# Can a poverty-reducing and progressive tax and transfer system hurt the poor?

**DOI:** 10.1016/j.jdeveco.2016.04.001

**Published:** 2016-09

**Authors:** Sean Higgins, Nora Lustig

**Affiliations:** a Department of Economics, Tulane University, 206 Tilton Hall, 6823 St. Charles Ave., New Orleans, LA 70118, United States; b Haas School of Business, University of California, Berkeley, 2220 Piedmont Avenue, Berkeley, CA 94720, United States; c Center for Global Development, 2055 L Street NW, Washington, D.C. 20036, United States; d Inter-American Dialogue, 1211 Connecticut Avenue NW Suite 510, Washington, D.C. 20036, United States

**Keywords:** Poverty, Horizontal equity, Progressivity, Fiscal impoverishment

## Abstract

To analyze anti-poverty policies in tandem with the taxes used to pay for them, comparisons of poverty before and after taxes and transfers are often used. We show that these comparisons, as well as measures of horizontal equity and progressivity, can fail to capture an important aspect: that a substantial proportion of the poor are made poorer (or non-poor made poor) by the tax and transfer system. We illustrate with data from seventeen developing countries: in fifteen, the fiscal system is poverty-reducing and progressive, but in ten of these at least one-quarter of the poor pay more in taxes than they receive in transfers. We call this fiscal impoverishment, and axiomatically derive a measure of its extent. An analogous measure of fiscal gains of the poor is also derived, and we show that changes in the poverty gap can be decomposed into our axiomatic measures of fiscal impoverishment and gains.

## Highlights

• Poverty-reducing and progressive tax and transfer systems can make some poor poorer
• In ten developing countries, at least one-fourth of poor are fiscally impoverished
• We derive axiomatic measures of fiscal impoverishment and gains of the poor
• The change in the poverty gap can be decomposed into fiscal impoverishment and gains

## 1 Introduction

Anti-poverty policies are often evaluated in isolation from the taxes used to pay for them.^1^ If, however, taxes cancel out the benefits of transfers for many poor households, so that some poor pay more in taxes than they receive in transfers, the objective of these policies might be compromised. This is especially important when poverty traps exist at the individual level (e.g., Ghatak, 2015, Ravallion, 2015): a tax and transfer system in which many poor pay more in taxes than they receive in transfers risks pushing the transiently poor into chronic poverty by shifting their after tax and transfer incomes below their individual-specific poverty trap thresholds.

Recently, the connection between anti-poverty policies and the taxes used to pay for them has come into the spotlight in the debates over the United Nations' Post-2015 Sustainable Development Goals. In recognition of the resources necessary to achieve these ambitious development goals, and partly as a consequence of austerity in advanced countries (and thus lower anticipated flows of international aid to developing countries), much of the discussion has focused on how developing countries should collect the revenue necessary to achieve the goals.^2^ Influential organizations such as the International Monetary Fund and World Bank emphasize the importance of efficient taxes with minimal exemptions (International Monetary Fund 2013, World Bank 2013). When concerns are raised about these taxes—such as a no-exemption value added tax—falling disproportionately on the poor, many argue that higher tax burdens on the poor are acceptable if they are accompanied by sufficiently large targeted transfers: “spending instruments are available that are better targeted to the pursuit of equity concerns” (Keen and Lockwood, 2010, p.141). Similarly, Engel et al. (1999, p. 186) assert that “it is quite obvious that the disadvantages of a proportional tax are moderated by adequate targeting” of transfers, since “what the poor individual pays in taxes is returned to her.” These taxes “might conceivably be the best way to finance pro-poor expenditures, with the net effect being to relieve poverty” (Ebrill et al., 2001, p. 105).

How can we be sure that what the poor individual pays in taxes is returned to her? Even if the net effect of taxes and transfers is to relieve poverty, are some poor made worse off? When taxes and transfers are analyzed in tandem to determine how they affect the poor, it is common to compare poverty before taxes and transfers (“pre-fisc”) to poverty after taxes and transfers (“post-fisc”). As we show in this paper, however, a fiscal system can be unambiguously poverty-reducing for a range of poverty lines and any poverty measure, yet still make a substantial proportion of the poor worse off. This phenomenon does not only occur with regressive taxes: we show that taxes and transfers can be globally progressive, unambiguously equalizing, and unambiguously poverty-reducing and *still* make many poor worse off. In other words, conventional tools used to measure how the poor are affected by the tax and transfer system are inadequate to measure whether some of the poor pay more in taxes than they receive in transfers, a phenomenon we call fiscal impoverishment (FI).

We also show that in practice, there are a number of countries with poverty-reducing and progressive tax and transfer systems that nevertheless make a substantial proportion of the poor poorer (or non-poor poor), illustrating with data from seventeen developing countries.^3^ In fifteen of these countries, post-fisc poverty is unambiguously lower than pre-fisc poverty (measured with any poverty line up to $1.25 per person per day in low and lower-middle income countries and $2.50 per day in upper-middle income countries)^4^ and the tax and transfer system is globally progressive and unambiguously equalizing, i.e., we would conclude that the tax and transfer system unambiguously benefits the poor using conventional measures, potentially overlooking impoverishment. In all of these countries, some degree of FI occurs, and in ten of them we find that at least one-quarter of the poor pay more in taxes than they receive in transfers.

In light of the debate about financing anti-poverty policies and the Sustainable Development Goals, it is necessary to fill this gap in the measurement arsenal and develop a measure of this phenomenon that adheres to certain properties. We axiomatically derive a measure of FI, as well as an analogous measure for fiscal gains of the poor (FGP), which captures the extent to which some poor receive more in transfers than they pay in taxes.^5^ We then show how a commonly used measure of poverty that overlooks the extent of FI, the poverty gap, can be decomposed into FI and FGP components using our axiomatic measures, again illustrating with data from seventeen developing countries. Because the extent of FI and FGP depends on the particular poverty line used, we also propose dominance criteria that can be used to determine whether one fiscal system (such as the one that would occur after a proposed reform) causes unambiguously less FI or more FGP than another (such as the current system) over a range of poverty lines. We analyze FI and FGP over a range of poverty lines in Brazil, which is a pertinent example due to the coexistence of high tax burdens on the poor (Baer and Galvão, 2008, Goñi et al., 2011) and lauded poverty-reducing cash transfer programs: a large-scale conditional cash transfer program that reaches over one-fourth of all Brazilian households and a non-contributory pension program for the elderly poor that reaches one-third of all elderly (Levy and Schady, 2013, Table 1).

Section 2 uses hypothetical and empirical examples to show that common tools to assess how the tax and transfer system affects the poor can fail to capture FI. Section 3 axiomatically derives a measure that does capture FI; it then proposes a partial FI ordering that can be used to compare the level of FI induced by two fiscal systems for any poverty line. Section 4 derives an analogous measure and partial ordering for FGP and shows that the poverty gap can be decomposed into our axiomatic measures of FI and FGP. Section 5 uses data from seventeen developing countries to illustrate the axiomatic measures and poverty gap decomposition. Section 6 concludes, and the formal axioms and proofs are collected in the Appendix.

## 2 The problems with conventional measures

Through a number of examples, we illustrate and explain the problems with conventional measures of poverty, horizontal equity, and progressivity. Of course, these measures are still quite important for assessing a tax and transfer system; we merely aim to show that they do not capture everything we are interested in. First, in Section 2.1 we show the problem with poverty measures when they are used to compare poverty before and after taxes and transfers. Although comparisons of pre-fisc and post-fisc poverty are common in empirical studies (e.g., DeFina and Thanawala, 2004, Hoynes et al., 2006), poverty measures can overlook fiscal impoverishment because they obey the anonymity axiom (which is usually taken as an innocuous and desirable axiom): the tax and transfer system can reduce poverty while simultaneously making a substantial portion of the poor poorer, or making some non-poor poor. The anonymity axiom is not the only culprit for the shortcomings of existing measures, however: in Section 2.2 we show that measures designed to incorporate information about individuals' pre-fisc positions, such as measures of horizontal equity and progressivity, can also fail to capture FI.^6^ To show that these shortcomings of conventional measures are not confined to contrived hypothetical examples, but rather occur frequently in practice, in Section 2.3 we present examples from seventeen developing countries: in ten, the tax and transfer system is poverty-reducing and progressive, but hurts a substantial portion of the poor by pushing them deeper into poverty.

### 2.1 Poverty measures

Suppose the change in poverty caused by the fiscal system will be evaluated over a range of poverty lines, including lines greater than 6 and less than or equal to 10. Suppose there are three individuals in society with pre-fisc incomes of 5, 8, and 20, and (retaining the order of the individuals) post-fisc incomes 9, 6, and 18. For any poverty line in the range we are considering, and for any poverty measure in a broad class of measures, poverty has either not changed or decreased. This is because the poorest individual in the pre-fisc income distribution has an income of 5 and the second-poorest 8, while in the post-fisc distribution, the poorest has an income of 6 and the second-poorest 9. Poverty comparisons do not take into account that the poorest individual in the post-fisc distribution, with an income of 6, is not the poorest individual in the pre-fisc distribution who has an income of 5, but instead had an income of 8 in the pre-fisc distirbution and paid 2 more in taxes than she received in transfers. Depending on the exact poverty line chosen within the range we are considering, this individual was either pre-fisc poor and lost income to the fiscal system, or pre-fisc non-poor and pushed into poverty by the fiscal system.

It is clear, then, that poverty measures are inadequate to measure whether some of the poor pay more in taxes than they receive in transfers. Stochastic dominance tests, which are used to determine whether poverty is unambiguously lower in one income distribution than another for any poverty line and a broad class of poverty measures (Atkinson, 1987, Foster and Shorrocks, 1988), are also inadequate. This is because poverty measures and stochastic dominance tests are anonymous with respect to pre-fisc income: they compare the pre- and post-fisc income distributions without paying attention to the specific pre-fisc to post-fisc trajectory of particular individuals' incomes. The anonymity axiom, normally considered an innocuous and desirable property, becomes problematic when we are concerned with how the fiscal system affects the poor: in the words of Amiel and Cowell (1994, p. 448–9), “anonymity itself may be questionable as a welfare criterion when the social-welfare function is to take into account something more than the end-state distribution of incomes.” Anonymity implies that poverty measures fail to take into account individuals' initial positions, and thus whether some are being made poorer by the tax and transfer system.^7^

**Fig. 1:**
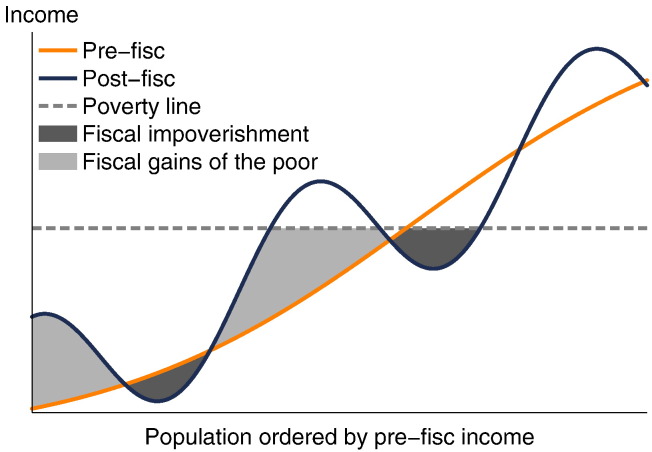
Stylistic illustration of fiscal impoverishment and gains to the poor.

To illustrate visually, Fig. 1 shows a stylistic representation of the pre- and post-fisc incomes of a population ordered by pre-fisc income. The increasing curve represents pre-fisc income, the wavy curve post-fisc income, and the dashed line the poverty line; because some individuals receive more in transfers than they pay in taxes, while others pay more in taxes than they receive in transfers, the post-fisc income curve is sometimes above and sometimes below the pre-fisc income curve. Although post-fisc poverty is lower than pre-fisc poverty because the losses of some poor are more than compensated by the gains of other poor, there is FI. The extent of FI is shown by the dark-shaded areas, while the light-shaded areas represent the extent of FGP (using the measures we axiomatically derive in 3, 4).

### 2.2 Horizontal equity and progressivity

Anonymity is not the only reason conventional measures overlook fiscal impoverishment: non-anonymous measures such as horizontal equity and progressivity, which are designed to incorporate information about an individual's pre-fisc position, can fail to capture FI because they are not concerned with whether her net tax burden (taxes paid minus transfers received) is positive or negative. Denote income before taxes and transfers by $y_{i}^{0}\in R_{+}$ and income after taxes and transfers by $y_{i}^{1}\in R_{+}$ for each *i* ∈ *S*, where *S* is the set of individuals in society. Consider a range of potential poverty lines $Z\subset R_{+}$. Each individual's income before or after taxes and transfers is arranged in the vector *y*^0^ or *y*^1^, both ordered in ascending order of pre-fisc income $y_{i}^{0}$—even if reranking occurs, the order of the *y*^1^ vector reflects the pre-fisc income ranking.

Horizontal equity can be defined in two ways: the reranking definition, which requires that no pair of individuals switch ranks, and the classical definition, which requires that pre-fisc equals are treated equally by the tax and transfer system. Under either definition, the existence or absence of horizontal equity among the poor does not tell us whether FI has occurred. Even if some are impoverished by the tax and transfer system, the ranking among the poor may not change (so there is horizontal equity by the reranking definition) and pre-fisc equals may be impoverished to the same degree (so there is classical horizontal equity): e.g., $Z=(6,10],\;y^{0}=(1,1,7,7,13),\;y^{1}=(3,3,6,6,11)$. Nor does horizontal inequity among the poor necessarily imply FI, because there could be reranking among the poor or unequal treatment among pre-fisc equals when the tax and transfer system lifts incomes of some of the poor without decreasing incomes of any poor: e.g., $Z=(6,10],\;y^{0}=(5,5,6,20),\;y^{1}=(5,7,6,18)$.

A tax and transfer system is everywhere progressive when net taxes (i.e., taxes minus benefits), relative to pre-fisc income, increase with income (Duclos, 1997, Lambert, 1988). The tax and transfer system can be progressive (and unambiguously equalizing) but cause fiscal impoverishment: e.g., Z=(6,10], *y*^0^ = (1,3,7,13), *y*^1^ = (3,4,6,11); net taxes relative to pre-fisc income increase with income, but the third individual whose income falls from 7 to 6 is fiscally impoverished. Thus, progressivity is not a sufficient condition to ensure that FI does not occur. Nor is progressivity a necessary condition for the absence of FI: e.g., Z=(6,10], *y^0^* = (1,3,7,14), *y^1^* = (1,5,8,11), which involves no FI but is not everywhere progressive because net taxes first decrease with income when moving from the poorest to the second-poorest, then increase with income thereafter.

Table 1 summarizes the examples presented in 2.1, 2.2 to show that conventional tools—specifically, poverty measures (and stochastic dominance tests) and measures of or tests for horizontal equity and progressivity—can overlook FI.

### 2.3 Real-world examples

The problems with conventional measures are not limited to contrived hypothetical examples. In a number of countries, we observe an unambiguous reduction in poverty and a globally progressive tax and transfer system, while a significant proportion of the poor are fiscally impoverished. Using the income concepts from Higgins et al. (2015), we compare market income (before taxes and transfers) to post-fiscal income (after direct and indirect taxes, direct cash and food transfers, and indirect subsidies) in seventeen developing countries. We use post-fiscal income as the after taxes and transfers income concept even though taxes are used to fund more than just direct cash and food transfers and indirect subsidies from the government (e.g., they are used to fund public goods and services, many of which also reach the poor) because this is the income concept relevant for measuring poverty: it is “disposable money and near-money income” that should be compared to the poverty line when the latter is based on “a poverty budget for food, clothing, shelter, and similar items” (Citro and Michael, 1995, p. 212, 237). For low and lower-middle income countries, we use a poverty line of $1.25 per person per day; for upper middle income countries, $2.50 per day. Table 2 column 1 shows the pre-fisc (market income) poverty headcount and column 2 shows the change in poverty from the pre-fisc to the post-fisc income distribution; countries in which poverty increased due to the fiscal system are excluded.^8^

Moving to the progressivity of the tax and transfer system and change in inequality in each country, column 3 shows the pre-fisc Gini coefficient and column 4 shows the Reynolds and Smolensky (1977) index, which is a summary indicator corresponding to tests of global progressivity; the Reynolds–Smolensky equals the pre-fisc Gini minus the concentration coefficient of post-fisc income with respect to pre-fisc income, and thus globally progressive systems have a positive Reynolds–Smolensky index. Column 5 shows the change in inequality, with negative numbers indicating that inequality fell as a result of the tax and transfer system.^9^

Since we do not derive an axiomatic measure of FI until Section 3, here we use two intuitively appealing measures likely to have policy traction. Column 6 shows the percent *of the population* that are fiscally impoverished and column 7 the percent *of the post-fisc poor* that are fiscally impoverished. Although all of the countries in Table 2 experienced a reduction in poverty and inequality due to the tax and transfer system, the amount of FI varies greatly between countries. In ten countries—Armenia, Bolivia, Brazil, El Salvador, Guatemala, Indonesia, Mexico, Russia, Sri Lanka, and Tunisia—between one-quarter and two-thirds of the post-fisc poor lost income to the fiscal system.^10^ In other countries, this figure is much lower, at 13.3% of the post-fisc poor in South Africa (but, due to the high proportion of the total population that is poor, still 5.9% of the total population) and 3.2% of the post-fisc poor in Ecuador.

Even when poverty increases from pre-fisc to post-fisc income and hence we know that FI has occurred (as in Ghana and Ethiopia), it is impossible to tell its extent without explicit measures like the ones we propose in Section 3. A stark example of this comes from Ethiopia, where looking at poverty and progressivity numbers alone greatly masks the extent of FI: the headcount ratio at $1.25 per day increases from 31.9% to 33.2% of the population, while the squared poverty gap and Gini coefficient fall as a result of taxes and transfers (World Bank, 2015). Nevertheless, applying our measures to the same data, Hill et al. (forthcoming) find that 28.5% of Ethiopians and over 80% of the post-fisc poor experience FI.

Even if we add the value of public spending on education and health (imputed at their government cost to families who report a child attending public school or who report using public health facilities), fiscal impoverishment is still high in several countries: in Armenia, Ethiopia, Indonesia, Russia, and Tunisia, between 25 and 50% of those who are fiscally impoverished before adding in benefits from public spending on health and education are still fiscally impoverished when these benefits are included as transfers.

## 3 Measures of fiscal impoverishment

To assess anti-poverty policies in tandem with the taxes used to finance them, it is important to have measures of the extent of fiscal impoverishment. In the last section, we provided a glimpse of FI in several developing countries using two simple, straight-forward, and intuitive measures that—given these features—can be useful for policy discussions. These two measures also have drawbacks, however. To illustrate their limitations, we begin by providing more detail about the two measures. For a particular poverty line z∈Z, there is *fiscal impoverishment* if $y_{i}^{1}<y_{i}^{0}$ and $y_{i}^{1}<z$ for some individual *i* ∈ *S*. In other words, the individual could be poor before taxes and transfers and made poorer by the fiscal system, or non-poor before taxes and transfers but poor after. Both straight-forward measures count the number of individuals who meet this condition (and are thus fiscally impoverished) in the numerator. The proportion *of the population* who are fiscally impoverished (column 6 of Table 2) divides this numerator by the number of individuals in society, while the proportion *of the post-fisc poor* who are fiscally impoverished (column 7) divides it by the number who are post-fisc poor $\left(\text{with}\;y_{i}^{1}<z\right)$.

In the context of poverty measurement, Sen (1976, p. 219) proposes a monotonicity axiom requiring that, all else equal, “a reduction in income of a person below the poverty line must increase the poverty measure.” We propose a similar axiom for FI measures requiring that a larger decrease in post-fisc income for an impoverished person, all else equal, must increase the FI measure. Monotonicity is violated by the straight-forward measures, which do not increase when an impoverished person becomes more impoverished because she counts as one impoverished individual in the measure's numerator regardless of how much income she loses to the fiscal system.^11^

### 3.1 Axioms

We propose eight properties desirable for a robust measure of FI; we describe these properties here and formally define them in the Appendix. Throughout, we assume that income is measured in real terms and has been converted to a common currency such as US dollars adjusted for purchasing power parity, thereby simplifying away concerns about inflation or currency conversions if comparing FI over time or across countries.

Our *FI monotonicity* axiom described above implies not only that the FI measure must be strictly increasing in the extent to which an impoverished individual is impoverished (ceteris paribus), but also that the measure must be strictly increasing in the number of individuals that are impoverished, holding fixed the amount of FI experienced by others. The *focus* axiom, analogous to Sen’s (1981) focus axiom for poverty measurement, says that different income changes to the non-impoverished—provided that they remain non-impoverished—leave the FI measure unchanged. Given the focus axiom, it is natural to impose a *normalization* that if no one is impoverished, the FI measure equals zero. Note that this normalization axiom is not instrumental to our result: if we did not impose it, our result would be that our axioms uniquely determine a measure of FI up to a linear (rather than proportional) transformation.^12^

Similar to Chakravarty's (1983)*continuity* axiom for poverty measures, we require the FI measure to be continuous in pre-fisc income, post-fisc income, and the poverty line (since we may want to assess FI for a range of possible poverty lines). This is stronger than Foster and Shorrocks's (1991) restricted continuity axiom which only requires the measure to be continuous in incomes *below* the poverty line and left-continuous *at* the poverty line, thus allowing the measure to jump discontinuously at the poverty line; see Zheng, 1997, Permanyer, 2014 for arguments in favor of using the stronger continuity axiom in the contexts of unidimensional and multidimensional poverty measures.

Because “the names of income recipients do not matter” (Zheng, 1997, p. 131), we impose a *permutability* axiom requiring that if we take each individual's pre- and post-fisc income pair and (keeping each pre- and post-fisc income pair as a bundle) shuffle these around the population, FI is unchanged. We use the term “permutability” rather than symmetry or anonymity because—although both have been used in the same way we use permutability above (e.g., Cowell, 1985, Fields and Fei, 1978, Plotnick, 1982)—symmetry and anonymity have also taken on different definitions. Symmetry can instead mean, for two income distributions *X* and *Y* and a distance measure *d*, that *d*(*X*,*Y*) = *d*(*Y*,*X*); the two income distributions are treated symmetrically: losses are not distinguishable from gains (Ebert, 1984, Fields and Ok, 1999). Anonymity can instead mean that the measure compares the cumulative distribution of pre-fisc income, *F*_0_, to that of post-fisc income, *F*_1_, without regard to where a particular individual at position *j* in *F*_0_ ended in *F*_1_ (e.g., Bourguignon, 2011, Bourguignon, 2011). In other words, an anonymous measure would compare the pre-fisc income of the *j*th poorest individual in *F*_0_ to the post-fisc income of the *j*th poorest individual in *F*_1_, even though “they are not necessarily the same individuals” because of reranking (Bourguignon, 2011a, p. 607).

Next, we must decide whether our measure of FI should be absolute or relative (recalling that we assume income to be in real terms of a constant currency, so arguments about inflation or currency exchange should not affect the decision). Suppose each poor individual's pre-fisc income increases by $1, taxes and transfers are held fixed, and the price of one essential good in the basic goods basket, normalized to have one unit in the basket, also increases by $1 per unit.^13^ Each poor individual remains the same distance below the poverty line; that distance represents the amount of additional income she needs to afford adequate nutrition and other basic necessities. For those who experience FI, it is the absolute increase in the distance between that individual's income and the poverty line that matters in terms of the quantity of basic goods she can buy. Hence, we assume that if all pre- and post-fisc incomes increase by $1 and the poverty line also increases by $1, FI should remain unchanged. We thus impose *translation invariance*.

Given our above argument for absolute measures, we also impose *linear homogeneity*: if all incomes and the poverty line are multiplied by the same factor, the measure of FI changes by that factor. Instead, specifying homogeneity of degree zero (scale invariance) would be incompatible with translation invariance for the reasons explored in Zheng (1994). Since we assume that income is expressed in real terms and a common currency, our measure is nevertheless insensitive to inflation or currency changes. The translation invariance and linear homogeneity axioms have been used together in axiomatic derivations of measures of inequality (Kolm, 1976), poverty (Blackorby and Donaldson, 1980), economic distance (Chakravarty and Dutta, 1987, Ebert, 1984), and mobility (Fields and Ok, 1996, Mitra and Ok, 1998).^14^

Our final axiom is based on a concept introduced to the poverty literature by Foster et al. (1984, p. 761), who argue that “at the very least, one would expect that a decrease in the poverty level of one subgroup ceteris paribus should lead to less poverty for the population as a whole.” Similarly, it would be desirable for a measure of FI if a decrease in the measured FI for one subgroup of the population and no change in the measured FI for all other subgroups results in a decrease in the measured FI of the entire population. Hence, we impose a *subgroup consistency* axiom analogous to the one used for poverty measurement by Foster and Shorrocks (1991). In his survey of axiomatic poverty measurement, Zheng (1997, p. 137) notes that subgroup consistency “has gained wide recognition in the literature.”

### 3.2 An axiomatic measure of fiscal impoverishment

Proposition 1 *A measure satisfying FI monotonicity, focus, normalization, continuity, permutability, translation invariance, linear homogeneity, and subgroup consistency is uniquely determined up to a proportional transformation, and given by*

(1) $$f\left(y^{0},\;y^{1};\;z\right)=\kappa \sum_{i\in S}\left(\min\left\{y_{i}^{0},\;z\right\}-\min\left\{y_{i}^{0},\;y_{i}^{1},\;z\right\}\right).$$

The summand for individual *i* behaves as follows. For an individual who was poor before taxes and transfers and is impoverished $\left(y_{i}^{1}<y_{i}^{0}<z\right)$, it is equal to her fall in income, $y_{i}^{0}-y_{i}^{1}$. For an individual who was non-poor before taxes and transfers and is impoverished $\left(y_{i}^{1}<z\leq y_{i}^{0}\right)$, it equals her post-fisc poverty gap, or the amount that would need to be transferred to her to move her back to the poverty line (equivalently, to prevent her from becoming impoverished), $z-y_{i}^{1}$. For a non-impoverished pre-fisc non-poor individual $\left(y_{i}^{0}\geq z\;\text{and}\;y_{i}^{1}\geq z\right)$ it equals *z* − *z* = 0. For a non-impoverished pre-fisc poor individual $\left(y_{i}^{0}<z\;\text{and}\;y_{i}^{1}\geq y_{i}^{0}\right)$ it equals $y_{i}^{0}-y_{i}^{0}=0$. Hence, *f* sums the total amount of FI, multiplied by a factor of proportionality. This constant can be chosen based on the preferences of the practitioner: for example, *κ* = 1 gives total FI (the dark-shaded area in Fig. 1), while *κ* = |*S*|^ −1^ gives per capita FI.^15^

### 3.3 Fiscal impoverishment dominance criteria

Having identified the existence of FI in a country, a useful implementation of our FI measure would be to compare the degree of FI in two situations, e.g. by comparing the current fiscal system to a proposed reform. The choice of poverty line might, however, influence our conclusion about which situation entails higher FI. We thus present a partial FI ordering that can be used to determine if FI is unambiguously lower in one situation than another for any poverty line and any measure that satisfies FI monotonicity, focus, normalization, continuity, permutability, translation invariance, linear homogeneity, and subgroup consistency. Since we have already shown that a FI measure satisfies these axioms if and only if it takes the form in Eq. (1), a simple way to test for FI dominance for any measure satisfying those axioms and any poverty line in the domain of poverty lines Z is to simply compare the curves *f* (*y*^0^, *y*^1^; *z*) and *f* (*x*^0^,*x*^1^; *z*) across Z. Interestingly, if the minimum poverty line being considered is 0 (so $Z=[0,z^{+}]$, where *z*^ +^ is the maximum poverty line), there is an alternative (equivalent) way to test whether FI is unambiguously lower in one situation than another that uses a dominance test already developed in the mobility literature: Foster and Rothbaum's (2014) second order downward mobility dominance.

Proposition 2 *The following are equivalent.*

a) *FI is unambiguously lower in* (y^0^,y^1^) *than* (x^0^, x^1^) *for any poverty line in* [0, *z*^ +^] *and any measure satisfying FI monotonicity, focus, normalization, continuity, permutability, translation invariance, linear homogeneity, and subgroup consistency.*
b) *f* (y^0^, y^1^; *z*) < *f* (x^0^, x^1^; *z*) *for allz* ∈ [0, *z*^ +^].
c) (y^0^, y^1^) *second order downward mobility dominates* (x^0^, x^1^) *on* [0, *z*^ +^].

## 4 Fiscal gains of the poor

Most likely, we will be interested in more than just the extent to which some poor are not compensated for their tax burden with transfers: we will also want to know about the gains of other poor families, and the way in which a comparison of poverty before and after taxes and transfers can be decomposed into the losses and gains of different poor households. In this section, we formally define fiscal gains of the poor, briefly present the axioms for a measure of FGP analogous to those in Section 3.1 for a measure of FI, and present an axiomatic measure and partial ordering of FGP. We then show that a commonly used measure of poverty, the poverty gap, can be decomposed into our axiomatic measures of FI and FGP.

### 4.1 An axiomatic measure of fiscal gains of the poor

There are *fiscal gains of the poor* if $y_{i}^{0}<y_{i}^{1}$ and $y_{i}^{0}<z$ for some individual *i* ∈ *S*. The individual may or may not receive enough in net transfers to be post-fisc non-poor (i.e., it is possible that $z\leq y_{i}^{1}$ or $y_{i}^{1}<z$). Consider a pre-fisc poor individual who receives more in transfers than she pays in taxes. If she is given even more transfer income, while the pre- and post-fisc incomes of all others experiencing FGP do not change, FGP should not decrease; if she would have remained in poverty post-fisc without the additional transfer income, FGP should increase with the additional transfer. We impose these conditions in the *FGP monotonicity* axiom; we also impose FGP analogues of the other axioms from Section 3.1.

Proposition 3 *A measure satisfying FGP monotonicity, focus, normalization, continuity, permutability, translation invariance, linear homogeneity, and subgroup consistency is uniquely determined up to a proportional transformation, and given by*

(2) $$g\left(y^{0},\;y^{1};\;z\right)=\kappa \sum_{i\in S}\;\left(\min\left\{y_{i}^{1},\;z\right\}-\min\left\{y_{i}^{0},\;y_{i}^{1},\;z\right\}\right).$$

An individual who is pre-fisc poor and gains income from the tax and transfer system, but remains post-fisc poor $\left(y_{i}^{0}<y_{i}^{1}<z\right)$, contributes the amount of her income gain, $y_{i}^{1}-y_{i}^{0}$, to the measure of FGP. A pre-fisc poor individual that gains income and as a result has post-fisc income above the poverty line $\left(y_{i}^{0}<z\leq y_{i}^{1}\right)$ contributes the amount of net transfers that pulled her pre-fisc income to the poverty line, $z-y_{i}^{0}$. Someone who is pre-fisc poor and does not gain income $\left(y_{i}^{1}\leq y_{i}^{0}<z\right)$ contributes $y_{i}^{1}-y_{i}^{1}=0$. Someone who is pre-fisc non-poor $\left(z<y_{i}^{0}\right)$ also contributes 0 (for her, the summand equals *z* − *z* if she remains non-poor or $y_{i}^{1}-y_{i}^{1}$ if she loses income and becomes poor). For *κ* = 1, *g* equals the light-shaded area in Fig. 1.

As with fiscal impoverishment orderings, a fiscal gain partial ordering can be used to make unambiguous FGP comparisons for any poverty line and any measure satisfying our axioms. The ordering compares *g* (*y*^0^, *y*^1^; *z*) to *g* (*x*^0^, *x*^1^; *z*) for all z∈Z, and for $Z=[0,\;z^{+}]$ coincides with Foster and Rothbaum's (2014) second order upward mobility dominance (the proof proceeds similarly to the proof of Proposition 2 for FI).

### 4.2 Decomposition of the difference between pre-fisc and post-fisc poverty

The most common measures of poverty used in both policy circles and scholarly papers (e.g., Chen and Ravallion, 2010, Ravallion, 2012) are the poverty headcount ratio, which enumerates the proportion of the population that is poor, and the poverty gap, which takes into account how far the poor fall below the poverty line. The latter might be expressed in absolute terms, summing the gap between each poor person's income and the poverty line, in which case it can be thought of as the total amount that would need to be given to the poor to eliminate poverty (if targeting were perfect). Or it can be normalized, dividing the absolute poverty gap by the poverty line and population size, for example, to create a scale- and population-invariant measure. We use a general definition of the poverty gap that encompasses its absolute and normalized forms:

(3) $$p(y;\;z)=\nu (S,\;z)\underset{i\in S}{\sum }\;\left(z-y_{i}\right)I\left(y_{i}<z\right),$$

where *ν*(*S*,*z*) is a normalization factor. Two special cases are the *absolute poverty gap*, where *ν*(*S*,*z*) = 1, and the *poverty gap ratio*, where *ν*(*S*,*z*) = (*z*|*S*|)^ −1^. For simplicity and because a comparison of pre- and post-fisc poverty usually occurs for a fixed population and given poverty line, we assume that *S* and *z* are fixed in what follows.

Proposition 4 *A change in the poverty gap before and after taxes and transfers is equal to the difference between the axiomatic measures of FI and FGP from* Eqs. (1)*and*(2)*, multiplied by a constant.*

Given the assumption that the population and poverty line are fixed, *ν*(*S*,*z*) is a constant that we denote $\bar{\nu }$. The poverty gap in Eq. (3) can be rewritten as $p(y;z)=\bar{\nu }\sum_{i\in S}\left(z-y_{i}\right)I\left(y_{i}<z\right)=\bar{\nu }\sum_{i\in S}\left(z-\min\left\{y_{i},\;z\right\}\right)$, so we have $p\left(y^{1};\;z\right)-p\left(y^{0};\;z\right)=\bar{\nu }\sum_{i\in S}\left(z-\min\left\{y_{i}^{1},\;z\right\}\right)-\bar{\nu }\sum_{i\in S}\left(z-\min\left\{y_{i}^{0},\;z\right\}\right)$, or

$$\begin{array}{ll} p\left(y^{1};\;z\right)-p\left(y^{0};\;z\right) & =\bar{\nu }\left[\underset{i\in S}{\sum }\left(\min\left\{y_{i}^{0},\;z\right\}-\min\left\{y_{i}^{0},\;y_{i}^{1},\;z\right\}\right)\right)\\ & \;-\left(\underset{i\in S}{\sum }\left(\min\left\{y_{i}^{1},\;z\right\}-\min\left\{y_{i}^{0},\;y_{i}^{1},\;z\right\}\right)\right]\\ & =\frac{\bar{\nu }}{\kappa }\left[f\left(y^{0},\;y^{1};\;z\right)-g\left(y^{0},\;y^{1};\;z\right)\right]. \end{array}$$

Comparisons of pre- and post-fisc poverty are often used to assess whether the tax and transfer system helps or hurts the poor. This decomposition can be used to dig deeper into that net effect and observe the extent to which a net reduction in poverty masks the offsetting gains of some poor and impoverishment of others at the hands of the (possibly progressive) tax and transfer system.

## 5 Illustration

### 5.1 Results for seventeen developing countries

We saw in Section 2 that in fifteen of seventeen developing countries for which we have data, the tax and transfer system is poverty-reducing and progressive but, in many cases, fiscally impoverishes a significant proportion of the poor. In Table 3, we present FI and FGP results for these countries using the axiomatic measures derived in 3, 4. Column 1 gives total FI (i.e., the axiomatic measure from Eq. (1) with *κ* = 1) and column 2 total FGP, both expressed in millions of 2005 US dollars per year using purchasing power parity adjusted exchange rates. Because the axiomatic measure with *κ* = 1 is population variant, FI and FGP tend to be higher in more populous countries; these absolute amounts of FI and FGP can be useful, for example, in comparisons to the size of a country's main cash transfer program, as we show for Brazil below. To ease interpretation and comparison across countries, column 3 shows FI expressed as a percent of FGP, while columns 4 and 5 show FI and FGP per capita (where per capita refers to dividing by the entire population), normalized by the poverty line; each of these is population invariant.

There is large heterogeneity in the extent to which some poor are hurt by the tax and transfer system relative to the extent to which other poor gain, despite that the same range of policies, including direct taxes, direct cash and near-cash transfers, indirect consumption taxes, and indirect subsidies were considered in each country study. Among the upper-middle income countries, FI as a percent of FGP (using a poverty line of $2.50 per day) ranges from less than 1% in Ecuador to 40% in Tunisia. In low and lower-middle income countries, FI as a percent of FGP (using a poverty line of $1.25 per day) is even higher in some countries, reaching 55% in Guatemala and 81% in Bolivia; in Ethiopia and Ghana—the two countries in which post-fisc poverty is higher than pre-fisc poverty—FI exceeds FGP.

Column 6 shows the change in the poverty gap ratio from pre-fisc to post-fisc income, which by Proposition 4 can be decomposed into FI per capita minus FGP per capita, both normalized by the poverty line like the poverty gap ratio. This decomposition reveals some interesting traits of each country's tax and transfer system. For example, Ecuador achieves the same FGP per capita as Brazil but with nearly no FI, compared to substantial FI in Brazil; as a result, the poverty gap is reduced by more in Ecuador. The difference in FI might be attributable to the multiple consumption taxes levied at the state and federal levels in Brazil: these are high and often cascading, and consumption tax exemptions for basic goods are almost non-existent (Corbacho et al., 2013), compared to a system that exempts food, basic necessities, and medicine in Ecuador (Llerena Pinto et al., 2015). Interestingly, most of those experiencing FI are *not* excluded from the safety net; they *do* receive government transfers or subsidies: 65% of the impoverished in Brazil receive cash transfers from Bolsa Família, for example.

It is also noteworthy that Peru, one of the countries in which *less than* a quarter of the post-fisc poor experience FI, nevertheless redistributes low amounts to the poor, and thus has a low reduction in the poverty gap; this is consistent with Jaramillo's (2014, p. 391) finding that Peru's low poverty reduction induced by fiscal policy is “associated with low social spending rather than with inefficient spending.” Among three lower-middle income countries that each reduce the poverty gap ratio by about 0.3 percentage points (El Salvador, Guatemala, and Indonesia), Guatemala has high FI but also higher FGP, while El Salvador has lower FGP but very low FI, and Indonesia falls in the middle. We do not attempt to answer whether a lower-FI, lower-FGP or higher-FI, higher-FGP system is preferable from a welfare perspective, but note that this decomposition enables a substantially richer analysis than the typical comparison of poverty before and after taxes and transfers.

### 5.2 Results for a range of poverty lines in Brazil

So far, the FI and FGP results we have presented use a fixed poverty line ($1.25 in low and lower-middle income countries and $2.50 in upper-middle income countries). We now extend the analysis to a range of poverty lines, focusing the illustration on data from Brazil, using the Pesquisa de Orçamentos Familiares (Family Expenditure Survey) 2008–2009. The precise direct and indirect taxes, direct cash and food transfers, and indirect subsidies included in our analysis are described in detail in Higgins and Pereira (2014).

**Fig. 2:**
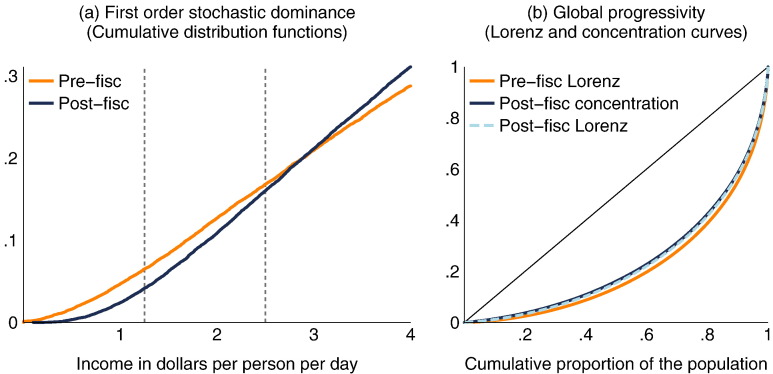
Conventional tools to assess the tax and transfer system in Brazil. Note: Dashed vertical lines included at common "international" poverty lines of $1.25 and $2.50 per person per day.

As we stated in Section 2.3, the tax and transfer system in Brazil is unambiguously poverty-reducing for any poverty line up to $2.50 per person per day, globally progressive, and unambiguously equalizing.^16^ This is shown in Fig. 2, where cumulative distribution functions reveal that the post-fisc distribution first order stochastically dominates the pre-fisc distribution on the domain [0,2.5], which implies an unambiguous reduction in poverty for any poverty line in this domain and any measure in a broad class (Atkinson, 1987, Foster and Shorrocks, 1988);^17^ the post-fisc concentration curve with respect to pre-fisc income dominates the pre-fisc Lorenz curve, which implies global progressivity (in the income redistribution sense; see Duclos, 2008); and the post-fisc Lorenz curve dominates the pre-fisc Lorenz curve, which implies that the fiscal system is unambiguously equalizing (Atkinson, 1970). If, however, we extend the maximum poverty line to, say, $4 per person per day—a poverty line frequently used by the World Bank when studying middle-income Latin American countries (e.g., Ferreira et al., 2013)— poverty is no longer unambiguously lowered by the fiscal system: for poverty lines above about $3 per day, the poverty headcount is higher after taxes and transfers than before. We would thus know that FI occurred using conventional measures and a poverty line above $3 per day, but would still be unaware of its extent without FI measures.^18^

Using the $2.50 line, we know that 5.6% of Brazil's population and over one-third of its post-fisc poor experience FI (Table 2); these impoverished individuals pay a total of $676 million more in taxes than they receive in transfers annually (Table 3), which is equivalent to 10% of the 2009 budget of Bolsa Família, Brazil's flagship anti-poverty program that reaches over one-fourth of the country's population. While substantial in size, this FI is dwarfed by FGP from Brazil's transfer programs, which totals over $3.5 billion. The absolute poverty gap, or the minimum amount that would need to be transferred to the poor to eliminate poverty if transfers were perfectly targeted, falls from $12.4 billion before taxes and transfers to $9.6 billion after. The change in the absolute poverty gap, $2.8 billion, looks impressive, but masks differential trends in two groups of the poor: those who gain (a total of $3.5 billion) and those who lose (a total of $676 million), as revealed by the decomposition of the change in the poverty gap derived in Section 4.

**Fig. 3:**
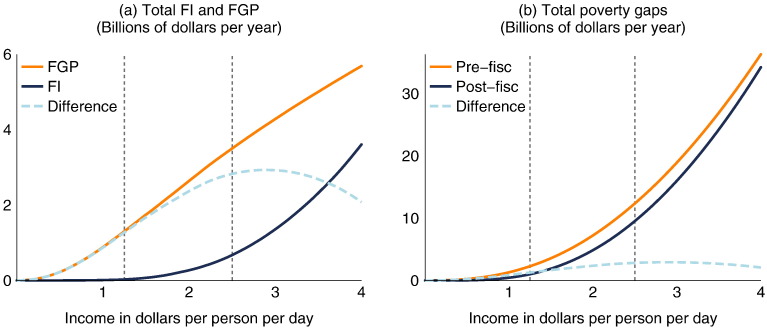
FI, FGP, and poverty gaps in Brazil for various poverty lines. Note: Dashed vertical lines included at common "international" poverty lines of $1.25 and $2.50 per person per day.

Fig. 3 shows how this decomposition and our axiomatic measures of total FI and FGP in Brazil vary with the poverty line. For low poverty lines, FI is essentially non-existent: at $1.25 per day, for example, total FI is $28 million per year, or 0.4% of the 2009 budget of Bolsa Família (Fig. 3a). This is not surprising in light of the unconditional component of the government cash transfer program Bolsa Família, available to households with income below 70 reais per person per month ($1.22 per day), regardless of whether the household has children or elderly members, and without conditions. At higher poverty lines, FI begins to increase more rapidly, and at a poverty line of $2.88 the rate of increase of FI exceeds the rate of increase of FGP: this can be seen by comparing the slopes of the solid curves in Fig. 3a, or by looking at the point where the difference between the two curves (plotted as the dashed curve in Fig. 3a) is at its maximum. By Proposition 4, this is also the point at which the absolute poverty gap reduction acheived by the fiscal system reaches its maximum, as seen by the dashed curve in Fig. 3b.

At this poverty line of $2.88 per day, where maximum poverty reduction is achieved, the difference between the pre-fisc and post-fisc poverty gaps is $2.9 billion. The eligibility cut-off for the conditional component of Bolsa Família, available to families with children who comply with certain education and health requirements, is $2.45 per person per day. Just above this line, a number of families still receive benefits due to program leakages, variable and mismeasured income, or components of income we are measuring that are not taken into account in the estimation of eligible income; not far above the line, however, families become much less likely to receive the program and we see a simultaneous deceleration of fiscal gains and acceleration of impoverishment.

## 6 Conclusions

Anti-poverty policies are increasingly being discussed in the same breath as the taxes used to pay for them. One example is the focus on mobilizing domestic resources to finance the policies necessary to achieve the United Nations' Post-2015 Sustainable Development Goals. To analyze transfers, subsidies, and taxes together, poverty comparisons and progressivity measures are often used. These measures, however, can lead us to conclude that the tax and transfer system unambiguously benefits the poor, when in fact a substantial number of poor are not compensated with transfers for their tax burdens. Indeed, we observe this in a number of developing countries: out of seventeen developing countries for which we have data, fifteen have tax and transfer systems that unambiguously reduce poverty and are globally progressive, but in ten of these at least one-quarter of the poor pay more in taxes than they receive in transfers and subsidies. In Brazil, for example, over one-third of the post-fisc poor experience fiscal impoverishment, paying a total of $676 million more in taxes than they receive in transfers and subsidies.

Given this shortcoming of conventional criteria and the debate about anti-poverty policies and the taxes used to pay for them, we propose a set of axioms that should be met by a measure of FI, and show that these uniquely determine the measure up to a proportional transformation. We also propose a partial ordering to determine when one fiscal system, such as that under a proposed reform, induces unambiguously less FI than another, such as the current system, over a range of possible poverty lines. To obtain a complete picture of the fiscal system's effect on the poor, we propose an analogous measure of fiscal gains of the poor, and show that the difference between the pre-fisc and post-fisc poverty gaps can be decomposed into our axiomatic measures of FI and FGP.

Our results can be extended to comparisons between two points in time or before and after a policy reform, rather than pre- and post-fisc. In comparison to the tools used to assess whether the tax and transfer system hurts the poor, tools from the literatures on pro-poor growth and policy reforms (tax and subsidy reforms, trade liberalization, etc.) suffer from similar limitations. For pro-poor growth,^19^ poverty measures and stochastic dominance tests are often used to assess whether poverty is unambiguously reduced over time; it directly follows from the first row of Table 1 that these will not necessarily capture that some of the poor become poorer over time. Hence, growth can appear unambiguously pro-poor even if a significant proportion of the poor are immiserized. Growth incidence curves (Ravallion and Chen, 2003) and related pro-poor partial orderings (Duclos, 2009) can fail to capture impoverishment for the same reason that stochastic dominance tests do: they are anonymous with respect to initial income. Although their non-anonymous counterparts (Bourguignon, 2011, Grimm, 2007, Van Kerm, 2009) resolve this issue in theory, in practice—to become graphically tractable—they average within percentiles, and hence impoverishment can still be overlooked if within some percentiles, some poor are “hurting behind the averages” (Ravallion, 2001, p. 1811).

For consumption tax and subsidy reform, Besley and Kanbur (1988) derive poverty-reducing conditions for reallocating food subsidies; these results are extended to commodity taxes and a broader class of poverty measures by Makdissi and Wodon, 2002, Duclos et al., 2008. Again, by the first row of Table 1, unambiguous poverty reduction does not guarantee that a substantial portion of the poor are not hurt by the reform. Studies that evaluate indirect tax reform with measures that take pre-fisc positions into account but average within groups, such as the percent gain or loss caused by the reform for each income or expenditure decile (Mirrlees et al., 2011, Chapter 9), can again overlook FI that occurs within each group.

In the literature on trade liberalization, Harrison et al. (2003 p. 97) note that “even the most attractive reforms will typically result in some households losing,” and recent efforts to measure welfare impact at the household level have been made following Porto (2006). Nevertheless, because results are presented at some aggregate level (e.g., by state or percentile), impoverishment due to trade reform could still be overlooked. For example, Nicita's (2009, p. 26) finding that “on average all income groups benefited from [Mexico's] trade liberalization, but to a varying extent” does not tell us the extent to which some households within each group were made worse off by the reform.

In each of these cases, our axiomatically derived FI measure could be used to quantify the impoverishment of those becoming poorer over time or the extent to which losers are hurt by policy reforms. Our decomposition could be used to examine the extent to which a decrease in poverty over time or due to a reform balances out the gains and losses of different households. Doing so, we will cease to overlook cases where growth, policy reform, or the tax and transfer system is poverty-reducing and progressive, yet hurts a substantial proportion of the poor.

## Acknowledgments

For detailed comments on earlier versions of the paper, we are grateful to Francesco Andreoli, Alan Barreca, Jean-Yves Duclos, John Edwards, Charles Kenny, Peter Lambert, Darryl McLeod, Mauricio Reis, Kathleen Short, Rafael Salas, Jay Shimshack, Harry Tsang, Paolo Verme, two anonymous referees, and Maitreesh Ghatak, the Editor. We are also grateful to Karim Araar, Abhijit Banerjee, François Bourguignon, Satya Chakravarty, Nachiketa Chattopadhyay, Chico Ferreira, Gary Fields, James Foster, Ravi Kanbur, Doug Nelson, Jukka Pirttilä, John Roemer, Jon Rothbaum, and Shlomo Yitzhaki for their useful feedback. For applying our measures to microdata in a number of developing countries and sharing the results with us for this paper, we are grateful to the many country study authors we cite in the paper. We thank Claudiney Pereira for collaboration on the analysis of Brazilian data and Ruoxi Li, Sandra Martínez-Aguilar, Adam Ratzlaff, Mel Reitcheck, and William Smith for research assistance. Work on this project was partially completed when S. Higgins was visiting Haas School of Business at UC Berkeley and the Center for Economic Studies at El Colegio de México with funding from the Fulbright–García Robles Public Policy Initiative. This paper is part of a larger project that received funding from the Bill & Melinda Gates Foundation (grant numbers OPP1097490 and OPP1335502). These institutions and funders are gratefully acknowledged.

## Figures and Tables

**Table 1:** Summary of the problems with conventional measures.

Measure	Issue	Example with Z=(6,10]
Poverty (and stochastic dominance)	*↓* poverty ⇏ no FI (anonymity)	*y*^0^ = (5,8,20), *y*^1^ = (9,6,18)
Horizontal equity	Horizontally equitable ⇏ no FI	*y*^0^= (1,1,7,7,13), *y*^1^ = (3,3,6,6,11)
	No FI ⇏ horizontally equitable	*y*^0^ = (5,5,6,20), *y*^1^ = (5,7,6,18)
Progressivity	Progressive ⇏ no FI	*y*^0^ = (1,3,7,13), *y*^1^ = (3,4,6,11)
	No FI ⇏ progressive	*y*^0^ = (1,3,7,14), *y*^1^ = (1,5,8,11)

**Table 2:** Poverty, inequality, and fiscal impoverishment in developing countries.

	(1)	(2)	(3)	(4)	(5)	(6)	(7)
	Pre-fisc	Change in	Pre-fisc	Reynolds–	Change in	Fiscally	Fiscally
	poverty	poverty	inequality	Smolensky	inequality	impoverished	impoverished
	headcount	headcount	(Gini)	(post-fisc	(*Δ*Gini)	as % of	as % of post-
Country (survey year)	(%)	(p.p.)		w.r.t. pre-fisc)		population	fisc poor
*Panel A: Upper-middle income countries, using a poverty line of $2.50 per day*							
Brazil (2008–2009)	16.8	−0.8	57.5	4.6	−3.5	5.6	34.9
Chile (2013)	2.8	−1.4	49.4	3.2	−3.0	0.3	19.2
Ecuador (2011–2012)	10.8	−3.8	47.8	3.5	−3.3	0.2	3.2
Mexico (2012)	13.3	−1.2	54.4	3.8	−2.5	4.0	32.7
Peru (2011)	13.8	−0.2	45.9	0.9	−0.8	3.2	23.8
Russia (2010)	4.3	−1.3	39.7	3.9	−2.6	1.1	34.4
South Africa (2010–2011)	49.3	−5.2	77.1	8.3	−7.7	5.9	13.3
Tunisia (2010)	7.8	−0.1	44.7	8.0	−6.9	3.0	38.5

*Panel B: Lower-middle income countries, using a poverty line of $1.25 per day*							
Armenia (2011)	21.4	−8.4	47.4	12.9	−9.2	6.2	52.3
Bolivia (2009)	10.9	−0.5	50.3	0.6	−0.3	6.6	63.2
Dominican Republic (2007)	6.8	−0.9	50.2	2.2	−2.2	1.0	16.3
El Salvador (2011)	4.3	−0.7	44.0	2.2	−2.1	1.0	27.0
Guatemala (2010)	12.0	−0.8	49.0	1.4	−1.2	7.0	62.2
Indonesia (2012)	12.0	−1.5	39.8	1.1	−0.8	4.1	39.2
Sri Lanka (2009–2010)	5.0	−0.7	37.1	1.3	−1.1	1.6	36.4

Sources: For Brazil, authors' calculations. For other countries, provided to us by the authors of the studies cited in Footnote 3.

Notes: p.p.=percentage points. w.r.t.=with respect to. Ethiopia and Ghana are not included in the table because poverty with a $1.25 per day poverty line increased from pre-fisc to post-fisc income (and hence they do not illustrate shortcomings of conventional measures). Country classifications are from the World Bank for the year of the survey.

**Table 3:** Fiscal impoverishment and gains of the poor in developing countries.

	(1)	(2)	(3)	(4)	(5)	(6)
	Total FI	Total FGP	FI as %	Per capita	Per capita	Change in
	($ millions	($ millions	of FGP	FI as %	FGP as %	poverty gap
Country (survey year)	per year)	per year)		of *z*	of *z*	ratio (p.p.)
*Panel A: Upper-middle income countries, using a poverty line of $2.50 per day*						
Brazil (2008–2009)	676.0	3503.6	19.3	0.39	2.02	−1.63
Chile (2013)	2.0	93.3	2.1	0.01	0.59	−0.58
Ecuador (2011–2012)	1.1	277.8	0.4	0.01	2.00	−1.99
Mexico (2012)	227.7	1446.5	15.7	0.21	1.35	−1.14
Peru (2011)	53.7	177.0	30.3	0.20	0.65	−0.45
Russia (2010)	84.9	1561.4	5.4	0.07	1.24	−1.17
South Africa (2010–2011)	186.6	5964.0	3.1	0.41	12.96	−12.56
Tunisia (2010)	20.8	52.0	40.0	0.23	0.59	−0.35

*Panel B: Low and lower-middle income countries, using a poverty line of $1.25 per day*						
Armenia (2011)	6.3	117.9	5.3	0.44	8.17	−7.74
Bolivia (2009)	25.9	32.2	80.6	0.55	0.68	−0.13
Dominican Republic (2007)	4.4	105.1	4.2	0.02	0.53	−0.51
El Salvador (2011)	1.2	11.1	11.1	0.04	0.39	−0.35
Ethiopia (2010–2011)	408.9	392.8	104.1	1.18	1.13	0.05
Ghana (2013)	25.9	9.9	262.1	0.22	0.08	0.13
Guatemala (2010)	20.7	37.8	54.9	0.33	0.61	−0.27
Indonesia (2012)	150.2	531.5	28.3	0.13	0.47	−0.34
Sri Lanka (2009–2010)	4.4	25.5	17.1	0.05	0.27	−0.23

Sources: For Brazil, authors' calculations. For other countries, provided to us by the authors of the studies cited in Footnote 3.

Notes: p.p. = percentage points. *z* denotes the poverty line. “$ millions” denotes millions of 2005 US dollars, at purchasing power parity adjusted exchange rates. Country classifications are from the World Bank for the year of the survey.

## Appendix A

### A.1 FI axioms

Consider pre- and post-fisc incomes $y_{i}^{0},y_{i}^{1}\in R_{+}$ for each *i* ∈ *S*; denote the vectors of pre- and post-fisc income for these individuals by *y*^0^ and *y*^1^, both ordered by pre-fisc income $y_{i}^{0}$. Now consider income vectors for the same individuals under different pre- and post-fisc scenarios, denoted by *x*^0^ and *x*^1^, both ordered by pre-fisc income $x_{i}^{0}$. The sets of impoverished individuals in scenarios (*y*^0^,*y*^1^) and (*x*^0^,*x*^1^) are denoted $I_{y}\equiv \{i\in S\;|\;y_{i}^{1}<y_{i}^{0}\text{ and}y_{i}^{1}<z\}$ and $I_{x}\equiv \left\{i\in S\;|\;x_{i}^{1}<x_{i}^{0}\text{ and}x_{i}^{1}<z\right\}$. A measure of FI is a function $f:{⋃}_{n=1}^{\infty }R_{+}^{n}\times {⋃}_{n=1}^{\infty }R_{+}^{n}\times R_{+}\to R$ , which takes as arguments the pre- and post-fisc income vectors and the poverty line.

Axiom 1 FI Monotonicity If $y_{i}^{0}=x_{i}^{0}$ for all *i* ∈ *S* and there exists *j* ∈ *I*_*y*_ ∪ *I*_*x*_ such that $y_{j}^{1}>x_{j}^{1}$, while $y_{k}^{1}=x_{k}^{1}$ for all *k* ∈ *I*_*y*_ ∪ *I*_*x*_ ∖{*j*}, then *f* (*y*^0^,*y*^1^;*z*) < *f* (*x*^0^,*x*^1^;*z*).

Axiom 2 Focus If $y_{i}^{0}=x_{i}^{0}$ and $y_{i}^{1}=x_{i}^{1}$ for all *i* ∈ *I*_*y*_ ∪ *I*_*x*_, then *f* (*y*^0^,*y*^1^;*z*) = *f* (*x*^0^,*x*^1^;*z*).

Axiom 3 Normalization $I_{y}=\emptyset \Rightarrow f\left(y^{0},y^{1};z\right)=0$.

Axiom 4 Continuity *f* is jointly continuous in $y_{i}^{0}$, $y_{i}^{1}$, and *z*.

Axiom 5 Permutability *f* (y^0^,y^1^;*z*) = *f* (y_*σ*_^0^,y_*σ*_^1^;*z*) for any permutation function *σ* : *S* → *S*, where $y_{\sigma }^{0}\equiv \left(y_{\sigma (1)}^{0},\ldots ,y_{\sigma (|S|)}^{0}\right)$ and $y_{\sigma }^{1}\equiv \left(y_{\sigma (1)}^{1},\ldots ,y_{\sigma (|S|)}^{1}\right)$.

Axiom 6 Translation Invariance *f* (*y*^0^ +*α* 1_|S|_,*y*^1^ +*α* 1_|S|_;*z* +*α*) = *f* (*y*^0^,*y*^1^;*z*) for all *α* ∈ℝ , where 1_|S|_ denotes a vector of ones with length |*S*|.

Axiom 7 Linear Homogeneity *f* (*λy*^0^,*λy*^1^;*λz*) = *λf* (*y*^0^,*y*^1^;*z*) for all *λ* ∈ℝ_ ++_.

Axiom 8 Subgroup Consistency Partition *S* into *m* subsets $S_{1},\ldots ,S_{m}$, and denote the vectors of pre- and post-fisc incomes for individuals belonging to subset *S*_*a*_, a∈{1,…,m}, by y_a_^0^ and y_a_^1^ or x_a_^0^ and x_a_^1^. If $f\left(y_{a}^{0},y_{a}^{1};z\right)<f\left(x_{a}^{0},x_{a}^{1};z\right)$ for some a∈{1,…,m} and $f\left(y_{b}^{0},y_{b}^{1};z\right)=f\left(x_{b}^{0},x_{b}^{1};z\right)$ for all b∈{1,…,m}∖{a}, then *f* (*y*^0^,*y*^1^;*z*) < *f* (*x*^0^,*x*^1^;*z*).

### A.2 FGP axioms

Let the sets of pre-fisc poor individuals experiencing fiscal gains under two scenarios be denoted $G_{y}\equiv \left\{i\in S\;|\;y_{i}^{0}<y_{i}^{1}\text{ and}y_{i}^{0}<z\right\}$ and $G_{x}\equiv \left\{i\in S\;|\;x_{i}^{0}<x_{i}^{1}\text{ and}x_{i}^{0}<z\right\}$. A measure of FGP is a function $g:{⋃}_{n=1}^{\infty }R_{+}^{n}\times {⋃}_{n=1}^{\infty }R_{+}^{n}\times R_{+}\to R$ , which takes as arguments the pre- and post-fisc income vectors and the poverty line.

Axiom 1′ FGP Monotonicity If $y_{i}^{0}=x_{i}^{0}$ for all *i* ∈ *S* and there exists *j* ∈ *G*_*y*_ ∪ *G*_*x*_ such that $y_{j}^{1}<x_{j}^{1}$, while $y_{k}^{1}=x_{k}^{1}$ for all *k* ∈ *G*_*y*_ ∪ *G*_*x*_ ∖{*j*}, then *g* (*y*^0^,*y*^1^;*z*) ≤ *g* (*x*^0^,*x*^1^;*z*), with strict inequality if $y_{j}^{1}<z$.

The remaining axioms for FI are desirable for a measure of FGP as well, and carry over directly to FGP after replacing *f* with *g*, *I*_*y*_ with *G*_*y*_, and *I*_*x*_ with *G*_*x*_.

### A.3 Proofs

Proof of Proposition 1 We begin with a lemma analogous to one of the propositions in Foster and Shorrocks (1991). To simplify notation, *y*_*a*_ ≡ (y_a_^0^,y_a_^1^) for a subset *S*_*a*_ of a partition of *S* into *m* subgroups a=1,…,m; similarly, *x*_*a*_ ≡ (x_a_^0^,x_a_^1^). We also define vectors $y_{-a}^{t}\equiv {\left(y_{b}^{t}\right)}_{b\neq a\in \{1,\ldots ,m\}}$, *t* ∈{0,1} as the vector of pre- or post-fisc incomes of all *i*∉*S*_*a*_ (similarly for $x_{-a}^{t}$) and $y_{-a}\equiv \left(y_{-a}^{0},y_{-a}^{1}\right)$, $x_{-a}\equiv \left(x_{-a}^{0},x_{-a}^{1}\right)$.

Lemma 1 **f*(y_a_,y_−a_;*z*) ≥ *f*(x_a_,y_−a_;*z*) ⇒ *f*(y_a_,x_−a_;*z*) ≥ *f*(x_a_,x_−a_;*z*).*

Proof By subgroup consistency, *f*(*y_a_*,*y*_−*a*_;*z*) ≥ *f*(*x_a_*,*y*_−a_;*z*) ⇒ *f*(*y_a_*;*z*) ≥ *f*(*x_a_*;*z*). (Suppose not. Then *f*(*y_a_*;*z*) < *f*(*x_a_*;*z*), which by subgroup consistency implies *f*(*y_a_*,*y*_−*a*_;*z*) < *f*(*x_a_*,*y*_−*a*_;*z*), a contradiction.) *f*(*y_a_*;*z*) ≥ *f*(*x_a_*;*z*) implies either *f*(*y_a_*;*z*) > *f*(*x_a_*;*z*) or *f*(*y_a_*;*z*) = *f*(*x_a_*;*z*). In the former case, it immediately follows by subgroup consistency that *f*(*y_a_*,*x*_−*a*_;*z*) ≥ *f*(*x_a_*,*x*_−*a*_;*z*). In the latter case, the implication is shown by contradiction. Suppose that *f*(*y_a_*,*x*_−*a*_;*z*) < *f*(*x_a_*,*x*_−*a*_;*z*). Then by subgroup consistency we have (since *f*(*y_a_*;*z*) = *f*(*x_a_*;*z*)) *f*(*y_a_*,*x*_−*a*_,*x_a_*;*z*) < *f*(*x_a_*,*x*_−*a*_,*y_a_*;*z*), which contradicts permutability. □

This lemma shows that a subgroup-consistent and permutable measure of FI is separable by group, using a definition of separability analogous to that used for preferences in the utility literature. Because the lemma can be reiterated within any particular subgroup to further separate individuals in that subgroup, we have that each set of individuals is separable (which is analogous to the “each set of sectors is separable” requirement in Gorman (1968, p. 368)). Hence, from Debreu (1960, Theorem 3), there exists a continuous FI function determined up to an increasing linear transformation of the form

$$f\left(y^{0},y^{1};z\right)=\alpha +\beta \sum_{i\in S}\phi_{i}\left(y_{i}^{0},y_{i}^{1},z\right)$$

where *ϕ*_*i*_ is a real-valued function for each *i* ∈ *S*. The additional requirement for Debreu's (1960) proof that more than two of the |*S*| elements of *S* are essential is satisfied as long as |*S*|≥ 3 and *f* is non-constant on [0,*z*], which in turn is implied by monotonicity as long as at least one individual is impoverished.^20^

Permutability implies that *ϕ*_*i*_ = *ϕ*_*j*_ for all *i*,*j* ∈ *S*, so we have $f\left(y^{0},y^{1};z\right)=\alpha +\beta \sum \phi \left(y_{i}^{0},y_{i}^{1},z\right)$ where *ϕ* is a real-valued function. By the focus and normalization axioms:

(4) $$\phi \left(y_{i}^{0},y_{i}^{1},z\right)=\left\{\begin{array}{cc} \tilde{\phi }\left(y_{i}^{0},y_{i}^{1},z\right)\; & \text{ if}y_{i}^{1}<y_{i}^{0}\text{ and}y_{i}^{1}<z\\ 0\; & \text{ otherwise.} \end{array}\right)$$

By the continuity of *f*, *ϕ* and $\tilde{\phi }$ must also be continuous. Consider an individual with $y_{i}^{0}>z$ and $y_{i}^{1}=z$. Since $y_{i}^{1}$ is not less than *z*, *i* is not impoverished, so by Eq. (4), $\phi \left(y_{i}^{0},y_{i}^{1},z\right)=0$. Now consider an alternative situation where ${\tilde{y}}_{i}^{1}=z-\epsilon$ for a sufficiently small *ϵ* > 0. In this scenario, $\tilde{\phi }$ cannot be a direct function of $y_{i}^{0}$ or *ϕ* would be discontinuous at *z*; instead, $\tilde{\phi }$ must be a direct function of just $y_{i}^{1}$ and *z* so that an infinitesimal decrease in $y_{i}^{1}$ below *z* results in an infinitesimal increase in *ϕ*. By a similar argument, for an individual with $y_{i}^{0}<z$, $y_{i}^{1}=y_{i}^{0}$, and ${\tilde{y}}_{i}^{1}=y_{i}^{0}-\epsilon$, $\tilde{\phi }$ cannot be a direct function of *z* and instead must directly depend only on $y_{i}^{1}$ and $y_{i}^{0}$ so that an infinitesimal decrease in $y_{i}^{1}$ below $y_{i}^{0}<z$ results in an infinitesimal increase in *ϕ*.

Given this, we can rewrite $\tilde{\phi }\left(y_{i}^{0},y_{i}^{1},z\right)=\tilde{\phi }\left(\min\left\{y_{i}^{0},z\right\},y_{i}^{1}\right)$. Since $\tilde{\phi }$ is only defined for those who are impoverished (i.e., those for whom $\min\left\{y_{i}^{0},y_{i}^{1},z\right\}=y_{i}^{1}$), we have

(5) $$\tilde{\phi }\left(y_{i}^{0},y_{i}^{1},z\right)=\tilde{\phi }\left(\min\left\{y_{i}^{0},z\right\},\min\left\{y_{i}^{0},y_{i}^{1},z\right\}\right)$$

(6) $$=\tilde{\phi }\left(\min\left\{y_{i}^{0},z\right\}-\min\left\{y_{i}^{0},y_{i}^{1},z\right\},0\right)$$

(7) $$=\left(\min\left\{y_{i}^{0},z\right\}-\min\left\{y_{i}^{0},y_{i}^{1},z\right\}\right)\tilde{\phi }(1,0)$$

where Eq. (6) follows from translation invariance and Eq. (7) from linear homogeneity. Noting that $\tilde{\phi }(1,0)$ is a constant (that is positive by monotonicity) and denoting it *γ*, we have

$$\phi \left(y_{i}^{0},y_{i}^{1},z\right)=\left\{\begin{array}{cc} \left(\min\left\{y_{i}^{0},z\right\}-\min\left\{y_{i}^{0},y_{i}^{1},z\right\}\right)\gamma \; & \text{ if}i\in I_{y}\\ 0\; & \text{ otherwise.} \end{array}\right)$$

For *i*∉*I*_*y*_ we can also write $\phi \left(y_{i}^{0},y_{i}^{1},z\right)=\left(\min\left\{y_{i}^{0},z\right\}-\right)\left(\min\left\{y_{i}^{0},y_{i}^{1},z\right\}\right)\gamma$ since the non-impoverished are either non-poor before taxes and transfers and non-poor after $\left(\Rightarrow \min\left\{y_{i}^{0},z\right\}=\right)\left(\min\left\{y_{i}^{0},y_{i}^{1},z\right\}=z\right)$ or poor before taxes and transfers but do not lose income to the fiscal system $\left(\Rightarrow \min\left\{y_{i}^{0},z\right\}=\min\left\{y_{i}^{0},y_{i}^{1},z\right\}=y_{i}^{0}\right)$. Therefore $f\left(y^{0},y^{1};z\right)=\alpha +\beta \gamma \sum_{i\in S}\left(\min\left\{y_{i}^{0},z\right\}-\min\left\{y_{i}^{0},y_{i}^{1},z\right\}\right)$. By normalization, *α* = 0, which completes the proof. □

Proof of Proposition 2 (a)⇔(b) follows immediately from Proposition 1. For (b)⇔(c), we begin by defining Foster and Rothbaum's (2014) second order downward mobility dominance.

Definition 1 (y^0^,y^1^) *second order downward mobility dominates* (x^0^,x^1^) on [0,*z*^ +^] if

$$\int_{0}^{z}m\left(y^{0},y^{1};c\right)dc<\int_{0}^{z}m\left(x^{0},x^{1};c\right)dc\;\;\forall \;z\in [0,z^{+}],$$

where $m\left(y^{0},y^{1};z\right)=|S{|}^{-1}\sum_{i\in S}I\left(y_{i}^{1}<z<y_{i}^{0}\right)$ is Foster and Rothbaum's (2014)*downward mobility curve*, measuring the proportion of the population that begins with income above each poverty line and ends with income below the line.

A sufficient condition for (b) being equivalent to (c) is $f\left(y^{0},y^{1};z\right)\propto \int_{0}^{z}m\left(y^{0},y^{1};c\right)dc$. For a given poverty line $z=\hat{z}$, partition the set *S* into four subsets: $S_{1}=\left\{i\in S\;|\;y_{i}^{1}<y_{i}^{0}<\hat{z}\right\}$, $S_{2}=\left\{i\in S\;|\;y_{i}^{1}<\hat{z}\leq y_{i}^{0}\right\}$, $S_{3}=\left\{i\in S\;|\;y_{i}^{0}\geq \hat{z},y_{i}^{1}\geq \hat{z}\right\}$, $S_{4}=\left\{i\in S\;|\;y_{i}^{0}<\hat{z},y_{i}^{0}\leq y_{i}^{1}\right\}$. For any subset *S*_*a*_ ⊂ *S*, denote $f_{a}(\cdot ;z)\equiv \kappa \sum_{i\in S_{a}}\left(\min\left\{y_{i}^{0},z\right\}-\min\left\{y_{i}^{0},y_{i}^{1},z\right\}\right)$ and $m_{a}(\cdot ;z)\equiv |S{|}^{-1}\sum_{i\in S_{a}}I\left(y_{i}^{1}<z<y_{i}^{0}\right)$.

Each *i* ∈ *S*_1_ experiences downward mobility on the interval $\left[0,\hat{z}\right]$ for all $z\in \left(y_{i}^{1},y_{i}^{0}\right)$ ⇒ individual *i* ∈ *S*_1_ increases *m*_1_(⋅;*z*) by |*S*|^ −1^ for $z\in \left(y_{i}^{1},y_{i}^{0}\right)$ and by zero for $z\leq y_{i}^{1}$ and $z\geq y_{i}^{0}$ ⇒ individual *i* ∈ *S*_1_ increases $\int_{0}^{\hat{z}}m_{1}(\cdot ;c)dc$ by $|S{|}^{-1}\left(y_{i}^{0}-y_{i}^{1}\right)$. Summing over all *i* ∈ *S*_1_, $\int_{0}^{\hat{z}}m_{1}(\cdot ;c)dc=\sum_{i\in S_{1}}|S{|}^{-1}\left(y_{i}^{0}-y_{i}^{1}\right)$.

$y_{i}^{1}<y_{i}^{0}<\hat{z}\;\;\forall \;i\in S_{1}\Rightarrow f_{1}(\cdot ;\hat{z})=\kappa \sum_{i\in S_{1}}\left(y_{i}^{0}-y_{i}^{1}\right)\Rightarrow$

(8) $$f_{1}(\cdot ;\hat{z})=\kappa |S|\int_{0}^{\hat{z}}m_{1}(\cdot ;c)dc.$$

Each *i* ∈ *S*_2_ experiences downward mobility on the interval $\left[0,\hat{z}\right]$ for all $z\in \left(y_{i}^{1},\hat{z}\right]$, which increases *m*_2_(⋅;*z*) by |*S*|^ −1^ for $z\in \left(y_{i}^{1},\hat{z}\right]$ and by zero for all other *z*⇒ individual *i* ∈ *S*_2_ increases $\int_{0}^{\hat{z}}m_{2}(\cdot ;c)dc$ by $|S{|}^{-1}\left(\hat{z}-y_{i}^{1}\right)$. Summing over all *i* ∈ *S*_2_, $\int_{0}^{\hat{z}}m_{2}(\cdot ;c)dc=\sum_{i\in S_{2}}|S{|}^{-1}\left(\hat{z}-y_{i}^{1}\right)$.

$y_{i}^{1}<\hat{z}\leq y_{i}^{0}\;\;\forall \;i\in S_{2}\Rightarrow f_{2}\left(\hat{z},\cdot \right)=\kappa \sum_{i\in S_{2}}\left(\hat{z}-y_{i}^{1}\right)\Rightarrow$

(9) $$f_{2}\left(\cdot ;\hat{z}\right)=\kappa |S|\int_{0}^{\hat{z}}m_{2}(\cdot ;c)dc.$$

Each *i* ∈ *S*_3_ does not experience downward mobility on the interval $\left[0,\hat{z}\right]$; summing over all *i* ∈ *S*_3_ and integrating over our domain, we have $\int_{0}^{\hat{z}}m_{3}(\cdot ;c)dc=0$. $y_{i}^{0}\geq \hat{z}$ and $y_{i}^{1}\geq \hat{z}\;\;\forall \;i\in S_{3}\Rightarrow$

(10) $$f_{3}(\cdot ;\hat{z})=\kappa \sum_{i\in S_{3}}(\hat{z}-\hat{z})=0=\kappa |S|\int_{0}^{\hat{z}}m_{3}(\cdot ;c)dc.$$

Similarly $\int_{0}^{\hat{z}}m_{4}(\cdot ;c)dc=0$ because each *i* ∈ *S*_4_ does not experience downward mobility on $\left[0,\hat{z}\right]$. $y_{i}^{0}<\hat{z}$ and $y_{i}^{0}\leq y_{i}^{1}\;\;\forall \;i\in S_{4}\Rightarrow$

(11) $$f_{4}(\cdot ;\hat{z})=\kappa \sum_{i\in S_{4}}\left(y_{i}^{0}-y_{i}^{0}\right)=0=\kappa |S|\int_{0}^{\hat{z}}m_{4}(\cdot ;c)dc.$$

Given the definitions of *f*_*a*_(⋅;*z*) and *m*_*a*_(⋅;*z*) and that *S* = *S*_1_ ∪ *S*_2_ ∪ *S*_3_ ∪ *S*_4_ and $S_{1}\cap S_{2}\cap S_{3}\cap S_{4}=\emptyset$, we have $f\left(y^{0},,,y^{1},;,z,),=,\sum_{a=1}^{4},f_{a},(,\cdot ,;,z\right)$ and $m\left(y^{0},y^{1};z\right)=\sum_{a=1}^{4}m_{a}(\cdot ;z)$. Hence, by Eqs. (8)–(11), $f\left(y^{0},y^{1};\hat{z}\right)=\kappa |S|\int_{0}^{\hat{z}}m(\cdot ;c)dc$. This holds for all $\hat{z}\in [0,z^{+}]$ since the choice of $\hat{z}$ was arbitrary, which completes the proof. □

Proof of Proposition 3 Analogous to the proof of Proposition 1 for FI. □

Proof of Proposition 4 Given in text. □

## Footnotes

1 We focus on anti-poverty policies that are redistributive in nature, one of the three categories of anti-poverty policies described in Ghatak (2015).

2 See, for example, the focus on domestic resource mobilization in United Nations (2015).

3 Our illustration uses results provided to us by the authors of country studies conducted as part of the Commitment to Equity (CEQ) Institute, located at Tulane University (www.commitmenttoequity.org). The countries included are Armenia (Younger and Khachatryan, 2016, Younger and Khachatryan, 2016), Bolivia (Paz Arauco et al., 2014), Brazil (authors' calculations), Chile (Martínez-Aguilar and Ortiz-Juarez, 2015), the Dominican Republic (Aristy-Escuder et al., 2016, Aristy-Escuder et al., 2016), Ecuador (Llerena Pinto et al., 2015), El Salvador (Beneke et al., 2015), Ethiopia (Hill et. al, 2016, Hill et. al, 2016), Ghana (Younger et al., 2015), Guatemala (Cabrera et al., 2015), Indonesia (Afkar et al., 2016, Afkar et al., 2016), Mexico (Aranda and Scott, 2015), Peru (Jaramillo et al., 2015), Russia (Lopez-Calva et al., 2016, Lopez-Calva et al., 2016), South Africa (Inchauste et al., 2016, Inchauste et al., 2016), Sri Lanka (Arunatilake et al., 2016, Arunatilake et al., 2016), and Tunisia (Shimeles et al., 2016, Shimeles et al., 2016). For an overview of the impact of taxes and social spending on inequality and poverty in many of these countries, see Lustig (2015).

4 The $1.25 per person per day poverty line (in 2005 US dollars adjusted for purchasing power parity) is approximately equal to the median poverty line of the fifteen poorest countries for which poverty line data are available, and the $2.50 line to the median of the world's low and middle income countries excluding the fifteen poorest (Chen and Ravallion, 2010).

5 Our axioms are adapted from the axiomatic poverty and mobility measurement literatures (see Foster, 2006, Zheng, 1997 for surveys of axiomatic poverty measurement and Fields, 2001 for a survey of axiomatic mobility measurement). Our resulting measure can be viewed as a censored directional version of the mobility measure derived by Fields and Ok (1996).

6 Other measures that are sometimes used, such as the percent of income gained or lost by each pre-fisc income decile, overlook FI for a distinct reason: they average over individuals, so for example the poorest decile could gain income on average while a substantial number of poor within the first decile lose income. We do not include these measures in this paper since the reason they overlook FI is obvious.

7 Amiel and Cowell (1994) also point out that the respect for income dominance axiom is only equivalent to the monotonicity axiom when anonymity is imposed. In the example from the previous paragraph, the post-fisc income distribution first order stochastically dominates the pre-fisc distribution on the domain from 0 to the maximum poverty line, so it would be evaluated as superior by any measure satisfying poverty focus and respect for income dominance (or, equivalently, poverty focus and both monotonicity and anonymity). It would not necessarily be evaluated as superior by a measure satisfying poverty focus and monotonicity but not anonymity, however. Other concerns with the anonymity axiom have also been pointed out: for example, it can clash with the Pigou–Dalton transfer axiom when there are households of different types (Ebert, 1997) and with the subgroup sensitivity axiom, an extension of the Pigou–Dalton transfer axiom to subgroups (Subramanian, 2006).

8 Although the table only shows poverty for a particular poverty line and poverty measure, it is also true that the post-fisc distribution first order stochastically dominates the pre-fisc distribution from 0 to the poverty line used for each country, meaning that poverty unambiguously fell for all poverty lines up to $1.25 or $2.50 and all poverty measures in a broad class.

9 We test global progressivity by dominance of the concentration curve of post-fisc with respect to pre-fisc income over the pre-fisc Lorenz curve, and test unambiguously equalizing by comparing the post-fisc and pre-fisc Lorenz curves.

10 If we instead scale down taxes so that they equal the transfers included in our analysis, which we avoid in the main analysis for the reasons mentioned above in defense of post-fiscal income as the after taxes and transfers income concept, FI is lower: for example, in Brazil 10.8% of the post-fisc poor are fiscally impoverished using this method.

11 Another simple tool to examine FI is the *q* × *q* transition matrix *P*, whose typical element *p*_*kl*_ represents the probability of being in post-fisc income group l∈{1,…,q} for an individual in pre-fisc income group k∈{1,…,q}. Measures based on *P* also fail to satisfy FI monotonicity and have the large drawback of not capturing FI among the poorest pre-fisc group (*k* = 1).

12 It is also possible to normalize by the measure's upper bound so that it always lies on the interval [0, 1] by specifying an axiom that if everyone loses all of their income to the fiscal system (the maximum possible FI), the measure of FI equals 1. We prefer to avoid normalizing in this way so that the class of axiomatic FI measures is more general.

13 To avoid inflation in this thought experiment, assume that there is an offsetting fall in the price of a good *not* in the basic goods basket and not consumed by the poor.

14 By requiring translation invariance and linear homogeneity, we are deriving a measure of *absolute* FI; from there, the measure can nevertheless be modified to obtain other types of desired measures such as a scale invariant measure. This is similar to the approach taken by Fields and Ok (1996), who axiomatically derive a measure of absolute mobility from which other desired measures such as mobility proportional to income can be obtained.

15 We do not impose a population invariance axiom; this axiom is commonly imposed but is criticized by Hassoun and Subramanian (2012). A subset of measures of form (1) are population invariant: choosing *κ* = |*S*|^ −1^ gives a measure that satisfies population invariance, while *κ* = 1 gives a measure that does not.

16 Nevertheless, the tax and transfer system reduces poverty by less than its potential under the type of optimal redistribution considered by Fellman et al. (1999), which follows a lexicographic maximin principle. Replacing the actual tax system with optimal taxes of this type (which, in total, equal the size of actual taxes), and replacing the actual distribution of Bolsa Família benefits with the optimal one (redistributing all transfers this way would completely eliminate poverty, so we only optimally redistribute Bolsa Família for illustration), the lowest income in the population would be $1.92 per day, the post-fisc poverty gap ratio would be 2.6% of the poverty line rather than 5.5%, and the post-fisc Gini would be 45.3 rather than 53.9.

17 We verify that this first order dominance is statistically significant at the 5% level using the asymptotic sampling distribution derived by Davidson and Duclos (2000) with a null hypothesis of non-dominance; the result is also robust to the type of data contamination considered in Cowell and Victoria-Feser (2002).

18 It is easy to show that if the post-fisc distribution does not first order stochastically dominate the pre-fisc distribution on the domain from 0 to the maximum poverty line, then FI has occurred.

19 Here, we are using the poverty-reducing or weak absolute definition of pro-poor (in the respective taxonomies of Kakwani and Son, 2008, Klasen, 2008), by which “growth is pro-poor if the poverty measure of interest falls” (Kraay, 2006, p. 198). We could instead adopt a relative definition of pro-poor growth (Kakwani and Pernia, 2000); growth-adjusted stochastic dominance tests can be used to determine when growth is unambiguously relatively pro-poor (Duclos, 2009), and it can be shown that this type of dominance can also occur despite a significant portion of the poor becoming poorer.

## References

[bb0005] Afkar R., Jellema J., Wai-Poi M., Inchauste G., Lustig N. (2016). The distributional impact of fiscal policy in Indonesia. The Distributional Impact of Fiscal Policy: Experience from Developing Countries.

[bb0010] Amiel Y., Cowell F. (1994). Monotonicity, dominance and the Pareto principle. Econ. Lett..

[bb0015] Aranda R., Scott J. (2015). CEQ Master Workbook for Mexico. Mimeo.

[bb0020] Aristy-Escuder J., Cabrera M., Sánchez-Martín M.E. (2016). An analysis of fiscal policy and income redistribution in the Dominican Republic. CEQ Working Paper 37.

[bb0025] Arunatilake N., Hewawasam J., Gunasekara N., Inchauste G., Lustig N. (2016). The distributional impact of fiscal policy in Sri Lanka. The Distributional Impact of Fiscal Policy: Experience from Developing Countries.

[bb0030] Atkinson A.B. (1970). On the measurement of inequality. J. Econ. Theory.

[bb0035] Atkinson A.B. (1987). On the measurement of poverty. Econometrica.

[bb0040] Baer W., Galvão A.F. (2008). Tax burden, government expenditures and income distribution in Brazil. Q. Rev. Econ. Finance.

[bb0045] Beneke M., Lustig N., Oliva J.A. (2015). El impacto de los impuestos y el gasto social en la desigualded y la pobreza en El Salvador. CEQ Working Paper 26.

[bb0050] Besley T., Kanbur R. (1988). Food subsidies and poverty alleviation. Econ. J..

[bb0055] Blackorby C., Donaldson D. (1980). Ethical indices for the measurement of poverty. Econometrica.

[bb0060] Bourguignon F. (2011). Non-anonymous growth incidence curves, income mobility and social welfare dominance. J. Econ. Inequal..

[bb0065] Bourguignon F. (2011). Status quo in the welfare analysis of tax reforms. Rev. Income Wealth.

[bb0070] Cabrera M., Lustig N., Morán H. (2015). Fiscal policy, inequality, and the ethnic divide in Guatemala. World Dev..

[bb0075] Chakravarty S.R. (1983). A new index of poverty. Math. Soc. Sci..

[bb0080] Chakravarty S.R., Dutta B. (1987). A note on measures of distance between income distributions. J. Econ. Theory.

[bb0085] Chen S., Ravallion M. (2010). The developing world is poorer than we thought, but no less successful in the fight against poverty. Q. J. Econ..

[bb0090] Citro C.F., Michael R.T. (1995). Measuring Poverty: A New Approach.

[bb0095] Corbacho A., Cibils V.F., Lora E. (2013). More than Revenue: Taxation as a Development Tool.

[bb0100] Cowell F.A. (1985). Measures of distributional change: an axiomatic approach. Rev. Econ. Stud..

[bb0105] Cowell F.A., Victoria-Feser M.-P. (2002). Welfare rankings in the presence of contaminated data. Econometrica.

[bb0110] Davidson R., Duclos J.-Y. (2000). Statistical inference for stochastic dominance and for the measurement of poverty and inequality. Econometrica.

[bb0115] Debreu G., Arrow K.J., Karlin S., Suppes P. (1960). Topological methods in cardinal utility theory. Mathematical Methods in the Social Sciences.

[bb0120] DeFina R.H., Thanawala K. (2004). International evidence on the impact of transfers and taxes on alternative poverty indexes. Soc. Sci. Res..

[bb0125] Duclos J.-Y. (1997). Measuring progressivity and inequality. Res. Econ. Inequal..

[bb0130] Duclos J.-Y., Durlauf S.N., Blume L.E. (2008). Horizontal and vertical equity. The New Palgrave Dictionary of Economics.

[bb0135] Duclos J.-Y. (2009). What is pro-poor?. Soc. Choice Welf..

[bb0140] Duclos J.-Y., Makdissi P., Wodon Q. (2008). Socially improving tax reforms. Int. Econ. Rev..

[bb0145] Ebert U. (1984). Measures of distance between income distributions. J. Econ. Theory.

[bb0150] Ebert U. (1997). Social welfare when needs differ: an axiomatic approach. Economica.

[bb0155] Ebrill L.P., Keen M., Summers V.P. (2001). The Modern VAT.

[bb0160] Engel E.M.R.A., Galetovic A., Raddatz C.E. (1999). Taxes and income distribution in Chile: some unpleasant redistributive arithmetic. J. Dev. Econ..

[bb0165] Fellman J., Jäntti M., Lambert P.J. (1999). Optimal tax-transfer systems and redistributive policy. Scand. J. Econ..

[bb0170] Ferreira F.H.G., Messina J., Rigolini J., López-Calva L.F., Lugo M.A., Vakis R. (2013). Economic Mobility and the Rise of the Latin American Middle Class.

[bb0175] Fields G.S. (2001). Distribution and Development.

[bb0180] Fields G.S., Fei J.C.H. (1978). On inequality comparisons. Econometrica.

[bb0185] Fields G.S., Ok E.A. (1996). The meaning and measurement of income mobility. J. Econ. Theory.

[bb0190] Fields G.S., Ok E.A. (1999). Measuring movement of incomes. Economica.

[bb0195] Foster J., de Janvry A., Kanbur R. (2006). Poverty indices. Poverty, Inequality and Development: Essays in Honor of Erik Thorbecke.

[bb0200] Foster J., Greer J., Thorbecke E. (1984). A class of decomposable poverty measures. Econometrica.

[bb0205] Foster J., Rothbaum J. (2014). The mobility curve: measuring the impact of mobility on welfare. Working Paper.

[bb0210] Foster J., Shorrocks A.F. (1988). Poverty orderings. Econometrica.

[bb0215] Foster J., Shorrocks A.F. (1991). Subgroup consistent poverty indices. Econometrica.

[bb0220] Ghatak M. (2015). Theories of poverty traps and anti-poverty policies. World Bank Econ. Rev..

[bb0225] Goñi E., López J.H., Servén L. (2011). Fiscal redistribution and income inequality in Latin America. World Dev..

[bb0230] Gorman W.M. (1968). The structure of utility functions. Rev. Econ. Stud..

[bb0235] Grimm M. (2007). Removing the anonymity axiom in assessing pro-poor growth. J. Econ. Inequal..

[bb0240] Harrison G.W., Rutherford T.F., Tarr D.G. (2003). Trade liberalization, poverty and efficient equity. J. Dev. Econ..

[bb0245] Hassoun N., Subramanian S. (2012). An aspect of variable population poverty comparisons. J. Dev. Econ..

[bb0250] Higgins S., Lustig N., Ruble W., Smeeding T.M. (2015). Comparing the incidence of taxes and social spending in Brazil and the United States. Rev. Income Wealth.

[bb0255] Higgins S., Pereira C. (2014). The effects of Brazil's taxation and social spending on the distribution of household income. Public Financ. Rev..

[bb0260] Hill R., Inchauste G., Lustig N., Tsehaye E., Woldehanna T., Inchauste G., Lustig N. (2016). A fiscal incidence analysis for Ethiopia. The Distributional Impact of Fiscal Policy: Experience from Developing Countries.

[bb0265] Hoynes H.W., Page M.E., Stevens A.H. (2006). Poverty in America: trends and explanations. J. Econ. Perspect..

[bb0270] Inchauste G., Lustig N., Maboshe M., Purfield C., Woolard I., Inchauste G., Lustig N. (2016). The distributional impact of fiscal policy in South Africa. The Distributional Impact of Fiscal Policy: Experience from Developing Countries.

[bb0275] International Monetary Fund (2013). Fiscal Monitor: Taxing Times. https://www.imf.org/external/pubs/ft/fm/2013/02/pdf/fm1302.pdf.

[bb0280] Jaramillo M. (2014). The incidence of social spending in Peru. Public Financ. Rev..

[bb0285] Jaramillo M., de la Flor L., Sparrow B. (2015). Are ethnic groupings invisible for fiscal policy in Peru? An incidence analysis of taxes and transfers on indigenous and non-indigenous Peruvians. Mimeo.

[bb0290] Kakwani N.C., Pernia E.M. (2000). What is pro-poor growth?. Asian Dev. Rev..

[bb0295] Kakwani N.C., Son H.H. (2008). Poverty equivalent growth rate. Rev. Income Wealth.

[bb0300] Keen M., Lockwood B. (2010). The value added tax: its causes and consequences. J. Dev. Econ..

[bb0305] Klasen S. (2008). Economic growth and poverty reduction: measurement issues using income and non-income indicators. World Dev..

[bb0310] Kolm S.-C. (1976). Unequal inequalities I. J. Econ. Theory.

[bb0315] Kraay A. (2006). When is growth pro-poor? Evidence from a panel of countries. J. Dev. Econ..

[bb0320] Lambert P.J., Eichhorn W. (1988). Net fiscal incidence progressivity: some approaches to measurement. Measurement in Economics: Theory and Application of Economics Indices.

[bb0325] Levy S., Schady N. (2013). Latin America's social policy challenge: education, social insurance, redistribution. J. Econ. Perspect..

[bb0330] Llerena Pinto F.P., Llerena Pinto M.C., Llerena Pinto M.A., Perez G. (2015). Social spending, taxes and income redistribution in Ecuador. CEQ Working Paper 28.

[bb0335] Lopez-Calva L.F., Lustig N., Matytsin M., Popova D., Inchauste G., Lustig N. (2016). Who benefits from fiscal redistribution in Russia?. The Distributional Impact of Fiscal Policy: Experience from Developing Countries.

[bb0340] Lustig N., Gupta S., Keen M., Clements B., de Mooij R. (2015). The redistributive impact of government spending on education and health: evidence from 13 developing countries in the Commitment to Equity project. Inequality and Fiscal Policy.

[bb0345] Makdissi P., Wodon Q. (2002). Consumption dominance curves: testing for the impact of indirect tax reforms on poverty. Econ. Lett..

[bb0350] Martínez-Aguilar S., Ortiz-Juarez E. (2015). CEQ Master Workbook for Chile. Mimeo.

[bb0355] Mirrlees J., Adam S., Besley T., Blundell R., Bond S., Chote R., Gammie M., Johnson P., Myles G., Poterba J. (2011). Tax by Design.

[bb0360] Mitra T., Ok E. (1998). The measurement of income mobility: a partial ordering approach. Economic Theory.

[bb0365] Nicita A. (2009). The price effect of tariff liberalization: measuring the impact on household welfare. J. Dev. Econ..

[bb0370] Paz Arauco V., Gray Molina G., Yáñez Aguilar E., Jiménez Pozo W. (2014). Explaining low redistributive impact in Bolivia. Public Financ. Rev..

[bb0375] Permanyer I. (2014). Assessing individuals' deprivation in a multidimensional framework. J. Dev. Econ..

[bb0380] Plotnick R. (1982). The concept and measurement of horizontal inequity. J. Public Econ..

[bb0385] Porto G.G. (2006). Using survey data to assess the distributional effects of trade policy. J. Int. Econ..

[bb0390] Ravallion M. (2001). Growth, inequality and poverty: looking beyond averages. World Dev..

[bb0395] Ravallion M. (2012). Why don’t we see poverty convergence?. Am. Econ. Rev..

[bb0400] Ravallion M., Atkinson A.B., Bourguignon F. (2015). The idea of antipoverty policy. Handbook of Income Distribution.

[bb0405] Ravallion M., Chen S. (2003). Measuring pro-poor growth. Econ. Lett..

[bb0410] Reynolds M., Smolensky E. (1977). Public Expenditures, Taxes and the Distribution of Income: The United States, 1950, 1961, 1970.

[bb0415] Sen A. (1976). Poverty: an ordinal approach to measurement. Econometrica.

[bb0420] Sen A. (1981). Poverty and Famines: An Essay on Entitlement and Deprivation.

[bb0425] Shimeles A., Moummi A., Jouini N., Lustig N. (2016). Fiscal incidence and poverty reduction: evidence from Tunisia. CEQ Working Paper 38.

[bb0430] Subramanian S., McGillivray M. (2006). Social groups and economic poverty: a problem in measurement. Inequality, Poverty, and Wellbeing.

[bb0435] United Nations (2015). Addis Ababa Action Agenda of the Third International Conference on Financing for Development.

[bb0440] Van Kerm P. (2009). Income mobility profiles. Econ. Lett..

[bb0445] World Bank (2013). Financing for Development Post-2015.

[bb0450] World Bank (2015). Ethiopia Poverty Assessment 2014.

[bb0455] Younger S.D., Khachatryan A., Inchauste G., Lustig N. (2016). Fiscal incidence in Armenia. The Distributional Impact of Fiscal Policy: Experience From Developing Countries.

[bb0460] Younger S.D., Osei-Assibey E., Oppong F. (2015). Fiscal incidence in Ghana. CEQ Working Paper 35.

[bb0465] Zheng B. (1994). Can a poverty index be both relative and absolute?. Econometrica.

[bb0470] Zheng B. (1997). Aggregate poverty measures. J. Econ. Surv..

