# Supplementary material for: Can a poverty-reducing and progressive tax and transfer system hurt the poor?
Source: J Dev Econ. 2016 Sep;122:63–75. doi: 10.1016/j.jdeveco.2016.04.001 (PMC4988485; doi:10.1016/j.jdeveco.2016.04.001)
Supplement: Supplementary file 1 — Supplementary material. [file mmc1.zip › documentation/POF0809/POF_questionnaire.pdf]

Ministério do Planejamento, Orçamento e Gestão

IBGE
Instituto Brasileiro de Geografia e Estatística

Diretoria de Pesquisas
Coordenação de Trabalho e Rendimento
Gerência da Pesquisa de Orçamentos Familiares

Pesquisa de Orçamentos Familiares 2008- 2009

POF 1 - Características do Domicílio e dos Moradores

01 IDENTIFICAÇÃO E CONTROLE DO QUESTIONÁRIO

01 IDENTIFICAÇÃO GERAL

UF	MUNICÍPIO	DISTRI-TO	SUBDIS-TRITO	SETOR	Nº DE ORDEM NA LISTAGEM

02 IDENTIFICAÇÃO POF

UF	SEQÜENCIAL	DV	CÓDIGO DO DOMICÍLIO	PERÍODO TEÓRICO	PERÍODO REAL

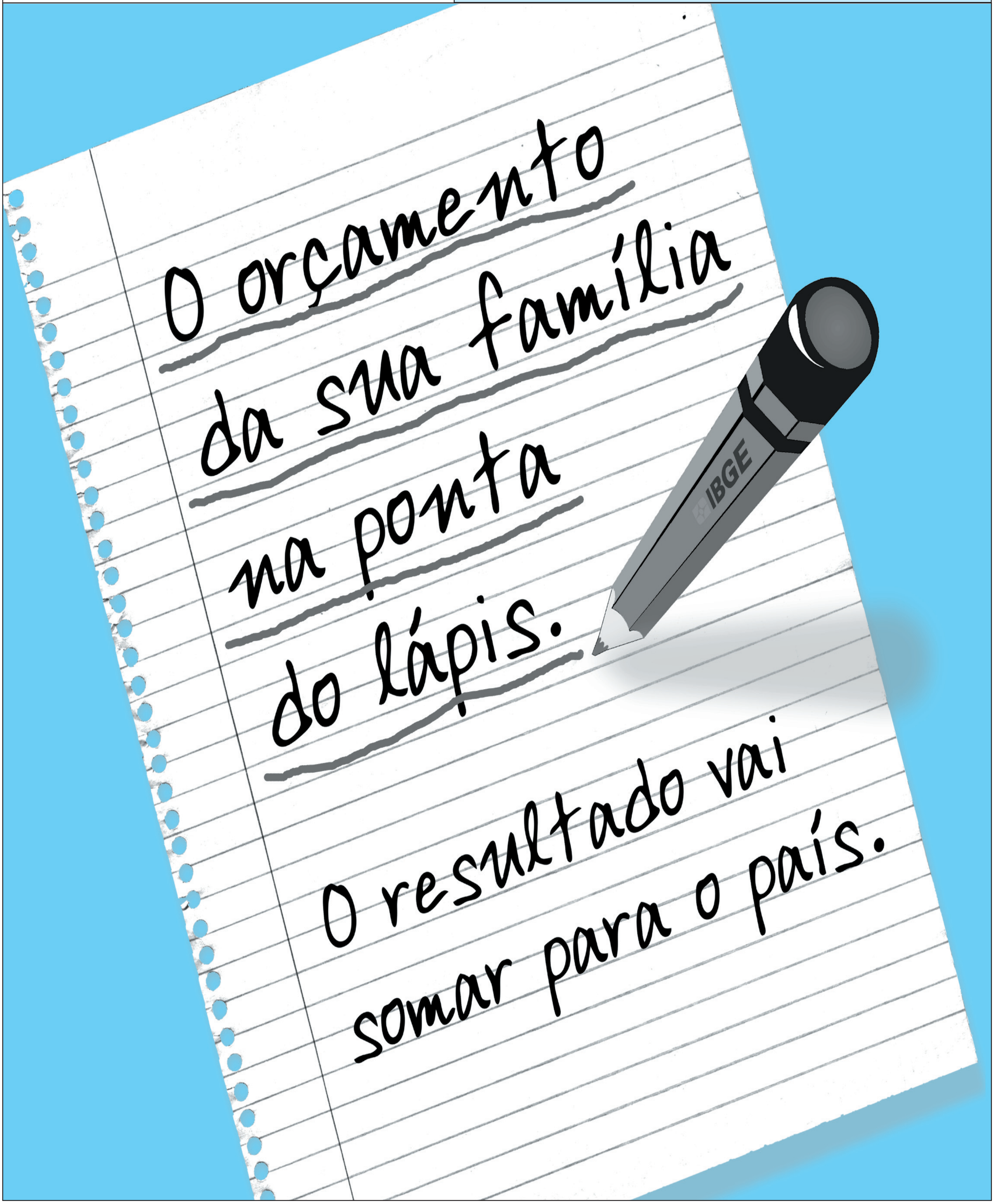

02 CARACTERÍSTICAS DO DOMICÍLIO

01 ESTE DOMICÍLIO ESTÁ LOCALIZADO EM OU PRÓXIMO A:

01 ☐ ESTRADA DE GRANDE CIRCULAÇÃO DE VEÍCULOS
02 ☐ ÁREA INDUSTRIAL
03 ☐ ESTRADA DE FERRO EM USO
04 ☐ PASSAGEM DE FIOS DE ALTA TENSÃO
05 ☐ GASODUTO OU OLEODUTO (REDE OU DEPÓSITO)
06 ☐ LIXÃO OU DEPÓSITO DE LIXO TÓXICO OU PERIGOSO
07 ☐ ESGOTO A CÉU ABERTO OU VALÃO
08 ☐ RIO, BAÍA, LAGO, AÇUDE OU REPRESA POLUÍDOS
09 ☐ ENCOSTA OU ÁREA SUJEITA A DESLIZAMENTO
10 ☐ NENHUMA SITUAÇÃO ACIMA

02 ESTE DOMICÍLIO É DO TIPO:

1 ☐ CASA 2 ☐ APARTAMENTO 3 ☐ CÔMODO

03 QUAL É O MATERIAL QUE PREDOMINA NA CONSTRUÇÃO DAS PAREDES EXTERNAS DESTE DOMICÍLIO?

1 ☐ ALVENARIA
2 ☐ MADEIRA PARA CONSTRUÇÃO
3 ☐ TAIPA NÃO-REVESTIDA
4 ☐ MADEIRA APROVEITADA
5 ☐ PALHA
6 ☐ OUTRO MATERIAL

04 QUAL É O MATERIAL QUE PREDOMINA NA COBERTURA (TELHADO) DESTE DOMICÍLIO?

1 ☐ TELHA QUALQUER
2 ☐ LAJE DE CONCRETO
3 ☐ MADEIRA PARA CONSTRUÇÃO
4 ☐ CHAPA METÁLICA
5 ☐ MADEIRA APROVEITADA
6 ☐ PALHA
7 ☐ OUTRO MATERIAL

05 QUAL É O MATERIAL QUE PREDOMINA NO PISO DESTE DOMICÍLIO?

1 ☐ CARPETE
2 ☐ CERÂMICA / LAJOTA / PEDRA
3 ☐ MADEIRA PARA CONSTRUÇÃO
4 ☐ CIMENTO
5 ☐ MADEIRA APROVEITADA
6 ☐ TERRA
7 ☐ OUTRO MATERIAL

06 QUANTOS CÔMODOS TEM ESTE DOMICÍLIO?

☐☐

07 QUANTOS CÔMODOS ESTÃO SERVINDO PERMANENTEMENTE DE DORMITÓRIO PARA OS MORADORES DESTE DOMICÍLIO?

☐☐

08 ESTE DOMICÍLIO TEM ÁGUA CANALIZADA PARA PELO MENOS UM CÔMODO?

1 ☐ SIM 2 ☐ NÃO

09 A ÁGUA UTILIZADA NESTE DOMICÍLIO É PROVENIENTE DE:

1 ☐ REDE GERAL DE DISTRIBUIÇÃO
2 ☐ POÇO OU NASCENTE
3 ☐ OUTRA PROVENIÊNCIA

10 QUANTOS BANHEIROS OU SANITÁRIOS TEM ESTE DOMICÍLIO PARA USO EXCLUSIVO?

☐☐

11 DE QUE FORMA É FEITO O ESCOADOURO DESTE(s) BANHEIRO(s) OU SANITÁRIO(s)?

1 ☐ REDE COLETORA DE ESGOTO OU PLUVIAL
2 ☐ FOSSA SÉPTICA
3 ☐ FOSSA RUDIMENTAR
4 ☐ VALA
5 ☐ DIRETO PARA O RIO, LAGO OU MAR
6 ☐ OUTRA FORMA
7 ☐ NÃO TEM

12 O LIXO DESTE DOMICÍLIO É SEPARADO EM MATERIAL BIODEGRADÁVEL (ALIMENTOS, PAPEL, PAPELÃO) E NÃO DEGRADÁVEL (PLÁSTICOS, VIDROS, METAIS)?

1 ☐ SIM → Siga quesito 13.
2 ☐ NÃO → Passe para o quesito 14.

13 A SEPARAÇÃO DO LIXO DESTE DOMICÍLIO TEM COMO FINALIDADE ATENDER À COLETA SELETIVA REALIZADA POR EMPRESA ESPECIALIZADA?

1 ☐ SIM
2 ☐ NÃO

14 O LIXO DESTE DOMICÍLIO É:

1 ☐ COLETADO DIRETAMENTE
2 ☐ COLETADO INDIRETAMENTE
3 ☐ QUEIMADO OU ENTERRADO NA PROPRIEDADE
4 ☐ JOGADO EM TERRENO BALDIO OU LOGRADOURO
5 ☐ JOGADO EM RIO, LAGO OU MAR
6 ☐ OUTRO DESTINO

02 CARACTERÍSTICAS DO DOMICÍLIO

15 QUAL(is) A(s) ORIGEM(ens) DA ENERGIA ELÉTRICA UTILIZADA(s) NESTE DOMICÍLIO?

1 ☐ REDE GERAL

2 ☐ PRÓPRIA — Se utiliza energia própria, siga quesito 16. Caso contrário, passe para o quesito 17.

3 ☐ NÃO DISPÕE

16 QUAL(is) FONTE(s) PRÓPRIA(s) DE ENERGIA É(são) USADA(s) NESTE DOMICÍLIO PARA GERAÇÃO DE ENERGIA ELÉTRICA?

1 ☐ DIESEL / GASOLINA / GÁS

2 ☐ SOLAR

3 ☐ EÓLICA

4 ☐ ÁGUA

5 ☐ BIODIESEL

6 ☐ SISTEMA MISTO

7 ☐ OUTRA FONTE

17 A ÁGUA CANALIZADA DESTE DOMICÍLIO É AQUECIDA POR QUAL(is) FONTE(s)?

1 ☐ ENERGIA ELÉTRICA

2 ☐ GÁS

3 ☐ ENERGIA SOLAR

4 ☐ LENHA / CARVÃO

5 ☐ OUTRA FORMA

6 ☐ NÃO TEM

18 O(s) FOGÃO(ões) DESTE DOMICÍLIO UTILIZA(m) COMO COMBUSTÍVEL:

1 ☐ GÁS BOTIJÃO/ENCANADO

2 ☐ LENHA

3 ☐ CARVÃO

4 ☐ ENERGIA ELÉTRICA

5 ☐ OUTRO COMBUSTÍVEL (ÓLEO, QUEROSENE, ETC.)

6 ☐ NÃO TEM

19 ESTE DOMICÍLIO É:

1 ☐ PRÓPRIO - JÁ PAGO

2 ☐ PRÓPRIO - AINDA PAGANDO

3 ☐ CEDIDO POR EMPREGADOR

4 ☐ CEDIDO DE OUTRA FORMA

5 ☐ OUTRA CONDIÇÃO

(1 a 5) → Siga quesito 20.

6 ☐ ALUGADO → Passe para o quesito 21.

20 CASO ESTE DOMICÍLIO FOSSE ALUGADO, QUAL SERIA O VALOR ESTIMADO DO ALUGUEL?

R$ ☐☐☐☐☐☐ , 0 0 → Passe para o quesito 23.

21 ESTE DOMICÍLIO É ALUGADO HÁ QUANTO TEMPO?

1 ☐ MENOS DE 12 MESES

2 ☐ DE 12 A 30 MESES

3 ☐ MAIS DE 30 MESES

22 ESTE CONTRATO DE ALUGUEL É:

1 ☐ VERBAL ("SÓ DE BOCA")

2 ☐ DOCUMENTADO ATRAVÉS DE IMOBILIÁRIA

3 ☐ DOCUMENTADO ATRAVÉS DE OUTRA FONTE

23 EXISTE PAVIMENTAÇÃO NA RUA ONDE SE LOCALIZA ESTE DOMICÍLIO?

1 ☐ SIM 2 ☐ NÃO

24 O SERVIÇO DE DISTRIBUIÇÃO DOS CORREIOS PARA ESTE DOMICÍLIO É REALIZADO:

1 ☐ POR ENTREGA DOMICILIÁRIA (PELO CARTEIRO)

2 ☐ EM CAIXA POSTAL COMUNITÁRIA - CPC

3 ☐ EM AGÊNCIA DE CORREIOS MAIS PRÓXIMA

4 ☐ POR ENTREGA EM OUTRO ENDEREÇO

5 ☐ POR OUTRA FORMA

OBSERVAÇÕES

_ _

_ _

_ _

_ _

03 RELAÇÃO DOS MORADORES

PERGUNTA INICIAL:

Quantas pessoas moram neste domicílio? |__|__|

CONDIÇÃO DOS MORADORES NA UNIDADE DE CONSUMO

Nº DE ORDEM	NOME	Nº DA UNIDADE DE CONSUMO	CONDIÇÃO NA UNIDADE DE CONSUMO

CÓDIGOS

CONDIÇÃO NA UNIDADE DE CONSUMO

1 - PESSOA DE REFERÊNCIA	5 - AGREGADO
2 - CÔNJUGE	6 - PENSIONISTA
3 - FILHO	7 - EMPREGADO DOMÉSTICO
4 - OUTRO PARENTE	8 - PARENTE DO EMPREGADO DOMÉSTICO

03 RELAÇÃO DOS MORADORES

CONDIÇÃO DOS MORADORES NA FAMÍLIA

Nº DE ORDEM	NOME	Nº DA FAMÍLIA	CONDIÇÃO NA FAMÍLIA

CÓDIGOS

CONDIÇÃO NA FAMÍLIA

1 - PESSOA DE REFERÊNCIA
2 - CÔNJUGE
3 - FILHO
4 - OUTRO PARENTE
5 - AGREGADO
6 - PENSIONISTA
7 - EMPREGADO DOMÉSTICO
8 - PARENTE DO EMPREGADO DOMÉSTICO

04 CARACTERÍSTICAS DO MORADOR

Nº DE ORDEM ☐☐

NOME ....

Nº DA UNIDADE DE CONSUMO: ☐

CONDIÇÃO NA UNIDADE DE CONSUMO:

1 ☐ PESSOA DE REFERÊNCIA
2 ☐ CÔNJUGE
3 ☐ FILHO
4 ☐ OUTRO PARENTE
5 ☐ AGREGADO
6 ☐ PENSIONISTA
7 ☐ EMPREGADO DOMÉSTICO
8 ☐ PARENTE DE EMPREGADO DOMÉSTICO

01 CONDIÇÃO DE PRESENÇA: 1 ☐ MORADOR PRESENTE → Siga quesito 02. 2 ☐ MORADOR AUSENTE → Passe para o quesito 04.

02 ALTURA / COMPRIMENTO INFORMADO EM CENTÍMETROS: ☐☐☐ , ☐

03 PESO INFORMADO EM QUILOGRAMAS: ☐☐☐ , ☐

04 DATA DE NASCIMENTO: ☐☐ DIA ☐☐ MÊS ☐☐☐☐ ANO

05 SEXO:

1 ☐ MASCULINO → Se idade menor que 4 anos, passe para o quesito 15. Caso contrário, passe para o quesito 18.

2 ☐ FEMININO → Se idade menor que 4 anos, passe para o quesito 15. Com idade de 4 a 9 anos, passe para o quesito 18. Com 10 anos ou mais de idade, siga quesito 06.

PARA MULHERES COM 10 ANOS OU MAIS DE IDADE

06 ______ ESTÁ GRÁVIDA? 1 ☐ SIM → Siga quesito 07. 2 ☐ NÃO → Passe para o quesito 08.

07 TEMPO DE GESTAÇÃO EM SEMANAS: ☐☐

08 ______ ESTÁ AMAMENTANDO? 1 ☐ SIM 2 ☐ NÃO

09 ______ TEVE ALGUM FILHO NASCIDO VIVO (OU SEJA, QUE APRESENTOU ALGUM SINAL DE VIDA AO NASCER)?

1 ☐ SIM → Siga quesito 10. 2 ☐ NÃO → Passe para o quesito 18.

10 QUANTOS FILHOS NASCIDOS VIVOS ______ TEVE? ☐☐ HOMENS ☐☐ MULHERES

11 DESTES FILHOS QUE ______ TEVE, QUANTOS ESTÃO VIVOS? ☐☐ HOMENS ☐☐ MULHERES

12 QUAL FOI O MÊS E O ANO DE NASCIMENTO DO ÚLTIMO FILHO NASCIDO VIVO QUE ______ TEVE? ☐☐ MÊS ☐☐☐☐ ANO

13 ESTE ÚLTIMO FILHO NASCIDO VIVO QUE ______ TEVE, AINDA ESTÁ VIVO?

1 ☐ SIM → Passe para o quesito 18. 2 ☐ NÃO → Siga quesito 14. 9 ☐ NÃO SABE → Passe para o quesito 18.

14 QUAL FOI O MÊS E O ANO DE FALECIMENTO DESTE ÚLTIMO FILHO NASCIDO VIVO QUE ______ TEVE? ☐☐ MÊS ☐☐☐☐ ANO

PARA AS CRIANÇAS MENORES QUE 4 ANOS (ENTRE 0 E 47 MESES)

15 ______ RECEBE LEITE MATERNO ATUALMENTE?

1 ☐ SIM → Siga quesito 16.
2 ☐ NÃO, MAS JÁ RECEBEU → Passe para o quesito 17.
3 ☐ NUNCA RECEBEU → Passe para o quesito 18.

16 ALÉM DO LEITE MATERNO, ______ RECEBE OUTRO TIPO DE ALIMENTO ATUALMENTE?

1 ☐ SIM → Siga quesito 17.
2 ☐ NÃO
9 ☐ NÃO SABE
(2 e 9) → Passe para o quesito 18.

17 POR QUANTOS MESES ______ RECEBEU EXCLUSIVAMENTE O LEITE MATERNO COMO FONTE DE ALIMENTAÇÃO? ☐☐

04 CARACTERÍSTICAS DO MORADOR

PARA TODAS AS PESSOAS

18 ______ SABE LER E ESCREVER?

1 ☐ SIM
2 ☐ NÃO

19 ______ FREQÜENTA ESCOLA OU CRECHE?

1 ☐ SIM, REDE PARTICULAR → Siga quesito 20.
2 ☐ SIM, REDE PÚBLICA → Siga quesito 20.
3 ☐ NÃO, MAS JÁ FREQÜENTOU → Passe para o quesito 25.
4 ☐ NUNCA FREQÜENTOU → Passe para o quesito 29.
9 ☐ NÃO SABE → Passe para o quesito 29.

PARA AS PESSOAS QUE FREQÜENTAM ESCOLA

20 QUAL É O CURSO QUE ______ FREQÜENTA?

01 ☐ CRECHE → Passe para o quesito 22.
02 ☐ PRÉ-ESCOLAR → Passe para o quesito 22.
03 ☐ CLASSE DE ALFABETIZAÇÃO DE CRIANÇAS → Passe para o quesito 22.
04 ☐ ALFABETIZAÇÃO DE ADULTOS → Passe para o quesito 22.
05 ☐ REGULAR DO ENSINO FUNDAMENTAL → Siga quesito 21.
06 ☐ EDUCAÇÃO DE JOVENS E ADULTOS OU SUPLETIVO DO ENSINO FUNDAMENTAL OU 1º GRAU → Passe para o quesito 22.
07 ☐ REGULAR DO ENSINO MÉDIO → Passe para o quesito 22.
08 ☐ EDUCAÇÃO DE JOVENS E ADULTOS OU SUPLETIVO DO ENSINO MÉDIO OU 2º GRAU → Passe para o quesito 22.
09 ☐ TECNOLÓGICO SUPERIOR → Passe para o quesito 22.
10 ☐ PRÉ-VESTIBULAR → Passe para o quesito 22.
11 ☐ SUPERIOR - GRADUAÇÃO → Passe para o quesito 22.
12 ☐ ESPECIALIZAÇÃO SUPERIOR → Passe para o quesito 22.
13 ☐ MESTRADO OU DOUTORADO → Passe para o quesito 22.
99 ☐ NÃO SABE → Passe para o quesito 22.

21 A DURAÇÃO DESTE CURSO DE ENSINO FUNDAMENTAL OU 1º GRAU É DE:

1 ☐ 8 ANOS
2 ☐ 9 ANOS
9 ☐ NÃO SABE

22 QUAL É A SÉRIE QUE ______ FREQÜENTA?

01 ☐ PRIMEIRA
02 ☐ SEGUNDA
03 ☐ TERCEIRA
04 ☐ QUARTA
05 ☐ QUINTA
06 ☐ SEXTA
07 ☐ SÉTIMA
08 ☐ OITAVA
09 ☐ NONA
10 ☐ CURSO NÃO-SERIADO
99 ☐ NÃO SABE

PARA AS PESSOAS MENORES DE 10 ANOS QUE FREQÜENTAM ESCOLA

23 COM QUE FREQÜÊNCIA ______ COSTUMA SE ALIMENTAR DIARIAMENTE NA ESCOLA?

1 ☐ UMA VEZ → Siga quesito 24.
2 ☐ DUAS VEZES → Siga quesito 24.
3 ☐ TRÊS VEZES → Siga quesito 24.
4 ☐ QUATRO VEZES OU MAIS → Siga quesito 24.
5 ☐ NENHUMA VEZ → Passe para o quesito 29.
9 ☐ NÃO SABE → Siga quesito 24.

24 OS ALIMENTOS QUE ______ CONSOME NA ESCOLA COSTUMAM SER:

1 ☐ FORNECIDOS PELA ESCOLA (MERENDA, ALMOÇO, ETC.)
2 ☐ LEVADOS DE CASA
3 ☐ COMPRADOS NA CANTINA
4 ☐ OUTROS

04 CARACTERÍSTICAS DO MORADOR

PARA AS PESSOAS QUE NÃO FREQÜENTAM ESCOLA, MAS JÁ FREQÜENTARAM

25 QUAL FOI O CURSO MAIS ELEVADO QUE ______ FREQÜENTOU?

01 ☐ CRECHE
02 ☐ PRÉ-ESCOLAR
03 ☐ CLASSE DE ALFABETIZAÇÃO DE CRIANÇAS
04 ☐ ALFABETIZAÇÃO DE ADULTOS
05 ☐ ANTIGO PRIMÁRIO
06 ☐ ANTIGO GINÁSIO
07 ☐ ANTIGO CLÁSSICO, CIENTÍFICO, ETC.
→ Passe para o quesito 27.

08 ☐ REGULAR ENSINO FUNDAMENTAL → Siga quesito 26.

09 ☐ EDUCAÇÃO DE JOVENS E ADULTOS OU SUPLETIVO DO ENSINO FUNDAMENTAL OU 1º GRAU
10 ☐ REGULAR DO ENSINO MÉDIO
11 ☐ EDUCAÇÃO DE JOVENS E ADULTOS OU SUPLETIVO DO ENSINO MÉDIO OU 2º GRAU
12 ☐ TECNOLÓGICO SUPERIOR
13 ☐ PRÉ-VESTIBULAR
14 ☐ SUPERIOR - GRADUAÇÃO
15 ☐ ESPECIALIZAÇÃO SUPERIOR
16 ☐ MESTRADO OU DOUTORADO
99 ☐ NÃO SABE
→ Passe para o quesito 27.

26 A DURAÇÃO DESTE CURSO DE ENSINO FUNDAMENTAL OU 1º GRAU ERA DE:

1 ☐ 8 ANOS 2 ☐ 9 ANOS 9 ☐ NÃO SABE

27 QUAL FOI A ÚLTIMA SÉRIE QUE ______ CONCLUIU COM APROVAÇÃO?

01 ☐ PRIMEIRA
02 ☐ SEGUNDA
03 ☐ TERCEIRA
04 ☐ QUARTA
05 ☐ QUINTA
06 ☐ SEXTA
07 ☐ SÉTIMA
08 ☐ OITAVA
09 ☐ NONA
10 ☐ CURSO NÃO-SERIADO
11 ☐ NENHUMA
99 ☐ NÃO SABE

28 ______ CONCLUIU O CURSO MAIS ELEVADO QUE FREQÜENTOU? 1 ☐ SIM 2 ☐ NÃO 9 ☐ NÃO SABE

PARA TODAS AS PESSOAS

29 A COR OU RAÇA DO(A) ______ É:

1 ☐ BRANCA 2 ☐ PRETA 3 ☐ AMARELA 4 ☐ PARDA 5 ☐ INDÍGENA 9 ☐ NÃO SABE

30 A RELIGIÃO OU CULTO DO(A) ______ É:

....

31 ______TEM REGISTRO DE NASCIMENTO EM CARTÓRIO?

1 ☐ SIM → Se idade menor que 3 anos, passe para o quesito 33. Caso contrário, passe para o quesito 34.
2 ☐ NÃO → Siga quesito 32.
9 ☐ NÃO SABE → Se idade menor que 3 anos, passe para o quesito 33. Caso contrário, passe para o quesito 34.

32 QUAL O MOTIVO DO(A) ______ NÃO TER O REGISTRO DE NASCIMENTO?

1 ☐ NÃO ACHA IMPORTANTE
2 ☐ PAI E / OU MÃE NÃO TEM DOCUMENTOS PRÓPRIOS
3 ☐ O CARTÓRIO É DISTANTE OU DE DIFÍCIL ACESSO
4 ☐ NÃO TEM DINHEIRO PARA SE LOCOMOVER ATÉ O CARTÓRIO
5 ☐ OUTROS MOTIVOS

04 CARACTERÍSTICAS DO MORADOR

PARA CRIANÇAS MENORES DE 3 ANOS (ENTRE 0 E 35 MESES)

33 COMPRIMENTO EM CENTÍMETROS: ☐☐☐ , ☐

PARA PESSOAS COM 2 ANOS OU MAIS (COM 24 MESES OU MAIS)

34 ALTURA EM CENTÍMETROS: ☐☐☐ , ☐

PARA TODAS AS PESSOAS

35 POSIÇÃO AO SER MEDIDO O COMPRIMENTO OU A ALTURA:

1 ☐ SÓ DEITADO(A)

2 ☐ DEITADO(A) E EM PÉ

3 ☐ SÓ EM PÉ

4 ☐ NENHUMA (DEVIDO A AUSÊNCIA, DOENÇA, RECUSA, ETC.)

PARA PESSOAS COM 2 ANOS OU MAIS (COM 24 MESES OU MAIS)

36 PESO EM QUILOGRAMAS: ☐☐☐ , ☐

PARA CRIANÇAS MENORES DE 2 ANOS (ENTRE 0 E 23 MESES)

37 PESO DO ADULTO COM A CRIANÇA NO COLO EM QUILOGRAMAS: ☐☐☐ , ☐

PESO DO ADULTO SOZINHO EM QUILOGRAMAS: ☐☐☐ , ☐

NOME DO ADULTO QUE SEGUROU A CRIANÇA NO COLO: ________

PARA TODAS AS PESSOAS

38 ______ TEM PLANO OU SEGURO-SAÚDE?

1 ☐ SIM → Siga quesito 39.

2 ☐ NÃO
9 ☐ NÃO SABE → Se idade maior ou igual a 10 anos, passe para o quesito 41. Caso contrário, encerre.

39 ______ É O(A) TITULAR DO PLANO OU SEGURO-SAÚDE?

1 ☐ SIM → Siga quesito 40.

2 ☐ NÃO
9 ☐ NÃO SABE → Se idade maior ou igual a 10 anos, passe para o quesito 41. Caso contrário, encerre.

40 NÚMERO DE DEPENDENTES DO(A) TITULAR DO PLANO OU SEGURO-SAÚDE: ☐☐

PARA PESSOAS COM 10 ANOS OU MAIS DE IDADE

41 ______ É UMA UNIDADE DE ORÇAMENTO TRABALHO E/OU RENDIMENTO? 1 ☐ SIM 2 ☐ NÃO 3 ☐ RECUSA 4 ☐ OUTRO

42 ______ É UMA UNIDADE DE ORÇAMENTO DESPESA?

1 ☐ SIM → Siga quesito 43.

2 ☐ NÃO → Passe para o quesito 47.

3 ☐ RECUSA
4 ☐ OUTRO → Siga quesito 43.

43 ______ TEM CARTÃO DE CRÉDITO?

1 ☐ SIM → Siga quesito 44.

2 ☐ NÃO
9 ☐ NÃO SABE → Passe para o quesito 45.

44 ______ É O(A) TITULAR DO CARTÃO DE CRÉDITO? 1 ☐ SIM 2 ☐ NÃO 9 ☐ NÃO SABE

45 ______ TEM CHEQUE ESPECIAL?

1 ☐ SIM → Siga quesito 46.

2 ☐ NÃO
9 ☐ NÃO SABE → Passe para o quesito 47.

46 ______ É O(A) TITULAR DA CONTA CORRENTE? 1 ☐ SIM 2 ☐ NÃO 9 ☐ NÃO SABE

47 ______ VAI RESPONDER O BLOCO DE CONSUMO ALIMENTAR? 1 ☐ SIM 2 ☐ NÃO 3 ☐ RECUSA 4 ☐ OUTRO

04 CARACTERÍSTICAS DO MORADOR

Nº DE ORDEM ☐☐ .... NOME

Nº DA UNIDADE DE CONSUMO: ☐

CONDIÇÃO NA UNIDADE DE CONSUMO:

1 ☐ PESSOA DE REFERÊNCIA
3 ☐ FILHO
5 ☐ AGREGADO
7 ☐ EMPREGADO DOMÉSTICO
2 ☐ CÔNJUGE
4 ☐ OUTRO PARENTE
6 ☐ PENSIONISTA
8 ☐ PARENTE DE EMPREGADO DOMÉSTICO

01 CONDIÇÃO DE PRESENÇA: 1 ☐ MORADOR PRESENTE → Siga quesito 02. 2 ☐ MORADOR AUSENTE → Passe para o quesito 04.

02 ALTURA / COMPRIMENTO INFORMADO EM CENTÍMETROS: ☐☐☐ , ☐

03 PESO INFORMADO EM QUILOGRAMAS: ☐☐☐ , ☐

04 DATA DE NASCIMENTO: ☐☐ DIA ☐☐ MÊS ☐☐☐☐ ANO

05 SEXO:

1 ☐ MASCULINO → Se idade menor que 4 anos, passe para o quesito 15. Caso contrário, passe para o quesito 18.

2 ☐ FEMININO → Se idade menor que 4 anos, passe para o quesito 15. Com idade de 4 a 9 anos, passe para o quesito 18. Com 10 anos ou mais de idade, siga quesito 06.

PARA MULHERES COM 10 ANOS OU MAIS DE IDADE

06 ______ ESTÁ GRÁVIDA? 1 ☐ SIM → Siga quesito 07. 2 ☐ NÃO → Passe para o quesito 08.

07 TEMPO DE GESTAÇÃO EM SEMANAS: ☐☐

08 ______ ESTÁ AMAMENTANDO? 1 ☐ SIM 2 ☐ NÃO

09 ______ TEVE ALGUM FILHO NASCIDO VIVO (OU SEJA, QUE APRESENTOU ALGUM SINAL DE VIDA AO NASCER)?

1 ☐ SIM → Siga quesito 10. 2 ☐ NÃO → Passe para o quesito 18.

10 QUANTOS FILHOS NASCIDOS VIVOS ______ TEVE? ☐☐ HOMENS ☐☐ MULHERES

11 DESTES FILHOS QUE ______ TEVE, QUANTOS ESTÃO VIVOS? ☐☐ HOMENS ☐☐ MULHERES

12 QUAL FOI O MÊS E O ANO DE NASCIMENTO DO ÚLTIMO FILHO NASCIDO VIVO QUE ______ TEVE? ☐☐ MÊS ☐☐☐☐ ANO

13 ESTE ÚLTIMO FILHO NASCIDO VIVO QUE ______ TEVE, AINDA ESTÁ VIVO?

1 ☐ SIM → Passe para o quesito 18. 2 ☐ NÃO → Siga quesito 14. 9 ☐ NÃO SABE → Passe para o quesito 18.

14 QUAL FOI O MÊS E O ANO DE FALECIMENTO DESTE ÚLTIMO FILHO NASCIDO VIVO QUE ______ TEVE? ☐☐ MÊS ☐☐☐☐ ANO

PARA AS CRIANÇAS MENORES QUE 4 ANOS (ENTRE 0 E 47 MESES)

15 ______ RECEBE LEITE MATERNO ATUALMENTE?

1 ☐ SIM → Siga quesito 16.
2 ☐ NÃO, MAS JÁ RECEBEU → Passe para o quesito 17.
3 ☐ NUNCA RECEBEU → Passe para o quesito 18.

16 ALÉM DO LEITE MATERNO, ______ RECEBE OUTRO TIPO DE ALIMENTO ATUALMENTE?

1 ☐ SIM → Siga quesito 17.
2 ☐ NÃO
9 ☐ NÃO SABE
(2 e 9) → Passe para o quesito 18.

17 POR QUANTOS MESES ______ RECEBEU EXCLUSIVAMENTE O LEITE MATERNO COMO FONTE DE ALIMENTAÇÃO? ☐☐

04 CARACTERÍSTICAS DO MORADOR

PARA TODAS AS PESSOAS

18 ________ SABE LER E ESCREVER?

1 ☐ SIM 2 ☐ NÃO

19 ________ FREQÜENTA ESCOLA OU CRECHE?

1 ☐ SIM, REDE PARTICULAR
2 ☐ SIM, REDE PÚBLICA
→ Siga quesito 20.

3 ☐ NÃO, MAS JÁ FREQÜENTOU → Passe para o quesito 25.

4 ☐ NUNCA FREQÜENTOU
9 ☐ NÃO SABE
→ Passe para o quesito 29.

PARA AS PESSOAS QUE FREQÜENTAM ESCOLA

20 QUAL É O CURSO QUE ________ FREQÜENTA?

01 ☐ CRECHE
02 ☐ PRÉ-ESCOLAR
03 ☐ CLASSE DE ALFABETIZAÇÃO DE CRIANÇAS
04 ☐ ALFABETIZAÇÃO DE ADULTOS
→ Passe para o quesito 22.

05 ☐ REGULAR DO ENSINO FUNDAMENTAL → Siga quesito 21.

06 ☐ EDUCAÇÃO DE JOVENS E ADULTOS OU SUPLETIVO DO ENSINO FUNDAMENTAL OU 1º GRAU
07 ☐ REGULAR DO ENSINO MÉDIO
08 ☐ EDUCAÇÃO DE JOVENS E ADULTOS OU SUPLETIVO DO ENSINO MÉDIO OU 2º GRAU
09 ☐ TECNOLÓGICO SUPERIOR
10 ☐ PRÉ-VESTIBULAR
11 ☐ SUPERIOR - GRADUAÇÃO
12 ☐ ESPECIALIZAÇÃO SUPERIOR
13 ☐ MESTRADO OU DOUTORADO
99 ☐ NÃO SABE
→ Passe para o quesito 22.

21 A DURAÇÃO DESTE CURSO DE ENSINO FUNDAMENTAL OU 1º GRAU É DE:

1 ☐ 8 ANOS 2 ☐ 9 ANOS 9 ☐ NÃO SABE

22 QUAL É A SÉRIE QUE ________ FREQÜENTA?

01 ☐ PRIMEIRA
02 ☐ SEGUNDA
03 ☐ TERCEIRA
04 ☐ QUARTA
05 ☐ QUINTA
06 ☐ SEXTA
07 ☐ SÉTIMA
08 ☐ OITAVA
09 ☐ NONA
10 ☐ CURSO NÃO-SERIADO
99 ☐ NÃO SABE

PARA AS PESSOAS MENORES DE 10 ANOS QUE FREQÜENTAM ESCOLA

23 COM QUE FREQÜÊNCIA ________ COSTUMA SE ALIMENTAR DIARIAMENTE NA ESCOLA?

1 ☐ UMA VEZ
2 ☐ DUAS VEZES
3 ☐ TRÊS VEZES
4 ☐ QUATRO VEZES OU MAIS
→ Siga quesito 24.

5 ☐ NENHUMA VEZ → Passe para o quesito 29.

9 ☐ NÃO SABE → Siga quesito 24.

24 OS ALIMENTOS QUE ________ CONSOME NA ESCOLA COSTUMAM SER:

1 ☐ FORNECIDOS PELA ESCOLA (MERENDA, ALMOÇO, ETC.)
2 ☐ LEVADOS DE CASA
3 ☐ COMPRADOS NA CANTINA
4 ☐ OUTROS

04 CARACTERÍSTICAS DO MORADOR

PARA AS PESSOAS QUE NÃO FREQÜENTAM ESCOLA, MAS JÁ FREQÜENTARAM

25 QUAL FOI O CURSO MAIS ELEVADO QUE _______ FREQÜENTOU?

01 ☐ CRECHE
02 ☐ PRÉ-ESCOLAR
03 ☐ CLASSE DE ALFABETIZAÇÃO DE CRIANÇAS
04 ☐ ALFABETIZAÇÃO DE ADULTOS
05 ☐ ANTIGO PRIMÁRIO
06 ☐ ANTIGO GINÁSIO
07 ☐ ANTIGO CLÁSSICO, CIENTÍFICO, ETC.
→ Passe para o quesito 27.

08 ☐ REGULAR ENSINO FUNDAMENTAL → Siga quesito 26.

09 ☐ EDUCAÇÃO DE JOVENS E ADULTOS OU SUPLETIVO DO ENSINO FUNDAMENTAL OU 1º GRAU
10 ☐ REGULAR DO ENSINO MÉDIO
11 ☐ EDUCAÇÃO DE JOVENS E ADULTOS OU SUPLETIVO DO ENSINO MÉDIO OU 2º GRAU
12 ☐ TECNOLÓGICO SUPERIOR
13 ☐ PRÉ-VESTIBULAR
14 ☐ SUPERIOR - GRADUAÇÃO
15 ☐ ESPECIALIZAÇÃO SUPERIOR
16 ☐ MESTRADO OU DOUTORADO
99 ☐ NÃO SABE
→ Passe para o quesito 27.

26 A DURAÇÃO DESTE CURSO DE ENSINO FUNDAMENTAL OU 1º GRAU ERA DE:

1 ☐ 8 ANOS   2 ☐ 9 ANOS   9 ☐ NÃO SABE

27 QUAL FOI A ÚLTIMA SÉRIE QUE _______ CONCLUIU COM APROVAÇÃO?

01 ☐ PRIMEIRA
02 ☐ SEGUNDA
03 ☐ TERCEIRA
04 ☐ QUARTA
05 ☐ QUINTA
06 ☐ SEXTA
07 ☐ SÉTIMA
08 ☐ OITAVA
09 ☐ NONA
10 ☐ CURSO NÃO-SERIADO
11 ☐ NENHUMA
99 ☐ NÃO SABE

28 _______ CONCLUIU O CURSO MAIS ELEVADO QUE FREQÜENTOU?   1 ☐ SIM   2 ☐ NÃO   9 ☐ NÃO SABE

PARA TODAS AS PESSOAS

29 A COR OU RAÇA DO(A) _______ É:

1 ☐ BRANCA   2 ☐ PRETA   3 ☐ AMARELA   4 ☐ PARDA   5 ☐ INDÍGENA   9 ☐ NÃO SABE

30 A RELIGIÃO OU CULTO DO(A) _______ É:

....

31 _______ TEM REGISTRO DE NASCIMENTO EM CARTÓRIO?

1 ☐ SIM → Se idade menor que 3 anos, passe para o quesito 33. Caso contrário, passe para o quesito 34.
2 ☐ NÃO → Siga quesito 32.
9 ☐ NÃO SABE → Se idade menor que 3 anos, passe para o quesito 33. Caso contrário, passe para o quesito 34.

32 QUAL O MOTIVO DO(A) _______ NÃO TER O REGISTRO DE NASCIMENTO?

1 ☐ NÃO ACHA IMPORTANTE
2 ☐ PAI E / OU MÃE NÃO TEM DOCUMENTOS PRÓPRIOS
3 ☐ O CARTÓRIO É DISTANTE OU DE DIFÍCIL ACESSO
4 ☐ NÃO TEM DINHEIRO PARA SE LOCOMOVER ATÉ O CARTÓRIO
5 ☐ OUTROS MOTIVOS

04 CARACTERÍSTICAS DO MORADOR

PARA CRIANÇAS MENORES DE 3 ANOS (ENTRE 0 E 35 MESES)

33 COMPRIMENTO EM CENTÍMETROS: ☐☐☐ , ☐

PARA PESSOAS COM 2 ANOS OU MAIS (COM 24 MESES OU MAIS)

34 ALTURA EM CENTÍMETROS: ☐☐☐ , ☐

PARA TODAS AS PESSOAS

35 POSIÇÃO AO SER MEDIDO O COMPRIMENTO OU A ALTURA:

1 ☐ SÓ DEITADO(A)

2 ☐ DEITADO(A) E EM PÉ

3 ☐ SÓ EM PÉ

4 ☐ NENHUMA (DEVIDO A AUSÊNCIA, DOENÇA, RECUSA, ETC.)

PARA PESSOAS COM 2 ANOS OU MAIS (COM 24 MESES OU MAIS)

36 PESO EM QUILOGRAMAS: ☐☐☐ , ☐

PARA CRIANÇAS MENORES DE 2 ANOS (ENTRE 0 E 23 MESES)

37 PESO DO ADULTO COM A CRIANÇA NO COLO EM QUILOGRAMAS: ☐☐☐ , ☐

PESO DO ADULTO SOZINHO EM QUILOGRAMAS: ☐☐☐ , ☐

NOME DO ADULTO QUE SEGUROU A CRIANÇA NO COLO: ____________

PARA TODAS AS PESSOAS

38 ______ TEM PLANO OU SEGURO-SAÚDE?

1 ☐ SIM → Siga quesito 39.

2 ☐ NÃO
9 ☐ NÃO SABE
→ Se idade maior ou igual a 10 anos, passe para o quesito 41. Caso contrário, encerre.

39 ______ É O(A) TITULAR DO PLANO OU SEGURO-SAÚDE?

1 ☐ SIM → Siga quesito 40.

2 ☐ NÃO
9 ☐ NÃO SABE
→ Se idade maior ou igual a 10 anos, passe para o quesito 41. Caso contrário, encerre.

40 NÚMERO DE DEPENDENTES DO(A) TITULAR DO PLANO OU SEGURO-SAÚDE: ☐☐

PARA PESSOAS COM 10 ANOS OU MAIS DE IDADE

41 ______ É UMA UNIDADE DE ORÇAMENTO TRABALHO E/OU RENDIMENTO? 1 ☐ SIM 2 ☐ NÃO 3 ☐ RECUSA 4 ☐ OUTRO

42 ______ É UMA UNIDADE DE ORÇAMENTO DESPESA?

1 ☐ SIM → Siga quesito 43.

2 ☐ NÃO → Passe para o quesito 47.

3 ☐ RECUSA
4 ☐ OUTRO
→ Siga quesito 43.

43 ______ TEM CARTÃO DE CRÉDITO?

1 ☐ SIM → Siga quesito 44.

2 ☐ NÃO
9 ☐ NÃO SABE
→ Passe para o quesito 45.

44 ______ É O(A) TITULAR DO CARTÃO DE CRÉDITO? 1 ☐ SIM 2 ☐ NÃO 9 ☐ NÃO SABE

45 ______ TEM CHEQUE ESPECIAL?

1 ☐ SIM → Siga quesito 46.

2 ☐ NÃO
9 ☐ NÃO SABE
→ Passe para o quesito 47.

46 ______ É O(A) TITULAR DA CONTA CORRENTE? 1 ☐ SIM 2 ☐ NÃO 9 ☐ NÃO SABE

47 ______ VAI RESPONDER O BLOCO DE CONSUMO ALIMENTAR? 1 ☐ SIM 2 ☐ NÃO 3 ☐ RECUSA 4 ☐ OUTRO

04 CARACTERÍSTICAS DO MORADOR

Nº DE ORDEM | NOME

Nº DA UNIDADE DE CONSUMO:

CONDIÇÃO NA UNIDADE DE CONSUMO:

1 PESSOA DE REFERÊNCIA
3 FILHO
5 AGREGADO
7 EMPREGADO DOMÉSTICO
2 CÔNJUGE
4 OUTRO PARENTE
6 PENSIONISTA
8 PARENTE DE EMPREGADO DOMÉSTICO

01 CONDIÇÃO DE PRESENÇA: 1 MORADOR PRESENTE → Siga quesito 02. 2 MORADOR AUSENTE → Passe para o quesito 04.

02 ALTURA / COMPRIMENTO INFORMADO EM CENTÍMETROS: ___ , _

03 PESO INFORMADO EM QUILOGRAMAS: ___ , _

04 DATA DE NASCIMENTO: DIA | MÊS | ANO

05 SEXO:

1 MASCULINO → Se idade menor que 4 anos, passe para o quesito 15. Caso contrário, passe para o quesito 18.

2 FEMININO → Se idade menor que 4 anos, passe para o quesito 15. Com idade de 4 a 9 anos, passe para o quesito 18. Com 10 anos ou mais de idade, siga quesito 06.

PARA MULHERES COM 10 ANOS OU MAIS DE IDADE

06 _______ ESTÁ GRÁVIDA? 1 SIM → Siga quesito 07. 2 NÃO → Passe para o quesito 08.

07 TEMPO DE GESTAÇÃO EM SEMANAS:

08 _______ ESTÁ AMAMENTANDO? 1 SIM 2 NÃO

09 _______ TEVE ALGUM FILHO NASCIDO VIVO (OU SEJA, QUE APRESENTOU ALGUM SINAL DE VIDA AO NASCER)?

1 SIM → Siga quesito 10. 2 NÃO → Passe para o quesito 18.

10 QUANTOS FILHOS NASCIDOS VIVOS _______ TEVE? HOMENS MULHERES

11 DESTES FILHOS QUE _______ TEVE, QUANTOS ESTÃO VIVOS? HOMENS MULHERES

12 QUAL FOI O MÊS E O ANO DE NASCIMENTO DO ÚLTIMO FILHO NASCIDO VIVO QUE _______ TEVE? MÊS ANO

13 ESTE ÚLTIMO FILHO NASCIDO VIVO QUE _______ TEVE, AINDA ESTÁ VIVO?

1 SIM → Passe para o quesito 18. 2 NÃO → Siga quesito 14. 9 NÃO SABE → Passe para o quesito 18.

14 QUAL FOI O MÊS E O ANO DE FALECIMENTO DESTE ÚLTIMO FILHO NASCIDO VIVO QUE _______ TEVE? MÊS ANO

PARA AS CRIANÇAS MENORES QUE 4 ANOS (ENTRE 0 E 47 MESES)

15 _______ RECEBE LEITE MATERNO ATUALMENTE?

1 SIM → Siga quesito 16.
2 NÃO, MAS JÁ RECEBEU → Passe para o quesito 17.
3 NUNCA RECEBEU → Passe para o quesito 18.

16 ALÉM DO LEITE MATERNO, _______ RECEBE OUTRO TIPO DE ALIMENTO ATUALMENTE?

1 SIM → Siga quesito 17.
2 NÃO
9 NÃO SABE
(2, 9) → Passe para o quesito 18.

17 POR QUANTOS MESES _______ RECEBEU EXCLUSIVAMENTE O LEITE MATERNO COMO FONTE DE ALIMENTAÇÃO?

04	CARACTERÍSTICAS DO MORADOR

PARA TODAS AS PESSOAS

18 ________ SABE LER E ESCREVER?

1 ☐ SIM 2 ☐ NÃO

19 ________ FREQÜENTA ESCOLA OU CRECHE?

1 ☐ SIM, REDE PARTICULAR → Siga quesito 20.
2 ☐ SIM, REDE PÚBLICA → Siga quesito 20.
3 ☐ NÃO, MAS JÁ FREQÜENTOU → Passe para o quesito 25.
4 ☐ NUNCA FREQÜENTOU → Passe para o quesito 29.
9 ☐ NÃO SABE → Passe para o quesito 29.

PARA AS PESSOAS QUE FREQÜENTAM ESCOLA

20 QUAL É O CURSO QUE ________ FREQÜENTA?

01 ☐ CRECHE
02 ☐ PRÉ-ESCOLAR
03 ☐ CLASSE DE ALFABETIZAÇÃO DE CRIANÇAS
04 ☐ ALFABETIZAÇÃO DE ADULTOS
→ Passe para o quesito 22.

05 ☐ REGULAR DO ENSINO FUNDAMENTAL → Siga quesito 21.

06 ☐ EDUCAÇÃO DE JOVENS E ADULTOS OU SUPLETIVO DO ENSINO FUNDAMENTAL OU 1º GRAU
07 ☐ REGULAR DO ENSINO MÉDIO
08 ☐ EDUCAÇÃO DE JOVENS E ADULTOS OU SUPLETIVO DO ENSINO MÉDIO OU 2º GRAU
09 ☐ TECNOLÓGICO SUPERIOR
10 ☐ PRÉ-VESTIBULAR
11 ☐ SUPERIOR - GRADUAÇÃO
12 ☐ ESPECIALIZAÇÃO SUPERIOR
13 ☐ MESTRADO OU DOUTORADO
99 ☐ NÃO SABE
→ Passe para o quesito 22.

21 A DURAÇÃO DESTE CURSO DE ENSINO FUNDAMENTAL OU 1º GRAU É DE:

1 ☐ 8 ANOS 2 ☐ 9 ANOS 9 ☐ NÃO SABE

22 QUAL É A SÉRIE QUE ________ FREQÜENTA?

01 ☐ PRIMEIRA
02 ☐ SEGUNDA
03 ☐ TERCEIRA
04 ☐ QUARTA
05 ☐ QUINTA
06 ☐ SEXTA
07 ☐ SÉTIMA
08 ☐ OITAVA
09 ☐ NONA
10 ☐ CURSO NÃO-SERIADO
99 ☐ NÃO SABE

PARA AS PESSOAS MENORES DE 10 ANOS QUE FREQÜENTAM ESCOLA

23 COM QUE FREQÜÊNCIA ________ COSTUMA SE ALIMENTAR DIARIAMENTE NA ESCOLA?

1 ☐ UMA VEZ
2 ☐ DUAS VEZES
3 ☐ TRÊS VEZES
4 ☐ QUATRO VEZES OU MAIS
→ Siga quesito 24.

5 ☐ NENHUMA VEZ → Passe para o quesito 29.
9 ☐ NÃO SABE → Siga quesito 24.

24 OS ALIMENTOS QUE ________ CONSOME NA ESCOLA COSTUMAM SER:

1 ☐ FORNECIDOS PELA ESCOLA (MERENDA, ALMOÇO, ETC.)
2 ☐ LEVADOS DE CASA
3 ☐ COMPRADOS NA CANTINA
4 ☐ OUTROS

04 CARACTERÍSTICAS DO MORADOR

PARA AS PESSOAS QUE NÃO FREQÜENTAM ESCOLA, MAS JÁ FREQÜENTARAM

25 QUAL FOI O CURSO MAIS ELEVADO QUE ______ FREQÜENTOU?

- 01 ☐ CRECHE
- 02 ☐ PRÉ-ESCOLAR
- 03 ☐ CLASSE DE ALFABETIZAÇÃO DE CRIANÇAS
- 04 ☐ ALFABETIZAÇÃO DE ADULTOS
- 05 ☐ ANTIGO PRIMÁRIO
- 06 ☐ ANTIGO GINÁSIO
- 07 ☐ ANTIGO CLÁSSICO, CIENTÍFICO, ETC.

(01–07) → Passe para o quesito 27.

- 08 ☐ REGULAR ENSINO FUNDAMENTAL → Siga quesito 26.
- 09 ☐ EDUCAÇÃO DE JOVENS E ADULTOS OU SUPLETIVO DO ENSINO FUNDAMENTAL OU 1º GRAU
- 10 ☐ REGULAR DO ENSINO MÉDIO
- 11 ☐ EDUCAÇÃO DE JOVENS E ADULTOS OU SUPLETIVO DO ENSINO MÉDIO OU 2º GRAU
- 12 ☐ TECNOLÓGICO SUPERIOR
- 13 ☐ PRÉ-VESTIBULAR
- 14 ☐ SUPERIOR - GRADUAÇÃO
- 15 ☐ ESPECIALIZAÇÃO SUPERIOR
- 16 ☐ MESTRADO OU DOUTORADO
- 99 ☐ NÃO SABE

(09–99) → Passe para o quesito 27.

26 A DURAÇÃO DESTE CURSO DE ENSINO FUNDAMENTAL OU 1º GRAU ERA DE:

1 ☐ 8 ANOS 2 ☐ 9 ANOS 9 ☐ NÃO SABE

27 QUAL FOI A ÚLTIMA SÉRIE QUE ______ CONCLUIU COM APROVAÇÃO?

01 ☐ PRIMEIRA	04 ☐ QUARTA	07 ☐ SÉTIMA	10 ☐ CURSO NÃO-SERIADO
02 ☐ SEGUNDA	05 ☐ QUINTA	08 ☐ OITAVA	11 ☐ NENHUMA
03 ☐ TERCEIRA	06 ☐ SEXTA	09 ☐ NONA	99 ☐ NÃO SABE

28 ______ CONCLUIU O CURSO MAIS ELEVADO QUE FREQÜENTOU? 1 ☐ SIM 2 ☐ NÃO 9 ☐ NÃO SABE

PARA TODAS AS PESSOAS

29 A COR OU RAÇA DO(A) ______ É:

1 ☐ BRANCA 2 ☐ PRETA 3 ☐ AMARELA 4 ☐ PARDA 5 ☐ INDÍGENA 9 ☐ NÃO SABE

30 A RELIGIÃO OU CULTO DO(A) ______ É:

....

31 ______ TEM REGISTRO DE NASCIMENTO EM CARTÓRIO?

- 1 ☐ SIM → Se idade menor que 3 anos, passe para o quesito 33. Caso contrário, passe para o quesito 34.
- 2 ☐ NÃO → Siga quesito 32.
- 9 ☐ NÃO SABE → Se idade menor que 3 anos, passe para o quesito 33. Caso contrário, passe para o quesito 34.

32 QUAL O MOTIVO DO(A) ______ NÃO TER O REGISTRO DE NASCIMENTO?

- 1 ☐ NÃO ACHA IMPORTANTE
- 2 ☐ PAI E / OU MÃE NÃO TEM DOCUMENTOS PRÓPRIOS
- 3 ☐ O CARTÓRIO É DISTANTE OU DE DIFÍCIL ACESSO
- 4 ☐ NÃO TEM DINHEIRO PARA SE LOCOMOVER ATÉ O CARTÓRIO
- 5 ☐ OUTROS MOTIVOS

04 CARACTERÍSTICAS DO MORADOR

PARA CRIANÇAS MENORES DE 3 ANOS (ENTRE 0 E 35 MESES)

33 COMPRIMENTO EM CENTÍMETROS: ☐☐☐ , ☐

PARA PESSOAS COM 2 ANOS OU MAIS (COM 24 MESES OU MAIS)

34 ALTURA EM CENTÍMETROS: ☐☐☐ , ☐

PARA TODAS AS PESSOAS

35 POSIÇÃO AO SER MEDIDO O COMPRIMENTO OU A ALTURA:

1 ☐ SÓ DEITADO(A)

2 ☐ DEITADO(A) E EM PÉ

3 ☐ SÓ EM PÉ

4 ☐ NENHUMA (DEVIDO A AUSÊNCIA, DOENÇA, RECUSA, ETC.)

PARA PESSOAS COM 2 ANOS OU MAIS (COM 24 MESES OU MAIS)

36 PESO EM QUILOGRAMAS: ☐☐☐ , ☐

PARA CRIANÇAS MENORES DE 2 ANOS (ENTRE 0 E 23 MESES)

37 PESO DO ADULTO COM A CRIANÇA NO COLO EM QUILOGRAMAS: ☐☐☐ , ☐

PESO DO ADULTO SOZINHO EM QUILOGRAMAS: ☐☐☐ , ☐

NOME DO ADULTO QUE SEGUROU A CRIANÇA NO COLO: ________

PARA TODAS AS PESSOAS

38 ______ TEM PLANO OU SEGURO-SAÚDE?

1 ☐ SIM → Siga quesito 39.

2 ☐ NÃO
9 ☐ NÃO SABE
→ Se idade maior ou igual a 10 anos, passe para o quesito 41. Caso contrário, encerre.

39 ______ É O(A) TITULAR DO PLANO OU SEGURO-SAÚDE?

1 ☐ SIM → Siga quesito 40.

2 ☐ NÃO
9 ☐ NÃO SABE
→ Se idade maior ou igual a 10 anos, passe para o quesito 41. Caso contrário, encerre.

40 NÚMERO DE DEPENDENTES DO(A) TITULAR DO PLANO OU SEGURO-SAÚDE: ☐☐

PARA PESSOAS COM 10 ANOS OU MAIS DE IDADE

41 ______ É UMA UNIDADE DE ORÇAMENTO TRABALHO E/OU RENDIMENTO? 1 ☐ SIM 2 ☐ NÃO 3 ☐ RECUSA 4 ☐ OUTRO

42 ______ É UMA UNIDADE DE ORÇAMENTO DESPESA?

1 ☐ SIM → Siga quesito 43.

2 ☐ NÃO → Passe para o quesito 47.

3 ☐ RECUSA
4 ☐ OUTRO
→ Siga quesito 43.

43 ______ TEM CARTÃO DE CRÉDITO?

1 ☐ SIM → Siga quesito 44.

2 ☐ NÃO
9 ☐ NÃO SABE
→ Passe para o quesito 45.

44 ______ É O(A) TITULAR DO CARTÃO DE CRÉDITO? 1 ☐ SIM 2 ☐ NÃO 9 ☐ NÃO SABE

45 ______ TEM CHEQUE ESPECIAL?

1 ☐ SIM → Siga quesito 46.

2 ☐ NÃO
9 ☐ NÃO SABE
→ Passe para o quesito 47.

46 ______ É O(A) TITULAR DA CONTA CORRENTE? 1 ☐ SIM 2 ☐ NÃO 9 ☐ NÃO SABE

47 ______ VAI RESPONDER O BLOCO DE CONSUMO ALIMENTAR? 1 ☐ SIM 2 ☐ NÃO 3 ☐ RECUSA 4 ☐ OUTRO

04 CARACTERÍSTICAS DO MORADOR

Nº DE ORDEM [|]

NOME ....

Nº DA UNIDADE DE CONSUMO: [ ]

CONDIÇÃO NA UNIDADE DE CONSUMO:

1 [ ] PESSOA DE REFERÊNCIA
2 [ ] CÔNJUGE
3 [ ] FILHO
4 [ ] OUTRO PARENTE
5 [ ] AGREGADO
6 [ ] PENSIONISTA
7 [ ] EMPREGADO DOMÉSTICO
8 [ ] PARENTE DE EMPREGADO DOMÉSTICO

01 CONDIÇÃO DE PRESENÇA: 1 [ ] MORADOR PRESENTE → Siga quesito 02. 2 [ ] MORADOR AUSENTE → Passe para o quesito 04.

02 ALTURA / COMPRIMENTO INFORMADO EM CENTÍMETROS: [| |] , [ ]

03 PESO INFORMADO EM QUILOGRAMAS: [| |] , [ ]

04 DATA DE NASCIMENTO: [|] DIA [|] MÊS [| | |] ANO

05 SEXO:

1 [ ] MASCULINO → Se idade menor que 4 anos, passe para o quesito 15. Caso contrário, passe para o quesito 18.

2 [ ] FEMININO → Se idade menor que 4 anos, passe para o quesito 15. Com idade de 4 a 9 anos, passe para o quesito 18. Com 10 anos ou mais de idade, siga quesito 06.

PARA MULHERES COM 10 ANOS OU MAIS DE IDADE

06 ______ ESTÁ GRÁVIDA? 1 [ ] SIM → Siga quesito 07. 2 [ ] NÃO → Passe para o quesito 08.

07 TEMPO DE GESTAÇÃO EM SEMANAS: [|]

08 ______ ESTÁ AMAMENTANDO? 1 [ ] SIM 2 [ ] NÃO

09 ______ TEVE ALGUM FILHO NASCIDO VIVO (OU SEJA, QUE APRESENTOU ALGUM SINAL DE VIDA AO NASCER)?

1 [ ] SIM → Siga quesito 10. 2 [ ] NÃO → Passe para o quesito 18.

10 QUANTOS FILHOS NASCIDOS VIVOS ______ TEVE? [|] HOMENS [|] MULHERES

11 DESTES FILHOS QUE ______ TEVE, QUANTOS ESTÃO VIVOS? [|] HOMENS [|] MULHERES

12 QUAL FOI O MÊS E O ANO DE NASCIMENTO DO ÚLTIMO FILHO NASCIDO VIVO QUE ______ TEVE? [|] MÊS [| | |] ANO

13 ESTE ÚLTIMO FILHO NASCIDO VIVO QUE ______ TEVE, AINDA ESTÁ VIVO?

1 [ ] SIM → Passe para o quesito 18. 2 [ ] NÃO → Siga quesito 14. 9 [ ] NÃO SABE → Passe para o quesito 18.

14 QUAL FOI O MÊS E O ANO DE FALECIMENTO DESTE ÚLTIMO FILHO NASCIDO VIVO QUE ______ TEVE? [|] MÊS [| | |] ANO

PARA AS CRIANÇAS MENORES QUE 4 ANOS (ENTRE 0 E 47 MESES)

15 ______ RECEBE LEITE MATERNO ATUALMENTE?

1 [ ] SIM → Siga quesito 16.
2 [ ] NÃO, MAS JÁ RECEBEU → Passe para o quesito 17.
3 [ ] NUNCA RECEBEU → Passe para o quesito 18.

16 ALÉM DO LEITE MATERNO, ______ RECEBE OUTRO TIPO DE ALIMENTO ATUALMENTE?

1 [ ] SIM → Siga quesito 17.
2 [ ] NÃO
9 [ ] NÃO SABE
(2, 9) → Passe para o quesito 18.

17 POR QUANTOS MESES ______ RECEBEU EXCLUSIVAMENTE O LEITE MATERNO COMO FONTE DE ALIMENTAÇÃO? [|]

04 CARACTERÍSTICAS DO MORADOR

PARA TODAS AS PESSOAS

18 ________ SABE LER E ESCREVER?
- 1 ☐ SIM
- 2 ☐ NÃO

19 ________ FREQÜENTA ESCOLA OU CRECHE?
- 1 ☐ SIM, REDE PARTICULAR → Siga quesito 20.
- 2 ☐ SIM, REDE PÚBLICA → Siga quesito 20.
- 3 ☐ NÃO, MAS JÁ FREQÜENTOU → Passe para o quesito 25.
- 4 ☐ NUNCA FREQÜENTOU → Passe para o quesito 29.
- 9 ☐ NÃO SABE → Passe para o quesito 29.

PARA AS PESSOAS QUE FREQÜENTAM ESCOLA

20 QUAL É O CURSO QUE ________ FREQÜENTA?
- 01 ☐ CRECHE → Passe para o quesito 22.
- 02 ☐ PRÉ-ESCOLAR → Passe para o quesito 22.
- 03 ☐ CLASSE DE ALFABETIZAÇÃO DE CRIANÇAS → Passe para o quesito 22.
- 04 ☐ ALFABETIZAÇÃO DE ADULTOS → Passe para o quesito 22.
- 05 ☐ REGULAR DO ENSINO FUNDAMENTAL → Siga quesito 21.
- 06 ☐ EDUCAÇÃO DE JOVENS E ADULTOS OU SUPLETIVO DO ENSINO FUNDAMENTAL OU 1º GRAU
- 07 ☐ REGULAR DO ENSINO MÉDIO
- 08 ☐ EDUCAÇÃO DE JOVENS E ADULTOS OU SUPLETIVO DO ENSINO MÉDIO OU 2º GRAU
- 09 ☐ TECNOLÓGICO SUPERIOR
- 10 ☐ PRÉ-VESTIBULAR
- 11 ☐ SUPERIOR - GRADUAÇÃO
- 12 ☐ ESPECIALIZAÇÃO SUPERIOR
- 13 ☐ MESTRADO OU DOUTORADO
- 99 ☐ NÃO SABE

(06 a 99) → Passe para o quesito 22.

21 A DURAÇÃO DESTE CURSO DE ENSINO FUNDAMENTAL OU 1º GRAU É DE:
- 1 ☐ 8 ANOS
- 2 ☐ 9 ANOS
- 9 ☐ NÃO SABE

22 QUAL É A SÉRIE QUE ________ FREQÜENTA?
- 01 ☐ PRIMEIRA
- 02 ☐ SEGUNDA
- 03 ☐ TERCEIRA
- 04 ☐ QUARTA
- 05 ☐ QUINTA
- 06 ☐ SEXTA
- 07 ☐ SÉTIMA
- 08 ☐ OITAVA
- 09 ☐ NONA
- 10 ☐ CURSO NÃO-SERIADO
- 99 ☐ NÃO SABE

PARA AS PESSOAS MENORES DE 10 ANOS QUE FREQÜENTAM ESCOLA

23 COM QUE FREQÜÊNCIA ________ COSTUMA SE ALIMENTAR DIARIAMENTE NA ESCOLA?
- 1 ☐ UMA VEZ → Siga quesito 24.
- 2 ☐ DUAS VEZES → Siga quesito 24.
- 3 ☐ TRÊS VEZES → Siga quesito 24.
- 4 ☐ QUATRO VEZES OU MAIS → Siga quesito 24.
- 5 ☐ NENHUMA VEZ → Passe para o quesito 29.
- 9 ☐ NÃO SABE → Siga quesito 24.

24 OS ALIMENTOS QUE ________ CONSOME NA ESCOLA COSTUMAM SER:
- 1 ☐ FORNECIDOS PELA ESCOLA (MERENDA, ALMOÇO, ETC.)
- 2 ☐ LEVADOS DE CASA
- 3 ☐ COMPRADOS NA CANTINA
- 4 ☐ OUTROS

04 CARACTERÍSTICAS DO MORADOR

PARA AS PESSOAS QUE NÃO FREQÜENTAM ESCOLA, MAS JÁ FREQÜENTARAM

25 QUAL FOI O CURSO MAIS ELEVADO QUE ______ FREQÜENTOU?

01 ☐ CRECHE
02 ☐ PRÉ-ESCOLAR
03 ☐ CLASSE DE ALFABETIZAÇÃO DE CRIANÇAS
04 ☐ ALFABETIZAÇÃO DE ADULTOS
05 ☐ ANTIGO PRIMÁRIO
06 ☐ ANTIGO GINÁSIO
07 ☐ ANTIGO CLÁSSICO, CIENTÍFICO, ETC.
→ Passe para o quesito 27.

08 ☐ REGULAR ENSINO FUNDAMENTAL → Siga quesito 26.

09 ☐ EDUCAÇÃO DE JOVENS E ADULTOS OU SUPLETIVO DO ENSINO FUNDAMENTAL OU 1º GRAU
10 ☐ REGULAR DO ENSINO MÉDIO
11 ☐ EDUCAÇÃO DE JOVENS E ADULTOS OU SUPLETIVO DO ENSINO MÉDIO OU 2º GRAU
12 ☐ TECNOLÓGICO SUPERIOR
13 ☐ PRÉ-VESTIBULAR
14 ☐ SUPERIOR - GRADUAÇÃO
15 ☐ ESPECIALIZAÇÃO SUPERIOR
16 ☐ MESTRADO OU DOUTORADO
99 ☐ NÃO SABE
→ Passe para o quesito 27.

26 A DURAÇÃO DESTE CURSO DE ENSINO FUNDAMENTAL OU 1º GRAU ERA DE:

1 ☐ 8 ANOS   2 ☐ 9 ANOS   9 ☐ NÃO SABE

27 QUAL FOI A ÚLTIMA SÉRIE QUE ______ CONCLUIU COM APROVAÇÃO?

01 ☐ PRIMEIRA
02 ☐ SEGUNDA
03 ☐ TERCEIRA
04 ☐ QUARTA
05 ☐ QUINTA
06 ☐ SEXTA
07 ☐ SÉTIMA
08 ☐ OITAVA
09 ☐ NONA
10 ☐ CURSO NÃO-SERIADO
11 ☐ NENHUMA
99 ☐ NÃO SABE

28 ______ CONCLUIU O CURSO MAIS ELEVADO QUE FREQÜENTOU? 1 ☐ SIM   2 ☐ NÃO   9 ☐ NÃO SABE

PARA TODAS AS PESSOAS

29 A COR OU RAÇA DO(A) ______ É:

1 ☐ BRANCA   2 ☐ PRETA   3 ☐ AMARELA   4 ☐ PARDA   5 ☐ INDÍGENA   9 ☐ NÃO SABE

30 A RELIGIÃO OU CULTO DO(A) ______ É:

....

31 ______ TEM REGISTRO DE NASCIMENTO EM CARTÓRIO?

1 ☐ SIM → Se idade menor que 3 anos, passe para o quesito 33. Caso contrário, passe para o quesito 34.
2 ☐ NÃO → Siga quesito 32.
9 ☐ NÃO SABE → Se idade menor que 3 anos, passe para o quesito 33. Caso contrário, passe para o quesito 34.

32 QUAL O MOTIVO DO(A) ______ NÃO TER O REGISTRO DE NASCIMENTO?

1 ☐ NÃO ACHA IMPORTANTE
2 ☐ PAI E / OU MÃE NÃO TEM DOCUMENTOS PRÓPRIOS
3 ☐ O CARTÓRIO É DISTANTE OU DE DIFÍCIL ACESSO
4 ☐ NÃO TEM DINHEIRO PARA SE LOCOMOVER ATÉ O CARTÓRIO
5 ☐ OUTROS MOTIVOS

04 **CARACTERÍSTICAS DO MORADOR**

PARA CRIANÇAS MENORES DE 3 ANOS (ENTRE 0 E 35 MESES)

33 COMPRIMENTO EM CENTÍMETROS: ☐☐☐ , ☐

PARA PESSOAS COM 2 ANOS OU MAIS (COM 24 MESES OU MAIS)

34 ALTURA EM CENTÍMETROS: ☐☐☐ , ☐

PARA TODAS AS PESSOAS

35 POSIÇÃO AO SER MEDIDO O COMPRIMENTO OU A ALTURA:

1 ☐ SÓ DEITADO(A)

2 ☐ DEITADO(A) E EM PÉ

3 ☐ SÓ EM PÉ

4 ☐ NENHUMA (DEVIDO A AUSÊNCIA, DOENÇA, RECUSA, ETC.)

PARA PESSOAS COM 2 ANOS OU MAIS (COM 24 MESES OU MAIS)

36 PESO EM QUILOGRAMAS: ☐☐☐ , ☐

PARA CRIANÇAS MENORES DE 2 ANOS (ENTRE 0 E 23 MESES)

37 PESO DO ADULTO COM A CRIANÇA NO COLO EM QUILOGRAMAS: ☐☐☐ , ☐

PESO DO ADULTO SOZINHO EM QUILOGRAMAS: ☐☐☐ , ☐

NOME DO ADULTO QUE SEGUROU A CRIANÇA NO COLO: ____________

PARA TODAS AS PESSOAS

38 ______ TEM PLANO OU SEGURO-SAÚDE?

1 ☐ SIM → Siga quesito 39.

2 ☐ NÃO / 9 ☐ NÃO SABE → Se idade maior ou igual a 10 anos, passe para o quesito 41. Caso contrário, encerre.

39 ______ É O(A) TITULAR DO PLANO OU SEGURO-SAÚDE?

1 ☐ SIM → Siga quesito 40.

2 ☐ NÃO / 9 ☐ NÃO SABE → Se idade maior ou igual a 10 anos, passe para o quesito 41. Caso contrário, encerre.

40 NÚMERO DE DEPENDENTES DO(A) TITULAR DO PLANO OU SEGURO-SAÚDE: ☐☐

PARA PESSOAS COM 10 ANOS OU MAIS DE IDADE

41 ______ É UMA UNIDADE DE ORÇAMENTO TRABALHO E/OU RENDIMENTO? 1 ☐ SIM 2 ☐ NÃO 3 ☐ RECUSA 4 ☐ OUTRO

42 ______ É UMA UNIDADE DE ORÇAMENTO DESPESA?

1 ☐ SIM → Siga quesito 43.

2 ☐ NÃO → Passe para o quesito 47.

3 ☐ RECUSA / 4 ☐ OUTRO → Siga quesito 43.

43 ______ TEM CARTÃO DE CRÉDITO?

1 ☐ SIM → Siga quesito 44.

2 ☐ NÃO / 9 ☐ NÃO SABE → Passe para o quesito 45.

44 ______ É O(A) TITULAR DO CARTÃO DE CRÉDITO? 1 ☐ SIM 2 ☐ NÃO 9 ☐ NÃO SABE

45 ______ TEM CHEQUE ESPECIAL?

1 ☐ SIM → Siga quesito 46.

2 ☐ NÃO / 9 ☐ NÃO SABE → Passe para o quesito 47.

46 ______ É O(A) TITULAR DA CONTA CORRENTE? 1 ☐ SIM 2 ☐ NÃO 9 ☐ NÃO SABE

47 ______ VAI RESPONDER O BLOCO DE CONSUMO ALIMENTAR? 1 ☐ SIM 2 ☐ NÃO 3 ☐ RECUSA 4 ☐ OUTRO

OBSERVAÇÕES

Ministério do Planejamento, Orçamento e Gestão

IBGE
Instituto Brasileiro de Geografia e Estatística

Diretoria de Pesquisas
Coordenação de Trabalho e Rendimento
Gerência da Pesquisa de Orçamentos Familiares

Pesquisa de Orçamentos Familiares 2008- 2009

POF 2 - Questionário de Aquisição Coletiva

05 IDENTIFICAÇÃO E CONTROLE DO QUESTIONÁRIO

01 IDENTIFICAÇÃO GERAL

UF	MUNICÍPIO	DISTRI-TO	SUBDIS-TRITO	SETOR	Nº DE ORDEM NA LISTAGEM

02 IDENTIFICAÇÃO POF

UF	SEQÜENCIAL	DV	CÓDIGO DO DOMICÍLIO	PERÍODO TEÓRICO	PERÍODO REAL	Nº DA UC	Nº DE MESES DA UC

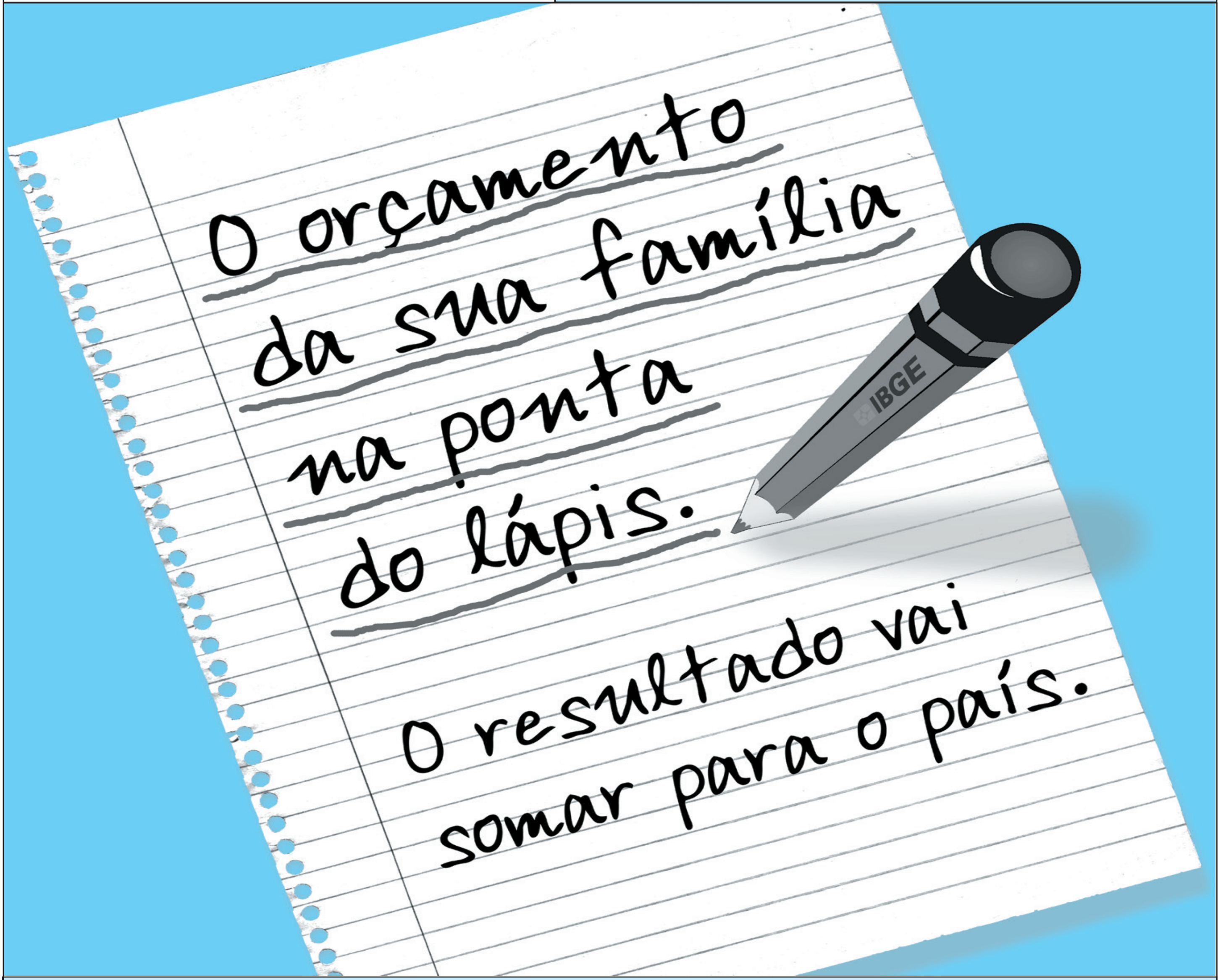

PERÍODOS DE REFERÊNCIA

PERÍODO DE 90 DIAS ____ a ____

PERÍODO DE 12 MESES ____ a ____

MÊS DA ÚLTIMA DESPESA

01 - Janeiro
02 - Fevereiro
03 - Março
04 - Abril
05 - Maio
06 - Junho
07 - Julho
08 - Agosto
09 - Setembro
10 - Outubro
11 - Novembro
12 - Dezembro
99 - Não sabe

FORMA DE AQUISIÇÃO

Escreva, nesta coluna, o código correspondente à Forma de Aquisição para cada um dos produtos ou serviços relacionados:

01 - Monetária à vista para a Unidade de Consumo
02 - Monetária à vista para Outra Unidade de Consumo
03 - Monetária a prazo para a Unidade de Consumo
04 - Monetária a prazo para Outra Unidade de Consumo
05 - Cartão de crédito à vista para a Unidade de Consumo
06 - Cartão de crédito à vista para Outra Unidade de Consumo
07 - Doação
08 - Retirada do Negócio
09 - Troca
10 - Produção Própria
11 - Outra

Por lei, todas as informações prestadas para as pesquisas do IBGE têm caráter confidencial e só podem ser utilizadas para fins estatísticos (Lei 5534 de 14/11/1968).

06 SERVIÇOS E TAXAS DO DOMICÍLIO PRINCIPAL NO PERÍODO DE REFERÊNCIA DE 90 DIAS

SITUAÇÃO DO QUADRO   1 ☐ PESQUISADO COM REGISTRO   3 ☐ PESQUISADO SEM REGISTRO   5 ☐ NÃO-PESQUISADO

TIPO	QUANTIDADE	VALOR DA ÚLTIMA CONTA
(1)	(2)	(3)
ENERGIA ELÉTRICA (kwh)		,
ÁGUA E ESGOTO (m^3)		,
GÁS ENCANADO (m^3)		,
TELEFONE RESIDENCIAL (despesa total)		,
TELEFONE FIXO, CELULAR E INTERNET (pacote)		,
TELEFONE FIXO E INTERNET (pacote)		,
ACESSO À INTERNET (provedor, a cabo, comunicação via satélite, etc.)		,
TV POR ASSINATURA (pacote)		,
		,
		,
		,
		,

07 AQUISIÇÃO DE COMBUSTÍVEIS DOMÉSTICOS E OUTROS DO DOMICÍLIO PRINCIPAL NO PERÍODO DE REFERÊNCIA DE 90 DIAS

SITUAÇÃO DO QUADRO   1 ☐ PESQUISADO COM REGISTRO   3 ☐ PESQUISADO SEM REGISTRO   5 ☐ NÃO-PESQUISADO

TIPO	FORMA DE AQUISIÇÃO	QUANTIDADE	UNIDADE DE MEDIDA	PESO OU VOLUME	VALOR	LOCAL DE AQUISIÇÃO
(1)	(2)	(3)	(4)	(5)	(6)	(7)
GÁS DE BOTIJÃO					,	
ÁGUA PARA USO GERAL					,	
QUEROSENE					,	
ÁLCOOL					,	
PÓ DE SERRA					,	
					,	
					,	
					,	
					,	
					,	
					,	
					,	
					,	
					,	
					,	

08 CONSERVAÇÃO, MANUTENÇÃO E PEQUENOS REPAROS COM HABITAÇÃO, JAZIGO E JARDINAGEM NO PERÍODO DE REFERÊNCIA DE 90 DIAS

SITUAÇÃO DO QUADRO 1 ☐ PESQUISADO COM REGISTRO 3 ☐ PESQUISADO SEM REGISTRO 5 ☐ NÃO-PESQUISADO

TIPO	FORMA DE AQUISIÇÃO	VALOR	LOCAL DE AQUISIÇÃO
(1)	(2)	(3)	(4)
MÃO-DE-OBRA (pedreiro, marceneiro, eletricista, pintor, etc.)		,	
REFORMA OU OBRA POR EMPREITADA		,	
CIMENTO		,	
TIJOLO		,	
TORNEIRA, CANO E MATERIAL HIDRÁULICO EM GERAL		,	
FERRAGENS (fechadura, ferro, arame, prego, etc.)		,	
MADEIRA E TACO		,	
MADEIRA E TACO DE MADEIRA CERTIFICADA (selo FSC)		,	
AZULEJO E PISO		,	
JANELA E PORTA		,	
MATERIAL DE PINTURA EM GERAL		,	
TINTA		,	
AREIA, TERRA, BARRO, SAIBRO, ETC.		,	
FIO E MATERIAL ELÉTRICO EM GERAL		,	
APARELHOS SANITÁRIOS (pia, vaso, bidê, banheira, etc.)		,	
TELHAS (barro, amianto, fibra, alumínio, etc.)		,	
VIDRO		,	
PEDRAS (britada, marroada, paralelepípedo, etc.)		,	
ARTIGOS DE JARDINAGEM (planta, terra, semente, xaxim, etc.)		,	
LAJE PRÉ-MOLDADA		,	
CARRETO DE MATERIAL DE CONSTRUÇÃO		,	
MANUTENÇÃO DE JAZIGO		,	
EXUMAÇÃO		,	
PLACA DE JAZIGO		,	
		,	
		,	
		,	
		,	
		,	
		,	
		,	
		,	
		,	
		,	

09 CONSERTOS E MANUTENÇÃO DE MÓVEIS, APARELHOS, MÁQUINAS E UTENSÍLIOS DE USO DOMÉSTICO NO PERÍODO DE REFERÊNCIA DE 90 DIAS

SITUAÇÃO DO QUADRO 1 ☐ PESQUISADO COM REGISTRO 3 ☐ PESQUISADO SEM REGISTRO 5 ☐ NÃO-PESQUISADO

TIPO	FORMA DE AQUISIÇÃO	VALOR	LOCAL DE AQUISIÇÃO
(1)	(2)	(3)	(4)
CONSERTO DE MÓVEIS (peça + mão-de-obra)		,	
MANUTENÇÃO DE MÓVEIS		,	
CONSERTO DE GELADEIRA (peça)		,	
CONSERTO DE GELADEIRA (mão-de-obra)		,	
CONSERTO DE GELADEIRA (peça + mão-de-obra)		,	
CONSERTO DE TELEVISÃO (peça)		,	
CONSERTO DE TELEVISÃO (mão-de-obra)		,	
CONSERTO DE TELEVISÃO (peça + mão-de-obra)		,	
CONSERTO DE APARELHO SONORO (peça)		,	
CONSERTO DE APARELHO SONORO (mão-de-obra)		,	
CONSERTO DE APARELHO SONORO (peça + mão-de-obra)		,	
MANUTENÇÃO DE APARELHOS DOMÉSTICOS		,	
MANUTENÇÃO DE UTENSÍLIOS DOMÉSTICOS ELÉTRICOS		,	
MANUTENÇÃO DE UTENSÍLIOS DOMÉSTICOS NÃO-ELÉTRICOS		,	
		,	
		,	
		,	

10 ALUGUEL, IMPOSTOS E OUTRAS TAXAS DO DOMICÍLIO PRINCIPAL NO PERÍODO DE REFERÊNCIA DE 12 MESES

SITUAÇÃO DO QUADRO 1 ☐ PESQUISADO COM REGISTRO 3 ☐ PESQUISADO SEM REGISTRO 5 ☐ NÃO-PESQUISADO

TIPO	FORMA DE AQUISIÇÃO	ÚLTIMA DESPESA MENSAL	MÊS	Nº DE MESES	LOCAL DE AQUISIÇÃO
(1)	(2)	(3)	(4)	(5)	(6)
ALUGUEL DO IMÓVEL		,			
ADICIONAIS DO ALUGUEL DO IMÓVEL (juros, multa, etc.)		,			
PRESTAÇÃO DO IMÓVEL		,			
CONDOMÍNIO		,			
IMPOSTO PREDIAL E TERRITORIAL URBANO (IPTU)		,			
ALUGUEL DE GARAGEM		,			
		,			
		,			

11 — **CONSTRUÇÃO E REFORMA DE HABITAÇÃO E JAZIGO NO PERÍODO DE REFERÊNCIA DE 12 MESES**

SITUAÇÃO DO QUADRO 1 ☐ PESQUISADO COM REGISTRO 3 ☐ PESQUISADO SEM REGISTRO 5 ☐ NÃO-PESQUISADO

TIPO	FORMA DE AQUISIÇÃO	VALOR	LOCAL DE AQUISIÇÃO
(1)	(2)	(3)	(4)
MÃO-DE-OBRA (pedreiro, marceneiro, eletricista, pintor, etc.)		,	
CIMENTO		,	
TIJOLO		,	
TORNEIRA, CANO E MATERIAL HIDRÁULICO EM GERAL		,	
FERRAGENS (fechadura, ferro, arame, prego, etc.)		,	
MADEIRA E TACO		,	
MADEIRA E TACO DE MADEIRA CERTIFICADA (selo FSC)		,	
AZULEJO E PISO		,	
JANELA E PORTA		,	
MATERIAL DE PINTURA EM GERAL		,	
TINTA		,	
AREIA, TERRA, BARRO, SAIBRO, ETC.		,	
FIO E MATERIAL ELÉTRICO EM GERAL		,	
APARELHOS SANITÁRIOS (pia, vaso, bidê, banheira, etc.)		,	
TELHAS (barro, amianto, fibra, alumínio, etc.)		,	
VIDRO		,	
PEDRAS (britada, marroada, paralelepípedo, etc.)		,	
LAJE PRÉ-MOLDADA		,	
CARRETO DE MATERIAL DE CONSTRUÇÃO		,	
REFORMA OU OBRA POR EMPREITADA		,	
CONSTRUÇÃO DE JAZIGO		,	
REFORMA DE JAZIGO		,	
LÁPIDE		,	
AQUISIÇÃO DE SEPULTURA		,	
		,	
		,	
		,	
		,	
		,	
		,	
		,	
		,	
		,	
		,	

12 OUTROS ITENS DO DOMICÍLIO PRINCIPAL COM SERVIÇOS PÚBLICOS, PRIVADOS E HABITAÇÃO NO PERÍODO DE REFERÊNCIA DE 12 MESES

SITUAÇÃO DO QUADRO 1 ☐ PESQUISADO COM REGISTRO 3 ☐ PESQUISADO SEM REGISTRO 5 ☐ NÃO-PESQUISADO

TIPO	FORMA DE AQUISIÇÃO	VALOR
(1)	(2)	(3)
AQUISIÇÃO DO IMÓVEL À VISTA (valor pago, imposto de transmissão, etc.)		,
AQUISIÇÃO DO IMÓVEL A PRAZO (entrada, parcelas, taxas, impostos, etc.)		,
LOCAÇÃO DO IMÓVEL (contrato, depósitos de locação e conservação, etc.)		,
MUDANÇA		,
SEGUROS SOBRE O IMÓVEL (incêndio, roubo, etc.)		,
TAXAS (coleta de lixo, prevenção e extinção de incêndio, segurança, etc.)		,
TAXA DE OCUPAÇÃO DO IMÓVEL		,
TAXA DE INSTALAÇÃO DE RELÓGIO DE ÁGUA		,
TAXA DE TRANSFERÊNCIA DE TELEFONE		,
TAXA DE ADESÃO DE TELEVISÃO POR ASSINATURA		,
TAXA DE INSTALAÇÃO DE TELEVISÃO POR ASSINATURA		,
TAXA DE INSTALAÇÃO DE INTERNET		,
TAXA EXTRA DE CONDOMÍNIO (reforma e manutenção)		,
VALOR DO IMÓVEL ADQUIRIDO EM PRIMEIRA LOCAÇÃO À VISTA		,
VALOR DO IMÓVEL ADQUIRIDO EM PRIMEIRA LOCAÇÃO A PRAZO		,
VALOR DO IMÓVEL ADQUIRIDO USADO À VISTA		,
VALOR DO IMÓVEL ADQUIRIDO USADO A PRAZO		,
....		,
....		,

13 ALUGUEL DE APARELHOS E UTILIDADES DE USO DOMÉSTICO NO PERÍODO DE REFERÊNCIA DE 12 MESES

SITUAÇÃO DO QUADRO 1 ☐ PESQUISADO COM REGISTRO 3 ☐ PESQUISADO SEM REGISTRO 5 ☐ NÃO-PESQUISADO

TIPO	FORMA DE AQUISIÇÃO	VALOR
(1)	(2)	(3)
TELEFONE RESIDENCIAL		,
TELEVISÃO		,
DVD		,
MICROCOMPUTADOR		,
....		,
....		,

14 INVENTÁRIO DE BENS DURÁVEIS DO DOMICÍLIO PRINCIPAL

SITUAÇÃO DO QUADRO 1 ☐ PESQUISADO COM REGISTRO 3 ☐ PESQUISADO SEM REGISTRO 5 ☐ NÃO-PESQUISADO

TIPO	QUANTIDADE	ÚLTIMA AQUISIÇÃO		
		FORMA DE AQUISIÇÃO	ANO	ESTADO<br>1 - Novo<br>3 - Usado<br>9 - Não sabe
(1)	(2)	(3)	(4)	(5)
FOGÃO				
FREEZER				
GELADEIRA DE 1 PORTA				
GELADEIRA DE 2 PORTAS				
CHUVEIRO ELÉTRICO				
LIQUIDIFICADOR				
PROCESSADOR DE ALIMENTOS				
GRILL				
ASPIRADOR DE PÓ				
FORNO ELÉTRICO				
FERRO ELÉTRICO				
MÁQUINA DE LAVAR ROUPAS				
TELEVISÃO EM CORES				
TELEVISÃO EM PRETO E BRANCO				
EQUIPAMENTO DE SOM				
RÁDIO				
AR-CONDICIONADO				
VENTILADOR E/OU CIRCULADOR DE AR				
MÁQUINA DE COSTURA				
FILTRO DE ÁGUA				
AUTOMÓVEL				
BICICLETA				
MOTOCICLETA				
MICROCOMPUTADOR				
PURIFICADOR DE ÁGUA				
FORNO DE MICROONDAS				
ANTENA PARABÓLICA				
APARELHO DE DVD				
MÁQUINA DE SECAR ROUPAS				
BATEDEIRA DE BOLO				
SECADOR DE CABELOS				
MÁQUINA DE LAVAR LOUÇAS				

15 **AQUISIÇÃO DE APARELHOS, MÁQUINAS E OUTRAS UTILIDADES DE USO DOMÉSTICO NO PERÍODO DE REFERÊNCIA DE 12 MESES (continua)**

SITUAÇÃO DO QUADRO 1 ☐ PESQUISADO COM REGISTRO 3 ☐ PESQUISADO SEM REGISTRO 5 ☐ NÃO-PESQUISADO

TIPO	FORMA DE AQUISIÇÃO	VALOR	ESTADO<br>1 - Novo<br>3 - Usado<br>9 - Não sabe	LOCAL DE AQUISIÇÃO
(1)	(2)	(3)	(4)	(5)
TELEVISÃO EM CORES		,		
TELEVISÃO EM PRETO E BRANCO		,		
TELEVISÃO E VIDEOCASSETE ACOPLADOS		,		
VIDEOCASSETE		,		
DVD		,		
GELADEIRA		,		
FREEZER		,		
FOGÃO A GÁS		,		
FOGÃO ELÉTRICO		,		
FOGÃO A LENHA		,		
FERRO ELÉTRICO		,		
MÁQUINA DE LAVAR ROUPAS		,		
LIQUIDIFICADOR		,		
GRAVADOR E TOCA-FITAS		,		
CAIXA DE SOM		,		
ALTO-FALANTE, *TWEETER,* MEGAFONE, MICROFONE, ETC.		,		
CONJUNTO DE SOM ACOPLADO		,		
WALKMAN		,		
RÁDIO-RELÓGIO OU DE MESA		,		
TELEFONE-RÁDIO-RELÓGIO		,		
RÁDIO PORTÁTIL		,		
VENTILADOR		,		
AR-CONDICIONADO		,		
CIRCULADOR DE AR		,		
CHUVEIRO OU DUCHA ELÉTRICOS		,		
SECADOR E MODELADOR DE CABELO		,		
MÁQUINA DE COSTURA ELÉTRICA		,		
MÁQUINA DE COSTURA A PEDAL		,		
VIDEO GAME		,		
ANTENA DE TELEVISÃO NÃO-PARABÓLICA		,		
ANTENA PARABÓLICA E EQUIPAMENTOS		,		
PURIFICADOR DE ÁGUA		,		

15 AQUISIÇÃO DE APARELHOS, MÁQUINAS E OUTRAS UTILIDADES DE USO DOMÉSTICO NO PERÍODO DE REFERÊNCIA DE **12 MESES** (conclusão)

TIPO	FORMA DE AQUISIÇÃO	VALOR	ESTADO<br>1 - Novo<br>3 - Usado<br>9 - Não sabe	LOCAL DE AQUISIÇÃO
(1)	(2)	(3)	(4)	(5)
MICROCOMPUTADOR		,		
IMPRESSORA DE MICROCOMPUTADOR		,		
ASPIRADOR DE PÓ		,		
EXAUSTOR		,		
ENCERADEIRA		,		
ESPREMEDOR DE FRUTAS ELÉTRICO		,		
PROCESSADOR DE ALIMENTOS ELÉTRICO		,		
FORNO ELÉTRICO		,		
FORNO DE MICROONDAS		,		
MÁQUINA DE SECAR ROUPAS		,		
MÁQUINA DE LAVAR PRATOS		,		
BOMBA DE ÁGUA		,		
CAFETEIRA ELÉTRICA		,		
BARBEADOR ELÉTRICO		,		
TANQUE DE ROUPA ELÉTRICO		,		
TOCA-DISCOS A LASER		,		
TORRADEIRA ELÉTRICA		,		
GRILL		,		
SECRETÁRIA ELETRÔNICA		,		
VAPORIZADOR ELÉTRICO		,		
MÁQUINA FOTOGRÁFICA		,		
LINHA TELEFÔNICA RESIDENCIAL		,		
APARELHO TELEFÔNICO NÃO CELULAR		,		
		,		
		,		
		,		
		,		
		,		
		,		
		,		
		,		
		,		
		,		

16	AQUISIÇÃO DE FERRAMENTAS, ANIMAIS DOMÉSTICOS, EQUIPAMENTOS MUSICAIS E DE ACAMPAMENTO NO PERÍODO DE REFERÊNCIA DE **12 MESES**

SITUAÇÃO DO QUADRO 1 ☐ PESQUISADO COM REGISTRO 3 ☐ PESQUISADO SEM REGISTRO 5 ☐ NÃO-PESQUISADO

TIPO	FORMA DE AQUISIÇÃO	VALOR	ESTADO<br>1 - Novo<br>3 - Usado<br>9 - Não sabe	LOCAL DE AQUISIÇÃO
(1)	(2)	(3)	(4)	(5)
ACESSÓRIOS DE INSTRUMENTOS MUSICAIS		,	0	
PIANO		,		
VIOLÃO		,		
BATERIA MUSICAL		,		
FERRAMENTAS EM GERAL		,		
CAIXA DE FERRAMENTAS		,		
COMPRA DE ANIMAL DOMÉSTICO		,		
TOSADEIRA DE ANIMAL DOMÉSTICO		,		
VETERINÁRIO		,		
TRATAMENTO DE SAÚDE DE ANIMAL DOMÉSTICO		,		
SERVIÇO DE HIGIENE DE ANIMAL DOMÉSTICO		,		
ADESTRAMENTO DE ANIMAL DOMÉSTICO		,		
ARTIGOS DE LONA OU PLÁSTICO PARA ACAMPAMENTO		,		
FOGÃO PARA CAMPING		,		
BARRACA DE ACAMPAMENTO		,		
LAMPIÃO		,		
		,		
		,		
		,		
		,		
		,		
		,		
		,		
		,		
		,		
		,		
		,		
		,		
		,		
		,		
		,		
		,		

17 AQUISIÇÃO DE MÓVEIS NO PERÍODO DE REFERÊNCIA DE 12 MESES (continua)

SITUAÇÃO DO QUADRO 1 ☐ PESQUISADO COM REGISTRO 3 ☐ PESQUISADO SEM REGISTRO 5 ☐ NÃO-PESQUISADO

TIPO	FORMA DE AQUISIÇÃO	VALOR	ESTADO<br>1 - Novo<br>3 - Usado<br>9 - Não sabe	LOCAL DE AQUISIÇÃO
(1)	(2)	(3)	(4)	(5)
**MÓVEIS DE SALA (ESTAR E JANTAR)**				
CONJUNTO ESTOFADO		,		
CONJUNTO ESTOFADO E MESINHA(s)		,		
POLTRONA		,		
ESTANTE		,		
MESA E CADEIRAS		,		
CADEIRA		,		
MESA		,		
CADEIRA DE BALANÇO		,		
SOFÁ OU SOFÁ-CAMA		,		
MESA PARA TV, SOM, VÍDEO E MICROCOMPUTADOR		,		
MESINHA DE CENTRO		,		
BAR		,		
ARCA COM OU SEM VITRINE OU ORATÓRIO		,		
CARRINHO DE CHÁ OU DE BEBIDAS		,		
....		,		
**MÓVEIS DE QUARTO (ADULTO)**				
ARMÁRIO SIMPLES, DÚPLEX OU EMBUTIDO		,		
DORMITÓRIO COMPLETO (armário, cama,etc.)		,		
CAMA		,		
BICAMA, BELICHE OU TRILICHE		,		
MESINHA DE CABECEIRA		,		
CÔMODA		,		
CONSOLE		,		
COLCHÃO		,		
....		,		
**MÓVEIS DE COPA E COZINHA (continua)**				
ARMÁRIO		,		
ARMÁRIO, MESA E CADEIRAS		,		
MESA E CADEIRAS		,		
CADEIRA		,		
MESA		,		
....		,		

17	AQUISIÇÃO DE MÓVEIS NO PERÍODO DE REFERÊNCIA DE 12 MESES (conclusão)			
TIPO	FORMA DE AQUISIÇÃO	VALOR	ESTADO<br>1 - Novo<br>3 - Usado<br>9 - Não sabe	LOCAL DE AQUISIÇÃO
(1)	(2)	(3)	(4)	(5)
**MÓVEIS DE COPA E COZINHA (conclusão)**				
.....		,		.....
.....		,		.....
.....		,		.....
**MÓVEIS INFANTIS**				
BERÇO		,		.....
CARRINHO DE BEBÊ E GÔNDOLA		,		.....
ARMÁRIO, CAMA E MESINHA DE CABECEIRA		,		.....
ARMÁRIO		,		.....
CAMA		,		.....
CADEIRINHA		,		.....
CADEIRA ALTA DE REFEIÇÃO		,		.....
CERCADO		,		.....
COLCHÃO		,		.....
CÔMODA		,		.....
.....		,		.....
.....		,		.....
.....		,		.....
.....		,		.....
**MÓVEIS EXTERNOS E DIVERSOS**				
REDE DE ADULTO E CRIANÇA		,		.....
MESA COM CADEIRAS (jardim, varanda, etc.)		,		.....
CADEIRA (jardim, varanda, etc.)		,		.....
BANCO (jardim, varanda, etc.)		,		.....
ESCRIVANINHA		,		.....
GUARDA-SOL		,		.....
MESA DE TELEFONE		,		.....
ARMÁRIO DE BANHEIRO (não plástico)		,		.....
PRATELEIRA		,		.....
COLCHONETE		,		.....
.....		,		.....
.....		,		.....
.....		,		.....

18	AQUISIÇÃO DE ARTIGOS DE DECORAÇÃO E FORRAÇÃO NO PERÍODO DE REFÊRENCIA DE **12 MESES**

SITUAÇÃO DO QUADRO 1 ☐ PESQUISADO COM REGISTRO 3 ☐ PESQUISADO SEM REGISTRO 5 ☐ NÃO-PESQUISADO

TIPO	FORMA DE AQUISIÇÃO	VALOR	ESTADO<br>1 - Novo<br>3 - Usado<br>9 - Não sabe	LOCAL DE AQUISIÇÃO
(1)	(2)	(3)	(4)	(5)
TAPETE DE SALA OU QUARTO		,		
TAPEÇARIA		,		
CORTINA PRONTA (exceto de plástico)		,		
QUADRO OU MOLDURA DE QUADRO, PÔSTER, PORTA-RETRATO, ETC.		,		
LUMINÁRIA, ABAJUR, LUSTRE, GLOBO, ETC.		,		
ALMOFADA		,		
ENFEITES DE ÁRVORE DE NATAL		,		
ESPELHO		,		
AQUÁRIO ORNAMENTAL E EQUIPAMENTOS		,		
PEÇA DE LOUÇA		,		
PEÇA DE CERÂMICA		,		
PEÇA DE METAL (exceto de prata)		,		
PEÇA DE PRATA		,		
PEÇA DE VIDRO		,		
PEÇA DE GESSO		,		
PEÇA DE CRISTAL		,		
PEÇA DE MADEIRA		,		
FLORES ARTIFICIAIS		,		
PERSIANAS		,		
TAPETE DE BANHEIRO (tecido)		,		
		,		
		,		
		,		
		,		
		,		
		,		
		,		
		,		
		,		
		,		
		,		
		,		
		,		

19	SERVIÇOS DOMÉSTICOS NO PERÍODO DE REFÊRENCIA DE 12 MESES

SITUAÇÃO DO QUADRO 1 ☐ PESQUISADO COM REGISTRO 3 ☐ PESQUISADO SEM REGISTRO 5 ☐ NÃO-PESQUISADO

TIPO	FORMA DE AQUISIÇÃO	ÚLTIMO GASTO MENSAL		ESPÉCIE<br>1 - Mensalista<br>3 - Diarista<br>9 - Não sabe	MÊS	Nº DE MESES
		VALOR	INSS			
(1)	(2)	(3)	(4)	(5)	(6)	(7)
EMPREGADA		,	,			
FAXINEIRA		,	,			
LAVADEIRA E PASSADEIRA		,	,			
LAVADEIRA		,	,			
PASSADEIRA		,	,			
COZINHEIRO		,	,			
BABÁ		,	,			
JARDINEIRO		,	,			
CASEIRO		,	,			
VIGIA		,	,			
MOTORISTA		,	,			
....		,	,			
....		,	,			
....		,	,			
....		,	,			
....		,	,			
....		,	,			
....		,	,			

OBSERVAÇÕES

_ _

_ _

_ _

_ _

_ _

_ _

_ _

_ _

_ _

_ _

_ _

Ministério do Planejamento, Orçamento e Gestão

IBGE
Instituto Brasileiro de Geografia e Estatística

Diretoria de Pesquisas
Coordenação de Trabalho e Rendimento
Gerência da Pesquisa de Orçamentos Familiares

Pesquisa de Orçamentos Familiares 2008- 2009

POF 3 - Caderneta de Aquisição Coletiva

62	IDENTIFICAÇÃO E CONTROLE DO QUESTIONÁRIO
01	IDENTIFICAÇÃO GERAL

UF	MUNICÍPIO	DISTRI-TO	SUBDIS-TRITO	SETOR	Nº DE ORDEM NA LISTAGEM

02	IDENTIFICAÇÃO POF

UF	SEQÜENCIAL	DV	CÓDIGO DO DOMICÍLIO	PERÍODO TEÓRICO	PERÍODO REAL	Nº DA UC

03	INFORMAÇÕES PREENCHIDAS POR:

INFORMANTE ☐ PESQUISADOR / ATRAVÉS DO INFORMANTE ☐

TOTAL DE DIAS PESQUISADOS ☐

NOME DO INFORMANTE ....

Prezado(a) senhor(a),

A sua colaboração no preenchimento diário desta caderneta representa uma efetiva contribuição para o sucesso da pesquisa de orçamentos familiares. Recordamos que as informações prestadas serão usadas exclusivamente para fins estatísticos e serão mantidas em sigilo, conforme estabelecido na lei 5.534 de 14/11/1968.

Muito obrigado por sua colaboração.

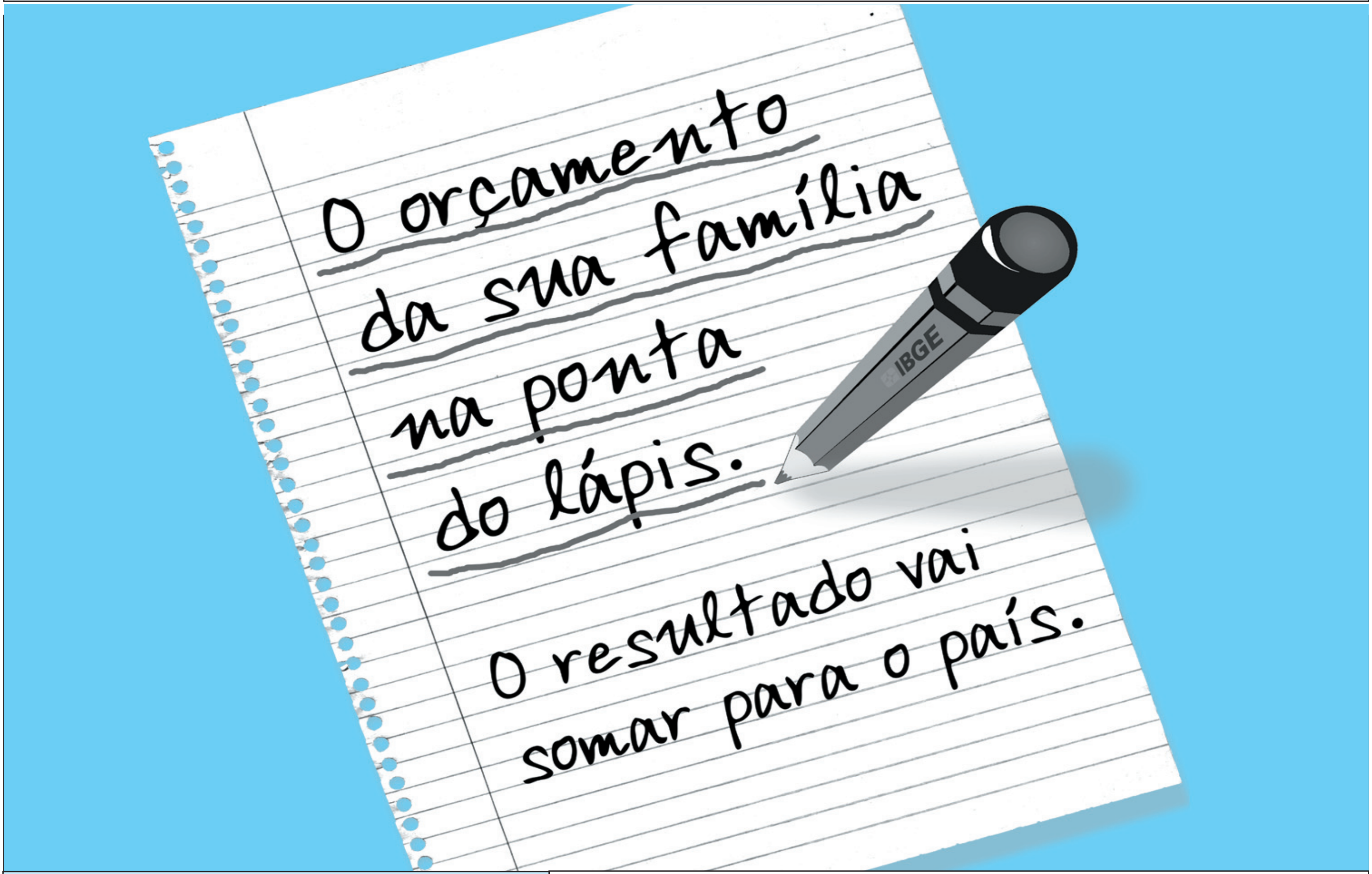

PERÍODO DE REFERÊNCIA

PERÍODO DE 7 DIAS

Data de início ☐ a ☐ Data de término

FORMA DE AQUISIÇÃO

Escreva nesta coluna, o código correspondente à Forma de Aquisição para cada um dos produtos relacionados:

01 - Monetária à vista para a Unidade de Consumo
02 - Monetária à vista para Outra Unidade de Consumo
03 - Monetária a prazo para a Unidade de Consumo
04 - Monetária a prazo para Outra Unidade de Consumo
05 - Cartão de crédito à vista para a Unidade de Consumo
06 - Cartão de crédito à vista para Outra Unidade de Consumo
07 - Doação
08 - Retirada do Negócio
09 - Troca
10 - Produção Própria
11 - Outra

INSTRUÇÕES PARA O PREENCHIMENTO

ANOTE NESTA CADERNETA TODAS AS AQUISIÇÕES COM:

ALIMENTOS E BEBIDAS
ARTIGOS DE HIGIENE PESSOAL E DE LIMPEZA DOMÉSTICA
COMBUSTÍVEIS DE USO DOMÉSTICO (EXCETO GÁS E LENHA)
COMIDA E ARTIGOS PARA ANIMAIS
OUTRAS PEQUENAS COMPRAS (VELA, PILHA, LÂMPADA, ETC.)

O PREENCHIMENTO DE CADA COLUNA DEVE SER FEITO DA SEGUINTE FORMA:

DESCRIÇÃO DETALHADA DO PRODUTO

Escreva, nesta coluna:
- A quantidade adquirida do produto;
- A unidade de medida pela qual o produto foi adquirido. Registre o peso ou volume da unidade, sempre que for possível; e
- O tipo do produto adquirido.

Exemplos: 2 pacotes de 500 gramas de macarrão com ovos;
1 lata de 400 gramas de leite em pó integral;
5 quilo de arroz polido;

VALOR (R$)

Escreva, nesta coluna, o total da aquisição do produto.
Considere os centavos.
Registre todas as aquisições efetuadas, inclusive as de pequeno valor.

LOCAL DE AQUISIÇÃO

Escreva, nesta coluna, o nome do local onde o produto foi adquirido.
Exemplos: supermercado;
vendedor ambulante;
padaria;
lanchonete;
açougue;
peixaria;
feira-livre ou feira;
drogaria.

ATENÇÃO

Para os produtos que possuem diferentes características , os registros deverão ter suas especificações: ***ORGÂNICO***, ***LIGHT*** **ou** ***DIET.***

PARA OS PRODUTOS ABAIXO RELACIONADOS, AS ANOTAÇÕES DEVEM SER FEITAS DA SEGUINTE FORMA:

ARROZ	Arroz polido, arroz integral orgânico, arroz com casca, etc.
FEIJÃO	Feijão-preto, feijão-jalo, feijão-mulatinho, feijão-macassar, feijão-preto orgânico, etc.
CARNE DE BOI	Alcatra orgânica, pá, contra-filé, costela de boi, chã-de-dentro, patinho, etc.
CARNE DE PORCO	Lombinho de porco, costelinha de porco, bisteca de porco, etc.
PEIXE	Peixe inteiro sardinha, peixe em postas badejo, peixe em filé fresco anchova, etc. (iniciar pela palavra peixe)
FILÉ DE PEIXE	Filé de peixe badejo fresco, filé de peixe merluza congelado, filé de peixe pescada embalado congelado, etc.
AVES	Frango vivo, frango abatido, frango congelado, coxa de frango, pato abatido, etc.
PÃO	Pão francês, pão doce, pão de fôrma light, pão de fôrma diet, etc.
LEITE	Leite de vaca in natura, leite em pó integral, leite de vaca orgânico, leite de vaca light, etc.
MACARRÃO	Macarrão com ovos, talharim sem ovos, macarrão instantâneo diet, macarrão com ovos light, etc.
ÓLEO	Óleo de soja, óleo de milho, óleo de algodão, óleo de girassol, óleo de granola, etc.
BISCOITO	Biscoito salgado, biscoito doce light, rosca doce, rosca salgada, etc.
CAFÉ	Café moído, café solúvel, café descafeinado, café moído orgânico, etc.
AÇÚCAR	Açúcar refinado, açúcar cristal, açúcar refinado light, açúcar mascavo, açúcar cristal orgânico, etc.
SAL e CONDIMENTOS	Sal refinado, sal grosso, sal refinado light, maionese diet, etc.
QUEIJO	Queijo-de-minas, queijo prato, queijo mussarela, queijo-de-minas light, etc.
LARANJA	Laranja-pêra, laranja-seleta, laranja-da-baía, laranja-lima, etc.
BANANA	Banana-prata, banana-maçã, banana-da-terra, banana-nanica, etc.
REFRIGERANTE	Refrigerante de cola, refrigerante de guaraná, refrigerante de laranja light, etc. (iniciar pela palavra refrigerante)
MAÇÃ	Maçã gala, maçã argentina, maçã orgânica

63	1 Nº DO DIA	DIA DA SEMANA	DATA
	1		___/___/___

2 SITUAÇÃO DO QUADRO			3 Nº DA FOLHA
1 ☐ PESQUISADO COM REGISTRO	3 ☐ PESQUISADO SEM REGISTRO	5 ☐ NÃO-PESQUISADO	0 1

DESCRIÇÃO DETALHADA DO PRODUTO (QUANTIDADE, UNIDADE DE MEDIDA E TIPO)	FORMA DE AQUISIÇÃO	VALOR (R$)	LOCAL DE AQUISIÇÃO
(4)	(5)	(6)	(7)
6 unidades de 50 gramas de pão francês	0 1	1,20	padaria
1 litro de leite de vaca semidesnatado	0 1	1,10	padaria
1 pacote de 500 gramas de café moído orgânico	0 1	2,75	supermercado
250 gramas de queijo prato	0 1	2,00	padaria
5 quilos de arroz polido	0 1	5,30	supermercado
2 latas de 400 gramas de leite em pó integral	0 1	4,90	supermercado
1 pacote de 500 gramas de macarrão com ovos	0 1	0,80	mercearia
1 pacote de 200 gramas de biscoito salgado	0 2	0,75	vendedor ambulante
1 pote de 500 gramas de margarina light	0 1	1,50	padaria
2 quilos de alcatra	0 1	10,40	açougue
2300 gramas de costela de boi	0 1	6,90	açougue
1300 gramas de frango inteiro congelado	0 1	6,20	açougue
2400 gramas de peixe corvina inteiro	1 1	8,40	natureza
2400 gramas de filé de peixe pescada congelado	0 1	7,20	açougue
6 unidades de 120 gramas de iogurte diet	0 1	2,40	supermercado
1 quilo de sal refinado	0 1	0,46	armazém
2 litros de refrigerante guaraná diet	0 1	1,55	supermercado
2 pacotes de 200 gramas de biscoito doce light	0 1	3,60	supermercado
12 latas de 350 mililitros de cerveja	0 1	8,40	supermercado
1 unidade de quentinha (refeição pronta)	0 1	4,50	restaurante
2 molhos de espinafre orgânico	0 7	1,00	feira-livre
1 caixa de 1 quilo de sabão em pó	0 1	3,20	feira-livre
2 dúzias de banana maçã	1 0	3,00	domicílio
1 quilo de banana prata	0 1	1,70	sacolão
4 unidades de 90 gramas de sabonete	0 1	1,20	drogaria
1 saco de 15 quilos de ração para cachorro	0 1	26,80	supermercado
2 pacotes com 10 unidades de absorvente higiênico	0 1	2,50	farmácia
	0 1		

EXEMPLO

EXEMPLOS DE DESCRIÇÃO DETALHADA DO PRODUTO

1 quilograma ou quilo de frango inteiro congelado
1 litro de leite de vaca integral
250 gramas de queijo prato light
1 porção de pimenta malagueta
1 molho de agrião orgânico
1 caixa com 450 gramas de sucrilhos
1 pé de alface crespa
1 unidade inteira de melancia
1 pote de 250 gramas de margarina
2 dúzias de ovos
1 saco de 1 quilo de açúcar
1 bandeja de 720 gramas de salaminho

EXEMPLOS DE LOCAIS DE AQUISIÇÃO

Supermercado	Padaria	Farmácia	Armazém	Bar	Feira-livre	Mercado
Vendedor ambulante	Peixaria	Mercearia	Loja de departamento	Varejão	Açougue	Lanchonete
Confeitaria	Quitanda	Sacolão	Adega	Sorveteria	Depósito de doces	

GUIA DE REGISTRO DE COMPRAS

PRINCIPAIS ALIMENTOS E BEBIDAS

LEITE
De vaca pasteurizado
De vaca "in natura"
Em pó integral
Em pó desnatado
Condensado

DERIVADOS DO LEITE
Iogurte
Manteiga
Creme de leite
Requeijão
Queijo prato
Queijo ralado
Queijo-minas
Queijo provolone

FARINHAS
Láctea
Maisena
Fubá
De rosca
De soja
Neston
De trigo
De mandioca
De aveia
Sustagem

PÃO
Francês
De milho
De forma
Doce
De queijo
De rabanada
De centeio
Integral

BISCOITO E BOLO
Biscoito doce
Biscoito salgado
Bolo industrializado
Rosca doce
Rosca salgada

AÇÚCAR
Cristal
Refinado
Demerara
Adoçante artificial
Mascavo

CAFÉ
Moído
Solúvel
Descafeinado
De cevada

MASSAS
Macarrão com ovos
Macarrão sem ovos
Massa para pastel
Massa com ovos para sopa
Massa para pizza
Talharim com ovos

TEMPEROS
Sal grosso
Sal refinado
Pimenta-do-reino
Massa de tomate
Alho
Vinagre de vinho
Vinagre de álcool
Maionese
Caldo de carne
Caldo de galinha

ÓLEOS E GORDURAS
Azeite de oliva
Azeite de dendê
Gordura vegetal
Óleo de soja
Óleo de girassol
Óleo de milho
Óleo de amendoim
Óleo de arroz
Banha de porco

CARNES FRESCAS E CONGELADAS
Lombinho de porco
Costelinha de porco
Alcatra
Patinho
Chã-de-dentro
Músculo
Contra-filé
Carne moída de segunda
Fígado de boi
Rabada
Mocotó

DERIVADOS DE CARNE
Toucinho de porco
Salsicha em conserva
Mortadela
Lingüiça
Carne-seca
Presunto
Salaminho
Patê de carne
Patê de presunto

PESCADOS
Camarão congelado
Peixe sardinha inteiro fresco
Peixe pescada amarela em postas
Peixe em filé pescada congelado
Caranguejo
Siri
Lula
Peixe em filé merluza congelado

AVES
Frango vivo
Peru abatido
Peito de frango
Frango congelado
Fígado de galinha
Asa de galinha

OVOS
De galinha
De codorna
De pata
De perua

FEIJÃO
Roxo
Preto
Mulatinho
Carioquinha
Fradinho
Rajado

VERDURAS, LEGUMES E TUBÉRCULOS
Batata-inglesa
Ervilha em conserva
Palmito em conserva
Alface
Espinafre
Abóbora
Cenoura
Tomate
Pimentão

FRUTAS
Maçã
Abacaxi
Laranja-lima
Banana-prata
Melão
Tangerina
Uva
Pêssego
Morango

BEBIDAS
Suco de fruta
Refrigerante de guaraná
Refrigerante de coca
Vinho tinto
Chá-mate
Aguardente
Cerveja
Água mineral

DOCES
Geléia de frutas
Sorvete
Gelatina
De frutas em calda
Bala
Bombom

REFEIÇÃO PRONTA
Marmita
Quentinha
Comida congelada
Frango assado
Salgadinho

OUTROS PRODUTOS

ALIMENTOS E ARTIGOS PARA ANIMAIS
Ração para cão
Ração para gato
Sabão para cão
Milho para galinha
Corrente para animal
Gaiola para passarinho

ARTIGOS DE HIGIENE PESSOAL E DE LIMPEZA
Creme dental
Fio dental
Escova de dentes
Sabonete
Desodorante
Absorvente higiênico
Xampu
Creme para barba
Pincel para barba
Lâmina de barbear
Detergente
Sabão em pó
Vassoura
Cera
Álcool

ARTIGOS DE PAPEL
Copo
Prato
Coador
Guardanapo
Lenço
Papel-toalha

ARTIGOS DE ILUMINAÇÃO E PILHA
Vela
Fósforos
Lâmpada

FLORES NATURAIS
Rosa
Cravo
Margarida
Violeta

63	1	Nº DO DIA	DIA DA SEMANA	DATA
		1		__/__/__

2	SITUAÇÃO DO QUADRO	3	Nº DA FOLHA
	1 ☐ PESQUISADO COM REGISTRO 3 ☐ PESQUISADO SEM REGISTRO 5 ☐ NÃO-PESQUISADO		0 1

DESCRIÇÃO DETALHADA DO PRODUTO (QUANTIDADE, UNIDADE DE MEDIDA E TIPO)	FORMA DE AQUISIÇÃO	VALOR (R$)	LOCAL DE AQUISIÇÃO
(4)	(5)	(6)	(7)

EXEMPLOS DE DESCRIÇÃO DETALHADA DO PRODUTO

1 quilograma ou quilo de frango inteiro congelado	1 molho de agrião orgânico	1 pote de 250 gramas de margarina
1 litro de leite integral	1 caixa com 450 gramas de sucrilhos	2 dúzias de ovos
250 gramas de queijo prato	1 pé de alface crespa	1 saco de 1 quilo de açúcar
1 porção de pimenta malagueta	1 unidade inteira de melancia	1 bandeja de 720 gramas de salaminho

EXEMPLOS DE LOCAIS DE AQUISIÇÃO

Supermercado	Padaria	Farmácia	Armazém	Bar	Feira-livre	Mercado
Vendedor ambulante	Peixaria	Mercearia	Loja de departamento	Varejão	Açougue	Lanchonete
Confeitaria	Quitanda	Sacolão	Adega	Sorveteria	Depósito de doces	

64	1	Nº DO DIA	DIA DA SEMANA	DATA
	2			___/___/___

2	SITUAÇÃO DO QUADRO	3	Nº DA FOLHA
1 ☐ PESQUISADO COM REGISTRO	3 ☐ PESQUISADO SEM REGISTRO	5 ☐ NÃO-PESQUISADO	0 1

DESCRIÇÃO DETALHADA DO PRODUTO (QUANTIDADE, UNIDADE DE MEDIDA E TIPO)	FORMA DE AQUISIÇÃO	VALOR (R$)	LOCAL DE AQUISIÇÃO
(4)	(5)	(6)	(7)

EXEMPLOS DE DESCRIÇÃO DETALHADA DO PRODUTO

1 quilograma ou quilo de frango inteiro congelado
1 litro de leite integral
250 gramas de queijo prato
1 porção de pimenta malagueta
1 molho de agrião orgânico
1 caixa com 450 gramas de sucrilhos
1 pé de alface crespa
1 unidade inteira de melancia
1 pote de 250 gramas de margarina
2 dúzias de ovos
1 saco de 1 quilo de açúcar
1 bandeja de 720 gramas de salaminho

EXEMPLOS DE LOCAIS DE AQUISIÇÃO

Supermercado	Padaria	Farmácia	Armazém	Bar	Feira-livre	Mercado
Vendedor ambulante	Peixaria	Mercearia	Loja de departamento	Varejão	Açougue	Lanchonete
Confeitaria	Quitanda	Sacolão	Adega	Sorveteria	Depósito de doces	

65	1 Nº DO DIA	DIA DA SEMANA	DATA
	3		___/___/___

2 SITUAÇÃO DO QUADRO	3 Nº DA FOLHA
1 ☐ PESQUISADO COM REGISTRO 3 ☐ PESQUISADO SEM REGISTRO 5 ☐ NÃO-PESQUISADO	0 1

DESCRIÇÃO DETALHADA DO PRODUTO (QUANTIDADE, UNIDADE DE MEDIDA E TIPO)	FORMA DE AQUISIÇÃO	VALOR (R$)	LOCAL DE AQUISIÇÃO
(4)	(5)	(6)	(7)

EXEMPLOS DE DESCRIÇÃO DETALHADA DO PRODUTO

1 quilograma ou quilo de frango inteiro congelado
1 litro de leite integral
250 gramas de queijo prato
1 porção de pimenta malagueta
1 molho de agrião orgânico
1 caixa com 450 gramas de sucrilhos
1 pé de alface crespa
1 unidade inteira de melancia
1 pote de 250 gramas de margarina
2 dúzias de ovos
1 saco de 1 quilo de açúcar
1 bandeja de 720 gramas de salaminho

EXEMPLOS DE LOCAIS DE AQUISIÇÃO

Supermercado
Vendedor ambulante
Confeitaria
Padaria
Peixaria
Quitanda
Farmácia
Mercearia
Sacolão
Armazém
Loja de departamento
Adega
Bar
Varejão
Sorveteria
Feira-livre
Açougue
Depósito de doces
Mercado
Lanchonete

66	1	Nº DO DIA	DIA DA SEMANA	DATA
		4	___	___ / ___ / ___

2	SITUAÇÃO DO QUADRO	3	Nº DA FOLHA
	1 ☐ PESQUISADO COM REGISTRO 3 ☐ PESQUISADO SEM REGISTRO 5 ☐ NÃO-PESQUISADO		0 1

DESCRIÇÃO DETALHADA DO PRODUTO (QUANTIDADE, UNIDADE DE MEDIDA E TIPO)	FORMA DE AQUISIÇÃO	VALOR (R$)	LOCAL DE AQUISIÇÃO
(4)	(5)	(6)	(7)

EXEMPLOS DE DESCRIÇÃO DETALHADA DO PRODUTO

1 quilograma ou quilo de frango inteiro congelado
1 litro de leite integral
250 gramas de queijo prato
1 porção de pimenta malagueta
1 molho de agrião orgânico
1 caixa com 450 gramas de sucrilhos
1 pé de alface crespa
1 unidade inteira de melancia
1 pote de 250 gramas de margarina
2 dúzias de ovos
1 saco de 1 quilo de açúcar
1 bandeja de 720 gramas de salaminho

EXEMPLOS DE LOCAIS DE AQUISIÇÃO

Supermercado	Padaria	Farmácia	Armazém	Bar	Feira-livre	Mercado
Vendedor ambulante	Peixaria	Mercearia	Loja de departamento	Varejão	Açougue	Lanchonete
Confeitaria	Quitanda	Sacolão	Adega	Sorveteria	Depósito de doces	

67	1 Nº DO DIA	DIA DA SEMANA	DATA
	5		___/___/___

2 SITUAÇÃO DO QUADRO			3 Nº DA FOLHA
1 ☐ PESQUISADO COM REGISTRO	3 ☐ PESQUISADO SEM REGISTRO	5 ☐ NÃO-PESQUISADO	0 1

DESCRIÇÃO DETALHADA DO PRODUTO (QUANTIDADE, UNIDADE DE MEDIDA E TIPO)	FORMA DE AQUISIÇÃO	VALOR (R$)	LOCAL DE AQUISIÇÃO
(4)	(5)	(6)	(7)

EXEMPLOS DE DESCRIÇÃO DETALHADA DO PRODUTO

1 quilograma ou quilo de frango inteiro congelado
1 litro de leite integral
250 gramas de queijo prato
1 porção de pimenta malagueta
1 molho de agrião orgânico
1 caixa com 450 gramas de sucrilhos
1 pé de alface crespa
1 unidade inteira de melancia
1 pote de 250 gramas de margarina
2 dúzias de ovos
1 saco de 1 quilo de açúcar
1 bandeja de 720 gramas de salaminho

EXEMPLOS DE LOCAIS DE AQUISIÇÃO

Supermercado
Vendedor ambulante
Confeitaria
Padaria
Peixaria
Quitanda
Farmácia
Mercearia
Sacolão
Armazém
Loja de departamento
Adega
Bar
Varejão
Sorveteria
Feira-livre
Açougue
Depósito de doces
Mercado
Lanchonete

68	1 Nº DO DIA	DIA DA SEMANA	DATA
	6		/ /

2 SITUAÇÃO DO QUADRO	3 Nº DA FOLHA
1 PESQUISADO COM REGISTRO 3 PESQUISADO SEM REGISTRO 5 NÃO-PESQUISADO	0 1

DESCRIÇÃO DETALHADA DO PRODUTO (QUANTIDADE, UNIDADE DE MEDIDA E TIPO)	FORMA DE AQUISIÇÃO	VALOR (R$)	LOCAL DE AQUISIÇÃO
(4)	(5)	(6)	(7)

EXEMPLOS DE DESCRIÇÃO DETALHADA DO PRODUTO

1 quilograma ou quilo de frango inteiro congelado
1 litro de leite integral
250 gramas de queijo prato
1 porção de pimenta malagueta
1 molho de agrião orgânico
1 caixa com 450 gramas de sucrilhos
1 pé de alface crespa
1 unidade inteira de melancia
1 pote de 250 gramas de margarina
2 dúzias de ovos
1 saco de 1 quilo de açúcar
1 bandeja de 720 gramas de salaminho

EXEMPLOS DE LOCAIS DE AQUISIÇÃO

Supermercado
Vendedor ambulante
Confeitaria
Padaria
Peixaria
Quitanda
Farmácia
Mercearia
Sacolão
Armazém
Loja de departamento
Adega
Bar
Varejão
Sorveteria
Feira-livre
Açougue
Depósito de doces
Mercado
Lanchonete

69

1	Nº DO DIA	DIA DA SEMANA	DATA
	7		___/___/___

2	SITUAÇÃO DO QUADRO	3	Nº DA FOLHA
	1 ☐ PESQUISADO COM REGISTRO 3 ☐ PESQUISADO SEM REGISTRO 5 ☐ NÃO-PESQUISADO		0 1

DESCRIÇÃO DETALHADA DO PRODUTO (QUANTIDADE, UNIDADE DE MEDIDA E TIPO)	FORMA DE AQUISIÇÃO	VALOR (R$)	LOCAL DE AQUISIÇÃO
(4)	(5)	(6)	(7)

EXEMPLOS DE DESCRIÇÃO DETALHADA DO PRODUTO

1 quilograma ou quilo de frango inteiro congelado
1 litro de leite integral
250 gramas de queijo prato
1 porção de pimenta malagueta
1 molho de agrião orgânico
1 caixa com 450 gramas de sucrilhos
1 pé de alface crespa
1 unidade inteira de melancia
1 pote de 250 gramas de margarina
2 dúzias de ovos
1 saco de 1 quilo de açúcar
1 bandeja de 720 gramas de salaminho

EXEMPLOS DE LOCAIS DE AQUISIÇÃO

Supermercado, Vendedor ambulante, Confeitaria, Padaria, Peixaria, Quitanda, Farmácia, Mercearia, Sacolão, Armazém, Loja de departamento, Adega, Bar, Varejão, Sorveteria, Feira-livre, Açougue, Depósito de doces, Mercado, Lanchonete

OBSERVAÇÕES

FOLHA SUPLEMENTAR DO POF 3 - CADERNETA DE AQUISIÇÃO COLETIVA

IDENTIFICAÇÃO E CONTROLE DO QUESTIONÁRIO

SETOR	Nº DE ORDEM NA LISTAGEM	PERÍODO TEÓRICO	PERÍODO REAL	UF SEQÜENCIAL DV	CÓDIGO DO DOMICÍLIO	Nº DA UC

1 Nº DO DIA	DIA DA SEMANA	DATA
		____/____/____

2 SITUAÇÃO DO QUADRO	3 Nº DA FOLHA
1 ☐ PESQUISADO COM REGISTRO 3 ☐ PESQUISADO SEM REGISTRO 5 ☐ NÃO-PESQUISADO	

DESCRIÇÃO DETALHADA DO PRODUTO (QUANTIDADE, UNIDADE DE MEDIDA E TIPO)	FORMA DE AQUISIÇÃO	VALOR (R$)	LOCAL DE AQUISIÇÃO
(4)	(5)	(6)	(7)

EXEMPLOS DE DESCRIÇÃO DETALHADA DO PRODUTO

1 quilograma ou quilo de frango inteiro congelado
1 litro de leite de vaca integral
250 gramas de queijo prato light
1 porção de pimenta malagueta
1 molho de agrião orgânico
1 caixa com 450 gramas de sucrilhos
1 pé de alface crespa
1 unidade inteira de melancia
1 pote de 250 gramas de margarina
2 dúzias de ovos
1 saco de 1 quilo de açúcar
1 bandeja de 720 gramas de salaminho

EXEMPLOS DE LOCAIS DE AQUISIÇÃO

Supermercado
Vendedor ambulante
Confeitaria
Padaria
Peixaria
Quitanda
Farmácia
Mercearia
Sacolão
Armazém
Loja de departamento
Adega
Bar
Varejão
Sorveteria
Feira-livre
Açougue
Depósito de doces
Mercado
Lanchonete

1	Nº DO DIA	DIA DA SEMANA	DATA
			___ / ___ / ___

2	SITUAÇÃO DO QUADRO	3	Nº DA FOLHA
	1 ☐ PESQUISADO COM REGISTRO 3 ☐ PESQUISADO SEM REGISTRO 5 ☐ NÃO-PESQUISADO		

DESCRIÇÃO DETALHADA DO PRODUTO (QUANTIDADE, UNIDADE DE MEDIDA E TIPO)	FORMA DE AQUISIÇÃO	VALOR (R$)	LOCAL DE AQUISIÇÃO
(4)	(5)	(6)	(7)

EXEMPLOS DE DESCRIÇÃO DETALHADA DO PRODUTO

1 quilograma ou quilo de frango inteiro congelado	1 molho de agrião orgânico	1 pote de 250 gramas de margarina
1 litro de leite de vaca integral	1 caixa com 450 gramas de sucrilhos	2 dúzias de ovos
250 gramas de queijo prato light	1 pé de alface crespa	1 saco de 1 quilo de açúcar
1 porção de pimenta malagueta	1 unidade inteira de melancia	1 bandeja de 720 gramas de salaminho

EXEMPLOS DE LOCAIS DE AQUISIÇÃO

Supermercado	Padaria	Farmácia	Armazém	Bar	Feira-livre	Mercado
Vendedor ambulante	Peixaria	Mercearia	Loja de departamento	Varejão	Açougue	Lanchonete
Confeitaria	Quitanda	Sacolão	Adega	Sorveteria	Depósito de doces	

Ministério do Planejamento, Orçamento e Gestão

IBGE
Instituto Brasileiro de Geografia e Estatística

Diretoria de Pesquisas
Coordenação de Trabalho e Rendimento
Gerência da Pesquisa de Orçamentos Familiares

Pesquisa de Orçamentos Familiares 2008 - 2009

POF 4 - Questionário de Aquisição Individual

21 IDENTIFICAÇÃO E CONTROLE DO QUESTIONÁRIO

01 IDENTIFICAÇÃO GERAL

UF	MUNICÍPIO	DISTRI-TO	SUBDIS-TRITO	SETOR	Nº DE ORDEM NA LISTAGEM

02 IDENTIFICAÇÃO POF

UF	SEQÜENCIAL	DV	CÓDIGO DO DOMICÍLIO	PERÍODO TEÓRICO	PERÍODO REAL	Nº DA UC	Nº DE ORDEM DO INFORMANTE

NOME DO INFORMANTE ....

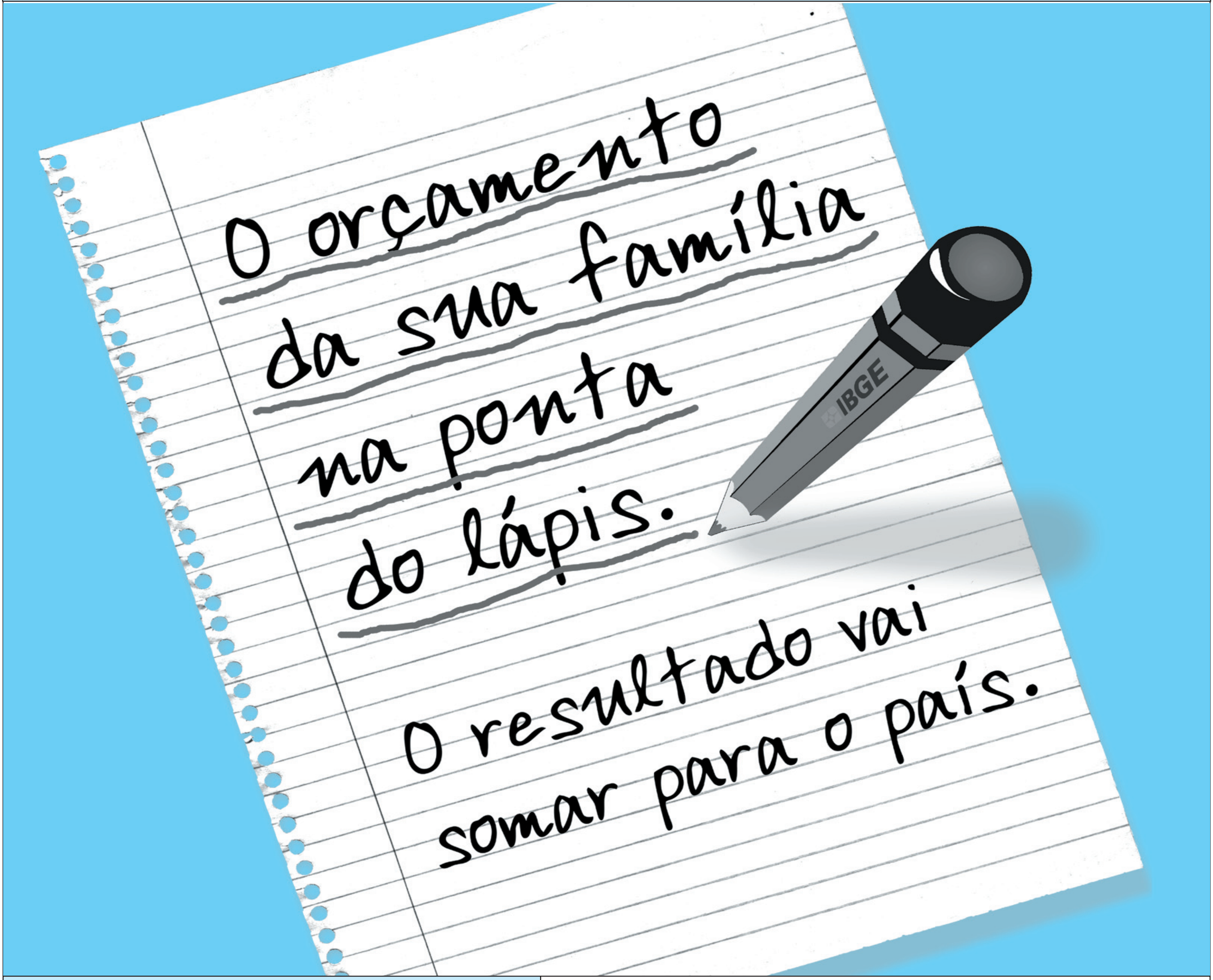

PERÍODOS DE REFERÊNCIA

PERÍODO DE 7 DIAS		a	
PERÍODO DE 30 DIAS		a	
PERÍODO DE 90 DIAS		a	
PERÍODO DE 12 MESES		a	

FORMA DE AQUISIÇÃO

Escreva, nesta coluna, o código correspondente à Forma de Aquisição para cada um dos produtos ou serviços relacionados:

01 - Monetária à vista para a Unidade de Consumo
02 - Monetária à vista para Outra Unidade de Consumo
03 - Monetária a prazo para a Unidade de Consumo
04 - Monetária a prazo para Outra Unidade de Consumo
05 - Cartão de crédito à vista para a Unidade de Consumo
06 - Cartão de crédito à vista para Outra Unidade de Consumo
07 - Doação
08 - Retirada do Negócio
09 - Troca
10 - Produção Própria
11 - Outra

Por lei, todas as informações prestadas para as pesquisas do IBGE têm caráter confidencial e só podem ser utilizadas para fins estatísticos (Lei 5534 de 14/11/1968).

22	COMUNICAÇÕES NO PERÍODO DE REFERÊNCIA DE 7 DIAS

SITUAÇÃO DO QUADRO 1 ☐ PESQUISADO COM REGISTRO 3 ☐ PESQUISADO SEM REGISTRO 5 ☐ NÃO-PESQUISADO

TIPO	FORMA DE AQUISIÇÃO	VALOR
(1)	(2)	(3)
TELEFONE PÚBLICO		,
CORREIO (carta, selo, etc.)		,
TELEMENSAGEM		,
TELEGRAMA FONADO		,
ACESSO À INTERNET		,
		,
		,
		,
		,
		,
		,
		,
		,
		,
		,
		,
		,
		,
		,
		,
		,
		,
		,
		,
		,
		,

23 TRANSPORTES COLETIVOS E PRÓPRIOS NO PERÍODO DE REFERÊNCIA DE 7 DIAS

SITUAÇÃO DO QUADRO 1 ☐ PESQUISADO COM REGISTRO 3 ☐ PESQUISADO SEM REGISTRO 5 ☐ NÃO-PESQUISADO

TIPO	FORMA DE AQUISIÇÃO	VALOR
(1)	(2)	(3)
ÔNIBUS URBANO CONVENCIONAL		,
ÔNIBUS INTERMUNICIPAL CONVENCIONAL		,
ÔNIBUS INTERESTADUAL CONVENCIONAL		,
ÔNIBUS URBANO RÁPIDO		,
ÔNIBUS INTERESTADUAL COM AR-CONDICIONADO		,
TRANSPORTE ALTERNATIVO		,
TÁXI		,
METRÔ		,
TREM		,
BARCA		,
AEROBARCO		,
GASOLINA COMUM (combustível de veículo)		,
GASOLINA ESPECIAL (combustível de veículo)		,
ÁLCOOL (combustível de veículo)		,
ÓLEO DIESEL (combutível de veículo)		,
GÁS VEICULAR		,
ESTACIONAMENTO		,
PEDÁGIO		,
INTEGRAÇÃO ÔNIBUS-METRÔ		,
INTEGRAÇÃO TREM-METRÔ		,
INTEGRAÇÃO TREM-ÔNIBUS		,
LAVAGEM SEM LUBRIFICAÇÃO		,
		,
		,
		,
		,

24 | **ALIMENTAÇÃO FORA DE CASA NO PERÍODO DE REFERÊNCIA DE 7 DIAS**

SITUAÇÃO DO QUADRO 1 ☐ PESQUISADO COM REGISTRO 3 ☐ PESQUISADO SEM REGISTRO 5 ☐ NÃO-PESQUISADO

TIPO	FORMA DE AQUISIÇÃO	VALOR	LOCAL DE AQUISIÇÃO
(1)	(2)	(3)	(4)
ALMOÇO OU JANTAR A PESO		,	
ALMOÇO OU JANTAR À LA CARTE		,	
ALMOÇO OU JANTAR FAST FOOD		,	
ALMOÇO DA ESCOLA		,	
JANTAR DA ESCOLA		,	
LANCHE DA ESCOLA		,	
CAFÉ DA ESCOLA		,	
REFRIGERANTE DE COLA		,	
CERVEJA CLARA OU PRETA		,	
SUCO DE FRUTAS OU VEGETAIS		,	
CAFEZINHO		,	
PÃO COM OU SEM MANTEIGA		,	
PÃO INTEGRAL LIGHT COM OU SEM MANTEIGA		,	
QUEIJO PRATO LIGHT		,	
BISCOITO SALGADO		,	
SANDUÍCHE		,	
BALA OU CHICLETE		,	
SORVETE DE QUALQUER SABOR DIET		,	
CHOCOLATE EM BARRA, TABLETE OU BOMBOM		,	
FRUTA		,	
LEITE DE VACA ORGÂNICO		,	
QUEIJO DE MINAS FRESCAL ORGÂNICO		,	
		,	
		,	
		,	
		,	

25 AQUISIÇÃO DE ARTIGOS DE FUMO NO PERÍODO DE REFERÊNCIA DE 7 DIAS

SITUAÇÃO DO QUADRO 1 ☐ PESQUISADO COM REGISTRO 3 ☐ PESQUISADO SEM REGISTRO 5 ☐ NÃO-PESQUISADO

TIPO	FORMA DE AQUISIÇÃO	VALOR	LOCAL DE AQUISIÇÃO
(1)	(2)	(3)	(4)
CIGARRO		,	
FÓSFORO		,	
FUMO DESFIADO		,	
FUMO DE ROLO		,	
PALHA PARA CIGARRO		,	
CHARUTO		,	
CIGARRILHA		,	
		,	
		,	
		,	
		,	
		,	
		,	
		,	
		,	
		,	
		,	
		,	
		,	
		,	
		,	
		,	
		,	
		,	
		,	
		,	

26	JOGOS E APOSTAS NO PERÍODO DE REFERÊNCIA DE 7 DIAS

SITUAÇÃO DO QUADRO 1 ☐ PESQUISADO COM REGISTRO 3 ☐ PESQUISADO SEM REGISTRO 5 ☐ NÃO-PESQUISADO

TIPO	FORMA DE AQUISIÇÃO	VALOR	LOCAL DE AQUISIÇÃO
(1)	(2)	(3)	(4)
MEGA SENA		,	
JOGO DO BICHO		,	
LOTERIA FEDERAL E ESTADUAL		,	
LOTERIA ESPORTIVA		,	
TELESENA		,	
RASPADINHA		,	
QUINA		,	
BINGO		,	
RIFA		,	
LOTOMANIA		,	
		,	
		,	
		,	
		,	
		,	
		,	
		,	
		,	
		,	
		,	
		,	
		,	
		,	
		,	
		,	
		,	
		,	

27	AQUISIÇÃO DE JORNAIS, REVISTAS E PASSATEMPOS IMPRESSOS NO PERÍODO DE REFERÊNCIA DE 7 DIAS

SITUAÇÃO DO QUADRO 1 ☐ PESQUISADO COM REGISTRO 3 ☐ PESQUISADO SEM REGISTRO 5 ☐ NÃO-PESQUISADO

TIPO	FORMA DE AQUISIÇÃO	VALOR	LOCAL DE AQUISIÇÃO
(1)	(2)	(3)	(4)
JORNAL		,	
REVISTA INFANTIL		,	
FOTONOVELA		,	
REVISTA ESPORTIVA		,	
REVISTA MASCULINA		,	
REVISTA DE CASA E DECORAÇÃO		,	
REVISTA DE CARROS		,	
ÁLBUM E FIGURINHAS		,	
		,	
		,	
		,	
		,	
		,	
		,	
		,	
		,	
		,	
		,	
		,	
		,	
		,	
		,	
		,	
		,	
		,	
		,	

28 DIVERSÕES, INGRESSOS PARA EVENTOS ESPORTIVOS E CULTURAIS E USO DE CELULARES NO PERÍODO DE REFERÊNCIA DE 30 DIAS

SITUAÇÃO DO QUADRO 1 ☐ PESQUISADO COM REGISTRO 3 ☐ PESQUISADO SEM REGISTRO 5 ☐ NÃO-PESQUISADO

TIPO	FORMA DE AQUISIÇÃO	VALOR	LOCAL DE AQUISIÇÃO
(1)	(2)	(3)	(4)
CINEMA		,	
TEATRO		,	
ALUGUEL DE DVD (disco)		,	
DISCO LASER		,	
FITA DE VIDEOCASSETE GRAVADA		,	
FITA DE VIDEOCASSETE VIRGEM		,	
ALUGUEL DE FITA DE VIDEOCASSETE		,	
FITA DE VIDEOGAME		,	
ALUGUEL DE FITA DE VIDEOGAME		,	
FITA CASSETE GRAVADA		,	
FITA CASSETE VIRGEM		,	
CLUBE (mensalidade e taxa)		,	
REVELAÇÃO E CÓPIA		,	
FILME E FLASH DESCARTÁVEL		,	
FUTEBOL		,	
MOTEL		,	
BOITE, DANCETERIA, DISCOTECA, ETC.		,	
CARTÃO DE TELEFONE CELULAR		,	
CONTA DE TELEFONE CELULAR		,	
		,	
		,	
		,	
		,	
		,	
		,	
		,	

29	AQUISIÇÃO DE PRODUTOS FARMACÊUTICOS NO PERÍODO DE REFERÊNCIA DE **30 DIAS** (continua)

SITUAÇÃO DO QUADRO 1 ☐ PESQUISADO COM REGISTRO 3 ☐ PESQUISADO SEM REGISTRO 5 ☐ NÃO-PESQUISADO

TIPO	FORMA DE AQUISIÇÃO	VALOR	LOCAL DE AQUISIÇÃO	CARAC-TERÍS-TICA
(1)	(2)	(3)	(4)	(5)
**REMÉDIOS**				
PARA DOR E FEBRE (Analgésico e Antitérmico)		,		
PARA TOSSE E RESFRIADO (Antigripal e Antitussígeno)		,		
PARA ALERGIA (Antialérgico)		,		
PARA AZIA (Antiácido)		,		
PARA ENJÔO E VÔMITO (Antiemético)		,		
PARA PRISÃO DE VENTRE (Laxante)		,		
PARA INFLAMAÇÃO E REUMATISMO (Antiinflamatório e Anti-reumático)		,		
PARA INFECÇÃO		,		
PARA DIABETE		,		
PARA PRESSÃO ALTA (Anti-hipertensivo)		,		
PARA PROBLEMAS CARDÍACOS E CIRCULATÓRIOS		,		
REDUTOR DE COLESTEROL OU TRIGLICERÍDEOS		,		
PARA VERMES (Vermífugo)		,		
PARA DEPRESSÃO (Antidepressivo)		,		
VITAMINAS		,		
ANTICONCEPCIONAL		,		
HORMÔNIO		,		
PARA SISTEMA NERVOSO		,		
PARA PROBLEMAS DE PELE		,		
PARA PROBLEMAS GINECOLÓGICOS		,		
PARA DIARRÉIA		,		
PARA PROBLEMAS DE BOCA, OUVIDO, NARIZ E GARGANTA		,		

29	AQUISIÇÃO DE PRODUTOS FARMACÊUTICOS NO PERÍODO DE REFERÊNCIA DE 30 DIAS (conclusão)			
TIPO	FORMA DE AQUISIÇÃO	VALOR	LOCAL DE AQUISIÇÃO	CARAC-TERÍS-TICA
(1)	(2)	(3)	(4)	(5)
**REMÉDIOS**				
PARA PROBLEMAS OCULARES (Oftalmológicos)		,		
CHÁS E REMÉDIOS ALTERNATIVOS		,		
		,		
		,		
		,		
		,		
**OUTROS PRODUTOS FARMACÊUTICOS**				
MATERIAL PARA CURATIVO		,		
ANTI-SÉPTICOS E DESINFETANTES TÓPICOS		,		
MATERIAL PARA CUIDADO COM BEBÊ		,		
MATERIAL PARA AUTODIAGNÓSTICO		,		
PRODUTOS PARA CUIDADOS BUCAIS E DENTAIS		,		
MATERIAL PARA APOIO TERAPÊUTICO		,		
PRESERVATIVO E LUBRIFICANTE ÍNTIMO		,		
MATERIAIS PARA CUIDADOS COM IDOSOS		,		
		,		
		,		
		,		
		,		
		,		

CARACTERÍSTICA

1 - de marca
2 - genérico
3 - plantas e ervas medicinais
4 - manipulado
9 - não sabe
5 - homeopático

ATENÇÃO: RESTRIÇÃO ORÇAMENTÁRIA

_____ teve necessidade de algum medicamento no período de referência de 30 dias e não o adquiriu por falta de dinheiro?

1 ☐ SIM 2 ☐ NÃO 9 ☐ NÃO SABE

30 AQUISIÇÃO DE ARTIGOS DE HIGIENE PESSOAL E MAQUIAGEM NO PERÍODO DE REFERÊNCIA DE 30 DIAS

SITUAÇÃO DO QUADRO 1 ☐ PESQUISADO COM REGISTRO 3 ☐ PESQUISADO SEM REGISTRO 5 ☐ NÃO-PESQUISADO

TIPO	FORMA DE AQUISIÇÃO	VALOR	LOCAL DE AQUISIÇÃO
(1)	(2)	(3)	(4)
PERFUME		,	
CREME DE PELE E BRONZEADOR		,	
ARTIGOS DE MAQUIAGEM		,	
ESMALTE, BASE, ACETONA, ETC.		,	
ESCOVA DE CABELOS E PENTE		,	
TALCO		,	
GRAMPO, PREGADOR DE CABELO, ETC.		,	
PRODUTOS DE LIMPEZA DE PELE		,	
ALICATE, CORTADOR DE UNHA, ETC.		,	
ESTOJO (talco, sabonete, perfume, etc.)		,	
ESTOJO DE MANICURO		,	
		,	
		,	
		,	
		,	
		,	
		,	
		,	
		,	
		,	
		,	
		,	
		,	
		,	
		,	
		,	

31	SERVIÇOS DE CUIDADOS PESSOAIS E OUTROS NO PERÍODO DE REFERÊNCIA DE **90 DIAS**

SITUAÇÃO DO QUADRO 1 ☐ PESQUISADO COM REGISTRO 3 ☐ PESQUISADO SEM REGISTRO 5 ☐ NÃO-PESQUISADO

TIPO	FORMA DE AQUISIÇÃO	VALOR
(1)	(2)	(3)
CABELEIREIRO		,
BARBEIRO		,
MANICURO E PEDICURO		,
COSTUREIRA		,
ALFAIATE		,
BORDADEIRA		,
CERZIDEIRA		,
SAPATEIRO		,
TINTURARIA E LAVANDERIA		,
RELOJOEIRO		,
ENGRAXATE		,
MASSAGEM E SAUNA		,
MASSAGEM		,
SAUNA		,
BARBA		,
CORTE DE CABELO		,
DEPILAÇÃO		,
JOALHEIRO		,
		,
		,
		,
		,
		,
		,
		,
		,

32 **AQUISIÇÃO DE ARTIGOS DE PAPELARIA, LIVROS NÃO-DIDÁTICOS E ASSINATURA DE PERIÓDICOS NO PERÍODO DE REFERÊNCIA DE 90 DIAS**

SITUAÇÃO DO QUADRO 1 ☐ PESQUISADO COM REGISTRO 3 ☐ PESQUISADO SEM REGISTRO 5 ☐ NÃO-PESQUISADO

TIPO	FORMA DE AQUISIÇÃO	VALOR	LOCAL DE AQUISIÇÃO
(1)	(2)	(3)	(4)
CADERNO		,	
LÁPIS, BORRACHA, CANETA, ETC.		,	
ÁLBUM DE FOTOGRAFIA (exceto de cerimônia familiar)		,	
PASTA PLÁSTICA NÃO-ESCOLAR		,	
OUTROS ARTIGOS DE PAPELARIA		,	
LIVROS NÃO-DIDÁTICOS		,	
ASSINATURA DE PERIÓDICOS		,	
FOTOCÓPIA XEROX		,	
		,	
		,	
		,	
		,	
		,	
		,	
		,	
		,	
		,	
		,	
		,	
		,	
		,	
		,	
		,	
		,	
		,	

33 AQUISIÇÃO DE BRINQUEDOS E MATERIAL DE RECREAÇÃO NO PERÍODO DE REFERÊNCIA DE 90 DIAS

SITUAÇÃO DO QUADRO 1 ☐ PESQUISADO COM REGISTRO 3 ☐ PESQUISADO SEM REGISTRO 5 ☐ NÃO-PESQUISADO

TIPO	FORMA DE AQUISIÇÃO	VALOR	LOCAL DE AQUISIÇÃO
(1)	(2)	(3)	(4)
BRINQUEDOS E JOGOS		,	
BONECA		,	
BOLA DE CRIANÇA		,	
PATIM, SKATE, VELOCÍPEDE		,	
EQUIPAMENTOS ESPORTIVOS		,	
MATERIAL DE CAÇA E PESCA		,	
ARMAS DE FOGO (exceto de caça)		,	
ARTIGOS PARA PRAIA		,	
BARRACA DE PRAIA		,	
CADEIRA DE PRAIA		,	
BICICLETA DE GINÁSTICA		,	
APARELHO DE MUSCULAÇÃO		,	
		,	
		,	
		,	
		,	
		,	
		,	
		,	
		,	
		,	
		,	
		,	
		,	
		,	
		,	

34	AQUISIÇÃO E ALUGUEL DE ROUPAS DE HOMEM NO PERÍODO DE REFERÊNCIA DE 90 DIAS

SITUAÇÃO DO QUADRO 1 ☐ PESQUISADO COM REGISTRO 3 ☐ PESQUISADO SEM REGISTRO 5 ☐ NÃO-PESQUISADO

TIPO	FORMA DE AQUISIÇÃO	VALOR	LOCAL DE AQUISIÇÃO
(1)	(2)	(3)	(4)
CAMISA		,	
CALÇA COMPRIDA		,	
BERMUDA, CALÇÃO E SHORT		,	
CUECA		,	
CAMISETA		,	
MEIAS		,	
AGASALHO (casaco, suéter, etc.)		,	
TERNO		,	
PALETÓ E BLAZER		,	
CONJUNTO ESPORTIVO		,	
PIJAMA E ROBE		,	
GRAVATA		,	
FARDAMENTO MILITAR		,	
UNIFORME DE TRABALHO		,	
MACACÃO		,	
CONJUNTO DE SHORT E CAMISA		,	
CONJUNTO DE CALÇA E CAMISA		,	
ALUGUEL DE ROUPA		,	
		,	
		,	
		,	
		,	
		,	
		,	
		,	
		,	

35 AQUISIÇÃO E ALUGUEL DE ROUPAS DE MULHER NO PERÍODO DE REFERÊNCIA DE 90 DIAS

SITUAÇÃO DO QUADRO 1 ☐ PESQUISADO COM REGISTRO 3 ☐ PESQUISADO SEM REGISTRO 5 ☐ NÃO-PESQUISADO

TIPO	FORMA DE AQUISIÇÃO	VALOR	LOCAL DE AQUISIÇÃO
(1)	(2)	(3)	(4)
BLUSA OU CAMISA		,	
CALÇA COMPRIDA		,	
BERMUDA E SHORT		,	
CONJUNTO DE CALCINHA E SUTIÃ		,	
CAMISETA		,	
VESTIDO		,	
SAIA		,	
MEIAS		,	
MAIÔ E BIQUÍNI		,	
CONJUNTO ESPORTIVO		,	
AGASALHO (casado, suéter, etc.)		,	
CAMISOLA, PIJAMA, ROBE, ETC.		,	
MACACÃO		,	
CONJUNTO DE SAIA E BLUSA		,	
CONJUNTO DE SHORT E BLUSA		,	
CONJUNTO DE CALÇA E BLUSA		,	
CONJUNTO DE SAIA E BLAZER		,	
VESTIDO DE NOIVA		,	
ALUGUEL DE ROUPA		,	
		,	
		,	
		,	
		,	
		,	
		,	
		,	

36 AQUISIÇÃO E ALUGUEL DE ROUPAS DE CRIANÇA ATÉ 14 ANOS NO PERÍODO DE REFERÊNCIA DE 90 DIAS

SITUAÇÃO DO QUADRO 1 ☐ PESQUISADO COM REGISTRO 3 ☐ PESQUISADO SEM REGISTRO 5 ☐ NÃO-PESQUISADO

TIPO	FORMA DE AQUISIÇÃO	VALOR	LOCAL DE AQUISIÇÃO
(1)	(2)	(3)	(4)
BLUSA OU CAMISA		,	
CALÇA COMPRIDA		,	
BERMUDA, SHORT E CALÇÃO		,	
VESTIDO		,	
CONJUNTO ESPORTIVO		,	
SAIA		,	
CAMISETA		,	
CONJUNTO DE CALCINHA E SUTIÃ		,	
CUECA		,	
MEIAS		,	
AGASALHO (casaco, suéter, etc.)		,	
SUNGA, MAIÔ E BIQUÍNI		,	
CONJUNTO DE SHORT E CAMISA		,	
CONJUNTO DE SAIA E BLUSA		,	
CONJUNTO DE CALÇA E CAMISA		,	
MACACÃO		,	
FRALDA DE TECIDO		,	
CAMISOLA, PIJAMA, ROBE, ETC.		,	
		,	
		,	
		,	
		,	
		,	
		,	
		,	
		,	

37	AQUISIÇÃO DE ARTIGOS DE ARMARINHO, TECIDOS E ROUPAS DE BANHO, CAMA E MESA NO PERÍODO DE REFERÊNCIA DE **90 DIAS**

SITUAÇÃO DO QUADRO 1 ☐ PESQUISADO COM REGISTRO 3 ☐ PESQUISADO SEM REGISTRO 5 ☐ NÃO-PESQUISADO

TIPO	FORMA DE AQUISIÇÃO	VALOR	LOCAL DE AQUISIÇÃO
(1)	(2)	(3)	(4)
ARTIGOS DE ARMARINHO		,	
TECIDOS		,	
CORTINADO		,	
TINTA DE TECIDO		,	
PLÁSTICO		,	
NAPA		,	
ROUPA DE BANHO		,	
ROUPA DE CAMA		,	
ROUPA DE MESA DE TECIDO		,	
ROUPA DE MESA DE PLÁSTICO		,	
ENCHIMENTO DE ALMOFADA, DE TRAVESSEIRO, ETC.		,	
TRAVESSEIRO		,	
		,	
		,	
		,	
		,	
		,	
		,	
		,	
		,	
		,	
		,	
		,	
		,	
		,	
		,	

38 AQUISIÇÃO E ALUGUEL DE BOLSAS, CALÇADOS, CINTOS E OUTROS ACESSÓRIOS NO PERÍODO DE REFERÊNCIA DE **90 DIAS**

SITUAÇÃO DO QUADRO 1 ☐ PESQUISADO COM REGISTRO 3 ☐ PESQUISADO SEM REGISTRO 5 ☐ NÃO-PESQUISADO

TIPO	FORMA DE AQUISIÇÃO	VALOR	LOCAL DE AQUISIÇÃO
(1)	(2)	(3)	(4)
BOLSA E CARTEIRA DE MULHER		,	
BOLSA, CARTEIRA, PASTA E MALETA DE HOMEM		,	
MALA		,	
MOCHILA (exceto escolar)		,	
TÊNIS DE HOMEM		,	
TÊNIS DE MULHER		,	
TÊNIS DE CRIANÇA		,	
SAPATO E BOTA DE HOMEM (exceto de plástico)		,	
SAPATO E BOTA DE MULHER (exceto de plástico)		,	
SAPATO E BOTA DE CRIANÇA (exceto de plástico)		,	
SANDÁLIA DE HOMEM (exceto de borracha ou plástico)		,	
SANDÁLIA DE MULHER (exceto de borracha ou plástico)		,	
SANDÁLIA DE CRIANÇA (exceto de borracha ou plástico)		,	
SANDÁLIA E SAPATO DE PLÁSTICO DE MULHER		,	
SANDÁLIA E SAPATO DE PLÁSTICO DE CRIANÇA		,	
CINTO DE MULHER		,	
CINTO DE HOMEM		,	
CHINELO		,	
SAPATILHA		,	
SANDÁLIA DE BORRACHA DE CRIANÇA		,	
SANDÁLIA DE BORRACHA DE HOMEM		,	
SANDÁLIA DE BORRACHA DE MULHER		,	
		,	
		,	
		,	
		,	

39 AQUISIÇÃO DE UTENSÍLIOS AVULSOS E ARTIGOS DE BANHEIRO, COPA E COZINHA NO PERÍODO DE REFERÊNCIA DE 90 DIAS

SITUAÇÃO DO QUADRO 1 ☐ PESQUISADO COM REGISTRO 3 ☐ PESQUISADO SEM REGISTRO 5 ☐ NÃO-PESQUISADO

TIPO	FORMA DE AQUISIÇÃO	VALOR	LOCAL DE AQUISIÇÃO
(1)	(2)	(3)	(4)
UTENSÍLIOS DE PLÁSTICO		,	
UTENSÍLIOS DE ISOPOR		,	
UTENSÍLIOS DE MADEIRA		,	
FRIGIDEIRA, LEITEIRA, ETC.		,	
TALHERES		,	
BOTIJÃO DE GÁS VAZIO		,	
BANDEJA DE INOX		,	
OUTROS UTENSÍLIOS DE METAL		,	
COPO DE VIDRO COMUM		,	
PRATO DE LOUÇA		,	
XÍCARA COMUM		,	
TRAVESSA DE VIDRO OU LOUÇA		,	
OUTROS UTENSÍLIOS DE VIDRO OU LOUÇA		,	
GARRAFA TÉRMICA		,	
CAPACHO		,	
		,	
		,	
		,	
		,	
		,	
		,	
		,	
		,	
		,	
		,	
		,	

40 OUTRAS AQUISIÇÕES NO PERÍODO DE REFERÊNCIA DE 90 DIAS

SITUAÇÃO DO QUADRO 1 ☐ PESQUISADO COM REGISTRO 3 ☐ PESQUISADO SEM REGISTRO 5 ☐ NÃO-PESQUISADO

TIPO	FORMA DE AQUISIÇÃO	VALOR	LOCAL DE AQUISIÇÃO
(1)	(2)	(3)	(4)
BIJUTERIA		,	
ÓCULOS SEM GRAU		,	
TRANSPORTE DE MERCADORIAS		,	
BATERIA DE RELÓGIO, BRINQUEDO, APARELHO AUDITIVO, ETC.		,	
PINCEL, TINTA E VERNIZ PARA PINTURA DE TELA, VASO, ETC.		,	
TELA PARA PINTURA		,	
SELO (hobby)		,	
CALENDÁRIO		,	
MONÓCULO		,	
CAVALETE DE PINTURA DE TELA		,	
MÁSCARA PROTETORA (proteção profissional)		,	
CANIVETE		,	
PORTA-ÓCULOS		,	
COFRE		,	
CHAVEIRO		,	
PARAFINA		,	
BOMBA E CUIA OU PORONGO DE CHIMARRÃO		,	
		,	
		,	
		,	
		,	
		,	
		,	
		,	
		,	
		,	

41 **VIAGENS NO PERÍODO DE REFERÊNCIA DE 90 DIAS**

SITUAÇÃO DO QUADRO 1 ☐ PESQUISADO COM REGISTRO 3 ☐ PESQUISADO SEM REGISTRO 5 ☐ NÃO-PESQUISADO

TIPO	FORMA DE AQUISIÇÃO	VALOR	MOTIVO DA VIAGEM	UNIDADE DA FEDERAÇÃO
(1)	(2)	(3)	(4)	(5)
ALIMENTAÇÃO		,		
ALOJAMENTO		,		
ÔNIBUS URBANO		,		
ÔNIBUS INTERMUNICIPAL		,		
ÔNIBUS INTERNACIONAL		,		
ÔNIBUS INTERESTADUAL		,		
AVIÃO		,		
NAVIO		,		
TÁXI		,		
TREM		,		
BARCO		,		
ALUGUEL DE VEÍCULO SEM CONDUTOR		,		
COMBUSTÍVEL DE VEÍCULO		,		
PEDÁGIO		,		
ALUGUEL DE IMÓVEIS POR TEMPORADA		,		
ATIVIDADES CULTURAIS (INGRESSO)		,		
ATIVIDADES DESPORTIVAS (INGRESSO)		,		
PACOTES TURÍSTICOS NACIONAIS		,		
PACOTES TURÍSTICOS INTERNACIONAIS		,		
		,		
		,		
		,		

MOTIVO DA VIAGEM

1 - lazer, recreio e férias
2 - visita a parentes e amigos
3 - negócios e motivos profissionais
4 - educação
5 - tratamentos médicos
6 - religião, peregrinações
7 - outros motivos
9 - não sabe

UNIDADE DA FEDERAÇÃO

11 - Rondônia
12 - Acre
13 - Amazonas
14 - Roraima
15 - Pará
16 - Amapá
17 -Tocantins
21 - Maranhão
22 - Piauí
23 - Ceará
24 - R. G. do Norte
25 - Paraíba
26 - Pernambuco
27 - Alagoas
28 - Sergipe
29 - Bahia
31 - Minas Gerais
32 - Espírito Santo
33 - R. de Janeiro
35 - São Paulo
41 - Paraná
42 - Sta Catarina
43 - R. G. do Sul
50 - M. G. do Sul
51 - Mato Grosso
52 - Goiás
53 - Distrito Federal
60 - Fora do País
99 - Não sabe

42	SERVIÇOS DE ASSISTÊNCIA À SAÚDE NO PERÍODO DE REFERÊNCIA DE **90 DIAS** (continua)

SITUAÇÃO DO QUADRO 1 ☐ PESQUISADO COM REGISTRO 3 ☐ PESQUISADO SEM REGISTRO 5 ☐ NÃO-PESQUISADO

TIPO	FORMA DE AQUISIÇÃO	VALOR	LOCAL DE AQUISIÇÃO
(1)	(2)	(3)	(4)
PLANO DE ASSISTÊNCIA MÉDICA (empresa)		,	
PLANO DE ASSISTÊNCIA MÉDICA (particular)		,	
PLANO EXCLUSIVAMENTE ODONTOLÓGICO (empresa)		,	
PLANO EXCLUSIVAMENTE ODONTOLÓGICO (particular)		,	
MENSALIDADE DE CLÍNICA		,	
ATENDIMENTO EM PRONTO-SOCORRO/PRONTO-ATENDIMENTO		,	
SERVIÇOS DE CIRURGIA, ANESTESIA E OBSTETRÍCIA		,	
HOSPITALIZAÇÃO		,	
AMBULÂNCIA E UTI MÓVEL (remoção)		,	
CONSULTA MÉDICA COM CLÍNICO GERAL		,	
CONSULTA MÉDICA PEDIÁTRICA		,	
CONSULTA MÉDICA GINECO-OBSTETRÍCIA		,	
OUTRAS CONSULTAS DIFERENTES DE GERAL, PEDIÁTRICA E GINECO-OBSTETRÍCIA		,	
EXAME DE LABORATÓRIO		,	
TOMOGRAFIA		,	
RESSONÂNCIA MAGNÉTICA		,	
ULTRASSONOGRAFIA		,	
RADIOGRAFIA		,	
ELETROCARDIOGRAMA E OUTROS MÉTODOS GRÁFICOS		,	
SERVIÇO DE HEMOTERAPIA (transfusão)		,	
PRÓTESE E APARELHO DENTÁRIO		,	
SERVIÇOS DE ENFERMAGEM		,	
NUTRICIONISTA		,	
PSICÓLOGO E PSICOTERAPEUTA		,	
TRATAMENTO FISIOTERÁPICO E DE TERAPIA OCUPACIONAL		,	
TRATAMENTO FONOAUDIOLÓGICO		,	
ACUPUNTURA		,	

42	SERVIÇOS DE ASSISTÊNCIA À SAÚDE NO PERÍODO DE REFERÊNCIA DE 90 DIAS (conclusão)		
TIPO	FORMA DE AQUISIÇÃO	VALOR	LOCAL DE AQUISIÇÃO
(1)	(2)	(3)	(4)
CURANDEIRO, CURIOSO E PARTEIRA		,	
CLÍNICA E RESIDÊNCIA PARA IDOSOS		,	
CLÍNICA PSIQUIÁTRICA OU PARA DEPENDENTES QUÍMICOS		,	
ARMAÇÃO DE ÓCULOS		,	
ARTIGOS ORTOPÉDICOS E PRÓTESES DIVERSAS		,	
ALUGUEL E REPARO DE APARELHO MÉDICO		,	
EXAMES EM POSTOS OU PROFISSIONAIS VOLANTES		,	
		,	
		,	
		,	
		,	
		,	
		,	
		,	
		,	
		,	
		,	
		,	
		,	
		,	
		,	
		,	
		,	
		,	
		,	

ATENÇÃO: RESTRIÇÃO ORÇAMENTÁRIA

_______ precisou de algum serviço de saúde no período de referência de 90 dias e não usou por falta de dinheiro?

1 ☐ SIM 2 ☐ NÃO 9 ☐ NÃO SABE

43 **ACESSÓRIOS E MANUTENÇÃO DE VEÍCULOS NO PERÍODO DE REFERÊNCIA DE 90 DIAS**

SITUAÇÃO DO QUADRO 1 ☐ PESQUISADO COM REGISTRO 3 ☐ PESQUISADO SEM REGISTRO 5 ☐ NÃO-PESQUISADO

TIPO	FORMA DE AQUISIÇÃO	VALOR	LOCAL DE AQUISIÇÃO
(1)	(2)	(3)	(4)
CONSERTO (peça + mão-de-obra)		,	
CONSERTO (mão-de-obra)		,	
PEÇA ELÉTRICA OU MECÂNICA		,	
LUBRIFICAÇÃO (com ou sem lavagem)		,	
CÂMARA E PNEU		,	
ÓLEO LUBRIFICANTE (complementação ou troca)		,	
BATERIA		,	
TOCA-FITAS		,	
RÁDIO COM CD		,	
RÁDIO		,	
AMPLIFICADOR		,	
AR-CONDICIONADO		,	
MOTOR DE AUTOMÓVEL		,	
ADITIVOS		,	
PINTURA (material e mão-de-obra)		,	
OUTRAS PEÇAS E ACESSÓRIOS (veículos motorizados)		,	
REBOQUE		,	
		,	
		,	
		,	
		,	
		,	
		,	
		,	
		,	
		,	

44 SERVIÇOS BANCÁRIOS, DE CARTÓRIO, DE ADVOGADO, DE DESPACHANTE E SIMILARES NO PERÍODO DE REFERÊNCIA DE 90 DIAS

SITUAÇÃO DO QUADRO 1 ☐ PESQUISADO COM REGISTRO 3 ☐ PESQUISADO SEM REGISTRO 5 ☐ NÃO-PESQUISADO

TIPO	FORMA DE AQUISIÇÃO	VALOR
(1)	(2)	(3)
ANUIDADE DE CARTÃO DE CRÉDITO		,
TALÃO DE CHEQUES		,
EXTRATO BANCÁRIO		,
CADASTRO BANCÁRIO		,
TRANSFERÊNCIA INTERBANCÁRIA		,
CARTÃO ESPECIAL (taxa)		,
TARIFA DE CONTA BANCÁRIA		,
TAXA DE SAQUE ELETRÔNICO		,
ENCARGOS FINANCEIROS DE CHEQUE ESPECIAL		,
CARTÓRIO		,
ADVOGADO		,
DESPACHANTE		,
COMISSÃO DE IMOBILIÁRIA		,
CONTADOR		,
ENGENHEIRO		,
CORRETOR		,
ENCARGOS FINANCEIROS DE CARTÃO DE CRÉDITO		,
		,
		,
		,
		,
		,
		,
		,
		,
		,

45 CERIMÔNIAS FAMILIARES, PRÁTICAS RELIGIOSAS, OUTRAS FESTAS E RECEPÇÕES NO PERÍODO DE REFERÊNCIA DE **12 MESES**

SITUAÇÃO DO QUADRO 1 ☐ PESQUISADO COM REGISTRO 3 ☐ PESQUISADO SEM REGISTRO 5 ☐ NÃO-PESQUISADO

TIPO	FORMA DE AQUISIÇÃO	VALOR
(1)	(2)	(3)
CASAMENTO		,
OUTRAS CERIMÔNIAS RELIGIOSAS		,
FUNERAL E ARTIGOS RELIGIOSOS		,
ANIVERSÁRIO		,
OUTRAS FESTAS OU RECEPÇÕES		,
FOTOGRAFIAS DE CERIMÔNIAS (fotógrafo profissional)		,
FILMAGEM DE CERIMÔNIA		,
		,
		,
		,
		,
		,
		,
		,
		,
		,
		,
		,
		,
		,
		,
		,
		,
		,
		,

46	AQUISIÇÕES DE JÓIAS, RELÓGIOS, APARELHOS E ACESSÓRIOS DE TELEFONIA CELULAR NO PERÍODO DE REFERÊNCIA DE **12 MESES**

SITUAÇÃO DO QUADRO 1 ☐ PESQUISADO COM REGISTRO 3 ☐ PESQUISADO SEM REGISTRO 5 ☐ NÃO-PESQUISADO

TIPO	FORMA DE AQUISIÇÃO	VALOR	LOCAL DE AQUISIÇÃO
(1)	(2)	(3)	(4)
JÓIAS (aliança, anel, etc.)		,	
RELÓGIO DE PULSO DE HOMEM		,	
RELÓGIO DE PULSO DE MULHER		,	
RELÓGIO DE PULSO DE CRIANÇA		,	
RELÓGIO DE BOLSO		,	
CANETA DE OURO OU PRATA		,	
ISQUEIRO DE OURO OU PRATA		,	
CARTÃO DE PRATA		,	
TELEFONE CELULAR (aparelho)		,	
ACESSÓRIOS DE TELEFONE CELULAR		,	
		,	
		,	
		,	
		,	
		,	
		,	
		,	
		,	
		,	
		,	
		,	
		,	
		,	
		,	
		,	
		,	

47 OUTROS IMÓVEIS NO PERÍODO DE REFERÊNCIA DE 12 MESES

SITUAÇÃO DO QUADRO 1 ☐ PESQUISADO COM REGISTRO 3 ☐ PESQUISADO SEM REGISTRO 5 ☐ NÃO-PESQUISADO

TIPO	FORMA DE AQUISIÇÃO	VALOR
(1)	(2)	(3)
AQUISIÇÃO DE OUTROS IMÓVEIS À VISTA OU A PRAZO (parcelas, taxas, etc.)		,
AQUISIÇÃO DE OUTROS TERRENOS OU LOTES À VISTA OU A PRAZO (parcelas, taxas, etc.)		,
TAXAS DE LOCAÇÃO DE OUTROS IMÓVEIS (contrato, depósito, multa, etc.)		,
ALUGUEL DE OUTROS IMÓVEIS		,
ALUGUEL DE GARAGEM DE OUTROS IMÓVEIS		,
ENERGIA ELÉTRICA DE OUTROS IMÓVEIS (Kwh)		,
IMPOSTO PREDIAL E TERRITORIAL URBANO (IPTU) DE OUTROS IMÓVEIS		,
CONDOMÍNIO DE OUTROS IMÓVEIS		,
AQUISIÇÃO DE TÍTULO DE CLUBE		,
TAXA (incêndio, lixo, segurança, etc.) DE OUTROS IMÓVEIS		,
IMPOSTO SOBRE PROPRIEDADE TERRITORIAL RURAL (IPTR) DE OUTROS IMÓVEIS		,
SEGURO CONTRA ROUBO E INCÊNDIO DE OUTROS IMÓVEIS		,
MUDANÇA		,
VALOR DE OUTRO IMÓVEL ADQUIRIDO EM PRIMEIRA LOCAÇÃO (novo e valor total)		,
VALOR DE OUTRO IMÓVEL ADQUIRIDO USADO (valor total)		,
		,
		,
		,
		,
		,
		,
		,
		,
		,
		,
		,

48 CONTRIBUIÇÕES, TRANSFERÊNCIAS E ENCARGOS FINANCEIROS NO PERÍODO DE REFERÊNCIA DE 12 MESES

SITUAÇÃO DO QUADRO 1 ☐ PESQUISADO COM REGISTRO 3 ☐ PESQUISADO SEM REGISTRO 5 ☐ NÃO-PESQUISADO

TIPO	FORMA DE AQUISIÇÃO	VALOR
(1)	(2)	(3)
MESADA, DINHEIRO PARA OUTRA UC		,
PAGAMENTO DE EMPRÉSTIMO		,
SEGURO DE VIDA		,
DOAÇÕES EM DINHEIRO (igreja, orfanato, instituição de caridade, museu, etc.)		,
CONSELHO E ASSOCIAÇÃO DE CLASSE		,
PREVIDÊNCIA PRIVADA ABERTA		,
PREVIDÊNCIA PRIVADA FECHADA		,
PREVIDÊNCIA PÚBLICA (Federal, Estadual, Municipal e Militar)		,
JUROS E SEGURO DE EMPRÉSTIMO		,
PENSÃO ALIMENTÍCIA EM DINHEIRO		,
SEGURO DE ACIDENTES PESSOAIS		,
CAIXA ESCOLAR (contribuição em dinheiro)		,
		,
		,
		,
		,
		,
		,
		,
		,
		,
		,
		,
		,
		,
		,

49 **CURSOS, LIVROS DIDÁTICOS, REVISTAS TÉCNICAS E OUTROS ITENS REFERENTES A EDUCAÇÃO NO PERÍODO DE REFERÊNCIA DE 12 MESES**

SITUAÇÃO DO QUADRO 1 ☐ PESQUISADO COM REGISTRO 3 ☐ PESQUISADO SEM REGISTRO 5 ☐ NÃO-PESQUISADO

TIPO	FORMA DE AQUISIÇÃO	VALOR	LOCAL DE AQUISIÇÃO
(1)	(2)	(3)	(4)
CURSO PRÉ-ESCOLAR		,	
CURSO REGULAR DE 1º GRAU		,	
CURSO REGULAR DE 2º GRAU		,	
CURSO REGULAR DE 3º GRAU		,	
CURSO SUPLETIVO		,	
CURSO PRÉ-VESTIBULAR		,	
CURSO PRÉ-TÉCNICO		,	
CURSO DE PÓS-GRADUAÇÃO (mestrado)		,	
AULA PARTICULAR		,	
CURSO DE MÚSICA		,	
CURSO DE INFORMÁTICA		,	
CRECHE		,	
AULA DE GINÁSTICA		,	
CURSO E AULA DE BALÉ		,	
AULA DE NATAÇÃO		,	
CURSO DE IDIOMA (exceto linguafone)		,	
LIVRO ESCOLAR DE 1º E 2º GRAU		,	
LIVRO TÉCNICO, REVISTA TÉCNICA E OUTROS LIVROS DIDÁTICOS		,	
TAXAS ESCOLARES		,	
CARNÊ DE FORMATURA		,	
UNIFORME ESCOLAR		,	
TRANSPORTE ESCOLAR		,	
		,	
		,	
		,	
		,	

50 VEÍCULOS: DOCUMENTAÇÃO, SEGURO E OUTROS NO PERÍODO DE REFERÊNCIA DE 12 MESES

SITUAÇÃO DO QUADRO 1 ☐ PESQUISADO COM REGISTRO 3 ☐ PESQUISADO SEM REGISTRO 5 ☐ NÃO-PESQUISADO

TIPO	FORMA DE AQUISIÇÃO	VALOR
(1)	(2)	(3)
IMPOSTO SOBRE PROPRIEDADE DE VEÍCULOS AUTOMOTORES (IPVA)		,
SEGURO OBRIGATÓRIO		,
MULTAS		,
SEGURO VOLUNTÁRIO DE AUTOMÓVEIS/UTILITÁRIOS		,
EMPLACAMENTO DE AUTOMÓVEL		,
EMPLACAMENTO DE CAMINHÃO		,
EMPLACAMENTO DE MOTO		,
EMPLACAMENTO DE EMBARCAÇÃO		,
VAGA CATIVA DE ESTACIONAMENTO DE VEÍCULO		,
TAXA DE EXPEDIENTE (DETRAN)		,
ASSISTÊNCIA AUTOMOBILÍSTICA (mensalidade e taxa)		,
ALUGUEL DE VEÍCULO		,
TRANSFERÊNCIA DE CONSÓRCIO		,
SERVIÇO DE SEGURANÇA VIA SATÉLITE		,
		,
		,
		,
		,
		,
		,
		,
		,
		,
		,
		,
		,

51 **AQUISIÇÃO DE VEÍCULOS NO PERÍODO DE REFERÊNCIA DE 12 MESES**

SITUAÇÃO DO QUADRO 1 ☐ PESQUISADO COM REGISTRO 3 ☐ PESQUISADO SEM REGISTRO 5 ☐ NÃO-PESQUISADO

TIPO	FORMA DE AQUISIÇÃO	VALOR	ESTADO<br>1 - Novo<br>3 - Usado<br>9 - Não sabe	LOCAL DE AQUISIÇÃO
(1)	(2)	(3)	(4)	(5)
AUTOMÓVEL DE PASSEIO NACIONAL		,		
AUTOMÓVEL DE PASSEIO IMPORTADO		,		
AUTOMÓVEL UTILITÁRIO NACIONAL		,		
AUTOMÓVEL UTILITÁRIO IMPORTADO		,		
MOTOCICLETA		,		
BICICLETA		,		
		,		
		,		
		,		
		,		
		,		
		,		
		,		

OBSERVAÇÕES

Ministério do Planejamento, Orçamento e Gestão

IBGE
Instituto Brasileiro de Geografia e Estatística

Diretoria de Pesquisas
Coordenação de Trabalho e Rendimento
Gerência da Pesquisa de Orçamentos Familiares

Pesquisa de Orçamentos Familiares 2008 - 2009

POF 5 - Questionário de Trabalho e Rendimento Individual

52 IDENTIFICAÇÃO E CONTROLE DO QUESTIONÁRIO

01 IDENTIFICAÇÃO GERAL

UF	MUNICÍPIO	DISTRI-TO	SUBDIS-TRITO	SETOR	Nº DE ORDEM NA LISTAGEM

02 IDENTIFICAÇÃO POF

UF	SEQÜENCIAL	DV	CÓDIGO DO DOMICÍLIO	PERÍODO TEÓRICO	PERÍODO REAL	Nº DA UC	Nº DE ORDEM DO INFORMANTE

NOME DO INFORMANTE ____

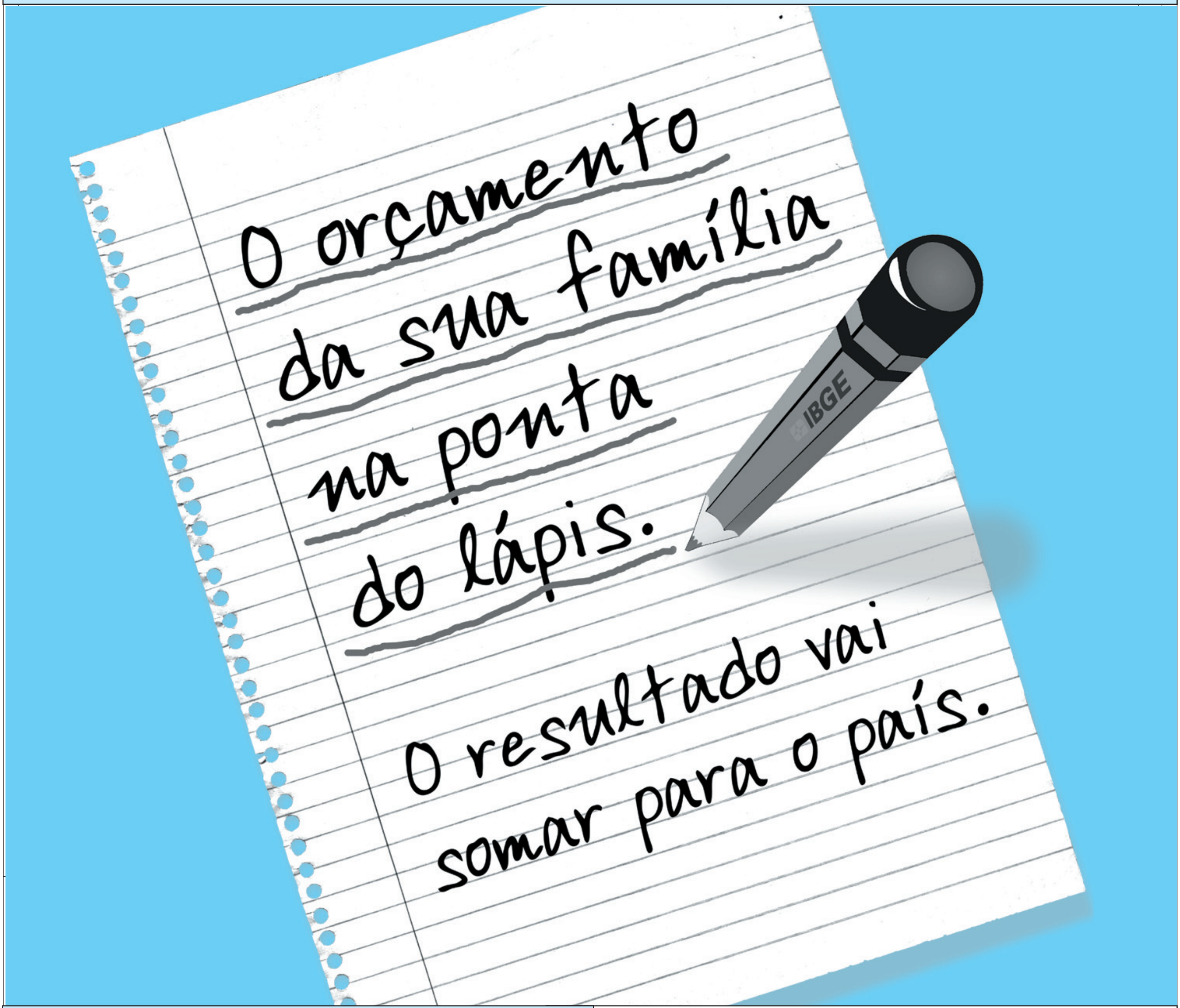

PERÍODO DE REFERÊNCIA

PERÍODO DE 12 MESES

|_|_|_|_| a |_|_|_|_|

MÊS DO ÚLTIMO RENDIMENTO

01 - Janeiro
02 - Fevereiro
03 - Março
04 - Abril
05 - Maio
06 - Junho
07 - Julho
08 - Agosto
09 - Setembro
10 - Outubro
11 - Novembro
12 - Dezembro
99 - Não sabe

Por lei, as informações prestadas para as pesquisas do IBGE têm caráter confidencial e só podem ser utilizadas para fins estatísticos (Lei nº 5534 de 14/11/1968).

53	TRABALHOS, RENDIMENTOS E DEDUÇÕES NO PERÍODO DE REFERÊNCIA DE 12 MESES

SITUAÇÃO DO QUADRO 1 ☐ PESQUISADO COM REGISTRO 3 ☐ PESQUISADO SEM REGISTRO 5 ☐ NÃO-PESQUISADO

QUANTOS TRABALHOS _______ TEVE NO PERÍODO? ☐

TRABALHO PRINCIPAL

1	**OCUPAÇÃO**

2	**ATIVIDADE**

3	**POSIÇÃO NA OCUPAÇÃO**

1 ☐ EMPREGADO PRIVADO
4 ☐ EMPREGADO TEMPORÁRIO NA ÁREA RURAL
7 ☐ APRENDIZ OU ESTAGIÁRIO
2 ☐ EMPREGADO PÚBLICO
5 ☐ EMPREGADOR
8 ☐ NÃO-REMUNERADO EM AJUDA A MEMBRO DO DOMICÍLIO
3 ☐ EMPREGADO DOMÉSTICO
6 ☐ CONTA-PRÓPRIA
9 ☐ TRABALHADOR NA PRODUÇÃO PARA O PRÓPRIO CONSUMO

4	**ÚLTIMO RENDIMENTO BRUTO MENSAL RECEBIDO**
4.1	NÚMERO DE HORAS TRABALHADAS HABITUALMENTE POR SEMANA: ☐☐
4.2	FORMA

0 ☐ NÃO TEM ⇨ Passe para Outro Trabalho
2 ☐ EM DINHEIRO E BENEFÍCIOS ⇨ Siga quesito 4.3
1 ☐ EM DINHEIRO ⇨ Siga quesito 4.3
3 ☐ SOMENTE BENEFÍCIOS ⇨ Passe para Outro Trabalho

4.3 VALOR	4.4 MÊS DO ÚLTIMO RENDIMENTO	4.5 Nº DE MESES RECEBIDOS
R$ ☐☐☐☐☐☐ , 0 0	☐☐	☐☐

5	**NO VALOR INFORMADO, TEVE DEDUÇÃO(ÕES)?** 1 ☐ SIM ⇨ Siga quesito 6 2 ☐ NÃO ⇨ Passe para Outro Trabalho

6	**DEDUÇÕES DO ÚLTIMO RENDIMENTO BRUTO MENSAL RECEBIDO**

6.1 PREVIDÊNCIA PÚBLICA	6.2 IMPOSTO DE RENDA	6.3 OUTRAS DEDUÇÕES
R$ ☐☐☐☐☐☐ , 0 0	R$ ☐☐☐☐☐☐ , 0 0	R$ ☐☐☐☐☐☐ , 0 0

TRABALHOS, RENDIMENTOS E DEDUÇÕES NO PERÍODO DE REFERÊNCIA DE 12 MESES

OUTRO TRABALHO

1 **OCUPAÇÃO**

....

2 **ATIVIDADE**

....

3 **POSIÇÃO NA OCUPAÇÃO**

1 ☐ EMPREGADO PRIVADO
2 ☐ EMPREGADO PÚBLICO
3 ☐ EMPREGADO DOMÉSTICO
4 ☐ EMPREGADO TEMPORÁRIO NA ÁREA RURAL
5 ☐ EMPREGADOR
6 ☐ CONTA-PRÓPRIA
7 ☐ APRENDIZ OU ESTAGIÁRIO
8 ☐ NÃO-REMUNERADO EM AJUDA A MEMBRO DO DOMICÍLIO
9 ☐ TRABALHADOR NA PRODUÇÃO PARA O PRÓPRIO CONSUMO

4 **ÚLTIMO RENDIMENTO BRUTO MENSAL RECEBIDO**

4.1 NÚMERO DE HORAS TRABALHADAS HABITUALMENTE POR SEMANA: ☐☐

4.2 FORMA

0 ☐ NÃO TEM ⇨ Passe para Outro Trabalho
1 ☐ EM DINHEIRO ⇨ Siga quesito 4.3
2 ☐ EM DINHEIRO E BENEFÍCIOS ⇨ Siga quesito 4.3
3 ☐ SOMENTE BENEFÍCIOS ⇨ Passe para Outro Trabalho

4.3 VALOR	4.4 MÊS DO ÚLTIMO RENDIMENTO	4.5 Nº DE MESES RECEBIDOS
R$ ☐☐☐☐☐☐ , 0 0	☐☐	☐☐

5 **NO VALOR INFORMADO, TEVE DEDUÇÃO(ÕES)?** 1 ☐ SIM ⇨ Siga quesito 6 2 ☐ NÃO ⇨ Passe para Outro Trabalho

6 **DEDUÇÕES DO ÚLTIMO RENDIMENTO BRUTO MENSAL RECEBIDO**

6.1 PREVIDÊNCIA PÚBLICA	6.2 IMPOSTO DE RENDA	6.3 OUTRAS DEDUÇÕES
R$ ☐☐☐☐☐☐ , 0 0	R$ ☐☐☐☐☐☐ , 0 0	R$ ☐☐☐☐☐☐ , 0 0

53 | TRABALHOS, RENDIMENTOS E DEDUÇÕES NO PERÍODO DE REFERÊNCIA DE 12 MESES

OUTRO TRABALHO

1 **OCUPAÇÃO**

2 **ATIVIDADE**

3 **POSIÇÃO NA OCUPAÇÃO**

1 ☐ EMPREGADO PRIVADO	4 ☐ EMPREGADO TEMPORÁRIO NA ÁREA RURAL	7 ☐ APRENDIZ OU ESTAGIÁRIO
2 ☐ EMPREGADO PÚBLICO	5 ☐ EMPREGADOR	8 ☐ NÃO-REMUNERADO EM AJUDA A MEMBRO DO DOMICÍLIO
3 ☐ EMPREGADO DOMÉSTICO	6 ☐ CONTA-PRÓPRIA	9 ☐ TRABALHADOR NA PRODUÇÃO PARA O PRÓPRIO CONSUMO

4 **ÚLTIMO RENDIMENTO BRUTO MENSAL RECEBIDO**

4.1 NÚMERO DE HORAS TRABALHADAS HABITUALMENTE POR SEMANA: |__|__|

4.2 FORMA

0 ☐ NÃO TEM

2 ☐ EM DINHEIRO E BENEFÍCIOS ⇨ Siga quesito 4.3

1 ☐ EM DINHEIRO ⇨ Siga quesito 4.3

3 ☐ SOMENTE BENEFÍCIOS

4.3 VALOR	4.4 MÊS DO ÚLTIMO RENDIMENTO	4.5 Nº DE MESES RECEBIDOS
R$ |__|__|__|__|__|__| , |0|0|	|__|__|	|__|__|

5 **NO VALOR INFORMADO, TEVE DEDUÇÃO(ÕES)?** 1 ☐ SIM ⇨ Siga quesito 6 2 ☐ NÃO

6 **DEDUÇÕES DO ÚLTIMO RENDIMENTO BRUTO MENSAL RECEBIDO**

6.1 PREVIDÊNCIA PÚBLICA	6.2 IMPOSTO DE RENDA	6.3 OUTRAS DEDUÇÕES
R$ |__|__|__|__|__|__| , |0|0|	R$ |__|__|__|__|__|__| , |0|0|	R$ |__|__|__|__|__|__| , |0|0|

54 APOSENTADORIAS, PENSÕES, AUXÍLIOS, OUTROS RENDIMENTOS HABITUAIS E DEDUÇÕES NO PERÍDO DE REFERÊNCIA DE 12 MESES

SITUAÇÃO DO QUADRO 1 ☐ PESQUISADO COM REGISTRO 3 ☐ PESQUISADO SEM REGISTRO 5 ☐ NÃO-PESQUISADO

TIPO	ÚLTIMO RENDIMENTO MENSAL (R$)	DEDUÇÃO	MÊS DO ÚLTIMO RENDIMENTO	NÚMERO DE MESES
(1)	(2)	(3)	(4)	(5)
Aposentadoria do Instituto Nacional de Seguridade Social (INSS)	, 0 0	, 0 0		
Pensão do Instituto Nacional de Seguridade Social (INSS).	, 0 0	, 0 0		
Aposentadoria (Municipal, Estadual, Federal) da Previdência Pública	, 0 0	, 0 0		
Pensão (Municipal, Estadual, Federal) da Previdência Pública	, 0 0	, 0 0		
Aposentadoria, Suplementação da Previdência Privada (Aberta ou Fechada)	, 0 0	, 0 0		
Bolsa-família	, 0 0	, 0 0		
Benefício de prestação continuada (BPC, LOAS)	, 0 0	, 0 0		
Programa de Erradicação do Trabalho Infantil (PETI)	, 0 0	, 0 0		
Bolsa de estudo	, 0 0	, 0 0		
Pensão alimentícia de não-morador	, 0 0	, 0 0		
Mesada de não-morador	, 0 0	, 0 0		
Doação de não-morador	, 0 0	, 0 0		
Aluguel, uso ou exploração de bens imóveis	, 0 0	, 0 0		
Aluguel, direitos autorais, patentes de bens móveis	, 0 0	, 0 0		
Auxílio-alimentação	, 0 0	, 0 0		
Auxílio-transporte	, 0 0	, 0 0		
....	, 0 0	, 0 0		
....	, 0 0	, 0 0		
....	, 0 0	, 0 0		

55	OUTROS RENDIMENTOS, RECEITAS, EMPRÉSTIMOS E DEDUÇÕES ESPORÁDICAS NO PERÍODO DE REFERÊNCIA DE **12 MESES**

SITUAÇÃO DO QUADRO 1 ☐ PESQUISADO COM REGISTRO 3 ☐ PESQUISADO SEM REGISTRO 5 ☐ NÃO-PESQUISADO

TIPO	VALOR	DEDUÇÃO
(1)	(2)	(3)
Décimo Terceiro Salário	,00	,00
Férias	,00	,00
Saque do PIS/PASEP (total)	,00	,00
Rendimento do PIS/PASEP	,00	,00
Saque do FGTS	,00	,00
Indenização trabalhista	,00	,00
Participação nos lucros de negócios (empregador)	,00	,00
Participação nos lucros de empresa (empregado)	,00	,00
Comissão de venda (esporádica)	,00	,00
Hora extra	,00	,00
Empréstimo	,00	,00
Seguro-desemprego	,00	,00
Restituição do Imposto de Renda	,00	,00
Herança e outras transferências patrimoniais	,00	,00
Venda de imóvel (exceto terreno)	,00	,00
Venda de automóvel	,00	,00
Venda de consórcio, carnê e outros	,00	,00
Indenização e restituição de seguro pessoal	,00	,00
Indenização e restituição de seguro obrigatório de auto/utilitários pessoal	,00	,00
Indenização e restituição de seguro voluntário de auto/utilitários pessoal	,00	,00
Indenização e restituição de seguro obrigatório de moto	,00	,00
Indenização e restituição de seguro voluntário de moto	,00	,00
....	,00	,00
....	,00	,00
....	,00	,00
....	,00	,00
....	,00	,00
....	,00	,00
....	,00	,00
....	,00	,00
....	,00	,00
....	,00	,00
....	,00	,00
....	,00	,00

56	DEPÓSITO/APLICAÇÃO DE ATIVO FINANCEIRO NO PERÍODO DE REFERÊNCIA DE 12 MESES

SITUAÇÃO DO QUADRO 1 ☐ PESQUISADO COM REGISTRO 3 ☐ PESQUISADO SEM REGISTRO 5 ☐ NÃO-PESQUISADO

TIPO	DEPÓSITO/APLICAÇÃO<br>VALOR
(1)	(2)
Poupança	,00
Fundo de aplicação financeira (CDB, RDB, Commodities, etc.)	,00
Ações	,00
Outros (ouro, moedas estrangeiras, etc.)	,00

57	RETIRADA/RESGATE DE ATIVO FINANCEIRO NO PERÍODO DE REFERÊNCIA DE 12 MESES

SITUAÇÃO DO QUADRO 1 ☐ PESQUISADO COM REGISTRO 3 ☐ PESQUISADO SEM REGISTRO 5 ☐ NÃO-PESQUISADO

TIPO	RETIRADA/RESGATE<br>VALOR
(1)	(2)
Poupança	,00
Fundo de aplicação financeira (CDB, RDB, Commodities, etc.)	,00
Ações	,00
Outros (ouro, moedas estrangeiras, etc.)	,00

OBSERVAÇÕES

Ministério do Planejamento, Orçamento e Gestão

IBGE
Instituto Brasileiro de Geografia e Estatística

Diretoria de Pesquisas
Coordenação de Trabalho e Rendimento
Gerência da Pesquisas de Orçamentos Familiares

Pesquisa de Orçamentos Familiares 2008 - 2009

POF 6 - Avaliação das Condições de Vida

60 IDENTIFICAÇÃO E CONTROLE DO QUESTIONÁRIO

01 IDENTIFICAÇÃO GERAL

UF	MUNICÍPIO	DISTRI-TO	SUBDIS-TRITO	SETOR	Nº DE ORDEM NA LISTAGEM

02 IDENTIFICAÇÃO POF

UF	SEQÜENCIAL	DV	CÓDIGO DO DOMICÍLIO	PERÍODO TEÓRICO	PERÍODO REAL	Nº DA UC

03 SITUAÇÃO DO QUESTIONÁRIO ☐ NÃO-PESQUISADO

04 NOME DO INFORMANTE: ....

61 AVALIAÇÃO DAS CONDIÇÕES DE VIDA

01 Na sua opinião a renda total de sua família permite que você(s) leve(m) a vida até o fim do mês com:

1 ☐ Muita dificuldade
2 ☐ Dificuldade
3 ☐ Alguma dificuldade
4 ☐ Alguma facilidade
5 ☐ Facilidade
6 ☐ Muita facilidade

02 Levando em conta a situação atual de sua família, qual seria a renda mensal familiar mínima necessária para chegar até o final do mês?

R$ ☐☐☐☐☐☐ , 0 0

03 Levando em conta a situação atual de sua família, qual seria o valor mínimo mensal de recursos para cobrir os gastos com alimentação de toda sua família?

R$ ☐☐☐☐☐☐ , 0 0

04 Das afirmativas a seguir, qual aquela que melhor descreve a quantidade de alimento consumido por sua família?

1 ☐ Normalmente não é suficiente
2 ☐ Às vezes não é suficiente
3 ☐ É sempre suficiente

05 Das afirmativas a seguir, qual aquela que melhor descreve o tipo de alimento consumido por sua família?

1 ☐ Sempre do tipo que quer → Passe para o quesito 07.
2 ☐ Nem sempre do tipo que quer
3 ☐ Raramente do tipo que quer
(2 e 3) → Siga quesito 06.

06 Qual a razão de sua família não estar se alimentando do tipo que quer?

1 ☐ Porque a renda familiar não permite
2 ☐ Os alimentos que a família quer não são encontrados no mercado
3 ☐ Outras razões

07 Como avalia as condições de moradia de sua família em relação ao serviço de:

	1 Bom	2 Ruim	3 Não tem
01 Água	☐	☐	☐
02 Coleta de lixo	☐	☐	☐
03 Iluminação de rua	☐	☐	☐
04 Escoamento da água de chuva na área	☐	☐	☐
05 Fornecimento de energia elétrica	☐	☐	☐
06 Transporte coletivo	☐	☐	☐
07 Educação	☐	☐	☐
08 Saúde	☐	☐	☐
09 Lazer e esporte	☐	☐	☐
10 Limpeza e manutenção de rua	☐	☐	☐
11 Esgotamento sanitário	☐	☐	☐

08 Há algum dos seguintes problemas no seu domicílio?

	1 Sim	2 Não
1 Pouco espaço	☐	☐
2 Rua ou vizinhos barulhentos	☐	☐
3 Casa escura	☐	☐
4 Telhado com goteira	☐	☐
5 Fundação, paredes ou chão úmidos	☐	☐
6 Madeira das janelas, portas ou assoalhos deteriorados	☐	☐
7 Poluição ou problemas ambientais causados pelo trânsito ou indústria	☐	☐
8 Violência ou vandalismo na sua área de residência	☐	☐
9 Sujeito à inundação	☐	☐

09 Na sua opinião, as condições de moradia de sua família são:

1 ☐ Boas 2 ☐ Satisfatórias 3 ☐ Ruins

10 Nos últimos 12 meses, sua família atrasou o pagamento de alguma das seguintes despesas?

	1 Sim	2 Não	3 Não se aplica
1 Aluguel ou prestação da casa, apartamento	☐	☐	☐
2 Água, eletricidade e gás	☐	☐	☐
3 Prestações de bens ou serviços adquiridos	☐	☐	☐

Ministério do Planejamento, Orçamento e Gestão

IBGE
Instituto Brasileiro de Geografia e Estatística

Diretoria de Pesquisas
Coordenação de Trabalho e Rendimento
Gerência da Pesquisa de Orçamentos Familiares

Pesquisa de Orçamentos Familiares 2008 - 2009

POF 7 - Bloco de Consumo Alimentar Pessoal

**70**	**IDENTIFICAÇÃO E CONTROLE DO QUESTIONÁRIO**
01	IDENTIFICAÇÃO GERAL<br>UF ☐☐ MUNICÍPIO ☐☐☐☐☐ ☐☐ SUBDIS-TRITO ☐☐ SETOR ☐☐☐☐ Nº DE ORDEM NA LISTAGEM ☐☐☐☐
02	IDENTIFICAÇÃO POF<br>UF ☐☐ SEQÜENCIAL ☐☐☐ DV ☐ CÓDIGO DO DOMICÍLIO ☐☐ PERÍODO TEÓRICO ☐☐ PERÍODO REAL ☐☐ Nº DA UC ☐ Nº DO INFORMANTE ☐☐
03	NOME DO INFORMANTE ______________________________
04	TOTAL DE DIAS PESQUISADOS ☐☐
05	REGISTROS FEITOS PELO PRÓPRIO INFORMANTE? 1 ☐ SIM 3 ☐ NÃO

Prezado(a) senhor(a),

A sua colaboração no preenchimento deste bloco representa uma efetiva contribuição para o sucesso da Pesquisa de Orçamentos Familiares. Recordamos que as informações prestadas serão usadas exclusivamente para fins estatísticos e serão mantidas em sigilo, conforme estabelecido na lei 5.534 de 14/11/1968.

Muito obrigado por sua colaboração.

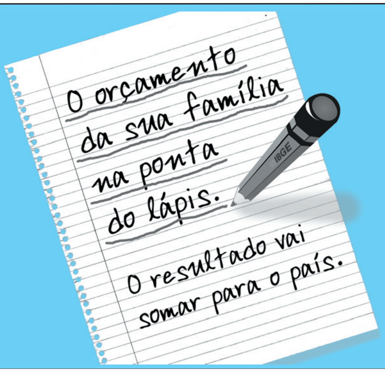

71	1 DATA: 17 / 06 / 2008	DIA DO POF 3: 1	DIA DA SEMANA: *Terça-feira*	1º DIA (CONTINUA)

2 **SITUAÇÃO DO QUADRO**

1 [X] PESQUISADO COM REGISTRO   3 [ ] PESQUISADO SEM REGISTRO   5 [ ] NÃO-PESQUISADO

INTRUÇÕES PARA PREENCHIMENTO DO QUADRO

1 - Registre a quantidade de todos os alimentos consumidos por você, no domicílio ou fora, conforme exemplo de preenchimento desta página.

D = Domicílio: o alimento consumido é proveniente do seu domicílio.

F = Fora: o alimento é adquirido e também consumido fora do seu domicílio.

2 - Procure descrever separadamente todos os itens consumidos. Se não for possível, registre junto, conforme o seguinte exemplo: 1 pão francês com manteiga.

3 - Escolha dois dias da semana, com um intervalo de pelo menos um dia entre eles, para registrar seu consumo.

______ utiliza com freqüência: [ ] Açúcar [X] Adoçante [ ] Açúcar e Adoçante [ ] Não utiliza

FONTE DO ALIMENTO	HORÁRIO	DESCRIÇÃO DO ALIMENTO CONSUMIDO
(3)	(4)	(5)
D	7h	1 copo médio de café
D	7h	2 ovos de galinha fritos
D	7h	3 pontas de faca de manteiga
D	7h	1 copo grande de leite com sabor
D	7h	1 pão francês
F	7h	2 balas
F	9h	1 laranja
F	10h	2 bifes de alcatra fritos
F	13h	1 porção de batata-inglesa cozida
F	13h	0,5 copo médio de café
F	13h	1 taça de salada de frutas
F	13h	3 escumadeiras de arroz
F	13h	1 copo grande de refrigerante de guaraná
F	13h	2 fatias de queijo prato
F	16h	2 rodelas de abacaxi
D	18h	3 conchas de sopa de legumes
D	20h	1 colher de chá de azeite de oliva
D	20h	3 colheres de sopa de doce de abóbora
F	23h	1 lata de 350 ml de cerveja

EXEMPLO

PESQUISA DE ORÇAMENTOS FAMILIARES 2008-2009

Anexo auxiliar do POF 7 - Bloco de Consumo Alimentar Pessoal

Para facilitar o preenchimento deste instrumento de coleta, utilize os exemplos abaixo.

MEDIDAS

Asa
Bago
Banda
Barra
Bife
Bisnaga
Bola
Cacho
Caneca
Caneco
Casquinha
Colher de arroz / servir
Colher de café
Colher de chá
Colher de sobremesa
Colher de sopa
Concha
Copo americano

Copo de cafezinho
Copo de requeijão
Copo grande
Copo médio
Copo tulipa
Costela
Coxa
Cumbuca
Dose
Escumadeira
Espetinho
Espeto
Espiga
Fatia
Filé
Folha
Garfada
Garrafa de _____ ml

Gomo
Grama
Lata de ___ ml
Litro
Maço
Metade
Mililitros
Pacote
Pedaço
Pegador
Peito
Pescoço
Pires
Ponta de faca
Porção
Punhado
Posta
Pote

Prato de sobremesa
Prato fundo
Prato raso
Quilo
Ramo
Rodela
Sachê
Saco
Sobrecoxa
Tablete
Taça
Tigela
Unidade
Unidade pequena
Xícara de café
Xícara de chá

PREPARAÇÕES

Cru(a)
Cozido(a)
Grelhado(a)/brasa/churrasco
Assado(a)
Frito(a)

Empanado(a)/à milanesa
Refogado(a)
Molho vermelho
Molho branco
Ao alho e óleo

Com manteiga/óleo
Ao vinagrete
Ensopado(a)
Mingau
Sopa

71	1 DATA: ____/____/____	DIA DO POF 3: ____	DIA DA SEMANA: ____________	1º DIA (CONTINUA)

2 SITUAÇÃO DO QUADRO

1 ☐ PESQUISADO COM REGISTRO   3 ☐ PESQUISADO SEM REGISTRO   5 ☐ NÃO-PESQUISADO

INTRUÇÕES PARA PREENCHIMENTO DO QUADRO

1 - Registre a quantidade de todos os alimentos consumidos por você, no domicílio ou fora, conforme exemplo de preenchimento desta página.

D = Domicílio: o alimento consumido é proveniente do seu domicílio.

F = Fora: o alimento é adquirido e também consumido fora do seu domicílio.

2 - Procure descrever separadamente todos os itens consumidos. Se não for possível, registre junto, conforme o seguinte exemplo: 1 pão francês com manteiga.

3 - Escolha dois dias da semana, com um intervalo de pelo menos um dia entre eles, para registrar seu consumo.

______ utiliza com freqüência: ☐ Açúcar ☐ Adoçante ☐ Açúcar e Adoçante ☐ Não utiliza

FONTE DO ALIMENTO	HORÁRIO	DESCRIÇÃO DO ALIMENTO CONSUMIDO
(3)	(4)	(5)

FONTE DO ALIMENTO	HORÁRIO	DESCRIÇÃO DO ALIMENTO CONSUMIDO
(3)	(4)	(5)

72	1 DATA: ____/____/____	DIA DO POF 3: ____	DIA DA SEMANA: ____________	2º DIA (CONTINUA)

2 SITUAÇÃO DO QUADRO

1 ☐ PESQUISADO COM REGISTRO   3 ☐ PESQUISADO SEM REGISTRO   5 ☐ NÃO-PESQUISADO

INTRUÇÕES PARA PREENCHIMENTO DO QUADRO

1 - Registre a quantidade de todos os alimentos consumidos por você, no domicílio ou fora, conforme exemplo de preenchimento desta página.

D = Domicílio: o alimento consumido é proveniente do seu domicílio.

F = Fora: o alimento é adquirido e também consumido fora do seu domicílio.

2 - Procure descrever separadamente todos os itens consumidos. Se não for possível, registre junto, conforme o seguinte exemplo: 1 pão francês com manteiga.

3 - Escolha dois dias da semana, com um intervalo de pelo menos um dia entre eles, para registrar seu consumo.

FONTE DO ALIMENTO	HORÁRIO	DESCRIÇÃO DO ALIMENTO CONSUMIDO
(3)	(4)	(5)

2º DIA (CONCLUSÃO)

FONTE DO ALIMENTO	HORÁRIO	DESCRIÇÃO DO ALIMENTO CONSUMIDO
(3)	(4)	(5)

OBSERVAÇÕES
