# Supplementary material for: Can a poverty-reducing and progressive tax and transfer system hurt the poor?
Source: J Dev Econ. 2016 Sep;122:63–75. doi: 10.1016/j.jdeveco.2016.04.001 (PMC4988485; doi:10.1016/j.jdeveco.2016.04.001)
Supplement: Supplementary file 1 — Supplementary material. [file mmc1.zip › documentation/POF0809/Alimentaçao 2008 - 2009.doc]

COMPONENTES DAS LINHAS DAS

TABELAS DE DESPESA MONETÁRIA E NÃO MONETÁRIA MÉDIA MENSAL FAMILIAR,

COM ALIMENTAÇÃO

Observações Iniciais:

- Este documento apresenta os códigos dos produtos componentes de cada linha das tabelas de Despesa Monetária e Não Monetária Média Mensal Familiar, com Alimentação.

- Cada código com 5 dígitos foi composto através dos 2 dígitos da variável “Número do Quadro” e dos 3 primeiros dígitos da variável “Código do Item”.

- Os 2 últimos dígitos da variável “Código do Item” se referem a números sequenciais, utilizados para identificar sinônimos ou diferentes denominações regionais de um mesmo produto. Como estes sequenciais são agregados em um único código na construção do plano tabular, não precisam ser descritos na construção das linhas da tabela.

**Despesa com alimentação** - 1 + 2

**1 - Alimentação no domicílio** - 1.1 + 1.2 + ------------------- + 1.16

**1.1 - Cereais, leguminosas e oleaginosas** - 1.1.1 + 1.1.2 + 1.1.3 + 1.14

1.1.1 - Arroz – 63001 a 63003, 63018, 63033 a 63034

1.1.2 - Feijão – 63012 a 63017, 63019, 63021 a 63026, 63031, 63046

1.1.3 - Orgânicos – 63037 a 63041, 63043 a 63045

1.1.4 - Outros – 63004 a 63011, 63020, 63027 a 63030, 63032, 63035 a 63036, 63042, 63047 a 63049, 66001 a 66016, 66018 a 66022, 66024 a 66025, 66027 a 66028

**1.2 - Farinhas, féculas e massas** - 1.2.1 + 1.2.2 + 1.2.3 + 1.2.4

1.2.1 - Macarrão – 65032 a 65034, 65049, 65069

1.2.2 - Farinha de trigo – 65010

1.2.3 - Farinha de mandioca – 65014

1.2.4 - Outras – 65001 a 65009, 65011 a 65013, 65015 a 65031, 65035 a 65048, 65050 a 65068, 65070 a 65071

**1.3 - Tubérculos e raízes** - 1.3.1 + 1.3.2 + 1.3.3 + 1.3.4

1.3.1 - Batata inglesa – 64001

1.3.2 - Cenoura – 64012

1.3.3 - Mandioca – 64006

1.3.4 - Outros – 64002 a 64005, 64007 a 64011, 64013 a 64020

1.4 - **Açúcares e derivados** - 1.4.1 + 1.4.2 + 1.4.3 + 1.4.4

1.4.1 - Açúcar refinado – 69001, 69086

1.4.2 - Açúcar cristal – 69002, 69087

1.4.3 - Light e Diet – 69069 a 69071, 69079 a 69081, 69091 a 69141

1.4.4 - Outros – 69003 a 69056, 69058 a 69063, 69066 a 69068, 69072 a 69074, 69076 a 69078, 69082 a 69085, 69088 a 69090, 69142 a 69148

**1.5 - Legumes e verduras** - 1.5.1 + 1.5.2 + 1.5.3 + 1.5.4

1.5.1 - Tomate – 67051, 67079

1.5.2 - Cebola – 67057

1.5.3 - Alface – 67001, 67078

1.5.4 - Outros – 67002 a 67050, 67052 a 67056, 67058 a 67061, 67063 a 67077, 67080 a 67082, 67084 a 67091, 67093, 67096, 67098 a 67109

**1.6 - Frutas** - 1.6.1 + 1.6.2 + 1.6.3 + 1.6.4

1.6.1 - Banana – 68001 a 68011, 68091, 68098

1.6.2 - Laranja – 68012 a 68015, 68017 a 68018, 68095, 68097

1.6.3 - Maça – 68030, 68086

1.6.4 - Outras frutas – 68016, 68019 a 68029, 68031 a 68085, 68087 a 68090, 68092 a 68094, 68096, 68099 a 68102

**1.7 - Carnes, vísceras e pescados** - 1.7.1 + 1.7.2 + ------------------- + 1.7.6

1.7.1 - Carne de boi de primeira – 71001 a 71007, 71014, 71016, 71064, 71113 a 71120

1.7.2 - Carne de boi de segunda – 71008 a 71013, 71015, 71017, 71026, 71108, 71122

1.7.3 - Carne de suíno – 71033 a 71037, 71060, 71065, 71068, 71105 a 71106

1.7.4 - Carnes e peixes industrializados – 72004 a 72005, 72014 a 72015, 72024 a 72025, 72034 a 72035, 72044 a 72045, 72054 a 72055, 72064 a 72065, 72074 a 72075, 72084 a 72085, 72094 a 72095, 72104 a 72105, 72114 a 72116, 72124 a 72125, 72134 a 72135, 72144 a 72145, 72154 a 72155, 72164 a 72165, 72174 a 72175, 72184 a 72185, 72194 a 72195, 72204 a 72205, 72214 a 72215, 72224 a 72225, 72234 a 72235, 72244 a 72245, 72254 a 72255, 72264 a 72265, 72274 a 72275, 72284 a 72285, 72294 a 72295, 72304 a 72305, 72314 a 72315, 72324 a 72325, 72334 a 72335, 72344 a 72345, 72354 a 72355, 72364 a 72365, 72374 a 72375, 72384 a 72385, 72394 a 72395, 72404 a 72405, 72414 a 72415, 72424 a 72425, 72434 a 72435, 72444 a 72445, 72454 a 72455, 72464 a 72465, 72474 a 72475, 72484 a 72485, 72494 a 72495, 72504 a 72505, 72514 a 72515, 72524 a 72525, 72534 a 72535, 72544 a 72545, 72554 a 72555, 72564 a 72565, 72574 a 72575, 72584 a 72585, 72594 a 72595, 72604, 72606, 72614, 72624, 72634, 72644, 72664, 72674, 72684, 72694 a 72695, 72704, 72714 a 72715, 72724 a 72725, 72734, 72744, 72754 a 72755, 72805, 74004 a 74005, 74014 a 74015, 74024 a 74025, 74034 a 74035, 74044 a 74045, 74054 a 74055, 74064 a 74065, 74074 a 74075, 74084 a 74085, 74094 a 74095, 74104 a 74105, 74114 a 74115, 74124 a 74125, 74134 a 74135, 74144 a 74145, 74154 a 74155, 74164 a 74165, 74174 a 74175, 74184 a 74185, 74194 a 74195, 74204 a 74205, 74214 a 74215, 74224 a 74225, 74234 a 74235, 74244 a 74245, 74254 a 74255, 74264 a 74265, 74274 a 74275, 74284 a 74285, 74294 a 74295, 74304 a 74305, 74314 a 74315, 74324 a 74325, 74334 a 74335, 74344 a 74345, 74354 a 74355, 74364 a 74365, 74374 a 74375, 74384 a 74385, 74394 a 74395, 74404 a 74405, 74414 a 74415, 74424 a 74425, 74434, 74444 a 74445, 74454 a 74455, 74464 a 74465, 74474 a 74475, 74484 a 74485, 74494 a 74495, 74504, 74514 a 74515, 76004 a 76005, 78029, 78031, 81001 a 81067

1.7.5 - Pescados frescos – 72001 a 72003, 72011 a 72013, 72021 a 72023, 72031 a 72033, 72039, 72041 a 72043, 72049, 72051 a 72053, 72061 a 72063, 72071 a 72073, 72081 a 72083, 72091 a 72093, 72101 a 72103, 72111 a 72113, 72121 a 72123, 72131 a 72133, 72141 a 72143, 72149, 72151 a 72153, 72161 a 72163, 72169, 72171 a 72173, 72181 a 72183, 72191 a 72193, 72201 a 72203, 72207, 72211 a 72213, 72221 a 72223, 72231 a 72233, 72241 a 72243, 72249, 72251 a 72253, 72261 a 72263, 72271 a 72273, 72279, 72281 a 72283, 72291 a 72293, 72301 a 72303, 72311 a 72313, 72321 a 72323, 72331 a 72333, 72341 a 72343, 72351 a 72353, 72361 a 72363, 72371 a 72373, 72381 a 72383, 72391 a 72393, 72399, 72401 a 72403, 72411 a 72413, 72421 a 72423, 72431 a 72433, 72441 a 72443, 72451 a 72453, 72461 a 72463, 72471 a 72473, 72481 a 72483, 72491 a 72493, 72501 a 72503, 72511 a 72513, 72521 a 72523, 72531 a 72533, 72541 a 72543, 72551 a 72553, 72561 a 72563, 72571 a 72573, 72581 a 72583, 72591 a 72593, 72601 a 72602, 72611 a 72612, 72621 a 72622, 72631 a 72632, 72641 a 72642, 72651, 72661 a 72662, 72671 a 72672, 72681 a 72682, 72691 a 72693, 72711 a 72713, 72721 a 72723, 72731, 72741 a 72742, 72751 a 72753, 72761, 72771, 72781, 72791, 72802, 72811, 74001 a 74003, 74011 a 74013, 74021 a 74023, 74031 a 74033, 74041 a 74043, 74047, 74049, 74051 a 74053, 74061 a 74063, 74071 a 74073, 74081 a 74083, 74091 a 74093, 74101 a 74103, 74109, 74111 a 74113, 74121 a 74123, 74131 a 74133, 74141 a 74143, 74151 a 74153, 74161 a 74163, 74171 a 74173, 74181 a 74183, 74191 a 74193, 74201 a 74203, 74211 a 74213, 74221 a 74223, 74227, 74229, 74231 a 74233, 74241 a 74243, 74251 a 74253, 74261 a 74263, 74271 a 74273, 74276, 74281 a 74283, 74291 a 74293, 74301 a 74303, 74311 a 74313, 74321 a 74323, 74329, 74331 a 74333, 74341 a 74343, 74349, 74351 a 74353, 74361 a 74363, 74371 a 74373, 74381 a 74383, 74391 a 74393, 74401 a 74403, 74411 a 74413, 74421 a 74423, 74431 a 74432, 74441 a 74443, 74451 a 74453, 74461 a 74463, 74471 a 74473, 74481 a 74483, 74491 a 74493, 74501 a 74502, 74511 a 74513, 74519, 74521, 74531, 74541, 74551, 74553, 74561, 74571, 74581, 74591, 74601, 74611, 76001 a 76003, 76007, 76009, 76011

1.7.6 - Outros – 71018 a 71025, 71027 a 71032, 71038 a 71059, 71061 a 71063, 71066 a 71067, 71069 a 71104, 71107, 71109 a 71112, 71121, 71123 a 71124

**1.8 - Aves e ovos** - 1.8.1 + 1.8.2 + 1.8.3 + 1.8.4

1.8.1 - Frango – 78001 a 78014, 78025 a 78027, 78032, 78036, 78038, 78047 a 78048, 78062, 78066, 78068

1.8.2 - Ovo de galinha – 78033, 78065

1.8.3 - Orgânicos – 78055 a 78059, 78064

1.8.4 - Outros – 78015 a 78024, 78028, 78030, 78034 a 78035, 78037, 78039 a 78046, 78049 a 78054, 78060 a 78061, 78063, 78067, 78069

**1.9 - Leites e derivados** - 1.9.1 + 1.9.2 + 1.9.3 + 1.9.4 + 1.9.5 + 1.9.6

1.9.1 - Leite de vaca – 79001 a 79002, 79031, 79036 a 79038

1.9.2 - Leite em pó – 79006 a 79008, 79070

1.9.3 - Queijos – 79017 a 79022, 79024 a 79030, 79033, 79078 a 79079, 79081 a 79084

1.9.4 - Light e Diet – 79039 a 79042, 79051 a 79069, 79071 a 79072, 79076 a 79077, 79080, 79086 a 79087

1.9.5 - Orgânicos – 79043 a 79050, 79073 a 79074

1.9.6 - Outros – 79003 a 79005, 79009 a 79016, 79023, 79032, 79034 a 79035, 79075, 79085

**1.10 - Panificados** - 1.10.1 + 1.10.2 + 1.10.3 + 1.10.4

1.10.1 - Pão francês – 80001

1.10.2 - Biscoito – 80022 a 80024, 80048 a 80049, 80052 a 80054

1.10.3 - Light e diet – 80065 a 80105, 80108 a 80109, 80112 a 80115

1.10.4 - Outros panificados – 80002 a 80021, 80025 a 80047, 80050 a 80051, 80055 a 80064, 80106 a 80107, 80110 a 80111

1.11 - **Óleos e gorduras** - 1.11.1 + 1.11.2 + 1.11.3

1.11.1 - Óleo de soja – 84003

1.11.2 - Azeite de oliva – 84001

1.11.3 - Outros – 84002, 84004 a 84043

**1.12 - Bebidas e infusões** - 1.12.1 + 1.12.2 + 1.12.3 + 1.12.4 + 1.12.5

1.12.1 - Café moído – 82025, 82106

1.12.2 - Refrigerantes – 82001 a 82006, 82009 a 82014, 82017 a 82018, 82035, 82041 a 82043, 82046, 82049, 82054 a 82055, 82057, 82067 a 82073, 82147, 82150, 82155

1.12.3 - Bebidas não alcoólicas light e diet – 82007 a 82008, 82015 a 82016, 82019, 82040, 82052, 82074 a 82079, 82081 a 82085, 82087 a 82100, 82110 a 82129, 82134, 82139 a 82141, 82145, 82152, 82156, 83043 a 83044

1.12.4 - Cervejas e chopes – 83001 a 83002

1.12.5 - Outras bebidas alcoólicas – 83003 a 83032, 83034 a 83042, 83045 a 83047

1.12.6 - Outras – 66017, 66023, 66026, 69057, 69064 a 69065, 69075, 82020 a 82024, 82026 a 82034, 82036 a 82039, 82044 a 82045, 82047 a 82048, 82050 a 82051, 82053, 82056, 82058 a 82066, 82080, 82086, 82101 a 82105, 82107 a 82109, 82130 a 82133, 82135 a 82138, 82142 a 82144, 82146, 82148 a 82149, 82151, 82153 a 82154, 83033

**1.13.1 - Enlatados e conservas** – 77001 a 77076

**1.14 - Sal e condimentos** - 1.14.1 + 1.14.2 + 1.14.3 + 1.14.4

1.14.1 - Massa de tomate – 70047

1.14.2 - Maionese – 70043, 70104

1.14.3 - Sal refinado – 70001, 70110

1.14.4 - Outros – 67062, 67083, 67092, 67094 a 67095, 67097, 70002 a 70042, 70044 a 70046, 70048 a 70103, 70105 a 70109, 70111 a 70126

**1.15 - Alimentos preparados** – 85001 a 85098

**1.16 - Outros** - 90001 a 90009

**2 - Alimentação fora do domicílio** - 2.1 + 2.2 + 2.3 + 2.4 + 2.5 + 2.6 + 2.7 + 2.8 + 2.9

2.1 - Almoço e jantar – 24001, 24035 a 24036, 24038, 24040, 24054 a 24056, 41006, 48033

2.2 - Café, leite, café/leite e chocolate – 24002, 24005, 24016, 24034, 24061, 24064

2.3 - Sanduíches e salgados – 24004, 24037, 24039, 24062

2.4 - Refrigerantes e outras bebidas não alcoólicas – 24006 a 24007, 24013, 24017, 24020 a 24033, 24050 a 24053, 24066, 24077, 24099, 24139

2.5 - Lanches – 24041 a 24042

2.6 - Cervejas, chopes e outras bebidas alcoólicas – 24009 a 24012, 24046 a 24049, 24067 a 24068

2.7 - Alimentação na escola – 24057 a 24060, 49026

2.8 - Alimentação light e diet – 24069 a 24076, 24078 a 24098, 24100 a 24138

2.9 - Outras – 24003, 24008, 24014 a 24015, 24018 a 24019, 24043 a 24045, 24063, 24065, 24999
