# Supplementary material for: Can a poverty-reducing and progressive tax and transfer system hurt the poor?
Source: J Dev Econ. 2016 Sep;122:63–75. doi: 10.1016/j.jdeveco.2016.04.001 (PMC4988485; doi:10.1016/j.jdeveco.2016.04.001)
Supplement: Supplementary file 1 — Supplementary material. [file mmc1.zip › documentation/POF0809/ClassificaçSes POF 2008-2009.doc]

# DOCUMENTAÇÃO DOS MICRODADOS

# PESQUISA DE ORÇAMENTOS FAMILIARES 2008-2009

```
CLASSIFICAÇÕES DAS VARIÁVEIS CATEGORIZADAS


VARIÁVEIS ESPECÍFICAS DO REGISTRO TIPO 01: DOMICÍLIO - POF1 / QUADRO 2


CLASSIFICAÇÃO: 100 – TIPO DE DOMICÍLIO

     Quantidade de Categorias: 3
     Número de Dígitos: 2

Categorias por Código:

              01 – Casa
            02 – Apartamento
		03 - Cômodo

       Categorias por Nome:

            02 – Apartamento
            01 – Casa
		03 - Cômodo


CLASSIFICAÇÃO: 101 – MATERIAL QUE PREDOMINA NAS PAREDES EXTERNAS

     Quantidade de Categorias: 6
     Número de Dígitos: 2

     Categorias por Código:

     01 – Alvenaria
     02 - Madeira para Construção
     03 - Taipa Não-Revestida
     04 - Madeira Aproveitada
     05 - Palha
     06 - Outro Material.


     Categorias por Nome:

     01 – Alvenaria
     04 - Madeira Aproveitada
     02 - Madeira para Construção
     06 - Outro Material.
     05 - Palha
     03 - Taipa Não-Revestida


CLASSIFICAÇÃO: 102 – MATERIAL QUE PREDOMINA NA COBERTURA

     Quantidade de Categorias: 7
     Número de Dígitos: 2

     Categorias por Código:

     01 - Telha Qualquer
     02 - Laje de Concreto
     03 - Madeira para Construção
     04 - Chapa Metálica
     05 - Madeira Aproveitada
     06 - Palha
     07 - Outro Material
     Categorias por Nome:

     04 - Chapa Metálica
     02 - Laje de Concreto
     05 - Madeira Aproveitada
     03 - Madeira para Construção
     07 - Outro Material.
     06 - Palha
     01 - Telha Qualquer


CLASSIFICAÇÃO: 103 – MATERIAL QUE PREDOMINA NO PISO

     Quantidade de Categorias: 7
     Número de Dígitos: 2

Categorias por Código:

01 – Carpete
02 – Cerâmica, Lajota ou pedra
03 – Madeira para Construção
04 – Cimento
05 – Madeira Aproveitada
06 – Terra
07 – Outro Material

 	Categorias por Nome:

   	01 – Carpete
   	02 – Cerâmica, Lajota ou pedra
   	04 – Cimento
05 – Madeira Aproveitada
03 – Madeira para Construção
	07 – Outro Material
	06 – Terra


CLASSIFICAÇÃO: 104 – EXISTÊNCIA DE ÁGUA CANALIZADA

     Quantidade de Categorias: 2
     Número de Dígitos: 2

Categorias por Código:

01 – Sim
02 – Não

 	Categorias por Nome:

02 – Não
01 – Sim


CLASSIFICAÇÃO: 105 – PROVENIÊNCIA DA ÁGUA

     Quantidade de Categorias: 3
     Número de Dígitos: 2

Categorias por Código:

01 - Rede Geral de Distribuição
02 - Poço ou Nascente
03 - Outra Proveniência.

 	Categorias por Nome:

03 - Outra Proveniência
02 - Poço ou Nascente
01 - Rede Geral de Distribuição

CLASSIFICAÇÃO: 106 – ESCOADOURO SANITÁRIO

     Quantidade de Categorias: 7
     Número de Dígitos: 2

Categorias por Código:

01 - Rede Coletora de Esgoto ou Pluvial
02 - Fossa Séptica
03 - Fossa Rudimentar
04 - Vala
05 – Direto para o Rio, Lago ou Mar
06 – Outra Forma
07 - Não Tem


 	Categorias por Nome:

05 – Direto para o Rio, Lago ou Mar
   	03 - Fossa Rudimentar
   	02 - Fossa Séptica
   	07 - Não Tem
   	06 – Outro Forma
   	01 - Rede Coletora de Esgoto ou Pluvial
04 - Vala


CLASSIFICAÇÃO: 107 – CONDIÇÃO DE OCUPAÇÃO

     Quantidade de Categorias: 6
     Número de Dígitos: 2

Categorias por Código:

01 - Próprio - já pago
02 - Próprio – ainda pagando
03 - Cedido por empregador
04 - Cedido de outra forma
05 – Outra condição
06 - Alugado

 	Categorias por Nome:

   	06 - Alugado
04 - Cedido de outra forma
03 - Cedido por empregador
   	05 – Outra condição
02 - Próprio – ainda pagando
01 - Próprio já pago


CLASSIFICAÇÃO: 108 – TEMPO DE ALUGUEL

     Quantidade de Categorias: 4
     Número de Dígitos: 2

     Categorias por Código:

     	–1 - Não Aplicável.
      01 - Menos de 12 meses
02 - De 12 a 30 meses
      03 - Mais de 30 meses

	Categorias por Nome:


      02 - De 12 a 30 meses
      03 - Mais de 30 meses
      01 - Menos de 12 meses
      –1 - Não Aplicável


CLASSIFICAÇÃO: 109 – TIPO DE CONTRATO DE ALUGUEL

     Quantidade de Categorias: 4
     Número de Dígitos: 2

     Categorias por Código:

     –1 - Não Aplicável
     01 - Verbal (“Só de Boca”)
     02 - Documentado através de Imobiliária
     03 - Documentado através de Outra Fonte


    Categorias por Nome:

     02 - Documentado através de Imobiliária
     03 - Documentado através de Outra Fonte
     –1 - Não Aplicável
     01 - Verbal (“Só de Boca”)


CLASSIFICAÇÃO: 110 – EXISTÊNCIA DE PAVIMENTAÇÃO NA RUA

     Quantidade de Categorias: 2
     Número de Dígitos: 2

     Categorias por Código:

      01 – Sim
	02 - Não


Categorias por Nome:

      02 - Não
      01 – Sim


CLASSIFICAÇÃO: 111 – IMPUTAÇÃO – QUANTIDADE DE CÔMODOS

     Quantidade de Categorias: 2
     Número de Dígitos: 1

     Categorias por Código:

      0 – Não
	1 - Sim


Categorias por Nome:

0 – Não
	1 - Sim


CLASSIFICAÇÃO: 112 – IMPUTAÇÃO – QUANTIDADE DE BANHEIROS

     Quantidade de Categorias: 2
     Número de Dígitos: 1

     Categorias por Código:

      0 – Não
	1 - Sim


Categorias por Nome:

0 – Não
	1 - Sim


CLASSIFICAÇÃO: 113 – IMPUTAÇÃO - ESCOADOURO SANITÁRIO

     Quantidade de Categorias: 2
     Número de Dígitos: 1

     Categorias por Código:

      0 – Não
	1 - Sim

Categorias por Nome:

0 – Não
	1 - Sim


VARIÁVEIS ESPECÍFICAS DO REGISTRO TIPO 02: PESSOAS - POF1 / QUADROS 3 E 4


CLASSIFICAÇÃO: 200 – CONDIÇÃO NA UNIDADE DE CONSUMO

     Quantidade de Categorias: 8
     Número de Dígitos: 2

Categorias por Código:

01 - Pessoa de referência
02 - Cônjuge
03 - Filho
  	04 - Outro parente
05 - Agregado
06 - Pensionista
07 - Empregado doméstico
08 - Parente do empregado doméstico

 	Categorias por Nome:

05 - Agregado
	02 - Cônjuge
   	07 - Empregado doméstico
	03 – Filho
	04 - Outro parente
	08 - Parente do empregado doméstico
	06 – Pensionista
	01 - Pessoa de referência


CLASSIFICAÇÃO: 201 – CONDIÇÃO NA FAMÍLIA

     Quantidade de Categorias: 8
     Número de Dígitos: 2

Categorias por Código:

01 - Pessoa de referência
02 - Cônjuge
03 - Filho
  	04 - Outro parente
05 - Agregado
06 - Pensionista
07 - Empregado doméstico
08 - Parente do empregado doméstico

 	Categorias por Nome:

05 - Agregado
	02 - Cônjuge
   	07 - Empregado doméstico
	03 – Filho
	04 - Outro parente
	08 - Parente do empregado doméstico
	06 – Pensionista
	01 - Pessoa de referência


CLASSIFICAÇÃO: 202 - CONDIÇÃO DE PRESENÇA

     Quantidade de Categorias: 2
     Número de Dígitos: 2

Categorias por Código:

01 - Morador Presente
02 - Morador Ausente

 	Categorias por Nome:

02 - Morador Ausente
	01 - Morador Presente


CLASSIFICAÇÃO: 203 – SEXO

     Quantidade de Categorias: 2
     Número de Dígitos: 2

Categorias por Código:

01 - Masculino
02 – Feminino


 	Categorias por Nome:

02 - Feminino
	01 - Masculino


CLASSIFICAÇÃO: 204 – SABE LER E ESCREVER

     Quantidade de Categorias: 2
     Número de Dígitos: 2

Categorias por Código:

01 - Sim
02 – Não

 	Categorias por Nome:

02 - Não
	01 – Sim


CLASSIFICAÇÃO: 205 – FREQUÊNCIA À ESCOLA OU CRECHE

     Quantidade de Categorias: 4
     Número de Dígitos: 2

Categorias por Código:

01 – Sim, rede particular
02 – Sim, rede pública
03 – Não, mas já frequentou
04 – Nunca frequentou

 	Categorias por Nome:

03 – Não, mas já frequentou
	04 – Nunca frequentou
	01 – Sim, rede particular
	02 – Sim, rede pública


CLASSIFICAÇÃO: 206 – CURSO QUE FREQUENTA

     Quantidade de Categorias: 14
     Número de Dígitos: 2

Categorias por Código:

-1 – Não Aplicável
01 - Creche
02 - Pré-Escolar
03 – Classe de Alfabetização de crianças
04 – Alfabetização de adultos
05 – Regular do Ensino fundamental
06 – Educação de Jovens e Adultos ou Supletivo do Ensino Fundamental ou 1º grau
07 – Regular do Ensino Médio
08 - Educação de Jovens e Adultos ou Supletivo do Ensino Médio ou 2º grau
09 – Tecnológico Superior
10 – Pré-Vestibular
11 – Superior – graduação
12 – Especialização superior
13 - Mestrado ou doutorado

 	Categorias por Nome:

04 – Alfabetização de adultos
03 – Classe de Alfabetização de crianças
01 - Creche
06 – Educação de Jovens e Adultos ou Supletivo do Ensino Fundamental ou 1º grau
08 - Educação de Jovens e Adultos ou Supletivo do Ensino Médio ou 2º grau
12 – Especialização superior
13 - Mestrado ou doutorado
-1 – Não Aplicável
02 - Pré-Escolar
10 – Pré-Vestibular
05 – Regular do Ensino fundamental
07 – Regular do Ensino Médio
11 – Superior – graduação
09 – Tecnológico Superior


CLASSIFICAÇÃO: 207 – QUAL A DURAÇÃO DO CURSO DE PRIMEIRO GRAU

     Quantidade de Categorias: 3
     Número de Dígitos: 2

Categorias por Código:

-1 – Não Aplicável
01 – 8 anos
02 – 9 anos

Categorias por nome:

01 – 8 anos
02 – 9 anos
-1 – Não Aplicável


CLASSIFICAÇÃO: 208 – SÉRIE QUE FREQUENTA

     Quantidade de Categorias: 11
     Número de Dígitos: 2

Categorias por Código:

-1 – Não Aplicável
01 - Primeira
02 - Segunda
03 – Terceira
04 – Quarta
05 – Quinta
06 - Sexta
07 – Sétima
08 – Oitava
09 - Nona
10 – Curso não seriado


	Categorias por Nome:

10 – Curso não seriado
-1 – Não Aplicável
09 - Nona
	08 - Oitava
	01 - Primeira
	04 – Quarta
	05 – Quinta
	02 – Segunda
	07 – Sétima
	06 – Sexta
03 – Terceira


CLASSIFICAÇÃO: 209 – CURSO MAIS ELEVADO QUE FREQUENTOU

     Quantidade de Categorias: 17
     Número de Dígitos: 2

Categorias por Código:

-1 – Não Aplicável
01 - Creche
02 - Pré-Escolar
03 – Classe de Alfabetização de crianças
04 – Alfabetização de adultos
05 – Antigo Primário
06 – Antigo Ginásio
07 – Antigo Clássico, Científico, etc.
08 – Regular do Ensino fundamental
09 – Educação de Jovens e Adultos ou Supletivo do Ensino Fundamental ou 1º grau
10 – Regular do Ensino Médio
11 - Educação de Jovens e Adultos ou Supletivo do Ensino Médio ou 2º grau
12 – Tecnológico Superior
13 – Pré-Vestibular
14 – Superior – graduação
15 – Especialização superior
16 - Mestrado ou doutorado

 	Categorias por Nome:

07 – Antigo Clássico, Científico, etc.
06 – Antigo Ginásio
05 – Antigo Primário
04 – Alfabetização de adultos
03 – Classe de Alfabetização de crianças
01 - Creche
09 – Educação de Jovens e Adultos ou Supletivo do Ensino Fundamental ou 1º grau
11 - Educação de Jovens e Adultos ou Supletivo do Ensino Médio ou 2º grau
15 – Especialização superior
16 - Mestrado ou doutorado
-1 – Não Aplicável
02 - Pré-Escolar
13 – Pré-Vestibular
08 – Regular do Ensino fundamental
10 – Regular do Ensino Médio
14 – Superior – graduação
12 – Tecnológico Superior


CLASSIFICAÇÃO: 210 – QUAL ERA A DURAÇÃO DO CURSO DE PRIMEIRO GRAU

     Quantidade de Categorias: 3
     Número de Dígitos: 2

Categorias por Código:

-1 – Não Aplicável
01 – 8 anos
02 – 9 anos

Categorias por nome:

01 – 8 anos
02 – 9 anos
-1 – Não Aplicável


CLASSIFICAÇÃO: 211 – ÚLTIMA SÉRIE CONCLUÍDA

     Quantidade de Categorias: 12
     Número de Dígitos: 2

Categorias por Código:

-1 – Não Aplicável
01 - Primeira
02 - Segunda
03 – Terceira
04 – Quarta
05 – Quinta
06 - Sexta
07 – Sétima
08 – Oitava
09 - Nona
10 – Curso não seriado
11 - Nenhuma

	Categorias por Nome:

10 – Curso não seriado
11 - Nenhuma
-1 – Não Aplicável
09 - Nona
	08 - Oitava
	01 - Primeira
	04 – Quarta
	05 – Quinta
	02 – Segunda
	07 – Sétima
	06 – Sexta
03 – Terceira


CLASSIFICAÇÃO: 212 – CONCLUSÃO DO CURSO MAIS ELEVADO

     Quantidade de Categorias: 3
     Número de Dígitos: 2

Categorias por Código:

-1 – Não Aplicável
01 - Sim
02 – Não

 Categorias por Nome:

02 - Não
-1 – Não Aplicável
	01 - Sim


CLASSIFICAÇÃO: 213 – ANOS DE ESTUDO

     Quantidade de Categorias: 17
     Número de Dígitos: 2


Categorias por Código:

00 - Zero
01 - Um
02 - Dois
03 - Três
04 - Quatro
05 - Cinco
06 - Seis
07 - Sete
08 - Oito
09 - Nove
10 - Dez
11 - Onze
      12 - Doze
13 - Treze
14 - Quatorze
      15 - Quinze ou mais
      88 - Não determinado


CLASSIFICAÇÃO: 214 – COR OU RAÇA

     Quantidade de Categorias: 6
     Número de Dígitos: 2

Categorias por Código:

01 - Branca
02 - Preta
03 – Amarela
04 – Parda
05 – Indígena
09 – Não Sabe

 	Categorias por Nome:

03 – Amarela
01 - Branca
   	05 – Indígena
09 – Não Sabe
	04 – Parda
	02 - Preta


CLASSIFICAÇÃO: 215 – ORÇAMENTO TRABALHO E/OU RENDIMENTO

     Quantidade de Categorias: 5
     Número de Dígitos: 2

Categorias por Código:

-1 – Não Aplicável
01 - Sim
02 – Não
03 – Recusa
04 - Outro

 	Categorias por Nome:

02 – Não
-1 – Não Aplicável
04 - Outro
	03 – Recusa
01 – Sim


CLASSIFICAÇÃO: 216 – ORÇAMENTO DESPESA

     Quantidade de Categorias: 5
     Número de Dígitos: 2

Categorias por Código:

-1 – Não Aplicável
01 - Sim
02 – Não
03 – Recusa
04 - Outro

 	Categorias por Nome:

02 – Não
-1 – Não Aplicável
04 - Outro
	03 – Recusa
01 - Sim


CLASSIFICAÇÃO: 217 – TEM CARTÃO DE CRÉDITO

     Quantidade de Categorias: 3
     Número de Dígitos: 2

Categorias por Código:

-1 – Não Aplicável
01 - Sim
02 – Não


 	Categorias por Nome:

02 - Não
-1 – Não Aplicável
	01 - Sim


CLASSIFICAÇÃO: 218 – TITULAR DO CARTÃO DE CRÉDITO

     Quantidade de Categorias: 3
     Número de Dígitos: 2

Categorias por Código:

-1 – Não Aplicável
01 - Sim
02 – Não


 	Categorias por Nome:

02 - Não
-1 – Não Aplicável
	01 - Sim


CLASSIFICAÇÃO: 219 – TEM CHEQUE ESPECIAL

     Quantidade de Categorias: 3
     Número de Dígitos: 2

Categorias por Código:

-1 – Não Aplicável
01 - Sim
02 – Não


 	Categorias por Nome:

02 - Não
-1 – Não Aplicável
	01 - Sim


CLASSIFICAÇÃO: 220 – TITULAR DA CONTA CORRENTE

     Quantidade de Categorias: 3
     Número de Dígitos: 2

Categorias por Código:


-1 – Não Aplicável
01 - Sim
02 – Não


 	Categorias por Nome:

02 - Não
-1 – Não Aplicável
	01 - Sim


CLASSIFICAÇÃO: 221 – ESTÁ GRÁVIDA

     Quantidade de Categorias: 3
     Número de Dígitos: 2

Categorias por Código:

-1 – Não Aplicável
01 - Sim
02 – Não


 	Categorias por Nome:

02 - Não
-1 – Não Aplicável
	01 - Sim


VARIÁVEIS ESPECÍFICAS DO REGISTRO TIPO 03: PESSOAS - IMPUTAÇÃO - POF1/QUADRO 4


CLASSIFICAÇÃO: 300 – IMPUTAÇÃO - SABE LER E ESCREVER

     Quantidade de Categorias: 2
     Número de Dígitos: 1

Categorias por Código e por Nome:

0 – Não
1 - Sim


CLASSIFICAÇÃO: 301 – IMPUTAÇÃO - FREQUÊNCIA À ESCOLA OU CRECHE

     Quantidade de Categorias: 2
     Número de Dígitos: 1

Categorias por Código e por Nome:

0 – Não
1 - Sim


CLASSIFICAÇÃO: 302 – IMPUTAÇÃO - CURSO QUE FREQUENTA

     Quantidade de Categorias: 2
     Número de Dígitos: 1

Categorias por Código e por Nome:

0 – Não
1 - Sim


CLASSIFICAÇÃO: 303 – IMPUTAÇÃO - QUAL A DURAÇÃO DO CURSO DE PRIMEIRO GRAU

     Quantidade de Categorias: 2
     Número de Dígitos: 1

Categorias por Código e por Nome:

0 – Não
1 - Sim


CLASSIFICAÇÃO: 304 – IMPUTAÇÃO - SÉRIE QUE FREQUENTA

     Quantidade de Categorias: 2
     Número de Dígitos: 1

Categorias por Código e por Nome:

0 – Não
1 - Sim


CLASSIFICAÇÃO: 305 – IMPUTAÇÃO - CURSO MAIS ELEVADO QUE FREQUENTOU

     Quantidade de Categorias: 2
     Número de Dígitos: 1

Categorias por Código e por Nome:

0 – Não
1 - Sim


CLASSIFICAÇÃO: 306 – IMPUTAÇÃO - QUAL ERA A DURAÇÃO DO CURSO DE PRIMEIRO GRAU

     Quantidade de Categorias: 2
     Número de Dígitos: 1

Categorias por Código e por Nome:

0 – Não
1 - Sim


CLASSIFICAÇÃO: 307 – IMPUTAÇÃO - ÚLTIMA SÉRIE CONCLUÍDA

     Quantidade de Categorias: 2
     Número de Dígitos: 1

Categorias por Código e por Nome:

0 – Não
1 - Sim


CLASSIFICAÇÃO: 308 – IMPUTAÇÃO - CONCLUSÃO DO CURSO MAIS ELEVADO

     Quantidade de Categorias: 2
     Número de Dígitos: 1

Categorias por Código e por Nome:

0 – Não
1 - Sim


CLASSIFICAÇÃO: 309 – IMPUTAÇÃO - TEM CARTÃO DE CRÉDITO

     Quantidade de Categorias: 2
     Número de Dígitos: 1

Categorias por Código e por Nome:

0 – Não
1 - Sim


CLASSIFICAÇÃO: 310 – IMPUTAÇÃO - TITULAR DO CARTÃO DE CRÉDITO

     Quantidade de Categorias: 2
     Número de Dígitos: 1

Categorias por Código e por Nome:


0 – Não
1 - Sim


CLASSIFICAÇÃO: 311 – IMPUTAÇÃO - TEM CHEQUE ESPECIAL

     Quantidade de Categorias: 2
     Número de Dígitos: 1

Categorias por Código e por Nome:

0 – Não
1 - Sim


CLASSIFICAÇÃO: 312 – IMPUTAÇÃO - TITULAR DA CONTA CORRENTE

     Quantidade de Categorias: 2
     Número de Dígitos: 1

Categorias por Código e por Nome:

0 – Não
1 - Sim


VARIÁVEIS ESPECÍFICAS DO REGISTRO TIPO 04: CONDIÇÕES DE VIDA - POF6

CLASSIFICAÇÃO: 400 – CÓDIGO DA RENDA FAMILIAR

     Quantidade de Categorias: 7
     Número de Dígitos: 1

     Categorias por Código:

     0 – Não Respondido
     1 – Muita Dificuldade
     2 – Dificuldade
     3 – Alguma Dificuldade
     4 – Alguma Facilidade
     5 – Facilidade
     6 – Muita Facilidade

      Categorias por Nome:

     3 – Alguma Dificuldade
     4 – Alguma Facilidade
     2 – Dificuldade
     5 – Facilidade
     1 – Muita Dificuldade
     6 – Muita Facilidade
      0 – Não Respondido


   CLASSIFICAÇÃO: 401 – QUANTIDADE DE ALIMENTO

        Quantidade de Categorias: 4
        Número de Dígitos: 1

        Categorias por Código:

        0 – Não Respondido
        1 – Normalmente não é Suficiente
        2 – Às Vezes não é Suficiente
        3 – É Sempre Suficiente

        Categorias por Nome:

        2 – Às Vezes não é Suficiente
        3 – É Sempre Suficiente
        0 – Não Respondido
        1 – Normalmente não é Suficiente


   CLASSIFICAÇÃO: 402 – TIPO DE ALIMENTO

     Quantidade de Categorias: 4
     Número de Dígitos: 1

     Categorias por Código:

     0 – Não Respondido
     1 – Sempre do Tipo que Quer
     2 – Nem Sempre do Tipo que Quer
     3 – Raramente do Tipo que Quer

     Categorias por Nome:

     0 – Não Respondido
     3 – Nem sempre do Tipo que Quer
     1 – Raramente do Tipo que Quer
     2 – Sempre do Tipo que Quer


VARIÁVEIS ESPECÍFICAS DO REGISTRO TIPO 09: DESPESAS COM SERVIÇOS DOMÉSTICOS - POF2 / QUADRO 19

CLASSIFICAÇÃO: 500 – CÓDIGO DO INSS


   Quantidade de Categorias: 54
   Número de Dígitos: 5


  Categorias por Código:
```

| 50101 | EMPREGADO (INSS) |
| --- | --- |
| 50102 | EMPREGADA (INSS) |
| 50201 | COZINHEIRO (INSS) |
| 50202 | COZINHEIRA (INSS) |
| 50301 | FAXINEIRO (INSS) |
| 50302 | FAXINEIRO (INSS) |
| 50401 | BABA (INSS) |
| 50501 | JARDINEIRO (INSS) |
| 50502 | JARDINEIRA (INSS) |
| 50601 | MOTORISTA (INSS) |
| 50701 | VIGIA (INSS) |
| 50702 | ZELADOR (INSS) |
| 50703 | PORTEIRO (INSS) |
| 50704 | PORTEIRA (INSS) |
| 50801 | ARRUMEDEIRA (INSS) |
| 50802 | ARRUMADOR (INSS) |
| 50901 | GOVERNANTA (INSS) |
| 51001 | MORDOMO (INSS) |
| 51101 | CASEIRO (INSS) |
| 51102 | CASEIRA (INSS) |
| 51201 | ACOMPANHANTE (INSS) |
| 51301 | LAVADEIRA (INSS) |
| 51302 | LAVADEIRO (INSS) |
| 51401 | AJUDANTE DE COZINHA (INSS) |
| 51501 | PASSADEIRA (EMPREGADA) (INSS) |
| 51601 | COPEIRA (INSS) |
| 51602 | COPEIRO (INSS) |
| 51701 | LAVADEIRA E PASSADEIRA (INSS) |
| 51702 | PASSADEIRA E LAVADEIRA (INSS) |
| 51801 | GARCOM (INSS) |
| 51802 | GARCONETE (INSS) |
| 51901 | CARREGADOR DE AGUA (INSS) |
| 51902 | PUXADOR DE AGUA (INSS) |
| 52001 | LIMPADOR DE TERRENO (CAPINADOR) (INSS) |
| 52002 | CAPINADOR (LIMPADOR DE TERRENO) (INSS) |
| 52003 | LIMPADOR DE TERRENO (INSS) |
| 52004 | CAPINADOR (INSS) |
| 52101 | LIMPADOR DE PISCINA (INSS) |
| 52102 | LAVADOR DE PISCINA (INSS) |
| 52201 | LAVADOR DE CARRO (INSS) |
| 52301 | BISCATEIRO (SERVICOS DIVERSOS) (INSS) |
| 52302 | BISCATEIRO (INSS) |
| 52401 | LIMPADOR DE FOSSA (INSS) |
| 52501 | ENFERMEIRA (SERVICO DOMESTICO) (INSS) |
| 52502 | ENFERMEIRA (INSS) |
| 52503 | ENFERMEIRO (INSS) |
| 52601 | CARREGADOR DE LENHA (INSS) |
| 52701 | TRATADOR DE ANIMAIS (INSS) |
| 52702 | BABA DE CACHORRO (INSS) |
| 52801 | SALVA-VIDAS (INSS) |
| 52901 | SERVENTE (INSS) |
| 53001 | MARINHEIRO (INSS) |
| 53002 | MARINHEIRA (INSS) |
| 59901 | AGREGADO (INSS) |

Categorias por Nome:

| 51201 | ACOMPANHANTE (INSS) |
| --- | --- |
| 59901 | AGREGADO (INSS) |
| 51401 | AJUDANTE DE COZINHA (INSS) |
| 50802 | ARRUMADOR (INSS) |
| 50801 | ARRUMEDEIRA (INSS) |
| 50401 | BABA (INSS) |
| 52702 | BABA DE CACHORRO (INSS) |
| 52302 | BISCATEIRO (INSS) |
| 52301 | BISCATEIRO (SERVICOS DIVERSOS) (INSS) |
| 52004 | CAPINADOR (INSS) |
| 52002 | CAPINADOR (LIMPADOR DE TERRENO) (INSS) |
| 51901 | CARREGADOR DE AGUA (INSS) |
| 52601 | CARREGADOR DE LENHA (INSS) |
| 51102 | CASEIRA (INSS) |
| 51101 | CASEIRO (INSS) |
| 51601 | COPEIRA (INSS) |
| 51602 | COPEIRO (INSS) |
| 50202 | COZINHEIRA (INSS) |
| 50201 | COZINHEIRO (INSS) |
| 50102 | EMPREGADA (INSS) |
| 50101 | EMPREGADO (INSS) |
| 52502 | ENFERMEIRA (INSS) |
| 52501 | ENFERMEIRA (SERVICO DOMESTICO) (INSS) |
| 52503 | ENFERMEIRO (INSS) |
| 50301 | FAXINEIRO (INSS) |
| 50302 | FAXINEIRO (INSS) |
| 51801 | GARCOM (INSS) |
| 51802 | GARCONETE (INSS) |
| 50901 | GOVERNANTA (INSS) |
| 50502 | JARDINEIRA (INSS) |
| 50501 | JARDINEIRO (INSS) |
| 51301 | LAVADEIRA (INSS) |
| 51701 | LAVADEIRA E PASSADEIRA (INSS) |
| 51302 | LAVADEIRO (INSS) |
| 52201 | LAVADOR DE CARRO (INSS) |
| 52102 | LAVADOR DE PISCINA (INSS) |
| 52401 | LIMPADOR DE FOSSA (INSS) |
| 52101 | LIMPADOR DE PISCINA (INSS) |
| 52001 | LIMPADOR DE TERRENO (CAPINADOR) (INSS) |
| 52003 | LIMPADOR DE TERRENO (INSS) |
| 53002 | MARINHEIRA (INSS) |
| 53001 | MARINHEIRO (INSS) |
| 51001 | MORDOMO (INSS) |
| 50601 | MOTORISTA (INSS) |
| 51501 | PASSADEIRA (EMPREGADA) (INSS) |
| 51702 | PASSADEIRA E LAVADEIRA (INSS) |
| 50704 | PORTEIRA (INSS) |
| 50703 | PORTEIRO (INSS) |
| 51902 | PUXADOR DE AGUA (INSS) |
| 52801 | SALVA-VIDAS (INSS) |
| 52901 | SERVENTE (INSS) |
| 52701 | TRATADOR DE ANIMAIS (INSS) |
| 50701 | VIGIA (INSS) |
| 50702 | ZELADOR (INSS) |

```
CLASSIFICAÇÃO: 501 – CÓDIGO DA ESPÉCIE

       Quantidade de Categorias: 3
       Número de Dígitos: 1

       Categorias por Código:

        1 – Mensalista
       3 – Diarista
       9 – Não Sabe

         Categorias por Nome:

       3 - Diarista
 1 - Mensalista
 9 – Não Sabe


VARIÁVEIS ESPECÍFICAS DO REGISTRO TIPO 11: CADERNETA DE DESPESA – POF 3

CLASSIFICAÇÃO: 600 - NÚMERO DO GRUPO DE DESPESA

    Quantidade de Categorias: 26
    Número de Dígitos: 2

  Categorias por Código:
```

| 63 | Cereais e leguminosas |
| --- | --- |
| 64 | Hortaliças tuberosas |
| 65 | Farinhas, féculas e massas |
| 66 | Cocos, castanhas e nozes |
| 67 | Hortaliças folhosas, frutosas e outras |
| 68 | Frutas |
| 69 | Açúcares e produtos de confeitaria |
| 70 | Sais e condimentos |
| 71 | Carnes e vísceras |
| 72 | Pescados marinhos |
| 74 | Pescados de água doce |
| 76 | Pescados não específicados |
| 77 | Enlatados e conservas |
| 78 | Aves e ovos |
| 79 | Laticínios |
| 80 | Panificados |
| 81 | Carnes industrializadas |
| 82 | Bebidas não alcoólicas e infusões |
| 83 | Bebidas alcoólicas |
| 84 | Óleos e gorduras |
| 85 | Alimentos preparados ou semi preparados |
| 86 | Produtos de limpeza doméstica e outros |
| 87 | Alimentos e produtos de higiene para animais |
| 88 | Flores naturais |
| 89 | Produtos de higiene corporal |
| 90 | Agregados |

Categorias por Nome:

| 69 | Açúcares e produtos de confeitaria |
| --- | --- |
| 90 | Agregados |
| 87 | Alimentos e produtos de higiene para animais |
| 85 | Alimentos preparados ou semi preparados |
| 78 | Aves e ovos |
| 83 | Bebidas alcoólicas |
| 82 | Bebidas não alcoólicas e infusões |
| 71 | Carnes e vísceras |
| 81 | Carnes industrializadas |
| 63 | Cereais e leguminosas |
| 66 | Cocos, castanhas e nozes |
| 77 | Enlatados e conservas |
| 65 | Farinhas, féculas e massas |
| 88 | Flores naturais |
| 68 | Frutas |
| 67 | Hortaliças folhosas, frutosas e outras |
| 64 | Hortaliças tuberosas |
| 79 | Laticínios |
| 84 | Óleos e gorduras |
| 80 | Panificados |
| 74 | Pescados de água doce |
| 72 | Pescados marinhos |
| 76 | Pescados não específicados |
| 89 | Produtos de higiene corporal |
| 86 | Produtos de limpeza doméstica e outros |
| 70 | Sais e condimentos |

```
CLASSIFICAÇÃO: 601 – MÉTODO DA QUANTIDADE ADQUIRIDA

    Quantidade de Categorias: 10
    Número de Dígitos: 2

  Categorias por Código:

(-2) Não aplicável (itens que não se referem a alimentos e bebidas)
(-1) Não houve tratamento (itens agregados)
      (0) Quando a quantidade adquirida em Kg foi calculada diretamente pelos valores informados nas variáveis quantidade, unidade de medida e peso ou volume.
           (1) Quando o peso não foi informado na coleta e a quantidade adquirida em Kg foi imputada pelo valor médio/ Kg do produto
(2) Quando a quantidade não foi informada na coleta e a quantidade adquirida em Kg foi imputada pelo valor médio/ Kg do produto
(3) Quando o peso e a quantidade não foram informados na coleta e a quantidade adquirida em Kg foi imputada pelo valor médio/ Kg do produto
(4) Quando o peso não foi informado na coleta e a quantidade adquirida em Kg foi imputada pela variável unidade de medida do produto.
(5) Quando a quantidade adquirida em Kg calculada foi considerada como outlier na cada inferior (menor que P05) e posteriormente foi imputada pelo valor médio/ Kg do produto
(6) Quando a quantidade adquirida em Kg calculada foi considerada como outlier na cada superior (maior que P95) e posteriormente foi imputada pelo valor médio/ Kg do produto
(7) Quando a quantidade adquirida em kg não pode ser imputada pelo valor médio/KG do produto devido a inexistência de parâmetros. Neste caso, a mesma foi imputada pelo valor médio/kg do sub grupo agregado.


VARIÁVEIS ESPECÍFICAS DO REGISTRO TIPO 14: RENDIMENTOS E DEDUÇÕES – POF5 / QUADRO 53


CLASSIFICAÇÃO: 700 – TIPO DE TRABALHO

     Quantidade de Categorias: 2
     Número de Dígitos: 1

Categorias por Código:
         1 - Principal
         2 - Secundário

Categorias por Nome:
         1 - Principal
         2 - Secundário


CLASSIFICAÇÃO: 701 – POSIÇÃO NA OCUPAÇÃO

       Quantidade de Categorias: 9
       Número de Dígitos: 2

       Categorias por Código:

                01 – Empregado Privado
                02 – Empregado Público
                03 – Empregado Doméstico
                04 – Empregado Temporário na Área Rural
                05 – Empregador
                06 – Conta-própria
                07 – Aprendiz ou Estagiário
                08 – Não-remunerado em Ajuda a Membro do Domicílio
                09 – Trabalhador na Produção para o Próprio Consumo


        Categorias por Nome:

                07 – Aprendiz ou Estagiário
                06 – Conta-própria
                03 – Empregado Doméstico
                01 – Empregado Privado
                02 – Empregado Público
                04 – Empregado Temporário na Área Rural
                05 – Empregador
                08 – Não-remunerado em Ajuda a Membro do Domicílio
                09 – Trabalhador na Produção para o Próprio Consumo


      CLASSIFICAÇÃO: 702 – FORMA DO ÚLTIMO RENDIMENTO

       Quantidade de Categorias: 4
       Número de Dígitos: 1

       Categorias por Código:


     0 – Não Tem
                1 – Em Dinheiro
                2 – Em Dinheiro e Benefícios
                3 – Somente Benefícios

         Categorias por Nome:

                    1 – Em Dinheiro
                    2 – Em Dinheiro e Benefícios
                    0 – Não Tem
                    3 – Somente Benefícios


       CLASSIFICAÇÃO: 703 – EXISTÊNCIA DE DEDUÇÃO

       Quantidade de Categorias: 3
       Número de Dígitos: 1


         Categorias por Código:

		0 - Não se aplica
        	1 – Sim
            2 - Não

          Categorias por Nome:

           	2 – Não
		0 - Não se aplica
        	1 – Sim


CLASSIFICAÇÃO: 704 – CÓDIGO DE PREVIDÊNCIA PÚBLICA

   Quantidade de Categorias: 9
   Número de Dígitos: 5


  Categorias por Código:
```

| 50101 | EMPREGADO PRIVADO (PREVIDENCIA PUBLICA) |
| --- | --- |
| 50201 | EMPREGADO PUBLICO (PREVIDENCIA PUBLICA) |
| 50301 | EMPREGADO DOMESTICO (PREVIDENCIA PUBLICA) |
| 50401 | EMPREGADO TEMPORARIO NA AREA RURAL (PREVIDENCIA PUBLICA) |
| 50501 | EMPREGADOR (PREVIDENCIA PUBLICA) |
| 50601 | CONTA-PROPRIA (PREVIDENCIA PUBLICA) |
| 50701 | APRENDIZ OU ESTAGIARIO (PREVIDENCIA PUBLICA) |
| 50801 | NAO-REMUNERADO EM AJUDA A MEMBRO DO DOMICILIO (PREVIDENCIA PUBLICA) |
| 50901 | TRABALHADOR NA PRODUCAO PARA O PROPRIO CONSUMO/USO (PREVIDENCIA PUBLICA) |

Categorias por Nome:

| 50701 | APRENDIZ OU ESTAGIARIO (PREVIDENCIA PUBLICA) |
| --- | --- |
| 50601 | CONTA-PROPRIA (PREVIDENCIA PUBLICA) |
| 50301 | EMPREGADO DOMESTICO (PREVIDENCIA PUBLICA) |
| 50101 | EMPREGADO PRIVADO (PREVIDENCIA PUBLICA) |
| 50201 | EMPREGADO PUBLICO (PREVIDENCIA PUBLICA) |
| 50401 | EMPREGADO TEMPORARIO NA AREA RURAL (PREVIDENCIA PUBLICA) |
| 50501 | EMPREGADOR (PREVIDENCIA PUBLICA) |
| 50801 | NAO-REMUNERADO EM AJUDA A MEMBRO DO DOMICILIO (PREVIDENCIA PUBLICA) |
| 50901 | TRABALHADOR NA PRODUCAO PARA O PROPRIO CONSUMO/USO (PREVIDENCIA PUBLICA) |

```
CLASSIFICAÇÃO: 705 – CÓDIGO DE IMPOSTO DE RENDA


   Quantidade de Categorias: 9
   Número de Dígitos: 5


  Categorias por Código:
```

| 60101 | EMPREGADO PRIVADO (PREVIDENCIA PUBLICA) |
| --- | --- |
| 60201 | EMPREGADO PUBLICO (PREVIDENCIA PUBLICA) |
| 60301 | EMPREGADO DOMESTICO (PREVIDENCIA PUBLICA) |
| 60401 | EMPREGADO TEMPORARIO NA AREA RURAL (PREVIDENCIA PUBLICA) |
| 60501 | EMPREGADOR (PREVIDENCIA PUBLICA) |
| 60601 | CONTA-PROPRIA (PREVIDENCIA PUBLICA) |
| 60701 | APRENDIZ OU ESTAGIARIO (PREVIDENCIA PUBLICA) |
| 60801 | NAO-REMUNERADO EM AJUDA A MEMBRO DO DOMICILIO (PREVIDENCIA PUBLICA) |
| 60901 | TRABALHADOR NA PRODUCAO PARA O PROPRIO CONSUMO/USO (PREVIDENCIA PUBLICA) |

Categorias por Nome:

| 60701 | APRENDIZ OU ESTAGIARIO (PREVIDENCIA PUBLICA) |
| --- | --- |
| 60601 | CONTA-PROPRIA (PREVIDENCIA PUBLICA) |
| 60301 | EMPREGADO DOMESTICO (PREVIDENCIA PUBLICA) |
| 60101 | EMPREGADO PRIVADO (PREVIDENCIA PUBLICA) |
| 60201 | EMPREGADO PUBLICO (PREVIDENCIA PUBLICA) |
| 60401 | EMPREGADO TEMPORARIO NA AREA RURAL (PREVIDENCIA PUBLICA) |
| 60501 | EMPREGADOR (PREVIDENCIA PUBLICA) |
| 60801 | NAO-REMUNERADO EM AJUDA A MEMBRO DO DOMICILIO (PREVIDENCIA PUBLICA) |
| 60901 | TRABALHADOR NA PRODUCAO PARA O PROPRIO CONSUMO/USO (PREVIDENCIA PUBLICA) |

```
CLASSIFICAÇÃO: 706 – CÓDIGO DE OUTRAS DEDUÇÕES


   Quantidade de Categorias: 9
   Número de Dígitos: 5


  Categorias por Código:
```

| 70101 | EMPREGADO PRIVADO (PREVIDENCIA PUBLICA) |
| --- | --- |
| 70201 | EMPREGADO PUBLICO (PREVIDENCIA PUBLICA) |
| 70301 | EMPREGADO DOMESTICO (PREVIDENCIA PUBLICA) |
| 70401 | EMPREGADO TEMPORARIO NA AREA RURAL (PREVIDENCIA PUBLICA) |
| 70501 | EMPREGADOR (PREVIDENCIA PUBLICA) |
| 70601 | CONTA-PROPRIA (PREVIDENCIA PUBLICA) |
| 70701 | APRENDIZ OU ESTAGIARIO (PREVIDENCIA PUBLICA) |
| 70801 | NAO-REMUNERADO EM AJUDA A MEMBRO DO DOMICILIO (PREVIDENCIA PUBLICA) |
| 70901 | TRABALHADOR NA PRODUCAO PARA O PROPRIO CONSUMO/USO (PREVIDENCIA PUBLICA) |

Categorias por Nome:

| 70701 | APRENDIZ OU ESTAGIARIO (PREVIDENCIA PUBLICA) |
| --- | --- |
| 70601 | CONTA-PROPRIA (PREVIDENCIA PUBLICA) |
| 70301 | EMPREGADO DOMESTICO (PREVIDENCIA PUBLICA) |
| 70101 | EMPREGADO PRIVADO (PREVIDENCIA PUBLICA) |
| 70201 | EMPREGADO PUBLICO (PREVIDENCIA PUBLICA) |
| 70401 | EMPREGADO TEMPORARIO NA AREA RURAL (PREVIDENCIA PUBLICA) |
| 70501 | EMPREGADOR (PREVIDENCIA PUBLICA) |
| 70801 | NAO-REMUNERADO EM AJUDA A MEMBRO DO DOMICILIO (PREVIDENCIA PUBLICA) |
| 70901 | TRABALHADOR NA PRODUCAO PARA O PROPRIO CONSUMO/USO (PREVIDENCIA PUBLICA) |

```
VARIÁVEIS ESPECÍFICAS DO REGISTRO TIPO 15: OUTROS RENDIMENTOS – POF5 / QUADROS 54 A 57


CLASSIFICAÇÃO: 800 – CÓDIGO DA DEDUÇÃO

   Quantidade de Categorias: 206
   Número de Dígitos: 5


  Categorias por Código:
```

| 50101 | APOSENTADORIA DO INSTITUTO NACIONAL DE SEGURIDADE SOCIAL (INSS) (DEDUCAO) |
| --- | --- |
| 50102 | APOSENTADORIA (INSS) (DEDUCAO) |
| 50201 | PENSAO DO INSTITUTO NACIONAL DE SEGURIDADE SOCIAL (INSS) (DEDUCAO) |
| 50202 | PENSAO (INSS) (DEDUCAO) |
| 50301 | APOSENTADORIA ( MUNICIPAL, ESTADUAL, FEDERAL) DA PREVIDENCIA PUBLICA (DEDUCAO) |
| 50302 | APOSENTADORIA ( MUNICIPAL, ESTADUAL, FEDERAL) (DEDUCAO) |
| 50401 | PENSAO ( MUNICIPAL, ESTADUAL, FEDERAL) DA PREVIDENCIA PUBLICA (DEDUCAO) |
| 50402 | PENSAO ( MUNICIPAL, ESTADUAL, FEDERAL) (DEDUCAO) |
| 50501 | APOSENTADORIA, SUPLEM., COMPLEM. DA PREVIDENCIA PRIVADA (ABERTA OU FECHADA) (DEDUCAO) |
| 50502 | APOSENTADORIA, COMPLEMENTACAO DE PREVIDENCIA PRIVADA (ABERTA OU FECHADA) (DEDUCAO) |
| 50601 | BOLSA DE ESTUDO (DEDUCAO) |
| 50701 | PENSAO ALIMENTICIA DE NAO MORADOR (DEDUCAO) |
| 50702 | PENSAO ALIMENTICIA DE NAO-MORADOR (DEDUCAO) |
| 50801 | ALUGUEL, USO OU EXPLORACAO DE BENS IMOVEIS (DEDUCAO) |
| 50802 | ARRENDAMENTO DE BENS IMOVEIS (DEDUCAO) |
| 50901 | ALUGUEL, DIREITOS AUTORAIS, PATENTES DE BENS MOVEIS (DEDUCAO) |
| 50902 | ALUGUEL, USO OU EXPLORACAO DE BENS MOVEIS (DEDUCAO) |
| 50903 | ALUGUEL/USO/EXPLORACAO DE BENS MOVEIS (DEDUCAO) |
| 50904 | DIREITOS AUTORAIS (DEDUCAO) |
| 50905 | EXPLORACAO DE PATENTES (DEDUCAO) |
| 51001 | BOLSA-FAMILIA (DEDUCAO) |
| 51101 | BENEFICIO DE PRESTACAO CONTINUADA (BPC, LOAS) (DEDUCAO) |
| 51201 | PROGRAMA DE ERRADICACAO DO TRABALHO INFANTIL (PETI) (DEDUCAO) |
| 51301 | BOLSA-ESCOLA (DEDUCAO) |
| 51401 | PROGRAMAS DE RENDA MINIMA (OUTRAS; MUN.EST.FEDERAL) (DEDUCAO) |
| 51501 | AUXILIO/TIQUETE/CARTAO ALIMENTACAO (DEDUCAO) |
| 51502 | TIQUETE-ALIMENTACAO (DEDUCAO) |
| 51503 | TIQUETE-REFEICAO (DEDUCAO) |
| 51504 | CARTAO-REFEICAO (DEDUCAO) |
| 51601 | AUXILIO/VALE/CARTAO TRANSPORTE E COMBUSTIVEL (DEDUCAO) |
| 51602 | VALE-TRANSPORTE (DEDUCAO) |
| 51603 | AUXILIO-COMBUSTIVEL (DEDUCAO) |
| 51604 | CARTAO-TRANSPORTE (DEDUCAO) |
| 51701 | AUXILIO-ATIVIDADE (DEDUCAO) |
| 51702 | ADICIONAL DE INSALUBRIDADE (DEDUCAO) |
| 51703 | ADICIONAL DE PERICULOSIDADE (DEDUCAO) |
| 51704 | ADICIONAL NOTURNO (DEDUCAO) |
| 51801 | AUXILIO-FARDAMENTO (DEDUCAO) |
| 51901 | AUXILIO-ESTIAGEM (DEDUCAO) |
| 52001 | AUXILIO-COMUNICACAO (DEDUCAO) |
| 52101 | AUXILIO ENERGIA ELETRICA (DEDUCAO) |
| 52201 | AGENTE JOVEM - PROGRAMA GOVERNAMENTAL PARA JOVEM DESEMPREGADO (DEDUCAO) |
| 52301 | AUXILIO-DOENCA DA PREVIDENCIA PRIVADA (DEDUCAO) |
| 52401 | AUXILIO A PORTADORES DE DEFICIENCIA FISICA (DEDUCAO) |
| 52501 | AUXILIO-DOENCA DA PREVIDENCIA PUBLICA (DEDUCAO) |
| 52601 | MESADA DE NAO-MORADOR (DEDUCAO) |
| 52602 | MESADA DE NAO MORADOR (DEDUCAO) |
| 52701 | CESTA BASICA (DEDUCAO) |
| 52801 | CARTAO CIDADAO (DEDUCAO) |
| 52901 | AUXILIO-LEITE (DEDUCAO) |
| 53001 | BOLSA-RENDA (DEDUCAO) |
| 53101 | AUXILIO-GAS (DEDUCAO) |
| 53201 | DOACAO DE NAO-MORADOR (DEDUCAO) |
| 53202 | DOACAO DE NAO MORADOR (DEDUCAO) |
| 53301 | RENDIMENTO DE MORADOR AUSENTE (DEDUCAO) |
| 53401 | RENDIMENTO DE MENORES DE DEZ ANOS (DEDUCAO) |
| 53501 | AUXILIO DEFESO (DEDUCAO) |
| 53601 | AUXILIO-CRECHE (DEDUCAO) |
| 53701 | AUXILIO PARA PLANO MEDICO (DEDUCAO) |
| 53801 | AUXILIO MORADIA (DEDUCAO) |
| 50101 | RENDIMENTO E ABONO DO PIS/PASEP (DEDUCAO) |
| 50102 | RENDIMENTO DO PIS/PASEP (DEDUCAO) |
| 50103 | ABONO DO PIS/PASEP (DEDUCAO) |
| 50104 | PIS/PASEP (RENDIMENTOS) (DEDUCAO) |
| 50105 | PIS/PASEP (ABONO) (DEDUCAO) |
| 50201 | SAQUE DO PIS/PASEP (TOTAL) (DEDUCAO) |
| 50202 | SAQUE TOTAL DO PIS/PASEP (DEDUCAO) |
| 50301 | PARTICIPACAO NOS LUCROS DE NEGOCIOS (EMPREGADOR) (DEDUCAO) |
| 50401 | RESTITUICAO DO IMPOSTO DE RENDA (DEDUCAO) |
| 50402 | DEVOLUCAO DO IMPOSTO DE RENDA (DEDUCAO) |
| 50501 | HERANCA E OUTRAS TRANSFERENCIAS PATRIMONIAIS (DEDUCAO) |
| 50502 | HERANCA/TRANSFERENCIA PATRIMONIAL (DEDUCAO) |
| 50601 | VENDA DE TERRENO (DEDUCAO) |
| 50701 | VENDA DE AUTOMOVEL (DEDUCAO) |
| 50801 | VENDA DE IMOVEL (EXCETO TERRENO) (DEDUCAO) |
| 50901 | VENDA DE MOTOCICLETA (DEDUCAO) |
| 51001 | GANHO EM JOGOS (LOTERIA, MEGA, ETC.) OU PREMIO (DEDUCAO) |
| 51002 | PREMIO OU GANHO EM JOGOS (LOTERIA, MEGA, ETC.) (DEDUCAO) |
| 51003 | GANHO EM JOGOS (LOTERIA, MEGA, ETC.) (DEDUCAO) |
| 51101 | GANHO COM VIAGEM A TRABALHO (DEDUCAO) |
| 51102 | AJUDA DE CUSTO EM TRABALHO (DEDUCAO) |
| 51201 | RESTITUICAO DE DINHEIRO NA COMPRA POR CARNET (DEDUCAO) |
| 51301 | JUROS DE EMPRESTIMO (DEDUCAO) |
| 51401 | EMPRESTIMO (DEDUCAO) |
| 51401 | RECEBIMENTO DE EMPRESTIMO (DEDUCAO) |
| 51401 | PENHORA (EMPRESTIMO) (DEDUCAO) |
| 51501 | DIVIDENDOS DE ACOES (DINHEIRO) (DEDUCAO) |
| 51601 | VENDA DE BARCO (DEDUCAO) |
| 51701 | SEGURO-DESEMPREGO (DEDUCAO) |
| 51702 | SALARIO-DESEMPREGO (DEDUCAO) |
| 51703 | AUXILIO-DESEMPREGO (DEDUCAO) |
| 51801 | AUXILIO-MATERNIDADE (DEDUCAO) |
| 51802 | AUXILIO-NATALIDADE (DEDUCAO) |
| 51901 | CREDITO-EDUCATIVO (DEDUCAO) |
| 52001 | ACAO JUDICIAL TRABALHISTA (DEDUCAO) |
| 52101 | INDENIZACAO JUDICIAL (DEDUCAO) |
| 52201 | ACIDENTE DE TRABALHO (PREVIDENCIA PUBLICA) (DEDUCAO) |
| 52301 | RESTITUICAO DA PREVIDENCIA PUBLICA (DEDUCAO) |
| 52302 | DEVOLUCAO DA PREVIDENCIA PUBLICA (DEDUCAO) |
| 52401 | PENSIONISTA (RENDIMENTO) (DEDUCAO) |
| 52501 | RECEBIMENTO DE PECULIO (DEDUCAO) |
| 52502 | PECULIO (RECEBIMENTO) (DEDUCAO) |
| 52601 | AUXILIO-EDUCACAO (DEDUCAO) |
| 52602 | AUXILIO-CRECHE (DEDUCAO) |
| 52603 | SALARIO-EDUCACAO (DEDUCAO) |
| 52604 | AUXILIO-ESCOLA (DEDUCAO) |
| 52605 | AUXILIO-MAE GUARDIA (DEDUCAO) |
| 52701 | RESTITUICAO JUDICIAL (DEDUCAO) |
| 52702 | DEVOLUCAO JUDICIAL (DEDUCAO) |
| 52801 | CAIXINHA (DEDUCAO) |
| 52901 | AUXILIO-FUNERAL (DEDUCAO) |
| 53001 | RESTITUICAO DE PENSAO ALIMENTICIA (DEDUCAO) |
| 53002 | DEVOLUCAO DE PENSAO ALIMENTICIA (DEDUCAO) , |
| 53101 | AUXILIO-VELHICE (DEDUCAO) |
| 53201 | DINHEIRO ACHADO (DEDUCAO) |
| 53301 | RESTITUICAO DA PREVIDENCIA PRIVADA (DEDUCAO) |
| 53302 | DEVOLUCAO DA PREVIDENCIA PRIVADA (DEDUCAO) |
| 53401 | SEGURO-HABITACAO (RESTITUICAO) (DEDUCAO) |
| 53402 | TAXA DE CONSERVACAO DE IMOVEL (RESTITUICAO) (DEDUCAO) |
| 53403 | RESTITUICAO DE SEGURO-HABITACAO (DEDUCAO) |
| 53404 | RESTITUICAO DE TAXA DE CONSERVACAO DE IMOVEL (DEDUCAO) |
| 53405 | DEVOLUCAO DE SEGURO-HABITACAO (DEDUCAO) |
| 53406 | DEVOLUCAO DE TAXA DE CONSERVACAO DE IMOVEL (DEDUCAO) |
| 53407 | DEVOLUCAO DE TAXA DE ESGOTO (CIA DE SANEAMENTO ESTADUAL) (DEDUCAO) |
| 53501 | AUXILIO-TRATAMENTO (DEDUCAO) |
| 53601 | VENDA DE CONSORCIO, CARNET E OUTROS (DEDUCAO) |
| 53602 | VENDA DE RELOGIO (DEDUCAO) |
| 53603 | VENDA DE ROUPA (DEDUCAO) |
| 53604 | VENDA DE CAMISA (DEDUCAO) |
| 53605 | VENDA DE CALCA (DEDUCAO) |
| 53606 | VENDA DE VIDEOGAME (DEDUCAO) |
| 53607 | VENDA DE APARELHO SONORO (DEDUCAO) |
| 53608 | VENDA DE TELEVISAO (DEDUCAO) |
| 53609 | VENDA DE COLCHA (DEDUCAO) |
| 53610 | VENDA DE TECIDO (DEDUCAO) |
| 53611 | VENDA DE MAQUINA DE COSTURA (DEDUCAO) |
| 53612 | VENDA DE MOVEL (DEDUCAO) |
| 53613 | VENDA DE VIDEOCASSETE (DEDUCAO) |
| 53614 | VENDA DE APARELHO ELETRODOMESTICO (DEDUCAO) |
| 53615 | VENDA DE CHURRASQUEIRA (DEDUCAO) |
| 53616 | VENDA DE GAIOLA (DEDUCAO) |
| 53617 | VENDA DE JOIA (DEDUCAO) |
| 53618 | VENDA DE MICROCOMPUTADOR (DEDUCAO) |
| 53619 | VENDA DE INSTRUMENTO MUSICAL (DEDUCAO) |
| 53620 | VENDA DE PECAS DE VEICULO (DEDUCAO) |
| 53621 | VENDA DE FRUTAS (DEDUCAO) |
| 53622 | VENDA DE APARELHO DE PRESSAO (TENSIOMETRO) (DEDUCAO) |
| 53623 | VENDA DE REDE (DEDUCAO) |
| 53624 | VENDA DE FURADEIRA (DEDUCAO) |
| 53625 | VENDA DE BUJAO DE GAS (DEDUCAO) |
| 53626 | VENDA DE BOTIJAO DE GAS (DEDUCAO) |
| 53627 | VENDA DE PANELA (DEDUCAO) |
| 53628 | VENDA DE CONSORCIO, CARNE E OUTROS (DEDUCAO) |
| 53629 | VENDA DE ANTENA PARABOLICA (DEDUCAO) |
| 53630 | VENDA DE APARELHO CELULAR (DEDUCAO) |
| 53631 | VENDA DE MAQUINA AGRICOLA (DEDUCAO) |
| 53632 | VENDA DE PRODUTOS AGRICOLAS (DEDUCAO) |
| 53633 | VENDA DE MIUDEZAS EM GERAL (DEDUCAO) |
| 53701 | DECIMO TERCEIRO SALARIO (DEDUCAO) |
| 53702 | 13° SALARIO (DEDUCAO) |
| 53801 | FERIAS (DEDUCAO) |
| 53802 | ADIANTAMENTO DE FERIAS (DEDUCAO) |
| 53803 | ABONO DE FERIAS (DEDUCAO) |
| 53804 | AGREGADO FERIAS E DECIMO TERCEIRO (DEDUCAO) |
| 53805 | ABONO PECUNIARIO DE FERIAS (DEDUCAO) |
| 53901 | DIFERENCA SALARIAL (DEDUCAO) |
| 53902 | PREMIO POR DESEMPENHO DE ATIVIDADE (DEDUCAO) |
| 53903 | BONIFICACAO RECEBIDA DA EMPRESA (DEDUCAO) |
| 54001 | AVISO PREVIO (DEDUCAO) |
| 54002 | RESCISAO CONTRATUAL (DEDUCAO) |
| 54101 | HORA EXTRA (DEDUCAO) |
| 54201 | REVISAO CONTRATUAL (DEDUCAO) |
| 54301 | AJUDA DE CUSTO DE TRANSFERENCIA DE LOCAL DE TRABALHO (DEDUCAO) |
| 54401 | TITULO DE CAPITALIZACAO (RESGATE) (DEDUCAO) |
| 54402 | RESGATE DE TITULO DE CAPITALIZACAO (DEDUCAO) |
| 54501 | PARTICIPACAO NOS LUCROS DE EMPRESA (EMPREGADO) (DEDUCAO) |
| 54601 | VENDA DE MATERIAL DE CONSTRUCAO (DEDUCAO) |
| 54602 | VENDA DE TIJOLO (DEDUCAO) |
| 54701 | VENDA DE VEICULO DE TRACAO ANIMAL OU ANIMAL DE TRACAO (DEDUCAO) |
| 54702 | VENDA DE ANIMAL DE TRACAO (DEDUCAO) |
| 54703 | VENDA DE CARROCA (DEDUCAO) |
| 54704 | VENDA DE ANIMAL DOMESTICO (DEDUCAO) |
| 54705 | VENDA DE ANIMAL (DEDUCAO) |
| 54801 | VENDA DE BICICLETA (DEDUCAO) |
| 54901 | VENDA DE LINHA TELEFONICA (DEDUCAO) |
| 55001 | COMISSAO DE VENDA (ESPORADICA) (DEDUCAO) |
| 55002 | TRABALHOS EVENTUAIS (DEDUCAO) |
| 55101 | RESTITUICAO DE CONVENIO DE SAUDE (DEDUCAO) |
| 55102 | DEVOLUCAO DE CONVENIO DE SAUDE (DEDUCAO) |
| 55103 | RESTITUICAO DE PLANO DE SAUDE (DEDUCAO) |
| 55104 | DEVOLUCAO DE PLANO DE SAUDE (DEDUCAO) |
| 55201 | RESTITUICAO DE MULTA DE TRANSITO (DEDUCAO) |
| 55202 | DEVOLUCAO DE MULTA DE TRANSITO (DEDUCAO) |
| 55401 | PRESENTE EM DINHEIRO (ESPORADICO) (DEDUCAO) |
| 55501 | INDENIZACAO E RESTITUICAO DE SEGURO PESSOAL (DEDUCAO) |
| 55502 | PREMIO RESTITUIDO E INDENIZACAO PAGOS POR SEGURO PESSOAL (DEDUCAO) |
| 55601 | INDENIZACAO E RESTITUICAO DE SEGURO OBRIGATORIO DE AUTO/UTILITARIOS PESSOAL (DEDUCAO) |
| 55602 | PREMIO RESTITUIDO E INDENIZACAO PAGOS POR SEGURO OBRIGATORIO DE AUTO/UTILITARIOS (DEDUCAO) |
| 55701 | INDENIZACAO E RESTITUICAO DE SEGURO VOLUNTARIO DE AUTO/UTILITARIOS PESSOAL (DEDUCAO) |
| 55702 | PREMIO RESTITUIDO E INDENIZACAO PAGOS POR SEGURO VOLUNTARIO DE AUTO/UTILITARIOS (DEDUCAO) |
| 55801 | INDENIZACAO E RESTITUICAO DE SEGURO OBRIGATORIO DE MOTO (DEDUCAO) |
| 55802 | PREMIO RESTITUIDO E INDENIZACAO PAGOS POR SEGURO OBRIGATORIO DE MOTO (DEDUCAO) |
| 55901 | INDENIZACAO E RESTITUICAO DE SEGURO VOLUNTARIO DE MOTO (DEDUCAO) |
| 55902 | PREMIO RESTITUIDO E INDENIZACAO PAGOS POR SEGURO VOLUNTARIO DE MOTO (DEDUCAO) |
| 56201 | SAQUE DO FGTS (DEDUCAO) |
| 56301 | INDENIZACAO TRABALISTA (DEDUCAO) |

Categorias por Nome:

| 53702 | 13° SALARIO (DEDUCAO) |
| --- | --- |
| 53803 | ABONO DE FERIAS (DEDUCAO) |
| 50103 | ABONO DO PIS/PASEP (DEDUCAO) |
| 53805 | ABONO PECUNIARIO DE FERIAS (DEDUCAO) |
| 52001 | ACAO JUDICIAL TRABALHISTA (DEDUCAO) |
| 52201 | ACIDENTE DE TRABALHO (PREVIDENCIA PUBLICA) (DEDUCAO) |
| 53802 | ADIANTAMENTO DE FERIAS (DEDUCAO) |
| 51702 | ADICIONAL DE INSALUBRIDADE (DEDUCAO) |
| 51703 | ADICIONAL DE PERICULOSIDADE (DEDUCAO) |
| 51704 | ADICIONAL NOTURNO (DEDUCAO) |
| 52201 | AGENTE JOVEM - PROGRAMA GOVERNAMENTAL PARA JOVEM DESEMPREGADO (DEDUCAO) |
| 53804 | AGREGADO FERIAS E DECIMO TERCEIRO (DEDUCAO) |
| 54301 | AJUDA DE CUSTO DE TRANSFERENCIA DE LOCAL DE TRABALHO (DEDUCAO) |
| 51102 | AJUDA DE CUSTO EM TRABALHO (DEDUCAO) |
| 50901 | ALUGUEL, DIREITOS AUTORAIS, PATENTES DE BENS MOVEIS (DEDUCAO) |
| 50801 | ALUGUEL, USO OU EXPLORACAO DE BENS IMOVEIS (DEDUCAO) |
| 50902 | ALUGUEL, USO OU EXPLORACAO DE BENS MOVEIS (DEDUCAO) |
| 50903 | ALUGUEL/USO/EXPLORACAO DE BENS MOVEIS (DEDUCAO) |
| 50302 | APOSENTADORIA ( MUNICIPAL, ESTADUAL, FEDERAL) (DEDUCAO) |
| 50301 | APOSENTADORIA ( MUNICIPAL, ESTADUAL, FEDERAL) DA PREVIDENCIA PUBLICA (DEDUCAO) |
| 50102 | APOSENTADORIA (INSS) (DEDUCAO) |
| 50101 | APOSENTADORIA DO INSTITUTO NACIONAL DE SEGURIDADE SOCIAL (INSS) (DEDUCAO) |
| 50502 | APOSENTADORIA, COMPLEMENTACAO DE PREVIDENCIA PRIVADA (ABERTA OU FECHADA) (DEDUCAO) |
| 50501 | APOSENTADORIA, SUPLEM., COMPLEM. DA PREVIDENCIA PRIVADA (ABERTA OU FECHADA) (DEDUCAO) |
| 50802 | ARRENDAMENTO DE BENS IMOVEIS (DEDUCAO) |
| 52401 | AUXILIO A PORTADORES DE DEFICIENCIA FISICA (DEDUCAO) |
| 53501 | AUXILIO DEFESO (DEDUCAO) |
| 52101 | AUXILIO ENERGIA ELETRICA (DEDUCAO) |
| 53801 | AUXILIO MORADIA (DEDUCAO) |
| 53701 | AUXILIO PARA PLANO MEDICO (DEDUCAO) |
| 51501 | AUXILIO/TIQUETE/CARTAO ALIMENTACAO (DEDUCAO) |
| 51601 | AUXILIO/VALE/CARTAO TRANSPORTE E COMBUSTIVEL (DEDUCAO) |
| 51701 | AUXILIO-ATIVIDADE (DEDUCAO) |
| 51603 | AUXILIO-COMBUSTIVEL (DEDUCAO) |
| 52001 | AUXILIO-COMUNICACAO (DEDUCAO) |
| 53601 | AUXILIO-CRECHE (DEDUCAO) |
| 52602 | AUXILIO-CRECHE (DEDUCAO) |
| 51703 | AUXILIO-DESEMPREGO (DEDUCAO) |
| 52301 | AUXILIO-DOENCA DA PREVIDENCIA PRIVADA (DEDUCAO) |
| 52501 | AUXILIO-DOENCA DA PREVIDENCIA PUBLICA (DEDUCAO) |
| 52601 | AUXILIO-EDUCACAO (DEDUCAO) |
| 52604 | AUXILIO-ESCOLA (DEDUCAO) |
| 51901 | AUXILIO-ESTIAGEM (DEDUCAO) |
| 51801 | AUXILIO-FARDAMENTO (DEDUCAO) |
| 52901 | AUXILIO-FUNERAL (DEDUCAO) |
| 53101 | AUXILIO-GAS (DEDUCAO) |
| 52901 | AUXILIO-LEITE (DEDUCAO) |
| 52605 | AUXILIO-MAE GUARDIA (DEDUCAO) |
| 51801 | AUXILIO-MATERNIDADE (DEDUCAO) |
| 51802 | AUXILIO-NATALIDADE (DEDUCAO) |
| 53501 | AUXILIO-TRATAMENTO (DEDUCAO) |
| 53101 | AUXILIO-VELHICE (DEDUCAO) |
| 54001 | AVISO PREVIO (DEDUCAO) |
| 51101 | BENEFICIO DE PRESTACAO CONTINUADA (BPC, LOAS) (DEDUCAO) |
| 50601 | BOLSA DE ESTUDO (DEDUCAO) |
| 51301 | BOLSA-ESCOLA (DEDUCAO) |
| 51001 | BOLSA-FAMILIA (DEDUCAO) |
| 53001 | BOLSA-RENDA (DEDUCAO) |
| 53903 | BONIFICACAO RECEBIDA DA EMPRESA (DEDUCAO) |
| 52801 | CAIXINHA (DEDUCAO) |
| 52801 | CARTAO CIDADAO (DEDUCAO) |
| 51504 | CARTAO-REFEICAO (DEDUCAO) |
| 51604 | CARTAO-TRANSPORTE (DEDUCAO) |
| 52701 | CESTA BASICA (DEDUCAO) |
| 55001 | COMISSAO DE VENDA (ESPORADICA) (DEDUCAO) |
| 51901 | CREDITO-EDUCATIVO (DEDUCAO) |
| 53701 | DECIMO TERCEIRO SALARIO (DEDUCAO) |
| 53302 | DEVOLUCAO DA PREVIDENCIA PRIVADA (DEDUCAO) |
| 52302 | DEVOLUCAO DA PREVIDENCIA PUBLICA (DEDUCAO) |
| 55102 | DEVOLUCAO DE CONVENIO DE SAUDE (DEDUCAO) |
| 55202 | DEVOLUCAO DE MULTA DE TRANSITO (DEDUCAO) |
| 53002 | DEVOLUCAO DE PENSAO ALIMENTICIA (DEDUCAO) , |
| 55104 | DEVOLUCAO DE PLANO DE SAUDE (DEDUCAO) |
| 53405 | DEVOLUCAO DE SEGURO-HABITACAO (DEDUCAO) |
| 53406 | DEVOLUCAO DE TAXA DE CONSERVACAO DE IMOVEL (DEDUCAO) |
| 53407 | DEVOLUCAO DE TAXA DE ESGOTO (CIA DE SANEAMENTO ESTADUAL) (DEDUCAO) |
| 50402 | DEVOLUCAO DO IMPOSTO DE RENDA (DEDUCAO) |
| 52702 | DEVOLUCAO JUDICIAL (DEDUCAO) |
| 53901 | DIFERENCA SALARIAL (DEDUCAO) |
| 53201 | DINHEIRO ACHADO (DEDUCAO) |
| 50904 | DIREITOS AUTORAIS (DEDUCAO) |
| 51501 | DIVIDENDOS DE ACOES (DINHEIRO) (DEDUCAO) |
| 53202 | DOACAO DE NAO MORADOR (DEDUCAO) |
| 53201 | DOACAO DE NAO-MORADOR (DEDUCAO) |
| 51401 | EMPRESTIMO (DEDUCAO) |
| 50905 | EXPLORACAO DE PATENTES (DEDUCAO) |
| 53801 | FERIAS (DEDUCAO) |
| 51101 | GANHO COM VIAGEM A TRABALHO (DEDUCAO) |
| 51003 | GANHO EM JOGOS (LOTERIA, MEGA, ETC.) (DEDUCAO) |
| 51001 | GANHO EM JOGOS (LOTERIA, MEGA, ETC.) OU PREMIO (DEDUCAO) |
| 50501 | HERANCA E OUTRAS TRANSFERENCIAS PATRIMONIAIS (DEDUCAO) |
| 50502 | HERANCA/TRANSFERENCIA PATRIMONIAL (DEDUCAO) |
| 54101 | HORA EXTRA (DEDUCAO) |
| 55601 | INDENIZACAO E RESTITUICAO DE SEGURO OBRIGATORIO DE AUTO/UTILITARIOS PESSOAL (DEDUCAO) |
| 55801 | INDENIZACAO E RESTITUICAO DE SEGURO OBRIGATORIO DE MOTO (DEDUCAO) |
| 55501 | INDENIZACAO E RESTITUICAO DE SEGURO PESSOAL (DEDUCAO) |
| 55701 | INDENIZACAO E RESTITUICAO DE SEGURO VOLUNTARIO DE AUTO/UTILITARIOS PESSOAL (DEDUCAO) |
| 55901 | INDENIZACAO E RESTITUICAO DE SEGURO VOLUNTARIO DE MOTO (DEDUCAO) |
| 52101 | INDENIZACAO JUDICIAL (DEDUCAO) |
| 56301 | INDENIZACAO TRABALISTA (DEDUCAO) |
| 51301 | JUROS DE EMPRESTIMO (DEDUCAO) |
| 52602 | MESADA DE NAO MORADOR (DEDUCAO) |
| 52601 | MESADA DE NAO-MORADOR (DEDUCAO) |
| 54501 | PARTICIPACAO NOS LUCROS DE EMPRESA (EMPREGADO) (DEDUCAO) |
| 50301 | PARTICIPACAO NOS LUCROS DE NEGOCIOS (EMPREGADOR) (DEDUCAO) |
| 52502 | PECULIO (RECEBIMENTO) (DEDUCAO) |
| 51401 | PENHORA (EMPRESTIMO) (DEDUCAO) |
| 50402 | PENSAO ( MUNICIPAL, ESTADUAL, FEDERAL) (DEDUCAO) |
| 50401 | PENSAO ( MUNICIPAL, ESTADUAL, FEDERAL) DA PREVIDENCIA PUBLICA (DEDUCAO) |
| 50202 | PENSAO (INSS) (DEDUCAO) |
| 50701 | PENSAO ALIMENTICIA DE NAO MORADOR (DEDUCAO) |
| 50702 | PENSAO ALIMENTICIA DE NAO-MORADOR (DEDUCAO) |
| 50201 | PENSAO DO INSTITUTO NACIONAL DE SEGURIDADE SOCIAL (INSS) (DEDUCAO) |
| 52401 | PENSIONISTA (RENDIMENTO) (DEDUCAO) |
| 50105 | PIS/PASEP (ABONO) (DEDUCAO) |
| 50104 | PIS/PASEP (RENDIMENTOS) (DEDUCAO) |
| 51002 | PREMIO OU GANHO EM JOGOS (LOTERIA, MEGA, ETC.) (DEDUCAO) |
| 53902 | PREMIO POR DESEMPENHO DE ATIVIDADE (DEDUCAO) |
| 55602 | PREMIO RESTITUIDO E INDENIZACAO PAGOS POR SEGURO OBRIGATORIO DE AUTO/UTILITARIOS (DEDUCAO) |
| 55802 | PREMIO RESTITUIDO E INDENIZACAO PAGOS POR SEGURO OBRIGATORIO DE MOTO (DEDUCAO) |
| 55502 | PREMIO RESTITUIDO E INDENIZACAO PAGOS POR SEGURO PESSOAL (DEDUCAO) |
| 55702 | PREMIO RESTITUIDO E INDENIZACAO PAGOS POR SEGURO VOLUNTARIO DE AUTO/UTILITARIOS (DEDUCAO) |
| 55902 | PREMIO RESTITUIDO E INDENIZACAO PAGOS POR SEGURO VOLUNTARIO DE MOTO (DEDUCAO) |
| 55401 | PRESENTE EM DINHEIRO (ESPORADICO) (DEDUCAO) |
| 51201 | PROGRAMA DE ERRADICACAO DO TRABALHO INFANTIL (PETI) (DEDUCAO) |
| 51401 | PROGRAMAS DE RENDA MINIMA (OUTRAS; MUN.EST.FEDERAL) (DEDUCAO) |
| 51401 | RECEBIMENTO DE EMPRESTIMO (DEDUCAO) |
| 52501 | RECEBIMENTO DE PECULIO (DEDUCAO) |
| 53401 | RENDIMENTO DE MENORES DE DEZ ANOS (DEDUCAO) |
| 53301 | RENDIMENTO DE MORADOR AUSENTE (DEDUCAO) |
| 50102 | RENDIMENTO DO PIS/PASEP (DEDUCAO) |
| 50101 | RENDIMENTO E ABONO DO PIS/PASEP (DEDUCAO) |
| 54002 | RESCISAO CONTRATUAL (DEDUCAO) |
| 54402 | RESGATE DE TITULO DE CAPITALIZACAO (DEDUCAO) |
| 53301 | RESTITUICAO DA PREVIDENCIA PRIVADA (DEDUCAO) |
| 52301 | RESTITUICAO DA PREVIDENCIA PUBLICA (DEDUCAO) |
| 55101 | RESTITUICAO DE CONVENIO DE SAUDE (DEDUCAO) |
| 51201 | RESTITUICAO DE DINHEIRO NA COMPRA POR CARNET (DEDUCAO) |
| 55201 | RESTITUICAO DE MULTA DE TRANSITO (DEDUCAO) |
| 53001 | RESTITUICAO DE PENSAO ALIMENTICIA (DEDUCAO) |
| 55103 | RESTITUICAO DE PLANO DE SAUDE (DEDUCAO) |
| 53403 | RESTITUICAO DE SEGURO-HABITACAO (DEDUCAO) |
| 53404 | RESTITUICAO DE TAXA DE CONSERVACAO DE IMOVEL (DEDUCAO) |
| 50401 | RESTITUICAO DO IMPOSTO DE RENDA (DEDUCAO) |
| 52701 | RESTITUICAO JUDICIAL (DEDUCAO) |
| 54201 | REVISAO CONTRATUAL (DEDUCAO) |
| 51702 | SALARIO-DESEMPREGO (DEDUCAO) |
| 52603 | SALARIO-EDUCACAO (DEDUCAO) |
| 56201 | SAQUE DO FGTS (DEDUCAO) |
| 50201 | SAQUE DO PIS/PASEP (TOTAL) (DEDUCAO) |
| 50202 | SAQUE TOTAL DO PIS/PASEP (DEDUCAO) |
| 51701 | SEGURO-DESEMPREGO (DEDUCAO) |
| 53401 | SEGURO-HABITACAO (RESTITUICAO) (DEDUCAO) |
| 53402 | TAXA DE CONSERVACAO DE IMOVEL (RESTITUICAO) (DEDUCAO) |
| 51502 | TIQUETE-ALIMENTACAO (DEDUCAO) |
| 51503 | TIQUETE-REFEICAO (DEDUCAO) |
| 54401 | TITULO DE CAPITALIZACAO (RESGATE) (DEDUCAO) |
| 55002 | TRABALHOS EVENTUAIS (DEDUCAO) |
| 51602 | VALE-TRANSPORTE (DEDUCAO) |
| 54705 | VENDA DE ANIMAL (DEDUCAO) |
| 54702 | VENDA DE ANIMAL DE TRACAO (DEDUCAO) |
| 54704 | VENDA DE ANIMAL DOMESTICO (DEDUCAO) |
| 53629 | VENDA DE ANTENA PARABOLICA (DEDUCAO) |
| 53630 | VENDA DE APARELHO CELULAR (DEDUCAO) |
| 53622 | VENDA DE APARELHO DE PRESSAO (TENSIOMETRO) (DEDUCAO) |
| 53614 | VENDA DE APARELHO ELETRODOMESTICO (DEDUCAO) |
| 53607 | VENDA DE APARELHO SONORO (DEDUCAO) |
| 50701 | VENDA DE AUTOMOVEL (DEDUCAO) |
| 51601 | VENDA DE BARCO (DEDUCAO) |
| 54801 | VENDA DE BICICLETA (DEDUCAO) |
| 53626 | VENDA DE BOTIJAO DE GAS (DEDUCAO) |
| 53625 | VENDA DE BUJAO DE GAS (DEDUCAO) |
| 53605 | VENDA DE CALCA (DEDUCAO) |
| 53604 | VENDA DE CAMISA (DEDUCAO) |
| 54703 | VENDA DE CARROCA (DEDUCAO) |
| 53615 | VENDA DE CHURRASQUEIRA (DEDUCAO) |
| 53609 | VENDA DE COLCHA (DEDUCAO) |
| 53628 | VENDA DE CONSORCIO, CARNE E OUTROS (DEDUCAO) |
| 53601 | VENDA DE CONSORCIO, CARNET E OUTROS (DEDUCAO) |
| 53621 | VENDA DE FRUTAS (DEDUCAO) |
| 53624 | VENDA DE FURADEIRA (DEDUCAO) |
| 53616 | VENDA DE GAIOLA (DEDUCAO) |
| 50801 | VENDA DE IMOVEL (EXCETO TERRENO) (DEDUCAO) |
| 53619 | VENDA DE INSTRUMENTO MUSICAL (DEDUCAO) |
| 53617 | VENDA DE JOIA (DEDUCAO) |
| 54901 | VENDA DE LINHA TELEFONICA (DEDUCAO) |
| 53631 | VENDA DE MAQUINA AGRICOLA (DEDUCAO) |
| 53611 | VENDA DE MAQUINA DE COSTURA (DEDUCAO) |
| 54601 | VENDA DE MATERIAL DE CONSTRUCAO (DEDUCAO) |
| 53618 | VENDA DE MICROCOMPUTADOR (DEDUCAO) |
| 53633 | VENDA DE MIUDEZAS EM GERAL (DEDUCAO) |
| 50901 | VENDA DE MOTOCICLETA (DEDUCAO) |
| 53612 | VENDA DE MOVEL (DEDUCAO) |
| 53627 | VENDA DE PANELA (DEDUCAO) |
| 53620 | VENDA DE PECAS DE VEICULO (DEDUCAO) |
| 53632 | VENDA DE PRODUTOS AGRICOLAS (DEDUCAO) |
| 53623 | VENDA DE REDE (DEDUCAO) |
| 53602 | VENDA DE RELOGIO (DEDUCAO) |
| 53603 | VENDA DE ROUPA (DEDUCAO) |
| 53610 | VENDA DE TECIDO (DEDUCAO) |
| 53608 | VENDA DE TELEVISAO (DEDUCAO) |
| 50601 | VENDA DE TERRENO (DEDUCAO) |
| 54602 | VENDA DE TIJOLO (DEDUCAO) |
| 54701 | VENDA DE VEICULO DE TRACAO ANIMAL OU ANIMAL DE TRACAO (DEDUCAO) |
| 53613 | VENDA DE VIDEOCASSETE (DEDUCAO) |
| 53606 | VENDA DE VIDEOGAME (DEDUCAO) |

```
VARIÁVEIS UTILIZADAS EM MAIS DE UM REGISTRO

CLASSIFICAÇÃO: 900 - CÓDIGO DA UNIDADE DA FEDERAÇÃO

    Quantidade de Categorias: 27
    Número de Dígitos: 2

  Categorias por Código:

   11 – Rondônia
   12 – Acre
   13 – Amazonas
   14 – Roraima
   15 – Pará
   16 – Amapá
   17 – Tocantins
   21 – Maranhão
   22 – Piauí
   23 – Ceará
   24 – Rio Grande do Norte
   25 – Paraíba
   26 – Pernambuco
   27 – Alagoas
   28 – Sergipe
   29 – Bahia
   31 – Minas Gerais
   32 – Espírito Santo
   33 – Rio de Janeiro
   35 – São Paulo
   41 – Paraná
   42 – Santa Catarina
   43 – Rio Grande do Sul
   50 – Mato Grosso do Sul
   51 – Mato Grosso
   52 – Goiás
   53 – Distrito Federal

   Categorias por Nome:

      12 – Acre
      27 – Alagoas
      16 – Amapá
      13 – Amazonas
      29 – Bahia
      23 – Ceará
      53 – Distrito Federal
      32 – Espírito Santo
      52 – Goiás
      21 – Maranhão
      51 – Mato Grosso
      50 – Mato Grosso do Sul
      31 – Minas Gerais
      15 – Pará
      25 – Paraíba
      41 – Paraná
      26 – Pernambuco
      22 – Piauí
      33 – Rio de Janeiro
      24 – Rio Grande do Norte
      43 – Rio Grande do Sul
      11 – Rondônia
      14 – Roraima
      42 – Santa Catarina
      35 – São Paulo
      28 – Sergipe
      17 – Tocantins

CLASSIFICAÇÃO: 901 – ESTRATO GEOGRÁFICO

   Quantidade de Categorias: 51
   Número de Dígitos: 2

   Categorias por Código e Nome:

          01 – Estrato 1
          02 – Estrato 2
        03 - Estrato 3
          04 – Estrato 4
          05 – Estrato 5
          06 – Estrato 6
          07 – Estrato 7
        08 – Estrato 8
        09 – Estrato 9
        10 – Estrato 10
        11 – Estrato 11
        12 – Estrato 12
        13 – Estrato 13
        14 – Estrato 14
          15 – Estrato 15
          16 – Estrato 16
        17 – Estrato 17
        18 – Estrato 18
        19 – Estrato 19
        20 – Estrato 20
        21 – Estrato 21
          22 – Estrato 22
        23 – Estrato 23
        24 – Estrato 24
          25 – Estrato 25
        26 – Estrato 26
        27 – Estrato 27
          28 – Estrato 28
        29 – Estrato 29
        30 – Estrato 30
        31 – Estrato 31
          32 – Estrato 32
        33 – Estrato 33
        34 – Estrato 34
        35 – Estrato 35
        36 – Estrato 36
        37 – Estrato 37
        38 – Estrato 38
        39 – Estrato 39
        40 – Estrato 40
        41 – Estrato 41
        42 – Estrato 42
        43 – Estrato 43
        44 – Estrato 44
        45 – Estrato 45
        46 – Estrato 46
        47 – Estrato 47
        48 – Estrato 48
        49 – Estrato 49
        50 – Estrato 50
        51 – Estrato 51


CLASSIFICAÇÃO: 902 – CÓDIGO DE IMPUTAÇÃO (OU CÓDIGO DE IMPUTAÇÃO DO INSS EXCLUSIVO DO TIPO REG = 9)

     Quantidade de Categorias: 4
     Número de Dígitos: 2

     Categorias por Código:

            00 – Não Imputado (dado original)
            01 – Imputação do Valor do Item
            02 – Imputação de Quadro
            03 – Imputação de Questionário

     Categorias por Nome:

            02 – Imputação de Quadro
            03 – Imputação de Questionário
01 – Imputação do Valor do Item
         	00 – Não Imputado (dado original)


  CLASSIFICAÇÃO: 903 – FORMA DE AQUISIÇÃO (OU FORMA DA ÚLTIMA AQUISIÇÃO NO CASO DO TIPO REG = 5)


  Quantidade de Categorias: 12
  Número de Dígitos: 2

  Categorias por Código:

    00 – Não Aplicável (somente para os itens do quadro 6)
   01 – Monetária à vista para a unidade de consumo
   02 – Monetária à vista para outra unidade de consumo
   03 – Monetária a prazo para a unidade de consumo
   04 – Monetária a prazo para outra unidade de consumo
   05 - Cartão de crédito à vista para a Unidade de Consumo
   06 - Cartão de crédito à vista para outra Unidade de Consumo
   07 - Doação
   08 – Retirada de negócio
   09 – Troca
   10 – Produção própria
   11 - Outra


   Categorias por Nome:

   05 - Cartão de crédito à vista para a Unidade de Consumo
   06 - Cartão de crédito à vista para outra Unidade de Consumo
    07 - Doação
   03 – Monetária a prazo para a unidade de consumo
   04 – Monetária a prazo para outra unidade de consumo
   01 – Monetária à vista para a unidade de consumo
   02 – Monetária à vista para outra unidade de consumo
   00 – Não Aplicável
   11 - Outra
   10 – Produção própria
   08 – Retirada de negócio
   09 – Troca


   CLASSIFICAÇÃO: 904 – MÊS DA ÚLTIMA DESPESA OU RENDIMENTO


  Quantidade de Categorias: 13
  Número de Dígitos: 2

   Categorias por Código:

   00 - Não se aplica
   01 - Janeiro
   02 - Fevereiro
   03 - Março
   04 - Abril
   05 - Maio
   06 - Junho
   07 - Julho
   08 - Agosto
   09 - Setembro
   10 - Outubro
   11 - Novembro
   12 - Dezembro

   Categorias por nome:

   04 - Abril
   08 - Agosto
   12 - Dezembro
   02 - Fevereiro
   01 - Janeiro
   07 - Julho
   06 – Junho
   05 - Maio
   03 - Março
   00 - Não se aplica
   11 – Novembro
   10 - Outubro
   09 - Setembro


   CLASSIFICAÇÃO: 905 - ESTADO DE AQUISIÇÃO (OU ESTADO DA ÚLTIMA AQUISIÇÃO NO CASO DO TIPO REG = 5)


   Quantidade de Categorias: 3
   Número de Dígitos: 1

Categorias por Código:

   1 - Novo
   3 - Usado
   9 - Não Sabe

  Categorias por Nome:

   9 - Não Sabe
   1 - Novo
   3 - Usado


     CLASSIFICAÇÃO: 906 – NÚMERO DO QUADRO

     Quantidade de Categorias: 56
     Número de Dígitos: 2

     Categorias por Código:
```

| 6 | Serviços e taxas do domicílio principal |
| --- | --- |
| 7 | Aquisição de combustíveis domésticos e outros do domicílio principal |
| 8 | Conservação, manutenção e pequenos reparos com habitação, jazigo e jardinagem |
| 9 | Consertos e manutenção de móveis, aparelhos, máquinas e utensílios de uso doméstico |
| 10 | Aluguel, impostos e outras taxas do domicílio principal |
| 11 | Construção e reforma de habitação e jazigo |
| 12 | Outros itens do domicílio principal com serviços públicos, privados e habitação |
| 13 | Aluguel de aparelhos e utilidades de uso doméstico |
| 14 | Inventário de bens duráveis |
| 15 | Aquisição de aparelhos, máquinas e outras utilidades de uso doméstico |
| 16 | Aquisição de ferramentas, animais domésticos, equipamentos musicais e de acampamento |
| 17 | Aquisição de móveis |
| 18 | Aquisição de artigos de decoração e forração |
| 19 | Serviços domésticos |
| 22 | Comunicações |
| 23 | Transportes coletivos e próprios |
| 24 | Alimentação fora de casa |
| 25 | Aquisição de artigos de fumo |
| 26 | Jogos e Apostas |
| 27 | Aquisição de jornais, revistas e passatempos impressos |
| 28 | Diversões, ingressos para eventos esportivos e culturais e uso de celulares |
| 29 | Aquisição de produtos famacêuticos |
| 30 | Aquisição de artigos de higiene pessoal e maquiagem |
| 31 | Serviços de cuidados pessoais e outros |
| 32 | Aquisição de artigos de papelaria, livros não didáticos e assinatura de periódicos |
| 33 | Aquisição de brinquedos e material de recreação |
| 34 | Aquisição e aluguel de roupas de homem |
| 35 | Aquisição e aluguel de roupas de mulher |
| 36 | Aquisição e aluguel de roupas de criança até 14 anos |
| 37 | Aquisição de artigos de armarinho, tecidos e roupas de banho, cama e mesa |
| 38 | Aquisição e aluguel de bolsas, calçados, cintos e outros acessórios |
| 39 | Aquisição de utensílios avulsos e artigos de banheiro, copa e cozinha |
| 40 | Outras aquisições |
| 41 | Viagens |
| 42 | Serviços de assistência à saúde |
| 43 | Acessórios e manutenção de veículos |
| 44 | Serviços bancários, de cartório, de advogado, de despachante e similares |
| 45 | Cerimônias familiares, práticas religiosas, outras festas e recepções |
| 46 | Aquisição de jóias, relógios, aparelhos e acessórios de telefonia celular |
| 47 | Outros Imóveis |
| 48 | Contribuições, transferências e encargos financeiros |
| 49 | Cursos, livros didáticos, revistas técnicas e outros itens referentes a educação |
| 50 | Veículos: documentação, seguro e outros |
| 51 | Aquisição de veículos |
| 53 | Trabalhos, rendimentos e deduções |
| 54 | Aposentadorias, pensões, auxílios e outros rendimentos habituais e deduções |
| 55 | Outros rendimentos, receitas, empréstimos e deduções esporádicas |
| 56 | Depósito/Aplicação de ativo financeiro |
| 57 | Retirada/Resgate de ativo financeiro |
| 63 | 1° Dia da Caderneta de Aquisição Coletiva |
| 64 | 2° Dia da Caderneta de Aquisição Coletiva |
| 65 | 3° Dia da Caderneta de Aquisição Coletiva |
| 66 | 4° Dia da Caderneta de Aquisição Coletiva |
| 67 | 5° Dia da Caderneta de Aquisição Coletiva |
| 68 | 6° Dia da Caderneta de Aquisição Coletiva |
| 69 | 7° Dia da Caderneta de Aquisição Coletiva |

Categorias por Nome:

| 63 | 1° Dia da Caderneta de Aquisição Coletiva |
| --- | --- |
| 64 | 2° Dia da Caderneta de Aquisição Coletiva |
| 65 | 3° Dia da Caderneta de Aquisição Coletiva |
| 66 | 4° Dia da Caderneta de Aquisição Coletiva |
| 67 | 5° Dia da Caderneta de Aquisição Coletiva |
| 68 | 6° Dia da Caderneta de Aquisição Coletiva |
| 69 | 7° Dia da Caderneta de Aquisição Coletiva |
| 43 | Acessórios e manutenção de veículos |
| 24 | Alimentação fora de casa |
| 13 | Aluguel de aparelhos e utilidades de uso doméstico |
| 10 | Aluguel, impostos e outras taxas do domicílio principal |
| 54 | Aposentadorias, pensões, auxílios e outros rendimentos habituais e deduções |
| 15 | Aquisição de aparelhos, máquinas e outras utilidades de uso doméstico |
| 37 | Aquisição de artigos de armarinho, tecidos e roupas de banho, cama e mesa |
| 18 | Aquisição de artigos de decoração e forração |
| 25 | Aquisição de artigos de fumo |
| 30 | Aquisição de artigos de higiene pessoal e maquiagem |
| 32 | Aquisição de artigos de papelaria, livros não didáticos e assinatura de periódicos |
| 33 | Aquisição de brinquedos e material de recreação |
| 7 | Aquisição de combustíveis domésticos e outros do domicílio principal |
| 16 | Aquisição de ferramentas, animais domésticos, equipamentos musicais e de acampamento |
| 46 | Aquisição de jóias, relógios, aparelhos e acessórios de telefonia celular |
| 27 | Aquisição de jornais, revistas e passatempos impressos |
| 17 | Aquisição de móveis |
| 29 | Aquisição de produtos famacêuticos |
| 39 | Aquisição de utensílios avulsos e artigos de banheiro, copa e cozinha |
| 51 | Aquisição de veículos |
| 38 | Aquisição e aluguel de bolsas, calçados, cintos e outros acessórios |
| 36 | Aquisição e aluguel de roupas de criança até 14 anos |
| 34 | Aquisição e aluguel de roupas de homem |
| 35 | Aquisição e aluguel de roupas de mulher |
| 45 | Cerimônias familiares, práticas religiosas, outras festas e recepções |
| 22 | Comunicações |
| 9 | Consertos e manutenção de móveis, aparelhos, máquinas e utensílios de uso doméstico |
| 8 | Conservação, manutenção e pequenos reparos com habitação, jazigo e jardinagem |
| 11 | Construção e reforma de habitação e jazigo |
| 48 | Contribuições, transferências e encargos financeiros |
| 49 | Cursos, livros didáticos, revistas técnicas e outros itens referentes a educação |
| 56 | Depósito/Aplicação de ativo financeiro |
| 28 | Diversões, ingressos para eventos esportivos e culturais e uso de celulares |
| 14 | Inventário de bens duráveis |
| 26 | Jogos e Apostas |
| 40 | Outras aquisições |
| 47 | Outros Imóveis |
| 12 | Outros itens do domicílio principal com serviços públicos, privados e habitação |
| 55 | Outros rendimentos, receitas, empréstimos e deduções esporádicas |
| 57 | Retirada/Resgate de ativo financeiro |
| 44 | Serviços bancários, de cartório, de advogado, de despachante e similares |
| 42 | Serviços de assistência à saúde |
| 31 | Serviços de cuidados pessoais e outros |
| 19 | Serviços domésticos |
| 6 | Serviços e taxas do domicílio principal |
| 53 | Trabalhos, rendimentos e deduções |
| 23 | Transportes coletivos e próprios |
| 50 | Veículos: documentação, seguro e outros |
| 41 | Viagens |

```
CLASSIFICAÇÃO: 907 – CÓDIGO DO ITEM


   Quantidade de Categorias: 13476
   Número de Dígitos: 5

  Categorias por Código:
```

| 00101 | AGUA E ESGOTO (M3) |
| --- | --- |
| 00201 | ENERGIA ELETRICA (KWH) |
| 00301 | GAS ENCANADO (M3) |
| 00401 | TELEFONE RESIDENCIAL |
| 00501 | ACESSO A INTERNET (PROVEDOR, A CABO, COMUNICACAO VIA SATELITE, ETC.) |
| 00502 | INTERNET BANDA LARGA |
| 00503 | PROVEDOR DE ACESSO A INTERNET DISCADA |
| 00504 | INTERNET SEM FIO |
| 00505 | INTERNET VIA SATELITE |
| 00506 | INTERNET VIA RADIO |
| 00701 | TV POR ASSINATURA (MENSALIDADE/PACOTE) |
| 00801 | TELEFONE FIXO, CELULAR E INTERNET (PACOTE) |
| 00901 | TELEFONE FIXO E INTERNET (PACOTE) |
| 01001 | TV POR ASSINATURA E INTERNET (PACOTE) |
| 01101 | TELEFONE FIXO E CELULAR (PACOTE) |
| 01201 | TELEFONE VIRTUAL - MENSAGEM (MENSALIDADE, ASSINATURA, ALUGUEL) |
| 01202 | PAGERS (MENSALIDADE, ASSINATURA, ALUGUEL) |
| 01203 | MOBI (MENSALIDADE, ASSINATURA, ALUGUEL) |
| 01204 | BIP (MENSALIDADE, ASSINATURA, ALUGUEL) |
| 01301 | INTERNET E CELULAR (PACOTE) |
| 01401 | TELEFONE FIXO, INTERNET E TV POR ASSINATURA (PACOTE) |
| 01501 | TELEFONE FIXO E TV POR ASSINATURA (PACOTE) |
| 99901 | AGREGADO |
| 00101 | GAS DE BOTIJAO (COMBUSTIVEL DOMESTICO) |
| 00102 | GAS DE BOTIJAO (KG) (COMBUSTIVEL DOMESTICO) |
| 00103 | GAS DE BUJAO (COMBUSTIVEL DOMESTICO) |
| 00104 | GAS DE BUJAO (KG) (COMBUSTIVEL DOMESTICO) |
| 00201 | AGUA PARA USO GERAL |
| 00202 | AQUISICAO DE AGUA (USO GERAL) (LITROS) |
| 00203 | PIPA DE AGUA (LITROS) |
| 00204 | GARRAFAO DE AGUA (LITROS) |
| 00301 | RECARGA DE BATERIA (ILUMINACAO DOMICILIAR) |
| 00401 | GAS DE BUJAO PARA ILUMINACAO |
| 00402 | GAS DE BUJAO (KG) (PARA ILUMINACAO) |
| 00403 | GAS DE BOTIJAO (PARA ILUMINACAO) |
| 00501 | QUEROSENE (COMBUSTIVEL DOMESTICO) |
| 00502 | QUEROSENE (LITRO) (COMBUSTIVEL DOMESTICO) |
| 00601 | PO DE SERRA (COMBUSTIVEL DOMESTICO) |
| 00602 | PO DE SERRA (KG) (COMBUSTIVEL DOMESTICO) |
| 00701 | ALCOOL (COMBUSTIVEL DOMESTICO) |
| 00702 | ALCOOL (LITRO) (COMBUSTIVEL DOMESTICO) |
| 00801 | OLEO DIESEL (COMBUSTIVEL DOMESTICO) |
| 00802 | OLEO DIESEL (LITRO) (COMBUSTIVEL DOMESTICO) |
| 00901 | GASOLINA (COMBUSTIVEL DOMESTICO) |
| 00902 | GASOLINA (LITRO) (COMBUSTIVEL DOMESTICO) |
| 01001 | CARVAO PARA USO DOMESTICO |
| 01101 | LENHA PARA USO DOMESTICO |
| 99901 | AGREGADO |
| 00101 | AZULEJO E PISO |
| 00102 | PISO |
| 00103 | RODAPE (EXCETO DE MADEIRA) |
| 00104 | LAJOTA |
| 00105 | AZULEJO |
| 00201 | APARELHOS SANITARIOS (PIA, VASO, BIDE, BANHEIRA, ETC.) |
| 00202 | BIDE |
| 00203 | CONJUNTO SANITARIO |
| 00204 | VASO SANITARIO |
| 00205 | BANHEIRA |
| 00206 | PECA PARA SUPORTE DE PAPEL HIGIENICO |
| 00207 | SABONETEIRA (EXCETO DE PLASTICO) |
| 00208 | PIA |
| 00301 | JANELA E PORTA |
| 00302 | PORTA |
| 00303 | JANELA |
| 00401 | MADEIRA E TACO |
| 00402 | TACO |
| 00403 | CAIBRO |
| 00404 | RIPA |
| 00405 | SARRAFO |
| 00406 | MADEIRA |
| 00501 | CIMENTO |
| 00502 | CIMENTO BRANCO |
| 00503 | CIMENTCOLA OU CIMENTOCOLA |
| 00601 | TIJOLO |
| 00602 | BLOCO DE CERAMICA |
| 00701 | VIDRO |
| 00801 | TINTA |
| 00802 | VERNIZ |
| 00901 | MATERIAL DE PINTURA EM GERAL |
| 00902 | MASSA CORRIDA PARA PAREDE |
| 00903 | CAL |
| 00904 | CAL VIRGEM |
| 00905 | ROLO DE PINTURA |
| 00906 | LIXA DE MADEIRA |
| 01001 | TORNEIRA, CANO E MATERIAL HIDRAULICO EM GERAL |
| 01002 | CANO |
| 01003 | MATERIAL HIDRAULICO |
| 01101 | FERRAGENS (FECHADURA, FERRO, ARAME, PREGO, ETC.) |
| 01102 | FECHADURA |
| 01103 | FERRO |
| 01104 | ARAME |
| 01105 | PREGO |
| 01106 | CADEADO |
| 01107 | RALO DE ESGOTAMENTO DE AGUA |
| 01108 | TELA DE ARAME |
| 01109 | TRILHO METALICO |
| 01110 | ROLAMENTO PARA PORTA |
| 01201 | FIO E MATERIAL ELETRICO EM GERAL |
| 01202 | MATERIAL PARA INSTALACAO ELETRICA |
| 01203 | FIO PARA INSTALACAO ELETRICA |
| 01204 | FIO DE ANTENA |
| 01301 | MAO-DE-OBRA (PEDREIRO, MARCENEIRO, ELETRICISTA, PINTOR, ETC.) |
| 01302 | PEDREIRO |
| 01303 | MARCENEIRO |
| 01304 | ELETRICISTA |
| 01305 | PINTOR |
| 01306 | VIDRACEIRO |
| 01307 | MANUTENCAO DE CAIXA DE AGUA |
| 01308 | CONSERTO DE TOLDO |
| 01309 | CONSERTO DE PORTAO ELETRONICO |
| 01310 | MANUTENCAO DE FOSSA SEPTICA |
| 01401 | DEDETIZACAO, DESRATIZACAO, DESINSETIZACAO, ETC. |
| 01402 | DESRATIZACAO |
| 01403 | EXTERMINIO DE INSETOS |
| 01404 | DESINSETIZACAO |
| 01501 | VITRIFICACAO (SINTECO, POLIURETANO, ETC.) |
| 01502 | SINTECO |
| 01503 | POLIURETANO |
| 01601 | REFORMA OU OBRA POR EMPREITADA |
| 01602 | CONSTRUCAO POR EMPREITADA |
| 01603 | EMPREITEIRO |
| 01604 | EMPREITADA |
| 01605 | OBRA POR EMPREITADA |
| 01606 | TERRAPLANAGEM (EMPREITADA) |
| 01607 | SERVICO DE TERRAPLANAGEM (EMPREITADA) |
| 01701 | LICENCA PARA OBRA, PLANTA E DESEMBARACO DE DOCUMENTACAO |
| 01702 | PLANTA PARA OBRA |
| 01703 | DESEMBARACO DE DOCUMENTACAO PARA OBRA |
| 01704 | LICENCA PARA OBRA |
| 01801 | ARTIGOS DE JARDINAGEM (PLANTA, TERRA, SEMENTE, XAXIM, ETC.) |
| 01802 | PLANTAS NATURAIS |
| 01803 | TERRA VEGETAL |
| 01804 | SEMENTES |
| 01805 | XAXIM |
| 01806 | VASO PARA PLANTA |
| 01807 | SUPORTE DE VASO |
| 01808 | MUDAS |
| 01809 | PINHEIRO (MUDA) |
| 01901 | ALUGUEL DE CARRETO PARA MATERIAL DE CONSTRUCAO |
| 01902 | FRETE DE MATERIAL DE CONSTRUCAO |
| 01903 | ALUGUEL DE CACAMBA PARA RETIRAR ENTULHOS |
| 01904 | RETIRADA DE ENTULHO |
| 01905 | CARRETO DE MATERIAL DE CONSTRUCAO |
| 01906 | ALUGUEL DE MATERIAL DE CONSTRUCAO |
| 01907 | ALUGUEL DE MAQUINAS |
| 02001 | CAIXA DE CORREIO |
| 02002 | CAIXA DE CORRESPONDENCIA |
| 02101 | LAJE PRE-MOLDADA |
| 02201 | AREIA, TERRA, BARRO, SAIBRO, ETC. |
| 02202 | ARENOSO |
| 02203 | BARRO |
| 02204 | SAIBRO |
| 02205 | CAULIM |
| 02206 | TERRA |
| 02207 | ATERRO |
| 02208 | CASCALHO |
| 02301 | PEDRAS (BRITADA, MARROADA, PARALELEPIPEDO, ETC.) |
| 02302 | PEDRA BRITADA |
| 02303 | BRITA |
| 02304 | PO DE PEDRA |
| 02305 | PEDRA MARROADA |
| 02306 | PEDRA DE ALICERCE |
| 02307 | ASFALTO |
| 02401 | PORTAO |
| 02402 | PORTAO DE FERRO |
| 02403 | PORTAO ELETRONICO |
| 02404 | PORTAO DE MADEIRA |
| 02501 | INPS DE OBRA |
| 02601 | BASCULANTE |
| 02701 | COLA (EXCETO DUREPOX) |
| 02702 | COLA DE MADEIRA |
| 02703 | COLA SUPER BOND |
| 02704 | SUPER BOND |
| 02705 | COLA DE SAPATEIRO |
| 02801 | ARGAMASSA |
| 02802 | REJUNTE |
| 02901 | TELHAS (BARRO, AMIANTO, FIBRA, ALUMINIO, ETC.) |
| 02902 | TELHA DE BARRO |
| 02903 | TELHA DE AMIANTO |
| 02904 | TELHA DE FIBRA |
| 02905 | TELHA DE ALUMINIO |
| 03001 | SOLEIRA E PARAPEITO DE JANELA DE QUALQUER MATERIAL |
| 03002 | PARAPEITO DE JANELA DE QUALQUER MATERIAL |
| 03101 | PEDRA MARMORE PARA PIA |
| 03102 | MARMORE (PEDRA) PARA PIA |
| 03103 | BALCAO DE MARMORE PARA PIA |
| 03104 | BALCAO DE PIA |
| 03105 | BALCAO DE COZINHA |
| 03106 | GRANITO (PEDRA) PARA PIA |
| 03107 | BALCAO DE GRANITO PARA PIA |
| 03108 | BALCAO |
| 03109 | PIA DE COZINHA |
| 03201 | CAIXA DE AGUA DE QUALQUER MATERIAL |
| 03202 | TAMPA DE CAIXA DE AGUA |
| 03203 | CAIXA DE AGUA |
| 03301 | GRADE DE FERRO |
| 03401 | BLOCO DE CIMENTO |
| 03402 | BLOCKET (BLOCO DE CIMENTO) |
| 03501 | BOX DE BANHEIRO DE QUALQUER MATERIAL |
| 03601 | TANQUE DE QUALQUER MATERIAL |
| 03701 | PORTAL |
| 03801 | CAIXA DE LUZ (PADRAO) |
| 03901 | CAIXA DE DESCARGA |
| 04001 | GESSO |
| 04101 | ESPELHO DE BANHEIRO |
| 04201 | MANILHA |
| 04301 | PLACA, ANEL, VIGA, PALANQUE, ETC. DE CIMENTO OU CONCRETO |
| 04302 | ANEL DE CIMENTO OU CONCRETO |
| 04303 | VIGA DE CIMENTO OU CONCRETO |
| 04304 | PALANQUE DE CIMENTO OU CONCRETO |
| 04305 | PLACA DE CIMENTO OU CONCRETO |
| 04401 | CAIXA DE REGISTRO DE AGUA |
| 04501 | MATERIAL PLASTICO PARA COBERTURA |
| 04502 | TOLDO PLASTICO |
| 04503 | PLASTICO (MATERIAL PARA COBERTURA) |
| 04504 | TOLDO DE LONA |
| 04601 | DUREPOX |
| 04602 | EPOX (COLA) |
| 04701 | CUBA PARA PIA (QUALQUER MATERIAL) |
| 04702 | PIA (CUBA) DE QUALQUER MATERIAL |
| 04801 | CALHA |
| 04901 | ESQUADRIA |
| 05001 | FORMICA (FOLHA) |
| 05002 | FOLHA DE FORMICA |
| 05101 | POSTE |
| 05102 | POSTE DE MADEIRA |
| 05103 | POSTE DE METAL |
| 05104 | POSTE DE CIMENTO |
| 05201 | CUPINICIDA |
| 05202 | VENENO CONTRA CUPIM (CUPINICIDA) |
| 05301 | PRODUTOS PARA PISCINA |
| 05302 | CLORO PARA PISCINA |
| 05303 | SULFATO DE ALUMINIO PARA PISCINA |
| 05401 | CARPETE |
| 05501 | ESCADA CARACOL |
| 05601 | IMPERMEABILIZANTE |
| 05602 | MASSA DE VEDACAO DE TELHA |
| 05701 | PALHA PARA COBERTURA |
| 05702 | SAPE PARA COBERTURA |
| 05801 | BOIA DE CAIXA DE AGUA |
| 05901 | MASSA DE FIXACAO E VEDACAO DE VIDRO |
| 06001 | FORRO PARA COBERTURA |
| 06002 | FORRO DE GESSO |
| 06003 | FORRO DE MADEIRA |
| 06004 | FORRO DE PVC |
| 06005 | FORRO PARA COBERTURA INDETERMINADO |
| 06101 | BANDEIRA DE JANELA |
| 06201 | CONCRETO USINADO |
| 06301 | LIXEIRA METALICA |
| 06401 | TETO SOLAR |
| 06501 | LATA VAZIA (USADA EM CONSTRUCAO) |
| 06502 | BALDE PARA CONSTRUCAO |
| 06601 | RUFO DE TELHADO |
| 06602 | RUFO PARA TELHADO |
| 06701 | FIXOTAC |
| 06801 | OXIDO DE FERRO (PARA PISO DE CERAMICA) |
| 07001 | PAPEL DE PAREDE |
| 07101 | ALUGUEL DE LIXADEIRA ELETRICA |
| 07201 | MOTOR ELETRICO PARA PORTAO |
| 07301 | MANUTENCAO DE JAZIGO |
| 07401 | EXUMACAO |
| 07501 | PLACA DE JAZIGO |
| 07601 | CONDOMINIO DE JAZIGO |
| 07602 | TAXA DE CONDOMINIO DE JAZIGO |
| 07701 | MADEIRA E TACO DE MADEIRA CERTIFICADA (SELO FSC) |
| 07702 | TACO DE MADEIRA CERTIFICADA (SELO FSC) |
| 07703 | CAIBRO DE MADEIRA CERTIFICADA (SELO FSC) |
| 07704 | RIPA DE MADEIRA CERTIFICADA (SELO FSC) |
| 07705 | SARRAFO DE MADEIRA CERTIFICADA (SELO FSC) |
| 07706 | MADEIRA COM CERTIFICADO (SELO FSC) |
| 07801 | ACESSORIOS PARA BANHEIRO |
| 07901 | CAIXA PARA AR CONDICIONADO |
| 99901 | AGREGADO |
| 00101 | CONSERTO DE MOVEIS (PECA+MAO-DE-OBRA) |
| 00102 | REFORMA DE MOVEIS (PECA+MAO-DE-OBRA) |
| 00201 | CONSERTO DE MOVEIS (PECA) |
| 00202 | REFORMA DE MOVEIS (PECA) |
| 00301 | CONSERTO DE MOVEIS (MAO-DE-OBRA) |
| 00302 | REFORMA DE MOVEIS (MAO-DE-OBRA) |
| 00401 | MANUTENCAO DE MOVEIS |
| 00501 | CONSERTO DE ASPIRADOR DE PO (PECA+MAO-DE-OBRA) |
| 00601 | CONSERTO DE ASPIRADOR DE PO (PECA) |
| 00701 | CONSERTO DE ASPIRADOR DE PO (MAO-DE-OBRA) |
| 00801 | MANUTENCAO DE APARELHOS DOMESTICOS |
| 00901 | CONSERTO DE GRILL (PECA+MAO-DE-OBRA) |
| 01001 | CONSERTO DE GRILL (PECA) |
| 01101 | CONSERTO DE GRILL (MAO-DE-OBRA) |
| 01201 | MANUTENCAO DE UTENSILIOS DOMESTICOS NAO-ELETRICOS |
| 01301 | MANUTENCAO DE UTENSILIOS DOMESTICOS ELETRICOS |
| 01401 | CONSERTO DE FERRO ELETRICO (PECA+MAO-DE-OBRA) |
| 01501 | CONSERTO DE FERRO ELETRICO (PECA) |
| 01601 | CONSERTO DE FERRO ELETRICO (MAO-DE-OBRA) |
| 01701 | CONSERTO DE ANTENA PARABOLICA (PECA+MAO-DE-OBRA) |
| 01801 | CONSERTO DE ANTENA PARABOLICA (PECA) |
| 01901 | CONSERTO DE ANTENA PARABOLICA (MAO-DE-OBRA) |
| 02001 | CONSERTO DE TELEFONE (PECA+MAO-DE-OBRA) |
| 02101 | CONSERTO DE TELEFONE (PECA) |
| 02201 | CONSERTO DE TELEFONE (MAO-DE-OBRA) |
| 02301 | CONSERTO DE PERSIANA (PECA+MAO-DE-OBRA) |
| 02401 | CONSERTO DE PERSIANA (PECA) |
| 02501 | CONSERTO DE PERSIANA (MAO-DE-OBRA) |
| 02601 | CONSERTO DE RECEPTOR DE ANTENA PARABOLICA (PECA+MAO-DE-OBRA) |
| 02701 | CONSERTO DE RECEPTOR DE ANTENA PARABOLICA (PECA) |
| 02801 | CONSERTO DE RECEPTOR DE ANTENA PARABOLICA (MAO-DE-OBRA) |
| 02901 | MONTAGEM DE MOVEIS |
| 03001 | CONSERTO DE GELADEIRA (PECA+MAO-DE-OBRA) |
| 03101 | CONSERTO DE GELADEIRA (PECA) |
| 03201 | CONSERTO DE GELADEIRA (MAO-DE-OBRA) |
| 03301 | CONSERTO DE FREEZER (PECA+MAO DE OBRA) |
| 03401 | CONSERTO DE FREEZER (PECA) |
| 03501 | CONSERTO DE FREEZER (MAO DE OBRA) |
| 03601 | CONSERTO DE TELEVISAO (PECA+MAO-DE-OBRA) |
| 03701 | CONSERTO DE TELEVISAO (PECA) |
| 03801 | CONSERTO DE TELEVISAO (MAO-DE-OBRA) |
| 03901 | CONSERTO DE TELAO (PECA+MAO-DE-OBRA) |
| 04001 | CONSERTO DE TELAO (PECA) |
| 04101 | CONSERTO DE TELAO (MAO-DE-OBRA) |
| 04201 | CONSERTO DE APARELHO SONORO (PECA+MAO-DE-OBRA) |
| 04301 | CONSERTO DE APARELHO SONORO (PECA) |
| 04401 | CONSERTO DE APARELHO SONORO (MAO-DE-OBRA) |
| 04501 | CONSERTO DE RADIO (PECA+MAO-DE-OBRA) |
| 04601 | CONSERTO DE RADIO (PECA) |
| 04701 | CONSERTO DE RADIO (MAO+DE+OBRA) |
| 04801 | CONSERTO DE RADIO-RELOGIO (PECA+MAO-DE-OBRA) |
| 04901 | CONSERTO DE RADIO-RELOGIO (PECA) |
| 05001 | CONSERTO DE RADIO-RELOGIO (MAO-DEOBRA) |
| 05101 | CONSERTO DE VIDEOCASSETE (PECA+MAO-DE-OBRA) |
| 05201 | CONSERTO DE VIDEOCASSETE (PECA) |
| 05301 | CONSERTO DE VIDEOCASSETE (MAO-DE-OBRA) |
| 05401 | CONSERTO DE MAQUINA DE LAVAR ROUPA (PECA+MAO-DE-OBRA) |
| 05501 | CONSERTO DE MAQUINA DE LAVAR ROUPA (PECA) |
| 05601 | CONSERTO DE MAQUINA DE LAVAR ROUPA (MAO-DE-OBRA) |
| 05701 | CONSERTO DE MAQUINA DE SECAR ROUPA (PECA+MAO-DE-OBRA) |
| 05801 | CONSERTO DE MAQUINA DE SECAR ROUPA (PECA) |
| 05901 | CONSERTO DE MAQUINA DE SECAR ROUPA (MAO-DE-OBRA) |
| 06001 | CONSERTO DE FOGAO (PECA+MAO-DE-OBRA) |
| 06101 | CONSERTO DE FOGAO (PECA) |
| 06201 | CONSERTO DE FOGAO (MAO-DE-OBRA) |
| 06301 | CONSERTO DE AR CONDICIONADO (PECA+MAO-DE-OBRA) |
| 06401 | CONSERTO DE AR CONDICIONADO (PECA) |
| 06501 | CONSERTO DE AR CONDICIONADO (MAO-DE-OBRA) |
| 06601 | CONSERTO DE FURADEIRA ELETRICA (PECA+MAO-DE-OBRA) |
| 06701 | CONSERTO DE FURADEIRA ELETRICA (PECA) |
| 06801 | CONSERTO DE FURADEIRA ELETRICA (MAO-DE-OBRA) |
| 06901 | CONSERTO DE ORGAO ELETRONICO (PECA+MAO-DE-OBRA) |
| 07001 | CONSERTO DE ORGAO ELETRONICO (PECA) |
| 07101 | CONSERTO DE ORGAO ELETRONICO (MAO-DE-OBRA) |
| 07201 | CONSERTO DE SECRETARIA ELETRONICA (PECA+MAO-DE-OBRA) |
| 07301 | CONSERTO DE SECRETARIA ELETRONICA (PECA) |
| 07401 | CONSERTO DE SECRETARIA ELETRONICA (MAO-DE-OBRA) |
| 07501 | CONSERTO DE MAQUINA DE COSTURA (PECA+MAO-DE-OBRA) |
| 07601 | CONSERTO DE MAQUINA DE COSTURA (PECA) |
| 07701 | CONSERTO DE MAQUINA DE COSTURA (MAO-DE-OBRA) |
| 07801 | CONSERTO DE MAQUINA DE OVERLOCK (PECA+MAO-DE-OBRA) |
| 07901 | CONSERTO DE MAQUINA DE OVERLOCK (PECA) |
| 08001 | CONSERTO DE MAQUINA DE OVERLOCK (MAO-DE-OBRA) |
| 08101 | CONSERTO DE BATEDEIRA DE BOLO (PECA+MAO-DE-OBRA) |
| 08201 | CONSERTO DE BATEDEIRA DE BOLO (PECA) |
| 08301 | CONSERTO DE BATEDEIRA DE BOLO (MAO-DE-OBRA) |
| 08401 | CONSERTO DE LIQUIDIFICADOR (PECA+MAO-DE-OBRA) |
| 08501 | CONSERTO DE LIQUIDIFICADOR (PECA) |
| 08601 | CONSERTO DE LIQUIDIFICADOR (MAO-DE-OBRA) |
| 08701 | CONSERTO DE MULTIPROCESSADOR (PECA+MAO-DE-OBRA) |
| 08801 | CONSERTO DE MULTIPROCESSADOR (PECA) |
| 08901 | CONSERTO DE MULTIPROCESSADOR (MAO-DE-OBRA) |
| 09001 | CONSERTO DE FORNO ELETRICO (PECA+MAO-DE-OBRA) |
| 09101 | CONSERTO DE FORNO ELETRICO (PECA) |
| 09201 | CONSERTO DE FORNO ELETRICO (MAO-DE-OBRA) |
| 09301 | CONSERTO DE MAQUINA DE LAVAR LOUCAS (PECA+MAO-DE-OBRA) |
| 09401 | CONSERTO DE MAQUINA DE LAVAR LOUCAS (PECA) |
| 09501 | CONSERTO DE MAQUINA DE LAVAR LOUCAS (MAO-DE-OBRA) |
| 09601 | CONSERTO DE RELOGIO DE MESA (PECA+MAO-DE-OBRA) |
| 09701 | CONSERTO DE RELOGIO DE MESA (PECA) |
| 09801 | CONSERTO DE RELOGIO DE MESA (MAO-DE-OBRA) |
| 09901 | CONSERTO DE RELOGIO DE PAREDE (PECA+MAO-DE-OBRA) |
| 10001 | CONSERTO DE RELOGIO DE PAREDE (PECA) |
| 10101 | CONSERTO DE RELOGIO DE PAREDE (MAO-DE-OBRA) |
| 10201 | CONSERTO DE VENTILADOR (PECA+MAO-DE-OBRA) |
| 10301 | CONSERTO DE VENTILADOR (PECA) |
| 10401 | CONSERTO DE VENTILADOR (MAO-DE-OBRA) |
| 10501 | CONSERTO DE OZONIZADOR (PECA+MAO-DE-OBRA) |
| 10601 | CONSERTO DE OZONIZADOR (PECA) |
| 10701 | CONSERTO DE OZONIZADOR (MAO-DE-OBRA) |
| 10801 | CONSERTO DE TORNEIRA ELETRICA (PECA+MAO-DE-OBRA) |
| 10901 | CONSERTO DE TORNEIRA ELETRICA (PECA) |
| 11001 | CONSERTO DE TORNEIRA ELETRICA (MAO-DE-OBRA) |
| 11101 | CONSERTO DE ENCERADEIRA (PECA+MAO-DE-OBRA) |
| 11201 | CONSERTO DE ENCERADEIRA (PECA) |
| 11301 | CONSERTO DE ENCERADEIRA (MAO-DE-OBRA) |
| 11401 | CONSERTO DE MICROCOMPUTADOR (PECA+MAO-DE-OBRA) |
| 11501 | CONSERTO DE MICROCOMPUTADOR (PECA) |
| 11601 | CONSERTO DE MICROCOMPUTADOR (MAO-DE-OBRA) |
| 11701 | CONSERTO DE CHUVEIRO ELETRICO (PECA+MAO-DE-OBRA) |
| 11801 | CONSERTO DE CHUVEIRO ELETRICO (PECA) |
| 11901 | CONSERTO DE CHUVEIRO ELETRICO (MAO-DE-OBRA) |
| 12001 | CONSERTO DE SECADOR DE CABELOS (PECA+MAO-DE-OBRA) |
| 12101 | CONSERTO DE SECADOR DE CABELOS (PECA) |
| 12201 | CONSERTO DE SECADOR DE CABELOS (MAO-DE-OBRA) |
| 12301 | CONSERTO DE MICROONDAS (PECA+MAO-DE-OBRA) |
| 12401 | CONSERTO DE MICROONDAS (PECA) |
| 12501 | CONSERTO DE MICROONDAS (MAO-DE-OBRA) |
| 12601 | CONSERTO DE VIDEOGAME (PECA+MAO-DE-OBRA) |
| 12701 | CONSERTO DE VIDEOGAME (PECA) |
| 12801 | CONSERTO DE VIDEOGAME (MAO-DE-OBRA) |
| 12901 | CONSERTO DE CENTRIFUGA (PECA+MAO-DE-OBRA) |
| 13001 | CONSERTO DE CENTRIFUGA (PECA) |
| 13101 | CONSERTO DE CENTRIFUGA (MAO-DE-OBRA) |
| 13201 | CONSERTO DE IMPRESSORA (PECA+MAO-DE-OBRA) |
| 13301 | CONSERTO DE IMPRESSORA (PECA) |
| 13401 | CONSERTO DE IMPRESSORA (MAO-DE-OBRA) |
| 13501 | CONSERTO DE CONTROLE REMOTO (PECA+MAO-DE-OBRA) |
| 13601 | CONSERTO DE CONTROLE REMOTO (PECA) |
| 13701 | CONSERTO DE CONTROLE REMOTO (MAO-DE-OBRA) |
| 13801 | CONSERTO DE FAX (PECA+MAO-DE-OBRA) |
| 13901 | CONSERTO DE FAX (PECA) |
| 14001 | CONSERTO DE FAX (MAO-DE-OBRA) |
| 14101 | CONSERTO DE DVD (PECA+MAO-DE-OBRA) |
| 14201 | CONSERTO DE DVD (PECA) |
| 14301 | CONSERTO DE DVD (MAO-DE-OBRA) |
| 14401 | REFORMA DE INSTRUMENTO MUSICAL (PECA+MAO-DE-OBRA) |
| 14501 | REFORMA DE INSTRUMENTO MUSICAL (PECA) |
| 14601 | REFORMA DE INSTRUMENTO MUSICAL (MAO-DE-OBRA) |
| 14701 | CONSERTO DE INTERFONE (PECA+MAO-DE-OBRA) |
| 14901 | CONSERTO DO INTERFONE (MAO-DE-OBRA) |
| 15001 | CONSERTO DE APARELHO ELETRONICO (PECA + MAO-DE-OBRA) |
| 15601 | CONSERTO DE BOMBA DE AGUA ELETRICA (PECA+MAO-DE-OBRA) |
| 15701 | CONSERTO DE BOMBA DE AGUA ELETRICA (PECA) |
| 15801 | CONSERTO DE BOMBA DE AGUA ELETRICA (MAO-DE-OBRA) |
| 15901 | CONSERTO DE BEBEDOURO ELETRICO (PECA+MAO-DE-OBRA) |
| 16001 | CONSERTO DE BEBEDOURO ELETRICO (PECA) |
| 16101 | CONSERTO DE BEBEDOURO ELETRICO (MAO DE OBRA) |
| 16201 | CONSERTO DE CAFETEIRA (PECA+MAO-DE-OBRA) |
| 99901 | AGREGADO |
| 00101 | ALUGUEL DO IMOVEL |
| 00201 | PRESTACAO DO IMOVEL |
| 00301 | ALUGUEL DE GARAGEM |
| 00401 | CONDOMINIO |
| 00501 | IMPOSTO PREDIAL E TERRITORIAL URBANO (IPTU) |
| 00502 | IPTU |
| 00601 | ADICIONAIS DO ALUGUEL DO IMOVEL (JUROS, MULTA, ETC.) |
| 00602 | JUROS DO ALUGUEL DO IMOVEL |
| 00603 | MULTA DO ALUGUEL DO IMOVEL |
| 00701 | ADICIONAIS DE PRESTACAO DO IMOVEL (JUROS, MULTA, ETC.) |
| 00702 | JUROS DE PRESTACAO DO IMOVEL |
| 00703 | MULTA DE PRESTACAO DO IMOVEL |
| 00801 | ADICIONAIS DE ALUGUEL DE GARAGEM (JUROS, MULTA, ETC.) |
| 00802 | JUROS DE ALUGUEL DE GARAGEM |
| 00803 | MULTA DE ALUGUEL DE GARAGEM |
| 00901 | ADICIONAIS DE CONDOMINIO (JUROS, MULTA, ETC.) |
| 00902 | JUROS DE CONDOMINIO |
| 00903 | MULTA DE CONDOMINIO |
| 01001 | ADICIONAIS DE IMPOSTO PREDIAL (JUROS, MULTA, ETC.) |
| 01002 | JUROS DE IMPOSTO PREDIAL |
| 01003 | MULTA DE IMPOSTO PREDIAL |
| 01101 | IMPOSTO SOBRE PROPRIEDADE TERRITORIAL RURAL (IPTR) |
| 01102 | IPTR |
| 01201 | SERVICO DE PATRIMONIO DA UNIAO (SPU) |
| 01301 | INCRA |
| 01401 | ADICIONAIS DE IPTR |
| 09001 | ALUGUEL ESTIMADO |
| 00101 | AZULEJO E PISO |
| 00102 | PISO |
| 00103 | RODAPE (EXCETO DE MADEIRA) |
| 00104 | AZULEJO |
| 00105 | LAJOTA |
| 00106 | CERAMICA |
| 00201 | APARELHOS SANITARIOS (PIA, VASO, BIDE, BANHEIRA, ETC.) |
| 00202 | BIDE |
| 00203 | CONJUNTO SANITARIO |
| 00204 | VASO SANITARIO |
| 00205 | BANHEIRA |
| 00206 | PECA PARA SUPORTE DE PAPEL HIGIENICO |
| 00207 | SABONETEIRA (EXCETO DE PLASTICO) |
| 00208 | PIA |
| 00301 | JANELA E PORTA |
| 00302 | PORTA |
| 00304 | JANELA |
| 00401 | MADEIRA E TACO |
| 00402 | TACO |
| 00403 | CAIBRO |
| 00404 | RIPA |
| 00405 | SARRAFO |
| 00406 | MADEIRA |
| 00407 | ALIZAR |
| 00501 | CIMENTO |
| 00502 | CIMENTO BRANCO |
| 00503 | CIMENTCOLA OU CIMENTOCOLA |
| 00601 | TIJOLO |
| 00602 | BLOCO DE CERAMICA |
| 00701 | VIDRO |
| 00801 | TINTA |
| 00802 | VERNIZ |
| 00901 | MATERIAL DE PINTURA EM GERAL |
| 00902 | MASSA CORRIDA PARA PAREDE |
| 00903 | CAL |
| 00904 | CAL VIRGEM |
| 00905 | ROLO DE PINTURA |
| 00906 | LIXA DE MADEIRA |
| 00907 | LIXA DE FERRO |
| 01001 | TORNEIRA, CANO E MATERIAL HIDRAULICO EM GERAL |
| 01002 | CANO |
| 01003 | MATERIAL HIDRAULICO |
| 01101 | FERRAGENS (FECHADURA, FERRO, ARAME, PREGO, ETC.) |
| 01102 | FECHADURA |
| 01103 | FERRO |
| 01104 | ARAME |
| 01105 | PREGO |
| 01106 | CADEADO |
| 01107 | RALO DE ESGOTAMENTO DE AGUA |
| 01108 | TELA DE ARAME |
| 01109 | TRILHO METALICO |
| 01110 | ROLAMENTO PARA PORTA |
| 01201 | FIO E MATERIAL ELETRICO EM GERAL |
| 01202 | MATERIAL PARA INSTALACAO ELETRICA |
| 01203 | FIO PARA INSTALACAO ELETRICA |
| 01301 | MAO-DE-OBRA (PEDREIRO, MARCENEIRO, ELETRICISTA, PINTOR, ETC.) |
| 01302 | PEDREIRO |
| 01303 | MARCENEIRO |
| 01304 | ELETRICISTA |
| 01305 | PINTOR |
| 01306 | VIDRACEIRO |
| 01401 | DEDETIZACAO, DESRATIZACAO, DESINSETIZACAO, ETC. |
| 01402 | DESRATIZACAO |
| 01403 | EXTERMINIO DE INSETOS |
| 01404 | DESINSETIZACAO |
| 01501 | VITRIFICACAO (SINTECO, POLIURETANO, ETC.) |
| 01502 | SINTECO |
| 01503 | POLIURETANO |
| 01601 | REFORMA OU OBRA POR EMPREITADA |
| 01602 | CONSTRUCAO POR EMPREITADA |
| 01603 | EMPREITEIRO |
| 01604 | EMPREITADA |
| 01605 | OBRA POR EMPREITADA |
| 01606 | TERRAPLANAGEM (EMPREITADA) |
| 01607 | SERVICO DE TERRAPLANAGEM (EMPREITADA) |
| 01701 | LICENCA PARA OBRA, PLANTA E DESEMBARACO DE DOCUMENTACAO |
| 01702 | PLANTA PARA OBRA |
| 01703 | DESEMBARACO DE DOCUMENTACAO PARA OBRA |
| 01704 | LICENCA PARA OBRA |
| 01801 | BOIA DE CAIXA DE AGUA |
| 01901 | ALUGUEL DE CARRETO PARA MATERIAL DE CONSTRUCAO |
| 01902 | FRETE DE MATERIAL DE CONSTRUCAO |
| 01903 | ALUGUEL DE CACAMBA PARA RETIRAR ENTULHOS |
| 01904 | RETIRADA DE ENTULHO |
| 01905 | CARRETO DE MATERIAL DE CONSTRUCAO |
| 01906 | ALUGUEL DE MATERIAL DE CONSTRUCAO |
| 02001 | CAIXA DE CORREIO |
| 02002 | CAIXA DE CORRESPONDENCIA |
| 02003 | CAIXA DE AR CONDICIONADO |
| 02101 | LAJE PRE-MOLDADA |
| 02201 | AREIA, TERRA, BARRO, SAIBRO, ETC. |
| 02202 | ARENOSO |
| 02203 | BARRO |
| 02204 | SAIBRO |
| 02205 | CAULIM |
| 02206 | TERRA |
| 02207 | ATERRO |
| 02208 | CASCALHO |
| 02301 | PEDRAS (BRITADA, MARROADA, PARALELEPIPEDO, ETC.) |
| 02302 | PEDRA BRITADA |
| 02303 | BRITA |
| 02304 | PO DE PEDRA |
| 02305 | PEDRA MARROADA |
| 02306 | PEDRA DE ALICERCE |
| 02307 | ASFALTO |
| 02401 | PORTAO |
| 02402 | PORTAO DE FERRO |
| 02403 | PORTAO ELETRICO |
| 02501 | INSS DE OBRA |
| 02502 | INPS DE OBRA |
| 02601 | BASCULANTE |
| 02701 | COLA (EXCETO DUREPOX) |
| 02702 | COLA DE MADEIRA |
| 02703 | COLA SUPER BOND |
| 02704 | SUPER BOND |
| 02705 | COLA DE SAPATEIRO |
| 02801 | ARGAMASSA |
| 02802 | REJUNTE |
| 02901 | TELHAS (BARRO, AMIANTO, FIBRA, ALUMINIO, ETC.) |
| 02902 | TELHA DE BARRO |
| 02903 | TELHA DE AMIANTO |
| 02904 | TELHA DE FIBRA |
| 02905 | TELHA DE ALUMINIO |
| 03001 | SOLEIRA E PARAPEITO DE JANELA DE QUALQUER MATERIAL |
| 03002 | PARAPEITO DE JANELA DE QUALQUER MATERIAL |
| 03101 | PEDRA MARMORE PARA PIA |
| 03102 | MARMORE (PEDRA) PARA PIA |
| 03103 | BALCAO DE MARMORE PARA PIA |
| 03104 | BALCAO DE PIA |
| 03105 | BALCAO DE COZINHA |
| 03106 | GRANITO (PEDRA) PARA PIA |
| 03107 | BALCAO DE GRANITO PARA PIA |
| 03108 | GRANITO |
| 03201 | CAIXA DE AGUA DE QUALQUER MATERIAL |
| 03202 | TAMPA DE CAIXA DE AGUA |
| 03203 | CAIXA DE AGUA |
| 03301 | GRADE DE FERRO |
| 03401 | BLOCO DE CIMENTO |
| 03402 | BLOCKET (BLOCO DE CIMENTO) |
| 03501 | BOX DE BANHEIRO DE QUALQUER MATERIAL |
| 03601 | TANQUE DE QUALQUER MATERIAL |
| 03701 | PORTAL |
| 03801 | CAIXA DE LUZ (PADRAO) |
| 03901 | CAIXA DE DESCARGA |
| 04001 | GESSO |
| 04101 | ESPELHO DE BANHEIRO |
| 04201 | MANILHA |
| 04301 | PLACA, ANEL, VIGA, PALANQUE, ETC. DE CIMENTO OU CONCRETO |
| 04302 | ANEL DE CIMENTO OU CONCRETO |
| 04303 | VIGA DE CIMENTO OU CONCRETO |
| 04304 | PALANQUE DE CIMENTO OU CONCRETO |
| 04305 | PLACA DE CIMENTO OU CONCRETO |
| 04401 | CAIXA DE REGISTRO DE AGUA |
| 04501 | MATERIAL PLASTICO PARA COBERTURA |
| 04502 | TOLDO PLASTICO |
| 04503 | PLASTICO (MATERIAL PARA COBERTURA) |
| 04504 | TOLDO DE LONA |
| 04505 | LONA |
| 04601 | DUREPOX |
| 04602 | EPOX (COLA) |
| 04701 | CUBA PARA PIA (QUALQUER MATERIAL) |
| 04702 | PIA (CUBA) DE QUALQUER MATERIAL |
| 04801 | CALHA |
| 04901 | ESQUADRIA |
| 05001 | FORMICA (FOLHA) |
| 05002 | FOLHA DE FORMICA |
| 05101 | POSTE |
| 05102 | POSTE DE MADEIRA |
| 05103 | POSTE METALICO |
| 05104 | POSTE DE CIMENTO |
| 05201 | CUPINICIDA |
| 05202 | VENENO CONTRA CUPIM (CUPINICIDA) |
| 05301 | PRODUTOS PARA PISCINA |
| 05302 | CLORO PARA PISCINA |
| 05303 | SULFATO DE ALUMINIO PARA PISCINA |
| 05401 | PAPEL DE PAREDE |
| 05501 | ESCADA CARACOL |
| 05601 | IMPERMEABILIZANTE |
| 05602 | MASSA DE VEDACAO DE TELHA |
| 05701 | PALHA PARA COBERTURA |
| 05702 | SAPE PARA COBERTURA |
| 05801 | ALUGUEL DE TERRENO PARA SEPULTURA |
| 05901 | MASSA DE FIXACAO E VEDACAO DE VIDRO |
| 06001 | CASA DE MADEIRA USADA |
| 06101 | BANDEIRA DE JANELA |
| 06201 | CONCRETO USINADO |
| 06301 | CIPO DE AMARRAR |
| 06401 | TETO SOLAR |
| 06501 | ALUGUEL DE MAQUINAS |
| 06601 | FOSSA SEPTICA |
| 06701 | FIXOTAC |
| 06901 | LATA VAZIA (USADA EM CONSTRUCAO) |
| 06902 | BALDE PARA CONSTRUCAO |
| 07001 | RUFO DE TELHADO |
| 07002 | RUFO PARA TELHADO |
| 07101 | OXIDO DE FERRO (PARA PISO DE CERAMICA) |
| 07201 | ESTACA PARA CERCA (QUALQUER MATERIAL) |
| 07301 | LIXEIRA METALICA |
| 07401 | MASSA PARA REJUNTE |
| 07501 | CHAPA METALICA |
| 07601 | ACESSORIOS PARA BANHEIRO |
| 07701 | FORRO PARA COBERTURA |
| 07702 | FORRO DE MADEIRA |
| 07703 | FORRO DE GESSO |
| 07704 | FORRO DE PVC |
| 07705 | FORRO PARA COBERTURA INDETERMINADO |
| 07801 | ALUGUEL DE LIXADEIRA ELETRICA |
| 07901 | MOTOR ELETRICO PARA PORTAO |
| 08001 | CONSTRUCAO DE JAZIGO |
| 08101 | REFORMA DE JAZIGO |
| 08201 | LAPIDE DE JAZIGO |
| 08202 | LAPIDE |
| 08301 | GRAVACAO EM JAZIGO |
| 08401 | GAVETA DE JAZIGO |
| 08501 | AQUISICAO DE SEPULTURA |
| 08502 | AQUISICAO DE TERRENO PARA SEPULTURA |
| 08601 | MADEIRA E TACO DE MADEIRA CERTIFICADA (SELO FSC) |
| 08602 | TACO DE MADEIRA CERTIFICADA (SELO FSC) |
| 08603 | CAIBRO DE MADEIRA CERTIFICADA (SELO FSC) |
| 08604 | RIPA DE MADEIRA CERTIFICADA (SELO FSC) |
| 08605 | SARRAFO DE MADEIRA CERTIFICADA (SELO FSC) |
| 08606 | MADEIRA COM CERTIFICADO (SELO FSC) |
| 08701 | CARPETE |
| 08801 | COMBOGO (CERAMICA VAZADA) |
| 08901 | LAREIRA |
| 99901 | AGREGADO |
| 00101 | AQUISICAO DO IMOVEL A VISTA (VALOR PAGO, IMPOSTO DE TRANSMISSAO, ETC.) |
| 00102 | IMPOSTO DE TRANSMISSAO DO IMOVEL (AQUISICAO A VISTA) |
| 00103 | AQUISICAO DO IMOVEL A VISTA (VALOR PAGO) |
| 00201 | AQUISICAO DO IMOVEL A PRAZO (ENTRADA, PARCELAS, TAXAS, IMPOSTOS, ETC.) |
| 00202 | TAXAS PARA AQUISICAO DO IMOVEL A PRAZO |
| 00203 | IMPOSTOS PARA AQUISICAO DO IMOVEL A PRAZO |
| 00204 | PARCELAS PARA AQUISICAO DO IMOVEL A PRAZO |
| 00205 | ENTRADA PARA AQUISICAO DO IMOVEL A PRAZO |
| 00301 | LOCACAO DO IMOVEL (CONTRATO, DEPOSITOS DE LOCACAO E CONSERVACAO, ETC.) |
| 00302 | CONTRATO DE LOCACAO DO IMOVEL |
| 00303 | DEPOSITOS DE LOCACAO DO IMOVEL |
| 00304 | DEPOSITOS DE CONSERVACAO DO IMOVEL |
| 00305 | SEGURO FIADOR |
| 00401 | MUDANCA |
| 00501 | SEGUROS SOBRE O IMOVEL (INCENDIO, ROUBO, ETC.) |
| 00502 | SEGURO CONTRA ROUBO DO IMOVEL |
| 00503 | SEGURO CONTRA INCENDIO DO IMOVEL |
| 00601 | TAXAS (COLETA DE LIXO, PREVENCAO E EXTINCAO DE INCENDIO, SEGURANCA, ETC.) |
| 00602 | TAXA DE COLETA DE LIXO |
| 00603 | TAXA DE PREVENCAO E EXTINCAO DE INCENDIO |
| 00604 | TAXA DE SEGURANCA |
| 00605 | TAXA DE ILUMINACAO PUBLICA |
| 00606 | TAXA DE CONSERVACAO URBANA |
| 00607 | TAXA DE LIXO |
| 00608 | TAXA DE ASFALTAMENTO |
| 00609 | TAXA DE INSTALACAO DE ESGOTO |
| 00610 | TAXA DE TRATAMENTO DE ESGOTO |
| 00701 | TAXA DE OCUPACAO DO IMOVEL |
| 00801 | TAXA DE INSTALACAO DE RELOGIO DE AGUA |
| 00802 | TAXA DE INSTALACAO DE HIDROMETRO |
| 00803 | TAXA DE RELIGACAO DE RELOGIO DE AGUA |
| 00804 | MANUTENCAO DE HIDROMETRO |
| 00901 | TAXA DE TRANSFERENCIA DE TELEFONE |
| 01001 | TAXA DE INSTALACAO DE INTERFONE,TELEFONE |
| 01002 | TAXA DE INSTALACAO DE INTERFONE |
| 01003 | TAXA DE INSTALACAO DE TELEFONE |
| 01101 | TAXA DE MANUTENCAO DE ELEVADOR |
| 01201 | TAXA DE LIGACAO DE LUZ |
| 01202 | TAXA DE RELIGACAO DE LUZ |
| 01301 | TAXA DE INSTALACAO DE POSTE DE ENERGIA ELETRICA |
| 01401 | TAXA DE ISENCAO DE IMPOSTO PREDIAL |
| 01501 | TAXA DE VERIFICACAO DE VAZAMENTO DE AGUA |
| 01601 | TAXA DE INSTALACAO DE CENTRAL DE GAS |
| 01602 | TAXA DE INSTALACAO DE MEDIDOR DE GAS |
| 01701 | TAXA DE UTILIZACAO E MANUTENCAO DE BOMBA DE AGUA |
| 01801 | TAXA DE MANUTENCAO DE LINHA TELEFONICA COMUNITARIA |
| 01901 | TAXA DE CERTIDAO DE IMPOSTO (DIVIDA ATIVA) |
| 02001 | TAXA DE INSTALACAO DE TELEVISAO POR ASSINATURA |
| 02002 | SERVICO DE INSTALACAO DE TV A CABO |
| 02101 | TAXA DE ADESAO DE TELEVISAO POR ASSINATURA |
| 02201 | TAXA DE INSTALACAO DE INTERNET |
| 02301 | TAXA EXTRA DE CONDOMINIO (REFORMA E MANUTENCAO) |
| 02302 | TAXA DE REFORMA DE PREDIO (EXTRA DE CONDOMINIO) |
| 02303 | TAXA DE MANUTENCAO DE PREDIO (EXTRA DE CONDOMINIO) |
| 02304 | TAXA EXTRA DE FUNDO DE RESERVA (CONDOMINIO) |
| 02401 | CERTIFICADO DE CADASTRO DE IMOVEL |
| 02501 | VIGILANCIA ELETRONICA |
| 02601 | VALOR DO IMOVEL ADQUIRIDO EM PRIMEIRA LOCACAO A VISTA |
| 02701 | VALOR DO IMOVEL ADQUIRIDO EM PRIMEIRA LOCACAO A PRAZO |
| 02801 | VALOR DO IMOVEL ADQUIRIDO USADO A VISTA |
| 02901 | VALOR DO IMOVEL ADQUIRIDO USADO A PRAZO |
| 03001 | TAXA DE RELIGACAO DE TELEFONE |
| 03101 | PARCELAMENTO DE DIVIDA DE CONTA DE AGUA |
| 03201 | PARCELAMENTO DE DIVIDA DE ENERGIA ELETRICA |
| 03301 | TAXA DE CAIXA POSTAL DE CORREIO |
| 03401 | MULTA DE VIOLACAO DE AGUA E ENERGIA ELETRICA |
| 03501 | SEGURO CONTRA DANOS DE ELETRODOMESTICOS DA COMPANHIA ELETRICA |
| 99901 | AGREGADO |
| 00101 | AR CONDICIONADO (ALUGUEL) |
| 00201 | TELEFONE RESIDENCIAL (ALUGUEL) |
| 00301 | TELEVISAO (ALUGUEL) |
| 00401 | VIDEOCASSETE (ALUGUEL) |
| 00501 | FREEZER (ALUGUEL) |
| 00601 | MAQUINA DE COSTURA (ALUGUEL) |
| 00701 | PIANO (ALUGUEL) |
| 00801 | BUJAO DE GAS (ALUGUEL) |
| 00802 | BOTIJAO DE GAS (ALUGUEL) |
| 00901 | SOM (ALUGUEL) |
| 01001 | MICROCOMPUTADOR (ALUGUEL) |
| 01101 | FILMADORA (ALUGUEL) |
| 01201 | TELAO (ALUGUEL) |
| 01301 | VIDEOGAME (ALUGUEL) |
| 01401 | KARAOKE OU VIDEOKE (ALUGUEL) |
| 01501 | MAQUINA DE ESCREVER ELETRICA (ALUGUEL) |
| 01601 | CORTADOR DE GRAMA (ALUGUEL) |
| 01701 | DVD (ALUGUEL) |
| 01801 | GELADEIRA (ALUGUEL) |
| 01901 | BATERIA (ALUGUEL) |
| 02001 | JOGO DE LUZ (ALUGUEL) |
| 02101 | FILTRO DE AGUA ELETRICO (ALUGUEL) |
| 02201 | MAQUINA DE LAVAR PEDRAS (ALUGUEL) |
| 99901 | AGREGADO |
| 00101 | FOGAO |
| 00201 | FREEZER |
| 00301 | GELADEIRA DE 1 PORTA |
| 00401 | GELADEIRA DE 2 PORTAS |
| 00501 | CHUVEIRO ELETRICO |
| 00601 | LIQUIDIFICADOR |
| 00701 | PROCESSADOR DE ALIMENTOS |
| 00801 | GRILL |
| 00901 | ASPIRADOR DE PO |
| 01001 | FORNO ELETRICO |
| 01101 | FERRO ELETRICO |
| 01201 | MAQUINA DE LAVAR ROUPAS |
| 01301 | TELEVISAO EM CORES |
| 01401 | TELEVISAO EM PRETO E BRANCO |
| 01501 | EQUIPAMENTO DE SOM |
| 01601 | RADIO |
| 01701 | AR-CONDICIONADO |
| 01801 | VENTILADOR E/OU CIRCULADOR DE AR |
| 01901 | MAQUINA DE COSTURA |
| 02001 | FILTRO DE AGUA |
| 02101 | AUTOMOVEL |
| 02201 | BICICLETA |
| 02301 | MOTOCICLETA |
| 02401 | MICROCOMPUTADOR |
| 02501 | PURIFICADOR DE AGUA |
| 02601 | FORNO DE MICROONDAS |
| 02701 | ANTENA PARABOLICA |
| 02801 | APARELHO DE DVD |
| 02901 | MAQUINA DE SECAR ROUPAS |
| 03001 | BATEDEIRA DE BOLO |
| 03101 | SECADOR DE CABELOS |
| 03201 | MAQUINA DE LAVAR LOUCAS |
| 00101 | EXAUSTOR |
| 00102 | PURIFICADOR DE AR |
| 00201 | FOGAO ELETRICO |
| 00301 | FOGAO A LENHA |
| 00401 | FORNO ELETRICO |
| 00501 | FREEZER |
| 00601 | GELADEIRA |
| 00602 | FRIGOBAR |
| 00701 | MAQUINA DE LAVAR PRATOS |
| 00801 | ABRIDOR E AFIADOR ELETRICO |
| 00802 | AFIADOR ELETRICO |
| 00803 | ABRIDOR ELETRICO |
| 00901 | BATEDEIRA DE BOLO |
| 01001 | CAFETEIRA ELETRICA |
| 01101 | ESPREMEDOR DE FRUTAS ELETRICO |
| 01201 | FACA ELETRICA |
| 01301 | GRILL |
| 01401 | LIQUIDIFICADOR |
| 01501 | MAQUINA DE MOER CARNE ELETRICA |
| 01601 | PURIFICADOR DE AGUA |
| 01602 | OZONIZADOR |
| 01701 | TORRADEIRA ELETRICA |
| 01801 | ASPIRADOR DE PO |
| 01802 | ASPIRADOR DE BOLINHAS |
| 01803 | ASPIRADOR PAPA BOLINHAS |
| 01804 | PAPA BOLINHAS (ASPIRADOR) |
| 01901 | ENCERADEIRA |
| 02001 | FERRO ELETRICO |
| 02101 | MAQUINA DE LAVAR ROUPAS |
| 02201 | MAQUINA DE SECAR ROUPAS |
| 02202 | CENTRIFUGA (SECAR ROUPA) |
| 02301 | ANTENA DE TELEVISAO NAO-PARABOLICA |
| 02401 | TELEVISAO EM CORES |
| 02501 | TELEVISAO EM PRETO E BRANCO |
| 02601 | VIDEOCASSETE |
| 02701 | DVD |
| 02702 | APARELHO DE DVD |
| 02801 | CAIXA DE SOM |
| 02901 | CONJUNTO DE SOM ACOPLADO |
| 02902 | APARELHO DE SOM |
| 02903 | EQUIPAMENTO DE SOM |
| 03001 | GRAVADOR E TOCA-FITAS |
| 03002 | TOCA-FITAS |
| 03003 | RADIO GRAVADOR |
| 03004 | RADIO TOCA-FITAS |
| 03005 | GRAVADOR |
| 03101 | TOCA-DISCOS A LASER |
| 03201 | RADIO PORTATIL |
| 03301 | RADIO RELOGIO OU DE MESA |
| 03302 | RADIO RELOGIO |
| 03303 | RADIO DE MESA |
| 03401 | AMPLIFICADOR |
| 03501 | TAPE-DECK |
| 03601 | TOCA-DISCOS DE AGULHA |
| 03701 | VIDEO GAME |
| 03801 | DISCMAN |
| 03802 | DISKMAN |
| 03803 | WALKMAN |
| 03901 | AR-CONDICIONADO |
| 04001 | AQUECEDOR DE AR |
| 04101 | CIRCULADOR DE AR |
| 04102 | CLIMATIZADOR |
| 04201 | VENTILADOR |
| 04301 | MAQUINA DE COSTURA A PEDAL |
| 04401 | MAQUINA DE COSTURA ELETRICA |
| 04501 | MAQUINA DE COSTURA NAO-ESPECIFICADA |
| 04601 | AQUECEDOR CENTRAL DE AGUA ELETRICO |
| 04701 | AQUECEDOR DE AGUA A GAS |
| 04801 | BOMBA DE AGUA |
| 04802 | MOTOR DE BOMBA DE AGUA |
| 04901 | CHUVEIRO OU DUCHA ELETRICOS |
| 04902 | CHUVEIRO ELETRICO |
| 04903 | DUCHA ELETRICA |
| 05001 | PARAFUSEIRA A BATERIA |
| 05101 | SECADOR E MODELADOR DE CABELO |
| 05102 | SECADOR DE CABELO |
| 05103 | MODELADOR DE CABELO |
| 05104 | PRANCHA ALISADORA DE CABELO |
| 05201 | BARBEADOR ELETRICO |
| 05301 | DEPILADOR ELETRICO |
| 05401 | KARAOKE |
| 05402 | VIDEOKE |
| 05601 | REGULADOR DE VOLTAGEM |
| 05701 | RECEPTOR DE TV A CABO |
| 05702 | APARELHO RECEPTOR DE TV A CABO |
| 05703 | RECEPTOR DE SINAL HD |
| 05801 | CENTRIFUGA DE ALIMENTOS |
| 05901 | TELEFONE-RADIO-RELOGIO |
| 05902 | RADIO-RELOGIO-TELEFONE |
| 06101 | PROCESSADOR DE ALIMENTOS ELETRICO |
| 06102 | PREPARADOR DE ALIMENTOS ELETRICO |
| 06103 | MULTIPROCESSADOR DE ALIMENTOS ELETRICO |
| 06104 | MULTIPREPARADOR DE ALIMENTOS ELETRICO |
| 06201 | MICROCOMPUTADOR |
| 06202 | COMPUTADOR |
| 06301 | ACESSORIOS DE MICROCOMPUTADOR |
| 06302 | ACESSORIOS DE COMPUTADOR |
| 06303 | IMPRESSORA DE MICROCOMPUTADOR |
| 06304 | IMPRESSORA DE COMPUTADOR |
| 06305 | SCANER DE MICROCOMPUTADOR |
| 06306 | SCANER DE COMPUTADOR |
| 06307 | TECLADO PARA COMPUTADOR |
| 06308 | MOUSE PARA COMPUTADOR |
| 06309 | CD-ROM |
| 06310 | CDROM |
| 06311 | DVD-ROM |
| 06312 | DVDROM |
| 06313 | GRAVADOR DE CD E DVD |
| 06314 | COMBO GRAVADOR |
| 06315 | MONITOR PARA COMPUTADOR |
| 06316 | CAIXAS DE SOM PARA COMPUTADOR |
| 06317 | GRAVADOR DE CD |
| 06318 | GRAVADOR DE DVD |
| 06501 | TANQUE DE ROUPA ELETRICO |
| 06502 | TANQUINHO ELETRICO |
| 06601 | FORNO DE MICROONDAS |
| 06701 | APARELHO DE FAX |
| 06702 | FAX (APARELHO) |
| 06801 | FRITADEIRA ELETRICA |
| 06901 | SELADORA |
| 06902 | MAQUINA SELADORA |
| 07101 | ESTERILIZADOR |
| 07201 | NOTEBOOK |
| 07202 | LAPTOP |
| 07203 | COMPUTADOR PORTATIL |
| 07301 | GERADOR DE ENERGIA |
| 07401 | SECRETARIA ELETRONICA |
| 07402 | SECRETARIA ELETRONICA -TELEFONE |
| 07403 | SECRETARIA ELETRONICA - FAX |
| 07501 | TRITURADOR ELETRICO |
| 07601 | BABA ELETRONICA |
| 07701 | ALTO-FALANTE, TWEETER, MEGAFONE, MICROFONE, ETC. |
| 07702 | TWEETER |
| 07703 | MEGAFONE |
| 07704 | MICROFONE |
| 07705 | ALTO-FALANTE |
| 07801 | CONTROLE REMOTO DE TV, SOM, VIDEOCASSETE, ETC. |
| 07802 | CONTROLE REMOTO DE TV |
| 07803 | CONTROLE REMOTO DE SOM |
| 07804 | CONTROLE REMOTO DE VIDEOCASSETE |
| 07901 | APARELHO DE SOLDA |
| 08001 | SAUNA RESIDENCIAL |
| 08201 | ACESSORIOS DE VIDEOCASSETE |
| 08301 | FORNO A LENHA |
| 08401 | LAVADORA DE ALTA PRESSAO |
| 08501 | FORNO A GAS |
| 08601 | MASSAGEADOR ELETRICO |
| 08701 | WATER PEACH |
| 08702 | ESCOVA ELETRICA |
| 08801 | MAQUINA DE FAZER PAO |
| 08901 | SANDUICHEIRA ELETRICA |
| 09001 | VAPORIZADOR ELETRICO |
| 09002 | NEBULIZADOR ELETRICO |
| 09101 | TELEVISAO E RADIO ACOPLADOS |
| 09201 | MAQUINA DE OVERLOCK |
| 09301 | TELEVISAO E VIDEOCASSETE ACOPLADOS |
| 09401 | ANTENA PARABOLICA E EQUIPAMENTOS |
| 09402 | RECEPTOR DE ANTENA PARABOLICA |
| 09501 | RETROPROJETOR |
| 09601 | LAVADORA A VAPOR |
| 09701 | PAINEL SOLAR (ENERGIA SOLAR) |
| 09702 | AQUECEDOR SOLAR |
| 09801 | SUPORTE ELETRICO PARA GARRAFAO DE AGUA |
| 09802 | GELAGUA |
| 09803 | BEBEDOURO |
| 09901 | UMIDIFICADOR DE AR |
| 10001 | TORNEIRA ELETRICA |
| 10101 | HOME THEATER |
| 10201 | IOGURTEIRA |
| 10301 | FILTRO ELETRICO |
| 10401 | ESTUFA ELETRICA |
| 10501 | FATIADOR DE ALIMENTOS |
| 10601 | PALM TOP |
| 10602 | PALMTOP |
| 10603 | PALM |
| 10604 | POCKET PC |
| 10605 | PDA |
| 10701 | MOTOR DE FREEZER |
| 10801 | MOTOR DE MAQUINA DE COSTURA |
| 10901 | AQUECEDOR DE AGUA ELETRICO |
| 10902 | MERGULHAO |
| 11001 | MAQUINA DE BATER ACAI ELETRICA |
| 11101 | MARMITA ELETRICA |
| 11201 | MAQUINA DE CORTAR PISO |
| 11301 | MAQUINA DE ALGODAO |
| 11401 | MAQUINA DE CREPE |
| 11501 | MAQUINA DE BATER SABAO ELETRICA |
| 11601 | MAQUINA DE ESTAMPAR |
| 11701 | MAQUINA DE POLIR |
| 11801 | MAQUINA DE RISCAR CERAMICA |
| 11901 | MAQUINA DE TRICO |
| 12001 | EASY BOX (APARELHO NAVEGADOR DE INTERNET) |
| 12002 | APARELHO NAVEGADOR DE INTERNET |
| 12101 | FONTE DE ALIMENTACAO DE APARELHO ELETRONICO |
| 12102 | ELIMINADOR DE PILHAS |
| 12201 | MOEDOR DE CAFE ELETRICO |
| 12301 | ACESSORIOS DE VIDEO GAME |
| 12401 | FOGAO A GAS |
| 12501 | BINOCULO, LUNETA, TELESCOPIO E LUPA |
| 12502 | LUNETA |
| 12503 | TELESCOPIO |
| 12504 | LUPA |
| 12505 | BINOCULO |
| 12601 | FILMADORA |
| 12701 | MAQUINA FOTOGRAFICA |
| 12702 | CAMERA FOTOGRAFICA DIGITAL |
| 12801 | CALCULADORA MECANICA |
| 12901 | CALCULADORA E AGENDA ELETRONICA |
| 12902 | CALCULADORA ELETRONICA |
| 12903 | AGENDA ELETRONICA |
| 13001 | MAQUINA DE ESCREVER MECANICA |
| 13101 | MAQUINA DE ESCREVER ELETRICA |
| 13201 | CHURRASQUEIRA |
| 13301 | APARELHO DE CHA OU CAFE |
| 13302 | APARELHO DE CHA |
| 13303 | APARELHO DE CAFE |
| 13401 | APARELHO DE JANTAR |
| 13402 | CONJUNTO DE JANTAR |
| 13403 | JOGO DE JANTAR |
| 13501 | BAIXELA |
| 13601 | BATERIA DE COZINHA |
| 13602 | CONJUNTO DE PANELAS |
| 13603 | JOGO DE PANELAS |
| 13701 | FAQUEIRO |
| 13801 | CONJUNTO COMPLETO DE COPOS FINOS OU DE LUXO |
| 13802 | JOGO COMPLETO DE COPOS FINOS OU DE LUXO |
| 13901 | SUPORTE DE TV GIRATORIO |
| 14001 | FILTRO DE AGUA |
| 14002 | FILTRO DE AGUA A VELA |
| 14101 | ACENDEDOR ELETRONICO |
| 14102 | ACENDEDOR DE FOGAO |
| 14201 | MAQUINA DE COSTURA MANUAL |
| 14202 | MINI MAQUINA DE COSTURA MANUAL |
| 14301 | HADFONE |
| 14302 | FONE DE OUVIDO |
| 14303 | EGOISTA (FONE DE OUVIDO) |
| 14401 | FURADEIRA, LIXADEIRA, ESMERIL E SERRA ELETRICA |
| 14402 | SERRADEIRA ELETRICA |
| 14403 | LIXADEIRA ELETRICA |
| 14404 | ESMERIL ELETRICO |
| 14405 | FURADEIRA ELETRICA |
| 14406 | SERRA MARMORE |
| 14407 | PLAINA ELETRICA |
| 14501 | MAQUINA DE PRESSAO PARA BOTAO |
| 14601 | MAQUINA DE MOER CARNE MANUAL |
| 14701 | EQUALIZADOR |
| 14801 | APARELHO TELEFONICO NAO-CELULAR |
| 14802 | APARELHO DE TELEFONE RESIDENCIAL |
| 14901 | LINHA TELEFONICA RESIDENCIAL |
| 15001 | CARRINHO DE FEIRA |
| 15101 | SECADOR DE ROUPA |
| 15201 | ESCADA |
| 15301 | LANTERNA DE PILHA |
| 15401 | TABUA DE PASSAR ROUPA |
| 15501 | INTERFONE |
| 15601 | MAQUINAS AGRICOLAS DE PEQUENO PORTE |
| 15602 | MOENDA DE CANA |
| 15603 | PULVERIZADOR DE AGROTOXICO |
| 15604 | RALADORA |
| 15605 | PICADEIRA |
| 15606 | TRITURADORA |
| 15607 | DEBULHADOR |
| 15608 | MOTOSSERRA |
| 15609 | CORTADOR DE GRAMA |
| 15801 | BALANCA PARA PESSOAS |
| 15802 | BALANCA DE BANHEIRO |
| 15901 | AQUECEDOR DE MAMADEIRA ELETRICO |
| 16001 | APARELHO PARA MASSAS |
| 16002 | MAQUINA PARA MASSAS |
| 16003 | APARELHO PARA MACARRAO |
| 16004 | MAQUINA PARA MACARRAO |
| 16005 | CILINDROS PARA MASSAS |
| 16101 | RELOGIO DESPERTADOR |
| 16301 | CAMPAINHA MUSICAL |
| 16302 | CAMPAINHA ELETRICA |
| 16303 | CAMPAINHA |
| 16401 | BALANCA PARA ALIMENTOS |
| 16501 | RAMAL TELEFONICO |
| 16502 | LINHA TELEFONICA COMUNITARIA (RAMAL) |
| 16601 | TELAO |
| 16701 | MICROSCOPIO |
| 16801 | CAFETEIRA NAO-ELETRICA |
| 16901 | BINA (IDENTIFICADOR DE TELEFONE) |
| 16902 | BINA |
| 17001 | APARELHO PARA FONDUE |
| 17101 | APARELHO TELEFONICO COM BINA |
| 17201 | APARELHO E LINHA TELEFONICA |
| 17301 | BLOQUEADOR DE CHAMADAS |
| 17401 | CENTRAL TELEFONICA |
| 17501 | ANTENA TELEFONICA |
| 17701 | SUPORTE PARA FORNO |
| 17801 | ACESSORIOS PARA MAQUINA FOTOGRAFICA |
| 17901 | FOTOMETRO |
| 18001 | FERRO A GAS |
| 18101 | FERRO A CARVAO |
| 18201 | FOGAREIRO |
| 18301 | FORNO DE TORRAR FARINHA |
| 18401 | BATERIA DE FILMADORA |
| 18501 | JOGO DE PORCELANA |
| 18601 | MAQUINA DE BATER ACAI MANUAL |
| 18602 | MAQUINA DE BATER ALIMENTOS MANUAL |
| 18701 | FORNO DE FERRO |
| 18801 | MAQUINA DE MOER MILHO MANUAL |
| 18802 | MAQUINA PARA RALAR MANDIOCA MANUAL |
| 18901 | COMPRESSOR DE AR |
| 19001 | PISCINA DE PLASTICO |
| 19002 | PISCINA DE FIBRA |
| 19101 | ANDADOR |
| 19102 | VOADOR (ANDADOR) |
| 19201 | BEBE-CONFORTO |
| 19301 | BABY BAG |
| 19302 | CANGURU (BABY BAG) |
| 19401 | RELOGIO DE MESA OU PAREDE |
| 19402 | RELOGIO DE MESA |
| 19403 | RELOGIO DE PAREDE |
| 19701 | CONVERSOR DIGITAL PARA TELEVISAO |
| 20001 | PEN DRIVE |
| 20601 | TOCADOR DE MP PLAYER |
| 20602 | MP3 PLAYER |
| 20603 | MP4 PLAYER |
| 20604 | MP5 PLAYER |
| 20605 | TOCADOR DE MP3/MP4/MP5 |
| 20701 | CARTAO DE MEMORIA |
| 20801 | CERCA ELETRICA |
| 20901 | CAMERA DE SEGURANCA |
| 21001 | RECARREGADOR DE PILHAS |
| 21101 | APARELHO DE FAZER PIPOCA ELETRICO |
| 21201 | ALMOFADA TERMICA VIBRATORIA |
| 21301 | ANTENA DE INTERNET VIA RADIO |
| 21401 | MAQUINA DE CORTAR CABELO ELETRICA |
| 21601 | PANELA ELETRICA |
| 21602 | JARRA ELETRICA |
| 99901 | AGREGADO |
| 00101 | ACESSORIOS DE INSTRUMENTOS MUSICAIS |
| 00201 | GUITARRA |
| 00301 | PIANO |
| 00401 | VIOLAO, VIOLA |
| 00501 | CAVAQUINHO |
| 00601 | ORGAO E TECLADO |
| 00602 | TECLADO |
| 00701 | ACORDEAO |
| 00801 | FLAUTA |
| 00901 | GAITA |
| 01001 | BATERIA MUSICAL |
| 01101 | CONTRABAIXO |
| 01201 | CITARA |
| 01301 | BERIMBAU |
| 01401 | SAXOFONE |
| 01501 | TROMBONE, TROMPETE |
| 01601 | FERRAMENTAS EM GERAL |
| 01602 | ALICATE E CHAVE (BOCA, ESTRIA, ETC.) |
| 01603 | CONJUNTO DE FERRAMENTAS |
| 01604 | CHAVE DE FENDA |
| 01605 | MARTELO |
| 01606 | SERRA MANUAL |
| 01607 | ARCO DE SERRA |
| 01608 | FURADEIRA MANUAL |
| 01609 | PUA |
| 01610 | SERROTE |
| 01611 | ENXADA |
| 01612 | CAVADEIRA |
| 01613 | ANCINHO |
| 01614 | TERCADO |
| 01615 | PICARETA |
| 01616 | PE-DE-CABRA |
| 01617 | FOICE |
| 01618 | PENEIRA |
| 01619 | NIVEL |
| 01620 | ESCALA |
| 01621 | RISCADOR |
| 01622 | PONTEIRA |
| 01623 | CORTADOR DE AZULEJO |
| 01624 | PA |
| 01625 | METRO |
| 01626 | MARRETA |
| 01627 | CHAVE INGLESA |
| 01628 | BROCA |
| 01629 | TARRACHA |
| 01630 | PLAINA |
| 01631 | FERRO DE SOLDAR |
| 01632 | MACARICO DE SOLDAR |
| 01633 | SUPORTE DE FERRO DE SOLDAR |
| 01634 | LIMA |
| 01635 | GROSA |
| 01636 | FORMAO |
| 01637 | CARRINHO DE MAO |
| 01638 | TESOURA DE CORTAR LATAO |
| 01639 | MACHADO |
| 01640 | CHAVE DE TESTE |
| 01641 | PISTOLA DE PINTURA |
| 01642 | REBITADEIRA |
| 01643 | TORNO DE MESA |
| 01644 | MORSA |
| 01645 | PODAO |
| 01646 | LAMINA DE SERRA |
| 01647 | SERRA (LAMINA DE ACO) |
| 01648 | DESEMPENADEIRA |
| 01649 | COLHER DE PEDREIRO |
| 01650 | JOGO DE FERRAMENTAS |
| 01652 | ALICATE |
| 01653 | CHAVE DE BOCA |
| 01654 | CORRENTE DE MOTOSSERA |
| 01655 | DISCO DE SERRA |
| 01656 | FACAO (FERRAMENTA) |
| 01657 | MANGUEIRA DE NIVEL |
| 01658 | MULTIMETRO |
| 01659 | TESOURA DE JARDINEIRO |
| 01660 | TRENA METRICA |
| 01661 | PLANTADEIRA MANUAL |
| 01701 | CAIXA DE FERRAMENTAS |
| 01801 | COMPRA DE ANIMAL DOMESTICO |
| 01802 | ANIMAL DOMESTICO |
| 01901 | TOSADEIRA DE ANIMAL DOMESTICO |
| 02001 | ARTIGOS DE LONA OU PLASTICO PARA ACAMPAMENTO |
| 02002 | PLASTICO PARA ACAMPAMENTO |
| 02003 | LONA PARA ACAMPAMENTO |
| 02004 | SACO DE DORMIR |
| 02005 | COLCHAO DE ACAMPAMENTO |
| 02006 | COLCHAO DE AR |
| 02101 | FOGAO PARA CAMPING |
| 02201 | BARRACA DE ACAMPAMENTO |
| 02301 | LAMPIAO |
| 02302 | LAMPARINA |
| 02401 | TRATAMENTO DE SAUDE DE ANIMAL DOMESTICO |
| 02402 | VACINA PARA ANIMAL DOMESTICO |
| 02403 | INJECAO PARA ANIMAL DOMESTICO |
| 02404 | MEDICAMENTO PARA ANIMAL DOMESTICO |
| 02405 | REMEDIO PARA ANIMAL DOMESTICO |
| 02406 | FORTIFICANTE PARA ANIMAL DOMESTICO |
| 02407 | COLEIRA ANTI PULGA |
| 02501 | SERVICO DE HIGIENE DE ANIMAL DOMESTICO |
| 02502 | TOSA DE PELOS DE ANIMAL DOMESTICO |
| 02503 | CORTE DE PELOS DE ANIMAL DOMESTICO |
| 02504 | BANHO DE ANIMAL DOMESTICO |
| 02505 | BANHO E TOSA DE ANIMAL DOMESTICO |
| 02506 | LIMPEZA DE DENTES DO CACHORRO |
| 02601 | VETERINARIO |
| 02701 | ADESTRAMENTO DE ANIMAL DOMESTICO |
| 02801 | VIOLINO |
| 02901 | PANDEIRO |
| 03001 | CLARINETE |
| 03101 | BANJO |
| 03201 | ATABAQUE |
| 03301 | BERRANTE |
| 03401 | BONGO |
| 03601 | DJEMBE |
| 03701 | TAM-TAM, CONGA |
| 03801 | SURDO, REPINIQUE, TAMBOR, CAIXA, BUMBO |
| 03901 | OBJETO DE ANIMAL |
| 03902 | CORRENTE DE ANIMAL |
| 03903 | COLEIRA DE ANIMAL |
| 03904 | GAIOLA DE ANIMAL |
| 03905 | OBJETO PARA ANIMAL |
| 03906 | CORRENTE PARA ANIMAL |
| 03907 | COLEIRA PARA ANIMAL |
| 03908 | GAIOLA PARA ANIMAL |
| 03909 | ENFORCADOR DE ANIMAL |
| 03910 | FOCINHEIRA DE ANIMAL |
| 03911 | ENFORCADOR PARA ANIMAL |
| 03912 | FOCINHEIRA PARA ANIMAL |
| 03913 | CASA DE ANIMAL |
| 03914 | AGASALHO PARA ANIMAL |
| 03915 | AREIA PARA GATO |
| 04001 | MARACA |
| 99901 | AGREGADO |
| 00101 | ARCA COM OU SEM VITRINE OU ORATORIO |
| 00201 | CADEIRA (SALA) |
| 00202 | CADEIRA (ESTAR E JANTAR) |
| 00301 | CARRINHO DE CHA OU DE BEBIDAS |
| 00302 | CARRINHO DE CHA |
| 00303 | CARRINHO DE BEBIDAS |
| 00401 | CRISTALEIRA OU VITRINE |
| 00402 | CRISTALEIRA |
| 00403 | VITRINE |
| 00501 | CONJUNTO ESTOFADO |
| 00601 | CONJUNTO ESTOFADO E MESINHA(S) |
| 00701 | ESTANTE |
| 00702 | RACK |
| 00703 | ARMARIO EMBUTIDO (SALA) |
| 00704 | ESTANTE EMBUTIDO (SALA) |
| 00801 | MESA E CADEIRAS (SALA) |
| 00802 | MESA E CADEIRAS (ESTAR E JANTAR) |
| 00901 | POLTRONA |
| 01001 | SOFA OU SOFA-CAMA |
| 01002 | SOFA |
| 01003 | SOFA-CAMA |
| 01101 | MESA PARA TV, SOM, VIDEO E MICROCOMPUTADOR |
| 01102 | MESA PARA TV |
| 01103 | MESA PARA SOM |
| 01104 | MESA PARA VIDEO |
| 01105 | MESA PARA COMPUTADOR |
| 01106 | MESA PARA MICROCOMPUTADOR |
| 01201 | PORTA-JORNAL E REVISTA |
| 01202 | PORTA-JORNAL |
| 01203 | PORTA-REVISTA |
| 01301 | CADEIRA DE BALANCO |
| 01401 | MESINHA DE CENTRO |
| 01501 | MESA (SALA) |
| 01502 | TAMPO DE MESA (SALA) (QUALQUER MATERIAL) |
| 01503 | BASE PARA MESA (SALA) (QUALQUER MATERIAL) |
| 01504 | MESA (ESTAR E JANTAR) |
| 01601 | BAU (SALA) |
| 01701 | BAR (SALA) |
| 01702 | BARZINHO (SALA) |
| 01703 | BAR |
| 01801 | BALCAO |
| 01901 | MOVEIS PARA SALA |
| 02001 | ARMARIO SIMPLES, DUPLEX OU EMBUTIDO (QUARTO DE ADULTO) |
| 02002 | ARMARIO SIMPLES, DUPLEX OU EMBUTIDO |
| 02003 | ARMARIO SIMPLES |
| 02004 | ARMARIO DUPLEX |
| 02005 | ARMARIO EMBUTIDO |
| 02006 | GUARDA ROUPA |
| 02007 | GUARDA ROUPAS |
| 02101 | DORMITORIO COMPLETO (ARMARIO, CAMA, MESINHA, ETC.) |
| 02102 | DORMITORIO COMPLETO (ARMARIO, CAMA, ETC.) |
| 02201 | BICAMA, BELICHE OU TRILICHE |
| 02202 | BELICHE |
| 02203 | TRILICHE |
| 02204 | BICAMA |
| 02301 | CAMA (QUARTO ADULTO) |
| 02302 | CAMA |
| 02303 | CAMA DE CASAL |
| 02304 | CAMA BOX |
| 02401 | COLCHAO (QUARTO ADULTO) |
| 02402 | COLCHAO DE ADULTO |
| 02403 | COLCHAO INFLAVEL |
| 02501 | COMODA (QUARTO ADULTO) |
| 02502 | COMODA |
| 02601 | CONSOLE |
| 02701 | PENTEADEIRA |
| 02801 | GAVETEIRO |
| 02901 | MESINHA DE CABECEIRA (QUARTO ADULTO) |
| 02902 | MESINHA DE CABECEIRA |
| 02903 | CRIADO MUDO |
| 03001 | CAMA E MESINHA DE CABECEIRA (QUARTO ADULTO) |
| 03101 | CABIDE DE PE |
| 03201 | BAU (QUARTO ADULTO) |
| 03301 | DIVISORIA (QUARTO ADULTO) |
| 03401 | CADEIRA (QUARTO ADULTO) |
| 03501 | BANCO (SALA) |
| 03601 | MOVEL DE SAPATOS |
| 03602 | SAPATARIA (MOVEL PARA SAPATOS) |
| 03701 | MOVEL PARA MAQUINA DE COSTURA (GABINETE) |
| 03702 | GABINETE (MOVEL PARA MAQUINA DE COSTURA) |
| 03801 | GUARDA LOUCA |
| 03901 | LAVANDERIA PLANEJADA |
| 04001 | ARMARIO (COPA E COZINHA) |
| 04002 | GABINETE DE PIA (COPA E COZINHA) |
| 04101 | ARMARIO, MESA E CADEIRAS (COPA E COZINHA) |
| 04102 | ARMARIO, MESA E CADEIRAS |
| 04201 | BANCO (COPA E COZINHA) |
| 04202 | BANCO |
| 04301 | BUFE ( COPA E COZINHA) |
| 04401 | MESA E CADEIRAS (COPA E COZINHA) |
| 04501 | MESA (COPA E COZINHA) |
| 04601 | CADEIRA (COPA E COZINHA) |
| 04701 | BALCAO PARA PASSAR ROUPA |
| 04801 | MESA E BANCOS NAO-ESPECIFICADOS |
| 04901 | MOVEIS DE COPA E COZINHA NAO-ESPECIFICADOS |
| 05001 | MESA E CADEIRAS NAO-ESPECIFICADAS |
| 05101 | MESA NAO-ESPECIFICADA |
| 05201 | GABINETE DE PIA (BANHEIRO) |
| 05301 | PUFE |
| 05401 | CADEIRA PARA COMPUTADOR |
| 05402 | CADEIRA GIRATORIA |
| 05501 | MOVEIS PARA ESCRITORIO |
| 05601 | MOVEIS DE QUARTO NAO-ESPECIFICADOS |
| 05701 | MOVEIS NAO-ESPECIFICADOS |
| 05801 | BIOMBO |
| 05901 | ARQUIVO DE ACO |
| 06001 | ARMARIO (MOVEL INFANTIL) |
| 06002 | ARMARIO INFANTIL |
| 06101 | ARMARIO, CAMA E MESINHA DE CABECEIRA (MOVEIS INFANTIS) |
| 06102 | ARMARIO CAMA E MESINHA DE CABECEIRA |
| 06201 | PRATELEIRA |
| 06202 | CANTONEIRA (PRATELEIRA) |
| 06301 | BERCO |
| 06401 | CADEIRA ALTA DE REFEICAO |
| 06501 | CAMA (MOVEL INFANTIL) |
| 06502 | CAMA INFANTIL |
| 06601 | CARRINHO DE BEBE E GONDOLA |
| 06602 | GONDOLA |
| 06603 | CARRINHO DE BEBE |
| 06701 | COLCHAO (INFANTIL) |
| 06702 | COLCHAO INFANTIL |
| 06801 | MESINHA E CADEIRAS (MOVEIS INFANTIS) |
| 06901 | CERCADO |
| 06902 | CERCADO DE CRIANCA |
| 07001 | MESINHA INFANTIL |
| 07101 | ARMARIO NAO-ESPECIFICADO |
| 07201 | CESTA DE VIME |
| 07301 | APARADOR |
| 07401 | BANHEIRA COM TROCA-FRALDAS |
| 07501 | CADEIRA NAO-ESPECIFICADA |
| 07601 | CADEIRINHA (INFANTIL) |
| 07602 | CADEIRA PEQUENA (INFANTIL) |
| 07603 | CADEIRINHA |
| 07701 | COMODA (MOVEL INFANTIL) |
| 07702 | COMODA INFANTIL |
| 07801 | ESTANTE (MOVEL INFANTIL) |
| 07901 | MOVEIS DE QUARTO INFANTIL NAO-ESPECIFICADOS |
| 08001 | BANCO (JARDIM, VARANDA, ETC.) |
| 08002 | BANCO DE JARDIM |
| 08003 | BANCO DE VARANDA |
| 08101 | CADEIRA (JARDIM, VARANDA, ETC.) |
| 08102 | CADEIRA DE JARDIM |
| 08103 | CADEIRA DE VARANDA |
| 08201 | GUARDA-SOL |
| 08301 | MESA (JARDIM, VARANDA, ETC.) |
| 08302 | MESA DE JARDIM |
| 08303 | MESA DE VARANDA |
| 08401 | MESA COM CADEIRAS (JARDIM, VARANDA, ETC.) |
| 08402 | MESA COM CADEIRAS DE JARDIM |
| 08403 | MESA COM CADEIRAS DE VARANDA |
| 08501 | REDE DE ADULTO E CRIANCA |
| 08502 | REDE DE ADULTO |
| 08503 | REDE DE CRIANCA |
| 08601 | ESPREGUICADEIRA |
| 08701 | COLCHONETE |
| 08801 | ESCRIVANINHA |
| 08802 | BUREAU |
| 08901 | MESA DE TELEFONE |
| 08902 | MESINHA DE TELEFONE, DE VENTILADOR, ETC. |
| 08903 | MESINHA DE TELEFONE |
| 08904 | MESINHA DE VENTILADOR |
| 09001 | ARMARIO DE BANHEIRO (NAO PLASTICO) |
| 09101 | ESTEIRA DE DORMIR |
| 99901 | AGREGADO |
| 00101 | TAPECARIA |
| 00201 | TAPETE DE SALA OU QUARTO |
| 00301 | PASSADEIRA |
| 00401 | TAPETE DE PANO |
| 00501 | TAPETE DE BANHEIRO (TECIDO) |
| 00601 | ALMOFADA |
| 00602 | CAPA DE ALMOFADA |
| 00603 | PROTETOR DE BERCO |
| 00604 | ALMOFADA (PROTETOR DE BERCO) |
| 00605 | ALMOFADAO (PROTETOR DE BERCO) |
| 00701 | CAPA PARA SOFA |
| 00801 | CORTINA PRONTA (EXCETO DE PLASTICO) |
| 00901 | ESCULTURA |
| 01001 | LUMINARIA, ABAJUR, LUSTRE, GLOBO DE LUZ, ETC. |
| 01002 | ABAJUR |
| 01003 | LUSTRE |
| 01004 | GLOBO DE LUZ |
| 01101 | PECA DE LOUCA (DECORACAO) |
| 01102 | LOUCA (PECA DE DECORACAO) |
| 01201 | PECA DE METAL (EXCETO PRATA) (DECORACAO) |
| 01202 | METAL EXCETO PRATA (PECA DE DECORACAO) |
| 01301 | PECA DE PRATA (DECORACAO) |
| 01302 | PRATA (PECA DE DECORACAO) |
| 01401 | ESPELHO |
| 01501 | QUADRO OU MOLDURA DE QUADRO, POSTER, PORTA-RETRATO, ETC. |
| 01502 | TELA A OLEO |
| 01503 | MOLDURA DE QUADRO OU FOTO |
| 01504 | GRAVURA |
| 01505 | POSTER |
| 01506 | PORTA-RETRATO |
| 01601 | PERSIANAS |
| 01701 | PUFE |
| 01801 | AQUARIO ORNAMENTAL E EQUIPAMENTOS |
| 01802 | EQUIPAMENTOS DE AQUARIO ORNAMENTAL |
| 01803 | FILTRO BIOLOGICO DE AQUARIO ORNAMENTAL |
| 01804 | OXIGENADOR DE AQUARIO ORNAMENTAL |
| 01901 | PECA DE CERAMICA (DECORACAO) |
| 01902 | CERAMICA (PECA DE DECORACAO) |
| 02001 | VELA DECORATIVA |
| 02101 | JARRO DE BARRO (DECORACAO) |
| 02201 | PECA DE GESSO (DECORACAO) |
| 02202 | GESSO (PECA DE DECORACAO) |
| 02301 | GAIOLA (DECORACAO) |
| 02401 | PECA DE MADEIRA (DECORACAO) |
| 02402 | MADEIRA (PECA DE DECORACAO) |
| 02403 | FLOREIRA DE MADEIRA |
| 02501 | ENFEITE DE BERCO |
| 02502 | MOBILE (ENFEITE DE BERCO) |
| 02601 | PECA DE CRISTAL (DECORACAO) |
| 02602 | CRISTAL (PECA DE DECORACAO) |
| 02701 | LEQUE (PECA DE DECORACAO) |
| 02801 | BIBELO DE QUALQUER MATERIAL |
| 02802 | BONEQUINHOS DE QUALQUER MATERIAL (DECORACAO) |
| 02803 | ARTESANATO DECORATIVO |
| 02901 | PECA DE PORCELANA (DECORACAO) |
| 02902 | PORCELANA (PECA DE DECORACAO) |
| 03001 | PECA DE VIDRO (DECORACAO) |
| 03002 | VIDRO (PECA DE DECORACAO) |
| 03101 | CINZEIRO |
| 03201 | ENFEITES DE ARVORE DE NATAL |
| 03202 | ARVORE DE NATAL (ARTIFICIAL) |
| 03203 | ENFEITES DE NATAL |
| 03204 | ENFEITES DIVERSOS |
| 03301 | PECA DE PALHA (DECORACAO) |
| 03302 | PALHA (PECA DE DECORACAO) |
| 03401 | SERIGRAFIA (ESTAMPARIA) |
| 03501 | BUZIOS, CONCHAS (DECORACAO) |
| 03601 | SUPORTE DE BIBLIA |
| 03701 | ISQUEIRO DE MESA |
| 03801 | PECA DE PLASTICO OU RESINA (DECORACAO) |
| 03802 | PLASTICO OU RESINA (PECA DE DECORACAO) |
| 03901 | PEDESTAL |
| 04001 | PECA DE CORDA (DECORACAO) |
| 04002 | CORDA (PECA DE DECORACAO) |
| 04101 | PECA DE PEDRA SABAO (DECORACAO) |
| 04102 | PEDRA SABAO (PECA DE DECORACAO) |
| 04201 | PAPIRO |
| 04301 | PECA DE TECIDO (DECORACAO) |
| 04302 | TECIDO (PECA DE DECORACAO) |
| 04401 | CASCO DE TARTARUGA |
| 04501 | BANDO |
| 04601 | FLORES ARTIFICIAIS |
| 04602 | PECAS DE CONFECCAO DE FLORES ARTIFICIAIS |
| 04603 | FORMAS DE CONFECCAO DE FLORES ARTIFICIAIS |
| 04604 | PLANTAS ARTIFICIAIS |
| 04701 | PORTA-JOIAS |
| 04801 | CAIXINHA DE MUSICA |
| 04901 | PECA DE AGATA OU ESMALTADA (DECORACAO) |
| 04902 | AGATA OU ESMALTADA (PECA DE DECORACAO) |
| 05001 | PORTA-LAPISEIRA |
| 05101 | PORTA-CD |
| 05102 | PORTA-FITAS CASSETE |
| 05103 | PORTA-COMPACT DISC |
| 05104 | PORTA-FITAS DE VIDEOCASSETE |
| 05201 | PEDRAS DECORATIVAS |
| 05301 | PRESEPIO (DECORACAO) |
| 99901 | AGREGADO |
| 00101 | EMPREGADA |
| 00102 | EMPREGADO |
| 00201 | COZINHEIRO |
| 00202 | COZINHEIRA |
| 00301 | FAXINEIRA |
| 00302 | FAXINEIRO |
| 00401 | BABA |
| 00501 | JARDINEIRO |
| 00502 | JARDINEIRA |
| 00601 | MOTORISTA |
| 00701 | VIGIA |
| 00702 | ZELADOR |
| 00703 | PORTEIRO |
| 00704 | PORTEIRA |
| 00801 | ARRUMADEIRA |
| 00802 | ARRUMADOR |
| 00901 | GOVERNANTA |
| 01001 | MORDOMO |
| 01101 | CASEIRO |
| 01102 | CASEIRA |
| 01201 | ACOMPANHANTE |
| 01301 | LAVADEIRA |
| 01302 | LAVADEIRO |
| 01401 | AJUDANTE DE COZINHA |
| 01501 | PASSADEIRA |
| 01601 | COPEIRA |
| 01602 | COPEIRO |
| 01701 | LAVADEIRA E PASSADEIRA |
| 01702 | PASSADEIRA E LAVADEIRA |
| 01801 | GARCOM |
| 01802 | GARCONETE |
| 01901 | CARREGADOR DE AGUA |
| 01902 | PUXADOR DE AGUA |
| 02001 | LIMPADOR DE TERRENO (CAPINADOR) |
| 02002 | CAPINADOR (LIMPADOR DE TERRENO) |
| 02003 | LIMPADOR DE TERRENO |
| 02004 | CAPINADOR |
| 02101 | LIMPADOR DE PISCINA |
| 02102 | LAVADOR DE PISCINA |
| 02201 | LAVADOR DE CARRO |
| 02301 | BISCATEIRO (SERVICOS DIVERSOS) |
| 02302 | BISCATEIRO |
| 02401 | LIMPADOR DE FOSSA |
| 02501 | ENFERMEIRA (SERVICO DOMESTICO) |
| 02502 | ENFERMEIRA |
| 02503 | ENFERMEIRO |
| 02601 | CARREGADOR DE LENHA |
| 02701 | TRATADOR DE ANIMAIS |
| 02702 | BABA DE CACHORRO |
| 02801 | SALVA-VIDAS |
| 02901 | SERVENTE |
| 03001 | MARINHEIRO |
| 03002 | MARINHEIRA |
| 09901 | AGREGADO |
| 00101 | CORREIO (CARTA, SELO, ETC.) |
| 00102 | CARTA |
| 00103 | TELEGRAMA COMUM |
| 00104 | SELO |
| 00105 | ENCOMENDA |
| 00106 | AEROGRAMA |
| 00107 | ORDEM DE PAGAMENTO (CORREIO) |
| 00108 | TELEX |
| 00109 | CAIXA POSTAL |
| 00110 | REEMBOLSO POSTAL |
| 00111 | FAX SIMILE |
| 00112 | SEDEX |
| 00123 | MALA DIRETA |
| 00201 | TELEFONE PUBLICO |
| 00202 | FICHA TELEFONICA |
| 00203 | CARTAO TELEFONICO |
| 00301 | TELEGRAMA FONADO |
| 00401 | TELEMENSAGEM |
| 00501 | ACESSO A INTERNET EM LOJA |
| 00502 | USO DE INTERNET |
| 00503 | SALA DE INTERNET |
| 99901 | AGREGADO |
| 00101 | ONIBUS URBANO |
| 00201 | TREM |
| 00301 | TAXI |
| 00302 | AUTOMOVEL DE ALUGUEL (TAXI) |
| 00401 | METRO |
| 00501 | BARCA |
| 00502 | BARCO |
| 00503 | BOTE |
| 00504 | CANOA |
| 00505 | CANOA MOTORIZADA |
| 00506 | CATRAIA |
| 00507 | CATAMARA |
| 00508 | AEROBARCO |
| 00509 | FERRY-BOAT |
| 00510 | BALSA |
| 00601 | ALCOOL (COMBUSTIVEL DE VEICULO) |
| 00701 | GASOLINA COMUM (COMBUSTIVEL DE VEICULO) |
| 00801 | ESTACIONAMENTO |
| 00802 | FLANELINHA |
| 00803 | ESTACIONAMENTO EM SHOPPING |
| 00804 | ESTACIONAMENTO-METRO |
| 00901 | PEDAGIO |
| 01001 | INTEGRACAO ONIBUS-METRO |
| 01101 | INTEGRACAO TREM-METRO |
| 01201 | BICICLETA |
| 01301 | OLEO DIESEL (COMBUSTIVEL DE VEICULO) |
| 01401 | BONDE, BONDINHO, ETC. |
| 01501 | PLANO INCLINADO |
| 01502 | ELEVADOR |
| 01601 | INTEGRACAO TREM-ONIBUS |
| 01701 | QUEROSENE (COMBUSTIVEL DE VEICULO) |
| 01801 | GAS VEICULAR |
| 01901 | LAVAGEM SEM LUBRIFICACAO |
| 01902 | LAVA-JATO |
| 02001 | ONIBUS INTERMUNICIPAL |
| 02101 | ONIBUS INTERESTADUAL |
| 02201 | AVIAO |
| 02301 | TRANSPORTE ALTERNATIVO |
| 02302 | LOTADA |
| 02303 | KOMBI |
| 02304 | VAN |
| 02305 | CAMINHAO |
| 02306 | CAMIONETE |
| 02307 | CARONA EM AUTOMOVEL |
| 02308 | JEEP |
| 02309 | TRATOR |
| 02501 | BANHEIRO PUBLICO |
| 02601 | MOTO-TAXI |
| 02602 | TAXI-MOTO |
| 02801 | GASOLINA ESPECIAL (COMBUSTIVEL DE VEICULO) |
| 02802 | GASOLINA ADITIVADA (COMBUSTIVEL DE VEICULO) |
| 99901 | AGREGADO |
| 00101 | ALMOCO OU JANTAR A PESO |
| 00102 | ALMOCO A PESO |
| 00103 | JANTAR A PESO |
| 00104 | ALMOCO A PESO E BEBIDA |
| 00105 | JANTAR A PESO E BEBIDA |
| 00106 | ALMOCO A PESO E SOBREMESA |
| 00107 | JANTAR A PESO E SOBREMESA |
| 00108 | ALMOCO A PESO, BEBIDA E SOBREMESA |
| 00109 | JANTAR A PESO, BEBIDA E SOBREMESA |
| 00201 | LEITE |
| 00301 | PAO COM OU SEM MANTEIGA |
| 00302 | PAO COM MANTEIGA |
| 00303 | PAO SEM MANTEIGA |
| 00304 | PAO DOCE |
| 00305 | PAO DE FORMA |
| 00306 | PAO FRANCES |
| 00401 | SANDUICHE |
| 00402 | CACHORRO QUENTE |
| 00403 | CHEESE BURGUER |
| 00404 | BAURU |
| 00405 | AMERICANO |
| 00406 | TORRADA COM QUEIJO, PRESUNTO OU OVO |
| 00407 | HAMBURGUER |
| 00408 | CHEESE EGG |
| 00409 | MISTO QUENTE OU FRIO |
| 00410 | CHEESE TUDO |
| 00411 | EGGSBURGUER |
| 00412 | SANDUICHE DE QUEIJO PRATO |
| 00413 | SANDUICHE DE SALAME |
| 00414 | SANDUICHE DE PRESUNTO |
| 00415 | SANDUICHE DE QUEIJO PRATO COM PRESUNTO |
| 00416 | SANDUICHE DE MORTADELA |
| 00417 | SANDUICHE DE QUEIJO MINAS |
| 00501 | CAFEZINHO |
| 00502 | CAFE TIPO EXPRESSO |
| 00503 | CAFE TIPO CAPUCCINO |
| 00601 | REFRIGERANTE DE COLA |
| 00602 | COCA-COLA |
| 00603 | PEPSI-COLA |
| 00701 | VITAMINA |
| 00702 | VITAMINA DE FRUTAS OU VEGETAIS |
| 00703 | VITAMINA DE BANANA |
| 00704 | VITAMINA DE BANANA COM AVEIA |
| 00705 | VITAMINA DE MAMAO |
| 00706 | VITAMINA DE ABACATE |
| 00707 | VITAMINA DE MORANGO |
| 00708 | VITAMINA MISTA |
| 00709 | VITAMINA DE MACA |
| 00710 | GEMADA |
| 00801 | BALA OU CHICLETE |
| 00802 | BALA |
| 00803 | CHICLETE |
| 00804 | PIRULITO |
| 00901 | AGUARDENTE |
| 00902 | CACHACA |
| 00903 | PINGA |
| 01001 | CERVEJA CLARA OU PRETA |
| 01002 | CERVEJA CLARA |
| 01003 | CERVEJA PRETA |
| 01004 | CERVEJA SEM ALCOOL |
| 01101 | OUTRAS BEBIDAS ALCOOLICAS |
| 01102 | CAIPIRINHA |
| 01201 | CHOPP CLARO OU ESCURO |
| 01202 | CHOPE CLARO OU ESCURO |
| 01301 | IOGURTE |
| 01302 | IOGURTE DE SABORES |
| 01401 | FRUTA |
| 01402 | BANANA |
| 01403 | LARANJA |
| 01404 | MACA |
| 01405 | MANGA |
| 01406 | PERA |
| 01407 | PUPUNHA |
| 01408 | ATA |
| 01501 | SALADA DE FRUTAS |
| 01601 | CAFE COM LEITE |
| 01701 | AGUA TONICA |
| 01801 | BISCOITO DOCE |
| 01802 | BOLACHA DOCE |
| 01803 | BISCOITO WAFFER |
| 01901 | BISCOITO SALGADO |
| 01902 | BOLACHA SALGADA |
| 02001 | ACAI (EMULSAO) |
| 02002 | ACAI COM GRANOLA |
| 02101 | CALDO DE CARURU, CARNE, FEIJAO OU MOCOTO |
| 02102 | CALDO DE CARURU |
| 02103 | CALDO DE CARNE |
| 02104 | CALDO DE FEIJAO |
| 02105 | CALDO DE MOCOTO |
| 02201 | CHA PREPARADO |
| 02202 | CHA MATE PREPARADO |
| 02203 | CHA PRETO PREPARADO |
| 02301 | REFRIGERANTE DE GUARANA |
| 02302 | GUARANA |
| 02303 | TUBAINA |
| 02401 | BEBIDA ISOTONICA |
| 02402 | GATORADE |
| 02403 | MARATHON |
| 02404 | SPORTADE |
| 02501 | AGUA MINERAL |
| 02502 | AGUA MINERAL COM GAS |
| 02503 | AGUA MINERAL SEM GAS |
| 02504 | AGUA GASEIFICADA COM SABOR |
| 02505 | H2O LIMAO |
| 02506 | H2O TANGERINA |
| 02601 | SUCO DE FRUTAS OU VEGETAIS |
| 02602 | SUCO DE FRUTAS |
| 02603 | SUCO DE VEGETAIS |
| 02604 | SUCO DE ABACAXI |
| 02605 | SUCO DE ACEROLA |
| 02606 | SUCO DE BETERRABA |
| 02607 | SUCO DE CUPUACU |
| 02608 | SUCO DE GOIABA |
| 02609 | SUCO DE LARANJA |
| 02610 | SUCO DE LARANJA E BANANA |
| 02611 | SUCO DE LARANJA E BETERRABA |
| 02612 | SUCO DE LARANJA E CENOURA |
| 02613 | SUCO DE LARANJA, CENOURA E BETERRABA |
| 02614 | SUCO DE MAMAO |
| 02615 | SUCO DE MANGA |
| 02616 | SUCO DE MARACUJA |
| 02617 | SUCO DE MELAO |
| 02618 | SUCO DE MORANGO |
| 02619 | SUCO DE PESSEGO |
| 02620 | SUCO DE PESSEGO EM CALDAS |
| 02621 | CALDO DE CANA |
| 02622 | CAJUINA (SUCO) |
| 02701 | REFRESCO DE FRUTAS |
| 02702 | REFRESCO DE MARACUJA |
| 02703 | REFRESCO DE CAJU |
| 02704 | REFRESCO DE GROSELHA |
| 02705 | REFRESCO DE LARANJA |
| 02801 | YAKULT |
| 02901 | COALHADA |
| 03001 | AGUA DE COCO |
| 03002 | COCO VERDE (AGUA DE COCO) |
| 03101 | BEBIDA ENERGETICA |
| 03102 | RED BULL |
| 03103 | FLYING HORSE |
| 03104 | FLASH POWER |
| 03201 | LEITE DE SOJA |
| 03301 | LEITE ACHOCOLATADO INDUSTRIALIZADO |
| 03302 | TODDYNHO |
| 03303 | LEITE ACHOCOLATADO TODDYNHO |
| 03304 | ACHOCOLATADO INDUSTRIALIZADO |
| 03401 | CHOCOLATE PREPARADO |
| 03501 | ALMOCO OU JANTAR A LA CARTE |
| 03502 | ALMOCO A LA CARTE |
| 03503 | JANTAR A LA CARTE |
| 03504 | ALMOCO A LA CARTE E BEBIDA |
| 03505 | JANTAR A LA CARTE E BEBIDA |
| 03506 | ALMOCO A LA CARTE E SOBREMESA |
| 03507 | JANTAR A LA CARTE E SOBREMESA |
| 03508 | ALMOCO A LA CARTE, BEBIDA E SOBREMESA |
| 03509 | JANTAR A LA CARTE, BEBIDA E SOBREMESA |
| 03601 | ALMOCO OU JANTAR FAST FOOD |
| 03602 | ALMOCO FAST FOOD |
| 03603 | JANTAR FAST FOOD |
| 03604 | ALMOCO FAST FOOD E BEBIDA |
| 03605 | JANTAR FAST FOOD E BEBIDA |
| 03606 | ALMOCO FAST FOOD E SOBREMESA |
| 03607 | JANTAR FAST FOOD E SOBREMESA |
| 03608 | ALMOCO FAST FOOD, BEBIDA E SOBREMESA |
| 03609 | JANTAR FAST FOOD, BEBIDA E SOBREMESA |
| 03701 | SALGADOS OU TIRA-GOSTO |
| 03702 | PASTEL |
| 03703 | CROQUETE |
| 03704 | BOLINHO SALGADO |
| 03705 | COXINHA |
| 03706 | EMPADA |
| 03707 | RISOLE |
| 03708 | PIZZA EM PEDACO |
| 03709 | TIRA-GOSTO |
| 03710 | PORCAO DE QUEIJO |
| 03711 | ACARAJE |
| 03712 | OVO COZIDO |
| 03713 | CHURRASQUINHO (ESPETINHO) |
| 03714 | PORCAO DE PEIXE FRITO |
| 03715 | BATATA FRITA |
| 03716 | CARANGUEJO |
| 03717 | REQUEIJAO |
| 03718 | PORCAO DE SALAME |
| 03719 | PORCAO DE SALAMINHO |
| 03720 | PORCAO DE CARNE |
| 03721 | AMENDOIN SALGADO |
| 03722 | CASTANHA DE CAJU |
| 03723 | TABUA DE FRIOS |
| 03724 | ESPETINHO DE CARNE |
| 03725 | QUEIJO COALHO |
| 03726 | SALSICHAO |
| 03727 | ENROLADINHO |
| 03728 | QUIBE |
| 03729 | ESFIRRA |
| 03730 | CREPE |
| 03731 | TORTA SALGADA |
| 03732 | ESPIGA DE MILHO ASSADA OU COZIDA |
| 03733 | MILHO ASSADO OU COZIDO |
| 03734 | TUBERCULOS |
| 03735 | BATATA DOCE |
| 03736 | INHAME |
| 03737 | TAPIOCA SALGADA |
| 03738 | ABARA |
| 03739 | ESFIRRA DE CARNE |
| 03740 | ESFIRRA DE QUEIJO |
| 03741 | ESFIRRA DE RICOTA |
| 03742 | ESFIRRA DE FRANGO |
| 03743 | PAO DE QUEIJO |
| 03744 | QUEIJO PRATO |
| 03745 | QUEIJO MINAS FRESCAL |
| 03746 | BARRA DE CEREAL SALGADA |
| 03747 | PIPOCA SALGADA |
| 03801 | ALMOCO OU JANTAR (OUTROS TIPOS) |
| 03802 | ALMOCO OU JANTAR (PRATO FEITO) |
| 03803 | ALMOCO OU JANTAR (QUENTINHA) |
| 03804 | ALMOCO OU JANTAR (MARMITA) |
| 03805 | ALMOCO OU JANTAR (BANDEJAO) |
| 03806 | ANGU A BAIANA |
| 03807 | SOPA DE QUALQUER TIPO |
| 03808 | TACACA |
| 03809 | VATAPA |
| 03901 | SANDUICHE NATURAL |
| 04001 | ALMOCO OU JANTAR NAO-ESPECIFICADO |
| 04101 | LANCHE |
| 04102 | SALGADO E SUCO |
| 04103 | BOLACHA E SUCO |
| 04104 | SANDUICHE E SUCO |
| 04105 | ACARAJE E REFRIGERANTE |
| 04106 | BATATA FRITA E REFRIGERANTE |
| 04107 | SANDUICHE E REFRIGERANTE |
| 04108 | SALGADO E REFRIGERANTE |
| 04109 | BOLO E REFRIGERANTE |
| 04110 | SANDUICHE, BATATA FRITA E REFRIGERANTE |
| 04111 | SANDUICHE, SALGADO E REFRIGERANTE |
| 04112 | SALGADO E CERVEJA |
| 04113 | PORCAO E CERVEJA |
| 04114 | SALGADO E VITAMINA |
| 04115 | SANDUICHE E REFRESCO |
| 04116 | SALGADO E REFRESCO |
| 04201 | CAFE DA MANHA |
| 04202 | CAFE E PAO |
| 04203 | CAFE TIPO COLONIAL |
| 04204 | CAFE E BOLO |
| 04205 | CAFE E BISCOITO |
| 04206 | CAFE E SALGADO |
| 04207 | CAFE DA TARDE |
| 04301 | DOCE OU BOLO |
| 04302 | DOCE |
| 04303 | BOLO |
| 04304 | PIPOCA DOCE |
| 04305 | AMENDOIM DOCE |
| 04306 | TORTA DOCE |
| 04307 | BROA DE MILHO |
| 04308 | SONHO |
| 04309 | RAPADURA |
| 04310 | BROA DE FUBA |
| 04311 | BANANA FRITA COM MEL |
| 04312 | CACAROLA |
| 04313 | CHURRO |
| 04314 | PUDIM |
| 04315 | COCADA |
| 04316 | ALGODAO DOCE |
| 04317 | ARROZ DOCE |
| 04318 | BOLINHO DOCE |
| 04319 | BARRA DE CEREAL DOCE |
| 04320 | MINGAU |
| 04321 | SUCRILHOS |
| 04322 | CUSCUZ |
| 04323 | CANJICA |
| 04324 | MUNGUNZA |
| 04325 | CURAU DE MILHO |
| 04326 | PAMONHA |
| 04327 | TAPIOCA DOCE |
| 04401 | CHOCOLATE EM BARRA, TABLETE OU BOMBOM |
| 04402 | CHOCOLATE EM BARRA |
| 04403 | CHOCOLATE EM TABLETE |
| 04404 | BOMBOM |
| 04405 | KINDER OVO |
| 04501 | SORVETE OU PICOLE |
| 04502 | SORVETE |
| 04503 | PICOLE |
| 04504 | DINDIM (SACOLE) |
| 04505 | SACOLE |
| 04506 | MILK SHAKE |
| 04601 | WHISKY |
| 04701 | VODKA |
| 04801 | VINHO |
| 04901 | CONHAQUE |
| 05001 | REFRIGERANTE DE UVA |
| 05101 | REFRIGERANTE DE LARANJA |
| 05201 | REFRIGERANTE DE LIMAO |
| 05301 | REFRIGERANTE DE TANGERINA |
| 05401 | ALMOCO OU JANTAR (RODIZIO DE CARNES) |
| 05402 | RODIZIO DE CARNES |
| 05501 | ALMOCO OU JANTAR (RODIZIO DE MASSAS) |
| 05502 | RODIZIO DE MASSAS |
| 05601 | ALMOCO OU JANTAR (RODIZIO DE FRUTOS DO MAR ) |
| 05602 | RODIZIO DE FRUTOS DO MAR |
| 05701 | ALMOCO DA ESCOLA |
| 05801 | JANTAR DA ESCOLA |
| 05901 | LANCHE DA ESCOLA |
| 05902 | MERENDA DA ESCOLA |
| 06001 | CAFE DA ESCOLA |
| 06101 | LEITE DE VACA ORGANICO |
| 06201 | QUEIJO DE MINAS FRESCAL ORGANICO |
| 06202 | PORCAO DE QUEIJO DE MINAS FRESCAL ORGANICO |
| 06301 | LEITE DE CABRA ORGANICO |
| 06401 | CAFE ORGANICO |
| 06402 | CAFEZINHO ORGANICO |
| 06501 | FRUTA ORGANICA |
| 06502 | ACEROLA ORGANICA |
| 06503 | UVA ORGANICA |
| 06504 | MACA ORGANICA |
| 06505 | PESSEGO ORGANICO |
| 06506 | MORANGO ORGANICO |
| 06601 | SUCO DE FRUTA OU VEGETAL ORGANICO |
| 06701 | VINHO ORGANICO |
| 06801 | AGUARDENTE ORGANICA |
| 06901 | DOCE DE FRUTA EM CALDA, EM PASTA OU CRISTALIZADO DIET |
| 06902 | DOCE DE FRUTA EM CALDA, EM PASTA OU CRISTALIZADO DIETETICO |
| 06903 | DOCE DE FRUTA EM CALDA DIET |
| 06904 | DOCE DE FRUTA EM CALDA DIETETICO |
| 06905 | DOCE DE FRUTA EM PASTA DIET |
| 06906 | DOCE DE FRUTA EM PASTA DIETETICO |
| 06907 | DOCE DE FRUTA CRISTALIZADO DIET |
| 06908 | DOCE DE FRUTA CRISTALIZADO DIETETICO |
| 07001 | SORVETE DE QUALQUER SABOR DIET |
| 07002 | SORVETE DE QUALQUER SABOR DIETETICO |
| 07003 | MILK SHAKE DIET |
| 07004 | MILK SHAKE DIETETICO |
| 07101 | BALA OU CHICLETE DIET |
| 07102 | CHICLETE DIET |
| 07103 | BALA OU CHICLETE DIETETICO |
| 07104 | CHICLETE DIETETICO |
| 07201 | CHOCOLATE EM BARRA, TABLETE OU BOMBOM DIET |
| 07202 | CHOCOLATE EM BARRA, TABLETE OU BOMBOM DIETETICO |
| 07203 | BARRA DE CHOCOLATE DIET |
| 07204 | BARRA DE CHOCOLATE DIETETICO |
| 07205 | TABLETE DE CHOCOLATE DIET |
| 07206 | TABLETE DE CHOCOLATE DIETETICO |
| 07207 | BOMBOM DIET |
| 07208 | BOMBOM DIETETICO |
| 07209 | OVO DE PASCOA DIET |
| 07210 | OVO DE PASCOA DIETETICO |
| 07301 | PUDIM DIET |
| 07302 | PUDIM DIETETICO |
| 07401 | BARRA DE CEREAIS DIET |
| 07402 | BARRA DE CEREAIS DIETETICA |
| 07501 | SOBREMESA DE QUALQUER SABOR DIET |
| 07502 | SOBREMESA DE QUALQUER SABOR DIETETICA |
| 07601 | DOCE OU BOLO DIETETICO |
| 07602 | MARIA MOLE DIET |
| 07603 | MARIA MOLE DIETETICA |
| 07604 | COCADA DIET |
| 07605 | COCADA DIETETICA |
| 07606 | BRIGADEIRO DIET |
| 07607 | BRIGADEIRO DIETETICO |
| 07608 | BOMBA DE QUALQUER TIPO DIET |
| 07609 | BOMBA DE QUALQUER TIPO DIETETICA |
| 07610 | DOCE DE QUEIJO DIET |
| 07611 | DOCE DE QUEIJO DIETETICO |
| 07612 | DOCE A BASE DE OVOS DIET |
| 07613 | DOCE A BASE DE OVOS DIETETICO |
| 07614 | DOCE A BASE DE LEITE DIET |
| 07615 | DOCE A BASE DE LEITE DIETETICO |
| 07616 | SONHO DIET |
| 07617 | SONHO DIETETICO |
| 07618 | BOLO DE QUALQUER SABOR DIET |
| 07619 | BOLO DE QUALQUER SABOR DIETETICO |
| 07701 | IOGURTE ORGANICO DE QUALQUER SABOR DIET |
| 07702 | IOGURTE ORGANICO DE QUALQUER SABOR DIETETICO |
| 07801 | TORTA DOCE DE QUALQUER SABOR DIET |
| 07802 | TORTA DOCE DE QUALQUER SABOR DIETETICA |
| 07901 | BISCOITO RECHEADO DIET |
| 07902 | BISCOITO RECHEADO DIETETICO |
| 08001 | BISCOITO DOCE DIET |
| 08002 | BISCOITO DOCE DIETETICO |
| 08003 | BOLACHA DOCE DIET |
| 08004 | BOLACHA DOCE DIETETICA |
| 08101 | PAO CARTEIRA DIET |
| 08102 | PAO CARTEIRA DIETETICO |
| 08201 | PAO DE FORMA DIET COM OU SEM MANTEIGA |
| 08202 | PAO DE FORMA DIET |
| 08203 | PAO DE FORMA DIETETICO |
| 08301 | PAO DE HAMBURGER OU CACHORRO QUENTE DIET COM OU SEM MANTEIGA |
| 08302 | PAO DE HAMBURGER DIET COM OU SEM MANTEIGA |
| 08303 | PAO DE HAMBURGER DIET |
| 08304 | PAO DE CACHORRO QUENTE DIET COM OU SEM MANTEIGA |
| 08305 | PAO DE CACHORRO QUENTE DIET |
| 08306 | PAO DE HAMBURGER DIETETICO |
| 08307 | PAO DE CACHORRO QUENTE DIETETICO |
| 08401 | ROSCA DIET |
| 08402 | ROSCA DIETETICA |
| 08501 | CHOCOLATE DIET PREPARADO |
| 08502 | CHOCOLATE DIETETICO PREPARADO |
| 08601 | SUCO DE FRUTA OU VEGETAL DIET |
| 08602 | SUCO DE FRUTA OU VEGETAL DIETETICO |
| 08603 | SUCO DE FRUTA DIET |
| 08604 | SUCO DE FRUTA DIETETICO |
| 08605 | SUCO DE VEGETAL DIET |
| 08606 | SUCO DE VEGETAL DIETETICO |
| 08701 | CAFE CAPUCCINO DIET |
| 08702 | CAFE CAPUCCINO DIETETICO |
| 08801 | CHA MATE DIET PREPARADO |
| 08802 | CHA MATE DIETETICO PREPARADO |
| 08901 | REFRIGERANTE AGUA TONICA DIET |
| 08902 | REFRIGERANTE AGUA TONICA DIETETICO |
| 08903 | AGUA TONICA DIET |
| 08904 | AGUA TONICA DIETETICA |
| 09001 | REFRIGERANTE DE COLA DIET |
| 09002 | REFRIGERANTE PEPSI COLA DIET |
| 09003 | PEPSI COLA DIET |
| 09004 | REFRIGERANTE DE COLA DIETETICO |
| 09005 | REFRIGERANTE PEPSI COLA DIETETICO |
| 09006 | PEPSI COLA DIETETICA |
| 09101 | REFRIGERANTE DE GUARANA DIET |
| 09102 | GUARANA DIET |
| 09103 | REFRIGERANTE DE GUARANA DIETETICO |
| 09104 | GUARANA DIETETICO |
| 09201 | REFRIGERANTE DE LIMAO DIET |
| 09202 | REFRIGERANTE SPRIT DIET |
| 09203 | SPRIT DIET |
| 09204 | REFRIGERANTE DE LIMAO DIETETICO |
| 09205 | REFRIGERANTE SPRIT DIETETICO |
| 09206 | SPRIT DIETETICO |
| 09301 | SORVETE DE QUALQUER SABOR LIGHT |
| 09302 | PICOLE LIGHT |
| 09401 | BALA OU CHICLETE LIGHT |
| 09402 | CHICLETE LIGHT |
| 09501 | CHOCOLATE EM BARRA, TABLETE OU BOMBOM LIGHT |
| 09502 | BARRA DE CHOCOLATE LIGHT |
| 09503 | TABLETE DE CHOCOLATE LIGHT |
| 09504 | BOMBOM LIGHT |
| 09505 | OVO DE PASCOA LIGHT |
| 09601 | DOCE OU BOLO LIGHT |
| 09602 | GELEIA DE FRUTA LIGHT |
| 09603 | MANJAR LIGHT |
| 09604 | GELATINA LIGHT |
| 09605 | TORTA DOCE LIGHT |
| 09606 | TORTA SALGADA LIGHT |
| 09607 | SONHO LIGHT |
| 09608 | BOLO DE QUALQUER SABOR LIGHT |
| 09609 | BOLO LIGHT DE QUALQUER SABOR |
| 09701 | SOBREMESA LIGHT |
| 09801 | ADOCANTE LIGHT |
| 09901 | IOGURTE ORGANICO LIGHT |
| 10001 | QUEIJO MUZZARELA LIGHT |
| 10002 | PORCAO DE QUEIJO MUZZARELA LIGHT |
| 10101 | LEITE DE SOJA LIGHT |
| 10201 | QUEIJO PRATO LIGHT |
| 10202 | PORCAO DE QUEIJO PRATO LIGHT |
| 10301 | LEITE DE CABRA LIGHT |
| 10401 | QUEIJO DE MINAS LIGHT |
| 10402 | PORCAO DE QUEIJO DE MINAS LIGHT |
| 10501 | QUEIJO RICOTA LIGHT |
| 10502 | PORCAO DE QUEIJO RICOTA LIGHT |
| 10601 | QUEIJO POLENGUINHO LIGHT |
| 10701 | OUTROS QUEIJOS LIGHT |
| 10702 | PORCAO DE OUTROS QUEIJOS LIGHT |
| 10801 | COALHADA LIGHT |
| 10901 | PAO DE QUEIJO LIGHT |
| 11001 | PAO INTEGRAL LIGHT COM OU SEM MANTEIGA |
| 11002 | PAO INTEGRAL LIGHT |
| 11101 | TORRADA LIGHT |
| 11201 | SALGADO LIGHT |
| 11202 | PIZZA EM FATIA LIGHT |
| 11203 | LAZANHA EM FATIA LIGHT |
| 11204 | CANELONI EM FATIA LIGHT |
| 11301 | REFEICAO LIGHT |
| 11302 | MASSA LIGHT |
| 11401 | BISCOITO RECHEADO LIGHT |
| 11501 | BISCOITO DOCE LIGHT |
| 11502 | BOLACHA DOCE LIGHT |
| 11601 | BISCOITO SALGADO LIGHT |
| 11602 | BOLACHA SALGADA LIGHT |
| 11701 | PAO CARTEIRA LIGHT |
| 11801 | PAO DE FORMA LIGHT COM OU SEM MANTEIGA |
| 11802 | PAO DE FORMA LIGHT |
| 11901 | PAO DE HAMBURGER OU CACHORRO QUENTE LIGHT COM OU SEM MANTEIGA |
| 11902 | PAO DE HAMBURGER LIGHT COM OU SEM MANTEIGA |
| 11903 | PAO DE HAMBURBER LIGHT |
| 11904 | PAO DE CACHORRO QUENTE LIGHT COM OU SEM MANTEIGA |
| 11905 | PAO DE CACHORRO QUENTE LIGHT |
| 12001 | ROSCA LIGHT |
| 12101 | PAO ARABE LIGHT |
| 12102 | PAO SIRIO LIGHT |
| 12201 | MORTADELA LIGHT |
| 12301 | SALAME LIGHT |
| 12401 | CHOCOLATE LIGHT PREPARADO |
| 12501 | SUCO DE FRUTA LIGHT |
| 12601 | CAFE CAPUCCINO LIGHT |
| 12701 | CHA PRETO LIGHT PREPARADO |
| 12801 | CHA MATE LIGHT PREPARADO |
| 12901 | REFRIGERANTE DE COLA LIGHT |
| 12902 | REFRIGERANTE COCA COLA LIGHT |
| 12903 | COCA COLA LIGHT |
| 13001 | REFRIGERANTE DE LARANJA LIGHT |
| 13002 | REFRIGERANTE FANTA LARANJA LIGHT |
| 13003 | FANTA LARANJA LIGHT |
| 13004 | REFRIGERANTE CRUSH LIGHT |
| 13005 | CRUSH LIGHT |
| 13006 | REFRIGERANTE MIRINDA LIGHT |
| 13007 | MIRINDA LIGHT |
| 13008 | REFRIGERANTE SUKITA LIGHT |
| 13009 | SUKITA LIGHT |
| 13010 | REFRIGERANTE POP LARANJA LIGHT |
| 13011 | POP LARANJA LIGHT |
| 13101 | REFRIGERANTE DE GUARANA LIGHT |
| 13102 | REFRIGERANTE GUARANA LIGHT |
| 13103 | GUARANA LIGHT |
| 13104 | REFRIGERANTE FLATELLY VITA LIGHT |
| 13105 | FLATELLY VITA LIGHT |
| 13106 | REFRIGERANTE XODO DA BAHIA LIGHT |
| 13107 | XODO DA BAHIA LIGHT |
| 13108 | REFRIGERANTE TUBAINA LIGHT |
| 13109 | TUBAINA LIGHT |
| 13201 | REFRIGERANTE DE MACA LIGHT |
| 13202 | REFRIGERANTE FANTA MACA LIGHT |
| 13203 | FANTA MACA LIGHT |
| 13301 | REFRIGERANTE DE LIMAO LIGHT |
| 13302 | REFRIGERANTE SODA LIMONADA LIGHT |
| 13303 | SODA LIMONADA LIGHT |
| 13304 | REFRIGERANTE FANTA LIMAO LIGHT |
| 13305 | FANTA LIMAO LIGHT |
| 13306 | REFRIGERANTE GASOSA LIGHT |
| 13307 | GASOSA LIGHT |
| 13401 | REFRIGERANTE DE UVA LIGHT |
| 13402 | REFRIGERANTE FANTA UVA LIGHT |
| 13403 | FANTA UVA LIGHT |
| 13404 | REFRIGERANTE GRAPETTE LIGHT |
| 13405 | GRAPETTE LIGHT |
| 13501 | REFRIGERANTES DE OUTROS SABORES LIGHT |
| 13502 | REFRIGERANTE MINUANO LIGHT |
| 13503 | MINUANO LIGHT |
| 13504 | REFRIGERANTE DE MATE LIGHT |
| 13505 | REFRIGERANTE MATE COURO LIGHT |
| 13506 | MATE COURO LIGHT |
| 13507 | REFRIGERANTE BIDU LIGHT |
| 13508 | BIDU LIGHT |
| 13509 | REFRIGERANTE JAO LIGHT |
| 13510 | JAO LIGHT |
| 13511 | REFRIGERANTE ALOA LIGHT |
| 13512 | ALOA LIGHT |
| 13513 | REFRIGERANTE DE TUTI FRUTI LIGHT |
| 13514 | REFRIGERANTE DE TANGERINA LIGHT |
| 13515 | REFRIGERANTE DE CAJU LIGHT |
| 13516 | REFRIGERANTE PARAGUAI LIGHT |
| 13517 | PARAGUAI LIGHT |
| 13518 | REFRIGERANTE GOIANINHA LIGHT |
| 13519 | GOIANINHA LIGHT |
| 13520 | REFRIGERANTE REAL LIGHT |
| 13521 | REAL LIGHT |
| 13522 | REFRIGERANTE CITRICO LIGHT |
| 13523 | REFRIGERANTE FANTA CITRUS LIGHT |
| 13524 | FANTA CITRUS LIGHT |
| 13525 | REFRIGERANTE DE ABACAXI LIGHT |
| 13526 | REFRIGERANTE DE FRAMBOESA LIGHT |
| 13527 | REFRIGERANTE DE MORANGO LIGHT |
| 13528 | REFRIGERANTE DE MARACUJA LIGHT |
| 13529 | REFRIGERANTE DE GENGIBRE LIGHT |
| 13601 | CERVEJA LIGHT |
| 13701 | CERVEJA SEM ALCOOL LIGHT |
| 13801 | DOCE DE FRUTA EM CALDA, EM PASTA OU CRISTALIZADO LIGHT |
| 13802 | DOCE DE FRUTA EM CALDA LIGHT |
| 13803 | DOCE DE FRUTA EM PASTA LIGHT |
| 13804 | DOCE DE FRUTA CRISTALIZADO LIGHT |
| 13901 | REFRIGERANTE NAO ESPECIFICADO |
| 99901 | AGREGADO |
| 00101 | CIGARRO |
| 00201 | ISQUEIRO DESCARTAVEL |
| 00301 | CHARUTO |
| 00401 | CIGARRILHA |
| 00501 | FUMO DESFIADO |
| 00502 | FUMO DESFIADO PARA CIGARRO |
| 00503 | FUMO DESFIADO PARA CACHIMBO |
| 00601 | FOSFORO (FUMANTE) |
| 00602 | FOSFORO |
| 00701 | PITEIRA |
| 00801 | FLUIDO DE ISQUEIRO |
| 00901 | GAS DE ISQUEIRO |
| 01001 | PEDRA DE ISQUEIRO |
| 01101 | FUMO DE ROLO |
| 01201 | RAPE |
| 01301 | CIGARRO DE PALHA |
| 01401 | PALHA PARA CIGARRO |
| 01501 | PAPEL DE CIGARRO |
| 01601 | MACONHA |
| 01602 | CIGARRO DE MACONHA |
| 01701 | COCAINA |
| 01801 | LANCA-PERFUME |
| 99901 | AGREGADO |
| 00101 | LOTECA |
| 00102 | LOTERIA ESPORTIVA |
| 00201 | LOTERIA FEDERAL E ESTADUAL |
| 00202 | BILHETE DE LOTERIA FEDERAL |
| 00203 | BILHETE DE LOTERIA ESTADUAL |
| 00301 | LOTOMANIA |
| 00401 | JOGO DO BICHO |
| 00501 | CORRIDA DE CAVALOS |
| 00601 | BRIGA DE GALOS |
| 00701 | JOGO DE CARTAS (APOSTA) |
| 00801 | OUTROS JOGOS E APOSTAS |
| 00802 | SINUCA (APOSTA) |
| 00804 | APOSTAS EM JOGO DE FUTEBOL |
| 00806 | BOLAO |
| 00807 | JOGO DE DOMINO (APOSTA) |
| 00901 | RIFA |
| 01001 | BINGO |
| 01101 | LOTOFACIL |
| 01201 | LOTERIA INSTANTANEA |
| 01301 | TELESENA |
| 01401 | RASPADINHA |
| 01501 | MAQUINA DE CACA NIQUEL |
| 01601 | QUINA |
| 01701 | DUPLA SENA |
| 01801 | MEGA SENA |
| 02001 | LOTOGOL |
| 02201 | CARUARU DA SORTE |
| 02401 | LOTINHA |
| 02701 | RIO DE PREMIOS |
| 02901 | TOTOLEC |
| 03001 | TRIMANIA |
| 99901 | AGREGADO |
| 00101 | JORNAL |
| 00201 | REVISTA INFANTIL |
| 00301 | FOTONOVELA |
| 00401 | REVISTA ESPORTIVA |
| 00501 | REVISTA DE MODA |
| 00601 | REVISTA MASCULINA |
| 00701 | REVISTA DE CULINARIA |
| 00801 | REVISTA INFORMATIVA |
| 00901 | REVISTA DE INFORMATICA |
| 01001 | REVISTA FEMININA |
| 01101 | REVISTA TEEN |
| 01201 | REVISTA DE CASA E DECORACAO |
| 01301 | REVISTA DE TV |
| 01401 | REVISTA DE ANIMAIS |
| 01501 | REVISTA DE CARROS |
| 01601 | REVISTA DE SAUDE |
| 01701 | ALBUM E FIGURINHAS |
| 01702 | FIGURINHAS DE ALBUM |
| 01801 | FOLHETO |
| 01802 | FOLHETO MUSICAL |
| 01901 | CRUZADINHA |
| 01902 | PALAVRA CRUZADA |
| 02001 | REVISTA DE ARTESANATO |
| 02101 | REVISTA RELIGIOSA |
| 99901 | AGREGADO |
| 00101 | CINEMA (INGRESSO) |
| 00201 | TEATRO (INGRESSO) |
| 00301 | FUTEBOL (INGRESSO) |
| 00401 | OUTROS EVENTOS ESPORTIVOS (INGRESSO) |
| 00402 | BASQUETEBOL (INGRESSO) |
| 00403 | FUTEBOL DE SALAO (INGRESSO) |
| 00404 | CORRIDA AUTOMOBILISTICA (INGRESSO) |
| 00405 | TENIS (INGRESSO) |
| 00406 | VOLEIBOL (INGRESSO) |
| 00501 | CLUBE (MENSALIDADE E TAXA) |
| 00502 | VIDEOCLUBE (TAXA) |
| 00503 | ASSOCIACAO RECREATIVA (MENSALIDADE E TAXA) |
| 00504 | MENSALIDADE DE CLUBE |
| 00505 | TAXA DE CLUBE |
| 00601 | DISCO DE VINIL |
| 00701 | FILME E FLASH DESCARTAVEL |
| 00702 | FLASH DESCARTAVEL |
| 00801 | REVELACAO E COPIA |
| 00802 | COPIA DE FILME |
| 00803 | REVELACAO DE FILME |
| 00901 | ALUGUEL DE FITA DE VIDEOCASSETE |
| 01001 | FITA DE VIDEOCASSETE GRAVADA |
| 01101 | SHOW (INGRESSO) |
| 01201 | PARQUE DE DIVERSOES (INGRESSO) |
| 01202 | PESCARIA EM PARQUE DE DIVERSOES |
| 01301 | JARDIM ZOOLOGICO (INGRESSO) |
| 01401 | MUSEU (INGRESSO) |
| 01501 | JOGOS ELETRONICOS (FICHA) |
| 01502 | FLIPERAMA (FICHA) |
| 01503 | VIDEOGAME (FICHA) |
| 01504 | VIDEOGAME (ALUGUEL DA HORA) |
| 01601 | LOCACAO DE CHARRETE (PASSEIO) |
| 01602 | LOCACAO DE CAVALO (PASSEIO) |
| 01603 | ALUGUEL DE CHARRETE (PASSEIO) |
| 01604 | ALUGUEL DE CAVALO (PASSEIO) |
| 01605 | ALUGUEL DE BICICLETA (PASSEIO) |
| 01606 | LOCACAO DE BICICLETA (PASSEIO) |
| 01607 | ALUGUEL DE BOTE (PASSEIO) |
| 01608 | LOCACAO DE BOTE (PASSEIO) |
| 01701 | ALUGUEL DE QUADRA E CAMPO |
| 01702 | ALUGUEL DE QUADRA |
| 01703 | ALUGUEL DE CAMPO |
| 01801 | EXPOSICAO OU FEIRA (INGRESSO) |
| 01802 | FEIRA DE INFORMATICA (INGRESSO) |
| 01901 | CIRCO (INGRESSO) |
| 02001 | BOITE, DANCETERIA, DISCOTECA, ETC. |
| 02002 | DANCETERIA (INGRESSO) |
| 02003 | DISCOTECA (INGRESSO) |
| 02004 | KARAOKE (INGRESSO) |
| 02005 | FORRO (INGRESSO) |
| 02006 | BOITE (INGRESSO) |
| 02007 | BAR (COUVERT ARTISTICO) |
| 02008 | VIDEOKE (INGRESSO) |
| 02101 | SINUCA,TOTO E BOLICHE |
| 02102 | TOTO |
| 02103 | BOLICHE |
| 02104 | SINUCA |
| 02105 | PEBOLIM |
| 02201 | FOGOS E ARTEFATOS JUNINOS |
| 02301 | CARTAO DE TELEFONE CELULAR |
| 02401 | CONTA DE TELEFONE CELULAR |
| 02402 | CONTA DE APARELHO NEXTEL |
| 02501 | MOTEL |
| 02601 | ALUGUEL DE DVD (DISCO) |
| 02701 | PONTOS TURISTICOS (INGRESSO) |
| 02801 | ALUGUEL DE CLUBE, TEATRO, ETC. PARA DIVERSAO |
| 02802 | LOCACAO DE CLUBE, TEATRO, ETC. PARA DIVERSAO |
| 02901 | PROSTITUTA |
| 03001 | PIQUENIQUE (TAXA) |
| 03002 | TAXA DE PIQUENIQUE |
| 03101 | TAXA DE SALTO (PARAQUEDISMO) |
| 03102 | PARAQUEDISMO |
| 03201 | PLANETARIO (INGRESSO) |
| 03301 | HIDRELETRICA (INGRESSO) |
| 03401 | PISCINA EM PARQUE, RESERVA, ETC. |
| 03501 | RINHA (INGRESSO) |
| 03601 | RODEIO (INGRESSO) |
| 03701 | CARREGADOR DE TACOS DE GOLFE |
| 03801 | SERESTA (INGRESSO) |
| 03901 | ALUGUEL DE BRINQUEDO ELETRICO OU ELETRONICO |
| 04001 | CD (DISCO) |
| 04002 | DISCO LASER |
| 04003 | CD DE VIDEOGAME (DISCO) |
| 04004 | CD DE MUSICA (DISCO) |
| 04005 | CD-ROM (DISCO) |
| 04101 | FITA DE VIDEOGAME |
| 04201 | ALUGUEL DE FITA DE VIDEOGAME |
| 04301 | FITA CASSETE GRAVADA |
| 04401 | FITA CASSETE VIRGEM |
| 04501 | FITA DE VIDEOCASSETE VIRGEM |
| 04601 | OPERA (INGRESSO) |
| 04701 | FITA DE FILMADORA |
| 04801 | DISQUETE PARA COMPUTADOR |
| 04901 | PISTA DE KART (INGRESSO) |
| 05001 | HOTEL |
| 05101 | PESQUE-PAGUE (INGRESSO) |
| 05102 | TAXA DE PESCA |
| 05103 | PESCA PAGA |
| 05201 | ACESSORIOS DE IMPRESSORA |
| 05202 | CARTUCHO DE IMPRESSORA DE COMPUTADOR |
| 05203 | RECARGA DE CARTUCHO PARA IMPRESSORA |
| 05301 | TITULO DE TURISMO (MENSALIDADE) |
| 05302 | MENSALIDADE DE TITULO DE TURISMO |
| 05401 | ALUGUEL DE BRINQUEDO NAO-ELETRICO OU NAO-ELETRONICO |
| 05501 | ACADEMIA |
| 05502 | ACADEMIA DE GINASTICA |
| 05503 | ACADEMIA DE MUSCULACAO |
| 05601 | BAILE E FESTA EM CLUBE (INGRESSO) |
| 05701 | DVD (DISCO) |
| 05801 | ALUGUEL DE CADEIRA DE PRAIA |
| 05901 | DANCA CLASSICA (INGRESSO) |
| 99901 | AGREGADO |
| 00101 | PARA DOR E FEBRE (ANALGESICO E ANTITERMICO) |
| 00102 | ANALGESICO E ANTITERMICO |
| 00103 | ANTITERMICO |
| 00104 | PARA ENXAQUECA |
| 00105 | ANALGESICO |
| 00106 | ANALGESICO E ANTIINFLAMATORIO |
| 00201 | PARA TOSSE E RESFRIADO (ANTIGRIPAL E ANTITUSSIGENO) |
| 00202 | ANTITUSSIGENO |
| 00203 | ANTIGRIPAL |
| 00204 | ANTIGRIPAL E ANALGESICO |
| 00205 | ANTIGRIPAL E ANTITERMICO |
| 00206 | ANTIGRIPAL HOMEOPATICO |
| 00207 | EXPECTORANTE (XAROPE) |
| 00208 | MEL MEDICINAL (QUALQUER TIPO) |
| 00209 | ANTIGRIPAL E ANTITUSSIGENO |
| 00301 | PARA ALERGIA (ANTIALERGICO) |
| 00302 | ANTIALERGICO HOMEOPATICO |
| 00303 | CREME ANTIALERGICO |
| 00304 | ANTIALERGICO |
| 00305 | SABONETE ANTIALERGICO |
| 00401 | PARA AZIA (ANTIACIDO) |
| 00402 | ANTI-REFLUXIVO GASTRICO |
| 00403 | PARA REFLUXO GASTRICO |
| 00404 | ANTIACIDO |
| 00501 | PARA ENJOO E VOMITO (ANTIEMETICO) |
| 00502 | PARA VOMITO |
| 00503 | PARA ENJOO |
| 00601 | PARA PRISAO DE VENTRE (LAXANTE) |
| 00602 | LAXANTE E PURGATIVO |
| 00603 | PURGATIVO |
| 00604 | PARA INTESTINO |
| 00605 | INTESTINAL |
| 00606 | LAXANTE |
| 00607 | OLEO (LAXATIVO) |
| 00701 | PARA INFLAMACAO E REUMATISMO (ANTIINFLAMATORIO E ANTI-REUMATICO) |
| 00702 | ANTIINFLAMATORIO E ANTI-REUMATICO |
| 00703 | ANTI-REUMATICO |
| 00704 | PARA REUMATISMO |
| 00705 | ANTIINFLAMATORIO |
| 00706 | CORTICOIDE |
| 00801 | PARA INFECCAO |
| 00802 | ANTIINFECCIOSO |
| 00803 | ANTIBIOTICO |
| 00804 | ANTIBACTERIANO |
| 00805 | ANTIINFECCIOSO E ANTIINFLAMATORIO |
| 00806 | ANTIVIROTICO |
| 00807 | PARA HERPES |
| 00901 | PARA DIABETE |
| 01001 | PARA PRESSAO ALTA (ANTI-HIPERTENSIVO) |
| 01002 | VASODILATADOR/PRESSAO ARTERIAL |
| 01003 | PARA PRESSAO ARTERIAL |
| 01101 | PARA PROBLEMAS CARDIACOS E CIRCULATORIOS |
| 01102 | PARA INSUFICIENCIA CARDIOVASCULAR |
| 01103 | PARA O CORACAO (INSUFICIENCIA CARDIOVASCULAR) |
| 01201 | REDUTOR DE COLESTEROL OU TRIGLICERIDEOS |
| 01202 | PARA COLESTEROL |
| 01203 | REDUTOR DE COLESTEROL |
| 01204 | PARA TRIGLICERIDEO |
| 01205 | REDUTOR DE TRIGLICERIDEOS |
| 01301 | PARA ASMA E BRONQUITE |
| 01302 | PARA ASMA |
| 01303 | PARA BRONQUITE |
| 01304 | PARA NEBULIZACAO |
| 01305 | BRONCODILATADORES |
| 01401 | PARA ANEMIA |
| 01402 | ANTIANEMICO |
| 01501 | PARA VERMES (VERMIFUGO) |
| 01502 | ANTI-HELMINTICO (VERMIFUGO) |
| 01503 | PARA VERMES |
| 01504 | VERMIFUGO |
| 01505 | ANTIHELMINTICO |
| 01506 | PARA PROTOZOARIOS |
| 01601 | PARA OS OSSOS E ARTICULACOES |
| 01602 | PARA OSTEOPOROSE |
| 01603 | ANTIOSTEOPOROSE |
| 01604 | CALCIO PARA OSTEOPOROSE |
| 01605 | CALCIO (CALCIFICANTE OSSEO) |
| 01606 | PARA OS OSSOS |
| 01607 | PARA AS ARTICULACOES |
| 01608 | ANTI OSTEOPENIA |
| 01701 | PARA DEPRESSAO (ANTIDEPRESSIVO) |
| 01702 | ANTI-DEPRESSIVO |
| 01801 | CALMANTE |
| 01802 | PARA ESTRESSE |
| 01803 | PARA STRESS |
| 01804 | ANSIOLITICO |
| 01805 | CALMANTE NATURAL |
| 01806 | SONIFERO |
| 01901 | MODERADOR DE APETITE |
| 01902 | CONTROLADOR DE APETITE |
| 01903 | EMAGRECEDOR |
| 01904 | PARA EMAGRECER |
| 02001 | VITAMINAS |
| 02002 | VITAMINA B12 |
| 02003 | COMPLEXO B |
| 02004 | COMPLEXO VITAMINICO |
| 02005 | VITAMINA C |
| 02101 | ANTICONCEPCIONAL |
| 02201 | HORMONIO |
| 02202 | ADESIVOS INTRADERMICOS (MENOPAUSA) |
| 02203 | FITO-HORMONIOS |
| 02204 | PARA MENOPAUSA |
| 02301 | PARA O FIGADO PANCREAS E VIAS BILIARES |
| 02302 | PARA O FIGADO (HEPATICO) |
| 02303 | HEPATICO (PARA O FIGADO) |
| 02304 | PARA HEPATITE |
| 02305 | PANCREATICO |
| 02306 | PARA O PANCREAS |
| 02307 | PARA VESICULA |
| 02401 | PARA OS RINS |
| 02402 | RENAL (PARA OS RINS) |
| 02403 | DIURETICO |
| 02501 | PARA O SISTEMA NERVOSO |
| 02502 | ANTIEPILEPTICO |
| 02503 | ANTICONVULSIVO |
| 02504 | PARA CONVULSAO |
| 02505 | PARA EPILEPSIA |
| 02506 | ANTIEPILETICO |
| 02507 | ANTIPSICOTICO |
| 02508 | PARA ALZHEIMER |
| 02509 | PARA DERRAME CEREBRAL |
| 02510 | ANTIPARKSONIANO |
| 02511 | PARA O MAL DE PARKINSON |
| 02512 | PARA SISTEMA NERVOSO |
| 02513 | NEUROLOGICO |
| 02514 | PARA MEMORIA |
| 02515 | PARA TONTURA |
| 02516 | PARA VERTIGEM |
| 02517 | PARA CIRCULACAO |
| 02518 | PARA PSICOSE |
| 02601 | PARA PROBLEMAS DE PELE |
| 02602 | DERMATOLOGICO |
| 02603 | PARA MICOSE (DERMATOLOGICO) |
| 02604 | ANTIFUNGICO |
| 02605 | ANTIMICOTICO |
| 02606 | CREME ANTIMICOTICO |
| 02607 | CREME DERMATOLOGICO |
| 02608 | CREME PARA O CORPO (MAO, PE, ETC.) |
| 02609 | CREME PARA QUEIMADURAS |
| 02610 | OLEO DERMATOLOGICO |
| 02611 | POMADA INDETERMINADA |
| 02612 | CICATRIZANTE |
| 02613 | CREME CICATRIZANTE |
| 02614 | PARA LUPUS |
| 02615 | LOCAO DERMATOLOGICA |
| 02616 | VASELINA |
| 02627 | OLEO PARA ASSADURA |
| 02628 | PARA ASSADURA |
| 02629 | CREME PARA ASSADURA |
| 02701 | PARA PROBLEMAS DE ESTOMAGO |
| 02702 | DIGESTIVO |
| 02703 | PARA GASTRITE |
| 02704 | PARA ESTOMAGO |
| 02801 | PARA PROBLEMAS GINECOLOGICOS |
| 02802 | CREME VAGINAL |
| 02803 | REMEDIO GINECOLOGICO |
| 02804 | POMADA VAGINAL |
| 02805 | ANTIABORTIVO |
| 02901 | PARA PROBLEMAS OCULARES (OFTALMOLOGICOS) |
| 02902 | COLIRIOS |
| 02903 | SOLUCAO OFTALMICA (PARA A VISTA) |
| 03001 | PARA DIARREIA |
| 03002 | ANTIDIARREICO |
| 03101 | PARA GASES E COLICA |
| 03102 | ANTIESPASMODICO |
| 03103 | ANTICOLICA |
| 03104 | OLEO DOCE (PARA GASES) |
| 03201 | FORTIFICANTES E ESTIMULANTES DO APETITE |
| 03202 | FORTIFICANTE |
| 03203 | OLEO FORTIFICANTE |
| 03204 | ESTIMULANTE DO APETITE |
| 03205 | COMPLEMENTO ALIMENTAR |
| 03206 | AMINOACIDO |
| 03301 | ESTIMULANTE SEXUAL |
| 03401 | PARA VARIZES |
| 03402 | REMEDIO PARA VARIZES |
| 03403 | PARA HEMORROIDAS |
| 03501 | PARA AIDS |
| 03502 | TRATAMENTO DE AIDS |
| 03601 | PARA PROBLEMAS DE BOCA, OUVIDO, NARIZ E GARGANTA |
| 03602 | OTOLOGICOS |
| 03603 | PASTILHA PARA GARGANTA |
| 03604 | PARA SINUSITE (ANTIINFLAMATORIO) |
| 03605 | PARA LABIRINTITE (ANTIINFLAMATORIO) |
| 03606 | PARA INFECCOES DA BOCA E GARGANTA |
| 03607 | PARA AFTA |
| 03608 | DESCONGESTIONANTE NASAL |
| 03609 | DESCONGESTIONANTE NASAL HOMEOPATICO |
| 03701 | PARA TIREOIDE |
| 03702 | ANTITIREOIDISMO |
| 03703 | PARA TIROIDE |
| 03801 | CHAS E REMEDIOS ALTERNATIVOS |
| 03802 | CHA DE QUALQUER TIPO |
| 03803 | EXTRATOS DE QUALQUER TIPO |
| 03804 | GARRAFADA DE QUALQUER TIPO |
| 03805 | GARRAFADA DE QUALQUER TRATAMENTO |
| 03806 | FLORAIS |
| 03807 | AGUA MEDICINAL |
| 03901 | CONTRASTES RADIOLOGICOS |
| 03902 | LIQUIDO PARA CONTRASTE (EXAME) |
| 03903 | LIQUIDO PARA UROGRAFIA (EXAME) |
| 04001 | COMBATE AO ALCOOLISMO E TABAGISMO |
| 04002 | ADESIVOS ANTITABAGISMO |
| 04003 | ANTITABAGISMO |
| 04004 | PARA TRATAMENTO DO ALCOOLISMO |
| 04101 | PARA DOENCAS INFECCIOSAS E ENDEMICAS |
| 04102 | PARA DOENCA DE CHAGAS |
| 04103 | PARA MALARIA |
| 04104 | PARA O PULMAO |
| 04105 | PARA TUBERCULOSE |
| 04106 | ANTI-HANSENIASE |
| 04201 | PARA PROBLEMAS DE COAGULACAO |
| 04202 | ANTICOAGULANTE |
| 04203 | COAGULANTE |
| 04204 | ANTI-HEMORRAGICO |
| 04301 | SOLUCOES HIDRATANTES |
| 04302 | REPOSITOR ELETROLITICO (CLORETO DE POTASSIO, SODIO, ETC.) |
| 04303 | SORO FISIOLOGICO HIDRATANTE |
| 04401 | PARA PROBLEMAS MUSCULARES |
| 04402 | ANESTESICO |
| 04403 | PARA COLUNA |
| 04404 | PARA DORES MUSCULARES |
| 04405 | RELAXANTE MUSCULAR |
| 04406 | PARA CONTUSAO (ANALGESICO) |
| 04407 | CANFORA |
| 04501 | ECTOPARASITOSE (SARNA, PIOLHO, ETC.) |
| 04502 | PARA PIOLHO |
| 04503 | SABONETE ANTI-PIOLHO |
| 04504 | REMEDIO PARA PIOLHO (SABONETE) |
| 04505 | PARA SARNA |
| 04601 | PARA PROSTATA E VIAS URINARIAS |
| 04602 | PARA PROSTATA |
| 04603 | UROLOGICO |
| 04701 | PARA CANCER |
| 04702 | ANTINEOPLASICO (TRATAMENTO DE CANCER) |
| 04703 | SEDATIVO (MORFINA) |
| 04704 | QUIMIOTERAPICO |
| 04705 | PARA QUIMIOTERAPIA |
| 04801 | VACINAS |
| 04802 | VACINA BCG |
| 04803 | VACINA ANTITETANICA |
| 04804 | VACINA DE HEPATITE B |
| 04805 | VACINA PARA OSTEOPOROSE |
| 04806 | VACINA ANTIPOLIO |
| 04807 | VACINA TRIPLICE |
| 04808 | VACINA ANTIOFIDICO |
| 04901 | MEDICAMENTO PARA GENGIVA DE BEBE |
| 30001 | REMEDIO INDETERMINADO |
| 30002 | SUPOSITORIO |
| 30003 | ADESIVOS |
| 30004 | DEPURATIVO SANGUINEO |
| 30005 | DEPURADOR SANGUINEO |
| 30006 | DEPURADOR DO SANGUE |
| 30007 | DEPURATIVO DO SANGUE |
| 30008 | ENXOFRE |
| 30009 | AMONIA |
| 30010 | REMEDIO HOMEOPATICO INDETERMINADO |
| 30011 | REMEDIO ORTOMOLECULAR |
| 30012 | REMEDIO MANIPULADO INDETERMINADO |
| 30013 | INJECAO AMPOLA (NAO-DETERMINADA) |
| 30101 | MATERIAL PARA CURATIVO |
| 30102 | ESPARADRAPO |
| 30103 | GAZE |
| 30104 | ALGODAO |
| 30105 | ATADURA |
| 30106 | FITA ADESIVA PARA CURATIVO |
| 30107 | LUVA CIRURGICA |
| 30201 | ANTI-SEPTICOS E DESINFETANTES TOPICOS |
| 30202 | ANTI-SEPTICO |
| 30203 | DESINFETANTE FARMACEUTICO |
| 30204 | MERCURIO CROMO |
| 30205 | AGUA OXIGENADA |
| 30206 | TINTURA DE ROMA |
| 30207 | VIOLETA GENCIANA |
| 30208 | MERTHIOLATE |
| 30209 | AGUA DESTILADA |
| 30210 | ALCOOL (PARA DESINFECCAO) |
| 30211 | ALCOOL IODADO |
| 30212 | ETER |
| 30213 | AGUA BORICADA |
| 30301 | MATERIAL DE INJECAO |
| 30302 | AGULHA DE INJECAO (MEDICAO DE GLICOSE) |
| 30303 | SERINGA |
| 30304 | GARROTE DE BORRACHA |
| 30305 | AGULHA DE INJECAO (DESCARTAVEL) |
| 30401 | MATERIAL PARA CUIDADO COM BEBE |
| 30402 | CHUPETA OU BICO |
| 30403 | BICO |
| 30404 | CHUCA |
| 30405 | MORDEDOR DE BEBE |
| 30406 | BORRACHA DE MORDER |
| 30407 | BORRACHA DE DENTICAO DE BEBE |
| 30408 | MORDEDOR DE DENTICAO DE BEBE |
| 30416 | MAMADEIRA |
| 30501 | MATERIAL PARA AUTODIAGNOSTICO |
| 30502 | TERMOMETRO |
| 30503 | UTENSILIO PARA COLETAR FEZES E URINA |
| 30504 | RECIPIENTE PARA COLETAR FEZES E URINA |
| 30505 | TESTE DE GRAVIDEZ |
| 30506 | FITA PARA MEDICAO DE GLICOSE |
| 30507 | APARELHO DE MEDIDOR DE GLICOSE |
| 30508 | APARELHO PARA MEDIR PRESSAO |
| 30601 | MATERIAL PARA HIGIENE DE LENTE DE CONTATO |
| 30602 | SOLUCAO DE LENTE DE CONTACTO |
| 30603 | SORO FISIOLOGICO PARA LENTE DE CONTATO |
| 30604 | LUBRIFICANTE PARA LENTE DE CONTATO |
| 30605 | LIQUIDO PARA LENTES DE CONTATO |
| 30701 | SABONETE E SHAMPU MEDICINAL |
| 30702 | SHAMPU MEDICINAL |
| 30703 | REMEDIO PARA CABELOS (SHAMPU E OUTROS) |
| 30704 | REMEDIO PARA QUEDA DE CABELOS (SHAMPU, CREME E OUTROS) |
| 30705 | SABONETE MEDICINAL |
| 30706 | ANTI-CASPA |
| 30801 | PRODUTOS PARA CUIDADOS BUCAIS E DENTAIS |
| 30802 | CREME DENTAL MEDICINAL |
| 30803 | MANTEIGA DE CACAU |
| 30804 | BALA DE GENGIBRE |
| 30806 | ODONTOLOGICO |
| 30807 | FIXADOR DE DENTADURA |
| 30901 | MATERIAL PARA APOIO TERAPEUTICO |
| 30902 | SONDA (QUALQUER TIPO) |
| 30903 | BOLSA DE AGUA QUENTE |
| 30904 | BOLSA TERMICA |
| 30905 | BOMBA PARA INALACAO |
| 30906 | BOMBA PARA HIGIENE NASAL |
| 30907 | EQUIPAMENTO DE MASSAGEM |
| 30908 | BOMBA DE TIRAR LEITE HUMANO |
| 30909 | PROTETOR DE SEIO |
| 31001 | PRESERVATIVO E LUBRIFICANTE INTIMO |
| 31002 | PRESERVATIVO MASCULINO |
| 31003 | CAMISINHA (PRESERVATIVO MASCULINO) |
| 31004 | PRESERVATIVO FEMININO |
| 31005 | LUBRIFICANTE INTIMO |
| 31006 | DIU |
| 31101 | PRODUTOS PARA MASSAGEM E ESTETICA |
| 31102 | IODO, SALICILATO (PARA MASSAGEM) |
| 31103 | SALICILATO, IODO (PARA MASSAGEM) |
| 31104 | OLEO PARA MASSAGEM |
| 31105 | GEL REDUTOR DE GORDURA |
| 31106 | REDUTOR DE GORDURA (GEL) |
| 31107 | GEL REDUTOR DE CELULITE |
| 31108 | REDUTOR DE CELULITE (GEL) |
| 31109 | REMEDIO MODELADOR DE CORPO (GEL) |
| 31110 | MODELADOR DE CORPO (GEL) |
| 31111 | OLEO INDETERMINADO |
| 31112 | GEL MASSAGEADOR |
| 31201 | MATERIAIS PARA CUIDADOS COM IDOSOS |
| 31202 | FRALDA GERIATRICA |
| 31203 | ABSORVENTE GERIATRICO |
| 31301 | APLICACAO DE INJECAO |
| 31401 | FRALDA DESCARTAVEL |
| 99901 | AGREGADO |
| 00101 | ESCOVA DE CABELOS E PENTE |
| 00102 | PENTE |
| 00103 | ESCOVA DE CABELO |
| 00201 | PERFUME |
| 00202 | AGUA DE COLONIA |
| 00203 | LAVANDA |
| 00206 | COLONIA |
| 00301 | TALCO |
| 00401 | ARTIGOS DE MAQUIAGEM |
| 00402 | CILIO POSTICO |
| 00403 | UNHA POSTICA |
| 00404 | PO COMPACTO |
| 00405 | PO DE ARROZ |
| 00406 | LAPIS PARA OLHOS |
| 00407 | APONTADOR DE LAPIS PARA OS OLHOS |
| 00408 | BATOM |
| 00409 | DELINEADOR |
| 00501 | CREME DE PELE E BRONZEADOR |
| 00502 | BRONZEADOR |
| 00503 | LOCAO DE PELE |
| 00504 | MASCARA DE BELEZA |
| 00505 | CREME PARA MAOS |
| 00506 | OLEO PARA PELE |
| 00507 | CREME HIDRATANTE |
| 00508 | HIDRATANTE (CREME) |
| 00509 | PROTETOR LABIAL |
| 00510 | PROTETOR SOLAR |
| 00511 | LEITE DE COLONIA |
| 00512 | BLOQUEADOR SOLAR |
| 00513 | OLEO DE AMENDOA |
| 00514 | CREME ANTI-RUGAS |
| 00515 | LEITE DE ROSAS |
| 00516 | CREME DE PELE |
| 00517 | CREME PARA AXILAS |
| 00518 | CREME PARA O ROSTO |
| 00519 | CREME PARA OS PES |
| 00520 | FILTRO SOLAR |
| 00521 | OLEO DE MASSAGEM |
| 00601 | ESMALTE, BASE, ACETONA, ETC. |
| 00602 | BASE |
| 00603 | ACETONA |
| 00604 | LIXA |
| 00605 | ESCOVA DE UNHA |
| 00606 | PAU DE LARANJEIRA |
| 00607 | RALADOR DE PE |
| 00608 | REMOVEDOR DE CUTICULA |
| 00609 | SERRINHA DE UNHA |
| 00610 | LIXA DE PE |
| 00612 | ESMALTE |
| 00701 | GRAMPO, PREGADOR DE CABELO, ETC. |
| 00702 | BOB DE CABELO |
| 00703 | TOUCA DE CABELO |
| 00704 | REDE DE CABELO |
| 00705 | PREGADOR DE CABELO |
| 00706 | PRENDEDOR DE CABELO |
| 00707 | TIARA |
| 00801 | ALICATE, CORTADOR DE UNHA, ETC. |
| 00802 | TESOURINHA DE UNHA |
| 00803 | CORTADOR DE UNHA |
| 00804 | PINCA |
| 00805 | ALICATE E CORTADOR DE UNHA |
| 00901 | PERUCA |
| 00902 | APLIQUE PARA CABELO |
| 01001 | PRODUTOS DE LIMPEZA DE PELE |
| 01002 | SABONETE DE LIMPEZA DE PELE |
| 01003 | SABONETE LIQUIDO DE LIMPEZA DE PELE |
| 01101 | ESPELHO DE BOLSO |
| 01201 | PORTA-MAQUIAGEM |
| 01301 | CERA DEPILATORIA |
| 01302 | CREME DEPILATORIO |
| 01303 | PAPEL PARA DEPILACAO |
| 01401 | ESTOJO (TALCO, SABONETE, PERFUME, ETC.) |
| 01501 | PURPURINA |
| 01601 | ESTOJO DE MANICURO |
| 01701 | ESTOJO (ESPELHO, ESCOVA, PENTE) |
| 01801 | APARELHO DE BARBEAR NAO-DESCARTAVEL |
| 01803 | CARTUCHO APARELHO BARBEAR |
| 01901 | ESTOJO (APARELHO DE BARBEAR, SABONETEIRA, PENTE) |
| 01902 | ESTOJO DE BARBEAR |
| 02001 | ESPONJA DE TALCO |
| 02101 | LOCAO APOS BARBA |
| 02301 | LEITE HIDRATANTE |
| 02501 | CREME ANTI-TRANSPIRANTE |
| 02601 | ARTIGOS DE TRATAMENTO DE CABELO |
| 02602 | REPARADOR DE PONTAS |
| 02603 | OLEO CAPILAR |
| 02604 | ALISANTE DE CABELO |
| 02605 | CHAPINHA DE ALISAR CABELOS |
| 02611 | FIXADOR DE CABELO |
| 02612 | GEL FIXADOR PARA CABELOS |
| 02613 | AGUA OXIGENADA PARA CABELO |
| 02614 | TINTA PARA CABELO |
| 02616 | DESCOLORANTE PARA CABELO |
| 02901 | PORTA-BARBEADOR |
| 03001 | PORTA-SECADOR |
| 03101 | DESODORANTE ESPECIAL PARA OS PES |
| 99901 | AGREGADO |
| 00101 | BARBEIRO |
| 00102 | LAVAGEM (BARBEIRO) |
| 00103 | MASSAGEM (BARBEIRO) |
| 00201 | CABELEIREIRO |
| 00202 | LAVAGEM (CABELEIREIRO) |
| 00203 | MASSAGEM (CABELEIREIRO) |
| 00204 | PENTEADO (CABELEIREIRO) |
| 00205 | TINGIMENTO (CABELEIREIRO) |
| 00206 | ALISAMENTO (CABELEIREIRO) |
| 00207 | SOMBRANCELHA (CABELEIREIRO) |
| 00208 | BANHO DE BRILHO (CABELEIREIRO) |
| 00209 | HIDRATACAO (CABELEIREIRO) |
| 00210 | LUZES (CABELEIREIRO) |
| 00211 | INTERLACE (CABELEIREIRO) |
| 00212 | PERMANENTE (CABELEIREIRO) |
| 00213 | RELAXAMENTO (CABELEIREIRO) |
| 00214 | ESCOVA (CABELEIREIRO) |
| 00301 | MANICURO E PEDICURO |
| 00302 | PEDICURO |
| 00303 | CALISTA |
| 00304 | MANICURO |
| 00401 | SAPATEIRO |
| 00402 | CONSERTO DE SAPATO |
| 00403 | CONSERTO DE CHINELO |
| 00404 | CONSERTO DE BOLSA |
| 00405 | SALTO DE SAPATO |
| 00406 | PALMILHA |
| 00501 | TINTURARIA E LAVANDERIA |
| 00502 | LAVANDERIA |
| 00601 | LIMPEZA DE PELE |
| 00602 | PEELING |
| 00701 | ENGRAXATE |
| 00801 | DEPILACAO |
| 00802 | DESCOLORACAO DE PELOS (BANHO DE LUA) |
| 00803 | BANHO DE LUA (DESCOLORACAO DE PELOS) |
| 00901 | ESTETICISTA |
| 00902 | BANDAGEM (ESTETICISTA) |
| 00903 | DRENAGEM LINFATICA |
| 01001 | AMOLAGEM DE FACA, TESOURA, ALICATE, ETC. |
| 01002 | SERVICO DE CUTELARIA |
| 01101 | MAQUIAGEM |
| 01201 | MASSAGEM E SAUNA |
| 01202 | SAUNA E MASSAGEM |
| 01301 | RELOJOEIRO |
| 01302 | CONSERTO DE RELOGIO |
| 01303 | PULSEIRA DE RELOGIO |
| 01304 | PINO DE RELOGIO |
| 01401 | JOALHEIRO |
| 01402 | CONSERTO DE JOIAS |
| 01403 | OURIVES (CONSERTO DE JOIAS) |
| 01501 | CONSERTO DE SOMBRINHA |
| 01601 | CONSERTO DE OCULOS |
| 01701 | COSTUREIRA |
| 01801 | CONSERTO DE MAQUINA FOTOGRAFICA, FLASH |
| 01802 | CONSERTO DE FLASH |
| 01901 | PINTURA EM CAMISA |
| 02001 | CONSERTO DE BRINQUEDOS E EQUIPAMENTOS ESPORTIVOS (EXCETO CACA E PESCA) |
| 02002 | CONSERTO DE VIDEOGAME |
| 02101 | CONSERTO DE EQUIPAMENTOS DE CACA (ARMA DE FOGO, ETC.) |
| 02201 | CONSERTO DE DISCMAN |
| 02202 | CONSERTO DE DISKMAN |
| 02203 | CONSERTO DE WALKMAN |
| 02301 | CHAVEIRO (SERVICO) |
| 02302 | COPIA DE CHAVE |
| 02401 | CONSERTO DE EQUIPAMENTOS DE PESCA (TARRAFA, MOLINETE, ETC.) |
| 02601 | CONSERTO DE CANETA, LAPISEIRA |
| 02701 | CONSERTO DE BARBEADOR ELETRICO |
| 02801 | GRAVACAO DE NOME EM OBJETOS PESSOAIS (CANETA, MEDALHA, ETC.) |
| 02901 | CONSERTO DE CALCULADORA |
| 03001 | CONSERTO DE ALTO-FALANTE |
| 03101 | CONSERTO DE VIOLAO |
| 03201 | EMOLDURADOR DE QUADRO |
| 03301 | CONSERTO DE PIANO |
| 03401 | MASSAGEM |
| 03501 | ALFAIATE |
| 03601 | BORDADEIRA |
| 03701 | CERZIDEIRA |
| 03801 | FOTOGRAFO (EXCETO DE CERIMONIAS FAMILIARES) |
| 03802 | FOTOGRAFIA 3X4 |
| 03803 | FOTOGRAFIA 5X7 |
| 03901 | CONSERTO DE LANTERNA |
| 04001 | TATUAGEM |
| 04101 | SAUNA |
| 04102 | BANHO TERMAL |
| 04201 | BARBA |
| 04301 | CORTE DE CABELO |
| 04302 | CORTE DE CABELO (FEMININO) |
| 04303 | CORTE DE CABELO (MASCULINO) |
| 04401 | CONSERTO DE ALICATE |
| 04501 | CONSERTO DE APARELHO CELULAR |
| 04601 | BRONZEAMENTO ARTIFICIAL |
| 04701 | CORTE DE CABELO E BARBA |
| 04801 | CROCHETEIRA |
| 04901 | TAPECEIRO |
| 05001 | TRICOTEIRA |
| 05101 | CONSERTO DE FILMADORA |
| 05201 | COLOCACAO DE PIERCING |
| 05202 | PIERCING |
| 05301 | PERSONAL TRAINER |
| 99901 | AGREGADO |
| 00101 | CADERNO |
| 00201 | LAPIS, BORRACHA, CANETA E OUTROS ARTIGOS DE PAPELARIA |
| 00202 | BORRACHA |
| 00203 | CANETA |
| 00204 | APONTADOR |
| 00205 | ESTOJO ESCOLAR |
| 00206 | CARGA DE CANETA |
| 00207 | CORRETIVO |
| 00208 | LAPIS DE COR |
| 00209 | PINCEL |
| 00210 | TESOURA ESCOLAR |
| 00211 | TINTA |
| 00212 | PAPEL |
| 00213 | CLIPS |
| 00214 | GRAMPO DE GRAMPEADOR |
| 00215 | GRAMPEADOR |
| 00216 | BLOCO DE PAPEL |
| 00217 | CARTOLINA |
| 00218 | COLA |
| 00219 | FITA ADESIVA |
| 00220 | REGUA |
| 00221 | COMPASSO |
| 00222 | TRANSFERIDOR |
| 00223 | ESQUADRO |
| 00224 | ENVELOPE |
| 00225 | BANDEIRINHAS DE FESTA |
| 00226 | ENFEITES DE PAREDE DE FESTA |
| 00227 | CARTAO DE ANIVERSARIO |
| 00228 | CARTAO DE VISITA |
| 00229 | CONVITE |
| 00230 | CARTAO DE NATAL |
| 00231 | GRAFITE |
| 00232 | CONFETE PARA CARNAVAL |
| 00233 | ENFEITE DE BOLO |
| 00234 | ARRANJO DE BOLO |
| 00235 | ETIQUETA |
| 00236 | AGENDA |
| 00237 | FITA DE MAQUINA DE ESCREVER |
| 00238 | CURRICULUM VITAE |
| 00239 | CARTELA DE RIFAR PREMIO |
| 00240 | FOLHA PARA FICHARIO |
| 00241 | BOBINA DE FAX |
| 00242 | ALBUM DE FOTOGRAFIAS (EXCETO DE CERIMONIA FAMILIAR) |
| 00243 | ALBUM DE FOTOGRAFIA |
| 00244 | PASTA PLASTICA NAO-ESCOLAR |
| 00245 | MASSA DE MODELAR |
| 00246 | FICHARIO DE CARTAS |
| 00247 | FICHARIO |
| 00248 | PASTA SUSPENSA |
| 00249 | PASTA DE PAPELAO |
| 00250 | PRANCHETA |
| 00251 | TRANSPARENCIA |
| 00252 | GIZ |
| 00253 | CONTACT |
| 00401 | LIVROS NAO-DIDATICOS |
| 00402 | COLECAO DE LIVROS NAO-DIDATICOS |
| 00403 | FASCICULO |
| 00404 | ROMANCE |
| 00405 | BIBLIA |
| 00406 | LIVRO DE CATECISMO |
| 00501 | ASSINATURA DE PERIODICOS |
| 00502 | ASSINATURA DE JORNAL |
| 00503 | ASSINATURA DE REVISTA |
| 00601 | FOTOCOPIA XEROX |
| 00602 | XEROX |
| 00603 | COPIA XEROX |
| 00604 | PLASTIFICACAO, ENCADERNACAO |
| 01501 | DIGITACAO, IMPRESSAO |
| 99901 | AGREGADO |
| 00101 | EQUIPAMENTOS ESPORTIVOS (BOLA, RAQUETE, REDE, TORNOZELEIRA, CHUTEIRA, ETC.) |
| 00102 | RAQUETE |
| 00103 | BOLA DE TENIS |
| 00104 | BOLA DE PINGUE-PONGUE |
| 00105 | REDE DE VOLEIBOL |
| 00106 | EQUIPAMENTOS DE ESPORTE |
| 00107 | BOLA DE SINUCA |
| 00108 | TORNOZELEIRA |
| 00109 | CHUTEIRA |
| 00110 | OCULOS DE NATACAO |
| 00111 | TOUCA DE NATACAO |
| 00112 | STRAP |
| 00113 | CANELEIRA |
| 00114 | PE-DE-PATO |
| 00115 | BOLA (EXCETO DE CRIANCA) |
| 00116 | TRAVA DE CHUTEIRA |
| 00117 | BOIA DE BRACO |
| 00118 | LUVA DE COURO (PARA GOLEIRO) |
| 00119 | EQUIPAMENTOS ESPORTIVOS |
| 00120 | PRANCHA DE SURFE |
| 00121 | MOREY BOGGIES (PRANCHA DE SURFE) |
| 00122 | BODY BOARDING (PRANCHA DE SURFE) |
| 00201 | BRINQUEDOS E JOGOS |
| 00202 | BOLA DE CRIANCA |
| 00203 | BALDINHO DE PRAIA |
| 00204 | BONECA |
| 00205 | JOGO DE ARMAR |
| 00206 | JOGO EDUCATIVO |
| 00207 | JOGO DE ADULTO E CRIANCA |
| 00208 | DAMA |
| 00209 | XADREZ |
| 00210 | BARALHO |
| 00211 | QUEBRA-CABECA |
| 00212 | CARRINHO DE BRINQUEDO |
| 00213 | CARRO DE BRINQUEDO |
| 00214 | MESA DE SINUCA |
| 00215 | MESA DE TOTO |
| 00216 | MESA DE PINGUE-PONGUE |
| 00217 | JOGO DE MONTAR |
| 00218 | QUADRO NEGRO |
| 00219 | BEXIGA |
| 00220 | BOLA DE SOPRAR |
| 00221 | BALAO |
| 00222 | BALAO DE GAS |
| 00223 | CHAPEUZINHO DE PAPEL |
| 00224 | MOTO DE BRINQUEDO |
| 00225 | XIQUE-XIQUE |
| 00226 | PIPA |
| 00227 | URSO DE PELUCIA |
| 00228 | BICHO DE PELUCIA |
| 00229 | CEROL |
| 00230 | MAQUIAGEM DE BRINQUEDO |
| 00231 | PETECA |
| 00232 | MESA INFANTIL |
| 00233 | CADEIRA INFANTIL |
| 00234 | BARRACA DE BRINQUEDO |
| 00301 | PATIM, SKATE, VELOCIPEDE |
| 00302 | SKATE |
| 00303 | VELOCIPEDE |
| 00307 | PATINETE |
| 00308 | TRICICLO |
| 00309 | ACESSORIOS PARA SKATE |
| 00310 | PATIM |
| 00401 | ARMAS DE FOGO (EXCETO DE CACA) |
| 00402 | ESPINGARDA (EXCETO DE CACA) |
| 00403 | REVOLVER (EXCETO DE CACA) |
| 00404 | PISTOLA (EXCETO DE CACA) |
| 00405 | MAUSA (EXCETO DE CACA) |
| 00406 | RIFLE (EXCETO DE CACA) |
| 00407 | CARABINA (EXCETO DE CACA) |
| 00408 | MUNICAO DE ARMA DE FOGO (EXCETO DE CACA) |
| 00501 | MATERIAL DE CACA E PESCA |
| 00502 | ESPINGARDA DE CACA |
| 00503 | ARMA DE CACA |
| 00504 | RIFLE DE CACA |
| 00505 | CARABINA DE CACA |
| 00506 | MUNICAO DE ARMA DE FOGO DE CACA |
| 00507 | MATERIAL DE PESCA |
| 00508 | ANZOL |
| 00509 | LINHA DE PESCA |
| 00510 | PESO DE CHUMBO DE PESCA |
| 00511 | CHUMBO DE PESCA |
| 00512 | PUCA |
| 00513 | REDE DE PESCA |
| 00514 | ARRASTAO |
| 00515 | SAMBURA |
| 00516 | ARPAO |
| 00517 | VARA DE PESCA |
| 00518 | ISCA ARTIFICIAL DE PESCA |
| 00519 | TARRAFA |
| 00520 | CANICO |
| 00521 | CORDA DE REDE DE PESCA |
| 00522 | CANTIL |
| 00601 | PORTE DE ARMA |
| 00701 | REGISTRO DE ARMA |
| 00801 | CELA DE CAVALO (RECREACAO) |
| 00802 | REDEA DE CAVALO |
| 00901 | BICICLETA DE GINASTICA |
| 00902 | PEDALETE (BICICLETA DE GINASTICA) |
| 00903 | BICICLETA ERGOMETRICA |
| 01001 | PORTA-PATINS |
| 01101 | ESTEIRA DE GINASTICA |
| 01102 | ESTEIRA DE CAMINHAR (GINASTICA) |
| 01201 | MINIGAME E AGENDA ELETRONICA INFANTIL |
| 01202 | AGENDA ELETRONICA INFANTIL |
| 01301 | KIT CARNAVALESCO |
| 01401 | LICENCA DE CACA E PESCA |
| 01402 | LICENCA PARA CACA E PESCA |
| 01403 | CACA E PESCA (LICENCA OU AUTORIZACAO) |
| 01404 | AUTORIZACAO DE CACA E PESCA |
| 01405 | AUTORIZACAO PARA CACA E PESCA |
| 01501 | REMO SECO (APARELHO DE GINASTICA) |
| 01502 | REMO (APARELHO DE GINASTICA) |
| 01601 | SOFTWARE DE JOGO |
| 01602 | SOFT DE JOGO |
| 01701 | ESTEPE (APARELHO DE GINASTICA) |
| 01801 | APARELHO DE ABDOMINAL |
| 01901 | APARELHO DE GINASTICA NAO-ESPECIFICADO |
| 02001 | APARELHO DE MUSCULACAO |
| 02101 | ARTIGOS PARA PRAIA |
| 02102 | BARRACA DE PRAIA |
| 02103 | CADEIRA DE PRAIA |
| 02104 | ESTEIRA DE PRAIA |
| 02105 | COLCHAO INFLAVEL |
| 02106 | BOTE INFLAVEL |
| 02201 | ESTEIRA MECANICA |
| 02301 | LICENCA DE RADIO AMADOR |
| 02401 | TROFEU |
| 02501 | ESCUDO DE TIME |
| 02601 | ARTIGOS EROTICOS |
| 02602 | VIBRADOR EROTICO |
| 02603 | BONECA INFLAVEL |
| 02604 | CALCINHA COMESTIVEL |
| 02605 | ALGEMA EROTICA |
| 02606 | CHICOTE EROTICO |
| 02607 | VIBRADOR |
| 99901 | AGREGADO |
| 00101 | AGASALHO (CASACO, SUETER, ETC.) |
| 00102 | SUETER |
| 00103 | BLUSA DE LA |
| 00104 | JAQUETA |
| 00105 | PULOVER |
| 00106 | CACHECOL |
| 00107 | CASACO |
| 00201 | BERMUDA, CALCAO E SHORT |
| 00202 | CALCAO |
| 00203 | SHORT |
| 00204 | BERMUDA |
| 00301 | CALCA COMPRIDA |
| 00401 | BLUSA OU CAMISA |
| 00402 | BLUSA |
| 00403 | CAMISA |
| 00404 | CAMISA MANGA CURTA |
| 00405 | CAMISA MANGA LONGA |
| 00501 | CAMISETA |
| 00601 | CONJUNTO ESPORTIVO |
| 00602 | JOGGING |
| 00603 | TRAINING |
| 00604 | ABRIGO |
| 00701 | CUECA |
| 00801 | GRAVATA |
| 00901 | SUNGA DE PRAIA |
| 01001 | MACACAO |
| 01002 | JARDINEIRA |
| 01101 | MEIAS |
| 01201 | PALETO E BLAZER |
| 01202 | BLAZER |
| 01203 | PALETO |
| 01301 | PIJAMA E ROBE |
| 01302 | ROBE |
| 01303 | ROUPAO |
| 01304 | ROUPAO DE BANHO |
| 01305 | PIJAMA |
| 01401 | TERNO |
| 01501 | COLETE |
| 01601 | GORRO |
| 01701 | LUVA DE COURO, MALHA,TECIDO |
| 01702 | LUVA DE COURO |
| 01703 | LUVA DE MALHA |
| 01704 | LUVA DE TECIDO |
| 01801 | ALUGUEL DE ROUPA |
| 01901 | FARDAMENTO MILITAR |
| 02001 | SOBRETUDO |
| 02101 | SLACK |
| 02201 | CAMISA (UNIFORME DE ESPORTE) |
| 02301 | CALCAO (UNIFORME DE ESPORTE) |
| 02401 | MEIAO (UNIFORME DE ESPORTE) |
| 02501 | FANTASIA |
| 02601 | MACACAO DE MOTOQUEIRO |
| 02701 | CONJUNTO DE CALCA E CAMISA |
| 02801 | CONJUNTO DE SHORT E CAMISA |
| 02901 | SMOKING |
| 03001 | QUIMONO |
| 03002 | UNIFORME DE JUDO (QUIMONO) |
| 03101 | CONJUNTO DE CALCA E JAQUETA |
| 03201 | UNIFORME DE TRABALHO |
| 03202 | JALECO |
| 03301 | FAIXA DE SMOKING |
| 99901 | AGREGADO |
| 00101 | AGASALHO (CASACO, SUETER, ETC.) |
| 00102 | CASACO |
| 00103 | SUETER |
| 00104 | PULOVER |
| 00105 | CACHECOL |
| 00106 | JAQUETA |
| 00107 | ECHARPE |
| 00108 | AGASALHO |
| 00109 | BLUSA DE LA |
| 00110 | CHALE |
| 00201 | BERMUDA E SHORT |
| 00202 | SHORT |
| 00203 | BERMUDA |
| 00301 | BLUSA OU CAMISA |
| 00302 | BLUSA |
| 00303 | CAMISA |
| 00304 | BATA |
| 00305 | BABY LOOK |
| 00306 | BODY |
| 00307 | BOLERO |
| 00401 | CALCA COMPRIDA |
| 00501 | CALCINHA |
| 00601 | COLLANT |
| 00602 | ROUPA DE GINASTICA |
| 00603 | MALHA DE GINASTICA |
| 00604 | ROUPA DE BALE |
| 00701 | CAMISOLA, PIJAMA, ROBE, ETC |
| 00702 | PIJAMA |
| 00703 | ROBE |
| 00704 | ROUPAO DE BANHO |
| 00705 | CAMISOLA |
| 00706 | BABY DOLL |
| 00801 | CONJUNTO ESPORTIVO |
| 00802 | JOGGING |
| 00803 | TRAINNING |
| 00901 | CAMISETA |
| 01001 | MACACAO |
| 01002 | JARDINEIRA |
| 01101 | MAIO E BIQUINI |
| 01102 | BIQUINI |
| 01103 | MAIO |
| 01201 | MEIAS |
| 01202 | MEIA-CALCA |
| 01301 | SAIA |
| 01401 | VESTIDO |
| 01501 | MODELADOR |
| 01601 | LUVA DE COURO, MALHA, TECIDO |
| 01602 | LUVA DE COURO |
| 01603 | LUVA DE MALHA |
| 01604 | LUVA DE TECIDO |
| 01701 | COMBINACAO |
| 01801 | GORRO |
| 01901 | ALUGUEL DE ROUPA |
| 02001 | CINTA |
| 02101 | SAIDA DE PRAIA |
| 02102 | CANGA |
| 02201 | PONCHO DE LA |
| 02301 | ANAGUA |
| 02401 | SLACK |
| 02501 | VESTIDO DE NOIVA |
| 02601 | CAMISA (UNIFORME DE ESPORTE) |
| 02701 | CALCAO (UNIFORME DE ESPORTE) |
| 02801 | MEIAO (UNIFORME DE ESPORTE) |
| 02901 | FANTASIA |
| 03001 | BUSTIE |
| 03101 | MACACAO DE MOTOQUEIRA |
| 03201 | POLAINAS |
| 03202 | CANELEIRA (POLAINA) |
| 03301 | CONJUNTO DE SAIA E BLUSA |
| 03401 | CONJUNTO DE SHORT E BLUSA |
| 03501 | BLAZER |
| 03601 | CONJUNTO DE SAIA E BLAZER |
| 03701 | TERNO OU TERNINHO |
| 03702 | TERNO |
| 03703 | TERNINHO |
| 03801 | MACAQUITO |
| 03802 | MACAQUINHO |
| 03901 | CONJUNTO DE CALCA E BLUSA |
| 04001 | SUTIA |
| 04101 | CALCA PLASTICA |
| 04201 | UNIFORME DE TRABALHO |
| 04202 | JALECO |
| 04301 | COLETE |
| 04401 | CONJUNTO DE CALCA E JAQUETA |
| 04501 | CONJUNTO DE SAIA E JAQUETA |
| 04601 | QUIMONO |
| 04701 | GRAVATA |
| 04801 | CALCA DE CAPOEIRA (ABADA) |
| 04901 | SAIA-CALCA |
| 05001 | TOP |
| 05101 | CALCA (UNIFORME DE ESPORTE) |
| 05201 | CONJUNTO DE BERMUDA E BLUSA |
| 05301 | CONJUNTO DE CALCA E BLAZER |
| 05401 | CONJUNTO DE CASACO E BLUSA |
| 05501 | SOBRETUDO |
| 05601 | FARDAMENTO MILITAR |
| 05701 | CONJUNTO DE CALCINHA E SUTIA |
| 05801 | CALCA (EXCETO COMPRIDA) |
| 05901 | CONJUNTO DE MALHA |
| 99901 | AGREGADO |
| 00101 | AGASALHO (CASACO, SUETER, ETC) |
| 00102 | SUETER |
| 00103 | JAQUETA |
| 00104 | PULOVER |
| 00105 | CACHECOL |
| 00106 | CASACO |
| 00201 | BERMUDA, SHORT E CALCAO |
| 00202 | SHORT |
| 00203 | BERMUDA |
| 00204 | CALCAO |
| 00301 | BLUSA OU CAMISA |
| 00302 | CAMISA |
| 00303 | BLUSA |
| 00401 | CALCA COMPRIDA |
| 00501 | SUNGA, MAIO E BIQUINI |
| 00502 | SUNGA |
| 00503 | MAIO |
| 00504 | BIQUINI |
| 00505 | TANGA |
| 00601 | CALCINHA |
| 00701 | ROUPA DE GINASTICA |
| 00702 | COLLANT |
| 00703 | MALHA DE GINASTICA |
| 00801 | CAMISOLA, PIJAMA, ROBE, ETC |
| 00802 | PIJAMA |
| 00803 | ROBE |
| 00804 | ROUPAO DE BANHO |
| 00805 | CAMISOLA |
| 00806 | BABY DOLL |
| 00901 | CONJUNTO ESPORTIVO |
| 00902 | JOGGING |
| 00903 | TRAINNING |
| 00904 | CONJUNTO DE MOLETON |
| 01001 | CUECA |
| 01101 | SUTIA |
| 01201 | FRALDA DE TECIDO |
| 01301 | MACACAO |
| 01302 | JARDINEIRA |
| 01401 | MEIAS |
| 01402 | MEIA-CALCA |
| 01501 | PALETO, BLAZER E TERNO |
| 01502 | BLAZER |
| 01503 | TERNO |
| 01504 | PALETO |
| 01601 | SAIA |
| 01701 | VESTIDO |
| 01702 | VESTIDO DE DAMA DE HONRA |
| 01801 | OUTRAS ROUPAS DE BEBE |
| 01802 | CALCA PLASTICA |
| 01803 | CUEIRO |
| 01804 | MANTA |
| 01805 | CINTEIRO |
| 01806 | BABADOR |
| 01807 | CALCA ENXUTA |
| 01808 | UMBIGUEIRO |
| 01809 | SAPATINHO DE LA OU LINHA |
| 01810 | FLANELA PARA BEBE |
| 01811 | MIJAOZINHO (CALCA DE MALHA PARA BEBE) |
| 01812 | CONJUNTO PAGAO |
| 01813 | CONJUNTINHO PAGAO |
| 01814 | TROCA-FRALDAS |
| 01901 | CAMISETA |
| 02001 | ANAGUA |
| 02101 | COMBINACAO |
| 02201 | GORRO |
| 02301 | SAIDA DE PRAIA |
| 02401 | LUVA DE COURO, MALHA,TECIDO |
| 02402 | LUVA DE COURO |
| 02403 | LUVA DE MALHA |
| 02404 | LUVA DE TECIDO |
| 02501 | CONJUNTO DE SHORT E CAMISA |
| 02601 | CONJUNTO DE BERMUDA E BLUSA |
| 02701 | ALUGUEL DE ROUPA |
| 02801 | UNIFORME PARA CORAL |
| 02802 | UNIFORME PARA CORAL DE IGREJA |
| 02803 | BECA |
| 02804 | CONJUNTO PARA PRIMEIRA COMUNHAO |
| 02901 | CAMISA (UNIFORME DE ESPORTE) |
| 03001 | CALCAO (UNIFORME DE ESPORTE) |
| 03101 | MEIAO (UNIFORME DE ESPORTE) |
| 03201 | FANTASIA |
| 03301 | BUSTIE |
| 03401 | CONJUNTO DE SAIA E BLUSA |
| 03501 | CONJUNTO DE CALCA E CAMISA |
| 03601 | QUIMONO |
| 03602 | UNIFORME DE JUDO (QUIMONO) |
| 03701 | CONJUNTO DE CALCA E JAQUETA |
| 03801 | PONCHO DE LA |
| 03901 | MACAQUITO |
| 04001 | GRAVATA |
| 04101 | POLAINAS |
| 04102 | CANELEIRA (POLAINA) |
| 04201 | COLETE |
| 04301 | UNIFORME DE BALE |
| 04401 | SLACK |
| 04501 | CONJUNTO DE SAIA E JAQUETA |
| 04601 | ENXOVAL DE BEBE |
| 04701 | CONJUNTO DE CALCINHA E SUTIA |
| 04801 | CALCA (EXCETO COMPRIDA) |
| 99901 | AGREGADO |
| 00101 | ARTIGOS DE ARMARINHO |
| 00102 | LINHA |
| 00103 | ALFINETE |
| 00104 | AGULHA |
| 00105 | BOTAO |
| 00106 | RENDA |
| 00107 | NOVELO DE LA |
| 00108 | CARRETEL |
| 00109 | FECHO |
| 00110 | COLCHETE |
| 00111 | TESOURA |
| 00112 | ELASTICO |
| 00113 | ESTOJO DE COSTURA |
| 00114 | FITA METRICA |
| 00115 | ILHOS |
| 00116 | DEDAL |
| 00117 | CARRETILHA DE LINHA (CARRETEL DE LINHA DE ARMARINHO) |
| 00118 | FITA DE FRALDA (ADESIVA) |
| 00119 | FIVELA DE CINTO |
| 00120 | AGULHA DE TRICO |
| 00121 | FITA DE TECIDO |
| 00122 | CADARCO |
| 00123 | CORREIA DE CHINELO |
| 00124 | BARBANTE |
| 00125 | CORDAO |
| 00126 | CORDA DE ALGODAO |
| 00127 | PRESILHA DE FRALDA |
| 00128 | LASTEX (ELASTICO) |
| 00129 | GUISO |
| 00130 | AGULHA DE CROCHE |
| 00131 | AGULHA DE MAQUINA DE COSTURA |
| 00201 | TECIDOS |
| 00301 | ROUPA DE BANHO |
| 00302 | TOALHA DE BANHO |
| 00303 | TOALHA INFANTIL DE BANHO |
| 00304 | JOGO DE TOALHAS DE BANHO |
| 00305 | TOALHA DE ROSTO |
| 00401 | ROUPA DE CAMA |
| 00402 | LENCOL |
| 00403 | MANTA DE CAMA |
| 00404 | COBERTOR |
| 00405 | FRONHA |
| 00406 | COLCHA |
| 00407 | EDREDON |
| 00408 | JOGO DE ROUPAS DE CAMA |
| 00409 | KIT DE BERCO |
| 00410 | MANTA PARA SOFA |
| 00501 | ROUPA DE MESA DE TECIDO |
| 00502 | TOALHA DE MESA DE TECIDO |
| 00503 | PANO DE PRATO |
| 00504 | JOGO DE MESA DE TECIDO |
| 00505 | JOGO AMERICANO DE TECIDO |
| 00506 | GUARDANAPO DE MESA DE TECIDO |
| 00601 | ROUPA DE MESA DE PLASTICO |
| 00602 | PANO DE CENTRO DE PLASTICO |
| 00603 | TOALHA DE MESA DE PLASTICO |
| 00604 | JOGO DE MESA DE PLASTICO |
| 00605 | JOGO AMERICANO DE PLASTICO |
| 00701 | LEQUE (USO PESSOAL) |
| 00801 | TINTA DE TECIDO |
| 00802 | TINTURA DE ROUPA |
| 00901 | PLUMAS |
| 01001 | CORTINADO |
| 01002 | ACORTINADO |
| 01003 | MOSQUITEIRO |
| 01004 | BLACKOUT (PLASTICO DE JANELA) |
| 01101 | ENCHIMENTO DE ALMOFADA, DE TRAVESSEIRO, ETC |
| 01102 | ESPUMA DE ENCHIMENTO DE ALMOFADA, DE TRAVESSEIRO, ETC. |
| 01103 | PAINA DE ENCHIMENTO DE ALMOFADA, DE TRAVESSEIRO, ETC. |
| 01104 | ESPONJA DE ENCHIMENTO DE ALMOFADA, DE TRAVESSEIRO, ETC. |
| 01201 | REVESTIMENTO DE TABUA DE PASSAR ROUPA |
| 01301 | AVENTAL, LUVA, ETC. PARA SERVICOS DOMESTICOS |
| 01302 | LUVA PARA SERVICOS DOMESTICOS |
| 01303 | LUVA MULTIUSO |
| 01304 | AVENTAL PARA SERVICOS DOMESTICOS |
| 01401 | OMBREIRA |
| 01501 | PLASTICO |
| 01601 | LENCOL DESCARTAVEL |
| 01701 | PUNHO DE REDE |
| 01801 | NAPA |
| 01802 | CORINO |
| 01901 | COURO |
| 02001 | PROTETOR DE COLCHAO |
| 02101 | TINTA DE CALCADO |
| 02201 | PE-DE-COELHO |
| 02301 | TRAVESSEIRO |
| 02401 | ENXOVAL (ROUPAS DE CAMA, MESA, BANHO) |
| 02501 | ESPUMA DE COLCHAO |
| 02601 | PRENDEDOR DE CHUPETA |
| 02701 | ALCA PARA SUTIA |
| 02801 | BASTIDOR |
| 02901 | BICO PARA VESTIDO |
| 03001 | BORDADO INGLES |
| 03101 | PROTETOR DE ENDREDON |
| 03201 | PROTETOR DE COBERTOR |
| 03301 | PROTETOR DE TRAVESSEIRO |
| 03401 | COLA PARA TECIDO |
| 03501 | GOLA |
| 03601 | LONA |
| 03701 | INTERTELA |
| 03801 | TOALHA DE MAO |
| 03901 | TOALHA DE PRAIA |
| 04001 | CABIDE |
| 04101 | CAIXA DE PRESENTE |
| 04201 | PAPEL DE PRESENTE |
| 04301 | TRILHO DE MESA |
| 04401 | FORRACAO DE BOTAO |
| 04501 | JOGO DE COZINHA (TECIDO) |
| 04601 | PORTA-FRALDA |
| 04701 | PORTA-CINTO |
| 99901 | AGREGADO |
| 00101 | BOLSA, CARTEIRA, PASTA E MALETA DE HOMEM |
| 00102 | PASTA DE HOMEM |
| 00103 | MALETA DE HOMEM |
| 00104 | CARTEIRA DE HOMEM |
| 00105 | BOLSA DE HOMEM |
| 00201 | BOLSA E CARTEIRA DE MULHER |
| 00202 | CARTEIRA DE MULHER |
| 00203 | BOLSA DE MULHER |
| 00204 | PORTA-CHEQUE DE MULHER |
| 00301 | FRASQUEIRA |
| 00401 | VALISE |
| 00501 | MALA |
| 00601 | SANDALIA DE CRIANCA (EXCETO DE BORRACHA OU PLASTICO) |
| 00701 | SANDALIA DE HOMEM (EXCETO DE BORRACHA OU PLASTICO) |
| 00801 | SANDALIA DE MULHER (EXCETO DE BORRACHA OU PLASTICO) |
| 00901 | SANDALIA DE BORRACHA DE CRIANCA |
| 01001 | SANDALIA E SAPATO DE PLASTICO DE CRIANCA |
| 01002 | SAPATO DE PLASTICO DE CRIANCA |
| 01003 | SANDALIA DE PLASTICO DE CRIANCA |
| 01101 | SANDALIA E SAPATO DE PLASTICO DE MULHER |
| 01102 | SAPATO DE PLASTICO DE MULHER |
| 01103 | SANDALIA DE PLASTICO DE MULHER |
| 01201 | SAPATO E BOTA DE CRIANCA (EXCETO DE PLASTICO) |
| 01202 | BOTA DE CRIANCA (EXCETO DE PLASTICO) |
| 01203 | SAPATO DE CRIANCA (EXCETO DE PLASTICO) |
| 01301 | SAPATO E BOTA DE HOMEM (EXCETO DE PLASTICO) |
| 01302 | BOTA DE HOMEM (EXCETO DE PLASTICO) |
| 01303 | SAPATO DE HOMEM (EXCETO DE PLASTICO) |
| 01401 | SAPATO E BOTA DE MULHER (EXCETO DE PLASTICO) |
| 01402 | BOTA DE MULHER (EXCETO DE PLASTICO) |
| 01403 | SAPATO DE MULHER (EXCETO DE PLASTICO) |
| 01501 | TENIS DE CRIANCA |
| 01601 | TENIS DE HOMEM |
| 01701 | CINTO DE HOMEM |
| 01801 | CINTO DE MULHER |
| 01901 | CHINELO |
| 01902 | PANTUFA |
| 02001 | TAMANCO |
| 02101 | CINTO DE CRIANCA |
| 02201 | SUSPENSORIO |
| 02301 | BOLSA DE CRIANCA |
| 02302 | CARTEIRA DE CRIANCA |
| 02303 | PONCHETE DE CRIANCA |
| 02304 | POCHETE DE CRIANCA |
| 02401 | SAPATINHO DE BEBE (EXCETO DE LA) |
| 02501 | MOCHILA (EXCETO ESCOLAR) |
| 02601 | ALPARGATA DE HOMEM |
| 02701 | ALPARGATA DE MULHER |
| 02801 | ALPARGATA DE CRIANCA |
| 02901 | ALUGUEL DE BOLSA, CALCADO, CINTO, CHAPEU, LUVA, ETC. |
| 03001 | SAPATILHA |
| 03002 | SAPATILHA DE BALE |
| 03003 | SAPATILHA DE GINASTICA |
| 03101 | CARTEIRA (NAO-ESPECIFICADA) |
| 03201 | SANDALIA E SAPATO DE PLASTICO DE HOMEM |
| 03202 | SAPATO DE PLASTICO DE HOMEM |
| 03203 | SANDALIA DE PLASTICO DE HOMEM |
| 03301 | GALOCHA DE CRIANCA, HOMEM E MULHER |
| 03401 | BOTA DE PLASTICO DE CRIANCA |
| 03501 | PORTA-NIQUEIS |
| 03601 | SACOLA DE BEBE |
| 03701 | SACOLA DE VIAGEM |
| 03702 | BOLSA DE VIAGEM |
| 03801 | CARRINHO DE CARREGAR MALA, VALISE, ETC. |
| 03901 | BOLSA (NAO-ESPECIFICADA) |
| 04001 | SANDALIA (NAO-ESPECIFICADA) |
| 04002 | RASTEIRINHA |
| 04101 | SAPATO (NAO-ESPECIFICADO) |
| 04201 | ALPARGATA (NAO-ESPECIFICADA) |
| 04301 | BOTA (NAO-ESPECIFICADA) |
| 04401 | COTURNO |
| 04501 | CINTO (NAO-ESPECIFICADO) |
| 04601 | TENIS (NAO-ESPECIFICADO) |
| 04701 | SANDALIA DE BORRACHA DE HOMEM |
| 04801 | SANDALIA DE PLASTICO (NAO-ESPECIFICADA) |
| 04901 | SANDALIA DE BORRACHA DE MULHER |
| 05001 | CAPA DE CHUVA |
| 05101 | LENCO DE CABECA |
| 05102 | VEU DE CABECA |
| 05201 | LENCINHO |
| 05202 | LENCO |
| 05301 | ISQUEIRO NAO-DESCARTAVEL (USO PESSOAL) |
| 05401 | GRINALDA |
| 05501 | ARRANJO DE CABELO |
| 05601 | LENCO DE PESCOCO |
| 05701 | ENFEITE DE CABELO |
| 05702 | LACINHO (ENFEITE DE CABELO) |
| 05801 | CHAPEU |
| 05901 | BONE E VISEIRA |
| 05902 | BONE |
| 05903 | VISEIRA |
| 05904 | TOUCA |
| 06001 | GUARDA-CHUVA E SOMBRINHA |
| 06002 | GUARDA-CHUVA |
| 06003 | SOMBRINHA |
| 06101 | FAIXA DE CABECA |
| 06201 | BENGALA |
| 06301 | BOTA DE PLASTICO (NAO-ESPECIFICADA) |
| 06401 | BOTA DE BORRACHA (NAO-ESPECIFICADA) |
| 06501 | SANDALIA DE BORRACHA (NAO-ESPECIFICADA) |
| 06601 | TENIS DE MULHER |
| 06701 | CACHIMBO |
| 06801 | BOLSA DE CACHIMBO |
| 06901 | CIGARREIRA |
| 07001 | LIMPADOR DE CACHIMBO |
| 07101 | SAPATENIS |
| 07201 | POCHETE |
| 99901 | AGREGADO |
| 00101 | COPO DE VIDRO COMUM |
| 00102 | CONJUNTO DE COPOS COMUNS DE VIDRO |
| 00103 | COPO COMUM DE VIDRO |
| 00201 | PRATO DE LOUCA |
| 00202 | CONJUNTO DE PRATOS DE LOUCA |
| 00203 | JOGO DE PRATOS DE LOUCA |
| 00301 | TRAVESSA DE VIDRO OU LOUCA |
| 00401 | XICARA COMUM |
| 00402 | CONJUNTO DE XICARAS COMUNS |
| 00501 | OUTROS UTENSILIOS DE VIDRO OU LOUCA |
| 00601 | FRIGIDEIRA, LEITEIRA, ETC. |
| 00602 | PANELA |
| 00603 | LEITEIRA |
| 00604 | PANELA DE PRESSAO |
| 00605 | PIPOQUEIRA (PANELA) |
| 00606 | CONJUNTO DE PANELAS |
| 00701 | TALHERES |
| 00702 | FACA |
| 00703 | GARFO |
| 00704 | COLHER |
| 00705 | CONJUNTO DE COLHERES |
| 00706 | CONJUNTO DE GARFOS |
| 00707 | CONJUNTO DE FACAS |
| 00708 | CONJUNTO DE ESCUMADEIRAS |
| 00709 | CONJUNTO DE CONCHAS |
| 00801 | OUTROS UTENSILIOS DE METAL |
| 00802 | SOCADOR DE CARNE DE METAL |
| 00803 | CANECA DE METAL |
| 00804 | RALADOR DE QUEIJO |
| 00805 | ESCORREDOR DE METAL |
| 00806 | ABRIDOR DE GARRAFA, LATA, ETC. |
| 00807 | TORRADEIRA DE PAO NAO-ELETRICA |
| 00808 | FACAO |
| 00809 | LATA DE CONDIMENTO (AVULSA) |
| 00810 | PORTA-CONDIMENTO (AVULSO) |
| 00811 | SUPORTE DE PANELAS |
| 00812 | FORMA DE BAURU |
| 00813 | FORMA DE BOLO, PIZZA, PUDIM, TORTA |
| 00814 | PORTA-COPOS |
| 00815 | RASPADOR DE COCO |
| 00816 | TRIPE DE PANELAS DE METAL |
| 00817 | BALDE DE ALUMINIO |
| 00818 | MARMITA DE ALUMINIO |
| 00819 | AGULHA DE DESENTUPIR FOGAO |
| 00820 | PROTETOR DE FERRO ELETRICO DE METAL |
| 00821 | CONJUNTO DE BALDES DE ALUMINIO |
| 00822 | CONJUNTO DE BACIAS DE ALUMINIO |
| 00823 | JOGO DE BACIAS DE ALUMINIO |
| 00824 | CONJUNTO DE MARMITAS |
| 00825 | JOGO DE MARMITAS |
| 00826 | CONJUNTO DE FORMAS DE BOLO, PIZZA, PUDIM, TORTA |
| 00827 | BACIA DE ALUMINIO |
| 00828 | BACIA DE ESMALTE |
| 00829 | BACIA DE INOX |
| 00830 | FRUTEIRA DE INOX |
| 00831 | BANDEJA DE INOX |
| 00832 | COPO DE INOX |
| 00833 | CONJUNTO DE ESPETOS DE METAL |
| 00834 | JOGO DE ESPETOS DE METAL |
| 00835 | ESPREMEDOR DE METAL |
| 00836 | COPO DE ALUMINIO |
| 00837 | RALADOR |
| 00901 | UTENSILIOS DE PLASTICO |
| 00902 | BALDE PLASTICO |
| 00903 | BACIA PLASTICA |
| 00904 | GARRAFA PLASTICA DE AGUA |
| 00905 | VASILHAME PLASTICO DE MANTIMENTOS |
| 00906 | GARRAFAO PLASTICO DE AGUA |
| 00907 | ENGRADADO PLASTICO |
| 00908 | PORTA-TOALHA DE PLASTICO |
| 00909 | ARMARIO DE PLASTICO DE BANHEIRO |
| 00910 | BANHEIRA PLASTICA DE CRIANCA OU BEBE |
| 00911 | CONJUNTO DE BANHEIRO DE PLASTICO |
| 00912 | CONJUNTO PLASTICO DE BANHEIRO |
| 00913 | ASSENTO DE VASO SANITARIO |
| 00914 | TAMPA DE TANQUE DE PLASTICO |
| 00915 | TORNEIRA DE FILTRO DE PLASTICO |
| 00916 | PORTA-PAPEL HIGIENICO |
| 00917 | VITASUCO (COPO DE LIQUIDIFICADOR) |
| 00918 | ENFEITE DE GELADEIRA DE PLASTICO |
| 00919 | OURINOL DE PLASTICO |
| 00920 | PENICO DE PLASTICO |
| 00921 | SUPORTE DE GARRAFA TERMICA DE PLASTICO |
| 00922 | COPO DE LIQUIDIFICADOR |
| 00923 | BASE DE LIQUIDIFICADOR |
| 00924 | COPO E BASE DE LIQUIDIFICADOR |
| 00925 | SABONETEIRA PLASTICA |
| 00926 | SUPORTE PARA PAPEL ALUMINIO DE PLASTICO |
| 00927 | SUPORTE PARA PAPEL HIGIENICO DE PLASTICO |
| 00928 | PRESSURIZADOR DE CHUVEIRO |
| 00929 | CONJUNTO DE BALDES DE PLASTICO |
| 00930 | CONJUNTO DE BACIAS DE PLASTICO |
| 00931 | CONJUNTO PLASTICO DE MANTIMENTOS OU CONDIMENTOS |
| 00932 | JOGO PLASTICO DE MANTIMENTOS OU CONDIMENTOS |
| 00933 | CONJUNTO PLASTICO DE CONDIMENTOS |
| 00934 | JOGO PLASTICO DE CONDIMENTOS |
| 00935 | IMA DE GELADEIRA |
| 00936 | COPOS PLASTICOS |
| 00937 | PRATO PLASTICO |
| 01001 | GARRAFA TERMICA |
| 01101 | VELA DE FILTRO |
| 01201 | CAPACHO |
| 01301 | CONJUNTO DE LATAS DE MANTIMENTOS |
| 01302 | LATA DE MANTIMENTOS |
| 01401 | MANGUEIRA DE PLASTICO OU BORRACHA |
| 01501 | CONJUNTO DE SALADEIRAS |
| 01502 | JOGO DE SALADEIRAS |
| 01503 | SALADEIRA |
| 01601 | UTENSILIOS DE MADEIRA |
| 01602 | ENGRADADO DE MADEIRA |
| 01603 | TABUA DE CARNE |
| 01604 | SOCADOR DE ALHO DE MADEIRA |
| 01605 | SUPORTE PARA PAPEL HIGIENICO DE MADEIRA |
| 01701 | CAPA DE LIQUIDIFICADOR, ENCERADEIRA, MAQUINA DE LAVAR, ETC. |
| 01801 | CONJUNTO DE FORMAS DE BARQUETE |
| 01802 | FORMA DE BARQUETE |
| 01803 | JOGO DE FORMAS DE BARQUETE |
| 01901 | COADOR DE TECIDO |
| 02001 | BUJAO DE GAS VAZIO |
| 02002 | BOTIJAO DE GAS VAZIO |
| 02101 | UTENSILIOS DE BARRO |
| 02102 | PANELA DE BARRO |
| 02103 | CONJUNTO DE PANELAS DE BARRO |
| 02104 | JOGO DE PANELAS DE BARRO |
| 02201 | UTENSILIOS DE ACRILICO |
| 02202 | COPO DE ACRILICO |
| 02203 | CESTA DE ACRILICO |
| 02301 | PRATO DE ESMALTE |
| 02401 | UTENSILIOS DE ISOPOR |
| 02402 | CAIXA DE ISOPOR |
| 02403 | PORTA-MAMADEIRA DE ISOPOR |
| 02501 | UTENSILIOS DE BORRACHA |
| 02502 | CORTINA E TAPETE DE BORRACHA |
| 02503 | TAPETES DE BORRACHA |
| 02504 | BORRACHA DE PANELA DE PRESSAO |
| 02601 | CESTO PARA ROUPA |
| 02602 | SACOLA PARA ROUPA |
| 02701 | APARELHO DE CORTAR VERDURA |
| 02702 | CORTADOR DE VERDURA (APARELHO) |
| 02801 | UTENSILIOS DE FIBRA VEGETAL |
| 02802 | PENEIRA DE FIBRA VEGETAL |
| 02901 | PORTA-TOALHA DE TECIDO |
| 03001 | CONJUNTO DE TECIDO DE BANHEIRO |
| 03002 | JOGO DE TECIDO DE BANHEIRO |
| 03101 | SAPATEIRA |
| 03201 | TACA DE SORVETE |
| 03202 | CONJUNTO PARA SORVETE |
| 03203 | CONJUNTO DE TACAS PARA SORVETE |
| 03301 | PROTETOR DE PORTA |
| 03401 | JARRA DE CRISTAL |
| 03501 | COPO DE CRISTAL |
| 03601 | LANCHEIRA (EXCETO ESCOLAR) |
| 03701 | CAMISA DE LAMPIAO A GAS |
| 03702 | CAMISINHA DE LAMPIAO A GAS |
| 03801 | PORTA-BOLO DE PORCELANA |
| 03802 | BANDEJA DE PORCELANA (PORTA-BOLO) |
| 03901 | EXTENSAO DE FERRO ELETRICO (ESPECIFICO) |
| 04001 | SUPORTE PARA GARRAFAO DE AGUA |
| 04002 | SUPORTE PARA AGUA MINERAL |
| 04101 | VARAL DE APARTAMENTO |
| 04102 | VARAL DE CORDINHAS REGULAVEL |
| 04201 | REGISTRO DE FOGAO |
| 04202 | RELOGIO DE FOGAO |
| 04303 | REGISTRO DE BOTIJAO DE GAS |
| 04304 | VALVULA DE BOTIJAO DE GAS |
| 04305 | RELOGIO DE BOTIJAO DE GAS |
| 04401 | TACA DE CRISTAL |
| 04402 | TACA DE VIDRO |
| 04501 | PES PARA GELADEIRA DE METAL OU LOUCA |
| 04502 | PES DE GELADEIRA DE METAL OU LOUCA |
| 04601 | CONJUNTO DE TRAVESSAS REFRATARIAS PARA MICROONDAS |
| 04602 | JOGO DE TRAVESSAS REFRATARIAS PARA MICROONDAS |
| 04701 | CAPA DE SOM, VIDEO |
| 04702 | CAPA DE COMPUTADOR |
| 04801 | BACIA INDETERMINADA |
| 04901 | BALDE |
| 05001 | ASSADEIRA |
| 05101 | ACUCAREIRO |
| 05201 | BANDEJA |
| 05302 | CONJUNTO DE BANDEJAS |
| 05401 | BATEDOR DE OVOS MANUAL |
| 05501 | BULE |
| 05601 | CALDEIRAO |
| 05701 | CANECA |
| 05801 | CHALEIRA |
| 05901 | COMPOTEIRA |
| 06001 | CONCHA |
| 06101 | CONJUNTO DE CHIMARRAO |
| 06201 | CONJUNTO DE PIREX |
| 06301 | CONJUNTO DE SOBREMESA |
| 06401 | CONJUNTO DE POTES |
| 06501 | CORTADOR DE BOLO |
| 06601 | CORTADOR DE PIZZA |
| 06701 | CUSCUZEIRA |
| 06801 | ESCORREDOR DE ARROZ |
| 06901 | ESCUMADEIRA |
| 07001 | FARINHEIRA |
| 07101 | FORMA DE CREPE |
| 07201 | FORMA DE GELO |
| 07301 | FORMA DE PAO |
| 07401 | FORMA DE MISTO-QUENTE |
| 07501 | FRUTEIRA |
| 07601 | FUNIL |
| 07701 | JARRA |
| 07801 | CONJUNTO DE JARRA E COPOS |
| 07901 | JOGO DE CANECAS |
| 08001 | GRELHA |
| 08101 | JOGO DE TIGELAS |
| 08102 | TIGELA |
| 08201 | CESTA DE PALHA |
| 08301 | GALHETEIRO |
| 08401 | MANTEIGUEIRA |
| 08501 | PALITEIRO |
| 08601 | OMELETEIRA |
| 08701 | PAPEIRO |
| 08801 | PES PARA FOGAO DE METAL OU LOUCA |
| 08901 | PILAO |
| 09001 | PINCEL DE COZINHA |
| 09101 | PORTA-OBJETOS (MADEIRA, ACRILICO, LOUCA, METAL, ETC.) |
| 09201 | SALEIRO |
| 09301 | SUPORTE PARA BUJAO DE GAS |
| 09401 | RALO DE PIA |
| 09501 | KIT PARA CHURRASCO |
| 09601 | TABULEIRO |
| 09701 | PONCHEIRA |
| 09801 | CESTO DE LIXO (PLASTICO) |
| 09901 | LIXEIRA METALICA |
| 99901 | AGREGADO |
| 00101 | BIJUTERIA |
| 00102 | COLAR |
| 00103 | PINGENTE |
| 00104 | BRINCO |
| 00105 | BROCHE |
| 00106 | GARGANTILHA |
| 00107 | ANEL |
| 00201 | TRANSPORTE DE ANIMAIS |
| 00301 | OCULOS SEM GRAU |
| 00302 | OCULOS DE SOL SEM GRAU |
| 00401 | LENTE DE CONTATO COLORIDA |
| 00501 | TRANSPORTE DE MERCADORIAS |
| 00601 | CALENDARIO |
| 00701 | MASCARA DE CARNAVAL |
| 00801 | ADESIVO PLASTICO OU PANFLETO |
| 00802 | PANFLETO |
| 00803 | ADESIVO PLASTICO |
| 00804 | DECALQUES |
| 00901 | MOEDA (HOBBY) |
| 01001 | SELO (HOBBY) |
| 01002 | ALBUM DE SELOS |
| 01003 | ALBUM DE CARTOES |
| 01004 | CARTAO TELEFONICO(HOBBY) |
| 01005 | OUTROS (HOBBY) |
| 01101 | BATERIA DE RELOGIO, BRINQUEDO, APARELHO AUDITIVO, ETC. |
| 01102 | BATERIA DE RELOGIO |
| 01103 | BATERIA DE BRINQUEDO |
| 01104 | BATERIA APARELHO AUDITIVO |
| 01201 | PARAFINA |
| 01301 | CHAVEIRO (PRODUTO) |
| 01401 | BOMBA E CUIA OU PORONGO DE CHIMARRAO |
| 01402 | BOMBA DE CHIMARRAO |
| 01403 | CUIA DE CHIMARRAO |
| 01404 | PORONGO DE CHIMARRAO |
| 01501 | PINCEL, TINTA E VERNIZ PARA PINTURA DE TELA, VASO, ETC. |
| 01502 | PINCEL |
| 01503 | TINTA PARA PINTURA DE TELA |
| 01504 | VERNIZ PARA PINTURA DE TELA |
| 01601 | MONOCULO |
| 01701 | CAVALETE DE PINTURA DE TELA |
| 01801 | MASCARA PROTETORA (PROTECAO PROFISSIONAL) |
| 01901 | PEDRA DE AMOLAR |
| 02001 | FLAMULA |
| 02101 | CANIVETE |
| 02201 | PRENDEDOR DE GRAVATA |
| 02301 | COFRE |
| 02401 | TELA PARA PINTURA |
| 02501 | PORTA-OCULOS |
| 02601 | RAQUETE DE MATAR MOSQUITO |
| 99901 | AGREGADO |
| 00101 | ONIBUS URBANO |
| 00201 | TREM |
| 00301 | AVIAO |
| 00401 | NAVIO |
| 00501 | COMBUSTIVEL DE VEICULO |
| 00601 | ALIMENTACAO |
| 00602 | BEBIDAS |
| 00603 | RESTAURANTES |
| 00604 | BARES |
| 00605 | LANCHONETES |
| 00606 | TENDAS |
| 00607 | TRAILERS |
| 00608 | QUIOSQUES |
| 00609 | CARROCINHAS |
| 00701 | ALOJAMENTO |
| 00702 | HOTEIS |
| 00703 | MOTEIS |
| 00704 | POUSADAS |
| 00705 | PENSOES |
| 00706 | DORMITORIOS |
| 00707 | ACAMPAMENTOS |
| 00708 | ALBERGUES |
| 00801 | EXCURSAO (EXCETO ESCOLAR) |
| 00901 | GARAGEM |
| 00902 | ESTACIONAMENTO |
| 01001 | ALUGUEL DE VEICULO SEM CONDUTOR |
| 01101 | TAXI |
| 01201 | BARCO |
| 01202 | FERRY-BOAT |
| 01203 | BALSA |
| 01301 | PEDAGIO |
| 01401 | METRO |
| 01501 | PASSAGEM DE CAMINHAO |
| 01502 | CAMINHAO |
| 01503 | PAU-DE-ARARA |
| 01601 | PASSAGEM EM AUTOMOVEL |
| 01602 | PASSAGEM EM AUTOMOVEL UTILITARIO |
| 01603 | AUTOMOVEL UTILITARIO TIPO VAN |
| 01604 | KOMBI |
| 01605 | TRANSPORTE ALTERNATIVO |
| 01701 | COLONIA DE FERIAS |
| 01801 | TAXA ALFANDEGARIA |
| 01802 | DOCUMENTACAO |
| 01901 | GUARDA-VOLUMES |
| 02001 | ONIBUS INTERMUNICIPAL |
| 02101 | ONIBUS INTERNACIONAL |
| 02201 | ONIBUS INTERESTADUAL |
| 02301 | CARREGADOR DE BAGAGEM |
| 02401 | GUIA TURISTICO |
| 02501 | ALUGUEL DE IMOVEIS POR TEMPORADA |
| 02601 | ATIVIDADES CULTURAIS (INGRESSO) |
| 02602 | CINEMA (INGRESSO) |
| 02603 | VIDEOTECAS (INGRESSO) |
| 02604 | CINEMATECAS (INGRESSO) |
| 02605 | TEATROS (INGRESSO) |
| 02606 | SHOWS (INGRESSO) |
| 02607 | CIRCOS (INGRESSO) |
| 02608 | RODEIOS (INGRESSO) |
| 02609 | DISCOTECAS (INGRESSO) |
| 02610 | DANCETERIAS (INGRESSO) |
| 02611 | BOATES (INGRESSO) |
| 03301 | ATIVIDADES DESPORTIVAS (INGRESSO) |
| 03302 | ESTADIOS (INGRESSO) |
| 03303 | GINASIOS (INGRESSO) |
| 03304 | QUADRAS DE TENIS (INGRESSO) |
| 03305 | HIPODROMOS (INGRESSO) |
| 03306 | CAMPOS DE GOLFE (INGRESSO) |
| 03307 | CIRCUITOS AUTOMOBILISTICOS (INGRESSO) |
| 03308 | PESCA |
| 03401 | BOLICHE |
| 03501 | BINGO |
| 03601 | LOCACAO DE BICICLETAS |
| 03701 | PEDALINHOS |
| 03801 | PARQUES DE DIVERSAO |
| 03901 | FLIPERAMA E SIMILARES |
| 04001 | PACOTES TURISTICOS NACIONAIS |
| 04101 | PACOTES TURISTICOS INTERNACIONAIS |
| 04201 | DESPESAS OPCIONAIS COM AGENCIAS DE VIAGEM |
| 99901 | AGREGADO |
| 00101 | PLANO DE ASSISTENCIA MEDICA (EMPRESA) |
| 00102 | FUNDO DE PARTICIPACAO EM PLANO/SEGURO-SAUDE (EMPRESA) |
| 00103 | POUPANCA COMPLEMENTAR DE PLANO/SEGURO-SAUDE (EMPRESA) |
| 00104 | COMPLEMENTACAO DE PLANO/SEGURO-SAUDE (EMPRESA) |
| 00105 | PLANO SEGURO-SAUDE (EMPRESA) |
| 00201 | PLANO EXCLUSIVAMENTE ODONTOLOGICO (PARTICULAR) |
| 00202 | PLANO ODONTOLOGICO (PARTICULAR) |
| 00203 | PLANO DE ASSISTENCIA ODONTOLOGICA (PARTICULAR) |
| 00301 | MENSALIDADE DE CLINICA |
| 00401 | ATENDIMENTO EM PRONTO-SOCORRO/PRONTO-ATENDIMENTO |
| 00402 | CAUTERIZACAO, CURATIVO, NEBULIZACAO, ETC. |
| 00403 | TRATAMENTO AMBULATORIAL |
| 00404 | NEBULIZACAO |
| 00405 | CURATIVO |
| 00501 | SERVICOS DE CIRURGIA, ANESTESIA E OBSTETRICIA |
| 00502 | ANESTESISTA PARA CIRURGIA E PARTO |
| 00503 | OBSTETRA PARA CIRURGIA E PARTO |
| 00504 | CIRURGIAO PARA OPERACAO E PARTO |
| 00505 | CIRURGIA (QUALQUER TIPO) |
| 00506 | PROCEDIMENTO CIRURGICO |
| 00507 | IMPLANTE DE CABELO (TRATAMENTO) |
| 00508 | TRATAMENTO PARA IMPLANTE DE CABELO |
| 00509 | INSTRUMENTADOR CIRURGICO |
| 00510 | RAIO LASER (APLICACAO) |
| 00511 | APLICACAO DE RAIO LASER |
| 00512 | FOTOGRAFIA RELATIVA A CIRURGIA |
| 00601 | HOSPITALIZACAO |
| 00602 | INTERNACAO EM PRONTO SOCORRO |
| 00603 | INTERNACAO EM MATERNIDADE |
| 00604 | INTERNACAO EM HOSPITAL |
| 00701 | AMBULANCIA E UTI MOVEL (REMOCAO) |
| 00702 | AMBULANCIA (REMOCAO) |
| 00801 | VACINACAO |
| 00901 | CONSULTA MEDICA COM CLINICO GERAL |
| 00902 | CONSULTA MEDICA GERAL |
| 01001 | CONSULTA MEDICA PEDIATRICA |
| 01101 | CONSULTA MEDICA GINECO-OBSTETRICIA |
| 01102 | EXAME PRE-NATAL |
| 01103 | EXAMES PREVENTIVOS |
| 01104 | EXAME GINECOLOGICO |
| 01105 | EXAME PREVENTIVO DE CANCER |
| 01106 | CONSULTA MEDICA GINECOLOGICA |
| 01107 | CONSULTA MEDICA OBSTETRICA |
| 01201 | OUTRAS CONSULTAS DIFERENTES DE GERAL, PEDIATRICA E GINECO-OBSTETRICIA |
| 01202 | CONSULTA MEDICA CARDIOLOGICA |
| 01203 | CONSULTA MEDICA OFTALMOLOGICA |
| 01204 | CONSULTA MEDICA ORTOPEDICA |
| 01205 | CONSULTA MEDICA NEUROLOGICA |
| 01206 | CONSULTA MEDICA PSIQUIATRICA |
| 01207 | CONSULTA MEDICA DERMATOLOGICA |
| 01208 | CONSULTA MEDICA GASTROENTEROLOGICA |
| 01209 | CONSULTA MEDICA (ALERGISTA) |
| 01210 | CONSULTA MEDICA GERIATRICA |
| 01211 | CONSULTA MEDICA HOMEOPATICA |
| 01212 | CONSULTA MEDICA NEFROLOGICA (RENAIS) |
| 01213 | CONSULTA MEDICA ONCOLOGICA |
| 01214 | CONSULTA MEDICA OTOLOGICA |
| 01215 | CONSULTA MEDICA OTORRINO |
| 01216 | CONSULTA MEDICA PNEUMOLOGICA |
| 01217 | CONSULTA MEDICA REUMATOLOGICA |
| 01218 | CONSULTA MEDICA ENDOCRINOLOGICA |
| 01219 | CONSULTA MEDICA PROTOLOGICA |
| 01220 | CONSULTA MEDICA UROLOGICA |
| 01221 | CONSULTA MEDICA ANGIOLOGICA |
| 01222 | CONSULTA MEDICA CIRURGIAO PLASTICO |
| 01223 | CONSULTA MEDICA HEMATOLOGICA |
| 01301 | EXAME DE LABORATORIO |
| 01302 | EXAME DE BACILO |
| 01303 | EXAME DE FEZES |
| 01304 | EXAME DE SANGUE |
| 01305 | EXAME DE URINA |
| 01306 | EXAME PRE-NUPCIAL |
| 01307 | BIOPSIA (EM GERAL) |
| 01308 | EXAMES EM GERAL |
| 01309 | ESPERMOGRAMA |
| 01401 | TOMOGRAFIA |
| 01402 | MAMOGRAFIA |
| 01403 | DENSITOMETRIA OSSEA |
| 01501 | RESSONANCIA MAGNETICA |
| 01601 | ULTRASSONOGRAFIA |
| 01602 | CARDIOTOCOGRAFIA |
| 01603 | ECOGRAFIA |
| 01604 | ECOCARDIOGRAMA |
| 01605 | ECOCARDIOGRAFIA |
| 01606 | ECOENDOSCOPIA |
| 01607 | EXAME DE CATETERISMO |
| 01701 | RADIOGRAFIA |
| 01702 | EXAME RADIOLOGICO |
| 01801 | ELETROCARDIOGRAMA E OUTROS METODOS GRAFICOS |
| 01802 | ELETROCARDIOGRAMA |
| 01803 | ELETROENCEFALOGRAMA |
| 01804 | EXAME ERGOMETRICO |
| 01805 | EXAME AUDIOMETRICO |
| 01901 | ENDOSCOPIA |
| 01902 | COLONOSCOPIA |
| 02001 | QUIMIOTERAPIA |
| 02002 | TRATAMENTO QUIMIOTERAPICO |
| 02101 | SERVICO DE HEMOTERAPIA (TRANSFUSAO) |
| 02102 | SANGUE HUMANO |
| 02201 | DIALISE |
| 02202 | HEMODIALISE |
| 02301 | PROTESE E APARELHO DENTARIO |
| 02302 | APARELHO ORTODONTICO |
| 02303 | PIVO |
| 02304 | DENTADURA |
| 02401 | TRATAMENTO DENTARIO |
| 02402 | TRATAMENTO DE CANAL DENTARIO |
| 02403 | OBTURACAO DENTARIA |
| 02404 | EXTRACAO DENTARIA |
| 02405 | COLOCACAO DE JAQUETA DENTARIA |
| 02406 | MANUTENCAO DE APARELHO DENTARIO |
| 02407 | MANUTENCAO DE APARELHO ORTODONTICO |
| 02408 | IMPLANTE DE DENTE |
| 02409 | CONSULTA ODONTOLOGICA |
| 02501 | SERVICOS DE ENFERMAGEM |
| 02502 | TRATAMENTO DE ENFERMAGEM |
| 02503 | ENFERMEIRA |
| 02601 | ACOMPANHANTE (CUIDADOR) |
| 02602 | ACOMPANHANTE DE QUARTO HOSPITALAR |
| 02701 | SERVICO DE INTERNACAO E CUIDADO DOMICILIAR |
| 02801 | NUTRICIONISTA |
| 02802 | TRATAMENTO DIETETICO |
| 02803 | CONSULTA MEDICA NUTRICIONISTA |
| 02901 | SERVICOS DE NUTRICAO ENTERAL E PARENTAL |
| 03001 | PSICOLOGO E PSICOTERAPEUTA |
| 03002 | TRATAMENTO PSICOLOGICO |
| 03101 | TRATAMENTO FISIOTERAPICO E DE TERAPIA OCUPACIONAL |
| 03102 | TRATAMENTO FISIOTERAPICO |
| 03201 | TRATAMENTO FONOAUDIOLOGICO |
| 03301 | MASSAGEM PARA FINS DE SAUDE SHIATZU DO IN E OUTROS TRATAMENTOS ALTERNATIVOS |
| 03302 | TRATAMENTO MASSAGISTICO |
| 03303 | TRATAMENTO HIDROTERAPICO |
| 03304 | HIDROTERAPIA (TRATAMENTO) |
| 03401 | ACUPUNTURA |
| 03402 | TRATAMENTO ACUPUNTURAL |
| 03403 | ACUPUNTURA (TRATAMENTO) |
| 03501 | CURANDEIRO, CURIOSO E PARTEIRA |
| 03502 | CURANDEIRO E CURIOSO |
| 03503 | CURIOSO |
| 03504 | PARTEIRA |
| 03601 | CLINICA E RESIDENCIA PARA IDOSOS |
| 03701 | CLINICA PARA DEFICIENTES FISICOS OU CONVALESCENTES |
| 03801 | CLINICA PSIQUIATRICA OU PARA DEPENDENTES QUIMICOS |
| 03901 | ARMACAO DE OCULOS |
| 04001 | LENTES DE OCULOS E DE CONTATO |
| 04002 | LENTE DE OCULOS (DE GRAU) |
| 04003 | OCULOS DE GRAU (ARMACAO+LENTE) |
| 04004 | LENTE DE CONTATO |
| 04101 | ARTIGOS ORTOPEDICOS E PROTESES DIVERSAS |
| 04102 | ARTIGOS ORTOPEDICOS E OUTROS |
| 04103 | SAPATO ORTOPEDICO |
| 04104 | MULETA |
| 04105 | PALMILHA (ORTOPEDICA) |
| 04106 | CAMA HOSPITALAR |
| 04107 | CADEIRA DE RODAS |
| 04108 | BOTA ORTOPEDICA |
| 04109 | OUTROS ARTIGOS MEDICOS |
| 04110 | SANDALIA ORTOPEDICA |
| 04111 | APARELHO DE SURDEZ |
| 04112 | FONOAUDITIVOS |
| 04113 | TORNOZELEIRA ORTOPEDICA |
| 04114 | CINTA TERMICA (CORRETIVA) |
| 04115 | MEIA ELASTICA (PARA VARIZES) |
| 04116 | CARRINHO PARA DEFICIENTE FISICO |
| 04117 | SUSPENSORIO ESCROTAL |
| 04118 | PROTETOR DE JOELHO |
| 04119 | LENTE INTRAOCULAR |
| 04120 | CRISTALINO (LENTE INTRAOCULAR) |
| 04201 | ALUGUEL E REPARO DE APARELHO MEDICO |
| 04202 | ALUGUEL DE APARELHO MEDICO |
| 04203 | ALUGUEL DE EQUIPAMENTO DE MASSAGEM |
| 04204 | ALUGUEL DE CADEIRA DE RODAS |
| 04205 | ALUGUEL DE CAMA HOSPITALAR |
| 04206 | CONSERTO E REPARO DE ARTIGO E APARELHO MEDICOS |
| 04207 | OXIGENIO (DESPESA COM SAUDE) |
| 04208 | TUBO OU CILINDRO DE OXIGENIO (DESPESA COM SAUDE) |
| 04209 | CILINDRO OU TUBO DE OXIGENIO (DESPESA COM SAUDE) |
| 04301 | EXAMES EM POSTOS OU PROFISSIONAIS VOLANTES |
| 04302 | PRESSAO ARTERIAL (EM POSTOS OU PROFISSIONAIS VOLANTES) |
| 04303 | EXAME DE PRESSAO ARTERIAL (EM POSTOS OU PROFISSIONAIS VOLANTES) |
| 04304 | VIGILANTES DO PESO (PROFISSIONAIS VOLANTES) |
| 04305 | PESAGEM (PROFISSIONAIS VOLANTES) |
| 04401 | PLANO EXCLUSIVAMENTE ODONTOLOGICO (EMPRESA) |
| 04402 | PLANO ODONTOLOGICO (EMPRESA) |
| 04403 | PLANO DE ASSISTENCIA ODONTOLOGICA (EMPRESA) |
| 04501 | PLANO DE ASSISTENCIA MEDICA (PARTICULAR) |
| 04502 | PLANO SEGURO-SAUDE (PARTICULAR) |
| 04601 | CINTILOGRAFIA OSSEA |
| 04602 | CINTILOGRAFIA |
| 04701 | PODOLOGO |
| 99901 | AGREGADO |
| 00101 | LUBRIFICACAO (COM OU SEM LAVAGEM) |
| 00102 | LUBRIFICACAO COM LAVAGEM |
| 00103 | LUBRIFICACAO SEM LAVAGEM |
| 00201 | OLEO LUBRIFICANTE (COMPLEMENTACAO OU TROCA) |
| 00202 | COMPLEMENTACAO DE OLEO LUBRIFICANTE |
| 00203 | TROCA DE OLEO LUBRIFICANTE |
| 00301 | PECA ELETRICA OU MECANICA |
| 00302 | PECA MECANICA |
| 00303 | PECA ELETRICA |
| 00401 | CAMARA E PNEU |
| 00402 | PNEU |
| 00403 | CAMARA |
| 00501 | CONSERTO E OUTROS SERVICOS (MAO-DE-OBRA) |
| 00502 | ACESSORIO (MAO-DE-OBRA) |
| 00503 | MECANICO (MAO-DE-OBRA) |
| 00504 | BORRACHEIRO (MAO-DE-OBRA) |
| 00505 | INSTALACAO DE SOM (MAO-DE-OBRA) |
| 00506 | ALINHAMENTO DE RODAS |
| 00507 | BALANCEAMENTO DE RODAS |
| 00508 | GEOMETRIA DE RODAS |
| 00509 | CONSERTO E REPARO DE VIDRO |
| 00510 | REPARO DE ARRANHOES DE PINTURA |
| 00511 | CARGA DE BATERIA |
| 00512 | RECARGA DE BATERIA |
| 00513 | GAS DE AR CONDICIONADO |
| 00514 | TRANSPORTE DE VEICULOS |
| 00515 | CONVERSAO DE MOTOR PARA GAS |
| 00601 | CONSERTO DE BICICLETA (PECA+MAO-DE-OBRA) |
| 00602 | CONSERTO DE BICICLETA (PECA) |
| 00603 | CONSERTO DE BICICLETA (MAO-DE-OBRA) |
| 00701 | BATERIA |
| 00702 | BATERIA DE AUTOMOVEL |
| 00703 | BATERIA DE MOTOCICLETA |
| 00801 | OUTRAS PECAS, ACESSORIOS E MATERIAIS (VEICULOS MOTORIZADOS) |
| 00802 | RETROVISOR |
| 00803 | TAPETE |
| 00804 | PARA-CHOQUE |
| 00805 | ARO DE FAROL |
| 00806 | FAROL |
| 00807 | ALARME E TRANCA |
| 00808 | TRANCA |
| 00809 | ALARME |
| 00810 | RADIO |
| 00811 | TOCA-FITAS |
| 00812 | RADIO TOCA-FITAS |
| 00813 | ESTOPA |
| 00814 | SHAMPOO |
| 00815 | LIMPA-PARA-BRISA |
| 00816 | CERA |
| 00817 | POLIDOR |
| 00818 | TINTA |
| 00819 | TINTA DE PINTURA |
| 00820 | LIXA DE FERRO |
| 00821 | CARROCERIA DE CAMIONETA |
| 00822 | SOLVENTE |
| 00823 | MOTOR DE LANCHA |
| 00824 | MASSA DE LANTERNAGEM |
| 00825 | PLASTIC |
| 00826 | ESTOFAMENTO |
| 00827 | FLUIDO DE FREIO |
| 00828 | OLEO DE FREIO |
| 00829 | MOTOR DE MOTOCICLETA |
| 00830 | CD PLAYER |
| 00831 | COMPACT DISC PLAYER |
| 00832 | INSULFILM |
| 00833 | CADEIRINHA INFANTIL PARA CARRO |
| 00834 | GPS VEICULAR |
| 00835 | DVD PARA CARRO |
| 01101 | AR CONDICIONADO |
| 01201 | OUTRAS PECAS E ACESSORIOS (BICICLETA) |
| 01202 | BORRACHA DE FREIO DE BICICLETA |
| 01203 | CABO DE FREIO PARA BICICLETA |
| 01204 | ARO PARA BICICLETA CROMADO |
| 01205 | ARO E RAIO PARA BICICLETA (CROMADO) |
| 01206 | RAIO PARA BICICLETA |
| 01207 | BOMBA PARA ENCHER PNEU (BICICLETA) |
| 01208 | CADEIRA DE BICICLETA |
| 01209 | BACIA DE BICICLETA |
| 01210 | EIXO DE BICICLETA |
| 01211 | CORRENTE DE BICICLETA |
| 01212 | PNEU DE BICICLETA |
| 01213 | CAMARA DE BICICLETA |
| 01301 | REBOQUE |
| 01302 | GUINCHO (REBOQUE) |
| 01701 | PINTURA (MATERIAL E MAO-DE-OBRA) |
| 01901 | CAPACETE DE MOTOQUEIRO |
| 02701 | MOTOR DE AUTOMOVEL |
| 02901 | AMPLIFICADOR |
| 03501 | RADIO COM CD |
| 04001 | ADITIVOS |
| 04002 | AGUA DESTILADA DE BATERIA |
| 04201 | CONSERTO E REVISAO (PECA+MAO-DE-OBRA) |
| 99901 | AGREGADO |
| 00101 | CARTORIO |
| 00102 | REGISTRO DE IMOVEL |
| 00103 | CERTIDAO DE CASAMENTO |
| 00104 | INVENTARIO |
| 00105 | ATESTADO DE OBITO |
| 00106 | CERTIDAO DE NASCIMENTO |
| 00107 | JUIZ DE PAZ |
| 00108 | ESCRITURA |
| 00109 | AUTENTICACAO DE DOCUMENTO |
| 00201 | ADVOGADO |
| 00301 | DESPACHANTE |
| 00401 | DESENHISTA |
| 00501 | DECORADOR |
| 00601 | CONTADOR |
| 00701 | TAXAS DE ORGAOS PUBLICOS |
| 00702 | ATESTADO DE BONS ANTECEDENTES |
| 00703 | CERTIDAO DA POLINTER |
| 00704 | DOCUMENTACAO (EXCETO DE HABILITACAO E DE VEICULO) |
| 00705 | OFICIAL DE JUSTICA |
| 00801 | ENGENHEIRO |
| 00901 | CORRETOR |
| 01001 | DATILOGRAFO |
| 01002 | DATILOGRAFIA COMPUTADORIZADA |
| 01003 | PROFISSIONAL DE DESIGN |
| 01101 | TOPOGRAFO |
| 01102 | AGRIMENSOR |
| 01201 | COMISSAO DE IMOBILIARIA |
| 01202 | ADMINISTRADORA DE IMOVEL (COMISSAO) |
| 01203 | COMISSAO DE CORRETOR DE IMOVEL |
| 01301 | FISCALIZACAO DE OBRA (PREFEITURA) |
| 01302 | FISCAL DE OBRA (PREFEITURA) |
| 01401 | ANUNCIO CLASSIFICADO |
| 01402 | PROMESSA EM JORNAL |
| 01501 | TRADUTOR JURAMENTADO |
| 01601 | DEFESA DO CONSUMIDOR (IDEC) |
| 01602 | IDEC (INSTITUTO DE DEFESA DO CONSUMIDOR) |
| 01603 | IDEC |
| 01701 | AVALIADOR DE IMOVEL |
| 01801 | INDENIZACAO A TERCEIROS |
| 01901 | TECNICO EM AGRICULTURA |
| 02001 | CONSORCIO DE DINHEIRO |
| 02101 | MULTA DE ALISTAMENTO MILITAR |
| 02201 | ANUIDADE DE CARTAO DE CREDITO |
| 02301 | TALAO DE CHEQUES |
| 02401 | EXTRATO BANCARIO |
| 02501 | CADASTRO BANCARIO |
| 02502 | ABERTURA DE CREDITO |
| 02503 | TARIFA DE MANUTENCAO DE CHEQUE ESPECIAL |
| 02504 | RENOVACAO DE CHEQUE ESPECIAL |
| 02505 | TAXA DE TRANSFERENCIA DE FINANCIAMENTO |
| 02601 | TRANSFERENCIA INTERBANCARIA |
| 02602 | DOC PESSOAL (TRANSFERENCIA INTERBANCARIA) |
| 02603 | TED PESSOAL (TRANSFERENCIA INTERBANCARIA) |
| 02701 | CARTAO ESPECIAL (TAXA) |
| 02702 | SEGUNDA VIA DE CARTAO ESPECIAL |
| 02801 | TARIFA DE CONTA BANCARIA |
| 02802 | MANUTENCAO DE CONTA BANCARIA |
| 02803 | PACOTE DE SERVICO DE CONTA BANCARIA |
| 02901 | TAXA DE SAQUE ELETRONICO |
| 03001 | TAXA PARA SUSTAR CHEQUES |
| 03101 | TAXA DE DEVOLUCAO DE CHEQUE |
| 03201 | ENCARGOS FINANCEIROS DE CHEQUE ESPECIAL |
| 03202 | JUROS DE CHEQUE ESPECIAL |
| 03301 | SEGURO DE CARTAO DE CREDITO |
| 03501 | ARQUITETO |
| 03601 | ENCARGOS FINANCEIROS DE CARTAO DE CREDITO |
| 03602 | JUROS DE CARTAO DE CREDITO |
| 03801 | SEGUNDA VIA CARTAO DE DEBITO |
| 03802 | SEGUNDA VIA CARTAO DE POUPANCA |
| 04201 | CHEQUE VISADO |
| 04701 | COPIA DE MICROFILME DE CHEQUE |
| 99901 | AGREGADO |
| 00101 | CASAMENTO |
| 00102 | ALUGUEL DE BUFFET DE CASAMENTO |
| 00103 | CONVITE DE CASAMENTO |
| 00104 | CASAMENTO EM TEMPLO RELIGIOSO |
| 00105 | CASAMENTO EM IGREJA |
| 00106 | ALUGUEL DE SALAO PARA CASAMENTO |
| 00107 | CURSO DE NOIVOS |
| 00108 | ALUGUEL DE MESA PARA FESTA DE CASAMENTO |
| 00201 | OUTRAS CERIMONIAS RELIGIOSAS |
| 00202 | BATIZADO |
| 00203 | COMUNHAO |
| 00204 | OFERENDA RELIGIOSA |
| 00205 | REUNIAO DE CULTO RELIGIOSO |
| 00206 | MISSA |
| 00207 | PROMESSA RELIGIOSA |
| 00301 | FUNERAL E ARTIGOS RELIGIOSOS |
| 00302 | ARTIGOS RELIGIOSOS |
| 00303 | CREMACAO |
| 00304 | COROA DE FLORES |
| 00305 | TAXA DE FUNERARIA |
| 00306 | CAIXAO |
| 00307 | URNA FUNERARIA |
| 00308 | OUTRAS DESPESAS COM FUNERAL |
| 00309 | FUNERAL |
| 00310 | EXUMACAO |
| 00401 | CARTOMANTE |
| 00501 | FOTOGRAFIAS DE CERIMONIAS (FOTOGRAFO PROFISSIONAL) |
| 00502 | FOTOS DE CERIMONIAS (FOTOGRAFO PROFISSIONAL) |
| 00503 | FOTOGRAFIAS DE CERIMONIA |
| 00601 | ANIVERSARIO |
| 00602 | ALUGUEL DE SALAO PARA FESTA DE ANIVERSARIO |
| 00603 | ALUGUEL DE MESA PARA FESTA DE ANIVERSARIO |
| 00604 | ALUGUEL DE BUFFET PARA FESTA DE ANIVERSARIO |
| 00701 | BAILE |
| 00801 | OUTRAS FESTAS OU RECEPCOES |
| 00802 | CERIMONIA MILITAR |
| 00901 | FILMAGEM DE CERIMONIA |
| 00902 | EDICAO DE FITA |
| 01001 | JOGO DE BUZIOS (PRATICA RELIGIOSA) |
| 01002 | JOGO DE BUZIOS |
| 01101 | COMEMORACAO NATALINA |
| 01201 | BATISTERIO |
| 01301 | FESTA DE ANO NOVO |
| 01401 | PRODUTOS DE CARNAVAL |
| 01402 | DESFILE DE ESCOLA DE SAMBA |
| 01403 | ABADA |
| 01404 | BAILE DE CARNAVAL |
| 01405 | CARNAVAL (INGRESSO) |
| 01601 | FESTA COMUNITARIA |
| 01701 | FESTA JUNINA (INGRESSO) |
| 01801 | QUERMESSE (INGRESSO) |
| 99901 | AGREGADO |
| 00101 | JOIAS (ALIANCA, ANEL, ETC.) |
| 00102 | PULSEIRA (JOIA) |
| 00103 | CORDAO (JOIA) |
| 00104 | ANEL (JOIA) |
| 00105 | ALIANCA (JOIA) |
| 00106 | SEMIJOIA |
| 00201 | RELOGIO DE PULSO DE CRIANCA |
| 00301 | RELOGIO DE PULSO DE HOMEM |
| 00401 | RELOGIO DE PULSO DE MULHER |
| 00501 | CANETA RELOGIO |
| 00601 | CANETA DE OURO OU PRATA |
| 00701 | ISQUEIRO DE OURO OU PRATA |
| 00801 | RELOGIO DE BOLSO |
| 00901 | TELEFONE VIRTUAL (APARELHO) |
| 00902 | MOBI (APARELHO) |
| 00903 | PAGER (APARELHO) |
| 00904 | BIP (APARELHO) |
| 01001 | CARTAO DE PRATA |
| 01101 | RELOGIO DE DEDO |
| 01201 | TELEFONE CELULAR (APARELHO) |
| 01301 | ACESSORIOS DE TELEFONE CELULAR |
| 01302 | BATERIA DE TELEFONE CELULAR |
| 01303 | CARREGADOR DE ENERGIA PARA CELULAR |
| 01304 | CHIP DE TELEFONE CELULAR |
| 01305 | CAPA DE CELULAR |
| 99901 | AGREGADO |
| 00101 | AQUISICAO DE OUTROS IMOVEIS A VISTA OU A PRAZO (PARCELAS, TAXAS, ETC.) |
| 00201 | IMPOSTO DE TRANSMISSAO DE OUTROS IMOVEIS (AQUISICAO A VISTA OU A PRAZO) |
| 00202 | ESCRITURA DE OUTROS TERRENOS OU IMOVEIS |
| 00301 | CONSORCIO DE OUTROS IMOVEIS (PRESTACAO) |
| 00401 | LOTE DOADO PELA PREFEITURA MUNICIPAL (OUTROS IMOVEIS) |
| 00501 | TAXAS DE LOCACAO DE OUTROS IMOVEIS (CONTRATO, DEPOSITO, MULTA, ETC.) |
| 00502 | CONTRATO DE LOCACAO DE OUTROS IMOVEIS |
| 00503 | DEPOSITO DE LOCACAO DE OUTROS IMOVEIS |
| 00504 | MULTA DE LOCACAO DE OUTROS IMOVEIS |
| 00505 | RESCISAO DE CONTRATO DE ALUGUEL DE OUTROS IMOVEIS |
| 00506 | FIANCA DE OUTROS IMOVEIS |
| 00601 | ALUGUEL DE GARAGEM DE OUTROS IMOVEIS |
| 00701 | CONDOMINIO DE OUTROS IMOVEIS |
| 00801 | IMPOSTO PREDIAL E TERRITORIAL URBANO (IPTU) DE OUTROS IMOVEIS |
| 00901 | SEGURO CONTRA ROUBO E INCENDIO DE OUTROS IMOVEIS |
| 00902 | SEGURO CONTRA ROUBO E ACIDENTES DE OUTROS IMOVEIS |
| 01001 | ENERGIA ELETRICA DE OUTROS IMOVEIS (KWH) |
| 01101 | TELEFONE RESIDENCIAL DE OUTROS IMOVEIS |
| 01201 | GAS ENCANADO DE OUTROS IMOVEIS (M3) |
| 01301 | AGUA E ESGOTO DE OUTROS IMOVEIS (M3) |
| 01401 | GAS DE BUTIJAO DE OUTROS IMOVEIS (KG) |
| 01501 | AGUA DE OUTROS IMOVEIS (CARRO PIPA) |
| 01601 | GASOLINA DE OUTROS IMOVEIS (COMBUSTIVEL DOMESTICO) |
| 01701 | OLEO DIESEL DE OUTROS IMOVEIS (COMBUSTIVEL DOMESTICO) |
| 01801 | ACESSO A INTERNET DE OUTROS IMOVEIS |
| 01901 | ACESSO A TV A CABO DE OUTROS IMOVEIS |
| 01902 | TAXA DE TELEVISAO POR ASSINATURA DE OUTROS IMOVEIS |
| 02001 | TAXA (INCENDIO, LIXO, SEGURANCA, ETC.) DE OUTROS IMOVEIS |
| 02002 | TAXA DE MELHORAMENTOS DE OUTROS IMOVEIS |
| 02003 | TAXA DE SEGURANCA DE OUTROS IMOVEIS |
| 02004 | TAXA DE LIXO DE OUTROS IMOVEIS |
| 02005 | TAXA DE INCENDIO DE OUTROS IMOVEIS |
| 02006 | COLETA DE LIXO DE OUTROS IMOVEIS |
| 02007 | TAXA DE INSTALACAO DE HIDROMETRO DE OUTROS IMOVEIS |
| 02008 | TAXA DE INSTALACAO DE RELOGIO DE AGUA DE OUTROS IMOVEIS |
| 02009 | TAXA DE HABITE |
| 02010 | MULTAS DIVERSAS DE OUTROS IMOVEIS |
| 02101 | AQUISICAO DE TITULO DE CLUBE |
| 02102 | TITULO DE CLUBE (AQUISICAO) |
| 02201 | IMPOSTO SOBRE PROPRIEDADE TERRITORIAL RURAL (IPTR) DE OUTROS IMOVEIS |
| 02202 | INCRA DE OUTROS IMOVEIS (IMPOSTO) |
| 02203 | PAGAMENTO DE SPU (SERVICO DE PATRIMONIO DA UNIAO) DE OUTROS IMOVEIS |
| 02301 | MUDANCA |
| 02401 | CERTIFICADO DE CADASTRO DE OUTROS IMOVEIS |
| 02501 | ALUGUEL DE OUTROS IMOVEIS |
| 02601 | AQUISICAO DE OUTROS TERRENOS OU LOTES A VISTA OU A PRAZO (PARCELAS, TAXAS, ETC.) |
| 02701 | VAGA CATIVA (ESTACIONAMENTO) |
| 09401 | VALOR DE OUTRO IMOVEL ADQUIRIDO EM PRIMEIRA LOCACAO (NOVO E VALOR TOTAL) |
| 09501 | VALOR DE OUTRO IMOVEL ADQUIRIDO USADO (VALOR TOTAL) |
| 99901 | AGREGADO |
| 00101 | CONSELHO E ASSOCIACAO DE CLASSE |
| 00102 | ASSOCIACAO DE CLASSE |
| 00103 | ASSOCIACAO DE TRABALHADORES |
| 00201 | DOACOES EM DINHEIRO (IGREJA, ORFANATO, INSTITUICAO DE CARIDADE, MUSEU, ETC.) |
| 00202 | MUSEU (DOACAO EM DINHEIRO) |
| 00203 | DOACAO PARA MUSEU EM DINHEIRO |
| 00204 | DOACAO EM DINHEIRO PARA INSTITUICAO DE CARIDADE |
| 00301 | CAIXA ESCOLAR (CONTRIBUICAO EM DINHEIRO) |
| 00302 | CONTRIBUICAO PARA CAIXA ESCOLAR EM DINHEIRO |
| 00401 | MESADA E PRESENTE EM DINHEIRO PARA OUTRA UC |
| 00402 | PRESENTE DOACAO EM DINHEIRO PARA OUTRA UC |
| 00403 | MESADA, DINHEIRO PARA OUTRA UC |
| 00404 | DOACAO EM DINHEIRO PARA OUTRA UC |
| 00501 | PENSAO ALIMENTICIA EM DINHEIRO |
| 00601 | PREVIDENCIA PRIVADA ABERTA |
| 00602 | JOIA (TAXA DE INGRESSO) DE PREVIDENCIA PRIVADA |
| 00603 | TAXA DE INGRESSO (JOIA) DE PREVIDENCIA PRIVADA |
| 00604 | PREVIDENCIA PRIVADA FECHADA |
| 00701 | SEGURO DE ACIDENTES PESSOAIS |
| 00801 | SEGURO DE VIDA |
| 00901 | FIANCA (PRESO) |
| 01001 | TAXA DE TRANSFERENCIA DE TELEFONE CELULAR |
| 01101 | JUROS E SEGURO DE EMPRESTIMO |
| 01102 | ENCARGOS FINANCEIROS DE FINANCIAMENTO |
| 01201 | PAGAMENTO DE EMPRESTIMO |
| 01202 | EMPRESTIMO (PAGAMENTO) |
| 01301 | SEGURO-EDUCACAO |
| 01302 | CREDITO EDUCATIVO (PAGAMENTO) |
| 01303 | PAGAMENTO DE CREDITO EDUCATIVO |
| 01401 | PAGAMENTO DE ASILO |
| 01402 | ASILO (PAGAMENTO) |
| 01501 | CARNET DE MERCADORIAS |
| 01701 | SPC (TAXAS) |
| 01702 | SERVICO DE PROTECAO AO CREDITO (TAXAS) |
| 01801 | PREVIDENCIA PUBLICA (FEDERAL, ESTADUAL, MUNICIPAL E MILITAR) |
| 01802 | FUNRURAL |
| 01803 | SEGURO COLETIVO DE TRABALHO (PREVIDENCIA PUBLICA) |
| 01804 | FUNDO ESPECIAL DA POLICIA MILITAR (PREVIDENCIA MILITAR) |
| 01805 | PREVIDENCIA ESTADUAL |
| 01806 | PREVIDENCIA MUNICIPAL |
| 01807 | PREVIDENCIA MILITAR |
| 01808 | PREVIDENCIA FEDERAL |
| 01809 | FUNDO DE PENSAO MILITAR |
| 01810 | PREVIDENCIA PUBLICA FECHADA |
| 01901 | INDENIZACAO A TERCEIROS |
| 01902 | AVISO PREVIO A EMPREGADOR |
| 01903 | PARTICIPACAO EM PREJUIZO DE EMPRESA |
| 02001 | PECULIO DOMICILIAR |
| 02002 | PECULIO |
| 02101 | ESMOLA |
| 02102 | ESMOLET |
| 02201 | PAX DOMINNI (AUXILIO FUNERAL) |
| 02202 | AUXILIO FUNERAL (PAX DOMINNI) |
| 02203 | ASSOCIACAO DE AJUDA FUNERARIA |
| 02204 | ASSOCIACAO POSTUMA |
| 02205 | ASSOCIACAO FUNERARIA |
| 02206 | CONSORCIO FUNERARIO |
| 02207 | TAXA DE CEMITERIO (MANUNTENCAO) |
| 02208 | PLANO DE FUNERAL |
| 02301 | MACONARIA |
| 02302 | ROTARY CLUBE |
| 02401 | CAIXINHA ENTRE ASSOCIADOS (FUNCIONARIOS, AMIGOS, ETC.) |
| 02402 | CONTRIBUICAO DE GRUPO (FUNCIONARIOS, AMIGOS, CASAIS, ETC.) |
| 02403 | CAIXA BENEFICENTE |
| 02404 | CONSORCIO DE DINHEIRO |
| 02405 | GREMIO (FUNCIONARIOS DE EMPRESA) |
| 02406 | CONTRIBUICAO PARA GREMIO (FUNCIONARIOS DE EMPRESA) |
| 02501 | PENSAO (PENSIONISTA) |
| 02601 | CONTRIBUICAO PARA OUTRAS ASSOCIACOES |
| 02602 | ASSOCIACOES OUTRAS (CONTRIBUICAO) |
| 02603 | COOPERATIVA (MENSALIDADE) |
| 02701 | PAGAMENTO DE TITULO PROTESTADO |
| 02702 | PROTESTO DE TITULO |
| 02703 | TITULO PROTESTADO |
| 02801 | ALISTAMENTO MILITAR (MULTA) |
| 02802 | MULTA DE ALISTAMENTO MILITAR |
| 02901 | SEGURO DE BENS MOVEIS (EXCETO DE VEICULOS) |
| 03001 | TAXA DE MANUTENCAO DE ARMAS |
| 03101 | DINHEIRO ROUBADO |
| 03201 | JUSTICA DO TRABALHO |
| 03301 | ALIMENTACAO SUBSIDIADA |
| 03302 | DESCONTO DE CESTA BASICA |
| 03303 | DESCONTO DE ALIMENTACAO (ALIMENTACAO SUBSIDIADA) |
| 03304 | DESCONTO ALIMENTACAO |
| 03401 | TRANSPORTE SUBSIDIADO |
| 03402 | DESCONTO TRANSPORTE |
| 03403 | DESCONTO DE TRANSPORTE (TRANSPORTE SUBSIDIADO) |
| 03501 | IMPOSTO SOBRE SERVICOS (ISS) (EVENTUAL) |
| 03502 | ISS (IMPOSTO SOBRE SERVICOS) (EVENTUAL) |
| 03601 | AUXILIO-EDUCACAO (DESCONTO) |
| 03602 | DESCONTO DE AUXILIO-EDUCACAO |
| 03701 | TAXA DE CADASTRAMENTO DO CPF |
| 03702 | TAXA CARTAO DE PRODUTOR RURAL |
| 03703 | TAXA PARA DOCUMENTOS PESSOAIS |
| 03704 | TAXA DE RENOVACAO DO CPF |
| 03801 | COMPLEMENTACAO DO IMPOSTO DE RENDA |
| 03802 | IMPOSTO DE RENDA DO EXERCICIO ANTERIOR |
| 03901 | IMPOSTO SINDICAL (ANUAL) |
| 03902 | CONTRIBUICAO SINDICAL (ANUAL) |
| 03903 | CONTRIBUICAO PARA SINDICATO E ASSOCIACAO DE TRABALHADORES (MENSAL) |
| 04001 | GORJETA |
| 04002 | CAIXINHA (GORJETA) |
| 04101 | TAXA DE INSCRICAO PARA CONCURSO (EMPREGO) |
| 04102 | TAXA DE INSCRICAO PARA CONGRESSO (NAO-EDUCACAO) |
| 04201 | TITULO DE CAPITALIZACAO (AQUISICAO) |
| 04202 | AQUISICAO DE TITULO DE CAPITALIZACAO |
| 04301 | CONTRIBUICAO PARA PARTIDO POLITICO |
| 04401 | DIZIMO PARA IGREJA |
| 04501 | EMPRESTIMO A TERCEIROS |
| 99901 | AGREGADO |
| 00101 | CURSO PRE-ESCOLAR |
| 00201 | CURSO SUPLETIVO |
| 00301 | AULA PARTICULAR |
| 00401 | CURSO EM DISCO OU FITA |
| 00402 | CURSO EM FITA CASSETE |
| 00403 | CURSO EM DISCO |
| 00404 | LINGUAFONE |
| 00501 | TAXAS ESCOLARES |
| 00502 | TAXA DE MATERIAL ESCOLAR |
| 00503 | TAXA DE EXAME BIOMEDICO |
| 00504 | TAXA DE TRANSFERENCIA ESCOLAR |
| 00505 | CERTIFICADO DE CONCLUSAO ESCOLAR |
| 00506 | DOCUMENTACAO ESCOLAR (EMOLUMENTO) |
| 00507 | EMOLUMENTO (DESPESA DE DOCUMENTACAO ESCOLAR) |
| 00508 | FICHA MODELO 18 E 19 |
| 00509 | DIPLOMA |
| 00510 | CARTEIRA DE ESTUDANTE |
| 00511 | CADERNETA ESCOLAR |
| 00512 | DECLARACAO ESCOLAR |
| 00513 | CARTAO MAGNETICO ESCOLAR |
| 00514 | CARTAO ESCOLAR MAGNETICO |
| 00515 | COTA EXTRA ESCOLAR |
| 00601 | ASSINATURA DE PERIODICO TECNICO |
| 00701 | LIVRO ESCOLAR DE PRIMEIRO E SEGUNDO GRAU |
| 00702 | LIVRO ESCOLAR DE 1º E 2º GRAU |
| 00801 | LIVRO TECNICO, REVISTA TECNICA E OUTROS LIVROS DIDATICOS |
| 00802 | LIVRO TECNICO |
| 00803 | REVISTA TECNICA |
| 00804 | OUTROS LIVROS DIDATICOS |
| 00805 | DICIONARIO |
| 00806 | APOSTILA |
| 00807 | MANUAL DIDADITO |
| 00808 | GUIA DE VESTIBULAR |
| 00809 | APOSTILA DE SUPLETIVO |
| 00810 | LIVRO PARADIDATICO |
| 00811 | ENCICLOPEDIA |
| 00812 | LIVRO E REVISTA TECNICA |
| 00813 | LIVRO PRE-ESCOLAR |
| 00814 | MANUAL DE VESTIBULAR |
| 00901 | TRANSPORTE ESCOLAR |
| 01001 | UNIFORME ESCOLAR |
| 01101 | CRECHE |
| 01102 | BERCARIO |
| 01201 | LOCACAO DE ALOJAMENTO ESTUDANTIL |
| 01202 | LOCACAO DE QUARTO |
| 01203 | LOCACAO DE VAGA |
| 01301 | FESTA ESCOLAR |
| 01401 | CARNE DE FORMATURA |
| 01402 | FORMATURA |
| 01403 | COLACAO DE GRAU |
| 01404 | CONVITE DE FORMATURA |
| 01405 | OUTROS ARTIGOS DE FORMATURA (BECA, CHAPEU, ETC) |
| 01501 | CURSO DE POS-GRADUACAO (ESPECIALIZACAO) |
| 01502 | ESPECIALIZACAO |
| 01503 | CURSO DE ESPECIALIZACAO |
| 01601 | EXCURSAO ESCOLAR |
| 01701 | TAXA DE INSCRICAO DE CONGRESSO, SEMINARIO, ETC. (EDUCACAO) |
| 01702 | TAXA DE INSCRICAO DE CONGRESSO (EDUCACAO) |
| 01703 | TAXA DE INSCRICAO DE SEMINARIO (EDUCACAO) |
| 01704 | TAXA DE INSCRICAO DE PALESTRA EDUCATIVA |
| 01801 | ASSOCIACAO DE PAIS E MESTRES |
| 01802 | CIRCULO DE PAIS E MESTRES |
| 01901 | PASTA ESCOLAR |
| 02001 | MERENDEIRA ESCOLAR |
| 02002 | LANCHEIRA ESCOLAR |
| 02003 | AGENDA ESCOLAR |
| 02101 | MOCHILA ESCOLAR |
| 02201 | CURSO DE POS-GRADUACAO (MESTRADO) |
| 02202 | MESTRADO |
| 02203 | CURSO DE MESTRADO |
| 02401 | COLONIA DE FERIAS (EDUCACAO) |
| 02501 | INSTRUMENTOS E APARELHOS DE CURSOS EDUCATIVOS |
| 02502 | APARELHOS DE CURSOS EDUCATIVOS |
| 02503 | ESTETOSCOPIO |
| 02504 | TENSIOMETRO |
| 02505 | AEROGRAFO |
| 02506 | BUSSOLA |
| 02507 | INSTRUMENTOS CIRURGICOS DE CURSO |
| 02508 | INSTRUMENTOS DE AULA PRATICA |
| 02509 | INSTRUMENTOS DE CURSOS EDUCATIVOS |
| 02601 | ALIMENTACAO ESCOLAR |
| 02602 | ALIMENTACAO NA ESCOLA |
| 02701 | BIBLIOTECA (MENSALIDADE, MULTA, ETC.) |
| 02702 | MENSALIDADE DE BIBLIOTECA |
| 02703 | MULTA DE BIBLIOTECA |
| 02801 | GREMIO ESTUDANTIL |
| 02901 | MAPA ESCOLAR |
| 03001 | TRADUTOR |
| 03101 | CURSO REGULAR DE PRIMEIRO GRAU |
| 03102 | CURSO REGULAR DE 1º GRAU |
| 03201 | CURSO REGULAR DE SEGUNDO GRAU |
| 03202 | CURSO REGULAR DE 2º GRAU |
| 03301 | CURSO REGULAR DE TERCEIRO GRAU |
| 03302 | CURSO REGULAR DE 3º GRAU |
| 03401 | AULA DE GINASTICA |
| 03501 | CURSO DE DATILOGRAFIA |
| 03601 | CURSO E AULA DE BALE |
| 03602 | CURSO DE BALE |
| 03603 | AULA DE BALE |
| 03604 | AULA DE DANCA |
| 03701 | CURSO E AULA DE IOGA |
| 03702 | CURSO DE IOGA |
| 03703 | AULA DE IOGA |
| 03801 | CURSO DE MUSICA |
| 03802 | AULA DE MUSICA |
| 03901 | CURSO DE INFORMATICA |
| 04001 | AULA DE HIDROGINASTICA |
| 04101 | CURSO DE POS-GRADUACAO (DOUTORADO) |
| 04102 | DOUTORADO |
| 04103 | CURSO DE DOUTORADO |
| 04201 | TAXA DE INSCRICAO DE VESTIBULAR |
| 04202 | INSCRICAO DE VESTIBULAR |
| 04301 | AULA DE NATACAO |
| 04401 | CURSO DE IDIOMA (EXCETO LINGUAFONE) |
| 04402 | CURSO DE ESPANHOL |
| 04403 | CURSO DE INGLES |
| 04404 | CURSO DE ALEMAO |
| 04405 | CURSO DE ITALIANO |
| 04406 | CURSO DE JAPONES |
| 04407 | CURSO DE FRANCES |
| 04501 | ALUGUEL DE LIVRO DE PRIMEIRO GRAU |
| 04601 | TAXA DE INSCRICAO PARA CONCURSO |
| 04701 | CURSO DE MECANICA EM REFRIGERACAO |
| 04801 | SOFTWARE DE CURSO |
| 04802 | SOFT DE CURSO |
| 04803 | PROGRAMA COMPUTACIONAL DE CURSO |
| 04901 | CURSO DE PRIMEIROS SOCORROS |
| 05001 | CURSO PARA EXCEPCIONAL |
| 05101 | CURSO PRE-VESTIBULAR |
| 05201 | CURSO PRE-TECNICO |
| 05301 | MATRICULA ESCOLAR |
| 05401 | AUTO ESCOLA |
| 05402 | CURSO DE DIRECAO DE CARRO |
| 05403 | CURSO DE DIRECAO DE MOTO |
| 05404 | CURSO DE DIRECAO DE ONIBUS |
| 05405 | CURSO DE DIRECAO DE CAMINHAO |
| 05501 | CURSO DE CABELEIREIRO |
| 05601 | CURSO DE CORTE E COSTURA |
| 05602 | CURSO DE BORDADO |
| 05701 | EDUCACAO A DISTANCIA (CURSOS) |
| 05801 | AULA DE CAPOEIRA |
| 05901 | AULA DE FUTEBOL |
| 06001 | AULA DE VOLEIBOL |
| 06101 | AULA DE BASQUETEBOL |
| 06201 | AULA DE TENIS |
| 06301 | AULA DE HANDEBOL |
| 06401 | AULA DE LUTAS |
| 06402 | AULA DE BOXE |
| 06403 | AULA DE JUDO |
| 06404 | AULA DE KARATE |
| 06501 | CURSO DE CULINARIA |
| 06601 | CURSO DE DANCA |
| 06701 | CURSO DE DESENHO |
| 06801 | CURSO DE ELETRICISTA |
| 06901 | CURSO DE ENFERMAGEM |
| 07001 | CURSO DE PINTURA |
| 07101 | CURSO DE SECRETARIADO |
| 07201 | CURSO DE TEATRO |
| 07301 | CURSO DE VIGILANTE |
| 07401 | CURSO DE DIRECAO DE OUTROS VEICULOS |
| 07402 | CURSO DE DIRECAO DE TREM |
| 07403 | CURSO DE DIRECAO DE METRO |
| 07404 | CURSO DE DIRECAO DE NAVIO |
| 07405 | CURSO DE DIRECAO DE AVIAO |
| 07501 | CURSO TECNICO |
| 07601 | CURSO PREPARATORIO PARA CONCURSO |
| 07701 | CURSOS RELIGIOSOS |
| 07801 | CURSO DE ARTESANATO |
| 07901 | CURSO DE MECANICA DE VEICULOS |
| 08001 | CURSO DE TELEMARKETING |
| 08002 | CURSO DE ATENDIMENTO AO CLIENTE |
| 08101 | CURSO DE MANICURE E PEDICURE |
| 08201 | CURSO DE MONTAGEM E MANUTENCAO DE COMPUTADORES |
| 08301 | CURSO DE ARTES |
| 08302 | AULA DE PINTURA |
| 08401 | CURSO DE ESTETICISTA |
| 08402 | CURSO DE DEPILACAO |
| 08501 | MATERIAL ESCOLAR (AGREGADO) |
| 08502 | KIT ESCOLAR (AGREGADO) |
| 08601 | CURSO DE MASSOTERAPIA (MASSAGEM) |
| 08701 | CURSO DE PROMOTOR DE VENDAS |
| 08801 | CURSO PREPARATORIO PARA OAB |
| 08901 | CURSO PREPARATORIO PARA ESCOLA MILITAR |
| 09001 | CURSOS DIVERSOS (MEDITACAO, ORATORIA, ETC.) |
| 09101 | OUTROS CURSOS DE CAPACITACAO PROFISSIONAL |
| 09102 | OUTROS CURSOS DE ATUALIZACAO, APERFEICOAMENTO OU EXTENSAO |
| 09201 | CURSO NAO-ESPECIFICADO |
| 09301 | REVISAO DE TESES, DISSERTACOES E TRABALHOS ESCOLARES |
| 99901 | AGREGADO |
| 00101 | EMPLACAMENTO DE AUTOMOVEL |
| 00102 | LICENCA DE AUTOMOVEL |
| 00201 | MULTAS |
| 00301 | SEGURO OBRIGATORIO |
| 00401 | IMPOSTO SOBRE PROPRIEDADE DE VEICULOS AUTOMOTORES (IPVA) |
| 00402 | IPVA (IMPOSTO SOBRE PROPRIEDADE DE VEICULOS AUTOMOTORES) |
| 00403 | IMPOSTO SOBRE PROPRIEDADE (IPVA) |
| 00501 | ASSISTENCIA AUTOMOBILISTICA (MENSALIDADE E TAXA) |
| 00502 | ASSOCIACAO DE ASSISTENCIA AUTOMOBILISTICA (MENSALIDADE E TAXA) |
| 00503 | ASSISTENCIA AUTOMOBILISTICA (MENSALIDADE) |
| 00504 | ASSISTENCIA AUTOMOBILISTICA (TAXAS) |
| 00601 | SEGURO VOLUNTARIO DE AUTOMOVEIS/UTILITARIOS |
| 00602 | SEGURO TOTAL DE AUTOMOVEIS/UTILITARIOS |
| 00603 | SEGURO PARCIAL DE AUTOMOVEIS/UTILITARIOS |
| 00701 | VAGA CATIVA DE ESTACIONAMENTO DE VEICULO |
| 00702 | VAGA DE ESTACIONAMENTO DE VEICULO |
| 00801 | ALUGUEL DE VEICULO |
| 00802 | ALUGUEL |
| 00901 | TAXA DE EXPEDIENTE (DETRAN) |
| 00902 | TAXA DE CARTEIRA DE HABILITACAO |
| 00903 | PERICIA DE VEICULO (TAXA) |
| 00904 | TAXA DE TRANSFERENCIA DE VEICULO |
| 00905 | TRANSFERENCIA DE VEICULO (TAXA) |
| 00906 | TAXA DE TRANSFERENCIA DE PLACA DE VEICULO |
| 00907 | TRANSFERENCIA DE PLACA DE VEICULO (TAXA) |
| 00908 | FOTOGRAFIA DE ACIDENTE (PERICIA) |
| 00909 | DOCUMENTO UNICO DE TRANSITO |
| 00910 | DUT (DOCUMENTO UNICO DE TRANSITO) |
| 00911 | TAXA DE NADA CONSTA |
| 00912 | NADA CONSTA (TAXA) |
| 00913 | BAIXA DE RESERVA DE REGISTRO DE VEICULO |
| 00914 | TAXA DE BAIXA DE RESERVA DE REGISTRO DE VEICULO |
| 00915 | EXAME PARA RENOVACAO DE CARTEIRA DE MOTORISTA |
| 00916 | EXAME PSICOTECNICO DE CARTEIRA DE MOTORISTA |
| 00917 | TAXA DE DESALIENACAO DE VEICULO |
| 00918 | TAXA DE LEGISLACAO (DETRAN) |
| 00919 | TAXA DE EXPEDIENTE |
| 00920 | TAXA DE LACRE DA PLACA |
| 00921 | TAXA DE VISTORIA |
| 01001 | TRANSFERENCIA DE CONSORCIO DE VEICULO |
| 01002 | TRANSFERENCIA DE CONSORCIO |
| 01101 | PROPINA |
| 01201 | LEASING DE AUTOMOVEL |
| 01301 | SEGURO VOLUNTARIO DE MOTO |
| 01302 | SEGURO VOLUNTARIO DE MOTOCICLETA |
| 01303 | SEGURO TOTAL DE MOTOCICLETA |
| 01401 | EMPLACAMENTO DE MOTO |
| 01402 | LICENCA DE MOTO |
| 01501 | EMPLACAMENTO DE EMBARCACAO |
| 01502 | LICENCA DE EMBARCACAO |
| 01601 | EMPLACAMENTO DE CAMINHAO |
| 01602 | LICENCA DE CAMINHAO |
| 01701 | SERVICO DE SEGURANCA VIA SATELITE |
| 01702 | SEGURANCA VEICULAR VIA SATELITE |
| 01801 | TAXA DE CONSERVACAO E MANUTENCAO DE VIAS PUBLICAS |
| 01901 | TAXAS INCLUIDAS NO LICENCIAMENTO ANUAL VEICULOS |
| 01902 | TAXA DE BOMBEIRO (DETRAN) |
| 01903 | TAXA DE CORREIO (DETRAN) |
| 99901 | AGREGADO |
| 00101 | AUTOMOVEL DE PASSEIO NACIONAL |
| 00201 | BARCO |
| 00202 | BARCO A MOTOR |
| 00301 | BICICLETA |
| 00401 | MOTOCICLETA |
| 00501 | WINDSURF |
| 00601 | ASA DELTA |
| 00701 | TRAILLER |
| 00801 | ULTRALEVE |
| 00901 | CAIQUE |
| 01001 | AUTOMOVEL UTILITARIO NACIONAL |
| 01002 | KOMBI |
| 01101 | CAMINHAO |
| 01201 | LANCHA |
| 01301 | JET SKI |
| 01401 | MOBILETE |
| 01501 | AUTOMOVEL UTILITARIO IMPORTADO |
| 01601 | AUTOMOVEL DE PASSEIO IMPORTADO |
| 01701 | VEICULO DE TRACAO ANIMAL E ANIMAL DE TRACAO |
| 01702 | ANIMAL DE TRACAO |
| 01801 | WALK MACHINE |
| 01901 | KART |
| 01902 | JIPE (KART) |
| 01903 | MINI-BUG |
| 02001 | BARCO A REMO |
| 02002 | CANOA A REMO |
| 02003 | PIROGA |
| 02101 | CONSORCIO DE AUTOMOVEL |
| 02201 | CONSORCIO DE MOTOCICLETA |
| 02301 | BARCO A VELA |
| 99901 | AGREGADO |
| 00101 | APOSENTADORIA DO INSTITUTO NACIONAL DE SEGURIDADE SOCIAL (INSS) |
| 00102 | APOSENTADORIA (INSS) |
| 00201 | PENSAO DO INSTITUTO NACIONAL DE SEGURIDADE SOCIAL (INSS) |
| 00202 | PENSAO (INSS) |
| 00301 | APOSENTADORIA ( MUNICIPAL, ESTADUAL, FEDERAL) DA PREVIDENCIA PUBLICA |
| 00302 | APOSENTADORIA ( MUNICIPAL, ESTADUAL, FEDERAL) |
| 00401 | PENSAO ( MUNICIPAL, ESTADUAL, FEDERAL) DA PREVIDENCIA PUBLICA |
| 00402 | PENSAO ( MUNICIPAL, ESTADUAL, FEDERAL) |
| 00501 | APOSENTADORIA, SUPLEM., COMPLEM. DA PREVIDENCIA PRIVADA (ABERTA OU FECHADA) |
| 00502 | APOSENTADORIA, COMPLEMENTACAO DE PREVIDENCIA PRIVADA (ABERTA OU FECHADA) |
| 00601 | BOLSA DE ESTUDO |
| 00701 | PENSAO ALIMENTICIA DE NAO MORADOR |
| 00702 | PENSAO ALIMENTICIA DE NAO-MORADOR |
| 00801 | ALUGUEL, USO OU EXPLORACAO DE BENS IMOVEIS |
| 00802 | ARRENDAMENTO DE BENS IMOVEIS |
| 00901 | ALUGUEL, DIREITOS AUTORAIS, PATENTES DE BENS MOVEIS |
| 00902 | ALUGUEL, USO OU EXPLORACAO DE BENS MOVEIS |
| 00903 | ALUGUEL/USO/EXPLORACAO DE BENS MOVEIS |
| 00904 | DIREITOS AUTORAIS |
| 00905 | EXPLORACAO DE PATENTES |
| 01001 | BOLSA-FAMILIA |
| 01101 | BENEFICIO DE PRESTACAO CONTINUADA (BPC, LOAS) |
| 01201 | PROGRAMA DE ERRADICACAO DO TRABALHO INFANTIL (PETI) |
| 01301 | BOLSA-ESCOLA |
| 01401 | PROGRAMAS DE RENDA MINIMA (OUTRAS; MUN.EST.FEDERAL) |
| 01501 | AUXILIO/TIQUETE/CARTAO ALIMENTACAO |
| 01502 | TIQUETE-ALIMENTACAO |
| 01503 | TIQUETE-REFEICAO |
| 01504 | CARTAO-REFEICAO |
| 01601 | AUXILIO/VALE/CARTAO TRANSPORTE E COMBUSTIVEL |
| 01602 | VALE-TRANSPORTE |
| 01603 | AUXILIO-COMBUSTIVEL |
| 01604 | CARTAO-TRANSPORTE |
| 01701 | AUXILIO-ATIVIDADE |
| 01702 | ADICIONAL DE INSALUBRIDADE |
| 01703 | ADICIONAL DE PERICULOSIDADE |
| 01704 | ADICIONAL NOTURNO |
| 01801 | AUXILIO-FARDAMENTO |
| 01901 | AUXILIO-ESTIAGEM |
| 02001 | AUXILIO-COMUNICACAO |
| 02101 | AUXILIO ENERGIA ELETRICA |
| 02201 | AGENTE JOVEM - PROGRAMA GOVERNAMENTAL PARA JOVEM DESEMPREGADO |
| 02301 | AUXILIO-DOENCA DA PREVIDENCIA PRIVADA |
| 02401 | AUXILIO A PORTADORES DE DEFICIENCIA FISICA |
| 02501 | AUXILIO-DOENCA DA PREVIDENCIA PUBLICA |
| 02601 | MESADA DE NAO-MORADOR |
| 02602 | MESADA DE NAO MORADOR |
| 02701 | CESTA BASICA |
| 02801 | CARTAO CIDADAO |
| 02901 | AUXILIO-LEITE |
| 03001 | BOLSA-RENDA |
| 03101 | AUXILIO-GAS |
| 03201 | DOACAO DE NAO-MORADOR |
| 03202 | DOACAO DE NAO MORADOR |
| 03301 | RENDIMENTO DE MORADOR AUSENTE |
| 03401 | RENDIMENTO DE MENORES DE DEZ ANOS |
| 03501 | AUXILIO DEFESO |
| 03601 | AUXILIO-CRECHE |
| 03701 | AUXILIO PARA PLANO MEDICO |
| 03801 | AUXILIO MORADIA |
| 00101 | RENDIMENTO E ABONO DO PIS/PASEP |
| 00102 | RENDIMENTO DO PIS/PASEP |
| 00103 | ABONO DO PIS/PASEP |
| 00104 | PIS/PASEP (RENDIMENTOS) |
| 00105 | PIS/PASEP (ABONO) |
| 00201 | SAQUE DO PIS/PASEP (TOTAL) |
| 00202 | SAQUE TOTAL DO PIS/PASEP |
| 00301 | PARTICIPACAO NOS LUCROS DE NEGOCIOS (EMPREGADOR) |
| 00401 | RESTITUICAO DO IMPOSTO DE RENDA |
| 00402 | DEVOLUCAO DO IMPOSTO DE RENDA |
| 00501 | HERANCA E OUTRAS TRANSFERENCIAS PATRIMONIAIS |
| 00502 | HERANCA/TRANSFERENCIA PATRIMONIAL |
| 00601 | VENDA DE TERRENO |
| 00701 | VENDA DE AUTOMOVEL |
| 00801 | VENDA DE IMOVEL (EXCETO TERRENO) |
| 00901 | VENDA DE MOTOCICLETA |
| 01001 | GANHO EM JOGOS (LOTERIA, MEGA, ETC.) OU PREMIO |
| 01002 | PREMIO OU GANHO EM JOGOS (LOTERIA, MEGA, ETC.) |
| 01003 | GANHO EM JOGOS (LOTERIA, MEGA, ETC.) |
| 01101 | GANHO COM VIAGEM A TRABALHO |
| 01102 | AJUDA DE CUSTO EM TRABALHO |
| 01201 | RESTITUICAO DE DINHEIRO NA COMPRA POR CARNET |
| 01301 | JUROS DE EMPRESTIMO |
| 01401 | EMPRESTIMO |
| 01402 | RECEBIMENTO DE EMPRESTIMO |
| 01403 | PENHORA (EMPRESTIMO) |
| 01501 | DIVIDENDOS DE ACOES (DINHEIRO) |
| 01601 | VENDA DE BARCO |
| 01701 | SEGURO-DESEMPREGO |
| 01702 | SALARIO-DESEMPREGO |
| 01703 | AUXILIO-DESEMPREGO |
| 01801 | AUXILIO-MATERNIDADE |
| 01802 | AUXILIO-NATALIDADE |
| 01901 | CREDITO-EDUCATIVO |
| 02001 | ACAO JUDICIAL TRABALHISTA |
| 02101 | INDENIZACAO JUDICIAL |
| 02201 | ACIDENTE DE TRABALHO (PREVIDENCIA PUBLICA) |
| 02301 | RESTITUICAO DA PREVIDENCIA PUBLICA |
| 02302 | DEVOLUCAO DA PREVIDENCIA PUBLICA |
| 02401 | PENSIONISTA (RENDIMENTO) |
| 02501 | RECEBIMENTO DE PECULIO |
| 02502 | PECULIO (RECEBIMENTO) |
| 02601 | AUXILIO-EDUCACAO |
| 02602 | AUXILIO-CRECHE |
| 02603 | SALARIO-EDUCACAO |
| 02604 | AUXILIO-ESCOLA |
| 02605 | AUXILIO-MAE GUARDIA |
| 02701 | RESTITUICAO JUDICIAL |
| 02702 | DEVOLUCAO JUDICIAL |
| 02801 | CAIXINHA |
| 02901 | AUXILIO-FUNERAL |
| 03001 | RESTITUICAO DE PENSAO ALIMENTICIA |
| 03002 | DEVOLUCAO DE PENSAO ALIMENTICIA |
| 03101 | AUXILIO-VELHICE |
| 03201 | DINHEIRO ACHADO |
| 03301 | RESTITUICAO DA PREVIDENCIA PRIVADA |
| 03302 | DEVOLUCAO DA PREVIDENCIA PRIVADA |
| 03401 | SEGURO-HABITACAO (RESTITUICAO) |
| 03402 | TAXA DE CONSERVACAO DE IMOVEL (RESTITUICAO) |
| 03403 | RESTITUICAO DE SEGURO-HABITACAO |
| 03404 | RESTITUICAO DE TAXA DE CONSERVACAO DE IMOVEL |
| 03405 | DEVOLUCAO DE SEGURO-HABITACAO |
| 03406 | DEVOLUCAO DE TAXA DE CONSERVACAO DE IMOVEL |
| 03407 | DEVOLUCAO DE TAXA DE ESGOTO (CIA DE SANEAMENTO ESTADUAL) |
| 03501 | AUXILIO-TRATAMENTO |
| 03601 | VENDA DE CONSORCIO, CARNET E OUTROS |
| 03602 | VENDA DE RELOGIO |
| 03603 | VENDA DE ROUPA |
| 03604 | VENDA DE CAMISA |
| 03605 | VENDA DE CALCA |
| 03606 | VENDA DE VIDEOGAME |
| 03607 | VENDA DE APARELHO SONORO |
| 03608 | VENDA DE TELEVISAO |
| 03609 | VENDA DE COLCHA |
| 03610 | VENDA DE TECIDO |
| 03611 | VENDA DE MAQUINA DE COSTURA |
| 03612 | VENDA DE MOVEL |
| 03613 | VENDA DE VIDEOCASSETE |
| 03614 | VENDA DE APARELHO ELETRODOMESTICO |
| 03615 | VENDA DE CHURRASQUEIRA |
| 03616 | VENDA DE GAIOLA |
| 03617 | VENDA DE JOIA |
| 03618 | VENDA DE MICROCOMPUTADOR |
| 03619 | VENDA DE INSTRUMENTO MUSICAL |
| 03620 | VENDA DE PECAS DE VEICULO |
| 03621 | VENDA DE FRUTAS |
| 03622 | VENDA DE APARELHO DE PRESSAO (TENSIOMETRO) |
| 03624 | VENDA DE REDE |
| 03625 | VENDA DE FURADEIRA |
| 03626 | VENDA DE BUJAO DE GAS |
| 03627 | VENDA DE BOTIJAO DE GAS |
| 03628 | VENDA DE PANELA |
| 03629 | VENDA DE CONSORCIO, CARNE E OUTROS |
| 03630 | VENDA DE ANTENA PARABOLICA |
| 03631 | VENDA DE APARELHO CELULAR |
| 03633 | VENDA DE MAQUINA AGRICOLA |
| 03634 | VENDA DE PRODUTOS AGRICOLAS |
| 03635 | VENDA DE MIUDEZAS EM GERAL |
| 03701 | DECIMO TERCEIRO SALARIO |
| 03702 | 13° SALARIO |
| 03801 | FERIAS |
| 03802 | ADIANTAMENTO DE FERIAS |
| 03803 | ABONO DE FERIAS |
| 03804 | AGREGADO FERIAS E DECIMO TERCEIRO |
| 03805 | ABONO PECUNIARIO DE FERIAS |
| 03901 | DIFERENCA SALARIAL |
| 03902 | PREMIO POR DESEMPENHO DE ATIVIDADE |
| 03903 | BONIFICACAO RECEBIDA DA EMPRESA |
| 04001 | AVISO PREVIO |
| 04002 | RESCISAO CONTRATUAL |
| 04101 | HORA EXTRA |
| 04201 | REVISAO CONTRATUAL |
| 04301 | AJUDA DE CUSTO DE TRANSFERENCIA DE LOCAL DE TRABALHO |
| 04401 | TITULO DE CAPITALIZACAO (RESGATE) |
| 04402 | RESGATE DE TITULO DE CAPITALIZACAO |
| 04501 | PARTICIPACAO NOS LUCROS DE EMPRESA (EMPREGADO) |
| 04601 | VENDA DE MATERIAL DE CONSTRUCAO |
| 04602 | VENDA DE TIJOLO |
| 04701 | VENDA DE VEICULO DE TRACAO ANIMAL OU ANIMAL DE TRACAO |
| 04702 | VENDA DE ANIMAL DE TRACAO |
| 04703 | VENDA DE CARROCA |
| 04704 | VENDA DE ANIMAL DOMESTICO |
| 04705 | VENDA DE ANIMAL |
| 04801 | VENDA DE BICICLETA |
| 04901 | VENDA DE LINHA TELEFONICA |
| 05001 | COMISSAO DE VENDA (ESPORADICA) |
| 05002 | TRABALHOS EVENTUAIS |
| 05101 | RESTITUICAO DE CONVENIO DE SAUDE |
| 05102 | DEVOLUCAO DE CONVENIO DE SAUDE |
| 05103 | RESTITUICAO DE PLANO DE SAUDE |
| 05104 | DEVOLUCAO DE PLANO DE SAUDE |
| 05201 | RESTITUICAO DE MULTA DE TRANSITO |
| 05202 | DEVOLUCAO DE MULTA DE TRANSITO |
| 05401 | PRESENTE EM DINHEIRO (ESPORADICO) |
| 05501 | INDENIZACAO E RESTITUICAO DE SEGURO PESSOAL |
| 05502 | PREMIO RESTITUIDO E INDENIZACAO PAGOS POR SEGURO PESSOAL |
| 05601 | INDENIZACAO E RESTITUICAO DE SEGURO OBRIGATORIO DE AUTO/UTILITARIOS PESSOAL |
| 05602 | PREMIO RESTITUIDO E INDENIZACAO PAGOS POR SEGURO OBRIGATORIO DE AUTO/UTILITARIOS |
| 05701 | INDENIZACAO E RESTITUICAO DE SEGURO VOLUNTARIO DE AUTO/UTILITARIOS PESSOAL |
| 05702 | PREMIO RESTITUIDO E INDENIZACAO PAGOS POR SEGURO VOLUNTARIO DE AUTO/UTILITARIOS |
| 05801 | INDENIZACAO E RESTITUICAO DE SEGURO OBRIGATORIO DE MOTO |
| 05802 | PREMIO RESTITUIDO E INDENIZACAO PAGOS POR SEGURO OBRIGATORIO DE MOTO |
| 05901 | INDENIZACAO E RESTITUICAO DE SEGURO VOLUNTARIO DE MOTO |
| 05902 | PREMIO RESTITUIDO E INDENIZACAO PAGOS POR SEGURO VOLUNTARIO DE MOTO |
| 06201 | SAQUE DO FGTS |
| 06301 | INDENIZACAO TRABALISTA |
| 00101 | POUPANCA (DEPOSITO) |
| 00102 | POUPANCA (DEPOSITO/APLICACAO) |
| 00201 | FUNDO DE APLICACAO FINANCEIRA (CDB, RDB, COMMODITIES, ETC.) (DEPOSITO/APLICACAO) |
| 00202 | FUNDO DE APLICACAO FINANCEIRA (CDB, RDB, COMMODITIES, ETC.) |
| 00203 | DEPOSITO DE APLICACAO FINANCEIRA (CDB, RDB, COMMODITIES, ETC.) |
| 00204 | FUNDO DE APLICACAO (DEPOSITO/APLICACAO) |
| 00301 | ACOES (APLICACAO) |
| 00302 | ACOES (DEPOSITO/APLICACAO) |
| 00401 | OUTROS (OURO, MOEDAS ESTRANGEIRAS, ETC.) (APLICACAO) |
| 00402 | OUTROS (DEPOSITO/APLICACAO) |
| 00101 | POUPANCA (RETIRADA) |
| 00102 | POUPANCA (RETIRADA/RESGATE) |
| 00201 | FUNDO DE APLICACAO FINANCEIRA (CDB, RDB, COMMODITIES, ETC.) (RETIRADA/RESGATE) |
| 00202 | FUNDO DE APLICACAO (RETIRADA/RESGATE) |
| 00301 | ACOES (RESGATE) |
| 00302 | ACOES (VENDA) |
| 00303 | RESGATE DE ACOES |
| 00304 | VENDA DE ACOES |
| 00305 | ACOES (RETIRADA/RESGATE) |
| 00401 | OUTROS (OURO, MOEDAS ESTRANGEIRAS, ETC.) (RESGATE) |
| 00402 | OUTROS (RETIRADA/RESGATE) |
| 00101 | ARROZ POLIDO |
| 00102 | ARROZ HIBRIDO |
| 00103 | ARROZ BICA CORRIDA |
| 00104 | ARROZ QUIRERA |
| 00105 | ARROZ LISO |
| 00106 | ARROZ MACERADO |
| 00107 | ARROZ PARBOILIZADO |
| 00108 | XEREM DE ARROZ |
| 00109 | ARROZ PARBORIZADO |
| 00110 | ARROZ COLONIAL |
| 00111 | ARROZ AGULHA |
| 00112 | ARROZ AGULHINHA |
| 00113 | ARROZ BRANCO |
| 00114 | ARROZ VERMELHO |
| 00115 | ARROZ AMARELO |
| 00201 | ARROZ INTEGRAL |
| 00202 | ARROZ PILADO |
| 00203 | ARROZ NAO-POLIDO |
| 00204 | ARROZ BENEFICIADO |
| 00301 | ARROZ COM CASCA |
| 00401 | AVEIA EM GRAO |
| 00402 | AVEIA EM MASSA |
| 00403 | MASSA DE AVEIA |
| 00501 | PIPOCA DE ARROZ INDUSTRIALIZADA |
| 00601 | CEVADA EM GRAO |
| 00701 | MILHO EM GRAO |
| 00702 | MILHO BRANCO EM GRAO |
| 00703 | MILHO DE CANJICA |
| 00704 | MILHO CANJIQUINHA |
| 00705 | MILHO DE PIPOCA |
| 00706 | CANJIQUINHA DE MILHO EM GRAO |
| 00707 | XEREM DE MILHO |
| 00708 | QUIRERA DE MILHO |
| 00709 | CANJICA DE MILHO EM GRAO |
| 00710 | MILHO AMARELO EM GRAO |
| 00711 | MILHO DE MUNGUZA |
| 00712 | MILHO ALHO |
| 00713 | MILHO SECO |
| 00714 | MILHO XEREM |
| 00801 | MILHO VERDE EM ESPIGA |
| 00901 | TRIGO EM GRAO |
| 00902 | TRIGO INTEGRAL |
| 00903 | QUIRERA DE TRIGO |
| 00904 | MASSA DE TRIGO |
| 00905 | FARELO DE TRIGO (QUIRERA) |
| 01001 | AMENDOIM EM GRAO IN NATURA |
| 01002 | MENDOIM |
| 01003 | MENDOBIM |
| 01004 | MEDUBIM |
| 01005 | AMENDOIM EM CASCA IN NATURA |
| 01006 | AMENDOIM NATURAL |
| 01101 | ERVILHA EM GRAO |
| 01201 | FAVA EM GRAO |
| 01202 | FAVA DE LIMA EM GRAO |
| 01203 | FEIJOA EM GRAO |
| 01204 | MANGALO AMARGO EM GRAO |
| 01205 | FEIJAO FAVA EM GRAO |
| 01301 | FEIJAO MANTEIGA |
| 01302 | FEIJAO BICO DE OURO |
| 01303 | FEIJAO CAETE |
| 01304 | FEIJAO PINGO DE OURO |
| 01305 | FEIJAO GRAO DE OURO |
| 01306 | FEIJAO BRANCO |
| 01307 | FEIJAO LOUCA |
| 01308 | FEIJAO CANARINHO |
| 01309 | FEIJAO BOLINHA |
| 01310 | FEIJAO PITOCO |
| 01311 | FEIJAO DOURADINHO |
| 01401 | FEIJAO MULATINHO |
| 01402 | FEIJAO COFELISTA |
| 01403 | FEIJAO PAULISTA |
| 01404 | FEIJAO MOURO |
| 01405 | FEIJAO SESSENTA DIAS |
| 01406 | FEIJAO MULATA GORDA |
| 01407 | FEIJAO MARROM |
| 01408 | FEIJAO MORENINHO |
| 01409 | FEIJAO CAFE COM LEITE |
| 01410 | FEIJAO NAGE |
| 01411 | FEIJAO NAVEGADOR MARROM |
| 01412 | FEIJAO CEARENCE |
| 01501 | FEIJAO PRETO |
| 01502 | FEIJAO ESCURINHO |
| 01503 | FEIJAO FLORESTA NEGRA |
| 01504 | FEIJAO BORBAO |
| 01505 | FEIJAO QUEBRANCHO |
| 01506 | FEIJAO REBENTA NEGRO |
| 01507 | FEIJAO DO MILHO |
| 01508 | FEIJAO BEIRA LINHA |
| 01509 | FEIJAO ITALIANINHO |
| 01510 | FEIJAO BANDINHA PRETO |
| 01511 | FEIJAO CAVALO |
| 01512 | FEIJAO DE COR |
| 01513 | FEIJAO PRETO BANDINHA |
| 01601 | FEIJAO FRADINHO |
| 01602 | FEIJAO MACASSAR |
| 01603 | FEIJAO DE CORDA |
| 01604 | FEIJAO DE METRO |
| 01605 | FEIJAO ORELHA DE FRADE |
| 01606 | FEIJAO CORUJA |
| 01607 | FEIJAO DE MOITA |
| 01608 | FEIJAO QUARENTINHA |
| 01609 | FEIJAO LIGEIRO |
| 01610 | FEIJAO CATADOR |
| 01611 | FEIJAO MIUDO |
| 01612 | FEIJAO GURGUTUBA |
| 01613 | FEIJAO GURUTUBA |
| 01614 | FEIJAO OLHO DE CABRA |
| 01615 | FEIJAO PENDANGA |
| 01616 | FEIJAO PITIUBA |
| 01617 | FEIJAO QUARENTA DIAS |
| 01618 | FEIJAO BOCA PRETA |
| 01619 | FEIJAO DE FRADE |
| 01620 | FEIJAO DE VARA |
| 01621 | FEIJAO OLHO PRETO |
| 01622 | FEIJAO DO RIO |
| 01623 | FEIJAO IBRA |
| 01624 | FEIJAO DE RAMA |
| 01625 | FEIJAO BAIANO |
| 01626 | FEIJAO VINAGRE |
| 01627 | FEIJAO SEMPRE VERDE |
| 01628 | FEIJAO DE ARRANCA |
| 01629 | FEIJAO BAJE PODRE (MACASSAR) |
| 01630 | FEIJAO BARRIGUDO (DE METRO) |
| 01631 | FEIJAO CANAPU |
| 01632 | FEIJAO CAUPI |
| 01633 | FEIJAO DA COLONIA |
| 01634 | FEIJAO VERDE |
| 01635 | FEIJAO PERUANO |
| 01636 | FEIJAO TREPA PAU |
| 01701 | FEIJAO JALO |
| 01702 | FEIJAO VERMELHO |
| 01703 | FEIJAO MOLEQUE |
| 01704 | FEIJAO ENCARNADO |
| 01705 | FEIJAO GORDO |
| 01706 | FEIJAO ENXOFRAO |
| 01707 | FEIJAO DO SUL |
| 01801 | ARROZ ESPECIAL JAPONES |
| 01802 | ARROZ JAPONES ESPECIAL |
| 01901 | FEIJAO ROXO |
| 01902 | FEIJAO ROCHEDO |
| 01903 | FEIJAO BICO ROXO |
| 01904 | FEIJAO BORDO |
| 01905 | FEIJAO BRABINHO |
| 01906 | FEIJAO MARUMBE |
| 01907 | FEIJAO ROXINHO |
| 01908 | FEIJAO ROXAO |
| 01909 | FEIJAO ROXOTI |
| 01910 | FEIJAO RAPE (ROXO) |
| 01911 | FEIJAO ANAO (ROXO) |
| 02001 | SEMENTE DE LINHACA |
| 02101 | FEIJAO RAJADO |
| 02102 | FEIJAO AMENDOIM |
| 02103 | FEIJAO CHITA FINA |
| 02104 | FEIJAO VERMELHO E BRANCO |
| 02105 | FEIJAO ZEBRINHA |
| 02106 | FEIJAO CASCA DE COCO |
| 02107 | FEIJAO CASCAO |
| 02108 | FEIJAO PINTADO |
| 02109 | FEIJAO PINTADINHO |
| 02110 | FEIJAO RISCADINHO |
| 02111 | FEIJAO CARNAVAL |
| 02112 | FEIJAO GROSSO |
| 02113 | FEIJAO CHOCOLATE |
| 02114 | FEIJAO CARIOCA |
| 02115 | FEIJAO RAJADINHO |
| 02116 | FEIJAO CARIOQUINHA |
| 02201 | FEIJAO ROSINHA |
| 02202 | FEIJAO MARIA ROSA |
| 02203 | FEIJAO MOURA ROSA |
| 02204 | FEIJAO ROSADO |
| 02205 | FEIJAO BARROSINHO |
| 02206 | FEIJAO GANCHEIRO |
| 02207 | FEIJAO PAQUETA |
| 02301 | FEIJAO ENXOFRE |
| 02302 | FEIJAO AMARELO |
| 02303 | FEIJAO OURO |
| 02304 | FEIJAO MINEIRO AMARELO |
| 02305 | FEIJAO MINEIRO |
| 02401 | FEIJAO PARDO |
| 02402 | FEIJAO FUMACA |
| 02501 | FEIJAO CHUMBINHO |
| 02502 | FEIJAO CAFEZINHO |
| 02503 | FEIJAO PAQUINHO |
| 02504 | FEIJAO PACO MINEIRO |
| 02505 | FEIJAO CAQUI |
| 02506 | FEIJAO OPAQUINHO |
| 02507 | FEIJAO CAFE |
| 02508 | FEIJAO TERRINHA |
| 02509 | FEIJAO CARA SUJA |
| 02510 | FEIJAO OPACO |
| 02511 | FEIJAO FIGADO DE GALINHA |
| 02512 | FEIJAO IMPERIAL |
| 02601 | GUANDO |
| 02602 | ANDU |
| 02603 | GUANDU |
| 02604 | FEIJAO GUANDO |
| 02605 | FEIJAO ANDU |
| 02606 | FEIJAO GUINE |
| 02607 | FEIJAO CRISTA |
| 02608 | FEIJAO SETE CAMADAS |
| 02609 | FEIJAO GUANDU |
| 02701 | TREMOCO |
| 02702 | TREMOCO BRANCO |
| 02703 | TREMOCO AMARELO |
| 02801 | GRAO DE BICO |
| 02901 | LENTILHA |
| 03001 | SOJA EM GRAO |
| 03002 | FEIJAO SOJA |
| 03101 | FEIJAO NAO-ESPECIFICADO |
| 03102 | FEIJAO (NAO-ESPECIFICADO) |
| 03201 | SEMENTE DE ABOBORA |
| 03301 | ARROZ PRE-COZIDO |
| 03302 | ARROZ SUICO PRE-COZIDO |
| 03303 | RIZOTO PRE-COZIDO |
| 03304 | RIZOTO CAMPONES PRE-COZIDO |
| 03305 | RIZOTO PRIMAVERA PRE-COZIDO |
| 03401 | ARROZ (NAO-ESPECIFICADO) |
| 03402 | ARROZ NAO-ESPECIFICADO |
| 03501 | QUIRERA NAO-ESPECIFICADA |
| 03502 | QUIRERA (NAO-ESPECIFICADA) |
| 03601 | MILHO DE PIPOCA PARA MICROONDAS |
| 03701 | SOJA EM GRAO ORGANICA |
| 03702 | FEIJAO SOJA ORGANICO |
| 03801 | FEIJAO MULATINHO ORGANICO |
| 03802 | FEIJAO COFELISTA ORGANICO |
| 03803 | FEIJAO PAULISTA ORGANICO |
| 03804 | FEIJAO MOURO ORGANICO |
| 03805 | FEIJAO SESSENTA DIAS ORGANICO |
| 03806 | FEIJAO MULATA GORDA ORGANICO |
| 03807 | FEIJAO MARROM ORGANICO |
| 03808 | FEIJAO MORENINHO ORGANICO |
| 03809 | FEIJAO CAFE COM LEITE ORGANICO |
| 03810 | FEIJAO NAGE ORGANICO |
| 03811 | FEIJAO NAVEGADOR MARROM ORGANICO |
| 03812 | FEIJAO CEARENCE ORGANICO |
| 03901 | FEIJAO PRETO ORGANICO |
| 03902 | FEIJAO ESCURINHO ORGANICO |
| 03903 | FEIJAO FLORESTA NEGRA ORGANICO |
| 03904 | FEIJAO BORBAO ORGANICO |
| 03905 | FEIJAO QUEBRANCHO ORGANICO |
| 03906 | FEIJAO REBENTA NEGRO ORGANICO |
| 03907 | FEIJAO DO MILHO ORGANICO |
| 03908 | FEIJAO BEIRA LINHA ORGANICO |
| 03909 | FEIJAO ITALIANINHO ORGANICO |
| 03910 | FEIJAO BANDINHA PRETO ORGANICO |
| 03911 | FEIJAO CAVALO ORGANICO |
| 03912 | FEIJAO DE COR ORGANICO |
| 03913 | FEIJAO PRETO BANDINHA ORGANICO |
| 04001 | FEIJAO FRADINHO ORGANICO |
| 04002 | FEIJAO MACASSAR ORGANICO |
| 04003 | FEIJAO DE CORDA ORGANICO |
| 04004 | FEIJAO DE METRO ORGANICO |
| 04005 | FEIJAO ORELHA DE FRADE ORGANICO |
| 04006 | FEIJAO CORUJA ORGANICO |
| 04007 | FEIJAO DE MOITA ORGANICO |
| 04008 | FEIJAO QUARENTINHA ORGANICO |
| 04009 | FEIJAO LIGEIRO ORGANICO |
| 04010 | FEIJAO CATADOR ORGANICO |
| 04011 | FEIJAO MIUDO ORGANICO |
| 04012 | FEIJAO GURGUTUBA ORGANICO |
| 04013 | FEIJAO GURUTUBA ORGANICO |
| 04014 | FEIJAO OLHO DE CABRA ORGANICO |
| 04015 | FEIJAO PENDANGA ORGANICO |
| 04016 | FEIJAO PITIUBA ORGANICO |
| 04017 | FEIJAO QUARENTA DIAS ORGANICO |
| 04018 | FEIJAO BOCA PRETA ORGANICO |
| 04019 | FEIJAO DE FRADE ORGANICO |
| 04020 | FEIJAO DE VARA ORGANICO |
| 04021 | FEIJAO OLHO PRETO ORGANICO |
| 04022 | FEIJAO DO RIO ORGANICO |
| 04023 | FEIJAO IBRA ORGANICO |
| 04024 | FEIJAO DE RAMA ORGANICO |
| 04025 | FEIJAO BAIANO ORGANICO |
| 04026 | FEIJAO VINAGRE ORGANICO |
| 04027 | FEIJAO SEMPRE VERDE ORGANICO |
| 04028 | FEIJAO DE ARRANCA ORGANICO |
| 04029 | FEIJAO BAJE PODRE (MACASSAR) ORGANICO |
| 04030 | FEIJAO BARRIGUDO (DE METRO) ORGANICO |
| 04031 | FEIJAO CANAPU ORGANICO |
| 04032 | FEIJAO CAUPI ORGANICO |
| 04033 | FEIJAO DA COLONIA ORGANICO |
| 04034 | FEIJAO VERDE ORGANICO |
| 04035 | FEIJAO PERUANO ORGANICO |
| 04101 | FEIJAO NAO-ESPECIFICADO ORGANICO |
| 04102 | FEIJAO (NAO-ESPECIFICADO) ORGANICO |
| 04201 | MILHO DE PIPOCA PARA MICROONDAS LIGHT |
| 04202 | MILHO DE PIPOCA LIGHT PARA MICROONDAS |
| 04301 | ARROZ POLIDO ORGANICO |
| 04302 | ARROZ HIBRIDO ORGANICO |
| 04303 | ARROZ BICA CORRIDA ORGANICO |
| 04304 | ARROZ QUIRERA ORGANICO |
| 04305 | ARROZ LISO ORGANICO |
| 04306 | ARROZ MACERADO ORGANICO |
| 04307 | ARROZ PARBOILIZADO ORGANICO |
| 04308 | XEREM DE ARROZ ORGANICO |
| 04309 | ARROZ PARBORIZADO ORGANICO |
| 04310 | ARROZ COLONIAL ORGANICO |
| 04311 | ARROZ AGULHA ORGANICO |
| 04312 | ARROZ AGULHINHA ORGANICO |
| 04313 | ARROZ BRANCO ORGANICO |
| 04314 | ARROZ VERMELHO ORGANICO |
| 04315 | ARROZ AMARELO ORGANICO |
| 04401 | ARROZ INTEGRAL ORGANICO |
| 04402 | ARROZ PILADO ORGANICO |
| 04403 | ARROZ NAO-POLIDO ORGANICO |
| 04404 | ARROZ BENEFICIADO ORGANICO |
| 04501 | ARROZ COM CASCA ORGANICO |
| 04601 | FEIJAO DA PRAIA |
| 04701 | LINHACA DOURADA |
| 04801 | SORGO EM GRAO |
| 04901 | QUINOA |
| 00101 | BATATA INGLESA |
| 00102 | BATATA DO REINO |
| 00103 | BATATA ROSA |
| 00104 | BATATA PORTUGUESA |
| 00105 | BATATA INGESA ROSA |
| 00106 | BATATINHA |
| 00107 | BATATA LISA |
| 00108 | BATATA HOLANDESA |
| 00109 | BATATA BINGE |
| 00110 | BATATA BRANCA |
| 00201 | ARARUTA |
| 00202 | MASSA DE ARARUTA |
| 00301 | BATATA BAROA |
| 00302 | BATATA BARONESA |
| 00303 | MANDIOQUINHA SALSA (BATATA BAROA) |
| 00304 | CENOURA AMARELA (BATATA BAROA) |
| 00305 | CEREFOLIO |
| 00401 | BATATA DOCE |
| 00402 | BATATA DOCE ROXA |
| 00403 | BATATA DA TERRA |
| 00404 | BATATA DE ANGOLA |
| 00405 | BATATA MASTRUS |
| 00406 | BATATA ROXA |
| 00501 | INHAME |
| 00502 | INHAME TAIA |
| 00503 | INHAME SAO TOME |
| 00504 | INHAME CHINES |
| 00505 | INHAME DA COSTA |
| 00506 | INHAME DE AGUA |
| 00507 | TAIOBA SAO TOME INHAME |
| 00508 | QUICARE |
| 00509 | TAIA JAPONES |
| 00510 | COIO ROSA |
| 00601 | MANDIOCA |
| 00602 | MANDIOCA DOCE |
| 00603 | MANDIOCA MANSA |
| 00604 | MANDIOCA BRANCA |
| 00605 | MANDIOCA CACAU |
| 00606 | MANDIOCA BRAVA |
| 00607 | MANDIOCA VASSOURINHA |
| 00608 | MANIVA |
| 00609 | AIPIM |
| 00610 | MACAXEIRA |
| 00611 | AIPI |
| 00612 | AIMPIM |
| 00613 | MANDIOCA SEM CASCA |
| 00614 | MASSA DE MACAXEIRA |
| 00701 | CARA |
| 00702 | CARA INHAME |
| 00703 | CARA CHINES |
| 00704 | CARA BRANCO |
| 00705 | CARA DA GUINE |
| 00706 | CARA DE SAPATEIRO |
| 00707 | CARA DO AR |
| 00708 | CARA MIMOSO |
| 00709 | CARATINGA |
| 00710 | ARRAS |
| 00711 | BATATA NAMBU |
| 00712 | INAMBU (CARA) |
| 00713 | INHAME CARAQUENTO (CARA) |
| 00714 | INHAME LISO (CARA) |
| 00715 | INHAME CASCO (CARA) |
| 00716 | BATATA DO AR |
| 00717 | BATATA DE RAMA |
| 00718 | CARA NAMBU |
| 00801 | BATATA NAO-ESPECIFICADA |
| 00802 | BATATA (NAO-ESPECIFICADA) |
| 00901 | BATATA AIPO |
| 00902 | BATATA SALSA |
| 00903 | BATATA DO BARAO |
| 00904 | BATATA SUICA |
| 00905 | BATATA CENOURA |
| 00906 | BATATA FIUZA |
| 00907 | AIPO DO PERU (BATATA AIPO) |
| 00908 | MANDIOQUINHA |
| 01001 | RABANETE |
| 01002 | NABO CHINES (RABANETE) |
| 01101 | BETERRABA |
| 01201 | CENOURA |
| 01202 | CENOURA VERMELHA |
| 01203 | CENOURINHA |
| 01301 | NABO |
| 01302 | NABO ROXO |
| 01303 | NABO BRANCO |
| 01401 | NABICA |
| 01402 | SARAMAGO |
| 01403 | RABANETE DE CAVALO (NABICA) |
| 01501 | GOBO |
| 01502 | BARDANA |
| 01601 | ACAFRAO |
| 01701 | GENGIBRE |
| 01801 | BATATA INGLESA ORGANICA |
| 01802 | BATATA DO REINO ORGANICA |
| 01803 | BATATA ROSA ORGANICA |
| 01804 | BATATA PORTUGUESA ORGANICA |
| 01805 | BATATA INGESA ROSA ORGANICA |
| 01806 | BATATINHA ORGANICA |
| 01807 | BATATA LISA ORGANICA |
| 01808 | BATATA HOLANDESA ORGANICA |
| 01809 | BATATA BINGE ORGANICA |
| 01901 | CENOURA ORGANICA |
| 01902 | CENOURA VERMELHA ORGANICA |
| 01903 | CENOURINHA ORGANICA |
| 02001 | BETERRABA ORGANICA |
| 00101 | CREME DE ARROZ |
| 00102 | CREME DE ARROZ VITAMINADO |
| 00103 | FARINHA DE ARROZ |
| 00104 | ARROZINA |
| 00105 | MUCILON |
| 00106 | MASSA DE ARROZ |
| 00107 | MUCILON DE ARROZ |
| 00108 | MINGAU DE ARROZ |
| 00109 | NUTRILON DE ARROZ |
| 00201 | FLOCOS DE ARROZ |
| 00202 | CROQUINHOS DE ARROZ |
| 00203 | FLOCAO DE ARROZ |
| 00204 | CEREAL MATINAL DE ARROZ EM FLOCOS |
| 00301 | FARINHA DE AVEIA |
| 00401 | AVEIA EM FLOCOS |
| 00402 | FLOCOS DE AVEIA |
| 00403 | AVEIA INSTANTANEA |
| 00404 | VITAVENA |
| 00501 | FARINHA DE CENTEIO |
| 00601 | FUBA DE MILHO |
| 00602 | FUBA |
| 00603 | FARINHA DE MILHO |
| 00604 | MILHARINA |
| 00605 | FLOR DE MILHO |
| 00606 | SEMOLA DE MILHO |
| 00607 | MASSA DE MILHO |
| 00608 | PUBA DE MILHO |
| 00609 | PUBA DE FUBA |
| 00610 | MASSA DE CANJICA |
| 00611 | FECULA DE MILHO |
| 00612 | MUCILON DE MILHO |
| 00613 | MILHO MOIDO |
| 00614 | MINGAU DE MILHO |
| 00615 | FARINHA DE FUBA |
| 00616 | MASSA DE PUBA |
| 00617 | MASSA PARA PAMONHA |
| 00618 | FARINHA AMARELA |
| 00701 | MAIZENA |
| 00702 | AMIDO DE MILHO |
| 00703 | GOMA DE MILHO |
| 00704 | AMIDOMIL |
| 00705 | AMIDO DE ARROZ |
| 00706 | AMIDO DE MANDIOCA |
| 00707 | MILHENA NUTRIL 5 |
| 00801 | CREMOGEMA |
| 00802 | VITAMILHO |
| 00803 | CREMOVITA |
| 00804 | CREME DE MILHO |
| 00805 | CREMOGEMA VITAMINADA |
| 00901 | FLOCOS DE MILHO |
| 00902 | SUCRILHOS DE MILHO |
| 00903 | CEREAL MATINAL DE MILHO EM FLOCOS |
| 00904 | FLOCAO DE MILHO |
| 01001 | FARINHA DE TRIGO |
| 01002 | FARINHA DO REINO |
| 01003 | SEMOLINA DE TRIGO |
| 01004 | FARINHA DE TRIGO COM FERMENTO |
| 01005 | FECULA DE TRIGO |
| 01006 | FARINHA DE TRIGO INTEGRAL |
| 01101 | FARINHA DE ROSCA |
| 01201 | FARINHA DE QUIBE |
| 01202 | FARINHA DE TRIGO DE QUIBE |
| 01203 | TRIGUILHO |
| 01204 | BULGOR |
| 01205 | TRIGO PARA QUIBE |
| 01301 | GERME DE TRIGO |
| 01302 | GLUTEN |
| 01303 | FIBRA DE TRIGO |
| 01304 | FIBRA DE CEREAL TRIGO |
| 01401 | FARINHA DE MANDIOCA |
| 01402 | FARINHA DE MANDIOCA CRUA |
| 01403 | FARINHA DE MANDIOCA TORRADA |
| 01404 | FARINHA DE MANDIOCA BIJU |
| 01405 | FARINHA DE MANDIOCA MORENA |
| 01406 | FARINHA DE MANDIOCA AMARELA |
| 01407 | FARINHA DE MANDIOCA BRANCA |
| 01408 | FARINHA DE MANDIOCA MISTURADA |
| 01409 | FARINHA DE MANDIOCA COMUM |
| 01410 | FARINHA DE COPIOBA |
| 01411 | FARINHA DE MESA |
| 01412 | FARINHA DE CARIMA |
| 01413 | FARINHA DE SURUI |
| 01414 | MASSA DE MANDIOCA |
| 01415 | FARINHA DE AGUA |
| 01416 | CRUERA |
| 01417 | FARINHA SECA |
| 01418 | FARINHA DE MANDIOCA TEMPERADA |
| 01419 | FARINHA DE MANDIOCA FLOCADA |
| 01501 | FECULA DE MANDIOCA |
| 01502 | GOMA DE MANDIOCA |
| 01503 | POLVILHO DE MANDIOCA |
| 01504 | POLVILHO AZEDO |
| 01505 | SAGU DE MANDIOCA |
| 01506 | GOMA DE CARIMA |
| 01507 | CREME DE MACAXEIRA |
| 01508 | GOMA DE TAPIOCA |
| 01509 | TAPIOCA GOMA |
| 01510 | FARINHA DE TAPIOCA |
| 01511 | FARINHA BEIJU |
| 01512 | POLVILHO DOCE |
| 01513 | CARIMA DE MANDIOCA |
| 01514 | PUBA DE MANDIOCA |
| 01515 | SAGU DE TAPIOCA |
| 01516 | TAPIOCA DE GOMA |
| 01517 | FARINHA DE GOMA |
| 01518 | FARINHA DE PUBA |
| 01519 | MASSA DE TAPIOCA |
| 01601 | FARINHA NAO-ESPECIFICADA |
| 01701 | FARINHA DE ARARUTA |
| 01801 | FECULA DE ARARUTA |
| 01802 | GOMA DE ARARUTA |
| 01901 | FARINHA DE SOJA |
| 02001 | FARINHA LACTEA |
| 02101 | NESTON |
| 02102 | FLOCOS DE CEREAIS |
| 02103 | ALIMENTOS INFANTIS QUATRO CEREAIS |
| 02104 | EXTRATO DE CEREAIS |
| 02105 | VITAFLOCOS |
| 02201 | MASSA DE PAO COMUM |
| 02202 | MISTURA PARA PAO DE TRIGO |
| 02301 | MASSA DE PAO DE QUEIJO |
| 02302 | PAO DE QUEIJO SEMIPRONTO |
| 02401 | MASSA DE PIZZA |
| 02402 | PIZZA SEMIPRONTA |
| 02403 | MINI PIZZA SEMIPRONTA |
| 02404 | MASSA PARA PIZZA |
| 02501 | FIBRA DE SOJA |
| 02601 | MASSA DE PASTEL |
| 02602 | FILHO EM MASSA PARA PASTEL |
| 02603 | MINI PASTEL |
| 02604 | PASTEL SEMIPRONTO |
| 02605 | MASSA PARA PASTEL |
| 02701 | MASSA DE NHOQUE |
| 02702 | NHOQUE SEMIPRONTO |
| 02703 | INHOQUE SEMIPRONTO |
| 02801 | MASSA DE RAVIOLE |
| 02802 | RAVIOLE SEMIPRONTO |
| 02901 | MASSA DE LASANHA |
| 02902 | LASANHA SEMIPRONTA |
| 02903 | MACARRAO PARA LASANHA |
| 02904 | MASSA PARA LASANHA |
| 02905 | LASANHA A BOLONHESA SEMIPRONTA |
| 03001 | MASSA DE CANELONE |
| 03002 | CANELONE SEMIPRONTO |
| 03101 | MASSA DE CAPELETI |
| 03102 | CAPELETI SEMIPRONTO |
| 03103 | ANHOLINE SEMIPRONTO |
| 03201 | MACARRAO SEM OVOS |
| 03202 | MACARRAO DE GLUTEN SEM OVOS |
| 03203 | MACARRAO DE SEMOLA SEM OVOS |
| 03204 | MACARRAO DE SEMOLINA SEM OVOS |
| 03205 | MACARRAO DE SOPA SEM OVOS |
| 03206 | MASSA SEM OVOS |
| 03207 | MASSA DE GLUTEN SEM OVOS |
| 03208 | MASSA DE SEMOLA SEM OVOS |
| 03209 | MASSA DE SEMOLINA SEM OVOS |
| 03210 | MASSA DE SOPA SEM OVOS |
| 03211 | TALHARIM SEM OVOS |
| 03212 | SPAGHETTI SEM OVOS |
| 03213 | ESPAGUETE SEM OVOS |
| 03214 | ALETRIA SEM OVOS |
| 03215 | MACARRAO SEM COLESTEROL |
| 03216 | MACARRAO VITAMINADO |
| 03217 | MACARRAO PARAFUSO SEM OVOS |
| 03218 | MACARRAO COM ESPINAFRE |
| 03219 | MACARRAO COM CENOURA |
| 03220 | MACARRAO COM MILHO |
| 03221 | MACARRAO PRE-COZIDO |
| 03222 | MACARRAO PASTEURIZADO |
| 03223 | MACARRAO PICADO |
| 03224 | MACARRAO PENNE SEM OVOS |
| 03301 | MACARRAO COM OVOS |
| 03302 | MACARRAO DE GLUTEN COM OVOS |
| 03303 | MACARRAO DE SEMOLA COM OVOS |
| 03304 | MACARRAO DE SEMOLINA COM OVOS |
| 03305 | MACARRAO DE SOPA COM OVOS |
| 03306 | MASSA COM OVOS |
| 03307 | MASSA DE GLUTEN COM OVOS |
| 03308 | MASSA DE SEMOLA COM OVOS |
| 03309 | MASSA DE SEMOLINA COM OVOS |
| 03310 | MASSA DE SOPA COM OVOS |
| 03311 | TALHARIM COM OVOS |
| 03312 | SPAGHETTI COM OVOS |
| 03313 | ESPAGUETE COM OVOS |
| 03314 | ALETRIA COM OVOS |
| 03315 | MACARRAO PARAFUSO COM OVOS |
| 03316 | MACARRAO PENNE COM OVOS |
| 03401 | MACARRAO (NAO-ESPECIFICADO) |
| 03402 | MACARRAO CASEIRO |
| 03403 | MACARRAO COM SEMOLA (NAO-ESPECIFICADO) |
| 03404 | ESPAGUETE (NAO-ESPECIFICADO) |
| 03405 | MACARRAO PARAFUSO (NAO-ESPECIFICADO) |
| 03406 | TALHARIM (NAO-ESPECIFICADO) |
| 03407 | MASSA DE SEMOLA (NAO-ESPECIFICADA) |
| 03408 | MASSA DE SOPA (NAO-ESPECIFICADA) |
| 03409 | MASSA (NAO-ESPECIFICADA) |
| 03410 | MACARRAO NAO-ESPECIFICADO |
| 03411 | MACARRAO COM SEMOLA NAO-ESPECIFICADO |
| 03412 | ESPAGUETE NAO-ESPECIFICADO |
| 03413 | MACARRAO PARAFUSO NAO-ESPECIFICADO |
| 03414 | TALHARIM NAO-ESPECIFICADO |
| 03415 | MASSA DE SEMOLA NAO-ESPECIFICADA |
| 03416 | MASSA DE SOPA NAO-ESPECIFICADA |
| 03417 | MASSA NAO-ESPECIFICADA |
| 03501 | PURE DE BATATA EM CAIXA |
| 03601 | BIFE VEGETAL |
| 03602 | MASSA DE SOJA |
| 03603 | CARNE VEGETAL |
| 03604 | PASTA DE SOJA |
| 03605 | CARNE DE SOJA |
| 03606 | ALIMENTO DE SOJA |
| 03701 | FARINHA DE AMENDOIM |
| 03702 | AMENDOIM MOIDO |
| 03801 | FARINHA DE PEIXE |
| 03802 | FARINHA DE MANDI |
| 03803 | PIRACUI (FARINHA DE PEIXE) |
| 03901 | FARINHA DE FEIJAO |
| 04001 | FARINHA DE BANANA |
| 04101 | GRANOLA |
| 04102 | FLOCOS DE GRANOLA |
| 04201 | MASSA DE COXINHA |
| 04202 | COXINHA DE FRITAR |
| 04203 | COXINHA SEMIPRONTA |
| 04301 | MASSA DE ROTOLONE |
| 04302 | ROTOLONE SEMIPRONTO |
| 04401 | MASSA DE CALZONE |
| 04402 | CALZONE SEMIPRONTO |
| 04501 | ANGU DE MILHO SEMIPRONTO |
| 04502 | POLENTA SEMIPRONTA |
| 04503 | MASSA PARA POLENTA |
| 04504 | MISTURA PARA POLENTA |
| 04505 | POLENTINA |
| 04601 | MUSLI |
| 04701 | MIX DE CEREAIS |
| 04801 | MIOJO |
| 04802 | MACARRAO INSTANTANEO |
| 04803 | MASSA INSTANTANEA |
| 04901 | MACARRAO INTEGRAL |
| 05001 | FARINHA VITAMINADA |
| 05002 | FARINHA DE CEREAIS VITAMINADA |
| 05101 | SUSTAGEM |
| 05102 | NOVOMILK EM PO |
| 05201 | GEVRAL |
| 05301 | LECITINA |
| 05401 | MERITENE |
| 05501 | PROTEINAC |
| 05601 | PROTEINA DE SOJA |
| 05602 | COMPLEMENTO VITAMINICO DE SOJA |
| 05603 | PROTEINA VEGETAL |
| 05701 | COMPLEMENTO ALIMENTAR DE QUALQUER SABOR |
| 05702 | COMPOSTO ALIMENTAR DE QUALQUER SABOR |
| 05801 | SUPLIGEM |
| 05901 | FLOCOS DE MILHO LIGHT |
| 05902 | SUCRILHOS DE MILHO LIGHT |
| 05903 | CEREAL MATINAL DE MILHO EM FLOCOS LIGHT |
| 06001 | MASSA DE PIZZA LIGHT |
| 06002 | PIZZA SEMIPRONTA LIGHT |
| 06003 | MINI PIZZA SEMIPRONTA LIGHT |
| 06101 | MASSA DE NHOQUE LIGHT |
| 06102 | NHOQUE SEMIPRONTO LIGHT |
| 06201 | MASSA DE LASANHA LIGHT |
| 06202 | LASANHA SEMIPRONTA LIGHT |
| 06301 | MIOJO LIGHT |
| 06302 | MACARRAO INSTANTANEO LIGHT |
| 06303 | MASSA INSTANTANEA LIGHT |
| 06401 | PROTEINA DE SOJA ORGANICA |
| 06402 | COMPLEMENTO VITAMINICO DE SOJA ORGANICA |
| 06403 | PROTEINA VEGETAL ORGANICA |
| 06501 | FECULA DE BATATA |
| 06601 | FAGOTINI |
| 06701 | MASSA DE PANQUECA |
| 06801 | FARINHA DE LINHACA |
| 06901 | MACARRAO SEMI PRONTO |
| 07001 | MASSA DE EMPADA (EMPADA SEMIPRONTA) |
| 07101 | FARINHA DE MARACUJA |
| 00101 | COCO DA BAHIA |
| 00102 | COCO DA PRAIA |
| 00103 | COCO DA INDIA |
| 00104 | COCO DA BAHIA SECO |
| 00105 | COCO DA BAHIA VERDE |
| 00201 | COCO RALADO INDUSTRIALIZADO |
| 00301 | CASTANHA PORTUGUESA |
| 00302 | CASTANHA DA EUROPA |
| 00401 | PINHAO |
| 00402 | PINHAO DO PARANA |
| 00403 | PINHA DO PARANA (PINHAO) |
| 00501 | AMENDOA |
| 00601 | AVELA |
| 00701 | CASTANHA DO PARA |
| 00702 | CASTANHA MARANHENSE |
| 00703 | AMENDOA DA AMERICA (CASTANHA DO PARA) |
| 00801 | CASTANHA DE CAJU |
| 00901 | COCO OURICURI |
| 00902 | ARICUI |
| 00903 | ARIRI |
| 00904 | NICURI |
| 00905 | ALICURI |
| 00906 | COCO ARICURI |
| 00907 | COCO ALICURI |
| 00908 | COCO ARIRI |
| 00909 | COCO NICURI |
| 00910 | OURICURI |
| 00911 | ARICURI |
| 00912 | BUTIA |
| 01001 | COCO DE BABACU |
| 01002 | COCO BABACU |
| 01003 | COCO BAUACU |
| 01004 | COCO BAGUACU |
| 01005 | BABACU |
| 01006 | BAUACU |
| 01007 | BAGUACU |
| 01008 | AMENDOA DE BABACU |
| 01101 | COCO DE TUCUM |
| 01102 | COCO DE TICUM |
| 01103 | COCO DE NATAL |
| 01104 | COCO MARAJA |
| 01105 | TUCUM |
| 01106 | TICUM |
| 01107 | MARAJA |
| 01108 | TUCUMA |
| 01201 | COCO MACAUBA |
| 01202 | COCO DE MACAUBA |
| 01203 | COCO DE CATARRO |
| 01204 | COCO DE ESPINHO |
| 01205 | COCO MACAIBA |
| 01206 | COCO BOCAIUVA |
| 01207 | COCO MACAJUBA |
| 01208 | COCO MUCAJA |
| 01209 | COCO MUCAIA |
| 01210 | COCO MOCAJUBA |
| 01211 | MACAUBA |
| 01212 | MACAIBA |
| 01213 | BOCAIUVA |
| 01214 | MACAJUBA |
| 01215 | MUCAIA |
| 01216 | MOCAJUBA |
| 01301 | COCO DENDE |
| 01302 | COCO DE DENDE |
| 01303 | DENDE |
| 01401 | COCO BABAO |
| 01402 | COCO CATOLE |
| 01403 | COCO AMARGOSO |
| 01404 | COCO DE QUARESMA |
| 01405 | BABAO |
| 01406 | CATOLE |
| 01501 | NOZ |
| 01601 | COCO INAJA |
| 01602 | COCO ANAJA |
| 01603 | COCO NAJA |
| 01604 | COCO ANAIA |
| 01605 | INAJA |
| 01606 | ANAJA |
| 01607 | NAJA |
| 01608 | ANAIA |
| 01701 | ACAI EM EMULSAO |
| 01702 | COCO JUCARA |
| 01703 | COCO JICARA |
| 01704 | COCO UACAI |
| 01705 | COCO PINA |
| 01706 | ACAI |
| 01707 | JUCARA |
| 01708 | JICARA |
| 01709 | UACAI |
| 01710 | PINA |
| 01711 | ACAI FRUTA |
| 01712 | JUSSARA |
| 01713 | EMULSAO DE ACAI |
| 01714 | ACAI EM PAPA |
| 01715 | PAPA DE ACAI |
| 01716 | VINHO DE ACAI |
| 01801 | COCO DE PUPUNHA |
| 01802 | COCO DE PIRAJA |
| 01803 | COCO PUPUNHA |
| 01804 | COCO PIRAJA |
| 01805 | PUPUNHA |
| 01806 | PIRAJA |
| 01901 | COCO BACABA |
| 01902 | BACABA |
| 01903 | BACABINHA |
| 01904 | BACABUCU |
| 01905 | BACABAI |
| 01906 | COCO BACABAI |
| 01907 | COCO BACABINHA |
| 01908 | COCO BACABUCU |
| 01909 | BACABA EM EMULSAO |
| 01910 | CAROCO DE BACABA |
| 01911 | EMULSAO DE BACABA |
| 01912 | BACABA EM PAPA |
| 01913 | PAPA DE BACABA |
| 02001 | CASTANHA DA INDIA |
| 02101 | COCO BURITI |
| 02102 | MASSA DE BURITI |
| 02103 | COCO MIRITI |
| 02104 | BURITI (COCO) |
| 02201 | PISTACHE |
| 02202 | PISTACIO |
| 02203 | NOZ DE PISTACHE |
| 02301 | BURITI EM EMULSAO |
| 02302 | VINHO DE BURITI |
| 02303 | BURITI EM PAPA |
| 02304 | PAPA DE BURITI |
| 02305 | EMULSAO DE BURITI EM EMULSAO |
| 02401 | COCO NAO-ESPECIFICADO |
| 02501 | CASTANHA NAO-ESPECIFICADA |
| 02601 | PATAUA EM EMULSAO |
| 02602 | EMULSAO DE PATAUA |
| 02603 | PASTA DE PATAUA |
| 02604 | VINHO DE PATAUA |
| 02701 | NOZ (NOGUEIRA) |
| 02801 | PATAUA (COCO) |
| 00101 | ALFACE |
| 00102 | ALFACE PAULISTA |
| 00103 | ALFACE REPOLHUDA |
| 00104 | ALFACE AMARGA |
| 00105 | ALFACE CRESPA |
| 00106 | ALFACE ROMANA |
| 00107 | ALFACE MANTEIGA |
| 00108 | ALFACE LISA |
| 00109 | ALFACE AMERICANA |
| 00110 | ALFACE HIDROPONICA |
| 00111 | ALFACE ROXA |
| 00112 | ALFACE BRANCA |
| 00113 | ALFACE MIMOSA |
| 00201 | BERTALHA |
| 00202 | BRETALHA |
| 00203 | BAIANO |
| 00301 | CHICORIA |
| 00302 | CHICORIA CRESPA |
| 00303 | CHICORIA FRISADA |
| 00304 | ESCAROLA |
| 00305 | ENDIVIA |
| 00401 | COENTRO (VERDURA) |
| 00402 | COENTRO EM FOLHA |
| 00501 | COUVE |
| 00502 | COUVE MANTEIGA |
| 00503 | COUVE REPOLHUDA |
| 00504 | COUVE PALMITO |
| 00505 | COUVE CRESPA |
| 00506 | COUVE DE FOLHAS |
| 00507 | COUVE PICADA |
| 00601 | COUVE FLOR |
| 00701 | ESPINAFRE |
| 00702 | ESPINAFRE JAPONES |
| 00703 | ORENCAO |
| 00704 | ORENCO |
| 00801 | MOSTARDA (VERDURA) |
| 00802 | MOSTARDA EM FOLHA |
| 00901 | REPOLHO |
| 00902 | REPOLHO BRANCO |
| 00903 | REPOLHO ROXO |
| 00904 | REPOLHO VERDE |
| 00905 | REPOLHO LISO |
| 01001 | SALSA |
| 01002 | SALSINHA |
| 01003 | SALSINHA VERDE |
| 01101 | CEBOLINHA |
| 01102 | CEBOLINHA VERDE |
| 01103 | CEBOLINHA DE TODO O ANO |
| 01104 | CEBOLA DE TODO O ANO (CEBOLINHA VERDE) |
| 01201 | CHEIRO VERDE |
| 01202 | SALSA E CEBOLINHA |
| 01203 | COENTRO E CEBOLINHA |
| 01204 | TEMPERO VERDE |
| 01301 | AGRIAO |
| 01302 | AGRIAO COMUM |
| 01303 | AGRIAO DAS HORTAS |
| 01304 | AGRIAO DE AGUA |
| 01305 | AGRIAO ORDINARIO |
| 01306 | AGRIAO DA TERRA |
| 01307 | AGRIAO DOS JARDINS |
| 01308 | AGRIAO HIDROPONICO |
| 01401 | TAIOBA |
| 01402 | TAIOBA SAO TOME |
| 01403 | TAIA |
| 01501 | ACELGA |
| 01502 | SELGA |
| 01503 | ROLESO |
| 01504 | RACUSAI |
| 01505 | BETERRABA BRANCA (ACELGA) |
| 01506 | BETERRABA CAMPESTRE (ACELGA) |
| 01601 | ALMEIRAO |
| 01602 | RADITE |
| 01603 | CHICORIA AMARGA (ALMEIRAO) |
| 01604 | CHICORIA SELVAGEM (ALMEIRAO) |
| 01605 | ALMEIRAO ROXO |
| 01701 | COUVE BROCOLOS |
| 01702 | COUVE BROCOLIS |
| 01703 | BROCOLOS |
| 01704 | BROCOLIS |
| 01705 | BROCOLIS HIBRIDO |
| 01706 | BROCOLIS JAPONES |
| 01801 | SERRALHA |
| 01901 | CARURU |
| 01902 | CARURU AZEDO |
| 01903 | CARURU DA GUINE |
| 01904 | AZEDA |
| 01905 | ROSELA |
| 01906 | CUXA |
| 01907 | VINAGREIRA |
| 01908 | CARIVEL |
| 01909 | QUIABO AZEDO (CARURU) |
| 01910 | QUIABO ROXO (CARURU) |
| 01911 | QUIABO ROSA (CARURU) |
| 01912 | QUIABO DE ANGOLA (CARURU) |
| 01913 | CARIRU |
| 02001 | RUCULA |
| 02002 | RUCOLA |
| 02003 | PINCHAO |
| 02004 | ROQUETE |
| 02101 | JAMBUACU |
| 02102 | ABECEDARIA |
| 02103 | AGRIAO DO PARA (JAMBUACU) |
| 02104 | AGRIAO DO BRASIL (JAMBUACU) |
| 02105 | JAMBU |
| 02201 | LINGUA DE VACA (VERDURA) |
| 02202 | MARIA GORDA |
| 02203 | MARIA GOMBE |
| 02204 | MANJONGOME |
| 02205 | JOAO GOMES |
| 02206 | MARIA GOMES |
| 02207 | LINGUA DE VACA EM FOLHA |
| 02301 | ALCACHOFRA |
| 02302 | ALCACHOFRA BRAVA |
| 02303 | CARDO |
| 02304 | CARDONA |
| 02401 | SAMAMBAIA DO CAMPO |
| 02501 | BREDO |
| 02502 | BREDO DE ESPINHO |
| 02503 | BREDO ROXO |
| 02504 | BREDO VERMELHO |
| 02505 | BREDO VERDADEIRO |
| 02506 | CARURU BRAVO (BREDO) |
| 02507 | CARURU DA MATA (BREDO) |
| 02508 | CARURU GRANDE (BREDO) |
| 02509 | CARURU VERMELHO (BREDO) |
| 02510 | CARURU DE PORCO (BREDO) |
| 02511 | AMARANTO |
| 02512 | CRISTA DE GALO |
| 02601 | AIPO |
| 02602 | AIPO DE AGUA |
| 02603 | AIPO DOS PANTANOS |
| 02604 | SALSAO (AIPO) |
| 02605 | SALSA ROMANA (AIPO) |
| 02701 | COUVE CHINESA |
| 02801 | ERVA DOCE |
| 02802 | FUNCHO |
| 02803 | FUNCHO DE FLORENCA |
| 02804 | RUNCHO |
| 02901 | AZEDINHA |
| 03001 | FOLHA DE MANDIOCA |
| 03002 | FOLHA DE MANIVA |
| 03003 | FOLHA DE AIPIM |
| 03004 | FOLHA DE MACAXEIRA |
| 03101 | BROTO DE FEIJAO |
| 03102 | MOYASHI |
| 03201 | BROTO DE BAMBU |
| 03301 | ABOBORA MOGANGA |
| 03302 | ABOBORA MOCANGA |
| 03303 | ABOBORA MORANGA |
| 03304 | ABOBORA CARNEIRA |
| 03305 | ABOBORA DE PORCO |
| 03306 | MORANGA |
| 03307 | MORANGA JAPONESA |
| 03308 | MORANGA CABUTIA |
| 03309 | ABOBORA MORANGUINHA |
| 03310 | ABOBORA JAPONESA |
| 03401 | ABOBORA MENINA |
| 03402 | ABOBORA GRANDE |
| 03403 | ABOBORA GIGANTE |
| 03404 | ABOBORA BAIANA |
| 03405 | ABOBORA VERMELHA |
| 03406 | ABOBORA DA BAHIA |
| 03407 | ABOBORA MARANHAO |
| 03501 | ABOBORA MELAO |
| 03502 | ABOBORA ALMISCAR |
| 03503 | ABOBORA CATINGA |
| 03504 | ABOBORA CHEIROSA |
| 03601 | ABOBORA DE AGUA |
| 03602 | ABOBORA DAGUA VERDE |
| 03603 | ABOBORA DA PRAIA |
| 03604 | ABOBORA DAGUA |
| 03605 | ABOBORA DA AGUA |
| 03701 | ABOBRINHA |
| 03702 | ABOBRINHA VERDE |
| 03703 | ABOBRINHA ITALIANA |
| 03704 | ABOBRINHA BRASILEIRA |
| 03705 | ABOBORA JAPONESA (ABOBRINHA) |
| 03706 | ABOBORA BAIANINHA (ABOBRINHA) |
| 03707 | CAXI |
| 03708 | XOXO |
| 03709 | ABOBORA VERDE |
| 03801 | ABOBORA DE PESCOCO |
| 03802 | ABOBORA DE CABOCLO |
| 03803 | ABOBORA DO SERTAO |
| 03804 | ABOBORA JACARE |
| 03805 | JERIMUM |
| 03806 | JERIMUM DE LEITE |
| 03807 | JERIMUM CABOCLO |
| 03808 | JERIMUM JACAREZINHO |
| 03809 | ABOBORA CABOCLO |
| 03810 | ABOBORA DE LEITE |
| 03901 | ABOBORA |
| 03902 | ABOBORA COMUM |
| 03903 | ABOBORA MADURA |
| 04001 | PEPINO |
| 04101 | CHUCHU |
| 04102 | MACHUCHU (CHUCHU) |
| 04103 | CAXIXE |
| 04104 | CHOCHO |
| 04105 | MACHUCHO (CHUCHU) |
| 04201 | JILO |
| 04202 | JILO PAULISTA |
| 04203 | JILO JAPONES |
| 04204 | JILO PRETO |
| 04301 | MAXIXE |
| 04302 | MAXIXO |
| 04303 | MAXIXE BRAVO |
| 04304 | MAXIXE DO MATO |
| 04305 | MAXIXOLA |
| 04306 | PEPININHO (MAXIXE) |
| 04307 | PEPINO DE ESPINHO (MAXIXE) |
| 04401 | PALMITO IN NATURA |
| 04402 | PALMITO DOCE IN NATURA |
| 04403 | PALMITO MOLE IN NATURA |
| 04404 | PALMITO AMARGOSO IN NATURA |
| 04405 | PALMITO DO CHAO IN NATURA |
| 04406 | PALMITO ACAI IN NATURA |
| 04407 | PALMITO JUCARA IN NATURA |
| 04408 | CAMARGO (PALMITO IN NATURA) |
| 04409 | GUARIROBA (PALMITO IN NATURA) |
| 04410 | GUEIROBA (PALMITO IN NATURA) |
| 04411 | GUEROBA (PALMITO IN NATURA) |
| 04501 | PIMENTAO |
| 04502 | PIMENTAO VERDE |
| 04503 | PIMENTAO MADURO |
| 04504 | PIMENTAO AMARELO |
| 04505 | PIMENTAO VERMELHO |
| 04601 | PIMENTA MALAGUETA |
| 04602 | PIMENTA CUMARI IN NATURA |
| 04603 | MALAGUETA IN NATURA |
| 04604 | CUMARI IN NATURA |
| 04605 | PIMENTA DE CUMARI IN NATURA |
| 04606 | PIMENTA BRANCA IN NATURA |
| 04607 | PIMENTA MADEIRA IN NATURA |
| 04608 | PIMENTA CHIFRE DE VEADO IN NATURA |
| 04701 | PIMENTA DE CHEIRO |
| 04702 | PIMENTA PITANGA IN NATURA |
| 04703 | PIMENTA MURICI IN NATURA |
| 04704 | PIMENTA CABACINHA IN NATURA |
| 04705 | PIMENTA MURUPI IN NATURA |
| 04706 | PIMENTA BODE IN NATURA |
| 04707 | PIMENTA DOCE IN NATURA |
| 04708 | PIMENTA SINO IN NATURA |
| 04709 | PIMENTA OLHO DE BODE IN NATURA |
| 04710 | PIMENTA MORANGA IN NATURA |
| 04711 | PIMENTA DE CHAPEU IN NATURA |
| 04712 | PIMENTA CHEIROSA |
| 04801 | PIMENTA POMBINHA IN NATURA |
| 04802 | PIMENTA PASSARINHO IN NATURA |
| 04803 | PIMENTA CUMARI MIUDA IN NATURA |
| 04804 | PIMENTINHA CUMARI |
| 04901 | CEBOLINHA BRANCA |
| 05001 | QUIABO |
| 05002 | GOMBO |
| 05003 | QUIGOMBO |
| 05101 | TOMATE |
| 05102 | TOMATE PAULISTA |
| 05103 | TOMATE MACA |
| 05104 | TOMATE PERA |
| 05105 | TOMATE JAPONES |
| 05106 | TOMATE VERDE |
| 05107 | TOMATE CAQUI |
| 05108 | TOMATE CEREJA |
| 05109 | TOMATE SALADA |
| 05201 | VAGEM |
| 05202 | VAGEM MANTEIGA |
| 05203 | FEIJAO VERDE EM VAGEM |
| 05204 | FEIJAO VAGEM |
| 05301 | COGUMELO IN NATURA |
| 05401 | BERINJELA |
| 05402 | BRINJELA |
| 05501 | ERVILHA EM VAGEM |
| 05502 | ERVILHA DE VAGEM |
| 05601 | CABACA |
| 05602 | CABACA PURUNGA |
| 05603 | CABACA VERDE |
| 05604 | CABACA PORONGO |
| 05701 | CEBOLA |
| 05702 | CEBOLA CABECA BRANCA |
| 05703 | CEBOLA BRANCA |
| 05704 | CEBOLA ROXA |
| 05705 | CEBOLA DO REINO |
| 05706 | CEBOLA PERA |
| 05707 | CEBOLA GIGANTE |
| 05708 | CEBOLA VERMELHA |
| 05709 | CEBOLA CABROBO BRANCA |
| 05710 | CEBOLA EM CABECA BRANCA |
| 05711 | CEBOLA ARGENTINA |
| 05712 | CEBOLA DE CABECA |
| 05713 | CEBOLA AMARELA |
| 05714 | CEBOLA NACIONAL |
| 05801 | CATALONHA |
| 05802 | CATALONIA |
| 05901 | COUVE RABANO |
| 05902 | COUVE RABAO |
| 05903 | RABANO PRETO |
| 05904 | COLORABA |
| 05905 | COUVE RABANO PRETO |
| 05906 | COUVE RABANA |
| 06001 | NIRA |
| 06101 | JARDINEIRA (SELETA) |
| 06102 | SELETA (JARDINEIRA) |
| 06201 | ALHO |
| 06202 | ALHO ROXO |
| 06203 | ALHO BRANCO |
| 06204 | ALHO ARGENTINO |
| 06205 | ALHO A GRANEL |
| 06206 | ALHO CABECA |
| 06207 | ALHO EM CABECA |
| 06208 | ALHO DESCASCADO |
| 06301 | ALHO PORRO |
| 06302 | ALHO MACHO |
| 06401 | ABOBORA PAULISTA |
| 06501 | ABOBORA CABOTIAN |
| 06502 | ABOBORA CABOTIA |
| 06503 | ABOBORA CABUTIA |
| 06504 | ABOBORA CABUTIAN |
| 06505 | ABOBORA KABUTIA |
| 06601 | ABOBORA CAXACO |
| 06701 | ABOBORA GOIANINHA |
| 06801 | ABOBORA HIBRIDA |
| 06901 | ABOBORA SECA |
| 07001 | ABOBRINHA CAIPIRA |
| 07101 | ABOBRINHA BRANCA |
| 07201 | ABOBRINHA MIRIM |
| 07301 | BROTO DE ALFAFA |
| 07401 | ALECRIN |
| 07402 | ALECRIM |
| 07501 | ALFAZEMA |
| 07601 | PIMENTA IN NATURA NAO-DISCRIMINADA |
| 07602 | PIMENTA IN NATURA (NAO-DISCRIMINADA) |
| 07603 | PIMENTA NAO ESPECIFICADA IN NATURA |
| 07701 | PALMITO FRESCO ORGANICO |
| 07702 | PALMITO IN NATURA ORGANICO |
| 07703 | PALMITO DOCE IN NATURA ORGANICO |
| 07704 | PALMITO MOLE IN NATURA ORGANICO |
| 07705 | PALMITO AMARGOSO IN NATURA ORGANICO |
| 07706 | PALMITO DO CHAO IN NATURA ORGANICO |
| 07707 | PALMITO ACAI IN NATURA ORGANICO |
| 07708 | PALMITO JUCARA IN NATURA ORGANICO |
| 07709 | CAMARGO (PALMITO IN NATURA) ORGANICO |
| 07710 | GUARIROBA (PALMITO IN NATURA) ORGANICO |
| 07711 | GUEIROBA (PALMITO IN NATURA) ORGANICO |
| 07712 | GUEROBA (PALMITO IN NATURA) ORGANICO |
| 07801 | ALFACE ORGANICA |
| 07802 | ALFACE PAULISTA ORGANICA |
| 07803 | ALFACE REPOLHUDA ORGANICA |
| 07804 | ALFACE AMARGA ORGANICA |
| 07805 | ALFACE CRESPA ORGANICA |
| 07806 | ALFACE ROMANA ORGANICA |
| 07807 | ALFACE MANTEIGA ORGANICA |
| 07808 | ALFACE LISA ORGANICA |
| 07809 | ALFACE AMERICANA ORGANICA |
| 07810 | ALFACE HIDROPONICA ORGANICA |
| 07811 | ALFACE ROXA ORGANICA |
| 07812 | ALFACE LISA HIDRORGANICA |
| 07901 | TOMATE ORGANICO |
| 07902 | TOMATE PAULISTA ORGANICO |
| 07903 | TOMATE MACA ORGANICO |
| 07904 | TOMATE PERA ORGANICO |
| 07905 | TOMATE JAPONES ORGANICO |
| 07906 | TOMATE VERDE ORGANICO |
| 07907 | TOMATE CAQUI ORGANICO |
| 08001 | PIMENTAO ORGANICO |
| 08002 | PIMENTAO VERDE ORGANICO |
| 08003 | PIMENTAO MADURO ORGANICO |
| 08101 | BROCOLIS ORGANICO |
| 08201 | COUVE ORGANICA |
| 08301 | HORTELA (MOLHO) |
| 08302 | HORTELA EM FOLHA |
| 08401 | ESPINAFRE ORGANICO |
| 08501 | PIMENTA DEDO DE MOCA |
| 08601 | VAGEM ORGANICA |
| 08701 | PIMENTA JAPONESA |
| 08801 | PIMENTA CAMBUCI |
| 08901 | COLONIA (CAPIM) |
| 09001 | SALSA ORGANICA |
| 09101 | ALMEIRAO ORGANICO |
| 09201 | ALFAVACA (MOLHO) |
| 09301 | FOLHA DE CENOURA |
| 09401 | MANGERICAO |
| 09402 | MANGERICAO ROXO |
| 09501 | MANJERONA |
| 09601 | CAPIM SANTO |
| 09602 | CAPIM LIMAO |
| 09701 | OREGANO (MOLHO) |
| 09801 | RUCULA ORGANICA |
| 09901 | ASPARGO (IN NATURA) |
| 10001 | FOLHA DE CEBOLA |
| 10101 | MASTRUZ |
| 10102 | MENTRUZ |
| 10103 | MASTRUCO |
| 10201 | COUVE DE BRUXELAS |
| 10301 | AGRIAO ORGANICO |
| 10401 | REPOLHO ORGANICO |
| 10501 | JILO ORGANICO |
| 10601 | PEPINO ORGANICO |
| 10701 | ORA PRO NOBIS |
| 10801 | CAMOMILA (EM FOLHA) |
| 10901 | CARQUEJA (EM FOLHA) |
| 00101 | BANANA DE AGUA |
| 00102 | BANANA NANICA |
| 00103 | BANANA DA CHINA |
| 00104 | BANANA DE ITALIANO |
| 00105 | BANANA MANGALO |
| 00106 | BANANA INGLESA |
| 00107 | BANANA TATU |
| 00108 | BANANA PETICA |
| 00109 | BANANA CATARINA |
| 00110 | BANANA CHORONA |
| 00111 | BANANA PEROA |
| 00112 | BANANA CASCA VERDE |
| 00113 | BANANA ACUCARINA |
| 00114 | BANANA CAMBOTA |
| 00115 | BANANA CATURRA |
| 00116 | BANANA ANA |
| 00117 | BANANA DAGUA |
| 00201 | BANANA PRATA |
| 00202 | BANANA ALTONA |
| 00203 | BANANA AMARELA |
| 00204 | BANANA BICO VERDE |
| 00205 | BANANA BRANCA |
| 00206 | BANANA DE CAMBOEIRO |
| 00207 | BANANA UMBIGO VERDE |
| 00208 | BANANA DA PRATA |
| 00301 | BANANA OURO |
| 00302 | BANANA CRAVO |
| 00303 | BANANA DOURADA |
| 00304 | BANANA IMPERIAL |
| 00305 | BANANA INAJA |
| 00306 | BANANA MARIQUINHA |
| 00307 | BANANA PISANGO REAL |
| 00308 | BANANA REAL |
| 00309 | BANANA URINHO |
| 00310 | BANANINHA |
| 00311 | BANANA CANELA |
| 00401 | BANANA MACA |
| 00402 | BANANA CARAPE |
| 00403 | BANANA COCO |
| 00404 | BANANA LEITE |
| 00405 | BANANA MACA PAINA |
| 00406 | BANANA PEDRA |
| 00407 | BANANA PERA |
| 00408 | BANANA MACAZINHA DA BAHIA |
| 00501 | BANANA FIGO |
| 00502 | BANANA ABOBORA |
| 00503 | BANANA ACU |
| 00504 | BANANA BABA DE BOI |
| 00505 | BANANA BABONA |
| 00506 | BANANA CACAU |
| 00507 | BANANA CAFE |
| 00508 | BANANA CAIXAO |
| 00509 | BANANA CAJU |
| 00510 | BANANA CARAMBOLA |
| 00511 | BANANA CASADA |
| 00512 | BANANA CASCA GROSSA |
| 00513 | BANANA CHOCOLATE |
| 00514 | BANANA CINZA |
| 00515 | BANANA CORUJA |
| 00516 | BANANA DE VELHO |
| 00517 | BANANA JACARE |
| 00518 | BANANA JASMIM |
| 00519 | BANANA MENCI |
| 00520 | BANANA MINEIRA |
| 00521 | BANANA MURICI |
| 00522 | BANANA PAO |
| 00523 | BANANA PARA |
| 00524 | BANANA PAU |
| 00525 | BANANA RIACHAO |
| 00526 | BANANA ROSA |
| 00527 | BANANA SAPA |
| 00528 | BANANA TAJAMAIA |
| 00529 | BANANA TANJA |
| 00530 | BANANA TRES QUINAS |
| 00531 | BANANA VERMELHA |
| 00532 | MASSA DE BANANA |
| 00601 | BANANA MARMELO |
| 00602 | BANANA MARECA |
| 00701 | BANANA ROXA |
| 00702 | BANANA CAMBORI |
| 00703 | BANANA DAS ALMAS |
| 00704 | BANANA MULATA |
| 00705 | BANANA PATROQUIA |
| 00706 | BANANA VINAGRE |
| 00801 | BANANA SAO DOMINGOS |
| 00802 | BANANA RAJADA |
| 00901 | BANANA SAO TOME |
| 00902 | BANANA CURTA |
| 00903 | BANANA DO PARAISO |
| 01001 | BANANA DA TERRA |
| 01002 | BANANA PACOVA |
| 01003 | BANANA PACOVEIRA |
| 01004 | BANANA PACOBEIRA |
| 01005 | BANANA PACOBUCU |
| 01006 | BANANA BURITI |
| 01007 | BANANA CHIFRE DE VACA |
| 01008 | BANANA COMPRIDA |
| 01009 | BANANA FARTA GENTE |
| 01010 | BANANA FARTA HOMEM |
| 01011 | BANANA FARTA VELHACO |
| 01012 | BANANA GRANDE |
| 01013 | BANANA GRANDE AMARELA |
| 01014 | BANANA MARANHENSE |
| 01015 | BANANA PACOVI |
| 01016 | BANANA ANGOLA |
| 01017 | PACOVA |
| 01018 | PACOVA GRANDE |
| 01019 | BANANA PACOVAN |
| 01101 | BANANA (NAO-ESPECIFICADA) |
| 01102 | BANANA NAO-ESPECIFICADA |
| 01201 | LARANJA LIMA |
| 01202 | LARANJA DOCINHA |
| 01203 | LARANJA SERRA DE AGUA |
| 01204 | LARANJA ACUCAR |
| 01205 | LARANJA DO CEU |
| 01206 | LARANJA DE AGUA |
| 01207 | LARANJA CELESTE |
| 01208 | LARANJA MIMO |
| 01209 | LARANJA ILHOA |
| 01210 | LARANJA MEL |
| 01211 | LARANJA PARATI |
| 01212 | LARANJA DA ILHA |
| 01213 | LARANJA MEL ROSA |
| 01214 | LARANJA MIMO DO CEU |
| 01215 | LARANJA BAIAO |
| 01301 | LARANJA SELETA |
| 01302 | LARANJA SELETA DO RIO |
| 01303 | LARANJA PAULISTA |
| 01304 | LARANJA CAMPISTA |
| 01305 | LARANJA MONJOLO |
| 01306 | LARANJA COCO |
| 01307 | LARANJA FOLHA MURCHA |
| 01308 | LARANJA ROSA |
| 01309 | LARANJA CAVALO |
| 01310 | LARANJA FLOR |
| 01311 | LARANJA RUSSA |
| 01312 | LARANJA JOAO NUNES |
| 01313 | LARANJA MACA |
| 01314 | LARANJA FEIJAO CRU |
| 01401 | LARANJA PERA |
| 01402 | LARANJA DE NATAL |
| 01403 | LARANJA NATAL |
| 01404 | LARANJA DE ABRIL |
| 01405 | LARANJA ABRIL |
| 01406 | LARANJA CAPETA |
| 01407 | LARANJA APANAZIA |
| 01408 | LARANJA SABARA |
| 01409 | LARANJA OURO |
| 01410 | LARANJA ACIDA |
| 01411 | LARANJA CASCA FINA |
| 01412 | LARANJA CITRICA |
| 01413 | LARANJA PERA DO RIO |
| 01414 | LARANJA BARAO |
| 01415 | LARANJA CARIOCA |
| 01416 | LARANJA COMUM |
| 01501 | LARANJA BAHIA |
| 01502 | LARANJA DE UMBIGO |
| 01503 | LARANJA SEM CAROCOS |
| 01504 | LARANJA BAIANINHA |
| 01505 | LARANJA TAQUARI |
| 01506 | LARANJA CATARINA |
| 01507 | LARANJA BANANA |
| 01508 | LARANJA VALENCIA |
| 01509 | LARANJA VALENCIANA |
| 01601 | MACADAMIA |
| 01701 | LARANJA DA TERRA |
| 01702 | LARANJA AMARGA |
| 01703 | LARANJA AZEDA |
| 01704 | LARANJA BRANCA |
| 01705 | LARANJA BIGARADE |
| 01706 | LARANJA DE SEVILHA |
| 01707 | LARANJA DE DOCE |
| 01801 | LARANJA (NAO-ESPECIFICADA) |
| 01802 | LARANJA NAO-ESPECIFICADA |
| 01901 | LIMA |
| 01902 | LIMA DA PERSIA |
| 01903 | LIMA DE BICO |
| 01904 | LIMA COMUM |
| 02001 | LIMAO COMUM |
| 02002 | LIMAO AMARGO |
| 02003 | LIMAO AZEDO |
| 02004 | LIMAO DE MOLHO |
| 02005 | LIMAO SILVESTRE |
| 02006 | LIMAO CHINA |
| 02007 | LIMAO ROSA |
| 02008 | LIMAO PAULISTA |
| 02009 | LIMAO MIRIM |
| 02010 | LIMAO TAITI |
| 02011 | LIMAO CRAVO |
| 02012 | LIMAO BRANCO |
| 02013 | LIMAO HAWAI |
| 02014 | LIMAO CAPETA |
| 02101 | LIMAO GALEGO |
| 02102 | LIMAO VERDADEIRO |
| 02103 | LIMAO VERMELHO |
| 02104 | LIMAO BERGAMOTA |
| 02105 | LIMAO BALAO |
| 02106 | LIMAO BOI |
| 02107 | LARANJA SANGUINEA (LIMAO GALEGO) |
| 02108 | LIMAO VINAGRE |
| 02201 | TANGERINA |
| 02202 | MEXERICA |
| 02203 | MIXIRICA |
| 02204 | MEXERICA RIO |
| 02205 | MIXIRICA RIO |
| 02206 | MEXIRICA MURKOT |
| 02207 | MIXIRICA MURKOT |
| 02208 | MEXIRICA POKAN |
| 02209 | MIXIRICA POKAN |
| 02210 | MEXIRICA CRAVO |
| 02211 | MIXIRICA CRAVO |
| 02212 | TANGERINA POKAN |
| 02213 | TANGERINA CRAVO |
| 02214 | MEXIRICA PERA |
| 02215 | MIXIRICA PERA |
| 02216 | POKAN |
| 02217 | BERGAMOTA |
| 02218 | MIMOSA |
| 02219 | TANGERINA MONGORT |
| 02220 | LARANJA MONGORT |
| 02221 | LARANJA CRAVO |
| 02222 | LARANJA MORGOT |
| 02223 | TANJA |
| 02224 | MEXIRICA CARIOCA |
| 02225 | MEXIRICA KAPUTIA |
| 02226 | MEXIRICA MARICOTA |
| 02227 | MARICOTA |
| 02228 | LARANJA POKAN |
| 02229 | MEXIRICA |
| 02230 | MURGOTE (TANGERINA) |
| 02231 | MORICOTA |
| 02301 | CIDRA |
| 02302 | POMELO |
| 02303 | TORANJA |
| 02304 | GREPE |
| 02305 | GRAPE FRUIT |
| 02401 | KINKAN |
| 02402 | KUNQUAT |
| 02403 | CUNQUATE |
| 02404 | LARANJA KUNQUAT |
| 02405 | LARANJA KINKAN |
| 02406 | LARANJA JABUTICABA |
| 02407 | LARANJINHA JAPONESA |
| 02501 | CANA DE ACUCAR |
| 02601 | ABACAXI |
| 02602 | ANANAS |
| 02603 | ABACAXI CAIANO |
| 02604 | ABACAXI PEROLA |
| 02701 | ABACATE |
| 02801 | CAQUI |
| 02802 | CAQUI CHOCOLATE |
| 02803 | CAQUI CAFE |
| 02804 | CAQUI DURO |
| 02805 | CAQUI FUJI |
| 02806 | CAQUI MOLE |
| 02901 | FIGO |
| 02902 | FIGO DA EUROPA |
| 02903 | FIGO DE VALINHOS |
| 03001 | MACA |
| 03002 | MACA ACIDA |
| 03003 | MACA DOCE |
| 03004 | MACA BRANCA |
| 03005 | MACA VERDE |
| 03006 | MACA VERMELHA |
| 03007 | MACA ARGENTINA |
| 03008 | MACA NACIONAL |
| 03009 | MACA FUGI |
| 03010 | MACA GALA |
| 03011 | MACA IMPORTADA |
| 03012 | MACA FUJI |
| 03101 | MAMAO |
| 03102 | MAMAO DA INDIA |
| 03103 | MAMAO MACHO |
| 03104 | MAMAO VERDE |
| 03105 | MAMAO HAVAI |
| 03106 | MAMAO DO AMAZONAS |
| 03107 | MAMAO PAPAIA |
| 03108 | PAPAIA |
| 03109 | MAMAO VERMELHO |
| 03110 | MAMAO BAIANO |
| 03111 | MAMAO FORMOSA |
| 03112 | MAMAO JAPONES |
| 03113 | MAMAO PAULISTA |
| 03114 | MAMAO DE CORDA |
| 03201 | MANGA |
| 03202 | MANGA COQUINHO |
| 03203 | MANGA ESPADINHA |
| 03204 | MANGA UBA |
| 03205 | MANGA CORACAO DE BOI |
| 03206 | MANGA ITAMARACA |
| 03207 | MANGA ROSINHA |
| 03208 | MANGA ROSA |
| 03209 | MANGA COITE |
| 03210 | MANGA CARLOTINHA |
| 03211 | MANGA ESPADA |
| 03212 | MANGA ABOBORA |
| 03213 | MANGA SAPATINHA |
| 03214 | MANGA JASMIM |
| 03215 | MANGUITO |
| 03216 | MANGA BACURI |
| 03217 | MANGA CATANA |
| 03218 | MANGA CORREIO |
| 03219 | MANGA DE FIAPO |
| 03220 | MANGA OURO |
| 03221 | MANGA TOMMY |
| 03222 | MANGA DE CHEIRO |
| 03223 | MANGA DE MESA |
| 03224 | MANGA HADEN |
| 03225 | MANGA PALMER |
| 03226 | MANGA RACHA |
| 03301 | MARACUJA |
| 03302 | MARACUJA MIRIM |
| 03303 | MARACUJA COMPRIDO |
| 03304 | MARACUJA AZUL |
| 03305 | MARACUJA REDONDO |
| 03306 | MARACUJA MELAO |
| 03307 | MARACUJA DE COBRA |
| 03308 | MARACUJA COMUM |
| 03309 | MARACUJA DOCE |
| 03310 | MARACUJA BRANCO |
| 03311 | MARACUJA SUSPIRO |
| 03312 | MARACUJA PEROBA |
| 03313 | MARACUJA SURURU |
| 03314 | MARACUJA ACU |
| 03315 | MARACUJA AMARELO |
| 03316 | MARACUJA MAMAO |
| 03317 | MARACUJAZINHO |
| 03318 | MARACUJA DE BOI |
| 03319 | MARACUJA ESCURO |
| 03320 | MARACUJA GRANDE |
| 03321 | MARACUJA PRETO |
| 03322 | MARACUJA ROXO |
| 03323 | MARACUJA PINTADO |
| 03324 | MARACUJA VERMELHO |
| 03325 | MARACUJA LARANJA |
| 03326 | MARACUJA ENCARNADO |
| 03327 | MARACUJA AZEDO |
| 03328 | MARACUJINA EM FRUTO |
| 03401 | MELANCIA |
| 03501 | MELAO |
| 03502 | MELAO AMARELO |
| 03503 | MELAO ESPANHOL |
| 03504 | MELAO GAUCHO |
| 03505 | MELAO JAPONES |
| 03506 | MELAO CAIPIRA |
| 03507 | MELAO COLONIAL |
| 03508 | MELAO ORANGE |
| 03509 | MELAO PINGO DE MEL |
| 03510 | MELAO VALENCIANO |
| 03511 | MELAO PELE DE SAPO |
| 03601 | PERA |
| 03602 | PERA DE AGUA |
| 03603 | PERA ACIDA |
| 03604 | PERA DAGUA |
| 03701 | PESSEGO |
| 03801 | FRUTA DE CONDE |
| 03802 | PINHA (FRUTA DE CONDE) |
| 03803 | ATA |
| 03901 | UVA |
| 03902 | UVA NIAGARA |
| 03903 | UVA MOSCATEL |
| 03904 | UVA PRETA |
| 03905 | UVA ROSA |
| 03906 | UVA NACIONAL |
| 03907 | UVA VERDE |
| 03908 | UVA ITALIA |
| 03909 | UVA ROXA |
| 03910 | UVA RUBI |
| 03911 | UVA BRANCA |
| 03912 | UVA PASSA |
| 03913 | UVA BENITAKA |
| 03914 | UVA RED GLOBE |
| 03915 | UVA THOMPSON |
| 04001 | JENIPAPO |
| 04002 | JENIPAPA |
| 04003 | JENIPAPINHO |
| 04004 | JENIPA |
| 04005 | JENIPAPO MANSO |
| 04101 | JACA |
| 04102 | JACA DURA |
| 04103 | JACA DA BAHIA |
| 04104 | JACA MOLE |
| 04105 | JACA MANTEIGA |
| 04201 | GOIABA |
| 04202 | ARACA GUAIABA (GOIABA) |
| 04203 | ARACA ACU (GOIABA) |
| 04204 | ARACA GOIABA (GOIABA) |
| 04205 | GUAIABA |
| 04301 | AMEIXA |
| 04302 | AMEIXA VERMELHA |
| 04303 | AMEIXA PRETA |
| 04304 | AMEIXA IMPORTADA |
| 04305 | AMEIXA FRESCA |
| 04401 | CAJU |
| 04501 | CEREJA |
| 04502 | CEREJA GALEGA |
| 04503 | CEREJA DOS PASSAROS |
| 04504 | CEREJA DA EUROPA |
| 04601 | CAJA MANGA |
| 04602 | CAJARANA |
| 04603 | CAJA ACU |
| 04604 | CAJA UMBU |
| 04605 | CAJA GRANDE |
| 04701 | TAPEREBA |
| 04702 | ACAJA (TAPEREBA) |
| 04703 | CAJA PEQUENO |
| 04704 | CAJA MIRIM |
| 04801 | CARAMBOLA |
| 04901 | JABOTICABA |
| 04902 | JABOTICABA DO MATO |
| 04903 | JABOTICABA PAULISTA |
| 04904 | JABOTICABA MURTA |
| 04905 | JABUTICATUBA |
| 04906 | JABOTICATUBA |
| 04907 | JABOTICABA ACU |
| 04908 | JABUTICABA |
| 04909 | JABOTICABA MIUDA |
| 05001 | GRAVIOLA |
| 05101 | ARATICUM |
| 05102 | ARATICUM DE COMER |
| 05103 | ARATICUM MANSO |
| 05104 | PASMADA |
| 05105 | MAROLO |
| 05201 | MORANGO |
| 05202 | MORANGUINHO |
| 05301 | JAMBO |
| 05302 | JAMBO ROSA |
| 05303 | JAMBO AMARELO |
| 05304 | JAMBO VERDADEIRO |
| 05305 | JAMBO MORENO |
| 05306 | JAMBO CHEIROSO |
| 05307 | JAMBO DA INDIA |
| 05308 | JAMBO COMUM |
| 05309 | JAMBO ENCARNADO |
| 05310 | JAMBO VERMELHO |
| 05311 | JAMBO ROXO |
| 05401 | ATEMOIA |
| 05501 | GROSELHA EM FRUTO |
| 05502 | GROSELHA PRETA EM FRUTO |
| 05503 | GROSELHA ESPINHOSA EM FRUTO |
| 05504 | GROSELHA BRANCA EM FRUTO |
| 05505 | GROSELHA VERMELHA EM FRUTO |
| 05601 | MANGABA |
| 05602 | MANGABINHA DO NORTE |
| 05603 | MANGABINHA |
| 05701 | ARACA |
| 05702 | GOIABA ARACA (ARACA) |
| 05801 | NESPERA |
| 05802 | AMEIXA DO CANADA (NESPERA) |
| 05803 | AMEIXA DO JAPAO (NESPERA) |
| 05804 | AMEIXA AMARELA (NESPERA) |
| 05805 | AMEIXA JAPONESA (NESPERA) |
| 05806 | NESPERA DO JAPAO |
| 05901 | FRUTA PAO |
| 06001 | SAPOTI |
| 06002 | SAPOTA |
| 06101 | UXI |
| 06201 | TAMARINDO |
| 06202 | TAMARINO |
| 06301 | CACAU |
| 06401 | PITOMBA |
| 06402 | PITOMBA DA MATA |
| 06403 | PITOMBA DE MACACO |
| 06501 | BACURI |
| 06502 | IBACUPARI |
| 06503 | BACURIUBA |
| 06504 | BACURIUVA |
| 06505 | IBACURUPARI |
| 06601 | INGA |
| 06602 | INGA CIPO |
| 06603 | INGA FEIJAO |
| 06604 | INGA DEDO |
| 06605 | INGA MIRIM |
| 06606 | INGA DA PRAIA |
| 06607 | INGA DE FERRADURA |
| 06608 | INGA XIXI |
| 06701 | PEQUI |
| 06702 | PIQUI |
| 06703 | PEQUIA |
| 06704 | PIQUIA |
| 06705 | PIQUIA BRAVO |
| 06706 | PIQUIA AMARELO |
| 06707 | PIQUIA DA AREIA |
| 06708 | PIQUIA PEDRA |
| 06801 | PITANGA |
| 06802 | PITANGA COMUM |
| 06803 | PITANGATUBA |
| 06804 | PITANGA MIUDA |
| 06805 | IBIPITANGA |
| 06806 | PITANGA VERMELHA |
| 06807 | PITANGA DE JARDIM |
| 06808 | PITANGA DO CAMPO |
| 06901 | CIRIGUELA |
| 07001 | MURICI |
| 07002 | MURICI RASTEIRO |
| 07003 | MURICI DE TABULEIRO |
| 07004 | MURUCHI |
| 07005 | MURICI DA MATA |
| 07006 | MURICI DA PRAIA |
| 07007 | MURICI PITANGA |
| 07008 | MURICI DO CAMPO |
| 07009 | MURICI DO BREJO |
| 07010 | MURICI DE CAPOEIRA |
| 07011 | MURICI PENIMA |
| 07012 | MURICI MIUDO |
| 07013 | DOURADINHA FALSA |
| 07014 | FALSA DOURADINHA |
| 07101 | UMBU |
| 07102 | AMBU |
| 07103 | IMBU |
| 07201 | BERIBA |
| 07202 | BIRIBA |
| 07203 | JACA DE POBRE (BERIBA) |
| 07301 | CUPUACU |
| 07401 | NECTARINA |
| 07501 | MARI |
| 07502 | MARI AMARELO |
| 07503 | MARI ROXO |
| 07504 | UMARI AMARELO |
| 07505 | UMARI |
| 07506 | UMARI ROXO |
| 07601 | ROMA |
| 07701 | ACEROLA |
| 07801 | KIWI |
| 07901 | MANGOSTIM |
| 07902 | MANGOSTAO |
| 08001 | ABIU |
| 08101 | ABRICO |
| 08201 | JURUBEBA |
| 08301 | GRUMIXAMA DA INDIA |
| 08302 | GRUMIXAMA |
| 08401 | FRUTA (NAO-ESPECIFICADA) |
| 08402 | FRUTA NAO-ESPECIFICADA |
| 08501 | UVA ORGANICA |
| 08502 | UVA NIAGARA ORGANICA |
| 08503 | UVA MOSCATEL ORGANICA |
| 08504 | UVA PRETA ORGANICA |
| 08505 | UVA ROSA ORGANICA |
| 08506 | UVA NACIONAL ORGANICA |
| 08507 | UVA VERDE ORGANICA |
| 08508 | UVA ITALIA ORGANICA |
| 08509 | UVA ROXA ORGANICA |
| 08510 | UVA RUBI ORGANICA |
| 08511 | UVA BRANCA ORGANICA |
| 08512 | UVA THOMPSON ORGANICA |
| 08513 | UVA BENITAKA ORGANICA |
| 08514 | UVA RED GLOBE ORGANICA |
| 08601 | MACA ORGANICA |
| 08602 | MACA ACIDA ORGANICA |
| 08603 | MACA DOCE ORGANICA |
| 08604 | MACA BRANCA ORGANICA |
| 08605 | MACA VERDE ORGANICA |
| 08606 | MACA VERMELHA ORGANICA |
| 08607 | MACA ARGENTINA ORGANICA |
| 08608 | MACA NACIONAL ORGANICA |
| 08609 | MACA FUGI ORGANICA |
| 08610 | MACA GALA ORGANICA |
| 08611 | MACA IMPORTADA ORGANICA |
| 08701 | PESSEGO ORGANICO |
| 08801 | MORANGO ORGANICO |
| 08802 | MORANGUINHO ORGANICO |
| 08901 | ACEROLA ORGANICA |
| 09001 | UVA PASSA ORGANICA |
| 09101 | BANANA PRATA ORGANICA |
| 09201 | CAJU ORGANICO |
| 09301 | LIMAO NAO ESPECIFICADO |
| 09401 | CAMBUI |
| 09501 | LARANJA CHAMPAGNE |
| 09502 | LARANJA CHAMPANHA |
| 09601 | GOIABA ORGANICA |
| 09701 | LARANJA CHINA |
| 09702 | LARANJA DA CHINA |
| 09801 | BANANA NAO-ESPECIFICADA ORGANICA |
| 09901 | AMORA |
| 10001 | LICHIA |
| 10101 | LIMAO COMUM ORGANICO |
| 10201 | JAMELAO |
| 00101 | ACUCAR REFINADO |
| 00102 | ACUCAR TRIFILTRADO |
| 00201 | ACUCAR CRISTAL |
| 00202 | ACUCAR GRANULADO |
| 00203 | ACUCAR CRISTALIZADO |
| 00204 | ACUCAR GROSSO |
| 00301 | ACUCAR DEMERARA |
| 00302 | DEMERARA |
| 00303 | ACUCAR AMARELO PRETO |
| 00304 | ACUCAR MASCAVO |
| 00305 | ACUCAR PRETO |
| 00306 | ACUCAR MASCAVADO |
| 00401 | RAPADURA |
| 00402 | ALFENIM DE CANA |
| 00403 | BATIDA (RAPADURA) |
| 00404 | RAPADURA COM COCO |
| 00405 | RAPADURA COM LEITE |
| 00406 | RAPADURA COM AMENDOIM |
| 00407 | RAPADURA COM CASTANHA |
| 00408 | RAPADURA COM CAJU |
| 00501 | SORVETE DE QUALQUER SABOR INDUSTRIALIZADO |
| 00502 | PICOLE DE QUALQUER SABOR INDUSTRIALIZADO |
| 00503 | MASSA DE SORVETE |
| 00504 | SORVETE INDUSTRIALIZADO |
| 00505 | PICOLE INDUSTRIALIZADO |
| 00506 | CREMOSINHO (SORVETE) |
| 00601 | CHICLETE |
| 00602 | MENTEX |
| 00603 | BALA |
| 00604 | CARAMELO (BALA) |
| 00605 | DROPS |
| 00606 | PASTILHA |
| 00607 | PIRULITO |
| 00608 | CHICLE |
| 00609 | GOMA DE MASCAR |
| 00610 | JUJUBA |
| 00611 | BALAS |
| 00612 | BALAS SORTIDAS |
| 00613 | BALINHA |
| 00614 | BALA DE GOMA |
| 00701 | TABLETE DE CHOCOLATE |
| 00702 | BARRA DE CHOCOLATE |
| 00703 | CHOCOLATE EM TABLETE |
| 00704 | CHOCOLATE EM BARRA |
| 00705 | CHOCOLATE BISS (TABLETE) |
| 00706 | CONFETE |
| 00707 | CHOCOLATE BATOM |
| 00708 | BATON CHOCOLATE |
| 00709 | TUBETE DE CHOCOLATE |
| 00801 | CHOCOLATE EM PO DE QUALQUER MARCA |
| 00802 | CHOCOLATE EM PO QUICK |
| 00803 | CHOCOLATE NESCAU |
| 00804 | CHOCOLATE EM PO MUQUI |
| 00805 | CHOCOLATE TODDY |
| 00806 | CHOCOLATE EM PO TODDY |
| 00807 | CHOCOLATE EM PO NESCAU |
| 00808 | TODDY INSTANTANEO |
| 00809 | QUICK |
| 00810 | TODDY VITAMINADO |
| 00811 | TODDY |
| 00812 | NESCAU |
| 00813 | NESCAU VITAMINADO |
| 00814 | MUQUI |
| 00815 | NESCAU INSTANTANEO |
| 00816 | QUICK SHAKE CRISTALINO |
| 00817 | QUICK SHAKE |
| 00818 | OVOMALTINE |
| 00819 | PO ACHOCOLATADO |
| 00820 | PO PARA MILK SHAKE |
| 00821 | ACHOCOLATADO EM PO |
| 00822 | TODDYNHO EM PO |
| 00823 | NESQUIK |
| 00901 | BOMBOM DE QUALQUER MARCA |
| 00902 | BOMBOM CARAMELIZADO DE QUALQUER MARCA |
| 00903 | BOMBONS SORTIDOS DE QUALQUER MARCA |
| 00904 | TRUFA |
| 00905 | BOBOM CASEIRO |
| 01001 | GELEIA DE FRUTAS DE QUALQUER MARCA OU SABOR |
| 01002 | MOUSSE DE QUALQUER MARCA (GELEIA) |
| 01003 | MOUSSE DE QUALQUER SABOR (GELEIA) |
| 01004 | GELEIA DE DAMASCO |
| 01101 | GELEIA DE MOCOTO |
| 01102 | GELEIA DE MOCOTO VITAMINADA |
| 01201 | DOCE DE FRUTAS EM PASTA DE QUALQUER SABOR |
| 01202 | PURE DE FRUTAS |
| 01203 | PASTA DE AMENDOIM |
| 01204 | PESSEGADA |
| 01205 | CREME DE AMENDOIM |
| 01206 | MARMELADA |
| 01207 | DOCE DE FRUTAS EM BARRA OU TIJOLO DE QUALQUER SABOR |
| 01208 | CREME DE GOIABA |
| 01209 | FIGADA |
| 01210 | BANANADA (EM BARRA OU TIJOLO) |
| 01211 | GOIABADA |
| 01212 | DOCE DE ABOBORA EM PASTA |
| 01213 | DOCE DE MORANGO EM PASTA |
| 01214 | CAJU EM PASTA |
| 01215 | DOCE DE CEREJA EM PASTA |
| 01216 | DOCE DE BATATA MARROM GLACE |
| 01217 | DOCE DE AMEIXA EM PASTA |
| 01218 | DOCE DE PERA EM PASTA |
| 01219 | DOCE DE CACAU EM PASTA |
| 01220 | PASTA DE CACAU |
| 01221 | CACAU EM PASTA |
| 01222 | DOCE DE FRUTAS EM TABLETE DE QUALQUER SABOR |
| 01223 | DOCE DE CAJU EM PASTA OU BARRA |
| 01224 | MARIOLA |
| 01225 | DOCE DE FEIJAO EM PASTA |
| 01226 | YOKAN (DOCE DE FEIJAO EM PASTA) |
| 01227 | DOCE DE MANDIOCA EM PASTA |
| 01228 | DOCE DE MAMAO EM PASTA |
| 01229 | DOCE DE MINGAU DE MILHO VERDE EM PASTA |
| 01230 | DOCE DE MILHO VERDE EM PASTA |
| 01231 | DOCE DE BANANA EM PASTA OU BARRA |
| 01232 | DOCE DE COCO EM PASTA OU BARRA |
| 01233 | DOCE DE BURITI EM PASTA OU BARRA |
| 01234 | DOCE DE CAJU EM BARRA |
| 01235 | DOCE DE GOIABA EM PASTA OU BARRA |
| 01236 | DOCE DE UVA EM PASTA OU BARRA |
| 01237 | DOCE DE ABOBORA COM COCO |
| 01238 | DOCE DE TAMARINDO (EM PASTA) |
| 01239 | PATE DE FRUTAS |
| 01240 | BANANADA |
| 01301 | DOCE DE FRUTAS EM CALDA DE QUALQUER SABOR |
| 01302 | FIGO EM CALDA |
| 01303 | AMEIXA EM CALDA |
| 01304 | PESSEGO EM CALDA |
| 01305 | SALADA DE FRUTAS EM CALDA |
| 01306 | ABACAXI EM CALDA |
| 01307 | BANANA EM CALDA |
| 01308 | GOIABA EM CALDA |
| 01309 | LARANJA EM CALDA |
| 01310 | CEREJA EM CALDA |
| 01311 | MORANGO EM CALDA |
| 01312 | DOCE DE JACA EM CALDA |
| 01313 | DOCE DE PESSEGO EM CALDA |
| 01401 | DOCE DE FRUTAS CRISTALIZADO DE QUALQUER SABOR |
| 01402 | DOCE DE ABACAXI CRISTALIZADO |
| 01403 | DOCE DE LARANJA CRISTALIZADO |
| 01404 | DOCE DE FIGO CRISTALIZADO |
| 01405 | DOCE DE GOIABA CRISTALIZADO |
| 01406 | DOCE DE CAJU CRISTALIZADO |
| 01407 | DOCE DE BANANA CRISTALIZADO |
| 01408 | FRUTAS CRISTALIZADAS (DOCE) |
| 01501 | MELADO |
| 01502 | MELADO DE CANA |
| 01601 | MEL DE ABELHA |
| 01602 | MEL |
| 01603 | MEL COM ALHO |
| 01604 | MEL COM PROPOLIS |
| 01605 | MEL COMPOSTO |
| 01606 | MEL DE ENGENHO |
| 01607 | MEL DE ROMA |
| 01608 | MEL COM AGRIAO |
| 01701 | GELATINA |
| 01702 | GELATINA EM PO |
| 01703 | GELATINA EM FOLHA |
| 01801 | ADOCANTE ARTIFICIAL |
| 01802 | SUITA |
| 01803 | SUCARIL |
| 01804 | SACARINA |
| 01805 | ZERO CAL |
| 01901 | GLICOSE DE MILHO |
| 01902 | GLUCOSE DE MILHO |
| 01903 | KARO |
| 02001 | CREME DE ACUCAR |
| 02002 | GLACUCAR |
| 02003 | GLACE |
| 02004 | MERENGUE |
| 02101 | CORANTE DE CAFE |
| 02102 | MISTURA DE CAFE |
| 02201 | CONFEITOS DE BOLOS E DOCES |
| 02202 | CORANTES DE BOLOS E DOCES |
| 02203 | CONFEITO DE CHOCOLATE |
| 02204 | CONFEITO DE AMENDOIM |
| 02205 | CONFEITO NAO-ESPECIFICADO |
| 02206 | CONFEITO DE MENTA |
| 02207 | CONFEITO DE LEITE |
| 02208 | CORANTE DE BOLO E DOCE |
| 02209 | MARSHMALLOW |
| 02301 | SCHIMIER DE CANA |
| 02302 | SCHIMIER DE COLONIA |
| 02401 | FRUTA SECA OU DESIDRATADA |
| 02402 | PASSA |
| 02403 | AMEIXA SECA OU DESIDRATADA |
| 02404 | TAMARA SECA OU DESIDRATADA |
| 02405 | MACA SECA OU DESIDRATADA |
| 02406 | DAMASCO SECO OU DESIDRATADO |
| 02407 | BANANA SECA OU DESIDRATADA |
| 02408 | PESSEGO SECO OU DESIDRATADO |
| 02409 | FIGO SECO OU DESIDRATADO |
| 02410 | ABACAXI SECO OU DESIDRATADO |
| 02411 | FRUTA DESIDRATADA |
| 02412 | AMEIXA DESIDRATADA |
| 02413 | TAMARA DESIDRATADA |
| 02414 | MACA DESIDRATADA |
| 02415 | DAMASCO DESIDRATADO |
| 02416 | BANANA DESIDRATADA |
| 02417 | PESSEGO DESIDRATADO |
| 02418 | FIGO DESIDRATADO |
| 02419 | ABACAXI DESIDRATADO |
| 02420 | BANANA PASSA |
| 02421 | CAJU DESIDRATADO |
| 02501 | SOBREMESA INFANTIL DE QUALQUER MARCA E SABOR |
| 02502 | DOCE INFANTIL EM POTE DE QUALQUER MARCA E SABOR |
| 02601 | PUDIM DE QUALQUER SABOR |
| 02602 | PUDIM DE COCO |
| 02603 | PUDIM DE QUEIJO |
| 02604 | PUDIM DE LEITE CONDENSADO |
| 02605 | PUDIM DE FUBA |
| 02606 | PUDIM DE LARANJA |
| 02607 | DANETTE PUDIM |
| 02608 | PUDIM DANETTE |
| 02701 | MANJAR |
| 02801 | MISTURA INDUSTRIAL DE DOCE, SORVETE E PUDIM |
| 02802 | FLAN EM PO |
| 02803 | PO DE DOCE, SORVETE E PUDIM |
| 02804 | PO DE FLAN |
| 02805 | FLAMBY EM PO |
| 02806 | PO DE MINGAU DE MILHO VERDE |
| 02807 | MASSA PARA MINGAU NAO-ESPECIFICADA |
| 02808 | MASSA DOCE NAO-ESPECIFICADA |
| 02809 | PO PARA MINGAU NAO-ESPECIFICADO |
| 02810 | MOUSSE EM PO |
| 02811 | PO PARA SORVETE |
| 02812 | BASE PARA SORVETE |
| 02813 | PO PARA PUDIM |
| 02901 | CUSCUZ |
| 02902 | CUSCUZ DE TAPIOCA |
| 02903 | CUSCUZ DE CARIMA |
| 02904 | CUSCUZ DE MANDIOCA |
| 02905 | CUSCUZ DE GOMA |
| 02906 | CUSCUZ DE ARROZ |
| 02907 | CUSCUZ DE FUBA |
| 02908 | CUSCUZ DE MILHO |
| 02909 | MASSA DE CUSCUZ |
| 03001 | MARIA MOLE |
| 03002 | BABA DE SAPO OU DE MOCA |
| 03101 | COCADA |
| 03102 | QUEBRA QUEIXO |
| 03201 | DOCE DE AMENDOIM |
| 03202 | PE DE MOLEQUE |
| 03203 | PACOCA |
| 03204 | TORRAO DE AMENDOIM |
| 03205 | PACOQUINHA DE AMENDOIM |
| 03206 | PACOCA DE CASTANHA DE CAJU |
| 03207 | AMENDOIM CARAMELIZADO |
| 03208 | AMENDOIM AMANTEIGADO |
| 03209 | AMENDOIM ACHOCOLATADO |
| 03301 | AMENDOIM SALGADO |
| 03302 | AMENDOIM TORRADO |
| 03303 | AMENDOIM APIMENTADO |
| 03304 | AMENDOIM COZIDO |
| 03305 | AMENDOIM DOCE |
| 03306 | AMENDOIM JAPONES |
| 03401 | DOCE DE ABOBORA |
| 03501 | DOCE DE BATATA DOCE |
| 03601 | BRIGADEIRO |
| 03602 | DOCE DE CHOCOLATE |
| 03603 | BOLINHO DE BRIGADEIRO |
| 03701 | BOMBA DE QUALQUER TIPO |
| 03801 | MIL FOLHAS |
| 03901 | QUEIJADINHA |
| 03902 | QUEIJADA |
| 04001 | DOCE DE QUEIJO |
| 04101 | DOCE A BASE DE OVOS |
| 04102 | DOCE DE OVOS COM AMENDOA |
| 04103 | SCHIMIER DE OVOS |
| 04104 | CACAROLA ITALIANA |
| 04105 | QUINDIM |
| 04106 | DOCE DE OVOS |
| 04107 | PAPO DE ANJO |
| 04108 | FIOS DE OVOS |
| 04201 | DOCE A BASE DE LEITE |
| 04202 | PAVE |
| 04203 | AMBROSIA |
| 04204 | LEITE GELEIFICADO |
| 04205 | CHANDELE DE QUALQUER SABOR |
| 04301 | DOCE DE LEITE |
| 04302 | MUMU |
| 04303 | DOCE DE LEITE COM AMEIXA |
| 04304 | DOCE DE LEITE COM AMENDOIM |
| 04305 | DOCE DE LEITE COM COCO |
| 04306 | GELEIA DE LEITE |
| 04401 | CANUDINHO RECHEADO |
| 04402 | CANUDINHO PARA RECHEAR |
| 04501 | SAROLHO |
| 04502 | BEIJU |
| 04503 | BEIJU DE TAPIOCA |
| 04504 | MASSA DE BEIJU |
| 04505 | BEIJU DE QUEIJO E PRESUNTO |
| 04506 | TAPIOCA (BEIJU) |
| 04507 | TAPIOCA COM COCO |
| 04508 | TAPIOCA DE QUEIJO |
| 04509 | BEIJU DE COCO |
| 04510 | MAL- CASADO |
| 04601 | SCHIMIER DE FRUTA (EXCETO DE CANA) |
| 04701 | BEIJO DE MOCA |
| 04702 | BEIJO AFRICANO |
| 04801 | PICOLE ENSACADO |
| 04802 | DINDIM (PICOLE ENSACADO) |
| 04803 | SACOLE (PICOLE ENSACADO) |
| 04804 | CHUP-CHUP (PICOLE ENSACADO) |
| 04805 | PICOLE CASEIRO |
| 04806 | SORVETE CASEIRO |
| 04807 | GELADINHO |
| 04901 | DOCE CONFEITADO DE QUALQUER TIPO |
| 05001 | CHURRO |
| 05101 | PAMONHA |
| 05201 | CHOCOLATE GRANULADO |
| 05301 | ADOCANTE NATURAL CONCENTRADO |
| 05302 | ADOCANTE CONCENTRADO NATURAL |
| 05401 | ALGODAO-DOCE |
| 05501 | COBERTURA DE QUALQUER SABOR (DE SORVETE, ETC.) |
| 05601 | CHOCOLATE EM CREME |
| 05602 | NUCITA (CHOCOLATE EM CREME) |
| 05603 | IOIO CREME (CHOCOLATE EM CREME) |
| 05604 | CREME DE BRIGADEIRO |
| 05605 | CREME DE CHOCOLATE |
| 05701 | GUARANA EM PO NATURAL |
| 05702 | GUARANA NATURAL EM PO |
| 05801 | OVO DE PASCOA |
| 05802 | COELHINHO DA PASCOA DE CHOCOLATE |
| 05803 | CHOCOLATE COELHINHO DA PASCOA |
| 05804 | KINDER OVO |
| 05901 | TORRONE |
| 05902 | DOCE TORRONE |
| 05903 | MANDOLATE |
| 06001 | ARROZ-DOCE |
| 06101 | PROPOLIS |
| 06201 | GELEIA REAL |
| 06301 | DOCE SIRIO |
| 06401 | POLPA DE FRUTA CONGELADA |
| 06402 | MARACUJA EM POLPA |
| 06403 | POLPA DE MARACUJA |
| 06404 | POLPA DE COCO |
| 06405 | MELAO EM POLPA |
| 06406 | POLPA DE ABACAXI |
| 06407 | POLPA DE CAJU |
| 06408 | POLPA DE CUPUACU |
| 06409 | POLPA DE GRAVIOLA |
| 06410 | POLPA DE JENIPAPO |
| 06411 | POLPA DE TANGERINA |
| 06412 | POLPA DE MANGA |
| 06413 | POLPA DE CAJA |
| 06414 | POLPA DE GOIABA |
| 06415 | POLPA DE PITANGA |
| 06416 | POLPA DE MORANGO |
| 06417 | POLPA DE UVA |
| 06418 | POLPA DE ACEROLA |
| 06419 | POLPA DE FRUTA DE MANGABA |
| 06420 | POLPA DE TAMARINDO |
| 06421 | POLPA DE BURITI |
| 06501 | DIET SHAKE |
| 06502 | CONCENTRADO ALIMENTAR DIET SHAKE |
| 06601 | ACUCAR INDETERMINADO |
| 06602 | ACUCAR |
| 06603 | ACUCAR COMUM |
| 06701 | ACUCAR DE BAUNILHA |
| 06801 | ACUCAR TRITURADO |
| 06802 | ACUCAR MOIDO |
| 06901 | ADOCANTE INDETERMINADO |
| 06902 | ADOCANTE |
| 06903 | ADOCANTE EM PO |
| 06904 | ADOCANTE LIQUIDO |
| 07001 | ACUCAR LIGHT |
| 07002 | ACUCAR REFINADO LIGHT |
| 07003 | ACUCAR TRIFILTRADO LIGHT |
| 07101 | ADOCANTE DIET |
| 07102 | ADOCANTE DIETETICO |
| 07103 | ADOCANTE EM PO DIET |
| 07104 | ADOCANTE LIQUIDO DIET |
| 07105 | ADOCANTE EM PO DIETETICO |
| 07106 | ADOCANTE LIQUIDO DIETETICO |
| 07201 | BARRA DE CEREAIS |
| 07202 | BARRA DE CEREAIS SALGADA |
| 07203 | BARRA DE CEREAIS DOCE |
| 07204 | BARRA DE CEREAL |
| 07301 | SOBREMESA DE QUALQUER TIPO (EXCETO INFANTIL) |
| 07401 | RABANADA |
| 07501 | MILK SHAKE |
| 07502 | MILK SHAKE DE MORANGO |
| 07601 | FRUTOSE |
| 07701 | DOCE NAO-ESPECIFICADO |
| 07702 | DOCE |
| 07801 | RECHEIO PARA BOLO |
| 07901 | GELEIA DE FRUTAS DE QUALQUER MARCA OU SABOR DIET |
| 07902 | MOUSSE DE QUALQUER MARCA (GELEIA) DIET |
| 07903 | MOUSSE DE QUALQUER SABOR (GELEIA) DIET |
| 07904 | GELEIA DIET |
| 07905 | GELEIA DE FRUTAS DIET |
| 07906 | GELEIA DE FRUTAS DE QUALQUER MARCA OU SABOR DIETETICA |
| 07907 | MOUSSE DE QUALQUER MARCA (GELEIA) DIETETICA |
| 07908 | MOUSSE DE QUALQUER SABOR (GELEIA) DIETETICA |
| 07909 | GELEIA DIETETICA |
| 07910 | GELEIA DE FRUTAS DIETETICA |
| 08001 | DOCE DE FRUTAS EM BARRA OU PASTA DIET |
| 08002 | DOCE DE FRUTAS EM BARRA OU PASTA DIETETICO |
| 08003 | DOCE DE FRUTAS EM PASTA DIETETICO |
| 08004 | DOCE DE FRUTAS EM BARRA DIETETICO |
| 08005 | DOCE DE FRUTAS EM PASTA DIET |
| 08006 | DOCE DE FRUTAS EM BARRA DIET |
| 08101 | GELATINA DIET |
| 08102 | GELATINA DIETETICA |
| 08103 | GELATINA DE DIETA |
| 08201 | NIDEX |
| 08301 | DEXTROSOL |
| 08401 | PASTEIS DE SANTA CLARA |
| 08501 | SUSPIRO |
| 08601 | ACUCAR REFINADO ORGANICO |
| 08602 | ACUCAR TRIFILTRADO ORGANICO |
| 08701 | ACUCAR CRISTAL ORGANICO |
| 08702 | ACUCAR GRANULADO ORGANICO |
| 08703 | ACUCAR CRISTALIZADO ORGANICO |
| 08801 | ACUCAR DEMERARA ORGANICO |
| 08802 | DEMERARA ORGANICO |
| 08803 | ACUCAR AMARELO PRETO ORGANICO |
| 08804 | ACUCAR MASCAVO ORGANICO |
| 08805 | ACUCAR PRETO ORGANICO |
| 08806 | ACUCAR MASCAVADO ORGANICO |
| 08901 | ACUCAR INDETERMINADO ORGANICO |
| 08902 | ACUCAR ORGANICO |
| 08903 | ACUCAR COMUM ORGANICO |
| 09001 | MEL DE ABELHA ORGANICO |
| 09002 | MEL ORGANICO |
| 09003 | MEL ORGANICO COM ALHO |
| 09004 | MEL ORGANICO COM PROPOLIS |
| 09005 | MEL ORGANICO COMPOSTO |
| 09006 | MEL ORGANICO DE ENGENHO |
| 09007 | MEL ORGANICO DE ROMA |
| 09101 | ACUCAR ORGANICO LIGHT |
| 09201 | DOCE DE FRUTAS EM CALDA DIET |
| 09202 | DOCE DE FRUTAS EM CALDA DIETETICO |
| 09203 | DOCE DE PESSEGO EM CALDA DIET |
| 09204 | DOCE DE PESSEGO EM CALDA DIETETICO |
| 09205 | FIGO EM CALDA DIET |
| 09206 | FIGO EM CALDA DIETETICO |
| 09207 | AMEIXA EM CALDA DIET |
| 09208 | AMEIXA EM CALDA DIETETICA |
| 09209 | ABACAXI EM CALDA DIET |
| 09210 | ABACAXI EM CALDA DIETETICO |
| 09211 | BANANA EM CALDA DIET |
| 09212 | BANANA EM CALDA DIETETICA |
| 09213 | GOIABA EM CALDA DIET |
| 09214 | GOIABA EM CALDA DIETETICA |
| 09215 | LARANJA EM CALDA DIET |
| 09216 | LARANJA EM CALDA DIETETICA |
| 09217 | CEREJA EM CALDA DIET |
| 09218 | CEREJA EM CALDA DIETETICA |
| 09219 | MORANGO EM CALDA DIET |
| 09220 | MORANGO EM CALDA DIETETICO |
| 09301 | DOCE DE FRUTAS CRISTALIZADO DE QUALQUER SABOR DIET |
| 09302 | DOCE DE FRUTAS CRISTALIZADO DE QUALQUER SABOR DIETETICO |
| 09303 | DOCE DE FRUTAS CRISTALIZADO DIET |
| 09304 | DOCE DE FRUTAS CRISTALIZADO DIETETICO |
| 09305 | DOCE DE GOIABA CRISTALIZADO DIET |
| 09306 | DOCE DE GOIABA CRISTALIZADO DIETETICO |
| 09307 | DOCE DE CAJU CRISTALIZADO DIET |
| 09308 | DOCE DE CAJU CRISTALIZADO DIETETICO |
| 09309 | DOCE DE BANANA CRISTALIZADO DIET |
| 09310 | DOCE DE BANANA CRISTALIZADO DIETETICO |
| 09401 | DOCE NAO-ESPECIFICADO LIGHT |
| 09402 | DOCE LIGHT NAO-ESPECIFICADO |
| 09501 | DOCE NAO-ESPECIFICADO DIET |
| 09502 | DOCE DIET NAO-ESPECIFICADO |
| 09503 | DOCE NAO-ESPECIFICADO DIETETICO |
| 09504 | DOCE DIETETICO NAO-ESPECIFICADO |
| 09601 | SORVETE DE QUALQUER SABOR INDUSTRIALIZADO LIGHT |
| 09602 | PICOLE DE QUALQUER SABOR INDUSTRIALIZADO LIGHT |
| 09603 | MASSA DE SORVETE LIGHT |
| 09604 | SORVETE INDUSTRIALIZADO LIGHT |
| 09605 | PICOLE INDUSTRIALIZADO LIGHT |
| 09701 | SORVETE DE QUALQUER SABOR INDUSTRIALIZADO DIET |
| 09702 | PICOLE DE QUALQUER SABOR INDUSTRIALIZADO DIET |
| 09703 | MASSA DE SORVETE DIET |
| 09704 | SORVETE INDUSTRIALIZADO DIET |
| 09705 | PICOLE INDUSTRIALIZADO DIET |
| 09706 | SORVETE DE QUALQUER SABOR INDUSTRIALIZADO DIETETICO |
| 09707 | PICOLE DE QUALQUER SABOR INDUSTRIALIZADO DIETETICO |
| 09708 | MASSA DE SORVETE DIETETICA |
| 09709 | SORVETE INDUSTRIALIZADO DIETETICO |
| 09710 | PICOLE INDUSTRIALIZADO DIETETICO |
| 09801 | CHICLETE LIGHT |
| 09802 | MENTEX LIGHT |
| 09803 | BALA LIGHT |
| 09804 | CARAMELO (BALA) LIGHT |
| 09805 | DROPS LIGHT |
| 09806 | PASTILHA LIGHT |
| 09807 | PIRULITO LIGHT |
| 09808 | CHICLE LIGHT |
| 09809 | GOMA DE MASCAR LIGHT |
| 09810 | JUJUBA LIGHT |
| 09811 | BALAS LIGHT |
| 09812 | BALAS SORTIDAS LIGHT |
| 09813 | BALINHA LIGHT |
| 09901 | CHICLETE DIET |
| 09902 | MENTEX DIET |
| 09903 | BALA DIET |
| 09904 | CARAMELO (BALA) DIET |
| 09905 | DROPS DIET |
| 09906 | PASTILHA DIET |
| 09907 | PIRULITO DIET |
| 09908 | CHICLE DIET |
| 09909 | GOMA DE MASCAR DIET |
| 09910 | JUJUBA DIET |
| 09911 | BALAS DIET |
| 09912 | BALAS SORTIDAS DIET |
| 09913 | BALINHA DIET |
| 09914 | CHICLETE DIETETICO |
| 09915 | MENTEX DIETETICO |
| 09916 | BALA DIETETICA |
| 09917 | CARAMELO (BALA) DIETETICO |
| 09918 | DROPS DIETETICO |
| 09919 | PASTILHA DIETETICA |
| 09920 | PIRULITO DIETETICO |
| 09921 | CHICLE DIETETICO |
| 09922 | GOMA DE MASCAR DIETETICA |
| 09923 | JUJUBA DIETETICA |
| 09924 | BALAS DIETETICAS |
| 09925 | BALAS SORTIDAS DIETETICAS |
| 09926 | BALINHA DIETETICA |
| 10001 | TABLETE DE CHOCOLATE LIGHT |
| 10002 | BARRA DE CHOCOLATE LIGHT |
| 10003 | CHOCOLATE EM TABLETE LIGHT |
| 10004 | CHOCOLATE EM BARRA LIGHT |
| 10005 | CHOCOLATE BISS (TABLETE) LIGHT |
| 10006 | CONFETE LIGHT |
| 10101 | TABLETE DE CHOCOLATE DIET |
| 10102 | BARRA DE CHOCOLATE DIET |
| 10103 | CHOCOLATE EM TABLETE DIET |
| 10104 | CHOCOLATE EM BARRA DIET |
| 10105 | CHOCOLATE BISS (TABLETE) DIET |
| 10106 | CONFETE DIET |
| 10107 | TABLETE DE CHOCOLATE DIETETICO |
| 10108 | BARRA DE CHOCOLATE DIETETICO |
| 10109 | CHOCOLATE EM TABLETE DIETETICO |
| 10110 | CHOCOLATE EM BARRA DIETETICO |
| 10111 | CHOCOLATE BISS (TABLETE) DIETETICO |
| 10112 | CONFETE DIETETICO |
| 10201 | CHOCOLATE EM PO DE QUALQUER MARCA LIGHT |
| 10202 | CHOCOLATE EM PO QUICK LIGHT |
| 10203 | CHOCOLATE NESCAU LIGHT |
| 10204 | CHOCOLATE EM PO MUQUI LIGHT |
| 10205 | CHOCOLATE TODDY LIGHT |
| 10206 | CHOCOLATE EM PO TODDY LIGHT |
| 10207 | CHOCOLATE EM PO NESCAU LIGHT |
| 10208 | TODDY INSTANTANEO LIGHT |
| 10209 | QUICK LIGHT |
| 10210 | TODDY VITAMINADO LIGHT |
| 10211 | TODDY LIGHT |
| 10212 | NESCAU LIGHT |
| 10213 | NESCAU VITAMINADO LIGHT |
| 10214 | MUQUI LIGHT |
| 10215 | NESCAU INSTANTANEO LIGHT |
| 10216 | QUICK SHAKE CRISTALINO LIGHT |
| 10217 | QUICK SHAKE LIGHT |
| 10218 | OVOMALTINE LIGHT |
| 10219 | PO ACHOCOLATADO LIGHT |
| 10220 | PO PARA MILK SHAKE LIGHT |
| 10221 | ACHOCOLATADO EM PO LIGHT |
| 10222 | TODDYNHO EM PO LIGHT |
| 10301 | CHOCOLATE EM PO DE QUALQUER MARCA DIET |
| 10302 | CHOCOLATE EM PO QUICK DIET |
| 10303 | CHOCOLATE NESCAU DIET |
| 10304 | CHOCOLATE EM PO MUQUI DIET |
| 10305 | CHOCOLATE TODDY DIET |
| 10306 | CHOCOLATE EM PO TODDY DIET |
| 10307 | CHOCOLATE EM PO NESCAU DIET |
| 10308 | TODDY INSTANTANEO DIET |
| 10309 | QUICK DIET |
| 10310 | TODDY VITAMINADO DIET |
| 10311 | TODDY DIET |
| 10312 | NESCAU DIET |
| 10313 | NESCAU VITAMINADO DIET |
| 10314 | MUQUI DIET |
| 10315 | NESCAU INSTANTANEO DIET |
| 10316 | QUICK SHAKE CRISTALINO DIET |
| 10317 | QUICK SHAKE DIET |
| 10318 | OVOMALTINE DIET |
| 10319 | PO ACHOCOLATADO DIET |
| 10320 | PO PARA MILK SHAKE DIET |
| 10321 | ACHOCOLATADO EM PO DIET |
| 10322 | TODDYNHO EM PO DIET |
| 10323 | CHOCOLATE EM PO DE QUALQUER MARCA DIETETICO |
| 10324 | CHOCOLATE EM PO QUICK DIETETICO |
| 10325 | CHOCOLATE NESCAU DIETETICO |
| 10326 | CHOCOLATE EM PO MUQUI DIETETICO |
| 10327 | CHOCOLATE TODDY DIETETICO |
| 10328 | CHOCOLATE EM PO TODDY DIETETICO |
| 10329 | CHOCOLATE EM PO NESCAU DIETETICO |
| 10330 | TODDY INSTANTANEO DIETETICO |
| 10331 | QUICK DIETETICO |
| 10332 | TODDY VITAMINADO DIETETICO |
| 10333 | TODDY DIETETICO |
| 10334 | NESCAU DIETETICO |
| 10335 | NESCAU VITAMINADO DIETETICO |
| 10336 | MUQUI DIETETICO |
| 10337 | NESCAU INSTANTANEO DIETETICO |
| 10338 | QUICK SHAKE CRISTALINO DIETETICO |
| 10339 | QUICK SHAKE DIETETICO |
| 10340 | OVOMALTINE DIETETICO |
| 10341 | PO ACHOCOLATADO DIETETICO |
| 10342 | PO PARA MILK SHAKE DIETETICO |
| 10343 | ACHOCOLATADO EM PO DIETETICO |
| 10344 | TODDYNHO EM PO DIETETICO |
| 10401 | BOMBOM DE QUALQUER MARCA LIGHT |
| 10402 | BOMBOM CARAMELIZADO DE QUALQUER MARCA LIGHT |
| 10403 | BOMBONS SORTIDOS DE QUALQUER MARCA LIGHT |
| 10404 | TRUFA LIGHT |
| 10405 | BOMBOM LIGHT |
| 10501 | BOMBOM DE QUALQUER MARCA DIET |
| 10502 | BOMBOM CARAMELIZADO DE QUALQUER MARCA DIET |
| 10503 | BOMBONS SORTIDOS DE QUALQUER MARCA DIET |
| 10504 | TRUFA DIET |
| 10505 | BOMBOM DE QUALQUER MARCA DIETETICO |
| 10506 | BOMBOM CARAMELIZADO DE QUALQUER MARCA DIETETICO |
| 10507 | BOMBONS SORTIDOS DE QUALQUER MARCA DIETETICO |
| 10508 | TRUFA DIETETICA |
| 10601 | GELEIA DE FRUTAS DE QUALQUER MARCA OU SABOR LIGHT |
| 10602 | MOUSSE DE QUALQUER MARCA (GELEIA) LIGHT |
| 10603 | MOUSSE DE QUALQUER SABOR (GELEIA) LIGHT |
| 10604 | GELEIA LIGHT |
| 10605 | GELEIA DE FRUTAS LIGHT |
| 10701 | DOCE DE FRUTAS EM BARRA OU PASTA LIGHT |
| 10702 | DOCE DE FRUTAS EM PASTA LIGHT |
| 10703 | DOCE DE FRUTAS EM BARRA LIGHT |
| 10801 | PUDIM DE QUALQUER SABOR LIGHT |
| 10802 | PUDIM DE COCO LIGHT |
| 10803 | PUDIM DE QUEIJO LIGHT |
| 10804 | PUDIM DE LEITE CONDENSADO LIGHT |
| 10805 | PUDIM DE FUBA LIGHT |
| 10806 | PUDIM DE LARANJA LIGHT |
| 10807 | DANETTE PUDIM LIGHT |
| 10808 | PUDIM DANETTE LIGHT |
| 10901 | PUDIM DE QUALQUER SABOR DIET |
| 10902 | PUDIM DE COCO DIET |
| 10903 | PUDIM DE QUEIJO DIET |
| 10904 | PUDIM DE LEITE CONDENSADO DIET |
| 10905 | PUDIM DE FUBA DIET |
| 10906 | PUDIM DE LARANJA DIET |
| 10907 | DANETTE PUDIM DIET |
| 10908 | PUDIM DANETTE DIET |
| 10909 | PUDIM DE QUALQUER SABOR DIETETICO |
| 10910 | PUDIM DE COCO DIETETICO |
| 10911 | PUDIM DE QUEIJO DIETETICO |
| 10912 | PUDIM DE LEITE CONDENSADO DIETETICO |
| 10913 | PUDIM DE FUBA DIETETICO |
| 10914 | PUDIM DE LARANJA DIETETICO |
| 10915 | DANETTE PUDIM DIETETICO |
| 10916 | PUDIM DANETTE DIETETICO |
| 11001 | MANJAR LIGHT |
| 11101 | MANJAR DIET |
| 11102 | MANJAR DIETETICO |
| 11201 | DOCE DE LEITE LIGHT |
| 11202 | MUMU LIGHT |
| 11301 | DOCE DE LEITE DIET |
| 11302 | MUMU DIET |
| 11303 | DOCE DE LEITE DIETETICO |
| 11304 | MUMU DIETETICO |
| 11401 | PICOLE ENSACADO LIGHT |
| 11402 | DINDIM (PICOLE ENSACADO) LIGHT |
| 11403 | SACOLE (PICOLE ENSACADO) LIGHT |
| 11404 | CHUP-CHUP (PICOLE ENSACADO) LIGHT |
| 11405 | PICOLE CASEIRO LIGHT |
| 11406 | SORVETE CASEIRO LIGHT |
| 11407 | GELADINHO LIGHT |
| 11501 | DOCE CONFEITADO DE QUALQUER TIPO LIGHT |
| 11601 | DOCE CONFEITADO DE QUALQUER TIPO DIET |
| 11602 | DOCE CONFEITADO DE QUALQUER TIPO DIETETICO |
| 11701 | CHOCOLATE GRANULADO LIGHT |
| 11801 | CHOCOLATE GRANULADO DIET |
| 11802 | CHOCOLATE GRANULADO DIETETICO |
| 11901 | COBERTURA DE QUALQUER SABOR (DE SORVETE, ETC.) LIGHT |
| 12001 | COBERTURA DE QUALQUER SABOR (DE SORVETE, ETC.) DIET |
| 12002 | COBERTURA DE QUALQUER SABOR (DE SORVETE, ETC.) DIETETICO |
| 12101 | CHOCOLATE EM CREME LIGHT |
| 12102 | NUCITA (CHOCOLATE EM CREME) LIGHT |
| 12103 | IOIO CREME (CHOCOLATE EM CREME) LIGHT |
| 12201 | CHOCOLATE EM CREME DIET |
| 12202 | NUCITA (CHOCOLATE EM CREME) DIET |
| 12203 | IOIO CREME (CHOCOLATE EM CREME) DIET |
| 12204 | CHOCOLATE EM CREME DIETETICO |
| 12205 | NUCITA (CHOCOLATE EM CREME) DIETETICO |
| 12206 | IOIO CREME (CHOCOLATE EM CREME) DIETETICO |
| 12301 | OVO DE PASCOA LIGHT |
| 12302 | COELHINHO DA PASCOA DE CHOCOLATE LIGHT |
| 12303 | CHOCOLATE COELHINHO DA PASCOA LIGHT |
| 12304 | KINDER OVO LIGHT |
| 12401 | OVO DE PASCOA DIET |
| 12402 | COELHINHO DA PASCOA DE CHOCOLATE DIET |
| 12403 | CHOCOLATE COELHINHO DA PASCOA DIET |
| 12404 | KINDER OVO DIET |
| 12405 | OVO DE PASCOA DIETETICO |
| 12406 | COELHINHO DA PASCOA DE CHOCOLATE DIETETICO |
| 12407 | CHOCOLATE COELHINHO DA PASCOA DIETETICO |
| 12408 | KINDER OVO DIETETICO |
| 12501 | BARRA DE CEREAIS DIET |
| 12502 | BARRA DE CEREAIS DOCE DIET |
| 12503 | BARRA DE CEREAIS DIETETICA |
| 12504 | BARRA DE CEREAIS DOCE DIETETICA |
| 12601 | SOBREMESA DE QUALQUER TIPO (EXCETO INFANTIL) LIGHT |
| 12701 | SOBREMESA DE QUALQUER TIPO (EXCETO INFANTIL) DIET |
| 12702 | SOBREMESA DE QUALQUER TIPO (EXCETO INFANTIL) DIETETICA |
| 12801 | MILK SHAKE DIET |
| 12802 | MILK SHAKE DE MORANGO DIET |
| 12803 | MILK SHAKE DIETETICO |
| 12804 | MILK SHAKE DE MORANGO DIETETICO |
| 12901 | MARIA MOLE DIET |
| 12902 | BABA DE SAPO OU DE MOCA DIET |
| 12903 | MARIA MOLE DIETETICA |
| 12904 | BABA DE SAPO OU DE MOCA DIETETICA |
| 13001 | COCADA DIET |
| 13002 | QUEBRA QUEIXO DIET |
| 13003 | COCADA DIETETICA |
| 13004 | QUEBRA QUEIXO DIETETICO |
| 13101 | DOCE DE AMENDOIM DIET |
| 13102 | PE DE MOLEQUE DIET |
| 13103 | PACOCA DIET |
| 13104 | TORRAO DE AMENDOIM DIET |
| 13105 | PACOQUINHA DE AMENDOIM DIET |
| 13106 | PACOCA DE CASTANHA DE CAJU DIET |
| 13107 | AMENDOIM CARAMELIZADO DIET |
| 13108 | AMENDOIM AMANTEIGADO DIET |
| 13109 | AMENDOIM ACHOCOLATADO DIET |
| 13110 | DOCE DE AMENDOIM DIETETICO |
| 13111 | PE DE MOLEQUE DIETETICO |
| 13112 | PACOCA DIETETICA |
| 13113 | TORRAO DE AMENDOIM DIETETICO |
| 13114 | PACOQUINHA DE AMENDOIM DIETETICA |
| 13115 | PACOCA DE CASTANHA DE CAJU DIETETICA |
| 13116 | AMENDOIM CARAMELIZADO DIETETICO |
| 13117 | AMENDOIM AMANTEIGADO DIETETICO |
| 13118 | AMENDOIM ACHOCOLATADO DIETETICO |
| 13201 | DOCE DE ABOBORA DIET |
| 13202 | DOCE DE ABOBORA DIETETICO |
| 13301 | DOCE DE BATATA DOCE DIET |
| 13302 | DOCE DE BATATA DOCE DIETETICO |
| 13401 | BRIGADEIRO DIET |
| 13402 | BRIGADEIRO DIETETICO |
| 13501 | BOMBA DE QUALQUER TIPO DIET |
| 13502 | BOMBA DE QUALQUER TIPO DIETETICA |
| 13601 | DOCE DE QUEIJO DIET |
| 13602 | DOCE DE QUEIJO DIETETICO |
| 13701 | DOCE A BASE DE OVOS DIET |
| 13702 | DOCE DE OVOS COM AMENDOA DIET |
| 13703 | SCHIMIER DE OVOS DIET |
| 13704 | CACAROLA ITALIANA DIET |
| 13705 | QUINDIM DIET |
| 13706 | DOCE DE OVOS DIET |
| 13707 | PAPO DE ANJO DIET |
| 13708 | FIOS DE OVOS DIET |
| 13709 | DOCE A BASE DE OVOS DIETETICO |
| 13710 | DOCE DE OVOS COM AMENDOA DIETETICO |
| 13711 | SCHIMIER DE OVOS DIETETICO |
| 13712 | CACAROLA ITALIANA DIETETICA |
| 13713 | QUINDIM DIETETICO |
| 13714 | DOCE DE OVOS DIETETICO |
| 13715 | PAPO DE ANJO DIETETICO |
| 13716 | FIOS DE OVOS DIETETICO |
| 13801 | DOCE A BASE DE LEITE DIET |
| 13802 | PAVE DIET |
| 13803 | AMBROSIA DIET |
| 13804 | LEITE GELEIFICADO DIET |
| 13805 | CHANDELE DE QUALQUER SABOR DIET |
| 13806 | DOCE A BASE DE LEITE DIETETICO |
| 13807 | PAVE DIETETICO |
| 13808 | AMBROSIA DIETETICA |
| 13809 | LEITE GELEIFICADO DIETETICO |
| 13810 | CHANDELE DE QUALQUER SABOR DIETETICO |
| 13901 | GELATINA LIGHT |
| 13902 | GELATINA EM PO LIGHT |
| 13903 | GELATINA EM FOLHA LIGHT |
| 14001 | ADOCANTE LIGHT |
| 14002 | ADOCANTE EM PO LIGHT |
| 14003 | ADOCANTE LIQUIDO LIGHT |
| 14101 | BARRA DE CEREAIS LIGHT |
| 14201 | ARROZ CARAMELIZADO |
| 14301 | DOCE DE GERGELIM |
| 14401 | CREME DE AVELA |
| 14501 | ARROZ DOCE |
| 14601 | GLICOSE DE MANDIOCA |
| 14701 | VITAMINA CONCENTRADA (SHAKE) |
| 14801 | CASQUINHA DE SORVETE |
| 00101 | SAL REFINADO |
| 00102 | SAL IODADO |
| 00201 | SAL GROSSO |
| 00202 | SAL TRITURADO |
| 00203 | SAL MOIDO |
| 00204 | SAL GROSSO TEMPERADO |
| 00301 | SAL AMONIACO |
| 00401 | TEMPERO MISTO INDUSTRIALIZADO EM PASTA |
| 00402 | CONDIMENTO MISTO INDUSTRIALIZADO EM PASTA |
| 00403 | SALHO |
| 00404 | ALHOSAL |
| 00405 | ARISCO |
| 00406 | TEMPERO ARISCO |
| 00407 | ALHO EM PASTA |
| 00408 | MINHOTO ALHO E SAL |
| 00409 | PURE DE ALHO |
| 00410 | CREME DE ALHO |
| 00411 | MASSA DE ALHO |
| 00412 | TEMPERO ALHO E SAL |
| 00413 | TEMPERO COMPLETO EM PASTA |
| 00501 | BICARBONATO DE SODIO |
| 00502 | BICARBONATO |
| 00601 | FERMENTO EM PO |
| 00602 | FERMENTO |
| 00603 | FERMENTO BIOLOGICO |
| 00701 | GLUTAMATO MONOSSODICO |
| 00702 | AJINOMOTO |
| 00703 | REALCADOR DE SABOR |
| 00801 | GERSAL |
| 00901 | BAUNILHA EM PO |
| 00902 | ESSENCIA DE BAUNILHA |
| 00903 | VANILINA |
| 01001 | CANELA EM PO |
| 01002 | CANELA EM PAU |
| 01003 | CANELA EM FOLHA |
| 01101 | CRAVO (TEMPERO INDUSTRIALIZADO) |
| 01102 | CRAVO TEMPERADO |
| 01103 | CRAVO DA INDIA |
| 01201 | COLORAU |
| 01202 | COLORIFICO |
| 01203 | URUCU |
| 01204 | URUCUM |
| 01205 | COLORAU COM PIMENTA |
| 01301 | PAPRIKA |
| 01401 | NOZ MOSCADA (TEMPERO INDUSTRIALIZADO) |
| 01501 | MOSTARDA (TEMPERO INDUSTRIALIZADO) |
| 01601 | LOURO (TEMPERO INDUSTRIALIZADO) |
| 01602 | LOURO EM PO |
| 01603 | LOURO EM FOLHA |
| 01604 | FOLHA DE LOURO |
| 01701 | ACAFRAO EM PO |
| 01801 | COMINHO EM GRAO |
| 01802 | CUMINHO EM GRAO |
| 01901 | SEGURELHA (TEMPERO INDUSTRIALIZADO) |
| 02001 | MOLHO DE TOMATE COM ERVAS |
| 02101 | ALFAVACA (TEMPERO INDUSTRIALIZADO) |
| 02102 | QUIOIO (TEMPERO INDUSTRIALIZADO) |
| 02103 | MANGERICAO DE MOLHO (TEMPERO INDUSTRIALIZADO) |
| 02104 | ALFAVACA DE CHEIRO (TEMPERO INDUSTRIALIZADO) |
| 02105 | MANGERICAO (TEMPERO INDUSTRIALIZADO) |
| 02106 | ALFAVACA CHEIROSA (TEMPERO INDUSTRIALIZADO) |
| 02107 | MOLHO DE MANGERICAO |
| 02201 | OREGANO (TEMPERO INDUSTRIALIZADO) |
| 02202 | OREGAO (TEMPERO INDUSTRIALIZADO) |
| 02203 | OUREGAO (TEMPERO INDUSTRIALIZADO) |
| 02301 | HORTELA (TEMPERO INDUSTRIALIZADO) |
| 02302 | HORTELA ROMANA (TEMPERO INDUSTRIALIZADO) |
| 02303 | HORTELA DAS HORTAS (TEMPERO INDUSTRIALIZADO) |
| 02304 | HORTELA COMUM (TEMPERO INDUSTRIALIZADO) |
| 02305 | LEVANTE (TEMPERO INDUSTRIALIZADO) |
| 02306 | HORTELA PRETA (TEMPERO INDUSTRIALIZADO) |
| 02307 | HORTELA PIMENTA (TEMPERO INDUSTRIALIZADO) |
| 02308 | ALEVANTE (TEMPERO INDUSTRIALIZADO) |
| 02401 | ALCAPARRA EM CONSERVA |
| 02501 | MANJERONA SECA |
| 02502 | MANJERONA VERDADEIRA SECA |
| 02503 | MANJERONA INGLESA SECA |
| 02504 | MANJERONA DESIDRATADA |
| 02505 | MANJERONA VERDADEIRA DESIDRATADA |
| 02506 | MANJERONA INGLESA DESIDRATADA |
| 02601 | COENTRO (TEMPERO INDUSTRIALIZADO) |
| 02602 | COENTRO EM PO |
| 02603 | SEMENTE DE COENTRO |
| 02604 | COENTRO EM GRAO |
| 02701 | ALHO EM PO |
| 02702 | ALHO DESIDRATADO |
| 02801 | CEBOLA EM PO |
| 02901 | CHEIRO VERDE EM PO |
| 03001 | PIMENTAO EM PO |
| 03101 | PIMENTA DO REINO EM PO OU EM GRAO |
| 03102 | PIMENTA DO REINO EM PO |
| 03103 | PIMENTA DO REINO EM GRAO |
| 03104 | PIMENTA DO REINO CLARA |
| 03105 | PIMENTA DA INDIA (PIMENTA DO REINO) |
| 03106 | PIMENTA DO REINO BRANCA |
| 03107 | PIMENTA DO REINO PRETA |
| 03108 | PIMENTA DO REINO ESCURA |
| 03109 | PIMENTA DO REINO |
| 03201 | PIMENTA DO REINO E COMINHO |
| 03202 | COMINHO E PIMENTA DO REINO |
| 03301 | VINAGRE DE ALCOOL |
| 03302 | VINAGRE DE CANA |
| 03401 | VINAGRE DE VINHO |
| 03402 | VINAGRE DE UVA |
| 03501 | VINAGRE DE MACA |
| 03601 | MOLHO DE SOJA |
| 03602 | NATOR |
| 03603 | MOLHO JAPONES |
| 03604 | SHOYO |
| 03605 | TSUQUEMONO |
| 03606 | SAKURO |
| 03607 | MOLHO SHOYU |
| 03701 | MOLHO INGLES |
| 03801 | LEITE DE COCO |
| 03901 | MOLHO TARTARO |
| 04001 | TEMPERO DE SALADAS |
| 04101 | MOLHO DE TUCUPI COM PIMENTA |
| 04102 | TUCUPI EM MOLHO COM PIMENTA |
| 04103 | TUCUPI COM PIMENTA |
| 04201 | MOLHO DE HORTELA |
| 04301 | MAIONESE |
| 04302 | MAIONESE COM LIMAO |
| 04303 | MAIONESE COM ATUM |
| 04304 | MAIONESE COM AZEITONA |
| 04305 | MOLHO DE MAIONESE |
| 04306 | MAIONESE DE QUALQUER SABOR |
| 04307 | MAIONESE DE LEITE |
| 04401 | CALDO DE GALINHA |
| 04501 | CALDO DE CARNE |
| 04502 | CALDO DE PICANHA |
| 04601 | CURRY |
| 04701 | MASSA DE TOMATE |
| 04702 | EXTRATO DE TOMATE |
| 04703 | GELEIA DE TOMATE |
| 04704 | PURE DE TOMATE |
| 04705 | POLPA DE TOMATE |
| 04801 | MOLHO DE TOMATE |
| 04802 | KETCHUP |
| 04803 | CATCHUP |
| 04804 | CAT-CHUP |
| 04805 | KAT-CHUP |
| 04806 | MOLHO REFOGADO DE TOMATE |
| 04807 | KATCHUP |
| 04901 | MOLHO BOLONHESA |
| 04902 | MOLHO A BOLONHESA |
| 04903 | MOLHO DE TOMATE COM CARNE |
| 04904 | MOLHO DE MACARRONADA |
| 05001 | MOLHO VEGETAL |
| 05101 | MOLHO XADREZ |
| 05201 | COMINHO EM PO |
| 05202 | CUMINHO EM PO |
| 05301 | PIMENTA DO REINO E ALHO EM PO (TEMPERO MISTO EM PO OU EM GRAO) |
| 05302 | TEMPERO MISTO EM PO |
| 05303 | PIMENTA DO REINO EM GRAO E ALHO (TEMPERO MISTO EM PO OU EM GRAO) |
| 05304 | TEMPERO MISTO EM GRAO |
| 05401 | CALDO DE LEGUMES |
| 05501 | PEIXE EM PO |
| 05502 | KATSUOBUSHI (PEIXE EM PO) |
| 05601 | TEMPERO DE MASSAS |
| 05701 | SAL DIET |
| 05702 | SAL DIETETICO |
| 05703 | SAL IODADO DIET |
| 05704 | SAL IODADO DIETETICO |
| 05801 | TEMPERO DE FEIJAO |
| 05802 | CALDO DE FEIJOADA KNORR |
| 05901 | AMACIANTE DE CARNE |
| 05902 | ZURITA |
| 06001 | FONDOR |
| 06101 | PIMENTA EM PO |
| 06201 | TEMPERO DE OMELETE EM PO |
| 06202 | OMELETE EM PO (TEMPERO) |
| 06301 | MASSA DE PEIXE |
| 06401 | MOLHO DE FUNGHI |
| 06501 | ERVILHA FRITA EM PO OU GRAO |
| 06502 | ERVILHA EM PO OU GRAO (FRITA) |
| 06601 | FAVA FRITA EM PO OU GRAO |
| 06602 | FAVA PO OU GRAO (FRITA) |
| 06701 | MOLHO DE MILHO VERDE |
| 06801 | MISSO |
| 06901 | SALSA DESIDRATADA |
| 07001 | MOLHO DE PIZZA |
| 07002 | MOLHO PARA PIZZA |
| 07101 | TUCUPI EM CALDO SEM PIMENTA |
| 07102 | CALDO DE TUCUPI SEM PIMENTA |
| 07201 | MOLHO BRANCO |
| 07301 | CALDO DE BACON |
| 07401 | CALDO DE CALABREZA |
| 07501 | CALDO DE QUEIJO |
| 07502 | CREME DE QUEIJO |
| 07503 | MOLHO QUATRO QUEIJOS |
| 07504 | PATE DE QUEIJO |
| 07601 | CALDO NAO-ESPECIFICADO |
| 07602 | TEMPERO NAO-ESPECIFICADO EM TABLETE |
| 07701 | CALDO DE PEIXE |
| 07702 | CALDO DE BACALHAU |
| 07801 | CALDO DE CAMARAO |
| 07802 | CAMARAO EM PO |
| 07901 | CALDO DE TOMATE |
| 08001 | MOLHO NAO-ESPECIFICADO |
| 08101 | MOLHO DE ALHO |
| 08102 | EXTRATO DE ALHO |
| 08201 | COALHO |
| 08202 | COAGULANTE LIQUIDO |
| 08301 | TEMPERO EM PO NAO-ESPECIFICADO |
| 08401 | TEMPERO NAO-ESPECIFICADO |
| 08402 | CONDIMENTO NAO-ESPECIFICADO |
| 08403 | TEMPERO CASEIRO NAO-ESPECIFICADO |
| 08501 | SAL AMARGO |
| 08601 | SALVIA EM FOLHA (TEMPERO INDUSTRIALIZADO) |
| 08701 | TOMILHO EM FOLHA (TEMPERO INDUSTRIALIZADO) |
| 08801 | VINAGRE NAO-ESPECIFICADO |
| 08901 | VINAGRE DE ARROZ |
| 09001 | VINAGRE DE MILHO |
| 09101 | GERGELIM EM PO |
| 09102 | GERGELIM EM GRAO |
| 09201 | MOLHO DE PIMENTA |
| 09301 | MOLHO MADEIRA |
| 09401 | MOLHO PARA SALADA |
| 09501 | MOLHO AGRIDOCE |
| 09601 | MOLHO AO SUGO |
| 09701 | MOLHO BARBECUE |
| 09801 | MOLHO DE FRANGO |
| 09901 | MOLHO PARA CARNE |
| 10001 | MOLHO SIRIO |
| 10101 | MOLHO TEMPERADO PARMESAO |
| 10201 | CEBOLINHA DESIDRATADA |
| 10301 | TOMATE SECO |
| 10302 | TOMATE DESIDRATADO |
| 10401 | MAIONESE LIGHT |
| 10402 | MAIONESE COM LIMAO LIGHT |
| 10403 | MAIONESE COM ATUM LIGHT |
| 10404 | MAIONESE COM AZEITONA LIGHT |
| 10405 | MOLHO DE MAIONESE LIGHT |
| 10406 | MAIONESE DE QUALQUER SABOR LIGHT |
| 10501 | MOLHO DE SOJA LIGHT |
| 10502 | NATOR LIGHT |
| 10503 | MOLHO JAPONES LIGHT |
| 10504 | SHOYO LIGHT |
| 10505 | TSUQUEMONO LIGHT |
| 10506 | SAKURO LIGHT |
| 10601 | LEITE DE COCO LIGHT |
| 10701 | TEMPERO DE SALADAS LIGHT |
| 10702 | MOLHO DE SALADAS LIGHT |
| 10801 | MOLHO DE HORTELA LIGHT |
| 10901 | MOLHO DE TOMATE LIGHT |
| 10902 | KETCHUP LIGHT |
| 10903 | CATCHUP LIGHT |
| 10904 | CAT-CHUP LIGHT |
| 10905 | KAT-CHUP LIGHT |
| 10906 | MOLHO REFOGADO DE TOMATE LIGHT |
| 11001 | SAL REFINADO LIGHT |
| 11002 | SAL IODADO LIGHT |
| 11101 | CALDO DE CEBOLA |
| 11201 | CALDO DE COSTELA |
| 11301 | CALDO DE MANDIOCA |
| 11401 | COCO EM FLOCOS |
| 11402 | FLOCOS DE COCO |
| 11501 | COAGULANTE LIQUIDO (CLORETO DE MAGNESIO) |
| 11601 | EMUSTAB (EMULSIFICANTE) |
| 11701 | PIMENTA CALABRESA |
| 11801 | TEMPERO SAZON |
| 11802 | SAZON (TEMPERO) |
| 11803 | SAZON |
| 11901 | VINAGRE DE ALCOOL E VINHO (COMPOSTO) |
| 11902 | AGRIN |
| 12001 | CALDO DE ARROZ |
| 12101 | TEMPERO NAO-ESPECIFICADO EM PACOTE |
| 12201 | TEMPERO MISTO LIQUIDO |
| 12202 | TEMPERO LIQUIDO MISTO |
| 12203 | TEMPERO LIQUIDO DE CEBOLA E ALHO |
| 12204 | TEMPERO LIQUIDO COMPLETO |
| 12301 | VINAGRE DE LIMAO |
| 12401 | VINAGRE BALSAMICO |
| 12501 | CALDO DE PERU |
| 12601 | ERVA DOCE (TEMPERO INDUSTRIALIZADO) |
| 00101 | FILE MIGNON |
| 00102 | FILE SEM MIGNON |
| 00103 | FILE COM MIGNON |
| 00104 | MIGNON |
| 00201 | CONTRAFILE |
| 00202 | FILE CURTO |
| 00203 | CHULETA COM OSSO (CONTRAFILE) |
| 00204 | FILE ESPECIAL |
| 00205 | BISTECA BOVINA |
| 00206 | CHULETA BOVINA |
| 00207 | PONTA DE CHULETA |
| 00301 | ALCATRA |
| 00302 | PONTA DE PATINHO |
| 00303 | MAMINHA |
| 00304 | PICANHA |
| 00305 | CHAPEU DE BISPO (MAMINHA) |
| 00306 | ALCATRA BOVINA |
| 00307 | PONTA DE ALCATRA |
| 00308 | ALCATRA COM OSSO |
| 00309 | MIOLO DE ALCATRA |
| 00401 | CHA DE DENTRO |
| 00402 | COXAO MOLE (CHA DE DENTRO) |
| 00403 | COXAO DE DENTRO (CHA DE DENTRO) |
| 00404 | COXAO MOLE |
| 00501 | PATINHO |
| 00502 | CABECA DE LOMBO (CARNE BOVINA) |
| 00503 | BOLA DO PATINHO |
| 00504 | PATINHO COM OSSO |
| 00601 | LAGARTO REDONDO |
| 00602 | LOMBO DOS MOCOS (LAGARTO REDONDO) |
| 00603 | PAULISTA REDONDO |
| 00604 | POSTA BRANCA |
| 00605 | LAGARTO PAULISTA |
| 00606 | TATU (LAGARTO REDONDO) |
| 00607 | PAULISTA |
| 00608 | LOMBO PAULISTA (CARNE BOVINA) |
| 00701 | LAGARTO COMUM |
| 00702 | POSTA VERMELHA |
| 00703 | LAGARTO PLANO |
| 00704 | PAULISTA PLANO |
| 00705 | COXAO DE FORA (LAGARTO COMUM) |
| 00706 | COXAO DURO (LAGARTO COMUM) |
| 00707 | CHA DE FORA |
| 00708 | COXAO DURO |
| 00709 | CHANDANGA BOVINA |
| 00801 | ACEM |
| 00802 | LOMBO BOVINO |
| 00803 | AGULHA (ACEM) |
| 00804 | ACEM COM OSSO |
| 00805 | LOMBO BOVINO COM OSSO |
| 00806 | AGULHA COM OSSO |
| 00807 | PONTA DE AGULHA |
| 00808 | MIOLO DE ACEM |
| 00809 | MIOLO DE AGULHA |
| 00901 | PA |
| 00902 | PONTA DA PA |
| 00903 | POSTA GORDA |
| 00904 | MIOLO DA PA |
| 00905 | PALETA |
| 00906 | CRUZ MACHADO |
| 00907 | PALETA COM OSSO |
| 00908 | PA COM OSSO |
| 00909 | MIOLO DE PALETA |
| 00910 | MIOLO DA PALETA |
| 00911 | PA SEM OSSO |
| 00912 | BOLA DA PA |
| 01001 | MUSCULO BOVINO |
| 01002 | MUSCULO DA PA |
| 01003 | MUSCULO DO TRASEIRO |
| 01004 | GARRAO |
| 01005 | MUSCULO DO DIANTEIRO |
| 01006 | CHAMBARIL |
| 01007 | CARNE DE PESCOCO |
| 01008 | MARICA |
| 01009 | CARNE MARICA BOVINA |
| 01010 | CARNE DE VAZIO |
| 01011 | VAZIO (CARNE BOVINA) |
| 01012 | CARNE BOVINA DO VAZIO |
| 01013 | MUSCULO COM OSSO |
| 01014 | OSSOBUCO |
| 01101 | PEITO BOVINO |
| 01102 | PONTA DE PEITO |
| 01103 | MIOLO DE PEITO BOVINO |
| 01201 | CAPA DE FILE |
| 01202 | FRALDINHA (CAPA DE FILE) |
| 01203 | ABA DE FILE |
| 01204 | CAPA DE CONTRAFILE |
| 01205 | CAPA DE MIGNON |
| 01206 | CAPA DE BISTECA BOVINA |
| 01301 | COSTELA BOVINA |
| 01302 | CARNE CHUPA MOLHO |
| 01303 | MATAMBRE |
| 01304 | PONTA DE COSTELA |
| 01305 | COSTELA DE BOI |
| 01401 | CARNE MOIDA DE PRIMEIRA |
| 01402 | GUIZADO (CARNE MOIDA DE PRIMEIRA) |
| 01501 | CARNE MOIDA DE SEGUNDA |
| 01502 | GUIZADO (CARNE MOIDA DE SEGUNDA) |
| 01601 | CARNE BOVINA DE PRIMEIRA |
| 01602 | CARNE BOVINA DE PRIMEIRA COM OSSO |
| 01701 | CARNE BOVINA DE SEGUNDA |
| 01702 | ALCATRINHA COM OSSO (DIANTEIRO DE SEGUNDA) |
| 01703 | CARNE BOVINA COM OSSO NAO-ESPECIFICADA |
| 01704 | JACARE (CARNE BOVINA DE SEGUNDA C/ OSSO) |
| 01705 | CONTRA FILE DE SEGUNDA |
| 01706 | FILE DE SEGUNDA |
| 01707 | ALCATRINHA |
| 01708 | CARNE BOVINA DE SEGUNDA COM OSSO |
| 01801 | VISCERA BOVINA NAO-ESPECIFICADA |
| 01802 | FRESSURA BOVINA NAO-ESPECIFICADA |
| 01803 | FRISSURA BOVINA NAO-ESPECIFICADA |
| 01804 | FISSURA BOVINA NAO-ESPECIFICADA |
| 01805 | MIUDO BOVINO NAO-ESPECIFICADO |
| 01806 | PANELADA (VISCERAS BOVINAS NAO-ESPECIFICADAS) |
| 01901 | CORACAO BOVINO |
| 02001 | RIM BOVINO |
| 02101 | PULMAO BOVINO |
| 02102 | BOFE BOVINO |
| 02103 | BOBO BOVINO |
| 02201 | MIOLO BOVINO |
| 02301 | BUCHO BOVINO |
| 02302 | ESTOMAGO BOVINO |
| 02303 | CUALHEIRA BOVINA |
| 02304 | BUCHADA BOVINA |
| 02401 | TRIPA BOVINA |
| 02402 | DOBRADINHA FRESCA |
| 02403 | FATO BOVINO |
| 02404 | FATO CAPRINO |
| 02405 | MONDONGO (TRIPA BOVINA) |
| 02501 | FIGADO BOVINO |
| 02502 | FIGADO DE BOI |
| 02601 | CUPIM |
| 02602 | CUPIM BOVINO |
| 02701 | LINGUA BOVINA |
| 02801 | RABADA BOVINA |
| 02802 | RABO BOVINO |
| 02901 | TESTICULO BOVINO |
| 03001 | CABECA BOVINA |
| 03002 | CASCO BOVINO (FRONTAL DA CABECA) |
| 03003 | FRONTAL DA CABECA (CASCO BOVINO) |
| 03101 | MOCOTO BOVINO |
| 03102 | PE BOVINO |
| 03103 | CORREDOR BOVINO |
| 03104 | BRACO BOVINO |
| 03105 | MAO BOVINA |
| 03106 | PERNA BOVINA |
| 03107 | MAO DE VACA |
| 03108 | CANELA BOVINA |
| 03201 | OSSO BOVINO |
| 03202 | OSSADA BOVINA |
| 03301 | CARRE |
| 03302 | CHULETA SUINA |
| 03303 | COSTELETA SUINA |
| 03304 | PALETA SUINA |
| 03305 | BISTECA SUINA |
| 03306 | PA SUINA |
| 03401 | PERNIL SUINO |
| 03402 | COXAO SUINO |
| 03403 | QUARTO SUINO |
| 03501 | COSTELA SUINA |
| 03502 | COSTELINHA SUINA |
| 03503 | COSTELA DE PORCO |
| 03504 | COSTELINHA DE PORCO |
| 03601 | CARNE SUINA COM OSSO NAO-ESPECIFICADA |
| 03701 | LOMBO SUINO |
| 03702 | LOMBINHO SUINO |
| 03703 | LOMBO SUINO RECHEADO E TEMPERADO |
| 03704 | COPA SUINA |
| 03705 | FILE MIGNON SUINO |
| 03801 | TOUCINHO |
| 03802 | TOUCINHO FRESCO |
| 03803 | TOUCINHO DE TORRESMO |
| 03804 | PELE SUINA FRESCA |
| 03901 | VISCERA SUINA NAO-ESPECIFICADA |
| 03902 | FRESSURA SUINA NAO-ESPECIFICADA |
| 03903 | MIUDO SUINO NAO-ESPECIFICADO |
| 03904 | FRISSURA SUINA NAO-ESPECIFICADA |
| 03905 | FISSURA SUINA NAO-ESPECIFICADA |
| 03906 | ARRASTO SUINO NAO-ESPECIFICADO |
| 03907 | SARAPATEL SUINO FRESCO |
| 04001 | CORACAO SUINO |
| 04101 | CARNE SUINA NAO-ESPECIFICADA |
| 04102 | CARNE DE PORCO NAO ESPECIFICADA |
| 04201 | BOFE SUINO |
| 04202 | PULMAO SUINO |
| 04301 | CARNE MOIDA NAO-ESPECIFICADA |
| 04302 | GUIZADO (CARNE MOIDA NAO-ESPECIFICADA) |
| 04401 | BUCHO SUINO |
| 04402 | ESTOMAGO SUINO |
| 04501 | TRIPA SUINA |
| 04502 | FATO SUINO |
| 04601 | FIGADO SUINO |
| 04701 | LINGUA SUINA |
| 04801 | RABO SUINO |
| 04802 | RABINHO SUINO |
| 04901 | CABECA SUINA |
| 04902 | CABECA DE PORCO |
| 05001 | ORELHA SUINA FRESCA |
| 05101 | SANGUE SUINO |
| 05102 | SANGUE COAGULADO SUINO |
| 05103 | SARRABULHO |
| 05104 | SANGUE SUINO COAGULADO |
| 05201 | OSSO SUINO |
| 05202 | OSSADA SUINA |
| 05203 | MOCOTO SUINO |
| 05204 | CARCACA SUINA |
| 05301 | PORCO VIVO |
| 05302 | SUINO VIVO |
| 05401 | CARNE DE CABRITO |
| 05402 | CARNE DE BODE |
| 05403 | CARNE CAPRINA |
| 05404 | COSTELA DE BODE |
| 05405 | CARNE DE CAPRINO |
| 05501 | CABRITO VIVO |
| 05502 | BODE VIVO |
| 05601 | CARNE DE CARNEIRO |
| 05602 | CARNE DE OVELHA |
| 05603 | COSTELA DE CARNEIRO |
| 05604 | COXAO DE CARNEIRO |
| 05605 | CARNE OVINA |
| 05606 | PALETA DE CARNEIRO |
| 05607 | PERNIL DE CARNEIRO |
| 05608 | DIANTEIRO DE CARNEIRO |
| 05701 | CARNEIRO VIVO |
| 05702 | OVELHA VIVA |
| 05801 | MOCOTO DE CAPRINO |
| 05901 | CHA BOVINA NAO-ESPECIFICADA |
| 06001 | CARNE SUINA DE PRIMEIRA NAO-ESPECIFICADA |
| 06101 | COSTELA NAO-ESPECIFICADA |
| 06201 | PE DE CAPRINO |
| 06301 | PERNIL NAO-ESPECIFICADO |
| 06401 | LAGARTO BOVINO NAO-ESPECIFICADO |
| 06402 | LAGARTO RECHEADO |
| 06501 | ALCATRA SUINA |
| 06601 | TANAJURA |
| 06701 | OUTROS ANIMAIS VIVOS |
| 06702 | VEADO VIVO |
| 06703 | PACA VIVA |
| 06704 | TATU VIVO |
| 06705 | COELHO VIVO |
| 06706 | LEBRE VIVA |
| 06707 | PREA VIVA |
| 06708 | CUTIA VIVA |
| 06709 | MACACO VIVO |
| 06801 | CARNE SUINA SEM OSSO NAO-ESPECIFICADA |
| 06802 | CARNE SUINA EM BIFE |
| 06901 | PASSARINHA BOVINA |
| 06902 | BACO BOVINO |
| 07001 | PASSARINHA SUINA |
| 07002 | BACO SUINO |
| 07101 | BRAJOLA |
| 07102 | BRACHOLA |
| 07103 | BIFE ROLE CRU |
| 07104 | ROLETA |
| 07201 | BUCHO DE CARNEIRO |
| 07202 | BUCHADA DE CARNEIRO |
| 07203 | ESTOMAGO DE CARNEIRO |
| 07204 | BUCHADA DE BODE |
| 07205 | BUCHO DE BODE |
| 07301 | LIMPEZA DE CARNE BOVINA |
| 07302 | APARA DE CARNE BOVINA |
| 07303 | MUXIBA (GORDURA BOVINA) |
| 07304 | GORDURA BOVINA |
| 07305 | XAXADO BOVINO |
| 07401 | BUCHO NAO-ESPECIFICADO |
| 07402 | BUCHADA NAO-ESPECIFICADA |
| 07501 | SUA SUINA |
| 07502 | ASSUA SUINA |
| 07601 | CARNE DE OUTROS ANIMAIS |
| 07602 | CARNE DE VEADO |
| 07603 | CARNE DE PACA |
| 07604 | CARNE DE TATU |
| 07605 | CARNE DE COELHO |
| 07606 | CARNE DE LEBRE |
| 07607 | CARNE DE PREA |
| 07608 | CARNE DE CAVALO |
| 07609 | CARNE DE ANTA |
| 07610 | FIGADO DE CARNEIRO |
| 07611 | CARNE DE JACARE |
| 07612 | CARNE DE CUTIA |
| 07613 | CARNE DE JACARE SALGADA |
| 07614 | CARNE DE PORCO SILVESTRE |
| 07615 | CARNE DE CAPIVARA |
| 07616 | CARNE DE MACACO |
| 07617 | CARNE DE COTIA |
| 07619 | CARNE DE QUEIXADA |
| 07620 | CARNE DE COBRA JIBOIA |
| 07621 | CARNE DE PORCO DO MATO |
| 07622 | CARNE DE JABUTI |
| 07623 | CARNE DE PREGUICA |
| 07624 | CARNE DE TAMANDUA |
| 07625 | CARNE DE CUTIARA |
| 07701 | CARNE DE BUFALO |
| 07801 | CARNE DE PRIMEIRA NAO-ESPECIFICADA |
| 07802 | CARNE DE PRIMEIRA COM OSSO NAO-ESPECIFICADA |
| 07803 | BISTECA NAO-ESPECIFICADA |
| 07804 | CHULETA NAO-ESPECIFICADA |
| 07805 | FILE NAO-ESPECIFICADO |
| 07806 | COXAO NAO-ESPECIFICADO |
| 07901 | KIT DE CARNE DE CHURRASCO |
| 07902 | CARNE DE CHURRASCO (KIT) |
| 08001 | ESTOMAGO NAO-ESPECIFICADO |
| 08101 | VISCERA DE CARNEIRO |
| 08102 | FRESSURA DE CARNEIRO |
| 08103 | MIUDO DE CARNEIRO |
| 08104 | FRISSURA DE CARNEIRO |
| 08105 | FISSURA DE CARNEIRO |
| 08106 | FISSURA DE BODE |
| 08107 | MIUDO DE BODE NAO-ESPECIFICADO |
| 08201 | NARIZ SUINO |
| 08301 | PE SUINO FRESCO |
| 08302 | JOELHO SUINO |
| 08401 | PORCO OU LEITAO MORTO EVISCERADO |
| 08402 | LEITAO MORTO EVISCERADO |
| 08403 | SUINO MORTO EVISCERADO |
| 08404 | PORCO ABATIDO |
| 08501 | APARA DE CARNE SUINA |
| 08502 | LIMPEZA DE CARNE SUINA |
| 08601 | GARGANTA BOVINA |
| 08701 | CABECA DE BODE |
| 08702 | CABECA DE CARNEIRO |
| 08703 | CABECA CAPRINA |
| 08801 | CARNE DE TERNEIRO NAO-NASCIDO |
| 08901 | CARNE NAO-ESPECIFICADA |
| 08902 | CARNE EM BIFE |
| 09001 | CARNE BOVINA SEM OSSO NAO-ESPECIFICADA |
| 09101 | CARNE BOVINA NAO-ESPECIFICADA |
| 09201 | BACO NAO-ESPECIFICADO |
| 09301 | SANGUE DE CARNEIRO |
| 09401 | BODE ABATIDO |
| 09501 | BOFE NAO-ESPECIFICADO |
| 09601 | RIM DE BODE |
| 09701 | RIM SUINO |
| 09801 | BOFE DE CAPRINO |
| 09802 | PULMAO DE CAPRINO |
| 09901 | RIM NAO-ESPECIFICADO |
| 10001 | APARA NAO-ESPECIFICADA |
| 10101 | CARNE COM OSSO NAO-ESPECIFICADA |
| 10201 | CARNE BOVINA DE TERCEIRA |
| 10301 | CORACAO DE CAPRINO |
| 10401 | ORELHA CAPRINA FRESCA |
| 10501 | CARNE SUINA DE SEGUNDA NAO-ESPECIFICADA |
| 10502 | MUSCULO SUINO |
| 10601 | LOMBO NAO-ESPECIFICADO |
| 10701 | CARNE MOIDA DE TERCEIRA |
| 10702 | GUIZADO (CARNE MOIDA DE TERCEIRA) |
| 10801 | MUSCULO NAO-ESPECIFICADO |
| 10901 | OSSO NAO-ESPECIFICADO |
| 10902 | OSSADA NAO-ESPECIFICADA |
| 11001 | CARNE DE TERCEIRA NAO-ESPECIFICADA |
| 11101 | CARNE SEM OSSO NAO-ESPECIFICADA |
| 11201 | CARNE DE SEGUNDA SEM OSSO NAO-ESPECIFICADA |
| 11202 | CARNE DE SEGUNDA NAO-ESPECIFICADA |
| 11301 | FILE MIGNON ORGANICO |
| 11302 | FILE SEM MIGNON ORGANICO |
| 11303 | FILE COM MIGNON ORGANICO |
| 11304 | MIGNON ORGANICO |
| 11401 | CONTRAFILE ORGANICO |
| 11402 | FILE CURTO ORGANICO |
| 11403 | CHULETA COM OSSO (CONTRAFILE) ORGANICA |
| 11404 | FILE ESPECIAL ORGANICO |
| 11405 | BISTECA ORGANICA |
| 11406 | CHULETA ORGANICA |
| 11407 | PONTA DE CHULETA ORGANICA |
| 11501 | ALCATRA ORGANICA |
| 11502 | PONTA DE PATINHO ORGANICA |
| 11503 | MAMINHA ORGANICA |
| 11504 | PICANHA ORGANICA |
| 11505 | CHAPEU DE BISPO (MAMINHA) ORGANICO |
| 11506 | ALCATRA BOVINA ORGANICA |
| 11507 | PONTA DE ALCATRA ORGANICA |
| 11508 | ALCATRA COM OSSO ORGANICA |
| 11509 | MIOLO DE ALCATRA ORGANICO |
| 11601 | CHA DE DENTRO ORGANICA |
| 11602 | COXAO MOLE (CHA DE DENTRO) ORGANICO |
| 11603 | COXAO DE DENTRO (CHA DE DENTRO) ORGANICO |
| 11701 | PATINHO ORGANICO |
| 11702 | CABECA DE LOMBO (CARNE BOVINA) ORGANICA |
| 11703 | BOLA DO PATINHO ORGANICA |
| 11704 | PATINHO COM OSSO ORGANICO |
| 11801 | LAGARTO REDONDO ORGANICO |
| 11802 | LOMBO DOS MOCOS (LAGARTO REDONDO) ORGANICO |
| 11803 | PAULISTA REDONDO ORGANICO |
| 11804 | POSTA BRANCA ORGANICA |
| 11805 | LAGARTO PAULISTA ORGANICO |
| 11806 | TATU (LAGARTO REDONDO) ORGANICO |
| 11807 | PAULISTA ORGANICO |
| 11808 | LOMBO PAULISTA (CARNE BOVINA) ORGANICO |
| 11901 | LAGARTO COMUM ORGANICO |
| 11902 | POSTA VERMELHA ORGANICA |
| 11903 | LAGARTO PLANO ORGANICO |
| 11904 | PAULISTA PLANO ORGANICO |
| 11905 | COXAO DE FORA (LAGARTO COMUM) ORGANICO |
| 11906 | COXAO DURO (LAGARTO COMUM) ORGANICO |
| 11907 | CHA DE FORA ORGANICA |
| 12001 | CARNE BOVINA DE PRIMEIRA ORGANICA |
| 12002 | CARNE BOVINA DE PRIMEIRA COM OSSO ORGANICA |
| 12101 | PANCETA SUINA |
| 12201 | PEIXINHO (LAGARTO DO BRACO) |
| 12301 | SANGUE BOVINO |
| 12401 | SUA BOVINA |
| 00101 | PEIXE INTEIRO DE MAR NAO-ESPECIFICADO |
| 00201 | PEIXE EM POSTAS DE MAR NAO-ESPECIFICADO |
| 00301 | PEIXE EM FILE FRESCO DE MAR NAO-ESPECIFICADO |
| 00401 | PEIXE SALGADO DE MAR NAO-ESPECIFICADO |
| 00501 | PEIXE EM FILE CONGELADO DE MAR NAO-ESPECIFICADO |
| 01101 | PEIXE INTEIRO ANCHOVA |
| 01102 | PEIXE INTEIRO ENCHOVA |
| 01103 | PEIXE INTEIRO ANCHOVETA |
| 01104 | PEIXE INTEIRO ENCHOVINHA |
| 01105 | PEIXE INTEIRO ANCHOVA BAETA |
| 01106 | PEIXE INTEIRO ENCHOVA BAETA |
| 01107 | PEIXE INTEIRO ENCHOVETA |
| 01108 | PEIXE INTEIRO ANCHOVINHA |
| 01109 | PEIXE INTEIRO CHAMA CHUVA |
| 01201 | PEIXE EM POSTAS ANCHOVA |
| 01301 | PEIXE EM FILE FRESCO ANCHOVA |
| 01401 | PEIXE SALGADO ANCHOVA |
| 01501 | PEIXE EM FILE CONGELADO ANCHOVA |
| 02101 | PEIXE INTEIRO BADEJO |
| 02102 | PEIXE INTEIRO BADEJO BRANCO |
| 02103 | PEIXE INTEIRO BADEJO FERRO |
| 02104 | PEIXE INTEIRO BADEJO PRETO |
| 02105 | PEIXE INTEIRO BADEJO MIRA |
| 02106 | PEIXE INTEIRO BADEJO SALTAO |
| 02107 | PEIXE INTEIRO BADEJO BICUDO |
| 02108 | PEIXE INTEIRO BADEJO SABAO |
| 02109 | PEIXE INTEIRO MIRA |
| 02110 | PEIXE INTEIRO BADEJETE |
| 02201 | PEIXE EM POSTAS BADEJO |
| 02301 | PEIXE EM FILE FRESCO BADEJO |
| 02401 | PEIXE SALGADO BADEJO |
| 02501 | PEIXE EM FILE CONGELADO BADEJO |
| 03101 | PEIXE INTEIRO CORVINA |
| 03102 | PEIXE INTEIRO CORVINA RISCADA |
| 03103 | PEIXE INTEIRO CORVINA DE LISTA |
| 03104 | PEIXE INTEIRO CORVINA BRASILEIRA |
| 03105 | PEIXE INTEIRO CORVINA BACALHAU |
| 03106 | PEIXE INTEIRO CORVINA DE LINHA |
| 03107 | PEIXE INTEIRO CUCURUCA |
| 03108 | PEIXE INTEIRO CURUCA |
| 03109 | PEIXE INTEIRO CURURUCA |
| 03110 | PEIXE INTEIRO CHORA CHORA |
| 03111 | PEIXE INTEIRO CURURUQUIRA |
| 03112 | PEIXE INTEIRO CORVINOTA |
| 03113 | PEIXE INTEIRO DENTUDA |
| 03201 | PEIXE EM POSTAS CORVINA |
| 03301 | PEIXE EM FILE FRESCO CORVINA |
| 03401 | PEIXE SALGADO CORVINA |
| 03501 | PEIXE EM FILE CONGELADO CORVINA |
| 03901 | PEIXE CORVINA (FORMA DE COMERCIALIZACAO NAO-DISCRIMINADA) |
| 04101 | PEIXE INTEIRO CASTANHA |
| 04201 | PEIXE EM POSTAS CASTANHA |
| 04301 | PEIXE EM FILE FRESCO CASTANHA |
| 04401 | PEIXE SALGADO CASTANHA |
| 04501 | PEIXE EM FILE CONGELADO CASTANHA |
| 04901 | PEIXE CASTANHA (FORMA DE COMERCIALIZACAO NAO-DISCRIMINADA) |
| 05101 | PEIXE INTEIRO CAVALINHA |
| 05102 | PEIXE INTEIRO CAVALINHA DO NORTE |
| 05103 | PEIXE INTEIRO CARAPAU |
| 05104 | PEIXE INTEIRO LANCETA |
| 05201 | PEIXE EM POSTAS CAVALINHA |
| 05301 | PEIXE EM FILE FRESCO CAVALINHA |
| 05401 | PEIXE SALGADO CAVALINHA |
| 05501 | PEIXE EM FILE CONGELADO CAVALINHA |
| 06101 | PEIXE INTEIRO CHICHARRO |
| 06102 | PEIXE INTEIRO CHICHARRO PINTADO |
| 06103 | PEIXE INTEIRO CHICHARRO BRANCO |
| 06104 | PEIXE INTEIRO CHICHARRO CARAPAU |
| 06105 | PEIXE INTEIRO CHICHARRO ALAMARIM |
| 06106 | PEIXE INTEIRO CHICHARRO OLHO GRANDE |
| 06107 | PEIXE INTEIRO CHICHARRO CALABAR |
| 06108 | PEIXE INTEIRO CHICHARRO CAVALA |
| 06109 | PEIXE INTEIRO SUREL |
| 06110 | PEIXE INTEIRO CAVALINHA DE REIS (CHICHARRO) |
| 06201 | PEIXE EM POSTAS CHICHARRO |
| 06301 | PEIXE EM FILE FRESCO CHICHARRO |
| 06401 | PEIXE SALGADO CHICHARRO |
| 06501 | PEIXE EM FILE CONGELADO CHICHARRO |
| 07101 | PEIXE INTEIRO RAIA |
| 07102 | PEIXE INTEIRO ARRAIA |
| 07103 | PEIXE INTEIRO GUITARRA |
| 07104 | PEIXE INTEIRO RAIA CARIMBADA |
| 07105 | PEIXE INTEIRO RAIA VIOLA |
| 07106 | PEIXE INTEIRO ARRAIA CHITA |
| 07107 | PEIXE INTEIRO RAIA LIXA |
| 07108 | PEIXE INTEIRO VIOLA |
| 07109 | PEIXE INTEIRO ARRAIA PREGO |
| 07110 | PEIXE INTEIRO ARRAIA VIOLA |
| 07111 | PEIXE INTEIRO RAIA PREGO |
| 07112 | PEIXE INTEIRO CACAO VIOLA |
| 07113 | PEIXE INTEIRO ARRAIA AMARELA |
| 07114 | PEIXE INTEIRO RAIA CHITA |
| 07115 | PEIXE INTEIRO RAIA AMARELA |
| 07116 | PEIXE INTEIRO ARRAIA LIXA |
| 07117 | PEIXE INTEIRO ARRAIA BORBOLETA |
| 07118 | PEIXE INTEIRO RAIA MANTEIGA |
| 07119 | PEIXE INTEIRO RAIA BORBOLETA |
| 07120 | PEIXE INTEIRO ARRAIA MANTEIGA |
| 07121 | PEIXE INTEIRO ARRAIA CARIMBADA |
| 07122 | PEIXE INTEIRO TICONHA |
| 07123 | PEIXE INTEIRO ARRAIA ARARA |
| 07124 | PEIXE INTEIRO RAIA PINTADA |
| 07125 | PEIXE INTEIRO RAIA ARARA |
| 07126 | PEIXE INTEIRO ARRAIA PINTADA |
| 07127 | PEIXE INTEIRO JABIRETE |
| 07128 | PEIXE INTEIRO BORBOLETA |
| 07129 | PEIXE INTEIRO NARINARI |
| 07130 | PEIXE INTEIRO JABIBIRETE |
| 07201 | PEIXE EM POSTAS RAIA |
| 07301 | PEIXE EM FILE FRESCO RAIA |
| 07302 | PEIXE EM FILE FRESCO VIOLA |
| 07401 | PEIXE SALGADO RAIA |
| 07501 | PEIXE EM FILE CONGELADO RAIA |
| 08101 | PEIXE INTEIRO NAMORADO |
| 08102 | PEIXE INTEIRO QUATI |
| 08103 | PEIXE INTEIRO BATATA |
| 08201 | PEIXE EM POSTAS NAMORADO |
| 08301 | PEIXE EM FILE FRESCO NAMORADO |
| 08401 | PEIXE SALGADO NAMORADO |
| 08501 | PEIXE EM FILE CONGELADO NAMORADO |
| 09101 | PEIXE INTEIRO PESCADINHA |
| 09102 | PEIXE INTEIRO PESCADINHA TORTINHA |
| 09103 | PEIXE INTEIRO PESCADINHA MALHEIRA |
| 09104 | PEIXE INTEIRO PESCADINHA DO ALTO |
| 09105 | PEIXE INTEIRO PESCADINHA MANTEIGA |
| 09106 | PEIXE INTEIRO PESCADINHA DO ALTO MAR |
| 09201 | PEIXE EM POSTAS PESCADINHA |
| 09301 | PEIXE EM FILE FRESCO PESCADINHA |
| 09401 | PEIXE SALGADO PESCADINHA |
| 09501 | PEIXE EM FILE CONGELADO PESCADINHA |
| 10101 | PEIXE INTEIRO TAINHA |
| 10102 | PEIXE INTEIRO TAINHA VERDADEIRA |
| 10103 | PEIXE INTEIRO TAINHA DE CORSO |
| 10104 | PEIXE INTEIRO TAINHA CURIMA |
| 10105 | PEIXE INTEIRO TAINHA DE ENTRADA |
| 10106 | PEIXE INTEIRO TAINHA DE CORRIDA |
| 10107 | PEIXE INTEIRO TAINHA SECA |
| 10108 | PEIXE INTEIRO CAMBAO |
| 10109 | PEIXE INTEIRO TAINHA OLHO PRETO |
| 10110 | PEIXE INTEIRO CACETAO |
| 10111 | PEIXE INTEIRO TAINHA URICHOCA |
| 10112 | PEIXE INTEIRO TAINHA PAU |
| 10113 | PEIXE INTEIRO TAINHA PITIUBA |
| 10114 | PEIXE INTEIRO QUIRA |
| 10115 | PEIXE INTEIRO CAMBIRA |
| 10116 | PEIXE INTEIRO TAINHA PITIUA |
| 10117 | PEIXE INTEIRO TACUJI |
| 10118 | PEIXE INTEIRO CURIMA |
| 10119 | PEIXE INTEIRO SAJUBA |
| 10120 | PEIXE INTEIRO TAMARATANA |
| 10121 | PEIXE INTEIRO CURUMA |
| 10122 | PEIXE INTEIRO URICHOCA |
| 10123 | PEIXE INTEIRO TAPIARA |
| 10124 | PEIXE INTEIRO VIROTE |
| 10125 | PEIXE INTEIRO SAJUNA |
| 10126 | PEIXE INTEIRO SAUNA |
| 10127 | PEIXE INTEIRO AZEITEIRA |
| 10128 | PEIXE INTEIRO AZEITONA |
| 10129 | PEIXE INTEIRO NEGRAO |
| 10130 | PEIXE INTEIRO TAINHOTA |
| 10131 | PEIXE INTEIRO TANHOTA |
| 10132 | PEIXE INTEIRO URIACICA |
| 10133 | PEIXE INTEIRO TAMATARANA |
| 10201 | PEIXE EM POSTAS TAINHA |
| 10301 | PEIXE EM FILE FRESCO TAINHA |
| 10401 | PEIXE SALGADO TAINHA |
| 10501 | PEIXE EM FILE CONGELADO TAINHA |
| 11101 | PEIXE INTEIRO SARDINHA |
| 11102 | PEIXE INTEIRO SARDINHA LAJE |
| 11103 | PEIXE INTEIRO SARDINHA CASCUDA |
| 11104 | PEIXE INTEIRO SARDINHA NAVALHA |
| 11105 | PEIXE INTEIRO SARDINHA BRANCA |
| 11106 | PEIXE INTEIRO SARDINHA DOURADA |
| 11107 | PEIXE INTEIRO SARDINHA PRATA |
| 11108 | PEIXE INTEIRO MACACO DE MAR |
| 11109 | PEIXE INTEIRO SARDINHA MAROMBA |
| 11110 | PEIXE INTEIRO SARDINHA VERDADEIRA |
| 11111 | PEIXE INTEIRO SARDINHA IMPERIAL |
| 11112 | PEIXE INTEIRO CHARUTO |
| 11113 | PEIXE INTEIRO MACACA DE MAR |
| 11114 | PEIXE INTEIRO CARDOSA |
| 11115 | PEIXE INTEIRO MAROMBA |
| 11116 | PEIXE INTEIRO MANE |
| 11117 | PEIXE INTEIRO CAICARA |
| 11118 | PEIXE INTEIRO MIRIM |
| 11119 | PEIXE INTEIRO MUSSOLINA |
| 11120 | PEIXE INTEIRO ARENGA BRANCA |
| 11121 | PEIXE INTEIRO MACAMBE |
| 11122 | PEIXE INTEIRO MASSAMBE |
| 11201 | PEIXE EM POSTAS SARDINHA |
| 11301 | PEIXE EM FILE FRESCO SARDINHA |
| 11401 | PEIXE SALGADO SARDINHA |
| 11501 | PEIXE EM FILE CONGELADO SARDINHA |
| 11601 | PEIXE DEFUMADO SARDINHA |
| 12101 | PEIXE INTEIRO VERMELHO |
| 12102 | PEIXE INTEIRO ARIACO |
| 12103 | PEIXE INTEIRO ARIOCO |
| 12104 | PEIXE INTEIRO BOCA PRETA |
| 12105 | PEIXE INTEIRO ARICO |
| 12106 | PEIXE INTEIRO VERMELHO DO FUNDO |
| 12107 | PEIXE INTEIRO VERMELHO VERDADEIRO |
| 12108 | PEIXE INTEIRO VERMELHO HENRIQUE |
| 12109 | PEIXE INTEIRO VERMELHO BOCA NEGRA |
| 12110 | PEIXE INTEIRO ARIECO |
| 12201 | PEIXE EM POSTAS VERMELHO |
| 12301 | PEIXE EM FILE FRESCO VERMELHO |
| 12401 | PEIXE SALGADO VERMELHO |
| 12501 | PEIXE EM FILE CONGELADO VERMELHO |
| 13101 | PEIXE INTEIRO CIOBA |
| 13102 | PEIXE INTEIRO CHIOVA |
| 13103 | PEIXE INTEIRO SAUBA |
| 13104 | PEIXE INTEIRO GUAIUBA |
| 13105 | PEIXE INTEIRO CHIOBA |
| 13106 | PEIXE INTEIRO GUAJUBA |
| 13107 | PEIXE INTEIRO GUATUBA |
| 13201 | PEIXE EM POSTAS CIOBA |
| 13301 | PEIXE EM FILE FRESCO CIOBA |
| 13401 | PEIXE SALGADO CIOBA |
| 13501 | PEIXE EM FILE CONGELADO CIOBA |
| 14101 | PEIXE INTEIRO BAGRE DE MAR |
| 14102 | PEIXE INTEIRO BAGRE CANGUITO |
| 14103 | PEIXE INTEIRO IRIDECA DE MAR |
| 14104 | PEIXE INTEIRO BAGRE AMARELO |
| 14105 | PEIXE INTEIRO GUARAJUBA DE MAR |
| 14106 | PEIXE INTEIRO BAGRE BANDEIRA |
| 14107 | PEIXE INTEIRO BAGRE URUTU |
| 14108 | PEIXE INTEIRO BAGRE ARIACU |
| 14109 | PEIXE INTEIRO BAGRE MANDIM DE MAR |
| 14110 | PEIXE INTEIRO PELADO |
| 14111 | PEIXE INTEIRO IRITINGA |
| 14112 | PEIXE INTEIRO BANDEIRADA |
| 14113 | PEIXE INTEIRO SARGENTO |
| 14114 | PEIXE INTEIRO BANDIM |
| 14115 | PEIXE INTEIRO URITINGA |
| 14116 | PEIXE INTEIRO BRAGALHAO |
| 14117 | PEIXE INTEIRO CALAFATE |
| 14118 | PEIXE INTEIRO IRICECA |
| 14119 | PEIXE INTEIRO CUBANGO |
| 14120 | PEIXE INTEIRO IREDECA |
| 14121 | PEIXE INTEIRO BOCA ABERTA |
| 14122 | PEIXE INTEIRO JAGUAR |
| 14123 | PEIXE INTEIRO IRIDECA |
| 14124 | PEIXE INTEIRO JURUPIRANGA |
| 14125 | PEIXE INTEIRO JUJUBA |
| 14126 | PEIXE INTEIRO CANGATA |
| 14127 | PEIXE INTEIRO CATINGUEIRA |
| 14128 | PEIXE INTEIRO CANGATI |
| 14201 | PEIXE EM POSTAS BAGRE DE MAR |
| 14301 | PEIXE EM FILE FRESCO BAGRE DE MAR |
| 14401 | PEIXE SALGADO BAGRE DE MAR |
| 14501 | PEIXE EM FILE CONGELADO BAGRE DE MAR |
| 14901 | PEIXE BAGRE DE MAR ( FORMA DE COMERCIALIZACAO NAO-DISCRIMINADA) |
| 15101 | PEIXE INTEIRO CARAPEBA |
| 15102 | PEIXE INTEIRO CARAPEBA LISTRADA |
| 15103 | PEIXE INTEIRO CARAPEBA BRANCA |
| 15104 | PEIXE INTEIRO CARAPEBA RAJADA |
| 15105 | PEIXE INTEIRO CARATINGA |
| 15106 | PEIXE INTEIRO ACARAPEBA DE MAR |
| 15107 | PEIXE INTEIRO CARAPEVA |
| 15108 | PEIXE INTEIRO ACARAPEVA |
| 15109 | PEIXE INTEIRO MULATINHO |
| 15110 | PEIXE INTEIRO CARA SUJA |
| 15111 | PEIXE INTEIRO MULATINHA |
| 15201 | PEIXE EM POSTAS CARAPEBA |
| 15301 | PEIXE EM FILE FRESCO CARAPEBA |
| 15401 | PEIXE SALGADO CARAPEBA |
| 15501 | PEIXE EM FILE CONGELADO CARAPEBA |
| 16101 | PEIXE INTEIRO CAVALA |
| 16102 | PEIXE INTEIRO CAVALA PINTADA |
| 16103 | PEIXE INTEIRO CAVALA BRANCA |
| 16104 | PEIXE INTEIRO CAVALA PERNA DE MOCA |
| 16105 | PEIXE INTEIRO CAVALA SARDINHEIRA |
| 16106 | PEIXE INTEIRO CAVALA PRETA |
| 16107 | PEIXE INTEIRO CAVALA VERDADEIRA |
| 16108 | PEIXE INTEIRO CAVALA DO REINO |
| 16109 | PEIXE INTEIRO SERRA PIMA |
| 16110 | PEIXE INTEIRO SOROROCA |
| 16201 | PEIXE EM POSTAS CAVALA |
| 16301 | PEIXE EM FILE FRESCO CAVALA |
| 16401 | PEIXE SALGADO CAVALA |
| 16501 | PEIXE EM FILE CONGELADO CAVALA |
| 16901 | PEIXE CAVALA (FORMA DE COMERCIALIZACAO NAO-DISCRIMINADA) |
| 17101 | PEIXE INTEIRO GALO |
| 17102 | PEIXE INTEIRO GALO BANDEIRA |
| 17103 | PEIXE INTEIRO DOUTOR |
| 17104 | PEIXE INTEIRO GALINHO |
| 17105 | PEIXE INTEIRO GALO BRANCO |
| 17106 | PEIXE INTEIRO ARACORANA |
| 17107 | PEIXE INTEIRO GALO PENA |
| 17108 | PEIXE INTEIRO GALO DE PENACHO |
| 17109 | PEIXE INTEIRO FAQUECO |
| 17110 | PEIXE INTEIRO TESTUDO |
| 17111 | PEIXE INTEIRO CAPAO |
| 17112 | PEIXE INTEIRO ALFAQUIM |
| 17113 | PEIXE INTEIRO ZABUCAI |
| 17201 | PEIXE EM POSTAS GALO |
| 17301 | PEIXE EM FILE FRESCO GALO |
| 17401 | PEIXE SALGADO GALO |
| 17501 | PEIXE EM FILE CONGELADO GALO |
| 18101 | PEIXE INTEIRO CORCOROCA |
| 18102 | PEIXE INTEIRO COCOROCA |
| 18103 | PEIXE INTEIRO CORCOROCA BOCA LARGA |
| 18104 | PEIXE INTEIRO COROCOROCA |
| 18105 | PEIXE INTEIRO CORCOROCA BOCA DE FOGO |
| 18106 | PEIXE INTEIRO CORCOROCA MULATA |
| 18107 | PEIXE INTEIRO CORCOROCA JURUMIRIM |
| 18108 | PEIXE INTEIRO BIQUARA |
| 18109 | PEIXE INTEIRO BOCA VELHA |
| 18110 | PEIXE INTEIRO BOCA DE VELHA |
| 18111 | PEIXE INTEIRO SAPURUNA |
| 18112 | PEIXE INTEIRO NEGRA MINA |
| 18113 | PEIXE INTEIRO XIRA |
| 18114 | PEIXE INTEIRO SAPURANA |
| 18115 | PEIXE INTEIRO ABIQUARA |
| 18116 | PEIXE INTEIRO ARREBENTA PANELA |
| 18117 | PEIXE INTEIRO CAMBUBA |
| 18118 | PEIXE INTEIRO CANGUITO |
| 18119 | PEIXE INTEIRO CAMBIMBA |
| 18120 | PEIXE INTEIRO COCORO |
| 18121 | PEIXE INTEIRO CAPIUNA |
| 18122 | PEIXE INTEIRO FAROFA |
| 18123 | PEIXE INTEIRO URIBACO |
| 18124 | PEIXE INTEIRO MACACA |
| 18201 | PEIXE EM POSTAS CORCOROCA |
| 18301 | PEIXE EM FILE FRESCO CORCOROCA |
| 18401 | PEIXE SALGADO CORCOROCA |
| 18501 | PEIXE EM FILE CONGELADO CORCOROCA |
| 19101 | PEIXE INTEIRO MANJUBA |
| 19102 | PEIXE INTEIRO XANGO |
| 19103 | PEIXE INTEIRO PIPITINGA |
| 19104 | PEIXE INTEIRO PITITINGA |
| 19105 | PEIXE INTEIRO MANJUVA |
| 19106 | PEIXE INTEIRO ANCHOITA |
| 19107 | PEIXE INTEIRO GINGA |
| 19201 | PEIXE EM POSTAS MANJUBA |
| 19301 | PEIXE EM FILE FRESCO MANJUBA |
| 19401 | PEIXE SALGADO MANJUBA |
| 19501 | PEIXE EM FILE CONGELADO MANJUBA |
| 20101 | PEIXE INTEIRO PAPATERRA |
| 20102 | PEIXE INTEIRO TAMETARA |
| 20103 | PEIXE INTEIRO SINHARA |
| 20104 | PEIXE INTEIRO TREMETARA |
| 20105 | PEIXE INTEIRO JUDEU |
| 20106 | PEIXE INTEIRO PESCADA CACHORRO (PAPATERRA) |
| 20107 | PEIXE INTEIRO BETARA |
| 20108 | PEIXE INTEIRO TAMBETARA |
| 20109 | PEIXE INTEIRO SAMBETARA |
| 20110 | PEIXE INTEIRO CARAMETARA |
| 20111 | PEIXE INTEIRO CORVINA CACHORRO (PAPATERRA) |
| 20112 | PEIXE INTEIRO IMBETARA |
| 20113 | PEIXE INTEIRO CARAMUTARA |
| 20114 | PEIXE INTEIRO PIRA SIRIRICA |
| 20115 | PEIXE INTEIRO EMBETARA |
| 20116 | PEIXE INTEIRO CHUPA AREIA |
| 20117 | PEIXE INTEIRO TEMBETARA |
| 20201 | PEIXE EM POSTAS PAPATERRA |
| 20301 | PEIXE EM FILE FRESCO PAPATERRA |
| 20401 | PEIXE SALGADO PAPATERRA |
| 20501 | PEIXE EM FILE CONGELADO PAPATERRA |
| 20701 | CABECA DE PEIXE PESCADA |
| 21101 | PEIXE INTEIRO DOURADO DE MAR |
| 21102 | PEIXE INTEIRO DALFINHO |
| 21103 | PEIXE INTEIRO GRASSAPE |
| 21104 | PEIXE INTEIRO MACACO |
| 21105 | PEIXE INTEIRO GUARACAPEMA |
| 21201 | PEIXE EM POSTAS DOURADO DE MAR |
| 21301 | PEIXE EM FILE FRESCO DOURADO DE MAR |
| 21401 | PEIXE SALGADO DOURADO DE MAR |
| 21501 | PEIXE EM FILE CONGELADO DOURADO DE MAR |
| 22101 | PEIXE INTEIRO ATUM |
| 22102 | PEIXE INTEIRO ALBACORA AZUL |
| 22201 | PEIXE EM POSTAS ATUM |
| 22301 | PEIXE EM FILE FRESCO ATUM |
| 22401 | PEIXE SALGADO ATUM |
| 22501 | PEIXE EM FILE CONGELADO ATUM |
| 23101 | PEIXE INTEIRO OLHO DE BOI |
| 23102 | PEIXE INTEIRO PITANGOLA |
| 23103 | PEIXE INTEIRO OLHETE |
| 23104 | PEIXE INTEIRO JURICO |
| 23105 | PEIXE INTEIRO URUBAIANA |
| 23106 | PEIXE INTEIRO ARABAIANA |
| 23107 | PEIXE INTEIRO CANTANTE |
| 23108 | PEIXE INTEIRO TAPIRECA |
| 23109 | PEIXE INTEIRO TAPIRANGA |
| 23201 | PEIXE EM POSTAS OLHO DE BOI |
| 23301 | PEIXE EM FILE FRESCO OLHO DE BOI |
| 23401 | PEIXE SALGADO OLHO DE BOI |
| 23501 | PEIXE EM FILE CONGELADO OLHO DE BOI |
| 24101 | PEIXE INTEIRO SERRA |
| 24102 | PEIXE INTEIRO SARDA |
| 24103 | PEIXE INTEIRO SERRA DE ESCAMA |
| 24104 | PEIXE INTEIRO CHERRO |
| 24201 | PEIXE EM POSTAS SERRA |
| 24301 | PEIXE EM FILE FRESCO SERRA |
| 24401 | PEIXE SALGADO SERRA |
| 24501 | PEIXE EM FILE CONGELADO SERRA |
| 24901 | PEIXE SERRA (FORMA DE COMERCIALIZACAO NAO-DISCRIMINADA) |
| 25101 | PEIXE INTEIRO PARGO |
| 25102 | PEIXE INTEIRO CALUNGA |
| 25103 | PEIXE INTEIRO PAGRO |
| 25104 | PEIXE INTEIRO PARGO OLHO DE VIDRO |
| 25105 | PEIXE INTEIRO PARGO ROSA |
| 25106 | PEIXE INTEIRO PARGO CACHUCHO |
| 25201 | PEIXE EM POSTAS PARGO |
| 25301 | PEIXE EM FILE FRESCO PARGO |
| 25401 | PEIXE SALGADO PARGO |
| 25501 | PEIXE EM FILE CONGELADO PARGO |
| 26101 | PEIXE INTEIRO RONCADOR |
| 26102 | PEIXE INTEIRO CORRO DE MAR |
| 26103 | PEIXE INTEIRO CORO |
| 26104 | PEIXE INTEIRO CARDEIRO |
| 26105 | PEIXE INTEIRO COROQUE |
| 26106 | PEIXE INTEIRO FERREIRO |
| 26107 | PEIXE INTEIRO CORO AMARELO |
| 26108 | PEIXE INTEIRO PARGO BRANCO (RONCADOR) |
| 26201 | PEIXE EM POSTAS RONCADOR |
| 26301 | PEIXE EM FILE FRESCO RONCADOR |
| 26401 | PEIXE SALGADO RONCADOR |
| 26501 | PEIXE EM FILE CONGELADO RONCADOR |
| 27101 | PEIXE INTEIRO PESCADA |
| 27102 | PEIXE INTEIRO PESCADA AMARELA |
| 27103 | PEIXE INTEIRO PIXIMA |
| 27104 | PEIXE INTEIRO PESCADA ARATANHA |
| 27105 | PEIXE INTEIRO PESCADA BRANCA |
| 27106 | PEIXE INTEIRO PESCADA PERNA DE MOCA |
| 27107 | PEIXE INTEIRO PESCADA CAMBUCI |
| 27108 | PEIXE INTEIRO PESCADA GOETE |
| 27109 | PEIXE INTEIRO PESCADA OLHUDA |
| 27110 | PEIXE INTEIRO PESCADA CAMBUCU |
| 27111 | PEIXE INTEIRO PESCADA JAGUARA |
| 27112 | PEIXE INTEIRO PESCADA ESCAMOSA |
| 27113 | PEIXE INTEIRO PESCADA REAL |
| 27114 | PEIXE INTEIRO MALHEIRA |
| 27115 | PEIXE INTEIRO PESCADA ARAUJA |
| 27116 | PEIXE INTEIRO PESCADA MARIA MOLE |
| 27117 | PEIXE INTEIRO PESCADA GALHETEIRA |
| 27118 | PEIXE INTEIRO PESCADA DO REINO |
| 27119 | PEIXE INTEIRO PESCADA GO |
| 27120 | PEIXE INTEIRO PESCADA LEGITIMA |
| 27121 | PEIXE INTEIRO PESCADA DE DENTE |
| 27122 | PEIXE INTEIRO PESCADA CUTUPANHA |
| 27123 | PEIXE INTEIRO PESCADA TRAMITARA |
| 27124 | PEIXE INTEIRO PESCADA PRETA |
| 27125 | PEIXE INTEIRO PESCADA CABECA DURA |
| 27126 | PEIXE INTEIRO PESCADA CORVINA |
| 27127 | PEIXE INTEIRO ARANJA |
| 27128 | PEIXE INTEIRO PESCADA COBRA |
| 27129 | PEIXE INTEIRO PESCADA SELVAGEM |
| 27130 | PEIXE INTEIRO PESCADA BANANA |
| 27131 | PEIXE INTEIRO MARIA MOLE |
| 27132 | PEIXE INTEIRO PESCADA FOGUETE |
| 27133 | PEIXE INTEIRO PESCADA DENTUCA |
| 27134 | PEIXE INTEIRO PESCADA DE ALTO |
| 27135 | PEIXE INTEIRO BOCA MOLE |
| 27136 | PEIXE INTEIRO RABO SECO |
| 27137 | PEIXE INTEIRO PESCADA DE ALTO MAR |
| 27138 | PEIXE INTEIRO COACA |
| 27139 | PEIXE INTEIRO GOETE |
| 27140 | PEIXE INTEIRO TICOPA |
| 27141 | PEIXE INTEIRO BORORO |
| 27142 | PEIXE INTEIRO GUETE |
| 27143 | PEIXE INTEIRO GORETE |
| 27144 | PEIXE INTEIRO PERNA DE MOCA |
| 27145 | PEIXE INTEIRO CALAFETAO |
| 27146 | PEIXE INTEIRO PIRAMBEBE |
| 27147 | PEIXE INTEIRO MEMBECA |
| 27148 | PEIXE INTEIRO GALHETEIRA |
| 27149 | PEIXE INTEIRO GO |
| 27201 | PEIXE EM POSTAS PESCADA |
| 27301 | PEIXE EM FILE FRESCO PESCADA |
| 27401 | PEIXE SALGADO PESCADA |
| 27501 | PEIXE EM FILE CONGELADO PESCADA |
| 27901 | PEIXE PESCADA (FORMA DE COMERCIALIZACAO NAO-DISCRIMINADA) |
| 27902 | PEIXE PESCADA AMARELA (FORMA DE COMERCIALIZACAO NAO-DISCRIMINADA) |
| 27903 | PEIXE PESCADA BRANCA (FORMA DE COMERCIALIZACAO NAO-DISCRIMINADA) |
| 28101 | PEIXE INTEIRO GAROUPA |
| 28102 | PEIXE INTEIRO GAROUPA VERDADEIRA |
| 28103 | PEIXE INTEIRO GAROUPA SAO TOME |
| 28104 | PEIXE INTEIRO GAROUPA CRIOULA |
| 28105 | PEIXE INTEIRO GAROUPA PRETA |
| 28106 | PEIXE INTEIRO GAROUPA GATO |
| 28107 | PEIXE INTEIRO GAROUPA SENHOR DE ENGENHO |
| 28108 | PEIXE INTEIRO GAROUPA DE SEGUNDA |
| 28109 | PEIXE INTEIRO GAROUPA CHITA |
| 28110 | PEIXE INTEIRO GAROUPA BICHADA |
| 28111 | PEIXE INTEIRO GAROUPINHA |
| 28112 | PEIXE INTEIRO SAPA |
| 28113 | PEIXE INTEIRO SULAPEBA |
| 28114 | PEIXE INTEIRO SIRIGAITA |
| 28115 | PEIXE INTEIRO PIRAJIA |
| 28116 | PEIXE INTEIRO PIRACUCA |
| 28117 | PEIXE INTEIRO SERIGADO MERO (GAROUPA) |
| 28118 | PEIXE INTEIRO SERIGADO FOCINHUDO (GAROUPA) |
| 28119 | PEIXE INTEIRO SERIGADO VERMELHO (GAROUPA) |
| 28201 | PEIXE EM POSTAS GAROUPA |
| 28301 | PEIXE EM FILE FRESCO GAROUPA |
| 28401 | PEIXE SALGADO GAROUPA |
| 28501 | PEIXE EM FILE CONGELADO GAROUPA |
| 29101 | PEIXE INTEIRO LINGUADO |
| 29102 | PEIXE INTEIRO LINGUADO DA AREIA |
| 29103 | PEIXE INTEIRO SOLHA |
| 29104 | PEIXE INTEIRO SOIA |
| 29105 | PEIXE INTEIRO ARAMACA |
| 29106 | PEIXE INTEIRO ARAMATA |
| 29107 | PEIXE INTEIRO ARUMATA |
| 29108 | PEIXE INTEIRO ARUMACA |
| 29109 | PEIXE INTEIRO RODOVALHO |
| 29110 | PEIXE INTEIRO CATRAIO |
| 29111 | PEIXE INTEIRO ARAMACACA |
| 29112 | PEIXE INTEIRO LINGUA |
| 29201 | PEIXE EM POSTAS LINGUADO |
| 29301 | PEIXE EM FILE FRESCO LINGUADO |
| 29401 | PEIXE SALGADO LINGUADO |
| 29501 | PEIXE EM FILE CONGELADO LINGUADO |
| 30101 | PEIXE INTEIRO ROBALO |
| 30102 | PEIXE INTEIRO CAMURIM BRANCO |
| 30103 | PEIXE INTEIRO CAMURIM APUA |
| 30104 | PEIXE INTEIRO CAMURIM SOVELA |
| 30105 | PEIXE INTEIRO ROBALO PEBA |
| 30106 | PEIXE INTEIRO CAMURIM ACU |
| 30107 | PEIXE INTEIRO CAMURIMPEBA |
| 30108 | PEIXE INTEIRO ROBALO BICUDO |
| 30109 | PEIXE INTEIRO ROBALAO |
| 30110 | PEIXE INTEIRO ROBALETE |
| 30111 | PEIXE INTEIRO ROBALO FLECHA |
| 30112 | PEIXE INTEIRO CAMURI |
| 30113 | PEIXE INTEIRO CAMURIPEBA |
| 30114 | PEIXE INTEIRO CORCUNDA |
| 30115 | PEIXE INTEIRO CORCUNDO |
| 30116 | PEIXE INTEIRO CAMORIM |
| 30117 | PEIXE INTEIRO CAMBRIACU |
| 30118 | PEIXE INTEIRO CONGOROPEBA |
| 30119 | PEIXE INTEIRO FURAO |
| 30120 | PEIXE INTEIRO ESCALHO |
| 30121 | PEIXE INTEIRO ROBAO |
| 30122 | PEIXE INTEIRO BICUDO |
| 30201 | PEIXE EM POSTAS ROBALO |
| 30301 | PEIXE EM FILE FRESCO ROBALO |
| 30401 | PEIXE SALGADO ROBALO |
| 30501 | PEIXE EM FILE CONGELADO ROBALO |
| 31101 | PEIXE INTEIRO MERO |
| 31102 | PEIXE INTEIRO CANAPUACU |
| 31103 | PEIXE INTEIRO MERO CANAPU |
| 31104 | PEIXE INTEIRO CANAPUM |
| 31105 | PEIXE INTEIRO CANAPU |
| 31106 | PEIXE INTEIRO MERO CANAPUM |
| 31201 | PEIXE EM POSTAS MERO |
| 31301 | PEIXE EM FILE FRESCO MERO |
| 31401 | PEIXE SALGADO MERO |
| 31501 | PEIXE EM FILE CONGELADO MERO |
| 32101 | PEIXE INTEIRO XERELETE |
| 32102 | PEIXE INTEIRO CAVACO |
| 32103 | PEIXE INTEIRO FLAMINGUETE |
| 32104 | PEIXE INTEIRO XERERETE |
| 32105 | PEIXE INTEIRO XARELETE |
| 32106 | PEIXE INTEIRO CHUMBERGA |
| 32107 | PEIXE INTEIRO FRAMIGUETA |
| 32108 | PEIXE INTEIRO XAREU DOURADO (XERELETE) |
| 32109 | PEIXE INTEIRO FRAMIGUETO |
| 32110 | PEIXE INTEIRO XAREU PEQUENO (XERELETE) |
| 32111 | PEIXE INTEIRO GUARECIMA |
| 32112 | PEIXE INTEIRO GUARICEMA |
| 32201 | PEIXE EM POSTAS XERELETE |
| 32301 | PEIXE EM FILE FRESCO XERELETE |
| 32401 | PEIXE SALGADO XERELETE |
| 32501 | PEIXE EM FILE CONGELADO XERELETE |
| 33101 | PEIXE INTEIRO ESPADA |
| 33102 | PEIXE INTEIRO IMBIRA |
| 33103 | PEIXE INTEIRO EMBIRA |
| 33104 | PEIXE INTEIRO CATANA |
| 33201 | PEIXE EM POSTAS ESPADA |
| 33301 | PEIXE EM FILE FRESCO ESPADA |
| 33401 | PEIXE SALGADO ESPADA |
| 33501 | PEIXE EM FILE CONGELADO ESPADA |
| 34101 | PEIXE INTEIRO PARATI |
| 34102 | PEIXE INTEIRO PARATI OLHO DE FOGO |
| 34103 | PEIXE INTEIRO PRATIQUEIRA |
| 34104 | PEIXE INTEIRO SOLE |
| 34105 | PEIXE INTEIRO CAICA |
| 34106 | PEIXE INTEIRO PARATICU |
| 34107 | PEIXE INTEIRO MONDEGO |
| 34108 | PEIXE INTEIRO TAINHA DE OLHO AMARELO (PARATI) |
| 34109 | PEIXE INTEIRO TAINHA CHATA (PARATI) |
| 34110 | PEIXE INTEIRO PIRATIQUEIRA |
| 34111 | PEIXE INTEIRO PRATIBU |
| 34201 | PEIXE EM POSTAS PARATI |
| 34301 | PEIXE EM FILE FRESCO PARATI |
| 34401 | PEIXE SALGADO PARATI |
| 34501 | PEIXE EM FILE CONGELADO PARATI |
| 35101 | PEIXE INTEIRO CACAO |
| 35102 | PEIXE INTEIRO CACAO PENA |
| 35103 | PEIXE INTEIRO CORNUDA |
| 35104 | PEIXE INTEIRO CACAO FIUZO |
| 35105 | PEIXE INTEIRO CACAO RAPOSA |
| 35106 | PEIXE INTEIRO CACAO SEBASTIAO |
| 35107 | PEIXE INTEIRO CACAO ANJO |
| 35108 | PEIXE INTEIRO CACAO TORRADOR |
| 35109 | PEIXE INTEIRO CACAO GALHUDO |
| 35110 | PEIXE INTEIRO CACAO PATA |
| 35111 | PEIXE INTEIRO CACAO CAMBEBA |
| 35112 | PEIXE INTEIRO CACAO ALECRIM |
| 35113 | PEIXE INTEIRO CACAO FRANGO |
| 35114 | PEIXE INTEIRO CACAO PANA |
| 35115 | PEIXE INTEIRO CACAO MARTELO |
| 35116 | PEIXE INTEIRO CACAO RODELA |
| 35117 | PEIXE INTEIRO CACAO CHAPEU |
| 35118 | PEIXE INTEIRO CACAO GATA |
| 35119 | PEIXE INTEIRO CACAO PINTO |
| 35120 | PEIXE INTEIRO CACAO MANGONGA |
| 35121 | PEIXE INTEIRO CACAO LIXA |
| 35122 | PEIXE INTEIRO CACAO DE AREIA |
| 35123 | PEIXE INTEIRO CANGA |
| 35124 | PEIXE INTEIRO CACAO DE ESPINHO |
| 35125 | PEIXE INTEIRO CACAO BAGRE |
| 35126 | PEIXE INTEIRO BARROSO |
| 35127 | PEIXE INTEIRO MARTELO |
| 35128 | PEIXE INTEIRO CACAO MAGONGA |
| 35129 | PEIXE INTEIRO CACAO MANGONA |
| 35130 | PEIXE INTEIRO GATA (CACAO) |
| 35131 | PEIXE INTEIRO PATA |
| 35132 | PEIXE INTEIRO MARRAXO |
| 35133 | PEIXE INTEIRO PINTO |
| 35134 | PEIXE INTEIRO ANJO |
| 35135 | PEIXE INTEIRO CUCURI |
| 35136 | PEIXE INTEIRO BODINHO |
| 35137 | PEIXE INTEIRO FRANGO |
| 35138 | PEIXE INTEIRO JOAO DIAS |
| 35139 | PEIXE INTEIRO TOLO |
| 35140 | PEIXE INTEIRO CACONETE |
| 35141 | PEIXE INTEIRO SEBASTIAO |
| 35142 | PEIXE INTEIRO MANGONA |
| 35201 | PEIXE EM POSTAS CACAO |
| 35301 | PEIXE EM FILE FRESCO CACAO |
| 35401 | PEIXE SALGADO CACAO |
| 35501 | PEIXE EM FILE CONGELADO CACAO |
| 36101 | PEIXE INTEIRO MERLUZA |
| 36102 | PEIXE INTEIRO MELOSO |
| 36103 | PEIXE INTEIRO PESCADA PORTUGUESA (MERLUZA) |
| 36201 | PEIXE EM POSTAS MERLUZA |
| 36301 | PEIXE EM FILE FRESCO MERLUZA |
| 36401 | PEIXE SALGADO MERLUZA |
| 36501 | PEIXE EM FILE CONGELADO MERLUZA |
| 37101 | PEIXE INTEIRO BEIJUPIRA |
| 37102 | PEIXE INTEIRO BEIUPIRA |
| 37103 | PEIXE INTEIRO BEJUPIRA |
| 37104 | PEIXE INTEIRO BIJUPIRA |
| 37105 | PEIXE INTEIRO BEIJOPIRA |
| 37106 | PEIXE INTEIRO CANADO |
| 37107 | PEIXE INTEIRO REI DOS PEIXES (BEIJUPIRA) |
| 37108 | PEIXE INTEIRO CACAO DE ESCAMAS (BEIJUPIRA) |
| 37109 | PEIXE INTEIRO PIRABIJU |
| 37110 | PEIXE INTEIRO PIRABEIJU |
| 37111 | PEIXE INTEIRO PIRAMBIJU |
| 37112 | PEIXE INTEIRO PARABEIJU |
| 37113 | PEIXE INTEIRO TORANDIJU |
| 37114 | PEIXE INTEIRO PARANDIJU |
| 37115 | PEIXE INTEIRO PARAMBEJU |
| 37116 | PEIXE INTEIRO BUJUPIRA |
| 37201 | PEIXE EM POSTAS BEIJUPIRA |
| 37301 | PEIXE EM FILE FRESCO BEIJUPIRA |
| 37401 | PEIXE SALGADO BEIJUPIRA |
| 37501 | PEIXE EM FILE CONGELADO BEIJUPIRA |
| 38101 | PEIXE INTEIRO PAMPO |
| 38102 | PEIXE INTEIRO PAMPO CABECA MOLE |
| 38103 | PEIXE INTEIRO PAMPO VERMELHO |
| 38104 | PEIXE INTEIRO PAMPO LISTRADO |
| 38105 | PEIXE INTEIRO PAMPO MACHO |
| 38106 | PEIXE INTEIRO PAMPO SOLTEIRO |
| 38107 | PEIXE INTEIRO PAMPO DO ALTO |
| 38108 | PEIXE INTEIRO PAMPO GARABEBEL |
| 38109 | PEIXE INTEIRO PAMPO SARGENTO |
| 38110 | PEIXE INTEIRO PAMPO GIGANTE |
| 38111 | PEIXE INTEIRO PAMPO ARABEBEL |
| 38112 | PEIXE INTEIRO PAMPO GALHUDO |
| 38113 | PEIXE INTEIRO PAMPO ESPINHA MOLE |
| 38114 | PEIXE INTEIRO PAMPO ARATUBAIA |
| 38115 | PEIXE INTEIRO PAMPO RISCADO |
| 38116 | PEIXE INTEIRO PAROLA |
| 38117 | PEIXE INTEIRO ARABEBEL |
| 38118 | PEIXE INTEIRO TABUA |
| 38119 | PEIXE INTEIRO SEMENDUARA |
| 38120 | PEIXE INTEIRO PIRAROBA |
| 38121 | PEIXE INTEIRO PAMPLO |
| 38122 | PEIXE INTEIRO JIRIQUITI |
| 38123 | PEIXE INTEIRO PAMPANO |
| 38124 | PEIXE INTEIRO GARABEBEL |
| 38125 | PEIXE INTEIRO CABARARI |
| 38126 | PEIXE INTEIRO ARATOBAIA |
| 38127 | PEIXE INTEIRO ARACANGUITO |
| 38128 | PEIXE INTEIRO ARATUBAIA |
| 38201 | PEIXE EM POSTAS PAMPO |
| 38301 | PEIXE EM FILE FRESCO PAMPO |
| 38401 | PEIXE SALGADO PAMPO |
| 38501 | PEIXE EM FILE CONGELADO PAMPO |
| 39101 | PEIXE INTEIRO XAREU |
| 39102 | PEIXE INTEIRO XAREO |
| 39103 | PEIXE INTEIRO XAREU VERDADEIRO |
| 39104 | PEIXE INTEIRO XAREU VAQUEIRO |
| 39105 | PEIXE INTEIRO XAREU RONCADOR |
| 39106 | PEIXE INTEIRO XAREU BRANCO |
| 39107 | PEIXE INTEIRO CARIMBAMBA |
| 39108 | PEIXE INTEIRO GRACAIM |
| 39109 | PEIXE INTEIRO CABECUDO |
| 39110 | PEIXE INTEIRO XAREU PRETO |
| 39111 | PEIXE INTEIRO CORIMBAMBA |
| 39112 | PEIXE INTEIRO GUACAINHA |
| 39113 | PEIXE INTEIRO GUARACEMA |
| 39114 | PEIXE INTEIRO ARACAROBA |
| 39201 | PEIXE EM POSTAS XAREU |
| 39301 | PEIXE EM FILE FRESCO XAREU |
| 39401 | PEIXE SALGADO XAREU |
| 39501 | PEIXE EM FILE CONGELADO XAREU |
| 39901 | PEIXE XAREU (FORMA DE COMERCIALIZACAO NAO-DISCRIMINADA) |
| 40101 | PEIXE INTEIRO CARAMURU |
| 40102 | PEIXE INTEIRO CRAMURU |
| 40103 | PEIXE INTEIRO MIRORO |
| 40104 | PEIXE INTEIRO MOREIA VERDE (CARAMURU) |
| 40105 | PEIXE INTEIRO ENGUIA (CARAMURU) |
| 40106 | PEIXE INTEIRO MORORO |
| 40107 | PEIXE INTEIRO TORORO |
| 40201 | PEIXE EM POSTAS CARAMURU |
| 40301 | PEIXE EM FILE FRESCO CARAMURU |
| 40401 | PEIXE SALGADO CARAMURU |
| 40501 | PEIXE EM FILE CONGELADO CARAMURU |
| 41101 | PEIXE INTEIRO CANGULO |
| 41102 | PEIXE INTEIRO PEROA BRANCO |
| 41103 | PEIXE INTEIRO ESFALDADO |
| 41104 | PEIXE INTEIRO PEROA PRETO |
| 41105 | PEIXE INTEIRO PEROA |
| 41106 | PEIXE INTEIRO PEROATINGA |
| 41107 | PEIXE INTEIRO GUDUNHO |
| 41108 | PEIXE INTEIRO FANTASMA |
| 41109 | PEIXE INTEIRO CAPADO |
| 41110 | PEIXE INTEIRO ACARAMUCU (CANGULO) |
| 41111 | PEIXE INTEIRO CANGULO REI |
| 41112 | PEIXE INTEIRO PORCO |
| 41113 | PEIXE INTEIRO CANGULO BRANCO |
| 41114 | PEIXE INTEIRO CANGULO DE FERNANDO |
| 41115 | PEIXE INTEIRO ACARAMOCO (CANGULO) |
| 41116 | PEIXE INTEIRO CANGULO PRETO |
| 41117 | PEIXE INTEIRO CANGURRO |
| 41118 | PEIXE INTEIRO CANGULO DO ALTO |
| 41119 | PEIXE INTEIRO PORQUINHO |
| 41201 | PEIXE EM POSTAS CANGULO |
| 41301 | PEIXE EM FILE FRESCO CANGULO |
| 41401 | PEIXE SALGADO CANGULO |
| 41501 | PEIXE EM FILE CONGELADO CANGULO |
| 42101 | PEIXE INTEIRO CAMURUPIM |
| 42102 | PEIXE INTEIRO CAMARUPIM |
| 42103 | PEIXE INTEIRO PIRAPEMA |
| 42104 | PEIXE INTEIRO CAMARIPIM |
| 42105 | PEIXE INTEIRO CANJURUPI |
| 42106 | PEIXE INTEIRO CAMURIPIM |
| 42107 | PEIXE INTEIRO CAMURUPI |
| 42108 | PEIXE INTEIRO CAMURIPEMA |
| 42109 | PEIXE INTEIRO CANGURUPI |
| 42110 | PEIXE INTEIRO CANGURUPIM |
| 42111 | PEIXE INTEIRO CANJURUPIM |
| 42112 | PEIXE INTEIRO PARAPEMA |
| 42113 | PEIXE INTEIRO PEMA |
| 42114 | PEIXE INTEIRO MARIQUITA |
| 42115 | PEIXE INTEIRO PERAPEMA |
| 42116 | PEIXE INTEIRO TARPAO |
| 42117 | PEIXE INTEIRO LARGA ESCAMA |
| 42118 | PEIXE INTEIRO POMBOCA |
| 42119 | PEIXE INTEIRO JACUNDA DE RIO (CAMURUPIM) |
| 42120 | PEIXE INTEIRO CUMURUPIM |
| 42201 | PEIXE EM POSTAS CAMURUPIM |
| 42301 | PEIXE EM FILE FRESCO CAMURUPIM |
| 42401 | PEIXE SALGADO CAMURUPIM |
| 42501 | PEIXE EM FILE CONGELADO CAMURUPIM |
| 43101 | PEIXE INTEIRO GUARAJUBA |
| 43102 | PEIXE INTEIRO GUARAMBA |
| 43103 | PEIXE INTEIRO GUARAIUBA |
| 43104 | PEIXE INTEIRO GARAJUBA |
| 43105 | PEIXE INTEIRO GURIJUBA |
| 43106 | PEIXE INTEIRO URICECA |
| 43201 | PEIXE EM POSTAS GUARAJUBA |
| 43301 | PEIXE EM FILE FRESCO GUARAJUBA |
| 43401 | PEIXE SALGADO GUARAJUBA |
| 43501 | PEIXE EM FILE CONGELADO GUARAJUBA |
| 44101 | PEIXE INTEIRO AGULHA |
| 44102 | PEIXE INTEIRO AGULHA PRETA |
| 44103 | PEIXE INTEIRO AGULHA CRIOULA |
| 44104 | PEIXE INTEIRO FARNANGALHO |
| 44105 | PEIXE INTEIRO CARAPIA |
| 44106 | PEIXE INTEIRO AGULHA BRANCA |
| 44107 | PEIXE INTEIRO FARNANGAIO |
| 44108 | PEIXE INTEIRO TARANGAIO |
| 44109 | PEIXE INTEIRO TARANGALHO |
| 44201 | PEIXE EM POSTAS AGULHA |
| 44301 | PEIXE EM FILE FRESCO AGULHA |
| 44401 | PEIXE SALGADO AGULHA |
| 44501 | PEIXE EM FILE CONGELADO AGULHA |
| 45101 | PEIXE INTEIRO UBARANA |
| 45102 | PEIXE INTEIRO OBARANA |
| 45103 | PEIXE INTEIRO UBARANA RATO |
| 45104 | PEIXE INTEIRO ALBARANA |
| 45105 | PEIXE INTEIRO UBARANA ROLICA |
| 45106 | PEIXE INTEIRO UBARANA MIRIM |
| 45107 | PEIXE INTEIRO URUBARANA |
| 45108 | PEIXE INTEIRO OBARANACU |
| 45109 | PEIXE INTEIRO UBARANACU |
| 45110 | PEIXE INTEIRO TIJUBARANA |
| 45111 | PEIXE INTEIRO RATO |
| 45201 | PEIXE EM POSTAS UBARANA |
| 45301 | PEIXE EM FILE FRESCO UBARANA |
| 45401 | PEIXE SALGADO UBARANA |
| 45501 | PEIXE EM FILE CONGELADO UBARANA |
| 46101 | PEIXE INTEIRO GUAIVIRA |
| 46102 | PEIXE INTEIRO GUAIBIRA |
| 46103 | PEIXE INTEIRO TIBIRO |
| 46104 | PEIXE INTEIRO TIMBIRA |
| 46105 | PEIXE INTEIRO GUAJUVIRA |
| 46106 | PEIXE INTEIRO GOIVIRA |
| 46107 | PEIXE INTEIRO GUARAVIRA |
| 46108 | PEIXE INTEIRO PAMPARRONA |
| 46109 | PEIXE INTEIRO TIBIRIO |
| 46110 | PEIXE INTEIRO TIMBIRO |
| 46201 | PEIXE EM POSTAS GUAIVIRA |
| 46301 | PEIXE EM FILE FRESCO GUAIVIRA |
| 46401 | PEIXE SALGADO GUAIVIRA |
| 46501 | PEIXE EM FILE CONGELADO GUAIVIRA |
| 47101 | PEIXE INTEIRO BICUDA |
| 47102 | PEIXE INTEIRO BICUDA DE CORSO |
| 47103 | PEIXE INTEIRO MILONGO (BICUDA) DE MAR |
| 47104 | PEIXE INTEIRO BICUDA BRANCA |
| 47105 | PEIXE INTEIRO BICUDA CACHORRO |
| 47106 | PEIXE INTEIRO COROMA |
| 47107 | PEIXE INTEIRO BARRACUDA |
| 47108 | PEIXE INTEIRO BACUDA |
| 47109 | PEIXE INTEIRO MILONGA |
| 47201 | PEIXE EM POSTAS BICUDA |
| 47301 | PEIXE EM FILE FRESCO BICUDA |
| 47401 | PEIXE SALGADO BICUDA |
| 47501 | PEIXE EM FILE CONGELADO BICUDA |
| 48101 | PEIXE INTEIRO TRILHA |
| 48201 | PEIXE EM POSTAS TRILHA |
| 48301 | PEIXE EM FILE FRESCO TRILHA |
| 48401 | PEIXE SALGADO TRILHA |
| 48501 | PEIXE EM FILE CONGELADO TRILHA |
| 49101 | PEIXE INTEIRO BONITO |
| 49102 | PEIXE INTEIRO BONITO PINTADO |
| 49103 | PEIXE INTEIRO BONITO PEQUENO |
| 49104 | PEIXE INTEIRO BONITO CADELA |
| 49105 | PEIXE INTEIRO BONITO CARIJO |
| 49106 | PEIXE INTEIRO BONITO BARRIGA RISCADA |
| 49107 | PEIXE INTEIRO BONITO GRANDE |
| 49108 | PEIXE INTEIRO BONITO CACHORRO |
| 49201 | PEIXE EM POSTAS BONITO |
| 49301 | PEIXE EM FILE FRESCO BONITO |
| 49401 | PEIXE SALGADO BONITO |
| 49501 | PEIXE EM FILE CONGELADO BONITO |
| 50101 | PEIXE INTEIRO SAVELHA |
| 50102 | PEIXE INTEIRO SARDELA |
| 50103 | PEIXE INTEIRO COITADINHO |
| 50104 | PEIXE INTEIRO LEVANTA SAIA |
| 50105 | PEIXE INTEIRO SARAVELHA |
| 50106 | PEIXE INTEIRO SARDINHA SAVELHA (SAVELHA) |
| 50201 | PEIXE EM POSTAS SAVELHA |
| 50301 | PEIXE EM FILE FRESCO SAVELHA |
| 50401 | PEIXE SALGADO SAVELHA |
| 50501 | PEIXE EM FILE CONGELADO SAVELHA |
| 51101 | PEIXE INTEIRO BODIAO |
| 51102 | PEIXE INTEIRO PAPAGAIO |
| 51103 | PEIXE INTEIRO BODIAO BATATA |
| 51104 | PEIXE INTEIRO GUDIAO |
| 51105 | PEIXE INTEIRO BODIAO TUCANO |
| 51106 | PEIXE INTEIRO GODIAO |
| 51107 | PEIXE INTEIRO BODIAO VERMELHO |
| 51108 | PEIXE INTEIRO BODIAO PAPAGAIO |
| 51109 | PEIXE INTEIRO BUDIAO |
| 51110 | PEIXE INTEIRO BODIAO SABONETE |
| 51201 | PEIXE EM POSTAS BODIAO |
| 51301 | PEIXE EM FILE FRESCO BODIAO |
| 51401 | PEIXE SALGADO BODIAO |
| 51501 | PEIXE EM FILE CONGELADO BODIAO |
| 52101 | PEIXE INTEIRO PALOMBETA |
| 52102 | PEIXE INTEIRO PALOMETA |
| 52103 | PEIXE INTEIRO PILOMBETA |
| 52104 | PEIXE INTEIRO VENTO LESTE |
| 52105 | PEIXE INTEIRO JUVA |
| 52106 | PEIXE INTEIRO FOLHA DE MANGUE |
| 52107 | PEIXE INTEIRO FAVOLETA |
| 52108 | PEIXE INTEIRO FAVINHA |
| 52109 | PEIXE INTEIRO CAICO |
| 52201 | PEIXE EM POSTAS PALOMBETA |
| 52301 | PEIXE EM FILE FRESCO PALOMBETA |
| 52401 | PEIXE SALGADO PALOMBETA |
| 52501 | PEIXE EM FILE CONGELADO PALOMBETA |
| 53101 | PEIXE INTEIRO MIRAGAIA |
| 53102 | PEIXE INTEIRO PIRAUVA |
| 53103 | PEIXE INTEIRO MIRAGUAIA |
| 53104 | PEIXE INTEIRO PERUMBEBA |
| 53105 | PEIXE INTEIRO GORRETAO |
| 53106 | PEIXE INTEIRO PEROMBEBE |
| 53107 | PEIXE INTEIRO QUINDUNDE |
| 53108 | PEIXE INTEIRO VACA |
| 53109 | PEIXE INTEIRO PIRAUNA |
| 53201 | PEIXE EM POSTAS MIRAGAIA |
| 53301 | PEIXE EM FILE FRESCO MIRAGAIA |
| 53401 | PEIXE SALGADO MIRAGAIA |
| 53501 | PEIXE EM FILE CONGELADO MIRAGAIA |
| 54101 | PEIXE INTEIRO REI (PEIXE REI) |
| 54201 | PEIXE EM POSTAS REI (PEIXE REI) |
| 54301 | PEIXE EM FILE FRESCO REI (PEIXE REI) |
| 54401 | PEIXE SALGADO REI (PEIXE REI) |
| 54501 | PEIXE EM FILE CONGELADO REI (PEIXE REI) |
| 55101 | PEIXE INTEIRO MOREIA |
| 55102 | PEIXE INTEIRO MOREIA PINTADA |
| 55103 | PEIXE INTEIRO MOREIA AMARELA |
| 55104 | PEIXE INTEIRO MUTUTUCA |
| 55105 | PEIXE INTEIRO MUTUCA |
| 55201 | PEIXE EM POSTAS MOREIA |
| 55301 | PEIXE EM FILE FRESCO MOREIA |
| 55401 | PEIXE SALGADO MOREIA |
| 55501 | PEIXE EM FILE CONGELADO MOREIA |
| 56101 | PEIXE INTEIRO ALBACORA |
| 56102 | PEIXE INTEIRO ALBACORA LAJE |
| 56103 | PEIXE INTEIRO ALBACORA MIRIM |
| 56104 | PEIXE INTEIRO ALBACORA BRANCA |
| 56105 | PEIXE INTEIRO ALBACORA LAJEIRA |
| 56106 | PEIXE INTEIRO ALBACORA BANDOLIM |
| 56107 | PEIXE INTEIRO ALBACORINHA |
| 56108 | PEIXE INTEIRO ATUM BRANCO (ALBACORA) |
| 56109 | PEIXE INTEIRO ALVACORA |
| 56110 | PEIXE INTEIRO ALBACORA OLHAO |
| 56111 | PEIXE INTEIRO BANDOLIM |
| 56201 | PEIXE EM POSTAS ALBACORA |
| 56301 | PEIXE EM FILE FRESCO ALBACORA |
| 56401 | PEIXE SALGADO ALBACORA |
| 56501 | PEIXE EM FILE CONGELADO ALBACORA |
| 57101 | PEIXE INTEIRO CHERNE |
| 57102 | PEIXE INTEIRO CHERNE PRETO |
| 57103 | PEIXE INTEIRO CHERNETE |
| 57104 | PEIXE INTEIRO CHERNE PINTADO |
| 57105 | PEIXE INTEIRO CHERNOTE |
| 57106 | PEIXE INTEIRO MERO QUEIMADO (CHERNE) |
| 57107 | PEIXE INTEIRO CHERNA |
| 57108 | PEIXE INTEIRO MERO PRETO (CHERNE) |
| 57109 | PEIXE INTEIRO SERIGADO TAPOA (CHERNE) |
| 57110 | PEIXE INTEIRO SERIGADO CHERNE (CHERNE) |
| 57201 | PEIXE EM POSTAS CHERNE |
| 57301 | PEIXE EM FILE FRESCO CHERNE |
| 57401 | PEIXE SALGADO CHERNE |
| 57501 | PEIXE EM FILE CONGELADO CHERNE |
| 58101 | PEIXE INTEIRO AGULHAO |
| 58102 | PEIXE INTEIRO AGULHAO BANDEIRA |
| 58103 | PEIXE INTEIRO AGULHAO DE VELA |
| 58104 | PEIXE INTEIRO GUEBUCU |
| 58105 | PEIXE INTEIRO MARLIM |
| 58106 | PEIXE INTEIRO AGULHAO PRETO |
| 58107 | PEIXE INTEIRO DE VELA |
| 58108 | PEIXE INTEIRO GUEBO |
| 58109 | PEIXE INTEIRO ESPADARTE MECA |
| 58110 | PEIXE INTEIRO ESPADARTE |
| 58111 | PEIXE INTEIRO AGULHAO BRANCO |
| 58112 | PEIXE INTEIRO AGULHAO VELA |
| 58201 | PEIXE EM POSTAS AGULHAO |
| 58301 | PEIXE EM FILE FRESCO AGULHAO |
| 58401 | PEIXE SALGADO AGULHAO |
| 58501 | PEIXE EM FILE CONGELADO AGULHAO |
| 59101 | PEIXE INTEIRO SALMONETE |
| 59102 | PEIXE INTEIRO SALMAO PEQUENO (SALMONETE) |
| 59103 | PEIXE INTEIRO SARAMONETE |
| 59104 | PEIXE INTEIRO SALMONEJO |
| 59105 | PEIXE INTEIRO PIRAMETARA |
| 59106 | PEIXE INTEIRO SABONETE |
| 59107 | PEIXE INTEIRO SARAMUNETE |
| 59201 | PEIXE EM POSTAS SALMONETE |
| 59301 | PEIXE EM FILE FRESCO SALMONETE |
| 59401 | PEIXE SALGADO SALMONETE |
| 59501 | PEIXE EM FILE CONGELADO SALMONETE |
| 60101 | CAMARAO |
| 60102 | CAMARAO CABOCLO |
| 60103 | CAMARAO VERMELHO |
| 60104 | CAMARAO SERRINHA |
| 60105 | CAMARAO LEGITIMO |
| 60106 | CAMARAO ROSA |
| 60107 | CAMARAO POTITINGA |
| 60108 | POTITINGA |
| 60109 | CAMARAO LIXO |
| 60110 | CAMARAO SETE BARBAS |
| 60111 | CAMARAO FERRINHO |
| 60112 | VILA FRANCA |
| 60113 | PITICAIA |
| 60114 | CAMARAO POTIMIRIM |
| 60115 | PITIGAIA |
| 60116 | POTIMIRIM |
| 60117 | CAMARAO CINZA |
| 60118 | CAMARAO DE AGUA SALGADA |
| 60119 | CAMARAO PISTOLA |
| 60120 | CAMARAO FRESCO |
| 60201 | CAMARAO SEM CASCA |
| 60401 | CAMARAO SECO |
| 60402 | CAMARAO SALGADO |
| 60601 | CAMARAO DEFUMADO |
| 61101 | SIRI |
| 61102 | GUAIA |
| 61103 | SIRI PIMENTA |
| 61104 | GUAJA |
| 61105 | SIRI DO MANGUE |
| 61106 | SIRI BRANCO |
| 61107 | CAXANGA |
| 61108 | SIRI CANELA |
| 61109 | SIRI BAU |
| 61110 | SIRI GUAJA |
| 61111 | SIRI AZUL |
| 61112 | PUA |
| 61113 | SIRIMIRIM |
| 61114 | SIRIACU |
| 61115 | SIRI PATOLA |
| 61116 | SIRIPUA |
| 61117 | CHORA MARE |
| 61118 | CHAMA MARE |
| 61119 | TESOURA (SIRI) |
| 61120 | BAU (SIRI) |
| 61121 | GOIA |
| 61201 | SIRI SEM CASCA |
| 61202 | CARNE DE SIRI |
| 61203 | FILE DE SIRI |
| 61401 | SIRI SALGADO |
| 62101 | CARANGUEJO |
| 62102 | GUAIAUMA |
| 62103 | ARATU |
| 62104 | GOIAMUM |
| 62105 | GUAIAMU |
| 62106 | GUAIAMUM |
| 62107 | FUBAMBA |
| 62108 | FUMBAMBA |
| 62109 | GOIAMU |
| 62110 | CARANGUEJO CATADO |
| 62111 | CARANQUEJO VIVO |
| 62201 | CARANGUEJO SEM CASCA |
| 62202 | CARNE DE CARANGUEJO |
| 62401 | CARANGUEJO SALGADO |
| 63101 | MARISCO |
| 63102 | MEXILHAO |
| 63103 | OSTRA DE POBRE (MARISCO) |
| 63104 | SURURU DAS PEDRAS (MARISCO) |
| 63105 | MACUNIM |
| 63201 | MARISCO SEM CASCA |
| 63401 | MARISCO SALGADO |
| 64101 | OSTRA |
| 64102 | OSTRA DO FUNDO |
| 64103 | OSTRA AMERICANA |
| 64104 | OSTRA DAS PEDRAS |
| 64105 | OSTRA DO MANGUE |
| 64106 | GUERIRI |
| 64107 | GORIRI |
| 64108 | LAMBRETA (OSTRA) |
| 64109 | OSTRA DA VIRGINIA |
| 64201 | OSTRA SEM CASCA |
| 64401 | OSTRA SALGADA |
| 65101 | LULA |
| 65102 | CALAMAR |
| 66101 | SURURU |
| 66102 | SURURU DE ALAGOAS |
| 66103 | SIRIRI |
| 66104 | BACUCU |
| 66105 | ALASTRIM |
| 66201 | SURURU SEM CASCA |
| 66401 | SURURU SALGADO |
| 67101 | LAGOSTA |
| 67102 | SAPATEIRA (LAGOSTA) |
| 67103 | LAGOSTA JAPONESA |
| 67104 | LAGOSTIM |
| 67105 | LAGOSTA SAPATEIRA |
| 67106 | LAGOSTA VERDE |
| 67107 | LAGOSTA DE ESPINHO |
| 67108 | LAGOSTA COMUM |
| 67109 | CAVAQUEIRA |
| 67110 | LAGOSTA VERMELHA |
| 67111 | LAGOSTA CABO VERDE |
| 67112 | LAGOSTA PINTADA |
| 67113 | LAGOSTA VERDADEIRA |
| 67201 | LAGOSTA SEM CASCA |
| 67401 | LAGOSTA SALGADA |
| 68101 | VIEIRA |
| 68102 | LEQUE (MOLUSCO) |
| 68201 | VIEIRA SEM CASCA |
| 68401 | VIEIRA SALGADA |
| 69101 | TARTARUGA DE MAR |
| 69102 | TARTARUGA DE PENTE |
| 69103 | TARTARUGA VERDADEIRA |
| 69104 | TARTARUGA VERDE |
| 69105 | SURUANA |
| 69201 | TARTARUGA EM POSTAS |
| 69301 | TARTARUGA EM FILE FRESCO |
| 69401 | TARTARUGA SALGADA |
| 69501 | TARTARUGA EM FILE CONGELADO |
| 70401 | BACALHAU |
| 71101 | PEIXE INTEIRO CAICANHA |
| 71102 | PEIXE INTEIRO CARCANHA |
| 71103 | PEIXE INTEIRO SAGUA |
| 71104 | PEIXE INTEIRO SANHOA |
| 71105 | PEIXE INTEIRO CHOUPA |
| 71106 | PEIXE INTEIRO SAUARA |
| 71107 | PEIXE INTEIRO PEDRA |
| 71108 | PEIXE INTEIRO CORO BRANCO |
| 71109 | PEIXE INTEIRO GOLOSA |
| 71110 | PEIXE INTEIRO GULOSA |
| 71201 | PEIXE EM POSTAS CAICANHA |
| 71301 | PEIXE EM FILE FRESCO CAICANHA |
| 71401 | PEIXE SALGADO CAICANHA |
| 71501 | PEIXE EM FILE CONGELADO CAICANHA |
| 72101 | PEIXE INTEIRO TIRAVIRA |
| 72102 | PEIXE INTEIRO CONGRO REAL |
| 72103 | PEIXE INTEIRO VIUVA |
| 72104 | PEIXE INTEIRO TIRIVIRA |
| 72105 | PEIXE INTEIRO TIRAVIDA |
| 72201 | PEIXE EM POSTAS TIRAVIRA |
| 72301 | PEIXE EM FILE FRESCO TIRAVIRA |
| 72401 | PEIXE SALGADO TIRAVIRA |
| 72501 | PEIXE EM FILE CONGELADO TIRAVIRA |
| 73101 | OVAS DE PEIXE (QUALQUER ESPECIE) |
| 73401 | OVAS DE PEIXE SALGADAS (QUALQUER ESPECIE) |
| 74101 | POLVO |
| 74201 | POLVO EM POSTAS |
| 74401 | POLVO SALGADO |
| 75101 | PEIXE INTEIRO ABROTE |
| 75201 | PEIXE EM POSTAS ABROTE |
| 75301 | PEIXE EM FILE FRESCO ABROTE |
| 75401 | PEIXE SALGADO ABROTE |
| 75501 | PEIXE EM FILE CONGELADO ABROTE |
| 76101 | PEIXE INTEIRO PARU |
| 77101 | PEIXE INTEIRO PIRUCAIA |
| 78101 | PEIXE INTEIRO SAMBUIO |
| 78102 | PEIXE INTEIRO SAMBUDA |
| 79101 | PEIXE INTEIRO VERDE |
| 80201 | PEIXE EM POSTAS SALMAO |
| 80501 | PEIXE EM FILE CONGELADO SALMAO |
| 81101 | PEIXE INTEIRO CARAPICU |
| 00101 | PEIXE INTEIRO DE AGUA DOCE NAO-ESPECIFICADO |
| 00201 | PEIXE EM POSTAS DE AGUA DOCE NAO-ESPECIFICADO |
| 00301 | PEIXE EM FILE FRESCO DE AGUA DOCE NAO-ESPECIFICADO |
| 00401 | PEIXE SALGADO DE AGUA DOCE NAO-ESPECIFICADO |
| 00501 | PEIXE EM FILE CONGELADO DE AGUA DOCE NAO-ESPECIFICADO |
| 01101 | PEIXE INTEIRO TRAIRA |
| 01102 | PEIXE INTEIRO TARARIRA |
| 01103 | PEIXE INTEIRO TARIEIRA |
| 01104 | PEIXE INTEIRO TARIIRA |
| 01105 | PEIXE INTEIRO TARAIRA |
| 01106 | PEIXE INTEIRO CONGUACU |
| 01107 | PEIXE INTEIRO TRARIEIRA |
| 01108 | PEIXE INTEIRO ROBAFO |
| 01109 | PEIXE INTEIRO DORME DORME |
| 01110 | PEIXE INTEIRO RUBAFO |
| 01111 | PEIXE INTEIRO MATURAQUE |
| 01201 | PEIXE EM POSTAS TRAIRA |
| 01301 | PEIXE EM FILE FRESCO TRAIRA |
| 01401 | PEIXE SALGADO TRAIRA |
| 01501 | PEIXE EM FILE CONGELADO TRAIRA |
| 02101 | PEIXE INTEIRO ACARA |
| 02102 | PEIXE INTEIRO CARA SEVERO |
| 02103 | PEIXE INTEIRO CARA TOPETE |
| 02104 | PEIXE INTEIRO CARA BERE |
| 02105 | PEIXE INTEIRO ACARAPEBA |
| 02106 | PEIXE INTEIRO CARA |
| 02107 | PEIXE INTEIRO CARA DIADEMA |
| 02108 | PEIXE INTEIRO CARA BERERE |
| 02109 | PEIXE INTEIRO ACARAUNA |
| 02110 | PEIXE INTEIRO ACARA ACU |
| 02111 | PEIXE INTEIRO CARA DE TOPETE |
| 02112 | PEIXE INTEIRO ACARA FUSO |
| 02113 | PEIXE INTEIRO CARA PRETO |
| 02114 | PEIXE INTEIRO CARA CASCUDO |
| 02115 | PEIXE INTEIRO CARA PATAQUIRA |
| 02116 | PEIXE INTEIRO CARA BOBO |
| 02117 | PEIXE INTEIRO CARA GUACU |
| 02118 | PEIXE INTEIRO CARA VACU |
| 02119 | PEIXE INTEIRO CARA GIGANTE |
| 02120 | PEIXE INTEIRO CARA TONTO |
| 02121 | PEIXE INTEIRO CARA ZEBU |
| 02122 | PEIXE INTEIRO CARA FERREIRO |
| 02123 | PEIXE INTEIRO MERERE |
| 02124 | PEIXE INTEIRO CORRO |
| 02125 | PEIXE INTEIRO MORERE |
| 02126 | PEIXE INTEIRO APAIARI |
| 02127 | PEIXE INTEIRO ARARUA |
| 02128 | PEIXE INTEIRO CARAUNA |
| 02129 | PEIXE INTEIRO ESPANHARINA |
| 02130 | PEIXE INTEIRO BUVUARI |
| 02131 | PEIXE INTEIRO CARAMBANGE |
| 02132 | PEIXE INTEIRO PILAPE |
| 02133 | PEIXE INTEIRO BARARUA |
| 02134 | PEIXE INTEIRO CARA ACU |
| 02201 | PEIXE EM POSTAS ACARA |
| 02301 | PEIXE EM FILE FRESCO ACARA |
| 02401 | PEIXE SALGADO ACARA |
| 02501 | PEIXE EM FILE CONGELADO ACARA |
| 03101 | PEIXE INTEIRO LAMBARI |
| 03102 | PEIXE INTEIRO PIABA PIXUNA |
| 03103 | PEIXE INTEIRO PIABA BRANCA |
| 03104 | PEIXE INTEIRO CASCA GROSSA |
| 03105 | PEIXE INTEIRO ISCA BRANCA |
| 03106 | PEIXE INTEIRO CEVELHA |
| 03107 | PEIXE INTEIRO MOCINHO |
| 03108 | PEIXE INTEIRO MOCINHA |
| 03109 | PEIXE INTEIRO TAMBIU |
| 03110 | PEIXE INTEIRO CATUNGA |
| 03111 | PEIXE INTEIRO TAPIOCA |
| 03112 | PEIXE INTEIRO MATUPIRI |
| 03113 | PEIXE INTEIRO CANIVETE |
| 03114 | PEIXE INTEIRO PIQUIRA |
| 03115 | PEIXE INTEIRO BRANQUINHA |
| 03116 | PEIXE INTEIRO BRANQUINHO |
| 03117 | PEIXE INTEIRO PIABUCU |
| 03118 | PEIXE INTEIRO PIABINHA |
| 03119 | PEIXE INTEIRO ALAMBARI |
| 03120 | PEIXE INTEIRO PIABA DA LAGOA |
| 03121 | PEIXE INTEIRO PIAVUCU |
| 03122 | PEIXE INTEIRO PIAVA |
| 03123 | PEIXE INTEIRO PIABA |
| 03124 | PEIXE INTEIRO PIABA CRIOULA |
| 03201 | PEIXE EM POSTAS LAMBARI |
| 03301 | PEIXE EM FILE FRESCO LAMBARI |
| 03401 | PEIXE SALGADO LAMBARI |
| 03501 | PEIXE EM FILE CONGELADO LAMBARI |
| 04101 | PEIXE INTEIRO SURUBIM |
| 04102 | PEIXE INTEIRO PIRAUACA |
| 04103 | PEIXE INTEIRO JURUPOCA |
| 04104 | PEIXE INTEIRO PIRAMBUCU |
| 04105 | PEIXE INTEIRO BOCA DE COLHER |
| 04106 | PEIXE INTEIRO JIRIPOCA |
| 04107 | PEIXE INTEIRO JERUPOCA |
| 04108 | PEIXE INTEIRO SURUBIM CAPARARI |
| 04109 | PEIXE INTEIRO SURUBIM PINTADO |
| 04110 | PEIXE INTEIRO POQUINHA |
| 04111 | PEIXE INTEIRO SURUBIM RAJADO |
| 04112 | PEIXE INTEIRO SURUBIM LIMA |
| 04113 | PEIXE INTEIRO JURUPENSEM |
| 04114 | PEIXE INTEIRO SURUMI |
| 04115 | PEIXE INTEIRO LOANGO |
| 04116 | PEIXE INTEIRO SURUBIM CHICOTE |
| 04117 | PEIXE INTEIRO PINTADO |
| 04118 | PEIXE INTEIRO SURUBI |
| 04119 | PEIXE INTEIRO SORUBI |
| 04120 | PEIXE INTEIRO SORUBIM |
| 04121 | PEIXE INTEIRO SURUMBI |
| 04122 | PEIXE INTEIRO CAPARARI |
| 04123 | PEIXE INTEIRO PIRAACA |
| 04124 | PEIXE INTEIRO BRUTELO |
| 04125 | PEIXE INTEIRO CACONETE DE RIO (SURUBIM) |
| 04126 | PEIXE INTEIRO CAPARI |
| 04127 | PEIXE INTEIRO BICO DE PATO |
| 04128 | PEIXE INTEIRO PONQUINHA |
| 04129 | PEIXE INTEIRO CAXARA |
| 04201 | PEIXE EM POSTAS SURUBIM |
| 04301 | PEIXE EM FILE FRESCO SURUBIM |
| 04401 | PEIXE SALGADO SURUBIM |
| 04501 | PEIXE EM FILE CONGELADO SURUBIM |
| 04701 | CABECA DE PINTADO |
| 04901 | PEIXE PINTADO (FORMA DE COMERCIALIZACAO NAO-DISCRIMINADA) |
| 05101 | PEIXE INTEIRO JACUNDA |
| 05102 | PEIXE INTEIRO CABECA AMARGA |
| 05103 | PEIXE INTEIRO MIXORNE |
| 05104 | PEIXE INTEIRO JACUNDA CABECUDO |
| 05105 | PEIXE INTEIRO JOANA GUENZA |
| 05106 | PEIXE INTEIRO JACUNDA VERDE |
| 05107 | PEIXE INTEIRO MARIA GUENZA |
| 05108 | PEIXE INTEIRO JACUNDA BRANCO |
| 05109 | PEIXE INTEIRO JACUNDA PIRANGA |
| 05110 | PEIXE INTEIRO JACUNDA ACU |
| 05111 | PEIXE INTEIRO JACUNDA PINIMA |
| 05112 | PEIXE INTEIRO JACUNDA TOURO |
| 05113 | PEIXE INTEIRO GUENZA |
| 05114 | PEIXE INTEIRO JOANINHA |
| 05115 | PEIXE INTEIRO JACUNDA COROA |
| 05116 | PEIXE INTEIRO JACUNDA TOTO |
| 05117 | PEIXE INTEIRO LOPE |
| 05118 | PEIXE INTEIRO SABAO |
| 05119 | PEIXE INTEIRO MICHOLA |
| 05120 | PEIXE INTEIRO SERRA DE AGUA DOCE (JACUNDA) |
| 05121 | PEIXE INTEIRO GUENZA VERDE |
| 05122 | PEIXE INTEIRO JOANINHA GUENZA |
| 05201 | PEIXE EM POSTAS JACUNDA |
| 05301 | PEIXE EM FILE FRESCO JACUNDA |
| 05401 | PEIXE SALGADO JACUNDA |
| 05501 | PEIXE EM FILE CONGELADO JACUNDA |
| 06101 | PEIXE INTEIRO PIRARUCU |
| 06102 | PEIXE INTEIRO PIRACU |
| 06103 | PEIXE INTEIRO TUBARAO DE MARIA (PIRARUCU) |
| 06104 | PEIXE INTEIRO BODECO |
| 06105 | PEIXE INTEIRO ANATO |
| 06106 | PEIXE INTEIRO PIRAURUCU |
| 06201 | PEIXE EM POSTAS PIRARUCU |
| 06301 | PEIXE EM FILE FRESCO PIRARUCU |
| 06401 | PEIXE SALGADO PIRARUCU |
| 06501 | PEIXE EM FILE CONGELADO PIRARUCU |
| 07101 | PEIXE INTEIRO ARACU |
| 07102 | PEIXE INTEIRO ARACU PINIMA |
| 07103 | PEIXE INTEIRO ARACU BRANCO |
| 07104 | PEIXE INTEIRO ARACU TAQUARA |
| 07105 | PEIXE INTEIRO ARACU PINTADO |
| 07106 | PEIXE INTEIRO TAGUARA |
| 07107 | PEIXE INTEIRO CAGUARA |
| 07108 | PEIXE INTEIRO ARACUTINGA |
| 07109 | PEIXE INTEIRO TAQUARA |
| 07201 | PEIXE EM POSTAS ARACU |
| 07301 | PEIXE EM FILE FRESCO ARACU |
| 07401 | PEIXE SALGADO ARACU |
| 07501 | PEIXE EM FILE CONGELADO ARACU |
| 08101 | PEIXE INTEIRO TILAPIA |
| 08201 | PEIXE EM POSTAS TILAPIA |
| 08301 | PEIXE EM FILE FRESCO TILAPIA |
| 08401 | PEIXE SALGADO TILAPIA |
| 08501 | PEIXE EM FILE CONGELADO TILAPIA |
| 09101 | PEIXE INTEIRO JEJU |
| 09102 | PEIXE INTEIRO TRAIRA PIXUNA (JEJU) |
| 09103 | PEIXE INTEIRO JIJU |
| 09104 | PEIXE INTEIRO TRAIRA PIXURIA (JEJU) |
| 09201 | PEIXE EM POSTAS JEJU |
| 09301 | PEIXE EM FILE FRESCO JEJU |
| 09401 | PEIXE SALGADO JEJU |
| 09501 | PEIXE EM FILE CONGELADO JEJU |
| 10101 | PEIXE INTEIRO CURIMATA |
| 10102 | PEIXE INTEIRO CURIMATAU |
| 10103 | PEIXE INTEIRO CURIMATA (RIO) |
| 10104 | PEIXE INTEIRO CHIRA |
| 10105 | PEIXE INTEIRO CURUMBATA |
| 10106 | PEIXE INTEIRO CORIMBA |
| 10107 | PEIXE INTEIRO MORE (CURIMATA) |
| 10108 | PEIXE INTEIRO CURIBATA |
| 10109 | PEIXE INTEIRO CURIMBA |
| 10110 | PEIXE INTEIRO CURIMBATA |
| 10111 | PEIXE INTEIRO CORUMBATA |
| 10112 | PEIXE INTEIRO CRUMATA |
| 10113 | PEIXE INTEIRO CURIMBOTA |
| 10114 | PEIXE INTEIRO BAMBA |
| 10115 | PEIXE INTEIRO GRUMEXA |
| 10116 | PEIXE INTEIRO GERICOIA |
| 10117 | PEIXE INTEIRO GRUMATA |
| 10118 | PEIXE INTEIRO GRAMATAO |
| 10201 | PEIXE EM POSTAS CURIMATA |
| 10301 | PEIXE EM FILE FRESCO CURIMATA |
| 10401 | PEIXE SALGADO CURIMATA |
| 10501 | PEIXE EM FILE CONGELADO CURIMATA |
| 10901 | PEIXE CURIMATA (FORMA DE COMERCIALIZACAO NAO-DISCRIMINADA) |
| 11101 | PEIXE INTEIRO TUCUNARE |
| 11102 | PEIXE INTEIRO TUCUNARE ACU |
| 11103 | PEIXE INTEIRO BURRIQUETI DE RIO (TUCUNARE) |
| 11104 | PEIXE INTEIRO TUCUNARE ACO |
| 11105 | PEIXE INTEIRO PUNARE |
| 11106 | PEIXE INTEIRO LUCANARI |
| 11107 | PEIXE INTEIRO TUCUNARE LUMBINHO |
| 11108 | PEIXE INTEIRO LACANARI |
| 11109 | PEIXE INTEIRO TUCUNARE BRANCO |
| 11110 | PEIXE INTEIRO TUCUNARE PUTANGA |
| 11111 | PEIXE INTEIRO TUCUNARETINGA |
| 11112 | PEIXE INTEIRO TUCUNARE PITANGA |
| 11113 | PEIXE INTEIRO TUCUNARE PINIMA |
| 11114 | PEIXE INTEIRO BURRIQUETA DE RIO (TUCUNARE) |
| 11115 | PEIXE INTEIRO BURRIQUETE DE RIO (TUCUNARE) |
| 11116 | PEIXE INTEIRO SARABIANA |
| 11117 | PEIXE INTEIRO PUTANGA |
| 11118 | PEIXE INTEIRO PITANGA |
| 11201 | PEIXE EM POSTAS TUCUNARE |
| 11301 | PEIXE EM FILE FRESCO TUCUNARE |
| 11401 | PEIXE SALGADO TUCUNARE |
| 11501 | PEIXE EM FILE CONGELADO TUCUNARE |
| 12101 | PEIXE INTEIRO ACARI |
| 12102 | PEIXE INTEIRO CASCUDO BARBADO |
| 12103 | PEIXE INTEIRO CARITO |
| 12104 | PEIXE INTEIRO CACHIMBAU |
| 12105 | PEIXE INTEIRO PILATOS |
| 12106 | PEIXE INTEIRO UACARI |
| 12107 | PEIXE INTEIRO BODO |
| 12108 | PEIXE INTEIRO GUACARI |
| 12109 | PEIXE INTEIRO CASCUDO ESPINHO |
| 12110 | PEIXE INTEIRO CASCUDINHO |
| 12111 | PEIXE INTEIRO CASCUDO LIMA |
| 12112 | PEIXE INTEIRO CASCUDO COMUM |
| 12113 | PEIXE INTEIRO CASCUDO ESPADA |
| 12114 | PEIXE INTEIRO CARI |
| 12115 | PEIXE INTEIRO BOI DE GUARA (ACARI) |
| 12116 | PEIXE INTEIRO ACARI PINTADO |
| 12117 | PEIXE INTEIRO APERTA GALHA |
| 12118 | PEIXE INTEIRO ACARIJUBA |
| 12119 | PEIXE INTEIRO ACARI MOLE |
| 12120 | PEIXE INTEIRO ACARI VIOLA |
| 12121 | PEIXE INTEIRO ACARI LARANJA |
| 12122 | PEIXE INTEIRO ACARI CHICOTE |
| 12123 | PEIXE INTEIRO ACARI ESPADA |
| 12124 | PEIXE INTEIRO ACARI CACHIMBO |
| 12125 | PEIXE INTEIRO ACARI BODO |
| 12126 | PEIXE INTEIRO CASCUDO |
| 12127 | PEIXE INTEIRO ANAIA |
| 12128 | PEIXE INTEIRO BARBUDO |
| 12201 | PEIXE EM POSTAS ACARI |
| 12301 | PEIXE EM FILE FRESCO ACARI |
| 12401 | PEIXE SALGADO ACARI |
| 12501 | PEIXE EM FILE CONGELADO ACARI |
| 12502 | PEIXE EM FILE CONGELADO CASCUDO |
| 13101 | PEIXE INTEIRO PIRAMUTABA |
| 13102 | PEIXE INTEIRO MULHER INGRATA |
| 13103 | PEIXE INTEIRO PIRAMUTA |
| 13104 | PEIXE INTEIRO PIRAMUTAVA |
| 13105 | PEIXE INTEIRO BARBA CHATA |
| 13201 | PEIXE EM POSTAS PIRAMUTABA |
| 13301 | PEIXE EM FILE FRESCO PIRAMUTABA |
| 13401 | PEIXE SALGADO PIRAMUTABA |
| 13501 | PEIXE EM FILE CONGELADO PIRAMUTABA |
| 14101 | PEIXE INTEIRO PIAU |
| 14102 | PEIXE INTEIRO PIAU VERDADEIRO |
| 14103 | PEIXE INTEIRO PIAU LAVRADO |
| 14104 | PEIXE INTEIRO PIAU GALO |
| 14105 | PEIXE INTEIRO PAIUDO |
| 14201 | PEIXE EM POSTAS PIAU |
| 14301 | PEIXE EM FILE FRESCO PIAU |
| 14401 | PEIXE SALGADO PIAU |
| 14501 | PEIXE EM FILE CONGELADO PIAU |
| 15101 | PEIXE INTEIRO MUCUM |
| 15102 | PEIXE INTEIRO COBRA |
| 15103 | PEIXE INTEIRO MUCU |
| 15104 | PEIXE INTEIRO MUCUM DE PELE |
| 15105 | PEIXE INTEIRO MUCUM DE ESCAMA |
| 15106 | PEIXE INTEIRO ENGUIA DE AGUA DOCE (MUCUM) |
| 15201 | PEIXE EM POSTAS MUCUM |
| 15301 | PEIXE EM FILE FRESCO MUCUM |
| 15401 | PEIXE SALGADO MUCUM |
| 15501 | PEIXE EM FILE CONGELADO MUCUM |
| 16101 | PEIXE INTEIRO DOURADO DE AGUA DOCE |
| 16102 | PEIXE INTEIRO PIRAJU |
| 16103 | PEIXE INTEIRO PIRAJUBA |
| 16104 | PEIXE INTEIRO SAIPE |
| 16105 | PEIXE INTEIRO SAIJE |
| 16106 | PEIXE INTEIRO PIRAUVU |
| 16107 | PEIXE INTEIRO PIRAIU |
| 16201 | PEIXE EM POSTAS DOURADO DE AGUA DOCE |
| 16301 | PEIXE EM FILE FRESCO DOURADO DE AGUA DOCE |
| 16401 | PEIXE SALGADO DOURADO DE AGUA DOCE |
| 16501 | PEIXE EM FILE CONGELADO DOURADO DE AGUA DOCE |
| 17101 | PEIXE INTEIRO TAMOATA |
| 17102 | PEIXE INTEIRO TAMBUATA |
| 17103 | PEIXE INTEIRO TAMBOATA |
| 17104 | PEIXE INTEIRO TAMOATA PINIMA |
| 17105 | PEIXE INTEIRO TAMUATA |
| 17106 | PEIXE INTEIRO TAMATA |
| 17107 | PEIXE INTEIRO CAMBOTA |
| 17108 | PEIXE INTEIRO CAMBOATA |
| 17109 | PEIXE INTEIRO SOLDADO DE AGUA DOCE (TAMOATA) |
| 17201 | PEIXE EM POSTAS TAMOATA |
| 17301 | PEIXE EM FILE FRESCO TAMOATA |
| 17401 | PEIXE SALGADO TAMOATA |
| 17501 | PEIXE EM FILE CONGELADO TAMOATA |
| 18101 | PEIXE INTEIRO PIRAPEUA |
| 18102 | PEIXE INTEIRO LENHA |
| 18103 | PEIXE INTEIRO SURUBIM MENA (PIRAPEUA) |
| 18104 | PEIXE INTEIRO PIRAJUPEVA |
| 18105 | PEIXE INTEIRO PIRAPEUAUA |
| 18201 | PEIXE EM POSTAS PIRAPEUA |
| 18301 | PEIXE EM FILE FRESCO PIRAPEUA |
| 18401 | PEIXE SALGADO PIRAPEUA |
| 18501 | PEIXE EM FILE CONGELADO PIRAPEUA |
| 19101 | PEIXE INTEIRO MAPARA |
| 19102 | PEIXE INTEIRO BRACO DE MULATA |
| 19103 | PEIXE INTEIRO OLELEIRO |
| 19104 | PEIXE INTEIRO SARDA BRACO DE MOCA (MAPARA) |
| 19105 | PEIXE INTEIRO MAPARA DE CAMETA |
| 19106 | PEIXE INTEIRO MANDI PERUANO (MAPARA) |
| 19107 | PEIXE INTEIRO BRACO DE MOCA |
| 19108 | PEIXE INTEIRO LAULAU |
| 19109 | PEIXE INTEIRO MAPURA |
| 19110 | PEIXE INTEIRO MAMPARA |
| 19201 | PEIXE EM POSTAS MAPARA |
| 19301 | PEIXE EM FILE FRESCO MAPARA |
| 19401 | PEIXE SALGADO MAPARA |
| 19501 | PEIXE EM FILE CONGELADO MAPARA |
| 20101 | PEIXE INTEIRO PIRANHA |
| 20102 | PEIXE INTEIRO PIRANHA CAJU |
| 20103 | PEIXE INTEIRO PIRANHA VERMELHA |
| 20104 | PEIXE INTEIRO PIRANHA CHATA |
| 20105 | PEIXE INTEIRO PIRANHA PINTADA |
| 20106 | PEIXE INTEIRO PIRANHA AMARELA |
| 20107 | PEIXE INTEIRO PIRANHA BEBA |
| 20108 | PEIXE INTEIRO PIRANHA DA LAGOA |
| 20109 | PEIXE INTEIRO PIRANHA PRETA |
| 20110 | PEIXE INTEIRO PIRANHA BRANCA |
| 20111 | PEIXE INTEIRO PIRANHA PEBA |
| 20112 | PEIXE INTEIRO PIRANHA CACHORRA |
| 20113 | PEIXE INTEIRO PIRANHA MAPARA |
| 20114 | PEIXE INTEIRO PIRAMPEVA |
| 20115 | PEIXE INTEIRO PIRAMBE |
| 20116 | PEIXE INTEIRO PIRAMPEBA |
| 20117 | PEIXE INTEIRO PIRAMBEVA |
| 20118 | PEIXE INTEIRO PIRAMBEBA |
| 20119 | PEIXE INTEIRO PIRANHA DOCE |
| 20120 | PEIXE INTEIRO COICOA |
| 20121 | PEIXE INTEIRO PIRAMPE |
| 20122 | PEIXE INTEIRO RODOLEIRA |
| 20123 | PEIXE INTEIRO CHUPITA |
| 20201 | PEIXE EM POSTAS PIRANHA |
| 20301 | PEIXE EM FILE FRESCO PIRANHA |
| 20401 | PEIXE SALGADO PIRANHA |
| 20501 | PEIXE EM FILE CONGELADO PIRANHA |
| 21101 | PEIXE INTEIRO PACU |
| 21102 | PEIXE INTEIRO PACUACU |
| 21103 | PEIXE INTEIRO PACU CHICO |
| 21104 | PEIXE INTEIRO PACU MIRIM |
| 21105 | PEIXE INTEIRO PACU CHATO |
| 21106 | PEIXE INTEIRO PACU BRANCO |
| 21107 | PEIXE INTEIRO PACUGUACU |
| 21108 | PEIXE INTEIRO PACU AZUL |
| 21109 | PEIXE INTEIRO BATUQUEIRO |
| 21110 | PEIXE INTEIRO PIROSCA |
| 21111 | PEIXE INTEIRO PACUTINGA |
| 21112 | PEIXE INTEIRO PEVA |
| 21113 | PEIXE INTEIRO PACUPEVA |
| 21114 | PEIXE INTEIRO PACUPEBA |
| 21115 | PEIXE INTEIRO MAFURA |
| 21116 | PEIXE INTEIRO PEBA |
| 21117 | PEIXE INTEIRO CAPADINHO |
| 21118 | PEIXE INTEIRO ABEL |
| 21119 | PEIXE INTEIRO CORONA |
| 21120 | PEIXE INTEIRO CARANHA |
| 21121 | PEIXE INTEIRO PANARE |
| 21201 | PEIXE EM POSTAS PACU |
| 21301 | PEIXE EM FILE FRESCO PACU |
| 21401 | PEIXE SALGADO PACU |
| 21501 | PEIXE EM FILE CONGELADO PACU |
| 22101 | PEIXE INTEIRO TAMBAQUI |
| 22102 | PEIXE INTEIRO CURUPETE |
| 22103 | PEIXE INTEIRO PACU DO AMAZONAS (TAMBAQUI) |
| 22201 | PEIXE EM POSTAS TAMBAQUI |
| 22301 | PEIXE EM FILE FRESCO TAMBAQUI |
| 22401 | PEIXE SALGADO TAMBAQUI |
| 22501 | PEIXE EM FILE CONGELADO TAMBAQUI |
| 22701 | CABECA DE PACU |
| 22901 | PEIXE TAMBAQUI (FORMA DE COMERCIALIZACAO NAO-DISCRIMINADA) |
| 23101 | PEIXE INTEIRO ANUJA |
| 23102 | PEIXE INTEIRO CACHORRINHO DE PADRE |
| 23103 | PEIXE INTEIRO ANOJADO |
| 23104 | PEIXE INTEIRO CACHORRO DE PADRE |
| 23105 | PEIXE INTEIRO CACHORRINHO |
| 23106 | PEIXE INTEIRO ANUIA |
| 23107 | PEIXE INTEIRO BODE |
| 23108 | PEIXE INTEIRO MANDIZINHO (ANUJA) |
| 23109 | PEIXE INTEIRO MANDICUMBA |
| 23110 | PEIXE INTEIRO CUMBACA (DE AGUA DOCE) (ANUJA) |
| 23111 | PEIXE INTEIRO CANGATINHA |
| 23112 | PEIXE INTEIRO MULATO |
| 23113 | PEIXE INTEIRO CHORADEIRA |
| 23114 | PEIXE INTEIRO BOCA LISA (DE AGUA DOCE) (ANUJA) |
| 23115 | PEIXE INTEIRO BAGRE CHORA (ANUJA) |
| 23116 | PEIXE INTEIRO CABECA DE FERRO |
| 23117 | PEIXE INTEIRO LIRIO |
| 23118 | PEIXE BAGRE DE RIO |
| 23119 | PEIXE INTEIRO MANDI |
| 23120 | PEIXE INTEIRO JUNDIA |
| 23121 | PEIXE INTEIRO BOGA |
| 23122 | PEIXE INTEIRO MANDIUVA |
| 23123 | PEIXE INTEIRO JANDIA |
| 23201 | PEIXE EM POSTAS ANUJA |
| 23301 | PEIXE EM FILE FRESCO ANUJA |
| 23401 | PEIXE SALGADO ANUJA |
| 23501 | PEIXE EM FILE CONGELADO ANUJA |
| 24101 | PEIXE INTEIRO SARDINHA DE AGUA DOCE |
| 24102 | PEIXE INTEIRO APAPA |
| 24103 | PEIXE INTEIRO SARDINHA GRANDE DE AGUA DOCE |
| 24104 | PEIXE INTEIRO SARDINHAO (AGUA DOCE) |
| 24105 | PEIXE INTEIRO SARDA DE AGUA DOCE |
| 24106 | PEIXE INTEIRO ARANGAL |
| 24201 | PEIXE EM POSTAS SARDINHA DE AGUA DOCE |
| 24301 | PEIXE EM FILE FRESCO SARDINHA DE AGUA DOCE |
| 24401 | PEIXE SALGADO SARDINHA DE AGUA DOCE |
| 24501 | PEIXE EM FILE CONGELADO SARDINHA DE AGUA DOCE |
| 25101 | PEIXE INTEIRO JARAQUI |
| 25102 | PEIXE INTEIRO JERAQUI |
| 25103 | PEIXE INTEIRO JERIQUI |
| 25201 | PEIXE EM POSTAS JARAQUI |
| 25301 | PEIXE EM FILE FRESCO JARAQUI |
| 25401 | PEIXE SALGADO JARAQUI |
| 25501 | PEIXE EM FILE CONGELADO JARAQUI |
| 26101 | PEIXE INTEIRO CACHORRO |
| 26102 | PEIXE INTEIRO CIGARRA |
| 26103 | PEIXE INTEIRO ICANGA |
| 26104 | PEIXE INTEIRO PIRAPUCU |
| 26105 | PEIXE INTEIRO TAJIBUCU |
| 26106 | PEIXE INTEIRO TAJABUCU |
| 26107 | PEIXE INTEIRO PIRAPUCA |
| 26108 | PEIXE INTEIRO TAJAPECO |
| 26109 | PEIXE INTEIRO PIRA ANDIRA |
| 26110 | PEIXE INTEIRO SAICANGA |
| 26111 | PEIXE INTEIRO SARANHA |
| 26112 | PEIXE INTEIRO BICUDA DE RIO (CACHORRO) |
| 26113 | PEIXE INTEIRO DENTUDO PINTADO |
| 26114 | PEIXE INTEIRO BOCARRA |
| 26115 | PEIXE INTEIRO CUBIO |
| 26116 | PEIXE INTEIRO CUBIU |
| 26117 | PEIXE INTEIRO BOCUDO |
| 26118 | PEIXE INTEIRO JAUARANA |
| 26201 | PEIXE EM POSTAS CACHORRO |
| 26301 | PEIXE EM FILE FRESCO CACHORRO |
| 26401 | PEIXE SALGADO CACHORRO |
| 26501 | PEIXE EM FILE CONGELADO CACHORRO |
| 27101 | PEIXE INTEIRO PIABANHA |
| 27102 | PEIXE INTEIRO PIRAPITINGA |
| 27103 | PEIXE INTEIRO TARAPITINGA |
| 27104 | PEIXE INTEIRO TRAPITINGA |
| 27201 | PEIXE EM POSTAS PIABANHA |
| 27301 | PEIXE EM FILE FRESCO PIABANHA |
| 27401 | PEIXE SALGADO PIABANHA |
| 27501 | PEIXE EM FILE CONGELADO PIABANHA |
| 27601 | PEIXE EM FILE FRESCO DE PIRAPITINGA |
| 28101 | PEIXE INTEIRO PIRAIBA |
| 28102 | PEIXE INTEIRO FILHOTE |
| 28103 | PEIXE INTEIRO PIRATINGA (PIRAIBA) |
| 28201 | PEIXE EM POSTAS PIRAIBA |
| 28301 | PEIXE EM FILE FRESCO PIRAIBA |
| 28401 | PEIXE SALGADO PIRAIBA |
| 28501 | PEIXE EM FILE CONGELADO PIRAIBA |
| 29101 | PEIXE INTEIRO BACU |
| 29102 | PEIXE INTEIRO ABOTOADO |
| 29103 | PEIXE INTEIRO ARMAU |
| 29104 | PEIXE INTEIRO DAQUEIRO |
| 29105 | PEIXE INTEIRO BACU DE PEDRA |
| 29106 | PEIXE INTEIRO BACU PEDRA |
| 29107 | PEIXE INTEIRO VACU |
| 29108 | PEIXE INTEIRO BOTOADO |
| 29109 | PEIXE INTEIRO CUIU CUIU |
| 29201 | PEIXE EM POSTAS BACU |
| 29301 | PEIXE EM FILE FRESCO BACU |
| 29401 | PEIXE SALGADO BACU |
| 29501 | PEIXE EM FILE CONGELADO BACU |
| 30101 | PEIXE INTEIRO PIRARARA |
| 30102 | PEIXE INTEIRO PIRAUARARA |
| 30103 | PEIXE INTEIRO ARARA |
| 30104 | PEIXE INTEIRO CAJARO |
| 30105 | PEIXE INTEIRO UARARA |
| 30106 | PEIXE INTEIRO PIRAMBREPE |
| 30107 | PEIXE INTEIRO LAITU |
| 30201 | PEIXE EM POSTAS PIRARARA |
| 30301 | PEIXE EM FILE FRESCO PIRARARA |
| 30401 | PEIXE SALGADO PIRARARA |
| 30501 | PEIXE EM FILE CONGELADO PIRARARA |
| 31101 | PEIXE INTEIRO PACAMAO DE AGUA DOCE |
| 31102 | PEIXE INTEIRO APACAMA DE AGUA DOCE |
| 31103 | PEIXE INTEIRO PACUMA DE AGUA DOCE |
| 31104 | PEIXE INTEIRO PIRACURURU |
| 31105 | PEIXE INTEIRO SAPO |
| 31106 | PEIXE INTEIRO BAGRE SAPO (PACAMAO DE AGUA DOCE) |
| 31107 | PEIXE INTEIRO PACAMA DE AGUA DOCE |
| 31108 | PEIXE INTEIRO PIACURURU |
| 31109 | PEIXE INTEIRO MANGURUIU |
| 31110 | PEIXE INTEIRO MANGURIU |
| 31111 | PEIXE INTEIRO PRECAMBUCU |
| 31112 | PEIXE INTEIRO PRECUMBUCU |
| 31201 | PEIXE EM POSTAS PACAMAO DE AGUA DOCE |
| 31301 | PEIXE EM FILE FRESCO PACAMAO DE AGUA DOCE |
| 31401 | PEIXE SALGADO PACAMAO DE AGUA DOCE |
| 31501 | PEIXE EM FILE CONGELADO PACAMAO DE AGUA DOCE |
| 32101 | PEIXE INTEIRO DOURADA |
| 32102 | PEIXE INTEIRO DOURADA DO AMAZONAS |
| 32103 | PEIXE INTEIRO DOIRADA DO AMAZONAS |
| 32104 | PEIXE INTEIRO DOIRADA |
| 32201 | PEIXE EM POSTAS DOURADA |
| 32301 | PEIXE EM FILE FRESCO DOURADA |
| 32401 | PEIXE SALGADO DOURADA |
| 32501 | PEIXE EM FILE CONGELADO DOURADA |
| 32901 | PEIXE DOURADA (FORMA DE COMERCIALIZACAO NAO-DISCRIMINADA) |
| 33101 | PEIXE INTEIRO PIRANAMBU |
| 33102 | PEIXE INTEIRO GALINHA |
| 33103 | PEIXE INTEIRO INAMBU |
| 33104 | PEIXE INTEIRO INHAMBU |
| 33105 | PEIXE INTEIRO PIRAMBU (DE AGUA DOCE) (PIRANAMBU) |
| 33106 | PEIXE INTEIRO PIRANIAMBU |
| 33107 | PEIXE INTEIRO PINTADINHO (DE AGUA DOCE) (PIRANAMBU) |
| 33108 | PEIXE INTEIRO PIRANIAMPU |
| 33109 | PEIXE INTEIRO PIRANAMPU |
| 33110 | PEIXE INTEIRO NAMBU |
| 33111 | PEIXE INTEIRO MANTOPAQUE |
| 33112 | PEIXE INTEIRO MOELA |
| 33113 | PEIXE INTEIRO BARBADO (DE AGUA DOCE) (PIRANAMBU) |
| 33114 | PEIXE INTEIRO PIRAMAPU |
| 33201 | PEIXE EM POSTAS PIRANAMBU |
| 33301 | PEIXE EM FILE FRESCO PIRANAMBU |
| 33401 | PEIXE SALGADO PIRANAMBU |
| 33501 | PEIXE EM FILE CONGELADO PIRANAMBU |
| 34101 | PEIXE INTEIRO FIDALGO |
| 34102 | PEIXE INTEIRO BAGRE FIDALGO (FIDALGO) |
| 34201 | PEIXE EM POSTAS FIDALGO |
| 34301 | PEIXE EM FILE FRESCO FIDALGO |
| 34401 | PEIXE SALGADO FIDALGO |
| 34501 | PEIXE EM FILE CONGELADO FIDALGO |
| 34901 | PEIXE FIDALGO (FORMA DE COMERCIALIZACAO NAO-DISCRIMINADA) |
| 35101 | PEIXE INTEIRO TABARANA |
| 35102 | PEIXE INTEIRO JATUBARANA |
| 35103 | PEIXE INTEIRO JUTUBARANA |
| 35104 | PEIXE INTEIRO TUBARANA |
| 35105 | PEIXE INTEIRO TRAGUIRA |
| 35201 | PEIXE EM POSTAS TABARANA |
| 35301 | PEIXE EM FILE FRESCO TABARANA |
| 35401 | PEIXE SALGADO TABARANA |
| 35501 | PEIXE EM FILE CONGELADO TABARANA |
| 36101 | PEIXE INTEIRO MANDUBE |
| 36102 | PEIXE INTEIRO ANDUBE |
| 36103 | PEIXE INTEIRO MANDURI |
| 36104 | PEIXE INTEIRO MANDUVA |
| 36105 | PEIXE INTEIRO MANDUBE MIRIM |
| 36106 | PEIXE INTEIRO MANDIBE |
| 36107 | PEIXE INTEIRO MANDUBI |
| 36108 | PEIXE INTEIRO ANDUBI |
| 36201 | PEIXE EM POSTAS MANDUBE |
| 36301 | PEIXE EM FILE FRESCO MANDUBE |
| 36401 | PEIXE SALGADO MANDUBI |
| 36501 | PEIXE EM FILE CONGELADO MANDUBI |
| 37101 | PEIXE INTEIRO PIRAPUTANGA |
| 37102 | PEIXE INTEIRO PIRAPITANGA |
| 37103 | PEIXE INTEIRO PIRAPITA |
| 37201 | PEIXE EM POSTAS PIRAPUTANGA |
| 37301 | PEIXE EM FILE FRESCO PIRAPUTANGA |
| 37401 | PEIXE SALGADO PIRAPUTANGA |
| 37501 | PEIXE EM FILE CONGELADO PIRAPUTANGA |
| 38101 | PEIXE INTEIRO JAU |
| 38102 | PEIXE INTEIRO JARIPOCA |
| 38103 | PEIXE INTEIRO JUNDIA DE LAGOA (JAU) |
| 38104 | PEIXE INTEIRO JAUPOCA |
| 38201 | PEIXE EM POSTAS JAU |
| 38301 | PEIXE EM FILE FRESCO JAU |
| 38401 | PEIXE SALGADO JAU |
| 38501 | PEIXE EM FILE CONGELADO JAU |
| 39101 | PEIXE INTEIRO PIAPARA |
| 39102 | PEIXE INTEIRO PIAMPARA |
| 39103 | PEIXE INTEIRO PIRAPARA |
| 39201 | PEIXE EM POSTAS PIAPARA |
| 39301 | PEIXE EM FILE FRESCO PIAPARA |
| 39401 | PEIXE SALGADO PIAPARA |
| 39501 | PEIXE EM FILE CONGELADO PIAPARA |
| 40101 | PEIXE INTEIRO ARUANA |
| 40102 | PEIXE INTEIRO CARAPANA |
| 40103 | PEIXE INTEIRO AMANA |
| 40104 | PEIXE INTEIRO URUANA |
| 40105 | PEIXE INTEIRO ARAUANA |
| 40106 | PEIXE INTEIRO SULAMBA |
| 40201 | PEIXE EM POSTAS ARUANA |
| 40301 | PEIXE EM FILE FRESCO ARUANA |
| 40401 | PEIXE SALGADO ARUANA |
| 40501 | PEIXE EM FILE CONGELADO ARUANA |
| 41101 | PEIXE INTEIRO PIRACANJUBA |
| 41102 | PEIXE INTEIRO PIRACANJUVA |
| 41103 | PEIXE INTEIRO PIRACANJUVIRA |
| 41104 | PEIXE INTEIRO TRAITINGA |
| 41201 | PEIXE EM POSTAS PIRACANJUBA |
| 41301 | PEIXE EM FILE FRESCO PIRACANJUBA |
| 41401 | PEIXE SALGADO PIRACANJUBA |
| 41501 | PEIXE EM FILE CONGELADO PIRACANJUBA |
| 42101 | PEIXE INTEIRO SAIRU |
| 42102 | PEIXE INTEIRO SAIRU DE RIO |
| 42103 | PEIXE INTEIRO SAGUIRU |
| 42104 | PEIXE INTEIRO SAGUIRI |
| 42105 | PEIXE INTEIRO SAGARU |
| 42106 | PEIXE INTEIRO SABARU |
| 42107 | PEIXE INTEIRO SAGUIRA |
| 42108 | PEIXE INTEIRO BIRUBA |
| 42109 | PEIXE INTEIRO SAGUACU |
| 42110 | PEIXE INTEIRO SABURU |
| 42111 | PEIXE INTEIRO LAMBARI BOCARRA (SAIRU) |
| 42201 | PEIXE EM POSTAS SAIRU |
| 42301 | PEIXE EM FILE FRESCO SAIRU |
| 42401 | PEIXE SALGADO SAIRU |
| 42501 | PEIXE EM FILE CONGELADO SAIRU |
| 43101 | CAMARAO DE AGUA DOCE |
| 43102 | CAMARAO LAGOSTA (AGUA DOCE) |
| 43103 | PITU (CAMARAO DE AGUA DOCE) |
| 43104 | ZABUMBA |
| 43105 | CAMARAO ZABUMBA (AGUA DOCE) |
| 43106 | CAMARAO BRACO FORTE (AGUA DOCE) |
| 43107 | CAMARAO CASTANHO (AGUA DOCE) |
| 43201 | CAMARAO DE AGUA DOCE SEM CASCA |
| 43401 | CAMARAO DE AGUA DOCE SALGADO |
| 44101 | TRACAJA |
| 44102 | GANGAPARRA |
| 44103 | AIACA |
| 44104 | TARTARUGA DE AGUA DOCE |
| 44105 | TARTARUGA DO AMAZONAS |
| 44106 | TRACAJA CABECADA |
| 44107 | CABECADA |
| 44108 | ARAU |
| 44109 | AIUCA |
| 44110 | JURARA ACU |
| 44111 | TERECAI |
| 44112 | JURARA |
| 44113 | BRACAJA |
| 44114 | IURURA |
| 44115 | IURARA |
| 44116 | PITIU |
| 44117 | IACA |
| 44118 | CAPITARI |
| 44119 | JURURA |
| 44120 | CAPININGA |
| 44121 | CAPINIMA |
| 44122 | ANURI |
| 44123 | TARECAI |
| 44124 | TARACAIA |
| 44201 | TRACAJA EM POSTAS |
| 44301 | TRACAJA EM FILE FRESCO |
| 44401 | TRACAJA SALGADA |
| 44501 | TRACAJA EM FILE CONGELADO |
| 45101 | RA |
| 45102 | RA CACOTE |
| 45103 | RA COMUM |
| 45104 | RA VERDADEIRA |
| 45105 | RA TOURO GIGANTE |
| 45106 | RA NIMBUIA |
| 45107 | RA PIMENTA |
| 45108 | RA TOURO |
| 45109 | JIA |
| 45110 | RA PAULISTINHA |
| 45111 | RA MIRIM |
| 45112 | RA JIA |
| 45113 | CACOTE |
| 45114 | RA GIMBUIA |
| 45115 | PAULISTINHA |
| 45116 | NIMBUIA |
| 45117 | GIMBUIA |
| 45201 | RA EM POSTAS |
| 45301 | RA EM FILE FRESCO |
| 45401 | RA SALGADA |
| 45501 | RA EM FILE CONGELADO |
| 46101 | PEIXE INTEIRO BAGRE DE AGUA DOCE |
| 46201 | PEIXE EM POSTAS BAGRE DE AGUA DOCE |
| 46301 | PEIXE EM FILE FRESCO BAGRE DE AGUA DOCE |
| 46401 | PEIXE SALGADO BAGRE DE AGUA DOCE |
| 46501 | PEIXE EM FILE CONGELADO BAGRE DE AGUA DOCE |
| 47101 | MUCUA |
| 47201 | MUCUA EM POSTAS |
| 47301 | MUCUA EM FILE FRESCO |
| 47401 | MUCUA SALGADA |
| 47501 | MUCUA EM FILE CONGELADO |
| 48101 | PEIXE INTEIRO ITUI |
| 48102 | PEIXE INTEIRO TUI |
| 48103 | PEIXE INTEIRO SARAPO |
| 48201 | PEIXE EM POSTAS ITUI |
| 48301 | PEIXE EM FILE FRESCO ITUI |
| 48401 | PEIXE SALGADO ITUI |
| 48501 | PEIXE EM FILE CONGELADO ITUI |
| 49101 | PEIXE INTEIRO TRUTA |
| 49201 | PEIXE EM POSTAS TRUTA |
| 49301 | PEIXE EM FILE FRESCO TRUTA |
| 49401 | PEIXE SALGADO TRUTA |
| 49501 | PEIXE EM FILE CONGELADO TRUTA |
| 50101 | AVIU |
| 50201 | AVIU SEM CASCA |
| 50401 | AVIU SALGADO |
| 51101 | PEIXE INTEIRO MATRINXA |
| 51102 | PEIXE INTEIRO MATRINXAO |
| 51103 | PEIXE INTEIRO MAMURI |
| 51104 | PEIXE INTEIRO JATUARANA |
| 51201 | PEIXE EM POSTAS MATRINXA |
| 51301 | PEIXE EM FILE FRESCO MATRINXA |
| 51401 | PEIXE SALGADO MATRINXA |
| 51501 | PEIXE EM FILE CONGELADO MATRINXA |
| 51901 | PEIXE MATRINXA (FORMA DE COMERCIALIZACAO NAO-DISCRIMINADA) |
| 52101 | PEIXE INTEIRO ARACAPURI |
| 53101 | PEIXE INTEIRO ARUIRI |
| 54101 | PEIXE INTEIRO BAIACU |
| 55101 | PEIXE INTEIRO CARPA |
| 55301 | PEIXE EM FILE FRESCO CARPA |
| 56101 | PEIXE INTEIRO CUJUBA |
| 57101 | PEIXE INTEIRO VOADOR |
| 57102 | PEIXE INTEIRO FLECHEIRO |
| 58101 | PEIXE INTEIRO PIRACATINGA |
| 59101 | PEIXE INTEIRO TRAIRAO |
| 60101 | PEIXE INTEIRO CHAPEREMA |
| 61101 | CARAMUJO |
| 61102 | URUA ( MOLUSCO) |
| 00101 | PEIXE INTEIRO NAO-ESPECIFICADO |
| 00201 | PEIXE EM POSTAS NAO-ESPECIFICADO |
| 00301 | PEIXE EM FILE FRESCO NAO-ESPECIFICADO |
| 00401 | PEIXE SALGADO NAO-ESPECIFICADO |
| 00402 | PEIXE SECO NAO-ESPECIFICADO |
| 00501 | PEIXE EM FILE CONGELADO NAO-ESPECIFICADO |
| 00701 | CABECA DE PEIXE |
| 00901 | PEIXE NAO-ESPECIFICADO |
| 01102 | PEIXE EM POSTAS TAMBACU |
| 00101 | AZEITONA |
| 00102 | AZEITONA EM CONSERVA |
| 00103 | AZEITONA VERDE |
| 00104 | AZEITONA PRETA |
| 00105 | AZEITONA RECHEADA EN CONSERVA |
| 00106 | AZEITONA SEM CAROCO |
| 00107 | AZEITONA FATIADA EM CONSERVA |
| 00201 | ERVILHA EM CONSERVA |
| 00202 | PETIT POIS |
| 00301 | ERVILHA COM CENOURA EM CONSERVA |
| 00302 | ERVILHA E CENOURA EM CONSERVA |
| 00401 | MILHO VERDE EM CONSERVA |
| 00402 | MILHO EM CONSERVA |
| 00501 | PALMITO EM CONSERVA |
| 00601 | COGUMELO EM CONSERVA |
| 00602 | CHAMPIGNON EM CONSERVA |
| 00603 | FUNGHI SECO |
| 00701 | ASPARGO EM CONSERVA |
| 00801 | CENOURA EM CONSERVA |
| 00901 | REPOLHO EM CONSERVA |
| 00902 | CHUCRUTE |
| 01001 | TOMATE SEM PELE EM CONSERVA |
| 01002 | TOMATE COM PELE EM CONSERVA |
| 01101 | LEGUME NAO-ESPECIFICADO EM CONSERVA |
| 01102 | LEGUME EM CONSERVA NAO-ESPECIFICADO |
| 01201 | SOPA INFANTIL DE QUALQUER SABOR |
| 01202 | SOPA INFANTIL COM LEGUMES OU VERDURAS |
| 01203 | ALIMENTO INFANTIL CREMOSO (EXCETO SOBREMESA) |
| 01204 | ALIMENTO INFANTIL EM PASTA (EXCETO SOBREMESA) |
| 01205 | PATE INFANTIL (EXCETO SOBREMESA) |
| 01206 | CREME INFANTIL (EXCETO SOBREMESA) |
| 01207 | PAPINHA (ALIMENTO INFANTIL) |
| 01208 | SOPINHA PARA BEBE |
| 01301 | SOPA DE LEGUMES EM CONSERVA |
| 01401 | SOPA DESIDRATADA DE QUALQUER SABOR |
| 01402 | SOPA EM PACOTE DE QUALQUER SABOR |
| 01403 | SOPA CONCENTRADA EM PACOTE DE QUALQUER SABOR |
| 01404 | CREME DE CEBOLA (SOPA DESIDRATADA) |
| 01405 | CREME DE LEGUMES (SOPA DESIDRATADA) |
| 01406 | YAKISSOBA (SOPA DE LEGUMES DESIDRATADA) |
| 01407 | CANJA DESIDRATADA |
| 01408 | CREME DE ERVILHA (SOPA DESIDRATADA) |
| 01409 | SOPA DE LEGUMES (SOPA DESIDRATADA) |
| 01501 | MILHO VERDE COM ERVILHA EM CONSERVA |
| 01502 | ERVILHA E MILHO EM CONSERVA |
| 01503 | MILHO E ERVILHA EM CONSERVA |
| 01504 | ERVILHA E MILHO VERDE EM CONSERVA |
| 01505 | MILHO VERDE E ERVILHA EM CONSERVA |
| 01601 | PIMENTA MALAGUETA EM CONSERVA |
| 01602 | MALAGUETA EM CONSERVA |
| 01701 | PIMENTA DE CHEIRO EM CONSERVA |
| 01702 | PIMENTA BODE EM CONSERVA |
| 01801 | PIMENTA NAO-ESPECIFICADA EM CONSERVA |
| 01802 | MOLHO DE PIMENTA NAO-ESPECIFICADA |
| 01803 | PIMENTA EM CONSERVA NAO-ESPECIFICADA |
| 01901 | FEIJOADA EM CONSERVA |
| 01902 | FEIJOADA COMPLETA EM CONSERVA |
| 01903 | FEIJOADA PRONTA EM CONSERVA |
| 02001 | FEIJAO BRANCO COM DOBRADINHA EM CONSERVA |
| 02002 | DOBRADINHA COM FEIJAO BRANCO EM CONSERVA |
| 02101 | COZIDO EM CONSERVA |
| 02201 | PATE DE FIGADO EM CONSERVA |
| 02202 | PASTA DE FIGADO EM CONSERVA |
| 02301 | PATE DE CARNE EM CONSERVA |
| 02302 | PASTA DE CARNE EM CONSERVA |
| 02303 | PATE DE CALABRESA |
| 02401 | PATE DE PRESUNTO EM CONSERVA |
| 02402 | PASTA DE PRESUNTO EM CONSERVA |
| 02501 | PATE DE GALINHA EM CONSERVA |
| 02502 | PASTA DE GALINHA EM CONSERVA |
| 02503 | PATE DE FRANGO EM CONSERVA |
| 02601 | CARNE BOVINA EM CONSERVA |
| 02602 | ALMONDEGA EM CONSERVA |
| 02603 | KITUTE BOVINO |
| 02604 | ALMONDEGA AO MOLHO EM CONSERVA |
| 02605 | VITELADA |
| 02606 | CORNED BEEF |
| 02607 | QUITUTE BOVINO |
| 02701 | CARNE SUINA EM CONSERVA |
| 02702 | FIAMBRADA SUINA |
| 02703 | VIANDADA SUINA |
| 02704 | KITUTE SUINO |
| 02705 | PRESUNTADA |
| 02706 | QUITUTE SUINO |
| 02707 | FIAMBRE |
| 02708 | AFIAMBRADO |
| 02801 | SALSICHA EM CONSERVA |
| 02901 | LINGUICA EM CONSERVA |
| 03001 | PEIXE SARDINHA EM CONSERVA |
| 03002 | SARDINHA EM CONSERVA |
| 03003 | SARDINHA EM LATA |
| 03101 | PEIXE ANCHOVA EM CONSERVA |
| 03102 | PEIXE ENCHOVA EM CONSERVA |
| 03103 | ANCHOVA EM CONSERVA |
| 03104 | ENCHOVA EM CONSERVA |
| 03201 | PEIXE CORVINA EM CONSERVA |
| 03202 | CORVINA EM CONSERVA |
| 03301 | PEIXE CAVALINHA EM CONSERVA |
| 03302 | CAVALINHA EM CONSERVA |
| 03401 | PEIXE ATUM EM CONSERVA |
| 03402 | ATUM EM CONSERVA |
| 03403 | ATUM RALADO |
| 03404 | ATUM SOLIDO |
| 03501 | PEIXE SAVELHA EM CONSERVA |
| 03502 | SAVELHA EM CONSERVA |
| 03601 | CAMARAO EM CONSERVA |
| 03701 | PEPINO EM CONSERVA |
| 03801 | CEBOLA EM CONSERVA |
| 03901 | PATE DE PEIXE EM CONSERVA |
| 03902 | PASTA DE PEIXE EM CONSERVA |
| 04001 | PICLES |
| 04101 | PEIXE BONITO EM CONSERVA |
| 04102 | BONITO EM CONSERVA |
| 04201 | LENTILHA EM CONSERVA |
| 04301 | MORTADELA EM CONSERVA |
| 04401 | ALGAS EM CONSERVA |
| 04402 | ALGA EM CONSERVA |
| 04501 | PEIXE PESCADA EM CONSERVA |
| 04502 | PESCADA EM CONSERVA |
| 04601 | CARNE DE FRANGO EM CONSERVA |
| 04602 | QUITUTE DE FRANGO |
| 04603 | QUITUTE DE FRANGO EM CONSERVA |
| 04604 | CARNE DE GALINHA EM CONSERVA |
| 04701 | PATE DE PERU EM CONSERVA |
| 04702 | PASTA DE PERU EM CONSERVA |
| 04801 | PATE DE LINGUA EM CONSERVA |
| 04802 | PASTA DE LINGUA EM CONSERVA |
| 04901 | PEIXE TAINHA EM CONSERVA |
| 04902 | TAINHA EM CONSERVA |
| 04903 | KAMA BOKU (TAINHA EM CONSERVA) |
| 04904 | KAMABOKU (TAINHA EM CONSERVA) |
| 05001 | BERBIGAO EM CONSERVA |
| 05101 | FIAMBRE DE CHESTER |
| 05102 | PRESUNTO DE CHESTER |
| 05201 | NABO EM CONSERVA |
| 05301 | ALICHE |
| 05401 | STROGONOFF EM CONSERVA |
| 05402 | STROGONOFF ENCARTONADO |
| 05403 | STROGONOFF EM PACOTE |
| 05501 | CONSERVANTE PARA ALIMENTOS |
| 05601 | CARNE EM CONSERVA NAO-ESPECIFICADA |
| 05701 | PIMENTA DE CUMARI EM CONSERVA |
| 05702 | PIMENTA CUMARI EM CONSERVA |
| 05703 | CUMARI EM CONSERVA |
| 05801 | ALIMENTO INFANTIL NAO-ESPECIFICADO |
| 05802 | NESTLE BABY |
| 05901 | KANI KAMA |
| 06001 | SALMAO EM CONSERVA |
| 06101 | SOPA DESIDRATADA DE QUALQUER SABOR LIGHT |
| 06102 | SOPA EM PACOTE DE QUALQUER SABOR LIGHT |
| 06103 | SOPA CONCENTRADA EM PACOTE DE QUALQUER SABOR LIGHT |
| 06104 | CREME DE CEBOLA (SOPA DESIDRATADA) LIGHT |
| 06105 | CREME DE LEGUMES (SOPA DESIDRATADA) LIGHT |
| 06106 | YAKISSOBA (SOPA DE LEGUMES DESIDRATADA) LIGHT |
| 06107 | CANJA DESIDRATADA LIGHT |
| 06201 | PEIXE SARDINHA EM CONSERVA LIGHT |
| 06202 | SARDINHA EM CONSERVA LIGHT |
| 06203 | SARDINHA EM LATA LIGHT |
| 06301 | PEIXE ATUM EM CONSERVA LIGHT |
| 06302 | ATUM EM CONSERVA LIGHT |
| 06303 | ATUM RALADO LIGHT |
| 06304 | ATUM SOLIDO LIGHT |
| 06401 | MORTADELA EM CONSERVA LIGHT |
| 06501 | FIAMBRE DE CHESTER LIGHT |
| 06502 | PRESUNTO DE CHESTER LIGHT |
| 06601 | BERINJELA AGRIDOCE |
| 06701 | BETERRABA EM CONSERVA |
| 06801 | LANCHE PEPERI (PRESUNTOS SUINO E DE FRANGO) |
| 06901 | OVO DE CODORNA EM CONSERVA |
| 07001 | SELETA DE LEGUMES EM CONSERVA |
| 07101 | PASTA DE AZEITONA |
| 07102 | PATE DE AZEITONA |
| 07201 | COUVE FLOR EM CONSERVA |
| 07301 | CEBOLINHA CRISTAL EM CONSERVA |
| 07401 | AZEITONA VERDE LIGHT |
| 07501 | SALSICHA VEGETAL EM CONSERVA |
| 07601 | BROCOLIS EM CONSERVA |
| 00101 | FRANGO ABATIDO |
| 00102 | GALINHA ABATIDA |
| 00103 | FRANGO INTEIRO |
| 00104 | FRANGO CAIPIRA |
| 00201 | FRANGO CONGELADO |
| 00202 | GALINHA CONGELADA |
| 00203 | FRANGO TEMPERADO CONGELADO |
| 00204 | GALINHA TEMPERADA CONGELADA |
| 00205 | FRANGO RESFRIADO |
| 00206 | GALINHA RESFRIADA |
| 00207 | FRANGO CONGELADO TEMPERADO |
| 00208 | GALINHA CONGELADA TEMPERADA |
| 00209 | FRANGO INTEIRO CONGELADO |
| 00301 | PARTE DE GALINHA OU FRANGO NAO-ESPECIFICADA |
| 00302 | FRANGO EM PEDACOS NAO-ESPECIFICADOS |
| 00303 | GALINHA EM PEDACOS NAO-ESPECIFICADOS |
| 00304 | CARNE DE GALINHA OU FRANGO NAO-ESPECIFICADA |
| 00305 | CARNE DE FRANGO NAO ESPECIFICADA |
| 00306 | CARNE DE FRANGO DESFIADA |
| 00307 | FRANGO DESFIADO |
| 00401 | PEITO DE GALINHA OU FRANGO |
| 00402 | FILE DE FRANGO |
| 00403 | PEITO DE FRANGO |
| 00404 | ESCALOPE DE FRANGO |
| 00405 | FILE DE PEITO DE FRANGO |
| 00406 | MEDALHAO DE FRANGO |
| 00501 | COXA DE GALINHA OU FRANGO |
| 00502 | SOBRECOXA DE GALINHA OU FRANGO |
| 00503 | COXA E SOBRECOXA DE GALINHA OU FRANGO |
| 00504 | COXA DE FRANGO |
| 00505 | SOBRECOXA DE FRANGO |
| 00506 | COXA E SOBRECOXA DE FRANGO |
| 00601 | DORSO DE GALINHA OU FRANGO |
| 00602 | CARCACA DE GALINHA OU FRANGO |
| 00603 | DORSO DE FRANGO |
| 00604 | CARCACA FRANGO |
| 00701 | ASA DE GALINHA OU FRANGO |
| 00702 | DRUMETE DE GALINHA OU FRANGO |
| 00703 | ASA DE FRANGO |
| 00704 | ASA DE GALINHA |
| 00705 | ASA DE FRANGO TEMPERADO |
| 00706 | DRUMETE DE FRANGO |
| 00707 | COXINHA DA ASA DE GALINHA OU FRANGO |
| 00708 | ASINHA DE FRANGO |
| 00709 | COXA DE ASA DE FRANGO |
| 00801 | PESCOCO DE GALINHA OU FRANGO |
| 00802 | PESCOCO DE FRANGO |
| 00901 | PE DE GALINHA OU FRANGO |
| 00902 | PE DE FRANGO |
| 01001 | MIUDO DE GALINHA OU FRANGO NAO-ESPECIFICADO |
| 01002 | MIUDO DE FRANGO NAO-ESPECIFICADO |
| 01101 | MOELA DE GALINHA OU FRANGO |
| 01102 | MOELA DE FRANGO |
| 01201 | CORACAO DE GALINHA OU FRANGO |
| 01202 | CORACAO DE FRANGO |
| 01301 | FIGADO DE GALINHA OU FRANGO |
| 01302 | FIGADO DE FRANGO |
| 01401 | FRANGO VIVO |
| 01402 | GALINHA VIVA |
| 01403 | GALINHA EM PE |
| 01404 | FRANGO EM PE |
| 01501 | PERU ABATIDO |
| 01601 | PERU CONGELADO |
| 01602 | PERU TEMPERADO CONGELADO |
| 01603 | PERU CONGELADO TEMPERADO |
| 01701 | PARTE DE PERU NAO-ESPECIFICADA |
| 01702 | PERU EM PEDACO NAO-ESPECIFICADO |
| 01703 | CARNE DE PERU NAO-ESPECIFICADA |
| 01801 | PEITO DE PERU |
| 01802 | PEITO DE PERU DEFUMADO |
| 01901 | COXA DE PERU |
| 01902 | SOBRECOXA DE PERU |
| 02001 | DORSO DE PERU |
| 02002 | CARCACA DE PERU |
| 02101 | ASA DE PERU |
| 02201 | PERU VIVO |
| 02202 | PERU EM PE |
| 02301 | COXA DE AVE NAO-ESPECIFICADA |
| 02401 | COXA E SOBRECOXA DE AVE NAO-ESPECIFICADA |
| 02501 | COXA E ASA DE GALINHA OU FRANGO |
| 02502 | COXA E ASA DE FRANGO |
| 02601 | COXA E PEITO DE GALINHA OU FRANGO |
| 02602 | COXA E PEITO DE FRANGO |
| 02701 | CHESTER |
| 02702 | TENDER |
| 02801 | NAMBU ABATIDO, CONGELADO OU VIVO |
| 02802 | NAMBU CONGELADO |
| 02803 | NAMBU VIVO |
| 02804 | INHAMBU ABATIDO |
| 02901 | NUGGETS DE FRANGO |
| 02902 | STEAK DE FRANGO |
| 02903 | NUGGETS DE GALINHA |
| 02904 | STEAK DE GALINHA |
| 03001 | CODORNA ABATIDA, CONGELADA OU VIVA |
| 03002 | CODORNA CONGELADA |
| 03003 | CODORNA VIVA |
| 03101 | PERU EMPANADO |
| 03201 | PE E ASA DE GALINHA OU FRANGO |
| 03202 | PE E ASA DE FRANGO |
| 03301 | OVO DE GALINHA |
| 03302 | OVOS DE GALINHA |
| 03303 | OVO DE GALINHA CAIPIRA |
| 03304 | OVOS DE GALINHA BRANCO |
| 03305 | OVOS DE GALINHA VERMELHO |
| 03401 | OVO DE PATA |
| 03501 | OVO DE CODORNA |
| 03601 | COSTELA E PE DE GALINHA OU FRANGO |
| 03602 | COSTELA E PE DE FRANGO |
| 03701 | OUTRA AVE ABATIDA, CONGELADA OU VIVA |
| 03702 | GALINHA DE ANGOLA ABATIDA, CONGELADA OU VIVA |
| 03703 | CAPOTE ABATIDA, CONGELADA OU VIVA |
| 03704 | PERDIZ ABATIDA, CONGELADA OU VIVA |
| 03705 | GANSO ABATIDO, CONGELADO OU VIVO |
| 03706 | POMBO ABATIDO, CONGELADO OU VIVO |
| 03707 | MUTUM ABATIDO, CONGELADO OU VIVO |
| 03708 | AVOANTE ABATIDA, CONGELADA OU VIVA |
| 03709 | OUTRA AVE CONGELADA |
| 03710 | OUTRA AVE VIVA |
| 03711 | GALINHA DE ANGOLA CONGELADA |
| 03712 | GALINHA DE ANGOLA VIVA |
| 03713 | CAPOTE CONGELADA |
| 03714 | CAPOTE VIVA |
| 03715 | PERDIZ CONGELADA |
| 03716 | PERDIZ VIVA |
| 03717 | GANSO CONGELADO |
| 03718 | GANSO VIVO |
| 03719 | POMBO CONGELADO |
| 03720 | POMBO VIVO |
| 03721 | MUTUM CONGELADO |
| 03722 | MUTUM VIVO |
| 03723 | AVOANTE CONGELADA |
| 03724 | AVOANTE VIVA |
| 03725 | JACANA ABATIDA, CONGELADA OU VIVA |
| 03726 | INAMBU ABATIDA, CONGELADA OU VIVA |
| 03727 | GARCA ABATIDA, CONGELADA OU VIVA |
| 03728 | JACU ABATIDO |
| 03729 | GALINHA DAGUA |
| 03801 | OSSO DE GALINHA |
| 03901 | PATO ABATIDO, CONGELADO OU VIVO |
| 03902 | PATO VIVO |
| 03903 | PATO EM PE |
| 03904 | PATO ABATIDO |
| 03905 | PATO CONGELADO |
| 03906 | PARTE DE PATO (NAO-ESPECIFICADA) |
| 03907 | PATO EM PEDACOS (NAO-ESPECIFICADOS) |
| 03908 | PEITO DE PATO |
| 03909 | COXA DE PATO |
| 03910 | SOBRECOXA DE PATO |
| 03911 | DORSO DE PATO |
| 03912 | CARCACA DE PATO |
| 03913 | CARNE DE PATO NAO-ESPECIFICADA |
| 04001 | CABECA DE GALINHA, PATO, PERU, MARRECO, GALINHA DE ANGOLA |
| 04101 | PESCOCO DE PERU |
| 04201 | SOBRECOXA NAO-ESPECIFICADA |
| 04301 | OVO DE MARRECA |
| 04401 | OVO DE TRACAJA |
| 04501 | OVO DE PERU |
| 04601 | OSSO DE PERU |
| 04701 | RIM DE GALINHA |
| 04801 | SAMBIQUIRA DE GALINHA OU FRANGO |
| 04901 | SAMBIQUIRA DE PERU |
| 05001 | SAMBIQUIRA NAO-ESPECIFICADA |
| 05101 | ROLE DE PERU |
| 05201 | COXA E ASA NAO-ESPECIFICADA |
| 05301 | COXA E ASA DE PERU |
| 05401 | PEITO DE PERU LIGHT |
| 05501 | FRANGO INTEIRO ORGANICO |
| 05502 | FRANGO ABATIDO ORGANICO |
| 05503 | GALINHA ABATIDA ORGANICA |
| 05504 | FRANGO CAIPIRA ORGANICO |
| 05601 | FRANGO CONGELADO ORGANICO |
| 05602 | GALINHA CONGELADA ORGANICA |
| 05603 | FRANGO TEMPERADO CONGELADO ORGANICO |
| 05604 | GALINHA TEMPERADA CONGELADA ORGANICA |
| 05605 | FRANGO RESFRIADO ORGANICO |
| 05606 | GALINHA RESFRIADA ORGANICA |
| 05607 | FRANGO CONGELADO TEMPERADO ORGANICO |
| 05608 | GALINHA CONGELADA TEMPERADA ORGANICA |
| 05701 | PEITO DE FRANGO ORGANICO |
| 05702 | FILE DE FRANGO ORGANICO |
| 05703 | FILE DE GALINHA OU FRANGO ORGANICO |
| 05704 | PEITO DE GALINHA OU FRANGO ORGANICO |
| 05801 | COXA E SOBRECOXA DE FRANGO ORGANICA |
| 05802 | COXA DE GALINHA OU FRANGO ORGANICA |
| 05803 | SOBRECOXA DE GALINHA OU FRANGO ORGANICA |
| 05804 | COXA DE FRANGO ORGANICA |
| 05805 | SOBRECOXA DE FRANGO ORGANICA |
| 05806 | COXA E SOBRECOXA DE GALINHA OU FRANGO ORGANICA |
| 05901 | PARTE DE FRANGO ORGANICO NAO-ESPECIFICADA |
| 05902 | FRANGO EM PEDACOS NAO-ESPECIFICADOS ORGANICO |
| 05903 | GALINHA EM PEDACOS NAO-ESPECIFICADOS ORGANICA |
| 05904 | CARNE DE GALINHA OU FRANGO NAO-ESPECIFICADA ORGANICA |
| 05905 | PARTE DE GALINHA OU FRANGO NAO-ESPECIFICADA ORGANICA |
| 06001 | MARRECO ABATIDO, CONGELADO OU VIVO |
| 06002 | CARNE DE MARRECO |
| 06101 | CORACAO DE PERU |
| 06201 | CARNE MOIDA DE FRANGO |
| 06301 | OVO DE GUINE |
| 06401 | FRANGO ORGANICO INTEIRO |
| 06501 | OVO DE GALINHA ORGANICO |
| 06601 | PELE DE FRANGO |
| 06701 | OVO DE JACARE |
| 06801 | PEITO DE FRANGO LIGHT |
| 06901 | CARNE DE AVESTRUZ |
| 00101 | LEITE DE VACA INTEGRAL |
| 00102 | LEITE TIPO B INTEGRAL |
| 00103 | LEITE TIPO C INTEGRAL |
| 00104 | LEITE ESPECIAL INTEGRAL |
| 00105 | LEITE LONGA VIDA INTEGRAL |
| 00106 | LEITE GLUT LONGA VIDA INTEGRAL |
| 00107 | LEITE TIPO A INTEGRAL |
| 00108 | LEITE PASTEURIZADO INTEGRAL |
| 00109 | LEITE ESTERILIZADO INTEGRAL |
| 00110 | LEITE INTEGRAL |
| 00111 | LEITE DE VACA PASTEURIZADO INTEGRAL |
| 00112 | LEITE TIPO C COMUM |
| 00201 | LEITE DE VACA FRESCO |
| 00202 | LEITE DA ROCA |
| 00203 | LEITE DE VACA IN NATURA |
| 00204 | LEITE FRESCO |
| 00205 | LEITE IN NATURA |
| 00301 | LEITE DE CABRA |
| 00401 | LEITE DE BUFALA |
| 00501 | LEITE DE JUMENTA |
| 00601 | LEITE EM PO INTEGRAL |
| 00602 | LEITE EM PO NINHO INTEGRAL |
| 00603 | LEITE EM PO GLORIA INTEGRAL |
| 00604 | LEITE EM PO NINHO INSTANTANEO INTEGRAL |
| 00605 | LEITE EM PO NESTOGENO 2. SEMESTRE INTEGRAL |
| 00606 | LEITE EM PO LACTOGENO INTEGRAL |
| 00607 | LEITE EM PO NANON INTEGRAL |
| 00608 | LEITE EM PO FRIGOR INTEGRAL |
| 00701 | LEITE EM PO DESENGORDURADO |
| 00702 | LEITE EM PO PELARGON DESENGORDURADO |
| 00703 | LEITE EM PO ELEDON DESENGORDURADO |
| 00704 | LEITE EM PO MOCOCA DESENGORDURADO |
| 00705 | LEITE EM PO DESNATADO OU DESENGORDURADO |
| 00706 | LEITE EM PO SEMILKO DESENGORDURADO |
| 00707 | LEITE EM PO NESTOGENO 1. SEMESTRE DESENGORDURADO |
| 00708 | LEITE EM PO MOLICO DESENGORDURADO |
| 00709 | LEITE EM PO PRODIETON DESENGORDURADO |
| 00710 | LEITE EM PO DESNATADO |
| 00801 | LEITE EM PO NAO-ESPECIFICADO |
| 00901 | LEITE CONDENSADO |
| 00902 | LEITE MOCA |
| 00903 | LEITE BEIJINHO |
| 00904 | MOCA FESTA |
| 01001 | CREME DE LEITE |
| 01101 | CHANTILLY |
| 01102 | CHANTIBON |
| 01201 | IOGURTE DE QUALQUER SABOR |
| 01202 | IOGURTE DANONE |
| 01203 | IOGURTE NATURAL |
| 01204 | DANONE IOGURTE |
| 01205 | DANONINHO |
| 01206 | PETIT SUISSE (IOGURTE) |
| 01207 | CHAMBINHO (IOGURTE) |
| 01208 | IOGURTE ACTIVIA |
| 01209 | DANONETE |
| 01210 | IOGURTE DESNATADO |
| 01211 | IOGURTE LIQUIDO |
| 01212 | ACTIVIA (IOGURTE) |
| 01301 | YAKULT DE QUALQUER SABOR |
| 01302 | LEITE FERMENTADO YAKULT |
| 01303 | BEBIDA LACTEA |
| 01304 | LEITE FERMENTADO |
| 01305 | BEBIDA LACTEA DE QUALQUER SABOR |
| 01306 | BEBIDA LACTEA COM FRUTA |
| 01307 | BEBIDA LACTEA DE FRUTA |
| 01308 | LEITE FERMENTADO COM SUCRILHOS |
| 01309 | LEITE FERMENTADO INTEGRAL |
| 01401 | COALHADA |
| 01402 | COALHADA DESNATADA |
| 01403 | COALHADA COM ABACAXI |
| 01404 | COALHADA COM AMEIXA |
| 01405 | COALHADA COM MORANGO |
| 01501 | MANTEIGA COM OU SEM SAL |
| 01502 | MANTEIGA COM SAL |
| 01503 | MANTEIGA SEM SAL |
| 01504 | MANTEIGA DA TERRA |
| 01505 | MANTEIGA DE GARRAFA |
| 01506 | MANTEIGA DE LEITE |
| 01507 | MANTEIGA |
| 01508 | MANTEIGA CASEIRA |
| 01509 | MANTEIGA DO SERTAO |
| 01601 | MARGARINA VEGETAL COM OU SEM SAL |
| 01602 | MARGARINA COM OU SEM SAL |
| 01603 | MARGARINA COM SAL |
| 01604 | MARGARINA DE MILHO |
| 01605 | MARGARINA DE SOJA |
| 01606 | MARGARINA SEM SAL |
| 01607 | MARGARINA VEGETAL COM SAL |
| 01608 | MARGARINA VEGETAL SEM SAL |
| 01609 | MARGARINA |
| 01701 | QUEIJO PRATO |
| 01702 | QUEIJO TIPO LANCHE |
| 01703 | QUEIJO COLONIAL |
| 01704 | QUEIJO LANCHE |
| 01705 | QUEIJO DE COLONIA |
| 01706 | QUEIJO BOLA |
| 01801 | QUEIJO MUZARELLA |
| 01802 | MUZARELLA |
| 01803 | MUSSARELA |
| 01804 | QUEIJO MUSSARELA |
| 01805 | MUSSARELA FATIADA |
| 01806 | RETALHO DE MUSSARELA |
| 01807 | MUSSARELA DE BUFALA |
| 01808 | QUEIJO MUSSARELA FATIADO |
| 01809 | QUEIJO DE BUFALA (MUSSARELA) |
| 01901 | QUEIJO DO REINO |
| 01902 | QUEIJO PALMIRA |
| 01903 | QUEIJO CUIA |
| 02001 | QUEIJO DE MINAS |
| 02002 | QUEIJO MINAS |
| 02003 | QUEIJO DE MANTEIGA |
| 02004 | QUECHIMIA |
| 02005 | QUEIJO DE COALHO |
| 02006 | QUEIJO TIPO MINAS |
| 02007 | QUEIJO MINAS CURADO |
| 02008 | QUEIJO CANASTRA |
| 02009 | QUEIJO DE MINAS CURADO |
| 02010 | QUEIJO DO SERRO |
| 02011 | QUEIJO CATIARA |
| 02012 | QUEIJO COALHO |
| 02013 | QUEIJO TIPO ESTEPE |
| 02101 | OUTROS QUEIJOS |
| 02102 | QUEIJO SUICO |
| 02103 | QUEIJO DE TRANCA |
| 02104 | QUEIJO CAVALO |
| 02105 | QUEIJO TIPO GOLDA |
| 02106 | QUEIJO QUARCK |
| 02107 | QUEIJO TIPO QUARCK |
| 02108 | QUEIJO MAASDAMER |
| 02109 | QUEIJO FOL EPI |
| 02110 | NUGGET DE QUEIJO |
| 02111 | QUEIJO ESTEPE |
| 02201 | QUEIJO RICOTA |
| 02301 | LEITE DE SOJA COM SABOR |
| 02302 | MAMY |
| 02303 | ADES ORIGINAL |
| 02304 | LEITE DE SOJA INTEGRAL |
| 02305 | LEITE DE SOJA NATURAL |
| 02306 | LEITE DE SOJA |
| 02307 | BEBIDA DE SOJA |
| 02308 | IOGURTE DE SOJA |
| 02309 | SUCO DE SOJA |
| 02310 | TONYU |
| 02401 | QUEIJO PARMEZON |
| 02402 | QUEIJO PARMEZAO |
| 02403 | QUEIJO RALADO |
| 02404 | QUEIJO PARMESAO |
| 02405 | QUEIJO PARMESON |
| 02501 | QUEIJO PROVOLONE |
| 02601 | QUEIJO CAMEMBERT |
| 02602 | QUEIJO ROQUEFORT |
| 02701 | QUEIJO DE MARAJO |
| 02801 | QUEIJO POLENGUINHO |
| 02802 | QUEIJO FUNDIDO |
| 02803 | QUEIJO PASTEURIZADO |
| 02804 | QUEIJO PASTEURIZADO POLENGUINHO |
| 02805 | QUEIJO POLENGUINHO PASTEURIZADO |
| 02806 | QUEIJO ALPESTRE (FUNDIDO) |
| 02901 | REQUEIJAO |
| 02902 | QUEIJO CREMOSO |
| 02903 | QUEIJO TIPO CREME |
| 02904 | QUEIJO EM PASTA |
| 02905 | QUEIJO TIPO DANONINHO |
| 02906 | QUEIJO DANONINHO |
| 02907 | REQUEIJAO CREMOSO |
| 02908 | REQUEIJAO CATUPIRI |
| 02909 | QUEIJO MANTEIGA |
| 03001 | QUEIJO NAO-ESPECIFICADO |
| 03101 | LEITE COM SABOR |
| 03102 | LEITE ACHOCOLATADO |
| 03103 | LEITE LONGA VIDA COM SABOR |
| 03104 | ACHOCOLATADO EM LEITE |
| 03105 | LEITE VITAMINADO |
| 03106 | LEITE VITAMINADO TODDYNHO |
| 03107 | LEITE TODDYNHO |
| 03108 | SUPERLAC |
| 03109 | LEITE AROMATIZADO |
| 03110 | BEBIDA ACHOCOLATADA |
| 03111 | NESCAU LIQUIDO |
| 03112 | TODDYNHO LIQUIDO |
| 03113 | ACHOCOLATADO LIQUIDO |
| 03201 | NATA DOCE OU SALGADA |
| 03202 | NATA COLONIAL |
| 03203 | NATA DOCE |
| 03204 | NATA SALGADA |
| 03301 | QUEIJO GORGONZOLA |
| 03401 | QUEIJO DE SOJA |
| 03402 | TOFU |
| 03403 | OTOFU |
| 03404 | QUEIJO DE FEIJAO SOJA |
| 03501 | LEITE DE SOJA EM PO |
| 03502 | EXTRATO DE SOJA EM PO |
| 03601 | LEITE DE VACA DESNATADO |
| 03602 | LEITE DE VACA PASTEURIZADO DESNATADO |
| 03603 | LEITE DESNATADO DE VACA |
| 03604 | LEITE LIGHT DE VACA |
| 03605 | LEITE DE VACA LIGHT |
| 03701 | LEITE SEMIDESNATADO DE VACA |
| 03702 | LEITE DE VACA SEMIDESNATADO |
| 03703 | LEITE DE VACA PASTEURIZADO SEMIDESNATADO |
| 03801 | LEITE NAO-ESPECIFICADO PASTEURIZADO |
| 03802 | LEITE PASTEURIZADO NAO-ESPECIFICADO |
| 03803 | LEITE DE VACA PASTEURIZADO NAO ESPECIFICADO |
| 03901 | IOGURTE LIGHT |
| 03902 | IOGURTE DANONE LIGHT |
| 03903 | IOGURTE DESNATADO (LIGHT) |
| 03904 | IOGURTE DE QUALQUER SABOR LIGHT |
| 03905 | DANONE IOGURTE LIGHT |
| 03906 | CORPUS BREAK LIGHT |
| 03907 | BIO FIBRAS LIQUIDO LIGHT |
| 04001 | IOGURTE DIET |
| 04002 | IOGURTE DANONE DIET |
| 04003 | IOGURTE DE QUALQUER SABOR DIET |
| 04004 | DANONE IOGURTE DIET |
| 04005 | IOGURTE DIETETICO |
| 04006 | IOGURTE DANONE DIETETICO |
| 04007 | IOGURTE DE QUALQUER SABOR DIETETICO |
| 04008 | DANONE IOGURTE DIETETICO |
| 04101 | MARGARINA VEGETAL COM OU SEM SAL DIET |
| 04102 | MARGARINA COM OU SEM SAL DIET |
| 04103 | MARGARINA COM SAL DIET |
| 04104 | MARGARINA DE MILHO DIET |
| 04105 | MARGARINA DE SOJA DIET |
| 04106 | MARGARINA SEM SAL DIET |
| 04107 | MARGARINA VEGETAL COM SAL DIET |
| 04108 | MARGARINA VEGETAL SEM SAL DIET |
| 04109 | MARGARINA VEGETAL COM OU SEM SAL DIETETICA |
| 04110 | MARGARINA COM OU SEM SAL DIETETICA |
| 04111 | MARGARINA COM SAL DIETETICA |
| 04112 | MARGARINA DE MILHO DIETETICA |
| 04113 | MARGARINA DE SOJA DIETETICA |
| 04114 | MARGARINA SEM SAL DIETETICA |
| 04115 | MARGARINA VEGETAL COM SAL DIETETICA |
| 04116 | MARGARINA VEGETAL SEM SAL DIETETICA |
| 04201 | MARGARINAVEGETAL COM OU SEM SAL LIGHT |
| 04202 | MARGARINA COM OU SEM SAL LIGHT |
| 04203 | MARGARINA COM SAL LIGHT |
| 04204 | MARGARINA DE MILHO LIGHT |
| 04205 | MARGARINA DE SOJA LIGHT |
| 04206 | MARGARINA SEM SAL LIGHT |
| 04207 | MARGARINA VEGETAL COM SAL LIGHT |
| 04208 | MARGARINA VEGETAL SEM SAL LIGHT |
| 04301 | LEITE DE VACA INTEGRAL ORGANICO |
| 04302 | LEITE TIPO B INTEGRAL ORGANICO |
| 04303 | LEITE TIPO C INTEGRAL ORGANICO |
| 04304 | LEITE ESPECIAL INTEGRAL ORGANICO |
| 04305 | LEITE LONGA VIDA INTEGRAL ORGANICO |
| 04306 | LEITE GLUT LONGA VIDA INTEGRAL ORGANICO |
| 04307 | LEITE TIPO A INTEGRAL ORGANICO |
| 04308 | LEITE PASTEURIZADO INTEGRAL ORGANICO |
| 04309 | LEITE ESTERILIZADO INTEGRAL ORGANICO |
| 04310 | LEITE INTEGRAL ORGANICO |
| 04311 | LEITE DE VACA PASTEURIZADO INTEGRAL ORGANICO |
| 04401 | LEITE DE VACA FRESCO ORGANICO |
| 04402 | LEITE DA ROCA ORGANICO |
| 04403 | LEITE DE VACA IN NATURA ORGANICO |
| 04501 | LEITE DE VACA DESNATADO ORGANICO |
| 04502 | LEITE DE VACA PASTEURIZADO DESNATADO ORGANICO |
| 04503 | LEITE DESNATADO DE VACA ORGANICO |
| 04601 | LEITE SEMIDESNATADO DE VACA ORGANICO |
| 04602 | LEITE DE VACA SEMIDESNATADO ORGANICO |
| 04603 | LEITE DE VACA PASTEURIZADO SEMIDESNATADO ORGANICO |
| 04701 | CREME DE LEITE ORGANICO |
| 04801 | IOGURTE ORGANICO |
| 04802 | IOGURTE DANONE ORGANICO |
| 04803 | IOGURTE DESNATADO ORGANICO |
| 04804 | IOGURTE NATURAL ORGANICO |
| 04805 | DANONE IOGURTE ORGANICO |
| 04901 | IOGURTE ORGANICO LIGHT |
| 05001 | IOGURTE ORGANICO DIET |
| 05002 | IOGURTE DIETETICO ORGANICO |
| 05101 | QUEIJO MUZARELLA LIGHT |
| 05102 | MUZARELLA LIGHT |
| 05103 | MUSSARELA LIGHT |
| 05104 | QUEIJO MUSSARELA LIGHT |
| 05105 | MUSSARELA FATIADA LIGHT |
| 05106 | RETALHO DE MUSSARELA LIGHT |
| 05107 | MUSSARELA DE BUFALA LIGHT |
| 05201 | LEITE DE SOJA COM SABOR LIGHT |
| 05202 | MAMY LIGHT |
| 05203 | ADES LIGHT |
| 05301 | LEITE DE CABRA LIGHT |
| 05401 | LEITE CONDENSADO LIGHT |
| 05402 | LEITE MOCA LIGHT |
| 05403 | LEITE BEIJINHO LIGHT |
| 05404 | MOCA FESTA LIGHT |
| 05501 | LEITE CONDENSADO DIET |
| 05502 | LEITE MOCA DIET |
| 05503 | LEITE BEIJINHO DIET |
| 05504 | MOCA FESTA DIET |
| 05505 | LEITE CONDENSADO DIETETICO |
| 05506 | LEITE MOCA DIETETICO |
| 05507 | LEITE BEIJINHO DIETETICO |
| 05508 | MOCA FESTA DIETETICO |
| 05601 | CREME DE LEITE LIGHT |
| 05701 | CHANTILLY LIGHT |
| 05702 | CHANTIBON LIGHT |
| 05801 | CHANTILLY DIET |
| 05802 | CHANTIBON DIET |
| 05803 | CHANTILLY DIETETICO |
| 05804 | CHANTIBON DIETETICO |
| 05901 | QUEIJO PRATO LIGHT |
| 05902 | QUEIJO TIPO LANCHE LIGHT |
| 05903 | QUEIJO COLONIAL LIGHT |
| 06001 | QUEIJO DE MINAS LIGHT |
| 06002 | QUEIJO MINAS LIGHT |
| 06003 | QUEIJO DE MANTEIGA LIGHT |
| 06004 | QUECHIMIA LIGHT |
| 06005 | QUEIJO DE COALHO LIGHT |
| 06006 | QUEIJO TIPO MINAS LIGHT |
| 06007 | QUEIJO MINAS CURADO LIGHT |
| 06008 | QUEIJO CANASTRA LIGHT |
| 06009 | QUEIJO DE MINAS CURADO LIGHT |
| 06010 | QUEIJO DO SERRO LIGHT |
| 06011 | QUEIJO CATIARA LIGHT |
| 06101 | OUTROS QUEIJOS LIGHT |
| 06102 | QUEIJO SUICO LIGHT |
| 06103 | QUEIJO DE TRANCA LIGHT |
| 06104 | QUEIJO CAVALO LIGHT |
| 06105 | QUEIJO TIPO GOLDA LIGHT |
| 06106 | QUEIJO QUARCK LIGHT |
| 06107 | QUEIJO TIPO QUARCK LIGHT |
| 06108 | QUEIJO MAASDAMER LIGHT |
| 06109 | QUEIJO FOL EPI LIGHT |
| 06201 | QUEIJO RICOTA LIGHT |
| 06301 | QUEIJO PARMEZON LIGHT |
| 06302 | QUEIJO PARMEZAO LIGHT |
| 06303 | QUEIJO RALADO LIGHT |
| 06304 | QUEIJO PARMESAO LIGHT |
| 06305 | QUEIJO PARMESON LIGHT |
| 06401 | QUEIJO POLENGUINHO LIGHT |
| 06402 | QUEIJO FUNDIDO LIGHT |
| 06403 | QUEIJO PASTEURIZADO LIGHT |
| 06404 | QUEIJO PASTEURIZADO POLENGUINHO LIGHT |
| 06405 | QUEIJO POLENGUINHO PASTEURIZADO LIGHT |
| 06501 | REQUEIJAO LIGHT |
| 06502 | QUEIJO CREMOSO LIGHT |
| 06503 | QUEIJO TIPO CREME LIGHT |
| 06504 | QUEIJO EM PASTA LIGHT |
| 06505 | QUEIJO TIPO DANONINHO LIGHT |
| 06506 | QUEIJO DANONINHO LIGHT |
| 06507 | REQUEIJAO CREMOSO LIGHT |
| 06508 | REQUEIJAO CATUPIRI LIGHT |
| 06601 | QUEIJO NAO-ESPECIFICADO LIGHT |
| 06701 | LEITE COM SABOR LIGHT |
| 06702 | LEITE ACHOCOLATADO LIGHT |
| 06703 | LEITE LONGA VIDA COM SABOR LIGHT |
| 06704 | ACHOCOLATADO EM LEITE LIGHT |
| 06705 | LEITE VITAMINADO LIGHT |
| 06706 | LEITE VITAMINADO TODDYNHO LIGHT |
| 06707 | LEITE TODDYNHO LIGHT |
| 06708 | SUPERLAC LIGHT |
| 06709 | LEITE AROMATIZADO LIGHT |
| 06710 | BEBIDA ACHOCOLATADA LIGHT |
| 06801 | LEITE COM SABOR DIET |
| 06802 | LEITE ACHOCOLATADO DIET |
| 06803 | LEITE LONGA VIDA COM SABOR DIET |
| 06804 | ACHOCOLATADO EM LEITE DIET |
| 06805 | LEITE VITAMINADO DIET |
| 06806 | LEITE VITAMINADO TODDYNHO DIET |
| 06807 | LEITE TODDYNHO DIET |
| 06808 | SUPERLAC DIET |
| 06809 | LEITE AROMATIZADO DIET |
| 06810 | BEBIDA ACHOCOLATADA DIET |
| 06811 | LEITE COM SABOR DIETETICO |
| 06812 | LEITE ACHOCOLATADO DIETETICO |
| 06813 | LEITE LONGA VIDA COM SABOR DIETETICO |
| 06814 | ACHOCOLATADO EM LEITE DIETETICO |
| 06815 | LEITE VITAMINADO DIETETICO |
| 06816 | LEITE VITAMINADO TODDYNHO DIETETICO |
| 06817 | LEITE TODDYNHO DIETETICO |
| 06818 | SUPERLAC DIETETICO |
| 06819 | LEITE AROMATIZADO DIETETICO |
| 06820 | BEBIDA ACHOCOLATADA DIETETICA |
| 06901 | LEITE DE SOJA EM PO LIGHT |
| 07001 | LEITE EM PO COM SABOR |
| 07101 | MANTEIGA COM OU SEM SAL LIGHT |
| 07102 | MANTEIGA COM SAL LIGHT |
| 07103 | MANTEIGA SEM SAL LIGHT |
| 07104 | MANTEIGA DA TERRA LIGHT |
| 07105 | MANTEIGA DE GARRAFA LIGHT |
| 07106 | MANTEIGA DE LEITE LIGHT |
| 07201 | COALHADA LIGHT |
| 07301 | QUEIJO DE MINAS FRESCAL ORGANICO |
| 07302 | QUEIJO MINAS FRESCAL ORGANICO |
| 07303 | QUEIJO DE MANTEIGA FRESCAL ORGANICO |
| 07304 | QUECHIMIA FRESCAL ORGANICO |
| 07305 | QUEIJO DE COALHO FRESCAL ORGANICO |
| 07306 | QUEIJO TIPO MINAS FRESCAL ORGANICO |
| 07307 | QUEIJO MINAS CURADO FRESCAL ORGANICO |
| 07308 | QUEIJO CANASTRA FRESCAL ORGANICO |
| 07309 | QUEIJO DE MINAS CURADO FRESCAL ORGANICO |
| 07310 | QUEIJO DO SERRO FRESCAL ORGANICO |
| 07311 | QUEIJO CATIARA FRESCAL ORGANICO |
| 07401 | LEITE DE CABRA ORGANICO |
| 07501 | CREME DE LEITE DE SOJA |
| 07502 | CREME DE SOJA |
| 07601 | LEITE FERMENTADO DIET |
| 07701 | LEITE EM PO LIGHT |
| 07801 | QUEIJO CHEDAR |
| 07802 | QUEIJO CHEDDAR |
| 07803 | QUEIJO CHEEDAR |
| 07901 | FONDUE DE QUEIJO |
| 08001 | LEITE DE SOJA EM PO DIET |
| 08101 | QUEIJO BRIE |
| 08201 | QUEIJO COTTAGE |
| 08301 | QUEIJO CHANCLICHE |
| 08401 | QUEIJO GOUDA |
| 08501 | CREAM CHEESE |
| 08601 | BEBIDA LACTEA LIGHT |
| 08602 | LEITE FERMENTADO LIGHT |
| 08701 | NATA DOCE OU SALGADA LIGHT |
| 00101 | PAO FRANCES |
| 00102 | PAO DE AGUA |
| 00103 | PAO DE HAMBURGUER |
| 00104 | BISNAGA |
| 00105 | PAO DE SAL |
| 00106 | PAO DE TRIGO |
| 00107 | PAO CACETINHO |
| 00108 | PAO SUICO |
| 00109 | PAO CARECA |
| 00110 | PAO FILAO |
| 00111 | PAO DE SEMOLINA |
| 00112 | PAO BENGALA |
| 00113 | PAO BISNAGA |
| 00114 | BENGALA |
| 00115 | PAO SOVADO |
| 00116 | PAO SOVADO CABRITO |
| 00117 | PAO CAVAQUINHO |
| 00118 | PAO FRANCES BISNAGA |
| 00119 | PAO DE CHA |
| 00120 | PAO PROVENCO |
| 00121 | PAO PROVENCAL |
| 00122 | PAO MASSA FINA |
| 00123 | PAO VITA SALGADO |
| 00124 | PAO CARIOQUINHA |
| 00125 | PAO PRESUNTO |
| 00126 | PAO DE BANHA |
| 00127 | PAO BANQUETE |
| 00128 | PAO BAQUETE |
| 00129 | PAO BAGUETE |
| 00130 | PAO SERIDO |
| 00131 | PAO CARTEIRA (SALGADO) |
| 00132 | PAO SEDA |
| 00133 | PAO TABICA |
| 00134 | PAO PALITO |
| 00135 | PAO SACADURA |
| 00136 | PAO BAIANO |
| 00137 | PAO MANUAL |
| 00138 | MINI PAO |
| 00139 | MINI PAO FRANCES |
| 00140 | MINI BAGUETE |
| 00141 | MINI BENGALA |
| 00142 | PAO MASSA GROSSA |
| 00143 | PAO PIZZA |
| 00144 | PAO RECHEADO (SALGADO) |
| 00145 | PAO CIABATA |
| 00146 | PAO CERVEJINHA |
| 00147 | PAO DE CARA |
| 00148 | PAO PORTUGUES |
| 00149 | PAO SEDINHA |
| 00150 | CHIPA |
| 00151 | PAO CHIPA |
| 00152 | PAO JACO |
| 00153 | PAO CILINDRO |
| 00154 | PAO MINEIRO (SALGADO) |
| 00155 | PAO DE INHAME |
| 00156 | PAO CARIOCA |
| 00201 | PAO DOCE |
| 00202 | PAO ALMOFADINHA |
| 00203 | CHINEQUE |
| 00204 | PAO DOCE COM COCO |
| 00205 | PAO DOCE COMUM |
| 00206 | PAO BOLACHAO |
| 00207 | PAO DOCE COM CREME |
| 00208 | PAO DOCE COM RECHEIO |
| 00209 | PAO DOCE SEM COCO |
| 00210 | PAO DOCE ESPECIAL |
| 00211 | PAO TRANCA COM FAROFA |
| 00212 | PAO DOCE CARECA |
| 00213 | PAO DE LEITE (PAO DOCE) |
| 00214 | PAO CARECA DOCE |
| 00215 | PAO DOCE DE TRIGO |
| 00216 | MASSINHA DOCE (PAO DOCE) |
| 00217 | PAO DOCE DE LEITE |
| 00218 | CHINEQUE COM FAROFA |
| 00219 | PAO LANCHE |
| 00220 | PAO DE LANCHE |
| 00221 | PAO DOCE COM MEL |
| 00222 | PAO DOCE COM OVOS |
| 00223 | PAO DE COCO |
| 00224 | PAO AMANTEIGADO (DOCE) |
| 00225 | XISBAG |
| 00226 | CHISBAG |
| 00227 | PAO SUPER FOFO |
| 00228 | PAO VITA DOCE |
| 00229 | PAO DE MEL |
| 00230 | PAO CARTEIRA (DOCE) |
| 00231 | PAO ALEMAO |
| 00232 | PAO MANDI |
| 00233 | PAO MANDIM |
| 00234 | PANETONE |
| 00235 | PAO MANTEIGA |
| 00236 | PAO RECHEADO (DOCE) |
| 00237 | BISNAGUINHA |
| 00238 | FORROZINHO (PAO DOCE) |
| 00239 | COLOMBA DE PASCOA |
| 00240 | CROISSANT |
| 00241 | CHOCOTONE |
| 00242 | PAO BISNAGUINHA |
| 00243 | PAO MANDINHO |
| 00244 | PAO DE CHOCOLATE |
| 00245 | PAO AUSTRALIANO (DOCE) |
| 00246 | PAO FOLHADO |
| 00247 | PAO MADRILENHO |
| 00248 | PAO CABRITINHO |
| 00301 | PAO DE FORMA DE PADARIA (ADOCICADO) |
| 00302 | PAO DE SANDUICHE DE PADARIA (ADOCICADO) |
| 00401 | PAO DE FORMA DE PADARIA (SALGADO) |
| 00402 | PAO DE SANDUICHE DE PADARIA (SALGADO) |
| 00501 | PAO DE FORMA INDUSTRIALIZADO DE QUALQUER MARCA |
| 00502 | PAO SEVEN BOYS |
| 00503 | PAO PULMEX |
| 00504 | PAO DE FORMA PLUS VITA |
| 00505 | PAO DE FORMA DE LEITE PULLMAN |
| 00506 | PAO DE FORMA SEVEN BOYS |
| 00507 | PAO DE FORMA PETROPOLIS |
| 00508 | PAO PLUS VITA |
| 00509 | PAO DE CAIXA |
| 00510 | PAO INGLES |
| 00511 | PAO RECIFE |
| 00512 | PAO ITALIANO |
| 00513 | PAO PULLMAN |
| 00514 | PAO DE FORMA PULMEX |
| 00515 | PAO DE FORMA PULLMAN |
| 00516 | PAO DE SANDUICHE INDUSTRIALIZADO DE QUALQUER MARCA |
| 00601 | PAO DE ARROZ |
| 00701 | PAO DE GLUTEN |
| 00801 | PAO DE QUEIJO |
| 00901 | PAO DE CACHORRO QUENTE |
| 01001 | PAO DE BATATA |
| 01002 | PAO DE FECULA DE BATATA |
| 01101 | PAO DE MILHO |
| 01102 | PAO DE FORMA DE MILHO |
| 01103 | PAO DE FUBA |
| 01104 | PANHOCA DE MILHO |
| 01105 | PAO BROTE |
| 01106 | BROTE (PAO) |
| 01201 | PAO DE AIPIM |
| 01202 | PAO DE GOMA |
| 01203 | PAO DE MANDIOCA |
| 01204 | PAO DE MACAXEIRA |
| 01301 | PAO DE CENTEIO |
| 01302 | PAO PRETO |
| 01303 | PAO CRIOULO |
| 01401 | PAO INTEGRAL |
| 01402 | PAO DE GRAHAM PLUS VITA |
| 01403 | PAO DE GRAHAM |
| 01404 | PAO INTEGRAL PLUS VITA |
| 01405 | PAO DE FORMA INTEGRAL |
| 01406 | PAO BOA VIDA (INTEGRAL) |
| 01501 | PAO NAO-ESPECIFICADO |
| 01601 | PAO CASEIRO |
| 01701 | PAO DE AVEIA |
| 01801 | PAO DE RABANADA |
| 01802 | RABANADA (PAO) |
| 01901 | TORRADA DE QUALQUER PAO |
| 01902 | PAO TORRADO (TORRADA) |
| 01903 | TORRADA DE QUALQUER TIPO |
| 02001 | ROSCA DOCE |
| 02002 | ROSQUINHA DOCE |
| 02003 | ROSQUINHA DE NATA |
| 02004 | ROSCA DE TRIGO DOCE |
| 02005 | ROSCA DE NATA |
| 02006 | ROSCA DE MILHO |
| 02007 | ROSQUINHA AMANTEIGADA |
| 02008 | ROSQUINHA DE MILHO |
| 02009 | ROSCA DOCE DE TRIGO |
| 02010 | ROSCA DE COCO |
| 02011 | ROSQUINHA DE COCO |
| 02012 | ROSCA DE GLUTEN DOCE |
| 02013 | ROSCA AMANTEIGADA |
| 02014 | ROSCA DE CHOCOLATE |
| 02101 | ROSCA SALGADA |
| 02102 | ROSCA SALGADA DE TRIGO |
| 02103 | ROSCA DE BATATA |
| 02104 | ROSCA DE TRIGO SALGADA |
| 02105 | ROSQUINHA DE BATATA |
| 02106 | ROSQUINHA SALGADA |
| 02107 | ROSCA DE GLUTEN SALGADA |
| 02201 | BISCOITO SALGADO |
| 02202 | BISCOITO DO REINO |
| 02203 | BISCOITO GRISSINI |
| 02204 | BISCOITO CREAM CRACKER |
| 02205 | PRESUNTINHO BISCOITO |
| 02206 | BISCOITO TIPO ITALIANO |
| 02207 | BISCOITO DE AGUA |
| 02208 | BISCOITO QUEIJINHO |
| 02209 | BISCOITO DE COCO SALGADO |
| 02210 | BISCOITO DE CAMARAO |
| 02211 | JOBS DE CAMARAO |
| 02212 | BISCOITO DE POLVILHO SALGADO |
| 02213 | BISCOITO PRESUNTINHO |
| 02214 | BISCOITO INTEGRAL DE AGUA |
| 02215 | BISCOITO SALGADO DE COCO |
| 02216 | BISCOITO BIT |
| 02217 | BISKUIT GRISSINI |
| 02218 | BISCOITO SALGADINHO |
| 02219 | BISCOITO SALGADO DE POLVILHO |
| 02220 | BISCOITO CLUB CRACKER |
| 02221 | SALGADINHO (BISCOITO) |
| 02222 | BISCOITO DE AGUA INTEGRAL |
| 02223 | QUEIJINHO BISCOITO |
| 02224 | BISCOITO ITALIANO |
| 02225 | BOLACHA SALGADA |
| 02226 | BOLACHA DE AGUA E SAL |
| 02227 | CHIPS (SALGADINHOS) |
| 02228 | CROCK (SALGADINHOS) |
| 02229 | OKAKI (BISCOITO SALGADO) |
| 02230 | BACONZITOS |
| 02231 | BISCOITO DE AGUA E SAL |
| 02232 | BISCOITO CREAM CRACKER INTEGRAL |
| 02233 | BISCOITO DE SAL |
| 02234 | BISCOITO SALGADO DE MILHO |
| 02235 | BISCOITO SALGADO DE PIZZA |
| 02236 | BISCOITO SALGADO INTEGRAL |
| 02237 | BOLACHA CREAM CRACK |
| 02238 | BOLACHA INTEGRAL |
| 02239 | BISCOITO DE CEBOLA |
| 02240 | BISCOITO DE QUEIJO |
| 02241 | BISCOITO AGUA E SAL |
| 02242 | BISCOITO CLUB SOCIAL |
| 02243 | BISCOITO CLUBE SOCIAL |
| 02244 | BOLACHA AGUA E SAL |
| 02245 | BOLACHA DE SAL |
| 02246 | SALGADINHO DE MILHO |
| 02247 | SALGADINHO DE QUEIJO |
| 02248 | BISCOITO SALGADO DE GOMA |
| 02249 | MENTIRA (BISCOITO SALGADO) |
| 02250 | BISCOITO RUFFLES SALGADO |
| 02251 | BISCOITO PALITO SALGADO |
| 02301 | BISCOITO DOCE |
| 02302 | CAVACO CHINES |
| 02303 | BISCOITO DE MANTEIGA |
| 02304 | BOLACHA AMANTEIGADA |
| 02305 | SEQUILHO |
| 02306 | TARECO |
| 02307 | BISCOITO DE GERGELIM |
| 02308 | BISCOITO AMANTEIGADO |
| 02309 | SOLDA |
| 02310 | BISCOITO TOSTINE |
| 02311 | TOSTINE (BISCOITO) |
| 02312 | BOLACHA DE FUBA |
| 02313 | BOLACHA DE MANTEIGA |
| 02314 | DEDITOS (BISCOITO) |
| 02315 | BREVIDADE |
| 02316 | BISCOITO DE MAIZENA |
| 02317 | BOLACHA DE MILHO |
| 02318 | SEQUILHO DE MILHO |
| 02319 | BISCOITO DOCE DE QUEIJO |
| 02320 | SEQUILHO DE MAIZENA |
| 02321 | BISCOITO DOCE SORTIDO |
| 02322 | BISCOITO DE FUBA |
| 02323 | BISCOITO DEDITOS |
| 02324 | BISCOITO SORTIDO |
| 02325 | BISCOITO DE COCO DOCE |
| 02326 | BISCOITO CREME |
| 02327 | BISCOITO DE MILHO |
| 02328 | BISCOITO CHAMPANHE |
| 02329 | BISCOITO ROSQUINHA DE COCO |
| 02330 | BISCOITO DE QUEIJO DOCE |
| 02331 | BISCOITO BROA DE COCO |
| 02332 | BISCOITO ROSCA DE COCO |
| 02333 | BOLACHA COM LEITE |
| 02334 | BOLACHA DOCE |
| 02335 | MARIA MALUCA (BOLACHA DOCE) |
| 02336 | BISCOITO DE CHAMPAGNE |
| 02337 | BISCOITO MARIA |
| 02338 | BISCOITO DE LEITE |
| 02339 | BISCOITO DE COCO |
| 02340 | BOLACHA DE COCO |
| 02341 | BISCOITO DE CHOCOLATE |
| 02342 | BISCOITO DE TAPIOCA |
| 02343 | BISCOITO ROSQUINHA DE CHOCOLATE |
| 02344 | BISCOITO ROSQUINHA DE LEITE |
| 02345 | BOLACHA DE CHOCOLATE |
| 02346 | BOLACHA DE LEITE |
| 02347 | BOLACHA DE LEITE E MEL |
| 02348 | BOLACHA DE MAIZENA |
| 02349 | BOLACHAO DOCE |
| 02350 | BISCOITO DE NATA |
| 02351 | CUECA VIRADA (BISCOITO DOCE) |
| 02352 | BISCOITO ORELHA DE GATO |
| 02353 | BOLACHA PALITO |
| 02354 | CAVACO |
| 02355 | BISCOITO DE GOMA |
| 02356 | BOLACHA DE MEL |
| 02357 | MARIA MALUCA |
| 02358 | ORELHA DE GATO (BISCOITO DOCE) |
| 02359 | BOLACHAO DE COCO |
| 02360 | BISCOITO PALITO DE CHOCOLATE |
| 02361 | BOLACHA DE GOMA |
| 02362 | CROSTOLI |
| 02363 | MENTIRA DOCE |
| 02401 | BISCOITO DE POLVILHO DOCE |
| 02402 | BISCOITO DE POLVILHO SEQUILHO |
| 02403 | BISCOITO QUEBRA QUEBRA |
| 02404 | BISCOITO DE ARARUTA |
| 02405 | QUEBRA QUEBRA |
| 02406 | SEQUILHO DE POLVILHO |
| 02407 | BISCOITO DOCE DE POLVILHO |
| 02408 | PETA |
| 02409 | PETA DE POLVILHO |
| 02410 | PETA DE GOMA |
| 02411 | PETAS |
| 02501 | BOLO DE QUALQUER MARCA E SABOR |
| 02502 | ROCAMBOLE |
| 02503 | CUCA DE QUALQUER TIPO |
| 02504 | FATIA DE BOLO DE QUALQUER SABOR |
| 02505 | BOLO DE OVOS |
| 02506 | BOLO DE QUEIJO |
| 02507 | BOLINHO DE QUEIJO |
| 02601 | BOLO DE MILHO |
| 02602 | BROA DE MILHO |
| 02603 | BROINHA DE MILHO ADOCICADA |
| 02604 | AMIDOMIL (BOLINHO) |
| 02605 | ANGUSOR DE MILHO |
| 02606 | BROA DE FUBA |
| 02607 | BROINHA DE FUBA |
| 02608 | BOLINHO AMIDOMIL |
| 02609 | GRUSTOLI (BOLINHO DOCE) |
| 02610 | BOLINHO DE MILHO |
| 02611 | BOLINHO DE FUBA |
| 02612 | BOLO DE FUBA |
| 02701 | BOLO DE AIPIM |
| 02702 | BOLO DE TAPIOCA |
| 02703 | BOLO DE MACAXEIRA |
| 02704 | BOLO DE GOMA |
| 02705 | BROA DE MANDIOCA |
| 02706 | BROINHA DE MANDIOCA |
| 02707 | BROA DE GOMA |
| 02708 | BOLO DE MANDIOCA |
| 02801 | BOLO DE BATATA DOCE |
| 02901 | BOLO DE ARROZ |
| 03001 | BOLO DE CENTEIO |
| 03002 | BROA DE CENTEIO |
| 03003 | BROINHA DE CENTEIO |
| 03004 | BROA PRETA |
| 03005 | BROINHA PRETA |
| 03101 | BOLO DE CARA |
| 03102 | BROA DE CARA |
| 03103 | BROINHA DE CARA |
| 03201 | BOLO DE INHAME |
| 03202 | BROA DE INHAME |
| 03203 | BROINHA DE INHAME |
| 03301 | MISTURAS INDUSTRIAIS DE PAES |
| 03302 | MASSAS INDUSTRIAIS DE PAES |
| 03401 | MISTURAS INDUSTRIAIS DE BOLOS, TORTAS, BISCOITOS, ETC. |
| 03402 | MASSAS INDUSTRIAIS DE BOLOS, TORTAS, BISCOITOS, ETC. |
| 03403 | MASSA FOLHADA |
| 03404 | MASSA PARA BOLO |
| 03405 | MASSA DE BOLO |
| 03406 | MISTURA DE BOLO |
| 03407 | MISTURA PARA BOLO |
| 03408 | PREPARO PARA BOLO |
| 03501 | TORTAS DOCES DE QUALQUER SABOR |
| 03502 | MORENINHA (TORTA) |
| 03601 | TORTAS SALGADAS DE QUALQUER SABOR |
| 03701 | SONHO |
| 03702 | FILHOS (BOLINHO DE FARINHA DE TRIGO E OVOS) |
| 03801 | BOLO DE CHOCOLATE |
| 03901 | PAO BRIOCHE |
| 03902 | BRIOCHE |
| 03903 | PAO DE MASSA FOLHADA |
| 04001 | BOLO DE LARANJA |
| 04101 | BOLO DE COCO |
| 04201 | BOLO DE CENOURA |
| 04301 | PAO DE SOJA |
| 04302 | PAO DE FORMA DE SOJA |
| 04303 | PAO DE SOJA DE FORMA |
| 04401 | PAO DE CENOURA, ALHO, CEBOLA, TORRESMO, ETC. |
| 04402 | PAO DE TORRESMO |
| 04403 | PAO DE ALHO |
| 04404 | PAO DE CEBOLA |
| 04405 | PAO DE GERGELIM |
| 04406 | PAO COM ERVA FINA |
| 04407 | PAO DE ERVA DOCE |
| 04408 | PAO DE CALABRESA |
| 04409 | PAO DE FRIOS |
| 04410 | PAO DE LARANJA |
| 04411 | PAO DE PASSAS |
| 04412 | PAO DE GOIABA |
| 04501 | BROA DE COCO |
| 04502 | BROINHA DE COCO |
| 04601 | BOLO DE TRIGO |
| 04602 | PAO DE LO |
| 04701 | BROA |
| 04801 | BISCOITO RECHEADO |
| 04802 | MIRABEL |
| 04803 | BISCOITO RECHEADO TOSTINE |
| 04804 | BISCOITO LANCHE MIRABEL |
| 04805 | BISCOITO WAFFER |
| 04806 | LANCHE MIRABEL |
| 04807 | WAFFER (BISCOITO) |
| 04808 | ROSQUINHA RECHEADA DE QUALQUER SABOR |
| 04809 | ALFAJORES (BISCOITO) |
| 04810 | BOLACHA RECHEADA |
| 04811 | BISCOITO CROCANTE |
| 04812 | BISCOITO MIRABEL |
| 04813 | ROSCA RECHEADA |
| 04814 | BISCOITO RECHEADO DE CHOCOLATE |
| 04815 | BISCOITO RECHEADO DE MORANGO |
| 04816 | CASADINHA (BISCOITO RECHEADO) |
| 04817 | AMANDITA (BISCOITO RECHEADO) |
| 04818 | BEM CASADO (BISCOITO RECHEADO) |
| 04819 | TRAQUINAS (BISCOITO RECHEADO) |
| 04901 | BISCOITO CASEIRO |
| 04902 | BOLACHA CASEIRA |
| 05001 | BROINHA |
| 05101 | BROA DE LEITE |
| 05201 | BISCOITO NAO-ESPECIFICADO |
| 05301 | BISCOITO DE POLVILHO NAO-ESPECIFICADO |
| 05401 | BOLACHA NAO-ESPECIFICADA |
| 05501 | PAO CARTEIRA NAO-ESPECIFICADO |
| 05601 | PAO DE FORMA NAO-ESPECIFICADO |
| 05701 | PAO DE SANDUICHE NAO-ESPECIFICADO |
| 05801 | TORTA NAO-ESPECIFICADA |
| 05901 | ROSCA NAO-ESPECIFICADA |
| 05902 | ROSQUINHA NAO-ESPECIFICADA |
| 05903 | ROSCA DE TRIGO NAO-ESPECIFICADA |
| 06001 | ROSCA DE POLVILHO NAO-ESPECIFICADA |
| 06002 | ROSQUINHA DE POLVILHO NAO-ESPECIFICADA |
| 06101 | CREPE |
| 06201 | CASCA PARA TORTA |
| 06301 | PAO BOLA |
| 06401 | PAO ARABE |
| 06402 | PAO SIRIO |
| 06501 | PAO DE FORMA INDUSTRIALIZADO DE QUALQUER MARCA DIET |
| 06502 | PAO SEVEN BOYS DIET |
| 06503 | PAO PULMEX DIET |
| 06504 | PAO DE FORMA PLUS VITA DIET |
| 06505 | PAO DE FORMA DE LEITE PULLMAN DIET |
| 06506 | PAO DE FORMA SEVEN BOYS DIET |
| 06507 | PAO DE FORMA PETROPOLIS DIET |
| 06508 | PAO PLUS VITA DIET |
| 06509 | PAO DE CAIXA DIET |
| 06510 | PAO DE FORMA INDUSTRIALIZADO DIET |
| 06511 | PAO DIET (DE FORMA INDUSTRIALIZADO) |
| 06512 | PAO DIET WICK BOLD |
| 06513 | PAO DE FORMA INDUSTRIALIZADO DE QUALQUER MARCA DIETETICO |
| 06514 | PAO SEVEN BOYS DIETETICO |
| 06515 | PAO PULMEX DIETETICO |
| 06516 | PAO DE FORMA PLUS VITA DIETETICO |
| 06517 | PAO DE FORMA DE LEITE PULLMAN DIETETICO |
| 06518 | PAO DE FORMA SEVEN BOYS DIETETICO |
| 06519 | PAO DE FORMA PETROPOLIS DIETETICO |
| 06520 | PAO PLUS VITA DIETETICO |
| 06521 | PAO DE CAIXA DIETETICO |
| 06522 | PAO DE FORMA INDUSTRIALIZADO DIETETICO |
| 06523 | PAO DIETETICO (DE FORMA INDUSTRIALIZADO) |
| 06524 | PAO DIETETICO WICK BOLD |
| 06601 | PAO DE FORMA INDUSTRIALIZADO DE QUALQUER MARCA LIGHT |
| 06602 | PAO SEVEN BOYS LIGHT |
| 06603 | PAO PULMEX LIGHT |
| 06604 | PAO DE FORMA PLUS VITA LIGHT |
| 06605 | PAO DE FORMA DE LEITE PULLMAN LIGHT |
| 06606 | PAO DE FORMA SEVEN BOYS LIGHT |
| 06607 | PAO DE FORMA PETROPOLIS LIGHT |
| 06608 | PAO PLUS VITA LIGHT |
| 06609 | PAO DE CAIXA LIGHT |
| 06610 | PAO DE FORMA INDUSTRIALIZADO LIGHT |
| 06701 | PAO DE QUEIJO LIGHT |
| 06801 | PAO INTEGRAL LIGHT |
| 06802 | PAO DE GRAHAM PLUS VITA LIGHT |
| 06803 | PAO DE GRAHAM LIGHT |
| 06804 | PAO INTEGRAL PLUS VITA LIGHT |
| 06901 | TORRADA DE QUALQUER PAO LIGHT |
| 06902 | PAO TORRADO (TORRADA) LIGHT |
| 06903 | TORRADA DE QUALQUER TIPO LIGHT |
| 07001 | TORTAS DOCES DE QUALQUER SABOR LIGHT |
| 07101 | TORTAS DOCES DE QUALQUER SABOR DIET |
| 07102 | TORTAS DOCES DE QUALQUER SABOR DIETETICAS |
| 07201 | TORTAS SALGADAS DE QUALQUER SABOR LIGHT |
| 07301 | SONHO LIGHT |
| 07302 | FILHOS (BOLINHO DE FARINHA DE TRIGO E OVOS) LIGHT |
| 07401 | SONHO DIET |
| 07402 | FILHOS (BOLINHO DE FARINHA DE TRIGO E OVOS) DIET |
| 07403 | SONHO DIETETICO |
| 07404 | FILHOS (BOLINHO DE FARINHA DE TRIGO E OVOS) DIETETICO |
| 07501 | BOLO DE CHOCOLATE LIGHT |
| 07601 | BOLO DE CHOCOLATE DIET |
| 07602 | BOLO DE CHOCOLATE DIETETICO |
| 07701 | BOLO DE LARANJA LIGHT |
| 07801 | BOLO DE LARANJA DIET |
| 07802 | BOLO DE LARANJA DIETETICO |
| 07901 | BOLO DE COCO LIGHT |
| 08001 | BOLO DE COCO DIET |
| 08002 | BOLO DE COCO DIETETICO |
| 08101 | BOLO DE CENOURA LIGHT |
| 08201 | BOLO DE CENOURA DIET |
| 08202 | BOLO DE CENOURA DIETETICO |
| 08301 | BISCOITO RECHEADO LIGHT |
| 08302 | MIRABEL LIGHT |
| 08303 | BISCOITO RECHEADO TOSTINE LIGHT |
| 08304 | BISCOITO LANCHE MIRABEL LIGHT |
| 08305 | BISCOITO WAFFER LIGHT |
| 08306 | LANCHE MIRABEL LIGHT |
| 08307 | WAFFER (BISCOITO) LIGHT |
| 08308 | ROSQUINHA RECHEADA DE QUALQUER SABOR LIGHT |
| 08309 | ALFAJORES (BISCOITO) LIGHT |
| 08310 | BOLACHA RECHEADA LIGHT |
| 08311 | BISCOITO CROCANTE LIGHT |
| 08312 | BISCOITO MIRABEL LIGHT |
| 08313 | ROSCA RECHEADA LIGHT |
| 08401 | BISCOITO RECHEADO DIET |
| 08402 | MIRABEL DIET |
| 08403 | BISCOITO RECHEADO TOSTINE DIET |
| 08404 | BISCOITO LANCHE MIRABEL DIET |
| 08405 | BISCOITO WAFFER DIET |
| 08406 | LANCHE MIRABEL DIET |
| 08407 | WAFFER (BISCOITO) DIET |
| 08408 | ROSQUINHA RECHEADA DE QUALQUER SABOR DIET |
| 08409 | ALFAJORES (BISCOITO) DIET |
| 08410 | BOLACHA RECHEADA DIET |
| 08411 | BISCOITO CROCANTE DIET |
| 08412 | BISCOITO MIRABEL DIET |
| 08413 | ROSCA RECHEADA DIET |
| 08414 | BISCOITO RECHEADO DIETETICO |
| 08415 | MIRABEL DIETETICO |
| 08416 | BISCOITO RECHEADO TOSTINE DIETETICO |
| 08417 | BISCOITO LANCHE MIRABEL DIETETICO |
| 08418 | BISCOITO WAFFER DIETETICO |
| 08419 | LANCHE MIRABEL DIETETICO |
| 08420 | WAFFER (BISCOITO) DIETETICO |
| 08421 | ROSQUINHA RECHEADA DE QUALQUER SABOR DIETETICA |
| 08422 | ALFAJORES (BISCOITO) DIETETICO |
| 08423 | BOLACHA RECHEADA DIETETICA |
| 08424 | BISCOITO CROCANTE DIETETICO |
| 08425 | BISCOITO MIRABEL DIETETICO |
| 08426 | ROSCA RECHEADA DIETETICA |
| 08501 | BISCOITO NAO-ESPECIFICADO LIGHT |
| 08601 | BISCOITO NAO-ESPECIFICADO DIET |
| 08602 | BISCOITO NAO-ESPECIFICADO DIETETICO |
| 08701 | BOLACHA NAO-ESPECIFICADA LIGHT |
| 08801 | BOLACHA NAO-ESPECIFICADA DIET |
| 08802 | BOLACHA NAO-ESPECIFICADA DIETETICA |
| 08901 | PAO CARTEIRA NAO-ESPECIFICADO LIGHT |
| 09001 | PAO CARTEIRA NAO-ESPECIFICADO DIET |
| 09002 | PAO CARTEIRA NAO-ESPECIFICADO DIETETICO |
| 09101 | PAO DE FORMA NAO-ESPECIFICADO LIGHT |
| 09201 | PAO DE FORMA NAO-ESPECIFICADO DIET |
| 09202 | PAO DE FORMA NAO-ESPECIFICADO DIETETICO |
| 09301 | PAO DE SANDUICHE NAO-ESPECIFICADO LIGHT |
| 09401 | PAO DE SANDUICHE NAO-ESPECIFICADO DIET |
| 09402 | PAO DE SANDUICHE NAO-ESPECIFICADO DIETETICO |
| 09501 | TORTA NAO-ESPECIFICADA LIGHT |
| 09601 | TORTA NAO-ESPECIFICADA DIET |
| 09602 | TORTA NAO-ESPECIFICADA DIETETICA |
| 09701 | ROSCA NAO-ESPECIFICADA LIGHT |
| 09702 | ROSQUINHA NAO-ESPECIFICADA LIGHT |
| 09703 | ROSCA DE TRIGO NAO-ESPECIFICADA LIGHT |
| 09801 | ROSCA NAO-ESPECIFICADA DIET |
| 09802 | ROSQUINHA NAO-ESPECIFICADA DIET |
| 09803 | ROSCA DE TRIGO NAO-ESPECIFICADA DIET |
| 09804 | ROSCA NAO-ESPECIFICADA DIETETICA |
| 09805 | ROSQUINHA NAO-ESPECIFICADA DIETETICA |
| 09806 | ROSCA DE TRIGO NAO-ESPECIFICADA DIETETICA |
| 09901 | PAO ARABE LIGHT |
| 09902 | PAO SIRIO LIGHT |
| 10001 | PAO DOCE DIET |
| 10002 | PAO ALMOFADINHA DIET |
| 10003 | CHINEQUE DIET |
| 10004 | PAO DOCE COM COCO DIET |
| 10005 | PAO DOCE COMUM DIET |
| 10006 | PAO BOLACHAO DIET |
| 10007 | PAO DOCE COM CREME DIET |
| 10008 | PAO DOCE COM RECHEIO DIET |
| 10009 | PAO DOCE SEM COCO DIET |
| 10010 | PAO DOCE ESPECIAL DIET |
| 10011 | PAO TRANCA COM FAROFA DIET |
| 10012 | PAO DOCE CARECA DIET |
| 10013 | PAO DE LEITE (PAO DOCE) DIET |
| 10014 | PAO CARECA DOCE DIET |
| 10015 | PAO DOCE DE TRIGO DIET |
| 10016 | MASSINHA DOCE (PAO DOCE) DIET |
| 10017 | PAO DOCE DE LEITE DIET |
| 10018 | CHINEQUE COM FAROFA DIET |
| 10019 | PAO LANCHE DIET |
| 10020 | PAO DE LANCHE DIET |
| 10021 | PAO DOCE COM MEL DIET |
| 10022 | PAO DOCE COM OVOS DIET |
| 10023 | PAO DE COCO DIET |
| 10024 | PAO AMANTEIGADO (DOCE) DIET |
| 10025 | XISBAG DIET |
| 10026 | CHISBAG DIET |
| 10027 | PAO SUPER FOFO DIET |
| 10028 | PAO VITA DOCE DIET |
| 10029 | PAO DE MEL DIET |
| 10030 | PAO CARTEIRA (DOCE) DIET |
| 10031 | PAO ALEMAO DIET |
| 10032 | PAO MANDI DIET |
| 10033 | PAO MANDIM DIET |
| 10034 | PANETONE DIET |
| 10035 | PAO MANTEIGA DIET |
| 10036 | PAO RECHEADO (DOCE) DIET |
| 10037 | BISNAGUINHA DIET |
| 10038 | FORROZINHO (PAO DOCE) DIET |
| 10039 | COLOMBA DE PASCOA DIET |
| 10040 | CROISSANT DIET |
| 10041 | PAO DOCE DIETETICO |
| 10042 | PAO ALMOFADINHA DIETETICO |
| 10043 | CHINEQUE DIETETICO |
| 10044 | PAO DOCE COM COCO DIETETICO |
| 10045 | PAO DOCE COMUM DIETETICO |
| 10046 | PAO BOLACHAO DIETETICO |
| 10047 | PAO DOCE COM CREME DIETETICO |
| 10048 | PAO DOCE COM RECHEIO DIETETICO |
| 10049 | PAO DOCE SEM COCO DIETETICO |
| 10050 | PAO DOCE ESPECIAL DIETETICO |
| 10051 | PAO TRANCA COM FAROFA DIETETICO |
| 10052 | PAO DOCE CARECA DIETETICO |
| 10053 | PAO DE LEITE (PAO DOCE) DIETETICO |
| 10054 | PAO CARECA DOCE DIETETICO |
| 10055 | PAO DOCE DE TRIGO DIETETICO |
| 10056 | MASSINHA DOCE (PAO DOCE) DIETETICO |
| 10057 | PAO DOCE DE LEITE DIETETICO |
| 10058 | CHINEQUE COM FAROFA DIETETICO |
| 10059 | PAO LANCHE DIETETICO |
| 10060 | PAO DE LANCHE DIETETICO |
| 10061 | PAO DOCE COM MEL DIETETICO |
| 10062 | PAO DOCE COM OVOS DIETETICO |
| 10063 | PAO DE COCO DIETETICO |
| 10064 | PAO AMANTEIGADO (DOCE) DIETETICO |
| 10065 | XISBAG DIETETICO |
| 10066 | CHISBAG DIETETICO |
| 10067 | PAO SUPER FOFO DIETETICO |
| 10068 | PAO VITA DOCE DIETETICO |
| 10069 | PAO DE MEL DIETETICO |
| 10070 | PAO CARTEIRA (DOCE) DIETETICO |
| 10071 | PAO ALEMAO DIETETICO |
| 10072 | PAO MANDI DIETETICO |
| 10073 | PAO MANDIM DIETETICO |
| 10074 | PANETONE DIETETICO |
| 10075 | PAO MANTEIGA DIETETICO |
| 10076 | PAO RECHEADO (DOCE) DIETETICO |
| 10077 | BISNAGUINHA DIETETICO |
| 10078 | FORROZINHO (PAO DOCE) DIETETICO |
| 10079 | COLOMBA DE PASCOA DIETETICA |
| 10080 | CROISSANT DIETETICO |
| 10101 | BISCOITO SALGADO LIGHT |
| 10102 | BISCOITO DO REINO LIGHT |
| 10103 | BISCOITO GRISSINI LIGHT |
| 10104 | BISCOITO CREAM CRACKER LIGHT |
| 10105 | PRESUNTINHO BISCOITO LIGHT |
| 10106 | BISCOITO TIPO ITALIANO LIGHT |
| 10107 | BISCOITO DE AGUA LIGHT |
| 10108 | BISCOITO QUEIJINHO LIGHT |
| 10109 | BISCOITO DE COCO SALGADO LIGHT |
| 10110 | BISCOITO DE CAMARAO LIGHT |
| 10111 | JOBS DE CAMARAO LIGHT |
| 10112 | BISCOITO DE POLVILHO SALGADO LIGHT |
| 10113 | BISCOITO PRESUNTINHO LIGHT |
| 10114 | BISCOITO INTEGRAL DE AGUA LIGHT |
| 10115 | BISCOITO SALGADO DE COCO LIGHT |
| 10116 | BISCOITO BIT LIGHT |
| 10117 | BISKUIT GRISSINI LIGHT |
| 10118 | BISCOITO SALGADINHO LIGHT |
| 10119 | BISCOITO SALGADO DE POLVILHO LIGHT |
| 10120 | BISCOITO CLUB CRACKER LIGHT |
| 10121 | SALGADINHO (BISCOITO) LIGHT |
| 10122 | BISCOITO DE AGUA INTEGRAL LIGHT |
| 10123 | QUEIJINHO BISCOITO LIGHT |
| 10124 | BISCOITO ITALIANO LIGHT |
| 10125 | BOLACHA SALGADA LIGHT |
| 10126 | BOLACHA DE AGUA E SAL LIGHT |
| 10127 | CHIPS (SALGADINHOS) LIGHT |
| 10128 | CROCK (SALGADINHOS) LIGHT |
| 10129 | OKAKI (BISCOITO SALGADO) LIGHT |
| 10130 | BACONZITOS LIGHT |
| 10201 | BOLO DE QUALQUER MARCA E SABOR LIGHT |
| 10202 | ROCAMBOLE LIGHT |
| 10203 | CUCA DE QUALQUER TIPO LIGHT |
| 10204 | FATIA DE BOLO DE QUALQUER SABOR LIGHT |
| 10301 | BOLO DE QUALQUER MARCA E SABOR DIET |
| 10302 | ROCAMBOLE DIET |
| 10303 | CUCA DE QUALQUER TIPO DIET |
| 10304 | FATIA DE BOLO DE QUALQUER SABOR DIET |
| 10305 | BOLO DE QUALQUER MARCA E SABOR DIETETICO |
| 10306 | ROCAMBOLE DIETETICO |
| 10307 | CUCA DE QUALQUER TIPO DIETETICO |
| 10308 | FATIA DE BOLO DE QUALQUER SABOR DIETETICO |
| 10401 | BISCOITO DOCE LIGHT |
| 10402 | CAVACO CHINES LIGHT |
| 10403 | BISCOITO DE MANTEIGA LIGHT |
| 10404 | BOLACHA AMANTEIGADA LIGHT |
| 10405 | SEQUILHO LIGHT |
| 10406 | TARECO LIGHT |
| 10407 | BISCOITO DE GERGELIM LIGHT |
| 10408 | BISCOITO AMANTEIGADO LIGHT |
| 10409 | SOLDA LIGHT |
| 10410 | BISCOITO TOSTINE LIGHT |
| 10411 | TOSTINE (BISCOITO) LIGHT |
| 10412 | BOLACHA DE FUBA LIGHT |
| 10413 | BOLACHA DE MANTEIGA LIGHT |
| 10414 | DEDITOS (BISCOITO) LIGHT |
| 10415 | BREVIDADE LIGHT |
| 10416 | BISCOITO DE MAIZENA LIGHT |
| 10417 | BOLACHA DE MILHO LIGHT |
| 10418 | SEQUILHO DE MILHO LIGHT |
| 10419 | BISCOITO DOCE DE QUEIJO LIGHT |
| 10420 | SEQUILHO DE MAIZENA LIGHT |
| 10421 | BISCOITO DOCE SORTIDO LIGHT |
| 10422 | BISCOITO DE FUBA LIGHT |
| 10423 | BISCOITO DEDITOS LIGHT |
| 10424 | BISCOITO SORTIDO LIGHT |
| 10425 | BISCOITO DE COCO DOCE LIGHT |
| 10426 | BISCOITO CREME LIGHT |
| 10427 | BISCOITO DE MILHO LIGHT |
| 10428 | BISCOITO CHAMPANHE LIGHT |
| 10429 | BISCOITO ROSQUINHA DE COCO LIGHT |
| 10430 | BISCOITO DE QUEIJO DOCE LIGHT |
| 10431 | BISCOITO BROA DE COCO LIGHT |
| 10432 | BISCOITO ROSCA DE COCO LIGHT |
| 10433 | BOLACHA COM LEITE LIGHT |
| 10434 | BOLACHA DOCE LIGHT |
| 10435 | MARIA MALUCA (BOLACHA DOCE) LIGHT |
| 10436 | BISCOITO CHAMPAGNE LIGHT |
| 10437 | BISCOITO MARIA LIGHT |
| 10438 | BISCOITO DE LEITE LIGHT |
| 10439 | BISCOITO DE COCO LIGHT |
| 10440 | BOLACHA DE COCO LIGHT |
| 10501 | BISCOITO DOCE DIET |
| 10502 | CAVACO CHINES DIET |
| 10503 | BISCOITO DE MANTEIGA DIET |
| 10504 | BOLACHA AMANTEIGADA DIET |
| 10505 | SEQUILHO DIET |
| 10506 | TARECO DIET |
| 10507 | BISCOITO DE GERGELIM DIET |
| 10508 | BISCOITO AMANTEIGADO DIET |
| 10509 | SOLDA DIET |
| 10510 | BISCOITO TOSTINE DIET |
| 10511 | TOSTINE (BISCOITO) DIET |
| 10512 | BOLACHA DE FUBA DIET |
| 10513 | BOLACHA DE MANTEIGA DIET |
| 10514 | DEDITOS (BISCOITO) DIET |
| 10515 | BREVIDADE DIET |
| 10516 | BISCOITO DE MAIZENA DIET |
| 10517 | BOLACHA DE MILHO DIET |
| 10518 | SEQUILHO DE MILHO DIET |
| 10519 | BISCOITO DOCE DE QUEIJO DIET |
| 10520 | SEQUILHO DE MAIZENA DIET |
| 10521 | BISCOITO DOCE SORTIDO DIET |
| 10522 | BISCOITO DE FUBA DIET |
| 10523 | BISCOITO DEDITOS DIET |
| 10524 | BISCOITO SORTIDO DIET |
| 10525 | BISCOITO DE COCO DOCE DIET |
| 10526 | BISCOITO CREME DIET |
| 10527 | BISCOITO DE MILHO DIET |
| 10528 | BISCOITO CHAMPANHE DIET |
| 10529 | BISCOITO ROSQUINHA DE COCO DIET |
| 10530 | BISCOITO DE QUEIJO DOCE DIET |
| 10531 | BISCOITO BROA DE COCO DIET |
| 10532 | BISCOITO ROSCA DE COCO DIET |
| 10533 | BOLACHA COM LEITE DIET |
| 10534 | BOLACHA DOCE DIET |
| 10535 | MARIA MALUCA (BOLACHA DOCE) DIET |
| 10536 | BISCOITO CHAMPAGNE DIET |
| 10537 | BISCOITO MARIA DIET |
| 10538 | BISCOITO DE LEITE DIET |
| 10539 | BISCOITO DE COCO DIET |
| 10540 | BOLACHA DE COCO DIET |
| 10541 | BISCOITO DOCE DIETETICO |
| 10542 | CAVACO CHINES DIETETICO |
| 10543 | BISCOITO DE MANTEIGA DIETETICO |
| 10544 | BOLACHA AMANTEIGADA DIETETICA |
| 10545 | SEQUILHO DIETETICO |
| 10546 | TARECO DIETETICO |
| 10547 | BISCOITO DE GERGELIM DIETETICO |
| 10548 | BISCOITO AMANTEIGADO DIETETICO |
| 10549 | SOLDA DIETETICA |
| 10550 | BISCOITO TOSTINE DIETETICO |
| 10551 | TOSTINE (BISCOITO) DIETETICO |
| 10552 | BOLACHA DE FUBA DIETETICA |
| 10553 | BOLACHA DE MANTEIGA DIETETICA |
| 10554 | DEDITOS (BISCOITO) DIETETICO |
| 10555 | BREVIDADE DIETETICA |
| 10556 | BISCOITO DE MAIZENA DIETETICO |
| 10557 | BOLACHA DE MILHO DIETETICA |
| 10558 | SEQUILHO DE MILHO DIETETICO |
| 10559 | BISCOITO DOCE DE QUEIJO DIETETICO |
| 10560 | SEQUILHO DE MAIZENA DIETETICO |
| 10561 | BISCOITO DOCE SORTIDO DIETETICO |
| 10562 | BISCOITO DE FUBA DIETETICO |
| 10563 | BISCOITO DEDITOS DIETETICO |
| 10564 | BISCOITO SORTIDO DIETETICO |
| 10565 | BISCOITO DE COCO DOCE DIETETICO |
| 10566 | BISCOITO CREME DIETETICO |
| 10567 | BISCOITO DE MILHO DIETETICO |
| 10568 | BISCOITO CHAMPANHE DIETETICO |
| 10569 | BISCOITO ROSQUINHA DE COCO DIETETICO |
| 10570 | BISCOITO DE QUEIJO DOCE DIETETICO |
| 10571 | BISCOITO BROA DE COCO DIETETICO |
| 10572 | BISCOITO ROSCA DE COCO DIETETICO |
| 10573 | BOLACHA COM LEITE DIETETICA |
| 10574 | BOLACHA DOCE DIETETICA |
| 10575 | MARIA MALUCA (BOLACHA DOCE) DIETETICA |
| 10576 | BISCOITO DE CHAMPAGNE DIETETICO |
| 10577 | BISCOITO MARIA DIETETICO |
| 10578 | BISCOITO DE LEITE DIETETICO |
| 10579 | BISCOITO DE COCO DIETETICO |
| 10580 | BOLACHA DE COCO DIETETICA |
| 10601 | BOLO DE BANANA |
| 10701 | BROA DE QUEIJO |
| 10801 | PAO DE FORMA NAO ESPECIFICADO LIGHT |
| 10901 | PAO PRETO LIGHT |
| 10902 | PAO DE CENTEIO LIGHT |
| 11001 | AVOADOR |
| 11101 | PAO DE LINHO OU LINHACA |
| 11201 | PAO FRANCES DIET |
| 11301 | PAO DOCE LIGHT |
| 11401 | ROSCA DOCE LIGHT |
| 11501 | PAO DE SOJA LIGHT |
| 00101 | CARNE SECA |
| 00102 | CARNE DE CHARQUE |
| 00103 | JABA |
| 00104 | CHARQUE |
| 00105 | CARNE DE SAL PRESA |
| 00106 | CARNE DO SUL |
| 00201 | CARNE DE SOL |
| 00202 | CARNE DO SERTAO |
| 00203 | CARNE DE VENTO |
| 00204 | CARNE DO SOL |
| 00301 | CARNE BOVINA DEFUMADA |
| 00401 | CARNE BOVINA SALGADA |
| 00402 | FIGADO ALEMAO |
| 00403 | COSTELA BOVINA SALGADA |
| 00501 | CARNE BOVINA DE HAMBURGUER |
| 00502 | HAMBURGUER DE CARNE BOVINA |
| 00503 | CARNE DE HAMBURGUER BOVINA |
| 00601 | CARNE SUINA DEFUMADA |
| 00701 | ORELHA DE PORCO DEFUMADA |
| 00801 | LOMBO DE PORCO DEFUMADO |
| 00802 | COPA DE PORCO DEFUMADA |
| 00901 | COSTELA DE PORCO DEFUMADA |
| 01001 | TOUCINHO DE PORCO DEFUMADO |
| 01002 | BACON NO VAREJO |
| 01003 | TOUCINHO DEFUMADO |
| 01004 | BACON EMBALADO |
| 01005 | BACON |
| 01006 | BACON DEFUMADO |
| 01007 | RETALHO DE BACON |
| 01008 | BACON EM RETALHO |
| 01101 | HAMBURGUER DE FRANGO |
| 01102 | CARNE DE FRANGO DE HAMBURGUER |
| 01201 | CHISPE DEFUMADO |
| 01202 | PE DE PORCO DEFUMADO |
| 01301 | CARNE SUINA SALGADA |
| 01401 | ORELHA DE PORCO SALGADA |
| 01501 | COSTELA DE PORCO SALGADA |
| 01601 | LOMBO DE PORCO SALGADO |
| 01602 | COPA DE PORCO SALGADA |
| 01701 | PE DE PORCO SALGADO |
| 01702 | CHISPE SALGADO |
| 01703 | CHISPE |
| 01801 | RABO DE PORCO SALGADO |
| 01901 | TOUCINHO DE PORCO SALGADO |
| 01902 | TOUCINHO SALGADO |
| 02001 | VISCERA DE PORCO SALGADA |
| 02002 | MIUDO DE PORCO SALGADO |
| 02003 | FRESSURA DE PORCO SALGADA |
| 02004 | FRISSURA DE PORCO SALGADA |
| 02101 | SALSICHA NO VAREJO |
| 02102 | SALSICHA EM PACOTE |
| 02103 | SALSICHAO NO VAREJO |
| 02104 | SALSICHAO EM PACOTE |
| 02201 | LINGUICA NO VAREJO |
| 02202 | LINGUICA EM PACOTE |
| 02203 | LINGUICA PARA CHURRASCO |
| 02204 | LINGUICA NAO-ESPECIFICADA |
| 02205 | LINGUICA CALABRESA |
| 02206 | LINGUICA BOVINA |
| 02207 | LINGUICA DE FRANGO |
| 02208 | LINGUICA SUINA |
| 02209 | LINGUICA DEFUMADA |
| 02210 | LINGUICA MISTA |
| 02211 | LINGUICA TOSCANA |
| 02212 | LINGUICA SUINA DEFUMADA |
| 02213 | CALABRESA (LINGUICA) |
| 02214 | LINGUICA DE PERNIL |
| 02301 | RETALHO PARA PIZZA |
| 02302 | QUEIJO E PRESUNTO RETALHADOS PARA PIZZA |
| 02303 | PRESUNTO E QUEIJO RETALHADOS PARA PIZZA |
| 02304 | QUEIJO E PRESUNTO PICADOS |
| 02401 | CHOURICO |
| 02402 | MORCELA |
| 02403 | MORCILHA |
| 02404 | QUEIJO DE PORCO |
| 02405 | MURCILHA |
| 02501 | PAIO |
| 02502 | CODEGUIM |
| 02601 | MORTADELA |
| 02602 | MORTADELA BOVINA |
| 02603 | MORTADELA DE FRANGO |
| 02604 | MORTADELA SUINA |
| 02605 | MORTADELA FATIADA NAO-ESPECIFICADA |
| 02606 | RETALHO DE MORTADELA |
| 02607 | MORTADELA BOLONHESA |
| 02608 | MORTADELA DE CHESTER |
| 02609 | MORTADELA DE GALINHA |
| 02610 | MORTADELA DE PERU |
| 02611 | MORTADELA DEFUMADA |
| 02612 | MORTADELA MISTA |
| 02701 | SALAME |
| 02702 | SALAMINHO |
| 02703 | SALAME DEFUMADO |
| 02801 | BLANQUET DE PERU |
| 02802 | BLANQUETE DE PERU |
| 02901 | PRESUNTO DE QUALQUER TIPO |
| 02902 | PRESUNTO DE PERU |
| 02903 | PRESUNTO DE FRANGO |
| 02904 | PRESUNTO BOVINO |
| 03001 | HAMBURGUER DE PERU |
| 03002 | CARNE DE PERU DE HAMBURGUER |
| 03101 | HAMBURGUER NAO-ESPECIFICADO |
| 03102 | CARNE NAO-ESPECIFICADA DE HAMBURGUER |
| 03201 | PATE DE PRESUNTO EMBUTIDO |
| 03202 | PASTA DE PRESUNTO EMBUTIDA |
| 03301 | PATE DE CARNE EMBUTIDO |
| 03302 | PASTA DE CARNE EMBUTIDA |
| 03401 | PATE DE FIGADO EMBUTIDO |
| 03402 | PASTA DE FIGADO EMBUTIDA |
| 03501 | PATE DE GALINHA EMBUTIDO |
| 03502 | PASTA DE GALINHA EMBUTIDA |
| 03601 | APRESUNTADO |
| 03602 | APRESUNTADO FATIADO |
| 03603 | RETALHO DE APRESUNTADO |
| 03701 | PATE DE CEBOLA EM CONSERVA |
| 03702 | PATE DE CEBOLA |
| 03801 | PATE DE BERINJELA EM CONSERVA |
| 03802 | PATE DE BERINJELA |
| 03901 | PURURUCA DE PORCO |
| 03902 | PELE DE PORCO PREPARADA |
| 04001 | PATE NAO-ESPECIFICADO |
| 04101 | CARNE DE AVES DEFUMADA |
| 04102 | PEITO DE GALINHA DEFUMADO |
| 04201 | MASSA DE QUIBE, CROQUETE, SALGADINHO DE PEIXE, ETC. |
| 04202 | MASSA DE QUIBE |
| 04203 | MASSA DE CROQUETE |
| 04204 | MASSA DE SALGADINHO DE PEIXE |
| 04205 | QUIBE SEMIPRONTO |
| 04301 | CARNE SALGADA DE FEIJOADA |
| 04302 | MISTURA DE CARNES SALGADAS DE FEIJOADA |
| 04303 | CARNE SALGADA NAO-ESPECIFICADA PARA FEIJOADA |
| 04401 | CARNE BOVINA EMPANADA |
| 04501 | ALMONDEGA CONGELADA DE QUALQUER ANIMAL (CRUA) |
| 04502 | ALMONDEGA DE QUALQUER ANIMAL CONGELADA |
| 04503 | ALMONDEGA DE FRANGO CONGELADA |
| 04601 | CARNE SALGADA NAO-ESPECIFICADA |
| 04701 | ROSBIFE E BIFE A MILANEZA INDUSTRIALIZADO SEMIPRONTO |
| 04702 | BIFE A MILANEZA INDUSTRIALIZADO SEMIPRONTO |
| 04801 | CARNE SALGADA DE OUTROS ANIMAIS |
| 04802 | CARNE DE CAPIVARA SALGADA |
| 04803 | CARNE DE CABRITO SALGADA |
| 04804 | CARNE DE BODE SALGADA |
| 04805 | CARNE DE CARNEIRO SALGADA |
| 04806 | CARNE DE OVELHA SALGADA |
| 04807 | CARNE DE PREA SALGADA |
| 04901 | FRANGO EMPANADO |
| 04902 | NUGGET DE FRANGO EMPANADO |
| 04903 | FILE DE FRANGO EMPANADO |
| 04904 | MINI CHICKEN EMPANADO |
| 04905 | EMPANADO DE FRANGO |
| 04906 | TEKITOS (CARNE DE FRANGO EMPANADA) |
| 05001 | MORTADELA LIGHT |
| 05002 | MORTADELA BOVINA LIGHT |
| 05003 | MORTADELA DE FRANGO LIGHT |
| 05004 | MORTADELA SUINA LIGHT |
| 05005 | MORTADELA FATIADA NAO-ESPECIFICADA LIGHT |
| 05006 | RETALHO DE MORTADELA LIGHT |
| 05007 | MORTADELA BOLONHESA LIGHT |
| 05008 | MORTADELA DE CHESTER LIGHT |
| 05009 | MORTADELA DE GALINHA LIGHT |
| 05010 | MORTADELA DE PERU LIGHT |
| 05011 | MORTADELA DEFUMADA LIGHT |
| 05012 | MORTADELA MISTA LIGHT |
| 05101 | HAMBURGUER DE FRANGO LIGHT |
| 05102 | CARNE DE FRANGO DE HAMBURGUER LIGHT |
| 05201 | SALSICHA NO VAREJO LIGHT |
| 05202 | SALSICHA EM PACOTE LIGHT |
| 05203 | SALSICHAO NO VAREJO LIGHT |
| 05204 | SALSICHAO EM PACOTE LIGHT |
| 05301 | LINGUICA NO VAREJO LIGHT |
| 05302 | LINGUICA EM PACOTE LIGHT |
| 05303 | LINGUICA PARA CHURRASCO LIGHT |
| 05304 | LINGUICA NAO-ESPECIFICADA LIGHT |
| 05305 | LINGUICA CALABRESA LIGHT |
| 05306 | LINGUICA BOVINA LIGHT |
| 05307 | LINGUICA DE FRANGO LIGHT |
| 05308 | LINGUICA SUINA LIGHT |
| 05309 | LINGUICA DEFUMADA LIGHT |
| 05310 | LINGUICA MISTA LIGHT |
| 05311 | LINGUICA TOSCANA LIGHT |
| 05401 | SALAME LIGHT |
| 05402 | SALAMINHO LIGHT |
| 05501 | BLANQUET DE PERU LIGHT |
| 05601 | HAMBURGUER DE PERU LIGHT |
| 05602 | CARNE DE PERU DE HAMBURGUER LIGHT |
| 05701 | HAMBURGUER NAO-ESPECIFICADO LIGHT |
| 05702 | CARNE NAO-ESPECIFICADA DE HAMBURGUER LIGHT |
| 05801 | PATE DE PRESUNTO EMBUTIDO LIGHT |
| 05802 | PASTA DE PRESUNTO EMBUTIDA LIGHT |
| 05901 | PATE DE GALINHA EMBUTIDO LIGHT |
| 05902 | PASTA DE GALINHA EMBUTIDA LIGHT |
| 06001 | PRESUNTO DE FRANGO LIGTH |
| 06101 | PRESUNTO DE PERU LIGHT |
| 06102 | PRESUNTO DE PERU DEFUMADO LIGHT |
| 06201 | PRESUNTO SUINO LIGHT |
| 06202 | PRESUNTO SUINO SEM GORDURA |
| 06301 | ESPINHACO DE PORCO SALGADO |
| 06302 | ESPINHACO SUINO SALGADO |
| 06401 | DOBRADINHA SALGADA |
| 06501 | RABO SUINO DEFUMADO |
| 06601 | LINGUICA DE SOJA NO VAREJO |
| 06701 | GARGANTA SUINA SALGADA |
| 00101 | REFRIGERANTE DE COLA |
| 00102 | COCA COLA |
| 00103 | REFRIGERANTE COCA COLA |
| 00104 | REFRIGERANTE PEPSI |
| 00105 | PEPSI |
| 00106 | PEPSI COLA |
| 00107 | REFRIGERANTE PEPSI COLA |
| 00201 | REFRIGERANTE DE LARANJA |
| 00202 | FANTA LARANJA |
| 00203 | REFRIGERANTE CRUSH |
| 00204 | CRUSH |
| 00205 | REFRIGERANTE MIRINDA |
| 00206 | MIRINDA |
| 00207 | REFRIGERANTE SUKITA |
| 00208 | SUKITA |
| 00209 | REFRIGERANTE POP LARANJA |
| 00210 | POP LARANJA |
| 00301 | REFRIGERANTE DE GUARANA |
| 00302 | GUARANA |
| 00303 | REFRIGERANTE FRATELLY VITA |
| 00304 | FRATELLY VITA |
| 00305 | GUARAVITA |
| 00306 | GUARAVITON |
| 00307 | REFRIGERANTE GUARANA |
| 00401 | REFRIGERANTE DE LIMAO |
| 00402 | REFRIGERANTE SODA LIMONADA |
| 00403 | SODA LIMONADA |
| 00404 | LIMAO REFRIGERANTE |
| 00405 | REFRIGERANTE LIMAO |
| 00406 | REFRIGERANTE SPRIT |
| 00407 | SPRIT REFRIGERANTE |
| 00408 | REFRIGERANTE FANTA LIMAO |
| 00409 | REFRIGERANTE GASOSA |
| 00410 | GASOSA |
| 00501 | REFRIGERANTE DE UVA |
| 00502 | REFRIGERANTE FANTA UVA |
| 00503 | REFRIGERANTE GRAPETE |
| 00504 | GRAPETE |
| 00505 | FANTA UVA |
| 00506 | BEBIDA MISTA DE UVA |
| 00601 | REFRIGERANTE MINEIRINHO |
| 00602 | MINEIRINHO |
| 00701 | REFRIGERANTE DE COLA LIGHT |
| 00702 | COCA COLA LIGHT |
| 00703 | REFRIGERANTE COCA COLA LIGHT |
| 00801 | REFRIGERANTE DE COLA DIET |
| 00802 | REFRIGERANTE PEPSI DIET |
| 00803 | PEPSI DIET |
| 00804 | PEPSI COLA DIET |
| 00805 | REFRIGERANTE PEPSI COLA DIET |
| 00806 | REFRIGERANTE DE COLA DIETETICO |
| 00807 | REFRIGERANTE PEPSI DIETETICO |
| 00808 | PEPSI DIETETICA |
| 00809 | PEPSI COLA DIETETICA |
| 00810 | REFRIGERANTE PEPSI COLA DIETETICO |
| 00901 | REFRIGERANTE MINUANO |
| 00902 | MINUANO |
| 01001 | REFRIGERANTE AGUA MINERAL |
| 01002 | REFRIGERANTE MINALBA |
| 01003 | MINALBA |
| 01004 | AGUA MINERAL |
| 01005 | AGUA MINERAL SEM GAS |
| 01006 | AGUA COM GAS |
| 01007 | AGUA MINERAL COM GAS |
| 01008 | AGUA FLUORADA |
| 01009 | AGUA PURIFICADA |
| 01010 | AGUA MINERAL SABOR LIMAO |
| 01011 | H2O SABOR LIMAO |
| 01012 | AGUA COM GAS E LIMAO |
| 01013 | AGUA COM GAS SABOR LIMAO |
| 01014 | AGUA MINERAL SABOR TANGERINA |
| 01015 | AGUA COM SABOR GASEIFICADA |
| 01016 | AGUA MINERAL SABOR LARANJA |
| 01101 | REFRIGERANTE DE MATE |
| 01102 | REFRIGERANTE MATE COURO |
| 01103 | MATE COURO |
| 01104 | MATE |
| 01105 | MATE LIMAO |
| 01106 | MATE PESSEGO |
| 01107 | PESSEGO MATE |
| 01201 | REFRIGERANTE BIDU |
| 01202 | BIDU |
| 01301 | REFRIGERANTE JAO |
| 01302 | JAO |
| 01401 | REFRIGERANTE ALOA |
| 01402 | ALOA |
| 01501 | REFRIGERANTE DE LARANJA LIGHT |
| 01502 | FANTA LARANJA LIGHT |
| 01503 | REFRIGERANTE CRUSH LIGHT |
| 01504 | CRUSH LIGHT |
| 01505 | REFRIGERANTE MIRINDA LIGHT |
| 01506 | MIRINDA LIGHT |
| 01507 | REFRIGERANTE SUKITA LIGHT |
| 01508 | SUKITA LIGHT |
| 01509 | REFRIGERANTE POP LARANJA LIGHT |
| 01510 | POP LARANJA LIGHT |
| 01601 | REFRIGERANTE DE GUARANA LIGHT |
| 01602 | GUARANA LIGHT |
| 01603 | REFRIGERANTE FRATELLY VITA LIGHT |
| 01604 | FRATELLY VITA LIGHT |
| 01701 | REFRIGERANTE XODO DA BAHIA |
| 01702 | XODO DA BAHIA |
| 01801 | REFRIGERANTE TUBAINA |
| 01802 | TUBAINA |
| 01901 | REFRIGERANTE TUBAINA LIGHT |
| 01902 | TUBAINA LIGHT |
| 02001 | CALDO DE CANA |
| 02002 | GARAPA |
| 02101 | AGUA DE COCO |
| 02201 | SUCO DE FRUTA OU VEGETAL ENGARRAFADO |
| 02202 | XAROPE DE FRUTA OU VEGETAL ENGARRAFADO |
| 02203 | XAROPE DE GUARANA |
| 02204 | XAROPE DE GROSELHA |
| 02301 | REFRESCO DE FRUTA ENGARRAFADO |
| 02302 | REFRESCO DE FRUTA ENCARTONADO |
| 02401 | SUCO DE FRUTA OU VEGETAL ARTIFICIAL EM PO |
| 02402 | Q-SUCO |
| 02403 | Q-REFRESKO |
| 02404 | CONCENTRADO DE REFRESCO EM PO |
| 02405 | REFRESCO DE FRUTA ARTIFICIAL EM PO |
| 02406 | PO DE REFRESCO |
| 02407 | REFRESCO ADOCYL EM PO |
| 02408 | REFRESCO EM PO |
| 02409 | SUCO ARTIFICIAL EM PO |
| 02410 | REFRESCO ARTIFICIAL EM PO |
| 02411 | QSUCO |
| 02412 | SUCO EM PO |
| 02501 | CAFE MOIDO |
| 02502 | PO DE CAFE |
| 02503 | CAFE EM PO |
| 02601 | CAFE SOLUVEL |
| 02602 | NESCAFE |
| 02701 | CHA PRETO |
| 02702 | CHA DA INDIA |
| 02703 | CHA JAPONES |
| 02801 | CHA MATE |
| 02802 | MATE ERVA |
| 02803 | ERVA MATE |
| 02804 | CHIMARRAO |
| 02805 | ERVA DE TERERE |
| 02901 | ERVA DOCE CHA |
| 02902 | CHA DE ERVA DOCE |
| 02903 | ANIZ CHA |
| 02904 | CHA DE ANIZ |
| 03001 | HORTELA CHA |
| 03002 | CHA DE HORTELA |
| 03101 | CANELA CHA |
| 03102 | CHA DE CANELA |
| 03201 | CAMOMILA CHA |
| 03202 | CHA DE CAMOMILA |
| 03301 | ERVA CIDREIRA CHA |
| 03302 | CHA DE ERVA CIDREIRA |
| 03303 | CHA DE CIDREIRA |
| 03401 | LEVEDO DE CERVEJA |
| 03402 | CERVEJA EM PO |
| 03501 | REFRIGERANTE NAO-ESPECIFICADO |
| 03601 | CAFE DE CEVADA |
| 03602 | CEVADA CAFE |
| 03603 | CEVADA EM PO |
| 03604 | CEVADA MOIDA |
| 03701 | CAFE EM GRAO |
| 03801 | REFRESCO EM SACO PLASTICO |
| 03802 | REFRESCO DE FRUTA EM SACO PLASTICO |
| 03901 | CAFE COM LEITE EM PO |
| 04001 | REFRIGERANTE DE MACA LIGHT |
| 04002 | MACA REFRIGERANTE LIGHT |
| 04003 | FANTA MACA LIGHT |
| 04101 | REFRIGERANTE DE MACA |
| 04102 | MACA REFRIGERANTE |
| 04103 | FANTA MACA |
| 04104 | BEBIDA MISTA DE MACA |
| 04201 | REFRIGERANTE DE TUTI FRUTI |
| 04202 | TUTI FRUTI REFRIGERANTE |
| 04301 | REFRIGERANTE DE TANGERINA |
| 04302 | TANGERINA REFRIGERANTE |
| 04401 | MARCELA CHA |
| 04402 | CHA DE MARCELA |
| 04501 | CHOCOLATE ENGARRAFADO OU ENCARTONADO |
| 04502 | CHOCOMILK ENGARRAFADO |
| 04503 | CHOCOMILK ENCARTONADO |
| 04504 | CHOCOLATE LIQUIDO |
| 04601 | REFRIGERANTE DE CAJU |
| 04602 | CAJU REFRIGERANTE |
| 04603 | CAJUINA |
| 04701 | SUCO DE FRUTA OU VEGETAL ENCARTONADO |
| 04702 | XAROPE DE FRUTA OU VEGETAL ENCARTONADO |
| 04703 | SUCO DE FRUTA OU VEGETAL EM CAIXA |
| 04704 | XAROPE DE FRUTA OU VEGETAL EM CAIXA |
| 04801 | GELO EM CUBO, PEDRA, ETC. |
| 04802 | GELIKO EM CUBO |
| 04803 | GELITO |
| 04901 | REFRIGERANTE AGUA TONICA |
| 04902 | AGUA TONICA |
| 04903 | REFRIGERANTE DE QUININO |
| 05001 | CAFE DESCAFEINADO |
| 05101 | SUCO DE FRUTA OU VEGETAL EM LATA |
| 05102 | XAROPE DE FRUTA OU VEGETAL EM LATA |
| 05201 | CHA DIET |
| 05202 | CHA VITA SANFER |
| 05203 | CHA EMAGRECEDOR |
| 05204 | CHA DIETETICO |
| 05205 | CHA VITA SANFER DIET |
| 05206 | CHA EMAGRECEDOR DIET |
| 05207 | CHA VITA SANFER DIETETICO |
| 05208 | CHA EMAGRECEDOR DIETETICO |
| 05301 | SUCO DE FRUTA OU VEGETAL EM SACO PLASTICO |
| 05302 | XAROPE DE FRUTA OU VEGETAL EM SACO PLASTICO |
| 05401 | REFRIGERANTE PARAGUAI |
| 05402 | PARAGUAI REFRIGERANTE |
| 05501 | REFRIGERANTE GOIANINHA |
| 05502 | GOIANINHA REFRIGERANTE |
| 05601 | MULTIERVAS CHA |
| 05602 | CHA MULTIERVAS |
| 05701 | REFRIGERANTE REAL |
| 05702 | REAL REFRIGERANTE |
| 05801 | REFRIGERANTE ENERGETICO |
| 05802 | TAFFMAN E |
| 05803 | GATORADE |
| 05804 | SNAPPLE |
| 05805 | BEBIDA ISOTONICA |
| 05806 | ISOTONICO |
| 05901 | BEBIDA ENERGETICA |
| 06001 | BEBIDA NAO-ALCOOLICA NAO-ESPECIFICADA |
| 06101 | OUTRO CHA |
| 06102 | CHA OUTRO |
| 06201 | SUCO DE FRUTA OU VEGETAL NAO-ESPECIFICADO |
| 06301 | CHA NAO-ESPECIFICADO |
| 06401 | CAFE CAPUCCINO SOLUVEL |
| 06402 | CAFE SOLUVEL CAPUCCINO |
| 06501 | ARRUDA CHA |
| 06502 | ARRUDA EM FOLHA |
| 06503 | FOLHA DE ARRUDA |
| 06504 | CHA DE ARRUDA |
| 06601 | BOLDO CHA |
| 06602 | CHA DE BOLDO |
| 06701 | REFRIGERANTE CITRICO |
| 06702 | FANTA CITRUS |
| 06801 | REFRIGERANTE DE ABACAXI |
| 06901 | REFRIGERANTE DE ABACAXI COM GUARANA |
| 07001 | REFRIGERANTE DE FRAMBOESA |
| 07101 | REFRIGERANTE DE MORANGO |
| 07201 | REFRIGERANTE DE MARACUJA |
| 07301 | REFRIGERANTE DE GENGIBRE |
| 07401 | REFRIGERANTE DE GUARANA DIET |
| 07402 | GUARANA DIET |
| 07403 | REFRIGERANTE DE GUARANA DIETETICO |
| 07404 | GUARANA DIETETICO |
| 07405 | REFRIGERANTE DE GUARANA ZERO |
| 07406 | GUARANA ZERO |
| 07501 | REFRIGERANTE DE LIMAO LIGHT |
| 07502 | REFRIGERANTE SODA LIMONADA LIGHT |
| 07503 | SODA LIMONADA LIGHT |
| 07504 | LIMAO REFRIGERANTE LIGHT |
| 07505 | REFRIGERANTE LIMAO LIGHT |
| 07506 | REFRIGERANTE FANTA LIMAO LIGHT |
| 07507 | REFRIGERANTE GASOSA LIGHT |
| 07508 | GASOSA LIGHT |
| 07601 | REFRIGERANTE DE LIMAO DIET |
| 07602 | REFRIGERANTE SPRIT DIET |
| 07603 | SPRIT REFRIGERANTE DIET |
| 07604 | REFRIGERANTE DE LIMAO DIETETICO |
| 07605 | REFRIGERANTE SPRIT DIETETICO |
| 07606 | SPRIT REFRIGERANTE DIETETICO |
| 07701 | REFRIGERANTE DE UVA LIGHT |
| 07702 | REFRIGERANTE FANTA UVA LIGHT |
| 07703 | REFRIGERANTE GRAPETE LIGHT |
| 07704 | GRAPETE LIGHT |
| 07705 | FANTA UVA LIGHT |
| 07801 | REFRIGERANTE MINEIRINHO LIGHT |
| 07802 | MINEIRINHO LIGHT |
| 07901 | REFRIGERANTE MINUANO LIGHT |
| 07902 | MINUANO LIGHT |
| 08001 | AGUA POTAVEL |
| 08002 | AGUA DOCE |
| 08003 | AGUA DE POCO |
| 08101 | REFRIGERANTE DE MATE LIGHT |
| 08102 | REFRIGERANTE MATE COURO LIGHT |
| 08103 | MATE COURO LIGHT |
| 08104 | MATE LIGHT |
| 08105 | MATE LIMAO LIGHT |
| 08106 | MATE PESSEGO LIGHT |
| 08201 | REFRIGERANTE BIDU LIGHT |
| 08202 | BIDU LIGHT |
| 08301 | REFRIGERANTE JAO LIGHT |
| 08302 | JAO LIGHT |
| 08401 | REFRIGERANTE ALOA LIGHT |
| 08402 | ALOA LIGHT |
| 08501 | REFRIGERANTE XODO DA BAHIA LIGHT |
| 08502 | XODO DA BAHIA LIGHT |
| 08601 | CAFE COM LEITE SOLUVEL |
| 08701 | REFRIGERANTE DE TUTI FRUTI LIGHT |
| 08702 | TUTI FRUTI REFRIGERANTE LIGHT |
| 08801 | REFRIGERANTE DE TANGERINA LIGHT |
| 08802 | TANGERINA REFRIGERANTE LIGHT |
| 08901 | REFRIGERANTE DE CAJU LIGHT |
| 08902 | CAJU REFRIGERANTE LIGHT |
| 08903 | CAJUINA LIGHT |
| 09001 | REFRIGERANTE AGUA TONICA LIGHT |
| 09002 | AGUA TONICA LIGHT |
| 09003 | REFRIGERANTE DE QUININO LIGHT |
| 09101 | REFRIGERANTE PARAGUAI LIGHT |
| 09102 | PARAGUAI REFRIGERANTE LIGHT |
| 09201 | REFRIGERANTE GOIANINHA LIGHT |
| 09202 | GOIANINHA REFRIGERANTE LIGHT |
| 09301 | REFRIGERANTE REAL LIGHT |
| 09302 | REAL REFRIGERANTE LIGHT |
| 09401 | REFRIGERANTE CITRICO LIGHT |
| 09402 | FANTA CITRUS LIGHT |
| 09501 | REFRIGERANTE DE ABACAXI LIGHT |
| 09601 | REFRIGERANTE DE ABACAXI COM GUARANA LIGHT |
| 09701 | REFRIGERANTE DE FRAMBOESA LIGHT |
| 09801 | REFRIGERANTE DE MORANGO LIGHT |
| 09901 | REFRIGERANTE DE MARACUJA LIGHT |
| 10001 | REFRIGERANTE DE GENGIBRE LIGHT |
| 10101 | SUCO DE FRUTA OU VEGETAL ORGANICO ENGARRAFADO |
| 10102 | XAROPE DE FRUTA OU VEGETAL ORGANICO ENGARRAFADO |
| 10201 | SUCO DE FRUTA OU VEGETAL ORGANICO ENCARTONADO |
| 10202 | XAROPE DE FRUTA OU VEGETAL ORGANICO ENCARTONADO |
| 10203 | SUCO DE FRUTA OU VEGETAL ORGANICO EM CAIXA |
| 10204 | XAROPE DE FRUTA OU VEGETAL ORGANICO EM CAIXA |
| 10301 | SUCO DE FRUTA OU VEGETAL ORGANICO EM LATA |
| 10302 | XAROPE DE FRUTA OU VEGETAL ORGANICO EM LATA |
| 10401 | SUCO DE FRUTA OU VEGETAL ORGANICO EM SACO PLASTICO |
| 10402 | XAROPE DE FRUTA OU VEGETAL ORGANICO EM SACO PLASTICO |
| 10501 | SUCO DE FRUTA OU VEGETAL ORGANICO NAO-ESPECIFICADO |
| 10601 | CAFE MOIDO ORGANICO |
| 10602 | PO DE CAFE ORGANICO |
| 10701 | CAFE SOLUVEL ORGANICO |
| 10702 | NESCAFE ORGANICO |
| 10801 | CAFE EM GRAO ORGANICO |
| 10901 | CAFE DESCAFEINADO ORGANICO |
| 11001 | SUCO DE FRUTA OU VEGETAL ARTIFICIAL EM PO LIGHT |
| 11002 | Q-SUCO LIGHT |
| 11003 | Q-REFRESKO LIGHT |
| 11004 | CONCENTRADO DE REFRESCO EM PO LIGHT |
| 11005 | REFRESCO DE FRUTA ARTIFICIAL EM PO LIGHT |
| 11006 | PO DE REFRESCO LIGHT |
| 11007 | REFRESCO ADOCYL EM PO LIGHT |
| 11101 | SUCO DE FRUTA OU VEGETAL ARTIFICIAL EM PO DIET |
| 11102 | Q-SUCO DIET |
| 11103 | Q-REFRESKO DIET |
| 11104 | CONCENTRADO DE REFRESCO EM PO DIET |
| 11105 | REFRESCO DE FRUTA ARTIFICIAL EM PO DIET |
| 11106 | PO DE REFRESCO DIET |
| 11107 | REFRESCO ADOCYL EM PO DIET |
| 11108 | SUCO DE FRUTA OU VEGETAL ARTIFICIAL EM PO DIETETICO |
| 11109 | Q-SUCO DIETETICO |
| 11110 | Q-REFRESKO DIETETICO |
| 11111 | CONCENTRADO DE REFRESCO EM PO DIETETICO |
| 11112 | REFRESCO DE FRUTA ARTIFICIAL EM PO DIETETICO |
| 11113 | PO DE REFRESCO DIETETICO |
| 11114 | REFRESCO ADOCYL EM PO DIETETICO |
| 11201 | CHOCOLATE ENGARRAFADO OU ENCARTONADO LIGHT |
| 11202 | CHOCOMILK ENGARRAFADO LIGHT |
| 11203 | CHOCOMILK ENCARTONADO LIGHT |
| 11301 | CHOCOLATE ENGARRAFADO OU ENCARTONADO DIET |
| 11302 | CHOCOMILK ENGARRAFADO DIET |
| 11303 | CHOCOMILK ENCARTONADO DIET |
| 11304 | CHOCOLATE ENGARRAFADO OU ENCARTONADO DIETETICO |
| 11305 | CHOCOMILK ENGARRAFADO LIGHT DIETETICO |
| 11306 | CHOCOMILK ENCARTONADO LIGHT DIETETICO |
| 11401 | SUCO DE FRUTA OU VEGETAL ENGARRAFADO LIGHT |
| 11402 | XAROPE DE FRUTA OU VEGETAL ENGARRAFADO LIGHT |
| 11501 | SUCO DE FRUTA OU VEGETAL ENGARRAFADO DIET |
| 11502 | XAROPE DE FRUTA OU VEGETAL ENGARRAFADO DIET |
| 11503 | SUCO DE FRUTA OU VEGETAL ENGARRAFADO DIETETICO |
| 11504 | XAROPE DE FRUTA OU VEGETAL ENGARRAFADO DIETETICO |
| 11601 | SUCO DE FRUTA OU VEGETAL ENCARTONADO LIGHT |
| 11602 | XAROPE DE FRUTA OU VEGETAL ENCARTONADO LIGHT |
| 11603 | SUCO DE FRUTA OU VEGETAL EM CAIXA LIGHT |
| 11604 | XAROPE DE FRUTA OU VEGETAL EM CAIXA LIGHT |
| 11701 | SUCO DE FRUTA OU VEGETAL ENCARTONADO DIET |
| 11702 | XAROPE DE FRUTA OU VEGETAL ENCARTONADO DIET |
| 11703 | SUCO DE FRUTA OU VEGETAL EM CAIXA DIET |
| 11704 | XAROPE DE FRUTA OU VEGETAL EM CAIXA DIET |
| 11705 | SUCO DE FRUTA OU VEGETAL ENCARTONADO DIETETICO |
| 11706 | XAROPE DE FRUTA OU VEGETAL ENCARTONADO DIETETICO |
| 11707 | SUCO DE FRUTA OU VEGETAL EM CAIXA DIETETICO |
| 11708 | XAROPE DE FRUTA OU VEGETAL EM CAIXA DIETETICO |
| 11801 | SUCO DE FRUTA OU VEGETAL EM LATA LIGHT |
| 11802 | XAROPE DE FRUTA OU VEGETAL EM LATA LIGHT |
| 11901 | SUCO DE FRUTA OU VEGETAL EM LATA DIET |
| 11902 | XAROPE DE FRUTA OU VEGETAL EM LATA DIET |
| 11903 | SUCO DE FRUTA OU VEGETAL EM LATA DIETETICO |
| 11904 | XAROPE DE FRUTA OU VEGETAL EM LATA DIETETICO |
| 12001 | SUCO DE FRUTA OU VEGETAL EM SACO PLASTICO LIGHT |
| 12002 | XAROPE DE FRUTA OU VEGETAL EM SACO PLASTICO LIGHT |
| 12101 | SUCO DE FRUTA OU VEGETAL EM SACO PLASTICO DIET |
| 12102 | XAROPE DE FRUTA OU VEGETAL EM SACO PLASTICO DIET |
| 12103 | SUCO DE FRUTA OU VEGETAL EM SACO PLASTICO DIETETICO |
| 12104 | XAROPE DE FRUTA OU VEGETAL EM SACO PLASTICO DIETETICO |
| 12201 | SUCO DE FRUTA OU VEGETAL NAO-ESPECIFICADO LIGHT |
| 12301 | SUCO DE FRUTA OU VEGETAL NAO-ESPECIFICADO DIET |
| 12302 | SUCO DE FRUTA OU VEGETAL NAO-ESPECIFICADO DIETETICO |
| 12401 | CAFE CAPUCCINO SOLUVEL LIGHT |
| 12402 | CAFE SOLUVEL CAPUCCINO LIGHT |
| 12501 | CAFE CAPUCCINO SOLUVEL DIET |
| 12502 | CAFE SOLUVEL CAPUCCINO DIET |
| 12503 | CAFE CAPUCCINO SOLUVEL DIETETICO |
| 12504 | CAFE SOLUVEL CAPUCCINO DIETETICO |
| 12601 | CHA PRETO LIGHT |
| 12602 | CHA DA INDIA LIGHT |
| 12603 | CHA JAPONES LIGHT |
| 12701 | CHA MATE LIGHT |
| 12702 | MATE ERVA LIGHT |
| 12703 | ERVA MATE LIGHT |
| 12801 | CHA MATE DIET |
| 12802 | MATE ERVA DIET |
| 12803 | ERVA MATE DIET |
| 12804 | CHA MATE DIETETICO |
| 12805 | MATE ERVA DIETETICO |
| 12806 | ERVA MATE DIETETICA |
| 12901 | REFRIGERANTE AGUA TONICA DIET |
| 12902 | AGUA TONICA DIET |
| 12903 | REFRIGERANTE DE QUININO DIET |
| 12904 | REFRIGERANTE AGUA TONICA DIETETICA |
| 12905 | AGUA TONICA DIETETICA |
| 12906 | REFRIGERANTE DE QUININO DIETETICO |
| 13001 | ERVA MATE ORGANICA |
| 13002 | MATE ERVA ORGANICO |
| 13003 | CHA MATE ORGANICO |
| 13004 | CHIMARRAO ORGANICO |
| 13101 | CHA DE LIMAO |
| 13201 | CHA DE ALFAZEMA |
| 13301 | CHA VERDE |
| 13401 | COCA COLA ZERO |
| 13402 | COCA COLA DIET |
| 13403 | REFRIGERANTE COCA COLA ZERO |
| 13501 | CHA DE MACA |
| 13601 | CLOROFILA (SUCO) |
| 13701 | CHA DE PESSEGO |
| 13801 | CHA DE SENE |
| 13901 | MATE COURO DIET |
| 14001 | REFRIGERANTE NAO-ESPECIFICADO DIET |
| 14101 | REFRIGERANTE NAO-ESPECIFICADO LIGHT |
| 14201 | CHA DE ENDRO |
| 14301 | SUCO DE FRUTA OU VEGETAL EM COPO PLASTICO |
| 14401 | BABOSA EM FOLHA |
| 14501 | REFRIGERANTE DE GENGIBRE DIET |
| 14601 | AROMATIZANTE DE BEBIDAS |
| 14602 | SABORIZADOR DE LEITE |
| 14701 | REFRIGERANTE JESUS |
| 14801 | CHA DE ABACAXI |
| 14901 | CHA DE MORANGO |
| 15001 | REFRIGERANTE GOIANINHA DIET |
| 15101 | CHA DE MALVA |
| 15201 | GRAPETE DIET |
| 15301 | CHA DE CARQUEJA |
| 15401 | FOLHAS DE AMORA (CHA) |
| 15501 | REFRIGERANTE DE ABACATE |
| 15601 | REFRIGERANTE DE LARANJA DIET |
| 00101 | CERVEJA |
| 00102 | CERVEJA PRETA |
| 00103 | CERVEJA BRANCA |
| 00201 | CHOPP |
| 00202 | CHOPP ESCURO |
| 00203 | CHOPP CLARO |
| 00204 | CHOPE |
| 00301 | AGUARDENTE DE CANA |
| 00302 | CACHACA |
| 00303 | DUELO DE CANA COM SABOR DE FRUTA |
| 00304 | AGUARDENTE DE CANA DE ACUCAR |
| 00305 | PINGA |
| 00401 | AGUARDENTE DE UVA |
| 00402 | GRASPA |
| 00403 | BAGACEIRA |
| 00501 | BATIDA DE QUALQUER SABOR |
| 00502 | LEITE DE ONCA |
| 00503 | BATIDA DE COCO |
| 00601 | STEINHARGER |
| 00701 | RUM |
| 00801 | VODKA |
| 00901 | GIN |
| 00902 | GENEBRA |
| 01001 | UISQUE |
| 01002 | WHISKY |
| 01101 | CAMPARI |
| 01201 | CHAMPANHE |
| 01202 | SIDRA CHAMPANHE |
| 01301 | VERMUTE |
| 01401 | CINZANO |
| 01501 | MARTINI |
| 01601 | CONHAQUE |
| 01701 | DRINK DREHER |
| 01801 | SAN RAFAEL |
| 01901 | LICOR DE QUALQUER SABOR |
| 02001 | SHERRY |
| 02101 | FERNET |
| 02201 | BITTER |
| 02301 | AGUARDENTE DE ARROZ |
| 02302 | SAQUE |
| 02401 | VINHO DE UVA E OUTROS |
| 02402 | VINHO ROSE |
| 02403 | VINHO TINTO |
| 02404 | VINHO VERDE |
| 02405 | VINHO SECO |
| 02406 | VINHO MOSCATEL |
| 02407 | VINHO LICOROSO |
| 02408 | VINHO QUINADO |
| 02409 | CAJUINA (VINHO) |
| 02410 | VINHO BRANCO |
| 02411 | JURUBEBA VINHO |
| 02412 | VINHO DE CATUABA |
| 02413 | VINHO SUAVE |
| 02414 | VINHO DE UVA |
| 02415 | VINHO |
| 02416 | CATUABA |
| 02417 | VINHO DE JURUBEBA |
| 02501 | AGUARDENTE DE BANANA |
| 02601 | AGUARDENTE DE ALCACHOFRA |
| 02701 | ST. REMY |
| 02801 | KEEP COOLER |
| 02901 | COQUETEL DE FRUTAS |
| 03001 | UNDERBERG |
| 03101 | CAIPIRINHA |
| 03201 | AGUARDENTE DE CATUABA |
| 03301 | CERVEJA SEM ALCOOL |
| 03401 | TEQUILA |
| 03501 | BEBIDA ALCOOLICA NAO-ESPECIFICADA |
| 03601 | VINHO DE UVA E OUTROS ORGANICO |
| 03602 | VINHO ROSE ORGANICO |
| 03603 | VINHO TINTO ORGANICO |
| 03604 | VINHO VERDE ORGANICO |
| 03605 | VINHO SECO ORGANICO |
| 03606 | VINHO MOSCATEL ORGANICO |
| 03607 | VINHO LICOROSO ORGANICO |
| 03608 | VINHO QUINADO ORGANICO |
| 03609 | CAJUINA ORGANICA |
| 03610 | VINHO BRANCO ORGANICO |
| 03611 | JURUBEBA VINHO ORGANICA |
| 03612 | VINHO DE CATUABA ORGANICO |
| 03613 | VINHO SUAVE ORGANICO |
| 03614 | VINHO DE UVA ORGANICO |
| 03615 | VINHO ORGANICO |
| 03616 | CATUABA ORGANICA |
| 03617 | VINHO DE JURUBEBA ORGANICO |
| 03701 | AGUARDENTE DE CANA ORGANICA |
| 03702 | CACHACA ORGANICA |
| 03703 | DUELO DE CANA COM SABOR DE FRUTA ORGANICO |
| 03704 | AGUARDENTE DE CANA DE ACUCAR ORGANICA |
| 03801 | AGUARDENTE DE UVA ORGANICA |
| 03802 | GRASPA ORGANICA |
| 03803 | BAGACEIRA ORGANICA |
| 03901 | AGUARDENTE DE ARROZ ORGANICA |
| 03902 | SAQUE ORGANICO |
| 04001 | AGUARDENTE DE BANANA ORGANICA |
| 04101 | AGUARDENTE DE ALCACHOFRA ORGANICA |
| 04201 | AGUARDENTE DE CATUABA ORGANICA |
| 04301 | CERVEJA LIGHT |
| 04302 | CERVEJA PRETA LIGHT |
| 04303 | CERVEJA BRANCA LIGHT |
| 04401 | CERVEJA SEM ALCOOL LIGHT |
| 04501 | AGUARDENTE DE MANDIOCA |
| 04502 | CAXIRI (AGUARDENTE DE MANDIOCA) |
| 04601 | QUENTAO |
| 04701 | AGUARDENTE DE JALAPA |
| 00101 | AZEITE DE OLIVA |
| 00102 | AZEITE IMPORTADO DE OLIVA |
| 00103 | AZEITE DOCE |
| 00104 | AZEITE TEMPERADO |
| 00105 | AZEITE COMPOSTO DE OLIVA E OLEO DE SOJA |
| 00106 | AZEITE VERDE |
| 00107 | AZEITE EXTRA VIRGEM |
| 00201 | AZEITE DE DENDE |
| 00301 | OLEO DE SOJA |
| 00401 | OLEO DE MILHO |
| 00501 | OLEO DE AMENDOIM |
| 00601 | OLEO DE ALGODAO |
| 00701 | OLEO DE COCO BABACU |
| 00702 | OLEO DE BABACU |
| 00801 | OLEO DE GIRASSOL |
| 00901 | OLEO DE ARROZ |
| 01001 | OLEO DE PEIXE |
| 01101 | OLEO DE AMENDOA |
| 01201 | OLEO DE GENGIBRE |
| 01301 | OLEO DE UVA |
| 01302 | OLEO DE SEMENTE DE UVA |
| 01401 | OLEO DE COCO TUCUM |
| 01402 | OLEO DE COCO TICUM |
| 01403 | OLEO DE TICUM |
| 01404 | OLEO DE TUCUM |
| 01501 | OLEO DE COCO OURICURI |
| 01502 | OLEO DE COCO LICURI |
| 01503 | OLEO DE OURICURI |
| 01504 | OLEO DE LICURI |
| 01601 | OLEO DE PEQUI |
| 01602 | OLEO DE PIQUI |
| 01603 | OLEO DE PIQUIA |
| 01604 | OLEO DE PEQUIA |
| 01701 | OLEO DE COCO MACAUBA |
| 01702 | OLEO DE MACAIBA |
| 01703 | OLEO DE MACAUBA |
| 01704 | OLEO DE COCO MACAIBA |
| 01801 | BANHA SUINA |
| 01802 | BANHA DE PORCO |
| 01901 | BANHA DE GALINHA |
| 02001 | BANHA VEGETAL |
| 02101 | GORDURA SUINA |
| 02201 | GORDURA DE COCO |
| 02202 | GORDURA VEGETAL |
| 02301 | GORDURA DE PEIXE |
| 02401 | GORDURA DE OVELHA |
| 02501 | GORDURA DE RINS DE BODE |
| 02601 | OLEO DE GERGELIM |
| 02701 | OLEO DE COCO DA BAHIA |
| 02801 | GORDURA DE GALINHA |
| 02901 | OLEO DE CANOLA |
| 03001 | BANHA BOVINA |
| 03101 | BANHA NAO-ESPECIFICADA |
| 03201 | OLEO NAO-ESPECIFICADO |
| 03301 | AZEITE MISTO DE SOJA E OLIVA |
| 03302 | AZEITE MISTO DE OLIVA E SOJA |
| 03303 | OLEO COMPOSTO DE SOJA E OLIVA |
| 03401 | OLEO DE BANANA |
| 03501 | OLEO DE DENDE |
| 03601 | OLEO DE COCO NAO-ESPECIFICADO |
| 03701 | OLEO DE BURITI |
| 03801 | GORDURA HIDROGENADA |
| 03802 | GORDURA PARA SORVETE |
| 03901 | AZEITE DE COCO BABACU |
| 04001 | AZEITE DE SOJA |
| 04101 | AZEITE DE GIRASSOL |
| 04201 | AZEITE DE MACAUBA |
| 04301 | AZEITE DE MAMONA |
| 00101 | REFEICAO PRONTA PARA VIAGEM |
| 00102 | MARMITA PARA VIAGEM |
| 00103 | QUENTINHA PARA VIAGEM |
| 00104 | MARMITEX PARA VIAGEM |
| 00105 | ALMOCO PARA VIAGEM |
| 00106 | ALMOCO EM QUENTINHA PARA VIAGEM |
| 00107 | JANTAR PARA VIAGEM |
| 00108 | JANTAR EM QUENTINHA PARA VIAGEM |
| 00109 | VIANDA PARA VIAGEM |
| 00201 | SALGADINHO PARA VIAGEM |
| 00202 | PASTEL PARA VIAGEM |
| 00203 | CROQUETE PARA VIAGEM |
| 00204 | BOLINHO SALGADO DE CARNE, CAMARAO, ETC. PARA VIAGEM |
| 00205 | COXINHA PARA VIAGEM |
| 00206 | EMPADA PARA VIAGEM |
| 00207 | RIZOLE PARA VIAGEM |
| 00208 | PIZZA EM PEDACO PARA VIAGEM |
| 00209 | ACARAJE PARA VIAGEM |
| 00210 | TORRESMO PARA VIAGEM |
| 00211 | QUIBE PARA VIAGEM |
| 00212 | ABARA PARA VIAGEM |
| 00213 | PACOCA DE CARNE DE SOL PARA VIAGEM |
| 00214 | POLENTA FRITA PARA VIAGEM |
| 00215 | ANGU FRITO PARA VIAGEM |
| 00216 | MACAXEIRA FRITA PARA VIAGEM |
| 00217 | ANGU DE MILHO PARA VIAGEM |
| 00218 | POLENTA PARA VIAGEM |
| 00219 | BOLINHO DE CARNE, CAMARAO, ETC. PARA VIAGEM |
| 00220 | BOLINHO SALGADO DE CAMARAO PARA VIAGEM |
| 00221 | BOLINHO DE CAMARAO PARA VIAGEM |
| 00222 | ESFIRRA PARA VIAGEM |
| 00223 | ESFIRRA DE CARNE PARA VIAGEM |
| 00224 | ESFIRRA DE QUEIJO PARA VIAGEM |
| 00225 | ESFIRRA DE RICOTA PARA VIAGEM |
| 00226 | ESFIRRA DE FRANGO PARA VIAGEM |
| 00227 | ENROLADINHO PARA VIAGEM |
| 00228 | BOLINHO DE AIPIM PARA VIAGEM |
| 00229 | EMPADINHA PARA VIAGEM |
| 00230 | FOGACCIA DE PRESUNTO E QUEIJO PARA VIAGEM |
| 00231 | CASQUINHA DE SIRI |
| 00232 | BOLINHO DE BACALHAU PARA VIAGEM |
| 00301 | SANDUICHE PARA VIAGEM |
| 00302 | CACHORRO QUENTE PARA VIAGEM |
| 00303 | HAMBURGUER PARA VIAGEM |
| 00304 | CHEESBURGUER PARA VIAGEM |
| 00305 | EGGSBURGUER PARA VIAGEM |
| 00306 | MISTO-QUENTE OU FRIO PARA VIAGEM |
| 00307 | BAURU PARA VIAGEM |
| 00308 | AMERICANO PARA VIAGEM |
| 00309 | TORRADA COM QUEIJO, PRESUNTO, OVO, ETC. PARA VIAGEM |
| 00310 | TORRADA COM PRESUNTO PARA VIAGEM |
| 00311 | TORRADA COM OVO PARA VIAGEM |
| 00312 | MISTO-FRIO PARA VIAGEM |
| 00313 | MISTO QUENTE OU FRIO PARA VIAGEM |
| 00314 | MISTO FRIO OU FRIO PARA VIAGEM |
| 00315 | SANDUICHE DE QUEIJO PRATO PARA VIAGEM |
| 00316 | SANDUICHE DE SALAME PARA VIAGEM |
| 00317 | SANDUICHE DE PRESUNTO PARA VIAGEM |
| 00318 | SANDUICHE DE QUEIJO PRATO COM PRESUNTO PARA VIAGEM |
| 00319 | SANDUICHE DE MORTADELA PARA VIAGEM |
| 00320 | SANDUICHE DE QUEIJO MINAS PARA VIAGEM |
| 00321 | CHEESE EGG PARA VIAGEM |
| 00322 | CHEESE TUDO PARA VIAGEM |
| 00323 | SANDUICHE NATURAL |
| 00324 | CHEESE CREAM |
| 00325 | CHEESE DE FILE |
| 00326 | CHEESE SALADA |
| 00327 | X SALADA |
| 00401 | SUCO PARA VIAGEM |
| 00402 | SUCO DE ABACAXI PARA VIAGEM |
| 00403 | SUCO DE ACEROLA PARA VIAGEM |
| 00404 | SUCO DE BETERRABA PARA VIAGEM |
| 00405 | SUCO DE CUPUACU PARA VIAGEM |
| 00406 | SUCO DE GOIABA PARA VIAGEM |
| 00407 | SUCO DE LARANJA PARA VIAGEM |
| 00408 | SUCO DE LARANJA COM BANANA PARA VIAGEM |
| 00409 | SUCO DE LARANJA E BETERRABA PARA VIAGEM |
| 00410 | SUCO DE LARANJA E CENOURA PARA VIAGEM |
| 00411 | SUCO DE LARANJA CENOURA E BETERRABA PARA VIAGEM |
| 00412 | SUCO DE MAMAO PARA VIAGEM |
| 00413 | SUCO DE MANGA PARA VIAGEM |
| 00414 | SUCO DE MARACUJA PARA VIAGEM |
| 00415 | SUCO DE MELAO PARA VIAGEM |
| 00416 | SUCO DE MORANGO PARA VIAGEM |
| 00417 | SUCO DE PESSEGO PARA VIAGEM |
| 00418 | SUCO DE PESSEGO EM CALDA PARA VIAGEM |
| 00419 | SUCO DE MACA PARA VIAGEM |
| 00501 | VITAMINA PARA VIAGEM |
| 00502 | VITAMINA DE FRUTAS OU VEGETAIS PARA VIAGEM |
| 00503 | VITAMINA DE BANANA PARA VIAGEM |
| 00504 | VITAMINA DE BANANA COM AVEIA PARA VIAGEM |
| 00505 | VITAMINA DE MAMAO PARA VIAGEM |
| 00506 | VITAMINA DE ABACATE PARA VIAGEM |
| 00507 | VITAMINA DE MORANGO PARA VIAGEM |
| 00508 | VITAMINA MISTA PARA VIAGEM |
| 00509 | VITAMINA DE MACA PARA VIAGEM |
| 00601 | REFRESCO PARA VIAGEM |
| 00602 | REFRESCO DE CAJU PARA VIAGEM |
| 00603 | REFRESCO DE GROSELHA PARA VIAGEM |
| 00604 | REFRESCO DE LARANJA PARA VIAGEM |
| 00605 | REFRESCO DE MARACUJA PARA VIAGEM |
| 00606 | REFRESCO DE LIMAO PARA VIAGEM |
| 00701 | ALIMENTO PRONTO CONGELADO PARA VIAGEM |
| 00702 | CONGELADO PRONTO PARA VIAGEM |
| 00703 | MANDIOCA CONGELADA PARA VIAGEM |
| 00704 | AIPIM CONGELADO PARA VIAGEM |
| 00801 | SALADA OU VERDURA COZIDA, EXCETO DE FRUTA PARA VIAGEM |
| 00802 | SALADA DE MAIONESE PARA VIAGEM |
| 00803 | VERDURA COZIDA OU FRITA PARA VIAGEM |
| 00804 | BERINJELA A MILANESA PARA VIAGEM |
| 00805 | MANDIOCA COZIDA PARA VIAGEM |
| 00806 | VERDURA FRITA PARA VIAGEM |
| 00807 | CARA COZIDO PARA VIAGEM |
| 00901 | MASSA PRONTA PARA VIAGEM |
| 00902 | PIZZA, NHOQUE, CANELONI, CAPELETI, FETUTINE, RAVIOLE PARA VIAGEM |
| 00903 | PIZZA PRONTA PARA VIAGEM |
| 00904 | LAZANHA PRONTA PARA VIAGEM |
| 00905 | NHOQUE PRONTO PARA VIAGEM |
| 00906 | CANELONI PRONTO PARA VIAGEM |
| 00907 | CAPELETI PRONTO PARA VIAGEM |
| 00908 | FETUTINE PRONTO PARA VIAGEM |
| 00909 | RAVIOLI PRONTO PARA VIAGEM |
| 00910 | CALZONE PRONTO PARA VIAGEM |
| 00911 | MASSA CASEIRA PRONTA PARA VIAGEM |
| 00912 | MACARRAO PRONTO PARA VIAGEM |
| 00913 | PANQUECA PRONTA PARA VIAGEM |
| 00914 | PIZZA CALABREZA PARA VIAGEM |
| 00915 | PIZZA MUZZARELA PARA VIAGEM |
| 00916 | PIZZA PRESUNTO PARA VIAGEM |
| 00917 | PIZZA PORTUGUESA |
| 00918 | MACARRAO COM MOLHO PARA VIAGEM |
| 00919 | MACARRONADA COM QUEIJO |
| 00920 | PIZZA PARA VIAGEM |
| 01001 | FRANGO ASSADO OU DEFUMADO PARA VIAGEM |
| 01002 | GALINHA ASSADA OU DEFUMADA PARA VIAGEM |
| 01003 | GALETO ASSADO OU DEFUMADO PARA VIAGEM |
| 01004 | GALETO PARA VIAGEM |
| 01005 | FRANGO A PASSARINHO PARA VIAGEM |
| 01006 | FRANGO DEFUMADO PARA VIAGEM |
| 01007 | GALINHA DEFUMADA PARA VIAGEM |
| 01008 | GALETO DEFUMADO PARA VIAGEM |
| 01009 | ESPETINHO DE FRANGO PARA VIAGEM |
| 01010 | COXA E SOBRECOXA DE FRANGO ASSADO PARA VIAGEM |
| 01011 | FRANGO ASSADO PARA VIAGEM |
| 01012 | CHURRASQUINHO DE FRANGO PARA VIAGEM |
| 01013 | FRANGO FRITO PARA VIAGEM |
| 01014 | CHURRASCO DE FRANGO PARA VIAGEM |
| 01101 | CARNE ASSADA OU BIFE PREPARADO PARA VIAGEM |
| 01102 | ESPETINHO DE CARNE PARA VIAGEM |
| 01103 | CHURRASQUINHO PARA VIAGEM |
| 01104 | COSTELA ASSADA PARA VIAGEM |
| 01105 | BIFE PREPARADO PARA VIAGEM |
| 01106 | CARNE COZIDA PARA VIAGEM |
| 01107 | CARNE DE SOL PREPARADA PARA VIAGEM |
| 01108 | BIFE A MILANEZA PARA VIAGEM |
| 01109 | CARNE ASSADA PARA VIAGEM |
| 01201 | PIPOCA PARA VIAGEM |
| 01202 | PIPOCA DOCE OU SALGADA PARA VIAGEM |
| 01203 | PIPOCA SALGADA PARA VIAGEM |
| 01204 | PIPOCA DE MILHO INDUSTRIALIZADA PARA VIAGEM |
| 01301 | CAFE PREPARADO PURO OU COM LEITE PARA VIAGEM |
| 01302 | CAFE PREPARADO PURO PARA VIAGEM |
| 01303 | CAFE COM LEITE PREPARADO PARA VIAGEM |
| 01304 | CAFE TIPO EXPRESSO PARA VIAGEM |
| 01305 | CAFE TIPO CAPUCCINO PARA VIAGEM |
| 01401 | MILHO VERDE COZIDO PARA VIAGEM |
| 01402 | MILHO COZIDO OU ASSADO PARA VIAGEM |
| 01403 | MILHO ASSADO PARA VIAGEM |
| 01501 | BATATA FRITA PARA VIAGEM |
| 01502 | BATATA PALITO PARA VIAGEM |
| 01503 | BATATA PALHA PARA VIAGEM |
| 01601 | BOLINHO DOCE DE AMENDOIM PARA VIAGEM |
| 01701 | PERNIL SUINO ASSADO PARA VIAGEM |
| 01801 | PEIXE FRITO PARA VIAGEM |
| 01802 | PEIXE ASSADO PARA VIAGEM |
| 01803 | PEIXE COZIDO PARA VIAGEM |
| 01901 | MUNGUZA PARA VIAGEM |
| 01902 | MUNGUNZA PARA VIAGEM |
| 01903 | MUNGUNZA PRE-COZIDO PARA VIAGEM |
| 01904 | CURAU PARA VIAGEM |
| 01905 | MUNGUZA PRE-COZIDO PARA VIAGEM |
| 02001 | BOLINHO NAO-ESPECIFICADO PARA VIAGEM |
| 02101 | CAMARAO COZIDO PARA VIAGEM |
| 02201 | FAROFA PARA VIAGEM |
| 02301 | MINGAU PARA VIAGEM |
| 02401 | TACACA PARA VIAGEM |
| 02501 | BOLINHO DE COCO |
| 02601 | PERU ASSADO OU DEFUMADO PARA VIAGEM |
| 02602 | PERU COZIDO PARA VIAGEM |
| 02603 | PERU DEFUMADO PARA VIAGEM |
| 02701 | RISOTO PARA VIAGEM |
| 02801 | VATAPA PARA VIAGEM |
| 02901 | FAROFA PRONTA EM PACOTE PARA VIAGEM |
| 03001 | CARPACCIO PARA VIAGEM |
| 03002 | CARPACCIO CONGELADO PARA VIAGEM |
| 03101 | BATATA CONGELADA PARA VIAGEM |
| 03102 | BATATA COZIDA PARA VIAGEM |
| 03103 | BATATA A VACUO PARA VIAGEM |
| 03104 | BATATA CONGELADA PARA FRITAR |
| 03201 | CALDO PARA VIAGEM |
| 03202 | CALDO DE CARNE PARA VIAGEM |
| 03203 | CALDO DE FEIJAO PARA VIAGEM |
| 03204 | CALDO DE MOCOTO PARA VIAGEM |
| 03205 | CALDO DE SIRI PARA VIAGEM |
| 03206 | CALDO DE FEIJOADA PARA VIAGEM |
| 03207 | CALDO DE FRANGO PARA VIAGEM |
| 03301 | BOLINHO DE POLVILHO PARA VIAGEM |
| 03401 | BANANA PARA VIAGEM |
| 03501 | BAIAO DE DOIS PARA VIAGEM |
| 03601 | LANCHE PARA VIAGEM |
| 03701 | CHESTER ASSADO OU DEFUMADO PARA VIAGEM |
| 03702 | CHESTER DEFUMADO PARA VIAGEM |
| 03801 | CHURRASCO PARA VIAGEM |
| 03901 | BOLINHO DOCE PARA VIAGEM |
| 04001 | BOLINHO DE ARROZ PARA VIAGEM |
| 04101 | BOLINHO DE COCO PARA VIAGEM |
| 04201 | SUSHI PARA VIAGEM |
| 04202 | SUSHI PRONTO PARA VIAGEM |
| 04301 | BOLINHO DE TAPIOCA PARA VIAGEM |
| 04401 | SALADA DE FRUTAS PARA VIAGEM |
| 04501 | SOPA PARA VIAGEM |
| 04601 | SALGADINHO SEMIPRONTO PARA VIAGEM |
| 04701 | SALSICHAO PARA VIAGEM |
| 04702 | SALSICHAO ASSADO PARA VIAGEM |
| 04801 | SALPICAO PARA VIAGEM |
| 04901 | ROSCA NAO-ESPECIFICADA PARA VIAGEM |
| 05001 | ARROZ PRONTO PARA VIAGEM |
| 05002 | ARROZ COZIDO PARA VIAGEM |
| 05003 | ARROZ COLORIDO PARA VIAGEM |
| 05101 | CANJICA PARA VIAGEM |
| 05201 | CHARUTO DE REPOLHO PARA VIAGEM |
| 05301 | SALGADINHO DE PELE (PACOTE) PARA VIAGEM |
| 05401 | ARROZ A GREGA PARA VIAGEM |
| 05501 | ARRUMADINHO PARA VIAGEM |
| 05601 | BOBO DE CAMARAO PARA VIAGEM |
| 05701 | CARURU PARA VIAGEM (QUIABO, AMENDOIM, CASTANHA DE CAJU, CAMARAO SECO, ETC.) |
| 05801 | CUSCUZ PAULISTA PARA VIAGEM |
| 05901 | EMPADAO PARA VIAGEM |
| 06001 | FAROFA DE BANANA PARA VIAGEM |
| 06101 | FEIJAO TROPEIRO PARA VIAGEM |
| 06201 | GALINHA COM ARROZ PARA VIAGEM |
| 06301 | GALINHADA PARA VIAGEM |
| 06401 | MOQUECA BAIANA PARA VIAGEM |
| 06402 | MOQUECA PARA VIAGEM |
| 06501 | OMELETE PARA VIAGEM |
| 06601 | PIRAO PARA VIAGEM |
| 06701 | QUIBEBE PARA VIAGEM |
| 06801 | QUICHE PARA VIAGEM |
| 06901 | SUFLE PARA VIAGEM |
| 07001 | TUTU PARA VIAGEM |
| 07101 | MOQUECA CAPIXABA PARA VIAGEM |
| 07201 | MANICOBA PARA VIAGEM |
| 07301 | CALDO VERDE PARA VIAGEM |
| 07401 | ARROZ DE CUXA PARA VIAGEM |
| 07501 | ANGU A BAIANA PARA VIAGEM |
| 07601 | CHOCOLATE PARA VIAGEM |
| 07701 | GEMADA PARA VIAGEM |
| 07801 | ACAI COM GRANOLA VIAGEM |
| 07901 | SUCO ORGANICO PARA VIAGEM |
| 07902 | SUCO DE ABACAXI ORGANICO PARA VIAGEM |
| 07903 | SUCO DE ACEROLA ORGANICO PARA VIAGEM |
| 07904 | SUCO DE BETERRABA ORGANICO PARA VIAGEM |
| 07905 | SUCO DE CUPUACU ORGANICO PARA VIAGEM |
| 07906 | SUCO DE GOIABA ORGANICO PARA VIAGEM |
| 07907 | SUCO DE LARANJA ORGANICO PARA VIAGEM |
| 07908 | SUCO DE LARANJA COM BANANA ORGANICO PARA VIAGEM |
| 07909 | SUCO DE LARANJA E BETERRABA ORGANICO PARA VIAGEM |
| 07910 | SUCO DE LARANJA E CENOURA ORGANICO PARA VIAGEM |
| 07911 | SUCO DE LARANJA CENOURA E BETERRABA ORGANICO PARA VIAGEM |
| 07912 | SUCO DE MAMAO ORGANICO PARA VIAGEM |
| 07913 | SUCO DE MANGA ORGANICO PARA VIAGEM |
| 07914 | SUCO DE MARACUJA ORGANICO PARA VIAGEM |
| 07915 | SUCO DE MELAO ORGANICO PARA VIAGEM |
| 07916 | SUCO DE MORANGO ORGANICO PARA VIAGEM |
| 07917 | SUCO DE PESSEGO ORGANICO PARA VIAGEM |
| 07918 | SUCO DE PESSEGO EM CALDA ORGANICO PARA VIAGEM |
| 08001 | MASSA PRONTA LIGHT PARA VIAGEM |
| 08002 | PIZZA, NHOQUE, CANELONI, CAPELETI, FETUTINE, RAVIOLE LIGHT PARA VIAGEM |
| 08003 | PIZZA PRONTA LIGHT PARA VIAGEM |
| 08004 | LAZANHA PRONTA LIGHT PARA VIAGEM |
| 08005 | NHOQUE PRONTO LIGHT PARA VIAGEM |
| 08006 | CANELONI PRONTO LIGHT PARA VIAGEM |
| 08007 | CAPELETI PRONTO LIGHT PARA VIAGEM |
| 08008 | FETUTINE PRONTO LIGHT PARA VIAGEM |
| 08009 | RAVIOLI PRONTO LIGHT PARA VIAGEM |
| 08010 | CALZONE PRONTO LIGHT PARA VIAGEM |
| 08011 | MASSA CASEIRA PRONTA LIGHT PARA VIAGEM |
| 08012 | MACARRAO PRONTO LIGHT PARA VIAGEM |
| 08013 | PANQUECA PRONTA LIGHT PARA VIAGEM |
| 08014 | PIZZA CALABREZA LIGHT PARA VIAGEM |
| 08015 | PIZZA MUZZARELA LIGHT PARA VIAGEM |
| 08016 | PIZZA PRESUNTO LIGHT PARA VIAGEM |
| 08017 | PIZZA PORTUGUESA LIGHT PARA VIAGEM |
| 08101 | FAROFA PRONTA LIGHT EM PACOTE PARA VIAGEM |
| 08201 | CAFTA PARA VIAGEM |
| 08301 | VACA ATOLADA PARA VIAGEM |
| 08401 | SALADA OU VERDURA CRUA (EXCETO DE FRUTA) PARA VIAGEM |
| 08402 | SALADA DE FOLHAS (VERDURAS) PARA VIAGEM |
| 08403 | SALADA DE LEGUMES PARA VIAGEM |
| 08501 | ESPETINHO DE QUEIJO PARA VIAGEM |
| 08601 | FEIJAO PREPARADO PARA VIAGEM |
| 08701 | FEIJOADA PARA VIAGEM |
| 08801 | LINGUICA ASSADA PARA VIAGEM |
| 08901 | BANANA DA TERRA FRITA PARA VIAGEM |
| 09001 | ARROZ COM CARNE SECA PARA VIAGEM |
| 09101 | CANJA DE GALINHA PARA VIAGEM |
| 09201 | YAKISSOBA PARA VIAGEM |
| 09301 | RIZOTO PARA VIAGEM |
| 09401 | FAMILIA FELIZ PARA VIAGEM (COMIDA JAPONESA) |
| 09501 | DOBRADINHA PARA VIAGEM |
| 09601 | BATATA PALHA LIGTH PARA VIAGEM |
| 09701 | CHA PREPARADO PARA VIAGEM |
| 09801 | CAFE DA MANHA (VARIOS ALIMENTOS) |
| 00101 | AGUA SANITARIA |
| 00102 | CANDIDA |
| 00103 | Q-BOA |
| 00201 | ESPONJA DE LIMPEZA |
| 00202 | BOMBRIL |
| 00203 | LIXA DE LIMPEZA DE FOGAO |
| 00204 | ESPONJA DE ACO |
| 00205 | ESPONJA DE NYLON |
| 00206 | LA DE ACO |
| 00207 | BUCHA PARA LIMPEZA |
| 00208 | BUCHA DE LIMPEZA |
| 00301 | ESCOVA DE LIMPEZA |
| 00302 | LIMPADOR DE GARRAFA |
| 00303 | LIXA DE FOGAO |
| 00304 | ESCOVA DE MAMADEIRA |
| 00305 | CONJUNTO DE ESCOVAS DE MAMADEIRA |
| 00401 | VASSOURA DE QUALQUER TIPO |
| 00402 | FEITICEIRA |
| 00403 | VASSOURA DE VASO SANITARIO |
| 00404 | ESCOVAO |
| 00405 | VASSOURINHA |
| 00406 | ESFREGAO |
| 00407 | CABO DE VASSOURA |
| 00408 | ESCOVA SANITARIA |
| 00501 | RODO |
| 00502 | PUXADOR |
| 00503 | REFIL PARA RODO |
| 00601 | ESPANADOR |
| 00701 | FLANELA |
| 00702 | PANO DE PO |
| 00703 | PERFEX |
| 00704 | PANO DE LIMPEZA DESCARTAVEL |
| 00705 | PANO DE PIA |
| 00801 | PANO DE CHAO |
| 00802 | SACO DE LIMPEZA DE CHAO |
| 00803 | PANO DE SACO PARA LIMPEZA |
| 00901 | LATA DE LIXO |
| 00902 | SUPORTE DE LATA DE LIXO |
| 00903 | LIXEIRA |
| 00904 | CESTO DE LIXO |
| 00905 | RECIPIENTE DE LIXO |
| 01001 | PA DE LIXO |
| 01101 | SACO DE LIXO DE QUALQUER MATERIAL |
| 01102 | SACOLA DE LIXO |
| 01201 | SACOLA DE COMPRA |
| 01202 | BOLSA DE COMPRA |
| 01203 | SACO DE COMPRA |
| 01301 | EMBALAGEM PLASTIFICADA |
| 01302 | ROLOPAC |
| 01303 | MAGIPAC |
| 01304 | SACO PLASTICO PARA EMBALAGEM |
| 01305 | FILME PLASTICO |
| 01306 | FILME PVC |
| 01401 | DESINFETANTE |
| 01402 | CREOLINA |
| 01403 | PINHO SOL (DESINFETANTE) |
| 01501 | SOLVENTE |
| 01502 | AGUARRAS |
| 01503 | BENZINA |
| 01504 | FAISCA |
| 01505 | REMOVEDOR |
| 01506 | GLICERINA |
| 01601 | SODA CAUSTICA |
| 01602 | DIABO VERDE |
| 01603 | ACIDO MURIATICO |
| 01701 | ALCOOL PARA LIMPEZA |
| 01702 | ALCOOL DE LIMPEZA |
| 01703 | ALCOOL EM GEL |
| 01704 | ALCOOL GEL |
| 01705 | ALCOOL DE CEREAIS |
| 01706 | ALCOOL HIDRATADO |
| 01801 | DETERGENTE |
| 01802 | LAVA LOUCA |
| 01803 | REMOVEDOR DE LIMO |
| 01804 | DESENGORDURANTE |
| 01805 | REMOVEDOR DE GORDURA |
| 01806 | DETERGENTE EM PO |
| 01807 | DETERGENTE LIQUIDO |
| 01901 | SABAO LIQUIDO |
| 02001 | SABAO PASTOSO |
| 02101 | SABAO DE COCO |
| 02102 | SABAO EM PEDRA |
| 02103 | SABAO EM BARRA |
| 02104 | SABAO DE BOLA |
| 02105 | SABAO EM BOLA |
| 02106 | SABAO RALADO |
| 02107 | SABAO EM PASTA |
| 02201 | SABAO EM PO |
| 02301 | SAPONACEO |
| 02302 | SAPOLEO |
| 02303 | PASTA DE POLIR PANELA |
| 02304 | SAPOLIO |
| 02305 | PASTA JOIA |
| 02401 | ALVEJANTE |
| 02402 | OLA |
| 02403 | CONFORT |
| 02404 | ANIL |
| 02405 | AMACIANTE DE ROUPA |
| 02406 | GOMA DE ROUPA |
| 02407 | PASSE BEM |
| 02408 | PRE-LAVAGEM DE QUALQUER MARCA |
| 02409 | TIRA-MANCHA |
| 02410 | REMOVEDOR DE MANCHA DE ROUPA |
| 02411 | AMACIANTE |
| 02412 | AMACIANTE PARA ROUPA |
| 02413 | QBOA (ALVEJANTE) |
| 02414 | VANISH |
| 02415 | LAVA ROUPAS |
| 02501 | DESODORANTE AMBIENTAL |
| 02502 | SACHET |
| 02503 | BOM AR |
| 02504 | DESODORANTE DE SANITARIO |
| 02505 | PURIFICADOR DE AR |
| 02506 | PASTILHA SANITARIA |
| 02507 | DESODORIZADOR DE GELADEIRA |
| 02508 | DESODORANTE DE GELADEIRA |
| 02509 | INCENSO |
| 02510 | HIGIENIZADOR SANITARIO |
| 02511 | REFIL DE DESINFETANTE SANITARIO |
| 02512 | AROMATIZANTE |
| 02513 | ESSENCIA DE EUCALIPTO |
| 02514 | DESODORIZADOR SANITARIO |
| 02515 | PEDRA SANITARIA |
| 02601 | INSETICIDA |
| 02602 | AEROSOL SBP |
| 02603 | REPELENTE |
| 02604 | ESPIRAL DURMA BEM OU OUTRA MARCA |
| 02605 | NAFTALINA |
| 02606 | REFIL INSETICIDA |
| 02607 | ISCA PARA INSETO |
| 02608 | JIMO CUPIM |
| 02609 | SENTINELA ESPIRAL |
| 02701 | RATICIDA |
| 02702 | VENENO PARA RATO |
| 02703 | CHUMBINHO MATA-RATOS |
| 02801 | BOMBA DE INSETICIDA |
| 02901 | RATOEIRA |
| 03001 | PALHA DE ACO |
| 03101 | CERA DE ASSOALHO |
| 03102 | CERA LIQUIDA DE ASSOALHO |
| 03201 | OLEO DE MOVEL |
| 03202 | OLEO DE PEROBA |
| 03203 | LUSTRA MOVEL |
| 03301 | POLIDOR DE METAL |
| 03302 | SILVO |
| 03303 | PASTA DE BRILHO |
| 03304 | LAVINOX |
| 03305 | BRASSO |
| 03306 | KAOL |
| 03307 | LIMPA ALUMINIO |
| 03308 | BRILHA ALUMINIO |
| 03401 | GRAXA DE SAPATO |
| 03402 | CERA DE SAPATO |
| 03403 | POMADA DE CALCADO |
| 03501 | ESCOVA DE ROUPA |
| 03502 | ESCOVA DE SAPATO |
| 03503 | ESCOVA DE LAVAR ROUPA |
| 03504 | ESCOVA DE ENGRAXAR SAPATO |
| 03601 | OLEO DE MAQUINA |
| 03701 | PREGADOR DE ROUPA |
| 03702 | PRENDEDOR DE ROUPA |
| 03801 | CORDA DE VARAL |
| 03802 | CORDA DE ROUPA |
| 03901 | PAPEL TOALHA |
| 04001 | DESENTUPIDOR DE RALO |
| 04002 | DESENTUPIDOR DE PIA |
| 04101 | PALITO |
| 04102 | PALITOS DE DENTE |
| 04103 | PALITO DE DENTE |
| 04201 | SULFATO DE ALUMINIO PARA LIMPEZA DOMESTICA |
| 04202 | SULFATO DE SODIO PARA LIMPEZA DOMESTICA |
| 04301 | GUARDANAPO DE PAPEL |
| 04401 | PRATO OU BANDEJA DE PAPEL ALUMINIZADO OU PLASTICO |
| 04402 | EMBALAGEM ALUMINIZADA DE CONGELAMENTO DE ALIMENTOS |
| 04403 | PRATO DESCARTAVEL |
| 04501 | SABAO NAO-ESPECIFICADO |
| 04601 | COADOR DE CAFE DE PAPEL |
| 04602 | FILTRO DE CAFE DE PAPEL |
| 04603 | FILTRO DE PAPEL |
| 04701 | TOALHA DE PAPEL |
| 04801 | CANUDINHO DE PAPEL OU PLASTICO |
| 04901 | FORMA, COPO, ETC. DE PAPEL OU PAPELAO |
| 04902 | FORMINHA DE PAPEL |
| 04903 | PAPEL DE BALA |
| 04904 | COPO DE PAPEL OU PLASTICO |
| 04905 | COPO DESCARTAVEL |
| 05001 | PAPEL ALUMINIO OU ALUMINIZADO |
| 05002 | FOGAO LIMPO |
| 05003 | LIMPA-FOGAO |
| 05004 | PAPEL ALUMINIO PARA FOGAO |
| 05005 | FORRA-FOGAO |
| 05101 | PAPEL ABSORVENTE DE COPA E COZINHA |
| 05201 | PAPEL CELOFANE |
| 05301 | FOSFOROS |
| 05302 | CAIXA DE FOSFOROS |
| 05303 | FOSFORO |
| 05401 | VELA DE ILUMINACAO |
| 05501 | LAMPADA DE QUALQUER TIPO |
| 05502 | LAMPADA |
| 05503 | LAMPADA FLUORESCENTE |
| 05504 | LAMPADA FRIA |
| 05505 | LAMPADA BRANCA |
| 05506 | LAMPADA ELETRONICA |
| 05507 | LAMPADA TIPO ECONOMICA |
| 05601 | QUEROSENE PARA LIMPEZA |
| 05701 | CARVAO VEGETAL PARA USO DOMESTICO |
| 05702 | CARVAO PARA USO DOMESTICO |
| 05703 | CARVAO VEGETAL PARA CHURRASCO |
| 05704 | CARVAO PARA CHURRASCO |
| 05801 | GASOLINA PARA LIMPEZA |
| 05901 | PILHA |
| 06001 | CERA DE MOVEIS |
| 06101 | SHAMPU DE CARPETE |
| 06102 | SHAMPU DE TAPETE |
| 06103 | LIMPA-TAPETE |
| 06104 | LIMPA-CARPETE |
| 06201 | TALHER DE PLASTICO |
| 06202 | GARFO DE PLASTICO |
| 06203 | FACA DE PLASTICO |
| 06204 | COLHER DE PLASTICO |
| 06301 | AMONIACO |
| 06302 | VEJA |
| 06303 | AJAX |
| 06304 | FURIA |
| 06305 | JATO |
| 06306 | MULTILIMPADOR INSTANTANEO |
| 06307 | CONCENTRADO DE LIMPEZA |
| 06308 | LIMPADOR INSTANTANEO |
| 06309 | PULVERIZADOR DE MULTIPLA ACAO |
| 06310 | LIMPADOR DE MULTIPLA ACAO |
| 06311 | MULTIUSO |
| 06312 | LIMPADOR MULTIUSO |
| 06401 | ANTIMOFO |
| 06402 | DESUMIDIFICADOR |
| 06403 | MOFIL |
| 06501 | LIMPA-VIDROS |
| 06601 | LIMPA-FORNO |
| 06602 | EASY OFF |
| 06701 | VELA DE ANIVERSARIO |
| 06801 | VAPORIZADOR ELETRICO CONTRA INSETO |
| 06802 | REPELENTE ELETRICO DE INSETO |
| 06803 | PROTECTOR |
| 06804 | FUMINSECT |
| 06901 | ESTOPA DE LIMPEZA DA CASA |
| 07001 | ALGODAO DE LIMPEZA |
| 07101 | ANTIFERRUGEM |
| 07102 | TIRA-FERRUGEM |
| 07201 | SAQUINHO DE LANCHE DE PLASTICO OU PAPEL |
| 07202 | SACO DE LANCHE DE PLASTICO OU PAPEL |
| 07301 | ANILINA E TINTA DE ASSOALHO |
| 07302 | TINTA DE ASSOALHO |
| 07303 | CORANTE DE CERA PARA ASSOALHO |
| 07401 | ESFREGADEIRA DE LAVAR ROUPA |
| 07501 | CLORO LIQUIDO DE LIMPEZA DOMESTICA |
| 07601 | TALHER DE MADEIRA |
| 07602 | PALITO PARA SORVETE |
| 07603 | PALITO PARA CHURRASCO |
| 07604 | ESPETINHO PARA CHURRASCO |
| 07605 | ESPETO PARA CHURRASCO (MADEIRA) |
| 07701 | SECADOR DE MAQUINA DE LAVAR LOUCA |
| 07702 | SABAO EM PO DE MAQUINA DE LAVAR LOUCA |
| 07801 | FOLHA DE BANANEIRA PARA EMBALAGEM |
| 07901 | FILTRO DE PAPEL DE EXAUSTOR |
| 08001 | PAPEL MANTEIGA |
| 08101 | CHAMINHA |
| 08201 | BALDE |
| 08301 | CARVAO MINERAL |
| 08401 | ISQUEIRO PARA FOGAO |
| 08402 | ISQUEIRO DESCARTAVEL PARA FOGAO |
| 08403 | ISQUEIRO PARA ACENDER FOGAO |
| 08501 | FOGOTINI PARA ACENDER CHURRASQUEIRA |
| 08502 | GEL FOGOTINI PARA ACENDER CHURRASQUEIRA |
| 08601 | OLEO DE EUCALIPTO |
| 08701 | BOBINA DE PLASTICO PARA FREEZER |
| 08801 | FILTRO PARA ASPIRADOR |
| 08901 | LUVA DE LIMPEZA DE BORRACHA |
| 08902 | LUVA DE LIMPEZA |
| 08903 | LUVA DE LIMPEZA DE PLASTICO |
| 08904 | AVENTAL DE PLASTICO |
| 09001 | LENHA PARA COMBUSTIVEL DOMESTICO |
| 09002 | ACHA DE LENHA PARA COMBUSTIVEL DOMESTICO |
| 09003 | GALHO DE LENHA PARA COMBUSTIVEL DOMESTICO |
| 09101 | COADOR DE CAFE DE PANO |
| 09201 | FUNGICIDA |
| 09301 | LIMPA CERAMICA |
| 09302 | LIMPADOR DE CERAMICA |
| 09303 | LIMPA ARDOSIA |
| 09304 | LIMPA PISO |
| 09305 | LIMPADOR DE PISO |
| 09401 | BACTERICIDA |
| 09501 | PEDRA PARA POLIR FOGAO |
| 00101 | ALIMENTO DE CAO |
| 00102 | RACAO DE CAO |
| 00103 | ALIMENTO PARA CAO |
| 00104 | RACAO PARA CAO |
| 00105 | BISCOITO CANINO |
| 00106 | OSSO PARA CAO |
| 00107 | BISCOITO PARA CAO |
| 00201 | ALIMENTO DE GATO |
| 00202 | RACAO DE GATO |
| 00203 | ALIMENTO PARA GATO |
| 00204 | RACAO PARA GATO |
| 00301 | ALIMENTO DE PEIXE |
| 00302 | RACAO DE PEIXE |
| 00303 | ALIMENTO PARA PEIXE |
| 00304 | RACAO PARA PEIXE |
| 00401 | ALIMENTO DE BOVINO |
| 00402 | RACAO DE BOVINO |
| 00403 | ALIMENTO PARA BOVINO |
| 00404 | RACAO PARA BOVINO |
| 00405 | FARELO DE TRIGO PARA BOVINO |
| 00501 | ALIMENTO DE EQUINO |
| 00502 | RACAO DE EQUINO |
| 00503 | ALIMENTO PARA EQUINO |
| 00504 | RACAO PARA EQUINO |
| 00601 | ALIMENTO DE CAPRINO |
| 00602 | RACAO DE CAPRINO |
| 00603 | ALIMENTO PARA CAPRINO |
| 00604 | RACAO PARA CAPRINO |
| 00701 | ALIMENTO DE GALINHA |
| 00702 | ALIMENTO PARA GALINHA |
| 00703 | RACAO DE GALINHA |
| 00704 | RACAO PARA GALINHA |
| 00705 | MILHO PARA GALINHA |
| 00706 | ALIMENTO DE FRANGO |
| 00707 | ALIMENTO PARA FRANGO |
| 00708 | RACAO DE FRANGO |
| 00709 | RACAO PARA FRANGO |
| 00710 | MILHO PARA FRANGO |
| 00711 | RACAO DE PINTO |
| 00801 | ALIMENTO DE OVINO |
| 00802 | RACAO DE OVINO |
| 00803 | ALIMENTO PARA OVINO |
| 00804 | RACAO PARA OVINO |
| 00901 | ALIMENTO DE PASSARINHO |
| 00902 | RACAO DE PASSARINHO |
| 00903 | ALIMENTO PARA PASSARINHO |
| 00904 | RACAO PARA PASSARINHO |
| 00905 | SEMENTE DE GIRASSOL |
| 00906 | ALPISTE |
| 00907 | GIRASSOL (SEMENTE) |
| 00908 | PAINCO |
| 01001 | ALIMENTO DE COELHO |
| 01002 | RACAO DE COELHO |
| 01003 | ALIMENTO PARA COELHO |
| 01004 | RACAO PARA COELHO |
| 01201 | ALIMENTO DE OUTRO ANIMAL |
| 01202 | RACAO DE OUTRO ANIMAL |
| 01203 | ALIMENTO PARA OUTRO ANIMAL |
| 01204 | RACAO PARA OUTRO ANIMAL |
| 01301 | ALIMENTO PARA ANIMAL NAO-ESPECIFICADO |
| 01302 | FARELO DE SOJA PARA ANIMAL NAO-ESPECIFICADO |
| 01303 | FARELO DE ARROZ PARA ANIMAL NAO-ESPECIFICADO |
| 01304 | FARELO DE MILHO PARA OUTRO ANIMAL NAO-ESPECIFICADO |
| 01305 | MANDIOCA PARA ANIMAL NAO-ESPECIFICADO |
| 01306 | ALFAFA PARA ANIMAL NAO-ESPECIFICADO |
| 01307 | CAROCO DE ALGODAO PARA ANIMAL NAO-ESPECIFICADO |
| 01308 | LEITE PARA ANIMAL NAO-ESPECIFICADO |
| 01309 | RACAO NAO-ESPECIFICADA PARA ANIMAL NAO-ESPECIFICADO |
| 01310 | FARELO DE TRIGO PARA ANIMAL NAO ESPECIFICADO |
| 01311 | ARROZ PARA ANIMAL NAO ESPECIFICADO |
| 01401 | PRODUTO DE LIMPEZA E HIGIENE DE ANIMAL |
| 01402 | FRONTLINE |
| 01403 | SHAMPU PARA CAO |
| 01501 | RACAO PARA SUINO |
| 01502 | RACAO DE PORCO |
| 00101 | FLOR NATURAL |
| 00102 | ROSA |
| 00103 | CRAVO |
| 00104 | MARGARIDA |
| 00105 | GLADIOLOS |
| 00106 | ORQUIDEA |
| 00107 | MONSENHOR |
| 00108 | FLOR DO CAMPO |
| 00109 | PALMA |
| 00110 | COPO DE LEITE |
| 00111 | CRISANTEMO |
| 00112 | ARRANJO DE FLORES NATURAIS |
| 00113 | KALANCHOE |
| 00114 | CALENDULA |
| 00115 | VIOLETA |
| 00116 | MALVA |
| 00101 | CREME DENTAL E FIO DENTAL |
| 00102 | FIO DENTAL |
| 00103 | PASTA DE DENTE |
| 00104 | GEL DENTAL |
| 00105 | CREME DENTAL |
| 00106 | PASTA DENTAL |
| 00107 | FITA DENTAL |
| 00201 | ESCOVA DE DENTE |
| 00202 | ESCOVA DENTAL |
| 00301 | SABONETE |
| 00302 | SABONETE LIQUIDO |
| 00303 | SABONETE EM GEL |
| 00401 | SHAMPU, CONDICIONADOR E OUTROS PRODUTOS DE CABELO |
| 00402 | CREME RINSE |
| 00411 | AGUA DE QUINA |
| 00412 | LOCAO DE CABELO |
| 00413 | RESTAURADOR CAPILAR |
| 00414 | SHAMPU |
| 00415 | CREME ENXAGUANTE DE CABELO |
| 00416 | NEUTROX |
| 00417 | CREME DE CABELO |
| 00420 | CONDICIONADOR CAPILAR |
| 00421 | LOCAO TONICA OU TONIFICANTE CAPILAR |
| 00422 | OUTROS PRODUTOS DE CABELO |
| 00423 | CREME DEFINIDOR DE CACHOS |
| 00424 | SHAMPU E CONDICIONADOR |
| 00425 | CREME HIDRATANTE CAPILAR |
| 00426 | HIDRATANTE CAPILAR |
| 00427 | RECONDICIONADOR CAPILAR |
| 00433 | XAMPU |
| 00434 | CREME PARA CABELO |
| 00501 | DESODORANTE PESSOAL |
| 00502 | DESODORANTE ANTITRANSPIRANTE |
| 00503 | DESODORANTE ANTISSEPTICO |
| 00601 | CREME, PINCEL E OUTROS PRODUTOS DE BARBA |
| 00602 | CREME DE BARBEAR |
| 00603 | PINCEL DE BARBA |
| 00605 | GEL PARA BARBA |
| 00606 | ESPUMA DE BARBEAR |
| 00701 | LAMINA DE BARBEAR |
| 00702 | GILETE |
| 00705 | PRESTOBARBA |
| 00706 | APARELHO DE BARBEAR DESCARTAVEL |
| 00707 | APARELHO DE BARBA DESCARTAVEL |
| 00708 | ESTOJO DE BARBEAR DESCARTAVEL |
| 00709 | BARBEADOR DESCARTAVEL |
| 00710 | CARGA DE LAMINA DE BARBEAR |
| 00711 | APARELHO DE BARBEAR NAO ELETRICO DESCARTAVEL |
| 00712 | REFIL PARA APARELHO DE BARBEAR DESCARTAVEL |
| 00801 | ABSORVENTE HIGIENICO |
| 00802 | ABSORVENTE FEMININO |
| 00901 | LENCO DE PAPEL |
| 00902 | LENCO DE BEBE UMEDECIDO |
| 00903 | LENCO UMEDECIDO DE BEBE |
| 00904 | LENCO DESCARTAVEL |
| 01001 | COTONETE DE USO COLETIVO |
| 01101 | PRODUTO DE BANHO |
| 01102 | SAIS DE BANHO |
| 01103 | BUCHA DE BANHO |
| 01104 | ESCOVA DE BANHO |
| 01105 | PEDRA POME |
| 01106 | ESPONJA DE BANHO |
| 01201 | PAPEL HIGIENICO |
| 01301 | BARBEADOR DE LAMINA |
| 01401 | BUCHA VEGETAL |
| 01501 | ANTI-SEPTICO BUCAL |
| 01502 | DESODORANTE BUCAL |
| 00101 | CESTA DE PASCOA |
| 00201 | CESTA DE NATAL |
| 00301 | CESTA DE CAFE DA MANHA |
| 00401 | VAREJAO |
| 00501 | CESTA BASICA |
| 00601 | SACOLA COBAL |
| 00701 | FEIRINHA |
| 00801 | SACOLAO |
| 00901 | AGREGADO |
| 00902 | AGREGADO (FRUTAS) |
| 00903 | AGREGADO (FRUTAS E LEGUMES) |
| 00904 | AGREGADO (LEGUMES E VERDURAS) |
| 00905 | AGREGADO (LEGUMES) |
| 00906 | AGREGADO (VERDURAS) |
| 00907 | AGREGADO (FRUTAS E VERDURAS) |
| 00908 | AGREGADO (ALIMENTOS) |
| 00909 | AGREGADO (CARNES BOVINA, SUINA, DE AVES) |
| 00910 | AGREGADO (FRUTAS, VERDURAS, LEGUMES) |
| 00911 | AGREGADO (FRIOS) |
| 00912 | AGREGADO (CARNES, VERDURAS, FRUTAS) |
| 00913 | AGREGADO (FRANGO, VERDURAS, LEGUMES) |
| 00914 | AGREGADO (PAES) |
| 00915 | AGREGADO (PRODUTOS DE LIMPEZA) |
| 00916 | AGREGADO COMPRAS |
| 00917 | AGREGADO (TEMPEROS) |
| 00918 | AGREGADO (FRUTAS E TEMPEROS) |
| 00919 | AGREGADO (CARNES BOVINAS) |
| 00920 | AGREGADO (BEBIDAS) |
| 00921 | AGREGADO (CARNE E CERVEJA) |
| 00922 | QUITANDAS (PAES, BOLOS, BISCOITOS, ROSCAS, DOCES, ETC) |
| 00923 | AGREGADO (VERDURAS E PEIXES) |
| 00924 | AGREGADO (PEIXES) |

Categorias por Nome:

| 03702 | 13° SALARIO |
| --- | --- |
| 01203 | ABA DE FILE |
| 02701 | ABACATE |
| 02601 | ABACAXI |
| 02603 | ABACAXI CAIANO |
| 02419 | ABACAXI DESIDRATADO |
| 01306 | ABACAXI EM CALDA |
| 09209 | ABACAXI EM CALDA DIET |
| 09210 | ABACAXI EM CALDA DIETETICO |
| 02604 | ABACAXI PEROLA |
| 02410 | ABACAXI SECO OU DESIDRATADO |
| 01403 | ABADA |
| 01002 | ABAJUR |
| 03738 | ABARA |
| 00212 | ABARA PARA VIAGEM |
| 02102 | ABECEDARIA |
| 02502 | ABERTURA DE CREDITO |
| 08001 | ABIU |
| 03901 | ABOBORA |
| 03502 | ABOBORA ALMISCAR |
| 03404 | ABOBORA BAIANA |
| 03706 | ABOBORA BAIANINHA (ABOBRINHA) |
| 03809 | ABOBORA CABOCLO |
| 06502 | ABOBORA CABOTIA |
| 06501 | ABOBORA CABOTIAN |
| 06503 | ABOBORA CABUTIA |
| 06504 | ABOBORA CABUTIAN |
| 03304 | ABOBORA CARNEIRA |
| 03503 | ABOBORA CATINGA |
| 06601 | ABOBORA CAXACO |
| 03504 | ABOBORA CHEIROSA |
| 03902 | ABOBORA COMUM |
| 03605 | ABOBORA DA AGUA |
| 03406 | ABOBORA DA BAHIA |
| 03603 | ABOBORA DA PRAIA |
| 03604 | ABOBORA DAGUA |
| 03602 | ABOBORA DAGUA VERDE |
| 03601 | ABOBORA DE AGUA |
| 03802 | ABOBORA DE CABOCLO |
| 03810 | ABOBORA DE LEITE |
| 03801 | ABOBORA DE PESCOCO |
| 03305 | ABOBORA DE PORCO |
| 03803 | ABOBORA DO SERTAO |
| 03403 | ABOBORA GIGANTE |
| 06701 | ABOBORA GOIANINHA |
| 03402 | ABOBORA GRANDE |
| 06801 | ABOBORA HIBRIDA |
| 03804 | ABOBORA JACARE |
| 03310 | ABOBORA JAPONESA |
| 03705 | ABOBORA JAPONESA (ABOBRINHA) |
| 06505 | ABOBORA KABUTIA |
| 03903 | ABOBORA MADURA |
| 03407 | ABOBORA MARANHAO |
| 03501 | ABOBORA MELAO |
| 03401 | ABOBORA MENINA |
| 03302 | ABOBORA MOCANGA |
| 03301 | ABOBORA MOGANGA |
| 03303 | ABOBORA MORANGA |
| 03309 | ABOBORA MORANGUINHA |
| 06401 | ABOBORA PAULISTA |
| 06901 | ABOBORA SECA |
| 03709 | ABOBORA VERDE |
| 03405 | ABOBORA VERMELHA |
| 03701 | ABOBRINHA |
| 07101 | ABOBRINHA BRANCA |
| 03704 | ABOBRINHA BRASILEIRA |
| 07001 | ABOBRINHA CAIPIRA |
| 03703 | ABOBRINHA ITALIANA |
| 07201 | ABOBRINHA MIRIM |
| 03702 | ABOBRINHA VERDE |
| 03803 | ABONO DE FERIAS |
| 00103 | ABONO DO PIS/PASEP |
| 03805 | ABONO PECUNIARIO DE FERIAS |
| 08101 | ABRICO |
| 00806 | ABRIDOR DE GARRAFA, LATA, ETC. |
| 00801 | ABRIDOR E AFIADOR ELETRICO |
| 00803 | ABRIDOR ELETRICO |
| 00604 | ABRIGO |
| 00802 | ABSORVENTE FEMININO |
| 31203 | ABSORVENTE GERIATRICO |
| 00801 | ABSORVENTE HIGIENICO |
| 05501 | ACADEMIA |
| 05502 | ACADEMIA DE GINASTICA |
| 05503 | ACADEMIA DE MUSCULACAO |
| 01601 | ACAFRAO |
| 01701 | ACAFRAO EM PO |
| 01706 | ACAI |
| 02001 | ACAI (EMULSAO) |
| 02002 | ACAI COM GRANOLA |
| 07801 | ACAI COM GRANOLA VIAGEM |
| 01701 | ACAI EM EMULSAO |
| 01714 | ACAI EM PAPA |
| 01711 | ACAI FRUTA |
| 04702 | ACAJA (TAPEREBA) |
| 00707 | ACAMPAMENTOS |
| 02001 | ACAO JUDICIAL TRABALHISTA |
| 03711 | ACARAJE |
| 04105 | ACARAJE E REFRIGERANTE |
| 00209 | ACARAJE PARA VIAGEM |
| 01501 | ACELGA |
| 00801 | ACEM |
| 00804 | ACEM COM OSSO |
| 14102 | ACENDEDOR DE FOGAO |
| 14101 | ACENDEDOR ELETRONICO |
| 07701 | ACEROLA |
| 06502 | ACEROLA ORGANICA |
| 08901 | ACEROLA ORGANICA |
| 00501 | ACESSO A INTERNET (PROVEDOR, A CABO, COMUNICACAO VIA SATELITE, ETC.) |
| 01801 | ACESSO A INTERNET DE OUTROS IMOVEIS |
| 00501 | ACESSO A INTERNET EM LOJA |
| 01901 | ACESSO A TV A CABO DE OUTROS IMOVEIS |
| 00502 | ACESSORIO (MAO-DE-OBRA) |
| 06302 | ACESSORIOS DE COMPUTADOR |
| 05201 | ACESSORIOS DE IMPRESSORA |
| 00101 | ACESSORIOS DE INSTRUMENTOS MUSICAIS |
| 06301 | ACESSORIOS DE MICROCOMPUTADOR |
| 01301 | ACESSORIOS DE TELEFONE CELULAR |
| 12301 | ACESSORIOS DE VIDEO GAME |
| 08201 | ACESSORIOS DE VIDEOCASSETE |
| 07801 | ACESSORIOS PARA BANHEIRO |
| 07601 | ACESSORIOS PARA BANHEIRO |
| 17801 | ACESSORIOS PARA MAQUINA FOTOGRAFICA |
| 00309 | ACESSORIOS PARA SKATE |
| 00603 | ACETONA |
| 09002 | ACHA DE LENHA PARA COMBUSTIVEL DOMESTICO |
| 03104 | ACHOCOLATADO EM LEITE |
| 06804 | ACHOCOLATADO EM LEITE DIET |
| 06814 | ACHOCOLATADO EM LEITE DIETETICO |
| 06704 | ACHOCOLATADO EM LEITE LIGHT |
| 00821 | ACHOCOLATADO EM PO |
| 10321 | ACHOCOLATADO EM PO DIET |
| 10343 | ACHOCOLATADO EM PO DIETETICO |
| 10221 | ACHOCOLATADO EM PO LIGHT |
| 03304 | ACHOCOLATADO INDUSTRIALIZADO |
| 03113 | ACHOCOLATADO LIQUIDO |
| 02201 | ACIDENTE DE TRABALHO (PREVIDENCIA PUBLICA) |
| 01603 | ACIDO MURIATICO |
| 00301 | ACOES (APLICACAO) |
| 00302 | ACOES (DEPOSITO/APLICACAO) |
| 00301 | ACOES (RESGATE) |
| 00305 | ACOES (RETIRADA/RESGATE) |
| 00302 | ACOES (VENDA) |
| 01201 | ACOMPANHANTE |
| 02601 | ACOMPANHANTE (CUIDADOR) |
| 02602 | ACOMPANHANTE DE QUARTO HOSPITALAR |
| 00701 | ACORDEAO |
| 01002 | ACORTINADO |
| 01212 | ACTIVIA (IOGURTE) |
| 06602 | ACUCAR |
| 00303 | ACUCAR AMARELO PRETO |
| 08803 | ACUCAR AMARELO PRETO ORGANICO |
| 06603 | ACUCAR COMUM |
| 08903 | ACUCAR COMUM ORGANICO |
| 00201 | ACUCAR CRISTAL |
| 08701 | ACUCAR CRISTAL ORGANICO |
| 00203 | ACUCAR CRISTALIZADO |
| 08703 | ACUCAR CRISTALIZADO ORGANICO |
| 06701 | ACUCAR DE BAUNILHA |
| 00301 | ACUCAR DEMERARA |
| 08801 | ACUCAR DEMERARA ORGANICO |
| 00202 | ACUCAR GRANULADO |
| 08702 | ACUCAR GRANULADO ORGANICO |
| 00204 | ACUCAR GROSSO |
| 06601 | ACUCAR INDETERMINADO |
| 08901 | ACUCAR INDETERMINADO ORGANICO |
| 07001 | ACUCAR LIGHT |
| 00306 | ACUCAR MASCAVADO |
| 08806 | ACUCAR MASCAVADO ORGANICO |
| 00304 | ACUCAR MASCAVO |
| 08804 | ACUCAR MASCAVO ORGANICO |
| 06802 | ACUCAR MOIDO |
| 08902 | ACUCAR ORGANICO |
| 09101 | ACUCAR ORGANICO LIGHT |
| 00305 | ACUCAR PRETO |
| 08805 | ACUCAR PRETO ORGANICO |
| 00101 | ACUCAR REFINADO |
| 07002 | ACUCAR REFINADO LIGHT |
| 08601 | ACUCAR REFINADO ORGANICO |
| 00102 | ACUCAR TRIFILTRADO |
| 07003 | ACUCAR TRIFILTRADO LIGHT |
| 08602 | ACUCAR TRIFILTRADO ORGANICO |
| 06801 | ACUCAR TRITURADO |
| 05101 | ACUCAREIRO |
| 03401 | ACUPUNTURA |
| 03403 | ACUPUNTURA (TRATAMENTO) |
| 05203 | ADES LIGHT |
| 02303 | ADES ORIGINAL |
| 00803 | ADESIVO PLASTICO |
| 00801 | ADESIVO PLASTICO OU PANFLETO |
| 30003 | ADESIVOS |
| 04002 | ADESIVOS ANTITABAGISMO |
| 02202 | ADESIVOS INTRADERMICOS (MENOPAUSA) |
| 02701 | ADESTRAMENTO DE ANIMAL DOMESTICO |
| 03802 | ADIANTAMENTO DE FERIAS |
| 00801 | ADICIONAIS DE ALUGUEL DE GARAGEM (JUROS, MULTA, ETC.) |
| 00901 | ADICIONAIS DE CONDOMINIO (JUROS, MULTA, ETC.) |
| 01001 | ADICIONAIS DE IMPOSTO PREDIAL (JUROS, MULTA, ETC.) |
| 01401 | ADICIONAIS DE IPTR |
| 00701 | ADICIONAIS DE PRESTACAO DO IMOVEL (JUROS, MULTA, ETC.) |
| 00601 | ADICIONAIS DO ALUGUEL DO IMOVEL (JUROS, MULTA, ETC.) |
| 01702 | ADICIONAL DE INSALUBRIDADE |
| 01703 | ADICIONAL DE PERICULOSIDADE |
| 01704 | ADICIONAL NOTURNO |
| 04001 | ADITIVOS |
| 01202 | ADMINISTRADORA DE IMOVEL (COMISSAO) |
| 06902 | ADOCANTE |
| 01801 | ADOCANTE ARTIFICIAL |
| 05302 | ADOCANTE CONCENTRADO NATURAL |
| 07101 | ADOCANTE DIET |
| 07102 | ADOCANTE DIETETICO |
| 06903 | ADOCANTE EM PO |
| 07103 | ADOCANTE EM PO DIET |
| 07105 | ADOCANTE EM PO DIETETICO |
| 14002 | ADOCANTE EM PO LIGHT |
| 06901 | ADOCANTE INDETERMINADO |
| 09801 | ADOCANTE LIGHT |
| 14001 | ADOCANTE LIGHT |
| 06904 | ADOCANTE LIQUIDO |
| 07104 | ADOCANTE LIQUIDO DIET |
| 07106 | ADOCANTE LIQUIDO DIETETICO |
| 14003 | ADOCANTE LIQUIDO LIGHT |
| 05301 | ADOCANTE NATURAL CONCENTRADO |
| 00201 | ADVOGADO |
| 00508 | AEROBARCO |
| 02505 | AEROGRAFO |
| 00106 | AEROGRAMA |
| 02602 | AEROSOL SBP |
| 00802 | AFIADOR ELETRICO |
| 02708 | AFIAMBRADO |
| 00108 | AGASALHO |
| 00101 | AGASALHO (CASACO, SUETER, ETC) |
| 00101 | AGASALHO (CASACO, SUETER, ETC.) |
| 00101 | AGASALHO (CASACO, SUETER, ETC.) |
| 03914 | AGASALHO PARA ANIMAL |
| 04902 | AGATA OU ESMALTADA (PECA DE DECORACAO) |
| 00236 | AGENDA |
| 12903 | AGENDA ELETRONICA |
| 01202 | AGENDA ELETRONICA INFANTIL |
| 02003 | AGENDA ESCOLAR |
| 02201 | AGENTE JOVEM - PROGRAMA GOVERNAMENTAL PARA JOVEM DESEMPREGADO |
| 99901 | AGREGADO |
| 99901 | AGREGADO |
| 99901 | AGREGADO |
| 99901 | AGREGADO |
| 99901 | AGREGADO |
| 99901 | AGREGADO |
| 99901 | AGREGADO |
| 99901 | AGREGADO |
| 99901 | AGREGADO |
| 99901 | AGREGADO |
| 99901 | AGREGADO |
| 09901 | AGREGADO |
| 99901 | AGREGADO |
| 99901 | AGREGADO |
| 99901 | AGREGADO |
| 99901 | AGREGADO |
| 99901 | AGREGADO |
| 99901 | AGREGADO |
| 99901 | AGREGADO |
| 99901 | AGREGADO |
| 99901 | AGREGADO |
| 99901 | AGREGADO |
| 99901 | AGREGADO |
| 99901 | AGREGADO |
| 99901 | AGREGADO |
| 99901 | AGREGADO |
| 99901 | AGREGADO |
| 99901 | AGREGADO |
| 99901 | AGREGADO |
| 99901 | AGREGADO |
| 99901 | AGREGADO |
| 99901 | AGREGADO |
| 99901 | AGREGADO |
| 99901 | AGREGADO |
| 99901 | AGREGADO |
| 99901 | AGREGADO |
| 99901 | AGREGADO |
| 99901 | AGREGADO |
| 99901 | AGREGADO |
| 99901 | AGREGADO |
| 99901 | AGREGADO |
| 99901 | AGREGADO |
| 00901 | AGREGADO |
| 00908 | AGREGADO (ALIMENTOS) |
| 00920 | AGREGADO (BEBIDAS) |
| 00921 | AGREGADO (CARNE E CERVEJA) |
| 00909 | AGREGADO (CARNES BOVINA, SUINA, DE AVES) |
| 00919 | AGREGADO (CARNES BOVINAS) |
| 00912 | AGREGADO (CARNES, VERDURAS, FRUTAS) |
| 00913 | AGREGADO (FRANGO, VERDURAS, LEGUMES) |
| 00911 | AGREGADO (FRIOS) |
| 00903 | AGREGADO (FRUTAS E LEGUMES) |
| 00918 | AGREGADO (FRUTAS E TEMPEROS) |
| 00907 | AGREGADO (FRUTAS E VERDURAS) |
| 00902 | AGREGADO (FRUTAS) |
| 00910 | AGREGADO (FRUTAS, VERDURAS, LEGUMES) |
| 00904 | AGREGADO (LEGUMES E VERDURAS) |
| 00905 | AGREGADO (LEGUMES) |
| 00914 | AGREGADO (PAES) |
| 00924 | AGREGADO (PEIXES) |
| 00915 | AGREGADO (PRODUTOS DE LIMPEZA) |
| 00917 | AGREGADO (TEMPEROS) |
| 00923 | AGREGADO (VERDURAS E PEIXES) |
| 00906 | AGREGADO (VERDURAS) |
| 00916 | AGREGADO COMPRAS |
| 03804 | AGREGADO FERIAS E DECIMO TERCEIRO |
| 01301 | AGRIAO |
| 01302 | AGRIAO COMUM |
| 01306 | AGRIAO DA TERRA |
| 01303 | AGRIAO DAS HORTAS |
| 01304 | AGRIAO DE AGUA |
| 02104 | AGRIAO DO BRASIL (JAMBUACU) |
| 02103 | AGRIAO DO PARA (JAMBUACU) |
| 01307 | AGRIAO DOS JARDINS |
| 01308 | AGRIAO HIDROPONICO |
| 01305 | AGRIAO ORDINARIO |
| 10301 | AGRIAO ORGANICO |
| 01102 | AGRIMENSOR |
| 11902 | AGRIN |
| 30213 | AGUA BORICADA |
| 01006 | AGUA COM GAS |
| 01012 | AGUA COM GAS E LIMAO |
| 01013 | AGUA COM GAS SABOR LIMAO |
| 01015 | AGUA COM SABOR GASEIFICADA |
| 03001 | AGUA DE COCO |
| 02101 | AGUA DE COCO |
| 00202 | AGUA DE COLONIA |
| 01501 | AGUA DE OUTROS IMOVEIS (CARRO PIPA) |
| 08003 | AGUA DE POCO |
| 00411 | AGUA DE QUINA |
| 30209 | AGUA DESTILADA |
| 04002 | AGUA DESTILADA DE BATERIA |
| 08002 | AGUA DOCE |
| 00101 | AGUA E ESGOTO (M3) |
| 01301 | AGUA E ESGOTO DE OUTROS IMOVEIS (M3) |
| 01008 | AGUA FLUORADA |
| 02504 | AGUA GASEIFICADA COM SABOR |
| 03807 | AGUA MEDICINAL |
| 02501 | AGUA MINERAL |
| 01004 | AGUA MINERAL |
| 02502 | AGUA MINERAL COM GAS |
| 01007 | AGUA MINERAL COM GAS |
| 01016 | AGUA MINERAL SABOR LARANJA |
| 01010 | AGUA MINERAL SABOR LIMAO |
| 01014 | AGUA MINERAL SABOR TANGERINA |
| 02503 | AGUA MINERAL SEM GAS |
| 01005 | AGUA MINERAL SEM GAS |
| 30205 | AGUA OXIGENADA |
| 02613 | AGUA OXIGENADA PARA CABELO |
| 00201 | AGUA PARA USO GERAL |
| 08001 | AGUA POTAVEL |
| 01009 | AGUA PURIFICADA |
| 00101 | AGUA SANITARIA |
| 01701 | AGUA TONICA |
| 04902 | AGUA TONICA |
| 08903 | AGUA TONICA DIET |
| 12902 | AGUA TONICA DIET |
| 08904 | AGUA TONICA DIETETICA |
| 12905 | AGUA TONICA DIETETICA |
| 09002 | AGUA TONICA LIGHT |
| 00901 | AGUARDENTE |
| 02601 | AGUARDENTE DE ALCACHOFRA |
| 04101 | AGUARDENTE DE ALCACHOFRA ORGANICA |
| 02301 | AGUARDENTE DE ARROZ |
| 03901 | AGUARDENTE DE ARROZ ORGANICA |
| 02501 | AGUARDENTE DE BANANA |
| 04001 | AGUARDENTE DE BANANA ORGANICA |
| 00301 | AGUARDENTE DE CANA |
| 00304 | AGUARDENTE DE CANA DE ACUCAR |
| 03704 | AGUARDENTE DE CANA DE ACUCAR ORGANICA |
| 03701 | AGUARDENTE DE CANA ORGANICA |
| 03201 | AGUARDENTE DE CATUABA |
| 04201 | AGUARDENTE DE CATUABA ORGANICA |
| 04701 | AGUARDENTE DE JALAPA |
| 04501 | AGUARDENTE DE MANDIOCA |
| 00401 | AGUARDENTE DE UVA |
| 03801 | AGUARDENTE DE UVA ORGANICA |
| 06801 | AGUARDENTE ORGANICA |
| 01502 | AGUARRAS |
| 00104 | AGULHA |
| 00803 | AGULHA (ACEM) |
| 00806 | AGULHA COM OSSO |
| 00130 | AGULHA DE CROCHE |
| 00819 | AGULHA DE DESENTUPIR FOGAO |
| 30305 | AGULHA DE INJECAO (DESCARTAVEL) |
| 30302 | AGULHA DE INJECAO (MEDICAO DE GLICOSE) |
| 00131 | AGULHA DE MAQUINA DE COSTURA |
| 00120 | AGULHA DE TRICO |
| 44103 | AIACA |
| 00612 | AIMPIM |
| 00611 | AIPI |
| 00609 | AIPIM |
| 00704 | AIPIM CONGELADO PARA VIAGEM |
| 02601 | AIPO |
| 02602 | AIPO DE AGUA |
| 00907 | AIPO DO PERU (BATATA AIPO) |
| 02603 | AIPO DOS PANTANOS |
| 44109 | AIUCA |
| 06303 | AJAX |
| 00702 | AJINOMOTO |
| 04301 | AJUDA DE CUSTO DE TRANSFERENCIA DE LOCAL DE TRABALHO |
| 01102 | AJUDA DE CUSTO EM TRABALHO |
| 01401 | AJUDANTE DE COZINHA |
| 00809 | ALARME |
| 00807 | ALARME E TRANCA |
| 66105 | ALASTRIM |
| 00708 | ALBERGUES |
| 01003 | ALBUM DE CARTOES |
| 00243 | ALBUM DE FOTOGRAFIA |
| 00242 | ALBUM DE FOTOGRAFIAS (EXCETO DE CERIMONIA FAMILIAR) |
| 01002 | ALBUM DE SELOS |
| 01701 | ALBUM E FIGURINHAS |
| 02701 | ALCA PARA SUTIA |
| 02301 | ALCACHOFRA |
| 02302 | ALCACHOFRA BRAVA |
| 02401 | ALCAPARRA EM CONSERVA |
| 00301 | ALCATRA |
| 00306 | ALCATRA BOVINA |
| 11506 | ALCATRA BOVINA ORGANICA |
| 00308 | ALCATRA COM OSSO |
| 11508 | ALCATRA COM OSSO ORGANICA |
| 11501 | ALCATRA ORGANICA |
| 06501 | ALCATRA SUINA |
| 01707 | ALCATRINHA |
| 01702 | ALCATRINHA COM OSSO (DIANTEIRO DE SEGUNDA) |
| 00601 | ALCOOL (COMBUSTIVEL DE VEICULO) |
| 00701 | ALCOOL (COMBUSTIVEL DOMESTICO) |
| 00702 | ALCOOL (LITRO) (COMBUSTIVEL DOMESTICO) |
| 30210 | ALCOOL (PARA DESINFECCAO) |
| 01705 | ALCOOL DE CEREAIS |
| 01702 | ALCOOL DE LIMPEZA |
| 01703 | ALCOOL EM GEL |
| 01704 | ALCOOL GEL |
| 01706 | ALCOOL HIDRATADO |
| 30211 | ALCOOL IODADO |
| 01701 | ALCOOL PARA LIMPEZA |
| 07402 | ALECRIM |
| 07401 | ALECRIN |
| 03314 | ALETRIA COM OVOS |
| 03214 | ALETRIA SEM OVOS |
| 02308 | ALEVANTE (TEMPERO INDUSTRIALIZADO) |
| 00101 | ALFACE |
| 00104 | ALFACE AMARGA |
| 07804 | ALFACE AMARGA ORGANICA |
| 00109 | ALFACE AMERICANA |
| 07809 | ALFACE AMERICANA ORGANICA |
| 00112 | ALFACE BRANCA |
| 00105 | ALFACE CRESPA |
| 07805 | ALFACE CRESPA ORGANICA |
| 00110 | ALFACE HIDROPONICA |
| 07810 | ALFACE HIDROPONICA ORGANICA |
| 00108 | ALFACE LISA |
| 07812 | ALFACE LISA HIDRORGANICA |
| 07808 | ALFACE LISA ORGANICA |
| 00107 | ALFACE MANTEIGA |
| 07807 | ALFACE MANTEIGA ORGANICA |
| 00113 | ALFACE MIMOSA |
| 07801 | ALFACE ORGANICA |
| 00102 | ALFACE PAULISTA |
| 07802 | ALFACE PAULISTA ORGANICA |
| 00103 | ALFACE REPOLHUDA |
| 07803 | ALFACE REPOLHUDA ORGANICA |
| 00106 | ALFACE ROMANA |
| 07806 | ALFACE ROMANA ORGANICA |
| 00111 | ALFACE ROXA |
| 07811 | ALFACE ROXA ORGANICA |
| 01306 | ALFAFA PARA ANIMAL NAO-ESPECIFICADO |
| 03501 | ALFAIATE |
| 04809 | ALFAJORES (BISCOITO) |
| 08409 | ALFAJORES (BISCOITO) DIET |
| 08422 | ALFAJORES (BISCOITO) DIETETICO |
| 08309 | ALFAJORES (BISCOITO) LIGHT |
| 09201 | ALFAVACA (MOLHO) |
| 02101 | ALFAVACA (TEMPERO INDUSTRIALIZADO) |
| 02106 | ALFAVACA CHEIROSA (TEMPERO INDUSTRIALIZADO) |
| 02104 | ALFAVACA DE CHEIRO (TEMPERO INDUSTRIALIZADO) |
| 07501 | ALFAZEMA |
| 00402 | ALFENIM DE CANA |
| 00103 | ALFINETE |
| 04402 | ALGA EM CONSERVA |
| 04401 | ALGAS EM CONSERVA |
| 02605 | ALGEMA EROTICA |
| 30104 | ALGODAO |
| 07001 | ALGODAO DE LIMPEZA |
| 04316 | ALGODAO DOCE |
| 05401 | ALGODAO-DOCE |
| 06201 | ALHO |
| 06205 | ALHO A GRANEL |
| 06204 | ALHO ARGENTINO |
| 06203 | ALHO BRANCO |
| 06206 | ALHO CABECA |
| 06208 | ALHO DESCASCADO |
| 02702 | ALHO DESIDRATADO |
| 06207 | ALHO EM CABECA |
| 00407 | ALHO EM PASTA |
| 02701 | ALHO EM PO |
| 06302 | ALHO MACHO |
| 06301 | ALHO PORRO |
| 06202 | ALHO ROXO |
| 00404 | ALHOSAL |
| 00105 | ALIANCA (JOIA) |
| 01652 | ALICATE |
| 01602 | ALICATE E CHAVE (BOCA, ESTRIA, ETC.) |
| 00805 | ALICATE E CORTADOR DE UNHA |
| 00801 | ALICATE, CORTADOR DE UNHA, ETC. |
| 05301 | ALICHE |
| 00905 | ALICURI |
| 00601 | ALIMENTACAO |
| 02601 | ALIMENTACAO ESCOLAR |
| 02602 | ALIMENTACAO NA ESCOLA |
| 03301 | ALIMENTACAO SUBSIDIADA |
| 00401 | ALIMENTO DE BOVINO |
| 00101 | ALIMENTO DE CAO |
| 00601 | ALIMENTO DE CAPRINO |
| 01001 | ALIMENTO DE COELHO |
| 00501 | ALIMENTO DE EQUINO |
| 00706 | ALIMENTO DE FRANGO |
| 00701 | ALIMENTO DE GALINHA |
| 00201 | ALIMENTO DE GATO |
| 01201 | ALIMENTO DE OUTRO ANIMAL |
| 00801 | ALIMENTO DE OVINO |
| 00901 | ALIMENTO DE PASSARINHO |
| 00301 | ALIMENTO DE PEIXE |
| 03606 | ALIMENTO DE SOJA |
| 01203 | ALIMENTO INFANTIL CREMOSO (EXCETO SOBREMESA) |
| 01204 | ALIMENTO INFANTIL EM PASTA (EXCETO SOBREMESA) |
| 05801 | ALIMENTO INFANTIL NAO-ESPECIFICADO |
| 01301 | ALIMENTO PARA ANIMAL NAO-ESPECIFICADO |
| 00403 | ALIMENTO PARA BOVINO |
| 00103 | ALIMENTO PARA CAO |
| 00603 | ALIMENTO PARA CAPRINO |
| 01003 | ALIMENTO PARA COELHO |
| 00503 | ALIMENTO PARA EQUINO |
| 00707 | ALIMENTO PARA FRANGO |
| 00702 | ALIMENTO PARA GALINHA |
| 00203 | ALIMENTO PARA GATO |
| 01203 | ALIMENTO PARA OUTRO ANIMAL |
| 00803 | ALIMENTO PARA OVINO |
| 00903 | ALIMENTO PARA PASSARINHO |
| 00303 | ALIMENTO PARA PEIXE |
| 00701 | ALIMENTO PRONTO CONGELADO PARA VIAGEM |
| 02103 | ALIMENTOS INFANTIS QUATRO CEREAIS |
| 00506 | ALINHAMENTO DE RODAS |
| 00206 | ALISAMENTO (CABELEIREIRO) |
| 02604 | ALISANTE DE CABELO |
| 02801 | ALISTAMENTO MILITAR (MULTA) |
| 00407 | ALIZAR |
| 01601 | ALMEIRAO |
| 09101 | ALMEIRAO ORGANICO |
| 01605 | ALMEIRAO ROXO |
| 03502 | ALMOCO A LA CARTE |
| 03504 | ALMOCO A LA CARTE E BEBIDA |
| 03506 | ALMOCO A LA CARTE E SOBREMESA |
| 03508 | ALMOCO A LA CARTE, BEBIDA E SOBREMESA |
| 00102 | ALMOCO A PESO |
| 00104 | ALMOCO A PESO E BEBIDA |
| 00106 | ALMOCO A PESO E SOBREMESA |
| 00108 | ALMOCO A PESO, BEBIDA E SOBREMESA |
| 05701 | ALMOCO DA ESCOLA |
| 00106 | ALMOCO EM QUENTINHA PARA VIAGEM |
| 03602 | ALMOCO FAST FOOD |
| 03604 | ALMOCO FAST FOOD E BEBIDA |
| 03606 | ALMOCO FAST FOOD E SOBREMESA |
| 03608 | ALMOCO FAST FOOD, BEBIDA E SOBREMESA |
| 03805 | ALMOCO OU JANTAR (BANDEJAO) |
| 03804 | ALMOCO OU JANTAR (MARMITA) |
| 03801 | ALMOCO OU JANTAR (OUTROS TIPOS) |
| 03802 | ALMOCO OU JANTAR (PRATO FEITO) |
| 03803 | ALMOCO OU JANTAR (QUENTINHA) |
| 05401 | ALMOCO OU JANTAR (RODIZIO DE CARNES) |
| 05601 | ALMOCO OU JANTAR (RODIZIO DE FRUTOS DO MAR ) |
| 05501 | ALMOCO OU JANTAR (RODIZIO DE MASSAS) |
| 03501 | ALMOCO OU JANTAR A LA CARTE |
| 00101 | ALMOCO OU JANTAR A PESO |
| 03601 | ALMOCO OU JANTAR FAST FOOD |
| 04001 | ALMOCO OU JANTAR NAO-ESPECIFICADO |
| 00105 | ALMOCO PARA VIAGEM |
| 00601 | ALMOFADA |
| 00604 | ALMOFADA (PROTETOR DE BERCO) |
| 21201 | ALMOFADA TERMICA VIBRATORIA |
| 00605 | ALMOFADAO (PROTETOR DE BERCO) |
| 02604 | ALMONDEGA AO MOLHO EM CONSERVA |
| 04501 | ALMONDEGA CONGELADA DE QUALQUER ANIMAL (CRUA) |
| 04503 | ALMONDEGA DE FRANGO CONGELADA |
| 04502 | ALMONDEGA DE QUALQUER ANIMAL CONGELADA |
| 02602 | ALMONDEGA EM CONSERVA |
| 01402 | ALOA |
| 13512 | ALOA LIGHT |
| 08402 | ALOA LIGHT |
| 00701 | ALOJAMENTO |
| 04201 | ALPARGATA (NAO-ESPECIFICADA) |
| 02801 | ALPARGATA DE CRIANCA |
| 02601 | ALPARGATA DE HOMEM |
| 02701 | ALPARGATA DE MULHER |
| 00906 | ALPISTE |
| 07705 | ALTO-FALANTE |
| 07701 | ALTO-FALANTE, TWEETER, MEGAFONE, MICROFONE, ETC. |
| 00802 | ALUGUEL |
| 04202 | ALUGUEL DE APARELHO MEDICO |
| 01605 | ALUGUEL DE BICICLETA (PASSEIO) |
| 02901 | ALUGUEL DE BOLSA, CALCADO, CINTO, CHAPEU, LUVA, ETC. |
| 01607 | ALUGUEL DE BOTE (PASSEIO) |
| 03901 | ALUGUEL DE BRINQUEDO ELETRICO OU ELETRONICO |
| 05401 | ALUGUEL DE BRINQUEDO NAO-ELETRICO OU NAO-ELETRONICO |
| 00102 | ALUGUEL DE BUFFET DE CASAMENTO |
| 00604 | ALUGUEL DE BUFFET PARA FESTA DE ANIVERSARIO |
| 01903 | ALUGUEL DE CACAMBA PARA RETIRAR ENTULHOS |
| 01903 | ALUGUEL DE CACAMBA PARA RETIRAR ENTULHOS |
| 05801 | ALUGUEL DE CADEIRA DE PRAIA |
| 04204 | ALUGUEL DE CADEIRA DE RODAS |
| 04205 | ALUGUEL DE CAMA HOSPITALAR |
| 01703 | ALUGUEL DE CAMPO |
| 01901 | ALUGUEL DE CARRETO PARA MATERIAL DE CONSTRUCAO |
| 01901 | ALUGUEL DE CARRETO PARA MATERIAL DE CONSTRUCAO |
| 01604 | ALUGUEL DE CAVALO (PASSEIO) |
| 01603 | ALUGUEL DE CHARRETE (PASSEIO) |
| 02801 | ALUGUEL DE CLUBE, TEATRO, ETC. PARA DIVERSAO |
| 02601 | ALUGUEL DE DVD (DISCO) |
| 04203 | ALUGUEL DE EQUIPAMENTO DE MASSAGEM |
| 00901 | ALUGUEL DE FITA DE VIDEOCASSETE |
| 04201 | ALUGUEL DE FITA DE VIDEOGAME |
| 00301 | ALUGUEL DE GARAGEM |
| 00601 | ALUGUEL DE GARAGEM DE OUTROS IMOVEIS |
| 02501 | ALUGUEL DE IMOVEIS POR TEMPORADA |
| 04501 | ALUGUEL DE LIVRO DE PRIMEIRO GRAU |
| 07101 | ALUGUEL DE LIXADEIRA ELETRICA |
| 07801 | ALUGUEL DE LIXADEIRA ELETRICA |
| 01907 | ALUGUEL DE MAQUINAS |
| 06501 | ALUGUEL DE MAQUINAS |
| 01906 | ALUGUEL DE MATERIAL DE CONSTRUCAO |
| 01906 | ALUGUEL DE MATERIAL DE CONSTRUCAO |
| 00603 | ALUGUEL DE MESA PARA FESTA DE ANIVERSARIO |
| 00108 | ALUGUEL DE MESA PARA FESTA DE CASAMENTO |
| 02501 | ALUGUEL DE OUTROS IMOVEIS |
| 01702 | ALUGUEL DE QUADRA |
| 01701 | ALUGUEL DE QUADRA E CAMPO |
| 01801 | ALUGUEL DE ROUPA |
| 01901 | ALUGUEL DE ROUPA |
| 02701 | ALUGUEL DE ROUPA |
| 00106 | ALUGUEL DE SALAO PARA CASAMENTO |
| 00602 | ALUGUEL DE SALAO PARA FESTA DE ANIVERSARIO |
| 05801 | ALUGUEL DE TERRENO PARA SEPULTURA |
| 00801 | ALUGUEL DE VEICULO |
| 01001 | ALUGUEL DE VEICULO SEM CONDUTOR |
| 00101 | ALUGUEL DO IMOVEL |
| 04201 | ALUGUEL E REPARO DE APARELHO MEDICO |
| 09001 | ALUGUEL ESTIMADO |
| 00901 | ALUGUEL, DIREITOS AUTORAIS, PATENTES DE BENS MOVEIS |
| 00801 | ALUGUEL, USO OU EXPLORACAO DE BENS IMOVEIS |
| 00902 | ALUGUEL, USO OU EXPLORACAO DE BENS MOVEIS |
| 00903 | ALUGUEL/USO/EXPLORACAO DE BENS MOVEIS |
| 02401 | ALVEJANTE |
| 02411 | AMACIANTE |
| 05901 | AMACIANTE DE CARNE |
| 02405 | AMACIANTE DE ROUPA |
| 02412 | AMACIANTE PARA ROUPA |
| 04817 | AMANDITA (BISCOITO RECHEADO) |
| 02511 | AMARANTO |
| 04203 | AMBROSIA |
| 13803 | AMBROSIA DIET |
| 13808 | AMBROSIA DIETETICA |
| 07102 | AMBU |
| 00702 | AMBULANCIA (REMOCAO) |
| 00701 | AMBULANCIA E UTI MOVEL (REMOCAO) |
| 04301 | AMEIXA |
| 05804 | AMEIXA AMARELA (NESPERA) |
| 02412 | AMEIXA DESIDRATADA |
| 05802 | AMEIXA DO CANADA (NESPERA) |
| 05803 | AMEIXA DO JAPAO (NESPERA) |
| 01303 | AMEIXA EM CALDA |
| 09207 | AMEIXA EM CALDA DIET |
| 09208 | AMEIXA EM CALDA DIETETICA |
| 04305 | AMEIXA FRESCA |
| 04304 | AMEIXA IMPORTADA |
| 05805 | AMEIXA JAPONESA (NESPERA) |
| 04303 | AMEIXA PRETA |
| 02403 | AMEIXA SECA OU DESIDRATADA |
| 04302 | AMEIXA VERMELHA |
| 00501 | AMENDOA |
| 00703 | AMENDOA DA AMERICA (CASTANHA DO PARA) |
| 01008 | AMENDOA DE BABACU |
| 03209 | AMENDOIM ACHOCOLATADO |
| 13109 | AMENDOIM ACHOCOLATADO DIET |
| 13118 | AMENDOIM ACHOCOLATADO DIETETICO |
| 03208 | AMENDOIM AMANTEIGADO |
| 13108 | AMENDOIM AMANTEIGADO DIET |
| 13117 | AMENDOIM AMANTEIGADO DIETETICO |
| 03303 | AMENDOIM APIMENTADO |
| 03207 | AMENDOIM CARAMELIZADO |
| 13107 | AMENDOIM CARAMELIZADO DIET |
| 13116 | AMENDOIM CARAMELIZADO DIETETICO |
| 03304 | AMENDOIM COZIDO |
| 04305 | AMENDOIM DOCE |
| 03305 | AMENDOIM DOCE |
| 01005 | AMENDOIM EM CASCA IN NATURA |
| 01001 | AMENDOIM EM GRAO IN NATURA |
| 03306 | AMENDOIM JAPONES |
| 03702 | AMENDOIM MOIDO |
| 01006 | AMENDOIM NATURAL |
| 03301 | AMENDOIM SALGADO |
| 03302 | AMENDOIM TORRADO |
| 03721 | AMENDOIN SALGADO |
| 00405 | AMERICANO |
| 00308 | AMERICANO PARA VIAGEM |
| 00705 | AMIDO DE ARROZ |
| 00706 | AMIDO DE MANDIOCA |
| 00702 | AMIDO DE MILHO |
| 00704 | AMIDOMIL |
| 02604 | AMIDOMIL (BOLINHO) |
| 03206 | AMINOACIDO |
| 01001 | AMOLAGEM DE FACA, TESOURA, ALICATE, ETC. |
| 30009 | AMONIA |
| 06301 | AMONIACO |
| 09901 | AMORA |
| 03401 | AMPLIFICADOR |
| 02901 | AMPLIFICADOR |
| 02301 | ANAGUA |
| 02001 | ANAGUA |
| 01608 | ANAIA |
| 01606 | ANAJA |
| 00105 | ANALGESICO |
| 00106 | ANALGESICO E ANTIINFLAMATORIO |
| 00102 | ANALGESICO E ANTITERMICO |
| 02602 | ANANAS |
| 03103 | ANCHOVA EM CONSERVA |
| 01613 | ANCINHO |
| 19101 | ANDADOR |
| 02602 | ANDU |
| 00107 | ANEL |
| 00104 | ANEL (JOIA) |
| 04302 | ANEL DE CIMENTO OU CONCRETO |
| 04302 | ANEL DE CIMENTO OU CONCRETO |
| 04402 | ANESTESICO |
| 00502 | ANESTESISTA PARA CIRURGIA E PARTO |
| 03806 | ANGU A BAIANA |
| 07501 | ANGU A BAIANA PARA VIAGEM |
| 00217 | ANGU DE MILHO PARA VIAGEM |
| 04501 | ANGU DE MILHO SEMIPRONTO |
| 00215 | ANGU FRITO PARA VIAGEM |
| 02605 | ANGUSOR DE MILHO |
| 03103 | ANHOLINE SEMIPRONTO |
| 02404 | ANIL |
| 07301 | ANILINA E TINTA DE ASSOALHO |
| 01702 | ANIMAL DE TRACAO |
| 01802 | ANIMAL DOMESTICO |
| 00601 | ANIVERSARIO |
| 02903 | ANIZ CHA |
| 01804 | ANSIOLITICO |
| 21301 | ANTENA DE INTERNET VIA RADIO |
| 02301 | ANTENA DE TELEVISAO NAO-PARABOLICA |
| 02701 | ANTENA PARABOLICA |
| 09401 | ANTENA PARABOLICA E EQUIPAMENTOS |
| 17501 | ANTENA TELEFONICA |
| 01608 | ANTI OSTEOPENIA |
| 02805 | ANTIABORTIVO |
| 00404 | ANTIACIDO |
| 00304 | ANTIALERGICO |
| 00302 | ANTIALERGICO HOMEOPATICO |
| 01402 | ANTIANEMICO |
| 00804 | ANTIBACTERIANO |
| 00803 | ANTIBIOTICO |
| 30706 | ANTI-CASPA |
| 04202 | ANTICOAGULANTE |
| 03103 | ANTICOLICA |
| 02101 | ANTICONCEPCIONAL |
| 02503 | ANTICONVULSIVO |
| 01702 | ANTI-DEPRESSIVO |
| 03002 | ANTIDIARREICO |
| 02502 | ANTIEPILEPTICO |
| 02506 | ANTIEPILETICO |
| 03102 | ANTIESPASMODICO |
| 07101 | ANTIFERRUGEM |
| 02604 | ANTIFUNGICO |
| 00203 | ANTIGRIPAL |
| 00204 | ANTIGRIPAL E ANALGESICO |
| 00205 | ANTIGRIPAL E ANTITERMICO |
| 00209 | ANTIGRIPAL E ANTITUSSIGENO |
| 00206 | ANTIGRIPAL HOMEOPATICO |
| 04106 | ANTI-HANSENIASE |
| 01505 | ANTIHELMINTICO |
| 01502 | ANTI-HELMINTICO (VERMIFUGO) |
| 04204 | ANTI-HEMORRAGICO |
| 00802 | ANTIINFECCIOSO |
| 00805 | ANTIINFECCIOSO E ANTIINFLAMATORIO |
| 00705 | ANTIINFLAMATORIO |
| 00702 | ANTIINFLAMATORIO E ANTI-REUMATICO |
| 02605 | ANTIMICOTICO |
| 06401 | ANTIMOFO |
| 04702 | ANTINEOPLASICO (TRATAMENTO DE CANCER) |
| 01603 | ANTIOSTEOPOROSE |
| 02510 | ANTIPARKSONIANO |
| 02507 | ANTIPSICOTICO |
| 00402 | ANTI-REFLUXIVO GASTRICO |
| 00703 | ANTI-REUMATICO |
| 30202 | ANTI-SEPTICO |
| 01501 | ANTI-SEPTICO BUCAL |
| 30201 | ANTI-SEPTICOS E DESINFETANTES TOPICOS |
| 04003 | ANTITABAGISMO |
| 00103 | ANTITERMICO |
| 03702 | ANTITIREOIDISMO |
| 00202 | ANTITUSSIGENO |
| 00806 | ANTIVIROTICO |
| 02201 | ANUIDADE DE CARTAO DE CREDITO |
| 01401 | ANUNCIO CLASSIFICADO |
| 44122 | ANURI |
| 00508 | ANZOL |
| 07302 | APARA DE CARNE BOVINA |
| 08501 | APARA DE CARNE SUINA |
| 10001 | APARA NAO-ESPECIFICADA |
| 07301 | APARADOR |
| 01801 | APARELHO DE ABDOMINAL |
| 00707 | APARELHO DE BARBA DESCARTAVEL |
| 00706 | APARELHO DE BARBEAR DESCARTAVEL |
| 00711 | APARELHO DE BARBEAR NAO ELETRICO DESCARTAVEL |
| 01801 | APARELHO DE BARBEAR NAO-DESCARTAVEL |
| 13303 | APARELHO DE CAFE |
| 13302 | APARELHO DE CHA |
| 13301 | APARELHO DE CHA OU CAFE |
| 02701 | APARELHO DE CORTAR VERDURA |
| 02801 | APARELHO DE DVD |
| 02702 | APARELHO DE DVD |
| 06701 | APARELHO DE FAX |
| 21101 | APARELHO DE FAZER PIPOCA ELETRICO |
| 01901 | APARELHO DE GINASTICA NAO-ESPECIFICADO |
| 13401 | APARELHO DE JANTAR |
| 30507 | APARELHO DE MEDIDOR DE GLICOSE |
| 02001 | APARELHO DE MUSCULACAO |
| 07901 | APARELHO DE SOLDA |
| 02902 | APARELHO DE SOM |
| 04111 | APARELHO DE SURDEZ |
| 14802 | APARELHO DE TELEFONE RESIDENCIAL |
| 17201 | APARELHO E LINHA TELEFONICA |
| 12002 | APARELHO NAVEGADOR DE INTERNET |
| 02302 | APARELHO ORTODONTICO |
| 17001 | APARELHO PARA FONDUE |
| 16003 | APARELHO PARA MACARRAO |
| 16001 | APARELHO PARA MASSAS |
| 30508 | APARELHO PARA MEDIR PRESSAO |
| 05702 | APARELHO RECEPTOR DE TV A CABO |
| 17101 | APARELHO TELEFONICO COM BINA |
| 14801 | APARELHO TELEFONICO NAO-CELULAR |
| 02502 | APARELHOS DE CURSOS EDUCATIVOS |
| 00201 | APARELHOS SANITARIOS (PIA, VASO, BIDE, BANHEIRA, ETC.) |
| 00201 | APARELHOS SANITARIOS (PIA, VASO, BIDE, BANHEIRA, ETC.) |
| 31301 | APLICACAO DE INJECAO |
| 00511 | APLICACAO DE RAIO LASER |
| 00902 | APLIQUE PARA CABELO |
| 00204 | APONTADOR |
| 00407 | APONTADOR DE LAPIS PARA OS OLHOS |
| 00302 | APOSENTADORIA ( MUNICIPAL, ESTADUAL, FEDERAL) |
| 00301 | APOSENTADORIA ( MUNICIPAL, ESTADUAL, FEDERAL) DA PREVIDENCIA PUBLICA |
| 00102 | APOSENTADORIA (INSS) |
| 00101 | APOSENTADORIA DO INSTITUTO NACIONAL DE SEGURIDADE SOCIAL (INSS) |
| 00502 | APOSENTADORIA, COMPLEMENTACAO DE PREVIDENCIA PRIVADA (ABERTA OU FECHADA) |
| 00501 | APOSENTADORIA, SUPLEM., COMPLEM. DA PREVIDENCIA PRIVADA (ABERTA OU FECHADA) |
| 00804 | APOSTAS EM JOGO DE FUTEBOL |
| 00806 | APOSTILA |
| 00809 | APOSTILA DE SUPLETIVO |
| 03601 | APRESUNTADO |
| 03602 | APRESUNTADO FATIADO |
| 01801 | AQUARIO ORNAMENTAL E EQUIPAMENTOS |
| 04601 | AQUECEDOR CENTRAL DE AGUA ELETRICO |
| 04701 | AQUECEDOR DE AGUA A GAS |
| 10901 | AQUECEDOR DE AGUA ELETRICO |
| 04001 | AQUECEDOR DE AR |
| 15901 | AQUECEDOR DE MAMADEIRA ELETRICO |
| 09702 | AQUECEDOR SOLAR |
| 00202 | AQUISICAO DE AGUA (USO GERAL) (LITROS) |
| 00101 | AQUISICAO DE OUTROS IMOVEIS A VISTA OU A PRAZO (PARCELAS, TAXAS, ETC.) |
| 02601 | AQUISICAO DE OUTROS TERRENOS OU LOTES A VISTA OU A PRAZO (PARCELAS, TAXAS, ETC.) |
| 08501 | AQUISICAO DE SEPULTURA |
| 08502 | AQUISICAO DE TERRENO PARA SEPULTURA |
| 04202 | AQUISICAO DE TITULO DE CAPITALIZACAO |
| 02101 | AQUISICAO DE TITULO DE CLUBE |
| 00201 | AQUISICAO DO IMOVEL A PRAZO (ENTRADA, PARCELAS, TAXAS, IMPOSTOS, ETC.) |
| 00103 | AQUISICAO DO IMOVEL A VISTA (VALOR PAGO) |
| 00101 | AQUISICAO DO IMOVEL A VISTA (VALOR PAGO, IMPOSTO DE TRANSMISSAO, ETC.) |
| 01101 | AR CONDICIONADO |
| 00101 | AR CONDICIONADO (ALUGUEL) |
| 05701 | ARACA |
| 04203 | ARACA ACU (GOIABA) |
| 04204 | ARACA GOIABA (GOIABA) |
| 04202 | ARACA GUAIABA (GOIABA) |
| 01104 | ARAME |
| 01104 | ARAME |
| 00201 | ARARUTA |
| 05101 | ARATICUM |
| 05102 | ARATICUM DE COMER |
| 05103 | ARATICUM MANSO |
| 62103 | ARATU |
| 44108 | ARAU |
| 00101 | ARCA COM OU SEM VITRINE OU ORATORIO |
| 01607 | ARCO DE SERRA |
| 01701 | AR-CONDICIONADO |
| 03901 | AR-CONDICIONADO |
| 03915 | AREIA PARA GATO |
| 02201 | AREIA, TERRA, BARRO, SAIBRO, ETC. |
| 02201 | AREIA, TERRA, BARRO, SAIBRO, ETC. |
| 02202 | ARENOSO |
| 02202 | ARENOSO |
| 02801 | ARGAMASSA |
| 02801 | ARGAMASSA |
| 00902 | ARICUI |
| 00911 | ARICURI |
| 00903 | ARIRI |
| 00405 | ARISCO |
| 00503 | ARMA DE CACA |
| 03901 | ARMACAO DE OCULOS |
| 04001 | ARMARIO (COPA E COZINHA) |
| 06001 | ARMARIO (MOVEL INFANTIL) |
| 06102 | ARMARIO CAMA E MESINHA DE CABECEIRA |
| 09001 | ARMARIO DE BANHEIRO (NAO PLASTICO) |
| 00909 | ARMARIO DE PLASTICO DE BANHEIRO |
| 02004 | ARMARIO DUPLEX |
| 02005 | ARMARIO EMBUTIDO |
| 00703 | ARMARIO EMBUTIDO (SALA) |
| 06002 | ARMARIO INFANTIL |
| 07101 | ARMARIO NAO-ESPECIFICADO |
| 02003 | ARMARIO SIMPLES |
| 02002 | ARMARIO SIMPLES, DUPLEX OU EMBUTIDO |
| 02001 | ARMARIO SIMPLES, DUPLEX OU EMBUTIDO (QUARTO DE ADULTO) |
| 06101 | ARMARIO, CAMA E MESINHA DE CABECEIRA (MOVEIS INFANTIS) |
| 04102 | ARMARIO, MESA E CADEIRAS |
| 04101 | ARMARIO, MESA E CADEIRAS (COPA E COZINHA) |
| 00401 | ARMAS DE FOGO (EXCETO DE CACA) |
| 00805 | ARO DE FAROL |
| 01205 | ARO E RAIO PARA BICICLETA (CROMADO) |
| 01204 | ARO PARA BICICLETA CROMADO |
| 02512 | AROMATIZANTE |
| 14601 | AROMATIZANTE DE BEBIDAS |
| 00516 | ARPAO |
| 03501 | ARQUITETO |
| 05901 | ARQUIVO DE ACO |
| 00234 | ARRANJO DE BOLO |
| 05501 | ARRANJO DE CABELO |
| 00112 | ARRANJO DE FLORES NATURAIS |
| 00710 | ARRAS |
| 00514 | ARRASTAO |
| 03906 | ARRASTO SUINO NAO-ESPECIFICADO |
| 00802 | ARRENDAMENTO DE BENS IMOVEIS |
| 03401 | ARROZ (NAO-ESPECIFICADO) |
| 05401 | ARROZ A GREGA PARA VIAGEM |
| 00111 | ARROZ AGULHA |
| 04311 | ARROZ AGULHA ORGANICO |
| 00112 | ARROZ AGULHINHA |
| 04312 | ARROZ AGULHINHA ORGANICO |
| 00115 | ARROZ AMARELO |
| 04315 | ARROZ AMARELO ORGANICO |
| 00204 | ARROZ BENEFICIADO |
| 04404 | ARROZ BENEFICIADO ORGANICO |
| 00103 | ARROZ BICA CORRIDA |
| 04303 | ARROZ BICA CORRIDA ORGANICO |
| 00113 | ARROZ BRANCO |
| 04313 | ARROZ BRANCO ORGANICO |
| 14201 | ARROZ CARAMELIZADO |
| 00110 | ARROZ COLONIAL |
| 04310 | ARROZ COLONIAL ORGANICO |
| 05003 | ARROZ COLORIDO PARA VIAGEM |
| 09001 | ARROZ COM CARNE SECA PARA VIAGEM |
| 00301 | ARROZ COM CASCA |
| 04501 | ARROZ COM CASCA ORGANICO |
| 05002 | ARROZ COZIDO PARA VIAGEM |
| 07401 | ARROZ DE CUXA PARA VIAGEM |
| 04317 | ARROZ DOCE |
| 14501 | ARROZ DOCE |
| 01801 | ARROZ ESPECIAL JAPONES |
| 00102 | ARROZ HIBRIDO |
| 04302 | ARROZ HIBRIDO ORGANICO |
| 00201 | ARROZ INTEGRAL |
| 04401 | ARROZ INTEGRAL ORGANICO |
| 01802 | ARROZ JAPONES ESPECIAL |
| 00105 | ARROZ LISO |
| 04305 | ARROZ LISO ORGANICO |
| 00106 | ARROZ MACERADO |
| 04306 | ARROZ MACERADO ORGANICO |
| 03402 | ARROZ NAO-ESPECIFICADO |
| 00203 | ARROZ NAO-POLIDO |
| 04403 | ARROZ NAO-POLIDO ORGANICO |
| 01311 | ARROZ PARA ANIMAL NAO ESPECIFICADO |
| 00107 | ARROZ PARBOILIZADO |
| 04307 | ARROZ PARBOILIZADO ORGANICO |
| 00109 | ARROZ PARBORIZADO |
| 04309 | ARROZ PARBORIZADO ORGANICO |
| 00202 | ARROZ PILADO |
| 04402 | ARROZ PILADO ORGANICO |
| 00101 | ARROZ POLIDO |
| 04301 | ARROZ POLIDO ORGANICO |
| 03301 | ARROZ PRE-COZIDO |
| 05001 | ARROZ PRONTO PARA VIAGEM |
| 00104 | ARROZ QUIRERA |
| 04304 | ARROZ QUIRERA ORGANICO |
| 03302 | ARROZ SUICO PRE-COZIDO |
| 00114 | ARROZ VERMELHO |
| 04314 | ARROZ VERMELHO ORGANICO |
| 06001 | ARROZ-DOCE |
| 00104 | ARROZINA |
| 06501 | ARRUDA CHA |
| 06502 | ARRUDA EM FOLHA |
| 00801 | ARRUMADEIRA |
| 05501 | ARRUMADINHO PARA VIAGEM |
| 00802 | ARRUMADOR |
| 02803 | ARTESANATO DECORATIVO |
| 00101 | ARTIGOS DE ARMARINHO |
| 01801 | ARTIGOS DE JARDINAGEM (PLANTA, TERRA, SEMENTE, XAXIM, ETC.) |
| 02001 | ARTIGOS DE LONA OU PLASTICO PARA ACAMPAMENTO |
| 00401 | ARTIGOS DE MAQUIAGEM |
| 02601 | ARTIGOS DE TRATAMENTO DE CABELO |
| 02601 | ARTIGOS EROTICOS |
| 04102 | ARTIGOS ORTOPEDICOS E OUTROS |
| 04101 | ARTIGOS ORTOPEDICOS E PROTESES DIVERSAS |
| 02101 | ARTIGOS PARA PRAIA |
| 00302 | ARTIGOS RELIGIOSOS |
| 03202 | ARVORE DE NATAL (ARTIFICIAL) |
| 00703 | ASA DE FRANGO |
| 00705 | ASA DE FRANGO TEMPERADO |
| 00704 | ASA DE GALINHA |
| 00701 | ASA DE GALINHA OU FRANGO |
| 02101 | ASA DE PERU |
| 00601 | ASA DELTA |
| 02307 | ASFALTO |
| 02307 | ASFALTO |
| 01402 | ASILO (PAGAMENTO) |
| 00708 | ASINHA DE FRANGO |
| 09901 | ASPARGO (IN NATURA) |
| 00701 | ASPARGO EM CONSERVA |
| 01802 | ASPIRADOR DE BOLINHAS |
| 00901 | ASPIRADOR DE PO |
| 01801 | ASPIRADOR DE PO |
| 01803 | ASPIRADOR PAPA BOLINHAS |
| 05001 | ASSADEIRA |
| 00913 | ASSENTO DE VASO SANITARIO |
| 00502 | ASSINATURA DE JORNAL |
| 00601 | ASSINATURA DE PERIODICO TECNICO |
| 00501 | ASSINATURA DE PERIODICOS |
| 00503 | ASSINATURA DE REVISTA |
| 00501 | ASSISTENCIA AUTOMOBILISTICA (MENSALIDADE E TAXA) |
| 00503 | ASSISTENCIA AUTOMOBILISTICA (MENSALIDADE) |
| 00504 | ASSISTENCIA AUTOMOBILISTICA (TAXAS) |
| 02203 | ASSOCIACAO DE AJUDA FUNERARIA |
| 00502 | ASSOCIACAO DE ASSISTENCIA AUTOMOBILISTICA (MENSALIDADE E TAXA) |
| 00102 | ASSOCIACAO DE CLASSE |
| 01801 | ASSOCIACAO DE PAIS E MESTRES |
| 00103 | ASSOCIACAO DE TRABALHADORES |
| 02205 | ASSOCIACAO FUNERARIA |
| 02204 | ASSOCIACAO POSTUMA |
| 00503 | ASSOCIACAO RECREATIVA (MENSALIDADE E TAXA) |
| 02602 | ASSOCIACOES OUTRAS (CONTRIBUICAO) |
| 07502 | ASSUA SUINA |
| 01408 | ATA |
| 03803 | ATA |
| 03201 | ATABAQUE |
| 30105 | ATADURA |
| 05401 | ATEMOIA |
| 00401 | ATENDIMENTO EM PRONTO-SOCORRO/PRONTO-ATENDIMENTO |
| 02207 | ATERRO |
| 02207 | ATERRO |
| 00702 | ATESTADO DE BONS ANTECEDENTES |
| 00105 | ATESTADO DE OBITO |
| 02601 | ATIVIDADES CULTURAIS (INGRESSO) |
| 03301 | ATIVIDADES DESPORTIVAS (INGRESSO) |
| 03402 | ATUM EM CONSERVA |
| 06302 | ATUM EM CONSERVA LIGHT |
| 03403 | ATUM RALADO |
| 06303 | ATUM RALADO LIGHT |
| 03404 | ATUM SOLIDO |
| 06304 | ATUM SOLIDO LIGHT |
| 03603 | AULA DE BALE |
| 06101 | AULA DE BASQUETEBOL |
| 06402 | AULA DE BOXE |
| 05801 | AULA DE CAPOEIRA |
| 03604 | AULA DE DANCA |
| 05901 | AULA DE FUTEBOL |
| 03401 | AULA DE GINASTICA |
| 06301 | AULA DE HANDEBOL |
| 04001 | AULA DE HIDROGINASTICA |
| 03703 | AULA DE IOGA |
| 06403 | AULA DE JUDO |
| 06404 | AULA DE KARATE |
| 06401 | AULA DE LUTAS |
| 03802 | AULA DE MUSICA |
| 04301 | AULA DE NATACAO |
| 08302 | AULA DE PINTURA |
| 06201 | AULA DE TENIS |
| 06001 | AULA DE VOLEIBOL |
| 00301 | AULA PARTICULAR |
| 00109 | AUTENTICACAO DE DOCUMENTO |
| 05401 | AUTO ESCOLA |
| 02101 | AUTOMOVEL |
| 00302 | AUTOMOVEL DE ALUGUEL (TAXI) |
| 01601 | AUTOMOVEL DE PASSEIO IMPORTADO |
| 00101 | AUTOMOVEL DE PASSEIO NACIONAL |
| 01501 | AUTOMOVEL UTILITARIO IMPORTADO |
| 01001 | AUTOMOVEL UTILITARIO NACIONAL |
| 01603 | AUTOMOVEL UTILITARIO TIPO VAN |
| 01404 | AUTORIZACAO DE CACA E PESCA |
| 01405 | AUTORIZACAO PARA CACA E PESCA |
| 02401 | AUXILIO A PORTADORES DE DEFICIENCIA FISICA |
| 03501 | AUXILIO DEFESO |
| 02101 | AUXILIO ENERGIA ELETRICA |
| 02202 | AUXILIO FUNERAL (PAX DOMINNI) |
| 03801 | AUXILIO MORADIA |
| 03701 | AUXILIO PARA PLANO MEDICO |
| 01501 | AUXILIO/TIQUETE/CARTAO ALIMENTACAO |
| 01601 | AUXILIO/VALE/CARTAO TRANSPORTE E COMBUSTIVEL |
| 01701 | AUXILIO-ATIVIDADE |
| 01603 | AUXILIO-COMBUSTIVEL |
| 02001 | AUXILIO-COMUNICACAO |
| 03601 | AUXILIO-CRECHE |
| 02602 | AUXILIO-CRECHE |
| 01703 | AUXILIO-DESEMPREGO |
| 02301 | AUXILIO-DOENCA DA PREVIDENCIA PRIVADA |
| 02501 | AUXILIO-DOENCA DA PREVIDENCIA PUBLICA |
| 02601 | AUXILIO-EDUCACAO |
| 03601 | AUXILIO-EDUCACAO (DESCONTO) |
| 02604 | AUXILIO-ESCOLA |
| 01901 | AUXILIO-ESTIAGEM |
| 01801 | AUXILIO-FARDAMENTO |
| 02901 | AUXILIO-FUNERAL |
| 03101 | AUXILIO-GAS |
| 02901 | AUXILIO-LEITE |
| 02605 | AUXILIO-MAE GUARDIA |
| 01801 | AUXILIO-MATERNIDADE |
| 01802 | AUXILIO-NATALIDADE |
| 03501 | AUXILIO-TRATAMENTO |
| 03101 | AUXILIO-VELHICE |
| 01701 | AVALIADOR DE IMOVEL |
| 00401 | AVEIA EM FLOCOS |
| 00401 | AVEIA EM GRAO |
| 00402 | AVEIA EM MASSA |
| 00403 | AVEIA INSTANTANEA |
| 00601 | AVELA |
| 08904 | AVENTAL DE PLASTICO |
| 01304 | AVENTAL PARA SERVICOS DOMESTICOS |
| 01301 | AVENTAL, LUVA, ETC. PARA SERVICOS DOMESTICOS |
| 02201 | AVIAO |
| 00301 | AVIAO |
| 04001 | AVISO PREVIO |
| 01902 | AVISO PREVIO A EMPREGADOR |
| 50101 | AVIU |
| 50401 | AVIU SALGADO |
| 50201 | AVIU SEM CASCA |
| 11001 | AVOADOR |
| 03708 | AVOANTE ABATIDA, CONGELADA OU VIVA |
| 03723 | AVOANTE CONGELADA |
| 03724 | AVOANTE VIVA |
| 01904 | AZEDA |
| 02901 | AZEDINHA |
| 00105 | AZEITE COMPOSTO DE OLIVA E OLEO DE SOJA |
| 03901 | AZEITE DE COCO BABACU |
| 00201 | AZEITE DE DENDE |
| 04101 | AZEITE DE GIRASSOL |
| 04201 | AZEITE DE MACAUBA |
| 04301 | AZEITE DE MAMONA |
| 00101 | AZEITE DE OLIVA |
| 04001 | AZEITE DE SOJA |
| 00103 | AZEITE DOCE |
| 00107 | AZEITE EXTRA VIRGEM |
| 00102 | AZEITE IMPORTADO DE OLIVA |
| 03302 | AZEITE MISTO DE OLIVA E SOJA |
| 03301 | AZEITE MISTO DE SOJA E OLIVA |
| 00104 | AZEITE TEMPERADO |
| 00106 | AZEITE VERDE |
| 00101 | AZEITONA |
| 00102 | AZEITONA EM CONSERVA |
| 00107 | AZEITONA FATIADA EM CONSERVA |
| 00104 | AZEITONA PRETA |
| 00105 | AZEITONA RECHEADA EN CONSERVA |
| 00106 | AZEITONA SEM CAROCO |
| 00103 | AZEITONA VERDE |
| 07401 | AZEITONA VERDE LIGHT |
| 00105 | AZULEJO |
| 00104 | AZULEJO |
| 00101 | AZULEJO E PISO |
| 00101 | AZULEJO E PISO |
| 00401 | BABA |
| 02702 | BABA DE CACHORRO |
| 03002 | BABA DE SAPO OU DE MOCA |
| 12902 | BABA DE SAPO OU DE MOCA DIET |
| 12904 | BABA DE SAPO OU DE MOCA DIETETICA |
| 07601 | BABA ELETRONICA |
| 01005 | BABACU |
| 01806 | BABADOR |
| 01405 | BABAO |
| 14401 | BABOSA EM FOLHA |
| 19301 | BABY BAG |
| 00706 | BABY DOLL |
| 00806 | BABY DOLL |
| 00305 | BABY LOOK |
| 01902 | BACABA |
| 01909 | BACABA EM EMULSAO |
| 01912 | BACABA EM PAPA |
| 01905 | BACABAI |
| 01903 | BACABINHA |
| 01904 | BACABUCU |
| 70401 | BACALHAU |
| 00827 | BACIA DE ALUMINIO |
| 01209 | BACIA DE BICICLETA |
| 00828 | BACIA DE ESMALTE |
| 00829 | BACIA DE INOX |
| 04801 | BACIA INDETERMINADA |
| 00903 | BACIA PLASTICA |
| 06902 | BACO BOVINO |
| 09201 | BACO NAO-ESPECIFICADO |
| 07002 | BACO SUINO |
| 01005 | BACON |
| 01006 | BACON DEFUMADO |
| 01008 | BACON EM RETALHO |
| 01004 | BACON EMBALADO |
| 01002 | BACON NO VAREJO |
| 02230 | BACONZITOS |
| 10130 | BACONZITOS LIGHT |
| 09401 | BACTERICIDA |
| 66104 | BACUCU |
| 06501 | BACURI |
| 06503 | BACURIUBA |
| 06504 | BACURIUVA |
| 00403 | BAGACEIRA |
| 03803 | BAGACEIRA ORGANICA |
| 01007 | BAGUACU |
| 00203 | BAIANO |
| 03501 | BAIAO DE DOIS PARA VIAGEM |
| 00701 | BAILE |
| 01404 | BAILE DE CARNAVAL |
| 05601 | BAILE E FESTA EM CLUBE (INGRESSO) |
| 00913 | BAIXA DE RESERVA DE REGISTRO DE VEICULO |
| 13501 | BAIXELA |
| 00802 | BALA |
| 00603 | BALA |
| 30804 | BALA DE GENGIBRE |
| 00614 | BALA DE GOMA |
| 09903 | BALA DIET |
| 09916 | BALA DIETETICA |
| 09803 | BALA LIGHT |
| 00801 | BALA OU CHICLETE |
| 07101 | BALA OU CHICLETE DIET |
| 07103 | BALA OU CHICLETE DIETETICO |
| 09401 | BALA OU CHICLETE LIGHT |
| 15802 | BALANCA DE BANHEIRO |
| 16401 | BALANCA PARA ALIMENTOS |
| 15801 | BALANCA PARA PESSOAS |
| 00507 | BALANCEAMENTO DE RODAS |
| 00221 | BALAO |
| 00222 | BALAO DE GAS |
| 00611 | BALAS |
| 09911 | BALAS DIET |
| 09924 | BALAS DIETETICAS |
| 09811 | BALAS LIGHT |
| 00612 | BALAS SORTIDAS |
| 09912 | BALAS SORTIDAS DIET |
| 09925 | BALAS SORTIDAS DIETETICAS |
| 09812 | BALAS SORTIDAS LIGHT |
| 03108 | BALCAO |
| 01801 | BALCAO |
| 03105 | BALCAO DE COZINHA |
| 03105 | BALCAO DE COZINHA |
| 03107 | BALCAO DE GRANITO PARA PIA |
| 03107 | BALCAO DE GRANITO PARA PIA |
| 03103 | BALCAO DE MARMORE PARA PIA |
| 03103 | BALCAO DE MARMORE PARA PIA |
| 03104 | BALCAO DE PIA |
| 03104 | BALCAO DE PIA |
| 04701 | BALCAO PARA PASSAR ROUPA |
| 04901 | BALDE |
| 08201 | BALDE |
| 00817 | BALDE DE ALUMINIO |
| 06502 | BALDE PARA CONSTRUCAO |
| 06902 | BALDE PARA CONSTRUCAO |
| 00902 | BALDE PLASTICO |
| 00203 | BALDINHO DE PRAIA |
| 00613 | BALINHA |
| 09913 | BALINHA DIET |
| 09926 | BALINHA DIETETICA |
| 09813 | BALINHA LIGHT |
| 00510 | BALSA |
| 01203 | BALSA |
| 01402 | BANANA |
| 01101 | BANANA (NAO-ESPECIFICADA) |
| 00502 | BANANA ABOBORA |
| 00503 | BANANA ACU |
| 00113 | BANANA ACUCARINA |
| 00202 | BANANA ALTONA |
| 00203 | BANANA AMARELA |
| 00116 | BANANA ANA |
| 01016 | BANANA ANGOLA |
| 00504 | BANANA BABA DE BOI |
| 00505 | BANANA BABONA |
| 00204 | BANANA BICO VERDE |
| 00205 | BANANA BRANCA |
| 01006 | BANANA BURITI |
| 00506 | BANANA CACAU |
| 00507 | BANANA CAFE |
| 00508 | BANANA CAIXAO |
| 00509 | BANANA CAJU |
| 00702 | BANANA CAMBORI |
| 00114 | BANANA CAMBOTA |
| 00311 | BANANA CANELA |
| 00510 | BANANA CARAMBOLA |
| 00402 | BANANA CARAPE |
| 00511 | BANANA CASADA |
| 00512 | BANANA CASCA GROSSA |
| 00112 | BANANA CASCA VERDE |
| 00109 | BANANA CATARINA |
| 00115 | BANANA CATURRA |
| 01007 | BANANA CHIFRE DE VACA |
| 00513 | BANANA CHOCOLATE |
| 00110 | BANANA CHORONA |
| 00514 | BANANA CINZA |
| 00403 | BANANA COCO |
| 01008 | BANANA COMPRIDA |
| 00515 | BANANA CORUJA |
| 00302 | BANANA CRAVO |
| 00902 | BANANA CURTA |
| 00103 | BANANA DA CHINA |
| 00208 | BANANA DA PRATA |
| 01001 | BANANA DA TERRA |
| 08901 | BANANA DA TERRA FRITA PARA VIAGEM |
| 00117 | BANANA DAGUA |
| 00703 | BANANA DAS ALMAS |
| 00101 | BANANA DE AGUA |
| 00206 | BANANA DE CAMBOEIRO |
| 00104 | BANANA DE ITALIANO |
| 00516 | BANANA DE VELHO |
| 02416 | BANANA DESIDRATADA |
| 00903 | BANANA DO PARAISO |
| 00303 | BANANA DOURADA |
| 01307 | BANANA EM CALDA |
| 09211 | BANANA EM CALDA DIET |
| 09212 | BANANA EM CALDA DIETETICA |
| 01009 | BANANA FARTA GENTE |
| 01010 | BANANA FARTA HOMEM |
| 01011 | BANANA FARTA VELHACO |
| 00501 | BANANA FIGO |
| 04311 | BANANA FRITA COM MEL |
| 01012 | BANANA GRANDE |
| 01013 | BANANA GRANDE AMARELA |
| 00304 | BANANA IMPERIAL |
| 00305 | BANANA INAJA |
| 00106 | BANANA INGLESA |
| 00517 | BANANA JACARE |
| 00518 | BANANA JASMIM |
| 00404 | BANANA LEITE |
| 00401 | BANANA MACA |
| 00405 | BANANA MACA PAINA |
| 00408 | BANANA MACAZINHA DA BAHIA |
| 00105 | BANANA MANGALO |
| 01014 | BANANA MARANHENSE |
| 00602 | BANANA MARECA |
| 00306 | BANANA MARIQUINHA |
| 00601 | BANANA MARMELO |
| 00519 | BANANA MENCI |
| 00520 | BANANA MINEIRA |
| 00704 | BANANA MULATA |
| 00521 | BANANA MURICI |
| 00102 | BANANA NANICA |
| 01102 | BANANA NAO-ESPECIFICADA |
| 09801 | BANANA NAO-ESPECIFICADA ORGANICA |
| 00301 | BANANA OURO |
| 01004 | BANANA PACOBEIRA |
| 01005 | BANANA PACOBUCU |
| 01002 | BANANA PACOVA |
| 01019 | BANANA PACOVAN |
| 01003 | BANANA PACOVEIRA |
| 01015 | BANANA PACOVI |
| 00522 | BANANA PAO |
| 00523 | BANANA PARA |
| 03401 | BANANA PARA VIAGEM |
| 02420 | BANANA PASSA |
| 00705 | BANANA PATROQUIA |
| 00524 | BANANA PAU |
| 00406 | BANANA PEDRA |
| 00407 | BANANA PERA |
| 00111 | BANANA PEROA |
| 00108 | BANANA PETICA |
| 00307 | BANANA PISANGO REAL |
| 00201 | BANANA PRATA |
| 09101 | BANANA PRATA ORGANICA |
| 00802 | BANANA RAJADA |
| 00308 | BANANA REAL |
| 00525 | BANANA RIACHAO |
| 00526 | BANANA ROSA |
| 00701 | BANANA ROXA |
| 00801 | BANANA SAO DOMINGOS |
| 00901 | BANANA SAO TOME |
| 00527 | BANANA SAPA |
| 02407 | BANANA SECA OU DESIDRATADA |
| 00528 | BANANA TAJAMAIA |
| 00529 | BANANA TANJA |
| 00107 | BANANA TATU |
| 00530 | BANANA TRES QUINAS |
| 00207 | BANANA UMBIGO VERDE |
| 00309 | BANANA URINHO |
| 00531 | BANANA VERMELHA |
| 00706 | BANANA VINAGRE |
| 01240 | BANANADA |
| 01210 | BANANADA (EM BARRA OU TIJOLO) |
| 00310 | BANANINHA |
| 04202 | BANCO |
| 04201 | BANCO (COPA E COZINHA) |
| 08001 | BANCO (JARDIM, VARANDA, ETC.) |
| 03501 | BANCO (SALA) |
| 08002 | BANCO DE JARDIM |
| 08003 | BANCO DE VARANDA |
| 00902 | BANDAGEM (ESTETICISTA) |
| 06101 | BANDEIRA DE JANELA |
| 06101 | BANDEIRA DE JANELA |
| 00225 | BANDEIRINHAS DE FESTA |
| 05201 | BANDEJA |
| 00831 | BANDEJA DE INOX |
| 03802 | BANDEJA DE PORCELANA (PORTA-BOLO) |
| 04501 | BANDO |
| 03001 | BANHA BOVINA |
| 01901 | BANHA DE GALINHA |
| 01802 | BANHA DE PORCO |
| 03101 | BANHA NAO-ESPECIFICADA |
| 01801 | BANHA SUINA |
| 02001 | BANHA VEGETAL |
| 00205 | BANHEIRA |
| 00205 | BANHEIRA |
| 07401 | BANHEIRA COM TROCA-FRALDAS |
| 00910 | BANHEIRA PLASTICA DE CRIANCA OU BEBE |
| 02501 | BANHEIRO PUBLICO |
| 02504 | BANHO DE ANIMAL DOMESTICO |
| 00208 | BANHO DE BRILHO (CABELEIREIRO) |
| 00803 | BANHO DE LUA (DESCOLORACAO DE PELOS) |
| 02505 | BANHO E TOSA DE ANIMAL DOMESTICO |
| 04102 | BANHO TERMAL |
| 03101 | BANJO |
| 01703 | BAR |
| 02007 | BAR (COUVERT ARTISTICO) |
| 01701 | BAR (SALA) |
| 00210 | BARALHO |
| 04201 | BARBA |
| 00124 | BARBANTE |
| 01301 | BARBEADOR DE LAMINA |
| 00709 | BARBEADOR DESCARTAVEL |
| 05201 | BARBEADOR ELETRICO |
| 00101 | BARBEIRO |
| 00501 | BARCA |
| 00502 | BARCO |
| 01201 | BARCO |
| 00201 | BARCO |
| 00202 | BARCO A MOTOR |
| 02001 | BARCO A REMO |
| 02301 | BARCO A VELA |
| 01502 | BARDANA |
| 00604 | BARES |
| 07201 | BARRA DE CEREAIS |
| 07401 | BARRA DE CEREAIS DIET |
| 12501 | BARRA DE CEREAIS DIET |
| 07402 | BARRA DE CEREAIS DIETETICA |
| 12503 | BARRA DE CEREAIS DIETETICA |
| 07203 | BARRA DE CEREAIS DOCE |
| 12502 | BARRA DE CEREAIS DOCE DIET |
| 12504 | BARRA DE CEREAIS DOCE DIETETICA |
| 14101 | BARRA DE CEREAIS LIGHT |
| 07202 | BARRA DE CEREAIS SALGADA |
| 07204 | BARRA DE CEREAL |
| 04319 | BARRA DE CEREAL DOCE |
| 03746 | BARRA DE CEREAL SALGADA |
| 00702 | BARRA DE CHOCOLATE |
| 07203 | BARRA DE CHOCOLATE DIET |
| 10102 | BARRA DE CHOCOLATE DIET |
| 07204 | BARRA DE CHOCOLATE DIETETICO |
| 10108 | BARRA DE CHOCOLATE DIETETICO |
| 09502 | BARRA DE CHOCOLATE LIGHT |
| 10002 | BARRA DE CHOCOLATE LIGHT |
| 02201 | BARRACA DE ACAMPAMENTO |
| 00234 | BARRACA DE BRINQUEDO |
| 02102 | BARRACA DE PRAIA |
| 02203 | BARRO |
| 02203 | BARRO |
| 01702 | BARZINHO (SALA) |
| 02601 | BASCULANTE |
| 02601 | BASCULANTE |
| 00602 | BASE |
| 00923 | BASE DE LIQUIDIFICADOR |
| 01503 | BASE PARA MESA (SALA) (QUALQUER MATERIAL) |
| 02812 | BASE PARA SORVETE |
| 00402 | BASQUETEBOL (INGRESSO) |
| 02801 | BASTIDOR |
| 00304 | BATA |
| 00802 | BATATA (NAO-ESPECIFICADA) |
| 03103 | BATATA A VACUO PARA VIAGEM |
| 00901 | BATATA AIPO |
| 00301 | BATATA BAROA |
| 00302 | BATATA BARONESA |
| 00109 | BATATA BINGE |
| 01809 | BATATA BINGE ORGANICA |
| 00110 | BATATA BRANCA |
| 00905 | BATATA CENOURA |
| 03104 | BATATA CONGELADA PARA FRITAR |
| 03101 | BATATA CONGELADA PARA VIAGEM |
| 03102 | BATATA COZIDA PARA VIAGEM |
| 00403 | BATATA DA TERRA |
| 00404 | BATATA DE ANGOLA |
| 00717 | BATATA DE RAMA |
| 00716 | BATATA DO AR |
| 00903 | BATATA DO BARAO |
| 00102 | BATATA DO REINO |
| 01802 | BATATA DO REINO ORGANICA |
| 03735 | BATATA DOCE |
| 00401 | BATATA DOCE |
| 00402 | BATATA DOCE ROXA |
| 00906 | BATATA FIUZA |
| 03715 | BATATA FRITA |
| 04106 | BATATA FRITA E REFRIGERANTE |
| 01501 | BATATA FRITA PARA VIAGEM |
| 00108 | BATATA HOLANDESA |
| 01808 | BATATA HOLANDESA ORGANICA |
| 00105 | BATATA INGESA ROSA |
| 01805 | BATATA INGESA ROSA ORGANICA |
| 00101 | BATATA INGLESA |
| 01801 | BATATA INGLESA ORGANICA |
| 00107 | BATATA LISA |
| 01807 | BATATA LISA ORGANICA |
| 00405 | BATATA MASTRUS |
| 00711 | BATATA NAMBU |
| 00801 | BATATA NAO-ESPECIFICADA |
| 09601 | BATATA PALHA LIGTH PARA VIAGEM |
| 01503 | BATATA PALHA PARA VIAGEM |
| 01502 | BATATA PALITO PARA VIAGEM |
| 00104 | BATATA PORTUGUESA |
| 01804 | BATATA PORTUGUESA ORGANICA |
| 00103 | BATATA ROSA |
| 01803 | BATATA ROSA ORGANICA |
| 00406 | BATATA ROXA |
| 00902 | BATATA SALSA |
| 00904 | BATATA SUICA |
| 00106 | BATATINHA |
| 01806 | BATATINHA ORGANICA |
| 03001 | BATEDEIRA DE BOLO |
| 00901 | BATEDEIRA DE BOLO |
| 05401 | BATEDOR DE OVOS MANUAL |
| 00701 | BATERIA |
| 01901 | BATERIA (ALUGUEL) |
| 01104 | BATERIA APARELHO AUDITIVO |
| 00702 | BATERIA DE AUTOMOVEL |
| 01103 | BATERIA DE BRINQUEDO |
| 13601 | BATERIA DE COZINHA |
| 18401 | BATERIA DE FILMADORA |
| 00703 | BATERIA DE MOTOCICLETA |
| 01102 | BATERIA DE RELOGIO |
| 01101 | BATERIA DE RELOGIO, BRINQUEDO, APARELHO AUDITIVO, ETC. |
| 01302 | BATERIA DE TELEFONE CELULAR |
| 01001 | BATERIA MUSICAL |
| 00403 | BATIDA (RAPADURA) |
| 00503 | BATIDA DE COCO |
| 00501 | BATIDA DE QUALQUER SABOR |
| 01201 | BATISTERIO |
| 00202 | BATIZADO |
| 00408 | BATOM |
| 00708 | BATON CHOCOLATE |
| 03201 | BAU (QUARTO ADULTO) |
| 01601 | BAU (SALA) |
| 61120 | BAU (SIRI) |
| 01006 | BAUACU |
| 00901 | BAUNILHA EM PO |
| 00404 | BAURU |
| 00307 | BAURU PARA VIAGEM |
| 19201 | BEBE-CONFORTO |
| 09803 | BEBEDOURO |
| 03110 | BEBIDA ACHOCOLATADA |
| 06810 | BEBIDA ACHOCOLATADA DIET |
| 06820 | BEBIDA ACHOCOLATADA DIETETICA |
| 06710 | BEBIDA ACHOCOLATADA LIGHT |
| 03501 | BEBIDA ALCOOLICA NAO-ESPECIFICADA |
| 02307 | BEBIDA DE SOJA |
| 03101 | BEBIDA ENERGETICA |
| 05901 | BEBIDA ENERGETICA |
| 02401 | BEBIDA ISOTONICA |
| 05805 | BEBIDA ISOTONICA |
| 01303 | BEBIDA LACTEA |
| 01306 | BEBIDA LACTEA COM FRUTA |
| 01307 | BEBIDA LACTEA DE FRUTA |
| 01305 | BEBIDA LACTEA DE QUALQUER SABOR |
| 08601 | BEBIDA LACTEA LIGHT |
| 04104 | BEBIDA MISTA DE MACA |
| 00506 | BEBIDA MISTA DE UVA |
| 06001 | BEBIDA NAO-ALCOOLICA NAO-ESPECIFICADA |
| 00602 | BEBIDAS |
| 02803 | BECA |
| 04702 | BEIJO AFRICANO |
| 04701 | BEIJO DE MOCA |
| 04502 | BEIJU |
| 04509 | BEIJU DE COCO |
| 04505 | BEIJU DE QUEIJO E PRESUNTO |
| 04503 | BEIJU DE TAPIOCA |
| 02202 | BELICHE |
| 04818 | BEM CASADO (BISCOITO RECHEADO) |
| 01101 | BENEFICIO DE PRESTACAO CONTINUADA (BPC, LOAS) |
| 06201 | BENGALA |
| 00114 | BENGALA |
| 01503 | BENZINA |
| 05001 | BERBIGAO EM CONSERVA |
| 01102 | BERCARIO |
| 06301 | BERCO |
| 02217 | BERGAMOTA |
| 07201 | BERIBA |
| 01301 | BERIMBAU |
| 05401 | BERINJELA |
| 00804 | BERINJELA A MILANESA PARA VIAGEM |
| 06601 | BERINJELA AGRIDOCE |
| 00204 | BERMUDA |
| 00203 | BERMUDA |
| 00203 | BERMUDA |
| 00201 | BERMUDA E SHORT |
| 00201 | BERMUDA, CALCAO E SHORT |
| 00201 | BERMUDA, SHORT E CALCAO |
| 03301 | BERRANTE |
| 00201 | BERTALHA |
| 01101 | BETERRABA |
| 01505 | BETERRABA BRANCA (ACELGA) |
| 01506 | BETERRABA CAMPESTRE (ACELGA) |
| 06701 | BETERRABA EM CONSERVA |
| 02001 | BETERRABA ORGANICA |
| 00219 | BEXIGA |
| 02801 | BIBELO DE QUALQUER MATERIAL |
| 00405 | BIBLIA |
| 02701 | BIBLIOTECA (MENSALIDADE, MULTA, ETC.) |
| 02204 | BICAMA |
| 02201 | BICAMA, BELICHE OU TRILICHE |
| 00502 | BICARBONATO |
| 00501 | BICARBONATO DE SODIO |
| 00228 | BICHO DE PELUCIA |
| 02201 | BICICLETA |
| 01201 | BICICLETA |
| 00301 | BICICLETA |
| 00901 | BICICLETA DE GINASTICA |
| 00903 | BICICLETA ERGOMETRICA |
| 30403 | BICO |
| 02901 | BICO PARA VESTIDO |
| 00202 | BIDE |
| 00202 | BIDE |
| 01202 | BIDU |
| 13508 | BIDU LIGHT |
| 08202 | BIDU LIGHT |
| 04702 | BIFE A MILANEZA INDUSTRIALIZADO SEMIPRONTO |
| 01108 | BIFE A MILANEZA PARA VIAGEM |
| 01105 | BIFE PREPARADO PARA VIAGEM |
| 07103 | BIFE ROLE CRU |
| 03601 | BIFE VEGETAL |
| 00101 | BIJUTERIA |
| 00203 | BILHETE DE LOTERIA ESTADUAL |
| 00202 | BILHETE DE LOTERIA FEDERAL |
| 16902 | BINA |
| 16901 | BINA (IDENTIFICADOR DE TELEFONE) |
| 01001 | BINGO |
| 03501 | BINGO |
| 12505 | BINOCULO |
| 12501 | BINOCULO, LUNETA, TELESCOPIO E LUPA |
| 03907 | BIO FIBRAS LIQUIDO LIGHT |
| 05801 | BIOMBO |
| 01307 | BIOPSIA (EM GERAL) |
| 00904 | BIP (APARELHO) |
| 01204 | BIP (MENSALIDADE, ASSINATURA, ALUGUEL) |
| 01102 | BIQUINI |
| 00504 | BIQUINI |
| 07202 | BIRIBA |
| 02302 | BISCATEIRO |
| 02301 | BISCATEIRO (SERVICOS DIVERSOS) |
| 02241 | BISCOITO AGUA E SAL |
| 02308 | BISCOITO AMANTEIGADO |
| 10508 | BISCOITO AMANTEIGADO DIET |
| 10548 | BISCOITO AMANTEIGADO DIETETICO |
| 10408 | BISCOITO AMANTEIGADO LIGHT |
| 02216 | BISCOITO BIT |
| 10116 | BISCOITO BIT LIGHT |
| 02331 | BISCOITO BROA DE COCO |
| 10531 | BISCOITO BROA DE COCO DIET |
| 10571 | BISCOITO BROA DE COCO DIETETICO |
| 10431 | BISCOITO BROA DE COCO LIGHT |
| 00105 | BISCOITO CANINO |
| 04901 | BISCOITO CASEIRO |
| 10536 | BISCOITO CHAMPAGNE DIET |
| 10436 | BISCOITO CHAMPAGNE LIGHT |
| 02328 | BISCOITO CHAMPANHE |
| 10528 | BISCOITO CHAMPANHE DIET |
| 10568 | BISCOITO CHAMPANHE DIETETICO |
| 10428 | BISCOITO CHAMPANHE LIGHT |
| 02220 | BISCOITO CLUB CRACKER |
| 10120 | BISCOITO CLUB CRACKER LIGHT |
| 02242 | BISCOITO CLUB SOCIAL |
| 02243 | BISCOITO CLUBE SOCIAL |
| 02204 | BISCOITO CREAM CRACKER |
| 02232 | BISCOITO CREAM CRACKER INTEGRAL |
| 10104 | BISCOITO CREAM CRACKER LIGHT |
| 02326 | BISCOITO CREME |
| 10526 | BISCOITO CREME DIET |
| 10566 | BISCOITO CREME DIETETICO |
| 10426 | BISCOITO CREME LIGHT |
| 04811 | BISCOITO CROCANTE |
| 08411 | BISCOITO CROCANTE DIET |
| 08424 | BISCOITO CROCANTE DIETETICO |
| 08311 | BISCOITO CROCANTE LIGHT |
| 02207 | BISCOITO DE AGUA |
| 02231 | BISCOITO DE AGUA E SAL |
| 02222 | BISCOITO DE AGUA INTEGRAL |
| 10122 | BISCOITO DE AGUA INTEGRAL LIGHT |
| 10107 | BISCOITO DE AGUA LIGHT |
| 02404 | BISCOITO DE ARARUTA |
| 02210 | BISCOITO DE CAMARAO |
| 10110 | BISCOITO DE CAMARAO LIGHT |
| 02239 | BISCOITO DE CEBOLA |
| 02336 | BISCOITO DE CHAMPAGNE |
| 10576 | BISCOITO DE CHAMPAGNE DIETETICO |
| 02341 | BISCOITO DE CHOCOLATE |
| 02339 | BISCOITO DE COCO |
| 10539 | BISCOITO DE COCO DIET |
| 10579 | BISCOITO DE COCO DIETETICO |
| 02325 | BISCOITO DE COCO DOCE |
| 10525 | BISCOITO DE COCO DOCE DIET |
| 10565 | BISCOITO DE COCO DOCE DIETETICO |
| 10425 | BISCOITO DE COCO DOCE LIGHT |
| 10439 | BISCOITO DE COCO LIGHT |
| 02209 | BISCOITO DE COCO SALGADO |
| 10109 | BISCOITO DE COCO SALGADO LIGHT |
| 02322 | BISCOITO DE FUBA |
| 10522 | BISCOITO DE FUBA DIET |
| 10562 | BISCOITO DE FUBA DIETETICO |
| 10422 | BISCOITO DE FUBA LIGHT |
| 02307 | BISCOITO DE GERGELIM |
| 10507 | BISCOITO DE GERGELIM DIET |
| 10547 | BISCOITO DE GERGELIM DIETETICO |
| 10407 | BISCOITO DE GERGELIM LIGHT |
| 02355 | BISCOITO DE GOMA |
| 02338 | BISCOITO DE LEITE |
| 10538 | BISCOITO DE LEITE DIET |
| 10578 | BISCOITO DE LEITE DIETETICO |
| 10438 | BISCOITO DE LEITE LIGHT |
| 02316 | BISCOITO DE MAIZENA |
| 10516 | BISCOITO DE MAIZENA DIET |
| 10556 | BISCOITO DE MAIZENA DIETETICO |
| 10416 | BISCOITO DE MAIZENA LIGHT |
| 02303 | BISCOITO DE MANTEIGA |
| 10503 | BISCOITO DE MANTEIGA DIET |
| 10543 | BISCOITO DE MANTEIGA DIETETICO |
| 10403 | BISCOITO DE MANTEIGA LIGHT |
| 02327 | BISCOITO DE MILHO |
| 10527 | BISCOITO DE MILHO DIET |
| 10567 | BISCOITO DE MILHO DIETETICO |
| 10427 | BISCOITO DE MILHO LIGHT |
| 02350 | BISCOITO DE NATA |
| 02401 | BISCOITO DE POLVILHO DOCE |
| 05301 | BISCOITO DE POLVILHO NAO-ESPECIFICADO |
| 02212 | BISCOITO DE POLVILHO SALGADO |
| 10112 | BISCOITO DE POLVILHO SALGADO LIGHT |
| 02402 | BISCOITO DE POLVILHO SEQUILHO |
| 02240 | BISCOITO DE QUEIJO |
| 02330 | BISCOITO DE QUEIJO DOCE |
| 10530 | BISCOITO DE QUEIJO DOCE DIET |
| 10570 | BISCOITO DE QUEIJO DOCE DIETETICO |
| 10430 | BISCOITO DE QUEIJO DOCE LIGHT |
| 02233 | BISCOITO DE SAL |
| 02342 | BISCOITO DE TAPIOCA |
| 02323 | BISCOITO DEDITOS |
| 10523 | BISCOITO DEDITOS DIET |
| 10563 | BISCOITO DEDITOS DIETETICO |
| 10423 | BISCOITO DEDITOS LIGHT |
| 02202 | BISCOITO DO REINO |
| 10102 | BISCOITO DO REINO LIGHT |
| 01801 | BISCOITO DOCE |
| 02301 | BISCOITO DOCE |
| 02407 | BISCOITO DOCE DE POLVILHO |
| 02319 | BISCOITO DOCE DE QUEIJO |
| 10519 | BISCOITO DOCE DE QUEIJO DIET |
| 10559 | BISCOITO DOCE DE QUEIJO DIETETICO |
| 10419 | BISCOITO DOCE DE QUEIJO LIGHT |
| 08001 | BISCOITO DOCE DIET |
| 10501 | BISCOITO DOCE DIET |
| 08002 | BISCOITO DOCE DIETETICO |
| 10541 | BISCOITO DOCE DIETETICO |
| 11501 | BISCOITO DOCE LIGHT |
| 10401 | BISCOITO DOCE LIGHT |
| 02321 | BISCOITO DOCE SORTIDO |
| 10521 | BISCOITO DOCE SORTIDO DIET |
| 10561 | BISCOITO DOCE SORTIDO DIETETICO |
| 10421 | BISCOITO DOCE SORTIDO LIGHT |
| 02203 | BISCOITO GRISSINI |
| 10103 | BISCOITO GRISSINI LIGHT |
| 02214 | BISCOITO INTEGRAL DE AGUA |
| 10114 | BISCOITO INTEGRAL DE AGUA LIGHT |
| 02224 | BISCOITO ITALIANO |
| 10124 | BISCOITO ITALIANO LIGHT |
| 04804 | BISCOITO LANCHE MIRABEL |
| 08404 | BISCOITO LANCHE MIRABEL DIET |
| 08417 | BISCOITO LANCHE MIRABEL DIETETICO |
| 08304 | BISCOITO LANCHE MIRABEL LIGHT |
| 02337 | BISCOITO MARIA |
| 10537 | BISCOITO MARIA DIET |
| 10577 | BISCOITO MARIA DIETETICO |
| 10437 | BISCOITO MARIA LIGHT |
| 04812 | BISCOITO MIRABEL |
| 08412 | BISCOITO MIRABEL DIET |
| 08425 | BISCOITO MIRABEL DIETETICO |
| 08312 | BISCOITO MIRABEL LIGHT |
| 05201 | BISCOITO NAO-ESPECIFICADO |
| 08601 | BISCOITO NAO-ESPECIFICADO DIET |
| 08602 | BISCOITO NAO-ESPECIFICADO DIETETICO |
| 08501 | BISCOITO NAO-ESPECIFICADO LIGHT |
| 02352 | BISCOITO ORELHA DE GATO |
| 02360 | BISCOITO PALITO DE CHOCOLATE |
| 02251 | BISCOITO PALITO SALGADO |
| 00107 | BISCOITO PARA CAO |
| 02213 | BISCOITO PRESUNTINHO |
| 10113 | BISCOITO PRESUNTINHO LIGHT |
| 02403 | BISCOITO QUEBRA QUEBRA |
| 02208 | BISCOITO QUEIJINHO |
| 10108 | BISCOITO QUEIJINHO LIGHT |
| 04801 | BISCOITO RECHEADO |
| 04814 | BISCOITO RECHEADO DE CHOCOLATE |
| 04815 | BISCOITO RECHEADO DE MORANGO |
| 07901 | BISCOITO RECHEADO DIET |
| 08401 | BISCOITO RECHEADO DIET |
| 07902 | BISCOITO RECHEADO DIETETICO |
| 08414 | BISCOITO RECHEADO DIETETICO |
| 11401 | BISCOITO RECHEADO LIGHT |
| 08301 | BISCOITO RECHEADO LIGHT |
| 04803 | BISCOITO RECHEADO TOSTINE |
| 08403 | BISCOITO RECHEADO TOSTINE DIET |
| 08416 | BISCOITO RECHEADO TOSTINE DIETETICO |
| 08303 | BISCOITO RECHEADO TOSTINE LIGHT |
| 02332 | BISCOITO ROSCA DE COCO |
| 10532 | BISCOITO ROSCA DE COCO DIET |
| 10572 | BISCOITO ROSCA DE COCO DIETETICO |
| 10432 | BISCOITO ROSCA DE COCO LIGHT |
| 02343 | BISCOITO ROSQUINHA DE CHOCOLATE |
| 02329 | BISCOITO ROSQUINHA DE COCO |
| 10529 | BISCOITO ROSQUINHA DE COCO DIET |
| 10569 | BISCOITO ROSQUINHA DE COCO DIETETICO |
| 10429 | BISCOITO ROSQUINHA DE COCO LIGHT |
| 02344 | BISCOITO ROSQUINHA DE LEITE |
| 02250 | BISCOITO RUFFLES SALGADO |
| 02218 | BISCOITO SALGADINHO |
| 10118 | BISCOITO SALGADINHO LIGHT |
| 01901 | BISCOITO SALGADO |
| 02201 | BISCOITO SALGADO |
| 02215 | BISCOITO SALGADO DE COCO |
| 10115 | BISCOITO SALGADO DE COCO LIGHT |
| 02248 | BISCOITO SALGADO DE GOMA |
| 02234 | BISCOITO SALGADO DE MILHO |
| 02235 | BISCOITO SALGADO DE PIZZA |
| 02219 | BISCOITO SALGADO DE POLVILHO |
| 10119 | BISCOITO SALGADO DE POLVILHO LIGHT |
| 02236 | BISCOITO SALGADO INTEGRAL |
| 11601 | BISCOITO SALGADO LIGHT |
| 10101 | BISCOITO SALGADO LIGHT |
| 02324 | BISCOITO SORTIDO |
| 10524 | BISCOITO SORTIDO DIET |
| 10564 | BISCOITO SORTIDO DIETETICO |
| 10424 | BISCOITO SORTIDO LIGHT |
| 02206 | BISCOITO TIPO ITALIANO |
| 10106 | BISCOITO TIPO ITALIANO LIGHT |
| 02310 | BISCOITO TOSTINE |
| 10510 | BISCOITO TOSTINE DIET |
| 10550 | BISCOITO TOSTINE DIETETICO |
| 10410 | BISCOITO TOSTINE LIGHT |
| 01803 | BISCOITO WAFFER |
| 04805 | BISCOITO WAFFER |
| 08405 | BISCOITO WAFFER DIET |
| 08418 | BISCOITO WAFFER DIETETICO |
| 08305 | BISCOITO WAFFER LIGHT |
| 02217 | BISKUIT GRISSINI |
| 10117 | BISKUIT GRISSINI LIGHT |
| 00104 | BISNAGA |
| 00237 | BISNAGUINHA |
| 10037 | BISNAGUINHA DIET |
| 10077 | BISNAGUINHA DIETETICO |
| 00205 | BISTECA BOVINA |
| 07803 | BISTECA NAO-ESPECIFICADA |
| 11405 | BISTECA ORGANICA |
| 03305 | BISTECA SUINA |
| 02201 | BITTER |
| 01004 | BLACKOUT (PLASTICO DE JANELA) |
| 02801 | BLANQUET DE PERU |
| 05501 | BLANQUET DE PERU LIGHT |
| 02802 | BLANQUETE DE PERU |
| 01202 | BLAZER |
| 03501 | BLAZER |
| 01502 | BLAZER |
| 03402 | BLOCKET (BLOCO DE CIMENTO) |
| 03402 | BLOCKET (BLOCO DE CIMENTO) |
| 00602 | BLOCO DE CERAMICA |
| 00602 | BLOCO DE CERAMICA |
| 03401 | BLOCO DE CIMENTO |
| 03401 | BLOCO DE CIMENTO |
| 00216 | BLOCO DE PAPEL |
| 17301 | BLOQUEADOR DE CHAMADAS |
| 00512 | BLOQUEADOR SOLAR |
| 00402 | BLUSA |
| 00302 | BLUSA |
| 00303 | BLUSA |
| 00103 | BLUSA DE LA |
| 00109 | BLUSA DE LA |
| 00401 | BLUSA OU CAMISA |
| 00301 | BLUSA OU CAMISA |
| 00301 | BLUSA OU CAMISA |
| 02611 | BOATES (INGRESSO) |
| 00702 | BOB DE CABELO |
| 00241 | BOBINA DE FAX |
| 08701 | BOBINA DE PLASTICO PARA FREEZER |
| 02103 | BOBO BOVINO |
| 05601 | BOBO DE CAMARAO PARA VIAGEM |
| 00905 | BOBOM CASEIRO |
| 01213 | BOCAIUVA |
| 09401 | BODE ABATIDO |
| 05502 | BODE VIVO |
| 00306 | BODY |
| 00122 | BODY BOARDING (PRANCHA DE SURFE) |
| 02102 | BOFE BOVINO |
| 09801 | BOFE DE CAPRINO |
| 09501 | BOFE NAO-ESPECIFICADO |
| 04201 | BOFE SUINO |
| 00117 | BOIA DE BRACO |
| 05801 | BOIA DE CAIXA DE AGUA |
| 01801 | BOIA DE CAIXA DE AGUA |
| 02006 | BOITE (INGRESSO) |
| 02001 | BOITE, DANCETERIA, DISCOTECA, ETC. |
| 00115 | BOLA (EXCETO DE CRIANCA) |
| 00912 | BOLA DA PA |
| 00202 | BOLA DE CRIANCA |
| 00104 | BOLA DE PINGUE-PONGUE |
| 00107 | BOLA DE SINUCA |
| 00220 | BOLA DE SOPRAR |
| 00103 | BOLA DE TENIS |
| 00503 | BOLA DO PATINHO |
| 11703 | BOLA DO PATINHO ORGANICA |
| 02244 | BOLACHA AGUA E SAL |
| 02304 | BOLACHA AMANTEIGADA |
| 10504 | BOLACHA AMANTEIGADA DIET |
| 10544 | BOLACHA AMANTEIGADA DIETETICA |
| 10404 | BOLACHA AMANTEIGADA LIGHT |
| 04902 | BOLACHA CASEIRA |
| 02333 | BOLACHA COM LEITE |
| 10533 | BOLACHA COM LEITE DIET |
| 10573 | BOLACHA COM LEITE DIETETICA |
| 10433 | BOLACHA COM LEITE LIGHT |
| 02237 | BOLACHA CREAM CRACK |
| 02226 | BOLACHA DE AGUA E SAL |
| 10126 | BOLACHA DE AGUA E SAL LIGHT |
| 02345 | BOLACHA DE CHOCOLATE |
| 02340 | BOLACHA DE COCO |
| 10540 | BOLACHA DE COCO DIET |
| 10580 | BOLACHA DE COCO DIETETICA |
| 10440 | BOLACHA DE COCO LIGHT |
| 02312 | BOLACHA DE FUBA |
| 10512 | BOLACHA DE FUBA DIET |
| 10552 | BOLACHA DE FUBA DIETETICA |
| 10412 | BOLACHA DE FUBA LIGHT |
| 02361 | BOLACHA DE GOMA |
| 02346 | BOLACHA DE LEITE |
| 02347 | BOLACHA DE LEITE E MEL |
| 02348 | BOLACHA DE MAIZENA |
| 02313 | BOLACHA DE MANTEIGA |
| 10513 | BOLACHA DE MANTEIGA DIET |
| 10553 | BOLACHA DE MANTEIGA DIETETICA |
| 10413 | BOLACHA DE MANTEIGA LIGHT |
| 02356 | BOLACHA DE MEL |
| 02317 | BOLACHA DE MILHO |
| 10517 | BOLACHA DE MILHO DIET |
| 10557 | BOLACHA DE MILHO DIETETICA |
| 10417 | BOLACHA DE MILHO LIGHT |
| 02245 | BOLACHA DE SAL |
| 01802 | BOLACHA DOCE |
| 02334 | BOLACHA DOCE |
| 08003 | BOLACHA DOCE DIET |
| 10534 | BOLACHA DOCE DIET |
| 08004 | BOLACHA DOCE DIETETICA |
| 10574 | BOLACHA DOCE DIETETICA |
| 11502 | BOLACHA DOCE LIGHT |
| 10434 | BOLACHA DOCE LIGHT |
| 04103 | BOLACHA E SUCO |
| 02238 | BOLACHA INTEGRAL |
| 05401 | BOLACHA NAO-ESPECIFICADA |
| 08801 | BOLACHA NAO-ESPECIFICADA DIET |
| 08802 | BOLACHA NAO-ESPECIFICADA DIETETICA |
| 08701 | BOLACHA NAO-ESPECIFICADA LIGHT |
| 02353 | BOLACHA PALITO |
| 04810 | BOLACHA RECHEADA |
| 08410 | BOLACHA RECHEADA DIET |
| 08423 | BOLACHA RECHEADA DIETETICA |
| 08310 | BOLACHA RECHEADA LIGHT |
| 01902 | BOLACHA SALGADA |
| 02225 | BOLACHA SALGADA |
| 11602 | BOLACHA SALGADA LIGHT |
| 10125 | BOLACHA SALGADA LIGHT |
| 02359 | BOLACHAO DE COCO |
| 02349 | BOLACHAO DOCE |
| 00806 | BOLAO |
| 06601 | BOLDO CHA |
| 00307 | BOLERO |
| 02103 | BOLICHE |
| 03401 | BOLICHE |
| 02608 | BOLINHO AMIDOMIL |
| 00228 | BOLINHO DE AIPIM PARA VIAGEM |
| 04001 | BOLINHO DE ARROZ PARA VIAGEM |
| 00232 | BOLINHO DE BACALHAU PARA VIAGEM |
| 03603 | BOLINHO DE BRIGADEIRO |
| 00221 | BOLINHO DE CAMARAO PARA VIAGEM |
| 00219 | BOLINHO DE CARNE, CAMARAO, ETC. PARA VIAGEM |
| 02501 | BOLINHO DE COCO |
| 04101 | BOLINHO DE COCO PARA VIAGEM |
| 02611 | BOLINHO DE FUBA |
| 02610 | BOLINHO DE MILHO |
| 03301 | BOLINHO DE POLVILHO PARA VIAGEM |
| 02507 | BOLINHO DE QUEIJO |
| 04301 | BOLINHO DE TAPIOCA PARA VIAGEM |
| 04318 | BOLINHO DOCE |
| 01601 | BOLINHO DOCE DE AMENDOIM PARA VIAGEM |
| 03901 | BOLINHO DOCE PARA VIAGEM |
| 02001 | BOLINHO NAO-ESPECIFICADO PARA VIAGEM |
| 03704 | BOLINHO SALGADO |
| 00220 | BOLINHO SALGADO DE CAMARAO PARA VIAGEM |
| 00204 | BOLINHO SALGADO DE CARNE, CAMARAO, ETC. PARA VIAGEM |
| 04303 | BOLO |
| 02701 | BOLO DE AIPIM |
| 02901 | BOLO DE ARROZ |
| 10601 | BOLO DE BANANA |
| 02801 | BOLO DE BATATA DOCE |
| 03101 | BOLO DE CARA |
| 04201 | BOLO DE CENOURA |
| 08201 | BOLO DE CENOURA DIET |
| 08202 | BOLO DE CENOURA DIETETICO |
| 08101 | BOLO DE CENOURA LIGHT |
| 03001 | BOLO DE CENTEIO |
| 03801 | BOLO DE CHOCOLATE |
| 07601 | BOLO DE CHOCOLATE DIET |
| 07602 | BOLO DE CHOCOLATE DIETETICO |
| 07501 | BOLO DE CHOCOLATE LIGHT |
| 04101 | BOLO DE COCO |
| 08001 | BOLO DE COCO DIET |
| 08002 | BOLO DE COCO DIETETICO |
| 07901 | BOLO DE COCO LIGHT |
| 02612 | BOLO DE FUBA |
| 02704 | BOLO DE GOMA |
| 03201 | BOLO DE INHAME |
| 04001 | BOLO DE LARANJA |
| 07801 | BOLO DE LARANJA DIET |
| 07802 | BOLO DE LARANJA DIETETICO |
| 07701 | BOLO DE LARANJA LIGHT |
| 02703 | BOLO DE MACAXEIRA |
| 02708 | BOLO DE MANDIOCA |
| 02601 | BOLO DE MILHO |
| 02505 | BOLO DE OVOS |
| 02501 | BOLO DE QUALQUER MARCA E SABOR |
| 10301 | BOLO DE QUALQUER MARCA E SABOR DIET |
| 10305 | BOLO DE QUALQUER MARCA E SABOR DIETETICO |
| 10201 | BOLO DE QUALQUER MARCA E SABOR LIGHT |
| 07618 | BOLO DE QUALQUER SABOR DIET |
| 07619 | BOLO DE QUALQUER SABOR DIETETICO |
| 09608 | BOLO DE QUALQUER SABOR LIGHT |
| 02506 | BOLO DE QUEIJO |
| 02702 | BOLO DE TAPIOCA |
| 04601 | BOLO DE TRIGO |
| 04109 | BOLO E REFRIGERANTE |
| 09609 | BOLO LIGHT DE QUALQUER SABOR |
| 03901 | BOLSA (NAO-ESPECIFICADA) |
| 30903 | BOLSA DE AGUA QUENTE |
| 06801 | BOLSA DE CACHIMBO |
| 01202 | BOLSA DE COMPRA |
| 02301 | BOLSA DE CRIANCA |
| 00601 | BOLSA DE ESTUDO |
| 00105 | BOLSA DE HOMEM |
| 00203 | BOLSA DE MULHER |
| 03702 | BOLSA DE VIAGEM |
| 00201 | BOLSA E CARTEIRA DE MULHER |
| 30904 | BOLSA TERMICA |
| 00101 | BOLSA, CARTEIRA, PASTA E MALETA DE HOMEM |
| 01301 | BOLSA-ESCOLA |
| 01001 | BOLSA-FAMILIA |
| 03001 | BOLSA-RENDA |
| 02503 | BOM AR |
| 04801 | BOMBA DE AGUA |
| 01402 | BOMBA DE CHIMARRAO |
| 02801 | BOMBA DE INSETICIDA |
| 03701 | BOMBA DE QUALQUER TIPO |
| 07608 | BOMBA DE QUALQUER TIPO DIET |
| 13501 | BOMBA DE QUALQUER TIPO DIET |
| 07609 | BOMBA DE QUALQUER TIPO DIETETICA |
| 13502 | BOMBA DE QUALQUER TIPO DIETETICA |
| 30908 | BOMBA DE TIRAR LEITE HUMANO |
| 01401 | BOMBA E CUIA OU PORONGO DE CHIMARRAO |
| 01207 | BOMBA PARA ENCHER PNEU (BICICLETA) |
| 30906 | BOMBA PARA HIGIENE NASAL |
| 30905 | BOMBA PARA INALACAO |
| 04404 | BOMBOM |
| 00902 | BOMBOM CARAMELIZADO DE QUALQUER MARCA |
| 10502 | BOMBOM CARAMELIZADO DE QUALQUER MARCA DIET |
| 10506 | BOMBOM CARAMELIZADO DE QUALQUER MARCA DIETETICO |
| 10402 | BOMBOM CARAMELIZADO DE QUALQUER MARCA LIGHT |
| 00901 | BOMBOM DE QUALQUER MARCA |
| 10501 | BOMBOM DE QUALQUER MARCA DIET |
| 10505 | BOMBOM DE QUALQUER MARCA DIETETICO |
| 10401 | BOMBOM DE QUALQUER MARCA LIGHT |
| 07207 | BOMBOM DIET |
| 07208 | BOMBOM DIETETICO |
| 09504 | BOMBOM LIGHT |
| 10405 | BOMBOM LIGHT |
| 00903 | BOMBONS SORTIDOS DE QUALQUER MARCA |
| 10503 | BOMBONS SORTIDOS DE QUALQUER MARCA DIET |
| 10507 | BOMBONS SORTIDOS DE QUALQUER MARCA DIETETICO |
| 10403 | BOMBONS SORTIDOS DE QUALQUER MARCA LIGHT |
| 00202 | BOMBRIL |
| 01401 | BONDE, BONDINHO, ETC. |
| 05902 | BONE |
| 05901 | BONE E VISEIRA |
| 00204 | BONECA |
| 02603 | BONECA INFLAVEL |
| 02802 | BONEQUINHOS DE QUALQUER MATERIAL (DECORACAO) |
| 03401 | BONGO |
| 03903 | BONIFICACAO RECEBIDA DA EMPRESA |
| 04102 | BONITO EM CONSERVA |
| 03601 | BORDADEIRA |
| 03001 | BORDADO INGLES |
| 00202 | BORRACHA |
| 30407 | BORRACHA DE DENTICAO DE BEBE |
| 01202 | BORRACHA DE FREIO DE BICICLETA |
| 30406 | BORRACHA DE MORDER |
| 02504 | BORRACHA DE PANELA DE PRESSAO |
| 00504 | BORRACHEIRO (MAO-DE-OBRA) |
| 04301 | BOTA (NAO-ESPECIFICADA) |
| 06401 | BOTA DE BORRACHA (NAO-ESPECIFICADA) |
| 01202 | BOTA DE CRIANCA (EXCETO DE PLASTICO) |
| 01302 | BOTA DE HOMEM (EXCETO DE PLASTICO) |
| 01402 | BOTA DE MULHER (EXCETO DE PLASTICO) |
| 06301 | BOTA DE PLASTICO (NAO-ESPECIFICADA) |
| 03401 | BOTA DE PLASTICO DE CRIANCA |
| 04108 | BOTA ORTOPEDICA |
| 00105 | BOTAO |
| 00503 | BOTE |
| 02106 | BOTE INFLAVEL |
| 00802 | BOTIJAO DE GAS (ALUGUEL) |
| 02002 | BOTIJAO DE GAS VAZIO |
| 03501 | BOX DE BANHEIRO DE QUALQUER MATERIAL |
| 03501 | BOX DE BANHEIRO DE QUALQUER MATERIAL |
| 44113 | BRACAJA |
| 07102 | BRACHOLA |
| 03104 | BRACO BOVINO |
| 07101 | BRAJOLA |
| 03305 | BRASSO |
| 02501 | BREDO |
| 02502 | BREDO DE ESPINHO |
| 02503 | BREDO ROXO |
| 02505 | BREDO VERDADEIRO |
| 02504 | BREDO VERMELHO |
| 00202 | BRETALHA |
| 02315 | BREVIDADE |
| 10515 | BREVIDADE DIET |
| 10555 | BREVIDADE DIETETICA |
| 10415 | BREVIDADE LIGHT |
| 00601 | BRIGA DE GALOS |
| 03601 | BRIGADEIRO |
| 07606 | BRIGADEIRO DIET |
| 13401 | BRIGADEIRO DIET |
| 07607 | BRIGADEIRO DIETETICO |
| 13402 | BRIGADEIRO DIETETICO |
| 03308 | BRILHA ALUMINIO |
| 00104 | BRINCO |
| 05402 | BRINJELA |
| 00201 | BRINQUEDOS E JOGOS |
| 03902 | BRIOCHE |
| 02303 | BRITA |
| 02303 | BRITA |
| 04701 | BROA |
| 03102 | BROA DE CARA |
| 03002 | BROA DE CENTEIO |
| 04501 | BROA DE COCO |
| 04310 | BROA DE FUBA |
| 02606 | BROA DE FUBA |
| 02707 | BROA DE GOMA |
| 03202 | BROA DE INHAME |
| 05101 | BROA DE LEITE |
| 02705 | BROA DE MANDIOCA |
| 04307 | BROA DE MILHO |
| 02602 | BROA DE MILHO |
| 10701 | BROA DE QUEIJO |
| 03004 | BROA PRETA |
| 01628 | BROCA |
| 00105 | BROCHE |
| 01704 | BROCOLIS |
| 07601 | BROCOLIS EM CONSERVA |
| 01705 | BROCOLIS HIBRIDO |
| 01706 | BROCOLIS JAPONES |
| 08101 | BROCOLIS ORGANICO |
| 01703 | BROCOLOS |
| 05001 | BROINHA |
| 03103 | BROINHA DE CARA |
| 03003 | BROINHA DE CENTEIO |
| 04502 | BROINHA DE COCO |
| 02607 | BROINHA DE FUBA |
| 03203 | BROINHA DE INHAME |
| 02706 | BROINHA DE MANDIOCA |
| 02603 | BROINHA DE MILHO ADOCICADA |
| 03005 | BROINHA PRETA |
| 01305 | BRONCODILATADORES |
| 00502 | BRONZEADOR |
| 04601 | BRONZEAMENTO ARTIFICIAL |
| 01106 | BROTE (PAO) |
| 07301 | BROTO DE ALFAFA |
| 03201 | BROTO DE BAMBU |
| 03101 | BROTO DE FEIJAO |
| 01103 | BUCHA DE BANHO |
| 00208 | BUCHA DE LIMPEZA |
| 00207 | BUCHA PARA LIMPEZA |
| 01401 | BUCHA VEGETAL |
| 02304 | BUCHADA BOVINA |
| 07204 | BUCHADA DE BODE |
| 07202 | BUCHADA DE CARNEIRO |
| 07402 | BUCHADA NAO-ESPECIFICADA |
| 02301 | BUCHO BOVINO |
| 07205 | BUCHO DE BODE |
| 07201 | BUCHO DE CARNEIRO |
| 07401 | BUCHO NAO-ESPECIFICADO |
| 04401 | BUCHO SUINO |
| 04301 | BUFE ( COPA E COZINHA) |
| 00801 | BUJAO DE GAS (ALUGUEL) |
| 02001 | BUJAO DE GAS VAZIO |
| 05501 | BULE |
| 01204 | BULGOR |
| 08802 | BUREAU |
| 02104 | BURITI (COCO) |
| 02301 | BURITI EM EMULSAO |
| 02303 | BURITI EM PAPA |
| 02506 | BUSSOLA |
| 03001 | BUSTIE |
| 03301 | BUSTIE |
| 00912 | BUTIA |
| 03501 | BUZIOS, CONCHAS (DECORACAO) |
| 05601 | CABACA |
| 05604 | CABACA PORONGO |
| 05602 | CABACA PURUNGA |
| 05603 | CABACA VERDE |
| 03001 | CABECA BOVINA |
| 08703 | CABECA CAPRINA |
| 08701 | CABECA DE BODE |
| 08702 | CABECA DE CARNEIRO |
| 04001 | CABECA DE GALINHA, PATO, PERU, MARRECO, GALINHA DE ANGOLA |
| 00502 | CABECA DE LOMBO (CARNE BOVINA) |
| 11702 | CABECA DE LOMBO (CARNE BOVINA) ORGANICA |
| 22701 | CABECA DE PACU |
| 00701 | CABECA DE PEIXE |
| 20701 | CABECA DE PEIXE PESCADA |
| 04701 | CABECA DE PINTADO |
| 04902 | CABECA DE PORCO |
| 04901 | CABECA SUINA |
| 44107 | CABECADA |
| 00201 | CABELEIREIRO |
| 04001 | CABIDE |
| 03101 | CABIDE DE PE |
| 01203 | CABO DE FREIO PARA BICICLETA |
| 00407 | CABO DE VASSOURA |
| 05501 | CABRITO VIVO |
| 01403 | CACA E PESCA (LICENCA OU AUTORIZACAO) |
| 04312 | CACAROLA |
| 04104 | CACAROLA ITALIANA |
| 13704 | CACAROLA ITALIANA DIET |
| 13712 | CACAROLA ITALIANA DIETETICA |
| 06301 | CACAU |
| 01221 | CACAU EM PASTA |
| 00902 | CACHACA |
| 00302 | CACHACA |
| 03702 | CACHACA ORGANICA |
| 00106 | CACHECOL |
| 00105 | CACHECOL |
| 00105 | CACHECOL |
| 06701 | CACHIMBO |
| 00402 | CACHORRO QUENTE |
| 00302 | CACHORRO QUENTE PARA VIAGEM |
| 45113 | CACOTE |
| 00122 | CADARCO |
| 02501 | CADASTRO BANCARIO |
| 01106 | CADEADO |
| 01106 | CADEADO |
| 04601 | CADEIRA (COPA E COZINHA) |
| 00202 | CADEIRA (ESTAR E JANTAR) |
| 08101 | CADEIRA (JARDIM, VARANDA, ETC.) |
| 03401 | CADEIRA (QUARTO ADULTO) |
| 00201 | CADEIRA (SALA) |
| 06401 | CADEIRA ALTA DE REFEICAO |
| 01301 | CADEIRA DE BALANCO |
| 01208 | CADEIRA DE BICICLETA |
| 08102 | CADEIRA DE JARDIM |
| 02103 | CADEIRA DE PRAIA |
| 04107 | CADEIRA DE RODAS |
| 08103 | CADEIRA DE VARANDA |
| 05402 | CADEIRA GIRATORIA |
| 00233 | CADEIRA INFANTIL |
| 07501 | CADEIRA NAO-ESPECIFICADA |
| 05401 | CADEIRA PARA COMPUTADOR |
| 07602 | CADEIRA PEQUENA (INFANTIL) |
| 07603 | CADEIRINHA |
| 07601 | CADEIRINHA (INFANTIL) |
| 00833 | CADEIRINHA INFANTIL PARA CARRO |
| 00511 | CADERNETA ESCOLAR |
| 00101 | CADERNO |
| 08701 | CAFE CAPUCCINO DIET |
| 08702 | CAFE CAPUCCINO DIETETICO |
| 12601 | CAFE CAPUCCINO LIGHT |
| 06401 | CAFE CAPUCCINO SOLUVEL |
| 12501 | CAFE CAPUCCINO SOLUVEL DIET |
| 12503 | CAFE CAPUCCINO SOLUVEL DIETETICO |
| 12401 | CAFE CAPUCCINO SOLUVEL LIGHT |
| 01601 | CAFE COM LEITE |
| 03901 | CAFE COM LEITE EM PO |
| 01303 | CAFE COM LEITE PREPARADO PARA VIAGEM |
| 08601 | CAFE COM LEITE SOLUVEL |
| 06001 | CAFE DA ESCOLA |
| 04201 | CAFE DA MANHA |
| 09801 | CAFE DA MANHA (VARIOS ALIMENTOS) |
| 04207 | CAFE DA TARDE |
| 03601 | CAFE DE CEVADA |
| 05001 | CAFE DESCAFEINADO |
| 10901 | CAFE DESCAFEINADO ORGANICO |
| 04205 | CAFE E BISCOITO |
| 04204 | CAFE E BOLO |
| 04202 | CAFE E PAO |
| 04206 | CAFE E SALGADO |
| 03701 | CAFE EM GRAO |
| 10801 | CAFE EM GRAO ORGANICO |
| 02503 | CAFE EM PO |
| 02501 | CAFE MOIDO |
| 10601 | CAFE MOIDO ORGANICO |
| 06401 | CAFE ORGANICO |
| 01301 | CAFE PREPARADO PURO OU COM LEITE PARA VIAGEM |
| 01302 | CAFE PREPARADO PURO PARA VIAGEM |
| 02601 | CAFE SOLUVEL |
| 06402 | CAFE SOLUVEL CAPUCCINO |
| 12502 | CAFE SOLUVEL CAPUCCINO DIET |
| 12504 | CAFE SOLUVEL CAPUCCINO DIETETICO |
| 12402 | CAFE SOLUVEL CAPUCCINO LIGHT |
| 10701 | CAFE SOLUVEL ORGANICO |
| 00503 | CAFE TIPO CAPUCCINO |
| 01305 | CAFE TIPO CAPUCCINO PARA VIAGEM |
| 04203 | CAFE TIPO COLONIAL |
| 00502 | CAFE TIPO EXPRESSO |
| 01304 | CAFE TIPO EXPRESSO PARA VIAGEM |
| 01001 | CAFETEIRA ELETRICA |
| 16801 | CAFETEIRA NAO-ELETRICA |
| 00501 | CAFEZINHO |
| 06402 | CAFEZINHO ORGANICO |
| 08201 | CAFTA PARA VIAGEM |
| 00403 | CAIBRO |
| 00403 | CAIBRO |
| 07703 | CAIBRO DE MADEIRA CERTIFICADA (SELO FSC) |
| 08603 | CAIBRO DE MADEIRA CERTIFICADA (SELO FSC) |
| 01102 | CAIPIRINHA |
| 03101 | CAIPIRINHA |
| 00901 | CAIQUE |
| 02403 | CAIXA BENEFICENTE |
| 03203 | CAIXA DE AGUA |
| 03203 | CAIXA DE AGUA |
| 03201 | CAIXA DE AGUA DE QUALQUER MATERIAL |
| 03201 | CAIXA DE AGUA DE QUALQUER MATERIAL |
| 02003 | CAIXA DE AR CONDICIONADO |
| 02001 | CAIXA DE CORREIO |
| 02001 | CAIXA DE CORREIO |
| 02002 | CAIXA DE CORRESPONDENCIA |
| 02002 | CAIXA DE CORRESPONDENCIA |
| 03901 | CAIXA DE DESCARGA |
| 03901 | CAIXA DE DESCARGA |
| 01701 | CAIXA DE FERRAMENTAS |
| 05302 | CAIXA DE FOSFOROS |
| 02402 | CAIXA DE ISOPOR |
| 03801 | CAIXA DE LUZ (PADRAO) |
| 03801 | CAIXA DE LUZ (PADRAO) |
| 04101 | CAIXA DE PRESENTE |
| 04401 | CAIXA DE REGISTRO DE AGUA |
| 04401 | CAIXA DE REGISTRO DE AGUA |
| 02801 | CAIXA DE SOM |
| 00301 | CAIXA ESCOLAR (CONTRIBUICAO EM DINHEIRO) |
| 07901 | CAIXA PARA AR CONDICIONADO |
| 00109 | CAIXA POSTAL |
| 00306 | CAIXAO |
| 06316 | CAIXAS DE SOM PARA COMPUTADOR |
| 02801 | CAIXINHA |
| 04002 | CAIXINHA (GORJETA) |
| 04801 | CAIXINHA DE MUSICA |
| 02401 | CAIXINHA ENTRE ASSOCIADOS (FUNCIONARIOS, AMIGOS, ETC.) |
| 04603 | CAJA ACU |
| 04605 | CAJA GRANDE |
| 04601 | CAJA MANGA |
| 04704 | CAJA MIRIM |
| 04703 | CAJA PEQUENO |
| 04604 | CAJA UMBU |
| 04602 | CAJARANA |
| 04401 | CAJU |
| 02421 | CAJU DESIDRATADO |
| 01214 | CAJU EM PASTA |
| 09201 | CAJU ORGANICO |
| 04602 | CAJU REFRIGERANTE |
| 08902 | CAJU REFRIGERANTE LIGHT |
| 04603 | CAJUINA |
| 02622 | CAJUINA (SUCO) |
| 02409 | CAJUINA (VINHO) |
| 08903 | CAJUINA LIGHT |
| 03609 | CAJUINA ORGANICA |
| 00903 | CAL |
| 00903 | CAL |
| 00904 | CAL VIRGEM |
| 00904 | CAL VIRGEM |
| 02213 | CALABRESA (LINGUICA) |
| 65102 | CALAMAR |
| 05801 | CALCA (EXCETO COMPRIDA) |
| 04801 | CALCA (EXCETO COMPRIDA) |
| 05101 | CALCA (UNIFORME DE ESPORTE) |
| 00301 | CALCA COMPRIDA |
| 00401 | CALCA COMPRIDA |
| 00401 | CALCA COMPRIDA |
| 04801 | CALCA DE CAPOEIRA (ABADA) |
| 01807 | CALCA ENXUTA |
| 04101 | CALCA PLASTICA |
| 01802 | CALCA PLASTICA |
| 00202 | CALCAO |
| 00204 | CALCAO |
| 02301 | CALCAO (UNIFORME DE ESPORTE) |
| 02701 | CALCAO (UNIFORME DE ESPORTE) |
| 03001 | CALCAO (UNIFORME DE ESPORTE) |
| 00501 | CALCINHA |
| 00601 | CALCINHA |
| 02604 | CALCINHA COMESTIVEL |
| 01605 | CALCIO (CALCIFICANTE OSSEO) |
| 01604 | CALCIO PARA OSTEOPOROSE |
| 12901 | CALCULADORA E AGENDA ELETRONICA |
| 12902 | CALCULADORA ELETRONICA |
| 12801 | CALCULADORA MECANICA |
| 05601 | CALDEIRAO |
| 12001 | CALDO DE ARROZ |
| 07702 | CALDO DE BACALHAU |
| 07301 | CALDO DE BACON |
| 07401 | CALDO DE CALABREZA |
| 07801 | CALDO DE CAMARAO |
| 02621 | CALDO DE CANA |
| 02001 | CALDO DE CANA |
| 02103 | CALDO DE CARNE |
| 04501 | CALDO DE CARNE |
| 03202 | CALDO DE CARNE PARA VIAGEM |
| 02102 | CALDO DE CARURU |
| 02101 | CALDO DE CARURU, CARNE, FEIJAO OU MOCOTO |
| 11101 | CALDO DE CEBOLA |
| 11201 | CALDO DE COSTELA |
| 02104 | CALDO DE FEIJAO |
| 03203 | CALDO DE FEIJAO PARA VIAGEM |
| 05802 | CALDO DE FEIJOADA KNORR |
| 03206 | CALDO DE FEIJOADA PARA VIAGEM |
| 03207 | CALDO DE FRANGO PARA VIAGEM |
| 04401 | CALDO DE GALINHA |
| 05401 | CALDO DE LEGUMES |
| 11301 | CALDO DE MANDIOCA |
| 02105 | CALDO DE MOCOTO |
| 03204 | CALDO DE MOCOTO PARA VIAGEM |
| 07701 | CALDO DE PEIXE |
| 12501 | CALDO DE PERU |
| 04502 | CALDO DE PICANHA |
| 07501 | CALDO DE QUEIJO |
| 03205 | CALDO DE SIRI PARA VIAGEM |
| 07901 | CALDO DE TOMATE |
| 07102 | CALDO DE TUCUPI SEM PIMENTA |
| 07601 | CALDO NAO-ESPECIFICADO |
| 03201 | CALDO PARA VIAGEM |
| 07301 | CALDO VERDE PARA VIAGEM |
| 00601 | CALENDARIO |
| 00114 | CALENDULA |
| 04801 | CALHA |
| 04801 | CALHA |
| 00303 | CALISTA |
| 01801 | CALMANTE |
| 01805 | CALMANTE NATURAL |
| 08010 | CALZONE PRONTO LIGHT PARA VIAGEM |
| 00910 | CALZONE PRONTO PARA VIAGEM |
| 04402 | CALZONE SEMIPRONTO |
| 02302 | CAMA |
| 06501 | CAMA (MOVEL INFANTIL) |
| 02301 | CAMA (QUARTO ADULTO) |
| 02304 | CAMA BOX |
| 02303 | CAMA DE CASAL |
| 03001 | CAMA E MESINHA DE CABECEIRA (QUARTO ADULTO) |
| 04106 | CAMA HOSPITALAR |
| 06502 | CAMA INFANTIL |
| 00403 | CAMARA |
| 01213 | CAMARA DE BICICLETA |
| 00401 | CAMARA E PNEU |
| 60101 | CAMARAO |
| 43106 | CAMARAO BRACO FORTE (AGUA DOCE) |
| 60102 | CAMARAO CABOCLO |
| 43107 | CAMARAO CASTANHO (AGUA DOCE) |
| 60117 | CAMARAO CINZA |
| 02101 | CAMARAO COZIDO PARA VIAGEM |
| 43101 | CAMARAO DE AGUA DOCE |
| 43401 | CAMARAO DE AGUA DOCE SALGADO |
| 43201 | CAMARAO DE AGUA DOCE SEM CASCA |
| 60118 | CAMARAO DE AGUA SALGADA |
| 60601 | CAMARAO DEFUMADO |
| 03601 | CAMARAO EM CONSERVA |
| 07802 | CAMARAO EM PO |
| 60111 | CAMARAO FERRINHO |
| 60120 | CAMARAO FRESCO |
| 43102 | CAMARAO LAGOSTA (AGUA DOCE) |
| 60105 | CAMARAO LEGITIMO |
| 60109 | CAMARAO LIXO |
| 60119 | CAMARAO PISTOLA |
| 60114 | CAMARAO POTIMIRIM |
| 60107 | CAMARAO POTITINGA |
| 60106 | CAMARAO ROSA |
| 60402 | CAMARAO SALGADO |
| 60401 | CAMARAO SECO |
| 60201 | CAMARAO SEM CASCA |
| 60104 | CAMARAO SERRINHA |
| 60110 | CAMARAO SETE BARBAS |
| 60103 | CAMARAO VERMELHO |
| 43105 | CAMARAO ZABUMBA (AGUA DOCE) |
| 04408 | CAMARGO (PALMITO IN NATURA) |
| 07709 | CAMARGO (PALMITO IN NATURA) ORGANICO |
| 09401 | CAMBUI |
| 20901 | CAMERA DE SEGURANCA |
| 12702 | CAMERA FOTOGRAFICA DIGITAL |
| 02305 | CAMINHAO |
| 01502 | CAMINHAO |
| 01101 | CAMINHAO |
| 02306 | CAMIONETE |
| 00403 | CAMISA |
| 00303 | CAMISA |
| 00302 | CAMISA |
| 02201 | CAMISA (UNIFORME DE ESPORTE) |
| 02601 | CAMISA (UNIFORME DE ESPORTE) |
| 02901 | CAMISA (UNIFORME DE ESPORTE) |
| 03701 | CAMISA DE LAMPIAO A GAS |
| 00404 | CAMISA MANGA CURTA |
| 00405 | CAMISA MANGA LONGA |
| 00501 | CAMISETA |
| 00901 | CAMISETA |
| 01901 | CAMISETA |
| 31003 | CAMISINHA (PRESERVATIVO MASCULINO) |
| 03702 | CAMISINHA DE LAMPIAO A GAS |
| 00705 | CAMISOLA |
| 00805 | CAMISOLA |
| 00701 | CAMISOLA, PIJAMA, ROBE, ETC |
| 00801 | CAMISOLA, PIJAMA, ROBE, ETC |
| 10801 | CAMOMILA (EM FOLHA) |
| 03201 | CAMOMILA CHA |
| 16303 | CAMPAINHA |
| 16302 | CAMPAINHA ELETRICA |
| 16301 | CAMPAINHA MUSICAL |
| 01101 | CAMPARI |
| 03306 | CAMPOS DE GOLFE (INGRESSO) |
| 02501 | CANA DE ACUCAR |
| 00102 | CANDIDA |
| 05701 | CANECA |
| 00803 | CANECA DE METAL |
| 03108 | CANELA BOVINA |
| 03101 | CANELA CHA |
| 01003 | CANELA EM FOLHA |
| 01002 | CANELA EM PAU |
| 01001 | CANELA EM PO |
| 00113 | CANELEIRA |
| 03202 | CANELEIRA (POLAINA) |
| 04102 | CANELEIRA (POLAINA) |
| 03002 | CANELONE SEMIPRONTO |
| 11204 | CANELONI EM FATIA LIGHT |
| 08006 | CANELONI PRONTO LIGHT PARA VIAGEM |
| 00906 | CANELONI PRONTO PARA VIAGEM |
| 00203 | CANETA |
| 00601 | CANETA DE OURO OU PRATA |
| 00501 | CANETA RELOGIO |
| 04407 | CANFORA |
| 02102 | CANGA |
| 19302 | CANGURU (BABY BAG) |
| 00520 | CANICO |
| 02101 | CANIVETE |
| 09101 | CANJA DE GALINHA PARA VIAGEM |
| 01407 | CANJA DESIDRATADA |
| 06107 | CANJA DESIDRATADA LIGHT |
| 04323 | CANJICA |
| 00709 | CANJICA DE MILHO EM GRAO |
| 05101 | CANJICA PARA VIAGEM |
| 00706 | CANJIQUINHA DE MILHO EM GRAO |
| 01002 | CANO |
| 01002 | CANO |
| 00504 | CANOA |
| 02002 | CANOA A REMO |
| 00505 | CANOA MOTORIZADA |
| 00522 | CANTIL |
| 06202 | CANTONEIRA (PRATELEIRA) |
| 04801 | CANUDINHO DE PAPEL OU PLASTICO |
| 04402 | CANUDINHO PARA RECHEAR |
| 04401 | CANUDINHO RECHEADO |
| 00602 | CAPA DE ALMOFADA |
| 01206 | CAPA DE BISTECA BOVINA |
| 01305 | CAPA DE CELULAR |
| 05001 | CAPA DE CHUVA |
| 04702 | CAPA DE COMPUTADOR |
| 01204 | CAPA DE CONTRAFILE |
| 01201 | CAPA DE FILE |
| 01701 | CAPA DE LIQUIDIFICADOR, ENCERADEIRA, MAQUINA DE LAVAR, ETC. |
| 01205 | CAPA DE MIGNON |
| 04701 | CAPA DE SOM, VIDEO |
| 00701 | CAPA PARA SOFA |
| 01901 | CAPACETE DE MOTOQUEIRO |
| 01201 | CAPACHO |
| 08007 | CAPELETI PRONTO LIGHT PARA VIAGEM |
| 00907 | CAPELETI PRONTO PARA VIAGEM |
| 03102 | CAPELETI SEMIPRONTO |
| 09602 | CAPIM LIMAO |
| 09601 | CAPIM SANTO |
| 02004 | CAPINADOR |
| 02002 | CAPINADOR (LIMPADOR DE TERRENO) |
| 44121 | CAPINIMA |
| 44120 | CAPININGA |
| 44118 | CAPITARI |
| 03703 | CAPOTE ABATIDA, CONGELADA OU VIVA |
| 03713 | CAPOTE CONGELADA |
| 03714 | CAPOTE VIVA |
| 02801 | CAQUI |
| 02803 | CAQUI CAFE |
| 02802 | CAQUI CHOCOLATE |
| 02804 | CAQUI DURO |
| 02805 | CAQUI FUJI |
| 02806 | CAQUI MOLE |
| 00701 | CARA |
| 00704 | CARA BRANCO |
| 00703 | CARA CHINES |
| 00807 | CARA COZIDO PARA VIAGEM |
| 00705 | CARA DA GUINE |
| 00706 | CARA DE SAPATEIRO |
| 00707 | CARA DO AR |
| 00702 | CARA INHAME |
| 00708 | CARA MIMOSO |
| 00718 | CARA NAMBU |
| 00407 | CARABINA (EXCETO DE CACA) |
| 00505 | CARABINA DE CACA |
| 04801 | CARAMBOLA |
| 00604 | CARAMELO (BALA) |
| 09904 | CARAMELO (BALA) DIET |
| 09917 | CARAMELO (BALA) DIETETICO |
| 09804 | CARAMELO (BALA) LIGHT |
| 61101 | CARAMUJO |
| 03716 | CARANGUEJO |
| 62101 | CARANGUEJO |
| 62110 | CARANGUEJO CATADO |
| 62401 | CARANGUEJO SALGADO |
| 62201 | CARANGUEJO SEM CASCA |
| 62111 | CARANQUEJO VIVO |
| 00709 | CARATINGA |
| 00602 | CARCACA DE GALINHA OU FRANGO |
| 03912 | CARCACA DE PATO |
| 02002 | CARCACA DE PERU |
| 00604 | CARCACA FRANGO |
| 05204 | CARCACA SUINA |
| 01602 | CARDIOTOCOGRAFIA |
| 02303 | CARDO |
| 02304 | CARDONA |
| 00511 | CARGA DE BATERIA |
| 00206 | CARGA DE CANETA |
| 00710 | CARGA DE LAMINA DE BARBEAR |
| 01513 | CARIMA DE MANDIOCA |
| 01913 | CARIRU |
| 01908 | CARIVEL |
| 01405 | CARNAVAL (INGRESSO) |
| 01101 | CARNE ASSADA OU BIFE PREPARADO PARA VIAGEM |
| 01109 | CARNE ASSADA PARA VIAGEM |
| 01703 | CARNE BOVINA COM OSSO NAO-ESPECIFICADA |
| 00501 | CARNE BOVINA DE HAMBURGUER |
| 01601 | CARNE BOVINA DE PRIMEIRA |
| 01602 | CARNE BOVINA DE PRIMEIRA COM OSSO |
| 12002 | CARNE BOVINA DE PRIMEIRA COM OSSO ORGANICA |
| 12001 | CARNE BOVINA DE PRIMEIRA ORGANICA |
| 01701 | CARNE BOVINA DE SEGUNDA |
| 01708 | CARNE BOVINA DE SEGUNDA COM OSSO |
| 10201 | CARNE BOVINA DE TERCEIRA |
| 00301 | CARNE BOVINA DEFUMADA |
| 01012 | CARNE BOVINA DO VAZIO |
| 02601 | CARNE BOVINA EM CONSERVA |
| 04401 | CARNE BOVINA EMPANADA |
| 09101 | CARNE BOVINA NAO-ESPECIFICADA |
| 00401 | CARNE BOVINA SALGADA |
| 09001 | CARNE BOVINA SEM OSSO NAO-ESPECIFICADA |
| 05403 | CARNE CAPRINA |
| 01302 | CARNE CHUPA MOLHO |
| 10101 | CARNE COM OSSO NAO-ESPECIFICADA |
| 01106 | CARNE COZIDA PARA VIAGEM |
| 07609 | CARNE DE ANTA |
| 04101 | CARNE DE AVES DEFUMADA |
| 06901 | CARNE DE AVESTRUZ |
| 05402 | CARNE DE BODE |
| 04804 | CARNE DE BODE SALGADA |
| 07701 | CARNE DE BUFALO |
| 05401 | CARNE DE CABRITO |
| 04803 | CARNE DE CABRITO SALGADA |
| 07615 | CARNE DE CAPIVARA |
| 04802 | CARNE DE CAPIVARA SALGADA |
| 05405 | CARNE DE CAPRINO |
| 62202 | CARNE DE CARANGUEJO |
| 05601 | CARNE DE CARNEIRO |
| 04805 | CARNE DE CARNEIRO SALGADA |
| 07608 | CARNE DE CAVALO |
| 00102 | CARNE DE CHARQUE |
| 07902 | CARNE DE CHURRASCO (KIT) |
| 07620 | CARNE DE COBRA JIBOIA |
| 07605 | CARNE DE COELHO |
| 07617 | CARNE DE COTIA |
| 07612 | CARNE DE CUTIA |
| 07625 | CARNE DE CUTIARA |
| 01401 | CARNE DE FORMATURA |
| 01102 | CARNE DE FRANGO DE HAMBURGUER |
| 05102 | CARNE DE FRANGO DE HAMBURGUER LIGHT |
| 00306 | CARNE DE FRANGO DESFIADA |
| 04601 | CARNE DE FRANGO EM CONSERVA |
| 00305 | CARNE DE FRANGO NAO ESPECIFICADA |
| 04604 | CARNE DE GALINHA EM CONSERVA |
| 00304 | CARNE DE GALINHA OU FRANGO NAO-ESPECIFICADA |
| 05904 | CARNE DE GALINHA OU FRANGO NAO-ESPECIFICADA ORGANICA |
| 00503 | CARNE DE HAMBURGUER BOVINA |
| 07622 | CARNE DE JABUTI |
| 07611 | CARNE DE JACARE |
| 07613 | CARNE DE JACARE SALGADA |
| 07606 | CARNE DE LEBRE |
| 07616 | CARNE DE MACACO |
| 06002 | CARNE DE MARRECO |
| 07601 | CARNE DE OUTROS ANIMAIS |
| 05602 | CARNE DE OVELHA |
| 04806 | CARNE DE OVELHA SALGADA |
| 07603 | CARNE DE PACA |
| 03913 | CARNE DE PATO NAO-ESPECIFICADA |
| 03002 | CARNE DE PERU DE HAMBURGUER |
| 05602 | CARNE DE PERU DE HAMBURGUER LIGHT |
| 01703 | CARNE DE PERU NAO-ESPECIFICADA |
| 01007 | CARNE DE PESCOCO |
| 07621 | CARNE DE PORCO DO MATO |
| 04102 | CARNE DE PORCO NAO ESPECIFICADA |
| 07614 | CARNE DE PORCO SILVESTRE |
| 07607 | CARNE DE PREA |
| 04807 | CARNE DE PREA SALGADA |
| 07623 | CARNE DE PREGUICA |
| 07802 | CARNE DE PRIMEIRA COM OSSO NAO-ESPECIFICADA |
| 07801 | CARNE DE PRIMEIRA NAO-ESPECIFICADA |
| 07619 | CARNE DE QUEIXADA |
| 00105 | CARNE DE SAL PRESA |
| 11202 | CARNE DE SEGUNDA NAO-ESPECIFICADA |
| 11201 | CARNE DE SEGUNDA SEM OSSO NAO-ESPECIFICADA |
| 61202 | CARNE DE SIRI |
| 03605 | CARNE DE SOJA |
| 00201 | CARNE DE SOL |
| 01107 | CARNE DE SOL PREPARADA PARA VIAGEM |
| 07624 | CARNE DE TAMANDUA |
| 07604 | CARNE DE TATU |
| 11001 | CARNE DE TERCEIRA NAO-ESPECIFICADA |
| 08801 | CARNE DE TERNEIRO NAO-NASCIDO |
| 01010 | CARNE DE VAZIO |
| 07602 | CARNE DE VEADO |
| 00203 | CARNE DE VENTO |
| 00202 | CARNE DO SERTAO |
| 00204 | CARNE DO SOL |
| 00106 | CARNE DO SUL |
| 08902 | CARNE EM BIFE |
| 05601 | CARNE EM CONSERVA NAO-ESPECIFICADA |
| 01009 | CARNE MARICA BOVINA |
| 06201 | CARNE MOIDA DE FRANGO |
| 01401 | CARNE MOIDA DE PRIMEIRA |
| 01501 | CARNE MOIDA DE SEGUNDA |
| 10701 | CARNE MOIDA DE TERCEIRA |
| 04301 | CARNE MOIDA NAO-ESPECIFICADA |
| 08901 | CARNE NAO-ESPECIFICADA |
| 03102 | CARNE NAO-ESPECIFICADA DE HAMBURGUER |
| 05702 | CARNE NAO-ESPECIFICADA DE HAMBURGUER LIGHT |
| 05605 | CARNE OVINA |
| 04301 | CARNE SALGADA DE FEIJOADA |
| 04801 | CARNE SALGADA DE OUTROS ANIMAIS |
| 04601 | CARNE SALGADA NAO-ESPECIFICADA |
| 04303 | CARNE SALGADA NAO-ESPECIFICADA PARA FEIJOADA |
| 00101 | CARNE SECA |
| 11101 | CARNE SEM OSSO NAO-ESPECIFICADA |
| 03601 | CARNE SUINA COM OSSO NAO-ESPECIFICADA |
| 06001 | CARNE SUINA DE PRIMEIRA NAO-ESPECIFICADA |
| 10501 | CARNE SUINA DE SEGUNDA NAO-ESPECIFICADA |
| 00601 | CARNE SUINA DEFUMADA |
| 06802 | CARNE SUINA EM BIFE |
| 02701 | CARNE SUINA EM CONSERVA |
| 04101 | CARNE SUINA NAO-ESPECIFICADA |
| 01301 | CARNE SUINA SALGADA |
| 06801 | CARNE SUINA SEM OSSO NAO-ESPECIFICADA |
| 03603 | CARNE VEGETAL |
| 05701 | CARNEIRO VIVO |
| 01501 | CARNET DE MERCADORIAS |
| 01307 | CAROCO DE ALGODAO PARA ANIMAL NAO-ESPECIFICADO |
| 01910 | CAROCO DE BACABA |
| 02307 | CARONA EM AUTOMOVEL |
| 03002 | CARPACCIO CONGELADO PARA VIAGEM |
| 03001 | CARPACCIO PARA VIAGEM |
| 05401 | CARPETE |
| 08701 | CARPETE |
| 10901 | CARQUEJA (EM FOLHA) |
| 03301 | CARRE |
| 01901 | CARREGADOR DE AGUA |
| 02301 | CARREGADOR DE BAGAGEM |
| 01303 | CARREGADOR DE ENERGIA PARA CELULAR |
| 02601 | CARREGADOR DE LENHA |
| 03701 | CARREGADOR DE TACOS DE GOLFE |
| 00108 | CARRETEL |
| 00117 | CARRETILHA DE LINHA (CARRETEL DE LINHA DE ARMARINHO) |
| 01905 | CARRETO DE MATERIAL DE CONSTRUCAO |
| 01905 | CARRETO DE MATERIAL DE CONSTRUCAO |
| 06603 | CARRINHO DE BEBE |
| 06601 | CARRINHO DE BEBE E GONDOLA |
| 00303 | CARRINHO DE BEBIDAS |
| 00212 | CARRINHO DE BRINQUEDO |
| 03801 | CARRINHO DE CARREGAR MALA, VALISE, ETC. |
| 00302 | CARRINHO DE CHA |
| 00301 | CARRINHO DE CHA OU DE BEBIDAS |
| 15001 | CARRINHO DE FEIRA |
| 01637 | CARRINHO DE MAO |
| 04116 | CARRINHO PARA DEFICIENTE FISICO |
| 00213 | CARRO DE BRINQUEDO |
| 00821 | CARROCERIA DE CAMIONETA |
| 00609 | CARROCINHAS |
| 00102 | CARTA |
| 02801 | CARTAO CIDADAO |
| 00227 | CARTAO DE ANIVERSARIO |
| 20701 | CARTAO DE MEMORIA |
| 00230 | CARTAO DE NATAL |
| 01001 | CARTAO DE PRATA |
| 02301 | CARTAO DE TELEFONE CELULAR |
| 00228 | CARTAO DE VISITA |
| 00514 | CARTAO ESCOLAR MAGNETICO |
| 02701 | CARTAO ESPECIAL (TAXA) |
| 00513 | CARTAO MAGNETICO ESCOLAR |
| 00203 | CARTAO TELEFONICO |
| 01004 | CARTAO TELEFONICO(HOBBY) |
| 01504 | CARTAO-REFEICAO |
| 01604 | CARTAO-TRANSPORTE |
| 03101 | CARTEIRA (NAO-ESPECIFICADA) |
| 02302 | CARTEIRA DE CRIANCA |
| 00510 | CARTEIRA DE ESTUDANTE |
| 00104 | CARTEIRA DE HOMEM |
| 00202 | CARTEIRA DE MULHER |
| 00239 | CARTELA DE RIFAR PREMIO |
| 00217 | CARTOLINA |
| 00401 | CARTOMANTE |
| 00101 | CARTORIO |
| 01803 | CARTUCHO APARELHO BARBEAR |
| 05202 | CARTUCHO DE IMPRESSORA DE COMPUTADOR |
| 02201 | CARUARU DA SORTE |
| 01901 | CARURU |
| 01902 | CARURU AZEDO |
| 02506 | CARURU BRAVO (BREDO) |
| 01903 | CARURU DA GUINE |
| 02507 | CARURU DA MATA (BREDO) |
| 02510 | CARURU DE PORCO (BREDO) |
| 02508 | CARURU GRANDE (BREDO) |
| 05701 | CARURU PARA VIAGEM (QUIABO, AMENDOIM, CASTANHA DE CAJU, CAMARAO SECO, ETC.) |
| 02509 | CARURU VERMELHO (BREDO) |
| 08301 | CARVAO MINERAL |
| 05704 | CARVAO PARA CHURRASCO |
| 01001 | CARVAO PARA USO DOMESTICO |
| 05702 | CARVAO PARA USO DOMESTICO |
| 05703 | CARVAO VEGETAL PARA CHURRASCO |
| 05701 | CARVAO VEGETAL PARA USO DOMESTICO |
| 03913 | CASA DE ANIMAL |
| 06001 | CASA DE MADEIRA USADA |
| 00107 | CASACO |
| 00102 | CASACO |
| 00106 | CASACO |
| 04816 | CASADINHA (BISCOITO RECHEADO) |
| 00101 | CASAMENTO |
| 00105 | CASAMENTO EM IGREJA |
| 00104 | CASAMENTO EM TEMPLO RELIGIOSO |
| 06201 | CASCA PARA TORTA |
| 02208 | CASCALHO |
| 02208 | CASCALHO |
| 03002 | CASCO BOVINO (FRONTAL DA CABECA) |
| 04401 | CASCO DE TARTARUGA |
| 01102 | CASEIRA |
| 01101 | CASEIRO |
| 00231 | CASQUINHA DE SIRI |
| 14801 | CASQUINHA DE SORVETE |
| 00302 | CASTANHA DA EUROPA |
| 02001 | CASTANHA DA INDIA |
| 03722 | CASTANHA DE CAJU |
| 00801 | CASTANHA DE CAJU |
| 00701 | CASTANHA DO PARA |
| 00702 | CASTANHA MARANHENSE |
| 02501 | CASTANHA NAO-ESPECIFICADA |
| 00301 | CASTANHA PORTUGUESA |
| 05801 | CATALONHA |
| 05802 | CATALONIA |
| 00507 | CATAMARA |
| 04803 | CATCHUP |
| 04804 | CAT-CHUP |
| 10903 | CATCHUP LIGHT |
| 10904 | CAT-CHUP LIGHT |
| 01406 | CATOLE |
| 00506 | CATRAIA |
| 02416 | CATUABA |
| 03616 | CATUABA ORGANICA |
| 02205 | CAULIM |
| 02205 | CAULIM |
| 00402 | CAUTERIZACAO, CURATIVO, NEBULIZACAO, ETC. |
| 02354 | CAVACO |
| 02302 | CAVACO CHINES |
| 10502 | CAVACO CHINES DIET |
| 10542 | CAVACO CHINES DIETETICO |
| 10402 | CAVACO CHINES LIGHT |
| 01612 | CAVADEIRA |
| 01701 | CAVALETE DE PINTURA DE TELA |
| 03302 | CAVALINHA EM CONSERVA |
| 67109 | CAVAQUEIRA |
| 00501 | CAVAQUINHO |
| 61107 | CAXANGA |
| 03707 | CAXI |
| 04502 | CAXIRI (AGUARDENTE DE MANDIOCA) |
| 04103 | CAXIXE |
| 04001 | CD (DISCO) |
| 04004 | CD DE MUSICA (DISCO) |
| 04003 | CD DE VIDEOGAME (DISCO) |
| 00830 | CD PLAYER |
| 06310 | CDROM |
| 06309 | CD-ROM |
| 04005 | CD-ROM (DISCO) |
| 05701 | CEBOLA |
| 05713 | CEBOLA AMARELA |
| 05711 | CEBOLA ARGENTINA |
| 05703 | CEBOLA BRANCA |
| 05702 | CEBOLA CABECA BRANCA |
| 05709 | CEBOLA CABROBO BRANCA |
| 05712 | CEBOLA DE CABECA |
| 01104 | CEBOLA DE TODO O ANO (CEBOLINHA VERDE) |
| 05705 | CEBOLA DO REINO |
| 05710 | CEBOLA EM CABECA BRANCA |
| 03801 | CEBOLA EM CONSERVA |
| 02801 | CEBOLA EM PO |
| 05707 | CEBOLA GIGANTE |
| 05714 | CEBOLA NACIONAL |
| 05706 | CEBOLA PERA |
| 05704 | CEBOLA ROXA |
| 05708 | CEBOLA VERMELHA |
| 01101 | CEBOLINHA |
| 04901 | CEBOLINHA BRANCA |
| 07301 | CEBOLINHA CRISTAL EM CONSERVA |
| 01103 | CEBOLINHA DE TODO O ANO |
| 10201 | CEBOLINHA DESIDRATADA |
| 01102 | CEBOLINHA VERDE |
| 00801 | CELA DE CAVALO (RECREACAO) |
| 01201 | CENOURA |
| 00304 | CENOURA AMARELA (BATATA BAROA) |
| 00801 | CENOURA EM CONSERVA |
| 01901 | CENOURA ORGANICA |
| 01202 | CENOURA VERMELHA |
| 01902 | CENOURA VERMELHA ORGANICA |
| 01203 | CENOURINHA |
| 01903 | CENOURINHA ORGANICA |
| 17401 | CENTRAL TELEFONICA |
| 02202 | CENTRIFUGA (SECAR ROUPA) |
| 05801 | CENTRIFUGA DE ALIMENTOS |
| 00816 | CERA |
| 03101 | CERA DE ASSOALHO |
| 06001 | CERA DE MOVEIS |
| 03402 | CERA DE SAPATO |
| 01301 | CERA DEPILATORIA |
| 03102 | CERA LIQUIDA DE ASSOALHO |
| 00106 | CERAMICA |
| 01902 | CERAMICA (PECA DE DECORACAO) |
| 20801 | CERCA ELETRICA |
| 06901 | CERCADO |
| 06902 | CERCADO DE CRIANCA |
| 00204 | CEREAL MATINAL DE ARROZ EM FLOCOS |
| 00903 | CEREAL MATINAL DE MILHO EM FLOCOS |
| 05903 | CEREAL MATINAL DE MILHO EM FLOCOS LIGHT |
| 00305 | CEREFOLIO |
| 04501 | CEREJA |
| 04504 | CEREJA DA EUROPA |
| 04503 | CEREJA DOS PASSAROS |
| 01310 | CEREJA EM CALDA |
| 09217 | CEREJA EM CALDA DIET |
| 09218 | CEREJA EM CALDA DIETETICA |
| 04502 | CEREJA GALEGA |
| 00802 | CERIMONIA MILITAR |
| 00229 | CEROL |
| 00703 | CERTIDAO DA POLINTER |
| 00103 | CERTIDAO DE CASAMENTO |
| 00106 | CERTIDAO DE NASCIMENTO |
| 02401 | CERTIFICADO DE CADASTRO DE IMOVEL |
| 02401 | CERTIFICADO DE CADASTRO DE OUTROS IMOVEIS |
| 00505 | CERTIFICADO DE CONCLUSAO ESCOLAR |
| 00101 | CERVEJA |
| 00103 | CERVEJA BRANCA |
| 04303 | CERVEJA BRANCA LIGHT |
| 01002 | CERVEJA CLARA |
| 01001 | CERVEJA CLARA OU PRETA |
| 03402 | CERVEJA EM PO |
| 13601 | CERVEJA LIGHT |
| 04301 | CERVEJA LIGHT |
| 01003 | CERVEJA PRETA |
| 00102 | CERVEJA PRETA |
| 04302 | CERVEJA PRETA LIGHT |
| 01004 | CERVEJA SEM ALCOOL |
| 03301 | CERVEJA SEM ALCOOL |
| 13701 | CERVEJA SEM ALCOOL LIGHT |
| 04401 | CERVEJA SEM ALCOOL LIGHT |
| 03701 | CERZIDEIRA |
| 02701 | CESTA BASICA |
| 00501 | CESTA BASICA |
| 02203 | CESTA DE ACRILICO |
| 00301 | CESTA DE CAFE DA MANHA |
| 00201 | CESTA DE NATAL |
| 08201 | CESTA DE PALHA |
| 00101 | CESTA DE PASCOA |
| 07201 | CESTA DE VIME |
| 00904 | CESTO DE LIXO |
| 09801 | CESTO DE LIXO (PLASTICO) |
| 02601 | CESTO PARA ROUPA |
| 03602 | CEVADA CAFE |
| 00601 | CEVADA EM GRAO |
| 03603 | CEVADA EM PO |
| 03604 | CEVADA MOIDA |
| 05901 | CHA BOVINA NAO-ESPECIFICADA |
| 02702 | CHA DA INDIA |
| 12602 | CHA DA INDIA LIGHT |
| 14801 | CHA DE ABACAXI |
| 13201 | CHA DE ALFAZEMA |
| 02904 | CHA DE ANIZ |
| 06504 | CHA DE ARRUDA |
| 06602 | CHA DE BOLDO |
| 03202 | CHA DE CAMOMILA |
| 03102 | CHA DE CANELA |
| 15301 | CHA DE CARQUEJA |
| 03303 | CHA DE CIDREIRA |
| 00401 | CHA DE DENTRO |
| 11601 | CHA DE DENTRO ORGANICA |
| 14201 | CHA DE ENDRO |
| 03302 | CHA DE ERVA CIDREIRA |
| 02902 | CHA DE ERVA DOCE |
| 00707 | CHA DE FORA |
| 11907 | CHA DE FORA ORGANICA |
| 03002 | CHA DE HORTELA |
| 13101 | CHA DE LIMAO |
| 13501 | CHA DE MACA |
| 15101 | CHA DE MALVA |
| 04402 | CHA DE MARCELA |
| 14901 | CHA DE MORANGO |
| 13701 | CHA DE PESSEGO |
| 03802 | CHA DE QUALQUER TIPO |
| 13801 | CHA DE SENE |
| 05201 | CHA DIET |
| 05204 | CHA DIETETICO |
| 05203 | CHA EMAGRECEDOR |
| 05206 | CHA EMAGRECEDOR DIET |
| 05208 | CHA EMAGRECEDOR DIETETICO |
| 02703 | CHA JAPONES |
| 12603 | CHA JAPONES LIGHT |
| 02801 | CHA MATE |
| 12801 | CHA MATE DIET |
| 08801 | CHA MATE DIET PREPARADO |
| 12804 | CHA MATE DIETETICO |
| 08802 | CHA MATE DIETETICO PREPARADO |
| 12701 | CHA MATE LIGHT |
| 12801 | CHA MATE LIGHT PREPARADO |
| 13003 | CHA MATE ORGANICO |
| 02202 | CHA MATE PREPARADO |
| 05602 | CHA MULTIERVAS |
| 06301 | CHA NAO-ESPECIFICADO |
| 06102 | CHA OUTRO |
| 02201 | CHA PREPARADO |
| 09701 | CHA PREPARADO PARA VIAGEM |
| 02701 | CHA PRETO |
| 12601 | CHA PRETO LIGHT |
| 12701 | CHA PRETO LIGHT PREPARADO |
| 02203 | CHA PRETO PREPARADO |
| 13301 | CHA VERDE |
| 05202 | CHA VITA SANFER |
| 05205 | CHA VITA SANFER DIET |
| 05207 | CHA VITA SANFER DIETETICO |
| 00110 | CHALE |
| 05801 | CHALEIRA |
| 61118 | CHAMA MARE |
| 01006 | CHAMBARIL |
| 01207 | CHAMBINHO (IOGURTE) |
| 08101 | CHAMINHA |
| 01201 | CHAMPANHE |
| 00602 | CHAMPIGNON EM CONSERVA |
| 00709 | CHANDANGA BOVINA |
| 04205 | CHANDELE DE QUALQUER SABOR |
| 13805 | CHANDELE DE QUALQUER SABOR DIET |
| 13810 | CHANDELE DE QUALQUER SABOR DIETETICO |
| 01102 | CHANTIBON |
| 05802 | CHANTIBON DIET |
| 05804 | CHANTIBON DIETETICO |
| 05702 | CHANTIBON LIGHT |
| 01101 | CHANTILLY |
| 05801 | CHANTILLY DIET |
| 05803 | CHANTILLY DIETETICO |
| 05701 | CHANTILLY LIGHT |
| 07501 | CHAPA METALICA |
| 05801 | CHAPEU |
| 00305 | CHAPEU DE BISPO (MAMINHA) |
| 11505 | CHAPEU DE BISPO (MAMINHA) ORGANICO |
| 00223 | CHAPEUZINHO DE PAPEL |
| 02605 | CHAPINHA DE ALISAR CABELOS |
| 00104 | CHARQUE |
| 00301 | CHARUTO |
| 05201 | CHARUTO DE REPOLHO PARA VIAGEM |
| 03801 | CHAS E REMEDIOS ALTERNATIVOS |
| 01653 | CHAVE DE BOCA |
| 01604 | CHAVE DE FENDA |
| 01640 | CHAVE DE TESTE |
| 01627 | CHAVE INGLESA |
| 01301 | CHAVEIRO (PRODUTO) |
| 02301 | CHAVEIRO (SERVICO) |
| 00304 | CHEESBURGUER PARA VIAGEM |
| 00403 | CHEESE BURGUER |
| 00324 | CHEESE CREAM |
| 00325 | CHEESE DE FILE |
| 00408 | CHEESE EGG |
| 00321 | CHEESE EGG PARA VIAGEM |
| 00326 | CHEESE SALADA |
| 00410 | CHEESE TUDO |
| 00322 | CHEESE TUDO PARA VIAGEM |
| 01201 | CHEIRO VERDE |
| 02901 | CHEIRO VERDE EM PO |
| 04201 | CHEQUE VISADO |
| 02701 | CHESTER |
| 03701 | CHESTER ASSADO OU DEFUMADO PARA VIAGEM |
| 03702 | CHESTER DEFUMADO PARA VIAGEM |
| 00608 | CHICLE |
| 09908 | CHICLE DIET |
| 09921 | CHICLE DIETETICO |
| 09808 | CHICLE LIGHT |
| 00803 | CHICLETE |
| 00601 | CHICLETE |
| 07102 | CHICLETE DIET |
| 09901 | CHICLETE DIET |
| 07104 | CHICLETE DIETETICO |
| 09914 | CHICLETE DIETETICO |
| 09402 | CHICLETE LIGHT |
| 09801 | CHICLETE LIGHT |
| 00301 | CHICORIA |
| 01603 | CHICORIA AMARGA (ALMEIRAO) |
| 00302 | CHICORIA CRESPA |
| 00303 | CHICORIA FRISADA |
| 01604 | CHICORIA SELVAGEM (ALMEIRAO) |
| 02606 | CHICOTE EROTICO |
| 02804 | CHIMARRAO |
| 13004 | CHIMARRAO ORGANICO |
| 01901 | CHINELO |
| 00203 | CHINEQUE |
| 00218 | CHINEQUE COM FAROFA |
| 10018 | CHINEQUE COM FAROFA DIET |
| 10058 | CHINEQUE COM FAROFA DIETETICO |
| 10003 | CHINEQUE DIET |
| 10043 | CHINEQUE DIETETICO |
| 01304 | CHIP DE TELEFONE CELULAR |
| 00150 | CHIPA |
| 02227 | CHIPS (SALGADINHOS) |
| 10127 | CHIPS (SALGADINHOS) LIGHT |
| 00226 | CHISBAG |
| 10026 | CHISBAG DIET |
| 10066 | CHISBAG DIETETICO |
| 01703 | CHISPE |
| 01201 | CHISPE DEFUMADO |
| 01702 | CHISPE SALGADO |
| 04104 | CHOCHO |
| 00707 | CHOCOLATE BATOM |
| 00705 | CHOCOLATE BISS (TABLETE) |
| 10105 | CHOCOLATE BISS (TABLETE) DIET |
| 10111 | CHOCOLATE BISS (TABLETE) DIETETICO |
| 10005 | CHOCOLATE BISS (TABLETE) LIGHT |
| 05803 | CHOCOLATE COELHINHO DA PASCOA |
| 12403 | CHOCOLATE COELHINHO DA PASCOA DIET |
| 12407 | CHOCOLATE COELHINHO DA PASCOA DIETETICO |
| 12303 | CHOCOLATE COELHINHO DA PASCOA LIGHT |
| 08501 | CHOCOLATE DIET PREPARADO |
| 08502 | CHOCOLATE DIETETICO PREPARADO |
| 04402 | CHOCOLATE EM BARRA |
| 00704 | CHOCOLATE EM BARRA |
| 10104 | CHOCOLATE EM BARRA DIET |
| 10110 | CHOCOLATE EM BARRA DIETETICO |
| 10004 | CHOCOLATE EM BARRA LIGHT |
| 04401 | CHOCOLATE EM BARRA, TABLETE OU BOMBOM |
| 07201 | CHOCOLATE EM BARRA, TABLETE OU BOMBOM DIET |
| 07202 | CHOCOLATE EM BARRA, TABLETE OU BOMBOM DIETETICO |
| 09501 | CHOCOLATE EM BARRA, TABLETE OU BOMBOM LIGHT |
| 05601 | CHOCOLATE EM CREME |
| 12201 | CHOCOLATE EM CREME DIET |
| 12204 | CHOCOLATE EM CREME DIETETICO |
| 12101 | CHOCOLATE EM CREME LIGHT |
| 00801 | CHOCOLATE EM PO DE QUALQUER MARCA |
| 10301 | CHOCOLATE EM PO DE QUALQUER MARCA DIET |
| 10323 | CHOCOLATE EM PO DE QUALQUER MARCA DIETETICO |
| 10201 | CHOCOLATE EM PO DE QUALQUER MARCA LIGHT |
| 00804 | CHOCOLATE EM PO MUQUI |
| 10304 | CHOCOLATE EM PO MUQUI DIET |
| 10326 | CHOCOLATE EM PO MUQUI DIETETICO |
| 10204 | CHOCOLATE EM PO MUQUI LIGHT |
| 00807 | CHOCOLATE EM PO NESCAU |
| 10307 | CHOCOLATE EM PO NESCAU DIET |
| 10329 | CHOCOLATE EM PO NESCAU DIETETICO |
| 10207 | CHOCOLATE EM PO NESCAU LIGHT |
| 00802 | CHOCOLATE EM PO QUICK |
| 10302 | CHOCOLATE EM PO QUICK DIET |
| 10324 | CHOCOLATE EM PO QUICK DIETETICO |
| 10202 | CHOCOLATE EM PO QUICK LIGHT |
| 00806 | CHOCOLATE EM PO TODDY |
| 10306 | CHOCOLATE EM PO TODDY DIET |
| 10328 | CHOCOLATE EM PO TODDY DIETETICO |
| 10206 | CHOCOLATE EM PO TODDY LIGHT |
| 04403 | CHOCOLATE EM TABLETE |
| 00703 | CHOCOLATE EM TABLETE |
| 10103 | CHOCOLATE EM TABLETE DIET |
| 10109 | CHOCOLATE EM TABLETE DIETETICO |
| 10003 | CHOCOLATE EM TABLETE LIGHT |
| 04501 | CHOCOLATE ENGARRAFADO OU ENCARTONADO |
| 11301 | CHOCOLATE ENGARRAFADO OU ENCARTONADO DIET |
| 11304 | CHOCOLATE ENGARRAFADO OU ENCARTONADO DIETETICO |
| 11201 | CHOCOLATE ENGARRAFADO OU ENCARTONADO LIGHT |
| 05201 | CHOCOLATE GRANULADO |
| 11801 | CHOCOLATE GRANULADO DIET |
| 11802 | CHOCOLATE GRANULADO DIETETICO |
| 11701 | CHOCOLATE GRANULADO LIGHT |
| 12401 | CHOCOLATE LIGHT PREPARADO |
| 04504 | CHOCOLATE LIQUIDO |
| 00803 | CHOCOLATE NESCAU |
| 10303 | CHOCOLATE NESCAU DIET |
| 10325 | CHOCOLATE NESCAU DIETETICO |
| 10203 | CHOCOLATE NESCAU LIGHT |
| 07601 | CHOCOLATE PARA VIAGEM |
| 03401 | CHOCOLATE PREPARADO |
| 00805 | CHOCOLATE TODDY |
| 10305 | CHOCOLATE TODDY DIET |
| 10327 | CHOCOLATE TODDY DIETETICO |
| 10205 | CHOCOLATE TODDY LIGHT |
| 04503 | CHOCOMILK ENCARTONADO |
| 11303 | CHOCOMILK ENCARTONADO DIET |
| 11203 | CHOCOMILK ENCARTONADO LIGHT |
| 11306 | CHOCOMILK ENCARTONADO LIGHT DIETETICO |
| 04502 | CHOCOMILK ENGARRAFADO |
| 11302 | CHOCOMILK ENGARRAFADO DIET |
| 11202 | CHOCOMILK ENGARRAFADO LIGHT |
| 11305 | CHOCOMILK ENGARRAFADO LIGHT DIETETICO |
| 00241 | CHOCOTONE |
| 00204 | CHOPE |
| 01202 | CHOPE CLARO OU ESCURO |
| 00201 | CHOPP |
| 00203 | CHOPP CLARO |
| 01201 | CHOPP CLARO OU ESCURO |
| 00202 | CHOPP ESCURO |
| 61117 | CHORA MARE |
| 02401 | CHOURICO |
| 30404 | CHUCA |
| 04101 | CHUCHU |
| 00902 | CHUCRUTE |
| 00206 | CHULETA BOVINA |
| 00203 | CHULETA COM OSSO (CONTRAFILE) |
| 11403 | CHULETA COM OSSO (CONTRAFILE) ORGANICA |
| 07804 | CHULETA NAO-ESPECIFICADA |
| 11406 | CHULETA ORGANICA |
| 03302 | CHULETA SUINA |
| 02703 | CHUMBINHO MATA-RATOS |
| 00511 | CHUMBO DE PESCA |
| 04804 | CHUP-CHUP (PICOLE ENSACADO) |
| 11404 | CHUP-CHUP (PICOLE ENSACADO) LIGHT |
| 30402 | CHUPETA OU BICO |
| 01014 | CHURRASCO DE FRANGO PARA VIAGEM |
| 03801 | CHURRASCO PARA VIAGEM |
| 13201 | CHURRASQUEIRA |
| 03713 | CHURRASQUINHO (ESPETINHO) |
| 01012 | CHURRASQUINHO DE FRANGO PARA VIAGEM |
| 01103 | CHURRASQUINHO PARA VIAGEM |
| 04313 | CHURRO |
| 05001 | CHURRO |
| 00109 | CHUTEIRA |
| 00501 | CHUVEIRO ELETRICO |
| 04902 | CHUVEIRO ELETRICO |
| 04901 | CHUVEIRO OU DUCHA ELETRICOS |
| 02612 | CICATRIZANTE |
| 02301 | CIDRA |
| 06901 | CIGARREIRA |
| 00401 | CIGARRILHA |
| 00101 | CIGARRO |
| 01602 | CIGARRO DE MACONHA |
| 01301 | CIGARRO DE PALHA |
| 04209 | CILINDRO OU TUBO DE OXIGENIO (DESPESA COM SAUDE) |
| 16005 | CILINDROS PARA MASSAS |
| 00402 | CILIO POSTICO |
| 00503 | CIMENTCOLA OU CIMENTOCOLA |
| 00503 | CIMENTCOLA OU CIMENTOCOLA |
| 00501 | CIMENTO |
| 00501 | CIMENTO |
| 00502 | CIMENTO BRANCO |
| 00502 | CIMENTO BRANCO |
| 00101 | CINEMA (INGRESSO) |
| 02602 | CINEMA (INGRESSO) |
| 02604 | CINEMATECAS (INGRESSO) |
| 02001 | CINTA |
| 04114 | CINTA TERMICA (CORRETIVA) |
| 01805 | CINTEIRO |
| 04602 | CINTILOGRAFIA |
| 04601 | CINTILOGRAFIA OSSEA |
| 04501 | CINTO (NAO-ESPECIFICADO) |
| 02101 | CINTO DE CRIANCA |
| 01701 | CINTO DE HOMEM |
| 01801 | CINTO DE MULHER |
| 01401 | CINZANO |
| 03101 | CINZEIRO |
| 06301 | CIPO DE AMARRAR |
| 01901 | CIRCO (INGRESSO) |
| 02607 | CIRCOS (INGRESSO) |
| 03307 | CIRCUITOS AUTOMOBILISTICOS (INGRESSO) |
| 04101 | CIRCULADOR DE AR |
| 01802 | CIRCULO DE PAIS E MESTRES |
| 06901 | CIRIGUELA |
| 00505 | CIRURGIA (QUALQUER TIPO) |
| 00504 | CIRURGIAO PARA OPERACAO E PARTO |
| 01201 | CITARA |
| 03001 | CLARINETE |
| 04102 | CLIMATIZADOR |
| 03601 | CLINICA E RESIDENCIA PARA IDOSOS |
| 03701 | CLINICA PARA DEFICIENTES FISICOS OU CONVALESCENTES |
| 03801 | CLINICA PSIQUIATRICA OU PARA DEPENDENTES QUIMICOS |
| 00213 | CLIPS |
| 07501 | CLORO LIQUIDO DE LIMPEZA DOMESTICA |
| 05302 | CLORO PARA PISCINA |
| 05302 | CLORO PARA PISCINA |
| 13601 | CLOROFILA (SUCO) |
| 00501 | CLUBE (MENSALIDADE E TAXA) |
| 09101 | COADOR DE CAFE DE PANO |
| 04601 | COADOR DE CAFE DE PAPEL |
| 01901 | COADOR DE TECIDO |
| 04203 | COAGULANTE |
| 08202 | COAGULANTE LIQUIDO |
| 11501 | COAGULANTE LIQUIDO (CLORETO DE MAGNESIO) |
| 02901 | COALHADA |
| 01401 | COALHADA |
| 01403 | COALHADA COM ABACAXI |
| 01404 | COALHADA COM AMEIXA |
| 01405 | COALHADA COM MORANGO |
| 01402 | COALHADA DESNATADA |
| 10801 | COALHADA LIGHT |
| 07201 | COALHADA LIGHT |
| 08201 | COALHO |
| 00404 | COBERTOR |
| 05501 | COBERTURA DE QUALQUER SABOR (DE SORVETE, ETC.) |
| 12001 | COBERTURA DE QUALQUER SABOR (DE SORVETE, ETC.) DIET |
| 12002 | COBERTURA DE QUALQUER SABOR (DE SORVETE, ETC.) DIETETICO |
| 11901 | COBERTURA DE QUALQUER SABOR (DE SORVETE, ETC.) LIGHT |
| 00102 | COCA COLA |
| 13402 | COCA COLA DIET |
| 12903 | COCA COLA LIGHT |
| 00702 | COCA COLA LIGHT |
| 13401 | COCA COLA ZERO |
| 00602 | COCA-COLA |
| 04315 | COCADA |
| 03101 | COCADA |
| 07604 | COCADA DIET |
| 13001 | COCADA DIET |
| 07605 | COCADA DIETETICA |
| 13003 | COCADA DIETETICA |
| 01701 | COCAINA |
| 00907 | COCO ALICURI |
| 01403 | COCO AMARGOSO |
| 01604 | COCO ANAIA |
| 01602 | COCO ANAJA |
| 00906 | COCO ARICURI |
| 00908 | COCO ARIRI |
| 01002 | COCO BABACU |
| 01401 | COCO BABAO |
| 01901 | COCO BACABA |
| 01906 | COCO BACABAI |
| 01907 | COCO BACABINHA |
| 01908 | COCO BACABUCU |
| 01004 | COCO BAGUACU |
| 01003 | COCO BAUACU |
| 01206 | COCO BOCAIUVA |
| 02101 | COCO BURITI |
| 01402 | COCO CATOLE |
| 00101 | COCO DA BAHIA |
| 00104 | COCO DA BAHIA SECO |
| 00105 | COCO DA BAHIA VERDE |
| 00103 | COCO DA INDIA |
| 00102 | COCO DA PRAIA |
| 01001 | COCO DE BABACU |
| 01203 | COCO DE CATARRO |
| 01302 | COCO DE DENDE |
| 01204 | COCO DE ESPINHO |
| 01202 | COCO DE MACAUBA |
| 01103 | COCO DE NATAL |
| 01802 | COCO DE PIRAJA |
| 01801 | COCO DE PUPUNHA |
| 01404 | COCO DE QUARESMA |
| 01102 | COCO DE TICUM |
| 01101 | COCO DE TUCUM |
| 01301 | COCO DENDE |
| 11401 | COCO EM FLOCOS |
| 01601 | COCO INAJA |
| 01703 | COCO JICARA |
| 01702 | COCO JUCARA |
| 01205 | COCO MACAIBA |
| 01207 | COCO MACAJUBA |
| 01201 | COCO MACAUBA |
| 01104 | COCO MARAJA |
| 02103 | COCO MIRITI |
| 01210 | COCO MOCAJUBA |
| 01209 | COCO MUCAIA |
| 01208 | COCO MUCAJA |
| 01603 | COCO NAJA |
| 02401 | COCO NAO-ESPECIFICADO |
| 00909 | COCO NICURI |
| 00901 | COCO OURICURI |
| 01705 | COCO PINA |
| 01804 | COCO PIRAJA |
| 01803 | COCO PUPUNHA |
| 00201 | COCO RALADO INDUSTRIALIZADO |
| 01704 | COCO UACAI |
| 03002 | COCO VERDE (AGUA DE COCO) |
| 02502 | CODEGUIM |
| 03001 | CODORNA ABATIDA, CONGELADA OU VIVA |
| 03002 | CODORNA CONGELADA |
| 03003 | CODORNA VIVA |
| 05802 | COELHINHO DA PASCOA DE CHOCOLATE |
| 12402 | COELHINHO DA PASCOA DE CHOCOLATE DIET |
| 12406 | COELHINHO DA PASCOA DE CHOCOLATE DIETETICO |
| 12302 | COELHINHO DA PASCOA DE CHOCOLATE LIGHT |
| 06705 | COELHO VIVO |
| 02601 | COENTRO (TEMPERO INDUSTRIALIZADO) |
| 00401 | COENTRO (VERDURA) |
| 01203 | COENTRO E CEBOLINHA |
| 00402 | COENTRO EM FOLHA |
| 02604 | COENTRO EM GRAO |
| 02602 | COENTRO EM PO |
| 02301 | COFRE |
| 00601 | COGUMELO EM CONSERVA |
| 05301 | COGUMELO IN NATURA |
| 00510 | COIO ROSA |
| 00218 | COLA |
| 02701 | COLA (EXCETO DUREPOX) |
| 02701 | COLA (EXCETO DUREPOX) |
| 02702 | COLA DE MADEIRA |
| 02702 | COLA DE MADEIRA |
| 02705 | COLA DE SAPATEIRO |
| 02705 | COLA DE SAPATEIRO |
| 03401 | COLA PARA TECIDO |
| 02703 | COLA SUPER BOND |
| 02703 | COLA SUPER BOND |
| 01403 | COLACAO DE GRAU |
| 00102 | COLAR |
| 00406 | COLCHA |
| 06701 | COLCHAO (INFANTIL) |
| 02401 | COLCHAO (QUARTO ADULTO) |
| 02005 | COLCHAO DE ACAMPAMENTO |
| 02402 | COLCHAO DE ADULTO |
| 02006 | COLCHAO DE AR |
| 06702 | COLCHAO INFANTIL |
| 02403 | COLCHAO INFLAVEL |
| 02105 | COLCHAO INFLAVEL |
| 00110 | COLCHETE |
| 08701 | COLCHONETE |
| 00402 | COLECAO DE LIVROS NAO-DIDATICOS |
| 02407 | COLEIRA ANTI PULGA |
| 03903 | COLEIRA DE ANIMAL |
| 03907 | COLEIRA PARA ANIMAL |
| 02006 | COLETA DE LIXO DE OUTROS IMOVEIS |
| 01501 | COLETE |
| 04301 | COLETE |
| 04201 | COLETE |
| 00704 | COLHER |
| 01649 | COLHER DE PEDREIRO |
| 06204 | COLHER DE PLASTICO |
| 02902 | COLIRIOS |
| 00601 | COLLANT |
| 00702 | COLLANT |
| 02405 | COLOCACAO DE JAQUETA DENTARIA |
| 05201 | COLOCACAO DE PIERCING |
| 00239 | COLOMBA DE PASCOA |
| 10039 | COLOMBA DE PASCOA DIET |
| 10079 | COLOMBA DE PASCOA DIETETICA |
| 00206 | COLONIA |
| 08901 | COLONIA (CAPIM) |
| 01701 | COLONIA DE FERIAS |
| 02401 | COLONIA DE FERIAS (EDUCACAO) |
| 01902 | COLONOSCOPIA |
| 05904 | COLORABA |
| 01201 | COLORAU |
| 01205 | COLORAU COM PIMENTA |
| 01202 | COLORIFICO |
| 04001 | COMBATE AO ALCOOLISMO E TABAGISMO |
| 01701 | COMBINACAO |
| 02101 | COMBINACAO |
| 06314 | COMBO GRAVADOR |
| 08801 | COMBOGO (CERAMICA VAZADA) |
| 00501 | COMBUSTIVEL DE VEICULO |
| 01101 | COMEMORACAO NATALINA |
| 03202 | COMINHO E PIMENTA DO REINO |
| 01801 | COMINHO EM GRAO |
| 05201 | COMINHO EM PO |
| 01203 | COMISSAO DE CORRETOR DE IMOVEL |
| 01201 | COMISSAO DE IMOBILIARIA |
| 05001 | COMISSAO DE VENDA (ESPORADICA) |
| 02502 | COMODA |
| 07701 | COMODA (MOVEL INFANTIL) |
| 02501 | COMODA (QUARTO ADULTO) |
| 07702 | COMODA INFANTIL |
| 00831 | COMPACT DISC PLAYER |
| 00221 | COMPASSO |
| 00202 | COMPLEMENTACAO DE OLEO LUBRIFICANTE |
| 00104 | COMPLEMENTACAO DE PLANO/SEGURO-SAUDE (EMPRESA) |
| 03801 | COMPLEMENTACAO DO IMPOSTO DE RENDA |
| 03205 | COMPLEMENTO ALIMENTAR |
| 05701 | COMPLEMENTO ALIMENTAR DE QUALQUER SABOR |
| 05602 | COMPLEMENTO VITAMINICO DE SOJA |
| 06402 | COMPLEMENTO VITAMINICO DE SOJA ORGANICA |
| 02003 | COMPLEXO B |
| 02004 | COMPLEXO VITAMINICO |
| 05702 | COMPOSTO ALIMENTAR DE QUALQUER SABOR |
| 05901 | COMPOTEIRA |
| 01801 | COMPRA DE ANIMAL DOMESTICO |
| 18901 | COMPRESSOR DE AR |
| 06202 | COMPUTADOR |
| 07203 | COMPUTADOR PORTATIL |
| 00203 | COMUNHAO |
| 06502 | CONCENTRADO ALIMENTAR DIET SHAKE |
| 06307 | CONCENTRADO DE LIMPEZA |
| 02404 | CONCENTRADO DE REFRESCO EM PO |
| 11104 | CONCENTRADO DE REFRESCO EM PO DIET |
| 11111 | CONCENTRADO DE REFRESCO EM PO DIETETICO |
| 11004 | CONCENTRADO DE REFRESCO EM PO LIGHT |
| 06001 | CONCHA |
| 06201 | CONCRETO USINADO |
| 06201 | CONCRETO USINADO |
| 00420 | CONDICIONADOR CAPILAR |
| 00402 | CONDIMENTO MISTO INDUSTRIALIZADO EM PASTA |
| 08402 | CONDIMENTO NAO-ESPECIFICADO |
| 00401 | CONDOMINIO |
| 07601 | CONDOMINIO DE JAZIGO |
| 00701 | CONDOMINIO DE OUTROS IMOVEIS |
| 02204 | CONFEITO DE AMENDOIM |
| 02203 | CONFEITO DE CHOCOLATE |
| 02207 | CONFEITO DE LEITE |
| 02206 | CONFEITO DE MENTA |
| 02205 | CONFEITO NAO-ESPECIFICADO |
| 02201 | CONFEITOS DE BOLOS E DOCES |
| 00706 | CONFETE |
| 10106 | CONFETE DIET |
| 10112 | CONFETE DIETETICO |
| 10006 | CONFETE LIGHT |
| 00232 | CONFETE PARA CARNAVAL |
| 02403 | CONFORT |
| 00702 | CONGELADO PRONTO PARA VIAGEM |
| 04901 | CONHAQUE |
| 01601 | CONHAQUE |
| 01813 | CONJUNTINHO PAGAO |
| 13801 | CONJUNTO COMPLETO DE COPOS FINOS OU DE LUXO |
| 00822 | CONJUNTO DE BACIAS DE ALUMINIO |
| 00930 | CONJUNTO DE BACIAS DE PLASTICO |
| 00821 | CONJUNTO DE BALDES DE ALUMINIO |
| 00929 | CONJUNTO DE BALDES DE PLASTICO |
| 05302 | CONJUNTO DE BANDEJAS |
| 00911 | CONJUNTO DE BANHEIRO DE PLASTICO |
| 05201 | CONJUNTO DE BERMUDA E BLUSA |
| 02601 | CONJUNTO DE BERMUDA E BLUSA |
| 05301 | CONJUNTO DE CALCA E BLAZER |
| 03901 | CONJUNTO DE CALCA E BLUSA |
| 02701 | CONJUNTO DE CALCA E CAMISA |
| 03501 | CONJUNTO DE CALCA E CAMISA |
| 03101 | CONJUNTO DE CALCA E JAQUETA |
| 04401 | CONJUNTO DE CALCA E JAQUETA |
| 03701 | CONJUNTO DE CALCA E JAQUETA |
| 05701 | CONJUNTO DE CALCINHA E SUTIA |
| 04701 | CONJUNTO DE CALCINHA E SUTIA |
| 05401 | CONJUNTO DE CASACO E BLUSA |
| 06101 | CONJUNTO DE CHIMARRAO |
| 00705 | CONJUNTO DE COLHERES |
| 00709 | CONJUNTO DE CONCHAS |
| 00102 | CONJUNTO DE COPOS COMUNS DE VIDRO |
| 00305 | CONJUNTO DE ESCOVAS DE MAMADEIRA |
| 00708 | CONJUNTO DE ESCUMADEIRAS |
| 00833 | CONJUNTO DE ESPETOS DE METAL |
| 00707 | CONJUNTO DE FACAS |
| 01603 | CONJUNTO DE FERRAMENTAS |
| 01801 | CONJUNTO DE FORMAS DE BARQUETE |
| 00826 | CONJUNTO DE FORMAS DE BOLO, PIZZA, PUDIM, TORTA |
| 00706 | CONJUNTO DE GARFOS |
| 13402 | CONJUNTO DE JANTAR |
| 07801 | CONJUNTO DE JARRA E COPOS |
| 01301 | CONJUNTO DE LATAS DE MANTIMENTOS |
| 05901 | CONJUNTO DE MALHA |
| 00824 | CONJUNTO DE MARMITAS |
| 00904 | CONJUNTO DE MOLETON |
| 13602 | CONJUNTO DE PANELAS |
| 00606 | CONJUNTO DE PANELAS |
| 02103 | CONJUNTO DE PANELAS DE BARRO |
| 06201 | CONJUNTO DE PIREX |
| 06401 | CONJUNTO DE POTES |
| 00202 | CONJUNTO DE PRATOS DE LOUCA |
| 03601 | CONJUNTO DE SAIA E BLAZER |
| 03301 | CONJUNTO DE SAIA E BLUSA |
| 03401 | CONJUNTO DE SAIA E BLUSA |
| 04501 | CONJUNTO DE SAIA E JAQUETA |
| 04501 | CONJUNTO DE SAIA E JAQUETA |
| 01501 | CONJUNTO DE SALADEIRAS |
| 03401 | CONJUNTO DE SHORT E BLUSA |
| 02801 | CONJUNTO DE SHORT E CAMISA |
| 02501 | CONJUNTO DE SHORT E CAMISA |
| 06301 | CONJUNTO DE SOBREMESA |
| 02901 | CONJUNTO DE SOM ACOPLADO |
| 03203 | CONJUNTO DE TACAS PARA SORVETE |
| 03001 | CONJUNTO DE TECIDO DE BANHEIRO |
| 04601 | CONJUNTO DE TRAVESSAS REFRATARIAS PARA MICROONDAS |
| 00402 | CONJUNTO DE XICARAS COMUNS |
| 00601 | CONJUNTO ESPORTIVO |
| 00801 | CONJUNTO ESPORTIVO |
| 00901 | CONJUNTO ESPORTIVO |
| 00501 | CONJUNTO ESTOFADO |
| 00601 | CONJUNTO ESTOFADO E MESINHA(S) |
| 01812 | CONJUNTO PAGAO |
| 02804 | CONJUNTO PARA PRIMEIRA COMUNHAO |
| 03202 | CONJUNTO PARA SORVETE |
| 00912 | CONJUNTO PLASTICO DE BANHEIRO |
| 00933 | CONJUNTO PLASTICO DE CONDIMENTOS |
| 00931 | CONJUNTO PLASTICO DE MANTIMENTOS OU CONDIMENTOS |
| 00203 | CONJUNTO SANITARIO |
| 00203 | CONJUNTO SANITARIO |
| 00101 | CONSELHO E ASSOCIACAO DE CLASSE |
| 04401 | CONSERTO DE ALICATE |
| 03001 | CONSERTO DE ALTO-FALANTE |
| 01901 | CONSERTO DE ANTENA PARABOLICA (MAO-DE-OBRA) |
| 01801 | CONSERTO DE ANTENA PARABOLICA (PECA) |
| 01701 | CONSERTO DE ANTENA PARABOLICA (PECA+MAO-DE-OBRA) |
| 04501 | CONSERTO DE APARELHO CELULAR |
| 15001 | CONSERTO DE APARELHO ELETRONICO (PECA + MAO-DE-OBRA) |
| 04401 | CONSERTO DE APARELHO SONORO (MAO-DE-OBRA) |
| 04301 | CONSERTO DE APARELHO SONORO (PECA) |
| 04201 | CONSERTO DE APARELHO SONORO (PECA+MAO-DE-OBRA) |
| 06501 | CONSERTO DE AR CONDICIONADO (MAO-DE-OBRA) |
| 06401 | CONSERTO DE AR CONDICIONADO (PECA) |
| 06301 | CONSERTO DE AR CONDICIONADO (PECA+MAO-DE-OBRA) |
| 00701 | CONSERTO DE ASPIRADOR DE PO (MAO-DE-OBRA) |
| 00601 | CONSERTO DE ASPIRADOR DE PO (PECA) |
| 00501 | CONSERTO DE ASPIRADOR DE PO (PECA+MAO-DE-OBRA) |
| 02701 | CONSERTO DE BARBEADOR ELETRICO |
| 08301 | CONSERTO DE BATEDEIRA DE BOLO (MAO-DE-OBRA) |
| 08201 | CONSERTO DE BATEDEIRA DE BOLO (PECA) |
| 08101 | CONSERTO DE BATEDEIRA DE BOLO (PECA+MAO-DE-OBRA) |
| 16101 | CONSERTO DE BEBEDOURO ELETRICO (MAO DE OBRA) |
| 16001 | CONSERTO DE BEBEDOURO ELETRICO (PECA) |
| 15901 | CONSERTO DE BEBEDOURO ELETRICO (PECA+MAO-DE-OBRA) |
| 00603 | CONSERTO DE BICICLETA (MAO-DE-OBRA) |
| 00602 | CONSERTO DE BICICLETA (PECA) |
| 00601 | CONSERTO DE BICICLETA (PECA+MAO-DE-OBRA) |
| 00404 | CONSERTO DE BOLSA |
| 15801 | CONSERTO DE BOMBA DE AGUA ELETRICA (MAO-DE-OBRA) |
| 15701 | CONSERTO DE BOMBA DE AGUA ELETRICA (PECA) |
| 15601 | CONSERTO DE BOMBA DE AGUA ELETRICA (PECA+MAO-DE-OBRA) |
| 02001 | CONSERTO DE BRINQUEDOS E EQUIPAMENTOS ESPORTIVOS (EXCETO CACA E PESCA) |
| 16201 | CONSERTO DE CAFETEIRA (PECA+MAO-DE-OBRA) |
| 02901 | CONSERTO DE CALCULADORA |
| 02601 | CONSERTO DE CANETA, LAPISEIRA |
| 13101 | CONSERTO DE CENTRIFUGA (MAO-DE-OBRA) |
| 13001 | CONSERTO DE CENTRIFUGA (PECA) |
| 12901 | CONSERTO DE CENTRIFUGA (PECA+MAO-DE-OBRA) |
| 00403 | CONSERTO DE CHINELO |
| 11901 | CONSERTO DE CHUVEIRO ELETRICO (MAO-DE-OBRA) |
| 11801 | CONSERTO DE CHUVEIRO ELETRICO (PECA) |
| 11701 | CONSERTO DE CHUVEIRO ELETRICO (PECA+MAO-DE-OBRA) |
| 13701 | CONSERTO DE CONTROLE REMOTO (MAO-DE-OBRA) |
| 13601 | CONSERTO DE CONTROLE REMOTO (PECA) |
| 13501 | CONSERTO DE CONTROLE REMOTO (PECA+MAO-DE-OBRA) |
| 02201 | CONSERTO DE DISCMAN |
| 02202 | CONSERTO DE DISKMAN |
| 14301 | CONSERTO DE DVD (MAO-DE-OBRA) |
| 14201 | CONSERTO DE DVD (PECA) |
| 14101 | CONSERTO DE DVD (PECA+MAO-DE-OBRA) |
| 11301 | CONSERTO DE ENCERADEIRA (MAO-DE-OBRA) |
| 11201 | CONSERTO DE ENCERADEIRA (PECA) |
| 11101 | CONSERTO DE ENCERADEIRA (PECA+MAO-DE-OBRA) |
| 02101 | CONSERTO DE EQUIPAMENTOS DE CACA (ARMA DE FOGO, ETC.) |
| 02401 | CONSERTO DE EQUIPAMENTOS DE PESCA (TARRAFA, MOLINETE, ETC.) |
| 14001 | CONSERTO DE FAX (MAO-DE-OBRA) |
| 13901 | CONSERTO DE FAX (PECA) |
| 13801 | CONSERTO DE FAX (PECA+MAO-DE-OBRA) |
| 01601 | CONSERTO DE FERRO ELETRICO (MAO-DE-OBRA) |
| 01501 | CONSERTO DE FERRO ELETRICO (PECA) |
| 01401 | CONSERTO DE FERRO ELETRICO (PECA+MAO-DE-OBRA) |
| 05101 | CONSERTO DE FILMADORA |
| 01802 | CONSERTO DE FLASH |
| 06201 | CONSERTO DE FOGAO (MAO-DE-OBRA) |
| 06101 | CONSERTO DE FOGAO (PECA) |
| 06001 | CONSERTO DE FOGAO (PECA+MAO-DE-OBRA) |
| 09201 | CONSERTO DE FORNO ELETRICO (MAO-DE-OBRA) |
| 09101 | CONSERTO DE FORNO ELETRICO (PECA) |
| 09001 | CONSERTO DE FORNO ELETRICO (PECA+MAO-DE-OBRA) |
| 03501 | CONSERTO DE FREEZER (MAO DE OBRA) |
| 03401 | CONSERTO DE FREEZER (PECA) |
| 03301 | CONSERTO DE FREEZER (PECA+MAO DE OBRA) |
| 06801 | CONSERTO DE FURADEIRA ELETRICA (MAO-DE-OBRA) |
| 06701 | CONSERTO DE FURADEIRA ELETRICA (PECA) |
| 06601 | CONSERTO DE FURADEIRA ELETRICA (PECA+MAO-DE-OBRA) |
| 03201 | CONSERTO DE GELADEIRA (MAO-DE-OBRA) |
| 03101 | CONSERTO DE GELADEIRA (PECA) |
| 03001 | CONSERTO DE GELADEIRA (PECA+MAO-DE-OBRA) |
| 01101 | CONSERTO DE GRILL (MAO-DE-OBRA) |
| 01001 | CONSERTO DE GRILL (PECA) |
| 00901 | CONSERTO DE GRILL (PECA+MAO-DE-OBRA) |
| 13401 | CONSERTO DE IMPRESSORA (MAO-DE-OBRA) |
| 13301 | CONSERTO DE IMPRESSORA (PECA) |
| 13201 | CONSERTO DE IMPRESSORA (PECA+MAO-DE-OBRA) |
| 14701 | CONSERTO DE INTERFONE (PECA+MAO-DE-OBRA) |
| 01402 | CONSERTO DE JOIAS |
| 03901 | CONSERTO DE LANTERNA |
| 08601 | CONSERTO DE LIQUIDIFICADOR (MAO-DE-OBRA) |
| 08501 | CONSERTO DE LIQUIDIFICADOR (PECA) |
| 08401 | CONSERTO DE LIQUIDIFICADOR (PECA+MAO-DE-OBRA) |
| 07701 | CONSERTO DE MAQUINA DE COSTURA (MAO-DE-OBRA) |
| 07601 | CONSERTO DE MAQUINA DE COSTURA (PECA) |
| 07501 | CONSERTO DE MAQUINA DE COSTURA (PECA+MAO-DE-OBRA) |
| 09501 | CONSERTO DE MAQUINA DE LAVAR LOUCAS (MAO-DE-OBRA) |
| 09401 | CONSERTO DE MAQUINA DE LAVAR LOUCAS (PECA) |
| 09301 | CONSERTO DE MAQUINA DE LAVAR LOUCAS (PECA+MAO-DE-OBRA) |
| 05601 | CONSERTO DE MAQUINA DE LAVAR ROUPA (MAO-DE-OBRA) |
| 05501 | CONSERTO DE MAQUINA DE LAVAR ROUPA (PECA) |
| 05401 | CONSERTO DE MAQUINA DE LAVAR ROUPA (PECA+MAO-DE-OBRA) |
| 08001 | CONSERTO DE MAQUINA DE OVERLOCK (MAO-DE-OBRA) |
| 07901 | CONSERTO DE MAQUINA DE OVERLOCK (PECA) |
| 07801 | CONSERTO DE MAQUINA DE OVERLOCK (PECA+MAO-DE-OBRA) |
| 05901 | CONSERTO DE MAQUINA DE SECAR ROUPA (MAO-DE-OBRA) |
| 05801 | CONSERTO DE MAQUINA DE SECAR ROUPA (PECA) |
| 05701 | CONSERTO DE MAQUINA DE SECAR ROUPA (PECA+MAO-DE-OBRA) |
| 01801 | CONSERTO DE MAQUINA FOTOGRAFICA, FLASH |
| 11601 | CONSERTO DE MICROCOMPUTADOR (MAO-DE-OBRA) |
| 11501 | CONSERTO DE MICROCOMPUTADOR (PECA) |
| 11401 | CONSERTO DE MICROCOMPUTADOR (PECA+MAO-DE-OBRA) |
| 12501 | CONSERTO DE MICROONDAS (MAO-DE-OBRA) |
| 12401 | CONSERTO DE MICROONDAS (PECA) |
| 12301 | CONSERTO DE MICROONDAS (PECA+MAO-DE-OBRA) |
| 00301 | CONSERTO DE MOVEIS (MAO-DE-OBRA) |
| 00201 | CONSERTO DE MOVEIS (PECA) |
| 00101 | CONSERTO DE MOVEIS (PECA+MAO-DE-OBRA) |
| 08901 | CONSERTO DE MULTIPROCESSADOR (MAO-DE-OBRA) |
| 08801 | CONSERTO DE MULTIPROCESSADOR (PECA) |
| 08701 | CONSERTO DE MULTIPROCESSADOR (PECA+MAO-DE-OBRA) |
| 01601 | CONSERTO DE OCULOS |
| 07101 | CONSERTO DE ORGAO ELETRONICO (MAO-DE-OBRA) |
| 07001 | CONSERTO DE ORGAO ELETRONICO (PECA) |
| 06901 | CONSERTO DE ORGAO ELETRONICO (PECA+MAO-DE-OBRA) |
| 10701 | CONSERTO DE OZONIZADOR (MAO-DE-OBRA) |
| 10601 | CONSERTO DE OZONIZADOR (PECA) |
| 10501 | CONSERTO DE OZONIZADOR (PECA+MAO-DE-OBRA) |
| 02501 | CONSERTO DE PERSIANA (MAO-DE-OBRA) |
| 02401 | CONSERTO DE PERSIANA (PECA) |
| 02301 | CONSERTO DE PERSIANA (PECA+MAO-DE-OBRA) |
| 03301 | CONSERTO DE PIANO |
| 01309 | CONSERTO DE PORTAO ELETRONICO |
| 04701 | CONSERTO DE RADIO (MAO+DE+OBRA) |
| 04601 | CONSERTO DE RADIO (PECA) |
| 04501 | CONSERTO DE RADIO (PECA+MAO-DE-OBRA) |
| 05001 | CONSERTO DE RADIO-RELOGIO (MAO-DEOBRA) |
| 04901 | CONSERTO DE RADIO-RELOGIO (PECA) |
| 04801 | CONSERTO DE RADIO-RELOGIO (PECA+MAO-DE-OBRA) |
| 02801 | CONSERTO DE RECEPTOR DE ANTENA PARABOLICA (MAO-DE-OBRA) |
| 02701 | CONSERTO DE RECEPTOR DE ANTENA PARABOLICA (PECA) |
| 02601 | CONSERTO DE RECEPTOR DE ANTENA PARABOLICA (PECA+MAO-DE-OBRA) |
| 01302 | CONSERTO DE RELOGIO |
| 09801 | CONSERTO DE RELOGIO DE MESA (MAO-DE-OBRA) |
| 09701 | CONSERTO DE RELOGIO DE MESA (PECA) |
| 09601 | CONSERTO DE RELOGIO DE MESA (PECA+MAO-DE-OBRA) |
| 10101 | CONSERTO DE RELOGIO DE PAREDE (MAO-DE-OBRA) |
| 10001 | CONSERTO DE RELOGIO DE PAREDE (PECA) |
| 09901 | CONSERTO DE RELOGIO DE PAREDE (PECA+MAO-DE-OBRA) |
| 00402 | CONSERTO DE SAPATO |
| 12201 | CONSERTO DE SECADOR DE CABELOS (MAO-DE-OBRA) |
| 12101 | CONSERTO DE SECADOR DE CABELOS (PECA) |
| 12001 | CONSERTO DE SECADOR DE CABELOS (PECA+MAO-DE-OBRA) |
| 07401 | CONSERTO DE SECRETARIA ELETRONICA (MAO-DE-OBRA) |
| 07301 | CONSERTO DE SECRETARIA ELETRONICA (PECA) |
| 07201 | CONSERTO DE SECRETARIA ELETRONICA (PECA+MAO-DE-OBRA) |
| 01501 | CONSERTO DE SOMBRINHA |
| 04101 | CONSERTO DE TELAO (MAO-DE-OBRA) |
| 04001 | CONSERTO DE TELAO (PECA) |
| 03901 | CONSERTO DE TELAO (PECA+MAO-DE-OBRA) |
| 02201 | CONSERTO DE TELEFONE (MAO-DE-OBRA) |
| 02101 | CONSERTO DE TELEFONE (PECA) |
| 02001 | CONSERTO DE TELEFONE (PECA+MAO-DE-OBRA) |
| 03801 | CONSERTO DE TELEVISAO (MAO-DE-OBRA) |
| 03701 | CONSERTO DE TELEVISAO (PECA) |
| 03601 | CONSERTO DE TELEVISAO (PECA+MAO-DE-OBRA) |
| 01308 | CONSERTO DE TOLDO |
| 11001 | CONSERTO DE TORNEIRA ELETRICA (MAO-DE-OBRA) |
| 10901 | CONSERTO DE TORNEIRA ELETRICA (PECA) |
| 10801 | CONSERTO DE TORNEIRA ELETRICA (PECA+MAO-DE-OBRA) |
| 10401 | CONSERTO DE VENTILADOR (MAO-DE-OBRA) |
| 10301 | CONSERTO DE VENTILADOR (PECA) |
| 10201 | CONSERTO DE VENTILADOR (PECA+MAO-DE-OBRA) |
| 05301 | CONSERTO DE VIDEOCASSETE (MAO-DE-OBRA) |
| 05201 | CONSERTO DE VIDEOCASSETE (PECA) |
| 05101 | CONSERTO DE VIDEOCASSETE (PECA+MAO-DE-OBRA) |
| 02002 | CONSERTO DE VIDEOGAME |
| 12801 | CONSERTO DE VIDEOGAME (MAO-DE-OBRA) |
| 12701 | CONSERTO DE VIDEOGAME (PECA) |
| 12601 | CONSERTO DE VIDEOGAME (PECA+MAO-DE-OBRA) |
| 03101 | CONSERTO DE VIOLAO |
| 02203 | CONSERTO DE WALKMAN |
| 14901 | CONSERTO DO INTERFONE (MAO-DE-OBRA) |
| 00501 | CONSERTO E OUTROS SERVICOS (MAO-DE-OBRA) |
| 04206 | CONSERTO E REPARO DE ARTIGO E APARELHO MEDICOS |
| 00509 | CONSERTO E REPARO DE VIDRO |
| 04201 | CONSERTO E REVISAO (PECA+MAO-DE-OBRA) |
| 05501 | CONSERVANTE PARA ALIMENTOS |
| 02601 | CONSOLE |
| 02101 | CONSORCIO DE AUTOMOVEL |
| 02001 | CONSORCIO DE DINHEIRO |
| 02404 | CONSORCIO DE DINHEIRO |
| 02201 | CONSORCIO DE MOTOCICLETA |
| 00301 | CONSORCIO DE OUTROS IMOVEIS (PRESTACAO) |
| 02206 | CONSORCIO FUNERARIO |
| 08001 | CONSTRUCAO DE JAZIGO |
| 01602 | CONSTRUCAO POR EMPREITADA |
| 01602 | CONSTRUCAO POR EMPREITADA |
| 01209 | CONSULTA MEDICA (ALERGISTA) |
| 01221 | CONSULTA MEDICA ANGIOLOGICA |
| 01202 | CONSULTA MEDICA CARDIOLOGICA |
| 01222 | CONSULTA MEDICA CIRURGIAO PLASTICO |
| 00901 | CONSULTA MEDICA COM CLINICO GERAL |
| 01207 | CONSULTA MEDICA DERMATOLOGICA |
| 01218 | CONSULTA MEDICA ENDOCRINOLOGICA |
| 01208 | CONSULTA MEDICA GASTROENTEROLOGICA |
| 00902 | CONSULTA MEDICA GERAL |
| 01210 | CONSULTA MEDICA GERIATRICA |
| 01106 | CONSULTA MEDICA GINECOLOGICA |
| 01101 | CONSULTA MEDICA GINECO-OBSTETRICIA |
| 01223 | CONSULTA MEDICA HEMATOLOGICA |
| 01211 | CONSULTA MEDICA HOMEOPATICA |
| 01212 | CONSULTA MEDICA NEFROLOGICA (RENAIS) |
| 01205 | CONSULTA MEDICA NEUROLOGICA |
| 02803 | CONSULTA MEDICA NUTRICIONISTA |
| 01107 | CONSULTA MEDICA OBSTETRICA |
| 01203 | CONSULTA MEDICA OFTALMOLOGICA |
| 01213 | CONSULTA MEDICA ONCOLOGICA |
| 01204 | CONSULTA MEDICA ORTOPEDICA |
| 01214 | CONSULTA MEDICA OTOLOGICA |
| 01215 | CONSULTA MEDICA OTORRINO |
| 01001 | CONSULTA MEDICA PEDIATRICA |
| 01216 | CONSULTA MEDICA PNEUMOLOGICA |
| 01219 | CONSULTA MEDICA PROTOLOGICA |
| 01206 | CONSULTA MEDICA PSIQUIATRICA |
| 01217 | CONSULTA MEDICA REUMATOLOGICA |
| 01220 | CONSULTA MEDICA UROLOGICA |
| 02409 | CONSULTA ODONTOLOGICA |
| 02402 | CONTA DE APARELHO NEXTEL |
| 02401 | CONTA DE TELEFONE CELULAR |
| 00253 | CONTACT |
| 00601 | CONTADOR |
| 01705 | CONTRA FILE DE SEGUNDA |
| 01101 | CONTRABAIXO |
| 00201 | CONTRAFILE |
| 11401 | CONTRAFILE ORGANICO |
| 03901 | CONTRASTES RADIOLOGICOS |
| 00502 | CONTRATO DE LOCACAO DE OUTROS IMOVEIS |
| 00302 | CONTRATO DE LOCACAO DO IMOVEL |
| 02402 | CONTRIBUICAO DE GRUPO (FUNCIONARIOS, AMIGOS, CASAIS, ETC.) |
| 00302 | CONTRIBUICAO PARA CAIXA ESCOLAR EM DINHEIRO |
| 02406 | CONTRIBUICAO PARA GREMIO (FUNCIONARIOS DE EMPRESA) |
| 02601 | CONTRIBUICAO PARA OUTRAS ASSOCIACOES |
| 04301 | CONTRIBUICAO PARA PARTIDO POLITICO |
| 03903 | CONTRIBUICAO PARA SINDICATO E ASSOCIACAO DE TRABALHADORES (MENSAL) |
| 03902 | CONTRIBUICAO SINDICAL (ANUAL) |
| 01902 | CONTROLADOR DE APETITE |
| 07803 | CONTROLE REMOTO DE SOM |
| 07802 | CONTROLE REMOTO DE TV |
| 07801 | CONTROLE REMOTO DE TV, SOM, VIDEOCASSETE, ETC. |
| 07804 | CONTROLE REMOTO DE VIDEOCASSETE |
| 00515 | CONVERSAO DE MOTOR PARA GAS |
| 19701 | CONVERSOR DIGITAL PARA TELEVISAO |
| 00229 | CONVITE |
| 00103 | CONVITE DE CASAMENTO |
| 01404 | CONVITE DE FORMATURA |
| 02603 | COOPERATIVA (MENSALIDADE) |
| 00802 | COPA DE PORCO DEFUMADA |
| 01602 | COPA DE PORCO SALGADA |
| 03704 | COPA SUINA |
| 01601 | COPEIRA |
| 01602 | COPEIRO |
| 02302 | COPIA DE CHAVE |
| 00802 | COPIA DE FILME |
| 04701 | COPIA DE MICROFILME DE CHEQUE |
| 00603 | COPIA XEROX |
| 00103 | COPO COMUM DE VIDRO |
| 02202 | COPO DE ACRILICO |
| 00836 | COPO DE ALUMINIO |
| 03501 | COPO DE CRISTAL |
| 00832 | COPO DE INOX |
| 00110 | COPO DE LEITE |
| 00922 | COPO DE LIQUIDIFICADOR |
| 04904 | COPO DE PAPEL OU PLASTICO |
| 00101 | COPO DE VIDRO COMUM |
| 04905 | COPO DESCARTAVEL |
| 00924 | COPO E BASE DE LIQUIDIFICADOR |
| 00936 | COPOS PLASTICOS |
| 02901 | COQUETEL DE FRUTAS |
| 01901 | CORACAO BOVINO |
| 10301 | CORACAO DE CAPRINO |
| 01202 | CORACAO DE FRANGO |
| 01201 | CORACAO DE GALINHA OU FRANGO |
| 06101 | CORACAO DE PERU |
| 04001 | CORACAO SUINO |
| 02208 | CORANTE DE BOLO E DOCE |
| 02101 | CORANTE DE CAFE |
| 07303 | CORANTE DE CERA PARA ASSOALHO |
| 02202 | CORANTES DE BOLOS E DOCES |
| 04002 | CORDA (PECA DE DECORACAO) |
| 00126 | CORDA DE ALGODAO |
| 00521 | CORDA DE REDE DE PESCA |
| 03802 | CORDA DE ROUPA |
| 03801 | CORDA DE VARAL |
| 00125 | CORDAO |
| 00103 | CORDAO (JOIA) |
| 01802 | CORINO |
| 02606 | CORNED BEEF |
| 00304 | COROA DE FLORES |
| 03906 | CORPUS BREAK LIGHT |
| 03103 | CORREDOR BOVINO |
| 00123 | CORREIA DE CHINELO |
| 00101 | CORREIO (CARTA, SELO, ETC.) |
| 03902 | CORRENTE DE ANIMAL |
| 01211 | CORRENTE DE BICICLETA |
| 01654 | CORRENTE DE MOTOSSERA |
| 03906 | CORRENTE PARA ANIMAL |
| 00207 | CORRETIVO |
| 00901 | CORRETOR |
| 00404 | CORRIDA AUTOMOBILISTICA (INGRESSO) |
| 00501 | CORRIDA DE CAVALOS |
| 01623 | CORTADOR DE AZULEJO |
| 06501 | CORTADOR DE BOLO |
| 15609 | CORTADOR DE GRAMA |
| 01601 | CORTADOR DE GRAMA (ALUGUEL) |
| 06601 | CORTADOR DE PIZZA |
| 00803 | CORTADOR DE UNHA |
| 02702 | CORTADOR DE VERDURA (APARELHO) |
| 04301 | CORTE DE CABELO |
| 04302 | CORTE DE CABELO (FEMININO) |
| 04303 | CORTE DE CABELO (MASCULINO) |
| 04701 | CORTE DE CABELO E BARBA |
| 02503 | CORTE DE PELOS DE ANIMAL DOMESTICO |
| 00706 | CORTICOIDE |
| 02502 | CORTINA E TAPETE DE BORRACHA |
| 00801 | CORTINA PRONTA (EXCETO DE PLASTICO) |
| 01001 | CORTINADO |
| 03202 | CORVINA EM CONSERVA |
| 01104 | COSTELA ASSADA PARA VIAGEM |
| 01301 | COSTELA BOVINA |
| 00403 | COSTELA BOVINA SALGADA |
| 05404 | COSTELA DE BODE |
| 01305 | COSTELA DE BOI |
| 05603 | COSTELA DE CARNEIRO |
| 03503 | COSTELA DE PORCO |
| 00901 | COSTELA DE PORCO DEFUMADA |
| 01501 | COSTELA DE PORCO SALGADA |
| 03602 | COSTELA E PE DE FRANGO |
| 03601 | COSTELA E PE DE GALINHA OU FRANGO |
| 06101 | COSTELA NAO-ESPECIFICADA |
| 03501 | COSTELA SUINA |
| 03303 | COSTELETA SUINA |
| 03504 | COSTELINHA DE PORCO |
| 03502 | COSTELINHA SUINA |
| 01701 | COSTUREIRA |
| 00515 | COTA EXTRA ESCOLAR |
| 01001 | COTONETE DE USO COLETIVO |
| 04401 | COTURNO |
| 01901 | COURO |
| 00501 | COUVE |
| 01702 | COUVE BROCOLIS |
| 01701 | COUVE BROCOLOS |
| 02701 | COUVE CHINESA |
| 00505 | COUVE CRESPA |
| 10201 | COUVE DE BRUXELAS |
| 00506 | COUVE DE FOLHAS |
| 00601 | COUVE FLOR |
| 07201 | COUVE FLOR EM CONSERVA |
| 00502 | COUVE MANTEIGA |
| 08201 | COUVE ORGANICA |
| 00504 | COUVE PALMITO |
| 00507 | COUVE PICADA |
| 05906 | COUVE RABANA |
| 05901 | COUVE RABANO |
| 05905 | COUVE RABANO PRETO |
| 05902 | COUVE RABAO |
| 00503 | COUVE REPOLHUDA |
| 00709 | COXA DE ASA DE FRANGO |
| 02301 | COXA DE AVE NAO-ESPECIFICADA |
| 00504 | COXA DE FRANGO |
| 05804 | COXA DE FRANGO ORGANICA |
| 00501 | COXA DE GALINHA OU FRANGO |
| 05802 | COXA DE GALINHA OU FRANGO ORGANICA |
| 03909 | COXA DE PATO |
| 01901 | COXA DE PERU |
| 02502 | COXA E ASA DE FRANGO |
| 02501 | COXA E ASA DE GALINHA OU FRANGO |
| 05301 | COXA E ASA DE PERU |
| 05201 | COXA E ASA NAO-ESPECIFICADA |
| 02602 | COXA E PEITO DE FRANGO |
| 02601 | COXA E PEITO DE GALINHA OU FRANGO |
| 02401 | COXA E SOBRECOXA DE AVE NAO-ESPECIFICADA |
| 00506 | COXA E SOBRECOXA DE FRANGO |
| 01010 | COXA E SOBRECOXA DE FRANGO ASSADO PARA VIAGEM |
| 05801 | COXA E SOBRECOXA DE FRANGO ORGANICA |
| 00503 | COXA E SOBRECOXA DE GALINHA OU FRANGO |
| 05806 | COXA E SOBRECOXA DE GALINHA OU FRANGO ORGANICA |
| 05604 | COXAO DE CARNEIRO |
| 00403 | COXAO DE DENTRO (CHA DE DENTRO) |
| 11603 | COXAO DE DENTRO (CHA DE DENTRO) ORGANICO |
| 00705 | COXAO DE FORA (LAGARTO COMUM) |
| 11905 | COXAO DE FORA (LAGARTO COMUM) ORGANICO |
| 00708 | COXAO DURO |
| 00706 | COXAO DURO (LAGARTO COMUM) |
| 11906 | COXAO DURO (LAGARTO COMUM) ORGANICO |
| 00404 | COXAO MOLE |
| 00402 | COXAO MOLE (CHA DE DENTRO) |
| 11602 | COXAO MOLE (CHA DE DENTRO) ORGANICO |
| 07806 | COXAO NAO-ESPECIFICADO |
| 03402 | COXAO SUINO |
| 03705 | COXINHA |
| 00707 | COXINHA DA ASA DE GALINHA OU FRANGO |
| 04202 | COXINHA DE FRITAR |
| 00205 | COXINHA PARA VIAGEM |
| 04203 | COXINHA SEMIPRONTA |
| 02101 | COZIDO EM CONSERVA |
| 00202 | COZINHEIRA |
| 00201 | COZINHEIRO |
| 00103 | CRAVO |
| 01101 | CRAVO (TEMPERO INDUSTRIALIZADO) |
| 01103 | CRAVO DA INDIA |
| 01102 | CRAVO TEMPERADO |
| 08501 | CREAM CHEESE |
| 01101 | CRECHE |
| 01302 | CREDITO EDUCATIVO (PAGAMENTO) |
| 01901 | CREDITO-EDUCATIVO |
| 00303 | CREMACAO |
| 00303 | CREME ANTIALERGICO |
| 02606 | CREME ANTIMICOTICO |
| 00514 | CREME ANTI-RUGAS |
| 02501 | CREME ANTI-TRANSPIRANTE |
| 02613 | CREME CICATRIZANTE |
| 02001 | CREME DE ACUCAR |
| 00410 | CREME DE ALHO |
| 01205 | CREME DE AMENDOIM |
| 00101 | CREME DE ARROZ |
| 00102 | CREME DE ARROZ VITAMINADO |
| 14401 | CREME DE AVELA |
| 00602 | CREME DE BARBEAR |
| 05604 | CREME DE BRIGADEIRO |
| 00417 | CREME DE CABELO |
| 01404 | CREME DE CEBOLA (SOPA DESIDRATADA) |
| 06104 | CREME DE CEBOLA (SOPA DESIDRATADA) LIGHT |
| 05605 | CREME DE CHOCOLATE |
| 01408 | CREME DE ERVILHA (SOPA DESIDRATADA) |
| 01208 | CREME DE GOIABA |
| 01405 | CREME DE LEGUMES (SOPA DESIDRATADA) |
| 06105 | CREME DE LEGUMES (SOPA DESIDRATADA) LIGHT |
| 01001 | CREME DE LEITE |
| 07501 | CREME DE LEITE DE SOJA |
| 05601 | CREME DE LEITE LIGHT |
| 04701 | CREME DE LEITE ORGANICO |
| 01507 | CREME DE MACAXEIRA |
| 00804 | CREME DE MILHO |
| 00516 | CREME DE PELE |
| 00501 | CREME DE PELE E BRONZEADOR |
| 07502 | CREME DE QUEIJO |
| 07502 | CREME DE SOJA |
| 00423 | CREME DEFINIDOR DE CACHOS |
| 00105 | CREME DENTAL |
| 00101 | CREME DENTAL E FIO DENTAL |
| 30802 | CREME DENTAL MEDICINAL |
| 01302 | CREME DEPILATORIO |
| 02607 | CREME DERMATOLOGICO |
| 00415 | CREME ENXAGUANTE DE CABELO |
| 00507 | CREME HIDRATANTE |
| 00425 | CREME HIDRATANTE CAPILAR |
| 01206 | CREME INFANTIL (EXCETO SOBREMESA) |
| 02629 | CREME PARA ASSADURA |
| 00517 | CREME PARA AXILAS |
| 00434 | CREME PARA CABELO |
| 00505 | CREME PARA MAOS |
| 02608 | CREME PARA O CORPO (MAO, PE, ETC.) |
| 00518 | CREME PARA O ROSTO |
| 00519 | CREME PARA OS PES |
| 02609 | CREME PARA QUEIMADURAS |
| 00402 | CREME RINSE |
| 02802 | CREME VAGINAL |
| 00601 | CREME, PINCEL E OUTROS PRODUTOS DE BARBA |
| 00801 | CREMOGEMA |
| 00805 | CREMOGEMA VITAMINADA |
| 00506 | CREMOSINHO (SORVETE) |
| 00803 | CREMOVITA |
| 01402 | CREOLINA |
| 03730 | CREPE |
| 06101 | CREPE |
| 02903 | CRIADO MUDO |
| 00111 | CRISANTEMO |
| 02512 | CRISTA DE GALO |
| 02602 | CRISTAL (PECA DE DECORACAO) |
| 00402 | CRISTALEIRA |
| 00401 | CRISTALEIRA OU VITRINE |
| 04120 | CRISTALINO (LENTE INTRAOCULAR) |
| 04801 | CROCHETEIRA |
| 02228 | CROCK (SALGADINHOS) |
| 10128 | CROCK (SALGADINHOS) LIGHT |
| 00240 | CROISSANT |
| 10040 | CROISSANT DIET |
| 10080 | CROISSANT DIETETICO |
| 03703 | CROQUETE |
| 00203 | CROQUETE PARA VIAGEM |
| 00202 | CROQUINHOS DE ARROZ |
| 02362 | CROSTOLI |
| 01416 | CRUERA |
| 00204 | CRUSH |
| 13005 | CRUSH LIGHT |
| 01504 | CRUSH LIGHT |
| 00906 | CRUZ MACHADO |
| 01901 | CRUZADINHA |
| 02303 | CUALHEIRA BOVINA |
| 04701 | CUBA PARA PIA (QUALQUER MATERIAL) |
| 04701 | CUBA PARA PIA (QUALQUER MATERIAL) |
| 02503 | CUCA DE QUALQUER TIPO |
| 10303 | CUCA DE QUALQUER TIPO DIET |
| 10307 | CUCA DE QUALQUER TIPO DIETETICO |
| 10203 | CUCA DE QUALQUER TIPO LIGHT |
| 00701 | CUECA |
| 01001 | CUECA |
| 02351 | CUECA VIRADA (BISCOITO DOCE) |
| 01803 | CUEIRO |
| 01403 | CUIA DE CHIMARRAO |
| 05703 | CUMARI EM CONSERVA |
| 04604 | CUMARI IN NATURA |
| 01802 | CUMINHO EM GRAO |
| 05202 | CUMINHO EM PO |
| 02403 | CUNQUATE |
| 02601 | CUPIM |
| 02602 | CUPIM BOVINO |
| 05201 | CUPINICIDA |
| 05201 | CUPINICIDA |
| 07301 | CUPUACU |
| 03502 | CURANDEIRO E CURIOSO |
| 03501 | CURANDEIRO, CURIOSO E PARTEIRA |
| 00405 | CURATIVO |
| 04325 | CURAU DE MILHO |
| 01904 | CURAU PARA VIAGEM |
| 03503 | CURIOSO |
| 00238 | CURRICULUM VITAE |
| 04601 | CURRY |
| 04404 | CURSO DE ALEMAO |
| 08301 | CURSO DE ARTES |
| 07801 | CURSO DE ARTESANATO |
| 08002 | CURSO DE ATENDIMENTO AO CLIENTE |
| 03602 | CURSO DE BALE |
| 05602 | CURSO DE BORDADO |
| 05501 | CURSO DE CABELEIREIRO |
| 05601 | CURSO DE CORTE E COSTURA |
| 06501 | CURSO DE CULINARIA |
| 06601 | CURSO DE DANCA |
| 03501 | CURSO DE DATILOGRAFIA |
| 08402 | CURSO DE DEPILACAO |
| 06701 | CURSO DE DESENHO |
| 07405 | CURSO DE DIRECAO DE AVIAO |
| 05405 | CURSO DE DIRECAO DE CAMINHAO |
| 05402 | CURSO DE DIRECAO DE CARRO |
| 07403 | CURSO DE DIRECAO DE METRO |
| 05403 | CURSO DE DIRECAO DE MOTO |
| 07404 | CURSO DE DIRECAO DE NAVIO |
| 05404 | CURSO DE DIRECAO DE ONIBUS |
| 07401 | CURSO DE DIRECAO DE OUTROS VEICULOS |
| 07402 | CURSO DE DIRECAO DE TREM |
| 04103 | CURSO DE DOUTORADO |
| 06801 | CURSO DE ELETRICISTA |
| 06901 | CURSO DE ENFERMAGEM |
| 04402 | CURSO DE ESPANHOL |
| 01503 | CURSO DE ESPECIALIZACAO |
| 08401 | CURSO DE ESTETICISTA |
| 04407 | CURSO DE FRANCES |
| 04401 | CURSO DE IDIOMA (EXCETO LINGUAFONE) |
| 03901 | CURSO DE INFORMATICA |
| 04403 | CURSO DE INGLES |
| 03702 | CURSO DE IOGA |
| 04405 | CURSO DE ITALIANO |
| 04406 | CURSO DE JAPONES |
| 08101 | CURSO DE MANICURE E PEDICURE |
| 08601 | CURSO DE MASSOTERAPIA (MASSAGEM) |
| 07901 | CURSO DE MECANICA DE VEICULOS |
| 04701 | CURSO DE MECANICA EM REFRIGERACAO |
| 02203 | CURSO DE MESTRADO |
| 08201 | CURSO DE MONTAGEM E MANUTENCAO DE COMPUTADORES |
| 03801 | CURSO DE MUSICA |
| 00107 | CURSO DE NOIVOS |
| 07001 | CURSO DE PINTURA |
| 04101 | CURSO DE POS-GRADUACAO (DOUTORADO) |
| 01501 | CURSO DE POS-GRADUACAO (ESPECIALIZACAO) |
| 02201 | CURSO DE POS-GRADUACAO (MESTRADO) |
| 04901 | CURSO DE PRIMEIROS SOCORROS |
| 08701 | CURSO DE PROMOTOR DE VENDAS |
| 07101 | CURSO DE SECRETARIADO |
| 07201 | CURSO DE TEATRO |
| 08001 | CURSO DE TELEMARKETING |
| 07301 | CURSO DE VIGILANTE |
| 03601 | CURSO E AULA DE BALE |
| 03701 | CURSO E AULA DE IOGA |
| 00403 | CURSO EM DISCO |
| 00401 | CURSO EM DISCO OU FITA |
| 00402 | CURSO EM FITA CASSETE |
| 09201 | CURSO NAO-ESPECIFICADO |
| 05001 | CURSO PARA EXCEPCIONAL |
| 00101 | CURSO PRE-ESCOLAR |
| 07601 | CURSO PREPARATORIO PARA CONCURSO |
| 08901 | CURSO PREPARATORIO PARA ESCOLA MILITAR |
| 08801 | CURSO PREPARATORIO PARA OAB |
| 05201 | CURSO PRE-TECNICO |
| 05101 | CURSO PRE-VESTIBULAR |
| 03102 | CURSO REGULAR DE 1º GRAU |
| 03202 | CURSO REGULAR DE 2º GRAU |
| 03302 | CURSO REGULAR DE 3º GRAU |
| 03101 | CURSO REGULAR DE PRIMEIRO GRAU |
| 03201 | CURSO REGULAR DE SEGUNDO GRAU |
| 03301 | CURSO REGULAR DE TERCEIRO GRAU |
| 00201 | CURSO SUPLETIVO |
| 07501 | CURSO TECNICO |
| 09001 | CURSOS DIVERSOS (MEDITACAO, ORATORIA, ETC.) |
| 07701 | CURSOS RELIGIOSOS |
| 04322 | CUSCUZ |
| 02901 | CUSCUZ |
| 02906 | CUSCUZ DE ARROZ |
| 02903 | CUSCUZ DE CARIMA |
| 02907 | CUSCUZ DE FUBA |
| 02905 | CUSCUZ DE GOMA |
| 02904 | CUSCUZ DE MANDIOCA |
| 02908 | CUSCUZ DE MILHO |
| 02902 | CUSCUZ DE TAPIOCA |
| 05801 | CUSCUZ PAULISTA PARA VIAGEM |
| 06701 | CUSCUZEIRA |
| 06708 | CUTIA VIVA |
| 01906 | CUXA |
| 00208 | DAMA |
| 02415 | DAMASCO DESIDRATADO |
| 02406 | DAMASCO SECO OU DESIDRATADO |
| 05901 | DANCA CLASSICA (INGRESSO) |
| 02002 | DANCETERIA (INGRESSO) |
| 02610 | DANCETERIAS (INGRESSO) |
| 02607 | DANETTE PUDIM |
| 10907 | DANETTE PUDIM DIET |
| 10915 | DANETTE PUDIM DIETETICO |
| 10807 | DANETTE PUDIM LIGHT |
| 01204 | DANONE IOGURTE |
| 04004 | DANONE IOGURTE DIET |
| 04008 | DANONE IOGURTE DIETETICO |
| 03905 | DANONE IOGURTE LIGHT |
| 04805 | DANONE IOGURTE ORGANICO |
| 01209 | DANONETE |
| 01205 | DANONINHO |
| 01002 | DATILOGRAFIA COMPUTADORIZADA |
| 01001 | DATILOGRAFO |
| 15607 | DEBULHADOR |
| 00804 | DECALQUES |
| 03701 | DECIMO TERCEIRO SALARIO |
| 00512 | DECLARACAO ESCOLAR |
| 00501 | DECORADOR |
| 00116 | DEDAL |
| 01401 | DEDETIZACAO, DESRATIZACAO, DESINSETIZACAO, ETC. |
| 01401 | DEDETIZACAO, DESRATIZACAO, DESINSETIZACAO, ETC. |
| 02314 | DEDITOS (BISCOITO) |
| 10514 | DEDITOS (BISCOITO) DIET |
| 10554 | DEDITOS (BISCOITO) DIETETICO |
| 10414 | DEDITOS (BISCOITO) LIGHT |
| 01601 | DEFESA DO CONSUMIDOR (IDEC) |
| 00409 | DELINEADOR |
| 00302 | DEMERARA |
| 08802 | DEMERARA ORGANICO |
| 01303 | DENDE |
| 01403 | DENSITOMETRIA OSSEA |
| 02304 | DENTADURA |
| 00801 | DEPILACAO |
| 05301 | DEPILADOR ELETRICO |
| 00203 | DEPOSITO DE APLICACAO FINANCEIRA (CDB, RDB, COMMODITIES, ETC.) |
| 00503 | DEPOSITO DE LOCACAO DE OUTROS IMOVEIS |
| 00304 | DEPOSITOS DE CONSERVACAO DO IMOVEL |
| 00303 | DEPOSITOS DE LOCACAO DO IMOVEL |
| 30006 | DEPURADOR DO SANGUE |
| 30005 | DEPURADOR SANGUINEO |
| 30007 | DEPURATIVO DO SANGUE |
| 30004 | DEPURATIVO SANGUINEO |
| 02602 | DERMATOLOGICO |
| 00802 | DESCOLORACAO DE PELOS (BANHO DE LUA) |
| 02616 | DESCOLORANTE PARA CABELO |
| 03608 | DESCONGESTIONANTE NASAL |
| 03609 | DESCONGESTIONANTE NASAL HOMEOPATICO |
| 03304 | DESCONTO ALIMENTACAO |
| 03303 | DESCONTO DE ALIMENTACAO (ALIMENTACAO SUBSIDIADA) |
| 03602 | DESCONTO DE AUXILIO-EDUCACAO |
| 03302 | DESCONTO DE CESTA BASICA |
| 03403 | DESCONTO DE TRANSPORTE (TRANSPORTE SUBSIDIADO) |
| 03402 | DESCONTO TRANSPORTE |
| 01703 | DESEMBARACO DE DOCUMENTACAO PARA OBRA |
| 01703 | DESEMBARACO DE DOCUMENTACAO PARA OBRA |
| 01648 | DESEMPENADEIRA |
| 01804 | DESENGORDURANTE |
| 00401 | DESENHISTA |
| 04002 | DESENTUPIDOR DE PIA |
| 04001 | DESENTUPIDOR DE RALO |
| 01402 | DESFILE DE ESCOLA DE SAMBA |
| 01401 | DESINFETANTE |
| 30203 | DESINFETANTE FARMACEUTICO |
| 01404 | DESINSETIZACAO |
| 01404 | DESINSETIZACAO |
| 02501 | DESODORANTE AMBIENTAL |
| 00503 | DESODORANTE ANTISSEPTICO |
| 00502 | DESODORANTE ANTITRANSPIRANTE |
| 01502 | DESODORANTE BUCAL |
| 02508 | DESODORANTE DE GELADEIRA |
| 02504 | DESODORANTE DE SANITARIO |
| 03101 | DESODORANTE ESPECIAL PARA OS PES |
| 00501 | DESODORANTE PESSOAL |
| 02507 | DESODORIZADOR DE GELADEIRA |
| 02514 | DESODORIZADOR SANITARIO |
| 00301 | DESPACHANTE |
| 04201 | DESPESAS OPCIONAIS COM AGENCIAS DE VIAGEM |
| 01402 | DESRATIZACAO |
| 01402 | DESRATIZACAO |
| 06402 | DESUMIDIFICADOR |
| 01801 | DETERGENTE |
| 01806 | DETERGENTE EM PO |
| 01807 | DETERGENTE LIQUIDO |
| 03302 | DEVOLUCAO DA PREVIDENCIA PRIVADA |
| 02302 | DEVOLUCAO DA PREVIDENCIA PUBLICA |
| 05102 | DEVOLUCAO DE CONVENIO DE SAUDE |
| 05202 | DEVOLUCAO DE MULTA DE TRANSITO |
| 03002 | DEVOLUCAO DE PENSAO ALIMENTICIA |
| 05104 | DEVOLUCAO DE PLANO DE SAUDE |
| 03405 | DEVOLUCAO DE SEGURO-HABITACAO |
| 03406 | DEVOLUCAO DE TAXA DE CONSERVACAO DE IMOVEL |
| 03407 | DEVOLUCAO DE TAXA DE ESGOTO (CIA DE SANEAMENTO ESTADUAL) |
| 00402 | DEVOLUCAO DO IMPOSTO DE RENDA |
| 02702 | DEVOLUCAO JUDICIAL |
| 08301 | DEXTROSOL |
| 01602 | DIABO VERDE |
| 02201 | DIALISE |
| 05608 | DIANTEIRO DE CARNEIRO |
| 00805 | DICIONARIO |
| 06501 | DIET SHAKE |
| 03901 | DIFERENCA SALARIAL |
| 02702 | DIGESTIVO |
| 01501 | DIGITACAO, IMPRESSAO |
| 04802 | DINDIM (PICOLE ENSACADO) |
| 11402 | DINDIM (PICOLE ENSACADO) LIGHT |
| 04504 | DINDIM (SACOLE) |
| 03201 | DINHEIRO ACHADO |
| 03101 | DINHEIRO ROUBADO |
| 00509 | DIPLOMA |
| 00904 | DIREITOS AUTORAIS |
| 03801 | DISCMAN |
| 01655 | DISCO DE SERRA |
| 00601 | DISCO DE VINIL |
| 04002 | DISCO LASER |
| 02003 | DISCOTECA (INGRESSO) |
| 02609 | DISCOTECAS (INGRESSO) |
| 03802 | DISKMAN |
| 04801 | DISQUETE PARA COMPUTADOR |
| 31006 | DIU |
| 02403 | DIURETICO |
| 01501 | DIVIDENDOS DE ACOES (DINHEIRO) |
| 03301 | DIVISORIA (QUARTO ADULTO) |
| 04401 | DIZIMO PARA IGREJA |
| 03601 | DJEMBE |
| 03202 | DOACAO DE NAO MORADOR |
| 03201 | DOACAO DE NAO-MORADOR |
| 00204 | DOACAO EM DINHEIRO PARA INSTITUICAO DE CARIDADE |
| 00404 | DOACAO EM DINHEIRO PARA OUTRA UC |
| 00203 | DOACAO PARA MUSEU EM DINHEIRO |
| 00201 | DOACOES EM DINHEIRO (IGREJA, ORFANATO, INSTITUICAO DE CARIDADE, MUSEU, ETC.) |
| 02002 | DOBRADINHA COM FEIJAO BRANCO EM CONSERVA |
| 02402 | DOBRADINHA FRESCA |
| 09501 | DOBRADINHA PARA VIAGEM |
| 06401 | DOBRADINHA SALGADA |
| 02602 | DOC PESSOAL (TRANSFERENCIA INTERBANCARIA) |
| 04302 | DOCE |
| 07702 | DOCE |
| 04201 | DOCE A BASE DE LEITE |
| 07614 | DOCE A BASE DE LEITE DIET |
| 13801 | DOCE A BASE DE LEITE DIET |
| 07615 | DOCE A BASE DE LEITE DIETETICO |
| 13806 | DOCE A BASE DE LEITE DIETETICO |
| 04101 | DOCE A BASE DE OVOS |
| 07612 | DOCE A BASE DE OVOS DIET |
| 13701 | DOCE A BASE DE OVOS DIET |
| 07613 | DOCE A BASE DE OVOS DIETETICO |
| 13709 | DOCE A BASE DE OVOS DIETETICO |
| 04901 | DOCE CONFEITADO DE QUALQUER TIPO |
| 11601 | DOCE CONFEITADO DE QUALQUER TIPO DIET |
| 11602 | DOCE CONFEITADO DE QUALQUER TIPO DIETETICO |
| 11501 | DOCE CONFEITADO DE QUALQUER TIPO LIGHT |
| 01402 | DOCE DE ABACAXI CRISTALIZADO |
| 03401 | DOCE DE ABOBORA |
| 01237 | DOCE DE ABOBORA COM COCO |
| 13201 | DOCE DE ABOBORA DIET |
| 13202 | DOCE DE ABOBORA DIETETICO |
| 01212 | DOCE DE ABOBORA EM PASTA |
| 01217 | DOCE DE AMEIXA EM PASTA |
| 03201 | DOCE DE AMENDOIM |
| 13101 | DOCE DE AMENDOIM DIET |
| 13110 | DOCE DE AMENDOIM DIETETICO |
| 01407 | DOCE DE BANANA CRISTALIZADO |
| 09309 | DOCE DE BANANA CRISTALIZADO DIET |
| 09310 | DOCE DE BANANA CRISTALIZADO DIETETICO |
| 01231 | DOCE DE BANANA EM PASTA OU BARRA |
| 03501 | DOCE DE BATATA DOCE |
| 13301 | DOCE DE BATATA DOCE DIET |
| 13302 | DOCE DE BATATA DOCE DIETETICO |
| 01216 | DOCE DE BATATA MARROM GLACE |
| 01233 | DOCE DE BURITI EM PASTA OU BARRA |
| 01219 | DOCE DE CACAU EM PASTA |
| 01406 | DOCE DE CAJU CRISTALIZADO |
| 09307 | DOCE DE CAJU CRISTALIZADO DIET |
| 09308 | DOCE DE CAJU CRISTALIZADO DIETETICO |
| 01234 | DOCE DE CAJU EM BARRA |
| 01223 | DOCE DE CAJU EM PASTA OU BARRA |
| 01215 | DOCE DE CEREJA EM PASTA |
| 03602 | DOCE DE CHOCOLATE |
| 01232 | DOCE DE COCO EM PASTA OU BARRA |
| 01225 | DOCE DE FEIJAO EM PASTA |
| 01404 | DOCE DE FIGO CRISTALIZADO |
| 06907 | DOCE DE FRUTA CRISTALIZADO DIET |
| 06908 | DOCE DE FRUTA CRISTALIZADO DIETETICO |
| 13804 | DOCE DE FRUTA CRISTALIZADO LIGHT |
| 06903 | DOCE DE FRUTA EM CALDA DIET |
| 06904 | DOCE DE FRUTA EM CALDA DIETETICO |
| 13802 | DOCE DE FRUTA EM CALDA LIGHT |
| 06901 | DOCE DE FRUTA EM CALDA, EM PASTA OU CRISTALIZADO DIET |
| 06902 | DOCE DE FRUTA EM CALDA, EM PASTA OU CRISTALIZADO DIETETICO |
| 13801 | DOCE DE FRUTA EM CALDA, EM PASTA OU CRISTALIZADO LIGHT |
| 06905 | DOCE DE FRUTA EM PASTA DIET |
| 06906 | DOCE DE FRUTA EM PASTA DIETETICO |
| 13803 | DOCE DE FRUTA EM PASTA LIGHT |
| 01401 | DOCE DE FRUTAS CRISTALIZADO DE QUALQUER SABOR |
| 09301 | DOCE DE FRUTAS CRISTALIZADO DE QUALQUER SABOR DIET |
| 09302 | DOCE DE FRUTAS CRISTALIZADO DE QUALQUER SABOR DIETETICO |
| 09303 | DOCE DE FRUTAS CRISTALIZADO DIET |
| 09304 | DOCE DE FRUTAS CRISTALIZADO DIETETICO |
| 08006 | DOCE DE FRUTAS EM BARRA DIET |
| 08004 | DOCE DE FRUTAS EM BARRA DIETETICO |
| 10703 | DOCE DE FRUTAS EM BARRA LIGHT |
| 08001 | DOCE DE FRUTAS EM BARRA OU PASTA DIET |
| 08002 | DOCE DE FRUTAS EM BARRA OU PASTA DIETETICO |
| 10701 | DOCE DE FRUTAS EM BARRA OU PASTA LIGHT |
| 01207 | DOCE DE FRUTAS EM BARRA OU TIJOLO DE QUALQUER SABOR |
| 01301 | DOCE DE FRUTAS EM CALDA DE QUALQUER SABOR |
| 09201 | DOCE DE FRUTAS EM CALDA DIET |
| 09202 | DOCE DE FRUTAS EM CALDA DIETETICO |
| 01201 | DOCE DE FRUTAS EM PASTA DE QUALQUER SABOR |
| 08005 | DOCE DE FRUTAS EM PASTA DIET |
| 08003 | DOCE DE FRUTAS EM PASTA DIETETICO |
| 10702 | DOCE DE FRUTAS EM PASTA LIGHT |
| 01222 | DOCE DE FRUTAS EM TABLETE DE QUALQUER SABOR |
| 14301 | DOCE DE GERGELIM |
| 01405 | DOCE DE GOIABA CRISTALIZADO |
| 09305 | DOCE DE GOIABA CRISTALIZADO DIET |
| 09306 | DOCE DE GOIABA CRISTALIZADO DIETETICO |
| 01235 | DOCE DE GOIABA EM PASTA OU BARRA |
| 01312 | DOCE DE JACA EM CALDA |
| 01403 | DOCE DE LARANJA CRISTALIZADO |
| 04301 | DOCE DE LEITE |
| 04303 | DOCE DE LEITE COM AMEIXA |
| 04304 | DOCE DE LEITE COM AMENDOIM |
| 04305 | DOCE DE LEITE COM COCO |
| 11301 | DOCE DE LEITE DIET |
| 11303 | DOCE DE LEITE DIETETICO |
| 11201 | DOCE DE LEITE LIGHT |
| 01228 | DOCE DE MAMAO EM PASTA |
| 01227 | DOCE DE MANDIOCA EM PASTA |
| 01230 | DOCE DE MILHO VERDE EM PASTA |
| 01229 | DOCE DE MINGAU DE MILHO VERDE EM PASTA |
| 01213 | DOCE DE MORANGO EM PASTA |
| 04106 | DOCE DE OVOS |
| 04102 | DOCE DE OVOS COM AMENDOA |
| 13702 | DOCE DE OVOS COM AMENDOA DIET |
| 13710 | DOCE DE OVOS COM AMENDOA DIETETICO |
| 13706 | DOCE DE OVOS DIET |
| 13714 | DOCE DE OVOS DIETETICO |
| 01218 | DOCE DE PERA EM PASTA |
| 01313 | DOCE DE PESSEGO EM CALDA |
| 09203 | DOCE DE PESSEGO EM CALDA DIET |
| 09204 | DOCE DE PESSEGO EM CALDA DIETETICO |
| 04001 | DOCE DE QUEIJO |
| 07610 | DOCE DE QUEIJO DIET |
| 13601 | DOCE DE QUEIJO DIET |
| 07611 | DOCE DE QUEIJO DIETETICO |
| 13602 | DOCE DE QUEIJO DIETETICO |
| 01238 | DOCE DE TAMARINDO (EM PASTA) |
| 01236 | DOCE DE UVA EM PASTA OU BARRA |
| 09502 | DOCE DIET NAO-ESPECIFICADO |
| 09504 | DOCE DIETETICO NAO-ESPECIFICADO |
| 02502 | DOCE INFANTIL EM POTE DE QUALQUER MARCA E SABOR |
| 09402 | DOCE LIGHT NAO-ESPECIFICADO |
| 07701 | DOCE NAO-ESPECIFICADO |
| 09501 | DOCE NAO-ESPECIFICADO DIET |
| 09503 | DOCE NAO-ESPECIFICADO DIETETICO |
| 09401 | DOCE NAO-ESPECIFICADO LIGHT |
| 04301 | DOCE OU BOLO |
| 07601 | DOCE OU BOLO DIETETICO |
| 09601 | DOCE OU BOLO LIGHT |
| 06301 | DOCE SIRIO |
| 05902 | DOCE TORRONE |
| 01802 | DOCUMENTACAO |
| 00704 | DOCUMENTACAO (EXCETO DE HABILITACAO E DE VEICULO) |
| 00506 | DOCUMENTACAO ESCOLAR (EMOLUMENTO) |
| 00909 | DOCUMENTO UNICO DE TRANSITO |
| 02102 | DORMITORIO COMPLETO (ARMARIO, CAMA, ETC.) |
| 02101 | DORMITORIO COMPLETO (ARMARIO, CAMA, MESINHA, ETC.) |
| 00706 | DORMITORIOS |
| 00603 | DORSO DE FRANGO |
| 00601 | DORSO DE GALINHA OU FRANGO |
| 03911 | DORSO DE PATO |
| 02001 | DORSO DE PERU |
| 07013 | DOURADINHA FALSA |
| 04102 | DOUTORADO |
| 00903 | DRENAGEM LINFATICA |
| 01701 | DRINK DREHER |
| 00605 | DROPS |
| 09905 | DROPS DIET |
| 09918 | DROPS DIETETICO |
| 09805 | DROPS LIGHT |
| 00706 | DRUMETE DE FRANGO |
| 00702 | DRUMETE DE GALINHA OU FRANGO |
| 04903 | DUCHA ELETRICA |
| 00303 | DUELO DE CANA COM SABOR DE FRUTA |
| 03703 | DUELO DE CANA COM SABOR DE FRUTA ORGANICO |
| 01701 | DUPLA SENA |
| 04601 | DUREPOX |
| 04601 | DUREPOX |
| 00910 | DUT (DOCUMENTO UNICO DE TRANSITO) |
| 02701 | DVD |
| 01701 | DVD (ALUGUEL) |
| 05701 | DVD (DISCO) |
| 00835 | DVD PARA CARRO |
| 06312 | DVDROM |
| 06311 | DVD-ROM |
| 12001 | EASY BOX (APARELHO NAVEGADOR DE INTERNET) |
| 06602 | EASY OFF |
| 00107 | ECHARPE |
| 01605 | ECOCARDIOGRAFIA |
| 01604 | ECOCARDIOGRAMA |
| 01606 | ECOENDOSCOPIA |
| 01603 | ECOGRAFIA |
| 04501 | ECTOPARASITOSE (SARNA, PIOLHO, ETC.) |
| 00902 | EDICAO DE FITA |
| 00407 | EDREDON |
| 05701 | EDUCACAO A DISTANCIA (CURSOS) |
| 00411 | EGGSBURGUER |
| 00305 | EGGSBURGUER PARA VIAGEM |
| 14303 | EGOISTA (FONE DE OUVIDO) |
| 01210 | EIXO DE BICICLETA |
| 00112 | ELASTICO |
| 01304 | ELETRICISTA |
| 01304 | ELETRICISTA |
| 01802 | ELETROCARDIOGRAMA |
| 01801 | ELETROCARDIOGRAMA E OUTROS METODOS GRAFICOS |
| 01803 | ELETROENCEFALOGRAMA |
| 01502 | ELEVADOR |
| 12102 | ELIMINADOR DE PILHAS |
| 01903 | EMAGRECEDOR |
| 04402 | EMBALAGEM ALUMINIZADA DE CONGELAMENTO DE ALIMENTOS |
| 01301 | EMBALAGEM PLASTIFICADA |
| 03201 | EMOLDURADOR DE QUADRO |
| 00507 | EMOLUMENTO (DESPESA DE DOCUMENTACAO ESCOLAR) |
| 03706 | EMPADA |
| 00206 | EMPADA PARA VIAGEM |
| 05901 | EMPADAO PARA VIAGEM |
| 00229 | EMPADINHA PARA VIAGEM |
| 04905 | EMPANADO DE FRANGO |
| 00101 | EMPLACAMENTO DE AUTOMOVEL |
| 01601 | EMPLACAMENTO DE CAMINHAO |
| 01501 | EMPLACAMENTO DE EMBARCACAO |
| 01401 | EMPLACAMENTO DE MOTO |
| 00101 | EMPREGADA |
| 00102 | EMPREGADO |
| 01604 | EMPREITADA |
| 01604 | EMPREITADA |
| 01603 | EMPREITEIRO |
| 01603 | EMPREITEIRO |
| 01401 | EMPRESTIMO |
| 01202 | EMPRESTIMO (PAGAMENTO) |
| 04501 | EMPRESTIMO A TERCEIROS |
| 01713 | EMULSAO DE ACAI |
| 01911 | EMULSAO DE BACABA |
| 02305 | EMULSAO DE BURITI EM EMULSAO |
| 02602 | EMULSAO DE PATAUA |
| 11601 | EMUSTAB (EMULSIFICANTE) |
| 03601 | ENCARGOS FINANCEIROS DE CARTAO DE CREDITO |
| 03201 | ENCARGOS FINANCEIROS DE CHEQUE ESPECIAL |
| 01102 | ENCARGOS FINANCEIROS DE FINANCIAMENTO |
| 01901 | ENCERADEIRA |
| 01101 | ENCHIMENTO DE ALMOFADA, DE TRAVESSEIRO, ETC |
| 03104 | ENCHOVA EM CONSERVA |
| 00811 | ENCICLOPEDIA |
| 00105 | ENCOMENDA |
| 00305 | ENDIVIA |
| 01901 | ENDOSCOPIA |
| 00201 | ENERGIA ELETRICA (KWH) |
| 01001 | ENERGIA ELETRICA DE OUTROS IMOVEIS (KWH) |
| 02501 | ENFEITE DE BERCO |
| 00233 | ENFEITE DE BOLO |
| 05701 | ENFEITE DE CABELO |
| 00918 | ENFEITE DE GELADEIRA DE PLASTICO |
| 03201 | ENFEITES DE ARVORE DE NATAL |
| 03203 | ENFEITES DE NATAL |
| 00226 | ENFEITES DE PAREDE DE FESTA |
| 03204 | ENFEITES DIVERSOS |
| 02502 | ENFERMEIRA |
| 02503 | ENFERMEIRA |
| 02501 | ENFERMEIRA (SERVICO DOMESTICO) |
| 02503 | ENFERMEIRO |
| 03909 | ENFORCADOR DE ANIMAL |
| 03911 | ENFORCADOR PARA ANIMAL |
| 00801 | ENGENHEIRO |
| 01602 | ENGRADADO DE MADEIRA |
| 00907 | ENGRADADO PLASTICO |
| 00701 | ENGRAXATE |
| 03727 | ENROLADINHO |
| 00227 | ENROLADINHO PARA VIAGEM |
| 00205 | ENTRADA PARA AQUISICAO DO IMOVEL A PRAZO |
| 00224 | ENVELOPE |
| 01611 | ENXADA |
| 30008 | ENXOFRE |
| 02401 | ENXOVAL (ROUPAS DE CAMA, MESA, BANHO) |
| 04601 | ENXOVAL DE BEBE |
| 04602 | EPOX (COLA) |
| 04602 | EPOX (COLA) |
| 14701 | EQUALIZADOR |
| 30907 | EQUIPAMENTO DE MASSAGEM |
| 01501 | EQUIPAMENTO DE SOM |
| 02903 | EQUIPAMENTO DE SOM |
| 01802 | EQUIPAMENTOS DE AQUARIO ORNAMENTAL |
| 00106 | EQUIPAMENTOS DE ESPORTE |
| 00119 | EQUIPAMENTOS ESPORTIVOS |
| 00101 | EQUIPAMENTOS ESPORTIVOS (BOLA, RAQUETE, REDE, TORNOZELEIRA, CHUTEIRA, ETC.) |
| 03301 | ERVA CIDREIRA CHA |
| 02805 | ERVA DE TERERE |
| 02801 | ERVA DOCE |
| 12601 | ERVA DOCE (TEMPERO INDUSTRIALIZADO) |
| 02901 | ERVA DOCE CHA |
| 02803 | ERVA MATE |
| 12803 | ERVA MATE DIET |
| 12806 | ERVA MATE DIETETICA |
| 12703 | ERVA MATE LIGHT |
| 13001 | ERVA MATE ORGANICA |
| 00301 | ERVILHA COM CENOURA EM CONSERVA |
| 05502 | ERVILHA DE VAGEM |
| 00302 | ERVILHA E CENOURA EM CONSERVA |
| 01502 | ERVILHA E MILHO EM CONSERVA |
| 01504 | ERVILHA E MILHO VERDE EM CONSERVA |
| 00201 | ERVILHA EM CONSERVA |
| 01101 | ERVILHA EM GRAO |
| 06502 | ERVILHA EM PO OU GRAO (FRITA) |
| 05501 | ERVILHA EM VAGEM |
| 06501 | ERVILHA FRITA EM PO OU GRAO |
| 15201 | ESCADA |
| 05501 | ESCADA CARACOL |
| 05501 | ESCADA CARACOL |
| 01620 | ESCALA |
| 00404 | ESCALOPE DE FRANGO |
| 00304 | ESCAROLA |
| 06801 | ESCORREDOR DE ARROZ |
| 00805 | ESCORREDOR DE METAL |
| 00214 | ESCOVA (CABELEIREIRO) |
| 01104 | ESCOVA DE BANHO |
| 00103 | ESCOVA DE CABELO |
| 00101 | ESCOVA DE CABELOS E PENTE |
| 00201 | ESCOVA DE DENTE |
| 03504 | ESCOVA DE ENGRAXAR SAPATO |
| 03503 | ESCOVA DE LAVAR ROUPA |
| 00301 | ESCOVA DE LIMPEZA |
| 00304 | ESCOVA DE MAMADEIRA |
| 03501 | ESCOVA DE ROUPA |
| 03502 | ESCOVA DE SAPATO |
| 00605 | ESCOVA DE UNHA |
| 00202 | ESCOVA DENTAL |
| 08702 | ESCOVA ELETRICA |
| 00408 | ESCOVA SANITARIA |
| 00404 | ESCOVAO |
| 00108 | ESCRITURA |
| 00202 | ESCRITURA DE OUTROS TERRENOS OU IMOVEIS |
| 08801 | ESCRIVANINHA |
| 02501 | ESCUDO DE TIME |
| 00901 | ESCULTURA |
| 06901 | ESCUMADEIRA |
| 03729 | ESFIRRA |
| 03739 | ESFIRRA DE CARNE |
| 00223 | ESFIRRA DE CARNE PARA VIAGEM |
| 03742 | ESFIRRA DE FRANGO |
| 00226 | ESFIRRA DE FRANGO PARA VIAGEM |
| 03740 | ESFIRRA DE QUEIJO |
| 00224 | ESFIRRA DE QUEIJO PARA VIAGEM |
| 03741 | ESFIRRA DE RICOTA |
| 00225 | ESFIRRA DE RICOTA PARA VIAGEM |
| 00222 | ESFIRRA PARA VIAGEM |
| 07401 | ESFREGADEIRA DE LAVAR ROUPA |
| 00406 | ESFREGAO |
| 00612 | ESMALTE |
| 00601 | ESMALTE, BASE, ACETONA, ETC. |
| 14404 | ESMERIL ELETRICO |
| 02101 | ESMOLA |
| 02102 | ESMOLET |
| 03404 | ESPAGUETE (NAO-ESPECIFICADO) |
| 03313 | ESPAGUETE COM OVOS |
| 03412 | ESPAGUETE NAO-ESPECIFICADO |
| 03213 | ESPAGUETE SEM OVOS |
| 00601 | ESPANADOR |
| 30102 | ESPARADRAPO |
| 01502 | ESPECIALIZACAO |
| 01401 | ESPELHO |
| 04101 | ESPELHO DE BANHEIRO |
| 04101 | ESPELHO DE BANHEIRO |
| 01101 | ESPELHO DE BOLSO |
| 01309 | ESPERMOGRAMA |
| 03724 | ESPETINHO DE CARNE |
| 01102 | ESPETINHO DE CARNE PARA VIAGEM |
| 01009 | ESPETINHO DE FRANGO PARA VIAGEM |
| 08501 | ESPETINHO DE QUEIJO PARA VIAGEM |
| 07604 | ESPETINHO PARA CHURRASCO |
| 07605 | ESPETO PARA CHURRASCO (MADEIRA) |
| 03732 | ESPIGA DE MILHO ASSADA OU COZIDA |
| 00701 | ESPINAFRE |
| 00702 | ESPINAFRE JAPONES |
| 08401 | ESPINAFRE ORGANICO |
| 00402 | ESPINGARDA (EXCETO DE CACA) |
| 00502 | ESPINGARDA DE CACA |
| 06301 | ESPINHACO DE PORCO SALGADO |
| 06302 | ESPINHACO SUINO SALGADO |
| 02604 | ESPIRAL DURMA BEM OU OUTRA MARCA |
| 00204 | ESPONJA DE ACO |
| 01106 | ESPONJA DE BANHO |
| 01104 | ESPONJA DE ENCHIMENTO DE ALMOFADA, DE TRAVESSEIRO, ETC. |
| 00201 | ESPONJA DE LIMPEZA |
| 00205 | ESPONJA DE NYLON |
| 02001 | ESPONJA DE TALCO |
| 08601 | ESPREGUICADEIRA |
| 01101 | ESPREMEDOR DE FRUTAS ELETRICO |
| 00835 | ESPREMEDOR DE METAL |
| 00606 | ESPUMA DE BARBEAR |
| 02501 | ESPUMA DE COLCHAO |
| 01102 | ESPUMA DE ENCHIMENTO DE ALMOFADA, DE TRAVESSEIRO, ETC. |
| 04901 | ESQUADRIA |
| 04901 | ESQUADRIA |
| 00223 | ESQUADRO |
| 00902 | ESSENCIA DE BAUNILHA |
| 02513 | ESSENCIA DE EUCALIPTO |
| 07201 | ESTACA PARA CERCA (QUALQUER MATERIAL) |
| 00801 | ESTACIONAMENTO |
| 00902 | ESTACIONAMENTO |
| 00803 | ESTACIONAMENTO EM SHOPPING |
| 00804 | ESTACIONAMENTO-METRO |
| 03302 | ESTADIOS (INGRESSO) |
| 00701 | ESTANTE |
| 07801 | ESTANTE (MOVEL INFANTIL) |
| 00704 | ESTANTE EMBUTIDO (SALA) |
| 01102 | ESTEIRA DE CAMINHAR (GINASTICA) |
| 09101 | ESTEIRA DE DORMIR |
| 01101 | ESTEIRA DE GINASTICA |
| 02104 | ESTEIRA DE PRAIA |
| 02201 | ESTEIRA MECANICA |
| 01701 | ESTEPE (APARELHO DE GINASTICA) |
| 07101 | ESTERILIZADOR |
| 00901 | ESTETICISTA |
| 02503 | ESTETOSCOPIO |
| 03204 | ESTIMULANTE DO APETITE |
| 03301 | ESTIMULANTE SEXUAL |
| 00826 | ESTOFAMENTO |
| 01901 | ESTOJO (APARELHO DE BARBEAR, SABONETEIRA, PENTE) |
| 01701 | ESTOJO (ESPELHO, ESCOVA, PENTE) |
| 01401 | ESTOJO (TALCO, SABONETE, PERFUME, ETC.) |
| 01902 | ESTOJO DE BARBEAR |
| 00708 | ESTOJO DE BARBEAR DESCARTAVEL |
| 00113 | ESTOJO DE COSTURA |
| 01601 | ESTOJO DE MANICURO |
| 00205 | ESTOJO ESCOLAR |
| 02302 | ESTOMAGO BOVINO |
| 07203 | ESTOMAGO DE CARNEIRO |
| 08001 | ESTOMAGO NAO-ESPECIFICADO |
| 04402 | ESTOMAGO SUINO |
| 00813 | ESTOPA |
| 06901 | ESTOPA DE LIMPEZA DA CASA |
| 10401 | ESTUFA ELETRICA |
| 30212 | ETER |
| 00235 | ETIQUETA |
| 01805 | EXAME AUDIOMETRICO |
| 01302 | EXAME DE BACILO |
| 01607 | EXAME DE CATETERISMO |
| 01303 | EXAME DE FEZES |
| 01301 | EXAME DE LABORATORIO |
| 04303 | EXAME DE PRESSAO ARTERIAL (EM POSTOS OU PROFISSIONAIS VOLANTES) |
| 01304 | EXAME DE SANGUE |
| 01305 | EXAME DE URINA |
| 01804 | EXAME ERGOMETRICO |
| 01104 | EXAME GINECOLOGICO |
| 00915 | EXAME PARA RENOVACAO DE CARTEIRA DE MOTORISTA |
| 01102 | EXAME PRE-NATAL |
| 01306 | EXAME PRE-NUPCIAL |
| 01105 | EXAME PREVENTIVO DE CANCER |
| 00916 | EXAME PSICOTECNICO DE CARTEIRA DE MOTORISTA |
| 01702 | EXAME RADIOLOGICO |
| 01308 | EXAMES EM GERAL |
| 04301 | EXAMES EM POSTOS OU PROFISSIONAIS VOLANTES |
| 01103 | EXAMES PREVENTIVOS |
| 00101 | EXAUSTOR |
| 00801 | EXCURSAO (EXCETO ESCOLAR) |
| 01601 | EXCURSAO ESCOLAR |
| 00207 | EXPECTORANTE (XAROPE) |
| 00905 | EXPLORACAO DE PATENTES |
| 01801 | EXPOSICAO OU FEIRA (INGRESSO) |
| 03901 | EXTENSAO DE FERRO ELETRICO (ESPECIFICO) |
| 01403 | EXTERMINIO DE INSETOS |
| 01403 | EXTERMINIO DE INSETOS |
| 02404 | EXTRACAO DENTARIA |
| 02401 | EXTRATO BANCARIO |
| 08102 | EXTRATO DE ALHO |
| 02104 | EXTRATO DE CEREAIS |
| 03502 | EXTRATO DE SOJA EM PO |
| 04702 | EXTRATO DE TOMATE |
| 03803 | EXTRATOS DE QUALQUER TIPO |
| 07401 | EXUMACAO |
| 00310 | EXUMACAO |
| 00702 | FACA |
| 06203 | FACA DE PLASTICO |
| 01201 | FACA ELETRICA |
| 00808 | FACAO |
| 01656 | FACAO (FERRAMENTA) |
| 06601 | FAGOTINI |
| 01504 | FAISCA |
| 06101 | FAIXA DE CABECA |
| 03301 | FAIXA DE SMOKING |
| 07014 | FALSA DOURADINHA |
| 09401 | FAMILIA FELIZ PARA VIAGEM (COMIDA JAPONESA) |
| 06702 | FANTA CITRUS |
| 13524 | FANTA CITRUS LIGHT |
| 09402 | FANTA CITRUS LIGHT |
| 00202 | FANTA LARANJA |
| 13003 | FANTA LARANJA LIGHT |
| 01502 | FANTA LARANJA LIGHT |
| 13305 | FANTA LIMAO LIGHT |
| 04103 | FANTA MACA |
| 13203 | FANTA MACA LIGHT |
| 04003 | FANTA MACA LIGHT |
| 00505 | FANTA UVA |
| 13403 | FANTA UVA LIGHT |
| 07705 | FANTA UVA LIGHT |
| 02501 | FANTASIA |
| 02901 | FANTASIA |
| 03201 | FANTASIA |
| 13701 | FAQUEIRO |
| 01901 | FARDAMENTO MILITAR |
| 05601 | FARDAMENTO MILITAR |
| 01303 | FARELO DE ARROZ PARA ANIMAL NAO-ESPECIFICADO |
| 01304 | FARELO DE MILHO PARA OUTRO ANIMAL NAO-ESPECIFICADO |
| 01302 | FARELO DE SOJA PARA ANIMAL NAO-ESPECIFICADO |
| 00905 | FARELO DE TRIGO (QUIRERA) |
| 01310 | FARELO DE TRIGO PARA ANIMAL NAO ESPECIFICADO |
| 00405 | FARELO DE TRIGO PARA BOVINO |
| 00618 | FARINHA AMARELA |
| 01511 | FARINHA BEIJU |
| 01415 | FARINHA DE AGUA |
| 03701 | FARINHA DE AMENDOIM |
| 01701 | FARINHA DE ARARUTA |
| 00103 | FARINHA DE ARROZ |
| 00301 | FARINHA DE AVEIA |
| 04001 | FARINHA DE BANANA |
| 01412 | FARINHA DE CARIMA |
| 00501 | FARINHA DE CENTEIO |
| 05002 | FARINHA DE CEREAIS VITAMINADA |
| 01410 | FARINHA DE COPIOBA |
| 03901 | FARINHA DE FEIJAO |
| 00615 | FARINHA DE FUBA |
| 01517 | FARINHA DE GOMA |
| 06801 | FARINHA DE LINHACA |
| 03802 | FARINHA DE MANDI |
| 01401 | FARINHA DE MANDIOCA |
| 01406 | FARINHA DE MANDIOCA AMARELA |
| 01404 | FARINHA DE MANDIOCA BIJU |
| 01407 | FARINHA DE MANDIOCA BRANCA |
| 01409 | FARINHA DE MANDIOCA COMUM |
| 01402 | FARINHA DE MANDIOCA CRUA |
| 01419 | FARINHA DE MANDIOCA FLOCADA |
| 01408 | FARINHA DE MANDIOCA MISTURADA |
| 01405 | FARINHA DE MANDIOCA MORENA |
| 01418 | FARINHA DE MANDIOCA TEMPERADA |
| 01403 | FARINHA DE MANDIOCA TORRADA |
| 07101 | FARINHA DE MARACUJA |
| 01411 | FARINHA DE MESA |
| 00603 | FARINHA DE MILHO |
| 03801 | FARINHA DE PEIXE |
| 01518 | FARINHA DE PUBA |
| 01201 | FARINHA DE QUIBE |
| 01101 | FARINHA DE ROSCA |
| 01901 | FARINHA DE SOJA |
| 01413 | FARINHA DE SURUI |
| 01510 | FARINHA DE TAPIOCA |
| 01001 | FARINHA DE TRIGO |
| 01004 | FARINHA DE TRIGO COM FERMENTO |
| 01202 | FARINHA DE TRIGO DE QUIBE |
| 01006 | FARINHA DE TRIGO INTEGRAL |
| 01002 | FARINHA DO REINO |
| 02001 | FARINHA LACTEA |
| 01601 | FARINHA NAO-ESPECIFICADA |
| 01417 | FARINHA SECA |
| 05001 | FARINHA VITAMINADA |
| 07001 | FARINHEIRA |
| 06001 | FAROFA DE BANANA PARA VIAGEM |
| 02201 | FAROFA PARA VIAGEM |
| 02901 | FAROFA PRONTA EM PACOTE PARA VIAGEM |
| 08101 | FAROFA PRONTA LIGHT EM PACOTE PARA VIAGEM |
| 00806 | FAROL |
| 00403 | FASCICULO |
| 02504 | FATIA DE BOLO DE QUALQUER SABOR |
| 10304 | FATIA DE BOLO DE QUALQUER SABOR DIET |
| 10308 | FATIA DE BOLO DE QUALQUER SABOR DIETETICO |
| 10204 | FATIA DE BOLO DE QUALQUER SABOR LIGHT |
| 10501 | FATIADOR DE ALIMENTOS |
| 02403 | FATO BOVINO |
| 02404 | FATO CAPRINO |
| 04502 | FATO SUINO |
| 01202 | FAVA DE LIMA EM GRAO |
| 01201 | FAVA EM GRAO |
| 06601 | FAVA FRITA EM PO OU GRAO |
| 06602 | FAVA PO OU GRAO (FRITA) |
| 06702 | FAX (APARELHO) |
| 00111 | FAX SIMILE |
| 00301 | FAXINEIRA |
| 00302 | FAXINEIRO |
| 01102 | FECHADURA |
| 01102 | FECHADURA |
| 00109 | FECHO |
| 01801 | FECULA DE ARARUTA |
| 06501 | FECULA DE BATATA |
| 01501 | FECULA DE MANDIOCA |
| 00611 | FECULA DE MILHO |
| 01005 | FECULA DE TRIGO |
| 03102 | FEIJAO (NAO-ESPECIFICADO) |
| 04102 | FEIJAO (NAO-ESPECIFICADO) ORGANICO |
| 02302 | FEIJAO AMARELO |
| 02102 | FEIJAO AMENDOIM |
| 01911 | FEIJAO ANAO (ROXO) |
| 02605 | FEIJAO ANDU |
| 01625 | FEIJAO BAIANO |
| 04025 | FEIJAO BAIANO ORGANICO |
| 01629 | FEIJAO BAJE PODRE (MACASSAR) |
| 04029 | FEIJAO BAJE PODRE (MACASSAR) ORGANICO |
| 01510 | FEIJAO BANDINHA PRETO |
| 03910 | FEIJAO BANDINHA PRETO ORGANICO |
| 01630 | FEIJAO BARRIGUDO (DE METRO) |
| 04030 | FEIJAO BARRIGUDO (DE METRO) ORGANICO |
| 02205 | FEIJAO BARROSINHO |
| 01508 | FEIJAO BEIRA LINHA |
| 03908 | FEIJAO BEIRA LINHA ORGANICO |
| 01302 | FEIJAO BICO DE OURO |
| 01903 | FEIJAO BICO ROXO |
| 01618 | FEIJAO BOCA PRETA |
| 04018 | FEIJAO BOCA PRETA ORGANICO |
| 01309 | FEIJAO BOLINHA |
| 01504 | FEIJAO BORBAO |
| 03904 | FEIJAO BORBAO ORGANICO |
| 01904 | FEIJAO BORDO |
| 01905 | FEIJAO BRABINHO |
| 01306 | FEIJAO BRANCO |
| 02001 | FEIJAO BRANCO COM DOBRADINHA EM CONSERVA |
| 01303 | FEIJAO CAETE |
| 02507 | FEIJAO CAFE |
| 01409 | FEIJAO CAFE COM LEITE |
| 03809 | FEIJAO CAFE COM LEITE ORGANICO |
| 02502 | FEIJAO CAFEZINHO |
| 01631 | FEIJAO CANAPU |
| 04031 | FEIJAO CANAPU ORGANICO |
| 01308 | FEIJAO CANARINHO |
| 02505 | FEIJAO CAQUI |
| 02509 | FEIJAO CARA SUJA |
| 02114 | FEIJAO CARIOCA |
| 02116 | FEIJAO CARIOQUINHA |
| 02111 | FEIJAO CARNAVAL |
| 02106 | FEIJAO CASCA DE COCO |
| 02107 | FEIJAO CASCAO |
| 01610 | FEIJAO CATADOR |
| 04010 | FEIJAO CATADOR ORGANICO |
| 01632 | FEIJAO CAUPI |
| 04032 | FEIJAO CAUPI ORGANICO |
| 01511 | FEIJAO CAVALO |
| 03911 | FEIJAO CAVALO ORGANICO |
| 01412 | FEIJAO CEARENCE |
| 03812 | FEIJAO CEARENCE ORGANICO |
| 02103 | FEIJAO CHITA FINA |
| 02113 | FEIJAO CHOCOLATE |
| 02501 | FEIJAO CHUMBINHO |
| 01402 | FEIJAO COFELISTA |
| 03802 | FEIJAO COFELISTA ORGANICO |
| 01606 | FEIJAO CORUJA |
| 04006 | FEIJAO CORUJA ORGANICO |
| 02607 | FEIJAO CRISTA |
| 01633 | FEIJAO DA COLONIA |
| 04033 | FEIJAO DA COLONIA ORGANICO |
| 04601 | FEIJAO DA PRAIA |
| 01628 | FEIJAO DE ARRANCA |
| 04028 | FEIJAO DE ARRANCA ORGANICO |
| 01512 | FEIJAO DE COR |
| 03912 | FEIJAO DE COR ORGANICO |
| 01603 | FEIJAO DE CORDA |
| 04003 | FEIJAO DE CORDA ORGANICO |
| 01619 | FEIJAO DE FRADE |
| 04019 | FEIJAO DE FRADE ORGANICO |
| 01604 | FEIJAO DE METRO |
| 04004 | FEIJAO DE METRO ORGANICO |
| 01607 | FEIJAO DE MOITA |
| 04007 | FEIJAO DE MOITA ORGANICO |
| 01624 | FEIJAO DE RAMA |
| 04024 | FEIJAO DE RAMA ORGANICO |
| 01620 | FEIJAO DE VARA |
| 04020 | FEIJAO DE VARA ORGANICO |
| 01507 | FEIJAO DO MILHO |
| 03907 | FEIJAO DO MILHO ORGANICO |
| 01622 | FEIJAO DO RIO |
| 04022 | FEIJAO DO RIO ORGANICO |
| 01707 | FEIJAO DO SUL |
| 01311 | FEIJAO DOURADINHO |
| 01704 | FEIJAO ENCARNADO |
| 01706 | FEIJAO ENXOFRAO |
| 02301 | FEIJAO ENXOFRE |
| 01502 | FEIJAO ESCURINHO |
| 03902 | FEIJAO ESCURINHO ORGANICO |
| 01205 | FEIJAO FAVA EM GRAO |
| 02511 | FEIJAO FIGADO DE GALINHA |
| 01503 | FEIJAO FLORESTA NEGRA |
| 03903 | FEIJAO FLORESTA NEGRA ORGANICO |
| 01601 | FEIJAO FRADINHO |
| 04001 | FEIJAO FRADINHO ORGANICO |
| 02402 | FEIJAO FUMACA |
| 02206 | FEIJAO GANCHEIRO |
| 01705 | FEIJAO GORDO |
| 01305 | FEIJAO GRAO DE OURO |
| 02112 | FEIJAO GROSSO |
| 02604 | FEIJAO GUANDO |
| 02609 | FEIJAO GUANDU |
| 02606 | FEIJAO GUINE |
| 01612 | FEIJAO GURGUTUBA |
| 04012 | FEIJAO GURGUTUBA ORGANICO |
| 01613 | FEIJAO GURUTUBA |
| 04013 | FEIJAO GURUTUBA ORGANICO |
| 01623 | FEIJAO IBRA |
| 04023 | FEIJAO IBRA ORGANICO |
| 02512 | FEIJAO IMPERIAL |
| 01509 | FEIJAO ITALIANINHO |
| 03909 | FEIJAO ITALIANINHO ORGANICO |
| 01701 | FEIJAO JALO |
| 01609 | FEIJAO LIGEIRO |
| 04009 | FEIJAO LIGEIRO ORGANICO |
| 01307 | FEIJAO LOUCA |
| 01602 | FEIJAO MACASSAR |
| 04002 | FEIJAO MACASSAR ORGANICO |
| 01301 | FEIJAO MANTEIGA |
| 02202 | FEIJAO MARIA ROSA |
| 01407 | FEIJAO MARROM |
| 03807 | FEIJAO MARROM ORGANICO |
| 01906 | FEIJAO MARUMBE |
| 02305 | FEIJAO MINEIRO |
| 02304 | FEIJAO MINEIRO AMARELO |
| 01611 | FEIJAO MIUDO |
| 04011 | FEIJAO MIUDO ORGANICO |
| 01703 | FEIJAO MOLEQUE |
| 01408 | FEIJAO MORENINHO |
| 03808 | FEIJAO MORENINHO ORGANICO |
| 02203 | FEIJAO MOURA ROSA |
| 01404 | FEIJAO MOURO |
| 03804 | FEIJAO MOURO ORGANICO |
| 01406 | FEIJAO MULATA GORDA |
| 03806 | FEIJAO MULATA GORDA ORGANICO |
| 01401 | FEIJAO MULATINHO |
| 03801 | FEIJAO MULATINHO ORGANICO |
| 01410 | FEIJAO NAGE |
| 03810 | FEIJAO NAGE ORGANICO |
| 03101 | FEIJAO NAO-ESPECIFICADO |
| 04101 | FEIJAO NAO-ESPECIFICADO ORGANICO |
| 01411 | FEIJAO NAVEGADOR MARROM |
| 03811 | FEIJAO NAVEGADOR MARROM ORGANICO |
| 01614 | FEIJAO OLHO DE CABRA |
| 04014 | FEIJAO OLHO DE CABRA ORGANICO |
| 01621 | FEIJAO OLHO PRETO |
| 04021 | FEIJAO OLHO PRETO ORGANICO |
| 02510 | FEIJAO OPACO |
| 02506 | FEIJAO OPAQUINHO |
| 01605 | FEIJAO ORELHA DE FRADE |
| 04005 | FEIJAO ORELHA DE FRADE ORGANICO |
| 02303 | FEIJAO OURO |
| 02504 | FEIJAO PACO MINEIRO |
| 02207 | FEIJAO PAQUETA |
| 02503 | FEIJAO PAQUINHO |
| 02401 | FEIJAO PARDO |
| 01403 | FEIJAO PAULISTA |
| 03803 | FEIJAO PAULISTA ORGANICO |
| 01615 | FEIJAO PENDANGA |
| 04015 | FEIJAO PENDANGA ORGANICO |
| 01635 | FEIJAO PERUANO |
| 04035 | FEIJAO PERUANO ORGANICO |
| 01304 | FEIJAO PINGO DE OURO |
| 02109 | FEIJAO PINTADINHO |
| 02108 | FEIJAO PINTADO |
| 01616 | FEIJAO PITIUBA |
| 04016 | FEIJAO PITIUBA ORGANICO |
| 01310 | FEIJAO PITOCO |
| 08601 | FEIJAO PREPARADO PARA VIAGEM |
| 01501 | FEIJAO PRETO |
| 01513 | FEIJAO PRETO BANDINHA |
| 03913 | FEIJAO PRETO BANDINHA ORGANICO |
| 03901 | FEIJAO PRETO ORGANICO |
| 01617 | FEIJAO QUARENTA DIAS |
| 04017 | FEIJAO QUARENTA DIAS ORGANICO |
| 01608 | FEIJAO QUARENTINHA |
| 04008 | FEIJAO QUARENTINHA ORGANICO |
| 01505 | FEIJAO QUEBRANCHO |
| 03905 | FEIJAO QUEBRANCHO ORGANICO |
| 02115 | FEIJAO RAJADINHO |
| 02101 | FEIJAO RAJADO |
| 01910 | FEIJAO RAPE (ROXO) |
| 01506 | FEIJAO REBENTA NEGRO |
| 03906 | FEIJAO REBENTA NEGRO ORGANICO |
| 02110 | FEIJAO RISCADINHO |
| 01902 | FEIJAO ROCHEDO |
| 02204 | FEIJAO ROSADO |
| 02201 | FEIJAO ROSINHA |
| 01908 | FEIJAO ROXAO |
| 01907 | FEIJAO ROXINHO |
| 01901 | FEIJAO ROXO |
| 01909 | FEIJAO ROXOTI |
| 01627 | FEIJAO SEMPRE VERDE |
| 04027 | FEIJAO SEMPRE VERDE ORGANICO |
| 01405 | FEIJAO SESSENTA DIAS |
| 03805 | FEIJAO SESSENTA DIAS ORGANICO |
| 02608 | FEIJAO SETE CAMADAS |
| 03002 | FEIJAO SOJA |
| 03702 | FEIJAO SOJA ORGANICO |
| 02508 | FEIJAO TERRINHA |
| 01636 | FEIJAO TREPA PAU |
| 06101 | FEIJAO TROPEIRO PARA VIAGEM |
| 05204 | FEIJAO VAGEM |
| 01634 | FEIJAO VERDE |
| 05203 | FEIJAO VERDE EM VAGEM |
| 04034 | FEIJAO VERDE ORGANICO |
| 01702 | FEIJAO VERMELHO |
| 02104 | FEIJAO VERMELHO E BRANCO |
| 01626 | FEIJAO VINAGRE |
| 04026 | FEIJAO VINAGRE ORGANICO |
| 02105 | FEIJAO ZEBRINHA |
| 01203 | FEIJOA EM GRAO |
| 01902 | FEIJOADA COMPLETA EM CONSERVA |
| 01901 | FEIJOADA EM CONSERVA |
| 08701 | FEIJOADA PARA VIAGEM |
| 01903 | FEIJOADA PRONTA EM CONSERVA |
| 01802 | FEIRA DE INFORMATICA (INGRESSO) |
| 00701 | FEIRINHA |
| 00402 | FEITICEIRA |
| 03801 | FERIAS |
| 00602 | FERMENTO |
| 00603 | FERMENTO BIOLOGICO |
| 00601 | FERMENTO EM PO |
| 02101 | FERNET |
| 01101 | FERRAGENS (FECHADURA, FERRO, ARAME, PREGO, ETC.) |
| 01101 | FERRAGENS (FECHADURA, FERRO, ARAME, PREGO, ETC.) |
| 01601 | FERRAMENTAS EM GERAL |
| 01103 | FERRO |
| 01103 | FERRO |
| 18101 | FERRO A CARVAO |
| 18001 | FERRO A GAS |
| 01631 | FERRO DE SOLDAR |
| 01101 | FERRO ELETRICO |
| 02001 | FERRO ELETRICO |
| 00509 | FERRY-BOAT |
| 01202 | FERRY-BOAT |
| 01601 | FESTA COMUNITARIA |
| 01301 | FESTA DE ANO NOVO |
| 01301 | FESTA ESCOLAR |
| 01701 | FESTA JUNINA (INGRESSO) |
| 08008 | FETUTINE PRONTO LIGHT PARA VIAGEM |
| 00908 | FETUTINE PRONTO PARA VIAGEM |
| 02702 | FIAMBRADA SUINA |
| 02707 | FIAMBRE |
| 05101 | FIAMBRE DE CHESTER |
| 06501 | FIAMBRE DE CHESTER LIGHT |
| 00901 | FIANCA (PRESO) |
| 00506 | FIANCA DE OUTROS IMOVEIS |
| 01304 | FIBRA DE CEREAL TRIGO |
| 02501 | FIBRA DE SOJA |
| 01303 | FIBRA DE TRIGO |
| 00508 | FICHA MODELO 18 E 19 |
| 00202 | FICHA TELEFONICA |
| 00247 | FICHARIO |
| 00246 | FICHARIO DE CARTAS |
| 01209 | FIGADA |
| 00402 | FIGADO ALEMAO |
| 02501 | FIGADO BOVINO |
| 02502 | FIGADO DE BOI |
| 07610 | FIGADO DE CARNEIRO |
| 01302 | FIGADO DE FRANGO |
| 01301 | FIGADO DE GALINHA OU FRANGO |
| 04601 | FIGADO SUINO |
| 02901 | FIGO |
| 02902 | FIGO DA EUROPA |
| 02903 | FIGO DE VALINHOS |
| 02418 | FIGO DESIDRATADO |
| 01302 | FIGO EM CALDA |
| 09205 | FIGO EM CALDA DIET |
| 09206 | FIGO EM CALDA DIETETICO |
| 02409 | FIGO SECO OU DESIDRATADO |
| 01702 | FIGURINHAS DE ALBUM |
| 00103 | FILE COM MIGNON |
| 11303 | FILE COM MIGNON ORGANICO |
| 00202 | FILE CURTO |
| 11402 | FILE CURTO ORGANICO |
| 00402 | FILE DE FRANGO |
| 04903 | FILE DE FRANGO EMPANADO |
| 05702 | FILE DE FRANGO ORGANICO |
| 05703 | FILE DE GALINHA OU FRANGO ORGANICO |
| 00405 | FILE DE PEITO DE FRANGO |
| 01706 | FILE DE SEGUNDA |
| 61203 | FILE DE SIRI |
| 00204 | FILE ESPECIAL |
| 11404 | FILE ESPECIAL ORGANICO |
| 00101 | FILE MIGNON |
| 11301 | FILE MIGNON ORGANICO |
| 03705 | FILE MIGNON SUINO |
| 07805 | FILE NAO-ESPECIFICADO |
| 00102 | FILE SEM MIGNON |
| 11302 | FILE SEM MIGNON ORGANICO |
| 02602 | FILHO EM MASSA PARA PASTEL |
| 03702 | FILHOS (BOLINHO DE FARINHA DE TRIGO E OVOS) |
| 07402 | FILHOS (BOLINHO DE FARINHA DE TRIGO E OVOS) DIET |
| 07404 | FILHOS (BOLINHO DE FARINHA DE TRIGO E OVOS) DIETETICO |
| 07302 | FILHOS (BOLINHO DE FARINHA DE TRIGO E OVOS) LIGHT |
| 12601 | FILMADORA |
| 01101 | FILMADORA (ALUGUEL) |
| 00901 | FILMAGEM DE CERIMONIA |
| 00701 | FILME E FLASH DESCARTAVEL |
| 01305 | FILME PLASTICO |
| 01306 | FILME PVC |
| 01803 | FILTRO BIOLOGICO DE AQUARIO ORNAMENTAL |
| 02001 | FILTRO DE AGUA |
| 14001 | FILTRO DE AGUA |
| 14002 | FILTRO DE AGUA A VELA |
| 02101 | FILTRO DE AGUA ELETRICO (ALUGUEL) |
| 04602 | FILTRO DE CAFE DE PAPEL |
| 04603 | FILTRO DE PAPEL |
| 07901 | FILTRO DE PAPEL DE EXAUSTOR |
| 10301 | FILTRO ELETRICO |
| 08801 | FILTRO PARA ASPIRADOR |
| 00520 | FILTRO SOLAR |
| 01204 | FIO DE ANTENA |
| 00102 | FIO DENTAL |
| 01201 | FIO E MATERIAL ELETRICO EM GERAL |
| 01201 | FIO E MATERIAL ELETRICO EM GERAL |
| 01203 | FIO PARA INSTALACAO ELETRICA |
| 01203 | FIO PARA INSTALACAO ELETRICA |
| 04108 | FIOS DE OVOS |
| 13708 | FIOS DE OVOS DIET |
| 13716 | FIOS DE OVOS DIETETICO |
| 01302 | FISCAL DE OBRA (PREFEITURA) |
| 01301 | FISCALIZACAO DE OBRA (PREFEITURA) |
| 01804 | FISSURA BOVINA NAO-ESPECIFICADA |
| 08106 | FISSURA DE BODE |
| 08105 | FISSURA DE CARNEIRO |
| 03905 | FISSURA SUINA NAO-ESPECIFICADA |
| 00219 | FITA ADESIVA |
| 30106 | FITA ADESIVA PARA CURATIVO |
| 04301 | FITA CASSETE GRAVADA |
| 04401 | FITA CASSETE VIRGEM |
| 04701 | FITA DE FILMADORA |
| 00118 | FITA DE FRALDA (ADESIVA) |
| 00237 | FITA DE MAQUINA DE ESCREVER |
| 00121 | FITA DE TECIDO |
| 01001 | FITA DE VIDEOCASSETE GRAVADA |
| 04501 | FITA DE VIDEOCASSETE VIRGEM |
| 04101 | FITA DE VIDEOGAME |
| 00107 | FITA DENTAL |
| 00114 | FITA METRICA |
| 30506 | FITA PARA MEDICAO DE GLICOSE |
| 02203 | FITO-HORMONIOS |
| 00119 | FIVELA DE CINTO |
| 02611 | FIXADOR DE CABELO |
| 30807 | FIXADOR DE DENTADURA |
| 06701 | FIXOTAC |
| 06701 | FIXOTAC |
| 02805 | FLAMBY EM PO |
| 02001 | FLAMULA |
| 02802 | FLAN EM PO |
| 00701 | FLANELA |
| 01810 | FLANELA PARA BEBE |
| 00802 | FLANELINHA |
| 00702 | FLASH DESCARTAVEL |
| 03104 | FLASH POWER |
| 13105 | FLATELLY VITA LIGHT |
| 00801 | FLAUTA |
| 01502 | FLIPERAMA (FICHA) |
| 03901 | FLIPERAMA E SIMILARES |
| 00203 | FLOCAO DE ARROZ |
| 00904 | FLOCAO DE MILHO |
| 00201 | FLOCOS DE ARROZ |
| 00402 | FLOCOS DE AVEIA |
| 02102 | FLOCOS DE CEREAIS |
| 11402 | FLOCOS DE COCO |
| 04102 | FLOCOS DE GRANOLA |
| 00901 | FLOCOS DE MILHO |
| 05901 | FLOCOS DE MILHO LIGHT |
| 00605 | FLOR DE MILHO |
| 00108 | FLOR DO CAMPO |
| 00101 | FLOR NATURAL |
| 03806 | FLORAIS |
| 02403 | FLOREIRA DE MADEIRA |
| 04601 | FLORES ARTIFICIAIS |
| 00827 | FLUIDO DE FREIO |
| 00801 | FLUIDO DE ISQUEIRO |
| 03103 | FLYING HORSE |
| 03910 | FOCINHEIRA DE ANIMAL |
| 03912 | FOCINHEIRA PARA ANIMAL |
| 00230 | FOGACCIA DE PRESUNTO E QUEIJO PARA VIAGEM |
| 00101 | FOGAO |
| 12401 | FOGAO A GAS |
| 00301 | FOGAO A LENHA |
| 00201 | FOGAO ELETRICO |
| 05002 | FOGAO LIMPO |
| 02101 | FOGAO PARA CAMPING |
| 18201 | FOGAREIRO |
| 02201 | FOGOS E ARTEFATOS JUNINOS |
| 08501 | FOGOTINI PARA ACENDER CHURRASQUEIRA |
| 01617 | FOICE |
| 03003 | FOLHA DE AIPIM |
| 06503 | FOLHA DE ARRUDA |
| 07801 | FOLHA DE BANANEIRA PARA EMBALAGEM |
| 10001 | FOLHA DE CEBOLA |
| 09301 | FOLHA DE CENOURA |
| 05002 | FOLHA DE FORMICA |
| 05002 | FOLHA DE FORMICA |
| 01604 | FOLHA DE LOURO |
| 03004 | FOLHA DE MACAXEIRA |
| 03001 | FOLHA DE MANDIOCA |
| 03002 | FOLHA DE MANIVA |
| 00240 | FOLHA PARA FICHARIO |
| 15401 | FOLHAS DE AMORA (CHA) |
| 01801 | FOLHETO |
| 01802 | FOLHETO MUSICAL |
| 06001 | FONDOR |
| 07901 | FONDUE DE QUEIJO |
| 14302 | FONE DE OUVIDO |
| 04112 | FONOAUDITIVOS |
| 12101 | FONTE DE ALIMENTACAO DE APARELHO ELETRONICO |
| 01802 | FORMA DE BARQUETE |
| 00812 | FORMA DE BAURU |
| 00813 | FORMA DE BOLO, PIZZA, PUDIM, TORTA |
| 07101 | FORMA DE CREPE |
| 07201 | FORMA DE GELO |
| 07401 | FORMA DE MISTO-QUENTE |
| 07301 | FORMA DE PAO |
| 04901 | FORMA, COPO, ETC. DE PAPEL OU PAPELAO |
| 01636 | FORMAO |
| 04603 | FORMAS DE CONFECCAO DE FLORES ARTIFICIAIS |
| 01402 | FORMATURA |
| 05001 | FORMICA (FOLHA) |
| 05001 | FORMICA (FOLHA) |
| 04902 | FORMINHA DE PAPEL |
| 08501 | FORNO A GAS |
| 08301 | FORNO A LENHA |
| 18701 | FORNO DE FERRO |
| 02601 | FORNO DE MICROONDAS |
| 06601 | FORNO DE MICROONDAS |
| 18301 | FORNO DE TORRAR FARINHA |
| 01001 | FORNO ELETRICO |
| 00401 | FORNO ELETRICO |
| 04401 | FORRACAO DE BOTAO |
| 05005 | FORRA-FOGAO |
| 02005 | FORRO (INGRESSO) |
| 06002 | FORRO DE GESSO |
| 07703 | FORRO DE GESSO |
| 06003 | FORRO DE MADEIRA |
| 07702 | FORRO DE MADEIRA |
| 06004 | FORRO DE PVC |
| 07704 | FORRO DE PVC |
| 06001 | FORRO PARA COBERTURA |
| 07701 | FORRO PARA COBERTURA |
| 06005 | FORRO PARA COBERTURA INDETERMINADO |
| 07705 | FORRO PARA COBERTURA INDETERMINADO |
| 00238 | FORROZINHO (PAO DOCE) |
| 10038 | FORROZINHO (PAO DOCE) DIET |
| 10078 | FORROZINHO (PAO DOCE) DIETETICO |
| 03202 | FORTIFICANTE |
| 02406 | FORTIFICANTE PARA ANIMAL DOMESTICO |
| 03201 | FORTIFICANTES E ESTIMULANTES DO APETITE |
| 00602 | FOSFORO |
| 05303 | FOSFORO |
| 00601 | FOSFORO (FUMANTE) |
| 05301 | FOSFOROS |
| 06601 | FOSSA SEPTICA |
| 00601 | FOTOCOPIA XEROX |
| 03802 | FOTOGRAFIA 3X4 |
| 03803 | FOTOGRAFIA 5X7 |
| 00908 | FOTOGRAFIA DE ACIDENTE (PERICIA) |
| 00512 | FOTOGRAFIA RELATIVA A CIRURGIA |
| 00503 | FOTOGRAFIAS DE CERIMONIA |
| 00501 | FOTOGRAFIAS DE CERIMONIAS (FOTOGRAFO PROFISSIONAL) |
| 03801 | FOTOGRAFO (EXCETO DE CERIMONIAS FAMILIARES) |
| 17901 | FOTOMETRO |
| 00301 | FOTONOVELA |
| 00502 | FOTOS DE CERIMONIAS (FOTOGRAFO PROFISSIONAL) |
| 01201 | FRALDA DE TECIDO |
| 31401 | FRALDA DESCARTAVEL |
| 31202 | FRALDA GERIATRICA |
| 01202 | FRALDINHA (CAPA DE FILE) |
| 01005 | FRANGO A PASSARINHO PARA VIAGEM |
| 00101 | FRANGO ABATIDO |
| 05502 | FRANGO ABATIDO ORGANICO |
| 01001 | FRANGO ASSADO OU DEFUMADO PARA VIAGEM |
| 01011 | FRANGO ASSADO PARA VIAGEM |
| 00104 | FRANGO CAIPIRA |
| 05504 | FRANGO CAIPIRA ORGANICO |
| 00201 | FRANGO CONGELADO |
| 05601 | FRANGO CONGELADO ORGANICO |
| 00207 | FRANGO CONGELADO TEMPERADO |
| 05607 | FRANGO CONGELADO TEMPERADO ORGANICO |
| 01006 | FRANGO DEFUMADO PARA VIAGEM |
| 00307 | FRANGO DESFIADO |
| 01404 | FRANGO EM PE |
| 00302 | FRANGO EM PEDACOS NAO-ESPECIFICADOS |
| 05902 | FRANGO EM PEDACOS NAO-ESPECIFICADOS ORGANICO |
| 04901 | FRANGO EMPANADO |
| 01013 | FRANGO FRITO PARA VIAGEM |
| 00103 | FRANGO INTEIRO |
| 00209 | FRANGO INTEIRO CONGELADO |
| 05501 | FRANGO INTEIRO ORGANICO |
| 06401 | FRANGO ORGANICO INTEIRO |
| 00205 | FRANGO RESFRIADO |
| 05605 | FRANGO RESFRIADO ORGANICO |
| 00203 | FRANGO TEMPERADO CONGELADO |
| 05603 | FRANGO TEMPERADO CONGELADO ORGANICO |
| 01401 | FRANGO VIVO |
| 00301 | FRASQUEIRA |
| 00304 | FRATELLY VITA |
| 01604 | FRATELLY VITA LIGHT |
| 00201 | FREEZER |
| 00501 | FREEZER |
| 00501 | FREEZER (ALUGUEL) |
| 01802 | FRESSURA BOVINA NAO-ESPECIFICADA |
| 08102 | FRESSURA DE CARNEIRO |
| 02003 | FRESSURA DE PORCO SALGADA |
| 03902 | FRESSURA SUINA NAO-ESPECIFICADA |
| 01902 | FRETE DE MATERIAL DE CONSTRUCAO |
| 01902 | FRETE DE MATERIAL DE CONSTRUCAO |
| 00601 | FRIGIDEIRA, LEITEIRA, ETC. |
| 00602 | FRIGOBAR |
| 01803 | FRISSURA BOVINA NAO-ESPECIFICADA |
| 08104 | FRISSURA DE CARNEIRO |
| 02004 | FRISSURA DE PORCO SALGADA |
| 03904 | FRISSURA SUINA NAO-ESPECIFICADA |
| 06801 | FRITADEIRA ELETRICA |
| 00405 | FRONHA |
| 03003 | FRONTAL DA CABECA (CASCO BOVINO) |
| 01402 | FRONTLINE |
| 01401 | FRUTA |
| 08401 | FRUTA (NAO-ESPECIFICADA) |
| 03801 | FRUTA DE CONDE |
| 02411 | FRUTA DESIDRATADA |
| 08402 | FRUTA NAO-ESPECIFICADA |
| 06501 | FRUTA ORGANICA |
| 05901 | FRUTA PAO |
| 02401 | FRUTA SECA OU DESIDRATADA |
| 01408 | FRUTAS CRISTALIZADAS (DOCE) |
| 07501 | FRUTEIRA |
| 00830 | FRUTEIRA DE INOX |
| 07601 | FRUTOSE |
| 00602 | FUBA |
| 00601 | FUBA DE MILHO |
| 62107 | FUBAMBA |
| 62108 | FUMBAMBA |
| 06804 | FUMINSECT |
| 01101 | FUMO DE ROLO |
| 00501 | FUMO DESFIADO |
| 00503 | FUMO DESFIADO PARA CACHIMBO |
| 00502 | FUMO DESFIADO PARA CIGARRO |
| 02802 | FUNCHO |
| 02803 | FUNCHO DE FLORENCA |
| 00204 | FUNDO DE APLICACAO (DEPOSITO/APLICACAO) |
| 00202 | FUNDO DE APLICACAO (RETIRADA/RESGATE) |
| 00202 | FUNDO DE APLICACAO FINANCEIRA (CDB, RDB, COMMODITIES, ETC.) |
| 00201 | FUNDO DE APLICACAO FINANCEIRA (CDB, RDB, COMMODITIES, ETC.) (DEPOSITO/APLICACAO) |
| 00201 | FUNDO DE APLICACAO FINANCEIRA (CDB, RDB, COMMODITIES, ETC.) (RETIRADA/RESGATE) |
| 00102 | FUNDO DE PARTICIPACAO EM PLANO/SEGURO-SAUDE (EMPRESA) |
| 01809 | FUNDO DE PENSAO MILITAR |
| 01804 | FUNDO ESPECIAL DA POLICIA MILITAR (PREVIDENCIA MILITAR) |
| 00309 | FUNERAL |
| 00301 | FUNERAL E ARTIGOS RELIGIOSOS |
| 00603 | FUNGHI SECO |
| 09201 | FUNGICIDA |
| 07601 | FUNIL |
| 01802 | FUNRURAL |
| 14405 | FURADEIRA ELETRICA |
| 01608 | FURADEIRA MANUAL |
| 14401 | FURADEIRA, LIXADEIRA, ESMERIL E SERRA ELETRICA |
| 06304 | FURIA |
| 00301 | FUTEBOL (INGRESSO) |
| 00403 | FUTEBOL DE SALAO (INGRESSO) |
| 03702 | GABINETE (MOVEL PARA MAQUINA DE COSTURA) |
| 05201 | GABINETE DE PIA (BANHEIRO) |
| 04002 | GABINETE DE PIA (COPA E COZINHA) |
| 02301 | GAIOLA (DECORACAO) |
| 03904 | GAIOLA DE ANIMAL |
| 03908 | GAIOLA PARA ANIMAL |
| 00901 | GAITA |
| 01003 | GALETO ASSADO OU DEFUMADO PARA VIAGEM |
| 01008 | GALETO DEFUMADO PARA VIAGEM |
| 01004 | GALETO PARA VIAGEM |
| 08301 | GALHETEIRO |
| 09003 | GALHO DE LENHA PARA COMBUSTIVEL DOMESTICO |
| 00102 | GALINHA ABATIDA |
| 05503 | GALINHA ABATIDA ORGANICA |
| 01002 | GALINHA ASSADA OU DEFUMADA PARA VIAGEM |
| 06201 | GALINHA COM ARROZ PARA VIAGEM |
| 00202 | GALINHA CONGELADA |
| 05602 | GALINHA CONGELADA ORGANICA |
| 00208 | GALINHA CONGELADA TEMPERADA |
| 05608 | GALINHA CONGELADA TEMPERADA ORGANICA |
| 03729 | GALINHA DAGUA |
| 03702 | GALINHA DE ANGOLA ABATIDA, CONGELADA OU VIVA |
| 03711 | GALINHA DE ANGOLA CONGELADA |
| 03712 | GALINHA DE ANGOLA VIVA |
| 01007 | GALINHA DEFUMADA PARA VIAGEM |
| 01403 | GALINHA EM PE |
| 00303 | GALINHA EM PEDACOS NAO-ESPECIFICADOS |
| 05903 | GALINHA EM PEDACOS NAO-ESPECIFICADOS ORGANICA |
| 00206 | GALINHA RESFRIADA |
| 05606 | GALINHA RESFRIADA ORGANICA |
| 00204 | GALINHA TEMPERADA CONGELADA |
| 05604 | GALINHA TEMPERADA CONGELADA ORGANICA |
| 01402 | GALINHA VIVA |
| 06301 | GALINHADA PARA VIAGEM |
| 03301 | GALOCHA DE CRIANCA, HOMEM E MULHER |
| 44102 | GANGAPARRA |
| 01101 | GANHO COM VIAGEM A TRABALHO |
| 01003 | GANHO EM JOGOS (LOTERIA, MEGA, ETC.) |
| 01001 | GANHO EM JOGOS (LOTERIA, MEGA, ETC.) OU PREMIO |
| 03705 | GANSO ABATIDO, CONGELADO OU VIVO |
| 03717 | GANSO CONGELADO |
| 03718 | GANSO VIVO |
| 00901 | GARAGEM |
| 02002 | GARAPA |
| 03727 | GARCA ABATIDA, CONGELADA OU VIVA |
| 01801 | GARCOM |
| 01802 | GARCONETE |
| 00703 | GARFO |
| 06202 | GARFO DE PLASTICO |
| 08601 | GARGANTA BOVINA |
| 06701 | GARGANTA SUINA SALGADA |
| 00106 | GARGANTILHA |
| 00904 | GARRAFA PLASTICA DE AGUA |
| 01001 | GARRAFA TERMICA |
| 03804 | GARRAFADA DE QUALQUER TIPO |
| 03805 | GARRAFADA DE QUALQUER TRATAMENTO |
| 00204 | GARRAFAO DE AGUA (LITROS) |
| 00906 | GARRAFAO PLASTICO DE AGUA |
| 01004 | GARRAO |
| 30304 | GARROTE DE BORRACHA |
| 00513 | GAS DE AR CONDICIONADO |
| 00101 | GAS DE BOTIJAO (COMBUSTIVEL DOMESTICO) |
| 00102 | GAS DE BOTIJAO (KG) (COMBUSTIVEL DOMESTICO) |
| 00403 | GAS DE BOTIJAO (PARA ILUMINACAO) |
| 00103 | GAS DE BUJAO (COMBUSTIVEL DOMESTICO) |
| 00104 | GAS DE BUJAO (KG) (COMBUSTIVEL DOMESTICO) |
| 00402 | GAS DE BUJAO (KG) (PARA ILUMINACAO) |
| 00401 | GAS DE BUJAO PARA ILUMINACAO |
| 01401 | GAS DE BUTIJAO DE OUTROS IMOVEIS (KG) |
| 00901 | GAS DE ISQUEIRO |
| 00301 | GAS ENCANADO (M3) |
| 01201 | GAS ENCANADO DE OUTROS IMOVEIS (M3) |
| 01801 | GAS VEICULAR |
| 00901 | GASOLINA (COMBUSTIVEL DOMESTICO) |
| 00902 | GASOLINA (LITRO) (COMBUSTIVEL DOMESTICO) |
| 02802 | GASOLINA ADITIVADA (COMBUSTIVEL DE VEICULO) |
| 00701 | GASOLINA COMUM (COMBUSTIVEL DE VEICULO) |
| 01601 | GASOLINA DE OUTROS IMOVEIS (COMBUSTIVEL DOMESTICO) |
| 02801 | GASOLINA ESPECIAL (COMBUSTIVEL DE VEICULO) |
| 05801 | GASOLINA PARA LIMPEZA |
| 00410 | GASOSA |
| 13307 | GASOSA LIGHT |
| 07508 | GASOSA LIGHT |
| 02402 | GATORADE |
| 05803 | GATORADE |
| 08401 | GAVETA DE JAZIGO |
| 02801 | GAVETEIRO |
| 30103 | GAZE |
| 00104 | GEL DENTAL |
| 02612 | GEL FIXADOR PARA CABELOS |
| 08502 | GEL FOGOTINI PARA ACENDER CHURRASQUEIRA |
| 31112 | GEL MASSAGEADOR |
| 00605 | GEL PARA BARBA |
| 31107 | GEL REDUTOR DE CELULITE |
| 31105 | GEL REDUTOR DE GORDURA |
| 00601 | GELADEIRA |
| 01801 | GELADEIRA (ALUGUEL) |
| 00301 | GELADEIRA DE 1 PORTA |
| 00401 | GELADEIRA DE 2 PORTAS |
| 04807 | GELADINHO |
| 11407 | GELADINHO LIGHT |
| 09802 | GELAGUA |
| 01701 | GELATINA |
| 08103 | GELATINA DE DIETA |
| 08101 | GELATINA DIET |
| 08102 | GELATINA DIETETICA |
| 01703 | GELATINA EM FOLHA |
| 13903 | GELATINA EM FOLHA LIGHT |
| 01702 | GELATINA EM PO |
| 13902 | GELATINA EM PO LIGHT |
| 09604 | GELATINA LIGHT |
| 13901 | GELATINA LIGHT |
| 01004 | GELEIA DE DAMASCO |
| 09602 | GELEIA DE FRUTA LIGHT |
| 01001 | GELEIA DE FRUTAS DE QUALQUER MARCA OU SABOR |
| 07901 | GELEIA DE FRUTAS DE QUALQUER MARCA OU SABOR DIET |
| 07906 | GELEIA DE FRUTAS DE QUALQUER MARCA OU SABOR DIETETICA |
| 10601 | GELEIA DE FRUTAS DE QUALQUER MARCA OU SABOR LIGHT |
| 07905 | GELEIA DE FRUTAS DIET |
| 07910 | GELEIA DE FRUTAS DIETETICA |
| 10605 | GELEIA DE FRUTAS LIGHT |
| 04306 | GELEIA DE LEITE |
| 01101 | GELEIA DE MOCOTO |
| 01102 | GELEIA DE MOCOTO VITAMINADA |
| 04703 | GELEIA DE TOMATE |
| 07904 | GELEIA DIET |
| 07909 | GELEIA DIETETICA |
| 10604 | GELEIA LIGHT |
| 06201 | GELEIA REAL |
| 04802 | GELIKO EM CUBO |
| 04803 | GELITO |
| 04801 | GELO EM CUBO, PEDRA, ETC. |
| 00710 | GEMADA |
| 07701 | GEMADA PARA VIAGEM |
| 00902 | GENEBRA |
| 01701 | GENGIBRE |
| 00508 | GEOMETRIA DE RODAS |
| 07301 | GERADOR DE ENERGIA |
| 09102 | GERGELIM EM GRAO |
| 09101 | GERGELIM EM PO |
| 01301 | GERME DE TRIGO |
| 00801 | GERSAL |
| 04001 | GESSO |
| 04001 | GESSO |
| 02202 | GESSO (PECA DE DECORACAO) |
| 05201 | GEVRAL |
| 00702 | GILETE |
| 45117 | GIMBUIA |
| 00901 | GIN |
| 03303 | GINASIOS (INGRESSO) |
| 00907 | GIRASSOL (SEMENTE) |
| 00252 | GIZ |
| 02003 | GLACE |
| 02002 | GLACUCAR |
| 00105 | GLADIOLOS |
| 01506 | GLICERINA |
| 14601 | GLICOSE DE MANDIOCA |
| 01901 | GLICOSE DE MILHO |
| 01004 | GLOBO DE LUZ |
| 01902 | GLUCOSE DE MILHO |
| 00701 | GLUTAMATO MONOSSODICO |
| 01302 | GLUTEN |
| 01501 | GOBO |
| 61121 | GOIA |
| 04201 | GOIABA |
| 05702 | GOIABA ARACA (ARACA) |
| 01308 | GOIABA EM CALDA |
| 09213 | GOIABA EM CALDA DIET |
| 09214 | GOIABA EM CALDA DIETETICA |
| 09601 | GOIABA ORGANICA |
| 01211 | GOIABADA |
| 62109 | GOIAMU |
| 62104 | GOIAMUM |
| 13519 | GOIANINHA LIGHT |
| 05502 | GOIANINHA REFRIGERANTE |
| 09202 | GOIANINHA REFRIGERANTE LIGHT |
| 03501 | GOLA |
| 01802 | GOMA DE ARARUTA |
| 01506 | GOMA DE CARIMA |
| 01502 | GOMA DE MANDIOCA |
| 00609 | GOMA DE MASCAR |
| 09909 | GOMA DE MASCAR DIET |
| 09922 | GOMA DE MASCAR DIETETICA |
| 09809 | GOMA DE MASCAR LIGHT |
| 00703 | GOMA DE MILHO |
| 02406 | GOMA DE ROUPA |
| 01508 | GOMA DE TAPIOCA |
| 05002 | GOMBO |
| 06602 | GONDOLA |
| 07304 | GORDURA BOVINA |
| 02201 | GORDURA DE COCO |
| 02801 | GORDURA DE GALINHA |
| 02401 | GORDURA DE OVELHA |
| 02301 | GORDURA DE PEIXE |
| 02501 | GORDURA DE RINS DE BODE |
| 03801 | GORDURA HIDROGENADA |
| 03802 | GORDURA PARA SORVETE |
| 02101 | GORDURA SUINA |
| 02202 | GORDURA VEGETAL |
| 64107 | GORIRI |
| 04001 | GORJETA |
| 01601 | GORRO |
| 01801 | GORRO |
| 02201 | GORRO |
| 00901 | GOVERNANTA |
| 00834 | GPS VEICULAR |
| 03301 | GRADE DE FERRO |
| 03301 | GRADE DE FERRO |
| 00231 | GRAFITE |
| 00215 | GRAMPEADOR |
| 00214 | GRAMPO DE GRAMPEADOR |
| 00701 | GRAMPO, PREGADOR DE CABELO, ETC. |
| 03108 | GRANITO |
| 03106 | GRANITO (PEDRA) PARA PIA |
| 03106 | GRANITO (PEDRA) PARA PIA |
| 04101 | GRANOLA |
| 02801 | GRAO DE BICO |
| 02305 | GRAPE FRUIT |
| 00504 | GRAPETE |
| 15201 | GRAPETE DIET |
| 07704 | GRAPETE LIGHT |
| 13405 | GRAPETTE LIGHT |
| 00402 | GRASPA |
| 03802 | GRASPA ORGANICA |
| 02801 | GRAVACAO DE NOME EM OBJETOS PESSOAIS (CANETA, MEDALHA, ETC.) |
| 08301 | GRAVACAO EM JAZIGO |
| 03005 | GRAVADOR |
| 06317 | GRAVADOR DE CD |
| 06313 | GRAVADOR DE CD E DVD |
| 06318 | GRAVADOR DE DVD |
| 03001 | GRAVADOR E TOCA-FITAS |
| 00801 | GRAVATA |
| 04701 | GRAVATA |
| 04001 | GRAVATA |
| 05001 | GRAVIOLA |
| 01504 | GRAVURA |
| 03401 | GRAXA DE SAPATO |
| 08001 | GRELHA |
| 02405 | GREMIO (FUNCIONARIOS DE EMPRESA) |
| 02801 | GREMIO ESTUDANTIL |
| 02304 | GREPE |
| 00801 | GRILL |
| 01301 | GRILL |
| 05401 | GRINALDA |
| 01635 | GROSA |
| 05504 | GROSELHA BRANCA EM FRUTO |
| 05501 | GROSELHA EM FRUTO |
| 05503 | GROSELHA ESPINHOSA EM FRUTO |
| 05502 | GROSELHA PRETA EM FRUTO |
| 05505 | GROSELHA VERMELHA EM FRUTO |
| 08302 | GRUMIXAMA |
| 08301 | GRUMIXAMA DA INDIA |
| 02609 | GRUSTOLI (BOLINHO DOCE) |
| 61102 | GUAIA |
| 04205 | GUAIABA |
| 62105 | GUAIAMU |
| 62106 | GUAIAMUM |
| 62102 | GUAIAUMA |
| 61104 | GUAJA |
| 02601 | GUANDO |
| 02603 | GUANDU |
| 02302 | GUARANA |
| 00302 | GUARANA |
| 09102 | GUARANA DIET |
| 07402 | GUARANA DIET |
| 09104 | GUARANA DIETETICO |
| 07404 | GUARANA DIETETICO |
| 05701 | GUARANA EM PO NATURAL |
| 13103 | GUARANA LIGHT |
| 01602 | GUARANA LIGHT |
| 05702 | GUARANA NATURAL EM PO |
| 07406 | GUARANA ZERO |
| 00305 | GUARAVITA |
| 00306 | GUARAVITON |
| 03801 | GUARDA LOUCA |
| 02006 | GUARDA ROUPA |
| 02007 | GUARDA ROUPAS |
| 06002 | GUARDA-CHUVA |
| 06001 | GUARDA-CHUVA E SOMBRINHA |
| 00506 | GUARDANAPO DE MESA DE TECIDO |
| 04301 | GUARDANAPO DE PAPEL |
| 08201 | GUARDA-SOL |
| 01901 | GUARDA-VOLUMES |
| 04409 | GUARIROBA (PALMITO IN NATURA) |
| 07710 | GUARIROBA (PALMITO IN NATURA) ORGANICO |
| 04410 | GUEIROBA (PALMITO IN NATURA) |
| 07711 | GUEIROBA (PALMITO IN NATURA) ORGANICO |
| 64106 | GUERIRI |
| 04411 | GUEROBA (PALMITO IN NATURA) |
| 07712 | GUEROBA (PALMITO IN NATURA) ORGANICO |
| 00808 | GUIA DE VESTIBULAR |
| 02401 | GUIA TURISTICO |
| 01302 | GUINCHO (REBOQUE) |
| 00129 | GUISO |
| 00201 | GUITARRA |
| 01402 | GUIZADO (CARNE MOIDA DE PRIMEIRA) |
| 01502 | GUIZADO (CARNE MOIDA DE SEGUNDA) |
| 10702 | GUIZADO (CARNE MOIDA DE TERCEIRA) |
| 04302 | GUIZADO (CARNE MOIDA NAO-ESPECIFICADA) |
| 02505 | H2O LIMAO |
| 01011 | H2O SABOR LIMAO |
| 02506 | H2O TANGERINA |
| 14301 | HADFONE |
| 00407 | HAMBURGUER |
| 00502 | HAMBURGUER DE CARNE BOVINA |
| 01101 | HAMBURGUER DE FRANGO |
| 05101 | HAMBURGUER DE FRANGO LIGHT |
| 03001 | HAMBURGUER DE PERU |
| 05601 | HAMBURGUER DE PERU LIGHT |
| 03101 | HAMBURGUER NAO-ESPECIFICADO |
| 05701 | HAMBURGUER NAO-ESPECIFICADO LIGHT |
| 00303 | HAMBURGUER PARA VIAGEM |
| 02202 | HEMODIALISE |
| 02303 | HEPATICO (PARA O FIGADO) |
| 00501 | HERANCA E OUTRAS TRANSFERENCIAS PATRIMONIAIS |
| 00502 | HERANCA/TRANSFERENCIA PATRIMONIAL |
| 00209 | HIDRATACAO (CABELEIREIRO) |
| 00508 | HIDRATANTE (CREME) |
| 00426 | HIDRATANTE CAPILAR |
| 03301 | HIDRELETRICA (INGRESSO) |
| 03304 | HIDROTERAPIA (TRATAMENTO) |
| 02510 | HIGIENIZADOR SANITARIO |
| 03305 | HIPODROMOS (INGRESSO) |
| 10101 | HOME THEATER |
| 04101 | HORA EXTRA |
| 02201 | HORMONIO |
| 08301 | HORTELA (MOLHO) |
| 02301 | HORTELA (TEMPERO INDUSTRIALIZADO) |
| 03001 | HORTELA CHA |
| 02304 | HORTELA COMUM (TEMPERO INDUSTRIALIZADO) |
| 02303 | HORTELA DAS HORTAS (TEMPERO INDUSTRIALIZADO) |
| 08302 | HORTELA EM FOLHA |
| 02307 | HORTELA PIMENTA (TEMPERO INDUSTRIALIZADO) |
| 02306 | HORTELA PRETA (TEMPERO INDUSTRIALIZADO) |
| 02302 | HORTELA ROMANA (TEMPERO INDUSTRIALIZADO) |
| 00601 | HOSPITALIZACAO |
| 00702 | HOTEIS |
| 05001 | HOTEL |
| 44117 | IACA |
| 06502 | IBACUPARI |
| 06505 | IBACURUPARI |
| 06805 | IBIPITANGA |
| 01603 | IDEC |
| 01602 | IDEC (INSTITUTO DE DEFESA DO CONSUMIDOR) |
| 00115 | ILHOS |
| 00935 | IMA DE GELADEIRA |
| 07103 | IMBU |
| 05601 | IMPERMEABILIZANTE |
| 05601 | IMPERMEABILIZANTE |
| 00507 | IMPLANTE DE CABELO (TRATAMENTO) |
| 02408 | IMPLANTE DE DENTE |
| 03802 | IMPOSTO DE RENDA DO EXERCICIO ANTERIOR |
| 00201 | IMPOSTO DE TRANSMISSAO DE OUTROS IMOVEIS (AQUISICAO A VISTA OU A PRAZO) |
| 00102 | IMPOSTO DE TRANSMISSAO DO IMOVEL (AQUISICAO A VISTA) |
| 00501 | IMPOSTO PREDIAL E TERRITORIAL URBANO (IPTU) |
| 00801 | IMPOSTO PREDIAL E TERRITORIAL URBANO (IPTU) DE OUTROS IMOVEIS |
| 03901 | IMPOSTO SINDICAL (ANUAL) |
| 00403 | IMPOSTO SOBRE PROPRIEDADE (IPVA) |
| 00401 | IMPOSTO SOBRE PROPRIEDADE DE VEICULOS AUTOMOTORES (IPVA) |
| 01101 | IMPOSTO SOBRE PROPRIEDADE TERRITORIAL RURAL (IPTR) |
| 02201 | IMPOSTO SOBRE PROPRIEDADE TERRITORIAL RURAL (IPTR) DE OUTROS IMOVEIS |
| 03501 | IMPOSTO SOBRE SERVICOS (ISS) (EVENTUAL) |
| 00203 | IMPOSTOS PARA AQUISICAO DO IMOVEL A PRAZO |
| 06304 | IMPRESSORA DE COMPUTADOR |
| 06303 | IMPRESSORA DE MICROCOMPUTADOR |
| 01605 | INAJA |
| 00712 | INAMBU (CARA) |
| 03726 | INAMBU ABATIDA, CONGELADA OU VIVA |
| 02509 | INCENSO |
| 01301 | INCRA |
| 02202 | INCRA DE OUTROS IMOVEIS (IMPOSTO) |
| 01801 | INDENIZACAO A TERCEIROS |
| 01901 | INDENIZACAO A TERCEIROS |
| 05601 | INDENIZACAO E RESTITUICAO DE SEGURO OBRIGATORIO DE AUTO/UTILITARIOS PESSOAL |
| 05801 | INDENIZACAO E RESTITUICAO DE SEGURO OBRIGATORIO DE MOTO |
| 05501 | INDENIZACAO E RESTITUICAO DE SEGURO PESSOAL |
| 05701 | INDENIZACAO E RESTITUICAO DE SEGURO VOLUNTARIO DE AUTO/UTILITARIOS PESSOAL |
| 05901 | INDENIZACAO E RESTITUICAO DE SEGURO VOLUNTARIO DE MOTO |
| 02101 | INDENIZACAO JUDICIAL |
| 06301 | INDENIZACAO TRABALISTA |
| 06601 | INGA |
| 06602 | INGA CIPO |
| 06606 | INGA DA PRAIA |
| 06607 | INGA DE FERRADURA |
| 06604 | INGA DEDO |
| 06603 | INGA FEIJAO |
| 06605 | INGA MIRIM |
| 06608 | INGA XIXI |
| 02804 | INHAMBU ABATIDO |
| 03736 | INHAME |
| 00501 | INHAME |
| 00713 | INHAME CARAQUENTO (CARA) |
| 00715 | INHAME CASCO (CARA) |
| 00504 | INHAME CHINES |
| 00505 | INHAME DA COSTA |
| 00506 | INHAME DE AGUA |
| 00714 | INHAME LISO (CARA) |
| 00503 | INHAME SAO TOME |
| 00502 | INHAME TAIA |
| 02703 | INHOQUE SEMIPRONTO |
| 30013 | INJECAO AMPOLA (NAO-DETERMINADA) |
| 02403 | INJECAO PARA ANIMAL DOMESTICO |
| 02501 | INPS DE OBRA |
| 02502 | INPS DE OBRA |
| 04202 | INSCRICAO DE VESTIBULAR |
| 02601 | INSETICIDA |
| 02501 | INSS DE OBRA |
| 00505 | INSTALACAO DE SOM (MAO-DE-OBRA) |
| 00509 | INSTRUMENTADOR CIRURGICO |
| 02507 | INSTRUMENTOS CIRURGICOS DE CURSO |
| 02508 | INSTRUMENTOS DE AULA PRATICA |
| 02509 | INSTRUMENTOS DE CURSOS EDUCATIVOS |
| 02501 | INSTRUMENTOS E APARELHOS DE CURSOS EDUCATIVOS |
| 00832 | INSULFILM |
| 01001 | INTEGRACAO ONIBUS-METRO |
| 01101 | INTEGRACAO TREM-METRO |
| 01601 | INTEGRACAO TREM-ONIBUS |
| 15501 | INTERFONE |
| 00211 | INTERLACE (CABELEIREIRO) |
| 00604 | INTERNACAO EM HOSPITAL |
| 00603 | INTERNACAO EM MATERNIDADE |
| 00602 | INTERNACAO EM PRONTO SOCORRO |
| 00502 | INTERNET BANDA LARGA |
| 01301 | INTERNET E CELULAR (PACOTE) |
| 00504 | INTERNET SEM FIO |
| 00506 | INTERNET VIA RADIO |
| 00505 | INTERNET VIA SATELITE |
| 03701 | INTERTELA |
| 00605 | INTESTINAL |
| 00104 | INVENTARIO |
| 31102 | IODO, SALICILATO (PARA MASSAGEM) |
| 01301 | IOGURTE |
| 01208 | IOGURTE ACTIVIA |
| 01202 | IOGURTE DANONE |
| 04002 | IOGURTE DANONE DIET |
| 04006 | IOGURTE DANONE DIETETICO |
| 03902 | IOGURTE DANONE LIGHT |
| 04802 | IOGURTE DANONE ORGANICO |
| 01201 | IOGURTE DE QUALQUER SABOR |
| 04003 | IOGURTE DE QUALQUER SABOR DIET |
| 04007 | IOGURTE DE QUALQUER SABOR DIETETICO |
| 03904 | IOGURTE DE QUALQUER SABOR LIGHT |
| 01302 | IOGURTE DE SABORES |
| 02308 | IOGURTE DE SOJA |
| 01210 | IOGURTE DESNATADO |
| 03903 | IOGURTE DESNATADO (LIGHT) |
| 04803 | IOGURTE DESNATADO ORGANICO |
| 04001 | IOGURTE DIET |
| 04005 | IOGURTE DIETETICO |
| 05002 | IOGURTE DIETETICO ORGANICO |
| 03901 | IOGURTE LIGHT |
| 01211 | IOGURTE LIQUIDO |
| 01203 | IOGURTE NATURAL |
| 04804 | IOGURTE NATURAL ORGANICO |
| 04801 | IOGURTE ORGANICO |
| 07701 | IOGURTE ORGANICO DE QUALQUER SABOR DIET |
| 07702 | IOGURTE ORGANICO DE QUALQUER SABOR DIETETICO |
| 05001 | IOGURTE ORGANICO DIET |
| 09901 | IOGURTE ORGANICO LIGHT |
| 04901 | IOGURTE ORGANICO LIGHT |
| 10201 | IOGURTEIRA |
| 05603 | IOIO CREME (CHOCOLATE EM CREME) |
| 12203 | IOIO CREME (CHOCOLATE EM CREME) DIET |
| 12206 | IOIO CREME (CHOCOLATE EM CREME) DIETETICO |
| 12103 | IOIO CREME (CHOCOLATE EM CREME) LIGHT |
| 01102 | IPTR |
| 00502 | IPTU |
| 00402 | IPVA (IMPOSTO SOBRE PROPRIEDADE DE VEICULOS AUTOMOTORES) |
| 00518 | ISCA ARTIFICIAL DE PESCA |
| 02607 | ISCA PARA INSETO |
| 05806 | ISOTONICO |
| 03701 | ISQUEIRO DE MESA |
| 00701 | ISQUEIRO DE OURO OU PRATA |
| 00201 | ISQUEIRO DESCARTAVEL |
| 08402 | ISQUEIRO DESCARTAVEL PARA FOGAO |
| 05301 | ISQUEIRO NAO-DESCARTAVEL (USO PESSOAL) |
| 08403 | ISQUEIRO PARA ACENDER FOGAO |
| 08401 | ISQUEIRO PARA FOGAO |
| 03502 | ISS (IMPOSTO SOBRE SERVICOS) (EVENTUAL) |
| 44115 | IURARA |
| 44114 | IURURA |
| 00103 | JABA |
| 04901 | JABOTICABA |
| 04907 | JABOTICABA ACU |
| 04902 | JABOTICABA DO MATO |
| 04909 | JABOTICABA MIUDA |
| 04904 | JABOTICABA MURTA |
| 04903 | JABOTICABA PAULISTA |
| 04906 | JABOTICATUBA |
| 04908 | JABUTICABA |
| 04905 | JABUTICATUBA |
| 04101 | JACA |
| 04103 | JACA DA BAHIA |
| 07203 | JACA DE POBRE (BERIBA) |
| 04102 | JACA DURA |
| 04105 | JACA MANTEIGA |
| 04104 | JACA MOLE |
| 03725 | JACANA ABATIDA, CONGELADA OU VIVA |
| 01704 | JACARE (CARNE BOVINA DE SEGUNDA C/ OSSO) |
| 03728 | JACU ABATIDO |
| 03202 | JALECO |
| 04202 | JALECO |
| 05301 | JAMBO |
| 05303 | JAMBO AMARELO |
| 05306 | JAMBO CHEIROSO |
| 05308 | JAMBO COMUM |
| 05307 | JAMBO DA INDIA |
| 05309 | JAMBO ENCARNADO |
| 05305 | JAMBO MORENO |
| 05302 | JAMBO ROSA |
| 05311 | JAMBO ROXO |
| 05304 | JAMBO VERDADEIRO |
| 05310 | JAMBO VERMELHO |
| 02105 | JAMBU |
| 02101 | JAMBUACU |
| 10201 | JAMELAO |
| 00303 | JANELA |
| 00304 | JANELA |
| 00301 | JANELA E PORTA |
| 00301 | JANELA E PORTA |
| 03503 | JANTAR A LA CARTE |
| 03505 | JANTAR A LA CARTE E BEBIDA |
| 03507 | JANTAR A LA CARTE E SOBREMESA |
| 03509 | JANTAR A LA CARTE, BEBIDA E SOBREMESA |
| 00103 | JANTAR A PESO |
| 00105 | JANTAR A PESO E BEBIDA |
| 00107 | JANTAR A PESO E SOBREMESA |
| 00109 | JANTAR A PESO, BEBIDA E SOBREMESA |
| 05801 | JANTAR DA ESCOLA |
| 00108 | JANTAR EM QUENTINHA PARA VIAGEM |
| 03603 | JANTAR FAST FOOD |
| 03605 | JANTAR FAST FOOD E BEBIDA |
| 03607 | JANTAR FAST FOOD E SOBREMESA |
| 03609 | JANTAR FAST FOOD, BEBIDA E SOBREMESA |
| 00107 | JANTAR PARA VIAGEM |
| 01302 | JAO |
| 13510 | JAO LIGHT |
| 08302 | JAO LIGHT |
| 00104 | JAQUETA |
| 00106 | JAQUETA |
| 00103 | JAQUETA |
| 01301 | JARDIM ZOOLOGICO (INGRESSO) |
| 00502 | JARDINEIRA |
| 01002 | JARDINEIRA |
| 01002 | JARDINEIRA |
| 01302 | JARDINEIRA |
| 06101 | JARDINEIRA (SELETA) |
| 00501 | JARDINEIRO |
| 07701 | JARRA |
| 03401 | JARRA DE CRISTAL |
| 21602 | JARRA ELETRICA |
| 02101 | JARRO DE BARRO (DECORACAO) |
| 06305 | JATO |
| 02308 | JEEP |
| 04004 | JENIPA |
| 04002 | JENIPAPA |
| 04003 | JENIPAPINHO |
| 04001 | JENIPAPO |
| 04005 | JENIPAPO MANSO |
| 03805 | JERIMUM |
| 03807 | JERIMUM CABOCLO |
| 03806 | JERIMUM DE LEITE |
| 03808 | JERIMUM JACAREZINHO |
| 01301 | JET SKI |
| 45109 | JIA |
| 01708 | JICARA |
| 04201 | JILO |
| 04203 | JILO JAPONES |
| 10501 | JILO ORGANICO |
| 04202 | JILO PAULISTA |
| 04204 | JILO PRETO |
| 02608 | JIMO CUPIM |
| 01902 | JIPE (KART) |
| 01401 | JOALHEIRO |
| 02205 | JOAO GOMES |
| 02211 | JOBS DE CAMARAO |
| 10111 | JOBS DE CAMARAO LIGHT |
| 08302 | JOELHO SUINO |
| 00602 | JOGGING |
| 00802 | JOGGING |
| 00902 | JOGGING |
| 00605 | JOGO AMERICANO DE PLASTICO |
| 00505 | JOGO AMERICANO DE TECIDO |
| 13802 | JOGO COMPLETO DE COPOS FINOS OU DE LUXO |
| 00207 | JOGO DE ADULTO E CRIANCA |
| 00205 | JOGO DE ARMAR |
| 00823 | JOGO DE BACIAS DE ALUMINIO |
| 01002 | JOGO DE BUZIOS |
| 01001 | JOGO DE BUZIOS (PRATICA RELIGIOSA) |
| 07901 | JOGO DE CANECAS |
| 00701 | JOGO DE CARTAS (APOSTA) |
| 04501 | JOGO DE COZINHA (TECIDO) |
| 00807 | JOGO DE DOMINO (APOSTA) |
| 00834 | JOGO DE ESPETOS DE METAL |
| 01650 | JOGO DE FERRAMENTAS |
| 01803 | JOGO DE FORMAS DE BARQUETE |
| 13403 | JOGO DE JANTAR |
| 02001 | JOGO DE LUZ (ALUGUEL) |
| 00825 | JOGO DE MARMITAS |
| 00604 | JOGO DE MESA DE PLASTICO |
| 00504 | JOGO DE MESA DE TECIDO |
| 00217 | JOGO DE MONTAR |
| 13603 | JOGO DE PANELAS |
| 02104 | JOGO DE PANELAS DE BARRO |
| 18501 | JOGO DE PORCELANA |
| 00203 | JOGO DE PRATOS DE LOUCA |
| 00408 | JOGO DE ROUPAS DE CAMA |
| 01502 | JOGO DE SALADEIRAS |
| 03002 | JOGO DE TECIDO DE BANHEIRO |
| 08101 | JOGO DE TIGELAS |
| 00304 | JOGO DE TOALHAS DE BANHO |
| 04602 | JOGO DE TRAVESSAS REFRATARIAS PARA MICROONDAS |
| 00401 | JOGO DO BICHO |
| 00206 | JOGO EDUCATIVO |
| 00934 | JOGO PLASTICO DE CONDIMENTOS |
| 00932 | JOGO PLASTICO DE MANTIMENTOS OU CONDIMENTOS |
| 01501 | JOGOS ELETRONICOS (FICHA) |
| 00602 | JOIA (TAXA DE INGRESSO) DE PREVIDENCIA PRIVADA |
| 00101 | JOIAS (ALIANCA, ANEL, ETC.) |
| 00101 | JORNAL |
| 01707 | JUCARA |
| 00107 | JUIZ DE PAZ |
| 00610 | JUJUBA |
| 09910 | JUJUBA DIET |
| 09923 | JUJUBA DIETETICA |
| 09810 | JUJUBA LIGHT |
| 44112 | JURARA |
| 44110 | JURARA ACU |
| 00802 | JUROS DE ALUGUEL DE GARAGEM |
| 03602 | JUROS DE CARTAO DE CREDITO |
| 03202 | JUROS DE CHEQUE ESPECIAL |
| 00902 | JUROS DE CONDOMINIO |
| 01301 | JUROS DE EMPRESTIMO |
| 01002 | JUROS DE IMPOSTO PREDIAL |
| 00702 | JUROS DE PRESTACAO DO IMOVEL |
| 00602 | JUROS DO ALUGUEL DO IMOVEL |
| 01101 | JUROS E SEGURO DE EMPRESTIMO |
| 08201 | JURUBEBA |
| 02411 | JURUBEBA VINHO |
| 03611 | JURUBEBA VINHO ORGANICA |
| 44119 | JURURA |
| 01712 | JUSSARA |
| 03201 | JUSTICA DO TRABALHO |
| 00113 | KALANCHOE |
| 04903 | KAMA BOKU (TAINHA EM CONSERVA) |
| 04904 | KAMABOKU (TAINHA EM CONSERVA) |
| 05901 | KANI KAMA |
| 03306 | KAOL |
| 05401 | KARAOKE |
| 02004 | KARAOKE (INGRESSO) |
| 01401 | KARAOKE OU VIDEOKE (ALUGUEL) |
| 01903 | KARO |
| 01901 | KART |
| 04807 | KATCHUP |
| 04805 | KAT-CHUP |
| 10905 | KAT-CHUP LIGHT |
| 05502 | KATSUOBUSHI (PEIXE EM PO) |
| 02801 | KEEP COOLER |
| 04802 | KETCHUP |
| 10902 | KETCHUP LIGHT |
| 04405 | KINDER OVO |
| 05804 | KINDER OVO |
| 12404 | KINDER OVO DIET |
| 12408 | KINDER OVO DIETETICO |
| 12304 | KINDER OVO LIGHT |
| 02401 | KINKAN |
| 01301 | KIT CARNAVALESCO |
| 00409 | KIT DE BERCO |
| 07901 | KIT DE CARNE DE CHURRASCO |
| 08502 | KIT ESCOLAR (AGREGADO) |
| 09501 | KIT PARA CHURRASCO |
| 02603 | KITUTE BOVINO |
| 02704 | KITUTE SUINO |
| 07801 | KIWI |
| 02303 | KOMBI |
| 01604 | KOMBI |
| 01002 | KOMBI |
| 02402 | KUNQUAT |
| 00206 | LA DE ACO |
| 05702 | LACINHO (ENFEITE DE CABELO) |
| 06401 | LAGARTO BOVINO NAO-ESPECIFICADO |
| 00701 | LAGARTO COMUM |
| 11901 | LAGARTO COMUM ORGANICO |
| 00605 | LAGARTO PAULISTA |
| 11805 | LAGARTO PAULISTA ORGANICO |
| 00703 | LAGARTO PLANO |
| 11903 | LAGARTO PLANO ORGANICO |
| 06402 | LAGARTO RECHEADO |
| 00601 | LAGARTO REDONDO |
| 11801 | LAGARTO REDONDO ORGANICO |
| 67101 | LAGOSTA |
| 67111 | LAGOSTA CABO VERDE |
| 67108 | LAGOSTA COMUM |
| 67107 | LAGOSTA DE ESPINHO |
| 67103 | LAGOSTA JAPONESA |
| 67112 | LAGOSTA PINTADA |
| 67401 | LAGOSTA SALGADA |
| 67105 | LAGOSTA SAPATEIRA |
| 67201 | LAGOSTA SEM CASCA |
| 67113 | LAGOSTA VERDADEIRA |
| 67106 | LAGOSTA VERDE |
| 67110 | LAGOSTA VERMELHA |
| 67104 | LAGOSTIM |
| 02101 | LAJE PRE-MOLDADA |
| 02101 | LAJE PRE-MOLDADA |
| 00104 | LAJOTA |
| 00105 | LAJOTA |
| 64108 | LAMBRETA (OSTRA) |
| 00701 | LAMINA DE BARBEAR |
| 01646 | LAMINA DE SERRA |
| 05502 | LAMPADA |
| 05505 | LAMPADA BRANCA |
| 05501 | LAMPADA DE QUALQUER TIPO |
| 05506 | LAMPADA ELETRONICA |
| 05503 | LAMPADA FLUORESCENTE |
| 05504 | LAMPADA FRIA |
| 05507 | LAMPADA TIPO ECONOMICA |
| 02302 | LAMPARINA |
| 02301 | LAMPIAO |
| 01801 | LANCA-PERFUME |
| 01201 | LANCHA |
| 04101 | LANCHE |
| 05901 | LANCHE DA ESCOLA |
| 04806 | LANCHE MIRABEL |
| 08406 | LANCHE MIRABEL DIET |
| 08419 | LANCHE MIRABEL DIETETICO |
| 08306 | LANCHE MIRABEL LIGHT |
| 03601 | LANCHE PARA VIAGEM |
| 06801 | LANCHE PEPERI (PRESUNTOS SUINO E DE FRANGO) |
| 03601 | LANCHEIRA (EXCETO ESCOLAR) |
| 02002 | LANCHEIRA ESCOLAR |
| 00605 | LANCHONETES |
| 15301 | LANTERNA DE PILHA |
| 08202 | LAPIDE |
| 08201 | LAPIDE DE JAZIGO |
| 00208 | LAPIS DE COR |
| 00406 | LAPIS PARA OLHOS |
| 00201 | LAPIS, BORRACHA, CANETA E OUTROS ARTIGOS DE PAPELARIA |
| 07202 | LAPTOP |
| 01403 | LARANJA |
| 01801 | LARANJA (NAO-ESPECIFICADA) |
| 01405 | LARANJA ABRIL |
| 01410 | LARANJA ACIDA |
| 01204 | LARANJA ACUCAR |
| 01702 | LARANJA AMARGA |
| 01407 | LARANJA APANAZIA |
| 01703 | LARANJA AZEDA |
| 01501 | LARANJA BAHIA |
| 01504 | LARANJA BAIANINHA |
| 01215 | LARANJA BAIAO |
| 01507 | LARANJA BANANA |
| 01414 | LARANJA BARAO |
| 01705 | LARANJA BIGARADE |
| 01704 | LARANJA BRANCA |
| 01304 | LARANJA CAMPISTA |
| 01406 | LARANJA CAPETA |
| 01415 | LARANJA CARIOCA |
| 01411 | LARANJA CASCA FINA |
| 01506 | LARANJA CATARINA |
| 01309 | LARANJA CAVALO |
| 01207 | LARANJA CELESTE |
| 09501 | LARANJA CHAMPAGNE |
| 09502 | LARANJA CHAMPANHA |
| 09701 | LARANJA CHINA |
| 01412 | LARANJA CITRICA |
| 01306 | LARANJA COCO |
| 01416 | LARANJA COMUM |
| 02221 | LARANJA CRAVO |
| 09702 | LARANJA DA CHINA |
| 01212 | LARANJA DA ILHA |
| 01701 | LARANJA DA TERRA |
| 01404 | LARANJA DE ABRIL |
| 01206 | LARANJA DE AGUA |
| 01707 | LARANJA DE DOCE |
| 01402 | LARANJA DE NATAL |
| 01706 | LARANJA DE SEVILHA |
| 01502 | LARANJA DE UMBIGO |
| 01205 | LARANJA DO CEU |
| 01202 | LARANJA DOCINHA |
| 01309 | LARANJA EM CALDA |
| 09215 | LARANJA EM CALDA DIET |
| 09216 | LARANJA EM CALDA DIETETICA |
| 01314 | LARANJA FEIJAO CRU |
| 01310 | LARANJA FLOR |
| 01307 | LARANJA FOLHA MURCHA |
| 01209 | LARANJA ILHOA |
| 02406 | LARANJA JABUTICABA |
| 01312 | LARANJA JOAO NUNES |
| 02405 | LARANJA KINKAN |
| 02404 | LARANJA KUNQUAT |
| 01201 | LARANJA LIMA |
| 01313 | LARANJA MACA |
| 01210 | LARANJA MEL |
| 01213 | LARANJA MEL ROSA |
| 01208 | LARANJA MIMO |
| 01214 | LARANJA MIMO DO CEU |
| 02220 | LARANJA MONGORT |
| 01305 | LARANJA MONJOLO |
| 02222 | LARANJA MORGOT |
| 01802 | LARANJA NAO-ESPECIFICADA |
| 01403 | LARANJA NATAL |
| 01409 | LARANJA OURO |
| 01211 | LARANJA PARATI |
| 01303 | LARANJA PAULISTA |
| 01401 | LARANJA PERA |
| 01413 | LARANJA PERA DO RIO |
| 02228 | LARANJA POKAN |
| 01308 | LARANJA ROSA |
| 01311 | LARANJA RUSSA |
| 01408 | LARANJA SABARA |
| 02107 | LARANJA SANGUINEA (LIMAO GALEGO) |
| 01301 | LARANJA SELETA |
| 01302 | LARANJA SELETA DO RIO |
| 01503 | LARANJA SEM CAROCOS |
| 01203 | LARANJA SERRA DE AGUA |
| 01505 | LARANJA TAQUARI |
| 01508 | LARANJA VALENCIA |
| 01509 | LARANJA VALENCIANA |
| 02407 | LARANJINHA JAPONESA |
| 08901 | LAREIRA |
| 02905 | LASANHA A BOLONHESA SEMIPRONTA |
| 02902 | LASANHA SEMIPRONTA |
| 06202 | LASANHA SEMIPRONTA LIGHT |
| 00128 | LASTEX (ELASTICO) |
| 00809 | LATA DE CONDIMENTO (AVULSA) |
| 00901 | LATA DE LIXO |
| 01302 | LATA DE MANTIMENTOS |
| 06501 | LATA VAZIA (USADA EM CONSTRUCAO) |
| 06901 | LATA VAZIA (USADA EM CONSTRUCAO) |
| 01802 | LAVA LOUCA |
| 02415 | LAVA ROUPAS |
| 01301 | LAVADEIRA |
| 01701 | LAVADEIRA E PASSADEIRA |
| 01302 | LAVADEIRO |
| 02201 | LAVADOR DE CARRO |
| 02102 | LAVADOR DE PISCINA |
| 09601 | LAVADORA A VAPOR |
| 08401 | LAVADORA DE ALTA PRESSAO |
| 00102 | LAVAGEM (BARBEIRO) |
| 00202 | LAVAGEM (CABELEIREIRO) |
| 01901 | LAVAGEM SEM LUBRIFICACAO |
| 01902 | LAVA-JATO |
| 00203 | LAVANDA |
| 00502 | LAVANDERIA |
| 03901 | LAVANDERIA PLANEJADA |
| 03304 | LAVINOX |
| 00606 | LAXANTE |
| 00602 | LAXANTE E PURGATIVO |
| 11203 | LAZANHA EM FATIA LIGHT |
| 08004 | LAZANHA PRONTA LIGHT PARA VIAGEM |
| 00904 | LAZANHA PRONTA PARA VIAGEM |
| 01201 | LEASING DE AUTOMOVEL |
| 06706 | LEBRE VIVA |
| 05301 | LECITINA |
| 01102 | LEGUME EM CONSERVA NAO-ESPECIFICADO |
| 01101 | LEGUME NAO-ESPECIFICADO EM CONSERVA |
| 08402 | LEITAO MORTO EVISCERADO |
| 00201 | LEITE |
| 03102 | LEITE ACHOCOLATADO |
| 06802 | LEITE ACHOCOLATADO DIET |
| 06812 | LEITE ACHOCOLATADO DIETETICO |
| 03301 | LEITE ACHOCOLATADO INDUSTRIALIZADO |
| 06702 | LEITE ACHOCOLATADO LIGHT |
| 03303 | LEITE ACHOCOLATADO TODDYNHO |
| 03109 | LEITE AROMATIZADO |
| 06809 | LEITE AROMATIZADO DIET |
| 06819 | LEITE AROMATIZADO DIETETICO |
| 06709 | LEITE AROMATIZADO LIGHT |
| 00903 | LEITE BEIJINHO |
| 05503 | LEITE BEIJINHO DIET |
| 05507 | LEITE BEIJINHO DIETETICO |
| 05403 | LEITE BEIJINHO LIGHT |
| 03101 | LEITE COM SABOR |
| 06801 | LEITE COM SABOR DIET |
| 06811 | LEITE COM SABOR DIETETICO |
| 06701 | LEITE COM SABOR LIGHT |
| 00901 | LEITE CONDENSADO |
| 05501 | LEITE CONDENSADO DIET |
| 05505 | LEITE CONDENSADO DIETETICO |
| 05401 | LEITE CONDENSADO LIGHT |
| 00202 | LEITE DA ROCA |
| 04402 | LEITE DA ROCA ORGANICO |
| 00401 | LEITE DE BUFALA |
| 00301 | LEITE DE CABRA |
| 10301 | LEITE DE CABRA LIGHT |
| 05301 | LEITE DE CABRA LIGHT |
| 06301 | LEITE DE CABRA ORGANICO |
| 07401 | LEITE DE CABRA ORGANICO |
| 03801 | LEITE DE COCO |
| 10601 | LEITE DE COCO LIGHT |
| 00511 | LEITE DE COLONIA |
| 00501 | LEITE DE JUMENTA |
| 00502 | LEITE DE ONCA |
| 00515 | LEITE DE ROSAS |
| 03201 | LEITE DE SOJA |
| 02306 | LEITE DE SOJA |
| 02301 | LEITE DE SOJA COM SABOR |
| 05201 | LEITE DE SOJA COM SABOR LIGHT |
| 03501 | LEITE DE SOJA EM PO |
| 08001 | LEITE DE SOJA EM PO DIET |
| 06901 | LEITE DE SOJA EM PO LIGHT |
| 02304 | LEITE DE SOJA INTEGRAL |
| 10101 | LEITE DE SOJA LIGHT |
| 02305 | LEITE DE SOJA NATURAL |
| 03601 | LEITE DE VACA DESNATADO |
| 04501 | LEITE DE VACA DESNATADO ORGANICO |
| 00201 | LEITE DE VACA FRESCO |
| 04401 | LEITE DE VACA FRESCO ORGANICO |
| 00203 | LEITE DE VACA IN NATURA |
| 04403 | LEITE DE VACA IN NATURA ORGANICO |
| 00101 | LEITE DE VACA INTEGRAL |
| 04301 | LEITE DE VACA INTEGRAL ORGANICO |
| 03605 | LEITE DE VACA LIGHT |
| 06101 | LEITE DE VACA ORGANICO |
| 03602 | LEITE DE VACA PASTEURIZADO DESNATADO |
| 04502 | LEITE DE VACA PASTEURIZADO DESNATADO ORGANICO |
| 00111 | LEITE DE VACA PASTEURIZADO INTEGRAL |
| 04311 | LEITE DE VACA PASTEURIZADO INTEGRAL ORGANICO |
| 03803 | LEITE DE VACA PASTEURIZADO NAO ESPECIFICADO |
| 03703 | LEITE DE VACA PASTEURIZADO SEMIDESNATADO |
| 04603 | LEITE DE VACA PASTEURIZADO SEMIDESNATADO ORGANICO |
| 03702 | LEITE DE VACA SEMIDESNATADO |
| 04602 | LEITE DE VACA SEMIDESNATADO ORGANICO |
| 03603 | LEITE DESNATADO DE VACA |
| 04503 | LEITE DESNATADO DE VACA ORGANICO |
| 07001 | LEITE EM PO COM SABOR |
| 00701 | LEITE EM PO DESENGORDURADO |
| 00710 | LEITE EM PO DESNATADO |
| 00705 | LEITE EM PO DESNATADO OU DESENGORDURADO |
| 00703 | LEITE EM PO ELEDON DESENGORDURADO |
| 00608 | LEITE EM PO FRIGOR INTEGRAL |
| 00603 | LEITE EM PO GLORIA INTEGRAL |
| 00601 | LEITE EM PO INTEGRAL |
| 00606 | LEITE EM PO LACTOGENO INTEGRAL |
| 07701 | LEITE EM PO LIGHT |
| 00704 | LEITE EM PO MOCOCA DESENGORDURADO |
| 00708 | LEITE EM PO MOLICO DESENGORDURADO |
| 00607 | LEITE EM PO NANON INTEGRAL |
| 00801 | LEITE EM PO NAO-ESPECIFICADO |
| 00707 | LEITE EM PO NESTOGENO 1. SEMESTRE DESENGORDURADO |
| 00605 | LEITE EM PO NESTOGENO 2. SEMESTRE INTEGRAL |
| 00604 | LEITE EM PO NINHO INSTANTANEO INTEGRAL |
| 00602 | LEITE EM PO NINHO INTEGRAL |
| 00702 | LEITE EM PO PELARGON DESENGORDURADO |
| 00709 | LEITE EM PO PRODIETON DESENGORDURADO |
| 00706 | LEITE EM PO SEMILKO DESENGORDURADO |
| 00104 | LEITE ESPECIAL INTEGRAL |
| 04304 | LEITE ESPECIAL INTEGRAL ORGANICO |
| 00109 | LEITE ESTERILIZADO INTEGRAL |
| 04309 | LEITE ESTERILIZADO INTEGRAL ORGANICO |
| 01304 | LEITE FERMENTADO |
| 01308 | LEITE FERMENTADO COM SUCRILHOS |
| 07601 | LEITE FERMENTADO DIET |
| 01309 | LEITE FERMENTADO INTEGRAL |
| 08602 | LEITE FERMENTADO LIGHT |
| 01302 | LEITE FERMENTADO YAKULT |
| 00204 | LEITE FRESCO |
| 04204 | LEITE GELEIFICADO |
| 13804 | LEITE GELEIFICADO DIET |
| 13809 | LEITE GELEIFICADO DIETETICO |
| 00106 | LEITE GLUT LONGA VIDA INTEGRAL |
| 04306 | LEITE GLUT LONGA VIDA INTEGRAL ORGANICO |
| 02301 | LEITE HIDRATANTE |
| 00205 | LEITE IN NATURA |
| 00110 | LEITE INTEGRAL |
| 04310 | LEITE INTEGRAL ORGANICO |
| 03604 | LEITE LIGHT DE VACA |
| 03103 | LEITE LONGA VIDA COM SABOR |
| 06803 | LEITE LONGA VIDA COM SABOR DIET |
| 06813 | LEITE LONGA VIDA COM SABOR DIETETICO |
| 06703 | LEITE LONGA VIDA COM SABOR LIGHT |
| 00105 | LEITE LONGA VIDA INTEGRAL |
| 04305 | LEITE LONGA VIDA INTEGRAL ORGANICO |
| 00902 | LEITE MOCA |
| 05502 | LEITE MOCA DIET |
| 05506 | LEITE MOCA DIETETICO |
| 05402 | LEITE MOCA LIGHT |
| 03801 | LEITE NAO-ESPECIFICADO PASTEURIZADO |
| 01308 | LEITE PARA ANIMAL NAO-ESPECIFICADO |
| 00108 | LEITE PASTEURIZADO INTEGRAL |
| 04308 | LEITE PASTEURIZADO INTEGRAL ORGANICO |
| 03802 | LEITE PASTEURIZADO NAO-ESPECIFICADO |
| 03701 | LEITE SEMIDESNATADO DE VACA |
| 04601 | LEITE SEMIDESNATADO DE VACA ORGANICO |
| 00107 | LEITE TIPO A INTEGRAL |
| 04307 | LEITE TIPO A INTEGRAL ORGANICO |
| 00102 | LEITE TIPO B INTEGRAL |
| 04302 | LEITE TIPO B INTEGRAL ORGANICO |
| 00112 | LEITE TIPO C COMUM |
| 00103 | LEITE TIPO C INTEGRAL |
| 04303 | LEITE TIPO C INTEGRAL ORGANICO |
| 03107 | LEITE TODDYNHO |
| 06807 | LEITE TODDYNHO DIET |
| 06817 | LEITE TODDYNHO DIETETICO |
| 06707 | LEITE TODDYNHO LIGHT |
| 03105 | LEITE VITAMINADO |
| 06805 | LEITE VITAMINADO DIET |
| 06815 | LEITE VITAMINADO DIETETICO |
| 06705 | LEITE VITAMINADO LIGHT |
| 03106 | LEITE VITAMINADO TODDYNHO |
| 06806 | LEITE VITAMINADO TODDYNHO DIET |
| 06816 | LEITE VITAMINADO TODDYNHO DIETETICO |
| 06706 | LEITE VITAMINADO TODDYNHO LIGHT |
| 00603 | LEITEIRA |
| 05201 | LENCINHO |
| 05202 | LENCO |
| 00902 | LENCO DE BEBE UMEDECIDO |
| 05101 | LENCO DE CABECA |
| 00901 | LENCO DE PAPEL |
| 05601 | LENCO DE PESCOCO |
| 00904 | LENCO DESCARTAVEL |
| 00903 | LENCO UMEDECIDO DE BEBE |
| 00402 | LENCOL |
| 01601 | LENCOL DESCARTAVEL |
| 09001 | LENHA PARA COMBUSTIVEL DOMESTICO |
| 01101 | LENHA PARA USO DOMESTICO |
| 04004 | LENTE DE CONTATO |
| 00401 | LENTE DE CONTATO COLORIDA |
| 04002 | LENTE DE OCULOS (DE GRAU) |
| 04119 | LENTE INTRAOCULAR |
| 04001 | LENTES DE OCULOS E DE CONTATO |
| 02901 | LENTILHA |
| 04201 | LENTILHA EM CONSERVA |
| 68102 | LEQUE (MOLUSCO) |
| 02701 | LEQUE (PECA DE DECORACAO) |
| 00701 | LEQUE (USO PESSOAL) |
| 02305 | LEVANTE (TEMPERO INDUSTRIALIZADO) |
| 03401 | LEVEDO DE CERVEJA |
| 00102 | LICENCA DE AUTOMOVEL |
| 01401 | LICENCA DE CACA E PESCA |
| 01602 | LICENCA DE CAMINHAO |
| 01502 | LICENCA DE EMBARCACAO |
| 01402 | LICENCA DE MOTO |
| 02301 | LICENCA DE RADIO AMADOR |
| 01402 | LICENCA PARA CACA E PESCA |
| 01704 | LICENCA PARA OBRA |
| 01704 | LICENCA PARA OBRA |
| 01701 | LICENCA PARA OBRA, PLANTA E DESEMBARACO DE DOCUMENTACAO |
| 01701 | LICENCA PARA OBRA, PLANTA E DESEMBARACO DE DOCUMENTACAO |
| 10001 | LICHIA |
| 01901 | LICOR DE QUALQUER SABOR |
| 01634 | LIMA |
| 01901 | LIMA |
| 01904 | LIMA COMUM |
| 01902 | LIMA DA PERSIA |
| 01903 | LIMA DE BICO |
| 02002 | LIMAO AMARGO |
| 02003 | LIMAO AZEDO |
| 02105 | LIMAO BALAO |
| 02104 | LIMAO BERGAMOTA |
| 02106 | LIMAO BOI |
| 02012 | LIMAO BRANCO |
| 02014 | LIMAO CAPETA |
| 02006 | LIMAO CHINA |
| 02001 | LIMAO COMUM |
| 10101 | LIMAO COMUM ORGANICO |
| 02011 | LIMAO CRAVO |
| 02004 | LIMAO DE MOLHO |
| 02101 | LIMAO GALEGO |
| 02013 | LIMAO HAWAI |
| 02009 | LIMAO MIRIM |
| 09301 | LIMAO NAO ESPECIFICADO |
| 02008 | LIMAO PAULISTA |
| 00404 | LIMAO REFRIGERANTE |
| 07504 | LIMAO REFRIGERANTE LIGHT |
| 02007 | LIMAO ROSA |
| 02005 | LIMAO SILVESTRE |
| 02010 | LIMAO TAITI |
| 02102 | LIMAO VERDADEIRO |
| 02103 | LIMAO VERMELHO |
| 02108 | LIMAO VINAGRE |
| 03307 | LIMPA ALUMINIO |
| 09303 | LIMPA ARDOSIA |
| 09301 | LIMPA CERAMICA |
| 09304 | LIMPA PISO |
| 06104 | LIMPA-CARPETE |
| 07001 | LIMPADOR DE CACHIMBO |
| 09302 | LIMPADOR DE CERAMICA |
| 02401 | LIMPADOR DE FOSSA |
| 00302 | LIMPADOR DE GARRAFA |
| 06310 | LIMPADOR DE MULTIPLA ACAO |
| 02101 | LIMPADOR DE PISCINA |
| 09305 | LIMPADOR DE PISO |
| 02003 | LIMPADOR DE TERRENO |
| 02001 | LIMPADOR DE TERRENO (CAPINADOR) |
| 06308 | LIMPADOR INSTANTANEO |
| 06312 | LIMPADOR MULTIUSO |
| 05003 | LIMPA-FOGAO |
| 06601 | LIMPA-FORNO |
| 00815 | LIMPA-PARA-BRISA |
| 06103 | LIMPA-TAPETE |
| 06501 | LIMPA-VIDROS |
| 07301 | LIMPEZA DE CARNE BOVINA |
| 08502 | LIMPEZA DE CARNE SUINA |
| 02506 | LIMPEZA DE DENTES DO CACHORRO |
| 00601 | LIMPEZA DE PELE |
| 02701 | LINGUA BOVINA |
| 02201 | LINGUA DE VACA (VERDURA) |
| 02207 | LINGUA DE VACA EM FOLHA |
| 04701 | LINGUA SUINA |
| 00404 | LINGUAFONE |
| 08801 | LINGUICA ASSADA PARA VIAGEM |
| 02206 | LINGUICA BOVINA |
| 05306 | LINGUICA BOVINA LIGHT |
| 02205 | LINGUICA CALABRESA |
| 05305 | LINGUICA CALABRESA LIGHT |
| 02207 | LINGUICA DE FRANGO |
| 05307 | LINGUICA DE FRANGO LIGHT |
| 02214 | LINGUICA DE PERNIL |
| 06601 | LINGUICA DE SOJA NO VAREJO |
| 02209 | LINGUICA DEFUMADA |
| 05309 | LINGUICA DEFUMADA LIGHT |
| 02901 | LINGUICA EM CONSERVA |
| 02202 | LINGUICA EM PACOTE |
| 05302 | LINGUICA EM PACOTE LIGHT |
| 02210 | LINGUICA MISTA |
| 05310 | LINGUICA MISTA LIGHT |
| 02204 | LINGUICA NAO-ESPECIFICADA |
| 05304 | LINGUICA NAO-ESPECIFICADA LIGHT |
| 02201 | LINGUICA NO VAREJO |
| 05301 | LINGUICA NO VAREJO LIGHT |
| 02203 | LINGUICA PARA CHURRASCO |
| 05303 | LINGUICA PARA CHURRASCO LIGHT |
| 02208 | LINGUICA SUINA |
| 02212 | LINGUICA SUINA DEFUMADA |
| 05308 | LINGUICA SUINA LIGHT |
| 02211 | LINGUICA TOSCANA |
| 05311 | LINGUICA TOSCANA LIGHT |
| 00102 | LINHA |
| 00509 | LINHA DE PESCA |
| 16502 | LINHA TELEFONICA COMUNITARIA (RAMAL) |
| 14901 | LINHA TELEFONICA RESIDENCIAL |
| 04701 | LINHACA DOURADA |
| 00601 | LIQUIDIFICADOR |
| 01401 | LIQUIDIFICADOR |
| 03902 | LIQUIDO PARA CONTRASTE (EXAME) |
| 30605 | LIQUIDO PARA LENTES DE CONTATO |
| 03903 | LIQUIDO PARA UROGRAFIA (EXAME) |
| 00406 | LIVRO DE CATECISMO |
| 00812 | LIVRO E REVISTA TECNICA |
| 00702 | LIVRO ESCOLAR DE 1º E 2º GRAU |
| 00701 | LIVRO ESCOLAR DE PRIMEIRO E SEGUNDO GRAU |
| 00810 | LIVRO PARADIDATICO |
| 00813 | LIVRO PRE-ESCOLAR |
| 00802 | LIVRO TECNICO |
| 00801 | LIVRO TECNICO, REVISTA TECNICA E OUTROS LIVROS DIDATICOS |
| 00401 | LIVROS NAO-DIDATICOS |
| 00604 | LIXA |
| 00907 | LIXA DE FERRO |
| 00820 | LIXA DE FERRO |
| 00303 | LIXA DE FOGAO |
| 00203 | LIXA DE LIMPEZA DE FOGAO |
| 00906 | LIXA DE MADEIRA |
| 00906 | LIXA DE MADEIRA |
| 00610 | LIXA DE PE |
| 14403 | LIXADEIRA ELETRICA |
| 00903 | LIXEIRA |
| 06301 | LIXEIRA METALICA |
| 07301 | LIXEIRA METALICA |
| 09901 | LIXEIRA METALICA |
| 01201 | LOCACAO DE ALOJAMENTO ESTUDANTIL |
| 01606 | LOCACAO DE BICICLETA (PASSEIO) |
| 03601 | LOCACAO DE BICICLETAS |
| 01608 | LOCACAO DE BOTE (PASSEIO) |
| 01602 | LOCACAO DE CAVALO (PASSEIO) |
| 01601 | LOCACAO DE CHARRETE (PASSEIO) |
| 02802 | LOCACAO DE CLUBE, TEATRO, ETC. PARA DIVERSAO |
| 01202 | LOCACAO DE QUARTO |
| 01203 | LOCACAO DE VAGA |
| 00301 | LOCACAO DO IMOVEL (CONTRATO, DEPOSITOS DE LOCACAO E CONSERVACAO, ETC.) |
| 02101 | LOCAO APOS BARBA |
| 00412 | LOCAO DE CABELO |
| 00503 | LOCAO DE PELE |
| 02615 | LOCAO DERMATOLOGICA |
| 00421 | LOCAO TONICA OU TONIFICANTE CAPILAR |
| 03702 | LOMBINHO SUINO |
| 00802 | LOMBO BOVINO |
| 00805 | LOMBO BOVINO COM OSSO |
| 00801 | LOMBO DE PORCO DEFUMADO |
| 01601 | LOMBO DE PORCO SALGADO |
| 00602 | LOMBO DOS MOCOS (LAGARTO REDONDO) |
| 11802 | LOMBO DOS MOCOS (LAGARTO REDONDO) ORGANICO |
| 10601 | LOMBO NAO-ESPECIFICADO |
| 00608 | LOMBO PAULISTA (CARNE BOVINA) |
| 11808 | LOMBO PAULISTA (CARNE BOVINA) ORGANICO |
| 03701 | LOMBO SUINO |
| 03703 | LOMBO SUINO RECHEADO E TEMPERADO |
| 04505 | LONA |
| 03601 | LONA |
| 02003 | LONA PARA ACAMPAMENTO |
| 02302 | LOTADA |
| 00401 | LOTE DOADO PELA PREFEITURA MUNICIPAL (OUTROS IMOVEIS) |
| 00101 | LOTECA |
| 00102 | LOTERIA ESPORTIVA |
| 00201 | LOTERIA FEDERAL E ESTADUAL |
| 01201 | LOTERIA INSTANTANEA |
| 02401 | LOTINHA |
| 01101 | LOTOFACIL |
| 02001 | LOTOGOL |
| 00301 | LOTOMANIA |
| 01102 | LOUCA (PECA DE DECORACAO) |
| 01601 | LOURO (TEMPERO INDUSTRIALIZADO) |
| 01603 | LOURO EM FOLHA |
| 01602 | LOURO EM PO |
| 00101 | LUBRIFICACAO (COM OU SEM LAVAGEM) |
| 00102 | LUBRIFICACAO COM LAVAGEM |
| 00103 | LUBRIFICACAO SEM LAVAGEM |
| 31005 | LUBRIFICANTE INTIMO |
| 30604 | LUBRIFICANTE PARA LENTE DE CONTATO |
| 65101 | LULA |
| 01001 | LUMINARIA, ABAJUR, LUSTRE, GLOBO DE LUZ, ETC. |
| 12502 | LUNETA |
| 12504 | LUPA |
| 03203 | LUSTRA MOVEL |
| 01003 | LUSTRE |
| 30107 | LUVA CIRURGICA |
| 01702 | LUVA DE COURO |
| 01602 | LUVA DE COURO |
| 02402 | LUVA DE COURO |
| 00118 | LUVA DE COURO (PARA GOLEIRO) |
| 01601 | LUVA DE COURO, MALHA, TECIDO |
| 01701 | LUVA DE COURO, MALHA,TECIDO |
| 02401 | LUVA DE COURO, MALHA,TECIDO |
| 08902 | LUVA DE LIMPEZA |
| 08901 | LUVA DE LIMPEZA DE BORRACHA |
| 08903 | LUVA DE LIMPEZA DE PLASTICO |
| 01703 | LUVA DE MALHA |
| 01603 | LUVA DE MALHA |
| 02403 | LUVA DE MALHA |
| 01704 | LUVA DE TECIDO |
| 01604 | LUVA DE TECIDO |
| 02404 | LUVA DE TECIDO |
| 01303 | LUVA MULTIUSO |
| 01302 | LUVA PARA SERVICOS DOMESTICOS |
| 00210 | LUZES (CABELEIREIRO) |
| 01404 | MACA |
| 03001 | MACA |
| 03002 | MACA ACIDA |
| 08602 | MACA ACIDA ORGANICA |
| 03007 | MACA ARGENTINA |
| 08607 | MACA ARGENTINA ORGANICA |
| 03004 | MACA BRANCA |
| 08604 | MACA BRANCA ORGANICA |
| 02414 | MACA DESIDRATADA |
| 03003 | MACA DOCE |
| 08603 | MACA DOCE ORGANICA |
| 03009 | MACA FUGI |
| 08609 | MACA FUGI ORGANICA |
| 03012 | MACA FUJI |
| 03010 | MACA GALA |
| 08610 | MACA GALA ORGANICA |
| 03011 | MACA IMPORTADA |
| 08611 | MACA IMPORTADA ORGANICA |
| 03008 | MACA NACIONAL |
| 08608 | MACA NACIONAL ORGANICA |
| 06504 | MACA ORGANICA |
| 08601 | MACA ORGANICA |
| 04102 | MACA REFRIGERANTE |
| 04002 | MACA REFRIGERANTE LIGHT |
| 02405 | MACA SECA OU DESIDRATADA |
| 03005 | MACA VERDE |
| 08605 | MACA VERDE ORGANICA |
| 03006 | MACA VERMELHA |
| 08606 | MACA VERMELHA ORGANICA |
| 01001 | MACACAO |
| 01001 | MACACAO |
| 01301 | MACACAO |
| 03101 | MACACAO DE MOTOQUEIRA |
| 02601 | MACACAO DE MOTOQUEIRO |
| 06709 | MACACO VIVO |
| 01601 | MACADAMIA |
| 01212 | MACAIBA |
| 01214 | MACAJUBA |
| 03802 | MACAQUINHO |
| 03801 | MACAQUITO |
| 03901 | MACAQUITO |
| 01632 | MACARICO DE SOLDAR |
| 03401 | MACARRAO (NAO-ESPECIFICADO) |
| 03402 | MACARRAO CASEIRO |
| 03219 | MACARRAO COM CENOURA |
| 03218 | MACARRAO COM ESPINAFRE |
| 03220 | MACARRAO COM MILHO |
| 00918 | MACARRAO COM MOLHO PARA VIAGEM |
| 03301 | MACARRAO COM OVOS |
| 03403 | MACARRAO COM SEMOLA (NAO-ESPECIFICADO) |
| 03411 | MACARRAO COM SEMOLA NAO-ESPECIFICADO |
| 03302 | MACARRAO DE GLUTEN COM OVOS |
| 03202 | MACARRAO DE GLUTEN SEM OVOS |
| 03303 | MACARRAO DE SEMOLA COM OVOS |
| 03203 | MACARRAO DE SEMOLA SEM OVOS |
| 03304 | MACARRAO DE SEMOLINA COM OVOS |
| 03204 | MACARRAO DE SEMOLINA SEM OVOS |
| 03305 | MACARRAO DE SOPA COM OVOS |
| 03205 | MACARRAO DE SOPA SEM OVOS |
| 04802 | MACARRAO INSTANTANEO |
| 06302 | MACARRAO INSTANTANEO LIGHT |
| 04901 | MACARRAO INTEGRAL |
| 03410 | MACARRAO NAO-ESPECIFICADO |
| 02903 | MACARRAO PARA LASANHA |
| 03405 | MACARRAO PARAFUSO (NAO-ESPECIFICADO) |
| 03315 | MACARRAO PARAFUSO COM OVOS |
| 03413 | MACARRAO PARAFUSO NAO-ESPECIFICADO |
| 03217 | MACARRAO PARAFUSO SEM OVOS |
| 03222 | MACARRAO PASTEURIZADO |
| 03316 | MACARRAO PENNE COM OVOS |
| 03224 | MACARRAO PENNE SEM OVOS |
| 03223 | MACARRAO PICADO |
| 03221 | MACARRAO PRE-COZIDO |
| 08012 | MACARRAO PRONTO LIGHT PARA VIAGEM |
| 00912 | MACARRAO PRONTO PARA VIAGEM |
| 03215 | MACARRAO SEM COLESTEROL |
| 03201 | MACARRAO SEM OVOS |
| 06901 | MACARRAO SEMI PRONTO |
| 03216 | MACARRAO VITAMINADO |
| 00919 | MACARRONADA COM QUEIJO |
| 01211 | MACAUBA |
| 00610 | MACAXEIRA |
| 00216 | MACAXEIRA FRITA PARA VIAGEM |
| 01639 | MACHADO |
| 04105 | MACHUCHO (CHUCHU) |
| 04102 | MACHUCHU (CHUCHU) |
| 02301 | MACONARIA |
| 01601 | MACONHA |
| 63105 | MACUNIM |
| 00406 | MADEIRA |
| 00406 | MADEIRA |
| 02402 | MADEIRA (PECA DE DECORACAO) |
| 07706 | MADEIRA COM CERTIFICADO (SELO FSC) |
| 08606 | MADEIRA COM CERTIFICADO (SELO FSC) |
| 00401 | MADEIRA E TACO |
| 00401 | MADEIRA E TACO |
| 07701 | MADEIRA E TACO DE MADEIRA CERTIFICADA (SELO FSC) |
| 08601 | MADEIRA E TACO DE MADEIRA CERTIFICADA (SELO FSC) |
| 01303 | MAGIPAC |
| 01103 | MAIO |
| 00503 | MAIO |
| 01101 | MAIO E BIQUINI |
| 04301 | MAIONESE |
| 04303 | MAIONESE COM ATUM |
| 10403 | MAIONESE COM ATUM LIGHT |
| 04304 | MAIONESE COM AZEITONA |
| 10404 | MAIONESE COM AZEITONA LIGHT |
| 04302 | MAIONESE COM LIMAO |
| 10402 | MAIONESE COM LIMAO LIGHT |
| 04307 | MAIONESE DE LEITE |
| 04306 | MAIONESE DE QUALQUER SABOR |
| 10406 | MAIONESE DE QUALQUER SABOR LIGHT |
| 10401 | MAIONESE LIGHT |
| 00701 | MAIZENA |
| 04510 | MAL- CASADO |
| 00501 | MALA |
| 00123 | MALA DIRETA |
| 01602 | MALAGUETA EM CONSERVA |
| 04603 | MALAGUETA IN NATURA |
| 00103 | MALETA DE HOMEM |
| 00603 | MALHA DE GINASTICA |
| 00703 | MALHA DE GINASTICA |
| 00116 | MALVA |
| 30416 | MAMADEIRA |
| 03101 | MAMAO |
| 03110 | MAMAO BAIANO |
| 03102 | MAMAO DA INDIA |
| 03114 | MAMAO DE CORDA |
| 03106 | MAMAO DO AMAZONAS |
| 03111 | MAMAO FORMOSA |
| 03105 | MAMAO HAVAI |
| 03112 | MAMAO JAPONES |
| 03103 | MAMAO MACHO |
| 03107 | MAMAO PAPAIA |
| 03113 | MAMAO PAULISTA |
| 03104 | MAMAO VERDE |
| 03109 | MAMAO VERMELHO |
| 00303 | MAMINHA |
| 11503 | MAMINHA ORGANICA |
| 01402 | MAMOGRAFIA |
| 02302 | MAMY |
| 05202 | MAMY LIGHT |
| 00601 | MANDIOCA |
| 00604 | MANDIOCA BRANCA |
| 00606 | MANDIOCA BRAVA |
| 00605 | MANDIOCA CACAU |
| 00703 | MANDIOCA CONGELADA PARA VIAGEM |
| 00805 | MANDIOCA COZIDA PARA VIAGEM |
| 00602 | MANDIOCA DOCE |
| 00603 | MANDIOCA MANSA |
| 01305 | MANDIOCA PARA ANIMAL NAO-ESPECIFICADO |
| 00613 | MANDIOCA SEM CASCA |
| 00607 | MANDIOCA VASSOURINHA |
| 00908 | MANDIOQUINHA |
| 00303 | MANDIOQUINHA SALSA (BATATA BAROA) |
| 05903 | MANDOLATE |
| 01405 | MANGA |
| 03201 | MANGA |
| 03212 | MANGA ABOBORA |
| 03216 | MANGA BACURI |
| 03210 | MANGA CARLOTINHA |
| 03217 | MANGA CATANA |
| 03209 | MANGA COITE |
| 03202 | MANGA COQUINHO |
| 03205 | MANGA CORACAO DE BOI |
| 03218 | MANGA CORREIO |
| 03222 | MANGA DE CHEIRO |
| 03219 | MANGA DE FIAPO |
| 03223 | MANGA DE MESA |
| 03211 | MANGA ESPADA |
| 03203 | MANGA ESPADINHA |
| 03224 | MANGA HADEN |
| 03206 | MANGA ITAMARACA |
| 03214 | MANGA JASMIM |
| 03220 | MANGA OURO |
| 03225 | MANGA PALMER |
| 03226 | MANGA RACHA |
| 03208 | MANGA ROSA |
| 03207 | MANGA ROSINHA |
| 03213 | MANGA SAPATINHA |
| 03221 | MANGA TOMMY |
| 03204 | MANGA UBA |
| 05601 | MANGABA |
| 05603 | MANGABINHA |
| 05602 | MANGABINHA DO NORTE |
| 01204 | MANGALO AMARGO EM GRAO |
| 09401 | MANGERICAO |
| 02105 | MANGERICAO (TEMPERO INDUSTRIALIZADO) |
| 02103 | MANGERICAO DE MOLHO (TEMPERO INDUSTRIALIZADO) |
| 09402 | MANGERICAO ROXO |
| 07902 | MANGOSTAO |
| 07901 | MANGOSTIM |
| 01657 | MANGUEIRA DE NIVEL |
| 01401 | MANGUEIRA DE PLASTICO OU BORRACHA |
| 03215 | MANGUITO |
| 07201 | MANICOBA PARA VIAGEM |
| 00304 | MANICURO |
| 00301 | MANICURO E PEDICURO |
| 04201 | MANILHA |
| 04201 | MANILHA |
| 00608 | MANIVA |
| 02701 | MANJAR |
| 11101 | MANJAR DIET |
| 11102 | MANJAR DIETETICO |
| 09603 | MANJAR LIGHT |
| 11001 | MANJAR LIGHT |
| 09501 | MANJERONA |
| 02504 | MANJERONA DESIDRATADA |
| 02506 | MANJERONA INGLESA DESIDRATADA |
| 02503 | MANJERONA INGLESA SECA |
| 02501 | MANJERONA SECA |
| 02505 | MANJERONA VERDADEIRA DESIDRATADA |
| 02502 | MANJERONA VERDADEIRA SECA |
| 02204 | MANJONGOME |
| 01804 | MANTA |
| 00403 | MANTA DE CAMA |
| 00410 | MANTA PARA SOFA |
| 01507 | MANTEIGA |
| 01508 | MANTEIGA CASEIRA |
| 01501 | MANTEIGA COM OU SEM SAL |
| 07101 | MANTEIGA COM OU SEM SAL LIGHT |
| 01502 | MANTEIGA COM SAL |
| 07102 | MANTEIGA COM SAL LIGHT |
| 01504 | MANTEIGA DA TERRA |
| 07104 | MANTEIGA DA TERRA LIGHT |
| 30803 | MANTEIGA DE CACAU |
| 01505 | MANTEIGA DE GARRAFA |
| 07105 | MANTEIGA DE GARRAFA LIGHT |
| 01506 | MANTEIGA DE LEITE |
| 07106 | MANTEIGA DE LEITE LIGHT |
| 01509 | MANTEIGA DO SERTAO |
| 01503 | MANTEIGA SEM SAL |
| 07103 | MANTEIGA SEM SAL LIGHT |
| 08401 | MANTEIGUEIRA |
| 00814 | MANUAL DE VESTIBULAR |
| 00807 | MANUAL DIDADITO |
| 02406 | MANUTENCAO DE APARELHO DENTARIO |
| 02407 | MANUTENCAO DE APARELHO ORTODONTICO |
| 00801 | MANUTENCAO DE APARELHOS DOMESTICOS |
| 01307 | MANUTENCAO DE CAIXA DE AGUA |
| 02802 | MANUTENCAO DE CONTA BANCARIA |
| 01310 | MANUTENCAO DE FOSSA SEPTICA |
| 00804 | MANUTENCAO DE HIDROMETRO |
| 07301 | MANUTENCAO DE JAZIGO |
| 00401 | MANUTENCAO DE MOVEIS |
| 01301 | MANUTENCAO DE UTENSILIOS DOMESTICOS ELETRICOS |
| 01201 | MANUTENCAO DE UTENSILIOS DOMESTICOS NAO-ELETRICOS |
| 03105 | MAO BOVINA |
| 03107 | MAO DE VACA |
| 01301 | MAO-DE-OBRA (PEDREIRO, MARCENEIRO, ELETRICISTA, PINTOR, ETC.) |
| 01301 | MAO-DE-OBRA (PEDREIRO, MARCENEIRO, ELETRICISTA, PINTOR, ETC.) |
| 02901 | MAPA ESCOLAR |
| 01101 | MAQUIAGEM |
| 00230 | MAQUIAGEM DE BRINQUEDO |
| 11301 | MAQUINA DE ALGODAO |
| 11001 | MAQUINA DE BATER ACAI ELETRICA |
| 18601 | MAQUINA DE BATER ACAI MANUAL |
| 18602 | MAQUINA DE BATER ALIMENTOS MANUAL |
| 11501 | MAQUINA DE BATER SABAO ELETRICA |
| 01501 | MAQUINA DE CACA NIQUEL |
| 21401 | MAQUINA DE CORTAR CABELO ELETRICA |
| 11201 | MAQUINA DE CORTAR PISO |
| 01901 | MAQUINA DE COSTURA |
| 00601 | MAQUINA DE COSTURA (ALUGUEL) |
| 04301 | MAQUINA DE COSTURA A PEDAL |
| 04401 | MAQUINA DE COSTURA ELETRICA |
| 14201 | MAQUINA DE COSTURA MANUAL |
| 04501 | MAQUINA DE COSTURA NAO-ESPECIFICADA |
| 11401 | MAQUINA DE CREPE |
| 13101 | MAQUINA DE ESCREVER ELETRICA |
| 01501 | MAQUINA DE ESCREVER ELETRICA (ALUGUEL) |
| 13001 | MAQUINA DE ESCREVER MECANICA |
| 11601 | MAQUINA DE ESTAMPAR |
| 08801 | MAQUINA DE FAZER PAO |
| 03201 | MAQUINA DE LAVAR LOUCAS |
| 02201 | MAQUINA DE LAVAR PEDRAS (ALUGUEL) |
| 00701 | MAQUINA DE LAVAR PRATOS |
| 01201 | MAQUINA DE LAVAR ROUPAS |
| 02101 | MAQUINA DE LAVAR ROUPAS |
| 01501 | MAQUINA DE MOER CARNE ELETRICA |
| 14601 | MAQUINA DE MOER CARNE MANUAL |
| 18801 | MAQUINA DE MOER MILHO MANUAL |
| 09201 | MAQUINA DE OVERLOCK |
| 11701 | MAQUINA DE POLIR |
| 14501 | MAQUINA DE PRESSAO PARA BOTAO |
| 11801 | MAQUINA DE RISCAR CERAMICA |
| 02901 | MAQUINA DE SECAR ROUPAS |
| 02201 | MAQUINA DE SECAR ROUPAS |
| 11901 | MAQUINA DE TRICO |
| 12701 | MAQUINA FOTOGRAFICA |
| 16004 | MAQUINA PARA MACARRAO |
| 16002 | MAQUINA PARA MASSAS |
| 18802 | MAQUINA PARA RALAR MANDIOCA MANUAL |
| 06902 | MAQUINA SELADORA |
| 15601 | MAQUINAS AGRICOLAS DE PEQUENO PORTE |
| 04001 | MARACA |
| 03301 | MARACUJA |
| 03314 | MARACUJA ACU |
| 03315 | MARACUJA AMARELO |
| 03327 | MARACUJA AZEDO |
| 03304 | MARACUJA AZUL |
| 03310 | MARACUJA BRANCO |
| 03303 | MARACUJA COMPRIDO |
| 03308 | MARACUJA COMUM |
| 03318 | MARACUJA DE BOI |
| 03307 | MARACUJA DE COBRA |
| 03309 | MARACUJA DOCE |
| 06402 | MARACUJA EM POLPA |
| 03326 | MARACUJA ENCARNADO |
| 03319 | MARACUJA ESCURO |
| 03320 | MARACUJA GRANDE |
| 03325 | MARACUJA LARANJA |
| 03316 | MARACUJA MAMAO |
| 03306 | MARACUJA MELAO |
| 03302 | MARACUJA MIRIM |
| 03312 | MARACUJA PEROBA |
| 03323 | MARACUJA PINTADO |
| 03321 | MARACUJA PRETO |
| 03305 | MARACUJA REDONDO |
| 03322 | MARACUJA ROXO |
| 03313 | MARACUJA SURURU |
| 03311 | MARACUJA SUSPIRO |
| 03324 | MARACUJA VERMELHO |
| 03317 | MARACUJAZINHO |
| 03328 | MARACUJINA EM FRUTO |
| 01107 | MARAJA |
| 02403 | MARATHON |
| 04401 | MARCELA CHA |
| 01303 | MARCENEIRO |
| 01303 | MARCENEIRO |
| 00104 | MARGARIDA |
| 01609 | MARGARINA |
| 01602 | MARGARINA COM OU SEM SAL |
| 04102 | MARGARINA COM OU SEM SAL DIET |
| 04110 | MARGARINA COM OU SEM SAL DIETETICA |
| 04202 | MARGARINA COM OU SEM SAL LIGHT |
| 01603 | MARGARINA COM SAL |
| 04103 | MARGARINA COM SAL DIET |
| 04111 | MARGARINA COM SAL DIETETICA |
| 04203 | MARGARINA COM SAL LIGHT |
| 01604 | MARGARINA DE MILHO |
| 04104 | MARGARINA DE MILHO DIET |
| 04112 | MARGARINA DE MILHO DIETETICA |
| 04204 | MARGARINA DE MILHO LIGHT |
| 01605 | MARGARINA DE SOJA |
| 04105 | MARGARINA DE SOJA DIET |
| 04113 | MARGARINA DE SOJA DIETETICA |
| 04205 | MARGARINA DE SOJA LIGHT |
| 01606 | MARGARINA SEM SAL |
| 04106 | MARGARINA SEM SAL DIET |
| 04114 | MARGARINA SEM SAL DIETETICA |
| 04206 | MARGARINA SEM SAL LIGHT |
| 01601 | MARGARINA VEGETAL COM OU SEM SAL |
| 04101 | MARGARINA VEGETAL COM OU SEM SAL DIET |
| 04109 | MARGARINA VEGETAL COM OU SEM SAL DIETETICA |
| 01607 | MARGARINA VEGETAL COM SAL |
| 04107 | MARGARINA VEGETAL COM SAL DIET |
| 04115 | MARGARINA VEGETAL COM SAL DIETETICA |
| 04207 | MARGARINA VEGETAL COM SAL LIGHT |
| 01608 | MARGARINA VEGETAL SEM SAL |
| 04108 | MARGARINA VEGETAL SEM SAL DIET |
| 04116 | MARGARINA VEGETAL SEM SAL DIETETICA |
| 04208 | MARGARINA VEGETAL SEM SAL LIGHT |
| 04201 | MARGARINAVEGETAL COM OU SEM SAL LIGHT |
| 07501 | MARI |
| 07502 | MARI AMARELO |
| 07503 | MARI ROXO |
| 02203 | MARIA GOMBE |
| 02206 | MARIA GOMES |
| 02202 | MARIA GORDA |
| 02357 | MARIA MALUCA |
| 02335 | MARIA MALUCA (BOLACHA DOCE) |
| 10535 | MARIA MALUCA (BOLACHA DOCE) DIET |
| 10575 | MARIA MALUCA (BOLACHA DOCE) DIETETICA |
| 10435 | MARIA MALUCA (BOLACHA DOCE) LIGHT |
| 03001 | MARIA MOLE |
| 07602 | MARIA MOLE DIET |
| 12901 | MARIA MOLE DIET |
| 07603 | MARIA MOLE DIETETICA |
| 12903 | MARIA MOLE DIETETICA |
| 01008 | MARICA |
| 02227 | MARICOTA |
| 03002 | MARINHEIRA |
| 03001 | MARINHEIRO |
| 01224 | MARIOLA |
| 63101 | MARISCO |
| 63401 | MARISCO SALGADO |
| 63201 | MARISCO SEM CASCA |
| 01206 | MARMELADA |
| 00818 | MARMITA DE ALUMINIO |
| 11101 | MARMITA ELETRICA |
| 00102 | MARMITA PARA VIAGEM |
| 00104 | MARMITEX PARA VIAGEM |
| 03102 | MARMORE (PEDRA) PARA PIA |
| 03102 | MARMORE (PEDRA) PARA PIA |
| 05105 | MAROLO |
| 06001 | MARRECO ABATIDO, CONGELADO OU VIVO |
| 01626 | MARRETA |
| 02209 | MARSHMALLOW |
| 01605 | MARTELO |
| 01501 | MARTINI |
| 00504 | MASCARA DE BELEZA |
| 00701 | MASCARA DE CARNAVAL |
| 01801 | MASCARA PROTETORA (PROTECAO PROFISSIONAL) |
| 03409 | MASSA (NAO-ESPECIFICADA) |
| 08011 | MASSA CASEIRA PRONTA LIGHT PARA VIAGEM |
| 00911 | MASSA CASEIRA PRONTA PARA VIAGEM |
| 03306 | MASSA COM OVOS |
| 00902 | MASSA CORRIDA PARA PAREDE |
| 00902 | MASSA CORRIDA PARA PAREDE |
| 00411 | MASSA DE ALHO |
| 00202 | MASSA DE ARARUTA |
| 00106 | MASSA DE ARROZ |
| 00403 | MASSA DE AVEIA |
| 00532 | MASSA DE BANANA |
| 04504 | MASSA DE BEIJU |
| 03405 | MASSA DE BOLO |
| 02102 | MASSA DE BURITI |
| 04401 | MASSA DE CALZONE |
| 03001 | MASSA DE CANELONE |
| 00610 | MASSA DE CANJICA |
| 03101 | MASSA DE CAPELETI |
| 04201 | MASSA DE COXINHA |
| 04203 | MASSA DE CROQUETE |
| 02909 | MASSA DE CUSCUZ |
| 07001 | MASSA DE EMPADA (EMPADA SEMIPRONTA) |
| 05901 | MASSA DE FIXACAO E VEDACAO DE VIDRO |
| 05901 | MASSA DE FIXACAO E VEDACAO DE VIDRO |
| 03307 | MASSA DE GLUTEN COM OVOS |
| 03207 | MASSA DE GLUTEN SEM OVOS |
| 00824 | MASSA DE LANTERNAGEM |
| 02901 | MASSA DE LASANHA |
| 06201 | MASSA DE LASANHA LIGHT |
| 00614 | MASSA DE MACAXEIRA |
| 01414 | MASSA DE MANDIOCA |
| 00607 | MASSA DE MILHO |
| 00245 | MASSA DE MODELAR |
| 02701 | MASSA DE NHOQUE |
| 06101 | MASSA DE NHOQUE LIGHT |
| 06701 | MASSA DE PANQUECA |
| 02201 | MASSA DE PAO COMUM |
| 02301 | MASSA DE PAO DE QUEIJO |
| 02601 | MASSA DE PASTEL |
| 06301 | MASSA DE PEIXE |
| 02401 | MASSA DE PIZZA |
| 06001 | MASSA DE PIZZA LIGHT |
| 00616 | MASSA DE PUBA |
| 04202 | MASSA DE QUIBE |
| 04201 | MASSA DE QUIBE, CROQUETE, SALGADINHO DE PEIXE, ETC. |
| 02801 | MASSA DE RAVIOLE |
| 04301 | MASSA DE ROTOLONE |
| 04204 | MASSA DE SALGADINHO DE PEIXE |
| 03407 | MASSA DE SEMOLA (NAO-ESPECIFICADA) |
| 03308 | MASSA DE SEMOLA COM OVOS |
| 03415 | MASSA DE SEMOLA NAO-ESPECIFICADA |
| 03208 | MASSA DE SEMOLA SEM OVOS |
| 03309 | MASSA DE SEMOLINA COM OVOS |
| 03209 | MASSA DE SEMOLINA SEM OVOS |
| 03602 | MASSA DE SOJA |
| 03408 | MASSA DE SOPA (NAO-ESPECIFICADA) |
| 03310 | MASSA DE SOPA COM OVOS |
| 03416 | MASSA DE SOPA NAO-ESPECIFICADA |
| 03210 | MASSA DE SOPA SEM OVOS |
| 00503 | MASSA DE SORVETE |
| 09703 | MASSA DE SORVETE DIET |
| 09708 | MASSA DE SORVETE DIETETICA |
| 09603 | MASSA DE SORVETE LIGHT |
| 01519 | MASSA DE TAPIOCA |
| 04701 | MASSA DE TOMATE |
| 00904 | MASSA DE TRIGO |
| 05602 | MASSA DE VEDACAO DE TELHA |
| 05602 | MASSA DE VEDACAO DE TELHA |
| 02808 | MASSA DOCE NAO-ESPECIFICADA |
| 03403 | MASSA FOLHADA |
| 04803 | MASSA INSTANTANEA |
| 06303 | MASSA INSTANTANEA LIGHT |
| 11302 | MASSA LIGHT |
| 03417 | MASSA NAO-ESPECIFICADA |
| 03404 | MASSA PARA BOLO |
| 02904 | MASSA PARA LASANHA |
| 02807 | MASSA PARA MINGAU NAO-ESPECIFICADA |
| 00617 | MASSA PARA PAMONHA |
| 02605 | MASSA PARA PASTEL |
| 02404 | MASSA PARA PIZZA |
| 04503 | MASSA PARA POLENTA |
| 07401 | MASSA PARA REJUNTE |
| 08001 | MASSA PRONTA LIGHT PARA VIAGEM |
| 00901 | MASSA PRONTA PARA VIAGEM |
| 03206 | MASSA SEM OVOS |
| 08601 | MASSAGEADOR ELETRICO |
| 03401 | MASSAGEM |
| 00103 | MASSAGEM (BARBEIRO) |
| 00203 | MASSAGEM (CABELEIREIRO) |
| 01201 | MASSAGEM E SAUNA |
| 03301 | MASSAGEM PARA FINS DE SAUDE SHIATZU DO IN E OUTROS TRATAMENTOS ALTERNATIVOS |
| 03402 | MASSAS INDUSTRIAIS DE BOLOS, TORTAS, BISCOITOS, ETC. |
| 03302 | MASSAS INDUSTRIAIS DE PAES |
| 00216 | MASSINHA DOCE (PAO DOCE) |
| 10016 | MASSINHA DOCE (PAO DOCE) DIET |
| 10056 | MASSINHA DOCE (PAO DOCE) DIETETICO |
| 10103 | MASTRUCO |
| 10101 | MASTRUZ |
| 01303 | MATAMBRE |
| 01104 | MATE |
| 01103 | MATE COURO |
| 13901 | MATE COURO DIET |
| 13506 | MATE COURO LIGHT |
| 08103 | MATE COURO LIGHT |
| 02802 | MATE ERVA |
| 12802 | MATE ERVA DIET |
| 12805 | MATE ERVA DIETETICO |
| 12702 | MATE ERVA LIGHT |
| 13002 | MATE ERVA ORGANICO |
| 08104 | MATE LIGHT |
| 01105 | MATE LIMAO |
| 08105 | MATE LIMAO LIGHT |
| 01106 | MATE PESSEGO |
| 08106 | MATE PESSEGO LIGHT |
| 31201 | MATERIAIS PARA CUIDADOS COM IDOSOS |
| 00501 | MATERIAL DE CACA E PESCA |
| 30301 | MATERIAL DE INJECAO |
| 00507 | MATERIAL DE PESCA |
| 00901 | MATERIAL DE PINTURA EM GERAL |
| 00901 | MATERIAL DE PINTURA EM GERAL |
| 08501 | MATERIAL ESCOLAR (AGREGADO) |
| 01003 | MATERIAL HIDRAULICO |
| 01003 | MATERIAL HIDRAULICO |
| 30901 | MATERIAL PARA APOIO TERAPEUTICO |
| 30501 | MATERIAL PARA AUTODIAGNOSTICO |
| 30401 | MATERIAL PARA CUIDADO COM BEBE |
| 30101 | MATERIAL PARA CURATIVO |
| 30601 | MATERIAL PARA HIGIENE DE LENTE DE CONTATO |
| 01202 | MATERIAL PARA INSTALACAO ELETRICA |
| 01202 | MATERIAL PARA INSTALACAO ELETRICA |
| 04501 | MATERIAL PLASTICO PARA COBERTURA |
| 04501 | MATERIAL PLASTICO PARA COBERTURA |
| 05301 | MATRICULA ESCOLAR |
| 00405 | MAUSA (EXCETO DE CACA) |
| 04301 | MAXIXE |
| 04303 | MAXIXE BRAVO |
| 04304 | MAXIXE DO MATO |
| 04302 | MAXIXO |
| 04305 | MAXIXOLA |
| 00503 | MECANICO (MAO-DE-OBRA) |
| 00406 | MEDALHAO DE FRANGO |
| 02404 | MEDICAMENTO PARA ANIMAL DOMESTICO |
| 04901 | MEDICAMENTO PARA GENGIVA DE BEBE |
| 01004 | MEDUBIM |
| 01801 | MEGA SENA |
| 07703 | MEGAFONE |
| 04115 | MEIA ELASTICA (PARA VARIZES) |
| 01202 | MEIA-CALCA |
| 01402 | MEIA-CALCA |
| 02401 | MEIAO (UNIFORME DE ESPORTE) |
| 02801 | MEIAO (UNIFORME DE ESPORTE) |
| 03101 | MEIAO (UNIFORME DE ESPORTE) |
| 01101 | MEIAS |
| 01201 | MEIAS |
| 01401 | MEIAS |
| 01602 | MEL |
| 01608 | MEL COM AGRIAO |
| 01603 | MEL COM ALHO |
| 01604 | MEL COM PROPOLIS |
| 01605 | MEL COMPOSTO |
| 01601 | MEL DE ABELHA |
| 09001 | MEL DE ABELHA ORGANICO |
| 01606 | MEL DE ENGENHO |
| 01607 | MEL DE ROMA |
| 00208 | MEL MEDICINAL (QUALQUER TIPO) |
| 09002 | MEL ORGANICO |
| 09003 | MEL ORGANICO COM ALHO |
| 09004 | MEL ORGANICO COM PROPOLIS |
| 09005 | MEL ORGANICO COMPOSTO |
| 09006 | MEL ORGANICO DE ENGENHO |
| 09007 | MEL ORGANICO DE ROMA |
| 01501 | MELADO |
| 01502 | MELADO DE CANA |
| 03401 | MELANCIA |
| 03501 | MELAO |
| 03502 | MELAO AMARELO |
| 03506 | MELAO CAIPIRA |
| 03507 | MELAO COLONIAL |
| 06405 | MELAO EM POLPA |
| 03503 | MELAO ESPANHOL |
| 03504 | MELAO GAUCHO |
| 03505 | MELAO JAPONES |
| 03508 | MELAO ORANGE |
| 03511 | MELAO PELE DE SAPO |
| 03509 | MELAO PINGO DE MEL |
| 03510 | MELAO VALENCIANO |
| 01003 | MENDOBIM |
| 01002 | MENDOIM |
| 02702 | MENSALIDADE DE BIBLIOTECA |
| 00301 | MENSALIDADE DE CLINICA |
| 00504 | MENSALIDADE DE CLUBE |
| 05302 | MENSALIDADE DE TITULO DE TURISMO |
| 00602 | MENTEX |
| 09902 | MENTEX DIET |
| 09915 | MENTEX DIETETICO |
| 09802 | MENTEX LIGHT |
| 02249 | MENTIRA (BISCOITO SALGADO) |
| 02363 | MENTIRA DOCE |
| 10102 | MENTRUZ |
| 30204 | MERCURIO CROMO |
| 05902 | MERENDA DA ESCOLA |
| 02001 | MERENDEIRA ESCOLAR |
| 02004 | MERENGUE |
| 10902 | MERGULHAO |
| 05401 | MERITENE |
| 30208 | MERTHIOLATE |
| 04501 | MESA (COPA E COZINHA) |
| 01504 | MESA (ESTAR E JANTAR) |
| 08301 | MESA (JARDIM, VARANDA, ETC.) |
| 01501 | MESA (SALA) |
| 08401 | MESA COM CADEIRAS (JARDIM, VARANDA, ETC.) |
| 08402 | MESA COM CADEIRAS DE JARDIM |
| 08403 | MESA COM CADEIRAS DE VARANDA |
| 08302 | MESA DE JARDIM |
| 00216 | MESA DE PINGUE-PONGUE |
| 00214 | MESA DE SINUCA |
| 08901 | MESA DE TELEFONE |
| 00215 | MESA DE TOTO |
| 08303 | MESA DE VARANDA |
| 04801 | MESA E BANCOS NAO-ESPECIFICADOS |
| 04401 | MESA E CADEIRAS (COPA E COZINHA) |
| 00802 | MESA E CADEIRAS (ESTAR E JANTAR) |
| 00801 | MESA E CADEIRAS (SALA) |
| 05001 | MESA E CADEIRAS NAO-ESPECIFICADAS |
| 00232 | MESA INFANTIL |
| 05101 | MESA NAO-ESPECIFICADA |
| 01105 | MESA PARA COMPUTADOR |
| 01106 | MESA PARA MICROCOMPUTADOR |
| 01103 | MESA PARA SOM |
| 01102 | MESA PARA TV |
| 01101 | MESA PARA TV, SOM, VIDEO E MICROCOMPUTADOR |
| 01104 | MESA PARA VIDEO |
| 02602 | MESADA DE NAO MORADOR |
| 02601 | MESADA DE NAO-MORADOR |
| 00401 | MESADA E PRESENTE EM DINHEIRO PARA OUTRA UC |
| 00403 | MESADA, DINHEIRO PARA OUTRA UC |
| 02902 | MESINHA DE CABECEIRA |
| 02901 | MESINHA DE CABECEIRA (QUARTO ADULTO) |
| 01401 | MESINHA DE CENTRO |
| 08903 | MESINHA DE TELEFONE |
| 08902 | MESINHA DE TELEFONE, DE VENTILADOR, ETC. |
| 08904 | MESINHA DE VENTILADOR |
| 06801 | MESINHA E CADEIRAS (MOVEIS INFANTIS) |
| 07001 | MESINHA INFANTIL |
| 02202 | MESTRADO |
| 01202 | METAL EXCETO PRATA (PECA DE DECORACAO) |
| 01625 | METRO |
| 00401 | METRO |
| 01401 | METRO |
| 02202 | MEXERICA |
| 02204 | MEXERICA RIO |
| 63102 | MEXILHAO |
| 02229 | MEXIRICA |
| 02224 | MEXIRICA CARIOCA |
| 02210 | MEXIRICA CRAVO |
| 02225 | MEXIRICA KAPUTIA |
| 02226 | MEXIRICA MARICOTA |
| 02206 | MEXIRICA MURKOT |
| 02214 | MEXIRICA PERA |
| 02208 | MEXIRICA POKAN |
| 02401 | MICROCOMPUTADOR |
| 06201 | MICROCOMPUTADOR |
| 01001 | MICROCOMPUTADOR (ALUGUEL) |
| 07704 | MICROFONE |
| 16701 | MICROSCOPIO |
| 00104 | MIGNON |
| 11304 | MIGNON ORGANICO |
| 01811 | MIJAOZINHO (CALCA DE MALHA PARA BEBE) |
| 03801 | MIL FOLHAS |
| 00604 | MILHARINA |
| 00707 | MILHENA NUTRIL 5 |
| 00712 | MILHO ALHO |
| 00710 | MILHO AMARELO EM GRAO |
| 03733 | MILHO ASSADO OU COZIDO |
| 01403 | MILHO ASSADO PARA VIAGEM |
| 00702 | MILHO BRANCO EM GRAO |
| 00704 | MILHO CANJIQUINHA |
| 01402 | MILHO COZIDO OU ASSADO PARA VIAGEM |
| 00703 | MILHO DE CANJICA |
| 00711 | MILHO DE MUNGUZA |
| 00705 | MILHO DE PIPOCA |
| 04202 | MILHO DE PIPOCA LIGHT PARA MICROONDAS |
| 03601 | MILHO DE PIPOCA PARA MICROONDAS |
| 04201 | MILHO DE PIPOCA PARA MICROONDAS LIGHT |
| 01503 | MILHO E ERVILHA EM CONSERVA |
| 00402 | MILHO EM CONSERVA |
| 00701 | MILHO EM GRAO |
| 00613 | MILHO MOIDO |
| 00710 | MILHO PARA FRANGO |
| 00705 | MILHO PARA GALINHA |
| 00713 | MILHO SECO |
| 01501 | MILHO VERDE COM ERVILHA EM CONSERVA |
| 01401 | MILHO VERDE COZIDO PARA VIAGEM |
| 01505 | MILHO VERDE E ERVILHA EM CONSERVA |
| 00401 | MILHO VERDE EM CONSERVA |
| 00801 | MILHO VERDE EM ESPIGA |
| 00714 | MILHO XEREM |
| 04506 | MILK SHAKE |
| 07501 | MILK SHAKE |
| 07502 | MILK SHAKE DE MORANGO |
| 12802 | MILK SHAKE DE MORANGO DIET |
| 12804 | MILK SHAKE DE MORANGO DIETETICO |
| 07003 | MILK SHAKE DIET |
| 12801 | MILK SHAKE DIET |
| 07004 | MILK SHAKE DIETETICO |
| 12803 | MILK SHAKE DIETETICO |
| 02218 | MIMOSA |
| 01003 | MINALBA |
| 00602 | MINEIRINHO |
| 07802 | MINEIRINHO LIGHT |
| 04320 | MINGAU |
| 00108 | MINGAU DE ARROZ |
| 00614 | MINGAU DE MILHO |
| 02301 | MINGAU PARA VIAGEM |
| 00408 | MINHOTO ALHO E SAL |
| 00140 | MINI BAGUETE |
| 00141 | MINI BENGALA |
| 04904 | MINI CHICKEN EMPANADO |
| 14202 | MINI MAQUINA DE COSTURA MANUAL |
| 00138 | MINI PAO |
| 00139 | MINI PAO FRANCES |
| 02603 | MINI PASTEL |
| 02403 | MINI PIZZA SEMIPRONTA |
| 06003 | MINI PIZZA SEMIPRONTA LIGHT |
| 01903 | MINI-BUG |
| 01201 | MINIGAME E AGENDA ELETRONICA INFANTIL |
| 00902 | MINUANO |
| 13503 | MINUANO LIGHT |
| 07902 | MINUANO LIGHT |
| 04801 | MIOJO |
| 06301 | MIOJO LIGHT |
| 02201 | MIOLO BOVINO |
| 00904 | MIOLO DA PA |
| 00910 | MIOLO DA PALETA |
| 00808 | MIOLO DE ACEM |
| 00809 | MIOLO DE AGULHA |
| 00309 | MIOLO DE ALCATRA |
| 11509 | MIOLO DE ALCATRA ORGANICO |
| 00909 | MIOLO DE PALETA |
| 01103 | MIOLO DE PEITO BOVINO |
| 04802 | MIRABEL |
| 08402 | MIRABEL DIET |
| 08415 | MIRABEL DIETETICO |
| 08302 | MIRABEL LIGHT |
| 00206 | MIRINDA |
| 13007 | MIRINDA LIGHT |
| 01506 | MIRINDA LIGHT |
| 00206 | MISSA |
| 06801 | MISSO |
| 00314 | MISTO FRIO OU FRIO PARA VIAGEM |
| 00409 | MISTO QUENTE OU FRIO |
| 00313 | MISTO QUENTE OU FRIO PARA VIAGEM |
| 00312 | MISTO-FRIO PARA VIAGEM |
| 00306 | MISTO-QUENTE OU FRIO PARA VIAGEM |
| 03406 | MISTURA DE BOLO |
| 02102 | MISTURA DE CAFE |
| 04302 | MISTURA DE CARNES SALGADAS DE FEIJOADA |
| 02801 | MISTURA INDUSTRIAL DE DOCE, SORVETE E PUDIM |
| 03407 | MISTURA PARA BOLO |
| 02202 | MISTURA PARA PAO DE TRIGO |
| 04504 | MISTURA PARA POLENTA |
| 03401 | MISTURAS INDUSTRIAIS DE BOLOS, TORTAS, BISCOITOS, ETC. |
| 03301 | MISTURAS INDUSTRIAIS DE PAES |
| 01805 | MIUDO BOVINO NAO-ESPECIFICADO |
| 08107 | MIUDO DE BODE NAO-ESPECIFICADO |
| 08103 | MIUDO DE CARNEIRO |
| 01002 | MIUDO DE FRANGO NAO-ESPECIFICADO |
| 01001 | MIUDO DE GALINHA OU FRANGO NAO-ESPECIFICADO |
| 02002 | MIUDO DE PORCO SALGADO |
| 03903 | MIUDO SUINO NAO-ESPECIFICADO |
| 04701 | MIX DE CEREAIS |
| 02203 | MIXIRICA |
| 02211 | MIXIRICA CRAVO |
| 02207 | MIXIRICA MURKOT |
| 02215 | MIXIRICA PERA |
| 02209 | MIXIRICA POKAN |
| 02205 | MIXIRICA RIO |
| 00902 | MOBI (APARELHO) |
| 01203 | MOBI (MENSALIDADE, ASSINATURA, ALUGUEL) |
| 02502 | MOBILE (ENFEITE DE BERCO) |
| 01401 | MOBILETE |
| 00904 | MOCA FESTA |
| 05504 | MOCA FESTA DIET |
| 05508 | MOCA FESTA DIETETICO |
| 05404 | MOCA FESTA LIGHT |
| 01216 | MOCAJUBA |
| 02501 | MOCHILA (EXCETO ESCOLAR) |
| 02101 | MOCHILA ESCOLAR |
| 03101 | MOCOTO BOVINO |
| 05801 | MOCOTO DE CAPRINO |
| 05203 | MOCOTO SUINO |
| 01501 | MODELADOR |
| 05103 | MODELADOR DE CABELO |
| 31110 | MODELADOR DE CORPO (GEL) |
| 01901 | MODERADOR DE APETITE |
| 00901 | MOEDA (HOBBY) |
| 12201 | MOEDOR DE CAFE ELETRICO |
| 01102 | MOELA DE FRANGO |
| 01101 | MOELA DE GALINHA OU FRANGO |
| 15602 | MOENDA DE CANA |
| 06403 | MOFIL |
| 01503 | MOLDURA DE QUADRO OU FOTO |
| 04902 | MOLHO A BOLONHESA |
| 09501 | MOLHO AGRIDOCE |
| 09601 | MOLHO AO SUGO |
| 09701 | MOLHO BARBECUE |
| 04901 | MOLHO BOLONHESA |
| 07201 | MOLHO BRANCO |
| 08101 | MOLHO DE ALHO |
| 09801 | MOLHO DE FRANGO |
| 06401 | MOLHO DE FUNGHI |
| 04201 | MOLHO DE HORTELA |
| 10801 | MOLHO DE HORTELA LIGHT |
| 04904 | MOLHO DE MACARRONADA |
| 04305 | MOLHO DE MAIONESE |
| 10405 | MOLHO DE MAIONESE LIGHT |
| 02107 | MOLHO DE MANGERICAO |
| 06701 | MOLHO DE MILHO VERDE |
| 09201 | MOLHO DE PIMENTA |
| 01802 | MOLHO DE PIMENTA NAO-ESPECIFICADA |
| 07001 | MOLHO DE PIZZA |
| 10702 | MOLHO DE SALADAS LIGHT |
| 03601 | MOLHO DE SOJA |
| 10501 | MOLHO DE SOJA LIGHT |
| 04801 | MOLHO DE TOMATE |
| 04903 | MOLHO DE TOMATE COM CARNE |
| 02001 | MOLHO DE TOMATE COM ERVAS |
| 10901 | MOLHO DE TOMATE LIGHT |
| 04101 | MOLHO DE TUCUPI COM PIMENTA |
| 03701 | MOLHO INGLES |
| 03603 | MOLHO JAPONES |
| 10503 | MOLHO JAPONES LIGHT |
| 09301 | MOLHO MADEIRA |
| 08001 | MOLHO NAO-ESPECIFICADO |
| 09901 | MOLHO PARA CARNE |
| 07002 | MOLHO PARA PIZZA |
| 09401 | MOLHO PARA SALADA |
| 07503 | MOLHO QUATRO QUEIJOS |
| 04806 | MOLHO REFOGADO DE TOMATE |
| 10906 | MOLHO REFOGADO DE TOMATE LIGHT |
| 03607 | MOLHO SHOYU |
| 10001 | MOLHO SIRIO |
| 03901 | MOLHO TARTARO |
| 10101 | MOLHO TEMPERADO PARMESAO |
| 05001 | MOLHO VEGETAL |
| 05101 | MOLHO XADREZ |
| 02405 | MONDONGO (TRIPA BOVINA) |
| 06315 | MONITOR PARA COMPUTADOR |
| 01601 | MONOCULO |
| 00107 | MONSENHOR |
| 02901 | MONTAGEM DE MOVEIS |
| 06401 | MOQUECA BAIANA PARA VIAGEM |
| 07101 | MOQUECA CAPIXABA PARA VIAGEM |
| 06402 | MOQUECA PARA VIAGEM |
| 03306 | MORANGA |
| 03308 | MORANGA CABUTIA |
| 03307 | MORANGA JAPONESA |
| 05201 | MORANGO |
| 01311 | MORANGO EM CALDA |
| 09219 | MORANGO EM CALDA DIET |
| 09220 | MORANGO EM CALDA DIETETICO |
| 06506 | MORANGO ORGANICO |
| 08801 | MORANGO ORGANICO |
| 05202 | MORANGUINHO |
| 08802 | MORANGUINHO ORGANICO |
| 02402 | MORCELA |
| 02403 | MORCILHA |
| 30405 | MORDEDOR DE BEBE |
| 30408 | MORDEDOR DE DENTICAO DE BEBE |
| 01001 | MORDOMO |
| 03502 | MORENINHA (TORTA) |
| 00121 | MOREY BOGGIES (PRANCHA DE SURFE) |
| 02231 | MORICOTA |
| 01644 | MORSA |
| 02601 | MORTADELA |
| 02607 | MORTADELA BOLONHESA |
| 05007 | MORTADELA BOLONHESA LIGHT |
| 02602 | MORTADELA BOVINA |
| 05002 | MORTADELA BOVINA LIGHT |
| 02608 | MORTADELA DE CHESTER |
| 05008 | MORTADELA DE CHESTER LIGHT |
| 02603 | MORTADELA DE FRANGO |
| 05003 | MORTADELA DE FRANGO LIGHT |
| 02609 | MORTADELA DE GALINHA |
| 05009 | MORTADELA DE GALINHA LIGHT |
| 02610 | MORTADELA DE PERU |
| 05010 | MORTADELA DE PERU LIGHT |
| 02611 | MORTADELA DEFUMADA |
| 05011 | MORTADELA DEFUMADA LIGHT |
| 04301 | MORTADELA EM CONSERVA |
| 06401 | MORTADELA EM CONSERVA LIGHT |
| 02605 | MORTADELA FATIADA NAO-ESPECIFICADA |
| 05005 | MORTADELA FATIADA NAO-ESPECIFICADA LIGHT |
| 12201 | MORTADELA LIGHT |
| 05001 | MORTADELA LIGHT |
| 02612 | MORTADELA MISTA |
| 05012 | MORTADELA MISTA LIGHT |
| 02604 | MORTADELA SUINA |
| 05004 | MORTADELA SUINA LIGHT |
| 01003 | MOSQUITEIRO |
| 01501 | MOSTARDA (TEMPERO INDUSTRIALIZADO) |
| 00801 | MOSTARDA (VERDURA) |
| 00802 | MOSTARDA EM FOLHA |
| 00703 | MOTEIS |
| 02501 | MOTEL |
| 00224 | MOTO DE BRINQUEDO |
| 02301 | MOTOCICLETA |
| 00401 | MOTOCICLETA |
| 02701 | MOTOR DE AUTOMOVEL |
| 04802 | MOTOR DE BOMBA DE AGUA |
| 10701 | MOTOR DE FREEZER |
| 00823 | MOTOR DE LANCHA |
| 10801 | MOTOR DE MAQUINA DE COSTURA |
| 00829 | MOTOR DE MOTOCICLETA |
| 07201 | MOTOR ELETRICO PARA PORTAO |
| 07901 | MOTOR ELETRICO PARA PORTAO |
| 00601 | MOTORISTA |
| 15608 | MOTOSSERRA |
| 02601 | MOTO-TAXI |
| 06308 | MOUSE PARA COMPUTADOR |
| 01002 | MOUSSE DE QUALQUER MARCA (GELEIA) |
| 07902 | MOUSSE DE QUALQUER MARCA (GELEIA) DIET |
| 07907 | MOUSSE DE QUALQUER MARCA (GELEIA) DIETETICA |
| 10602 | MOUSSE DE QUALQUER MARCA (GELEIA) LIGHT |
| 01003 | MOUSSE DE QUALQUER SABOR (GELEIA) |
| 07903 | MOUSSE DE QUALQUER SABOR (GELEIA) DIET |
| 07908 | MOUSSE DE QUALQUER SABOR (GELEIA) DIETETICA |
| 10603 | MOUSSE DE QUALQUER SABOR (GELEIA) LIGHT |
| 02810 | MOUSSE EM PO |
| 04901 | MOVEIS DE COPA E COZINHA NAO-ESPECIFICADOS |
| 07901 | MOVEIS DE QUARTO INFANTIL NAO-ESPECIFICADOS |
| 05601 | MOVEIS DE QUARTO NAO-ESPECIFICADOS |
| 05701 | MOVEIS NAO-ESPECIFICADOS |
| 05501 | MOVEIS PARA ESCRITORIO |
| 01901 | MOVEIS PARA SALA |
| 03601 | MOVEL DE SAPATOS |
| 03701 | MOVEL PARA MAQUINA DE COSTURA (GABINETE) |
| 03102 | MOYASHI |
| 20602 | MP3 PLAYER |
| 20603 | MP4 PLAYER |
| 20604 | MP5 PLAYER |
| 01215 | MUCAIA |
| 00105 | MUCILON |
| 00107 | MUCILON DE ARROZ |
| 00612 | MUCILON DE MILHO |
| 47101 | MUCUA |
| 47501 | MUCUA EM FILE CONGELADO |
| 47301 | MUCUA EM FILE FRESCO |
| 47201 | MUCUA EM POSTAS |
| 47401 | MUCUA SALGADA |
| 00401 | MUDANCA |
| 02301 | MUDANCA |
| 01808 | MUDAS |
| 04104 | MULETA |
| 02101 | MULTA DE ALISTAMENTO MILITAR |
| 02802 | MULTA DE ALISTAMENTO MILITAR |
| 00803 | MULTA DE ALUGUEL DE GARAGEM |
| 02703 | MULTA DE BIBLIOTECA |
| 00903 | MULTA DE CONDOMINIO |
| 01003 | MULTA DE IMPOSTO PREDIAL |
| 00504 | MULTA DE LOCACAO DE OUTROS IMOVEIS |
| 00703 | MULTA DE PRESTACAO DO IMOVEL |
| 03401 | MULTA DE VIOLACAO DE AGUA E ENERGIA ELETRICA |
| 00603 | MULTA DO ALUGUEL DO IMOVEL |
| 00201 | MULTAS |
| 02010 | MULTAS DIVERSAS DE OUTROS IMOVEIS |
| 05601 | MULTIERVAS CHA |
| 06306 | MULTILIMPADOR INSTANTANEO |
| 01658 | MULTIMETRO |
| 06104 | MULTIPREPARADOR DE ALIMENTOS ELETRICO |
| 06103 | MULTIPROCESSADOR DE ALIMENTOS ELETRICO |
| 06311 | MULTIUSO |
| 04302 | MUMU |
| 11302 | MUMU DIET |
| 11304 | MUMU DIETETICO |
| 11202 | MUMU LIGHT |
| 04324 | MUNGUNZA |
| 01902 | MUNGUNZA PARA VIAGEM |
| 01903 | MUNGUNZA PRE-COZIDO PARA VIAGEM |
| 01901 | MUNGUZA PARA VIAGEM |
| 01905 | MUNGUZA PRE-COZIDO PARA VIAGEM |
| 00408 | MUNICAO DE ARMA DE FOGO (EXCETO DE CACA) |
| 00506 | MUNICAO DE ARMA DE FOGO DE CACA |
| 00814 | MUQUI |
| 10314 | MUQUI DIET |
| 10336 | MUQUI DIETETICO |
| 10214 | MUQUI LIGHT |
| 02405 | MURCILHA |
| 02230 | MURGOTE (TANGERINA) |
| 07001 | MURICI |
| 07005 | MURICI DA MATA |
| 07006 | MURICI DA PRAIA |
| 07010 | MURICI DE CAPOEIRA |
| 07003 | MURICI DE TABULEIRO |
| 07009 | MURICI DO BREJO |
| 07008 | MURICI DO CAMPO |
| 07012 | MURICI MIUDO |
| 07011 | MURICI PENIMA |
| 07007 | MURICI PITANGA |
| 07002 | MURICI RASTEIRO |
| 07004 | MURUCHI |
| 01001 | MUSCULO BOVINO |
| 01013 | MUSCULO COM OSSO |
| 01002 | MUSCULO DA PA |
| 01005 | MUSCULO DO DIANTEIRO |
| 01003 | MUSCULO DO TRASEIRO |
| 10801 | MUSCULO NAO-ESPECIFICADO |
| 10502 | MUSCULO SUINO |
| 00202 | MUSEU (DOACAO EM DINHEIRO) |
| 01401 | MUSEU (INGRESSO) |
| 04601 | MUSLI |
| 01803 | MUSSARELA |
| 01807 | MUSSARELA DE BUFALA |
| 05107 | MUSSARELA DE BUFALA LIGHT |
| 01805 | MUSSARELA FATIADA |
| 05105 | MUSSARELA FATIADA LIGHT |
| 05103 | MUSSARELA LIGHT |
| 03707 | MUTUM ABATIDO, CONGELADO OU VIVO |
| 03721 | MUTUM CONGELADO |
| 03722 | MUTUM VIVO |
| 07303 | MUXIBA (GORDURA BOVINA) |
| 01802 | MUZARELLA |
| 05102 | MUZARELLA LIGHT |
| 01401 | NABICA |
| 01301 | NABO |
| 01303 | NABO BRANCO |
| 01002 | NABO CHINES (RABANETE) |
| 05201 | NABO EM CONSERVA |
| 01302 | NABO ROXO |
| 00912 | NADA CONSTA (TAXA) |
| 02605 | NAFTALINA |
| 01607 | NAJA |
| 02801 | NAMBU ABATIDO, CONGELADO OU VIVO |
| 02802 | NAMBU CONGELADO |
| 02803 | NAMBU VIVO |
| 01801 | NAPA |
| 08201 | NARIZ SUINO |
| 03202 | NATA COLONIAL |
| 03203 | NATA DOCE |
| 03201 | NATA DOCE OU SALGADA |
| 08701 | NATA DOCE OU SALGADA LIGHT |
| 03204 | NATA SALGADA |
| 03602 | NATOR |
| 10502 | NATOR LIGHT |
| 00401 | NAVIO |
| 00404 | NEBULIZACAO |
| 09002 | NEBULIZADOR ELETRICO |
| 07401 | NECTARINA |
| 02602 | NESCAFE |
| 10702 | NESCAFE ORGANICO |
| 00812 | NESCAU |
| 10312 | NESCAU DIET |
| 10334 | NESCAU DIETETICO |
| 00815 | NESCAU INSTANTANEO |
| 10315 | NESCAU INSTANTANEO DIET |
| 10337 | NESCAU INSTANTANEO DIETETICO |
| 10215 | NESCAU INSTANTANEO LIGHT |
| 10212 | NESCAU LIGHT |
| 03111 | NESCAU LIQUIDO |
| 00813 | NESCAU VITAMINADO |
| 10313 | NESCAU VITAMINADO DIET |
| 10335 | NESCAU VITAMINADO DIETETICO |
| 10213 | NESCAU VITAMINADO LIGHT |
| 05801 | NESPERA |
| 05806 | NESPERA DO JAPAO |
| 00823 | NESQUIK |
| 05802 | NESTLE BABY |
| 02101 | NESTON |
| 02513 | NEUROLOGICO |
| 00416 | NEUTROX |
| 08005 | NHOQUE PRONTO LIGHT PARA VIAGEM |
| 00905 | NHOQUE PRONTO PARA VIAGEM |
| 02702 | NHOQUE SEMIPRONTO |
| 06102 | NHOQUE SEMIPRONTO LIGHT |
| 00904 | NICURI |
| 08201 | NIDEX |
| 45116 | NIMBUIA |
| 06001 | NIRA |
| 01619 | NIVEL |
| 07201 | NOTEBOOK |
| 00107 | NOVELO DE LA |
| 05102 | NOVOMILK EM PO |
| 01501 | NOZ |
| 02701 | NOZ (NOGUEIRA) |
| 02203 | NOZ DE PISTACHE |
| 01401 | NOZ MOSCADA (TEMPERO INDUSTRIALIZADO) |
| 05602 | NUCITA (CHOCOLATE EM CREME) |
| 12202 | NUCITA (CHOCOLATE EM CREME) DIET |
| 12205 | NUCITA (CHOCOLATE EM CREME) DIETETICO |
| 12102 | NUCITA (CHOCOLATE EM CREME) LIGHT |
| 04902 | NUGGET DE FRANGO EMPANADO |
| 02110 | NUGGET DE QUEIJO |
| 02901 | NUGGETS DE FRANGO |
| 02903 | NUGGETS DE GALINHA |
| 02801 | NUTRICIONISTA |
| 00109 | NUTRILON DE ARROZ |
| 03901 | OBJETO DE ANIMAL |
| 03905 | OBJETO PARA ANIMAL |
| 01605 | OBRA POR EMPREITADA |
| 01605 | OBRA POR EMPREITADA |
| 00503 | OBSTETRA PARA CIRURGIA E PARTO |
| 02403 | OBTURACAO DENTARIA |
| 04003 | OCULOS DE GRAU (ARMACAO+LENTE) |
| 00110 | OCULOS DE NATACAO |
| 00302 | OCULOS DE SOL SEM GRAU |
| 00301 | OCULOS SEM GRAU |
| 30806 | ODONTOLOGICO |
| 00204 | OFERENDA RELIGIOSA |
| 00705 | OFICIAL DE JUSTICA |
| 02229 | OKAKI (BISCOITO SALGADO) |
| 10129 | OKAKI (BISCOITO SALGADO) LIGHT |
| 02402 | OLA |
| 00607 | OLEO (LAXATIVO) |
| 02603 | OLEO CAPILAR |
| 03303 | OLEO COMPOSTO DE SOJA E OLIVA |
| 00601 | OLEO DE ALGODAO |
| 00513 | OLEO DE AMENDOA |
| 01101 | OLEO DE AMENDOA |
| 00501 | OLEO DE AMENDOIM |
| 00901 | OLEO DE ARROZ |
| 00702 | OLEO DE BABACU |
| 03401 | OLEO DE BANANA |
| 03701 | OLEO DE BURITI |
| 02901 | OLEO DE CANOLA |
| 00701 | OLEO DE COCO BABACU |
| 02701 | OLEO DE COCO DA BAHIA |
| 01502 | OLEO DE COCO LICURI |
| 01704 | OLEO DE COCO MACAIBA |
| 01701 | OLEO DE COCO MACAUBA |
| 03601 | OLEO DE COCO NAO-ESPECIFICADO |
| 01501 | OLEO DE COCO OURICURI |
| 01402 | OLEO DE COCO TICUM |
| 01401 | OLEO DE COCO TUCUM |
| 03501 | OLEO DE DENDE |
| 08601 | OLEO DE EUCALIPTO |
| 00828 | OLEO DE FREIO |
| 01201 | OLEO DE GENGIBRE |
| 02601 | OLEO DE GERGELIM |
| 00801 | OLEO DE GIRASSOL |
| 01504 | OLEO DE LICURI |
| 01702 | OLEO DE MACAIBA |
| 01703 | OLEO DE MACAUBA |
| 03601 | OLEO DE MAQUINA |
| 00521 | OLEO DE MASSAGEM |
| 00401 | OLEO DE MILHO |
| 03201 | OLEO DE MOVEL |
| 01503 | OLEO DE OURICURI |
| 01001 | OLEO DE PEIXE |
| 01601 | OLEO DE PEQUI |
| 01604 | OLEO DE PEQUIA |
| 03202 | OLEO DE PEROBA |
| 01602 | OLEO DE PIQUI |
| 01603 | OLEO DE PIQUIA |
| 01302 | OLEO DE SEMENTE DE UVA |
| 00301 | OLEO DE SOJA |
| 01403 | OLEO DE TICUM |
| 01404 | OLEO DE TUCUM |
| 01301 | OLEO DE UVA |
| 02610 | OLEO DERMATOLOGICO |
| 01301 | OLEO DIESEL (COMBUSTIVEL DE VEICULO) |
| 00801 | OLEO DIESEL (COMBUSTIVEL DOMESTICO) |
| 00802 | OLEO DIESEL (LITRO) (COMBUSTIVEL DOMESTICO) |
| 01701 | OLEO DIESEL DE OUTROS IMOVEIS (COMBUSTIVEL DOMESTICO) |
| 03104 | OLEO DOCE (PARA GASES) |
| 03203 | OLEO FORTIFICANTE |
| 31111 | OLEO INDETERMINADO |
| 00201 | OLEO LUBRIFICANTE (COMPLEMENTACAO OU TROCA) |
| 03201 | OLEO NAO-ESPECIFICADO |
| 02627 | OLEO PARA ASSADURA |
| 31104 | OLEO PARA MASSAGEM |
| 00506 | OLEO PARA PELE |
| 01401 | OMBREIRA |
| 06202 | OMELETE EM PO (TEMPERO) |
| 06501 | OMELETE PARA VIAGEM |
| 08601 | OMELETEIRA |
| 02101 | ONIBUS INTERESTADUAL |
| 02201 | ONIBUS INTERESTADUAL |
| 02001 | ONIBUS INTERMUNICIPAL |
| 02001 | ONIBUS INTERMUNICIPAL |
| 02101 | ONIBUS INTERNACIONAL |
| 00101 | ONIBUS URBANO |
| 00101 | ONIBUS URBANO |
| 04601 | OPERA (INGRESSO) |
| 10701 | ORA PRO NOBIS |
| 00107 | ORDEM DE PAGAMENTO (CORREIO) |
| 09701 | OREGANO (MOLHO) |
| 02201 | OREGANO (TEMPERO INDUSTRIALIZADO) |
| 02202 | OREGAO (TEMPERO INDUSTRIALIZADO) |
| 10401 | ORELHA CAPRINA FRESCA |
| 02358 | ORELHA DE GATO (BISCOITO DOCE) |
| 00701 | ORELHA DE PORCO DEFUMADA |
| 01401 | ORELHA DE PORCO SALGADA |
| 05001 | ORELHA SUINA FRESCA |
| 00703 | ORENCAO |
| 00704 | ORENCO |
| 00601 | ORGAO E TECLADO |
| 00106 | ORQUIDEA |
| 03202 | OSSADA BOVINA |
| 10902 | OSSADA NAO-ESPECIFICADA |
| 05202 | OSSADA SUINA |
| 03201 | OSSO BOVINO |
| 03801 | OSSO DE GALINHA |
| 04601 | OSSO DE PERU |
| 10901 | OSSO NAO-ESPECIFICADO |
| 00106 | OSSO PARA CAO |
| 05201 | OSSO SUINO |
| 01014 | OSSOBUCO |
| 64101 | OSTRA |
| 64103 | OSTRA AMERICANA |
| 64109 | OSTRA DA VIRGINIA |
| 64104 | OSTRA DAS PEDRAS |
| 63103 | OSTRA DE POBRE (MARISCO) |
| 64102 | OSTRA DO FUNDO |
| 64105 | OSTRA DO MANGUE |
| 64401 | OSTRA SALGADA |
| 64201 | OSTRA SEM CASCA |
| 03403 | OTOFU |
| 03602 | OTOLOGICOS |
| 02203 | OUREGAO (TEMPERO INDUSTRIALIZADO) |
| 00910 | OURICURI |
| 00919 | OURINOL DE PLASTICO |
| 01403 | OURIVES (CONSERTO DE JOIAS) |
| 03701 | OUTRA AVE ABATIDA, CONGELADA OU VIVA |
| 03709 | OUTRA AVE CONGELADA |
| 03710 | OUTRA AVE VIVA |
| 01101 | OUTRAS BEBIDAS ALCOOLICAS |
| 00201 | OUTRAS CERIMONIAS RELIGIOSAS |
| 01201 | OUTRAS CONSULTAS DIFERENTES DE GERAL, PEDIATRICA E GINECO-OBSTETRICIA |
| 00308 | OUTRAS DESPESAS COM FUNERAL |
| 00801 | OUTRAS FESTAS OU RECEPCOES |
| 01201 | OUTRAS PECAS E ACESSORIOS (BICICLETA) |
| 00801 | OUTRAS PECAS, ACESSORIOS E MATERIAIS (VEICULOS MOTORIZADOS) |
| 01801 | OUTRAS ROUPAS DE BEBE |
| 06101 | OUTRO CHA |
| 00402 | OUTROS (DEPOSITO/APLICACAO) |
| 01005 | OUTROS (HOBBY) |
| 00401 | OUTROS (OURO, MOEDAS ESTRANGEIRAS, ETC.) (APLICACAO) |
| 00401 | OUTROS (OURO, MOEDAS ESTRANGEIRAS, ETC.) (RESGATE) |
| 00402 | OUTROS (RETIRADA/RESGATE) |
| 06701 | OUTROS ANIMAIS VIVOS |
| 01405 | OUTROS ARTIGOS DE FORMATURA (BECA, CHAPEU, ETC) |
| 04109 | OUTROS ARTIGOS MEDICOS |
| 09102 | OUTROS CURSOS DE ATUALIZACAO, APERFEICOAMENTO OU EXTENSAO |
| 09101 | OUTROS CURSOS DE CAPACITACAO PROFISSIONAL |
| 00401 | OUTROS EVENTOS ESPORTIVOS (INGRESSO) |
| 00801 | OUTROS JOGOS E APOSTAS |
| 00804 | OUTROS LIVROS DIDATICOS |
| 00422 | OUTROS PRODUTOS DE CABELO |
| 02101 | OUTROS QUEIJOS |
| 10701 | OUTROS QUEIJOS LIGHT |
| 06101 | OUTROS QUEIJOS LIGHT |
| 00801 | OUTROS UTENSILIOS DE METAL |
| 00501 | OUTROS UTENSILIOS DE VIDRO OU LOUCA |
| 73101 | OVAS DE PEIXE (QUALQUER ESPECIE) |
| 73401 | OVAS DE PEIXE SALGADAS (QUALQUER ESPECIE) |
| 05702 | OVELHA VIVA |
| 03712 | OVO COZIDO |
| 03501 | OVO DE CODORNA |
| 06901 | OVO DE CODORNA EM CONSERVA |
| 03301 | OVO DE GALINHA |
| 03303 | OVO DE GALINHA CAIPIRA |
| 06501 | OVO DE GALINHA ORGANICO |
| 06301 | OVO DE GUINE |
| 06701 | OVO DE JACARE |
| 04301 | OVO DE MARRECA |
| 05801 | OVO DE PASCOA |
| 07209 | OVO DE PASCOA DIET |
| 12401 | OVO DE PASCOA DIET |
| 07210 | OVO DE PASCOA DIETETICO |
| 12405 | OVO DE PASCOA DIETETICO |
| 09505 | OVO DE PASCOA LIGHT |
| 12301 | OVO DE PASCOA LIGHT |
| 03401 | OVO DE PATA |
| 04501 | OVO DE PERU |
| 04401 | OVO DE TRACAJA |
| 00818 | OVOMALTINE |
| 10318 | OVOMALTINE DIET |
| 10340 | OVOMALTINE DIETETICO |
| 10218 | OVOMALTINE LIGHT |
| 03302 | OVOS DE GALINHA |
| 03304 | OVOS DE GALINHA BRANCO |
| 03305 | OVOS DE GALINHA VERMELHO |
| 06801 | OXIDO DE FERRO (PARA PISO DE CERAMICA) |
| 07101 | OXIDO DE FERRO (PARA PISO DE CERAMICA) |
| 01804 | OXIGENADOR DE AQUARIO ORNAMENTAL |
| 04207 | OXIGENIO (DESPESA COM SAUDE) |
| 01602 | OZONIZADOR |
| 01624 | PA |
| 00901 | PA |
| 00908 | PA COM OSSO |
| 01001 | PA DE LIXO |
| 00911 | PA SEM OSSO |
| 03306 | PA SUINA |
| 06703 | PACA VIVA |
| 03203 | PACOCA |
| 00213 | PACOCA DE CARNE DE SOL PARA VIAGEM |
| 03206 | PACOCA DE CASTANHA DE CAJU |
| 13106 | PACOCA DE CASTANHA DE CAJU DIET |
| 13115 | PACOCA DE CASTANHA DE CAJU DIETETICA |
| 13103 | PACOCA DIET |
| 13112 | PACOCA DIETETICA |
| 03205 | PACOQUINHA DE AMENDOIM |
| 13105 | PACOQUINHA DE AMENDOIM DIET |
| 13114 | PACOQUINHA DE AMENDOIM DIETETICA |
| 02803 | PACOTE DE SERVICO DE CONTA BANCARIA |
| 04101 | PACOTES TURISTICOS INTERNACIONAIS |
| 04001 | PACOTES TURISTICOS NACIONAIS |
| 01017 | PACOVA |
| 01018 | PACOVA GRANDE |
| 01401 | PAGAMENTO DE ASILO |
| 01303 | PAGAMENTO DE CREDITO EDUCATIVO |
| 01201 | PAGAMENTO DE EMPRESTIMO |
| 02203 | PAGAMENTO DE SPU (SERVICO DE PATRIMONIO DA UNIAO) DE OUTROS IMOVEIS |
| 02701 | PAGAMENTO DE TITULO PROTESTADO |
| 00903 | PAGER (APARELHO) |
| 01202 | PAGERS (MENSALIDADE, ASSINATURA, ALUGUEL) |
| 01103 | PAINA DE ENCHIMENTO DE ALMOFADA, DE TRAVESSEIRO, ETC. |
| 00908 | PAINCO |
| 09701 | PAINEL SOLAR (ENERGIA SOLAR) |
| 02501 | PAIO |
| 04304 | PALANQUE DE CIMENTO OU CONCRETO |
| 04304 | PALANQUE DE CIMENTO OU CONCRETO |
| 01902 | PALAVRA CRUZADA |
| 00905 | PALETA |
| 00907 | PALETA COM OSSO |
| 05606 | PALETA DE CARNEIRO |
| 03304 | PALETA SUINA |
| 01203 | PALETO |
| 01504 | PALETO |
| 01201 | PALETO E BLAZER |
| 01501 | PALETO, BLAZER E TERNO |
| 03302 | PALHA (PECA DE DECORACAO) |
| 03001 | PALHA DE ACO |
| 01401 | PALHA PARA CIGARRO |
| 05701 | PALHA PARA COBERTURA |
| 05701 | PALHA PARA COBERTURA |
| 08501 | PALITEIRO |
| 04101 | PALITO |
| 04103 | PALITO DE DENTE |
| 07603 | PALITO PARA CHURRASCO |
| 07602 | PALITO PARA SORVETE |
| 04102 | PALITOS DE DENTE |
| 10603 | PALM |
| 10601 | PALM TOP |
| 00109 | PALMA |
| 00406 | PALMILHA |
| 04105 | PALMILHA (ORTOPEDICA) |
| 04406 | PALMITO ACAI IN NATURA |
| 07707 | PALMITO ACAI IN NATURA ORGANICO |
| 04404 | PALMITO AMARGOSO IN NATURA |
| 07705 | PALMITO AMARGOSO IN NATURA ORGANICO |
| 04405 | PALMITO DO CHAO IN NATURA |
| 07706 | PALMITO DO CHAO IN NATURA ORGANICO |
| 04402 | PALMITO DOCE IN NATURA |
| 07703 | PALMITO DOCE IN NATURA ORGANICO |
| 00501 | PALMITO EM CONSERVA |
| 07701 | PALMITO FRESCO ORGANICO |
| 04401 | PALMITO IN NATURA |
| 07702 | PALMITO IN NATURA ORGANICO |
| 04407 | PALMITO JUCARA IN NATURA |
| 07708 | PALMITO JUCARA IN NATURA ORGANICO |
| 04403 | PALMITO MOLE IN NATURA |
| 07704 | PALMITO MOLE IN NATURA ORGANICO |
| 10602 | PALMTOP |
| 04326 | PAMONHA |
| 05101 | PAMONHA |
| 12101 | PANCETA SUINA |
| 02305 | PANCREATICO |
| 02901 | PANDEIRO |
| 00602 | PANELA |
| 02102 | PANELA DE BARRO |
| 00604 | PANELA DE PRESSAO |
| 21601 | PANELA ELETRICA |
| 01806 | PANELADA (VISCERAS BOVINAS NAO-ESPECIFICADAS) |
| 00234 | PANETONE |
| 10034 | PANETONE DIET |
| 10074 | PANETONE DIETETICO |
| 00802 | PANFLETO |
| 01104 | PANHOCA DE MILHO |
| 00602 | PANO DE CENTRO DE PLASTICO |
| 00801 | PANO DE CHAO |
| 00704 | PANO DE LIMPEZA DESCARTAVEL |
| 00705 | PANO DE PIA |
| 00702 | PANO DE PO |
| 00503 | PANO DE PRATO |
| 00803 | PANO DE SACO PARA LIMPEZA |
| 08013 | PANQUECA PRONTA LIGHT PARA VIAGEM |
| 00913 | PANQUECA PRONTA PARA VIAGEM |
| 01902 | PANTUFA |
| 00231 | PAO ALEMAO |
| 10031 | PAO ALEMAO DIET |
| 10071 | PAO ALEMAO DIETETICO |
| 00202 | PAO ALMOFADINHA |
| 10002 | PAO ALMOFADINHA DIET |
| 10042 | PAO ALMOFADINHA DIETETICO |
| 00224 | PAO AMANTEIGADO (DOCE) |
| 10024 | PAO AMANTEIGADO (DOCE) DIET |
| 10064 | PAO AMANTEIGADO (DOCE) DIETETICO |
| 06401 | PAO ARABE |
| 12101 | PAO ARABE LIGHT |
| 09901 | PAO ARABE LIGHT |
| 00245 | PAO AUSTRALIANO (DOCE) |
| 00129 | PAO BAGUETE |
| 00136 | PAO BAIANO |
| 00127 | PAO BANQUETE |
| 00128 | PAO BAQUETE |
| 00112 | PAO BENGALA |
| 00113 | PAO BISNAGA |
| 00242 | PAO BISNAGUINHA |
| 01406 | PAO BOA VIDA (INTEGRAL) |
| 06301 | PAO BOLA |
| 00206 | PAO BOLACHAO |
| 10006 | PAO BOLACHAO DIET |
| 10046 | PAO BOLACHAO DIETETICO |
| 03901 | PAO BRIOCHE |
| 01105 | PAO BROTE |
| 00248 | PAO CABRITINHO |
| 00107 | PAO CACETINHO |
| 00109 | PAO CARECA |
| 00214 | PAO CARECA DOCE |
| 10014 | PAO CARECA DOCE DIET |
| 10054 | PAO CARECA DOCE DIETETICO |
| 00156 | PAO CARIOCA |
| 00124 | PAO CARIOQUINHA |
| 00230 | PAO CARTEIRA (DOCE) |
| 10030 | PAO CARTEIRA (DOCE) DIET |
| 10070 | PAO CARTEIRA (DOCE) DIETETICO |
| 00131 | PAO CARTEIRA (SALGADO) |
| 08101 | PAO CARTEIRA DIET |
| 08102 | PAO CARTEIRA DIETETICO |
| 11701 | PAO CARTEIRA LIGHT |
| 05501 | PAO CARTEIRA NAO-ESPECIFICADO |
| 09001 | PAO CARTEIRA NAO-ESPECIFICADO DIET |
| 09002 | PAO CARTEIRA NAO-ESPECIFICADO DIETETICO |
| 08901 | PAO CARTEIRA NAO-ESPECIFICADO LIGHT |
| 01601 | PAO CASEIRO |
| 00117 | PAO CAVAQUINHO |
| 00146 | PAO CERVEJINHA |
| 00151 | PAO CHIPA |
| 00145 | PAO CIABATA |
| 00153 | PAO CILINDRO |
| 04406 | PAO COM ERVA FINA |
| 00302 | PAO COM MANTEIGA |
| 00301 | PAO COM OU SEM MANTEIGA |
| 01303 | PAO CRIOULO |
| 00102 | PAO DE AGUA |
| 01201 | PAO DE AIPIM |
| 04403 | PAO DE ALHO |
| 00601 | PAO DE ARROZ |
| 01701 | PAO DE AVEIA |
| 00126 | PAO DE BANHA |
| 01001 | PAO DE BATATA |
| 00901 | PAO DE CACHORRO QUENTE |
| 08305 | PAO DE CACHORRO QUENTE DIET |
| 08304 | PAO DE CACHORRO QUENTE DIET COM OU SEM MANTEIGA |
| 08307 | PAO DE CACHORRO QUENTE DIETETICO |
| 11905 | PAO DE CACHORRO QUENTE LIGHT |
| 11904 | PAO DE CACHORRO QUENTE LIGHT COM OU SEM MANTEIGA |
| 00509 | PAO DE CAIXA |
| 06509 | PAO DE CAIXA DIET |
| 06521 | PAO DE CAIXA DIETETICO |
| 06609 | PAO DE CAIXA LIGHT |
| 04408 | PAO DE CALABRESA |
| 00147 | PAO DE CARA |
| 04404 | PAO DE CEBOLA |
| 04401 | PAO DE CENOURA, ALHO, CEBOLA, TORRESMO, ETC. |
| 01301 | PAO DE CENTEIO |
| 10902 | PAO DE CENTEIO LIGHT |
| 00119 | PAO DE CHA |
| 00244 | PAO DE CHOCOLATE |
| 00223 | PAO DE COCO |
| 10023 | PAO DE COCO DIET |
| 10063 | PAO DE COCO DIETETICO |
| 04407 | PAO DE ERVA DOCE |
| 01002 | PAO DE FECULA DE BATATA |
| 00305 | PAO DE FORMA |
| 00505 | PAO DE FORMA DE LEITE PULLMAN |
| 06505 | PAO DE FORMA DE LEITE PULLMAN DIET |
| 06517 | PAO DE FORMA DE LEITE PULLMAN DIETETICO |
| 06605 | PAO DE FORMA DE LEITE PULLMAN LIGHT |
| 01102 | PAO DE FORMA DE MILHO |
| 00301 | PAO DE FORMA DE PADARIA (ADOCICADO) |
| 00401 | PAO DE FORMA DE PADARIA (SALGADO) |
| 04302 | PAO DE FORMA DE SOJA |
| 08202 | PAO DE FORMA DIET |
| 08201 | PAO DE FORMA DIET COM OU SEM MANTEIGA |
| 08203 | PAO DE FORMA DIETETICO |
| 00501 | PAO DE FORMA INDUSTRIALIZADO DE QUALQUER MARCA |
| 06501 | PAO DE FORMA INDUSTRIALIZADO DE QUALQUER MARCA DIET |
| 06513 | PAO DE FORMA INDUSTRIALIZADO DE QUALQUER MARCA DIETETICO |
| 06601 | PAO DE FORMA INDUSTRIALIZADO DE QUALQUER MARCA LIGHT |
| 06510 | PAO DE FORMA INDUSTRIALIZADO DIET |
| 06522 | PAO DE FORMA INDUSTRIALIZADO DIETETICO |
| 06610 | PAO DE FORMA INDUSTRIALIZADO LIGHT |
| 01405 | PAO DE FORMA INTEGRAL |
| 11802 | PAO DE FORMA LIGHT |
| 11801 | PAO DE FORMA LIGHT COM OU SEM MANTEIGA |
| 10801 | PAO DE FORMA NAO ESPECIFICADO LIGHT |
| 05601 | PAO DE FORMA NAO-ESPECIFICADO |
| 09201 | PAO DE FORMA NAO-ESPECIFICADO DIET |
| 09202 | PAO DE FORMA NAO-ESPECIFICADO DIETETICO |
| 09101 | PAO DE FORMA NAO-ESPECIFICADO LIGHT |
| 00507 | PAO DE FORMA PETROPOLIS |
| 06507 | PAO DE FORMA PETROPOLIS DIET |
| 06519 | PAO DE FORMA PETROPOLIS DIETETICO |
| 06607 | PAO DE FORMA PETROPOLIS LIGHT |
| 00504 | PAO DE FORMA PLUS VITA |
| 06504 | PAO DE FORMA PLUS VITA DIET |
| 06516 | PAO DE FORMA PLUS VITA DIETETICO |
| 06604 | PAO DE FORMA PLUS VITA LIGHT |
| 00515 | PAO DE FORMA PULLMAN |
| 00514 | PAO DE FORMA PULMEX |
| 00506 | PAO DE FORMA SEVEN BOYS |
| 06506 | PAO DE FORMA SEVEN BOYS DIET |
| 06518 | PAO DE FORMA SEVEN BOYS DIETETICO |
| 06606 | PAO DE FORMA SEVEN BOYS LIGHT |
| 04409 | PAO DE FRIOS |
| 01103 | PAO DE FUBA |
| 04405 | PAO DE GERGELIM |
| 00701 | PAO DE GLUTEN |
| 04412 | PAO DE GOIABA |
| 01202 | PAO DE GOMA |
| 01403 | PAO DE GRAHAM |
| 06803 | PAO DE GRAHAM LIGHT |
| 01402 | PAO DE GRAHAM PLUS VITA |
| 06802 | PAO DE GRAHAM PLUS VITA LIGHT |
| 11903 | PAO DE HAMBURBER LIGHT |
| 08303 | PAO DE HAMBURGER DIET |
| 08302 | PAO DE HAMBURGER DIET COM OU SEM MANTEIGA |
| 08306 | PAO DE HAMBURGER DIETETICO |
| 11902 | PAO DE HAMBURGER LIGHT COM OU SEM MANTEIGA |
| 08301 | PAO DE HAMBURGER OU CACHORRO QUENTE DIET COM OU SEM MANTEIGA |
| 11901 | PAO DE HAMBURGER OU CACHORRO QUENTE LIGHT COM OU SEM MANTEIGA |
| 00103 | PAO DE HAMBURGUER |
| 00155 | PAO DE INHAME |
| 00220 | PAO DE LANCHE |
| 10020 | PAO DE LANCHE DIET |
| 10060 | PAO DE LANCHE DIETETICO |
| 04410 | PAO DE LARANJA |
| 00213 | PAO DE LEITE (PAO DOCE) |
| 10013 | PAO DE LEITE (PAO DOCE) DIET |
| 10053 | PAO DE LEITE (PAO DOCE) DIETETICO |
| 11101 | PAO DE LINHO OU LINHACA |
| 04602 | PAO DE LO |
| 01204 | PAO DE MACAXEIRA |
| 01203 | PAO DE MANDIOCA |
| 03903 | PAO DE MASSA FOLHADA |
| 00229 | PAO DE MEL |
| 10029 | PAO DE MEL DIET |
| 10069 | PAO DE MEL DIETETICO |
| 01101 | PAO DE MILHO |
| 04411 | PAO DE PASSAS |
| 03743 | PAO DE QUEIJO |
| 00801 | PAO DE QUEIJO |
| 10901 | PAO DE QUEIJO LIGHT |
| 06701 | PAO DE QUEIJO LIGHT |
| 02302 | PAO DE QUEIJO SEMIPRONTO |
| 01801 | PAO DE RABANADA |
| 00105 | PAO DE SAL |
| 00302 | PAO DE SANDUICHE DE PADARIA (ADOCICADO) |
| 00402 | PAO DE SANDUICHE DE PADARIA (SALGADO) |
| 00516 | PAO DE SANDUICHE INDUSTRIALIZADO DE QUALQUER MARCA |
| 05701 | PAO DE SANDUICHE NAO-ESPECIFICADO |
| 09401 | PAO DE SANDUICHE NAO-ESPECIFICADO DIET |
| 09402 | PAO DE SANDUICHE NAO-ESPECIFICADO DIETETICO |
| 09301 | PAO DE SANDUICHE NAO-ESPECIFICADO LIGHT |
| 00111 | PAO DE SEMOLINA |
| 04301 | PAO DE SOJA |
| 04303 | PAO DE SOJA DE FORMA |
| 11501 | PAO DE SOJA LIGHT |
| 04402 | PAO DE TORRESMO |
| 00106 | PAO DE TRIGO |
| 06511 | PAO DIET (DE FORMA INDUSTRIALIZADO) |
| 06512 | PAO DIET WICK BOLD |
| 06523 | PAO DIETETICO (DE FORMA INDUSTRIALIZADO) |
| 06524 | PAO DIETETICO WICK BOLD |
| 00304 | PAO DOCE |
| 00201 | PAO DOCE |
| 00212 | PAO DOCE CARECA |
| 10012 | PAO DOCE CARECA DIET |
| 10052 | PAO DOCE CARECA DIETETICO |
| 00204 | PAO DOCE COM COCO |
| 10004 | PAO DOCE COM COCO DIET |
| 10044 | PAO DOCE COM COCO DIETETICO |
| 00207 | PAO DOCE COM CREME |
| 10007 | PAO DOCE COM CREME DIET |
| 10047 | PAO DOCE COM CREME DIETETICO |
| 00221 | PAO DOCE COM MEL |
| 10021 | PAO DOCE COM MEL DIET |
| 10061 | PAO DOCE COM MEL DIETETICO |
| 00222 | PAO DOCE COM OVOS |
| 10022 | PAO DOCE COM OVOS DIET |
| 10062 | PAO DOCE COM OVOS DIETETICO |
| 00208 | PAO DOCE COM RECHEIO |
| 10008 | PAO DOCE COM RECHEIO DIET |
| 10048 | PAO DOCE COM RECHEIO DIETETICO |
| 00205 | PAO DOCE COMUM |
| 10005 | PAO DOCE COMUM DIET |
| 10045 | PAO DOCE COMUM DIETETICO |
| 00217 | PAO DOCE DE LEITE |
| 10017 | PAO DOCE DE LEITE DIET |
| 10057 | PAO DOCE DE LEITE DIETETICO |
| 00215 | PAO DOCE DE TRIGO |
| 10015 | PAO DOCE DE TRIGO DIET |
| 10055 | PAO DOCE DE TRIGO DIETETICO |
| 10001 | PAO DOCE DIET |
| 10041 | PAO DOCE DIETETICO |
| 00210 | PAO DOCE ESPECIAL |
| 10010 | PAO DOCE ESPECIAL DIET |
| 10050 | PAO DOCE ESPECIAL DIETETICO |
| 11301 | PAO DOCE LIGHT |
| 00209 | PAO DOCE SEM COCO |
| 10009 | PAO DOCE SEM COCO DIET |
| 10049 | PAO DOCE SEM COCO DIETETICO |
| 00110 | PAO FILAO |
| 00246 | PAO FOLHADO |
| 00306 | PAO FRANCES |
| 00101 | PAO FRANCES |
| 00118 | PAO FRANCES BISNAGA |
| 11201 | PAO FRANCES DIET |
| 00510 | PAO INGLES |
| 01401 | PAO INTEGRAL |
| 11002 | PAO INTEGRAL LIGHT |
| 06801 | PAO INTEGRAL LIGHT |
| 11001 | PAO INTEGRAL LIGHT COM OU SEM MANTEIGA |
| 01404 | PAO INTEGRAL PLUS VITA |
| 06804 | PAO INTEGRAL PLUS VITA LIGHT |
| 00512 | PAO ITALIANO |
| 00152 | PAO JACO |
| 00219 | PAO LANCHE |
| 10019 | PAO LANCHE DIET |
| 10059 | PAO LANCHE DIETETICO |
| 00247 | PAO MADRILENHO |
| 00232 | PAO MANDI |
| 10032 | PAO MANDI DIET |
| 10072 | PAO MANDI DIETETICO |
| 00233 | PAO MANDIM |
| 10033 | PAO MANDIM DIET |
| 10073 | PAO MANDIM DIETETICO |
| 00243 | PAO MANDINHO |
| 00235 | PAO MANTEIGA |
| 10035 | PAO MANTEIGA DIET |
| 10075 | PAO MANTEIGA DIETETICO |
| 00137 | PAO MANUAL |
| 00122 | PAO MASSA FINA |
| 00142 | PAO MASSA GROSSA |
| 00154 | PAO MINEIRO (SALGADO) |
| 01501 | PAO NAO-ESPECIFICADO |
| 00134 | PAO PALITO |
| 00143 | PAO PIZZA |
| 00508 | PAO PLUS VITA |
| 06508 | PAO PLUS VITA DIET |
| 06520 | PAO PLUS VITA DIETETICO |
| 06608 | PAO PLUS VITA LIGHT |
| 00148 | PAO PORTUGUES |
| 00125 | PAO PRESUNTO |
| 01302 | PAO PRETO |
| 10901 | PAO PRETO LIGHT |
| 00121 | PAO PROVENCAL |
| 00120 | PAO PROVENCO |
| 00513 | PAO PULLMAN |
| 00503 | PAO PULMEX |
| 06503 | PAO PULMEX DIET |
| 06515 | PAO PULMEX DIETETICO |
| 06603 | PAO PULMEX LIGHT |
| 00236 | PAO RECHEADO (DOCE) |
| 10036 | PAO RECHEADO (DOCE) DIET |
| 10076 | PAO RECHEADO (DOCE) DIETETICO |
| 00144 | PAO RECHEADO (SALGADO) |
| 00511 | PAO RECIFE |
| 00135 | PAO SACADURA |
| 00132 | PAO SEDA |
| 00149 | PAO SEDINHA |
| 00303 | PAO SEM MANTEIGA |
| 00130 | PAO SERIDO |
| 00502 | PAO SEVEN BOYS |
| 06502 | PAO SEVEN BOYS DIET |
| 06514 | PAO SEVEN BOYS DIETETICO |
| 06602 | PAO SEVEN BOYS LIGHT |
| 06402 | PAO SIRIO |
| 12102 | PAO SIRIO LIGHT |
| 09902 | PAO SIRIO LIGHT |
| 00115 | PAO SOVADO |
| 00116 | PAO SOVADO CABRITO |
| 00108 | PAO SUICO |
| 00227 | PAO SUPER FOFO |
| 10027 | PAO SUPER FOFO DIET |
| 10067 | PAO SUPER FOFO DIETETICO |
| 00133 | PAO TABICA |
| 01902 | PAO TORRADO (TORRADA) |
| 06902 | PAO TORRADO (TORRADA) LIGHT |
| 00211 | PAO TRANCA COM FAROFA |
| 10011 | PAO TRANCA COM FAROFA DIET |
| 10051 | PAO TRANCA COM FAROFA DIETETICO |
| 00228 | PAO VITA DOCE |
| 10028 | PAO VITA DOCE DIET |
| 10068 | PAO VITA DOCE DIETETICO |
| 00123 | PAO VITA SALGADO |
| 01804 | PAPA BOLINHAS (ASPIRADOR) |
| 01715 | PAPA DE ACAI |
| 01913 | PAPA DE BACABA |
| 02304 | PAPA DE BURITI |
| 03108 | PAPAIA |
| 08701 | PAPEIRO |
| 00212 | PAPEL |
| 05101 | PAPEL ABSORVENTE DE COPA E COZINHA |
| 05001 | PAPEL ALUMINIO OU ALUMINIZADO |
| 05004 | PAPEL ALUMINIO PARA FOGAO |
| 05201 | PAPEL CELOFANE |
| 04903 | PAPEL DE BALA |
| 01501 | PAPEL DE CIGARRO |
| 07001 | PAPEL DE PAREDE |
| 05401 | PAPEL DE PAREDE |
| 04201 | PAPEL DE PRESENTE |
| 01201 | PAPEL HIGIENICO |
| 08001 | PAPEL MANTEIGA |
| 01303 | PAPEL PARA DEPILACAO |
| 03901 | PAPEL TOALHA |
| 01207 | PAPINHA (ALIMENTO INFANTIL) |
| 04201 | PAPIRO |
| 04107 | PAPO DE ANJO |
| 13707 | PAPO DE ANJO DIET |
| 13715 | PAPO DE ANJO DIETETICO |
| 01301 | PAPRIKA |
| 03607 | PARA AFTA |
| 03501 | PARA AIDS |
| 00301 | PARA ALERGIA (ANTIALERGICO) |
| 02508 | PARA ALZHEIMER |
| 01401 | PARA ANEMIA |
| 01607 | PARA AS ARTICULACOES |
| 01302 | PARA ASMA |
| 01301 | PARA ASMA E BRONQUITE |
| 02628 | PARA ASSADURA |
| 00401 | PARA AZIA (ANTIACIDO) |
| 01303 | PARA BRONQUITE |
| 04701 | PARA CANCER |
| 02517 | PARA CIRCULACAO |
| 01202 | PARA COLESTEROL |
| 04403 | PARA COLUNA |
| 04406 | PARA CONTUSAO (ANALGESICO) |
| 02504 | PARA CONVULSAO |
| 01701 | PARA DEPRESSAO (ANTIDEPRESSIVO) |
| 02509 | PARA DERRAME CEREBRAL |
| 00901 | PARA DIABETE |
| 03001 | PARA DIARREIA |
| 04102 | PARA DOENCA DE CHAGAS |
| 04101 | PARA DOENCAS INFECCIOSAS E ENDEMICAS |
| 00101 | PARA DOR E FEBRE (ANALGESICO E ANTITERMICO) |
| 04404 | PARA DORES MUSCULARES |
| 01904 | PARA EMAGRECER |
| 00503 | PARA ENJOO |
| 00501 | PARA ENJOO E VOMITO (ANTIEMETICO) |
| 00104 | PARA ENXAQUECA |
| 02505 | PARA EPILEPSIA |
| 02704 | PARA ESTOMAGO |
| 01802 | PARA ESTRESSE |
| 03101 | PARA GASES E COLICA |
| 02703 | PARA GASTRITE |
| 03403 | PARA HEMORROIDAS |
| 02304 | PARA HEPATITE |
| 00807 | PARA HERPES |
| 00801 | PARA INFECCAO |
| 03606 | PARA INFECCOES DA BOCA E GARGANTA |
| 00701 | PARA INFLAMACAO E REUMATISMO (ANTIINFLAMATORIO E ANTI-REUMATICO) |
| 01102 | PARA INSUFICIENCIA CARDIOVASCULAR |
| 00604 | PARA INTESTINO |
| 03605 | PARA LABIRINTITE (ANTIINFLAMATORIO) |
| 02614 | PARA LUPUS |
| 04103 | PARA MALARIA |
| 02514 | PARA MEMORIA |
| 02204 | PARA MENOPAUSA |
| 02603 | PARA MICOSE (DERMATOLOGICO) |
| 01304 | PARA NEBULIZACAO |
| 01103 | PARA O CORACAO (INSUFICIENCIA CARDIOVASCULAR) |
| 02302 | PARA O FIGADO (HEPATICO) |
| 02301 | PARA O FIGADO PANCREAS E VIAS BILIARES |
| 02511 | PARA O MAL DE PARKINSON |
| 02306 | PARA O PANCREAS |
| 04104 | PARA O PULMAO |
| 02501 | PARA O SISTEMA NERVOSO |
| 01606 | PARA OS OSSOS |
| 01601 | PARA OS OSSOS E ARTICULACOES |
| 02401 | PARA OS RINS |
| 01602 | PARA OSTEOPOROSE |
| 04502 | PARA PIOLHO |
| 01001 | PARA PRESSAO ALTA (ANTI-HIPERTENSIVO) |
| 01003 | PARA PRESSAO ARTERIAL |
| 00601 | PARA PRISAO DE VENTRE (LAXANTE) |
| 01101 | PARA PROBLEMAS CARDIACOS E CIRCULATORIOS |
| 03601 | PARA PROBLEMAS DE BOCA, OUVIDO, NARIZ E GARGANTA |
| 04201 | PARA PROBLEMAS DE COAGULACAO |
| 02701 | PARA PROBLEMAS DE ESTOMAGO |
| 02601 | PARA PROBLEMAS DE PELE |
| 02801 | PARA PROBLEMAS GINECOLOGICOS |
| 04401 | PARA PROBLEMAS MUSCULARES |
| 02901 | PARA PROBLEMAS OCULARES (OFTALMOLOGICOS) |
| 04602 | PARA PROSTATA |
| 04601 | PARA PROSTATA E VIAS URINARIAS |
| 01506 | PARA PROTOZOARIOS |
| 02518 | PARA PSICOSE |
| 04705 | PARA QUIMIOTERAPIA |
| 00403 | PARA REFLUXO GASTRICO |
| 00704 | PARA REUMATISMO |
| 04505 | PARA SARNA |
| 03604 | PARA SINUSITE (ANTIINFLAMATORIO) |
| 02512 | PARA SISTEMA NERVOSO |
| 01803 | PARA STRESS |
| 03701 | PARA TIREOIDE |
| 03703 | PARA TIROIDE |
| 02515 | PARA TONTURA |
| 00201 | PARA TOSSE E RESFRIADO (ANTIGRIPAL E ANTITUSSIGENO) |
| 04004 | PARA TRATAMENTO DO ALCOOLISMO |
| 01204 | PARA TRIGLICERIDEO |
| 04105 | PARA TUBERCULOSE |
| 03401 | PARA VARIZES |
| 01503 | PARA VERMES |
| 01501 | PARA VERMES (VERMIFUGO) |
| 02516 | PARA VERTIGEM |
| 02307 | PARA VESICULA |
| 00502 | PARA VOMITO |
| 00804 | PARA-CHOQUE |
| 01201 | PARAFINA |
| 05001 | PARAFUSEIRA A BATERIA |
| 13517 | PARAGUAI LIGHT |
| 05402 | PARAGUAI REFRIGERANTE |
| 09102 | PARAGUAI REFRIGERANTE LIGHT |
| 03002 | PARAPEITO DE JANELA DE QUALQUER MATERIAL |
| 03002 | PARAPEITO DE JANELA DE QUALQUER MATERIAL |
| 03102 | PARAQUEDISMO |
| 03101 | PARCELAMENTO DE DIVIDA DE CONTA DE AGUA |
| 03201 | PARCELAMENTO DE DIVIDA DE ENERGIA ELETRICA |
| 00204 | PARCELAS PARA AQUISICAO DO IMOVEL A PRAZO |
| 01201 | PARQUE DE DIVERSOES (INGRESSO) |
| 03801 | PARQUES DE DIVERSAO |
| 05901 | PARTE DE FRANGO ORGANICO NAO-ESPECIFICADA |
| 00301 | PARTE DE GALINHA OU FRANGO NAO-ESPECIFICADA |
| 05905 | PARTE DE GALINHA OU FRANGO NAO-ESPECIFICADA ORGANICA |
| 03906 | PARTE DE PATO (NAO-ESPECIFICADA) |
| 01701 | PARTE DE PERU NAO-ESPECIFICADA |
| 03504 | PARTEIRA |
| 01903 | PARTICIPACAO EM PREJUIZO DE EMPRESA |
| 04501 | PARTICIPACAO NOS LUCROS DE EMPRESA (EMPREGADO) |
| 00301 | PARTICIPACAO NOS LUCROS DE NEGOCIOS (EMPREGADOR) |
| 05104 | PASMADA |
| 02402 | PASSA |
| 00301 | PASSADEIRA |
| 01501 | PASSADEIRA |
| 01702 | PASSADEIRA E LAVADEIRA |
| 01501 | PASSAGEM DE CAMINHAO |
| 01601 | PASSAGEM EM AUTOMOVEL |
| 01602 | PASSAGEM EM AUTOMOVEL UTILITARIO |
| 06901 | PASSARINHA BOVINA |
| 07001 | PASSARINHA SUINA |
| 02407 | PASSE BEM |
| 01203 | PASTA DE AMENDOIM |
| 07101 | PASTA DE AZEITONA |
| 03303 | PASTA DE BRILHO |
| 01220 | PASTA DE CACAU |
| 02302 | PASTA DE CARNE EM CONSERVA |
| 03302 | PASTA DE CARNE EMBUTIDA |
| 00103 | PASTA DE DENTE |
| 02202 | PASTA DE FIGADO EM CONSERVA |
| 03402 | PASTA DE FIGADO EMBUTIDA |
| 02502 | PASTA DE GALINHA EM CONSERVA |
| 03502 | PASTA DE GALINHA EMBUTIDA |
| 05902 | PASTA DE GALINHA EMBUTIDA LIGHT |
| 00102 | PASTA DE HOMEM |
| 04802 | PASTA DE LINGUA EM CONSERVA |
| 00249 | PASTA DE PAPELAO |
| 02603 | PASTA DE PATAUA |
| 03902 | PASTA DE PEIXE EM CONSERVA |
| 04702 | PASTA DE PERU EM CONSERVA |
| 02303 | PASTA DE POLIR PANELA |
| 02402 | PASTA DE PRESUNTO EM CONSERVA |
| 03202 | PASTA DE PRESUNTO EMBUTIDA |
| 05802 | PASTA DE PRESUNTO EMBUTIDA LIGHT |
| 03604 | PASTA DE SOJA |
| 00106 | PASTA DENTAL |
| 01901 | PASTA ESCOLAR |
| 02305 | PASTA JOIA |
| 00244 | PASTA PLASTICA NAO-ESCOLAR |
| 00248 | PASTA SUSPENSA |
| 08401 | PASTEIS DE SANTA CLARA |
| 03702 | PASTEL |
| 00202 | PASTEL PARA VIAGEM |
| 02604 | PASTEL SEMIPRONTO |
| 00606 | PASTILHA |
| 09906 | PASTILHA DIET |
| 09919 | PASTILHA DIETETICA |
| 09806 | PASTILHA LIGHT |
| 03603 | PASTILHA PARA GARGANTA |
| 02506 | PASTILHA SANITARIA |
| 02801 | PATAUA (COCO) |
| 02601 | PATAUA EM EMULSAO |
| 07102 | PATE DE AZEITONA |
| 03802 | PATE DE BERINJELA |
| 03801 | PATE DE BERINJELA EM CONSERVA |
| 02303 | PATE DE CALABRESA |
| 02301 | PATE DE CARNE EM CONSERVA |
| 03301 | PATE DE CARNE EMBUTIDO |
| 03702 | PATE DE CEBOLA |
| 03701 | PATE DE CEBOLA EM CONSERVA |
| 02201 | PATE DE FIGADO EM CONSERVA |
| 03401 | PATE DE FIGADO EMBUTIDO |
| 02503 | PATE DE FRANGO EM CONSERVA |
| 01239 | PATE DE FRUTAS |
| 02501 | PATE DE GALINHA EM CONSERVA |
| 03501 | PATE DE GALINHA EMBUTIDO |
| 05901 | PATE DE GALINHA EMBUTIDO LIGHT |
| 04801 | PATE DE LINGUA EM CONSERVA |
| 03901 | PATE DE PEIXE EM CONSERVA |
| 04701 | PATE DE PERU EM CONSERVA |
| 02401 | PATE DE PRESUNTO EM CONSERVA |
| 03201 | PATE DE PRESUNTO EMBUTIDO |
| 05801 | PATE DE PRESUNTO EMBUTIDO LIGHT |
| 07504 | PATE DE QUEIJO |
| 01205 | PATE INFANTIL (EXCETO SOBREMESA) |
| 04001 | PATE NAO-ESPECIFICADO |
| 00310 | PATIM |
| 00301 | PATIM, SKATE, VELOCIPEDE |
| 00307 | PATINETE |
| 00501 | PATINHO |
| 00504 | PATINHO COM OSSO |
| 11704 | PATINHO COM OSSO ORGANICO |
| 11701 | PATINHO ORGANICO |
| 03904 | PATO ABATIDO |
| 03901 | PATO ABATIDO, CONGELADO OU VIVO |
| 03905 | PATO CONGELADO |
| 03903 | PATO EM PE |
| 03907 | PATO EM PEDACOS (NAO-ESPECIFICADOS) |
| 03902 | PATO VIVO |
| 00606 | PAU DE LARANJEIRA |
| 01503 | PAU-DE-ARARA |
| 00607 | PAULISTA |
| 11807 | PAULISTA ORGANICO |
| 00704 | PAULISTA PLANO |
| 11904 | PAULISTA PLANO ORGANICO |
| 00603 | PAULISTA REDONDO |
| 11803 | PAULISTA REDONDO ORGANICO |
| 45115 | PAULISTINHA |
| 04202 | PAVE |
| 13802 | PAVE DIET |
| 13807 | PAVE DIETETICO |
| 02201 | PAX DOMINNI (AUXILIO FUNERAL) |
| 10605 | PDA |
| 03102 | PE BOVINO |
| 06201 | PE DE CAPRINO |
| 00902 | PE DE FRANGO |
| 00901 | PE DE GALINHA OU FRANGO |
| 03202 | PE DE MOLEQUE |
| 13102 | PE DE MOLEQUE DIET |
| 13111 | PE DE MOLEQUE DIETETICO |
| 01202 | PE DE PORCO DEFUMADO |
| 01701 | PE DE PORCO SALGADO |
| 03202 | PE E ASA DE FRANGO |
| 03201 | PE E ASA DE GALINHA OU FRANGO |
| 08301 | PE SUINO FRESCO |
| 02105 | PEBOLIM |
| 04901 | PECA DE AGATA OU ESMALTADA (DECORACAO) |
| 01901 | PECA DE CERAMICA (DECORACAO) |
| 04001 | PECA DE CORDA (DECORACAO) |
| 02601 | PECA DE CRISTAL (DECORACAO) |
| 02201 | PECA DE GESSO (DECORACAO) |
| 01101 | PECA DE LOUCA (DECORACAO) |
| 02401 | PECA DE MADEIRA (DECORACAO) |
| 01201 | PECA DE METAL (EXCETO PRATA) (DECORACAO) |
| 03301 | PECA DE PALHA (DECORACAO) |
| 04101 | PECA DE PEDRA SABAO (DECORACAO) |
| 03801 | PECA DE PLASTICO OU RESINA (DECORACAO) |
| 02901 | PECA DE PORCELANA (DECORACAO) |
| 01301 | PECA DE PRATA (DECORACAO) |
| 04301 | PECA DE TECIDO (DECORACAO) |
| 03001 | PECA DE VIDRO (DECORACAO) |
| 00303 | PECA ELETRICA |
| 00301 | PECA ELETRICA OU MECANICA |
| 00302 | PECA MECANICA |
| 00206 | PECA PARA SUPORTE DE PAPEL HIGIENICO |
| 00206 | PECA PARA SUPORTE DE PAPEL HIGIENICO |
| 04602 | PECAS DE CONFECCAO DE FLORES ARTIFICIAIS |
| 02002 | PECULIO |
| 02502 | PECULIO (RECEBIMENTO) |
| 02001 | PECULIO DOMICILIAR |
| 00901 | PEDAGIO |
| 01301 | PEDAGIO |
| 00902 | PEDALETE (BICICLETA DE GINASTICA) |
| 03701 | PEDALINHOS |
| 01616 | PE-DE-CABRA |
| 02201 | PE-DE-COELHO |
| 00114 | PE-DE-PATO |
| 03901 | PEDESTAL |
| 00302 | PEDICURO |
| 02302 | PEDRA BRITADA |
| 02302 | PEDRA BRITADA |
| 02306 | PEDRA DE ALICERCE |
| 02306 | PEDRA DE ALICERCE |
| 01901 | PEDRA DE AMOLAR |
| 01001 | PEDRA DE ISQUEIRO |
| 03101 | PEDRA MARMORE PARA PIA |
| 03101 | PEDRA MARMORE PARA PIA |
| 02305 | PEDRA MARROADA |
| 02305 | PEDRA MARROADA |
| 09501 | PEDRA PARA POLIR FOGAO |
| 01105 | PEDRA POME |
| 04102 | PEDRA SABAO (PECA DE DECORACAO) |
| 02515 | PEDRA SANITARIA |
| 02301 | PEDRAS (BRITADA, MARROADA, PARALELEPIPEDO, ETC.) |
| 02301 | PEDRAS (BRITADA, MARROADA, PARALELEPIPEDO, ETC.) |
| 05201 | PEDRAS DECORATIVAS |
| 01302 | PEDREIRO |
| 01302 | PEDREIRO |
| 00602 | PEELING |
| 01101 | PEITO BOVINO |
| 00403 | PEITO DE FRANGO |
| 06801 | PEITO DE FRANGO LIGHT |
| 05701 | PEITO DE FRANGO ORGANICO |
| 04102 | PEITO DE GALINHA DEFUMADO |
| 00401 | PEITO DE GALINHA OU FRANGO |
| 05704 | PEITO DE GALINHA OU FRANGO ORGANICO |
| 03908 | PEITO DE PATO |
| 01801 | PEITO DE PERU |
| 01802 | PEITO DE PERU DEFUMADO |
| 05401 | PEITO DE PERU LIGHT |
| 03101 | PEIXE ANCHOVA EM CONSERVA |
| 01802 | PEIXE ASSADO PARA VIAGEM |
| 03401 | PEIXE ATUM EM CONSERVA |
| 06301 | PEIXE ATUM EM CONSERVA LIGHT |
| 14901 | PEIXE BAGRE DE MAR ( FORMA DE COMERCIALIZACAO NAO-DISCRIMINADA) |
| 23118 | PEIXE BAGRE DE RIO |
| 04101 | PEIXE BONITO EM CONSERVA |
| 04901 | PEIXE CASTANHA (FORMA DE COMERCIALIZACAO NAO-DISCRIMINADA) |
| 16901 | PEIXE CAVALA (FORMA DE COMERCIALIZACAO NAO-DISCRIMINADA) |
| 03301 | PEIXE CAVALINHA EM CONSERVA |
| 03901 | PEIXE CORVINA (FORMA DE COMERCIALIZACAO NAO-DISCRIMINADA) |
| 03201 | PEIXE CORVINA EM CONSERVA |
| 01803 | PEIXE COZIDO PARA VIAGEM |
| 10901 | PEIXE CURIMATA (FORMA DE COMERCIALIZACAO NAO-DISCRIMINADA) |
| 11601 | PEIXE DEFUMADO SARDINHA |
| 32901 | PEIXE DOURADA (FORMA DE COMERCIALIZACAO NAO-DISCRIMINADA) |
| 75501 | PEIXE EM FILE CONGELADO ABROTE |
| 02501 | PEIXE EM FILE CONGELADO ACARA |
| 12501 | PEIXE EM FILE CONGELADO ACARI |
| 44501 | PEIXE EM FILE CONGELADO AGULHA |
| 58501 | PEIXE EM FILE CONGELADO AGULHAO |
| 56501 | PEIXE EM FILE CONGELADO ALBACORA |
| 01501 | PEIXE EM FILE CONGELADO ANCHOVA |
| 23501 | PEIXE EM FILE CONGELADO ANUJA |
| 07501 | PEIXE EM FILE CONGELADO ARACU |
| 40501 | PEIXE EM FILE CONGELADO ARUANA |
| 22501 | PEIXE EM FILE CONGELADO ATUM |
| 29501 | PEIXE EM FILE CONGELADO BACU |
| 02501 | PEIXE EM FILE CONGELADO BADEJO |
| 46501 | PEIXE EM FILE CONGELADO BAGRE DE AGUA DOCE |
| 14501 | PEIXE EM FILE CONGELADO BAGRE DE MAR |
| 37501 | PEIXE EM FILE CONGELADO BEIJUPIRA |
| 47501 | PEIXE EM FILE CONGELADO BICUDA |
| 51501 | PEIXE EM FILE CONGELADO BODIAO |
| 49501 | PEIXE EM FILE CONGELADO BONITO |
| 35501 | PEIXE EM FILE CONGELADO CACAO |
| 26501 | PEIXE EM FILE CONGELADO CACHORRO |
| 71501 | PEIXE EM FILE CONGELADO CAICANHA |
| 42501 | PEIXE EM FILE CONGELADO CAMURUPIM |
| 41501 | PEIXE EM FILE CONGELADO CANGULO |
| 40501 | PEIXE EM FILE CONGELADO CARAMURU |
| 15501 | PEIXE EM FILE CONGELADO CARAPEBA |
| 12502 | PEIXE EM FILE CONGELADO CASCUDO |
| 04501 | PEIXE EM FILE CONGELADO CASTANHA |
| 16501 | PEIXE EM FILE CONGELADO CAVALA |
| 05501 | PEIXE EM FILE CONGELADO CAVALINHA |
| 57501 | PEIXE EM FILE CONGELADO CHERNE |
| 06501 | PEIXE EM FILE CONGELADO CHICHARRO |
| 13501 | PEIXE EM FILE CONGELADO CIOBA |
| 18501 | PEIXE EM FILE CONGELADO CORCOROCA |
| 03501 | PEIXE EM FILE CONGELADO CORVINA |
| 10501 | PEIXE EM FILE CONGELADO CURIMATA |
| 00501 | PEIXE EM FILE CONGELADO DE AGUA DOCE NAO-ESPECIFICADO |
| 00501 | PEIXE EM FILE CONGELADO DE MAR NAO-ESPECIFICADO |
| 32501 | PEIXE EM FILE CONGELADO DOURADA |
| 16501 | PEIXE EM FILE CONGELADO DOURADO DE AGUA DOCE |
| 21501 | PEIXE EM FILE CONGELADO DOURADO DE MAR |
| 33501 | PEIXE EM FILE CONGELADO ESPADA |
| 34501 | PEIXE EM FILE CONGELADO FIDALGO |
| 17501 | PEIXE EM FILE CONGELADO GALO |
| 28501 | PEIXE EM FILE CONGELADO GAROUPA |
| 46501 | PEIXE EM FILE CONGELADO GUAIVIRA |
| 43501 | PEIXE EM FILE CONGELADO GUARAJUBA |
| 48501 | PEIXE EM FILE CONGELADO ITUI |
| 05501 | PEIXE EM FILE CONGELADO JACUNDA |
| 25501 | PEIXE EM FILE CONGELADO JARAQUI |
| 38501 | PEIXE EM FILE CONGELADO JAU |
| 09501 | PEIXE EM FILE CONGELADO JEJU |
| 03501 | PEIXE EM FILE CONGELADO LAMBARI |
| 29501 | PEIXE EM FILE CONGELADO LINGUADO |
| 36501 | PEIXE EM FILE CONGELADO MANDUBI |
| 19501 | PEIXE EM FILE CONGELADO MANJUBA |
| 19501 | PEIXE EM FILE CONGELADO MAPARA |
| 51501 | PEIXE EM FILE CONGELADO MATRINXA |
| 36501 | PEIXE EM FILE CONGELADO MERLUZA |
| 31501 | PEIXE EM FILE CONGELADO MERO |
| 53501 | PEIXE EM FILE CONGELADO MIRAGAIA |
| 55501 | PEIXE EM FILE CONGELADO MOREIA |
| 15501 | PEIXE EM FILE CONGELADO MUCUM |
| 08501 | PEIXE EM FILE CONGELADO NAMORADO |
| 00501 | PEIXE EM FILE CONGELADO NAO-ESPECIFICADO |
| 23501 | PEIXE EM FILE CONGELADO OLHO DE BOI |
| 31501 | PEIXE EM FILE CONGELADO PACAMAO DE AGUA DOCE |
| 21501 | PEIXE EM FILE CONGELADO PACU |
| 52501 | PEIXE EM FILE CONGELADO PALOMBETA |
| 38501 | PEIXE EM FILE CONGELADO PAMPO |
| 20501 | PEIXE EM FILE CONGELADO PAPATERRA |
| 34501 | PEIXE EM FILE CONGELADO PARATI |
| 25501 | PEIXE EM FILE CONGELADO PARGO |
| 27501 | PEIXE EM FILE CONGELADO PESCADA |
| 09501 | PEIXE EM FILE CONGELADO PESCADINHA |
| 27501 | PEIXE EM FILE CONGELADO PIABANHA |
| 39501 | PEIXE EM FILE CONGELADO PIAPARA |
| 14501 | PEIXE EM FILE CONGELADO PIAU |
| 41501 | PEIXE EM FILE CONGELADO PIRACANJUBA |
| 28501 | PEIXE EM FILE CONGELADO PIRAIBA |
| 13501 | PEIXE EM FILE CONGELADO PIRAMUTABA |
| 33501 | PEIXE EM FILE CONGELADO PIRANAMBU |
| 20501 | PEIXE EM FILE CONGELADO PIRANHA |
| 18501 | PEIXE EM FILE CONGELADO PIRAPEUA |
| 37501 | PEIXE EM FILE CONGELADO PIRAPUTANGA |
| 30501 | PEIXE EM FILE CONGELADO PIRARARA |
| 06501 | PEIXE EM FILE CONGELADO PIRARUCU |
| 07501 | PEIXE EM FILE CONGELADO RAIA |
| 54501 | PEIXE EM FILE CONGELADO REI (PEIXE REI) |
| 30501 | PEIXE EM FILE CONGELADO ROBALO |
| 26501 | PEIXE EM FILE CONGELADO RONCADOR |
| 42501 | PEIXE EM FILE CONGELADO SAIRU |
| 80501 | PEIXE EM FILE CONGELADO SALMAO |
| 59501 | PEIXE EM FILE CONGELADO SALMONETE |
| 11501 | PEIXE EM FILE CONGELADO SARDINHA |
| 24501 | PEIXE EM FILE CONGELADO SARDINHA DE AGUA DOCE |
| 50501 | PEIXE EM FILE CONGELADO SAVELHA |
| 24501 | PEIXE EM FILE CONGELADO SERRA |
| 04501 | PEIXE EM FILE CONGELADO SURUBIM |
| 35501 | PEIXE EM FILE CONGELADO TABARANA |
| 10501 | PEIXE EM FILE CONGELADO TAINHA |
| 22501 | PEIXE EM FILE CONGELADO TAMBAQUI |
| 17501 | PEIXE EM FILE CONGELADO TAMOATA |
| 08501 | PEIXE EM FILE CONGELADO TILAPIA |
| 72501 | PEIXE EM FILE CONGELADO TIRAVIRA |
| 01501 | PEIXE EM FILE CONGELADO TRAIRA |
| 48501 | PEIXE EM FILE CONGELADO TRILHA |
| 49501 | PEIXE EM FILE CONGELADO TRUTA |
| 11501 | PEIXE EM FILE CONGELADO TUCUNARE |
| 45501 | PEIXE EM FILE CONGELADO UBARANA |
| 12501 | PEIXE EM FILE CONGELADO VERMELHO |
| 39501 | PEIXE EM FILE CONGELADO XAREU |
| 32501 | PEIXE EM FILE CONGELADO XERELETE |
| 75301 | PEIXE EM FILE FRESCO ABROTE |
| 02301 | PEIXE EM FILE FRESCO ACARA |
| 12301 | PEIXE EM FILE FRESCO ACARI |
| 44301 | PEIXE EM FILE FRESCO AGULHA |
| 58301 | PEIXE EM FILE FRESCO AGULHAO |
| 56301 | PEIXE EM FILE FRESCO ALBACORA |
| 01301 | PEIXE EM FILE FRESCO ANCHOVA |
| 23301 | PEIXE EM FILE FRESCO ANUJA |
| 07301 | PEIXE EM FILE FRESCO ARACU |
| 40301 | PEIXE EM FILE FRESCO ARUANA |
| 22301 | PEIXE EM FILE FRESCO ATUM |
| 29301 | PEIXE EM FILE FRESCO BACU |
| 02301 | PEIXE EM FILE FRESCO BADEJO |
| 46301 | PEIXE EM FILE FRESCO BAGRE DE AGUA DOCE |
| 14301 | PEIXE EM FILE FRESCO BAGRE DE MAR |
| 37301 | PEIXE EM FILE FRESCO BEIJUPIRA |
| 47301 | PEIXE EM FILE FRESCO BICUDA |
| 51301 | PEIXE EM FILE FRESCO BODIAO |
| 49301 | PEIXE EM FILE FRESCO BONITO |
| 35301 | PEIXE EM FILE FRESCO CACAO |
| 26301 | PEIXE EM FILE FRESCO CACHORRO |
| 71301 | PEIXE EM FILE FRESCO CAICANHA |
| 42301 | PEIXE EM FILE FRESCO CAMURUPIM |
| 41301 | PEIXE EM FILE FRESCO CANGULO |
| 40301 | PEIXE EM FILE FRESCO CARAMURU |
| 15301 | PEIXE EM FILE FRESCO CARAPEBA |
| 55301 | PEIXE EM FILE FRESCO CARPA |
| 04301 | PEIXE EM FILE FRESCO CASTANHA |
| 16301 | PEIXE EM FILE FRESCO CAVALA |
| 05301 | PEIXE EM FILE FRESCO CAVALINHA |
| 57301 | PEIXE EM FILE FRESCO CHERNE |
| 06301 | PEIXE EM FILE FRESCO CHICHARRO |
| 13301 | PEIXE EM FILE FRESCO CIOBA |
| 18301 | PEIXE EM FILE FRESCO CORCOROCA |
| 03301 | PEIXE EM FILE FRESCO CORVINA |
| 10301 | PEIXE EM FILE FRESCO CURIMATA |
| 00301 | PEIXE EM FILE FRESCO DE AGUA DOCE NAO-ESPECIFICADO |
| 00301 | PEIXE EM FILE FRESCO DE MAR NAO-ESPECIFICADO |
| 27601 | PEIXE EM FILE FRESCO DE PIRAPITINGA |
| 32301 | PEIXE EM FILE FRESCO DOURADA |
| 16301 | PEIXE EM FILE FRESCO DOURADO DE AGUA DOCE |
| 21301 | PEIXE EM FILE FRESCO DOURADO DE MAR |
| 33301 | PEIXE EM FILE FRESCO ESPADA |
| 34301 | PEIXE EM FILE FRESCO FIDALGO |
| 17301 | PEIXE EM FILE FRESCO GALO |
| 28301 | PEIXE EM FILE FRESCO GAROUPA |
| 46301 | PEIXE EM FILE FRESCO GUAIVIRA |
| 43301 | PEIXE EM FILE FRESCO GUARAJUBA |
| 48301 | PEIXE EM FILE FRESCO ITUI |
| 05301 | PEIXE EM FILE FRESCO JACUNDA |
| 25301 | PEIXE EM FILE FRESCO JARAQUI |
| 38301 | PEIXE EM FILE FRESCO JAU |
| 09301 | PEIXE EM FILE FRESCO JEJU |
| 03301 | PEIXE EM FILE FRESCO LAMBARI |
| 29301 | PEIXE EM FILE FRESCO LINGUADO |
| 36301 | PEIXE EM FILE FRESCO MANDUBE |
| 19301 | PEIXE EM FILE FRESCO MANJUBA |
| 19301 | PEIXE EM FILE FRESCO MAPARA |
| 51301 | PEIXE EM FILE FRESCO MATRINXA |
| 36301 | PEIXE EM FILE FRESCO MERLUZA |
| 31301 | PEIXE EM FILE FRESCO MERO |
| 53301 | PEIXE EM FILE FRESCO MIRAGAIA |
| 55301 | PEIXE EM FILE FRESCO MOREIA |
| 15301 | PEIXE EM FILE FRESCO MUCUM |
| 08301 | PEIXE EM FILE FRESCO NAMORADO |
| 00301 | PEIXE EM FILE FRESCO NAO-ESPECIFICADO |
| 23301 | PEIXE EM FILE FRESCO OLHO DE BOI |
| 31301 | PEIXE EM FILE FRESCO PACAMAO DE AGUA DOCE |
| 21301 | PEIXE EM FILE FRESCO PACU |
| 52301 | PEIXE EM FILE FRESCO PALOMBETA |
| 38301 | PEIXE EM FILE FRESCO PAMPO |
| 20301 | PEIXE EM FILE FRESCO PAPATERRA |
| 34301 | PEIXE EM FILE FRESCO PARATI |
| 25301 | PEIXE EM FILE FRESCO PARGO |
| 27301 | PEIXE EM FILE FRESCO PESCADA |
| 09301 | PEIXE EM FILE FRESCO PESCADINHA |
| 27301 | PEIXE EM FILE FRESCO PIABANHA |
| 39301 | PEIXE EM FILE FRESCO PIAPARA |
| 14301 | PEIXE EM FILE FRESCO PIAU |
| 41301 | PEIXE EM FILE FRESCO PIRACANJUBA |
| 28301 | PEIXE EM FILE FRESCO PIRAIBA |
| 13301 | PEIXE EM FILE FRESCO PIRAMUTABA |
| 33301 | PEIXE EM FILE FRESCO PIRANAMBU |
| 20301 | PEIXE EM FILE FRESCO PIRANHA |
| 18301 | PEIXE EM FILE FRESCO PIRAPEUA |
| 37301 | PEIXE EM FILE FRESCO PIRAPUTANGA |
| 30301 | PEIXE EM FILE FRESCO PIRARARA |
| 06301 | PEIXE EM FILE FRESCO PIRARUCU |
| 07301 | PEIXE EM FILE FRESCO RAIA |
| 54301 | PEIXE EM FILE FRESCO REI (PEIXE REI) |
| 30301 | PEIXE EM FILE FRESCO ROBALO |
| 26301 | PEIXE EM FILE FRESCO RONCADOR |
| 42301 | PEIXE EM FILE FRESCO SAIRU |
| 59301 | PEIXE EM FILE FRESCO SALMONETE |
| 11301 | PEIXE EM FILE FRESCO SARDINHA |
| 24301 | PEIXE EM FILE FRESCO SARDINHA DE AGUA DOCE |
| 50301 | PEIXE EM FILE FRESCO SAVELHA |
| 24301 | PEIXE EM FILE FRESCO SERRA |
| 04301 | PEIXE EM FILE FRESCO SURUBIM |
| 35301 | PEIXE EM FILE FRESCO TABARANA |
| 10301 | PEIXE EM FILE FRESCO TAINHA |
| 22301 | PEIXE EM FILE FRESCO TAMBAQUI |
| 17301 | PEIXE EM FILE FRESCO TAMOATA |
| 08301 | PEIXE EM FILE FRESCO TILAPIA |
| 72301 | PEIXE EM FILE FRESCO TIRAVIRA |
| 01301 | PEIXE EM FILE FRESCO TRAIRA |
| 48301 | PEIXE EM FILE FRESCO TRILHA |
| 49301 | PEIXE EM FILE FRESCO TRUTA |
| 11301 | PEIXE EM FILE FRESCO TUCUNARE |
| 45301 | PEIXE EM FILE FRESCO UBARANA |
| 12301 | PEIXE EM FILE FRESCO VERMELHO |
| 07302 | PEIXE EM FILE FRESCO VIOLA |
| 39301 | PEIXE EM FILE FRESCO XAREU |
| 32301 | PEIXE EM FILE FRESCO XERELETE |
| 05501 | PEIXE EM PO |
| 75201 | PEIXE EM POSTAS ABROTE |
| 02201 | PEIXE EM POSTAS ACARA |
| 12201 | PEIXE EM POSTAS ACARI |
| 44201 | PEIXE EM POSTAS AGULHA |
| 58201 | PEIXE EM POSTAS AGULHAO |
| 56201 | PEIXE EM POSTAS ALBACORA |
| 01201 | PEIXE EM POSTAS ANCHOVA |
| 23201 | PEIXE EM POSTAS ANUJA |
| 07201 | PEIXE EM POSTAS ARACU |
| 40201 | PEIXE EM POSTAS ARUANA |
| 22201 | PEIXE EM POSTAS ATUM |
| 29201 | PEIXE EM POSTAS BACU |
| 02201 | PEIXE EM POSTAS BADEJO |
| 46201 | PEIXE EM POSTAS BAGRE DE AGUA DOCE |
| 14201 | PEIXE EM POSTAS BAGRE DE MAR |
| 37201 | PEIXE EM POSTAS BEIJUPIRA |
| 47201 | PEIXE EM POSTAS BICUDA |
| 51201 | PEIXE EM POSTAS BODIAO |
| 49201 | PEIXE EM POSTAS BONITO |
| 35201 | PEIXE EM POSTAS CACAO |
| 26201 | PEIXE EM POSTAS CACHORRO |
| 71201 | PEIXE EM POSTAS CAICANHA |
| 42201 | PEIXE EM POSTAS CAMURUPIM |
| 41201 | PEIXE EM POSTAS CANGULO |
| 40201 | PEIXE EM POSTAS CARAMURU |
| 15201 | PEIXE EM POSTAS CARAPEBA |
| 04201 | PEIXE EM POSTAS CASTANHA |
| 16201 | PEIXE EM POSTAS CAVALA |
| 05201 | PEIXE EM POSTAS CAVALINHA |
| 57201 | PEIXE EM POSTAS CHERNE |
| 06201 | PEIXE EM POSTAS CHICHARRO |
| 13201 | PEIXE EM POSTAS CIOBA |
| 18201 | PEIXE EM POSTAS CORCOROCA |
| 03201 | PEIXE EM POSTAS CORVINA |
| 10201 | PEIXE EM POSTAS CURIMATA |
| 00201 | PEIXE EM POSTAS DE AGUA DOCE NAO-ESPECIFICADO |
| 00201 | PEIXE EM POSTAS DE MAR NAO-ESPECIFICADO |
| 32201 | PEIXE EM POSTAS DOURADA |
| 16201 | PEIXE EM POSTAS DOURADO DE AGUA DOCE |
| 21201 | PEIXE EM POSTAS DOURADO DE MAR |
| 33201 | PEIXE EM POSTAS ESPADA |
| 34201 | PEIXE EM POSTAS FIDALGO |
| 17201 | PEIXE EM POSTAS GALO |
| 28201 | PEIXE EM POSTAS GAROUPA |
| 46201 | PEIXE EM POSTAS GUAIVIRA |
| 43201 | PEIXE EM POSTAS GUARAJUBA |
| 48201 | PEIXE EM POSTAS ITUI |
| 05201 | PEIXE EM POSTAS JACUNDA |
| 25201 | PEIXE EM POSTAS JARAQUI |
| 38201 | PEIXE EM POSTAS JAU |
| 09201 | PEIXE EM POSTAS JEJU |
| 03201 | PEIXE EM POSTAS LAMBARI |
| 29201 | PEIXE EM POSTAS LINGUADO |
| 36201 | PEIXE EM POSTAS MANDUBE |
| 19201 | PEIXE EM POSTAS MANJUBA |
| 19201 | PEIXE EM POSTAS MAPARA |
| 51201 | PEIXE EM POSTAS MATRINXA |
| 36201 | PEIXE EM POSTAS MERLUZA |
| 31201 | PEIXE EM POSTAS MERO |
| 53201 | PEIXE EM POSTAS MIRAGAIA |
| 55201 | PEIXE EM POSTAS MOREIA |
| 15201 | PEIXE EM POSTAS MUCUM |
| 08201 | PEIXE EM POSTAS NAMORADO |
| 00201 | PEIXE EM POSTAS NAO-ESPECIFICADO |
| 23201 | PEIXE EM POSTAS OLHO DE BOI |
| 31201 | PEIXE EM POSTAS PACAMAO DE AGUA DOCE |
| 21201 | PEIXE EM POSTAS PACU |
| 52201 | PEIXE EM POSTAS PALOMBETA |
| 38201 | PEIXE EM POSTAS PAMPO |
| 20201 | PEIXE EM POSTAS PAPATERRA |
| 34201 | PEIXE EM POSTAS PARATI |
| 25201 | PEIXE EM POSTAS PARGO |
| 27201 | PEIXE EM POSTAS PESCADA |
| 09201 | PEIXE EM POSTAS PESCADINHA |
| 27201 | PEIXE EM POSTAS PIABANHA |
| 39201 | PEIXE EM POSTAS PIAPARA |
| 14201 | PEIXE EM POSTAS PIAU |
| 41201 | PEIXE EM POSTAS PIRACANJUBA |
| 28201 | PEIXE EM POSTAS PIRAIBA |
| 13201 | PEIXE EM POSTAS PIRAMUTABA |
| 33201 | PEIXE EM POSTAS PIRANAMBU |
| 20201 | PEIXE EM POSTAS PIRANHA |
| 18201 | PEIXE EM POSTAS PIRAPEUA |
| 37201 | PEIXE EM POSTAS PIRAPUTANGA |
| 30201 | PEIXE EM POSTAS PIRARARA |
| 06201 | PEIXE EM POSTAS PIRARUCU |
| 07201 | PEIXE EM POSTAS RAIA |
| 54201 | PEIXE EM POSTAS REI (PEIXE REI) |
| 30201 | PEIXE EM POSTAS ROBALO |
| 26201 | PEIXE EM POSTAS RONCADOR |
| 42201 | PEIXE EM POSTAS SAIRU |
| 80201 | PEIXE EM POSTAS SALMAO |
| 59201 | PEIXE EM POSTAS SALMONETE |
| 11201 | PEIXE EM POSTAS SARDINHA |
| 24201 | PEIXE EM POSTAS SARDINHA DE AGUA DOCE |
| 50201 | PEIXE EM POSTAS SAVELHA |
| 24201 | PEIXE EM POSTAS SERRA |
| 04201 | PEIXE EM POSTAS SURUBIM |
| 35201 | PEIXE EM POSTAS TABARANA |
| 10201 | PEIXE EM POSTAS TAINHA |
| 01102 | PEIXE EM POSTAS TAMBACU |
| 22201 | PEIXE EM POSTAS TAMBAQUI |
| 17201 | PEIXE EM POSTAS TAMOATA |
| 08201 | PEIXE EM POSTAS TILAPIA |
| 72201 | PEIXE EM POSTAS TIRAVIRA |
| 01201 | PEIXE EM POSTAS TRAIRA |
| 48201 | PEIXE EM POSTAS TRILHA |
| 49201 | PEIXE EM POSTAS TRUTA |
| 11201 | PEIXE EM POSTAS TUCUNARE |
| 45201 | PEIXE EM POSTAS UBARANA |
| 12201 | PEIXE EM POSTAS VERMELHO |
| 39201 | PEIXE EM POSTAS XAREU |
| 32201 | PEIXE EM POSTAS XERELETE |
| 03102 | PEIXE ENCHOVA EM CONSERVA |
| 34901 | PEIXE FIDALGO (FORMA DE COMERCIALIZACAO NAO-DISCRIMINADA) |
| 01801 | PEIXE FRITO PARA VIAGEM |
| 21118 | PEIXE INTEIRO ABEL |
| 18115 | PEIXE INTEIRO ABIQUARA |
| 29102 | PEIXE INTEIRO ABOTOADO |
| 75101 | PEIXE INTEIRO ABROTE |
| 02101 | PEIXE INTEIRO ACARA |
| 02110 | PEIXE INTEIRO ACARA ACU |
| 02112 | PEIXE INTEIRO ACARA FUSO |
| 41115 | PEIXE INTEIRO ACARAMOCO (CANGULO) |
| 41110 | PEIXE INTEIRO ACARAMUCU (CANGULO) |
| 02105 | PEIXE INTEIRO ACARAPEBA |
| 15106 | PEIXE INTEIRO ACARAPEBA DE MAR |
| 15108 | PEIXE INTEIRO ACARAPEVA |
| 02109 | PEIXE INTEIRO ACARAUNA |
| 12101 | PEIXE INTEIRO ACARI |
| 12125 | PEIXE INTEIRO ACARI BODO |
| 12124 | PEIXE INTEIRO ACARI CACHIMBO |
| 12122 | PEIXE INTEIRO ACARI CHICOTE |
| 12123 | PEIXE INTEIRO ACARI ESPADA |
| 12121 | PEIXE INTEIRO ACARI LARANJA |
| 12119 | PEIXE INTEIRO ACARI MOLE |
| 12116 | PEIXE INTEIRO ACARI PINTADO |
| 12120 | PEIXE INTEIRO ACARI VIOLA |
| 12118 | PEIXE INTEIRO ACARIJUBA |
| 44101 | PEIXE INTEIRO AGULHA |
| 44106 | PEIXE INTEIRO AGULHA BRANCA |
| 44103 | PEIXE INTEIRO AGULHA CRIOULA |
| 44102 | PEIXE INTEIRO AGULHA PRETA |
| 58101 | PEIXE INTEIRO AGULHAO |
| 58102 | PEIXE INTEIRO AGULHAO BANDEIRA |
| 58111 | PEIXE INTEIRO AGULHAO BRANCO |
| 58103 | PEIXE INTEIRO AGULHAO DE VELA |
| 58106 | PEIXE INTEIRO AGULHAO PRETO |
| 58112 | PEIXE INTEIRO AGULHAO VELA |
| 03119 | PEIXE INTEIRO ALAMBARI |
| 56101 | PEIXE INTEIRO ALBACORA |
| 22102 | PEIXE INTEIRO ALBACORA AZUL |
| 56106 | PEIXE INTEIRO ALBACORA BANDOLIM |
| 56104 | PEIXE INTEIRO ALBACORA BRANCA |
| 56102 | PEIXE INTEIRO ALBACORA LAJE |
| 56105 | PEIXE INTEIRO ALBACORA LAJEIRA |
| 56103 | PEIXE INTEIRO ALBACORA MIRIM |
| 56110 | PEIXE INTEIRO ALBACORA OLHAO |
| 56107 | PEIXE INTEIRO ALBACORINHA |
| 45104 | PEIXE INTEIRO ALBARANA |
| 17112 | PEIXE INTEIRO ALFAQUIM |
| 56109 | PEIXE INTEIRO ALVACORA |
| 40103 | PEIXE INTEIRO AMANA |
| 12127 | PEIXE INTEIRO ANAIA |
| 06105 | PEIXE INTEIRO ANATO |
| 19106 | PEIXE INTEIRO ANCHOITA |
| 01101 | PEIXE INTEIRO ANCHOVA |
| 01105 | PEIXE INTEIRO ANCHOVA BAETA |
| 01103 | PEIXE INTEIRO ANCHOVETA |
| 01108 | PEIXE INTEIRO ANCHOVINHA |
| 36102 | PEIXE INTEIRO ANDUBE |
| 36108 | PEIXE INTEIRO ANDUBI |
| 35134 | PEIXE INTEIRO ANJO |
| 23103 | PEIXE INTEIRO ANOJADO |
| 23106 | PEIXE INTEIRO ANUIA |
| 23101 | PEIXE INTEIRO ANUJA |
| 31102 | PEIXE INTEIRO APACAMA DE AGUA DOCE |
| 02126 | PEIXE INTEIRO APAIARI |
| 24102 | PEIXE INTEIRO APAPA |
| 12117 | PEIXE INTEIRO APERTA GALHA |
| 23106 | PEIXE INTEIRO ARABAIANA |
| 38117 | PEIXE INTEIRO ARABEBEL |
| 38127 | PEIXE INTEIRO ARACANGUITO |
| 52101 | PEIXE INTEIRO ARACAPURI |
| 39114 | PEIXE INTEIRO ARACAROBA |
| 17106 | PEIXE INTEIRO ARACORANA |
| 07101 | PEIXE INTEIRO ARACU |
| 07103 | PEIXE INTEIRO ARACU BRANCO |
| 07102 | PEIXE INTEIRO ARACU PINIMA |
| 07105 | PEIXE INTEIRO ARACU PINTADO |
| 07104 | PEIXE INTEIRO ARACU TAQUARA |
| 07108 | PEIXE INTEIRO ARACUTINGA |
| 29105 | PEIXE INTEIRO ARAMACA |
| 29111 | PEIXE INTEIRO ARAMACACA |
| 29106 | PEIXE INTEIRO ARAMATA |
| 24106 | PEIXE INTEIRO ARANGAL |
| 27127 | PEIXE INTEIRO ARANJA |
| 30103 | PEIXE INTEIRO ARARA |
| 02127 | PEIXE INTEIRO ARARUA |
| 38126 | PEIXE INTEIRO ARATOBAIA |
| 38128 | PEIXE INTEIRO ARATUBAIA |
| 40105 | PEIXE INTEIRO ARAUANA |
| 11120 | PEIXE INTEIRO ARENGA BRANCA |
| 12102 | PEIXE INTEIRO ARIACO |
| 12105 | PEIXE INTEIRO ARICO |
| 12110 | PEIXE INTEIRO ARIECO |
| 12103 | PEIXE INTEIRO ARIOCO |
| 29103 | PEIXE INTEIRO ARMAU |
| 07102 | PEIXE INTEIRO ARRAIA |
| 07113 | PEIXE INTEIRO ARRAIA AMARELA |
| 07123 | PEIXE INTEIRO ARRAIA ARARA |
| 07117 | PEIXE INTEIRO ARRAIA BORBOLETA |
| 07121 | PEIXE INTEIRO ARRAIA CARIMBADA |
| 07106 | PEIXE INTEIRO ARRAIA CHITA |
| 07116 | PEIXE INTEIRO ARRAIA LIXA |
| 07120 | PEIXE INTEIRO ARRAIA MANTEIGA |
| 07126 | PEIXE INTEIRO ARRAIA PINTADA |
| 07109 | PEIXE INTEIRO ARRAIA PREGO |
| 07110 | PEIXE INTEIRO ARRAIA VIOLA |
| 18116 | PEIXE INTEIRO ARREBENTA PANELA |
| 40101 | PEIXE INTEIRO ARUANA |
| 53101 | PEIXE INTEIRO ARUIRI |
| 29108 | PEIXE INTEIRO ARUMACA |
| 29107 | PEIXE INTEIRO ARUMATA |
| 22101 | PEIXE INTEIRO ATUM |
| 56108 | PEIXE INTEIRO ATUM BRANCO (ALBACORA) |
| 10127 | PEIXE INTEIRO AZEITEIRA |
| 10128 | PEIXE INTEIRO AZEITONA |
| 29101 | PEIXE INTEIRO BACU |
| 29105 | PEIXE INTEIRO BACU DE PEDRA |
| 29106 | PEIXE INTEIRO BACU PEDRA |
| 47108 | PEIXE INTEIRO BACUDA |
| 02110 | PEIXE INTEIRO BADEJETE |
| 02101 | PEIXE INTEIRO BADEJO |
| 02107 | PEIXE INTEIRO BADEJO BICUDO |
| 02102 | PEIXE INTEIRO BADEJO BRANCO |
| 02103 | PEIXE INTEIRO BADEJO FERRO |
| 02105 | PEIXE INTEIRO BADEJO MIRA |
| 02104 | PEIXE INTEIRO BADEJO PRETO |
| 02108 | PEIXE INTEIRO BADEJO SABAO |
| 02106 | PEIXE INTEIRO BADEJO SALTAO |
| 14104 | PEIXE INTEIRO BAGRE AMARELO |
| 14108 | PEIXE INTEIRO BAGRE ARIACU |
| 14106 | PEIXE INTEIRO BAGRE BANDEIRA |
| 14102 | PEIXE INTEIRO BAGRE CANGUITO |
| 23115 | PEIXE INTEIRO BAGRE CHORA (ANUJA) |
| 46101 | PEIXE INTEIRO BAGRE DE AGUA DOCE |
| 14101 | PEIXE INTEIRO BAGRE DE MAR |
| 34102 | PEIXE INTEIRO BAGRE FIDALGO (FIDALGO) |
| 14109 | PEIXE INTEIRO BAGRE MANDIM DE MAR |
| 31106 | PEIXE INTEIRO BAGRE SAPO (PACAMAO DE AGUA DOCE) |
| 14107 | PEIXE INTEIRO BAGRE URUTU |
| 54101 | PEIXE INTEIRO BAIACU |
| 10114 | PEIXE INTEIRO BAMBA |
| 14112 | PEIXE INTEIRO BANDEIRADA |
| 14114 | PEIXE INTEIRO BANDIM |
| 56111 | PEIXE INTEIRO BANDOLIM |
| 02133 | PEIXE INTEIRO BARARUA |
| 13105 | PEIXE INTEIRO BARBA CHATA |
| 33113 | PEIXE INTEIRO BARBADO (DE AGUA DOCE) (PIRANAMBU) |
| 12128 | PEIXE INTEIRO BARBUDO |
| 47107 | PEIXE INTEIRO BARRACUDA |
| 35126 | PEIXE INTEIRO BARROSO |
| 08103 | PEIXE INTEIRO BATATA |
| 21109 | PEIXE INTEIRO BATUQUEIRO |
| 37105 | PEIXE INTEIRO BEIJOPIRA |
| 37101 | PEIXE INTEIRO BEIJUPIRA |
| 37102 | PEIXE INTEIRO BEIUPIRA |
| 37103 | PEIXE INTEIRO BEJUPIRA |
| 20107 | PEIXE INTEIRO BETARA |
| 04127 | PEIXE INTEIRO BICO DE PATO |
| 47101 | PEIXE INTEIRO BICUDA |
| 47104 | PEIXE INTEIRO BICUDA BRANCA |
| 47105 | PEIXE INTEIRO BICUDA CACHORRO |
| 47102 | PEIXE INTEIRO BICUDA DE CORSO |
| 26112 | PEIXE INTEIRO BICUDA DE RIO (CACHORRO) |
| 30122 | PEIXE INTEIRO BICUDO |
| 37104 | PEIXE INTEIRO BIJUPIRA |
| 18108 | PEIXE INTEIRO BIQUARA |
| 42108 | PEIXE INTEIRO BIRUBA |
| 14121 | PEIXE INTEIRO BOCA ABERTA |
| 04105 | PEIXE INTEIRO BOCA DE COLHER |
| 18110 | PEIXE INTEIRO BOCA DE VELHA |
| 23114 | PEIXE INTEIRO BOCA LISA (DE AGUA DOCE) (ANUJA) |
| 27135 | PEIXE INTEIRO BOCA MOLE |
| 12104 | PEIXE INTEIRO BOCA PRETA |
| 18109 | PEIXE INTEIRO BOCA VELHA |
| 26114 | PEIXE INTEIRO BOCARRA |
| 26117 | PEIXE INTEIRO BOCUDO |
| 23107 | PEIXE INTEIRO BODE |
| 06104 | PEIXE INTEIRO BODECO |
| 51101 | PEIXE INTEIRO BODIAO |
| 51103 | PEIXE INTEIRO BODIAO BATATA |
| 51108 | PEIXE INTEIRO BODIAO PAPAGAIO |
| 51110 | PEIXE INTEIRO BODIAO SABONETE |
| 51105 | PEIXE INTEIRO BODIAO TUCANO |
| 51107 | PEIXE INTEIRO BODIAO VERMELHO |
| 35136 | PEIXE INTEIRO BODINHO |
| 12107 | PEIXE INTEIRO BODO |
| 23121 | PEIXE INTEIRO BOGA |
| 12115 | PEIXE INTEIRO BOI DE GUARA (ACARI) |
| 49101 | PEIXE INTEIRO BONITO |
| 49106 | PEIXE INTEIRO BONITO BARRIGA RISCADA |
| 49108 | PEIXE INTEIRO BONITO CACHORRO |
| 49104 | PEIXE INTEIRO BONITO CADELA |
| 49105 | PEIXE INTEIRO BONITO CARIJO |
| 49107 | PEIXE INTEIRO BONITO GRANDE |
| 49103 | PEIXE INTEIRO BONITO PEQUENO |
| 49102 | PEIXE INTEIRO BONITO PINTADO |
| 07128 | PEIXE INTEIRO BORBOLETA |
| 27141 | PEIXE INTEIRO BORORO |
| 29108 | PEIXE INTEIRO BOTOADO |
| 19107 | PEIXE INTEIRO BRACO DE MOCA |
| 19102 | PEIXE INTEIRO BRACO DE MULATA |
| 14116 | PEIXE INTEIRO BRAGALHAO |
| 03115 | PEIXE INTEIRO BRANQUINHA |
| 03116 | PEIXE INTEIRO BRANQUINHO |
| 04124 | PEIXE INTEIRO BRUTELO |
| 51109 | PEIXE INTEIRO BUDIAO |
| 37116 | PEIXE INTEIRO BUJUPIRA |
| 11114 | PEIXE INTEIRO BURRIQUETA DE RIO (TUCUNARE) |
| 11115 | PEIXE INTEIRO BURRIQUETE DE RIO (TUCUNARE) |
| 11103 | PEIXE INTEIRO BURRIQUETI DE RIO (TUCUNARE) |
| 02130 | PEIXE INTEIRO BUVUARI |
| 38125 | PEIXE INTEIRO CABARARI |
| 05102 | PEIXE INTEIRO CABECA AMARGA |
| 23116 | PEIXE INTEIRO CABECA DE FERRO |
| 39109 | PEIXE INTEIRO CABECUDO |
| 35101 | PEIXE INTEIRO CACAO |
| 35112 | PEIXE INTEIRO CACAO ALECRIM |
| 35107 | PEIXE INTEIRO CACAO ANJO |
| 35125 | PEIXE INTEIRO CACAO BAGRE |
| 35111 | PEIXE INTEIRO CACAO CAMBEBA |
| 35117 | PEIXE INTEIRO CACAO CHAPEU |
| 35122 | PEIXE INTEIRO CACAO DE AREIA |
| 37108 | PEIXE INTEIRO CACAO DE ESCAMAS (BEIJUPIRA) |
| 35124 | PEIXE INTEIRO CACAO DE ESPINHO |
| 35104 | PEIXE INTEIRO CACAO FIUZO |
| 35113 | PEIXE INTEIRO CACAO FRANGO |
| 35109 | PEIXE INTEIRO CACAO GALHUDO |
| 35118 | PEIXE INTEIRO CACAO GATA |
| 35121 | PEIXE INTEIRO CACAO LIXA |
| 35128 | PEIXE INTEIRO CACAO MAGONGA |
| 35129 | PEIXE INTEIRO CACAO MANGONA |
| 35120 | PEIXE INTEIRO CACAO MANGONGA |
| 35115 | PEIXE INTEIRO CACAO MARTELO |
| 35114 | PEIXE INTEIRO CACAO PANA |
| 35110 | PEIXE INTEIRO CACAO PATA |
| 35102 | PEIXE INTEIRO CACAO PENA |
| 35119 | PEIXE INTEIRO CACAO PINTO |
| 35105 | PEIXE INTEIRO CACAO RAPOSA |
| 35116 | PEIXE INTEIRO CACAO RODELA |
| 35106 | PEIXE INTEIRO CACAO SEBASTIAO |
| 35108 | PEIXE INTEIRO CACAO TORRADOR |
| 07112 | PEIXE INTEIRO CACAO VIOLA |
| 10110 | PEIXE INTEIRO CACETAO |
| 12104 | PEIXE INTEIRO CACHIMBAU |
| 23105 | PEIXE INTEIRO CACHORRINHO |
| 23102 | PEIXE INTEIRO CACHORRINHO DE PADRE |
| 26101 | PEIXE INTEIRO CACHORRO |
| 23104 | PEIXE INTEIRO CACHORRO DE PADRE |
| 35140 | PEIXE INTEIRO CACONETE |
| 04125 | PEIXE INTEIRO CACONETE DE RIO (SURUBIM) |
| 07107 | PEIXE INTEIRO CAGUARA |
| 34105 | PEIXE INTEIRO CAICA |
| 71101 | PEIXE INTEIRO CAICANHA |
| 11117 | PEIXE INTEIRO CAICARA |
| 52109 | PEIXE INTEIRO CAICO |
| 30104 | PEIXE INTEIRO CAJARO |
| 14117 | PEIXE INTEIRO CALAFATE |
| 27145 | PEIXE INTEIRO CALAFETAO |
| 25102 | PEIXE INTEIRO CALUNGA |
| 42104 | PEIXE INTEIRO CAMARIPIM |
| 42102 | PEIXE INTEIRO CAMARUPIM |
| 10108 | PEIXE INTEIRO CAMBAO |
| 18119 | PEIXE INTEIRO CAMBIMBA |
| 10115 | PEIXE INTEIRO CAMBIRA |
| 17108 | PEIXE INTEIRO CAMBOATA |
| 17107 | PEIXE INTEIRO CAMBOTA |
| 30117 | PEIXE INTEIRO CAMBRIACU |
| 18117 | PEIXE INTEIRO CAMBUBA |
| 30116 | PEIXE INTEIRO CAMORIM |
| 30112 | PEIXE INTEIRO CAMURI |
| 30106 | PEIXE INTEIRO CAMURIM ACU |
| 30103 | PEIXE INTEIRO CAMURIM APUA |
| 30102 | PEIXE INTEIRO CAMURIM BRANCO |
| 30104 | PEIXE INTEIRO CAMURIM SOVELA |
| 30107 | PEIXE INTEIRO CAMURIMPEBA |
| 30113 | PEIXE INTEIRO CAMURIPEBA |
| 42108 | PEIXE INTEIRO CAMURIPEMA |
| 42106 | PEIXE INTEIRO CAMURIPIM |
| 42107 | PEIXE INTEIRO CAMURUPI |
| 42101 | PEIXE INTEIRO CAMURUPIM |
| 37106 | PEIXE INTEIRO CANADO |
| 31105 | PEIXE INTEIRO CANAPU |
| 31102 | PEIXE INTEIRO CANAPUACU |
| 31104 | PEIXE INTEIRO CANAPUM |
| 35123 | PEIXE INTEIRO CANGA |
| 14126 | PEIXE INTEIRO CANGATA |
| 14128 | PEIXE INTEIRO CANGATI |
| 23111 | PEIXE INTEIRO CANGATINHA |
| 18118 | PEIXE INTEIRO CANGUITO |
| 41101 | PEIXE INTEIRO CANGULO |
| 41113 | PEIXE INTEIRO CANGULO BRANCO |
| 41114 | PEIXE INTEIRO CANGULO DE FERNANDO |
| 41118 | PEIXE INTEIRO CANGULO DO ALTO |
| 41116 | PEIXE INTEIRO CANGULO PRETO |
| 41111 | PEIXE INTEIRO CANGULO REI |
| 41117 | PEIXE INTEIRO CANGURRO |
| 42109 | PEIXE INTEIRO CANGURUPI |
| 42110 | PEIXE INTEIRO CANGURUPIM |
| 03113 | PEIXE INTEIRO CANIVETE |
| 42105 | PEIXE INTEIRO CANJURUPI |
| 42111 | PEIXE INTEIRO CANJURUPIM |
| 23107 | PEIXE INTEIRO CANTANTE |
| 21117 | PEIXE INTEIRO CAPADINHO |
| 41109 | PEIXE INTEIRO CAPADO |
| 17111 | PEIXE INTEIRO CAPAO |
| 04122 | PEIXE INTEIRO CAPARARI |
| 04126 | PEIXE INTEIRO CAPARI |
| 18121 | PEIXE INTEIRO CAPIUNA |
| 02106 | PEIXE INTEIRO CARA |
| 02134 | PEIXE INTEIRO CARA ACU |
| 02104 | PEIXE INTEIRO CARA BERE |
| 02108 | PEIXE INTEIRO CARA BERERE |
| 02116 | PEIXE INTEIRO CARA BOBO |
| 02114 | PEIXE INTEIRO CARA CASCUDO |
| 02111 | PEIXE INTEIRO CARA DE TOPETE |
| 02107 | PEIXE INTEIRO CARA DIADEMA |
| 02122 | PEIXE INTEIRO CARA FERREIRO |
| 02119 | PEIXE INTEIRO CARA GIGANTE |
| 02117 | PEIXE INTEIRO CARA GUACU |
| 02115 | PEIXE INTEIRO CARA PATAQUIRA |
| 02113 | PEIXE INTEIRO CARA PRETO |
| 02102 | PEIXE INTEIRO CARA SEVERO |
| 15110 | PEIXE INTEIRO CARA SUJA |
| 02120 | PEIXE INTEIRO CARA TONTO |
| 02103 | PEIXE INTEIRO CARA TOPETE |
| 02118 | PEIXE INTEIRO CARA VACU |
| 02121 | PEIXE INTEIRO CARA ZEBU |
| 02131 | PEIXE INTEIRO CARAMBANGE |
| 20110 | PEIXE INTEIRO CARAMETARA |
| 40101 | PEIXE INTEIRO CARAMURU |
| 20113 | PEIXE INTEIRO CARAMUTARA |
| 21120 | PEIXE INTEIRO CARANHA |
| 40102 | PEIXE INTEIRO CARAPANA |
| 05103 | PEIXE INTEIRO CARAPAU |
| 15101 | PEIXE INTEIRO CARAPEBA |
| 15103 | PEIXE INTEIRO CARAPEBA BRANCA |
| 15102 | PEIXE INTEIRO CARAPEBA LISTRADA |
| 15104 | PEIXE INTEIRO CARAPEBA RAJADA |
| 15107 | PEIXE INTEIRO CARAPEVA |
| 44105 | PEIXE INTEIRO CARAPIA |
| 81101 | PEIXE INTEIRO CARAPICU |
| 15105 | PEIXE INTEIRO CARATINGA |
| 02128 | PEIXE INTEIRO CARAUNA |
| 71102 | PEIXE INTEIRO CARCANHA |
| 26104 | PEIXE INTEIRO CARDEIRO |
| 11114 | PEIXE INTEIRO CARDOSA |
| 12114 | PEIXE INTEIRO CARI |
| 39107 | PEIXE INTEIRO CARIMBAMBA |
| 12103 | PEIXE INTEIRO CARITO |
| 55101 | PEIXE INTEIRO CARPA |
| 03104 | PEIXE INTEIRO CASCA GROSSA |
| 12110 | PEIXE INTEIRO CASCUDINHO |
| 12126 | PEIXE INTEIRO CASCUDO |
| 12102 | PEIXE INTEIRO CASCUDO BARBADO |
| 12112 | PEIXE INTEIRO CASCUDO COMUM |
| 12113 | PEIXE INTEIRO CASCUDO ESPADA |
| 12109 | PEIXE INTEIRO CASCUDO ESPINHO |
| 12111 | PEIXE INTEIRO CASCUDO LIMA |
| 04101 | PEIXE INTEIRO CASTANHA |
| 33104 | PEIXE INTEIRO CATANA |
| 14127 | PEIXE INTEIRO CATINGUEIRA |
| 29110 | PEIXE INTEIRO CATRAIO |
| 03110 | PEIXE INTEIRO CATUNGA |
| 32102 | PEIXE INTEIRO CAVACO |
| 16101 | PEIXE INTEIRO CAVALA |
| 16103 | PEIXE INTEIRO CAVALA BRANCA |
| 16108 | PEIXE INTEIRO CAVALA DO REINO |
| 16104 | PEIXE INTEIRO CAVALA PERNA DE MOCA |
| 16102 | PEIXE INTEIRO CAVALA PINTADA |
| 16106 | PEIXE INTEIRO CAVALA PRETA |
| 16105 | PEIXE INTEIRO CAVALA SARDINHEIRA |
| 16107 | PEIXE INTEIRO CAVALA VERDADEIRA |
| 05101 | PEIXE INTEIRO CAVALINHA |
| 06110 | PEIXE INTEIRO CAVALINHA DE REIS (CHICHARRO) |
| 05102 | PEIXE INTEIRO CAVALINHA DO NORTE |
| 04129 | PEIXE INTEIRO CAXARA |
| 03106 | PEIXE INTEIRO CEVELHA |
| 01109 | PEIXE INTEIRO CHAMA CHUVA |
| 60101 | PEIXE INTEIRO CHAPEREMA |
| 11112 | PEIXE INTEIRO CHARUTO |
| 57107 | PEIXE INTEIRO CHERNA |
| 57101 | PEIXE INTEIRO CHERNE |
| 57104 | PEIXE INTEIRO CHERNE PINTADO |
| 57102 | PEIXE INTEIRO CHERNE PRETO |
| 57103 | PEIXE INTEIRO CHERNETE |
| 57105 | PEIXE INTEIRO CHERNOTE |
| 24104 | PEIXE INTEIRO CHERRO |
| 06101 | PEIXE INTEIRO CHICHARRO |
| 06105 | PEIXE INTEIRO CHICHARRO ALAMARIM |
| 06103 | PEIXE INTEIRO CHICHARRO BRANCO |
| 06107 | PEIXE INTEIRO CHICHARRO CALABAR |
| 06104 | PEIXE INTEIRO CHICHARRO CARAPAU |
| 06108 | PEIXE INTEIRO CHICHARRO CAVALA |
| 06106 | PEIXE INTEIRO CHICHARRO OLHO GRANDE |
| 06102 | PEIXE INTEIRO CHICHARRO PINTADO |
| 13105 | PEIXE INTEIRO CHIOBA |
| 13102 | PEIXE INTEIRO CHIOVA |
| 10104 | PEIXE INTEIRO CHIRA |
| 03110 | PEIXE INTEIRO CHORA CHORA |
| 23113 | PEIXE INTEIRO CHORADEIRA |
| 71105 | PEIXE INTEIRO CHOUPA |
| 32106 | PEIXE INTEIRO CHUMBERGA |
| 20116 | PEIXE INTEIRO CHUPA AREIA |
| 20123 | PEIXE INTEIRO CHUPITA |
| 26102 | PEIXE INTEIRO CIGARRA |
| 13101 | PEIXE INTEIRO CIOBA |
| 27138 | PEIXE INTEIRO COACA |
| 15102 | PEIXE INTEIRO COBRA |
| 18120 | PEIXE INTEIRO COCORO |
| 18102 | PEIXE INTEIRO COCOROCA |
| 20120 | PEIXE INTEIRO COICOA |
| 50103 | PEIXE INTEIRO COITADINHO |
| 30118 | PEIXE INTEIRO CONGOROPEBA |
| 72102 | PEIXE INTEIRO CONGRO REAL |
| 01106 | PEIXE INTEIRO CONGUACU |
| 18101 | PEIXE INTEIRO CORCOROCA |
| 18105 | PEIXE INTEIRO CORCOROCA BOCA DE FOGO |
| 18103 | PEIXE INTEIRO CORCOROCA BOCA LARGA |
| 18107 | PEIXE INTEIRO CORCOROCA JURUMIRIM |
| 18106 | PEIXE INTEIRO CORCOROCA MULATA |
| 30114 | PEIXE INTEIRO CORCUNDA |
| 30115 | PEIXE INTEIRO CORCUNDO |
| 10106 | PEIXE INTEIRO CORIMBA |
| 39111 | PEIXE INTEIRO CORIMBAMBA |
| 35103 | PEIXE INTEIRO CORNUDA |
| 26103 | PEIXE INTEIRO CORO |
| 26107 | PEIXE INTEIRO CORO AMARELO |
| 71108 | PEIXE INTEIRO CORO BRANCO |
| 18104 | PEIXE INTEIRO COROCOROCA |
| 47106 | PEIXE INTEIRO COROMA |
| 21119 | PEIXE INTEIRO CORONA |
| 26105 | PEIXE INTEIRO COROQUE |
| 02124 | PEIXE INTEIRO CORRO |
| 26102 | PEIXE INTEIRO CORRO DE MAR |
| 10111 | PEIXE INTEIRO CORUMBATA |
| 03101 | PEIXE INTEIRO CORVINA |
| 03105 | PEIXE INTEIRO CORVINA BACALHAU |
| 03104 | PEIXE INTEIRO CORVINA BRASILEIRA |
| 20111 | PEIXE INTEIRO CORVINA CACHORRO (PAPATERRA) |
| 03106 | PEIXE INTEIRO CORVINA DE LINHA |
| 03103 | PEIXE INTEIRO CORVINA DE LISTA |
| 03102 | PEIXE INTEIRO CORVINA RISCADA |
| 03112 | PEIXE INTEIRO CORVINOTA |
| 40102 | PEIXE INTEIRO CRAMURU |
| 10112 | PEIXE INTEIRO CRUMATA |
| 14119 | PEIXE INTEIRO CUBANGO |
| 26115 | PEIXE INTEIRO CUBIO |
| 26116 | PEIXE INTEIRO CUBIU |
| 35135 | PEIXE INTEIRO CUCURI |
| 03107 | PEIXE INTEIRO CUCURUCA |
| 29109 | PEIXE INTEIRO CUIU CUIU |
| 56101 | PEIXE INTEIRO CUJUBA |
| 23110 | PEIXE INTEIRO CUMBACA (DE AGUA DOCE) (ANUJA) |
| 42120 | PEIXE INTEIRO CUMURUPIM |
| 10108 | PEIXE INTEIRO CURIBATA |
| 10118 | PEIXE INTEIRO CURIMA |
| 10101 | PEIXE INTEIRO CURIMATA |
| 10103 | PEIXE INTEIRO CURIMATA (RIO) |
| 10102 | PEIXE INTEIRO CURIMATAU |
| 10109 | PEIXE INTEIRO CURIMBA |
| 10110 | PEIXE INTEIRO CURIMBATA |
| 10113 | PEIXE INTEIRO CURIMBOTA |
| 03108 | PEIXE INTEIRO CURUCA |
| 10121 | PEIXE INTEIRO CURUMA |
| 10105 | PEIXE INTEIRO CURUMBATA |
| 22102 | PEIXE INTEIRO CURUPETE |
| 03109 | PEIXE INTEIRO CURURUCA |
| 03111 | PEIXE INTEIRO CURURUQUIRA |
| 21102 | PEIXE INTEIRO DALFINHO |
| 29104 | PEIXE INTEIRO DAQUEIRO |
| 00101 | PEIXE INTEIRO DE AGUA DOCE NAO-ESPECIFICADO |
| 00101 | PEIXE INTEIRO DE MAR NAO-ESPECIFICADO |
| 58107 | PEIXE INTEIRO DE VELA |
| 03113 | PEIXE INTEIRO DENTUDA |
| 26113 | PEIXE INTEIRO DENTUDO PINTADO |
| 32104 | PEIXE INTEIRO DOIRADA |
| 32103 | PEIXE INTEIRO DOIRADA DO AMAZONAS |
| 01109 | PEIXE INTEIRO DORME DORME |
| 32101 | PEIXE INTEIRO DOURADA |
| 32102 | PEIXE INTEIRO DOURADA DO AMAZONAS |
| 16101 | PEIXE INTEIRO DOURADO DE AGUA DOCE |
| 21101 | PEIXE INTEIRO DOURADO DE MAR |
| 17103 | PEIXE INTEIRO DOUTOR |
| 20115 | PEIXE INTEIRO EMBETARA |
| 33103 | PEIXE INTEIRO EMBIRA |
| 01102 | PEIXE INTEIRO ENCHOVA |
| 01106 | PEIXE INTEIRO ENCHOVA BAETA |
| 01107 | PEIXE INTEIRO ENCHOVETA |
| 01104 | PEIXE INTEIRO ENCHOVINHA |
| 40105 | PEIXE INTEIRO ENGUIA (CARAMURU) |
| 15106 | PEIXE INTEIRO ENGUIA DE AGUA DOCE (MUCUM) |
| 30120 | PEIXE INTEIRO ESCALHO |
| 41103 | PEIXE INTEIRO ESFALDADO |
| 33101 | PEIXE INTEIRO ESPADA |
| 58110 | PEIXE INTEIRO ESPADARTE |
| 58109 | PEIXE INTEIRO ESPADARTE MECA |
| 02129 | PEIXE INTEIRO ESPANHARINA |
| 41108 | PEIXE INTEIRO FANTASMA |
| 17109 | PEIXE INTEIRO FAQUECO |
| 44107 | PEIXE INTEIRO FARNANGAIO |
| 44104 | PEIXE INTEIRO FARNANGALHO |
| 18122 | PEIXE INTEIRO FAROFA |
| 52108 | PEIXE INTEIRO FAVINHA |
| 52107 | PEIXE INTEIRO FAVOLETA |
| 26106 | PEIXE INTEIRO FERREIRO |
| 34101 | PEIXE INTEIRO FIDALGO |
| 28102 | PEIXE INTEIRO FILHOTE |
| 32103 | PEIXE INTEIRO FLAMINGUETE |
| 57102 | PEIXE INTEIRO FLECHEIRO |
| 52106 | PEIXE INTEIRO FOLHA DE MANGUE |
| 32107 | PEIXE INTEIRO FRAMIGUETA |
| 32109 | PEIXE INTEIRO FRAMIGUETO |
| 35137 | PEIXE INTEIRO FRANGO |
| 30119 | PEIXE INTEIRO FURAO |
| 27148 | PEIXE INTEIRO GALHETEIRA |
| 33102 | PEIXE INTEIRO GALINHA |
| 17104 | PEIXE INTEIRO GALINHO |
| 17101 | PEIXE INTEIRO GALO |
| 17102 | PEIXE INTEIRO GALO BANDEIRA |
| 17105 | PEIXE INTEIRO GALO BRANCO |
| 17108 | PEIXE INTEIRO GALO DE PENACHO |
| 17107 | PEIXE INTEIRO GALO PENA |
| 38124 | PEIXE INTEIRO GARABEBEL |
| 43104 | PEIXE INTEIRO GARAJUBA |
| 28101 | PEIXE INTEIRO GAROUPA |
| 28110 | PEIXE INTEIRO GAROUPA BICHADA |
| 28109 | PEIXE INTEIRO GAROUPA CHITA |
| 28104 | PEIXE INTEIRO GAROUPA CRIOULA |
| 28108 | PEIXE INTEIRO GAROUPA DE SEGUNDA |
| 28106 | PEIXE INTEIRO GAROUPA GATO |
| 28105 | PEIXE INTEIRO GAROUPA PRETA |
| 28103 | PEIXE INTEIRO GAROUPA SAO TOME |
| 28107 | PEIXE INTEIRO GAROUPA SENHOR DE ENGENHO |
| 28102 | PEIXE INTEIRO GAROUPA VERDADEIRA |
| 28111 | PEIXE INTEIRO GAROUPINHA |
| 35130 | PEIXE INTEIRO GATA (CACAO) |
| 10116 | PEIXE INTEIRO GERICOIA |
| 19107 | PEIXE INTEIRO GINGA |
| 27149 | PEIXE INTEIRO GO |
| 51106 | PEIXE INTEIRO GODIAO |
| 27139 | PEIXE INTEIRO GOETE |
| 46106 | PEIXE INTEIRO GOIVIRA |
| 71109 | PEIXE INTEIRO GOLOSA |
| 27143 | PEIXE INTEIRO GORETE |
| 53105 | PEIXE INTEIRO GORRETAO |
| 39108 | PEIXE INTEIRO GRACAIM |
| 10118 | PEIXE INTEIRO GRAMATAO |
| 21103 | PEIXE INTEIRO GRASSAPE |
| 10117 | PEIXE INTEIRO GRUMATA |
| 10115 | PEIXE INTEIRO GRUMEXA |
| 39112 | PEIXE INTEIRO GUACAINHA |
| 12108 | PEIXE INTEIRO GUACARI |
| 46102 | PEIXE INTEIRO GUAIBIRA |
| 13104 | PEIXE INTEIRO GUAIUBA |
| 46101 | PEIXE INTEIRO GUAIVIRA |
| 13106 | PEIXE INTEIRO GUAJUBA |
| 46105 | PEIXE INTEIRO GUAJUVIRA |
| 21105 | PEIXE INTEIRO GUARACAPEMA |
| 39113 | PEIXE INTEIRO GUARACEMA |
| 43103 | PEIXE INTEIRO GUARAIUBA |
| 43101 | PEIXE INTEIRO GUARAJUBA |
| 14105 | PEIXE INTEIRO GUARAJUBA DE MAR |
| 43102 | PEIXE INTEIRO GUARAMBA |
| 46107 | PEIXE INTEIRO GUARAVIRA |
| 32111 | PEIXE INTEIRO GUARECIMA |
| 32112 | PEIXE INTEIRO GUARICEMA |
| 13107 | PEIXE INTEIRO GUATUBA |
| 51104 | PEIXE INTEIRO GUDIAO |
| 41107 | PEIXE INTEIRO GUDUNHO |
| 58108 | PEIXE INTEIRO GUEBO |
| 58104 | PEIXE INTEIRO GUEBUCU |
| 05113 | PEIXE INTEIRO GUENZA |
| 05121 | PEIXE INTEIRO GUENZA VERDE |
| 27142 | PEIXE INTEIRO GUETE |
| 07103 | PEIXE INTEIRO GUITARRA |
| 71110 | PEIXE INTEIRO GULOSA |
| 43105 | PEIXE INTEIRO GURIJUBA |
| 26103 | PEIXE INTEIRO ICANGA |
| 20112 | PEIXE INTEIRO IMBETARA |
| 33102 | PEIXE INTEIRO IMBIRA |
| 33103 | PEIXE INTEIRO INAMBU |
| 33104 | PEIXE INTEIRO INHAMBU |
| 14120 | PEIXE INTEIRO IREDECA |
| 14118 | PEIXE INTEIRO IRICECA |
| 14123 | PEIXE INTEIRO IRIDECA |
| 14103 | PEIXE INTEIRO IRIDECA DE MAR |
| 14111 | PEIXE INTEIRO IRITINGA |
| 03105 | PEIXE INTEIRO ISCA BRANCA |
| 48101 | PEIXE INTEIRO ITUI |
| 07130 | PEIXE INTEIRO JABIBIRETE |
| 07127 | PEIXE INTEIRO JABIRETE |
| 05101 | PEIXE INTEIRO JACUNDA |
| 05110 | PEIXE INTEIRO JACUNDA ACU |
| 05108 | PEIXE INTEIRO JACUNDA BRANCO |
| 05104 | PEIXE INTEIRO JACUNDA CABECUDO |
| 05115 | PEIXE INTEIRO JACUNDA COROA |
| 42119 | PEIXE INTEIRO JACUNDA DE RIO (CAMURUPIM) |
| 05111 | PEIXE INTEIRO JACUNDA PINIMA |
| 05109 | PEIXE INTEIRO JACUNDA PIRANGA |
| 05116 | PEIXE INTEIRO JACUNDA TOTO |
| 05112 | PEIXE INTEIRO JACUNDA TOURO |
| 05106 | PEIXE INTEIRO JACUNDA VERDE |
| 14122 | PEIXE INTEIRO JAGUAR |
| 23123 | PEIXE INTEIRO JANDIA |
| 25101 | PEIXE INTEIRO JARAQUI |
| 38102 | PEIXE INTEIRO JARIPOCA |
| 51104 | PEIXE INTEIRO JATUARANA |
| 35102 | PEIXE INTEIRO JATUBARANA |
| 38101 | PEIXE INTEIRO JAU |
| 26118 | PEIXE INTEIRO JAUARANA |
| 38104 | PEIXE INTEIRO JAUPOCA |
| 09101 | PEIXE INTEIRO JEJU |
| 25102 | PEIXE INTEIRO JERAQUI |
| 25103 | PEIXE INTEIRO JERIQUI |
| 04107 | PEIXE INTEIRO JERUPOCA |
| 09103 | PEIXE INTEIRO JIJU |
| 04106 | PEIXE INTEIRO JIRIPOCA |
| 38122 | PEIXE INTEIRO JIRIQUITI |
| 05105 | PEIXE INTEIRO JOANA GUENZA |
| 05114 | PEIXE INTEIRO JOANINHA |
| 05122 | PEIXE INTEIRO JOANINHA GUENZA |
| 35138 | PEIXE INTEIRO JOAO DIAS |
| 20105 | PEIXE INTEIRO JUDEU |
| 14125 | PEIXE INTEIRO JUJUBA |
| 23120 | PEIXE INTEIRO JUNDIA |
| 38103 | PEIXE INTEIRO JUNDIA DE LAGOA (JAU) |
| 23104 | PEIXE INTEIRO JURICO |
| 04113 | PEIXE INTEIRO JURUPENSEM |
| 14124 | PEIXE INTEIRO JURUPIRANGA |
| 04103 | PEIXE INTEIRO JURUPOCA |
| 35103 | PEIXE INTEIRO JUTUBARANA |
| 52105 | PEIXE INTEIRO JUVA |
| 11108 | PEIXE INTEIRO LACANARI |
| 30107 | PEIXE INTEIRO LAITU |
| 03101 | PEIXE INTEIRO LAMBARI |
| 42111 | PEIXE INTEIRO LAMBARI BOCARRA (SAIRU) |
| 05104 | PEIXE INTEIRO LANCETA |
| 42117 | PEIXE INTEIRO LARGA ESCAMA |
| 19108 | PEIXE INTEIRO LAULAU |
| 18102 | PEIXE INTEIRO LENHA |
| 50104 | PEIXE INTEIRO LEVANTA SAIA |
| 29112 | PEIXE INTEIRO LINGUA |
| 29101 | PEIXE INTEIRO LINGUADO |
| 29102 | PEIXE INTEIRO LINGUADO DA AREIA |
| 23117 | PEIXE INTEIRO LIRIO |
| 04115 | PEIXE INTEIRO LOANGO |
| 05117 | PEIXE INTEIRO LOPE |
| 11106 | PEIXE INTEIRO LUCANARI |
| 18124 | PEIXE INTEIRO MACACA |
| 11113 | PEIXE INTEIRO MACACA DE MAR |
| 21104 | PEIXE INTEIRO MACACO |
| 11108 | PEIXE INTEIRO MACACO DE MAR |
| 11121 | PEIXE INTEIRO MACAMBE |
| 21115 | PEIXE INTEIRO MAFURA |
| 27114 | PEIXE INTEIRO MALHEIRA |
| 19110 | PEIXE INTEIRO MAMPARA |
| 51103 | PEIXE INTEIRO MAMURI |
| 23119 | PEIXE INTEIRO MANDI |
| 19106 | PEIXE INTEIRO MANDI PERUANO (MAPARA) |
| 36106 | PEIXE INTEIRO MANDIBE |
| 23109 | PEIXE INTEIRO MANDICUMBA |
| 23122 | PEIXE INTEIRO MANDIUVA |
| 23108 | PEIXE INTEIRO MANDIZINHO (ANUJA) |
| 36101 | PEIXE INTEIRO MANDUBE |
| 36105 | PEIXE INTEIRO MANDUBE MIRIM |
| 36107 | PEIXE INTEIRO MANDUBI |
| 36103 | PEIXE INTEIRO MANDURI |
| 36104 | PEIXE INTEIRO MANDUVA |
| 11116 | PEIXE INTEIRO MANE |
| 35142 | PEIXE INTEIRO MANGONA |
| 31110 | PEIXE INTEIRO MANGURIU |
| 31109 | PEIXE INTEIRO MANGURUIU |
| 19101 | PEIXE INTEIRO MANJUBA |
| 19105 | PEIXE INTEIRO MANJUVA |
| 33111 | PEIXE INTEIRO MANTOPAQUE |
| 19101 | PEIXE INTEIRO MAPARA |
| 19105 | PEIXE INTEIRO MAPARA DE CAMETA |
| 19109 | PEIXE INTEIRO MAPURA |
| 05107 | PEIXE INTEIRO MARIA GUENZA |
| 27131 | PEIXE INTEIRO MARIA MOLE |
| 42114 | PEIXE INTEIRO MARIQUITA |
| 58105 | PEIXE INTEIRO MARLIM |
| 11115 | PEIXE INTEIRO MAROMBA |
| 35132 | PEIXE INTEIRO MARRAXO |
| 35127 | PEIXE INTEIRO MARTELO |
| 11122 | PEIXE INTEIRO MASSAMBE |
| 51101 | PEIXE INTEIRO MATRINXA |
| 51102 | PEIXE INTEIRO MATRINXAO |
| 03112 | PEIXE INTEIRO MATUPIRI |
| 01111 | PEIXE INTEIRO MATURAQUE |
| 36102 | PEIXE INTEIRO MELOSO |
| 27147 | PEIXE INTEIRO MEMBECA |
| 02123 | PEIXE INTEIRO MERERE |
| 36101 | PEIXE INTEIRO MERLUZA |
| 31101 | PEIXE INTEIRO MERO |
| 31103 | PEIXE INTEIRO MERO CANAPU |
| 31106 | PEIXE INTEIRO MERO CANAPUM |
| 57108 | PEIXE INTEIRO MERO PRETO (CHERNE) |
| 57106 | PEIXE INTEIRO MERO QUEIMADO (CHERNE) |
| 05119 | PEIXE INTEIRO MICHOLA |
| 47109 | PEIXE INTEIRO MILONGA |
| 47103 | PEIXE INTEIRO MILONGO (BICUDA) DE MAR |
| 02109 | PEIXE INTEIRO MIRA |
| 53101 | PEIXE INTEIRO MIRAGAIA |
| 53103 | PEIXE INTEIRO MIRAGUAIA |
| 11118 | PEIXE INTEIRO MIRIM |
| 40103 | PEIXE INTEIRO MIRORO |
| 05103 | PEIXE INTEIRO MIXORNE |
| 03108 | PEIXE INTEIRO MOCINHA |
| 03107 | PEIXE INTEIRO MOCINHO |
| 33112 | PEIXE INTEIRO MOELA |
| 34107 | PEIXE INTEIRO MONDEGO |
| 10107 | PEIXE INTEIRO MORE (CURIMATA) |
| 55101 | PEIXE INTEIRO MOREIA |
| 55103 | PEIXE INTEIRO MOREIA AMARELA |
| 55102 | PEIXE INTEIRO MOREIA PINTADA |
| 40104 | PEIXE INTEIRO MOREIA VERDE (CARAMURU) |
| 02125 | PEIXE INTEIRO MORERE |
| 40106 | PEIXE INTEIRO MORORO |
| 15103 | PEIXE INTEIRO MUCU |
| 15101 | PEIXE INTEIRO MUCUM |
| 15105 | PEIXE INTEIRO MUCUM DE ESCAMA |
| 15104 | PEIXE INTEIRO MUCUM DE PELE |
| 15111 | PEIXE INTEIRO MULATINHA |
| 15109 | PEIXE INTEIRO MULATINHO |
| 23112 | PEIXE INTEIRO MULATO |
| 13102 | PEIXE INTEIRO MULHER INGRATA |
| 11119 | PEIXE INTEIRO MUSSOLINA |
| 55105 | PEIXE INTEIRO MUTUCA |
| 55104 | PEIXE INTEIRO MUTUTUCA |
| 33110 | PEIXE INTEIRO NAMBU |
| 08101 | PEIXE INTEIRO NAMORADO |
| 00101 | PEIXE INTEIRO NAO-ESPECIFICADO |
| 07129 | PEIXE INTEIRO NARINARI |
| 18112 | PEIXE INTEIRO NEGRA MINA |
| 10129 | PEIXE INTEIRO NEGRAO |
| 45102 | PEIXE INTEIRO OBARANA |
| 45108 | PEIXE INTEIRO OBARANACU |
| 19103 | PEIXE INTEIRO OLELEIRO |
| 23103 | PEIXE INTEIRO OLHETE |
| 23101 | PEIXE INTEIRO OLHO DE BOI |
| 31107 | PEIXE INTEIRO PACAMA DE AGUA DOCE |
| 31101 | PEIXE INTEIRO PACAMAO DE AGUA DOCE |
| 21101 | PEIXE INTEIRO PACU |
| 21108 | PEIXE INTEIRO PACU AZUL |
| 21106 | PEIXE INTEIRO PACU BRANCO |
| 21105 | PEIXE INTEIRO PACU CHATO |
| 21103 | PEIXE INTEIRO PACU CHICO |
| 22103 | PEIXE INTEIRO PACU DO AMAZONAS (TAMBAQUI) |
| 21104 | PEIXE INTEIRO PACU MIRIM |
| 21102 | PEIXE INTEIRO PACUACU |
| 21107 | PEIXE INTEIRO PACUGUACU |
| 31103 | PEIXE INTEIRO PACUMA DE AGUA DOCE |
| 21114 | PEIXE INTEIRO PACUPEBA |
| 21113 | PEIXE INTEIRO PACUPEVA |
| 21111 | PEIXE INTEIRO PACUTINGA |
| 25103 | PEIXE INTEIRO PAGRO |
| 14105 | PEIXE INTEIRO PAIUDO |
| 52101 | PEIXE INTEIRO PALOMBETA |
| 52102 | PEIXE INTEIRO PALOMETA |
| 38123 | PEIXE INTEIRO PAMPANO |
| 46108 | PEIXE INTEIRO PAMPARRONA |
| 38121 | PEIXE INTEIRO PAMPLO |
| 38101 | PEIXE INTEIRO PAMPO |
| 38111 | PEIXE INTEIRO PAMPO ARABEBEL |
| 38114 | PEIXE INTEIRO PAMPO ARATUBAIA |
| 38102 | PEIXE INTEIRO PAMPO CABECA MOLE |
| 38107 | PEIXE INTEIRO PAMPO DO ALTO |
| 38113 | PEIXE INTEIRO PAMPO ESPINHA MOLE |
| 38112 | PEIXE INTEIRO PAMPO GALHUDO |
| 38108 | PEIXE INTEIRO PAMPO GARABEBEL |
| 38110 | PEIXE INTEIRO PAMPO GIGANTE |
| 38104 | PEIXE INTEIRO PAMPO LISTRADO |
| 38105 | PEIXE INTEIRO PAMPO MACHO |
| 38115 | PEIXE INTEIRO PAMPO RISCADO |
| 38109 | PEIXE INTEIRO PAMPO SARGENTO |
| 38106 | PEIXE INTEIRO PAMPO SOLTEIRO |
| 38103 | PEIXE INTEIRO PAMPO VERMELHO |
| 21121 | PEIXE INTEIRO PANARE |
| 51102 | PEIXE INTEIRO PAPAGAIO |
| 20101 | PEIXE INTEIRO PAPATERRA |
| 37112 | PEIXE INTEIRO PARABEIJU |
| 37115 | PEIXE INTEIRO PARAMBEJU |
| 37114 | PEIXE INTEIRO PARANDIJU |
| 42112 | PEIXE INTEIRO PARAPEMA |
| 34101 | PEIXE INTEIRO PARATI |
| 34102 | PEIXE INTEIRO PARATI OLHO DE FOGO |
| 34106 | PEIXE INTEIRO PARATICU |
| 25101 | PEIXE INTEIRO PARGO |
| 26108 | PEIXE INTEIRO PARGO BRANCO (RONCADOR) |
| 25106 | PEIXE INTEIRO PARGO CACHUCHO |
| 25104 | PEIXE INTEIRO PARGO OLHO DE VIDRO |
| 25105 | PEIXE INTEIRO PARGO ROSA |
| 38116 | PEIXE INTEIRO PAROLA |
| 76101 | PEIXE INTEIRO PARU |
| 35131 | PEIXE INTEIRO PATA |
| 21116 | PEIXE INTEIRO PEBA |
| 71107 | PEIXE INTEIRO PEDRA |
| 14110 | PEIXE INTEIRO PELADO |
| 42113 | PEIXE INTEIRO PEMA |
| 42115 | PEIXE INTEIRO PERAPEMA |
| 27144 | PEIXE INTEIRO PERNA DE MOCA |
| 41105 | PEIXE INTEIRO PEROA |
| 41102 | PEIXE INTEIRO PEROA BRANCO |
| 41104 | PEIXE INTEIRO PEROA PRETO |
| 41106 | PEIXE INTEIRO PEROATINGA |
| 53106 | PEIXE INTEIRO PEROMBEBE |
| 53104 | PEIXE INTEIRO PERUMBEBA |
| 27101 | PEIXE INTEIRO PESCADA |
| 27102 | PEIXE INTEIRO PESCADA AMARELA |
| 27104 | PEIXE INTEIRO PESCADA ARATANHA |
| 27115 | PEIXE INTEIRO PESCADA ARAUJA |
| 27130 | PEIXE INTEIRO PESCADA BANANA |
| 27105 | PEIXE INTEIRO PESCADA BRANCA |
| 27125 | PEIXE INTEIRO PESCADA CABECA DURA |
| 20106 | PEIXE INTEIRO PESCADA CACHORRO (PAPATERRA) |
| 27107 | PEIXE INTEIRO PESCADA CAMBUCI |
| 27110 | PEIXE INTEIRO PESCADA CAMBUCU |
| 27128 | PEIXE INTEIRO PESCADA COBRA |
| 27126 | PEIXE INTEIRO PESCADA CORVINA |
| 27122 | PEIXE INTEIRO PESCADA CUTUPANHA |
| 27134 | PEIXE INTEIRO PESCADA DE ALTO |
| 27137 | PEIXE INTEIRO PESCADA DE ALTO MAR |
| 27121 | PEIXE INTEIRO PESCADA DE DENTE |
| 27133 | PEIXE INTEIRO PESCADA DENTUCA |
| 27118 | PEIXE INTEIRO PESCADA DO REINO |
| 27112 | PEIXE INTEIRO PESCADA ESCAMOSA |
| 27132 | PEIXE INTEIRO PESCADA FOGUETE |
| 27117 | PEIXE INTEIRO PESCADA GALHETEIRA |
| 27119 | PEIXE INTEIRO PESCADA GO |
| 27108 | PEIXE INTEIRO PESCADA GOETE |
| 27111 | PEIXE INTEIRO PESCADA JAGUARA |
| 27120 | PEIXE INTEIRO PESCADA LEGITIMA |
| 27116 | PEIXE INTEIRO PESCADA MARIA MOLE |
| 27109 | PEIXE INTEIRO PESCADA OLHUDA |
| 27106 | PEIXE INTEIRO PESCADA PERNA DE MOCA |
| 36103 | PEIXE INTEIRO PESCADA PORTUGUESA (MERLUZA) |
| 27124 | PEIXE INTEIRO PESCADA PRETA |
| 27113 | PEIXE INTEIRO PESCADA REAL |
| 27129 | PEIXE INTEIRO PESCADA SELVAGEM |
| 27123 | PEIXE INTEIRO PESCADA TRAMITARA |
| 09101 | PEIXE INTEIRO PESCADINHA |
| 09104 | PEIXE INTEIRO PESCADINHA DO ALTO |
| 09106 | PEIXE INTEIRO PESCADINHA DO ALTO MAR |
| 09103 | PEIXE INTEIRO PESCADINHA MALHEIRA |
| 09105 | PEIXE INTEIRO PESCADINHA MANTEIGA |
| 09102 | PEIXE INTEIRO PESCADINHA TORTINHA |
| 21112 | PEIXE INTEIRO PEVA |
| 03123 | PEIXE INTEIRO PIABA |
| 03103 | PEIXE INTEIRO PIABA BRANCA |
| 03124 | PEIXE INTEIRO PIABA CRIOULA |
| 03120 | PEIXE INTEIRO PIABA DA LAGOA |
| 03102 | PEIXE INTEIRO PIABA PIXUNA |
| 27101 | PEIXE INTEIRO PIABANHA |
| 03118 | PEIXE INTEIRO PIABINHA |
| 03117 | PEIXE INTEIRO PIABUCU |
| 31108 | PEIXE INTEIRO PIACURURU |
| 39102 | PEIXE INTEIRO PIAMPARA |
| 39101 | PEIXE INTEIRO PIAPARA |
| 14101 | PEIXE INTEIRO PIAU |
| 14104 | PEIXE INTEIRO PIAU GALO |
| 14103 | PEIXE INTEIRO PIAU LAVRADO |
| 14102 | PEIXE INTEIRO PIAU VERDADEIRO |
| 03122 | PEIXE INTEIRO PIAVA |
| 03121 | PEIXE INTEIRO PIAVUCU |
| 02132 | PEIXE INTEIRO PILAPE |
| 12105 | PEIXE INTEIRO PILATOS |
| 52103 | PEIXE INTEIRO PILOMBETA |
| 33107 | PEIXE INTEIRO PINTADINHO (DE AGUA DOCE) (PIRANAMBU) |
| 04117 | PEIXE INTEIRO PINTADO |
| 35133 | PEIXE INTEIRO PINTO |
| 19103 | PEIXE INTEIRO PIPITINGA |
| 03114 | PEIXE INTEIRO PIQUIRA |
| 26109 | PEIXE INTEIRO PIRA ANDIRA |
| 20114 | PEIXE INTEIRO PIRA SIRIRICA |
| 04123 | PEIXE INTEIRO PIRAACA |
| 37110 | PEIXE INTEIRO PIRABEIJU |
| 37109 | PEIXE INTEIRO PIRABIJU |
| 41101 | PEIXE INTEIRO PIRACANJUBA |
| 41102 | PEIXE INTEIRO PIRACANJUVA |
| 41103 | PEIXE INTEIRO PIRACANJUVIRA |
| 58101 | PEIXE INTEIRO PIRACATINGA |
| 06102 | PEIXE INTEIRO PIRACU |
| 28116 | PEIXE INTEIRO PIRACUCA |
| 31104 | PEIXE INTEIRO PIRACURURU |
| 28101 | PEIXE INTEIRO PIRAIBA |
| 16107 | PEIXE INTEIRO PIRAIU |
| 28115 | PEIXE INTEIRO PIRAJIA |
| 16102 | PEIXE INTEIRO PIRAJU |
| 16103 | PEIXE INTEIRO PIRAJUBA |
| 18104 | PEIXE INTEIRO PIRAJUPEVA |
| 33114 | PEIXE INTEIRO PIRAMAPU |
| 20115 | PEIXE INTEIRO PIRAMBE |
| 20118 | PEIXE INTEIRO PIRAMBEBA |
| 27146 | PEIXE INTEIRO PIRAMBEBE |
| 20117 | PEIXE INTEIRO PIRAMBEVA |
| 37111 | PEIXE INTEIRO PIRAMBIJU |
| 30106 | PEIXE INTEIRO PIRAMBREPE |
| 33105 | PEIXE INTEIRO PIRAMBU (DE AGUA DOCE) (PIRANAMBU) |
| 04104 | PEIXE INTEIRO PIRAMBUCU |
| 59105 | PEIXE INTEIRO PIRAMETARA |
| 20121 | PEIXE INTEIRO PIRAMPE |
| 20116 | PEIXE INTEIRO PIRAMPEBA |
| 20114 | PEIXE INTEIRO PIRAMPEVA |
| 13103 | PEIXE INTEIRO PIRAMUTA |
| 13101 | PEIXE INTEIRO PIRAMUTABA |
| 13104 | PEIXE INTEIRO PIRAMUTAVA |
| 33101 | PEIXE INTEIRO PIRANAMBU |
| 33109 | PEIXE INTEIRO PIRANAMPU |
| 20101 | PEIXE INTEIRO PIRANHA |
| 20106 | PEIXE INTEIRO PIRANHA AMARELA |
| 20107 | PEIXE INTEIRO PIRANHA BEBA |
| 20110 | PEIXE INTEIRO PIRANHA BRANCA |
| 20112 | PEIXE INTEIRO PIRANHA CACHORRA |
| 20102 | PEIXE INTEIRO PIRANHA CAJU |
| 20104 | PEIXE INTEIRO PIRANHA CHATA |
| 20108 | PEIXE INTEIRO PIRANHA DA LAGOA |
| 20119 | PEIXE INTEIRO PIRANHA DOCE |
| 20113 | PEIXE INTEIRO PIRANHA MAPARA |
| 20111 | PEIXE INTEIRO PIRANHA PEBA |
| 20105 | PEIXE INTEIRO PIRANHA PINTADA |
| 20109 | PEIXE INTEIRO PIRANHA PRETA |
| 20103 | PEIXE INTEIRO PIRANHA VERMELHA |
| 33106 | PEIXE INTEIRO PIRANIAMBU |
| 33108 | PEIXE INTEIRO PIRANIAMPU |
| 39103 | PEIXE INTEIRO PIRAPARA |
| 42103 | PEIXE INTEIRO PIRAPEMA |
| 18101 | PEIXE INTEIRO PIRAPEUA |
| 18105 | PEIXE INTEIRO PIRAPEUAUA |
| 37103 | PEIXE INTEIRO PIRAPITA |
| 37102 | PEIXE INTEIRO PIRAPITANGA |
| 27102 | PEIXE INTEIRO PIRAPITINGA |
| 26107 | PEIXE INTEIRO PIRAPUCA |
| 26104 | PEIXE INTEIRO PIRAPUCU |
| 37101 | PEIXE INTEIRO PIRAPUTANGA |
| 30101 | PEIXE INTEIRO PIRARARA |
| 38120 | PEIXE INTEIRO PIRAROBA |
| 06101 | PEIXE INTEIRO PIRARUCU |
| 28103 | PEIXE INTEIRO PIRATINGA (PIRAIBA) |
| 34110 | PEIXE INTEIRO PIRATIQUEIRA |
| 04102 | PEIXE INTEIRO PIRAUACA |
| 30102 | PEIXE INTEIRO PIRAUARARA |
| 53109 | PEIXE INTEIRO PIRAUNA |
| 06106 | PEIXE INTEIRO PIRAURUCU |
| 53102 | PEIXE INTEIRO PIRAUVA |
| 16106 | PEIXE INTEIRO PIRAUVU |
| 21110 | PEIXE INTEIRO PIROSCA |
| 77101 | PEIXE INTEIRO PIRUCAIA |
| 11118 | PEIXE INTEIRO PITANGA |
| 23102 | PEIXE INTEIRO PITANGOLA |
| 19104 | PEIXE INTEIRO PITITINGA |
| 27103 | PEIXE INTEIRO PIXIMA |
| 42118 | PEIXE INTEIRO POMBOCA |
| 04128 | PEIXE INTEIRO PONQUINHA |
| 04110 | PEIXE INTEIRO POQUINHA |
| 41112 | PEIXE INTEIRO PORCO |
| 41119 | PEIXE INTEIRO PORQUINHO |
| 34111 | PEIXE INTEIRO PRATIBU |
| 34103 | PEIXE INTEIRO PRATIQUEIRA |
| 31111 | PEIXE INTEIRO PRECAMBUCU |
| 31112 | PEIXE INTEIRO PRECUMBUCU |
| 11105 | PEIXE INTEIRO PUNARE |
| 11117 | PEIXE INTEIRO PUTANGA |
| 08102 | PEIXE INTEIRO QUATI |
| 53107 | PEIXE INTEIRO QUINDUNDE |
| 10114 | PEIXE INTEIRO QUIRA |
| 27136 | PEIXE INTEIRO RABO SECO |
| 07101 | PEIXE INTEIRO RAIA |
| 07115 | PEIXE INTEIRO RAIA AMARELA |
| 07125 | PEIXE INTEIRO RAIA ARARA |
| 07119 | PEIXE INTEIRO RAIA BORBOLETA |
| 07104 | PEIXE INTEIRO RAIA CARIMBADA |
| 07114 | PEIXE INTEIRO RAIA CHITA |
| 07107 | PEIXE INTEIRO RAIA LIXA |
| 07118 | PEIXE INTEIRO RAIA MANTEIGA |
| 07124 | PEIXE INTEIRO RAIA PINTADA |
| 07111 | PEIXE INTEIRO RAIA PREGO |
| 07105 | PEIXE INTEIRO RAIA VIOLA |
| 45111 | PEIXE INTEIRO RATO |
| 54101 | PEIXE INTEIRO REI (PEIXE REI) |
| 37107 | PEIXE INTEIRO REI DOS PEIXES (BEIJUPIRA) |
| 01108 | PEIXE INTEIRO ROBAFO |
| 30109 | PEIXE INTEIRO ROBALAO |
| 30110 | PEIXE INTEIRO ROBALETE |
| 30101 | PEIXE INTEIRO ROBALO |
| 30108 | PEIXE INTEIRO ROBALO BICUDO |
| 30111 | PEIXE INTEIRO ROBALO FLECHA |
| 30105 | PEIXE INTEIRO ROBALO PEBA |
| 30121 | PEIXE INTEIRO ROBAO |
| 20122 | PEIXE INTEIRO RODOLEIRA |
| 29109 | PEIXE INTEIRO RODOVALHO |
| 26101 | PEIXE INTEIRO RONCADOR |
| 01110 | PEIXE INTEIRO RUBAFO |
| 05118 | PEIXE INTEIRO SABAO |
| 42106 | PEIXE INTEIRO SABARU |
| 59106 | PEIXE INTEIRO SABONETE |
| 42110 | PEIXE INTEIRO SABURU |
| 42105 | PEIXE INTEIRO SAGARU |
| 71103 | PEIXE INTEIRO SAGUA |
| 42109 | PEIXE INTEIRO SAGUACU |
| 42107 | PEIXE INTEIRO SAGUIRA |
| 42104 | PEIXE INTEIRO SAGUIRI |
| 42103 | PEIXE INTEIRO SAGUIRU |
| 26110 | PEIXE INTEIRO SAICANGA |
| 16105 | PEIXE INTEIRO SAIJE |
| 16104 | PEIXE INTEIRO SAIPE |
| 42101 | PEIXE INTEIRO SAIRU |
| 42102 | PEIXE INTEIRO SAIRU DE RIO |
| 10119 | PEIXE INTEIRO SAJUBA |
| 10125 | PEIXE INTEIRO SAJUNA |
| 59102 | PEIXE INTEIRO SALMAO PEQUENO (SALMONETE) |
| 59104 | PEIXE INTEIRO SALMONEJO |
| 59101 | PEIXE INTEIRO SALMONETE |
| 20109 | PEIXE INTEIRO SAMBETARA |
| 78102 | PEIXE INTEIRO SAMBUDA |
| 78101 | PEIXE INTEIRO SAMBUIO |
| 71104 | PEIXE INTEIRO SANHOA |
| 28112 | PEIXE INTEIRO SAPA |
| 31105 | PEIXE INTEIRO SAPO |
| 18114 | PEIXE INTEIRO SAPURANA |
| 18111 | PEIXE INTEIRO SAPURUNA |
| 11116 | PEIXE INTEIRO SARABIANA |
| 59103 | PEIXE INTEIRO SARAMONETE |
| 59107 | PEIXE INTEIRO SARAMUNETE |
| 26111 | PEIXE INTEIRO SARANHA |
| 48103 | PEIXE INTEIRO SARAPO |
| 50105 | PEIXE INTEIRO SARAVELHA |
| 24102 | PEIXE INTEIRO SARDA |
| 19104 | PEIXE INTEIRO SARDA BRACO DE MOCA (MAPARA) |
| 24105 | PEIXE INTEIRO SARDA DE AGUA DOCE |
| 50102 | PEIXE INTEIRO SARDELA |
| 11101 | PEIXE INTEIRO SARDINHA |
| 11105 | PEIXE INTEIRO SARDINHA BRANCA |
| 11103 | PEIXE INTEIRO SARDINHA CASCUDA |
| 24101 | PEIXE INTEIRO SARDINHA DE AGUA DOCE |
| 11106 | PEIXE INTEIRO SARDINHA DOURADA |
| 24103 | PEIXE INTEIRO SARDINHA GRANDE DE AGUA DOCE |
| 11111 | PEIXE INTEIRO SARDINHA IMPERIAL |
| 11102 | PEIXE INTEIRO SARDINHA LAJE |
| 11109 | PEIXE INTEIRO SARDINHA MAROMBA |
| 11104 | PEIXE INTEIRO SARDINHA NAVALHA |
| 11107 | PEIXE INTEIRO SARDINHA PRATA |
| 50106 | PEIXE INTEIRO SARDINHA SAVELHA (SAVELHA) |
| 11110 | PEIXE INTEIRO SARDINHA VERDADEIRA |
| 24104 | PEIXE INTEIRO SARDINHAO (AGUA DOCE) |
| 14113 | PEIXE INTEIRO SARGENTO |
| 71106 | PEIXE INTEIRO SAUARA |
| 13103 | PEIXE INTEIRO SAUBA |
| 10126 | PEIXE INTEIRO SAUNA |
| 50101 | PEIXE INTEIRO SAVELHA |
| 35141 | PEIXE INTEIRO SEBASTIAO |
| 38119 | PEIXE INTEIRO SEMENDUARA |
| 57110 | PEIXE INTEIRO SERIGADO CHERNE (CHERNE) |
| 28118 | PEIXE INTEIRO SERIGADO FOCINHUDO (GAROUPA) |
| 28117 | PEIXE INTEIRO SERIGADO MERO (GAROUPA) |
| 57109 | PEIXE INTEIRO SERIGADO TAPOA (CHERNE) |
| 28119 | PEIXE INTEIRO SERIGADO VERMELHO (GAROUPA) |
| 24101 | PEIXE INTEIRO SERRA |
| 05120 | PEIXE INTEIRO SERRA DE AGUA DOCE (JACUNDA) |
| 24103 | PEIXE INTEIRO SERRA DE ESCAMA |
| 16109 | PEIXE INTEIRO SERRA PIMA |
| 20103 | PEIXE INTEIRO SINHARA |
| 28114 | PEIXE INTEIRO SIRIGAITA |
| 29104 | PEIXE INTEIRO SOIA |
| 17109 | PEIXE INTEIRO SOLDADO DE AGUA DOCE (TAMOATA) |
| 34104 | PEIXE INTEIRO SOLE |
| 29103 | PEIXE INTEIRO SOLHA |
| 16110 | PEIXE INTEIRO SOROROCA |
| 04119 | PEIXE INTEIRO SORUBI |
| 04120 | PEIXE INTEIRO SORUBIM |
| 40106 | PEIXE INTEIRO SULAMBA |
| 28113 | PEIXE INTEIRO SULAPEBA |
| 06109 | PEIXE INTEIRO SUREL |
| 04118 | PEIXE INTEIRO SURUBI |
| 04101 | PEIXE INTEIRO SURUBIM |
| 04108 | PEIXE INTEIRO SURUBIM CAPARARI |
| 04116 | PEIXE INTEIRO SURUBIM CHICOTE |
| 04112 | PEIXE INTEIRO SURUBIM LIMA |
| 18103 | PEIXE INTEIRO SURUBIM MENA (PIRAPEUA) |
| 04109 | PEIXE INTEIRO SURUBIM PINTADO |
| 04111 | PEIXE INTEIRO SURUBIM RAJADO |
| 04121 | PEIXE INTEIRO SURUMBI |
| 04114 | PEIXE INTEIRO SURUMI |
| 35101 | PEIXE INTEIRO TABARANA |
| 38118 | PEIXE INTEIRO TABUA |
| 10117 | PEIXE INTEIRO TACUJI |
| 07106 | PEIXE INTEIRO TAGUARA |
| 10101 | PEIXE INTEIRO TAINHA |
| 34109 | PEIXE INTEIRO TAINHA CHATA (PARATI) |
| 10104 | PEIXE INTEIRO TAINHA CURIMA |
| 10106 | PEIXE INTEIRO TAINHA DE CORRIDA |
| 10103 | PEIXE INTEIRO TAINHA DE CORSO |
| 10105 | PEIXE INTEIRO TAINHA DE ENTRADA |
| 34108 | PEIXE INTEIRO TAINHA DE OLHO AMARELO (PARATI) |
| 10109 | PEIXE INTEIRO TAINHA OLHO PRETO |
| 10112 | PEIXE INTEIRO TAINHA PAU |
| 10116 | PEIXE INTEIRO TAINHA PITIUA |
| 10113 | PEIXE INTEIRO TAINHA PITIUBA |
| 10107 | PEIXE INTEIRO TAINHA SECA |
| 10111 | PEIXE INTEIRO TAINHA URICHOCA |
| 10102 | PEIXE INTEIRO TAINHA VERDADEIRA |
| 10130 | PEIXE INTEIRO TAINHOTA |
| 26106 | PEIXE INTEIRO TAJABUCU |
| 26108 | PEIXE INTEIRO TAJAPECO |
| 26105 | PEIXE INTEIRO TAJIBUCU |
| 10120 | PEIXE INTEIRO TAMARATANA |
| 17106 | PEIXE INTEIRO TAMATA |
| 10133 | PEIXE INTEIRO TAMATARANA |
| 22101 | PEIXE INTEIRO TAMBAQUI |
| 20108 | PEIXE INTEIRO TAMBETARA |
| 03109 | PEIXE INTEIRO TAMBIU |
| 17103 | PEIXE INTEIRO TAMBOATA |
| 17102 | PEIXE INTEIRO TAMBUATA |
| 20102 | PEIXE INTEIRO TAMETARA |
| 17101 | PEIXE INTEIRO TAMOATA |
| 17104 | PEIXE INTEIRO TAMOATA PINIMA |
| 17105 | PEIXE INTEIRO TAMUATA |
| 10131 | PEIXE INTEIRO TANHOTA |
| 10123 | PEIXE INTEIRO TAPIARA |
| 03111 | PEIXE INTEIRO TAPIOCA |
| 23109 | PEIXE INTEIRO TAPIRANGA |
| 23108 | PEIXE INTEIRO TAPIRECA |
| 07109 | PEIXE INTEIRO TAQUARA |
| 01105 | PEIXE INTEIRO TARAIRA |
| 44108 | PEIXE INTEIRO TARANGAIO |
| 44109 | PEIXE INTEIRO TARANGALHO |
| 27103 | PEIXE INTEIRO TARAPITINGA |
| 01102 | PEIXE INTEIRO TARARIRA |
| 01103 | PEIXE INTEIRO TARIEIRA |
| 01104 | PEIXE INTEIRO TARIIRA |
| 42116 | PEIXE INTEIRO TARPAO |
| 20117 | PEIXE INTEIRO TEMBETARA |
| 17110 | PEIXE INTEIRO TESTUDO |
| 46109 | PEIXE INTEIRO TIBIRIO |
| 46103 | PEIXE INTEIRO TIBIRO |
| 07122 | PEIXE INTEIRO TICONHA |
| 27140 | PEIXE INTEIRO TICOPA |
| 45110 | PEIXE INTEIRO TIJUBARANA |
| 08101 | PEIXE INTEIRO TILAPIA |
| 46104 | PEIXE INTEIRO TIMBIRA |
| 46110 | PEIXE INTEIRO TIMBIRO |
| 72105 | PEIXE INTEIRO TIRAVIDA |
| 72101 | PEIXE INTEIRO TIRAVIRA |
| 72104 | PEIXE INTEIRO TIRIVIRA |
| 35139 | PEIXE INTEIRO TOLO |
| 37113 | PEIXE INTEIRO TORANDIJU |
| 40107 | PEIXE INTEIRO TORORO |
| 35105 | PEIXE INTEIRO TRAGUIRA |
| 01101 | PEIXE INTEIRO TRAIRA |
| 09102 | PEIXE INTEIRO TRAIRA PIXUNA (JEJU) |
| 09104 | PEIXE INTEIRO TRAIRA PIXURIA (JEJU) |
| 59101 | PEIXE INTEIRO TRAIRAO |
| 41104 | PEIXE INTEIRO TRAITINGA |
| 27104 | PEIXE INTEIRO TRAPITINGA |
| 01107 | PEIXE INTEIRO TRARIEIRA |
| 20104 | PEIXE INTEIRO TREMETARA |
| 48101 | PEIXE INTEIRO TRILHA |
| 49101 | PEIXE INTEIRO TRUTA |
| 35104 | PEIXE INTEIRO TUBARANA |
| 06103 | PEIXE INTEIRO TUBARAO DE MARIA (PIRARUCU) |
| 11101 | PEIXE INTEIRO TUCUNARE |
| 11104 | PEIXE INTEIRO TUCUNARE ACO |
| 11102 | PEIXE INTEIRO TUCUNARE ACU |
| 11109 | PEIXE INTEIRO TUCUNARE BRANCO |
| 11107 | PEIXE INTEIRO TUCUNARE LUMBINHO |
| 11113 | PEIXE INTEIRO TUCUNARE PINIMA |
| 11112 | PEIXE INTEIRO TUCUNARE PITANGA |
| 11110 | PEIXE INTEIRO TUCUNARE PUTANGA |
| 11111 | PEIXE INTEIRO TUCUNARETINGA |
| 48102 | PEIXE INTEIRO TUI |
| 12106 | PEIXE INTEIRO UACARI |
| 30105 | PEIXE INTEIRO UARARA |
| 45101 | PEIXE INTEIRO UBARANA |
| 45106 | PEIXE INTEIRO UBARANA MIRIM |
| 45103 | PEIXE INTEIRO UBARANA RATO |
| 45105 | PEIXE INTEIRO UBARANA ROLICA |
| 45109 | PEIXE INTEIRO UBARANACU |
| 10132 | PEIXE INTEIRO URIACICA |
| 18123 | PEIXE INTEIRO URIBACO |
| 43106 | PEIXE INTEIRO URICECA |
| 10122 | PEIXE INTEIRO URICHOCA |
| 14115 | PEIXE INTEIRO URITINGA |
| 40104 | PEIXE INTEIRO URUANA |
| 23105 | PEIXE INTEIRO URUBAIANA |
| 45107 | PEIXE INTEIRO URUBARANA |
| 53108 | PEIXE INTEIRO VACA |
| 29107 | PEIXE INTEIRO VACU |
| 52104 | PEIXE INTEIRO VENTO LESTE |
| 79101 | PEIXE INTEIRO VERDE |
| 12101 | PEIXE INTEIRO VERMELHO |
| 12109 | PEIXE INTEIRO VERMELHO BOCA NEGRA |
| 12106 | PEIXE INTEIRO VERMELHO DO FUNDO |
| 12108 | PEIXE INTEIRO VERMELHO HENRIQUE |
| 12107 | PEIXE INTEIRO VERMELHO VERDADEIRO |
| 07108 | PEIXE INTEIRO VIOLA |
| 10124 | PEIXE INTEIRO VIROTE |
| 72103 | PEIXE INTEIRO VIUVA |
| 57101 | PEIXE INTEIRO VOADOR |
| 19102 | PEIXE INTEIRO XANGO |
| 32105 | PEIXE INTEIRO XARELETE |
| 39102 | PEIXE INTEIRO XAREO |
| 39101 | PEIXE INTEIRO XAREU |
| 39106 | PEIXE INTEIRO XAREU BRANCO |
| 32108 | PEIXE INTEIRO XAREU DOURADO (XERELETE) |
| 32110 | PEIXE INTEIRO XAREU PEQUENO (XERELETE) |
| 39110 | PEIXE INTEIRO XAREU PRETO |
| 39105 | PEIXE INTEIRO XAREU RONCADOR |
| 39104 | PEIXE INTEIRO XAREU VAQUEIRO |
| 39103 | PEIXE INTEIRO XAREU VERDADEIRO |
| 32101 | PEIXE INTEIRO XERELETE |
| 32104 | PEIXE INTEIRO XERERETE |
| 18113 | PEIXE INTEIRO XIRA |
| 17113 | PEIXE INTEIRO ZABUCAI |
| 51901 | PEIXE MATRINXA (FORMA DE COMERCIALIZACAO NAO-DISCRIMINADA) |
| 00901 | PEIXE NAO-ESPECIFICADO |
| 27901 | PEIXE PESCADA (FORMA DE COMERCIALIZACAO NAO-DISCRIMINADA) |
| 27902 | PEIXE PESCADA AMARELA (FORMA DE COMERCIALIZACAO NAO-DISCRIMINADA) |
| 27903 | PEIXE PESCADA BRANCA (FORMA DE COMERCIALIZACAO NAO-DISCRIMINADA) |
| 04501 | PEIXE PESCADA EM CONSERVA |
| 04901 | PEIXE PINTADO (FORMA DE COMERCIALIZACAO NAO-DISCRIMINADA) |
| 75401 | PEIXE SALGADO ABROTE |
| 02401 | PEIXE SALGADO ACARA |
| 12401 | PEIXE SALGADO ACARI |
| 44401 | PEIXE SALGADO AGULHA |
| 58401 | PEIXE SALGADO AGULHAO |
| 56401 | PEIXE SALGADO ALBACORA |
| 01401 | PEIXE SALGADO ANCHOVA |
| 23401 | PEIXE SALGADO ANUJA |
| 07401 | PEIXE SALGADO ARACU |
| 40401 | PEIXE SALGADO ARUANA |
| 22401 | PEIXE SALGADO ATUM |
| 29401 | PEIXE SALGADO BACU |
| 02401 | PEIXE SALGADO BADEJO |
| 46401 | PEIXE SALGADO BAGRE DE AGUA DOCE |
| 14401 | PEIXE SALGADO BAGRE DE MAR |
| 37401 | PEIXE SALGADO BEIJUPIRA |
| 47401 | PEIXE SALGADO BICUDA |
| 51401 | PEIXE SALGADO BODIAO |
| 49401 | PEIXE SALGADO BONITO |
| 35401 | PEIXE SALGADO CACAO |
| 26401 | PEIXE SALGADO CACHORRO |
| 71401 | PEIXE SALGADO CAICANHA |
| 42401 | PEIXE SALGADO CAMURUPIM |
| 41401 | PEIXE SALGADO CANGULO |
| 40401 | PEIXE SALGADO CARAMURU |
| 15401 | PEIXE SALGADO CARAPEBA |
| 04401 | PEIXE SALGADO CASTANHA |
| 16401 | PEIXE SALGADO CAVALA |
| 05401 | PEIXE SALGADO CAVALINHA |
| 57401 | PEIXE SALGADO CHERNE |
| 06401 | PEIXE SALGADO CHICHARRO |
| 13401 | PEIXE SALGADO CIOBA |
| 18401 | PEIXE SALGADO CORCOROCA |
| 03401 | PEIXE SALGADO CORVINA |
| 10401 | PEIXE SALGADO CURIMATA |
| 00401 | PEIXE SALGADO DE AGUA DOCE NAO-ESPECIFICADO |
| 00401 | PEIXE SALGADO DE MAR NAO-ESPECIFICADO |
| 32401 | PEIXE SALGADO DOURADA |
| 16401 | PEIXE SALGADO DOURADO DE AGUA DOCE |
| 21401 | PEIXE SALGADO DOURADO DE MAR |
| 33401 | PEIXE SALGADO ESPADA |
| 34401 | PEIXE SALGADO FIDALGO |
| 17401 | PEIXE SALGADO GALO |
| 28401 | PEIXE SALGADO GAROUPA |
| 46401 | PEIXE SALGADO GUAIVIRA |
| 43401 | PEIXE SALGADO GUARAJUBA |
| 48401 | PEIXE SALGADO ITUI |
| 05401 | PEIXE SALGADO JACUNDA |
| 25401 | PEIXE SALGADO JARAQUI |
| 38401 | PEIXE SALGADO JAU |
| 09401 | PEIXE SALGADO JEJU |
| 03401 | PEIXE SALGADO LAMBARI |
| 29401 | PEIXE SALGADO LINGUADO |
| 36401 | PEIXE SALGADO MANDUBI |
| 19401 | PEIXE SALGADO MANJUBA |
| 19401 | PEIXE SALGADO MAPARA |
| 51401 | PEIXE SALGADO MATRINXA |
| 36401 | PEIXE SALGADO MERLUZA |
| 31401 | PEIXE SALGADO MERO |
| 53401 | PEIXE SALGADO MIRAGAIA |
| 55401 | PEIXE SALGADO MOREIA |
| 15401 | PEIXE SALGADO MUCUM |
| 08401 | PEIXE SALGADO NAMORADO |
| 00401 | PEIXE SALGADO NAO-ESPECIFICADO |
| 23401 | PEIXE SALGADO OLHO DE BOI |
| 31401 | PEIXE SALGADO PACAMAO DE AGUA DOCE |
| 21401 | PEIXE SALGADO PACU |
| 52401 | PEIXE SALGADO PALOMBETA |
| 38401 | PEIXE SALGADO PAMPO |
| 20401 | PEIXE SALGADO PAPATERRA |
| 34401 | PEIXE SALGADO PARATI |
| 25401 | PEIXE SALGADO PARGO |
| 27401 | PEIXE SALGADO PESCADA |
| 09401 | PEIXE SALGADO PESCADINHA |
| 27401 | PEIXE SALGADO PIABANHA |
| 39401 | PEIXE SALGADO PIAPARA |
| 14401 | PEIXE SALGADO PIAU |
| 41401 | PEIXE SALGADO PIRACANJUBA |
| 28401 | PEIXE SALGADO PIRAIBA |
| 13401 | PEIXE SALGADO PIRAMUTABA |
| 33401 | PEIXE SALGADO PIRANAMBU |
| 20401 | PEIXE SALGADO PIRANHA |
| 18401 | PEIXE SALGADO PIRAPEUA |
| 37401 | PEIXE SALGADO PIRAPUTANGA |
| 30401 | PEIXE SALGADO PIRARARA |
| 06401 | PEIXE SALGADO PIRARUCU |
| 07401 | PEIXE SALGADO RAIA |
| 54401 | PEIXE SALGADO REI (PEIXE REI) |
| 30401 | PEIXE SALGADO ROBALO |
| 26401 | PEIXE SALGADO RONCADOR |
| 42401 | PEIXE SALGADO SAIRU |
| 59401 | PEIXE SALGADO SALMONETE |
| 11401 | PEIXE SALGADO SARDINHA |
| 24401 | PEIXE SALGADO SARDINHA DE AGUA DOCE |
| 50401 | PEIXE SALGADO SAVELHA |
| 24401 | PEIXE SALGADO SERRA |
| 04401 | PEIXE SALGADO SURUBIM |
| 35401 | PEIXE SALGADO TABARANA |
| 10401 | PEIXE SALGADO TAINHA |
| 22401 | PEIXE SALGADO TAMBAQUI |
| 17401 | PEIXE SALGADO TAMOATA |
| 08401 | PEIXE SALGADO TILAPIA |
| 72401 | PEIXE SALGADO TIRAVIRA |
| 01401 | PEIXE SALGADO TRAIRA |
| 48401 | PEIXE SALGADO TRILHA |
| 49401 | PEIXE SALGADO TRUTA |
| 11401 | PEIXE SALGADO TUCUNARE |
| 45401 | PEIXE SALGADO UBARANA |
| 12401 | PEIXE SALGADO VERMELHO |
| 39401 | PEIXE SALGADO XAREU |
| 32401 | PEIXE SALGADO XERELETE |
| 03001 | PEIXE SARDINHA EM CONSERVA |
| 06201 | PEIXE SARDINHA EM CONSERVA LIGHT |
| 03501 | PEIXE SAVELHA EM CONSERVA |
| 00402 | PEIXE SECO NAO-ESPECIFICADO |
| 24901 | PEIXE SERRA (FORMA DE COMERCIALIZACAO NAO-DISCRIMINADA) |
| 04901 | PEIXE TAINHA EM CONSERVA |
| 22901 | PEIXE TAMBAQUI (FORMA DE COMERCIALIZACAO NAO-DISCRIMINADA) |
| 39901 | PEIXE XAREU (FORMA DE COMERCIALIZACAO NAO-DISCRIMINADA) |
| 12201 | PEIXINHO (LAGARTO DO BRACO) |
| 06601 | PELE DE FRANGO |
| 03902 | PELE DE PORCO PREPARADA |
| 03804 | PELE SUINA FRESCA |
| 20001 | PEN DRIVE |
| 01618 | PENEIRA |
| 02802 | PENEIRA DE FIBRA VEGETAL |
| 01403 | PENHORA (EMPRESTIMO) |
| 00920 | PENICO DE PLASTICO |
| 00402 | PENSAO ( MUNICIPAL, ESTADUAL, FEDERAL) |
| 00401 | PENSAO ( MUNICIPAL, ESTADUAL, FEDERAL) DA PREVIDENCIA PUBLICA |
| 00202 | PENSAO (INSS) |
| 02501 | PENSAO (PENSIONISTA) |
| 00701 | PENSAO ALIMENTICIA DE NAO MORADOR |
| 00702 | PENSAO ALIMENTICIA DE NAO-MORADOR |
| 00501 | PENSAO ALIMENTICIA EM DINHEIRO |
| 00201 | PENSAO DO INSTITUTO NACIONAL DE SEGURIDADE SOCIAL (INSS) |
| 02401 | PENSIONISTA (RENDIMENTO) |
| 00705 | PENSOES |
| 00102 | PENTE |
| 02701 | PENTEADEIRA |
| 00204 | PENTEADO (CABELEIREIRO) |
| 04306 | PEPININHO (MAXIXE) |
| 04001 | PEPINO |
| 04307 | PEPINO DE ESPINHO (MAXIXE) |
| 03701 | PEPINO EM CONSERVA |
| 10601 | PEPINO ORGANICO |
| 00105 | PEPSI |
| 00106 | PEPSI COLA |
| 09003 | PEPSI COLA DIET |
| 00804 | PEPSI COLA DIET |
| 09006 | PEPSI COLA DIETETICA |
| 00809 | PEPSI COLA DIETETICA |
| 00803 | PEPSI DIET |
| 00808 | PEPSI DIETETICA |
| 00603 | PEPSI-COLA |
| 06701 | PEQUI |
| 06703 | PEQUIA |
| 01406 | PERA |
| 03601 | PERA |
| 03603 | PERA ACIDA |
| 03604 | PERA DAGUA |
| 03602 | PERA DE AGUA |
| 03704 | PERDIZ ABATIDA, CONGELADA OU VIVA |
| 03715 | PERDIZ CONGELADA |
| 03716 | PERDIZ VIVA |
| 00703 | PERFEX |
| 00201 | PERFUME |
| 00903 | PERICIA DE VEICULO (TAXA) |
| 00212 | PERMANENTE (CABELEIREIRO) |
| 03106 | PERNA BOVINA |
| 05607 | PERNIL DE CARNEIRO |
| 06301 | PERNIL NAO-ESPECIFICADO |
| 03401 | PERNIL SUINO |
| 01701 | PERNIL SUINO ASSADO PARA VIAGEM |
| 01601 | PERSIANAS |
| 05301 | PERSONAL TRAINER |
| 01501 | PERU ABATIDO |
| 02601 | PERU ASSADO OU DEFUMADO PARA VIAGEM |
| 01601 | PERU CONGELADO |
| 01603 | PERU CONGELADO TEMPERADO |
| 02602 | PERU COZIDO PARA VIAGEM |
| 02603 | PERU DEFUMADO PARA VIAGEM |
| 02202 | PERU EM PE |
| 01702 | PERU EM PEDACO NAO-ESPECIFICADO |
| 03101 | PERU EMPANADO |
| 01602 | PERU TEMPERADO CONGELADO |
| 02201 | PERU VIVO |
| 00901 | PERUCA |
| 04502 | PES DE GELADEIRA DE METAL OU LOUCA |
| 08801 | PES PARA FOGAO DE METAL OU LOUCA |
| 04501 | PES PARA GELADEIRA DE METAL OU LOUCA |
| 04305 | PESAGEM (PROFISSIONAIS VOLANTES) |
| 03308 | PESCA |
| 05103 | PESCA PAGA |
| 04502 | PESCADA EM CONSERVA |
| 01202 | PESCARIA EM PARQUE DE DIVERSOES |
| 00802 | PESCOCO DE FRANGO |
| 00801 | PESCOCO DE GALINHA OU FRANGO |
| 04101 | PESCOCO DE PERU |
| 00510 | PESO DE CHUMBO DE PESCA |
| 05101 | PESQUE-PAGUE (INGRESSO) |
| 01204 | PESSEGADA |
| 03701 | PESSEGO |
| 02417 | PESSEGO DESIDRATADO |
| 01304 | PESSEGO EM CALDA |
| 01107 | PESSEGO MATE |
| 06505 | PESSEGO ORGANICO |
| 08701 | PESSEGO ORGANICO |
| 02408 | PESSEGO SECO OU DESIDRATADO |
| 02408 | PETA |
| 02410 | PETA DE GOMA |
| 02409 | PETA DE POLVILHO |
| 02411 | PETAS |
| 00231 | PETECA |
| 00202 | PETIT POIS |
| 01206 | PETIT SUISSE (IOGURTE) |
| 00208 | PIA |
| 00208 | PIA |
| 04702 | PIA (CUBA) DE QUALQUER MATERIAL |
| 04702 | PIA (CUBA) DE QUALQUER MATERIAL |
| 03109 | PIA DE COZINHA |
| 00301 | PIANO |
| 00701 | PIANO (ALUGUEL) |
| 15605 | PICADEIRA |
| 00304 | PICANHA |
| 11504 | PICANHA ORGANICA |
| 01615 | PICARETA |
| 04001 | PICLES |
| 04503 | PICOLE |
| 04805 | PICOLE CASEIRO |
| 11405 | PICOLE CASEIRO LIGHT |
| 00502 | PICOLE DE QUALQUER SABOR INDUSTRIALIZADO |
| 09702 | PICOLE DE QUALQUER SABOR INDUSTRIALIZADO DIET |
| 09707 | PICOLE DE QUALQUER SABOR INDUSTRIALIZADO DIETETICO |
| 09602 | PICOLE DE QUALQUER SABOR INDUSTRIALIZADO LIGHT |
| 04801 | PICOLE ENSACADO |
| 11401 | PICOLE ENSACADO LIGHT |
| 00505 | PICOLE INDUSTRIALIZADO |
| 09705 | PICOLE INDUSTRIALIZADO DIET |
| 09710 | PICOLE INDUSTRIALIZADO DIETETICO |
| 09605 | PICOLE INDUSTRIALIZADO LIGHT |
| 09302 | PICOLE LIGHT |
| 05202 | PIERCING |
| 01305 | PIJAMA |
| 00702 | PIJAMA |
| 00802 | PIJAMA |
| 01301 | PIJAMA E ROBE |
| 08901 | PILAO |
| 05901 | PILHA |
| 01702 | PIMENTA BODE EM CONSERVA |
| 04706 | PIMENTA BODE IN NATURA |
| 04606 | PIMENTA BRANCA IN NATURA |
| 04704 | PIMENTA CABACINHA IN NATURA |
| 11701 | PIMENTA CALABRESA |
| 08801 | PIMENTA CAMBUCI |
| 04712 | PIMENTA CHEIROSA |
| 04608 | PIMENTA CHIFRE DE VEADO IN NATURA |
| 05702 | PIMENTA CUMARI EM CONSERVA |
| 04602 | PIMENTA CUMARI IN NATURA |
| 04803 | PIMENTA CUMARI MIUDA IN NATURA |
| 03105 | PIMENTA DA INDIA (PIMENTA DO REINO) |
| 04711 | PIMENTA DE CHAPEU IN NATURA |
| 04701 | PIMENTA DE CHEIRO |
| 01701 | PIMENTA DE CHEIRO EM CONSERVA |
| 05701 | PIMENTA DE CUMARI EM CONSERVA |
| 04605 | PIMENTA DE CUMARI IN NATURA |
| 08501 | PIMENTA DEDO DE MOCA |
| 03109 | PIMENTA DO REINO |
| 03106 | PIMENTA DO REINO BRANCA |
| 03104 | PIMENTA DO REINO CLARA |
| 05301 | PIMENTA DO REINO E ALHO EM PO (TEMPERO MISTO EM PO OU EM GRAO) |
| 03201 | PIMENTA DO REINO E COMINHO |
| 03103 | PIMENTA DO REINO EM GRAO |
| 05303 | PIMENTA DO REINO EM GRAO E ALHO (TEMPERO MISTO EM PO OU EM GRAO) |
| 03102 | PIMENTA DO REINO EM PO |
| 03101 | PIMENTA DO REINO EM PO OU EM GRAO |
| 03108 | PIMENTA DO REINO ESCURA |
| 03107 | PIMENTA DO REINO PRETA |
| 04707 | PIMENTA DOCE IN NATURA |
| 01803 | PIMENTA EM CONSERVA NAO-ESPECIFICADA |
| 06101 | PIMENTA EM PO |
| 07602 | PIMENTA IN NATURA (NAO-DISCRIMINADA) |
| 07601 | PIMENTA IN NATURA NAO-DISCRIMINADA |
| 08701 | PIMENTA JAPONESA |
| 04607 | PIMENTA MADEIRA IN NATURA |
| 04601 | PIMENTA MALAGUETA |
| 01601 | PIMENTA MALAGUETA EM CONSERVA |
| 04710 | PIMENTA MORANGA IN NATURA |
| 04703 | PIMENTA MURICI IN NATURA |
| 04705 | PIMENTA MURUPI IN NATURA |
| 07603 | PIMENTA NAO ESPECIFICADA IN NATURA |
| 01801 | PIMENTA NAO-ESPECIFICADA EM CONSERVA |
| 04709 | PIMENTA OLHO DE BODE IN NATURA |
| 04802 | PIMENTA PASSARINHO IN NATURA |
| 04702 | PIMENTA PITANGA IN NATURA |
| 04801 | PIMENTA POMBINHA IN NATURA |
| 04708 | PIMENTA SINO IN NATURA |
| 04501 | PIMENTAO |
| 04504 | PIMENTAO AMARELO |
| 03001 | PIMENTAO EM PO |
| 04503 | PIMENTAO MADURO |
| 08003 | PIMENTAO MADURO ORGANICO |
| 08001 | PIMENTAO ORGANICO |
| 04502 | PIMENTAO VERDE |
| 08002 | PIMENTAO VERDE ORGANICO |
| 04505 | PIMENTAO VERMELHO |
| 04804 | PIMENTINHA CUMARI |
| 01710 | PINA |
| 00804 | PINCA |
| 00209 | PINCEL |
| 01502 | PINCEL |
| 00603 | PINCEL DE BARBA |
| 09001 | PINCEL DE COZINHA |
| 01501 | PINCEL, TINTA E VERNIZ PARA PINTURA DE TELA, VASO, ETC. |
| 02003 | PINCHAO |
| 00903 | PINGA |
| 00305 | PINGA |
| 00103 | PINGENTE |
| 03802 | PINHA (FRUTA DE CONDE) |
| 00403 | PINHA DO PARANA (PINHAO) |
| 00401 | PINHAO |
| 00402 | PINHAO DO PARANA |
| 01809 | PINHEIRO (MUDA) |
| 01403 | PINHO SOL (DESINFETANTE) |
| 01304 | PINO DE RELOGIO |
| 01305 | PINTOR |
| 01305 | PINTOR |
| 01701 | PINTURA (MATERIAL E MAO-DE-OBRA) |
| 01901 | PINTURA EM CAMISA |
| 00226 | PIPA |
| 00203 | PIPA DE AGUA (LITROS) |
| 00501 | PIPOCA DE ARROZ INDUSTRIALIZADA |
| 01204 | PIPOCA DE MILHO INDUSTRIALIZADA PARA VIAGEM |
| 04304 | PIPOCA DOCE |
| 01202 | PIPOCA DOCE OU SALGADA PARA VIAGEM |
| 01201 | PIPOCA PARA VIAGEM |
| 03747 | PIPOCA SALGADA |
| 01203 | PIPOCA SALGADA PARA VIAGEM |
| 00605 | PIPOQUEIRA (PANELA) |
| 03001 | PIQUENIQUE (TAXA) |
| 06702 | PIQUI |
| 06704 | PIQUIA |
| 06706 | PIQUIA AMARELO |
| 06705 | PIQUIA BRAVO |
| 06707 | PIQUIA DA AREIA |
| 06708 | PIQUIA PEDRA |
| 03803 | PIRACUI (FARINHA DE PEIXE) |
| 01806 | PIRAJA |
| 06601 | PIRAO PARA VIAGEM |
| 02003 | PIROGA |
| 00804 | PIRULITO |
| 00607 | PIRULITO |
| 09907 | PIRULITO DIET |
| 09920 | PIRULITO DIETETICO |
| 09807 | PIRULITO LIGHT |
| 00105 | PIS/PASEP (ABONO) |
| 00104 | PIS/PASEP (RENDIMENTOS) |
| 19002 | PISCINA DE FIBRA |
| 19001 | PISCINA DE PLASTICO |
| 03401 | PISCINA EM PARQUE, RESERVA, ETC. |
| 00102 | PISO |
| 00102 | PISO |
| 04901 | PISTA DE KART (INGRESSO) |
| 02201 | PISTACHE |
| 02202 | PISTACIO |
| 00404 | PISTOLA (EXCETO DE CACA) |
| 01641 | PISTOLA DE PINTURA |
| 06801 | PITANGA |
| 06802 | PITANGA COMUM |
| 06807 | PITANGA DE JARDIM |
| 06808 | PITANGA DO CAMPO |
| 06804 | PITANGA MIUDA |
| 06806 | PITANGA VERMELHA |
| 06803 | PITANGATUBA |
| 00701 | PITEIRA |
| 60113 | PITICAIA |
| 60115 | PITIGAIA |
| 44116 | PITIU |
| 06401 | PITOMBA |
| 06402 | PITOMBA DA MATA |
| 06403 | PITOMBA DE MACACO |
| 43103 | PITU (CAMARAO DE AGUA DOCE) |
| 02303 | PIVO |
| 08014 | PIZZA CALABREZA LIGHT PARA VIAGEM |
| 00914 | PIZZA CALABREZA PARA VIAGEM |
| 11202 | PIZZA EM FATIA LIGHT |
| 03708 | PIZZA EM PEDACO |
| 00208 | PIZZA EM PEDACO PARA VIAGEM |
| 08015 | PIZZA MUZZARELA LIGHT PARA VIAGEM |
| 00915 | PIZZA MUZZARELA PARA VIAGEM |
| 00920 | PIZZA PARA VIAGEM |
| 00917 | PIZZA PORTUGUESA |
| 08017 | PIZZA PORTUGUESA LIGHT PARA VIAGEM |
| 08016 | PIZZA PRESUNTO LIGHT PARA VIAGEM |
| 00916 | PIZZA PRESUNTO PARA VIAGEM |
| 08003 | PIZZA PRONTA LIGHT PARA VIAGEM |
| 00903 | PIZZA PRONTA PARA VIAGEM |
| 02402 | PIZZA SEMIPRONTA |
| 06002 | PIZZA SEMIPRONTA LIGHT |
| 08002 | PIZZA, NHOQUE, CANELONI, CAPELETI, FETUTINE, RAVIOLE LIGHT PARA VIAGEM |
| 00902 | PIZZA, NHOQUE, CANELONI, CAPELETI, FETUTINE, RAVIOLE PARA VIAGEM |
| 04305 | PLACA DE CIMENTO OU CONCRETO |
| 04305 | PLACA DE CIMENTO OU CONCRETO |
| 07501 | PLACA DE JAZIGO |
| 04301 | PLACA, ANEL, VIGA, PALANQUE, ETC. DE CIMENTO OU CONCRETO |
| 04301 | PLACA, ANEL, VIGA, PALANQUE, ETC. DE CIMENTO OU CONCRETO |
| 01630 | PLAINA |
| 14407 | PLAINA ELETRICA |
| 03201 | PLANETARIO (INGRESSO) |
| 00101 | PLANO DE ASSISTENCIA MEDICA (EMPRESA) |
| 04501 | PLANO DE ASSISTENCIA MEDICA (PARTICULAR) |
| 04403 | PLANO DE ASSISTENCIA ODONTOLOGICA (EMPRESA) |
| 00203 | PLANO DE ASSISTENCIA ODONTOLOGICA (PARTICULAR) |
| 02208 | PLANO DE FUNERAL |
| 04401 | PLANO EXCLUSIVAMENTE ODONTOLOGICO (EMPRESA) |
| 00201 | PLANO EXCLUSIVAMENTE ODONTOLOGICO (PARTICULAR) |
| 01501 | PLANO INCLINADO |
| 04402 | PLANO ODONTOLOGICO (EMPRESA) |
| 00202 | PLANO ODONTOLOGICO (PARTICULAR) |
| 00105 | PLANO SEGURO-SAUDE (EMPRESA) |
| 04502 | PLANO SEGURO-SAUDE (PARTICULAR) |
| 01702 | PLANTA PARA OBRA |
| 01702 | PLANTA PARA OBRA |
| 01661 | PLANTADEIRA MANUAL |
| 04604 | PLANTAS ARTIFICIAIS |
| 01802 | PLANTAS NATURAIS |
| 00825 | PLASTIC |
| 01501 | PLASTICO |
| 04503 | PLASTICO (MATERIAL PARA COBERTURA) |
| 04503 | PLASTICO (MATERIAL PARA COBERTURA) |
| 03802 | PLASTICO OU RESINA (PECA DE DECORACAO) |
| 02002 | PLASTICO PARA ACAMPAMENTO |
| 00604 | PLASTIFICACAO, ENCADERNACAO |
| 00901 | PLUMAS |
| 00402 | PNEU |
| 01212 | PNEU DE BICICLETA |
| 00819 | PO ACHOCOLATADO |
| 10319 | PO ACHOCOLATADO DIET |
| 10341 | PO ACHOCOLATADO DIETETICO |
| 10219 | PO ACHOCOLATADO LIGHT |
| 00404 | PO COMPACTO |
| 00405 | PO DE ARROZ |
| 02502 | PO DE CAFE |
| 10602 | PO DE CAFE ORGANICO |
| 02803 | PO DE DOCE, SORVETE E PUDIM |
| 02804 | PO DE FLAN |
| 02806 | PO DE MINGAU DE MILHO VERDE |
| 02304 | PO DE PEDRA |
| 02304 | PO DE PEDRA |
| 02406 | PO DE REFRESCO |
| 11106 | PO DE REFRESCO DIET |
| 11113 | PO DE REFRESCO DIETETICO |
| 11006 | PO DE REFRESCO LIGHT |
| 00601 | PO DE SERRA (COMBUSTIVEL DOMESTICO) |
| 00602 | PO DE SERRA (KG) (COMBUSTIVEL DOMESTICO) |
| 00820 | PO PARA MILK SHAKE |
| 10320 | PO PARA MILK SHAKE DIET |
| 10342 | PO PARA MILK SHAKE DIETETICO |
| 10220 | PO PARA MILK SHAKE LIGHT |
| 02809 | PO PARA MINGAU NAO-ESPECIFICADO |
| 02813 | PO PARA PUDIM |
| 02811 | PO PARA SORVETE |
| 07201 | POCHETE |
| 02304 | POCHETE DE CRIANCA |
| 10604 | POCKET PC |
| 01645 | PODAO |
| 04701 | PODOLOGO |
| 02216 | POKAN |
| 03201 | POLAINAS |
| 04101 | POLAINAS |
| 00214 | POLENTA FRITA PARA VIAGEM |
| 00218 | POLENTA PARA VIAGEM |
| 04502 | POLENTA SEMIPRONTA |
| 04505 | POLENTINA |
| 00817 | POLIDOR |
| 03301 | POLIDOR DE METAL |
| 01503 | POLIURETANO |
| 01503 | POLIURETANO |
| 06406 | POLPA DE ABACAXI |
| 06418 | POLPA DE ACEROLA |
| 06421 | POLPA DE BURITI |
| 06413 | POLPA DE CAJA |
| 06407 | POLPA DE CAJU |
| 06404 | POLPA DE COCO |
| 06408 | POLPA DE CUPUACU |
| 06401 | POLPA DE FRUTA CONGELADA |
| 06419 | POLPA DE FRUTA DE MANGABA |
| 06414 | POLPA DE GOIABA |
| 06409 | POLPA DE GRAVIOLA |
| 06410 | POLPA DE JENIPAPO |
| 06412 | POLPA DE MANGA |
| 06403 | POLPA DE MARACUJA |
| 06416 | POLPA DE MORANGO |
| 06415 | POLPA DE PITANGA |
| 06420 | POLPA DE TAMARINDO |
| 06411 | POLPA DE TANGERINA |
| 04705 | POLPA DE TOMATE |
| 06417 | POLPA DE UVA |
| 00901 | POLTRONA |
| 01504 | POLVILHO AZEDO |
| 01503 | POLVILHO DE MANDIOCA |
| 01512 | POLVILHO DOCE |
| 74101 | POLVO |
| 74201 | POLVO EM POSTAS |
| 74401 | POLVO SALGADO |
| 03403 | POMADA DE CALCADO |
| 02611 | POMADA INDETERMINADA |
| 02804 | POMADA VAGINAL |
| 03706 | POMBO ABATIDO, CONGELADO OU VIVO |
| 03719 | POMBO CONGELADO |
| 03720 | POMBO VIVO |
| 02302 | POMELO |
| 09701 | PONCHEIRA |
| 02303 | PONCHETE DE CRIANCA |
| 02201 | PONCHO DE LA |
| 03801 | PONCHO DE LA |
| 00902 | PONTA DA PA |
| 00807 | PONTA DE AGULHA |
| 00307 | PONTA DE ALCATRA |
| 11507 | PONTA DE ALCATRA ORGANICA |
| 00207 | PONTA DE CHULETA |
| 11407 | PONTA DE CHULETA ORGANICA |
| 01304 | PONTA DE COSTELA |
| 00302 | PONTA DE PATINHO |
| 11502 | PONTA DE PATINHO ORGANICA |
| 01102 | PONTA DE PEITO |
| 01622 | PONTEIRA |
| 02701 | PONTOS TURISTICOS (INGRESSO) |
| 00210 | POP LARANJA |
| 13011 | POP LARANJA LIGHT |
| 01510 | POP LARANJA LIGHT |
| 03720 | PORCAO DE CARNE |
| 10702 | PORCAO DE OUTROS QUEIJOS LIGHT |
| 03714 | PORCAO DE PEIXE FRITO |
| 03710 | PORCAO DE QUEIJO |
| 06202 | PORCAO DE QUEIJO DE MINAS FRESCAL ORGANICO |
| 10402 | PORCAO DE QUEIJO DE MINAS LIGHT |
| 10002 | PORCAO DE QUEIJO MUZZARELA LIGHT |
| 10202 | PORCAO DE QUEIJO PRATO LIGHT |
| 10502 | PORCAO DE QUEIJO RICOTA LIGHT |
| 03718 | PORCAO DE SALAME |
| 03719 | PORCAO DE SALAMINHO |
| 04113 | PORCAO E CERVEJA |
| 02902 | PORCELANA (PECA DE DECORACAO) |
| 08404 | PORCO ABATIDO |
| 08401 | PORCO OU LEITAO MORTO EVISCERADO |
| 05301 | PORCO VIVO |
| 01404 | PORONGO DE CHIMARRAO |
| 00302 | PORTA |
| 00302 | PORTA |
| 02901 | PORTA-BARBEADOR |
| 03801 | PORTA-BOLO DE PORCELANA |
| 05101 | PORTA-CD |
| 00204 | PORTA-CHEQUE DE MULHER |
| 04701 | PORTA-CINTO |
| 05103 | PORTA-COMPACT DISC |
| 00810 | PORTA-CONDIMENTO (AVULSO) |
| 00814 | PORTA-COPOS |
| 05102 | PORTA-FITAS CASSETE |
| 05104 | PORTA-FITAS DE VIDEOCASSETE |
| 04601 | PORTA-FRALDA |
| 04701 | PORTA-JOIAS |
| 01202 | PORTA-JORNAL |
| 01201 | PORTA-JORNAL E REVISTA |
| 03701 | PORTAL |
| 03701 | PORTAL |
| 05001 | PORTA-LAPISEIRA |
| 02403 | PORTA-MAMADEIRA DE ISOPOR |
| 01201 | PORTA-MAQUIAGEM |
| 03501 | PORTA-NIQUEIS |
| 02401 | PORTAO |
| 02401 | PORTAO |
| 02402 | PORTAO DE FERRO |
| 02402 | PORTAO DE FERRO |
| 02404 | PORTAO DE MADEIRA |
| 02403 | PORTAO ELETRICO |
| 02403 | PORTAO ELETRONICO |
| 09101 | PORTA-OBJETOS (MADEIRA, ACRILICO, LOUCA, METAL, ETC.) |
| 02501 | PORTA-OCULOS |
| 00916 | PORTA-PAPEL HIGIENICO |
| 01001 | PORTA-PATINS |
| 01506 | PORTA-RETRATO |
| 01203 | PORTA-REVISTA |
| 03001 | PORTA-SECADOR |
| 00908 | PORTA-TOALHA DE PLASTICO |
| 02901 | PORTA-TOALHA DE TECIDO |
| 00601 | PORTE DE ARMA |
| 00704 | PORTEIRA |
| 00703 | PORTEIRO |
| 00604 | POSTA BRANCA |
| 11804 | POSTA BRANCA ORGANICA |
| 00903 | POSTA GORDA |
| 00702 | POSTA VERMELHA |
| 11902 | POSTA VERMELHA ORGANICA |
| 05101 | POSTE |
| 05101 | POSTE |
| 05104 | POSTE DE CIMENTO |
| 05104 | POSTE DE CIMENTO |
| 05102 | POSTE DE MADEIRA |
| 05102 | POSTE DE MADEIRA |
| 05103 | POSTE DE METAL |
| 05103 | POSTE METALICO |
| 01505 | POSTER |
| 60116 | POTIMIRIM |
| 60108 | POTITINGA |
| 00101 | POUPANCA (DEPOSITO) |
| 00102 | POUPANCA (DEPOSITO/APLICACAO) |
| 00101 | POUPANCA (RETIRADA) |
| 00102 | POUPANCA (RETIRADA/RESGATE) |
| 00103 | POUPANCA COMPLEMENTAR DE PLANO/SEGURO-SAUDE (EMPRESA) |
| 00704 | POUSADAS |
| 05104 | PRANCHA ALISADORA DE CABELO |
| 00120 | PRANCHA DE SURFE |
| 00250 | PRANCHETA |
| 01302 | PRATA (PECA DE DECORACAO) |
| 06201 | PRATELEIRA |
| 02301 | PRATO DE ESMALTE |
| 00201 | PRATO DE LOUCA |
| 04403 | PRATO DESCARTAVEL |
| 04401 | PRATO OU BANDEJA DE PAPEL ALUMINIZADO OU PLASTICO |
| 00937 | PRATO PLASTICO |
| 06707 | PREA VIVA |
| 00705 | PREGADOR DE CABELO |
| 03701 | PREGADOR DE ROUPA |
| 01105 | PREGO |
| 01105 | PREGO |
| 02408 | PRE-LAVAGEM DE QUALQUER MARCA |
| 01002 | PREMIO OU GANHO EM JOGOS (LOTERIA, MEGA, ETC.) |
| 03902 | PREMIO POR DESEMPENHO DE ATIVIDADE |
| 05602 | PREMIO RESTITUIDO E INDENIZACAO PAGOS POR SEGURO OBRIGATORIO DE AUTO/UTILITARIOS |
| 05802 | PREMIO RESTITUIDO E INDENIZACAO PAGOS POR SEGURO OBRIGATORIO DE MOTO |
| 05502 | PREMIO RESTITUIDO E INDENIZACAO PAGOS POR SEGURO PESSOAL |
| 05702 | PREMIO RESTITUIDO E INDENIZACAO PAGOS POR SEGURO VOLUNTARIO DE AUTO/UTILITARIOS |
| 05902 | PREMIO RESTITUIDO E INDENIZACAO PAGOS POR SEGURO VOLUNTARIO DE MOTO |
| 00706 | PRENDEDOR DE CABELO |
| 02601 | PRENDEDOR DE CHUPETA |
| 02201 | PRENDEDOR DE GRAVATA |
| 03702 | PRENDEDOR DE ROUPA |
| 06102 | PREPARADOR DE ALIMENTOS ELETRICO |
| 03408 | PREPARO PARA BOLO |
| 00402 | PRESENTE DOACAO EM DINHEIRO PARA OUTRA UC |
| 05401 | PRESENTE EM DINHEIRO (ESPORADICO) |
| 05301 | PRESEPIO (DECORACAO) |
| 31001 | PRESERVATIVO E LUBRIFICANTE INTIMO |
| 31004 | PRESERVATIVO FEMININO |
| 31002 | PRESERVATIVO MASCULINO |
| 00127 | PRESILHA DE FRALDA |
| 04302 | PRESSAO ARTERIAL (EM POSTOS OU PROFISSIONAIS VOLANTES) |
| 00928 | PRESSURIZADOR DE CHUVEIRO |
| 00201 | PRESTACAO DO IMOVEL |
| 00705 | PRESTOBARBA |
| 02705 | PRESUNTADA |
| 02205 | PRESUNTINHO BISCOITO |
| 10105 | PRESUNTINHO BISCOITO LIGHT |
| 02904 | PRESUNTO BOVINO |
| 05102 | PRESUNTO DE CHESTER |
| 06502 | PRESUNTO DE CHESTER LIGHT |
| 02903 | PRESUNTO DE FRANGO |
| 06001 | PRESUNTO DE FRANGO LIGTH |
| 02902 | PRESUNTO DE PERU |
| 06102 | PRESUNTO DE PERU DEFUMADO LIGHT |
| 06101 | PRESUNTO DE PERU LIGHT |
| 02901 | PRESUNTO DE QUALQUER TIPO |
| 02303 | PRESUNTO E QUEIJO RETALHADOS PARA PIZZA |
| 06201 | PRESUNTO SUINO LIGHT |
| 06202 | PRESUNTO SUINO SEM GORDURA |
| 01805 | PREVIDENCIA ESTADUAL |
| 01808 | PREVIDENCIA FEDERAL |
| 01807 | PREVIDENCIA MILITAR |
| 01806 | PREVIDENCIA MUNICIPAL |
| 00601 | PREVIDENCIA PRIVADA ABERTA |
| 00604 | PREVIDENCIA PRIVADA FECHADA |
| 01801 | PREVIDENCIA PUBLICA (FEDERAL, ESTADUAL, MUNICIPAL E MILITAR) |
| 01810 | PREVIDENCIA PUBLICA FECHADA |
| 00506 | PROCEDIMENTO CIRURGICO |
| 00701 | PROCESSADOR DE ALIMENTOS |
| 06101 | PROCESSADOR DE ALIMENTOS ELETRICO |
| 01101 | PRODUTO DE BANHO |
| 01401 | PRODUTO DE LIMPEZA E HIGIENE DE ANIMAL |
| 01401 | PRODUTOS DE CARNAVAL |
| 01001 | PRODUTOS DE LIMPEZA DE PELE |
| 30801 | PRODUTOS PARA CUIDADOS BUCAIS E DENTAIS |
| 31101 | PRODUTOS PARA MASSAGEM E ESTETICA |
| 05301 | PRODUTOS PARA PISCINA |
| 05301 | PRODUTOS PARA PISCINA |
| 01003 | PROFISSIONAL DE DESIGN |
| 04803 | PROGRAMA COMPUTACIONAL DE CURSO |
| 01201 | PROGRAMA DE ERRADICACAO DO TRABALHO INFANTIL (PETI) |
| 01401 | PROGRAMAS DE RENDA MINIMA (OUTRAS; MUN.EST.FEDERAL) |
| 01402 | PROMESSA EM JORNAL |
| 00207 | PROMESSA RELIGIOSA |
| 01101 | PROPINA |
| 06101 | PROPOLIS |
| 02901 | PROSTITUTA |
| 06803 | PROTECTOR |
| 05601 | PROTEINA DE SOJA |
| 06401 | PROTEINA DE SOJA ORGANICA |
| 05603 | PROTEINA VEGETAL |
| 06403 | PROTEINA VEGETAL ORGANICA |
| 05501 | PROTEINAC |
| 02301 | PROTESE E APARELHO DENTARIO |
| 02702 | PROTESTO DE TITULO |
| 00603 | PROTETOR DE BERCO |
| 03201 | PROTETOR DE COBERTOR |
| 02001 | PROTETOR DE COLCHAO |
| 03101 | PROTETOR DE ENDREDON |
| 00820 | PROTETOR DE FERRO ELETRICO DE METAL |
| 04118 | PROTETOR DE JOELHO |
| 03301 | PROTETOR DE PORTA |
| 30909 | PROTETOR DE SEIO |
| 03301 | PROTETOR DE TRAVESSEIRO |
| 00509 | PROTETOR LABIAL |
| 00510 | PROTETOR SOLAR |
| 00503 | PROVEDOR DE ACESSO A INTERNET DISCADA |
| 03001 | PSICOLOGO E PSICOTERAPEUTA |
| 01609 | PUA |
| 61112 | PUA |
| 00609 | PUBA DE FUBA |
| 01514 | PUBA DE MANDIOCA |
| 00608 | PUBA DE MILHO |
| 00512 | PUCA |
| 04314 | PUDIM |
| 02608 | PUDIM DANETTE |
| 10908 | PUDIM DANETTE DIET |
| 10916 | PUDIM DANETTE DIETETICO |
| 10808 | PUDIM DANETTE LIGHT |
| 02602 | PUDIM DE COCO |
| 10902 | PUDIM DE COCO DIET |
| 10910 | PUDIM DE COCO DIETETICO |
| 10802 | PUDIM DE COCO LIGHT |
| 02605 | PUDIM DE FUBA |
| 10905 | PUDIM DE FUBA DIET |
| 10913 | PUDIM DE FUBA DIETETICO |
| 10805 | PUDIM DE FUBA LIGHT |
| 02606 | PUDIM DE LARANJA |
| 10906 | PUDIM DE LARANJA DIET |
| 10914 | PUDIM DE LARANJA DIETETICO |
| 10806 | PUDIM DE LARANJA LIGHT |
| 02604 | PUDIM DE LEITE CONDENSADO |
| 10904 | PUDIM DE LEITE CONDENSADO DIET |
| 10912 | PUDIM DE LEITE CONDENSADO DIETETICO |
| 10804 | PUDIM DE LEITE CONDENSADO LIGHT |
| 02601 | PUDIM DE QUALQUER SABOR |
| 10901 | PUDIM DE QUALQUER SABOR DIET |
| 10909 | PUDIM DE QUALQUER SABOR DIETETICO |
| 10801 | PUDIM DE QUALQUER SABOR LIGHT |
| 02603 | PUDIM DE QUEIJO |
| 10903 | PUDIM DE QUEIJO DIET |
| 10911 | PUDIM DE QUEIJO DIETETICO |
| 10803 | PUDIM DE QUEIJO LIGHT |
| 07301 | PUDIM DIET |
| 07302 | PUDIM DIETETICO |
| 05301 | PUFE |
| 01701 | PUFE |
| 02101 | PULMAO BOVINO |
| 09802 | PULMAO DE CAPRINO |
| 04202 | PULMAO SUINO |
| 00105 | PULOVER |
| 00104 | PULOVER |
| 00104 | PULOVER |
| 00102 | PULSEIRA (JOIA) |
| 01303 | PULSEIRA DE RELOGIO |
| 15603 | PULVERIZADOR DE AGROTOXICO |
| 06309 | PULVERIZADOR DE MULTIPLA ACAO |
| 01701 | PUNHO DE REDE |
| 01407 | PUPUNHA |
| 01805 | PUPUNHA |
| 00409 | PURE DE ALHO |
| 03501 | PURE DE BATATA EM CAIXA |
| 01202 | PURE DE FRUTAS |
| 04704 | PURE DE TOMATE |
| 00603 | PURGATIVO |
| 02501 | PURIFICADOR DE AGUA |
| 01601 | PURIFICADOR DE AGUA |
| 00102 | PURIFICADOR DE AR |
| 02505 | PURIFICADOR DE AR |
| 01501 | PURPURINA |
| 03901 | PURURUCA DE PORCO |
| 00502 | PUXADOR |
| 01902 | PUXADOR DE AGUA |
| 00103 | Q-BOA |
| 02413 | QBOA (ALVEJANTE) |
| 02403 | Q-REFRESKO |
| 11103 | Q-REFRESKO DIET |
| 11110 | Q-REFRESKO DIETETICO |
| 11003 | Q-REFRESKO LIGHT |
| 02411 | QSUCO |
| 02402 | Q-SUCO |
| 11102 | Q-SUCO DIET |
| 11109 | Q-SUCO DIETETICO |
| 11002 | Q-SUCO LIGHT |
| 03304 | QUADRAS DE TENIS (INGRESSO) |
| 00218 | QUADRO NEGRO |
| 01501 | QUADRO OU MOLDURA DE QUADRO, POSTER, PORTA-RETRATO, ETC. |
| 03403 | QUARTO SUINO |
| 02405 | QUEBRA QUEBRA |
| 03102 | QUEBRA QUEIXO |
| 13002 | QUEBRA QUEIXO DIET |
| 13004 | QUEBRA QUEIXO DIETETICO |
| 00211 | QUEBRA-CABECA |
| 02004 | QUECHIMIA |
| 07304 | QUECHIMIA FRESCAL ORGANICO |
| 06004 | QUECHIMIA LIGHT |
| 03902 | QUEIJADA |
| 03901 | QUEIJADINHA |
| 02223 | QUEIJINHO BISCOITO |
| 10123 | QUEIJINHO BISCOITO LIGHT |
| 02806 | QUEIJO ALPESTRE (FUNDIDO) |
| 01706 | QUEIJO BOLA |
| 08101 | QUEIJO BRIE |
| 02601 | QUEIJO CAMEMBERT |
| 02008 | QUEIJO CANASTRA |
| 07308 | QUEIJO CANASTRA FRESCAL ORGANICO |
| 06008 | QUEIJO CANASTRA LIGHT |
| 02011 | QUEIJO CATIARA |
| 07311 | QUEIJO CATIARA FRESCAL ORGANICO |
| 06011 | QUEIJO CATIARA LIGHT |
| 02104 | QUEIJO CAVALO |
| 06104 | QUEIJO CAVALO LIGHT |
| 08301 | QUEIJO CHANCLICHE |
| 07801 | QUEIJO CHEDAR |
| 07802 | QUEIJO CHEDDAR |
| 07803 | QUEIJO CHEEDAR |
| 03725 | QUEIJO COALHO |
| 02012 | QUEIJO COALHO |
| 01703 | QUEIJO COLONIAL |
| 05903 | QUEIJO COLONIAL LIGHT |
| 08201 | QUEIJO COTTAGE |
| 02902 | QUEIJO CREMOSO |
| 06502 | QUEIJO CREMOSO LIGHT |
| 01903 | QUEIJO CUIA |
| 02906 | QUEIJO DANONINHO |
| 06506 | QUEIJO DANONINHO LIGHT |
| 01809 | QUEIJO DE BUFALA (MUSSARELA) |
| 02005 | QUEIJO DE COALHO |
| 07305 | QUEIJO DE COALHO FRESCAL ORGANICO |
| 06005 | QUEIJO DE COALHO LIGHT |
| 01705 | QUEIJO DE COLONIA |
| 03404 | QUEIJO DE FEIJAO SOJA |
| 02003 | QUEIJO DE MANTEIGA |
| 07303 | QUEIJO DE MANTEIGA FRESCAL ORGANICO |
| 06003 | QUEIJO DE MANTEIGA LIGHT |
| 02701 | QUEIJO DE MARAJO |
| 02001 | QUEIJO DE MINAS |
| 02009 | QUEIJO DE MINAS CURADO |
| 07309 | QUEIJO DE MINAS CURADO FRESCAL ORGANICO |
| 06009 | QUEIJO DE MINAS CURADO LIGHT |
| 06201 | QUEIJO DE MINAS FRESCAL ORGANICO |
| 07301 | QUEIJO DE MINAS FRESCAL ORGANICO |
| 10401 | QUEIJO DE MINAS LIGHT |
| 06001 | QUEIJO DE MINAS LIGHT |
| 02404 | QUEIJO DE PORCO |
| 03401 | QUEIJO DE SOJA |
| 02103 | QUEIJO DE TRANCA |
| 06103 | QUEIJO DE TRANCA LIGHT |
| 01901 | QUEIJO DO REINO |
| 02010 | QUEIJO DO SERRO |
| 07310 | QUEIJO DO SERRO FRESCAL ORGANICO |
| 06010 | QUEIJO DO SERRO LIGHT |
| 02304 | QUEIJO E PRESUNTO PICADOS |
| 02302 | QUEIJO E PRESUNTO RETALHADOS PARA PIZZA |
| 02904 | QUEIJO EM PASTA |
| 06504 | QUEIJO EM PASTA LIGHT |
| 02111 | QUEIJO ESTEPE |
| 02109 | QUEIJO FOL EPI |
| 06109 | QUEIJO FOL EPI LIGHT |
| 02802 | QUEIJO FUNDIDO |
| 06402 | QUEIJO FUNDIDO LIGHT |
| 03301 | QUEIJO GORGONZOLA |
| 08401 | QUEIJO GOUDA |
| 01704 | QUEIJO LANCHE |
| 02108 | QUEIJO MAASDAMER |
| 06108 | QUEIJO MAASDAMER LIGHT |
| 02909 | QUEIJO MANTEIGA |
| 02002 | QUEIJO MINAS |
| 02007 | QUEIJO MINAS CURADO |
| 07307 | QUEIJO MINAS CURADO FRESCAL ORGANICO |
| 06007 | QUEIJO MINAS CURADO LIGHT |
| 03745 | QUEIJO MINAS FRESCAL |
| 07302 | QUEIJO MINAS FRESCAL ORGANICO |
| 06002 | QUEIJO MINAS LIGHT |
| 01804 | QUEIJO MUSSARELA |
| 01808 | QUEIJO MUSSARELA FATIADO |
| 05104 | QUEIJO MUSSARELA LIGHT |
| 01801 | QUEIJO MUZARELLA |
| 05101 | QUEIJO MUZARELLA LIGHT |
| 10001 | QUEIJO MUZZARELA LIGHT |
| 03001 | QUEIJO NAO-ESPECIFICADO |
| 06601 | QUEIJO NAO-ESPECIFICADO LIGHT |
| 01902 | QUEIJO PALMIRA |
| 02404 | QUEIJO PARMESAO |
| 06304 | QUEIJO PARMESAO LIGHT |
| 02405 | QUEIJO PARMESON |
| 06305 | QUEIJO PARMESON LIGHT |
| 02402 | QUEIJO PARMEZAO |
| 06302 | QUEIJO PARMEZAO LIGHT |
| 02401 | QUEIJO PARMEZON |
| 06301 | QUEIJO PARMEZON LIGHT |
| 02803 | QUEIJO PASTEURIZADO |
| 06403 | QUEIJO PASTEURIZADO LIGHT |
| 02804 | QUEIJO PASTEURIZADO POLENGUINHO |
| 06404 | QUEIJO PASTEURIZADO POLENGUINHO LIGHT |
| 02801 | QUEIJO POLENGUINHO |
| 10601 | QUEIJO POLENGUINHO LIGHT |
| 06401 | QUEIJO POLENGUINHO LIGHT |
| 02805 | QUEIJO POLENGUINHO PASTEURIZADO |
| 06405 | QUEIJO POLENGUINHO PASTEURIZADO LIGHT |
| 03744 | QUEIJO PRATO |
| 01701 | QUEIJO PRATO |
| 10201 | QUEIJO PRATO LIGHT |
| 05901 | QUEIJO PRATO LIGHT |
| 02501 | QUEIJO PROVOLONE |
| 02106 | QUEIJO QUARCK |
| 06106 | QUEIJO QUARCK LIGHT |
| 02403 | QUEIJO RALADO |
| 06303 | QUEIJO RALADO LIGHT |
| 02201 | QUEIJO RICOTA |
| 10501 | QUEIJO RICOTA LIGHT |
| 06201 | QUEIJO RICOTA LIGHT |
| 02602 | QUEIJO ROQUEFORT |
| 02102 | QUEIJO SUICO |
| 06102 | QUEIJO SUICO LIGHT |
| 02903 | QUEIJO TIPO CREME |
| 06503 | QUEIJO TIPO CREME LIGHT |
| 02905 | QUEIJO TIPO DANONINHO |
| 06505 | QUEIJO TIPO DANONINHO LIGHT |
| 02013 | QUEIJO TIPO ESTEPE |
| 02105 | QUEIJO TIPO GOLDA |
| 06105 | QUEIJO TIPO GOLDA LIGHT |
| 01702 | QUEIJO TIPO LANCHE |
| 05902 | QUEIJO TIPO LANCHE LIGHT |
| 02006 | QUEIJO TIPO MINAS |
| 07306 | QUEIJO TIPO MINAS FRESCAL ORGANICO |
| 06006 | QUEIJO TIPO MINAS LIGHT |
| 02107 | QUEIJO TIPO QUARCK |
| 06107 | QUEIJO TIPO QUARCK LIGHT |
| 04601 | QUENTAO |
| 00103 | QUENTINHA PARA VIAGEM |
| 01801 | QUERMESSE (INGRESSO) |
| 01701 | QUEROSENE (COMBUSTIVEL DE VEICULO) |
| 00501 | QUEROSENE (COMBUSTIVEL DOMESTICO) |
| 00502 | QUEROSENE (LITRO) (COMBUSTIVEL DOMESTICO) |
| 05601 | QUEROSENE PARA LIMPEZA |
| 05001 | QUIABO |
| 01909 | QUIABO AZEDO (CARURU) |
| 01912 | QUIABO DE ANGOLA (CARURU) |
| 01911 | QUIABO ROSA (CARURU) |
| 01910 | QUIABO ROXO (CARURU) |
| 03728 | QUIBE |
| 00211 | QUIBE PARA VIAGEM |
| 04205 | QUIBE SEMIPRONTO |
| 06701 | QUIBEBE PARA VIAGEM |
| 00508 | QUICARE |
| 06801 | QUICHE PARA VIAGEM |
| 00809 | QUICK |
| 10309 | QUICK DIET |
| 10331 | QUICK DIETETICO |
| 10209 | QUICK LIGHT |
| 00817 | QUICK SHAKE |
| 00816 | QUICK SHAKE CRISTALINO |
| 10316 | QUICK SHAKE CRISTALINO DIET |
| 10338 | QUICK SHAKE CRISTALINO DIETETICO |
| 10216 | QUICK SHAKE CRISTALINO LIGHT |
| 10317 | QUICK SHAKE DIET |
| 10339 | QUICK SHAKE DIETETICO |
| 10217 | QUICK SHAKE LIGHT |
| 05003 | QUIGOMBO |
| 02001 | QUIMIOTERAPIA |
| 04704 | QUIMIOTERAPICO |
| 03001 | QUIMONO |
| 04601 | QUIMONO |
| 03601 | QUIMONO |
| 01601 | QUINA |
| 04105 | QUINDIM |
| 13705 | QUINDIM DIET |
| 13713 | QUINDIM DIETETICO |
| 04901 | QUINOA |
| 02102 | QUIOIO (TEMPERO INDUSTRIALIZADO) |
| 00608 | QUIOSQUES |
| 03502 | QUIRERA (NAO-ESPECIFICADA) |
| 00708 | QUIRERA DE MILHO |
| 00903 | QUIRERA DE TRIGO |
| 03501 | QUIRERA NAO-ESPECIFICADA |
| 00922 | QUITANDAS (PAES, BOLOS, BISCOITOS, ROSCAS, DOCES, ETC) |
| 02607 | QUITUTE BOVINO |
| 04602 | QUITUTE DE FRANGO |
| 04603 | QUITUTE DE FRANGO EM CONSERVA |
| 02706 | QUITUTE SUINO |
| 45101 | RA |
| 45102 | RA CACOTE |
| 45103 | RA COMUM |
| 45501 | RA EM FILE CONGELADO |
| 45301 | RA EM FILE FRESCO |
| 45201 | RA EM POSTAS |
| 45114 | RA GIMBUIA |
| 45112 | RA JIA |
| 45111 | RA MIRIM |
| 45106 | RA NIMBUIA |
| 45110 | RA PAULISTINHA |
| 45107 | RA PIMENTA |
| 45401 | RA SALGADA |
| 45108 | RA TOURO |
| 45105 | RA TOURO GIGANTE |
| 45104 | RA VERDADEIRA |
| 02801 | RABADA BOVINA |
| 07401 | RABANADA |
| 01802 | RABANADA (PAO) |
| 01001 | RABANETE |
| 01403 | RABANETE DE CAVALO (NABICA) |
| 05903 | RABANO PRETO |
| 04802 | RABINHO SUINO |
| 02802 | RABO BOVINO |
| 01801 | RABO DE PORCO SALGADO |
| 04801 | RABO SUINO |
| 06501 | RABO SUINO DEFUMADO |
| 00402 | RACAO DE BOVINO |
| 00102 | RACAO DE CAO |
| 00602 | RACAO DE CAPRINO |
| 01002 | RACAO DE COELHO |
| 00502 | RACAO DE EQUINO |
| 00708 | RACAO DE FRANGO |
| 00703 | RACAO DE GALINHA |
| 00202 | RACAO DE GATO |
| 01202 | RACAO DE OUTRO ANIMAL |
| 00802 | RACAO DE OVINO |
| 00902 | RACAO DE PASSARINHO |
| 00302 | RACAO DE PEIXE |
| 00711 | RACAO DE PINTO |
| 01502 | RACAO DE PORCO |
| 01309 | RACAO NAO-ESPECIFICADA PARA ANIMAL NAO-ESPECIFICADO |
| 00404 | RACAO PARA BOVINO |
| 00104 | RACAO PARA CAO |
| 00604 | RACAO PARA CAPRINO |
| 01004 | RACAO PARA COELHO |
| 00504 | RACAO PARA EQUINO |
| 00709 | RACAO PARA FRANGO |
| 00704 | RACAO PARA GALINHA |
| 00204 | RACAO PARA GATO |
| 01204 | RACAO PARA OUTRO ANIMAL |
| 00804 | RACAO PARA OVINO |
| 00904 | RACAO PARA PASSARINHO |
| 00304 | RACAO PARA PEIXE |
| 01501 | RACAO PARA SUINO |
| 00702 | RACK |
| 01504 | RACUSAI |
| 01601 | RADIO |
| 00810 | RADIO |
| 03501 | RADIO COM CD |
| 03303 | RADIO DE MESA |
| 03003 | RADIO GRAVADOR |
| 03201 | RADIO PORTATIL |
| 03302 | RADIO RELOGIO |
| 03301 | RADIO RELOGIO OU DE MESA |
| 03004 | RADIO TOCA-FITAS |
| 00812 | RADIO TOCA-FITAS |
| 01701 | RADIOGRAFIA |
| 05902 | RADIO-RELOGIO-TELEFONE |
| 01602 | RADITE |
| 00510 | RAIO LASER (APLICACAO) |
| 01206 | RAIO PARA BICICLETA |
| 00837 | RALADOR |
| 00607 | RALADOR DE PE |
| 00804 | RALADOR DE QUEIJO |
| 15604 | RALADORA |
| 01107 | RALO DE ESGOTAMENTO DE AGUA |
| 01107 | RALO DE ESGOTAMENTO DE AGUA |
| 09401 | RALO DE PIA |
| 16501 | RAMAL TELEFONICO |
| 04309 | RAPADURA |
| 00401 | RAPADURA |
| 00406 | RAPADURA COM AMENDOIM |
| 00408 | RAPADURA COM CAJU |
| 00407 | RAPADURA COM CASTANHA |
| 00404 | RAPADURA COM COCO |
| 00405 | RAPADURA COM LEITE |
| 01201 | RAPE |
| 00102 | RAQUETE |
| 02601 | RAQUETE DE MATAR MOSQUITO |
| 01401 | RASPADINHA |
| 00815 | RASPADOR DE COCO |
| 04002 | RASTEIRINHA |
| 02701 | RATICIDA |
| 02901 | RATOEIRA |
| 02802 | RAVIOLE SEMIPRONTO |
| 08009 | RAVIOLI PRONTO LIGHT PARA VIAGEM |
| 00909 | RAVIOLI PRONTO PARA VIAGEM |
| 13521 | REAL LIGHT |
| 05702 | REAL REFRIGERANTE |
| 09302 | REAL REFRIGERANTE LIGHT |
| 00703 | REALCADOR DE SABOR |
| 01642 | REBITADEIRA |
| 01301 | REBOQUE |
| 00512 | RECARGA DE BATERIA |
| 00301 | RECARGA DE BATERIA (ILUMINACAO DOMICILIAR) |
| 05203 | RECARGA DE CARTUCHO PARA IMPRESSORA |
| 21001 | RECARREGADOR DE PILHAS |
| 01402 | RECEBIMENTO DE EMPRESTIMO |
| 02501 | RECEBIMENTO DE PECULIO |
| 09402 | RECEPTOR DE ANTENA PARABOLICA |
| 05703 | RECEPTOR DE SINAL HD |
| 05701 | RECEPTOR DE TV A CABO |
| 07801 | RECHEIO PARA BOLO |
| 00905 | RECIPIENTE DE LIXO |
| 30504 | RECIPIENTE PARA COLETAR FEZES E URINA |
| 00427 | RECONDICIONADOR CAPILAR |
| 03102 | RED BULL |
| 08502 | REDE DE ADULTO |
| 08501 | REDE DE ADULTO E CRIANCA |
| 00704 | REDE DE CABELO |
| 08503 | REDE DE CRIANCA |
| 00513 | REDE DE PESCA |
| 00105 | REDE DE VOLEIBOL |
| 00802 | REDEA DE CAVALO |
| 31108 | REDUTOR DE CELULITE (GEL) |
| 01203 | REDUTOR DE COLESTEROL |
| 01201 | REDUTOR DE COLESTEROL OU TRIGLICERIDEOS |
| 31106 | REDUTOR DE GORDURA (GEL) |
| 01205 | REDUTOR DE TRIGLICERIDEOS |
| 00110 | REEMBOLSO POSTAL |
| 11301 | REFEICAO LIGHT |
| 00101 | REFEICAO PRONTA PARA VIAGEM |
| 02511 | REFIL DE DESINFETANTE SANITARIO |
| 02606 | REFIL INSETICIDA |
| 00712 | REFIL PARA APARELHO DE BARBEAR DESCARTAVEL |
| 00503 | REFIL PARA RODO |
| 14601 | REFORMA DE INSTRUMENTO MUSICAL (MAO-DE-OBRA) |
| 14501 | REFORMA DE INSTRUMENTO MUSICAL (PECA) |
| 14401 | REFORMA DE INSTRUMENTO MUSICAL (PECA+MAO-DE-OBRA) |
| 08101 | REFORMA DE JAZIGO |
| 00302 | REFORMA DE MOVEIS (MAO-DE-OBRA) |
| 00202 | REFORMA DE MOVEIS (PECA) |
| 00102 | REFORMA DE MOVEIS (PECA+MAO-DE-OBRA) |
| 01601 | REFORMA OU OBRA POR EMPREITADA |
| 01601 | REFORMA OU OBRA POR EMPREITADA |
| 02407 | REFRESCO ADOCYL EM PO |
| 11107 | REFRESCO ADOCYL EM PO DIET |
| 11114 | REFRESCO ADOCYL EM PO DIETETICO |
| 11007 | REFRESCO ADOCYL EM PO LIGHT |
| 02410 | REFRESCO ARTIFICIAL EM PO |
| 02703 | REFRESCO DE CAJU |
| 00602 | REFRESCO DE CAJU PARA VIAGEM |
| 02405 | REFRESCO DE FRUTA ARTIFICIAL EM PO |
| 11105 | REFRESCO DE FRUTA ARTIFICIAL EM PO DIET |
| 11112 | REFRESCO DE FRUTA ARTIFICIAL EM PO DIETETICO |
| 11005 | REFRESCO DE FRUTA ARTIFICIAL EM PO LIGHT |
| 03802 | REFRESCO DE FRUTA EM SACO PLASTICO |
| 02302 | REFRESCO DE FRUTA ENCARTONADO |
| 02301 | REFRESCO DE FRUTA ENGARRAFADO |
| 02701 | REFRESCO DE FRUTAS |
| 02704 | REFRESCO DE GROSELHA |
| 00603 | REFRESCO DE GROSELHA PARA VIAGEM |
| 02705 | REFRESCO DE LARANJA |
| 00604 | REFRESCO DE LARANJA PARA VIAGEM |
| 00606 | REFRESCO DE LIMAO PARA VIAGEM |
| 02702 | REFRESCO DE MARACUJA |
| 00605 | REFRESCO DE MARACUJA PARA VIAGEM |
| 02408 | REFRESCO EM PO |
| 03801 | REFRESCO EM SACO PLASTICO |
| 00601 | REFRESCO PARA VIAGEM |
| 01001 | REFRIGERANTE AGUA MINERAL |
| 04901 | REFRIGERANTE AGUA TONICA |
| 08901 | REFRIGERANTE AGUA TONICA DIET |
| 12901 | REFRIGERANTE AGUA TONICA DIET |
| 12904 | REFRIGERANTE AGUA TONICA DIETETICA |
| 08902 | REFRIGERANTE AGUA TONICA DIETETICO |
| 09001 | REFRIGERANTE AGUA TONICA LIGHT |
| 01401 | REFRIGERANTE ALOA |
| 13511 | REFRIGERANTE ALOA LIGHT |
| 08401 | REFRIGERANTE ALOA LIGHT |
| 01201 | REFRIGERANTE BIDU |
| 13507 | REFRIGERANTE BIDU LIGHT |
| 08201 | REFRIGERANTE BIDU LIGHT |
| 06701 | REFRIGERANTE CITRICO |
| 13522 | REFRIGERANTE CITRICO LIGHT |
| 09401 | REFRIGERANTE CITRICO LIGHT |
| 00103 | REFRIGERANTE COCA COLA |
| 12902 | REFRIGERANTE COCA COLA LIGHT |
| 00703 | REFRIGERANTE COCA COLA LIGHT |
| 13403 | REFRIGERANTE COCA COLA ZERO |
| 00203 | REFRIGERANTE CRUSH |
| 13004 | REFRIGERANTE CRUSH LIGHT |
| 01503 | REFRIGERANTE CRUSH LIGHT |
| 15501 | REFRIGERANTE DE ABACATE |
| 06801 | REFRIGERANTE DE ABACAXI |
| 06901 | REFRIGERANTE DE ABACAXI COM GUARANA |
| 09601 | REFRIGERANTE DE ABACAXI COM GUARANA LIGHT |
| 13525 | REFRIGERANTE DE ABACAXI LIGHT |
| 09501 | REFRIGERANTE DE ABACAXI LIGHT |
| 04601 | REFRIGERANTE DE CAJU |
| 13515 | REFRIGERANTE DE CAJU LIGHT |
| 08901 | REFRIGERANTE DE CAJU LIGHT |
| 00601 | REFRIGERANTE DE COLA |
| 00101 | REFRIGERANTE DE COLA |
| 09001 | REFRIGERANTE DE COLA DIET |
| 00801 | REFRIGERANTE DE COLA DIET |
| 09004 | REFRIGERANTE DE COLA DIETETICO |
| 00806 | REFRIGERANTE DE COLA DIETETICO |
| 12901 | REFRIGERANTE DE COLA LIGHT |
| 00701 | REFRIGERANTE DE COLA LIGHT |
| 07001 | REFRIGERANTE DE FRAMBOESA |
| 13526 | REFRIGERANTE DE FRAMBOESA LIGHT |
| 09701 | REFRIGERANTE DE FRAMBOESA LIGHT |
| 07301 | REFRIGERANTE DE GENGIBRE |
| 14501 | REFRIGERANTE DE GENGIBRE DIET |
| 13529 | REFRIGERANTE DE GENGIBRE LIGHT |
| 10001 | REFRIGERANTE DE GENGIBRE LIGHT |
| 02301 | REFRIGERANTE DE GUARANA |
| 00301 | REFRIGERANTE DE GUARANA |
| 09101 | REFRIGERANTE DE GUARANA DIET |
| 07401 | REFRIGERANTE DE GUARANA DIET |
| 09103 | REFRIGERANTE DE GUARANA DIETETICO |
| 07403 | REFRIGERANTE DE GUARANA DIETETICO |
| 13101 | REFRIGERANTE DE GUARANA LIGHT |
| 01601 | REFRIGERANTE DE GUARANA LIGHT |
| 07405 | REFRIGERANTE DE GUARANA ZERO |
| 05101 | REFRIGERANTE DE LARANJA |
| 00201 | REFRIGERANTE DE LARANJA |
| 15601 | REFRIGERANTE DE LARANJA DIET |
| 13001 | REFRIGERANTE DE LARANJA LIGHT |
| 01501 | REFRIGERANTE DE LARANJA LIGHT |
| 05201 | REFRIGERANTE DE LIMAO |
| 00401 | REFRIGERANTE DE LIMAO |
| 09201 | REFRIGERANTE DE LIMAO DIET |
| 07601 | REFRIGERANTE DE LIMAO DIET |
| 09204 | REFRIGERANTE DE LIMAO DIETETICO |
| 07604 | REFRIGERANTE DE LIMAO DIETETICO |
| 13301 | REFRIGERANTE DE LIMAO LIGHT |
| 07501 | REFRIGERANTE DE LIMAO LIGHT |
| 04101 | REFRIGERANTE DE MACA |
| 13201 | REFRIGERANTE DE MACA LIGHT |
| 04001 | REFRIGERANTE DE MACA LIGHT |
| 07201 | REFRIGERANTE DE MARACUJA |
| 13528 | REFRIGERANTE DE MARACUJA LIGHT |
| 09901 | REFRIGERANTE DE MARACUJA LIGHT |
| 01101 | REFRIGERANTE DE MATE |
| 13504 | REFRIGERANTE DE MATE LIGHT |
| 08101 | REFRIGERANTE DE MATE LIGHT |
| 07101 | REFRIGERANTE DE MORANGO |
| 13527 | REFRIGERANTE DE MORANGO LIGHT |
| 09801 | REFRIGERANTE DE MORANGO LIGHT |
| 04903 | REFRIGERANTE DE QUININO |
| 12903 | REFRIGERANTE DE QUININO DIET |
| 12906 | REFRIGERANTE DE QUININO DIETETICO |
| 09003 | REFRIGERANTE DE QUININO LIGHT |
| 05301 | REFRIGERANTE DE TANGERINA |
| 04301 | REFRIGERANTE DE TANGERINA |
| 13514 | REFRIGERANTE DE TANGERINA LIGHT |
| 08801 | REFRIGERANTE DE TANGERINA LIGHT |
| 04201 | REFRIGERANTE DE TUTI FRUTI |
| 13513 | REFRIGERANTE DE TUTI FRUTI LIGHT |
| 08701 | REFRIGERANTE DE TUTI FRUTI LIGHT |
| 05001 | REFRIGERANTE DE UVA |
| 00501 | REFRIGERANTE DE UVA |
| 13401 | REFRIGERANTE DE UVA LIGHT |
| 07701 | REFRIGERANTE DE UVA LIGHT |
| 05801 | REFRIGERANTE ENERGETICO |
| 13523 | REFRIGERANTE FANTA CITRUS LIGHT |
| 13002 | REFRIGERANTE FANTA LARANJA LIGHT |
| 00408 | REFRIGERANTE FANTA LIMAO |
| 13304 | REFRIGERANTE FANTA LIMAO LIGHT |
| 07506 | REFRIGERANTE FANTA LIMAO LIGHT |
| 13202 | REFRIGERANTE FANTA MACA LIGHT |
| 00502 | REFRIGERANTE FANTA UVA |
| 13402 | REFRIGERANTE FANTA UVA LIGHT |
| 07702 | REFRIGERANTE FANTA UVA LIGHT |
| 13104 | REFRIGERANTE FLATELLY VITA LIGHT |
| 00303 | REFRIGERANTE FRATELLY VITA |
| 01603 | REFRIGERANTE FRATELLY VITA LIGHT |
| 00409 | REFRIGERANTE GASOSA |
| 13306 | REFRIGERANTE GASOSA LIGHT |
| 07507 | REFRIGERANTE GASOSA LIGHT |
| 05501 | REFRIGERANTE GOIANINHA |
| 15001 | REFRIGERANTE GOIANINHA DIET |
| 13518 | REFRIGERANTE GOIANINHA LIGHT |
| 09201 | REFRIGERANTE GOIANINHA LIGHT |
| 00503 | REFRIGERANTE GRAPETE |
| 07703 | REFRIGERANTE GRAPETE LIGHT |
| 13404 | REFRIGERANTE GRAPETTE LIGHT |
| 00307 | REFRIGERANTE GUARANA |
| 13102 | REFRIGERANTE GUARANA LIGHT |
| 01301 | REFRIGERANTE JAO |
| 13509 | REFRIGERANTE JAO LIGHT |
| 08301 | REFRIGERANTE JAO LIGHT |
| 14701 | REFRIGERANTE JESUS |
| 00405 | REFRIGERANTE LIMAO |
| 07505 | REFRIGERANTE LIMAO LIGHT |
| 01102 | REFRIGERANTE MATE COURO |
| 13505 | REFRIGERANTE MATE COURO LIGHT |
| 08102 | REFRIGERANTE MATE COURO LIGHT |
| 01002 | REFRIGERANTE MINALBA |
| 00601 | REFRIGERANTE MINEIRINHO |
| 07801 | REFRIGERANTE MINEIRINHO LIGHT |
| 00901 | REFRIGERANTE MINUANO |
| 13502 | REFRIGERANTE MINUANO LIGHT |
| 07901 | REFRIGERANTE MINUANO LIGHT |
| 00205 | REFRIGERANTE MIRINDA |
| 13006 | REFRIGERANTE MIRINDA LIGHT |
| 01505 | REFRIGERANTE MIRINDA LIGHT |
| 13901 | REFRIGERANTE NAO ESPECIFICADO |
| 03501 | REFRIGERANTE NAO-ESPECIFICADO |
| 14001 | REFRIGERANTE NAO-ESPECIFICADO DIET |
| 14101 | REFRIGERANTE NAO-ESPECIFICADO LIGHT |
| 05401 | REFRIGERANTE PARAGUAI |
| 13516 | REFRIGERANTE PARAGUAI LIGHT |
| 09101 | REFRIGERANTE PARAGUAI LIGHT |
| 00104 | REFRIGERANTE PEPSI |
| 00107 | REFRIGERANTE PEPSI COLA |
| 09002 | REFRIGERANTE PEPSI COLA DIET |
| 00805 | REFRIGERANTE PEPSI COLA DIET |
| 09005 | REFRIGERANTE PEPSI COLA DIETETICO |
| 00810 | REFRIGERANTE PEPSI COLA DIETETICO |
| 00802 | REFRIGERANTE PEPSI DIET |
| 00807 | REFRIGERANTE PEPSI DIETETICO |
| 00209 | REFRIGERANTE POP LARANJA |
| 13010 | REFRIGERANTE POP LARANJA LIGHT |
| 01509 | REFRIGERANTE POP LARANJA LIGHT |
| 05701 | REFRIGERANTE REAL |
| 13520 | REFRIGERANTE REAL LIGHT |
| 09301 | REFRIGERANTE REAL LIGHT |
| 00402 | REFRIGERANTE SODA LIMONADA |
| 13302 | REFRIGERANTE SODA LIMONADA LIGHT |
| 07502 | REFRIGERANTE SODA LIMONADA LIGHT |
| 00406 | REFRIGERANTE SPRIT |
| 09202 | REFRIGERANTE SPRIT DIET |
| 07602 | REFRIGERANTE SPRIT DIET |
| 09205 | REFRIGERANTE SPRIT DIETETICO |
| 07605 | REFRIGERANTE SPRIT DIETETICO |
| 00207 | REFRIGERANTE SUKITA |
| 13008 | REFRIGERANTE SUKITA LIGHT |
| 01507 | REFRIGERANTE SUKITA LIGHT |
| 01801 | REFRIGERANTE TUBAINA |
| 13108 | REFRIGERANTE TUBAINA LIGHT |
| 01901 | REFRIGERANTE TUBAINA LIGHT |
| 01701 | REFRIGERANTE XODO DA BAHIA |
| 13106 | REFRIGERANTE XODO DA BAHIA LIGHT |
| 08501 | REFRIGERANTE XODO DA BAHIA LIGHT |
| 13501 | REFRIGERANTES DE OUTROS SABORES LIGHT |
| 00701 | REGISTRO DE ARMA |
| 04303 | REGISTRO DE BOTIJAO DE GAS |
| 04201 | REGISTRO DE FOGAO |
| 00102 | REGISTRO DE IMOVEL |
| 00220 | REGUA |
| 05601 | REGULADOR DE VOLTAGEM |
| 02802 | REJUNTE |
| 02802 | REJUNTE |
| 00213 | RELAXAMENTO (CABELEIREIRO) |
| 04405 | RELAXANTE MUSCULAR |
| 00801 | RELOGIO DE BOLSO |
| 04305 | RELOGIO DE BOTIJAO DE GAS |
| 01101 | RELOGIO DE DEDO |
| 04202 | RELOGIO DE FOGAO |
| 19402 | RELOGIO DE MESA |
| 19401 | RELOGIO DE MESA OU PAREDE |
| 19403 | RELOGIO DE PAREDE |
| 00201 | RELOGIO DE PULSO DE CRIANCA |
| 00301 | RELOGIO DE PULSO DE HOMEM |
| 00401 | RELOGIO DE PULSO DE MULHER |
| 16101 | RELOGIO DESPERTADOR |
| 01301 | RELOJOEIRO |
| 02803 | REMEDIO GINECOLOGICO |
| 30010 | REMEDIO HOMEOPATICO INDETERMINADO |
| 30001 | REMEDIO INDETERMINADO |
| 30012 | REMEDIO MANIPULADO INDETERMINADO |
| 31109 | REMEDIO MODELADOR DE CORPO (GEL) |
| 30011 | REMEDIO ORTOMOLECULAR |
| 02405 | REMEDIO PARA ANIMAL DOMESTICO |
| 30703 | REMEDIO PARA CABELOS (SHAMPU E OUTROS) |
| 04504 | REMEDIO PARA PIOLHO (SABONETE) |
| 30704 | REMEDIO PARA QUEDA DE CABELOS (SHAMPU, CREME E OUTROS) |
| 03402 | REMEDIO PARA VARIZES |
| 01502 | REMO (APARELHO DE GINASTICA) |
| 01501 | REMO SECO (APARELHO DE GINASTICA) |
| 01505 | REMOVEDOR |
| 00608 | REMOVEDOR DE CUTICULA |
| 01805 | REMOVEDOR DE GORDURA |
| 01803 | REMOVEDOR DE LIMO |
| 02410 | REMOVEDOR DE MANCHA DE ROUPA |
| 02402 | RENAL (PARA OS RINS) |
| 00106 | RENDA |
| 03401 | RENDIMENTO DE MENORES DE DEZ ANOS |
| 03301 | RENDIMENTO DE MORADOR AUSENTE |
| 00102 | RENDIMENTO DO PIS/PASEP |
| 00101 | RENDIMENTO E ABONO DO PIS/PASEP |
| 02504 | RENOVACAO DE CHEQUE ESPECIAL |
| 02602 | REPARADOR DE PONTAS |
| 00510 | REPARO DE ARRANHOES DE PINTURA |
| 02603 | REPELENTE |
| 06802 | REPELENTE ELETRICO DE INSETO |
| 00901 | REPOLHO |
| 00902 | REPOLHO BRANCO |
| 00901 | REPOLHO EM CONSERVA |
| 00905 | REPOLHO LISO |
| 10401 | REPOLHO ORGANICO |
| 00903 | REPOLHO ROXO |
| 00904 | REPOLHO VERDE |
| 04302 | REPOSITOR ELETROLITICO (CLORETO DE POTASSIO, SODIO, ETC.) |
| 03717 | REQUEIJAO |
| 02901 | REQUEIJAO |
| 02908 | REQUEIJAO CATUPIRI |
| 06508 | REQUEIJAO CATUPIRI LIGHT |
| 02907 | REQUEIJAO CREMOSO |
| 06507 | REQUEIJAO CREMOSO LIGHT |
| 06501 | REQUEIJAO LIGHT |
| 04002 | RESCISAO CONTRATUAL |
| 00505 | RESCISAO DE CONTRATO DE ALUGUEL DE OUTROS IMOVEIS |
| 00303 | RESGATE DE ACOES |
| 04402 | RESGATE DE TITULO DE CAPITALIZACAO |
| 01501 | RESSONANCIA MAGNETICA |
| 00413 | RESTAURADOR CAPILAR |
| 00603 | RESTAURANTES |
| 03301 | RESTITUICAO DA PREVIDENCIA PRIVADA |
| 02301 | RESTITUICAO DA PREVIDENCIA PUBLICA |
| 05101 | RESTITUICAO DE CONVENIO DE SAUDE |
| 01201 | RESTITUICAO DE DINHEIRO NA COMPRA POR CARNET |
| 05201 | RESTITUICAO DE MULTA DE TRANSITO |
| 03001 | RESTITUICAO DE PENSAO ALIMENTICIA |
| 05103 | RESTITUICAO DE PLANO DE SAUDE |
| 03403 | RESTITUICAO DE SEGURO-HABITACAO |
| 03404 | RESTITUICAO DE TAXA DE CONSERVACAO DE IMOVEL |
| 00401 | RESTITUICAO DO IMPOSTO DE RENDA |
| 02701 | RESTITUICAO JUDICIAL |
| 03603 | RETALHO DE APRESUNTADO |
| 01007 | RETALHO DE BACON |
| 02606 | RETALHO DE MORTADELA |
| 05006 | RETALHO DE MORTADELA LIGHT |
| 01806 | RETALHO DE MUSSARELA |
| 05106 | RETALHO DE MUSSARELA LIGHT |
| 02301 | RETALHO PARA PIZZA |
| 01904 | RETIRADA DE ENTULHO |
| 01904 | RETIRADA DE ENTULHO |
| 09501 | RETROPROJETOR |
| 00802 | RETROVISOR |
| 00205 | REUNIAO DE CULTO RELIGIOSO |
| 00803 | REVELACAO DE FILME |
| 00801 | REVELACAO E COPIA |
| 01201 | REVESTIMENTO DE TABUA DE PASSAR ROUPA |
| 04201 | REVISAO CONTRATUAL |
| 09301 | REVISAO DE TESES, DISSERTACOES E TRABALHOS ESCOLARES |
| 01401 | REVISTA DE ANIMAIS |
| 02001 | REVISTA DE ARTESANATO |
| 01501 | REVISTA DE CARROS |
| 01201 | REVISTA DE CASA E DECORACAO |
| 00701 | REVISTA DE CULINARIA |
| 00901 | REVISTA DE INFORMATICA |
| 00501 | REVISTA DE MODA |
| 01601 | REVISTA DE SAUDE |
| 01301 | REVISTA DE TV |
| 00401 | REVISTA ESPORTIVA |
| 01001 | REVISTA FEMININA |
| 00201 | REVISTA INFANTIL |
| 00801 | REVISTA INFORMATIVA |
| 00601 | REVISTA MASCULINA |
| 02101 | REVISTA RELIGIOSA |
| 00803 | REVISTA TECNICA |
| 01101 | REVISTA TEEN |
| 00403 | REVOLVER (EXCETO DE CACA) |
| 00901 | RIFA |
| 00406 | RIFLE (EXCETO DE CACA) |
| 00504 | RIFLE DE CACA |
| 02001 | RIM BOVINO |
| 09601 | RIM DE BODE |
| 04701 | RIM DE GALINHA |
| 09901 | RIM NAO-ESPECIFICADO |
| 09701 | RIM SUINO |
| 03501 | RINHA (INGRESSO) |
| 02701 | RIO DE PREMIOS |
| 00404 | RIPA |
| 00404 | RIPA |
| 07704 | RIPA DE MADEIRA CERTIFICADA (SELO FSC) |
| 08604 | RIPA DE MADEIRA CERTIFICADA (SELO FSC) |
| 01621 | RISCADOR |
| 03707 | RISOLE |
| 02701 | RISOTO PARA VIAGEM |
| 00207 | RIZOLE PARA VIAGEM |
| 03304 | RIZOTO CAMPONES PRE-COZIDO |
| 09301 | RIZOTO PARA VIAGEM |
| 03303 | RIZOTO PRE-COZIDO |
| 03305 | RIZOTO PRIMAVERA PRE-COZIDO |
| 01302 | ROBE |
| 00703 | ROBE |
| 00803 | ROBE |
| 02502 | ROCAMBOLE |
| 10302 | ROCAMBOLE DIET |
| 10306 | ROCAMBOLE DIETETICO |
| 10202 | ROCAMBOLE LIGHT |
| 00103 | RODAPE (EXCETO DE MADEIRA) |
| 00103 | RODAPE (EXCETO DE MADEIRA) |
| 03601 | RODEIO (INGRESSO) |
| 02608 | RODEIOS (INGRESSO) |
| 05402 | RODIZIO DE CARNES |
| 05602 | RODIZIO DE FRUTOS DO MAR |
| 05502 | RODIZIO DE MASSAS |
| 00501 | RODO |
| 01110 | ROLAMENTO PARA PORTA |
| 01110 | ROLAMENTO PARA PORTA |
| 05101 | ROLE DE PERU |
| 01503 | ROLESO |
| 07104 | ROLETA |
| 00905 | ROLO DE PINTURA |
| 00905 | ROLO DE PINTURA |
| 01302 | ROLOPAC |
| 07601 | ROMA |
| 00404 | ROMANCE |
| 02004 | ROQUETE |
| 00102 | ROSA |
| 04701 | ROSBIFE E BIFE A MILANEZA INDUSTRIALIZADO SEMIPRONTO |
| 02013 | ROSCA AMANTEIGADA |
| 02103 | ROSCA DE BATATA |
| 02014 | ROSCA DE CHOCOLATE |
| 02010 | ROSCA DE COCO |
| 02012 | ROSCA DE GLUTEN DOCE |
| 02107 | ROSCA DE GLUTEN SALGADA |
| 02006 | ROSCA DE MILHO |
| 02005 | ROSCA DE NATA |
| 06001 | ROSCA DE POLVILHO NAO-ESPECIFICADA |
| 02004 | ROSCA DE TRIGO DOCE |
| 05903 | ROSCA DE TRIGO NAO-ESPECIFICADA |
| 09803 | ROSCA DE TRIGO NAO-ESPECIFICADA DIET |
| 09806 | ROSCA DE TRIGO NAO-ESPECIFICADA DIETETICA |
| 09703 | ROSCA DE TRIGO NAO-ESPECIFICADA LIGHT |
| 02104 | ROSCA DE TRIGO SALGADA |
| 08401 | ROSCA DIET |
| 08402 | ROSCA DIETETICA |
| 02001 | ROSCA DOCE |
| 02009 | ROSCA DOCE DE TRIGO |
| 11401 | ROSCA DOCE LIGHT |
| 12001 | ROSCA LIGHT |
| 05901 | ROSCA NAO-ESPECIFICADA |
| 09801 | ROSCA NAO-ESPECIFICADA DIET |
| 09804 | ROSCA NAO-ESPECIFICADA DIETETICA |
| 09701 | ROSCA NAO-ESPECIFICADA LIGHT |
| 04901 | ROSCA NAO-ESPECIFICADA PARA VIAGEM |
| 04813 | ROSCA RECHEADA |
| 08413 | ROSCA RECHEADA DIET |
| 08426 | ROSCA RECHEADA DIETETICA |
| 08313 | ROSCA RECHEADA LIGHT |
| 02101 | ROSCA SALGADA |
| 02102 | ROSCA SALGADA DE TRIGO |
| 01905 | ROSELA |
| 02007 | ROSQUINHA AMANTEIGADA |
| 02105 | ROSQUINHA DE BATATA |
| 02011 | ROSQUINHA DE COCO |
| 02008 | ROSQUINHA DE MILHO |
| 02003 | ROSQUINHA DE NATA |
| 06002 | ROSQUINHA DE POLVILHO NAO-ESPECIFICADA |
| 02002 | ROSQUINHA DOCE |
| 05902 | ROSQUINHA NAO-ESPECIFICADA |
| 09802 | ROSQUINHA NAO-ESPECIFICADA DIET |
| 09805 | ROSQUINHA NAO-ESPECIFICADA DIETETICA |
| 09702 | ROSQUINHA NAO-ESPECIFICADA LIGHT |
| 04808 | ROSQUINHA RECHEADA DE QUALQUER SABOR |
| 08408 | ROSQUINHA RECHEADA DE QUALQUER SABOR DIET |
| 08421 | ROSQUINHA RECHEADA DE QUALQUER SABOR DIETETICA |
| 08308 | ROSQUINHA RECHEADA DE QUALQUER SABOR LIGHT |
| 02106 | ROSQUINHA SALGADA |
| 02302 | ROTARY CLUBE |
| 04302 | ROTOLONE SEMIPRONTO |
| 00604 | ROUPA DE BALE |
| 00301 | ROUPA DE BANHO |
| 00401 | ROUPA DE CAMA |
| 00602 | ROUPA DE GINASTICA |
| 00701 | ROUPA DE GINASTICA |
| 00601 | ROUPA DE MESA DE PLASTICO |
| 00501 | ROUPA DE MESA DE TECIDO |
| 01303 | ROUPAO |
| 01304 | ROUPAO DE BANHO |
| 00704 | ROUPAO DE BANHO |
| 00804 | ROUPAO DE BANHO |
| 02002 | RUCOLA |
| 02001 | RUCULA |
| 09801 | RUCULA ORGANICA |
| 06601 | RUFO DE TELHADO |
| 07001 | RUFO DE TELHADO |
| 06602 | RUFO PARA TELHADO |
| 07002 | RUFO PARA TELHADO |
| 00701 | RUM |
| 02804 | RUNCHO |
| 02104 | SABAO DE BOLA |
| 02101 | SABAO DE COCO |
| 02103 | SABAO EM BARRA |
| 02105 | SABAO EM BOLA |
| 02107 | SABAO EM PASTA |
| 02102 | SABAO EM PEDRA |
| 02201 | SABAO EM PO |
| 07702 | SABAO EM PO DE MAQUINA DE LAVAR LOUCA |
| 01901 | SABAO LIQUIDO |
| 04501 | SABAO NAO-ESPECIFICADO |
| 02001 | SABAO PASTOSO |
| 02106 | SABAO RALADO |
| 00301 | SABONETE |
| 00305 | SABONETE ANTIALERGICO |
| 04503 | SABONETE ANTI-PIOLHO |
| 01002 | SABONETE DE LIMPEZA DE PELE |
| 30701 | SABONETE E SHAMPU MEDICINAL |
| 00303 | SABONETE EM GEL |
| 00302 | SABONETE LIQUIDO |
| 01003 | SABONETE LIQUIDO DE LIMPEZA DE PELE |
| 30705 | SABONETE MEDICINAL |
| 00207 | SABONETEIRA (EXCETO DE PLASTICO) |
| 00207 | SABONETEIRA (EXCETO DE PLASTICO) |
| 00925 | SABONETEIRA PLASTICA |
| 14602 | SABORIZADOR DE LEITE |
| 01804 | SACARINA |
| 02502 | SACHET |
| 01203 | SACO DE COMPRA |
| 02004 | SACO DE DORMIR |
| 07202 | SACO DE LANCHE DE PLASTICO OU PAPEL |
| 00802 | SACO DE LIMPEZA DE CHAO |
| 01101 | SACO DE LIXO DE QUALQUER MATERIAL |
| 01304 | SACO PLASTICO PARA EMBALAGEM |
| 00601 | SACOLA COBAL |
| 03601 | SACOLA DE BEBE |
| 01201 | SACOLA DE COMPRA |
| 01102 | SACOLA DE LIXO |
| 03701 | SACOLA DE VIAGEM |
| 02602 | SACOLA PARA ROUPA |
| 00801 | SACOLAO |
| 04505 | SACOLE |
| 04803 | SACOLE (PICOLE ENSACADO) |
| 11403 | SACOLE (PICOLE ENSACADO) LIGHT |
| 01505 | SAGU DE MANDIOCA |
| 01515 | SAGU DE TAPIOCA |
| 01301 | SAIA |
| 01601 | SAIA |
| 04901 | SAIA-CALCA |
| 02204 | SAIBRO |
| 02204 | SAIBRO |
| 02101 | SAIDA DE PRAIA |
| 02301 | SAIDA DE PRAIA |
| 01102 | SAIS DE BANHO |
| 03606 | SAKURO |
| 10506 | SAKURO LIGHT |
| 08501 | SAL AMARGO |
| 00301 | SAL AMONIACO |
| 05701 | SAL DIET |
| 05702 | SAL DIETETICO |
| 00201 | SAL GROSSO |
| 00204 | SAL GROSSO TEMPERADO |
| 00102 | SAL IODADO |
| 05703 | SAL IODADO DIET |
| 05704 | SAL IODADO DIETETICO |
| 11002 | SAL IODADO LIGHT |
| 00203 | SAL MOIDO |
| 00101 | SAL REFINADO |
| 11001 | SAL REFINADO LIGHT |
| 00202 | SAL TRITURADO |
| 00503 | SALA DE INTERNET |
| 08402 | SALADA DE FOLHAS (VERDURAS) PARA VIAGEM |
| 01501 | SALADA DE FRUTAS |
| 01305 | SALADA DE FRUTAS EM CALDA |
| 04401 | SALADA DE FRUTAS PARA VIAGEM |
| 08403 | SALADA DE LEGUMES PARA VIAGEM |
| 00802 | SALADA DE MAIONESE PARA VIAGEM |
| 00801 | SALADA OU VERDURA COZIDA, EXCETO DE FRUTA PARA VIAGEM |
| 08401 | SALADA OU VERDURA CRUA (EXCETO DE FRUTA) PARA VIAGEM |
| 01503 | SALADEIRA |
| 02701 | SALAME |
| 02703 | SALAME DEFUMADO |
| 12301 | SALAME LIGHT |
| 05401 | SALAME LIGHT |
| 02702 | SALAMINHO |
| 05402 | SALAMINHO LIGHT |
| 01702 | SALARIO-DESEMPREGO |
| 02603 | SALARIO-EDUCACAO |
| 09201 | SALEIRO |
| 02221 | SALGADINHO (BISCOITO) |
| 10121 | SALGADINHO (BISCOITO) LIGHT |
| 02246 | SALGADINHO DE MILHO |
| 05301 | SALGADINHO DE PELE (PACOTE) PARA VIAGEM |
| 02247 | SALGADINHO DE QUEIJO |
| 00201 | SALGADINHO PARA VIAGEM |
| 04601 | SALGADINHO SEMIPRONTO PARA VIAGEM |
| 04112 | SALGADO E CERVEJA |
| 04116 | SALGADO E REFRESCO |
| 04108 | SALGADO E REFRIGERANTE |
| 04102 | SALGADO E SUCO |
| 04114 | SALGADO E VITAMINA |
| 11201 | SALGADO LIGHT |
| 03701 | SALGADOS OU TIRA-GOSTO |
| 00403 | SALHO |
| 31103 | SALICILATO, IODO (PARA MASSAGEM) |
| 06001 | SALMAO EM CONSERVA |
| 04801 | SALPICAO PARA VIAGEM |
| 01001 | SALSA |
| 06901 | SALSA DESIDRATADA |
| 01202 | SALSA E CEBOLINHA |
| 09001 | SALSA ORGANICA |
| 02605 | SALSA ROMANA (AIPO) |
| 02604 | SALSAO (AIPO) |
| 02801 | SALSICHA EM CONSERVA |
| 02102 | SALSICHA EM PACOTE |
| 05202 | SALSICHA EM PACOTE LIGHT |
| 02101 | SALSICHA NO VAREJO |
| 05201 | SALSICHA NO VAREJO LIGHT |
| 07501 | SALSICHA VEGETAL EM CONSERVA |
| 03726 | SALSICHAO |
| 04702 | SALSICHAO ASSADO PARA VIAGEM |
| 02104 | SALSICHAO EM PACOTE |
| 05204 | SALSICHAO EM PACOTE LIGHT |
| 02103 | SALSICHAO NO VAREJO |
| 05203 | SALSICHAO NO VAREJO LIGHT |
| 04701 | SALSICHAO PARA VIAGEM |
| 01002 | SALSINHA |
| 01003 | SALSINHA VERDE |
| 00405 | SALTO DE SAPATO |
| 02801 | SALVA-VIDAS |
| 08601 | SALVIA EM FOLHA (TEMPERO INDUSTRIALIZADO) |
| 02401 | SAMAMBAIA DO CAMPO |
| 04801 | SAMBIQUIRA DE GALINHA OU FRANGO |
| 04901 | SAMBIQUIRA DE PERU |
| 05001 | SAMBIQUIRA NAO-ESPECIFICADA |
| 00515 | SAMBURA |
| 01801 | SAN RAFAEL |
| 04001 | SANDALIA (NAO-ESPECIFICADA) |
| 06501 | SANDALIA DE BORRACHA (NAO-ESPECIFICADA) |
| 00901 | SANDALIA DE BORRACHA DE CRIANCA |
| 04701 | SANDALIA DE BORRACHA DE HOMEM |
| 04901 | SANDALIA DE BORRACHA DE MULHER |
| 00601 | SANDALIA DE CRIANCA (EXCETO DE BORRACHA OU PLASTICO) |
| 00701 | SANDALIA DE HOMEM (EXCETO DE BORRACHA OU PLASTICO) |
| 00801 | SANDALIA DE MULHER (EXCETO DE BORRACHA OU PLASTICO) |
| 04801 | SANDALIA DE PLASTICO (NAO-ESPECIFICADA) |
| 01003 | SANDALIA DE PLASTICO DE CRIANCA |
| 03203 | SANDALIA DE PLASTICO DE HOMEM |
| 01103 | SANDALIA DE PLASTICO DE MULHER |
| 01001 | SANDALIA E SAPATO DE PLASTICO DE CRIANCA |
| 03201 | SANDALIA E SAPATO DE PLASTICO DE HOMEM |
| 01101 | SANDALIA E SAPATO DE PLASTICO DE MULHER |
| 04110 | SANDALIA ORTOPEDICA |
| 00401 | SANDUICHE |
| 00416 | SANDUICHE DE MORTADELA |
| 00319 | SANDUICHE DE MORTADELA PARA VIAGEM |
| 00414 | SANDUICHE DE PRESUNTO |
| 00317 | SANDUICHE DE PRESUNTO PARA VIAGEM |
| 00417 | SANDUICHE DE QUEIJO MINAS |
| 00320 | SANDUICHE DE QUEIJO MINAS PARA VIAGEM |
| 00412 | SANDUICHE DE QUEIJO PRATO |
| 00415 | SANDUICHE DE QUEIJO PRATO COM PRESUNTO |
| 00318 | SANDUICHE DE QUEIJO PRATO COM PRESUNTO PARA VIAGEM |
| 00315 | SANDUICHE DE QUEIJO PRATO PARA VIAGEM |
| 00413 | SANDUICHE DE SALAME |
| 00316 | SANDUICHE DE SALAME PARA VIAGEM |
| 04115 | SANDUICHE E REFRESCO |
| 04107 | SANDUICHE E REFRIGERANTE |
| 04104 | SANDUICHE E SUCO |
| 03901 | SANDUICHE NATURAL |
| 00323 | SANDUICHE NATURAL |
| 00301 | SANDUICHE PARA VIAGEM |
| 04110 | SANDUICHE, BATATA FRITA E REFRIGERANTE |
| 04111 | SANDUICHE, SALGADO E REFRIGERANTE |
| 08901 | SANDUICHEIRA ELETRICA |
| 12301 | SANGUE BOVINO |
| 05102 | SANGUE COAGULADO SUINO |
| 09301 | SANGUE DE CARNEIRO |
| 02102 | SANGUE HUMANO |
| 05101 | SANGUE SUINO |
| 05104 | SANGUE SUINO COAGULADO |
| 03602 | SAPATARIA (MOVEL PARA SAPATOS) |
| 03101 | SAPATEIRA |
| 67102 | SAPATEIRA (LAGOSTA) |
| 00401 | SAPATEIRO |
| 07101 | SAPATENIS |
| 03001 | SAPATILHA |
| 03002 | SAPATILHA DE BALE |
| 03003 | SAPATILHA DE GINASTICA |
| 02401 | SAPATINHO DE BEBE (EXCETO DE LA) |
| 01809 | SAPATINHO DE LA OU LINHA |
| 04101 | SAPATO (NAO-ESPECIFICADO) |
| 01203 | SAPATO DE CRIANCA (EXCETO DE PLASTICO) |
| 01303 | SAPATO DE HOMEM (EXCETO DE PLASTICO) |
| 01403 | SAPATO DE MULHER (EXCETO DE PLASTICO) |
| 01002 | SAPATO DE PLASTICO DE CRIANCA |
| 03202 | SAPATO DE PLASTICO DE HOMEM |
| 01102 | SAPATO DE PLASTICO DE MULHER |
| 01201 | SAPATO E BOTA DE CRIANCA (EXCETO DE PLASTICO) |
| 01301 | SAPATO E BOTA DE HOMEM (EXCETO DE PLASTICO) |
| 01401 | SAPATO E BOTA DE MULHER (EXCETO DE PLASTICO) |
| 04103 | SAPATO ORTOPEDICO |
| 05702 | SAPE PARA COBERTURA |
| 05702 | SAPE PARA COBERTURA |
| 02302 | SAPOLEO |
| 02304 | SAPOLIO |
| 02301 | SAPONACEO |
| 06002 | SAPOTA |
| 06001 | SAPOTI |
| 02302 | SAQUE |
| 06201 | SAQUE DO FGTS |
| 00201 | SAQUE DO PIS/PASEP (TOTAL) |
| 03902 | SAQUE ORGANICO |
| 00202 | SAQUE TOTAL DO PIS/PASEP |
| 07201 | SAQUINHO DE LANCHE DE PLASTICO OU PAPEL |
| 01402 | SARAMAGO |
| 03907 | SARAPATEL SUINO FRESCO |
| 03002 | SARDINHA EM CONSERVA |
| 06202 | SARDINHA EM CONSERVA LIGHT |
| 03003 | SARDINHA EM LATA |
| 06203 | SARDINHA EM LATA LIGHT |
| 04501 | SAROLHO |
| 05103 | SARRABULHO |
| 00405 | SARRAFO |
| 00405 | SARRAFO |
| 07705 | SARRAFO DE MADEIRA CERTIFICADA (SELO FSC) |
| 08605 | SARRAFO DE MADEIRA CERTIFICADA (SELO FSC) |
| 04101 | SAUNA |
| 01202 | SAUNA E MASSAGEM |
| 08001 | SAUNA RESIDENCIAL |
| 03502 | SAVELHA EM CONSERVA |
| 01401 | SAXOFONE |
| 11803 | SAZON |
| 11802 | SAZON (TEMPERO) |
| 06306 | SCANER DE COMPUTADOR |
| 06305 | SCANER DE MICROCOMPUTADOR |
| 02301 | SCHIMIER DE CANA |
| 02302 | SCHIMIER DE COLONIA |
| 04601 | SCHIMIER DE FRUTA (EXCETO DE CANA) |
| 04103 | SCHIMIER DE OVOS |
| 13703 | SCHIMIER DE OVOS DIET |
| 13711 | SCHIMIER DE OVOS DIETETICO |
| 05102 | SECADOR DE CABELO |
| 03101 | SECADOR DE CABELOS |
| 07701 | SECADOR DE MAQUINA DE LAVAR LOUCA |
| 15101 | SECADOR DE ROUPA |
| 05101 | SECADOR E MODELADOR DE CABELO |
| 07401 | SECRETARIA ELETRONICA |
| 07403 | SECRETARIA ELETRONICA - FAX |
| 07402 | SECRETARIA ELETRONICA -TELEFONE |
| 04703 | SEDATIVO (MORFINA) |
| 00112 | SEDEX |
| 03801 | SEGUNDA VIA CARTAO DE DEBITO |
| 03802 | SEGUNDA VIA CARTAO DE POUPANCA |
| 02702 | SEGUNDA VIA DE CARTAO ESPECIAL |
| 01702 | SEGURANCA VEICULAR VIA SATELITE |
| 01901 | SEGURELHA (TEMPERO INDUSTRIALIZADO) |
| 01803 | SEGURO COLETIVO DE TRABALHO (PREVIDENCIA PUBLICA) |
| 03501 | SEGURO CONTRA DANOS DE ELETRODOMESTICOS DA COMPANHIA ELETRICA |
| 00503 | SEGURO CONTRA INCENDIO DO IMOVEL |
| 00502 | SEGURO CONTRA ROUBO DO IMOVEL |
| 00902 | SEGURO CONTRA ROUBO E ACIDENTES DE OUTROS IMOVEIS |
| 00901 | SEGURO CONTRA ROUBO E INCENDIO DE OUTROS IMOVEIS |
| 00701 | SEGURO DE ACIDENTES PESSOAIS |
| 02901 | SEGURO DE BENS MOVEIS (EXCETO DE VEICULOS) |
| 03301 | SEGURO DE CARTAO DE CREDITO |
| 00801 | SEGURO DE VIDA |
| 00305 | SEGURO FIADOR |
| 00301 | SEGURO OBRIGATORIO |
| 00603 | SEGURO PARCIAL DE AUTOMOVEIS/UTILITARIOS |
| 00602 | SEGURO TOTAL DE AUTOMOVEIS/UTILITARIOS |
| 01303 | SEGURO TOTAL DE MOTOCICLETA |
| 00601 | SEGURO VOLUNTARIO DE AUTOMOVEIS/UTILITARIOS |
| 01301 | SEGURO VOLUNTARIO DE MOTO |
| 01302 | SEGURO VOLUNTARIO DE MOTOCICLETA |
| 01701 | SEGURO-DESEMPREGO |
| 01301 | SEGURO-EDUCACAO |
| 03401 | SEGURO-HABITACAO (RESTITUICAO) |
| 00501 | SEGUROS SOBRE O IMOVEL (INCENDIO, ROUBO, ETC.) |
| 06901 | SELADORA |
| 06102 | SELETA (JARDINEIRA) |
| 07001 | SELETA DE LEGUMES EM CONSERVA |
| 01502 | SELGA |
| 00104 | SELO |
| 01001 | SELO (HOBBY) |
| 03201 | SEMENTE DE ABOBORA |
| 02603 | SEMENTE DE COENTRO |
| 00905 | SEMENTE DE GIRASSOL |
| 02001 | SEMENTE DE LINHACA |
| 01804 | SEMENTES |
| 00106 | SEMIJOIA |
| 00606 | SEMOLA DE MILHO |
| 01003 | SEMOLINA DE TRIGO |
| 02609 | SENTINELA ESPIRAL |
| 02305 | SEQUILHO |
| 02320 | SEQUILHO DE MAIZENA |
| 10520 | SEQUILHO DE MAIZENA DIET |
| 10560 | SEQUILHO DE MAIZENA DIETETICO |
| 10420 | SEQUILHO DE MAIZENA LIGHT |
| 02318 | SEQUILHO DE MILHO |
| 10518 | SEQUILHO DE MILHO DIET |
| 10558 | SEQUILHO DE MILHO DIETETICO |
| 10418 | SEQUILHO DE MILHO LIGHT |
| 02406 | SEQUILHO DE POLVILHO |
| 10505 | SEQUILHO DIET |
| 10545 | SEQUILHO DIETETICO |
| 10405 | SEQUILHO LIGHT |
| 03801 | SERESTA (INGRESSO) |
| 03401 | SERIGRAFIA (ESTAMPARIA) |
| 30303 | SERINGA |
| 01647 | SERRA (LAMINA DE ACO) |
| 01606 | SERRA MANUAL |
| 14406 | SERRA MARMORE |
| 14402 | SERRADEIRA ELETRICA |
| 01801 | SERRALHA |
| 00609 | SERRINHA DE UNHA |
| 01610 | SERROTE |
| 02901 | SERVENTE |
| 01002 | SERVICO DE CUTELARIA |
| 02101 | SERVICO DE HEMOTERAPIA (TRANSFUSAO) |
| 02501 | SERVICO DE HIGIENE DE ANIMAL DOMESTICO |
| 02002 | SERVICO DE INSTALACAO DE TV A CABO |
| 02701 | SERVICO DE INTERNACAO E CUIDADO DOMICILIAR |
| 01201 | SERVICO DE PATRIMONIO DA UNIAO (SPU) |
| 01702 | SERVICO DE PROTECAO AO CREDITO (TAXAS) |
| 01701 | SERVICO DE SEGURANCA VIA SATELITE |
| 01607 | SERVICO DE TERRAPLANAGEM (EMPREITADA) |
| 01607 | SERVICO DE TERRAPLANAGEM (EMPREITADA) |
| 00501 | SERVICOS DE CIRURGIA, ANESTESIA E OBSTETRICIA |
| 02501 | SERVICOS DE ENFERMAGEM |
| 02901 | SERVICOS DE NUTRICAO ENTERAL E PARENTAL |
| 00814 | SHAMPOO |
| 00414 | SHAMPU |
| 06101 | SHAMPU DE CARPETE |
| 06102 | SHAMPU DE TAPETE |
| 00424 | SHAMPU E CONDICIONADOR |
| 30702 | SHAMPU MEDICINAL |
| 01403 | SHAMPU PARA CAO |
| 00401 | SHAMPU, CONDICIONADOR E OUTROS PRODUTOS DE CABELO |
| 02001 | SHERRY |
| 00203 | SHORT |
| 00202 | SHORT |
| 00202 | SHORT |
| 01101 | SHOW (INGRESSO) |
| 02606 | SHOWS (INGRESSO) |
| 03604 | SHOYO |
| 10504 | SHOYO LIGHT |
| 01202 | SIDRA CHAMPANHE |
| 03302 | SILVO |
| 01502 | SINTECO |
| 01502 | SINTECO |
| 02104 | SINUCA |
| 00802 | SINUCA (APOSTA) |
| 02101 | SINUCA,TOTO E BOLICHE |
| 61101 | SIRI |
| 61111 | SIRI AZUL |
| 61109 | SIRI BAU |
| 61106 | SIRI BRANCO |
| 61108 | SIRI CANELA |
| 61105 | SIRI DO MANGUE |
| 61110 | SIRI GUAJA |
| 61115 | SIRI PATOLA |
| 61103 | SIRI PIMENTA |
| 61401 | SIRI SALGADO |
| 61201 | SIRI SEM CASCA |
| 61114 | SIRIACU |
| 61113 | SIRIMIRIM |
| 61116 | SIRIPUA |
| 66103 | SIRIRI |
| 00302 | SKATE |
| 02101 | SLACK |
| 02401 | SLACK |
| 04401 | SLACK |
| 02901 | SMOKING |
| 05804 | SNAPPLE |
| 00505 | SOBRECOXA DE FRANGO |
| 05805 | SOBRECOXA DE FRANGO ORGANICA |
| 00502 | SOBRECOXA DE GALINHA OU FRANGO |
| 05803 | SOBRECOXA DE GALINHA OU FRANGO ORGANICA |
| 03910 | SOBRECOXA DE PATO |
| 01902 | SOBRECOXA DE PERU |
| 04201 | SOBRECOXA NAO-ESPECIFICADA |
| 07501 | SOBREMESA DE QUALQUER SABOR DIET |
| 07502 | SOBREMESA DE QUALQUER SABOR DIETETICA |
| 07301 | SOBREMESA DE QUALQUER TIPO (EXCETO INFANTIL) |
| 12701 | SOBREMESA DE QUALQUER TIPO (EXCETO INFANTIL) DIET |
| 12702 | SOBREMESA DE QUALQUER TIPO (EXCETO INFANTIL) DIETETICA |
| 12601 | SOBREMESA DE QUALQUER TIPO (EXCETO INFANTIL) LIGHT |
| 02501 | SOBREMESA INFANTIL DE QUALQUER MARCA E SABOR |
| 09701 | SOBREMESA LIGHT |
| 02001 | SOBRETUDO |
| 05501 | SOBRETUDO |
| 01604 | SOCADOR DE ALHO DE MADEIRA |
| 00802 | SOCADOR DE CARNE DE METAL |
| 01601 | SODA CAUSTICA |
| 00403 | SODA LIMONADA |
| 13303 | SODA LIMONADA LIGHT |
| 07503 | SODA LIMONADA LIGHT |
| 01002 | SOFA |
| 01001 | SOFA OU SOFA-CAMA |
| 01003 | SOFA-CAMA |
| 04802 | SOFT DE CURSO |
| 01602 | SOFT DE JOGO |
| 04801 | SOFTWARE DE CURSO |
| 01601 | SOFTWARE DE JOGO |
| 03001 | SOJA EM GRAO |
| 03701 | SOJA EM GRAO ORGANICA |
| 02309 | SOLDA |
| 10509 | SOLDA DIET |
| 10549 | SOLDA DIETETICA |
| 10409 | SOLDA LIGHT |
| 03001 | SOLEIRA E PARAPEITO DE JANELA DE QUALQUER MATERIAL |
| 03001 | SOLEIRA E PARAPEITO DE JANELA DE QUALQUER MATERIAL |
| 30602 | SOLUCAO DE LENTE DE CONTACTO |
| 02903 | SOLUCAO OFTALMICA (PARA A VISTA) |
| 04301 | SOLUCOES HIDRATANTES |
| 00822 | SOLVENTE |
| 01501 | SOLVENTE |
| 00901 | SOM (ALUGUEL) |
| 00207 | SOMBRANCELHA (CABELEIREIRO) |
| 06003 | SOMBRINHA |
| 30902 | SONDA (QUALQUER TIPO) |
| 04308 | SONHO |
| 03701 | SONHO |
| 07616 | SONHO DIET |
| 07401 | SONHO DIET |
| 07617 | SONHO DIETETICO |
| 07403 | SONHO DIETETICO |
| 09607 | SONHO LIGHT |
| 07301 | SONHO LIGHT |
| 01806 | SONIFERO |
| 01403 | SOPA CONCENTRADA EM PACOTE DE QUALQUER SABOR |
| 06103 | SOPA CONCENTRADA EM PACOTE DE QUALQUER SABOR LIGHT |
| 01409 | SOPA DE LEGUMES (SOPA DESIDRATADA) |
| 01301 | SOPA DE LEGUMES EM CONSERVA |
| 03807 | SOPA DE QUALQUER TIPO |
| 01401 | SOPA DESIDRATADA DE QUALQUER SABOR |
| 06101 | SOPA DESIDRATADA DE QUALQUER SABOR LIGHT |
| 01402 | SOPA EM PACOTE DE QUALQUER SABOR |
| 06102 | SOPA EM PACOTE DE QUALQUER SABOR LIGHT |
| 01202 | SOPA INFANTIL COM LEGUMES OU VERDURAS |
| 01201 | SOPA INFANTIL DE QUALQUER SABOR |
| 04501 | SOPA PARA VIAGEM |
| 01208 | SOPINHA PARA BEBE |
| 04801 | SORGO EM GRAO |
| 04303 | SORO FISIOLOGICO HIDRATANTE |
| 30603 | SORO FISIOLOGICO PARA LENTE DE CONTATO |
| 04502 | SORVETE |
| 04806 | SORVETE CASEIRO |
| 11406 | SORVETE CASEIRO LIGHT |
| 07001 | SORVETE DE QUALQUER SABOR DIET |
| 07002 | SORVETE DE QUALQUER SABOR DIETETICO |
| 00501 | SORVETE DE QUALQUER SABOR INDUSTRIALIZADO |
| 09701 | SORVETE DE QUALQUER SABOR INDUSTRIALIZADO DIET |
| 09706 | SORVETE DE QUALQUER SABOR INDUSTRIALIZADO DIETETICO |
| 09601 | SORVETE DE QUALQUER SABOR INDUSTRIALIZADO LIGHT |
| 09301 | SORVETE DE QUALQUER SABOR LIGHT |
| 00504 | SORVETE INDUSTRIALIZADO |
| 09704 | SORVETE INDUSTRIALIZADO DIET |
| 09709 | SORVETE INDUSTRIALIZADO DIETETICO |
| 09604 | SORVETE INDUSTRIALIZADO LIGHT |
| 04501 | SORVETE OU PICOLE |
| 03312 | SPAGHETTI COM OVOS |
| 03212 | SPAGHETTI SEM OVOS |
| 01701 | SPC (TAXAS) |
| 02404 | SPORTADE |
| 09203 | SPRIT DIET |
| 09206 | SPRIT DIETETICO |
| 00407 | SPRIT REFRIGERANTE |
| 07603 | SPRIT REFRIGERANTE DIET |
| 07606 | SPRIT REFRIGERANTE DIETETICO |
| 02701 | ST. REMY |
| 02902 | STEAK DE FRANGO |
| 02904 | STEAK DE GALINHA |
| 00601 | STEINHARGER |
| 00112 | STRAP |
| 05401 | STROGONOFF EM CONSERVA |
| 05403 | STROGONOFF EM PACOTE |
| 05402 | STROGONOFF ENCARTONADO |
| 12401 | SUA BOVINA |
| 07501 | SUA SUINA |
| 01803 | SUCARIL |
| 02409 | SUCO ARTIFICIAL EM PO |
| 02604 | SUCO DE ABACAXI |
| 07902 | SUCO DE ABACAXI ORGANICO PARA VIAGEM |
| 00402 | SUCO DE ABACAXI PARA VIAGEM |
| 02605 | SUCO DE ACEROLA |
| 07903 | SUCO DE ACEROLA ORGANICO PARA VIAGEM |
| 00403 | SUCO DE ACEROLA PARA VIAGEM |
| 02606 | SUCO DE BETERRABA |
| 07904 | SUCO DE BETERRABA ORGANICO PARA VIAGEM |
| 00404 | SUCO DE BETERRABA PARA VIAGEM |
| 02607 | SUCO DE CUPUACU |
| 07905 | SUCO DE CUPUACU ORGANICO PARA VIAGEM |
| 00405 | SUCO DE CUPUACU PARA VIAGEM |
| 08603 | SUCO DE FRUTA DIET |
| 08604 | SUCO DE FRUTA DIETETICO |
| 12501 | SUCO DE FRUTA LIGHT |
| 02401 | SUCO DE FRUTA OU VEGETAL ARTIFICIAL EM PO |
| 11101 | SUCO DE FRUTA OU VEGETAL ARTIFICIAL EM PO DIET |
| 11108 | SUCO DE FRUTA OU VEGETAL ARTIFICIAL EM PO DIETETICO |
| 11001 | SUCO DE FRUTA OU VEGETAL ARTIFICIAL EM PO LIGHT |
| 08601 | SUCO DE FRUTA OU VEGETAL DIET |
| 08602 | SUCO DE FRUTA OU VEGETAL DIETETICO |
| 04703 | SUCO DE FRUTA OU VEGETAL EM CAIXA |
| 11703 | SUCO DE FRUTA OU VEGETAL EM CAIXA DIET |
| 11707 | SUCO DE FRUTA OU VEGETAL EM CAIXA DIETETICO |
| 11603 | SUCO DE FRUTA OU VEGETAL EM CAIXA LIGHT |
| 14301 | SUCO DE FRUTA OU VEGETAL EM COPO PLASTICO |
| 05101 | SUCO DE FRUTA OU VEGETAL EM LATA |
| 11901 | SUCO DE FRUTA OU VEGETAL EM LATA DIET |
| 11903 | SUCO DE FRUTA OU VEGETAL EM LATA DIETETICO |
| 11801 | SUCO DE FRUTA OU VEGETAL EM LATA LIGHT |
| 05301 | SUCO DE FRUTA OU VEGETAL EM SACO PLASTICO |
| 12101 | SUCO DE FRUTA OU VEGETAL EM SACO PLASTICO DIET |
| 12103 | SUCO DE FRUTA OU VEGETAL EM SACO PLASTICO DIETETICO |
| 12001 | SUCO DE FRUTA OU VEGETAL EM SACO PLASTICO LIGHT |
| 04701 | SUCO DE FRUTA OU VEGETAL ENCARTONADO |
| 11701 | SUCO DE FRUTA OU VEGETAL ENCARTONADO DIET |
| 11705 | SUCO DE FRUTA OU VEGETAL ENCARTONADO DIETETICO |
| 11601 | SUCO DE FRUTA OU VEGETAL ENCARTONADO LIGHT |
| 02201 | SUCO DE FRUTA OU VEGETAL ENGARRAFADO |
| 11501 | SUCO DE FRUTA OU VEGETAL ENGARRAFADO DIET |
| 11503 | SUCO DE FRUTA OU VEGETAL ENGARRAFADO DIETETICO |
| 11401 | SUCO DE FRUTA OU VEGETAL ENGARRAFADO LIGHT |
| 06201 | SUCO DE FRUTA OU VEGETAL NAO-ESPECIFICADO |
| 12301 | SUCO DE FRUTA OU VEGETAL NAO-ESPECIFICADO DIET |
| 12302 | SUCO DE FRUTA OU VEGETAL NAO-ESPECIFICADO DIETETICO |
| 12201 | SUCO DE FRUTA OU VEGETAL NAO-ESPECIFICADO LIGHT |
| 06601 | SUCO DE FRUTA OU VEGETAL ORGANICO |
| 10203 | SUCO DE FRUTA OU VEGETAL ORGANICO EM CAIXA |
| 10301 | SUCO DE FRUTA OU VEGETAL ORGANICO EM LATA |
| 10401 | SUCO DE FRUTA OU VEGETAL ORGANICO EM SACO PLASTICO |
| 10201 | SUCO DE FRUTA OU VEGETAL ORGANICO ENCARTONADO |
| 10101 | SUCO DE FRUTA OU VEGETAL ORGANICO ENGARRAFADO |
| 10501 | SUCO DE FRUTA OU VEGETAL ORGANICO NAO-ESPECIFICADO |
| 02602 | SUCO DE FRUTAS |
| 02601 | SUCO DE FRUTAS OU VEGETAIS |
| 02608 | SUCO DE GOIABA |
| 07906 | SUCO DE GOIABA ORGANICO PARA VIAGEM |
| 00406 | SUCO DE GOIABA PARA VIAGEM |
| 02609 | SUCO DE LARANJA |
| 07911 | SUCO DE LARANJA CENOURA E BETERRABA ORGANICO PARA VIAGEM |
| 00411 | SUCO DE LARANJA CENOURA E BETERRABA PARA VIAGEM |
| 07908 | SUCO DE LARANJA COM BANANA ORGANICO PARA VIAGEM |
| 00408 | SUCO DE LARANJA COM BANANA PARA VIAGEM |
| 02610 | SUCO DE LARANJA E BANANA |
| 02611 | SUCO DE LARANJA E BETERRABA |
| 07909 | SUCO DE LARANJA E BETERRABA ORGANICO PARA VIAGEM |
| 00409 | SUCO DE LARANJA E BETERRABA PARA VIAGEM |
| 02612 | SUCO DE LARANJA E CENOURA |
| 07910 | SUCO DE LARANJA E CENOURA ORGANICO PARA VIAGEM |
| 00410 | SUCO DE LARANJA E CENOURA PARA VIAGEM |
| 07907 | SUCO DE LARANJA ORGANICO PARA VIAGEM |
| 00407 | SUCO DE LARANJA PARA VIAGEM |
| 02613 | SUCO DE LARANJA, CENOURA E BETERRABA |
| 00419 | SUCO DE MACA PARA VIAGEM |
| 02614 | SUCO DE MAMAO |
| 07912 | SUCO DE MAMAO ORGANICO PARA VIAGEM |
| 00412 | SUCO DE MAMAO PARA VIAGEM |
| 02615 | SUCO DE MANGA |
| 07913 | SUCO DE MANGA ORGANICO PARA VIAGEM |
| 00413 | SUCO DE MANGA PARA VIAGEM |
| 02616 | SUCO DE MARACUJA |
| 07914 | SUCO DE MARACUJA ORGANICO PARA VIAGEM |
| 00414 | SUCO DE MARACUJA PARA VIAGEM |
| 02617 | SUCO DE MELAO |
| 07915 | SUCO DE MELAO ORGANICO PARA VIAGEM |
| 00415 | SUCO DE MELAO PARA VIAGEM |
| 02618 | SUCO DE MORANGO |
| 07916 | SUCO DE MORANGO ORGANICO PARA VIAGEM |
| 00416 | SUCO DE MORANGO PARA VIAGEM |
| 02619 | SUCO DE PESSEGO |
| 07918 | SUCO DE PESSEGO EM CALDA ORGANICO PARA VIAGEM |
| 00418 | SUCO DE PESSEGO EM CALDA PARA VIAGEM |
| 02620 | SUCO DE PESSEGO EM CALDAS |
| 07917 | SUCO DE PESSEGO ORGANICO PARA VIAGEM |
| 00417 | SUCO DE PESSEGO PARA VIAGEM |
| 02309 | SUCO DE SOJA |
| 02603 | SUCO DE VEGETAIS |
| 08605 | SUCO DE VEGETAL DIET |
| 08606 | SUCO DE VEGETAL DIETETICO |
| 02412 | SUCO EM PO |
| 07901 | SUCO ORGANICO PARA VIAGEM |
| 00401 | SUCO PARA VIAGEM |
| 04321 | SUCRILHOS |
| 00902 | SUCRILHOS DE MILHO |
| 05902 | SUCRILHOS DE MILHO LIGHT |
| 00102 | SUETER |
| 00103 | SUETER |
| 00102 | SUETER |
| 06901 | SUFLE PARA VIAGEM |
| 08403 | SUINO MORTO EVISCERADO |
| 05302 | SUINO VIVO |
| 01802 | SUITA |
| 00208 | SUKITA |
| 13009 | SUKITA LIGHT |
| 01508 | SUKITA LIGHT |
| 04201 | SULFATO DE ALUMINIO PARA LIMPEZA DOMESTICA |
| 05303 | SULFATO DE ALUMINIO PARA PISCINA |
| 05303 | SULFATO DE ALUMINIO PARA PISCINA |
| 04202 | SULFATO DE SODIO PARA LIMPEZA DOMESTICA |
| 00502 | SUNGA |
| 00901 | SUNGA DE PRAIA |
| 00501 | SUNGA, MAIO E BIQUINI |
| 02704 | SUPER BOND |
| 02704 | SUPER BOND |
| 03108 | SUPERLAC |
| 06808 | SUPERLAC DIET |
| 06818 | SUPERLAC DIETETICO |
| 06708 | SUPERLAC LIGHT |
| 05801 | SUPLIGEM |
| 03601 | SUPORTE DE BIBLIA |
| 01633 | SUPORTE DE FERRO DE SOLDAR |
| 00921 | SUPORTE DE GARRAFA TERMICA DE PLASTICO |
| 00902 | SUPORTE DE LATA DE LIXO |
| 00811 | SUPORTE DE PANELAS |
| 13901 | SUPORTE DE TV GIRATORIO |
| 01807 | SUPORTE DE VASO |
| 09801 | SUPORTE ELETRICO PARA GARRAFAO DE AGUA |
| 04002 | SUPORTE PARA AGUA MINERAL |
| 09301 | SUPORTE PARA BUJAO DE GAS |
| 17701 | SUPORTE PARA FORNO |
| 04001 | SUPORTE PARA GARRAFAO DE AGUA |
| 00926 | SUPORTE PARA PAPEL ALUMINIO DE PLASTICO |
| 01605 | SUPORTE PARA PAPEL HIGIENICO DE MADEIRA |
| 00927 | SUPORTE PARA PAPEL HIGIENICO DE PLASTICO |
| 30002 | SUPOSITORIO |
| 03801 | SURDO, REPINIQUE, TAMBOR, CAIXA, BUMBO |
| 69105 | SURUANA |
| 66101 | SURURU |
| 63104 | SURURU DAS PEDRAS (MARISCO) |
| 66102 | SURURU DE ALAGOAS |
| 66401 | SURURU SALGADO |
| 66201 | SURURU SEM CASCA |
| 04201 | SUSHI PARA VIAGEM |
| 04202 | SUSHI PRONTO PARA VIAGEM |
| 02201 | SUSPENSORIO |
| 04117 | SUSPENSORIO ESCROTAL |
| 08501 | SUSPIRO |
| 05101 | SUSTAGEM |
| 04001 | SUTIA |
| 01101 | SUTIA |
| 00701 | TABLETE DE CHOCOLATE |
| 07205 | TABLETE DE CHOCOLATE DIET |
| 10101 | TABLETE DE CHOCOLATE DIET |
| 07206 | TABLETE DE CHOCOLATE DIETETICO |
| 10107 | TABLETE DE CHOCOLATE DIETETICO |
| 09503 | TABLETE DE CHOCOLATE LIGHT |
| 10001 | TABLETE DE CHOCOLATE LIGHT |
| 01603 | TABUA DE CARNE |
| 03723 | TABUA DE FRIOS |
| 15401 | TABUA DE PASSAR ROUPA |
| 09601 | TABULEIRO |
| 04401 | TACA DE CRISTAL |
| 03201 | TACA DE SORVETE |
| 04402 | TACA DE VIDRO |
| 03808 | TACACA |
| 02401 | TACACA PARA VIAGEM |
| 00402 | TACO |
| 00402 | TACO |
| 07702 | TACO DE MADEIRA CERTIFICADA (SELO FSC) |
| 08602 | TACO DE MADEIRA CERTIFICADA (SELO FSC) |
| 05802 | TAFFMAN E |
| 01403 | TAIA |
| 00509 | TAIA JAPONES |
| 04902 | TAINHA EM CONSERVA |
| 01401 | TAIOBA |
| 01402 | TAIOBA SAO TOME |
| 00507 | TAIOBA SAO TOME INHAME |
| 02301 | TALAO DE CHEQUES |
| 00301 | TALCO |
| 03406 | TALHARIM (NAO-ESPECIFICADO) |
| 03311 | TALHARIM COM OVOS |
| 03414 | TALHARIM NAO-ESPECIFICADO |
| 03211 | TALHARIM SEM OVOS |
| 07601 | TALHER DE MADEIRA |
| 06201 | TALHER DE PLASTICO |
| 00701 | TALHERES |
| 02001 | TAMANCO |
| 02413 | TAMARA DESIDRATADA |
| 02404 | TAMARA SECA OU DESIDRATADA |
| 06201 | TAMARINDO |
| 06202 | TAMARINO |
| 03202 | TAMPA DE CAIXA DE AGUA |
| 03202 | TAMPA DE CAIXA DE AGUA |
| 00914 | TAMPA DE TANQUE DE PLASTICO |
| 01502 | TAMPO DE MESA (SALA) (QUALQUER MATERIAL) |
| 03701 | TAM-TAM, CONGA |
| 06601 | TANAJURA |
| 00505 | TANGA |
| 02201 | TANGERINA |
| 02213 | TANGERINA CRAVO |
| 02219 | TANGERINA MONGORT |
| 02212 | TANGERINA POKAN |
| 04302 | TANGERINA REFRIGERANTE |
| 08802 | TANGERINA REFRIGERANTE LIGHT |
| 02223 | TANJA |
| 03601 | TANQUE DE QUALQUER MATERIAL |
| 03601 | TANQUE DE QUALQUER MATERIAL |
| 06501 | TANQUE DE ROUPA ELETRICO |
| 06502 | TANQUINHO ELETRICO |
| 00101 | TAPECARIA |
| 04901 | TAPECEIRO |
| 03501 | TAPE-DECK |
| 04701 | TAPEREBA |
| 00803 | TAPETE |
| 00501 | TAPETE DE BANHEIRO (TECIDO) |
| 00401 | TAPETE DE PANO |
| 00201 | TAPETE DE SALA OU QUARTO |
| 02503 | TAPETES DE BORRACHA |
| 04506 | TAPIOCA (BEIJU) |
| 04507 | TAPIOCA COM COCO |
| 01516 | TAPIOCA DE GOMA |
| 04508 | TAPIOCA DE QUEIJO |
| 04327 | TAPIOCA DOCE |
| 01509 | TAPIOCA GOMA |
| 03737 | TAPIOCA SALGADA |
| 44124 | TARACAIA |
| 44123 | TARECAI |
| 02306 | TARECO |
| 10506 | TARECO DIET |
| 10546 | TARECO DIETETICO |
| 10406 | TARECO LIGHT |
| 02801 | TARIFA DE CONTA BANCARIA |
| 02503 | TARIFA DE MANUTENCAO DE CHEQUE ESPECIAL |
| 01629 | TARRACHA |
| 00519 | TARRAFA |
| 44104 | TARTARUGA DE AGUA DOCE |
| 69101 | TARTARUGA DE MAR |
| 69102 | TARTARUGA DE PENTE |
| 44105 | TARTARUGA DO AMAZONAS |
| 69501 | TARTARUGA EM FILE CONGELADO |
| 69301 | TARTARUGA EM FILE FRESCO |
| 69201 | TARTARUGA EM POSTAS |
| 69401 | TARTARUGA SALGADA |
| 69103 | TARTARUGA VERDADEIRA |
| 69104 | TARTARUGA VERDE |
| 00606 | TATU (LAGARTO REDONDO) |
| 11806 | TATU (LAGARTO REDONDO) ORGANICO |
| 06704 | TATU VIVO |
| 04001 | TATUAGEM |
| 02001 | TAXA (INCENDIO, LIXO, SEGURANCA, ETC.) DE OUTROS IMOVEIS |
| 01801 | TAXA ALFANDEGARIA |
| 03702 | TAXA CARTAO DE PRODUTOR RURAL |
| 02101 | TAXA DE ADESAO DE TELEVISAO POR ASSINATURA |
| 00608 | TAXA DE ASFALTAMENTO |
| 00914 | TAXA DE BAIXA DE RESERVA DE REGISTRO DE VEICULO |
| 01902 | TAXA DE BOMBEIRO (DETRAN) |
| 03701 | TAXA DE CADASTRAMENTO DO CPF |
| 03301 | TAXA DE CAIXA POSTAL DE CORREIO |
| 00902 | TAXA DE CARTEIRA DE HABILITACAO |
| 02207 | TAXA DE CEMITERIO (MANUNTENCAO) |
| 01901 | TAXA DE CERTIDAO DE IMPOSTO (DIVIDA ATIVA) |
| 00505 | TAXA DE CLUBE |
| 00602 | TAXA DE COLETA DE LIXO |
| 07602 | TAXA DE CONDOMINIO DE JAZIGO |
| 03402 | TAXA DE CONSERVACAO DE IMOVEL (RESTITUICAO) |
| 01801 | TAXA DE CONSERVACAO E MANUTENCAO DE VIAS PUBLICAS |
| 00606 | TAXA DE CONSERVACAO URBANA |
| 01903 | TAXA DE CORREIO (DETRAN) |
| 00917 | TAXA DE DESALIENACAO DE VEICULO |
| 03101 | TAXA DE DEVOLUCAO DE CHEQUE |
| 00503 | TAXA DE EXAME BIOMEDICO |
| 00919 | TAXA DE EXPEDIENTE |
| 00901 | TAXA DE EXPEDIENTE (DETRAN) |
| 00305 | TAXA DE FUNERARIA |
| 02009 | TAXA DE HABITE |
| 00605 | TAXA DE ILUMINACAO PUBLICA |
| 02005 | TAXA DE INCENDIO DE OUTROS IMOVEIS |
| 00603 | TAXA DE INGRESSO (JOIA) DE PREVIDENCIA PRIVADA |
| 01702 | TAXA DE INSCRICAO DE CONGRESSO (EDUCACAO) |
| 01701 | TAXA DE INSCRICAO DE CONGRESSO, SEMINARIO, ETC. (EDUCACAO) |
| 01704 | TAXA DE INSCRICAO DE PALESTRA EDUCATIVA |
| 01703 | TAXA DE INSCRICAO DE SEMINARIO (EDUCACAO) |
| 04201 | TAXA DE INSCRICAO DE VESTIBULAR |
| 04601 | TAXA DE INSCRICAO PARA CONCURSO |
| 04101 | TAXA DE INSCRICAO PARA CONCURSO (EMPREGO) |
| 04102 | TAXA DE INSCRICAO PARA CONGRESSO (NAO-EDUCACAO) |
| 01601 | TAXA DE INSTALACAO DE CENTRAL DE GAS |
| 00609 | TAXA DE INSTALACAO DE ESGOTO |
| 00802 | TAXA DE INSTALACAO DE HIDROMETRO |
| 02007 | TAXA DE INSTALACAO DE HIDROMETRO DE OUTROS IMOVEIS |
| 01002 | TAXA DE INSTALACAO DE INTERFONE |
| 01001 | TAXA DE INSTALACAO DE INTERFONE,TELEFONE |
| 02201 | TAXA DE INSTALACAO DE INTERNET |
| 01602 | TAXA DE INSTALACAO DE MEDIDOR DE GAS |
| 01301 | TAXA DE INSTALACAO DE POSTE DE ENERGIA ELETRICA |
| 00801 | TAXA DE INSTALACAO DE RELOGIO DE AGUA |
| 02008 | TAXA DE INSTALACAO DE RELOGIO DE AGUA DE OUTROS IMOVEIS |
| 01003 | TAXA DE INSTALACAO DE TELEFONE |
| 02001 | TAXA DE INSTALACAO DE TELEVISAO POR ASSINATURA |
| 01401 | TAXA DE ISENCAO DE IMPOSTO PREDIAL |
| 00920 | TAXA DE LACRE DA PLACA |
| 00918 | TAXA DE LEGISLACAO (DETRAN) |
| 01201 | TAXA DE LIGACAO DE LUZ |
| 00607 | TAXA DE LIXO |
| 02004 | TAXA DE LIXO DE OUTROS IMOVEIS |
| 03001 | TAXA DE MANUTENCAO DE ARMAS |
| 01101 | TAXA DE MANUTENCAO DE ELEVADOR |
| 01801 | TAXA DE MANUTENCAO DE LINHA TELEFONICA COMUNITARIA |
| 02303 | TAXA DE MANUTENCAO DE PREDIO (EXTRA DE CONDOMINIO) |
| 00502 | TAXA DE MATERIAL ESCOLAR |
| 02002 | TAXA DE MELHORAMENTOS DE OUTROS IMOVEIS |
| 00911 | TAXA DE NADA CONSTA |
| 00701 | TAXA DE OCUPACAO DO IMOVEL |
| 05102 | TAXA DE PESCA |
| 03002 | TAXA DE PIQUENIQUE |
| 00603 | TAXA DE PREVENCAO E EXTINCAO DE INCENDIO |
| 02302 | TAXA DE REFORMA DE PREDIO (EXTRA DE CONDOMINIO) |
| 01202 | TAXA DE RELIGACAO DE LUZ |
| 00803 | TAXA DE RELIGACAO DE RELOGIO DE AGUA |
| 03001 | TAXA DE RELIGACAO DE TELEFONE |
| 03704 | TAXA DE RENOVACAO DO CPF |
| 03101 | TAXA DE SALTO (PARAQUEDISMO) |
| 02901 | TAXA DE SAQUE ELETRONICO |
| 00604 | TAXA DE SEGURANCA |
| 02003 | TAXA DE SEGURANCA DE OUTROS IMOVEIS |
| 01902 | TAXA DE TELEVISAO POR ASSINATURA DE OUTROS IMOVEIS |
| 02505 | TAXA DE TRANSFERENCIA DE FINANCIAMENTO |
| 00906 | TAXA DE TRANSFERENCIA DE PLACA DE VEICULO |
| 00901 | TAXA DE TRANSFERENCIA DE TELEFONE |
| 01001 | TAXA DE TRANSFERENCIA DE TELEFONE CELULAR |
| 00904 | TAXA DE TRANSFERENCIA DE VEICULO |
| 00504 | TAXA DE TRANSFERENCIA ESCOLAR |
| 00610 | TAXA DE TRATAMENTO DE ESGOTO |
| 01701 | TAXA DE UTILIZACAO E MANUTENCAO DE BOMBA DE AGUA |
| 01501 | TAXA DE VERIFICACAO DE VAZAMENTO DE AGUA |
| 00921 | TAXA DE VISTORIA |
| 02301 | TAXA EXTRA DE CONDOMINIO (REFORMA E MANUTENCAO) |
| 02304 | TAXA EXTRA DE FUNDO DE RESERVA (CONDOMINIO) |
| 03703 | TAXA PARA DOCUMENTOS PESSOAIS |
| 03001 | TAXA PARA SUSTAR CHEQUES |
| 00601 | TAXAS (COLETA DE LIXO, PREVENCAO E EXTINCAO DE INCENDIO, SEGURANCA, ETC.) |
| 00501 | TAXAS DE LOCACAO DE OUTROS IMOVEIS (CONTRATO, DEPOSITO, MULTA, ETC.) |
| 00701 | TAXAS DE ORGAOS PUBLICOS |
| 00501 | TAXAS ESCOLARES |
| 01901 | TAXAS INCLUIDAS NO LICENCIAMENTO ANUAL VEICULOS |
| 00202 | TAXAS PARA AQUISICAO DO IMOVEL A PRAZO |
| 00301 | TAXI |
| 01101 | TAXI |
| 02602 | TAXI-MOTO |
| 00201 | TEATRO (INGRESSO) |
| 02605 | TEATROS (INGRESSO) |
| 04302 | TECIDO (PECA DE DECORACAO) |
| 00201 | TECIDOS |
| 00602 | TECLADO |
| 06307 | TECLADO PARA COMPUTADOR |
| 01901 | TECNICO EM AGRICULTURA |
| 02603 | TED PESSOAL (TRANSFERENCIA INTERBANCARIA) |
| 04906 | TEKITOS (CARNE DE FRANGO EMPANADA) |
| 01502 | TELA A OLEO |
| 01108 | TELA DE ARAME |
| 01108 | TELA DE ARAME |
| 02401 | TELA PARA PINTURA |
| 16601 | TELAO |
| 01201 | TELAO (ALUGUEL) |
| 01201 | TELEFONE CELULAR (APARELHO) |
| 01101 | TELEFONE FIXO E CELULAR (PACOTE) |
| 00901 | TELEFONE FIXO E INTERNET (PACOTE) |
| 01501 | TELEFONE FIXO E TV POR ASSINATURA (PACOTE) |
| 00801 | TELEFONE FIXO, CELULAR E INTERNET (PACOTE) |
| 01401 | TELEFONE FIXO, INTERNET E TV POR ASSINATURA (PACOTE) |
| 00201 | TELEFONE PUBLICO |
| 00401 | TELEFONE RESIDENCIAL |
| 00201 | TELEFONE RESIDENCIAL (ALUGUEL) |
| 01101 | TELEFONE RESIDENCIAL DE OUTROS IMOVEIS |
| 01201 | TELEFONE VIRTUAL - MENSAGEM (MENSALIDADE, ASSINATURA, ALUGUEL) |
| 00901 | TELEFONE VIRTUAL (APARELHO) |
| 05901 | TELEFONE-RADIO-RELOGIO |
| 00103 | TELEGRAMA COMUM |
| 00301 | TELEGRAMA FONADO |
| 00401 | TELEMENSAGEM |
| 12503 | TELESCOPIO |
| 01301 | TELESENA |
| 00301 | TELEVISAO (ALUGUEL) |
| 09101 | TELEVISAO E RADIO ACOPLADOS |
| 09301 | TELEVISAO E VIDEOCASSETE ACOPLADOS |
| 01301 | TELEVISAO EM CORES |
| 02401 | TELEVISAO EM CORES |
| 01401 | TELEVISAO EM PRETO E BRANCO |
| 02501 | TELEVISAO EM PRETO E BRANCO |
| 00108 | TELEX |
| 02905 | TELHA DE ALUMINIO |
| 02905 | TELHA DE ALUMINIO |
| 02903 | TELHA DE AMIANTO |
| 02903 | TELHA DE AMIANTO |
| 02902 | TELHA DE BARRO |
| 02902 | TELHA DE BARRO |
| 02904 | TELHA DE FIBRA |
| 02904 | TELHA DE FIBRA |
| 02901 | TELHAS (BARRO, AMIANTO, FIBRA, ALUMINIO, ETC.) |
| 02901 | TELHAS (BARRO, AMIANTO, FIBRA, ALUMINIO, ETC.) |
| 00412 | TEMPERO ALHO E SAL |
| 00406 | TEMPERO ARISCO |
| 08403 | TEMPERO CASEIRO NAO-ESPECIFICADO |
| 00413 | TEMPERO COMPLETO EM PASTA |
| 05801 | TEMPERO DE FEIJAO |
| 05601 | TEMPERO DE MASSAS |
| 06201 | TEMPERO DE OMELETE EM PO |
| 04001 | TEMPERO DE SALADAS |
| 10701 | TEMPERO DE SALADAS LIGHT |
| 08301 | TEMPERO EM PO NAO-ESPECIFICADO |
| 12204 | TEMPERO LIQUIDO COMPLETO |
| 12203 | TEMPERO LIQUIDO DE CEBOLA E ALHO |
| 12202 | TEMPERO LIQUIDO MISTO |
| 05304 | TEMPERO MISTO EM GRAO |
| 05302 | TEMPERO MISTO EM PO |
| 00401 | TEMPERO MISTO INDUSTRIALIZADO EM PASTA |
| 12201 | TEMPERO MISTO LIQUIDO |
| 08401 | TEMPERO NAO-ESPECIFICADO |
| 12101 | TEMPERO NAO-ESPECIFICADO EM PACOTE |
| 07602 | TEMPERO NAO-ESPECIFICADO EM TABLETE |
| 11801 | TEMPERO SAZON |
| 01204 | TEMPERO VERDE |
| 00606 | TENDAS |
| 02702 | TENDER |
| 00405 | TENIS (INGRESSO) |
| 04601 | TENIS (NAO-ESPECIFICADO) |
| 01501 | TENIS DE CRIANCA |
| 01601 | TENIS DE HOMEM |
| 06601 | TENIS DE MULHER |
| 02504 | TENSIOMETRO |
| 03401 | TEQUILA |
| 01614 | TERCADO |
| 44111 | TERECAI |
| 30502 | TERMOMETRO |
| 03703 | TERNINHO |
| 01401 | TERNO |
| 03702 | TERNO |
| 01503 | TERNO |
| 03701 | TERNO OU TERNINHO |
| 02206 | TERRA |
| 02206 | TERRA |
| 01803 | TERRA VEGETAL |
| 01606 | TERRAPLANAGEM (EMPREITADA) |
| 01606 | TERRAPLANAGEM (EMPREITADA) |
| 00111 | TESOURA |
| 61119 | TESOURA (SIRI) |
| 01638 | TESOURA DE CORTAR LATAO |
| 01659 | TESOURA DE JARDINEIRO |
| 00210 | TESOURA ESCOLAR |
| 00802 | TESOURINHA DE UNHA |
| 30505 | TESTE DE GRAVIDEZ |
| 02901 | TESTICULO BOVINO |
| 06401 | TETO SOLAR |
| 06401 | TETO SOLAR |
| 00707 | TIARA |
| 01106 | TICUM |
| 08102 | TIGELA |
| 00601 | TIJOLO |
| 00601 | TIJOLO |
| 00205 | TINGIMENTO (CABELEIREIRO) |
| 00801 | TINTA |
| 00801 | TINTA |
| 00211 | TINTA |
| 00818 | TINTA |
| 07302 | TINTA DE ASSOALHO |
| 02101 | TINTA DE CALCADO |
| 00819 | TINTA DE PINTURA |
| 00801 | TINTA DE TECIDO |
| 02614 | TINTA PARA CABELO |
| 01503 | TINTA PARA PINTURA DE TELA |
| 30206 | TINTURA DE ROMA |
| 00802 | TINTURA DE ROUPA |
| 00501 | TINTURARIA E LAVANDERIA |
| 01502 | TIQUETE-ALIMENTACAO |
| 01503 | TIQUETE-REFEICAO |
| 07102 | TIRA-FERRUGEM |
| 03709 | TIRA-GOSTO |
| 02409 | TIRA-MANCHA |
| 04201 | TITULO DE CAPITALIZACAO (AQUISICAO) |
| 04401 | TITULO DE CAPITALIZACAO (RESGATE) |
| 02102 | TITULO DE CLUBE (AQUISICAO) |
| 05301 | TITULO DE TURISMO (MENSALIDADE) |
| 02703 | TITULO PROTESTADO |
| 00302 | TOALHA DE BANHO |
| 03801 | TOALHA DE MAO |
| 00603 | TOALHA DE MESA DE PLASTICO |
| 00502 | TOALHA DE MESA DE TECIDO |
| 04701 | TOALHA DE PAPEL |
| 03901 | TOALHA DE PRAIA |
| 00305 | TOALHA DE ROSTO |
| 00303 | TOALHA INFANTIL DE BANHO |
| 03101 | TOCA-DISCOS A LASER |
| 03601 | TOCA-DISCOS DE AGULHA |
| 20601 | TOCADOR DE MP PLAYER |
| 20605 | TOCADOR DE MP3/MP4/MP5 |
| 03002 | TOCA-FITAS |
| 00811 | TOCA-FITAS |
| 00811 | TODDY |
| 10311 | TODDY DIET |
| 10333 | TODDY DIETETICO |
| 00808 | TODDY INSTANTANEO |
| 10308 | TODDY INSTANTANEO DIET |
| 10330 | TODDY INSTANTANEO DIETETICO |
| 10208 | TODDY INSTANTANEO LIGHT |
| 10211 | TODDY LIGHT |
| 00810 | TODDY VITAMINADO |
| 10310 | TODDY VITAMINADO DIET |
| 10332 | TODDY VITAMINADO DIETETICO |
| 10210 | TODDY VITAMINADO LIGHT |
| 03302 | TODDYNHO |
| 00822 | TODDYNHO EM PO |
| 10322 | TODDYNHO EM PO DIET |
| 10344 | TODDYNHO EM PO DIETETICO |
| 10222 | TODDYNHO EM PO LIGHT |
| 03112 | TODDYNHO LIQUIDO |
| 03402 | TOFU |
| 04504 | TOLDO DE LONA |
| 04504 | TOLDO DE LONA |
| 04502 | TOLDO PLASTICO |
| 04502 | TOLDO PLASTICO |
| 05101 | TOMATE |
| 05107 | TOMATE CAQUI |
| 07907 | TOMATE CAQUI ORGANICO |
| 05108 | TOMATE CEREJA |
| 01002 | TOMATE COM PELE EM CONSERVA |
| 10302 | TOMATE DESIDRATADO |
| 05105 | TOMATE JAPONES |
| 07905 | TOMATE JAPONES ORGANICO |
| 05103 | TOMATE MACA |
| 07903 | TOMATE MACA ORGANICO |
| 07901 | TOMATE ORGANICO |
| 05102 | TOMATE PAULISTA |
| 07902 | TOMATE PAULISTA ORGANICO |
| 05104 | TOMATE PERA |
| 07904 | TOMATE PERA ORGANICO |
| 05109 | TOMATE SALADA |
| 10301 | TOMATE SECO |
| 01001 | TOMATE SEM PELE EM CONSERVA |
| 05106 | TOMATE VERDE |
| 07906 | TOMATE VERDE ORGANICO |
| 08701 | TOMILHO EM FOLHA (TEMPERO INDUSTRIALIZADO) |
| 01401 | TOMOGRAFIA |
| 02310 | TONYU |
| 05001 | TOP |
| 01101 | TOPOGRAFO |
| 02303 | TORANJA |
| 00915 | TORNEIRA DE FILTRO DE PLASTICO |
| 10001 | TORNEIRA ELETRICA |
| 01001 | TORNEIRA, CANO E MATERIAL HIDRAULICO EM GERAL |
| 01001 | TORNEIRA, CANO E MATERIAL HIDRAULICO EM GERAL |
| 01643 | TORNO DE MESA |
| 00108 | TORNOZELEIRA |
| 04113 | TORNOZELEIRA ORTOPEDICA |
| 00311 | TORRADA COM OVO PARA VIAGEM |
| 00310 | TORRADA COM PRESUNTO PARA VIAGEM |
| 00406 | TORRADA COM QUEIJO, PRESUNTO OU OVO |
| 00309 | TORRADA COM QUEIJO, PRESUNTO, OVO, ETC. PARA VIAGEM |
| 01901 | TORRADA DE QUALQUER PAO |
| 06901 | TORRADA DE QUALQUER PAO LIGHT |
| 01903 | TORRADA DE QUALQUER TIPO |
| 06903 | TORRADA DE QUALQUER TIPO LIGHT |
| 11101 | TORRADA LIGHT |
| 00807 | TORRADEIRA DE PAO NAO-ELETRICA |
| 01701 | TORRADEIRA ELETRICA |
| 03204 | TORRAO DE AMENDOIM |
| 13104 | TORRAO DE AMENDOIM DIET |
| 13113 | TORRAO DE AMENDOIM DIETETICO |
| 00210 | TORRESMO PARA VIAGEM |
| 05901 | TORRONE |
| 04306 | TORTA DOCE |
| 07801 | TORTA DOCE DE QUALQUER SABOR DIET |
| 07802 | TORTA DOCE DE QUALQUER SABOR DIETETICA |
| 09605 | TORTA DOCE LIGHT |
| 05801 | TORTA NAO-ESPECIFICADA |
| 09601 | TORTA NAO-ESPECIFICADA DIET |
| 09602 | TORTA NAO-ESPECIFICADA DIETETICA |
| 09501 | TORTA NAO-ESPECIFICADA LIGHT |
| 03731 | TORTA SALGADA |
| 09606 | TORTA SALGADA LIGHT |
| 03501 | TORTAS DOCES DE QUALQUER SABOR |
| 07101 | TORTAS DOCES DE QUALQUER SABOR DIET |
| 07102 | TORTAS DOCES DE QUALQUER SABOR DIETETICAS |
| 07001 | TORTAS DOCES DE QUALQUER SABOR LIGHT |
| 03601 | TORTAS SALGADAS DE QUALQUER SABOR |
| 07201 | TORTAS SALGADAS DE QUALQUER SABOR LIGHT |
| 02502 | TOSA DE PELOS DE ANIMAL DOMESTICO |
| 01901 | TOSADEIRA DE ANIMAL DOMESTICO |
| 02311 | TOSTINE (BISCOITO) |
| 10511 | TOSTINE (BISCOITO) DIET |
| 10551 | TOSTINE (BISCOITO) DIETETICO |
| 10411 | TOSTINE (BISCOITO) LIGHT |
| 02102 | TOTO |
| 02901 | TOTOLEC |
| 05904 | TOUCA |
| 00703 | TOUCA DE CABELO |
| 00111 | TOUCA DE NATACAO |
| 03801 | TOUCINHO |
| 01001 | TOUCINHO DE PORCO DEFUMADO |
| 01901 | TOUCINHO DE PORCO SALGADO |
| 03803 | TOUCINHO DE TORRESMO |
| 01003 | TOUCINHO DEFUMADO |
| 03802 | TOUCINHO FRESCO |
| 01902 | TOUCINHO SALGADO |
| 05002 | TRABALHOS EVENTUAIS |
| 44101 | TRACAJA |
| 44106 | TRACAJA CABECADA |
| 44501 | TRACAJA EM FILE CONGELADO |
| 44301 | TRACAJA EM FILE FRESCO |
| 44201 | TRACAJA EM POSTAS |
| 44401 | TRACAJA SALGADA |
| 03001 | TRADUTOR |
| 01501 | TRADUTOR JURAMENTADO |
| 00607 | TRAILERS |
| 00701 | TRAILLER |
| 00603 | TRAINING |
| 00803 | TRAINNING |
| 00903 | TRAINNING |
| 00808 | TRANCA |
| 01002 | TRANSFERENCIA DE CONSORCIO |
| 01001 | TRANSFERENCIA DE CONSORCIO DE VEICULO |
| 00907 | TRANSFERENCIA DE PLACA DE VEICULO (TAXA) |
| 00905 | TRANSFERENCIA DE VEICULO (TAXA) |
| 02601 | TRANSFERENCIA INTERBANCARIA |
| 00222 | TRANSFERIDOR |
| 00251 | TRANSPARENCIA |
| 02301 | TRANSPORTE ALTERNATIVO |
| 01605 | TRANSPORTE ALTERNATIVO |
| 00201 | TRANSPORTE DE ANIMAIS |
| 00501 | TRANSPORTE DE MERCADORIAS |
| 00514 | TRANSPORTE DE VEICULOS |
| 00901 | TRANSPORTE ESCOLAR |
| 03401 | TRANSPORTE SUBSIDIADO |
| 04819 | TRAQUINAS (BISCOITO RECHEADO) |
| 02701 | TRATADOR DE ANIMAIS |
| 03402 | TRATAMENTO ACUPUNTURAL |
| 00403 | TRATAMENTO AMBULATORIAL |
| 03502 | TRATAMENTO DE AIDS |
| 02402 | TRATAMENTO DE CANAL DENTARIO |
| 02502 | TRATAMENTO DE ENFERMAGEM |
| 02401 | TRATAMENTO DE SAUDE DE ANIMAL DOMESTICO |
| 02401 | TRATAMENTO DENTARIO |
| 02802 | TRATAMENTO DIETETICO |
| 03102 | TRATAMENTO FISIOTERAPICO |
| 03101 | TRATAMENTO FISIOTERAPICO E DE TERAPIA OCUPACIONAL |
| 03201 | TRATAMENTO FONOAUDIOLOGICO |
| 03303 | TRATAMENTO HIDROTERAPICO |
| 03302 | TRATAMENTO MASSAGISTICO |
| 00508 | TRATAMENTO PARA IMPLANTE DE CABELO |
| 03002 | TRATAMENTO PSICOLOGICO |
| 02002 | TRATAMENTO QUIMIOTERAPICO |
| 02309 | TRATOR |
| 00116 | TRAVA DE CHUTEIRA |
| 00301 | TRAVESSA DE VIDRO OU LOUCA |
| 02301 | TRAVESSEIRO |
| 00201 | TREM |
| 00201 | TREM |
| 02701 | TREMOCO |
| 02703 | TREMOCO AMARELO |
| 02702 | TREMOCO BRANCO |
| 01660 | TRENA METRICA |
| 00308 | TRICICLO |
| 05001 | TRICOTEIRA |
| 00901 | TRIGO EM GRAO |
| 00902 | TRIGO INTEGRAL |
| 01205 | TRIGO PARA QUIBE |
| 01203 | TRIGUILHO |
| 04301 | TRILHO DE MESA |
| 01109 | TRILHO METALICO |
| 01109 | TRILHO METALICO |
| 02203 | TRILICHE |
| 03001 | TRIMANIA |
| 02401 | TRIPA BOVINA |
| 04501 | TRIPA SUINA |
| 00816 | TRIPE DE PANELAS DE METAL |
| 07501 | TRITURADOR ELETRICO |
| 15606 | TRITURADORA |
| 00203 | TROCA DE OLEO LUBRIFICANTE |
| 01814 | TROCA-FRALDAS |
| 02401 | TROFEU |
| 01501 | TROMBONE, TROMPETE |
| 00904 | TRUFA |
| 10504 | TRUFA DIET |
| 10508 | TRUFA DIETETICA |
| 10404 | TRUFA LIGHT |
| 03605 | TSUQUEMONO |
| 10505 | TSUQUEMONO LIGHT |
| 02303 | TUBAINA |
| 01802 | TUBAINA |
| 13109 | TUBAINA LIGHT |
| 01902 | TUBAINA LIGHT |
| 03734 | TUBERCULOS |
| 00709 | TUBETE DE CHOCOLATE |
| 04208 | TUBO OU CILINDRO DE OXIGENIO (DESPESA COM SAUDE) |
| 01105 | TUCUM |
| 01108 | TUCUMA |
| 04103 | TUCUPI COM PIMENTA |
| 07101 | TUCUPI EM CALDO SEM PIMENTA |
| 04102 | TUCUPI EM MOLHO COM PIMENTA |
| 04202 | TUTI FRUTI REFRIGERANTE |
| 08702 | TUTI FRUTI REFRIGERANTE LIGHT |
| 07001 | TUTU PARA VIAGEM |
| 00701 | TV POR ASSINATURA (MENSALIDADE/PACOTE) |
| 01001 | TV POR ASSINATURA E INTERNET (PACOTE) |
| 07702 | TWEETER |
| 01709 | UACAI |
| 01001 | UISQUE |
| 00801 | ULTRALEVE |
| 01601 | ULTRASSONOGRAFIA |
| 07505 | UMARI |
| 07504 | UMARI AMARELO |
| 07506 | UMARI ROXO |
| 01808 | UMBIGUEIRO |
| 07101 | UMBU |
| 09901 | UMIDIFICADOR DE AR |
| 03001 | UNDERBERG |
| 00403 | UNHA POSTICA |
| 04301 | UNIFORME DE BALE |
| 03002 | UNIFORME DE JUDO (QUIMONO) |
| 03602 | UNIFORME DE JUDO (QUIMONO) |
| 03201 | UNIFORME DE TRABALHO |
| 04201 | UNIFORME DE TRABALHO |
| 01001 | UNIFORME ESCOLAR |
| 02801 | UNIFORME PARA CORAL |
| 02802 | UNIFORME PARA CORAL DE IGREJA |
| 00307 | URNA FUNERARIA |
| 04603 | UROLOGICO |
| 00227 | URSO DE PELUCIA |
| 61102 | URUA ( MOLUSCO) |
| 01203 | URUCU |
| 01204 | URUCUM |
| 00502 | USO DE INTERNET |
| 30503 | UTENSILIO PARA COLETAR FEZES E URINA |
| 02201 | UTENSILIOS DE ACRILICO |
| 02101 | UTENSILIOS DE BARRO |
| 02501 | UTENSILIOS DE BORRACHA |
| 02801 | UTENSILIOS DE FIBRA VEGETAL |
| 02401 | UTENSILIOS DE ISOPOR |
| 01601 | UTENSILIOS DE MADEIRA |
| 00901 | UTENSILIOS DE PLASTICO |
| 03901 | UVA |
| 03913 | UVA BENITAKA |
| 08513 | UVA BENITAKA ORGANICA |
| 03911 | UVA BRANCA |
| 08511 | UVA BRANCA ORGANICA |
| 03908 | UVA ITALIA |
| 08508 | UVA ITALIA ORGANICA |
| 03903 | UVA MOSCATEL |
| 08503 | UVA MOSCATEL ORGANICA |
| 03906 | UVA NACIONAL |
| 08506 | UVA NACIONAL ORGANICA |
| 03902 | UVA NIAGARA |
| 08502 | UVA NIAGARA ORGANICA |
| 06503 | UVA ORGANICA |
| 08501 | UVA ORGANICA |
| 03912 | UVA PASSA |
| 09001 | UVA PASSA ORGANICA |
| 03904 | UVA PRETA |
| 08504 | UVA PRETA ORGANICA |
| 03914 | UVA RED GLOBE |
| 08514 | UVA RED GLOBE ORGANICA |
| 03905 | UVA ROSA |
| 08505 | UVA ROSA ORGANICA |
| 03909 | UVA ROXA |
| 08509 | UVA ROXA ORGANICA |
| 03910 | UVA RUBI |
| 08510 | UVA RUBI ORGANICA |
| 03915 | UVA THOMPSON |
| 08512 | UVA THOMPSON ORGANICA |
| 03907 | UVA VERDE |
| 08507 | UVA VERDE ORGANICA |
| 06101 | UXI |
| 08301 | VACA ATOLADA PARA VIAGEM |
| 04808 | VACINA ANTIOFIDICO |
| 04806 | VACINA ANTIPOLIO |
| 04803 | VACINA ANTITETANICA |
| 04802 | VACINA BCG |
| 04804 | VACINA DE HEPATITE B |
| 02402 | VACINA PARA ANIMAL DOMESTICO |
| 04805 | VACINA PARA OSTEOPOROSE |
| 04807 | VACINA TRIPLICE |
| 00801 | VACINACAO |
| 04801 | VACINAS |
| 02701 | VAGA CATIVA (ESTACIONAMENTO) |
| 00701 | VAGA CATIVA DE ESTACIONAMENTO DE VEICULO |
| 00702 | VAGA DE ESTACIONAMENTO DE VEICULO |
| 05201 | VAGEM |
| 05202 | VAGEM MANTEIGA |
| 08601 | VAGEM ORGANICA |
| 01602 | VALE-TRANSPORTE |
| 00401 | VALISE |
| 09401 | VALOR DE OUTRO IMOVEL ADQUIRIDO EM PRIMEIRA LOCACAO (NOVO E VALOR TOTAL) |
| 09501 | VALOR DE OUTRO IMOVEL ADQUIRIDO USADO (VALOR TOTAL) |
| 02701 | VALOR DO IMOVEL ADQUIRIDO EM PRIMEIRA LOCACAO A PRAZO |
| 02601 | VALOR DO IMOVEL ADQUIRIDO EM PRIMEIRA LOCACAO A VISTA |
| 02901 | VALOR DO IMOVEL ADQUIRIDO USADO A PRAZO |
| 02801 | VALOR DO IMOVEL ADQUIRIDO USADO A VISTA |
| 04304 | VALVULA DE BOTIJAO DE GAS |
| 02304 | VAN |
| 00903 | VANILINA |
| 02414 | VANISH |
| 09001 | VAPORIZADOR ELETRICO |
| 06801 | VAPORIZADOR ELETRICO CONTRA INSETO |
| 00517 | VARA DE PESCA |
| 04101 | VARAL DE APARTAMENTO |
| 04102 | VARAL DE CORDINHAS REGULAVEL |
| 00401 | VAREJAO |
| 02616 | VASELINA |
| 00905 | VASILHAME PLASTICO DE MANTIMENTOS |
| 01806 | VASO PARA PLANTA |
| 00204 | VASO SANITARIO |
| 00204 | VASO SANITARIO |
| 01002 | VASODILATADOR/PRESSAO ARTERIAL |
| 00401 | VASSOURA DE QUALQUER TIPO |
| 00403 | VASSOURA DE VASO SANITARIO |
| 00405 | VASSOURINHA |
| 03809 | VATAPA |
| 02801 | VATAPA PARA VIAGEM |
| 01011 | VAZIO (CARNE BOVINA) |
| 06702 | VEADO VIVO |
| 01701 | VEICULO DE TRACAO ANIMAL E ANIMAL DE TRACAO |
| 06302 | VEJA |
| 06701 | VELA DE ANIVERSARIO |
| 01101 | VELA DE FILTRO |
| 05401 | VELA DE ILUMINACAO |
| 02001 | VELA DECORATIVA |
| 00303 | VELOCIPEDE |
| 00304 | VENDA DE ACOES |
| 04705 | VENDA DE ANIMAL |
| 04702 | VENDA DE ANIMAL DE TRACAO |
| 04704 | VENDA DE ANIMAL DOMESTICO |
| 03630 | VENDA DE ANTENA PARABOLICA |
| 03631 | VENDA DE APARELHO CELULAR |
| 03622 | VENDA DE APARELHO DE PRESSAO (TENSIOMETRO) |
| 03614 | VENDA DE APARELHO ELETRODOMESTICO |
| 03607 | VENDA DE APARELHO SONORO |
| 00701 | VENDA DE AUTOMOVEL |
| 01601 | VENDA DE BARCO |
| 04801 | VENDA DE BICICLETA |
| 03627 | VENDA DE BOTIJAO DE GAS |
| 03626 | VENDA DE BUJAO DE GAS |
| 03605 | VENDA DE CALCA |
| 03604 | VENDA DE CAMISA |
| 04703 | VENDA DE CARROCA |
| 03615 | VENDA DE CHURRASQUEIRA |
| 03609 | VENDA DE COLCHA |
| 03629 | VENDA DE CONSORCIO, CARNE E OUTROS |
| 03601 | VENDA DE CONSORCIO, CARNET E OUTROS |
| 03621 | VENDA DE FRUTAS |
| 03625 | VENDA DE FURADEIRA |
| 03616 | VENDA DE GAIOLA |
| 00801 | VENDA DE IMOVEL (EXCETO TERRENO) |
| 03619 | VENDA DE INSTRUMENTO MUSICAL |
| 03617 | VENDA DE JOIA |
| 04901 | VENDA DE LINHA TELEFONICA |
| 03633 | VENDA DE MAQUINA AGRICOLA |
| 03611 | VENDA DE MAQUINA DE COSTURA |
| 04601 | VENDA DE MATERIAL DE CONSTRUCAO |
| 03618 | VENDA DE MICROCOMPUTADOR |
| 03635 | VENDA DE MIUDEZAS EM GERAL |
| 00901 | VENDA DE MOTOCICLETA |
| 03612 | VENDA DE MOVEL |
| 03628 | VENDA DE PANELA |
| 03620 | VENDA DE PECAS DE VEICULO |
| 03634 | VENDA DE PRODUTOS AGRICOLAS |
| 03624 | VENDA DE REDE |
| 03602 | VENDA DE RELOGIO |
| 03603 | VENDA DE ROUPA |
| 03610 | VENDA DE TECIDO |
| 03608 | VENDA DE TELEVISAO |
| 00601 | VENDA DE TERRENO |
| 04602 | VENDA DE TIJOLO |
| 04701 | VENDA DE VEICULO DE TRACAO ANIMAL OU ANIMAL DE TRACAO |
| 03613 | VENDA DE VIDEOCASSETE |
| 03606 | VENDA DE VIDEOGAME |
| 05202 | VENENO CONTRA CUPIM (CUPINICIDA) |
| 05202 | VENENO CONTRA CUPIM (CUPINICIDA) |
| 02702 | VENENO PARA RATO |
| 04201 | VENTILADOR |
| 01801 | VENTILADOR E/OU CIRCULADOR DE AR |
| 00803 | VERDURA COZIDA OU FRITA PARA VIAGEM |
| 00806 | VERDURA FRITA PARA VIAGEM |
| 01504 | VERMIFUGO |
| 01301 | VERMUTE |
| 00802 | VERNIZ |
| 00802 | VERNIZ |
| 01504 | VERNIZ PARA PINTURA DE TELA |
| 01401 | VESTIDO |
| 01701 | VESTIDO |
| 01702 | VESTIDO DE DAMA DE HONRA |
| 02501 | VESTIDO DE NOIVA |
| 02601 | VETERINARIO |
| 05102 | VEU DE CABECA |
| 00109 | VIANDA PARA VIAGEM |
| 02703 | VIANDADA SUINA |
| 02607 | VIBRADOR |
| 02602 | VIBRADOR EROTICO |
| 03701 | VIDEO GAME |
| 02601 | VIDEOCASSETE |
| 00401 | VIDEOCASSETE (ALUGUEL) |
| 00502 | VIDEOCLUBE (TAXA) |
| 01504 | VIDEOGAME (ALUGUEL DA HORA) |
| 01301 | VIDEOGAME (ALUGUEL) |
| 01503 | VIDEOGAME (FICHA) |
| 05402 | VIDEOKE |
| 02008 | VIDEOKE (INGRESSO) |
| 02603 | VIDEOTECAS (INGRESSO) |
| 01306 | VIDRACEIRO |
| 01306 | VIDRACEIRO |
| 00701 | VIDRO |
| 00701 | VIDRO |
| 03002 | VIDRO (PECA DE DECORACAO) |
| 68101 | VIEIRA |
| 68401 | VIEIRA SALGADA |
| 68201 | VIEIRA SEM CASCA |
| 04303 | VIGA DE CIMENTO OU CONCRETO |
| 04303 | VIGA DE CIMENTO OU CONCRETO |
| 00701 | VIGIA |
| 02501 | VIGILANCIA ELETRONICA |
| 04304 | VIGILANTES DO PESO (PROFISSIONAIS VOLANTES) |
| 60112 | VILA FRANCA |
| 12401 | VINAGRE BALSAMICO |
| 03301 | VINAGRE DE ALCOOL |
| 11901 | VINAGRE DE ALCOOL E VINHO (COMPOSTO) |
| 08901 | VINAGRE DE ARROZ |
| 03302 | VINAGRE DE CANA |
| 12301 | VINAGRE DE LIMAO |
| 03501 | VINAGRE DE MACA |
| 09001 | VINAGRE DE MILHO |
| 03402 | VINAGRE DE UVA |
| 03401 | VINAGRE DE VINHO |
| 08801 | VINAGRE NAO-ESPECIFICADO |
| 01907 | VINAGREIRA |
| 04801 | VINHO |
| 02415 | VINHO |
| 02410 | VINHO BRANCO |
| 03610 | VINHO BRANCO ORGANICO |
| 01716 | VINHO DE ACAI |
| 02302 | VINHO DE BURITI |
| 02412 | VINHO DE CATUABA |
| 03612 | VINHO DE CATUABA ORGANICO |
| 02417 | VINHO DE JURUBEBA |
| 03617 | VINHO DE JURUBEBA ORGANICO |
| 02604 | VINHO DE PATAUA |
| 02414 | VINHO DE UVA |
| 02401 | VINHO DE UVA E OUTROS |
| 03601 | VINHO DE UVA E OUTROS ORGANICO |
| 03614 | VINHO DE UVA ORGANICO |
| 02407 | VINHO LICOROSO |
| 03607 | VINHO LICOROSO ORGANICO |
| 02406 | VINHO MOSCATEL |
| 03606 | VINHO MOSCATEL ORGANICO |
| 06701 | VINHO ORGANICO |
| 03615 | VINHO ORGANICO |
| 02408 | VINHO QUINADO |
| 03608 | VINHO QUINADO ORGANICO |
| 02402 | VINHO ROSE |
| 03602 | VINHO ROSE ORGANICO |
| 02405 | VINHO SECO |
| 03605 | VINHO SECO ORGANICO |
| 02413 | VINHO SUAVE |
| 03613 | VINHO SUAVE ORGANICO |
| 02403 | VINHO TINTO |
| 03603 | VINHO TINTO ORGANICO |
| 02404 | VINHO VERDE |
| 03604 | VINHO VERDE ORGANICO |
| 00401 | VIOLAO, VIOLA |
| 00115 | VIOLETA |
| 30207 | VIOLETA GENCIANA |
| 02801 | VIOLINO |
| 01801 | VISCERA BOVINA NAO-ESPECIFICADA |
| 08101 | VISCERA DE CARNEIRO |
| 02001 | VISCERA DE PORCO SALGADA |
| 03901 | VISCERA SUINA NAO-ESPECIFICADA |
| 05903 | VISEIRA |
| 02105 | VITAFLOCOS |
| 00802 | VITAMILHO |
| 00701 | VITAMINA |
| 02002 | VITAMINA B12 |
| 02005 | VITAMINA C |
| 14701 | VITAMINA CONCENTRADA (SHAKE) |
| 00706 | VITAMINA DE ABACATE |
| 00506 | VITAMINA DE ABACATE PARA VIAGEM |
| 00703 | VITAMINA DE BANANA |
| 00704 | VITAMINA DE BANANA COM AVEIA |
| 00504 | VITAMINA DE BANANA COM AVEIA PARA VIAGEM |
| 00503 | VITAMINA DE BANANA PARA VIAGEM |
| 00702 | VITAMINA DE FRUTAS OU VEGETAIS |
| 00502 | VITAMINA DE FRUTAS OU VEGETAIS PARA VIAGEM |
| 00709 | VITAMINA DE MACA |
| 00509 | VITAMINA DE MACA PARA VIAGEM |
| 00705 | VITAMINA DE MAMAO |
| 00505 | VITAMINA DE MAMAO PARA VIAGEM |
| 00707 | VITAMINA DE MORANGO |
| 00507 | VITAMINA DE MORANGO PARA VIAGEM |
| 00708 | VITAMINA MISTA |
| 00508 | VITAMINA MISTA PARA VIAGEM |
| 00501 | VITAMINA PARA VIAGEM |
| 02001 | VITAMINAS |
| 00917 | VITASUCO (COPO DE LIQUIDIFICADOR) |
| 00404 | VITAVENA |
| 02605 | VITELADA |
| 01501 | VITRIFICACAO (SINTECO, POLIURETANO, ETC.) |
| 01501 | VITRIFICACAO (SINTECO, POLIURETANO, ETC.) |
| 00403 | VITRINE |
| 19102 | VOADOR (ANDADOR) |
| 04701 | VODKA |
| 00801 | VODKA |
| 00406 | VOLEIBOL (INGRESSO) |
| 04807 | WAFFER (BISCOITO) |
| 08407 | WAFFER (BISCOITO) DIET |
| 08420 | WAFFER (BISCOITO) DIETETICO |
| 08307 | WAFFER (BISCOITO) LIGHT |
| 01801 | WALK MACHINE |
| 03803 | WALKMAN |
| 08701 | WATER PEACH |
| 04601 | WHISKY |
| 01002 | WHISKY |
| 00501 | WINDSURF |
| 00327 | X SALADA |
| 00209 | XADREZ |
| 00433 | XAMPU |
| 04704 | XAROPE DE FRUTA OU VEGETAL EM CAIXA |
| 11704 | XAROPE DE FRUTA OU VEGETAL EM CAIXA DIET |
| 11708 | XAROPE DE FRUTA OU VEGETAL EM CAIXA DIETETICO |
| 11604 | XAROPE DE FRUTA OU VEGETAL EM CAIXA LIGHT |
| 05102 | XAROPE DE FRUTA OU VEGETAL EM LATA |
| 11902 | XAROPE DE FRUTA OU VEGETAL EM LATA DIET |
| 11904 | XAROPE DE FRUTA OU VEGETAL EM LATA DIETETICO |
| 11802 | XAROPE DE FRUTA OU VEGETAL EM LATA LIGHT |
| 05302 | XAROPE DE FRUTA OU VEGETAL EM SACO PLASTICO |
| 12102 | XAROPE DE FRUTA OU VEGETAL EM SACO PLASTICO DIET |
| 12104 | XAROPE DE FRUTA OU VEGETAL EM SACO PLASTICO DIETETICO |
| 12002 | XAROPE DE FRUTA OU VEGETAL EM SACO PLASTICO LIGHT |
| 04702 | XAROPE DE FRUTA OU VEGETAL ENCARTONADO |
| 11702 | XAROPE DE FRUTA OU VEGETAL ENCARTONADO DIET |
| 11706 | XAROPE DE FRUTA OU VEGETAL ENCARTONADO DIETETICO |
| 11602 | XAROPE DE FRUTA OU VEGETAL ENCARTONADO LIGHT |
| 02202 | XAROPE DE FRUTA OU VEGETAL ENGARRAFADO |
| 11502 | XAROPE DE FRUTA OU VEGETAL ENGARRAFADO DIET |
| 11504 | XAROPE DE FRUTA OU VEGETAL ENGARRAFADO DIETETICO |
| 11402 | XAROPE DE FRUTA OU VEGETAL ENGARRAFADO LIGHT |
| 10204 | XAROPE DE FRUTA OU VEGETAL ORGANICO EM CAIXA |
| 10302 | XAROPE DE FRUTA OU VEGETAL ORGANICO EM LATA |
| 10402 | XAROPE DE FRUTA OU VEGETAL ORGANICO EM SACO PLASTICO |
| 10202 | XAROPE DE FRUTA OU VEGETAL ORGANICO ENCARTONADO |
| 10102 | XAROPE DE FRUTA OU VEGETAL ORGANICO ENGARRAFADO |
| 02204 | XAROPE DE GROSELHA |
| 02203 | XAROPE DE GUARANA |
| 07305 | XAXADO BOVINO |
| 01805 | XAXIM |
| 00108 | XEREM DE ARROZ |
| 04308 | XEREM DE ARROZ ORGANICO |
| 00707 | XEREM DE MILHO |
| 00602 | XEROX |
| 00401 | XICARA COMUM |
| 00225 | XIQUE-XIQUE |
| 00225 | XISBAG |
| 10025 | XISBAG DIET |
| 10065 | XISBAG DIETETICO |
| 01702 | XODO DA BAHIA |
| 13107 | XODO DA BAHIA LIGHT |
| 08502 | XODO DA BAHIA LIGHT |
| 03708 | XOXO |
| 01406 | YAKISSOBA (SOPA DE LEGUMES DESIDRATADA) |
| 06106 | YAKISSOBA (SOPA DE LEGUMES DESIDRATADA) LIGHT |
| 09201 | YAKISSOBA PARA VIAGEM |
| 02801 | YAKULT |
| 01301 | YAKULT DE QUALQUER SABOR |
| 01226 | YOKAN (DOCE DE FEIJAO EM PASTA) |
| 43104 | ZABUMBA |
| 00702 | ZELADOR |
| 01805 | ZERO CAL |
| 05902 | ZURITA |
