# Supplementary material for: Can a poverty-reducing and progressive tax and transfer system hurt the poor?
Source: J Dev Econ. 2016 Sep;122:63–75. doi: 10.1016/j.jdeveco.2016.04.001 (PMC4988485; doi:10.1016/j.jdeveco.2016.04.001)
Supplement: Supplementary file 1 — Supplementary material. [file mmc1.zip › documentation/POF0809/Descriç¦o dos Registros POF 2008-2009.doc]

#  DOCUMENTAÇÃO DOS MICRODADOS

# PESQUISA DE ORÇAMENTOS FAMILIARES 2008-2009

###### DESCRIÇÃO DOS REGISTROS

```
 Dados Gerais do Dicionário:

Formato do Registro: Fixo por Tipo
Número de Registros: 15
Tamanho Máximo: 286
Data da Última Atualização: 26/08/2010
Registros Descritos:

      01 – DOMICÍLIO - POF1 / QUADRO2
          Tamanho Máximo: 131

      02 – PESSOAS - POF1 / QUADROS 3 E 4
          Tamanho Máximo: 212

      03 – PESSOAS – IMPUTAÇÃO – POF 1 / QUADRO 4
          Tamanho Máximo: 104

      04 – CONDIÇÕES DE VIDA – POF 6
          Tamanho Máximo: 94

      05 – INVENTÁRIO DE BENS DUVÁVEIS - POF2 / QUADRO 14
          Tamanho Máximo: 107

      06 - DESPESAS DE 90 DIAS - POF2 / QUADROS 6 A 9
          Tamanho Máximo: 145

      07 - DESPESAS DE 12 MESES - POF2 / QUADROS 10 A 13
          Tamanho Máximo: 149

      08 - OUTRAS DESPESAS – POF2 / QUADROS 15 A 18
          Tamanho Máximo: 146

      09 – DESPESAS COM SERVIÇOS DOMESTICOS - POF2 / QUADRO 19
          Tamanho Máximo: 195

      10 – ALUGUEL ESTIMADO - POF1 / QUADRO 2
          Tamanho Máximo: 149

      11 - CADERNETA DE DESPESA - POF3
          Tamanho Máximo: 147

      12 - DESPESA INDIVIDUAL - POF4 / QUADROS 22 A 50
          Tamanho Máximo: 147

      13 - DESPESA COM VEICULOS - POF4 / QUADRO 51
          Tamanho Máximo: 148

      14 – RENDIMENTOS E DEDUÇÕES - POF5 / QUADRO 53
          Tamanho Máximo: 283

      15 - OUTROS RENDIMENTOS - POF5 / QUADROS 54 A 57
          Tamanho Máximo: 192


Classificações Utilizadas:


100 – TIPO DE DOMICÍLIO
101 – MATERIAL QUE PREDOMINA NAS PAREDES EXTERNAS
102 – MATERIAL QUE PREDOMINA NA COBERTURA
103 – MATERIAL QUE PREDOMINA NO PISO
104 – EXISTÊNCIA DE ÁGUA CANALIZADA
105 – PROVENIÊNCIA DA ÁGUA
106 – ESCOADOURO SANITÁRIO
107 – CONDIÇÃO DE OCUPAÇÃO
108 – TEMPO DE ALUGUEL
109 – TIPO DE CONTRATO DE ALUGUEL
110 – EXISTÊNCIA DE PAVIMENTAÇÃO NA RUA
111 – IMPUTAÇÃO – QUANTIDADE DE CÔMODOS
112 – IMPUTAÇÃO – QUANTIDADE DE BANHEIROS
113 – IMPUTAÇÃO - ESCOADOURO SANITÁRIO
200 – CONDIÇÃO NA UNIDADE DE CONSUMO
201 – CONDIÇÃO NA FAMÍLIA
202 - CONDIÇÃO DE PRESENÇA
203 – SEXO
204 – SABE LER E ESCREVER
205 – FREQUÊNCIA À ESCOLA OU CRECHE
206 – CURSO QUE FREQUENTA
207 – QUAL A DURAÇÃO DO CURSO DE PRIMEIRO GRAU
208 – SÉRIE QUE FREQUENTA
209 – CURSO MAIS ELEVADO QUE FREQUENTOU
210 – QUAL ERA A DURAÇÃO DO CURSO DE PRIMEIRO GRAU
211 – ÚLTIMA SÉRIE CONCLUÍDA
212 – CONCLUSÃO DO CURSO MAIS ELEVADO
213 – ANOS DE ESTUDO
214 – COR OU RAÇA
215 – ORÇAMENTO TRABALHO E/OU RENDIMENTO
216 – ORÇAMENTO DESPESA
217 – TEM CARTÃO DE CRÉDITO
218 – TITULAR DO CARTÃO DE CRÉDITO
219 – TEM CHEQUE ESPECIAL
220 – TITULAR DA CONTA CORRENTE
221 – ESTA GRÁVIDA
300 – IMPUTAÇÃO - SABE LER E ESCREVER
301 – IMPUTAÇÃO - FREQUENCIA À ESCOLA OU CRECHE
302 – IMPUTAÇÃO - CURSO QUE FREQUENTA
303 – IMPUTAÇÃO - QUAL A DURAÇÃO DO CURSO DE PRIMEIRO GRAU
304 – IMPUTAÇÃO - SÉRIE QUE FREQUENTA
305 – IMPUTAÇÃO - CURSO MAIS ELEVADO QUE FREQUENTOU
306 – IMPUTAÇÃO - QUAL ERA A DURAÇÃO DO CURSO DE PRIMEIRO GRAU
307 – IMPUTAÇÃO - ÚLTIMA SÉRIE CONCLUÍDA
308 – IMPUTAÇÃO - CONCLUSÃO DO CURSO MAIS ELEVADO
309 – IMPUTAÇÃO - TEM CARTÃO DE CRÉDITO
310 – IMPUTAÇÃO - TITULAR DO CARTÃO DE CRÉDITO
311 – IMPUTAÇÃO - TEM CHEQUE ESPECIAL
312 – IMPUTAÇÃO - TITULAR DA CONTA CORRENTE
400 – CÓDIGO DA RENDA FAMILIAR
401 – QUANTIDADE DE ALIMENTO
402 – TIPO DE ALIMENTO
500 – CÓDIGO DO INSS
501 – CÓDIGO DA ESPÉCIE
600 - NÚMERO DO GRUPO DE DESPESA
700 – TIPO DE TRABALHO
701 – POSIÇÃO NA OCUPAÇÃO
702 – FORMA DO ÚLTIMO RENDIMENTO
703 – EXISTÊNCIA DE DEDUÇÃO
704 – CÓDIGO DE PREVIDÊNCIA PÚBLICA
705 – CÓDIGO DE IMPOSTO DE RENDA
706 – CÓDIGO DE OUTRAS DEDUÇÕES
800 – CÓDIGO DA DEDUÇÃO
900 - UNIDADES DA FEDERAÇÃO
901 – ESTRATO GEOGRÁFICO
902 – CÓDIGO DE IMPUTAÇÃO
903 – FORMA DE AQUISIÇÃO
904 – MÊS DA ÚLTIMA DESPESA OU RENDIMENTO
905 - ESTADO DE AQUISIÇÃO
906 – NÚMERO DO QUADRO
907 – CÓDIGO DO ITEM
```

# Descrição dos Itens Físicos:

**REGISTRO TIPO 01: DOMICÍLIO - POF1 / QUADRO 2**

## Tamanho: 131

```
        1 - TIPO DE REGISTRO (Item de Identificação do Dicionário)
            Posição Inicial: 1
            Formato: N - Numérico
            Tamanho: 2 dígitos (2 bytes)
 		Descrição Sumária: Identifica o tipo de dado a que se referem os registros. Os registros tipo 01 englobam as informações sobre o Domicílio, os tipo 02 referem-se as informações sobre Pessoas Moradoras, os tipo 03 referem-se as informações de Imputação das variáveis referentes a Pessoas Moradoras, os tipo 04 referem-se as informações de Condições de Vida das Unidades de Consumo, os tipo 05 referem-se ao Inventário de Bens Duráveis das Unidades de Consumo, os tipo 06 referem-se as Despesas de 90 dias do Questionário de Aquisição Coletiva (POF2), os tipo 07 referem-se as Despesas de 12 meses do Questionário de Aquisição Coletiva (POF2), os tipo 08 referem-se as Outras Despesas do Questionário de Aquisição Coletiva (POF2), os tipo 09 referem-se as Despesas com Serviços Domésticos do Questionário de Aquisição Coletiva (POF2), os tipo 10 referem-se aos valores de Aluguel Estimado das Unidades de Consumo Principais, pesquisados no POF 1, os  tipo  11 referem-se  as Despesas da Caderneta de Despesa Coletiva (POF3), os tipo 12 referem-se as Despesas do Questionário de Aquisição Individual (POF4), os  tipo 13 referem-se as Despesas com Veículos do Questionário de Aquisição Individual (POF4), os tipo 14 referem-se as informações sobre Trabalhos, Rendimentos e Deduções do Questionário de Rendimento Individual (POF5), os tipo 15 referem-se aos Outros Rendimentos do Questionário de Rendimento Individual (POF5).


            2 – CÓDIGO DA UNIDADE DA FEDERAÇÃO
            Posição Inicial: 3
            Formato: N - Numérico
            Tamanho: 2 dígitos (2 bytes)
 		Quantidade de Categorias: 27
            Classificação: 900
            Descrição Sumária: Identifica o código da Unidade da Federação.


            3 – NÚMERO SEQUENCIAL
            Posição Inicial: 5
            Formato: N - Numérico
            Tamanho: 3 dígitos (3 bytes)
 		Descrição Sumária: Identifica o Número Seqüencial para cada um dos Setores da Amostra. É composto de três dígitos atribuído ao setor em cada área.
.


            4 – DV DO SEQUENCIAL
            Posição Inicial: 8
            Formato: N - Numérico
            Tamanho: 1 dígito (1 byte)
            Descrição Sumária: Identifica o Dígito Verificador do Número Seqüencial atribuído ao Setor da Amostra.


            5 – NÚMERO DO DOMICÍLIO
            Posição Inicial: 9
            Formato: N - Numérico
            Tamanho: 2 dígitos (2 bytes)
            Descrição Sumária: Identifica o Domicílio. Corresponde a um código de dois dígitos atribuído seqüencialmente a cada domicílio selecionado em cada setor.

            6 - ESTRATO GEOGRÁFICO
            Posição Inicial: 11
            Formato: N - Numérico
            Tamanho: 2 dígitos (2 bytes)
Quantidade de Categorias: 51
Classificação: 901
Descrição Sumária: Identifica os estratos do Plano Amostral da pesquisa: estratificações Geográfica e Estatística. No nível geográfico, a estratificação compreende: área urbana para o município da capital, resto da região metropolitana, resto da UF e área rural. A estratificação estatística foi realizada a partir das informações da renda total dos responsáveis pelos domicílios e a taxa de ocupação.


ESTRATOS DO PLANO AMOSTRAL
Estratificação Geográfica e Estatística
```

|  |  | ESTRATOS |  |  |
| --- | --- | --- | --- | --- |
| ÁREAS |  | URBANO |  | RURAL |
| DA PESQUISA | Município | Resto | Resto |  |
|  | da Capital | da RM | da UF |  |
|  |  |  |  |  |
| **Região Norte** |  |  |  |  |
| Rondônia 11 | 1 e 2 | - | 3 a 6 | 7 a 11 |
| Acre 12 | 1 | - | 2 | 3 e 4 |
| Amazonas 13 | 1 a 4 | - | 5 a 8 | 9 a 13 |
| Roraima 14 | 1 | - | 2 | 3 e 4 |
| Pará 15 | 1 e 2 | 3 e 4 belem | 5 a 8 | 9 a 14 |
| Amapá 16 | 1 | - | 2 e 3 | 4 a 6 |
| Tocantins 17 | 1 | - | 2 a 5 | 6 a 10 |
|  |  |  |  |  |
| **Região Nordeste** |  |  |  |  |
| Maranhão 21 | 1 a 3 | - | 4 a 12 | 13 a 24 |
| Piauí 22 | 1 a 3 | - | 4 a 9 | 10 a 19 |
| Ceará 23 | 1 a 9 | 10 a 12 fortaleza | 13 a 23 | 24 a 36 |
| Rio Grande do Norte 24 | 1 e 2 | - | 3 a 8 | 9 a 13 |
| Paraíba 25 | 1 a 3 | - | 4 a 9 | 10 a 16 |
| Pernambuco 26 | 1 a 3 | 4 a 6 recife | 7 a 15 | 16 a 25 |
| Alagoas 27 | 1 a 3 | - | 4 a 8 | 9 a 13 |
| Sergipe 28 | 1 e 2 | - | 3 a 7 | 8 a 9 |
| Bahia 29 | 1 a 6 | 7 a 8 salvador | 9 a 21 | 22 a 36 |
|  |  |  |  |  |
| **Região Sudeste** |  |  |  |  |
| Minas Gerais 31 | 1 a 6 | 7 a 9 belo horizonte | 10 a 27 | 28 a 45 |
| Espírito Santo 32 | 1 | - | 2 a 9 | 10 a 14 |
| Rio de Janeiro 33 | 1 a 9 | 10 a 18 rio | 19 a 30 | 31 a 37 |
| São Paulo 35 | 1 a 9 | 10 a 15 sao paulo | 16 a 30 | 31 a 51 |
|  |  |  |  |  |
| **Região Sul** |  |  |  |  |
| Paraná 41 | 1 a 6 | 7 a 9 curitiba | 10 a 18 | 19 a 29 |
| Santa Catarina 42 | 1 e 2 | - | 3 a 13 | 14 a 23 |
| Rio Grande Do Sul 43 | 1 a 6 | 7 a 9 porto alegre | 10 a 18 | 19 a 30 |
|  |  |  |  |  |
| **Região Centro-Oeste** |  |  |  |  |
| Mato Grosso do Sul 50 | 1 a 3 | - | 4 a 8 | 9 a 13 |
| Mato Grosso 51 | 1 a 3 | - | 4 a 10 | 11 a 18 |
| Goiás 52 | 1 a 3 | - | 4 a 17 | 18 a 28 |
| Distrito Federal 53 | 1 a 7 |  |  | 8 a 9 |

```
            7 – FATOR DE EXPANSÃO 1 (DESENHO AMOSTRAL)
            Posição Inicial: 13
            Formato: N - Numérico
            Tamanho: 14 dígitos (14 bytes)
            Quantidade de Decimais: 8
            Descrição Sumária: Identifica o fator de expansão atribuído ao             domicílio pertencente a um setor. A cada domicílio corresponde um fator de expansão, o qual é também atribuído para as unidades de consumo do  respectivo  domicílio.


            8 – FATOR DE EXPANSÃO 2 (AJUSTADO P/ ESTIMATIVAS)
            Posição Inicial: 27
            Formato: N - Numérico
            Tamanho: 14 dígitos (14 bytes)
            Quantidade de Decimais: 8
            Descrição Sumária: Identifica o fator de expansão ajustado às  previsões de totais (para 15 janeiro de 2009) de algumas variáveis do Universo, segundo alguns domínios de interesse. Este fator ajustado é definido para cada domicílio de um setor da amostra e também deve ser aplicado para cada Unidade de Consumo existente no Domicílio. Na atual pesquisa, os domicílios pertencentes à um mesmo setor tem o mesmo fator de expansão ajustado (fator de expansão 2). Este fator deve ser utilizado para efeito do cálculo de estimativas a partir dos dados da pesquisa.


            9 – PERÍODO REAL DA COLETA
            Posição Inicial: 41
            Formato: N - Numérico
            Tamanho: 4 dígitos (4 bytes)
 		Descrição Sumária: Identifica o período(semana) que o domicílio selecionado foi efetivamente pesquisado. Este período é definido em função do primeiro dia de preenchimento da caderneta de despesa coletiva (POF3).


            10 – QUANTIDADE DE MORADORES
            Posição Inicial: 45
            Formato: N - Numérico
            Tamanho: 4 dígitos (4 bytes)
            Descrição Sumária: Identifica o número total de moradores no domicílio.


            11 - QUANTIDADE DE UC
            Posição inicial: 49
            Formato: N - Numérico
            Tamanho: 2 dígitos (2 bytes)
Descrição Sumária: Identifica o número total de unidades de consumo no domicílio.


            12 – QUANTIDADE DE FAMÍLIAS
            Posição Inicial: 51
            Formato: N - Numérico
            Tamanho: 2 dígitos (2 bytes)
            Descrição Sumária: Identifica o número total de famílias no domicílio.


            13 - TIPO DE DOMICÍLIO
            Posição Inicial: 53
            Formato: N - Numérico
            Tamanho: 2 dígitos (2 bytes)
            Quantidade de Categorias: 3
            Classificação: 100
            Descrição Sumária: Identifica o tipo de domicílio. Categorias: (01) Casa, (02) Apartamento e (03) Cômodo.


            14 – MATERIAL QUE PREDOMINA NAS PAREDES EXTERNAS
            Posição Inicial: 55
            Formato: N - Numérico
            Tamanho: 2 dígitos (2 bytes)
            Quantidade de Categorias: 6
            Classificação: 101
            Descrição Sumária: Identifica o material que predomina na construção das paredes externas do domicílio. Categorias: (01) Alvenaria, (02) Madeira para Construção, (03) Taipa Não-Revestida, (04) Madeira Aproveitada, (05) Palha e (06) Outro Material.


            15 – MATERIAL QUE PREDOMINA NA COBERTURA
            Posição Inicial: 57
            Formato: N - Numérico
            Tamanho: 2 dígitos (2 bytes)
            Quantidade de Categorias: 7
            Classificação: 102
            Descrição Sumária: Identifica o material que predomina na cobertura (telhado) do domicílio. Categorias: (01) Telha Qualquer, (02) Laje de Concreto, (03) Madeira para Construção, (04) Chapa Metálica, (05) Madeira Aproveitada, (06) Palha e (07) Outro Material.


            16 – MATERIAL QUE PREDOMINA NO PISO
            Posição Inicial: 59
            Formato: N - Numérico
            Tamanho: 2 dígitos (2 bytes)
            Quantidade de Categorias: 7
            Classificação: 103
            Descrição Sumária: Identifica o material que predomina no piso do domicílio. Categorias: (01) Carpete, (02) Cerâmica/Lajota/Pedra, (03) Madeira para Construção, (04) Cimento, (05) Madeira Aproveitada, (06) Terra e (07) Outro Material.


            17 - QUANTIDADE DE CÔMODOS
            Posição Inicial: 61
            Formato: N - Numérico
            Tamanho: 2 dígitos (2 bytes)
 		Descrição Sumária: Identifica o total de cômodos existente no domicílio.


            18 - CÔMODOS SERVINDO DE DORMITÓRIO
            Posição Inicial: 63
            Formato: N - Numérico
            Tamanho: 2 dígitos (2 bytes)
            Descrição Sumária: Identifica o número de cômodos servindo de dormitório aos moradores do domicílio, em caráter permanente.


            19 - EXISTÊNCIA DE ÁGUA CANALIZADA
            Posição Inicial: 65
            Formato: N - Numérico
            Tamanho: 2 dígitos (2 bytes)
Quantidade de Categorias: 2
            Classificação: 104
            Descrição Sumária: Identifica se existe água canalizada para pelo menos um cômodo do domicílio. Categorias: (01) Sim e (02) Não.


            20 - PROVENIÊNCIA DA ÁGUA
            Posição Inicial: 67
            Formato: N - Numérico
            Tamanho: 2 dígitos (2 bytes)
Quantidade de Categorias: 3
            Classificação: 105
            Descrição Sumária: Identifica a proveniência da água canalizada utilizada no domicílio. Categorias: (01) Rede Geral de Distribuição, (02) Poço ou Nascente e (03) Outra Proveniência.


            21 – QUANTIDADE DE BANHEIROS
            Posição Inicial: 69
            Formato: N - Numérico
            Tamanho: 2 dígitos (2 bytes)
            Descrição Sumária: Identifica o número de banheiros ou sanitários localizados dentro da propriedade e de uso exclusivo dos moradores do domicílio.


            22 – ESCOADOURO SANITÁRIO
            Posição Inicial: 71
            Formato: N - Numérico
            Tamanho: 2 dígitos (2 bytes)
            Quantidade de Categorias: 7
            Classificação: 106
            Descrição Sumária: Identifica a forma de Escoadouro Sanitário do banheiro ou sanitário do domicílio. Categorias: (01) Rede Geral de Esgoto ou Pluvial, (02) Fossa Séptica, (03) Fossa Rudimentar, (04) Vala, (05) Direto para o Rio/ lago ou mar, (06) Outra Forma e (07) Não Tem.


            23 - CONDIÇÃO DE OCUPAÇÃO
            Posição Inicial: 73
            Formato: N - Numérico
            Tamanho: 2 dígitos (2 bytes)
            Quantidade de Categorias: 6
            Classificação: 107
            Descrição Sumária: Identifica a Condição de Ocupação do Domicílio. Categorias: (01) Próprio já pago, (02) Próprio – ainda pagando, (03) Cedido por empregador, (04) Cedido de outra forma, (05) Outra Condição e (06) Alugado.


            24 - TEMPO DE ALUGUEL
            Posição Inicial: 75
            Formato: N - Numérico
            Tamanho: 2 dígitos (2 bytes)
            Quantidade de Categorias: 4
            Classificação: 108
            Descrição Sumária: Identifica o tempo de aluguel para os domicílios cuja condição de ocupação é Alugado. Categorias: (–1) Não Aplicável, (01) Menos de 12 meses, (02) De 12 a 30 meses e (03) Mais de 30 meses.


            25 - TIPO DE CONTRATO DE ALUGUEL
            Posição Inicial: 77
            Formato: N - Numérico
            Tamanho: 2 dígitos (2 bytes)
            Quantidade de Categorias: 4
            Classificação: 109
            Descrição Sumária: Identifica o tipo de contrato de aluguel para os domicílios cuja condição de ocupação é Alugado. Categorias: (–1) Não Aplicável, (01) Verbal (“Só de Boca”), (02) Documentado através de Imobiliária e (03) Documentado através de Outra Fonte.


            26 – EXISTÊNCIA DE PAVIMENTAÇÃO NA RUA
            Posição Inicial: 79
            Formato: N - Numérico
            Tamanho: 2 dígitos (2 bytes)
            Quantidade de Categorias: 2
            Classificação: 110
            Descrição Sumária: Identifica se existe pavimentação na rua ou pelo menos no trecho onde se localiza o domicílio. Categorias: (01) Sim e (02) Não.


            27 – IMPUTAÇÃO – QUANTIDADE DE CÔMODOS
            Posição Inicial: 81
            Formato: N - Numérico
            Tamanho: 1 dígito (1 byte)
Quantidade de Categorias: 2
Classificação: 111
            Descrição Sumária: Identifica se o valor da variável Quantidade de Cômodos foi imputado. Categorias: (0) Não e (1) Sim.


            28 – IMPUTAÇÃO – QUANTIDADE DE BANHEIROS
            Posição Inicial: 82
            Formato: N - Numérico
            Tamanho: 1 dígito (1 byte)
Quantidade de Categorias: 2
Classificação: 112
            Descrição Sumária: Identifica se o valor da variável Quantidade de Banheiros foi imputado. Categorias: (0) Não e (1) Sim.


            29 – IMPUTAÇÃO - ESCOADOURO SANITÁRIO
            Posição Inicial: 83
            Formato: N - Numérico
            Tamanho: 1 dígito (1 byte)
Quantidade de Categorias: 2
Classificação: 113
            Descrição Sumária: Identifica se o valor da variável Escoadouro Sanitário foi imputado. Categorias: (0) Não e (1) Sim.

		Obs: não houve imputação dos valores das outras variáveis deste registro.


30 - RENDA MONETÁRIA MENSAL DO DOMICÍLIO
            Posição inicial: 84
            Formato: N - Numérico
            Tamanho: 16 dígitos (16 bytes)
            Quantidade de Decimais: 2
            Descrição Sumária: Identifica o valor em reais do rendimento bruto monetário mensal do domicílio que é obtido através do somatório dos rendimentos brutos monetários mensais de todos os moradores do domicílio, obtidos através do trabalho, transferências e outras rendas, acrescido da variação patrimonial, que compreende vendas de imóveis, recebimentos de heranças e o saldo positivo da movimentação   financeira.


            31 - RENDA NÃO MONETÁRIA MENSAL DO DOMICÍLIO
            Posição inicial: 100
            Formato: N - Numérico
            Tamanho: 16 dígitos (16 bytes)
            Quantidade de Decimais: 2
            Descrição Sumária:
Identifica o valor em reais do rendimento bruto não monetário mensal do domicílio.
      Considerou-se como rendimento não monetário a parcela equivalente às despesas não-monetárias que são aquelas efetuadas sem pagamento monetário, ou seja, aquisição obtida através doação, retirada do negócio, troca, produção própria, pescado, caçado e coletado durante o período de referência da pesquisa, disponíveis para utilização. As valorações das despesas não monetárias foram realizadas pelos próprios informantes, considerando os preços vigentes no mercado local.
		O aluguel atribuído ao domicílio cuja condição de ocupação era diferente de alugado foi o único serviço contabilizado como despesa não monetária.
       É importante observar que as despesas não-monetárias são iguais, em termos contábeis, às receitas não monetárias com exceção do valor do aluguel estimado.
       Para a imputação do valor do rendimento não monetário correspondente ao aluguel de imóveis, foram deduzidas, do valor do aluguel estimado pelas unidades de consumo, as despesas com manutenção e reparos, impostos, taxas de serviços e seguros com o domicílio, conforme as recomendações contidas no documento Informe Final da Décima Sétima Conferência Internacional de Estatísticos do Trabalho, realizada pela Organização Internacional do Trabalho em dezembro de 2003.


            32 - RENDA TOTAL MENSAL DO DOMICÍLIO
            Posição inicial: 116
            Formato: N - Numérico
            Tamanho: 16 dígitos (16 bytes)
            Quantidade de Decimais: 2
            Descrição Sumária: Identifica o valor em reais do rendimento bruto total mensal do domicílio. O rendimento total é obtido através do somatório dos rendimentos brutos monetários mensais de todos os moradores do domicílio, obtidos através do trabalho, transferências e outras rendas, mais a parcela relativa aos rendimentos não monetários mensais do domicílio, acrescido da variação patrimonial, que compreende vendas de imóveis, recebimentos de heranças e o saldo positivo da movimentação   financeira.


  REGISTRO TIPO 02: PESSOAS - POF1 / QUADROS 3 E 4
```

##  Tamanho: 212

```
  		1 - TIPO DE REGISTRO (Item de Identificação do Dicionário)
            Posição Inicial: 1
            Formato: N - Numérico
            Tamanho: 2 dígitos (2 bytes)
 		Descrição Sumária: Ver descrição da variável 1 do REGISTRO TIPO 01 – DOMICÍLIO.


  		2 – CÓDIGO DA UNIDADE DA FEDERAÇÃO
            Posição Inicial: 3
            Formato: N - Numérico
            Tamanho: 2 dígitos (2 bytes)
 		Quantidade de Categorias: 27
 		Classificação: 900
 		Descrição Sumária: Ver descrição da variável 2 do REGISTRO TIPO 01 – DOMICÍLIO.


  		3 – NÚMERO SEQUENCIAL
            Posição Inicial: 5
            Formato: N - Numérico
            Tamanho: 3 dígitos (3 bytes)
```

Descrição Sumária: Ver descrição da variável 3 do REGISTRO TIPO 01 – DOMICÍLIO.

```
  		4 – DV DO SEQUENCIAL
            Posição Inicial: 8
            Formato: N - Numérico
            Tamanho: 1 dígito (1 byte)
 		Descrição Sumária: Ver descrição da variável 4 do REGISTRO TIPO 01 – DOMICÍLIO.


  		5 – NÚMERO DO DOMICÍLIO
            Posição Inicial: 9
            Formato: N - Numérico
            Tamanho: 2 dígitos (2 bytes)
Descrição Sumária: Ver descrição da variável 5 do REGISTRO TIPO
01 – DOMICÍLIO.


  		6 - NÚMERO DA UC
             Posição inicial: 11
             Formato: N - Numérico
             Tamanho: 1 dígito (1 byte)
            Descrição Sumária: Identifica o número da unidade de consumo (UC) pertencente ao domicílio pesquisado. Este número varia de 1 até o número total de unidades de consumo do domicílio.


7 – NÚMERO DO INFORMANTE
            Posição Inicial: 12
            Formato: N - Numérico
            Tamanho: 2 dígitos (2 bytes)
Descrição Sumária: Corresponde ao número de ordem no qual o morador foi registrado no quadro 3 do POF1. Este número é usado para identificar o informante em todos questionários.


8 - ESTRATO GEOGRÁFICO
            Posição Inicial: 14
            Formato: N - Numérico
            Tamanho: 2 dígitos (2 bytes)
Quantidade de Categorias: 51
Classificação: 901
Descrição Sumária: Ver descrição da variável 6 do REGISTRO TIPO 01 – DOMICÍLIO.


  		9 – FATOR DE EXPANSÃO 1 (DESENHO AMOSTRAL)
            Posição Inicial: 16
            Formato: N - Numérico
            Tamanho: 14 dígitos (14 bytes)
            Quantidade de Decimais: 8
Descrição Sumária: Ver descrição da variável 7 do REGISTRO TIPO 01 – DOMICÍLIO.


  		10 – FATOR DE EXPANSÃO 2 (AJUSTADO P/ ESTIMATIVAS)
            Posição Inicial: 30
            Formato: N - Numérico
            Tamanho: 14 dígitos (14 bytes)
            Quantidade de Decimais: 8
Descrição Sumária: Ver descrição da variável 8 do REGISTRO TIPO 01 – DOMICÍLIO.


  		11 – CONDIÇÃO NA UNIDADE DE CONSUMO
            Posição Inicial: 44
            Formato: N - Numérico
            Tamanho: 2 dígitos (2 bytes)
            Quantidade de Categorias: 8
            Classificação: 200
            Descrição Sumária: Identifica o grau de parentesco ou a natureza da subordinação existente entre o morador e a pessoa de referência da sua unidade de consumo. Categorias: (01) Pessoa de Referência, (02) Cônjuge, (03) Filho, (04) Outro Parente, (05) Agregado, (06) Pensionista, (07) Empregado Doméstico e (08) Parente de Empregado Doméstico.


12 - NÚMERO DA FAMÍLIA
            Posição inicial: 46
            Formato: N - Numérico
            Tamanho: 2 dígitos (2 bytes)
 		Descrição Sumária: Identifica o número da família que pertence o morador. Este número varia de 1 até o número total de famílias do domicílio.


13 – CONDIÇÃO NA FAMÍLIA
            Posição Inicial: 48
            Formato: N - Numérico
            Tamanho: 2 dígitos (2 bytes)
            Quantidade de Categorias: 8
            Classificação: 201
            Descrição Sumária: Identifica o grau de parentesco ou a natureza da subordinação existente entre o morador e a pessoa de referência da sua família. Categorias: (01) Pessoa de Referência, (02) Cônjuge, (03) Filho, (04) Outro Parente, (05) Agregado, (06) Pensionista, (07) Empregado Doméstico e (08) Parente de Empregado Doméstico.


  		14 - CONDIÇÃO DE PRESENÇA
            Posição Inicial: 50
            Formato: N – Numérico
            Tamanho: 2 dígitos (2 bytes)
            Quantidade de Categorias: 2
            Classificação: 202
            Descrição Sumária: Identifica se o morador encontrava-se  presente ou  não  no período da pesquisa. Categorias: (01) Morador Presente e (02) Morador Ausente.


15 – DIA DE NASCIMENTO
            Posição Inicial: 52
            Formato: N - Numérico
            Tamanho: 2 dígitos (2 bytes)
Descrição Sumária: Identifica o dia de nascimento do morador do domicílio. Para as situações que o morador não soube precisar a data de nascimento própria ou de um dos moradores, registrou-se nesta variável 00 quando a idade foi presumida pelo morador ou estimada pelo agente de pesquisa.


  		16 – MÊS DE NASCIMENTO
            Posição Inicial: 54
            Formato: N - Numérico
            Tamanho: 2 dígitos (2 bytes)
            Descrição Sumária: Identifica o mês de nascimento do morador do domicílio. Para as situações que o morador não soube precisar a data             de nascimento própria ou de um dos moradores, registrou-se             nesta variável 20 quando a idade foi presumida pelo morador e 30 quando foi estimada pelo agente de pesquisa.


  		17 – ANO DE NASCIMENTO
            Posição Inicial: 56
            Formato: N - Numérico
            Tamanho: 4 dígitos (4 bytes)
 		Descrição Sumária: Identifica o ano de nascimento do morador do domicílio. Para as situações que o morador não soube precisar a data            de nascimento própria ou de um dos moradores, registrou-se             nesta variável a idade que foi presumida pelo morador ou quando foi estimada pelo agente de pesquisa.


  		18 - IDADE CALCULADA EM ANOS
            Posição Inicial: 60
            Formato: N - Numérico
            Tamanho: 3 dígitos (3 bytes)
   		Descrição Sumária: Identifica a idade do morador em anos completos. Foi calculada com base na data de nascimento e a             data de início de preenchimento da Caderneta de Despesa Coletiva (POF3). No caso  de  idade  estimada  ou  presumida foi obtida pela transferência da informação da variável "Ano de nascimento".


  		19 - IDADE CALCULADA EM MESES
            Posição Inicial: 63
            Formato: N - Numérico
            Tamanho: 6 dígitos (6 bytes)
Descrição Sumária: Identifica a idade do morador em meses completos. Foi calculada com base na data de nascimento e a             data de início de preenchimento da Caderneta de Despesa Coletiva (POF3).


  		20 - IDADE CALCULADA EM DIAS
            Posição Inicial: 69
            Formato: N - Numérico
            Tamanho: 7 dígitos (7 bytes)
Descrição Sumária: Identifica a idade do morador em dias completos. Foi calculada com base na data de nascimento e a             data de início de preenchimento da Caderneta de Despesa Coletiva (POF3).


  		21 – SEXO
            Posição Inicial: 76
            Formato: N - Numérico
            Tamanho: 2 dígitos (2 bytes)
            Quantidade de Categorias: 2
            Classificação: 203
            Descrição Sumária: Identifica o sexo do morador. Categorias: (01) Masculino e (02) Feminino.


22 – SABE LER E ESCREVER
            Posição Inicial: 78
            Formato: N - Numérico
            Tamanho: 2 dígitos (2 bytes)
            Quantidade de Categorias: 2
            Classificação: 204
            Descrição Sumária: Identifica se o morador sabe ler e escrever. Categorias: (01) Sim e (02) Não.


  		23 – FREQUÊNCIA À ESCOLA OU CRECHE
            Posição Inicial: 80
            Formato: N - Numérico
            Tamanho: 2 dígitos (2 bytes)
            Quantidade de Categorias: 4
            Classificação: 205
            Descrição Sumária: Identifica se o morador frequentava escola ou creche no período da pesquisa. Categorias: (01) Sim, Rede Particular (02) Sim, Rede Pública, (03) Não, mas já Frequentou, (04) Nunca Frequentou.


  		24 – CURSO QUE FREQUENTA
            Posição Inicial: 82
            Formato: N - Numérico
            Tamanho: 2 dígitos (2 bytes)
            Quantidade de Categorias: 14
            Classificação: 206
            Descrição Sumária: Identifica o curso que o morador frequentava no período da pesquisa. Categorias: (-1) Não Aplicável, (01) Creche, (02) Pré-escolar, (03) Classe de Alfabetização de Crianças, (04) Alfabetização de Adultos, (05) Regular do Ensino Fundamental, (06) Educação de Jovens e Adultos ou Supletivo do Ensino Fundamental ou 1º grau, (07) Regular do Ensino Médio, (08) Educação de Jovens e Adultos ou Supletivo do Ensino Médio ou 2º grau, (09) Tecnológico Superior, (10) Pré-Vestibular, (11) Superior-Graduação, (12) Especialização Superior e (13) Mestrado ou Doutorado.


            25 - QUAL A DURAÇÃO DO CURSO DE PRIMEIRO GRAU
            Posição Inicial: 84
            Formato: N - Numérico
            Tamanho: 2 dígitos (2 bytes)
            Quantidade de Categorias: 3
            Classificação: 207
            Descrição Sumária: Identifica a duração do curso de primeiro grau que o morador frequentava no período da pesquisa. Categorias: (-1) Não Aplicável, (01) 8 anos e (02) 9 anos.


 		26 – SÉRIE QUE FREQUENTA
Posição Inicial: 86
            Formato: N - Numérico
            Tamanho: 2 dígitos (2 bytes)
            Quantidade de Categorias: 11
            Classificação: 208
            Descrição Sumária: Identifica a série que o morador frequentava no período da pesquisa. Categorias: (-1) Não Aplicável, (01) Primeira, (02) Segunda, (03) Terceira, (04) Quarta, (05) Quinta, (06) Sexta, (07) Sétima, (08) Oitava, (09) Nona e (10) Curso Não Seriado.


27 – CURSO MAIS ELEVADO QUE FREQUENTOU
            Posição Inicial: 88
            Formato: N - Numérico
            Tamanho: 2 dígitos (2 bytes)
            Quantidade de Categorias: 17
            Classificação: 209
            Descrição Sumária: Identifica o curso mais elevado que o morador frequentou. Categorias: (-1) Não Aplicável, (01) Creche, (02) Pré-escolar, (03) Classe de Alfabetização de Crianças, (04) Alfabetização de Adultos, (05) Antigo Primário, (06) Antigo Ginásio, (07) Antigo Clássico, Científico, etc. (08) Regular do Ensino Fundamental, (09) Educação de Jovens e Adultos ou Supletivo do Ensino Fundamental ou 1º grau, (10) Regular do Ensino Médio, (11) Educação de Jovens e Adultos ou Supletivo do Ensino Médio ou 2º grau, (12) Tecnológico Superior, (13) Pré-Vestibular, (14) Superior-Graduação, (15) Especialização Superior e (16) Mestrado ou Doutorado.


28 - QUAL ERA A DURAÇÃO DO CURSO DE PRIMEIRO GRAU
            Posição Inicial: 90
            Formato: N - Numérico
            Tamanho: 2 dígitos (2 bytes)
            Quantidade de Categorias: 3
            Classificação: 210
            Descrição Sumária: Identifica a duração do curso de primeiro grau que o morador frequentou. Categorias: (-1) Não Aplicável, (1) 8 anos e (2) 9 anos.


29 - ÚLTIMA SÉRIE CONCLUÍDA
Posição Inicial: 92
            Formato: N - Numérico
            Tamanho: 2 dígitos (2 bytes)
            Quantidade de Categorias: 12
            Classificação: 211
            Descrição Sumária: Identifica a última série que o morador concluiu com aprovação. Categorias: (-1) Não Aplicável, (01) Primeira, (02) Segunda, (03) Terceira, (04) Quarta, (05) Quinta, (06) Sexta, (07) Sétima, (08) Oitava, (09) Nona, (10) Curso Não Seriado e (11) Nenhuma.


30 – CONCLUSÃO DO CURSO MAIS ELEVADO
Posição Inicial: 94
            Formato: N - Numérico
            Tamanho: 2 dígitos (2 bytes)
            Quantidade de Categorias: 3
            Classificação: 212
            Descrição Sumária: Identifica se o morador concluiu o curso mais elevado que frequentou. Categorias: (-1) Não Aplicável, (01) Sim e (02) Não.

31 – ANOS DE ESTUDO
            Posição Inicial: 96
            Formato: N - Numérico
            Tamanho: 2 dígitos (2 bytes)
Quantidade de Categorias: 17
		Classificação: 213
Descrição Sumária: Identifica o número de anos de estudo do  morador. Foi calculado com base nas variáveis 24 a 29 deste registro, referentes ao tema escolaridade. Categorias: (00) Zero, (01) Um, (02) Dois, (03) Três, (04) Quatro, (05) Cinco, (06) Seis, (07) Sete, (08) Oito, (09) Nove, (10) Dez, (11) Onze, (12) Doze, (13) Treze, (14) Quatorze, (15) Quinze ou mais e (88) Não determinado (que incluem as pessoas que frequentam ou frequentaram curso Supletivo não seriado de primeiro ou segundo grau).


32 – COR OU RAÇA
            Posição Inicial: 98
            Formato: N - Numérico
            Tamanho: 2 dígitos (2 bytes)
            Quantidade de Categorias: 6
            Classificação: 214
            Descrição Sumária:  Identifica a Cor ou Raça declarada pelo morador. Categorias: (01) Branca, (02) Preta, (03) Amarela, (04) Parda, (05) Indígena e (09) Não Sabe.


33 - ORÇAMENTO TRABALHO E/OU RENDIMENTO
            Posição Inicial: 100
            Formato: N - Numérico
            Tamanho: 2 dígitos (2 bytes)
            Quantidade de Categorias: 5
            Classificação: 215
            Descrição Sumária: Identifica se o morador com 10 anos ou mais é uma Unidade de Orçamento  Trabalho e/ou Rendimento e se respondeu o  questionário  de Trabalho e Rendimento Individual(POF5). Categorias:
(-1) Não Aplicável, (01) Sim, (02) Não, (03) Recusa e (04) Outro.


  		34 - ORÇAMENTO DESPESA
            Posição Inicial: 102
            Formato: N - Numérico
            Tamanho: 2 dígitos (2 bytes)
            Quantidade de Categorias: 5
            Classificação: 216
            Descrição Sumária: Identifica se o morador com 10 anos ou mais é uma Unidade de Orçamento Despesa e se respondeu o questionário  de Despesa Individual (POF4). Categorias:
(-1) Não Aplicável, (01) Sim, (02) Não, (03) Recusa e (04) Outro.


  		35 - TEM CARTÃO DE CRÉDITO
            Posição Inicial: 104
            Formato: N - Numérico
            Tamanho: 2 dígitos (2 bytes)
            Quantidade de Categorias: 3
            Classificação: 217
            Descrição Sumária: Identifica se o morador possui Cartão de Crédito. Categorias: (-1) Não Aplicável, (01) Sim e (02) Não.


	36 - TITULAR DO CARTÃO DE CRÉDITO
            Posição Inicial: 106
            Formato: N - Numérico
            Tamanho: 2 dígitos (2 bytes)
            Quantidade de Categorias: 3
            Classificação: 218
            Descrição Sumária: Identifica se o morador é titular do Cartão de Crédito. Categorias: (-1) Não Aplicável, (01) Sim e (02) Não.


  		37 - TEM CHEQUE ESPECIAL
            Posição Inicial: 108
            Formato: N - Numérico
            Tamanho: 2 dígitos (2 bytes)
            Quantidade de Categorias: 3
            Classificação: 219
            Descrição Sumária: Identifica se o morador possui Cheque Especial. Categorias: (-1) Não Aplicável, (01) Sim e (02) Não.


		38 - TITULAR DA CONTA CORRENTE
            Posição Inicial: 110
            Formato: N - Numérico
            Tamanho: 2 dígitos (2 bytes)
            Quantidade de Categorias: 3
            Classificação: 220
            Descrição Sumária: Identifica se o morador é titular da conta corrente. Categorias: (-1) Não Aplicável, (01) Sim e (02) Não.


39 - RENDA MONETÁRIA MENSAL DA UNIDADE DE CONSUMO
            Posição inicial: 112
            Formato: N - Numérico
            Tamanho: 16 dígitos (16 bytes)
            Quantidade de Decimais: 2
            Descrição Sumária: Identifica o valor em reais do rendimento bruto monetário mensal da unidade de consumo que é obtido através do somatório dos rendimentos brutos monetários mensais de todos os moradores da unidade de consumo, obtidos através do trabalho, transferências e outras rendas, acrescido da variação patrimonial, que compreende vendas de imóveis, recebimentos de heranças e o saldo positivo da movimentação financeira.


40 - RENDA NÃO MONETÁRIA MENSAL DA UNIDADE DE CONSUMO
Posição inicial: 128
            Formato: N - Numérico
            Tamanho: 16 dígitos (16 bytes)
            Quantidade de Decimais: 2
            Descrição Sumária:
Identifica o valor em reais do rendimento bruto não monetário mensal da unidade de consumo.
      Considerou-se como rendimento não monetário a parcela equivalente às despesas não monetárias que são aquelas efetuadas sem pagamento monetário, ou seja, aquisição obtida através doação, retirada do negócio, troca, produção própria, pescado, caçado e coletado durante o período de referência da pesquisa, disponíveis para utilização. As valorações das despesas não monetárias foram realizadas pelos próprios informantes, considerando os preços vigentes no mercado local.
	O aluguel atribuído ao domicílio cuja condição de ocupação era diferente de alugado foi o único serviço contabilizado como despesa não monetária.
       É importante observar que as despesas não monetárias são iguais, em termos contábeis, às receitas não monetárias com exceção do valor do aluguel estimado.
       Para a imputação do valor do rendimento não monetário correspondente ao aluguel de imóveis, foram deduzidas, do valor do aluguel estimado pelas unidades de consumo, as despesas com manutenção e reparos, impostos, taxas de serviços e seguros com o domicílio, conforme as recomendações contidas no documento Informe Final da Décima Sétima Conferência Internacional de Estatísticos do Trabalho, realizada pela Organização Internacional do Trabalho em dezembro de 2003.


41 - RENDA TOTAL MENSAL DA UNIDADE DE CONSUMO           Posição inicial: 144
Formato: N - Numérico
Tamanho: 16 dígitos (16 bytes)
Quantidade de Decimais: 2
Descrição Sumária: Identifica o valor em reais do rendimento bruto total mensal da unidade de consumo. O rendimento total é obtido através do somatório dos rendimentos brutos monetários mensais de todos os moradores da unidade de consumo, obtidos através do trabalho, transferências e outras rendas, mais a parcela relativa aos rendimentos não monetários mensais do domicílio, acrescido da variação patrimonial, que compreende vendas de imóveis, recebimentos de heranças e o saldo positivo da movimentação financeira.


42 – ESTÁ GRAVIDA
Posição inicial:160
Formato: N - Numérico
Tamanho: 2 dígitos (2 bytes)
Quantidade de Categorias: 3
            Classificação: 221
Descrição Sumária: Identifica se a moradora com 10 anos ou mais de idade se encontrava na condição de gestante no momento da pesquisa. Categorias: (-1) Não Aplicável, (01) Sim e (02) Não.


43 – COMPRIMENTO ORIGINAL
Posição inicial: 162
Formato: N - Numérico
Tamanho: 5 dígitos (5 bytes)
Quantidade de Decimais: 1
Descrição Sumária: Identifica o comprimento em centímetros e decimais das crianças menores de 36 meses.
Para moradores ausentes ou moradores com 36 meses ou mais, este campo está preenchido com ZEROS. Quando não foi possível obter o comprimento da criança por algum motivo (doença, recusa, etc.) este campo está preenchido com 999.9.


44 – ALTURA ORIGINAL
Posição inicial: 167
Formato: N - Numérico
Tamanho: 5 dígitos (5 bytes)
Quantidade de Decimais: 1
Descrição Sumária: Identifica a altura em centímetros e decimais dos moradores com 24 meses ou mais.
Para moradores ausentes ou moradores com menos de 24 meses, este campo está preenchido com ZEROS. Para mulheres que se encontravam na condição de gestante no período da pesquisa, este campo também está preenchido com ZEROS, pois as informações de antopometria para este grupo ainda não foram analisadas. Quando não foi possível obter a altura da pessoa por algum motivo (doença, recusa, etc.) este campo está preenchido com 999.9.


45 – PESO ORIGINAL
Posição inicial: 172
Formato: N - Numérico
Tamanho: 5 dígitos (5 bytes)
Quantidade de Decimais: 1
Descrição Sumária: Identifica o peso em quilogramas e decimais dos moradores com 24 meses ou mais.
Para moradores ausentes ou moradores com menos de 24 meses, este campo está preenchido com ZEROS. Para mulheres que se encontravam na condição de gestante no período da pesquisa, este campo também está preenchido com ZEROS, pois as informações de antopometria para este grupo ainda não foram analisadas. Quando não foi possível obter o peso da pessoa por algum motivo (doença, recusa, etc.) este campo está preenchido com 999.9 ou 555.5.


46 – PESO ORIGINAL DAS CRIANÇAS
Posição inicial: 177
Formato: N - Numérico
Tamanho: 5 dígitos (5 bytes)
Quantidade de Decimais: 1
Descrição Sumária: Identifica o peso em quilogramas e decimais das crianças menores de 24 meses.
Para moradores ausentes ou moradores com 24 meses ou mais, este campo está preenchido com ZEROS. Quando não foi possível obter o peso da criança por algum motivo (doença, recusa, etc.) este campo também está preenchido com ZEROS.


Tratamentos das informações antropométricas

            Crítica e imputação das variáveis peso e altura

Diversos tipos de erros podem afetar os resultados do levantamento. Alguns são possíveis de serem controlados, como, por exemplo, o erro amostral, que é controlado quando da definição do desenho e tamanho da amostra. Outros erros surgem nas diversas fases da pesquisa e, dentre estes, destacam-se os erros de não-resposta.

Em função da complexidade da Pesquisa de Orçamentos Familiares, situações de dados incompletos associados à não-resposta ocorreram. A ausência de resposta surgiu quando ocorreu perda das informações motivada por diversos fatores, como, por exemplo, a falta de contato com os moradores selecionados, falta de cooperação associada com a carga de informações a serem coletadas e outros.

Foi utilizado o procedimento de imputação para tratar a não-resposta e também  os erros de resposta associados a valores rejeitados na etapa de crítica.

     O processo de crítica e imputação

 As variáveis peso e altura foram inicialmente dispostas, segundo grupos de idade e sexo, em diagramas de dispersão com o objetivo de identificar valores que caracterizavam erros de medida. Após esta etapa, os dados foram submetidos a um sistema de crítica e imputação automática, denominado CIDAQ - Crítica e Imputação para dados Quantitativos - CIDAQ (Nascimento Silva,1988).

Por uma breve explanação sobre o funcionamento do sistema CIDAQ, pode-se ressaltar que suas funções foram definidas para tratamento de: erros de resposta ou de medida e não resposta parcial.
           Sobre os aspectos metodológicos pertinentes, vale ressaltar que o sistema incorpora:
```

- tratamento multivariado de dados, onde os dados podem ser avaliados levando-se em consideração o comportamento conjunto de várias variáveis, como por exemplo, idade, sexo, peso e altura;

- análise exploratória prévia e transformação de dados, que permite trabalhar com distribuições padronizadas;

- estimação robusta de parâmetros, a partir de dados incompletos, que implementa pesos diferentes para observações mais distantes do conjunto das observações;

- detecção de casos suspeitos (outliers) e

- imputação dos dados faltantes ou rejeitados.

A Tabela abaixo apresenta os totais de registros ignorados, rejeitados pelo CIDAQ e válidos, para pessoas menores de 20 anos, por sexo.

| **Critério de Avaliação** | **Masculino** | **Feminino** | **Total** |
| --- | --- | --- | --- |
| Ignorados | 16009 | 12096 | 28105 |
| Rejeitados (CIDAQ) | 197 | 218 | 415 |
| Válidos | 76969 | 82972 | 159941 |
| Total | 93175 | 95286 | 188461 |

47 – COMPRIMENTO IMPUTADO

Posição inicial: 182

Formato: N - Numérico

Tamanho: 5 dígitos (5 bytes)

Quantidade de Decimais: 1

Descrição Sumária: Identifica o comprimento em centímetros e decimais imputado das crianças menores de 24 meses.

Para moradores com 24 meses ou mais, este campo está preenchido com ZEROS.

48 – ALTURA IMPUTADA

Posição inicial: 187

Formato: N - Numérico

Tamanho: 5 dígitos (5 bytes)

Quantidade de Decimais: 1

Descrição Sumária: Identifica a altura em centímetros e decimais imputada dos moradores com 24 meses ou mais.

Para moradores com menos de 24 meses, este campo está preenchido com ZEROS. Para mulheres que se encontravam na condição de gestante no período da pesquisa, este campo também está preenchido com ZEROS, pois as informações de antopometria para este grupo ainda não foram analisadas.

49 – PESO IMPUTADO

Posição inicial: 192

Formato: N - Numérico

Tamanho: 5 dígitos (5 bytes)

Quantidade de Decimais: 1

Descrição Sumária: Identifica o peso em quilogramas e decimais imputado dos moradores.

Para mulheres que se encontravam na condição de gestante no período da pesquisa, este campo está preenchido com ZEROS, pois as informações de antopometria para este grupo ainda não foram analisadas.

50 – RENDA PER CAPITA DA UNIDADE DE CONSUMO

Posição inicial: 197

Formato: N - Numérico

Tamanho: 16 dígitos (16 bytes)

Quantidade de Decimais: 2

Descrição Sumária: Identifica o valor da renda total mensal da unidade de consumo dividida pelo número de integrantes da unidade de consumo.

**REGISTRO TIPO 03: PESSOAS - IMPUTAÇÃO - POF1/QUADRO 4**

###  Tamanho: 104

```
1 - TIPO DE REGISTRO (Item de Identificação do Dicionário)            Posição Inicial: 1
            Formato: N - Numérico
            Tamanho: 2 dígitos (2 bytes)
 		Descrição Sumária: Ver descrição da variável 1 do REGISTRO TIPO 01 – DOMICÍLIO.


  		2 – CÓDIGO DA UNIDADE DA FEDERAÇÃO
            Posição Inicial: 3
            Formato: N - Numérico
            Tamanho: 2 dígitos (2 bytes)
 		Quantidade de Categorias: 27
 		Classificação: 900
		Descrição Sumária: Ver descrição da variável 2 do REGISTRO TIPO 01 – DOMICÍLIO.


  		3 – NÚMERO SEQUENCIAL
            Posição Inicial: 5
            Formato: N - Numérico
            Tamanho: 3 dígitos (3 bytes)
 		Descrição Sumária: Ver descrição da variável 3 do REGISTRO TIPO 01 – DOMICÍLIO.


  		4 – DV DO SEQUENCIAL
            Posição Inicial: 8
            Formato: N - Numérico
            Tamanho: 1 dígito (1 byte)
 		Descrição Sumária: Ver descrição da variável 4 do REGISTRO TIPO 01 – DOMICÍLIO.


  		5 – NÚMERO DO DOMICÍLIO
            Posição Inicial: 9
            Formato: N - Numérico
            Tamanho: 2 dígitos (2 bytes)
Descrição Sumária: Ver descrição da variável 5 do REGISTRO TIPO
01 – DOMICÍLIO.


  		6 - NÚMERO DA UC
             Posição inicial: 11
             Formato: N - Numérico
             Tamanho: 1 dígito (1 byte)
            Descrição Sumária: Ver descrição da variável 6 do REGISTRO TIPO
02 – PESSOAS.


7 – NÚMERO DO INFORMANTE
            Posição Inicial: 12
            Formato: N - Numérico
            Tamanho: 2 dígitos (2 bytes)
Descrição Sumária: Ver descrição da variável 7 do REGISTRO TIPO
02 – PESSOAS.

8 - ESTRATO GEOGRÁFICO
            Posição Inicial: 14
            Formato: N - Numérico
            Tamanho: 2 dígitos (2 bytes)
Quantidade de Categorias: 51
Classificação: 901
Descrição Sumária: Ver descrição da variável 6 do REGISTRO TIPO 01 – DOMICÍLIO.


  		9 – FATOR DE EXPANSÃO 1 (DESENHO AMOSTRAL)
            Posição Inicial: 16
            Formato: N - Numérico
            Tamanho: 14 dígitos (14 bytes)
            Quantidade de Decimais: 8
Descrição Sumária: Ver descrição da variável 7 do REGISTRO TIPO 01 – DOMICÍLIO.


  		10 – FATOR DE EXPANSÃO 2 (AJUSTADO P/ ESTIMATIVAS)
            Posição Inicial: 30
            Formato: N - Numérico
            Tamanho: 14 dígitos (14 bytes)
            Quantidade de Decimais: 8
Descrição Sumária: Ver descrição da variável 8 do REGISTRO TIPO 01 – DOMICÍLIO.


  		11 – IMPUTAÇÃO - SABE LER E ESCREVER
             Posição Inicial: 44
             Formato: N - Numérico
             Tamanho: 1 dígito (1 byte)
             Quantidade de Categorias: 2
             Classificação: 300
             Descrição Sumária: Identifica se o valor da variável SABE LER E ESCREVER foi imputado. Categorias: 0 (Não) e 1 (Sim).


  		12 – IMPUTAÇÃO - FREQUÊNCIA À ESCOLA OU CRECHE
             Posição Inicial: 45
             Formato: N - Numérico
             Tamanho: 1 dígito (1 byte)
             Quantidade de Categorias: 2
             Classificação: 301
Descrição Sumária: Identifica se o valor da variável FREQUÊNCIA À ESCOLA OU CRECHE foi imputado. Categorias: 0 (Não) e 1 (Sim).


 13 – IMPUTAÇÃO - CURSO QUE FREQUENTA
 Posição Inicial: 46
 Formato: N - Numérico
             Tamanho: 1 dígito (1 byte)
             Quantidade de Categorias: 2
 Classificação: 302
 Descrição Sumária: Identifica se o valor da variável CURSO QUE FREQUENTA foi imputado. Categorias: 0 (Não) e 1 (Sim).


 14 – IMPUTAÇÃO - QUAL A DURAÇÃO DO CURSO DE PRIMEIRO GRAU
 Posição Inicial: 47
 Formato: N - Numérico
             Tamanho: 1 dígito (1 byte)
 Quantidade de Categorias: 2
 Classificação: 303
 Descrição Sumária: Identifica se o valor da variável QUAL A DURAÇÃO DO CURSO DE PRIMEIRO GRAU foi imputado. Categorias: 0 (Não) e 1 (Sim).


 15 – IMPUTAÇÃO - SÉRIE QUE FREQÜENTA
 Posição Inicial: 48
 Formato: N - Numérico
             Tamanho: 1 dígito (1 byte)
 Quantidade de Categorias: 2
 Classificação: 304
 Descrição Sumária: Identifica se o valor da variável SÉRIE QUE FREQÜENTA foi imputado. Categorias: 0 (Não) e 1 (Sim).


 16 – IMPUTAÇÃO - CURSO MAIS ELEVADO QUE FREQUENTOU
 Posição Inicial: 49
 Formato: N - Numérico
 Tamanho: 1 dígito (1 byte)
 Quantidade de Categorias: 2
 Classificação: 305
 Descrição Sumária: Identifica se o valor da variável CURSO MAIS ELEVADO QUE FREQUENTOU foi imputado. Categorias: 0 (Não) e 1 (Sim).


 17 – IMPUTAÇÃO - QUAL ERA A DURAÇÃO DO CURSO DE PRIMEIRO GRAU
 Posição Inicial: 50
 Formato: N - Numérico
 Tamanho: 1 dígito (1 byte)
 Quantidade de Categorias: 2
 Classificação: 306
 Descrição Sumária: Identifica se o valor da variável QUAL ERA A DURAÇÃO DO CURSO DE PRIMEIRO GRAU foi imputado. Categorias: 0 (Não) e 1 (Sim).


 18 – IMPUTAÇÃO - ÚLTIMA SÉRIE CONCLUÍDA
 Posição Inicial: 51
 Formato: N - Numérico
 Tamanho: 1 dígito (1 byte)
 Quantidade de Categorias: 2
 Classificação: 307
 Descrição Sumária: Identifica se o valor da variável ÚLTIMA SÉRIE CONCLUÍDA foi imputado. Categorias: 0 (Não) e 1 (Sim).


 19 – IMPUTAÇÃO - CONCLUSÃO DO CURSO MAIS ELEVADO
 Posição Inicial: 52
 Formato: N - Numérico
 Tamanho: 1 dígito (1 byte)
 Quantidade de Categorias: 2
 Classificação: 308
 Descrição Sumária: Identifica se o valor da variável CONCLUSÃO DO CURSO MAIS ELEVADO foi imputado. Categorias: 0 (Não) e 1 (Sim).


 20 – IMPUTAÇÃO - TEM CARTÃO DE CRÉDITO
 Posição Inicial: 53
 Formato: N - Numérico
 Tamanho: 1 dígito (1 byte)
 Quantidade de Categorias: 2
 Classificação: 309
 Descrição Sumária: Identifica se o valor da variável TEM CARTÃO DE CRÉDITO foi imputado. Categorias: 0 (Não) e 1 (Sim).


 21 – IMPUTAÇÃO - TITULAR DO CARTÃO DE CRÉDITO
 Posição Inicial: 54
 Formato: N - Numérico
 Tamanho: 1 dígito (1 byte)
 Quantidade de Categorias: 2
 Classificação: 310
 Descrição Sumária: Identifica se o valor da variável TITULAR DO CARTÃO DE CRÉDITO foi imputado. Categorias: 0 (Não) e 1 (Sim).


 22 – IMPUTAÇÃO - TEM CHEQUE ESPECIAL
 Posição Inicial: 55
 Formato: N - Numérico
 Tamanho: 1 dígito (1 byte)
 Quantidade de Categorias: 2
 Classificação: 311
 Descrição Sumária: Identifica se o valor da variável TEM CHEQUE ESPECIAL foi imputado. Categorias: 0 (Não) e 1 (Sim).


 23 – IMPUTAÇÃO - TITULAR DA CONTA CORRENTE
 Posição Inicial: 56
 Formato: N - Numérico
 Tamanho: 1 dígito (1 byte)
 Quantidade de Categorias: 2
 Classificação: 312
 Descrição Sumária: Identifica se o valor da variável TITULAR DA CONTA CORRENTE foi imputado. Categorias: 0 (Não) e 1 (Sim).


	Obs: não houve imputação dos valores das outras variáveis do registro de pessoas.


 24 - RENDA MONETÁRIA MENSAL DA UNIDADE DE CONSUMO
 Posição inicial: 57
 Formato: N - Numérico
 Tamanho: 16 dígitos (16 bytes)
 Quantidade de Decimais: 2
      Descrição Sumária: Ver descrição da variável 39 do REGISTRO TIPO 02 – PESSOAS.


 25 - RENDA NÃO MONETÁRIA MENSAL DA UNIDADE DE CONSUMO
 Posição inicial: 73
 Formato: N - Numérico
 Tamanho: 16 dígitos (16 bytes)
 Quantidade de Decimais: 2
      Descrição Sumária: Ver descrição da variável 40 do REGISTRO TIPO 02 – PESSOAS.


 26 - RENDA TOTAL MENSAL DA UNIDADE DE CONSUMO
 Posição inicial: 89
 Formato: N - Numérico
 Tamanho: 16 dígitos (16 bytes)
 Quantidade de Decimais: 2
 Descrição Sumária: Ver descrição da variável 41 do REGISTRO TIPO
02 – PESSOAS.


 REGISTRO TIPO 04: CONDIÇÕES DE VIDA - POF6 Tamanho Máximo: 94

1 - TIPO DE REGISTRO (Item de Identificação do Dicionário)
            Posição Inicial: 1
            Formato: N - Numérico
            Tamanho: 2 dígitos (2 bytes)
 		Descrição Sumária: Ver descrição da variável 1 do REGISTRO TIPO 01 – DOMICÍLIO.


  		2 – CÓDIGO DA UNIDADE DA FEDERAÇÃO
            Posição Inicial: 3
            Formato: N - Numérico
            Tamanho: 2 dígitos (2 bytes)
 		Quantidade de Categorias: 27
 		Classificação: 900
 		Descrição Sumária: Ver descrição da variável 2 do REGISTRO TIPO 01 – DOMICÍLIO.


  		3 – NÚMERO SEQUENCIAL
            Posição Inicial: 5
            Formato: N - Numérico
            Tamanho: 3 dígitos (3 bytes)
 		Descrição Sumária: Ver descrição da variável 3 do REGISTRO TIPO 01 – DOMICÍLIO.


  		4 – DV DO SEQUENCIAL
            Posição Inicial: 8
            Formato: N - Numérico
            Tamanho: 1 dígito (1 byte)
 		Descrição Sumária: Ver descrição da variável 4 do REGISTRO TIPO 01 – DOMICÍLIO.


  		5 – NÚMERO DO DOMICÍLIO
            Posição Inicial: 9
            Formato: N - Numérico
            Tamanho: 2 dígitos (2 bytes)
Descrição Sumária: Ver descrição da variável 5 do REGISTRO TIPO
01 – DOMICÍLIO.


  		6 - NÚMERO DA UC
             Posição inicial: 11
             Formato: N - Numérico
             Tamanho: 1 dígito (1 byte)
 		Descrição Sumária: Ver descrição da variável 6 do REGISTRO TIPO
02 – PESSOAS.


7 – NÚMERO DO INFORMANTE
            Posição Inicial: 12
            Formato: N - Numérico
            Tamanho: 2 dígitos (2 bytes)
Descrição Sumária: Ver descrição da variável 7 do REGISTRO TIPO
02 – PESSOAS.


8 - ESTRATO GEOGRÁFICO
            Posição Inicial: 14
            Formato: N - Numérico
            Tamanho: 2 dígitos (2 bytes)
Quantidade de Categorias: 51
Classificação: 901
Descrição Sumária: Ver descrição da variável 6 do REGISTRO TIPO 01 – DOMICÍLIO.


  		9 – FATOR DE EXPANSÃO 1 (DESENHO AMOSTRAL)
            Posição Inicial: 16
            Formato: N - Numérico
            Tamanho: 14 dígitos (14 bytes)
            Quantidade de Decimais: 8
Descrição Sumária: Ver descrição da variável 7 do REGISTRO TIPO 01 – DOMICÍLIO.


  		10 – FATOR DE EXPANSÃO 2 (AJUSTADO P/ ESTIMATIVAS)
            Posição Inicial: 30
            Formato: N - Numérico
            Tamanho: 14 dígitos (14 bytes)
            Quantidade de Decimais: 8
Descrição Sumária: Ver descrição da variável 8 do REGISTRO TIPO 01 – DOMICÍLIO.


11 - CODIGO DA RENDA FAMILIAR
            Posição Inicial: 44
            Formato: N - Numérico
            Tamanho: 1 dígito (1 byte)
            Quantidade de Categorias: 7
            Classificação: 400
            Descrição Sumária: Identifica a avaliação da família sobre como seu rendimento total permite levar a vida até o fim do mês. Categorias: (0) Não respondido, (1) Muita dificuldade, (2) Dificuldade, (3) Alguma dificuldade, (4) Alguma facilidade, (5) Facilidade e (6) Muita facilidade.


12 – QUANTIDADE DE ALIMENTO
            Posição Inicial: 45
            Formato: N - Numérico
            Tamanho: 1 dígito (1 byte)
            Quantidade de Categorias: 4
            Classificação: 401
            Descrição Sumária: Identifica a avaliação da família em relação a quantidade de alimentos consumidos. Categorias: (0) Não respondido, (1) Normalmente não é suficiente, (2) Às vezes não é suficiente e (3) É sempre suficiente.


13 – TIPO DE ALIMENTO
            Posição Inicial: 46
            Formato: N - Numérico
            Tamanho: 1 dígito (1 byte)
            Quantidade de Categorias: 4
            Classificação: 402
            Descrição Sumária: Identifica a avaliação da família em relação aos tipos de alimentos consumidos. Categorias: (0) Não respondido, (1) Sempre do tipo que quer, (2) Nem sempre do tipo que quer, (3) Raramente do tipo que quer.


 14 - RENDA MONETÁRIA MENSAL DA UNIDADE DE CONSUMO
 Posição inicial: 47
 Formato: N - Numérico
 Tamanho: 16 dígitos (16 bytes)
 Quantidade de Decimais: 2
      Descrição Sumária: Ver descrição da variável 39 do REGISTRO TIPO 02 – PESSOAS.


 15 - RENDA NÃO MONETÁRIA MENSAL DA UNIDADE DE CONSUMO
 Posição inicial: 63
 Formato: N - Numérico
 Tamanho: 16 dígitos (16 bytes)
 Quantidade de Decimais: 2
      Descrição Sumária: Ver descrição da variável 40 do REGISTRO TIPO 02 – PESSOAS.


 16 - RENDA TOTAL MENSAL DA UNIDADE DE CONSUMO
 Posição inicial: 79
 Formato: N - Numérico
 Tamanho: 16 dígitos (16 bytes)
 Quantidade de Decimais: 2
 Descrição Sumária: Ver descrição da variável 41 do REGISTRO TIPO
02 – PESSOAS.


REGISTRO TIPO 05: INVENTÁRIO DE BENS DURÁVEIS - POF2 / QUADRO 14
```

#### **Tamanho: 107**

```
1 - TIPO DE REGISTRO (Item de Identificação do Dicionário)
            Posição Inicial: 1
            Formato: N - Numérico
            Tamanho: 2 dígitos (2 bytes)
 		Descrição Sumária: Ver descrição da variável 1 do REGISTRO TIPO 01 – DOMICÍLIO.


  		2 – CÓDIGO DA UNIDADE DA FEDERAÇÃO
            Posição Inicial: 3
            Formato: N - Numérico
            Tamanho: 2 dígitos (2 bytes)
 		Quantidade de Categorias: 27
            Classificação: 900
 		Descrição Sumária: Ver descrição da variável 2 do REGISTRO TIPO 01 – DOMICÍLIO.


  		3 – NÚMERO SEQUENCIAL
            Posição Inicial: 5
            Formato: N - Numérico
            Tamanho: 3 dígitos (3 bytes)
 		Descrição Sumária: Ver descrição da variável 3 do REGISTRO TIPO 01 – DOMICÍLIO.


  		4 – DV DO SEQUENCIAL
            Posição Inicial: 8
            Formato: N - Numérico
            Tamanho: 1 dígito (1 byte)
 		Descrição Sumária: Ver descrição da variável 4 do REGISTRO TIPO 01 – DOMICÍLIO.


  		5 – NÚMERO DO DOMICÍLIO
            Posição Inicial: 9
            Formato: N - Numérico
            Tamanho: 2 dígitos (2 bytes)
Descrição Sumária: Ver descrição da variável 5 do REGISTRO TIPO
01 – DOMICÍLIO.


  		6 - NÚMERO DA UC
             Posição inicial: 11
             Formato: N - Numérico
             Tamanho: 1 dígito (1 byte)
 		Descrição Sumária: Ver descrição da variável 6 do REGISTRO TIPO
02 – PESSOAS.


7 - ESTRATO GEOGRÁFICO
            Posição Inicial: 12
            Formato: N - Numérico
            Tamanho: 2 dígitos (2 bytes)
Quantidade de Categorias: 51
Classificação: 901
Descrição Sumária: Ver descrição da variável 6 do REGISTRO TIPO 01 – DOMICÍLIO.


  		8 – FATOR DE EXPANSÃO 1 (DESENHO AMOSTRAL)
            Posição Inicial: 14
            Formato: N - Numérico
            Tamanho: 14 dígitos (14 bytes)
            Quantidade de Decimais: 8
Descrição Sumária: Ver descrição da variável 7 do REGISTRO TIPO 01 – DOMICÍLIO.


  		9 – FATOR DE EXPANSÃO 2 (AJUSTADO P/ ESTIMATIVAS)
            Posição Inicial: 28
            Formato: N - Numérico
            Tamanho: 14 dígitos (14 bytes)
            Quantidade de Decimais: 8
Descrição Sumária: Ver descrição da variável 8 do REGISTRO TIPO 01 – DOMICÍLIO.


		10 – NÚMERO DO QUADRO
            Posição Inicial: 42
            Formato: N - Numérico
            Tamanho: 2 dígitos (2 bytes)
            Quantidade de Categorias: 56
Classificação: 906
            Descrição Sumária: Identifica o número do quadro do Questionário de Aquisição Coletiva (POF2) onde são registradas as informações sobre os Bens Duráveis da Unidade de Consumo.
Neste registro, o número do quadro é sempre 14.


   		11 – CÓDIGO DO ITEM
            Posição Inicial: 44
            Formato: N - Numérico
            Tamanho: 5 dígitos (5 bytes)
            Quantidade de Categorias: 13476
            Classificação: 907
 			Descrição Sumária: Identifica o tipo de bem durável.         Esta variável deverá ser lida juntamente com a variável “NÚMERO DO QUADRO”, que irá compor o código do produto completo com 7 dígitos.


12 - QUANTIDADE DO ITEM
            Posição Inicial: 49
            Formato: N - Numérico
            Tamanho: 2 dígitos (2 bytes)
Descrição Sumária: Identifica a quantidade do bem durável, existente no domicílio principal da Unidade de Consumo no período da pesquisa. Nos casos de quantidade ignorada, atribuiu-se o valor de "99".


            13 - ANO DA ÚLTIMA AQUISIÇÃO
            Posição Inicial: 51
            Formato: N - Numérico
            Tamanho: 4 dígitos (4 bytes)
Descrição Sumária: Identifica o ano da última aquisição do bem durável, realizada pela unidade de consumo. Nos casos de ano ignorado, atribuiu-se o valor de "9999".


            14 - ESTADO DA ÚLTIMA AQUISIÇÃO
            Posição Inicial: 55
            Formato: N - Numérico
            Tamanho: 1 dígito (1 byte)
            Quantidade de Categorias: 3
            Classificação: 905
Descrição Sumária: Identifica o estado da última aquisição do bem durável, realizada pela unidade de consumo. Categorias: (1) Novo, (3) Usado e 9 (Não Sabe).


            15 – FORMA DA ÚLTIMA AQUISIÇÃO
            Posição Inicial: 56
            Formato: N - Numérico
            Tamanho: 2 dígitos (2 bytes)
            Quantidade de Categorias: 11
            Classificação: 903
Descrição Sumária: Identifica a forma da última aquisição do bem durável, realizada pela unidade de consumo. Categorias: (00) Não se aplica, (01) Monetária à vista para a Unidade de Consumo, (02) Monetária à vista para Outra Unidade de Consumo, (03) Monetária a prazo para a Unidade de Consumo, (04) Monetária a prazo outra Unidade de Consumo, (05) Cartão de crédito à vista para a Unidade de Consumo, (06) Cartão de crédito à vista para outra Unidade de Consumo, (07) Doação, (08) Retirada do Negócio, (09) Troca, (10) Produção Própria) e (11) Outra.


  		16 – CÓDIGO DE IMPUTAÇÃO
            Posição Inicial: 58
            Formato: N - Numérico
            Tamanho: 2 dígitos (2 bytes)
            Quantidade de Categorias: 4
            Classificação: 902
Descrição Sumária: Identifica o tipo de imputação realizada. Categorias: (00) Quando o registro não foi imputado, (01) Imputação do Valor do Item (quando o registro foi imputado em função da etapa de imputação do valor do item), (02) Imputação de Quadro (quando o registro foi imputado em função da etapa de imputação do quadro), (03) Imputação de Questionário (quando o registro foi imputado em função da etapa de imputação do questionário), (4) Imputação do Valor do Item informado como componente de um agregado com informação do valor total da despesa


 17 - RENDA MONETÁRIA MENSAL DA UNIDADE DE CONSUMO
 Posição inicial: 60
 Formato: N - Numérico
 Tamanho: 16 dígitos (16 bytes)
 Quantidade de Decimais: 2
      Descrição Sumária: Ver descrição da variável 39 do REGISTRO TIPO 02 – PESSOAS.


 18 - RENDA NÃO MONETÁRIA MENSAL DA UNIDADE DE CONSUMO
 Posição inicial: 76
 Formato: N - Numérico
 Tamanho: 16 dígitos (16 bytes)
 Quantidade de Decimais: 2
      Descrição Sumária: Ver descrição da variável 40 do REGISTRO TIPO 02 – PESSOAS.


 19 - RENDA TOTAL MENSAL DA UNIDADE DE CONSUMO
 Posição inicial: 92
 Formato: N - Numérico
 Tamanho: 16 dígitos (16 bytes)
 Quantidade de Decimais: 2
 Descrição Sumária: Ver descrição da variável 41 do REGISTRO TIPO
02 – PESSOAS.


IMPUTAÇÃO DE VALORES DE ITEM, QUESTIONÁRIO E QUADRO

I - IMPUTAÇÃO DOS VALORES DE ITENS
```

- **POF 2**

***Observações:***

1) No Quadro 12, os itens: Valor do imóvel adquirido em primeira locação à vista, Valor do imóvel adquirido em primeira locação a prazo, Valor do imóvel adquirido usado à vista e Valor do imóvel adquirido usado a prazo ignorados, não foram imputados. Ficando no campo Valor da Despesa Anualizado = 0.00.
2) No Quadro 19, quando o valor do último gasto mensal foi imputado, houve também imputação no Valor do INSS, a espécie e a forma de aquisição, proveniente do mesmo questionário doador.
3) No Quadro 19, quando o valor imputado foi apenas o INSS, utilizou-se para cálculo desta despesa a tabela de Contribuição INSS de Fevereiro de 2009.

- **POF 5**

***Observações:***

**Quadro 53**

1) Quando houve imputação na variável Valor do Rendimento, imputou-se também do mesmo doador caso houvesse, Valor de Dedução: Previdência Pública, Imposto de Renda e Outras Deduções**.**

2) Para a imputação do Valor de Dedução do Imposto de Renda e da Previdência Pública, no caso de EMPREGADOR, cuja posição na ocupação era 01, 02 ou 03, e tendo a informação do Valor do Rendimento, utilizou-se para cálculo as tabelas: Tabela do Imposto de Renda de Pessoa Física para o exercício de 2010, ano calendário de 2009 e Tabela de INSS a partir de fevereiro de 2009 respectivamente.

A variável Valor de Outras Deduções, nestes casos, não receberam imputação

A variável Valor da Dedução dos itens restantes do quadro 53, 54 e 55 não foram passíveis de imputação.

**II - IMPUTAÇÃO DE QUESTIONÁRIOS E QUADROS**

1) As informações dos questionários POF 3 com menos de 3 dias pesquisados, foram desprezadas, e foi imputado todo questionário.
2) Para os informantes em que houve imputação de POF 4 e POF 5, estes receberam as informações de um mesmo doador.
3) Para as situações de não preenchimento do Quadro 14 (Inventário de bens duráveis) do Questionário de Aquisição Coletiva (POF2), o mesmo foi imputado.
4) Para os Informantes em que apenas um dos questionários POF 4 ou POF 5 foi imputado, imputou-se apenas o questionários não preenchido, mesmo que o doador tivesse os dois questionários completos.

**REGISTRO TIPO 06: DESPESA DE 90 DIAS - POF2 / QUADROS 6 A 9**

#### **Tamanho: 145**

```
1 - TIPO DE REGISTRO (Item de Identificação do Dicionário)
            Posição Inicial: 1
            Formato: N - Numérico
            Tamanho: 2 dígitos (2 bytes)
 		Descrição Sumária: Ver descrição da variável 1 do REGISTRO TIPO 01 – DOMICÍLIO.


  		2 – CÓDIGO DA UNIDADE DA FEDERAÇÃO
            Posição Inicial: 3
            Formato: N - Numérico
            Tamanho: 2 dígitos (2 bytes)
 		Quantidade de Categorias: 27
 		Classificação: 900
 		Descrição Sumária: Ver descrição da variável 2 do REGISTRO TIPO 01 – DOMICÍLIO.


  		3 – NÚMERO SEQUENCIAL
            Posição Inicial: 5
            Formato: N - Numérico
            Tamanho: 3 dígitos (3 bytes)
 		Descrição Sumária: Ver descrição da variável 3 do REGISTRO TIPO 01 – DOMICÍLIO.


  		4 – DV DO SEQUENCIAL
            Posição Inicial: 8
            Formato: N - Numérico
            Tamanho: 1 dígito (1 byte)
 		Descrição Sumária: Ver descrição da variável 4 do REGISTRO TIPO 01 – DOMICÍLIO.


  		5 – NÚMERO DO DOMICÍLIO
            Posição Inicial: 9
            Formato: N - Numérico
            Tamanho: 2 dígitos (2 bytes)
Descrição Sumária: Ver descrição da variável 5 do REGISTRO TIPO
01 – DOMICÍLIO.


  	      6 - NÚMERO DA UC
             Posição inicial: 11
             Formato: N - Numérico
             Tamanho: 1 dígito (1 byte)
 	      Descrição Sumária: Ver descrição da variável 6 do REGISTRO TIPO
02 – PESSOAS.


7 - ESTRATO GEOGRÁFICO
            Posição Inicial: 12
            Formato: N - Numérico
            Tamanho: 2 dígitos (2 bytes)
Quantidade de Categorias: 51
Classificação: 901
Descrição Sumária: Ver descrição da variável 6 do REGISTRO TIPO 01 – DOMICÍLIO.


  		8 – FATOR DE EXPANSÃO 1 (DESENHO AMOSTRAL)
            Posição Inicial: 14
            Formato: N - Numérico
            Tamanho: 14 dígitos (14 bytes)
            Quantidade de Decimais: 8
Descrição Sumária: Ver descrição da variável 7 do REGISTRO TIPO 01 – DOMICÍLIO.


  		9 – FATOR DE EXPANSÃO 2 (AJUSTADO P/ ESTIMATIVAS)
            Posição Inicial: 28
            Formato: N - Numérico
            Tamanho: 14 dígitos (14 bytes)
            Quantidade de Decimais: 8
Descrição Sumária: Ver descrição da variável 8 do REGISTRO TIPO 01 – DOMICÍLIO.


10 – NÚMERO DO QUADRO
            Posição Inicial: 42
            Formato: N - Numérico
            Tamanho: 2 dígitos (2 bytes)
 		Quantidade de Categorias: 56
Classificação: 906
Descrição Sumária: Identifica o número do quadro do Questionário de Aquisição Coletiva (POF2) no qual o item de despesa foi registrado.
Neste registro, o número do quadro varia de 6 a 9.


   		11 – CÓDIGO DO ITEM
            Posição Inicial: 44
            Formato: N - Numérico
            Tamanho: 5 dígitos (5 bytes)
 		Quantidade de Categorias: 13476
            Classificação: 907
            Descrição Sumária: Identifica o tipo de despesa. Esta variável deverá ser lida juntamente com a variável “Número do Quadro”, que irá compor o código do produto completo com 7 dígitos.


12 – FORMA DE AQUISIÇÃO
            Posição Inicial: 49
            Formato: N - Numérico
            Tamanho: 2 dígitos (2 bytes)
            Quantidade de Categorias: 12
            Classificação: 903
 		Descrição Sumária: Identifica a forma pela qual o tipo de produto ou serviço foi adquirido pela unidade de consumo no período de referência da pesquisa. Categorias: (00) Não se aplica, (01) Monetária à vista para a Unidade de Consumo, (02) Monetária à vista para Outra Unidade de Consumo, (03) Monetária a prazo para a Unidade de Consumo, (04) Monetária a prazo outra Unidade de Consumo, (05) Cartão de crédito à vista para a Unidade de Consumo, (06) Cartão de crédito à vista para outra Unidade de Consumo, (07) Doação, (08) Retirada do Negócio, (09) Troca, (10) Produção Própria) e (11) Outra.


13 – VALOR DA DESPESA / AQUISIÇÃO
            Posição Inicial: 51
            Formato: N - Numérico
            Tamanho: 11 dígitos (11 bytes)
            Quantidade de Decimais: 2
            Descrição Sumária: Identifica o valor em reais (R$), considerando os centavos, da despesa realizada pela unidade de consumo no período de referência da pesquisa. Nos casos de valores ignorados, este campo está preenchido com “Noves”.
Para os itens do quadro 6, o valor registrado corresponde ao valor da última conta paga dentro do período de referência de 90 dias.


   		14 - FATOR DE ANUALIZAÇÃO
            Posição Inicial: 62
            Formato: N - Numérico
            Tamanho: 2 dígitos (2 bytes)
Descrição Sumária: Identifica o número utilizado para anualizar as despesas ou rendimentos, em função do período de referência definido para coleta. Fatores utilizados = 1, 4, 12 e 52.


15 – DEFLATOR FATOR
            Posição Inicial: 64
            Formato: N - Numérico
            Tamanho: 5 dígitos (5 bytes)
            Quantidade de Decimais: 2
            Descrição Sumária: Identifica o número que multiplicado pelo “VALOR DA DESPESA / AQUISIÇÃO” ou “VALOR DO RENDIMENTO” gera o “VALOR DA DESPESA OU RENDIMENTO DEFLACIONADO”.


16 – VALOR DA DESPESA DEFLACIONADO
            Posição Inicial: 69
            Formato: N - Numérico
            Tamanho: 11 dígitos (11 bytes)
            Quantidade de Decimais: 2
            Descrição Sumária: Identifica o valor resultante da operação “VALOR DA DESPESA / AQUISIÇÃO” multiplicado pelo “DEFLATOR FATOR”.


17 – VALOR DA DESPESA ANUALIZADO E EXPANDIDO (FATOR 2)
            Posição Inicial: 80
            Formato: N - Numérico
            Tamanho: 16 dígitos (16 bytes)
            Quantidade de Decimais: 2
Descrição Sumária: Identifica o valor resultante da operação do “VALOR DA DESPESA DEFLACIONADO”, pelo “FATOR DE ANUALIZAÇÃO” e pelo FATOR DE EXPANSÃO 2 (AJUSTADO P/ ESTIMATIVAS).


18 – CÓDIGO DE IMPUTAÇÃO
            Posição Inicial: 96
            Formato: N - Numérico
            Tamanho: 2 dígitos (2 bytes)
            Quantidade de Categorias: 4
            Classificação: 902
Descrição Sumária: Ver descrição da variável 16 do REGISTRO TIPO 05 – INVENTÁRIO DE BENS DURÁVEIS.


 19 - RENDA MONETÁRIA MENSAL DA UNIDADE DE CONSUMO
 Posição inicial: 98
 Formato: N - Numérico
 Tamanho: 16 dígitos (16 bytes)
 Quantidade de Decimais: 2
      Descrição Sumária: Ver descrição da variável 39 do REGISTRO TIPO 02 – PESSOAS.


 20 - RENDA NÃO MONETÁRIA MENSAL DA UNIDADE DE CONSUMO
 Posição inicial: 114
 Formato: N - Numérico
 Tamanho: 16 dígitos (16 bytes)
 Quantidade de Decimais: 2
      Descrição Sumária: Ver descrição da variável 40 do REGISTRO TIPO 02 – PESSOAS.


21 - RENDA TOTAL MENSAL DA UNIDADE DE CONSUMO
Posição inicial: 130
Formato: N - Numérico
Tamanho: 16 dígitos (16 bytes)
Quantidade de Decimais: 2
Descrição Sumária: Ver descrição da variável 41 do REGISTRO TIPO
02 – PESSOAS.


REGISTRO TIPO 07: DESPESA DE 12 MESES - POF2 / QUADROS 10 A 13
```

#### **Tamanho: 149**

```
1 - TIPO DE REGISTRO (Item de Identificação do Dicionário)            Posição Inicial: 1
            Formato: N - Numérico
            Tamanho: 2 dígitos (2 bytes)
 		Descrição Sumária: Ver descrição da variável 1 do REGISTRO TIPO 01 – DOMICÍLIO.


  		2 – CÓDIGO DA UNIDADE DA FEDERAÇÃO
            Posição Inicial: 3
            Formato: N - Numérico
            Tamanho: 2 dígitos (2 bytes)
 		Quantidade de Categorias: 27
 		Classificação: 900
 		Descrição Sumária: Ver descrição da variável 2 do REGISTRO TIPO 01 – DOMICÍLIO.


  		3 – NÚMERO SEQUENCIAL
            Posição Inicial: 5
            Formato: N - Numérico
            Tamanho: 3 dígitos (3 bytes)
 		Descrição Sumária: Ver descrição da variável 3 do REGISTRO TIPO 01 – DOMICÍLIO.


  		4 – DV DO SEQUENCIAL
            Posição Inicial: 8
            Formato: N - Numérico
            Tamanho: 1 dígito (1 byte)
 		Descrição Sumária: Ver descrição da variável 4 do REGISTRO TIPO 01 – DOMICÍLIO.


  		5 – NÚMERO DO DOMICÍLIO
            Posição Inicial: 9
            Formato: N - Numérico
            Tamanho: 2 dígitos (2 bytes)
Descrição Sumária: Ver descrição da variável 5 do REGISTRO TIPO
01 – DOMICÍLIO.


  		6 - NÚMERO DA UC
             Posição inicial: 11
             Formato: N - Numérico
             Tamanho: 1 dígito (1 byte)
 		Descrição Sumária: Ver descrição da variável 6 do REGISTRO TIPO
02 – PESSOAS.


7 - ESTRATO GEOGRÁFICO
            Posição Inicial: 12
            Formato: N - Numérico
            Tamanho: 2 dígitos (2 bytes)
Quantidade de Categorias: 51
Classificação: 901
Descrição Sumária: Ver descrição da variável 6 do REGISTRO TIPO 01 – DOMICÍLIO.


  		8 – FATOR DE EXPANSÃO 1 (DESENHO AMOSTRAL)
            Posição Inicial: 14
            Formato: N - Numérico
            Tamanho: 14 dígitos (14 bytes)
            Quantidade de Decimais: 8
Descrição Sumária: Ver descrição da variável 7 do REGISTRO TIPO 01 – DOMICÍLIO.


  		9 – FATOR DE EXPANSÃO 2 (AJUSTADO P/ ESTIMATIVAS)
            Posição Inicial: 28
            Formato: N - Numérico
            Tamanho: 14 dígitos (14 bytes)
            Quantidade de Decimais: 8
Descrição Sumária: Ver descrição da variável 8 do REGISTRO TIPO 01 – DOMICÍLIO.


10 – NÚMERO DO QUADRO
            Posição Inicial: 42
            Formato: N - Numérico
            Tamanho: 2 dígitos (2 bytes)
 		Quantidade de Categorias: 56
Classificação: 906
Descrição Sumária: Ver descrição da variável 10 do REGISTRO TIPO 06 – DESPESA DE 90 DIAS.
Neste registro, o número do quadro varia de 10 a 13.


   		11 – CÓDIGO DO ITEM
            Posição Inicial: 44
            Formato: N - Numérico
            Tamanho: 5 dígitos (5 bytes)
 		Quantidade de Categorias: 13476
            Classificação: 907
 		Descrição Sumária: Ver descrição da variável 11 do REGISTRO TIPO 06 – DESPESA DE 90 DIAS.


12 – FORMA DE AQUISIÇÃO
            Posição Inicial: 49
            Formato: N - Numérico
            Tamanho: 2 dígitos (2 bytes)
            Quantidade de Categorias: 12
 		Classificação: 903
 		Descrição Sumária: Ver descrição da variável 12 do REGISTRO TIPO 06 – DESPESA DE 90 DIAS.


13 – VALOR DA DESPESA / AQUISIÇÃO
            Posição Inicial: 51
            Formato: N - Numérico
            Tamanho: 11 dígitos (11 bytes)
            Quantidade de Decimais: 2
		Descrição Sumária: Ver descrição da variável 13 do REGISTRO TIPO 06 – DESPESA DE 90 DIAS.
Para os itens do quadro 10, o valor registrado corresponde ao valor da última despesa mensal realizada dentro do período de referência de 12 meses.


14 – MÊS DA ÚLTIMA DESPESA
            Posição Inicial: 62
            Formato: N – Numérico
            Tamanho: 2 dígitos (2 bytes)
            Quantidade de Categorias = 13
            Classificação: 904
Descrição Sumária: Identifica o último mês que a despesa foi realizada pela unidade de consumo no período de referência da pesquisa. Categorias: (00) Não se aplica, (01) Janeiro, (02) Fevereiro, (03) Março, (04) Abril, (05) Maio, (06) Junho, (07) Julho, (08) Agosto, (09) Setembro, (10) Outubro, (11) Novembro e (12) Dezembro.
Neste registro, esta variável é exclusiva para os itens do quadro 10.


            15 – NÚMERO DE MESES
            Posição Inicial: 64
            Formato: N – Numérico
            Tamanho: 2 dígitos (2 bytes)
Descrição Sumária: Identifica o número de meses que a despesa foi realizada pela unidade de consumo no período de referência da pesquisa. O valor desta variável é “Zero” para os casos onde não se aplica.
Neste registro, esta variável é exclusiva para os itens do quadro 10.


   		16 - FATOR DE ANUALIZAÇÃO
            Posição Inicial: 66
            Formato: N - Numérico
            Tamanho: 2 dígitos (2 bytes)
		Descrição Sumária: Ver descrição da variável 14 do REGISTRO TIPO 06 – DESPESA DE 90 DIAS.


17 – DEFLATOR FATOR
            Posição Inicial: 68
            Formato: N - Numérico
            Tamanho: 5 dígitos (5 bytes)
            Quantidade de Decimais: 2
Descrição Sumária: Ver descrição da variável 15 do REGISTRO TIPO 06 – DESPESA DE 90 DIAS.


18 – VALOR DA DESPESA DEFLACIONADO
            Posição Inicial: 73
            Formato: N - Numérico
            Tamanho: 11 dígitos (11 bytes)
            Quantidade de Decimais: 2
Descrição Sumária: Ver descrição da variável 16 do REGISTRO TIPO 06 – DESPESA DE 90 DIAS.


19 – VALOR DA DESPESA ANUALIZADO E EXPANDIDO (FATOR 2)
            Posição Inicial: 84
            Formato: N - Numérico
            Tamanho: 16 dígitos (16 bytes)
            Quantidade de Decimais: 2
 		Descrição Sumária: Ver descrição da variável 17 do REGISTRO TIPO 06 – DESPESA DE 90 DIAS.
		Para os itens do quadro 10, deve-se multiplicar também pela variável NÚMERO DE MESES.


20 – CÓDIGO DE IMPUTAÇÃO
            Posição Inicial: 100
            Formato: N - Numérico
            Tamanho: 2 dígitos (2 bytes)
            Quantidade de Categorias: 4
 		Classificação: 902
Descrição Sumária: Ver descrição da variável 16 do REGISTRO TIPO 05 – INVENTÁRIO DE BENS DURÁVEIS.


 21 - RENDA MONETÁRIA MENSAL DA UNIDADE DE CONSUMO
 Posição inicial: 102
 Formato: N - Numérico
 Tamanho: 16 dígitos (16 bytes)
 Quantidade de Decimais: 2
      Descrição Sumária: Ver descrição da variável 39 do REGISTRO TIPO 02 – PESSOAS.


 22 - RENDA NÃO MONETÁRIA MENSAL DA UNIDADE DE CONSUMO
 Posição inicial: 118
 Formato: N - Numérico
 Tamanho: 16 dígitos (16 bytes)
 Quantidade de Decimais: 2
      Descrição Sumária: Ver descrição da variável 40 do REGISTRO TIPO 02 – PESSOAS.


 23 - RENDA TOTAL MENSAL DA UNIDADE DE CONSUMO
 Posição inicial: 134
 Formato: N - Numérico
 Tamanho: 16 dígitos (16 bytes)
 Quantidade de Decimais: 2
 Descrição Sumária: Ver descrição da variável 41 do REGISTRO TIPO
02 – PESSOAS.


REGISTRO TIPO 08: OUTRAS DESPESAS - POF2 / QUADROS 15 A 18
```

#### **Tamanho: 146**

```
1 - TIPO DE REGISTRO (Item de Identificação do Dicionário)            Posição Inicial: 1
            Formato: N - Numérico
            Tamanho: 2 dígitos (2 bytes)
 		Descrição Sumária: Ver descrição da variável 1 do REGISTRO TIPO 01 – DOMICÍLIO.


  		2 – CÓDIGO DA UNIDADE DA FEDERAÇÃO
            Posição Inicial: 3
            Formato: N - Numérico
            Tamanho: 2 dígitos (2 bytes)
 		Quantidade de Categorias: 27
 		Classificação: 900
 		Descrição Sumária: Ver descrição da variável 2 do REGISTRO TIPO 01 – DOMICÍLIO.


  		3 – NÚMERO SEQUENCIAL
            Posição Inicial: 5
            Formato: N - Numérico
            Tamanho: 3 dígitos (3 bytes)
```

Descrição Sumária: Ver descrição da variável 3 do REGISTRO TIPO 01 – DOMICÍLIO.

```
  		4 – DV DO SEQUENCIAL
            Posição Inicial: 8
            Formato: N - Numérico
            Tamanho: 1 dígito (1 byte)
 		Descrição Sumária: Ver descrição da variável 4 do REGISTRO TIPO 01 – DOMICÍLIO.


  		5 – NÚMERO DO DOMICÍLIO
            Posição Inicial: 9
            Formato: N - Numérico
            Tamanho: 2 dígitos (2 bytes)
Descrição Sumária: Ver descrição da variável 5 do REGISTRO TIPO
01 – DOMICÍLIO.


  		6 - NÚMERO DA UC
             Posição inicial: 11
             Formato: N - Numérico
             Tamanho: 1 dígito (1 byte)
 		Descrição Sumária: Ver descrição da variável 6 do REGISTRO TIPO
02 – PESSOAS.


7 - ESTRATO GEOGRÁFICO
            Posição Inicial: 12
            Formato: N - Numérico
            Tamanho: 2 dígitos (2 bytes)
Quantidade de Categorias: 51
Classificação: 901
Descrição Sumária: Ver descrição da variável 6 do REGISTRO TIPO 01 – DOMICÍLIO.


  		8 – FATOR DE EXPANSÃO 1 (DESENHO AMOSTRAL)
            Posição Inicial: 14
            Formato: N - Numérico
            Tamanho: 14 dígitos (14 bytes)
            Quantidade de Decimais: 8
Descrição Sumária: Ver descrição da variável 7 do REGISTRO TIPO 01 – DOMICÍLIO.


  		9 – FATOR DE EXPANSÃO 2 (AJUSTADO P/ ESTIMATIVAS)
            Posição Inicial: 28
            Formato: N - Numérico
            Tamanho: 14 dígitos (14 bytes)
            Quantidade de Decimais: 8
Descrição Sumária: Ver descrição da variável 8 do REGISTRO TIPO 01 – DOMICÍLIO.


10 – NÚMERO DO QUADRO
            Posição Inicial: 42
            Formato: N - Numérico
            Tamanho: 2 dígitos (2 bytes)
 		Quantidade de Categorias: 56
Classificação: 906
Descrição Sumária: Ver descrição da variável 10 do REGISTRO TIPO 06 – DESPESA DE 90 DIAS.
Neste registro, o número do quadro varia de 15 a 18.


   		11 – CÓDIGO DO ITEM
            Posição Inicial: 44
            Formato: N - Numérico
            Tamanho: 5 dígitos (5 bytes)
 		Quantidade de Categorias: 13476
            Classificação: 907
 		Descrição Sumária: Ver descrição da variável 11 do REGISTRO TIPO 06 – DESPESA DE 90 DIAS.


12 – FORMA DE AQUISIÇÃO
            Posição Inicial: 49
            Formato: N - Numérico
            Tamanho: 2 dígitos (2 bytes)
            Quantidade de Categorias: 12
 		Classificação: 903
 		Descrição Sumária: Ver descrição da variável 12 do REGISTRO TIPO 06 – DESPESA DE 90 DIAS.


13 – VALOR DA DESPESA / AQUISIÇÃO
            Posição Inicial: 51
            Formato: N - Numérico
            Tamanho: 11 dígitos (11 bytes)
            Quantidade de Decimais: 2
		Descrição Sumária: Ver descrição da variável 13 do REGISTRO TIPO 06 – DESPESA DE 90 DIAS.


14 – ESTADO DE AQUISIÇÃO
Posição Inicial: 62
            Formato: N - Numérico
            Tamanho: 1 dígito (1 byte)
            Quantidade de Categorias: 3
 		Classificação: 905
Descrição Sumária: Identifica o estado do produto adquirido pela unidade de consumo no período de referencia da pesquisa. Categorias: (1) Novo, (3) Usado e 9 (Não Sabe).


   		15 - FATOR DE ANUALIZAÇÃO
            Posição Inicial: 63
            Formato: N - Numérico
            Tamanho: 2 dígitos (2 bytes)
		Descrição Sumária: Ver descrição da variável 14 do REGISTRO TIPO 06 – DESPESA DE 90 DIAS.


16 – DEFLATOR FATOR
            Posição Inicial: 65
            Formato: N - Numérico
            Tamanho: 5 dígitos (5 bytes)
            Quantidade de Decimais: 2
Descrição Sumária: Ver descrição da variável 15 do REGISTRO TIPO 06 – DESPESA DE 90 DIAS.


17 – VALOR DA DESPESA DEFLACIONADO
            Posição Inicial: 70
            Formato: N - Numérico
            Tamanho: 11 dígitos (11 bytes)
            Quantidade de Decimais: 2
Descrição Sumária: Ver descrição da variável 16 do REGISTRO TIPO 06 – DESPESA DE 90 DIAS.


18 – VALOR DA DESPESA ANUALIZADO E EXPANDIDO (FATOR 2)
            Posição Inicial: 81
            Formato: N - Numérico
            Tamanho: 16 dígitos (16 bytes)
            Quantidade de Decimais: 2
 		Descrição Sumária: Ver descrição da variável 17 do REGISTRO TIPO 06 – DESPESA DE 90 DIAS.


19 – CÓDIGO DE IMPUTAÇÃO
            Posição Inicial: 97
            Formato: N - Numérico
            Tamanho: 2 dígitos (2 bytes)
            Quantidade de Categorias: 4
 		Classificação: 902
Descrição Sumária: Ver descrição da variável 16 do REGISTRO TIPO 05 – INVENTÁRIO DE BENS DURÁVEIS.


 20 - RENDA MONETÁRIA MENSAL DA UNIDADE DE CONSUMO
 Posição inicial: 99
 Formato: N - Numérico
 Tamanho: 16 dígitos (16 bytes)
 Quantidade de Decimais: 2
      Descrição Sumária: Ver descrição da variável 39 do REGISTRO TIPO 02 – PESSOAS.


 21 - RENDA NÃO MONETÁRIA MENSAL DA UNIDADE DE CONSUMO
 Posição inicial: 115
 Formato: N - Numérico
 Tamanho: 16 dígitos (16 bytes)
 Quantidade de Decimais: 2
      Descrição Sumária: Ver descrição da variável 40 do REGISTRO TIPO 02 – PESSOAS.


 22 - RENDA TOTAL MENSAL DA UNIDADE DE CONSUMO
 Posição inicial: 131
 Formato: N - Numérico
 Tamanho: 16 dígitos (16 bytes)
 Quantidade de Decimais: 2
 Descrição Sumária: Ver descrição da variável 41 do REGISTRO TIPO
02 – PESSOAS.


REGISTRO TIPO 09: DESPESAS COM SERVIÇOS DOMÉSTICOS - POF2 / QUADRO 19
```

#### **Tamanho: 195**

```
1 - TIPO DE REGISTRO (Item de Identificação do Dicionário)            Posição Inicial: 1
 		Formato: N - Numérico
            Tamanho: 2 dígitos (2 bytes)
 		Descrição Sumária: Ver descrição da variável 1 do REGISTRO TIPO 01 – DOMICÍLIO.


  		2 – CÓDIGO DA UNIDADE DA FEDERAÇÃO
            Posição Inicial: 3
            Formato: N - Numérico
            Tamanho: 2 dígitos (2 bytes)
 		Quantidade de Categorias: 27
 		Classificação: 900
 		Descrição Sumária: Ver descrição da variável 2 do REGISTRO TIPO 01 – DOMICÍLIO.


  		3 – NÚMERO SEQUENCIAL
            Posição Inicial: 5
            Formato: N - Numérico
            Tamanho: 3 dígitos (3 bytes)
```

Descrição Sumária: Ver descrição da variável 3 do REGISTRO TIPO 01 – DOMICÍLIO.

```
  		4 – DV DO SEQUENCIAL
            Posição Inicial: 8
            Formato: N - Numérico
            Tamanho: 1 dígito (1 byte)
 		Descrição Sumária: Ver descrição da variável 4 do REGISTRO TIPO 01 – DOMICÍLIO.


  		5 – NÚMERO DO DOMICÍLIO
            Posição Inicial: 9
            Formato: N - Numérico
            Tamanho: 2 dígitos (2 bytes)
Descrição Sumária: Ver descrição da variável 5 do REGISTRO TIPO
01 – DOMICÍLIO.


  		6 - NÚMERO DA UC
             Posição inicial: 11
             Formato: N - Numérico
             Tamanho: 1 dígito (1 byte)
 		Descrição Sumária: Ver descrição da variável 6 do REGISTRO TIPO
02 – PESSOAS.


7 - ESTRATO GEOGRÁFICO
            Posição Inicial: 12
            Formato: N - Numérico
            Tamanho: 2 dígitos (2 bytes)
Quantidade de Categorias: 51
Classificação: 901
Descrição Sumária: Ver descrição da variável 6 do REGISTRO TIPO 01 – DOMICÍLIO.


  		8 – FATOR DE EXPANSÃO 1 (DESENHO AMOSTRAL)
            Posição Inicial: 14
            Formato: N - Numérico
            Tamanho: 14 dígitos (14 bytes)
            Quantidade de Decimais: 8
Descrição Sumária: Ver descrição da variável 7 do REGISTRO TIPO 01 – DOMICÍLIO.


  		9 – FATOR DE EXPANSÃO 2 (AJUSTADO P/ ESTIMATIVAS)
            Posição Inicial: 28
            Formato: N - Numérico
            Tamanho: 14 dígitos (14 bytes)
            Quantidade de Decimais: 8
Descrição Sumária: Ver descrição da variável 8 do REGISTRO TIPO 01 – DOMICÍLIO.


10 – NÚMERO DO QUADRO
            Posição Inicial: 42
            Formato: N - Numérico
            Tamanho: 2 dígitos (2 bytes)
 		Quantidade de Categorias: 56
Classificação: 906
Descrição Sumária: Ver descrição da variável 10 do REGISTRO TIPO 06 – DESPESA DE 90 DIAS.
Neste registro, o número do quadro é sempre 19.


   		11 – CÓDIGO DO ITEM
            Posição Inicial: 44
            Formato: N - Numérico
            Tamanho: 5 dígitos (5 bytes)
 		Quantidade de Categorias: 13476
            Classificação: 907
 		Descrição Sumária: Ver descrição da variável 11 do REGISTRO TIPO 06 – DESPESA DE 90 DIAS.


12 – FORMA DE AQUISIÇÃO
            Posição Inicial: 49
            Formato: N - Numérico
            Tamanho: 2 dígitos (2 bytes)
            Quantidade de Categorias: 12
 		Classificação: 903
 		Descrição Sumária: Ver descrição da variável 12 do REGISTRO TIPO 06 – DESPESA DE 90 DIAS.


13 – VALOR DA DESPESA / AQUISIÇÃO
            Posição Inicial: 51
            Formato: N - Numérico
            Tamanho: 11 dígitos (11 bytes)
            Quantidade de Decimais: 2
		Descrição Sumária: Ver descrição da variável 13 do REGISTRO TIPO 06 – DESPESA DE 90 DIAS.
Para os itens do quadro 19, o valor registrado corresponde ao valor da última despesa mensal realizada dentro do período de referência de 12 meses.


14 – CÓDIGO DO INSS
Posição Inicial: 62
            Formato: N - Numérico
            Tamanho: 5 dígitos (5 bytes)
            Quantidade de Categorias: 54
            Classificação: 500
            Descrição Sumária: Identifica o tipo de despesa com INSS. Esta variável deverá ser lida juntamente com a variável “Número do Quadro”, que irá compor o código do produto completo com 7 dígitos.


15 – VALOR DO INSS
            Posição Inicial: 67
            Formato: N - Numérico
            Tamanho: 11 dígitos (11 bytes)
            Quantidade de Decimais: 2
            Descrição Sumária: Identifica o valor em reais (R$), considerando os centavos, da despesa com INSS realizada pela unidade de consumo no período de referência da pesquisa. Nos casos de valores do INSS ignorados, este campo está preenchido com “Noves”. Nos casos em que não ocorreu despesa com INSS, este campo esta preenchido com “Zeros”.


   		16 – CÓDIGO DA ESPÉCIE
            Posição Inicial: 78
            Formato: N - Numérico
            Tamanho: 1 dígito (1 byte)
            Quantidade de Categorias: 3
            Classificação: 501
            Descrição Sumária: Identifica a espécie em que o serviço doméstico foi contratado pela unidade de consumo no período de referência da pesquisa. Categorias: (1) Mensalista, (3) Diarista e (9) Não Sabe.


17 – MÊS DA ÚLTIMA DESPESA
            Posição Inicial: 79
            Formato: N – Numérico
            Tamanho: 2 dígitos (2 bytes)
            Quantidade de Categorias = 13
            Classificação: 904
Descrição Sumária: Ver descrição da variável 14 do REGISTRO TIPO 07 – DESPESA DE 12 MESES.


            18 - NÚMERO DE MESES
            Posição Inicial: 81
            Formato: N – Numérico
            Tamanho: 2 dígitos (2 bytes)
Descrição Sumária: Ver descrição da variável 15 do REGISTRO TIPO 07 – DESPESA DE 12 MESES.


   		19 - FATOR DE ANUALIZAÇÃO
            Posição Inicial: 83
            Formato: N - Numérico
            Tamanho: 2 dígitos (2 bytes)
		Descrição Sumária: Ver descrição da variável 14 do REGISTRO TIPO 06 – DESPESA DE 90 DIAS.


20 – DEFLATOR FATOR
            Posição Inicial: 85
            Formato: N - Numérico
            Tamanho: 5 dígitos (5 bytes)
            Quantidade de Decimais: 2
Descrição Sumária: Ver descrição da variável 15 do REGISTRO TIPO 06 – DESPESA DE 90 DIAS.


21 – VALOR DA DESPESA DEFLACIONADO
            Posição Inicial: 90
            Formato: N - Numérico
            Tamanho: 11 dígitos (11 bytes)
            Quantidade de Decimais: 2
Descrição Sumária: Ver descrição da variável 16 do REGISTRO TIPO 06 – DESPESA DE 90 DIAS.


22 – VALOR DO INSS DEFLACIONADO
            Posição Inicial: 101
            Formato: N - Numérico
            Tamanho: 11 dígitos (11 bytes)
            Quantidade de Decimais: 2
 		Descrição Sumária: Identifica o valor resultante da operação “VALOR DO INSS” multiplicado pelo “DEFLATOR  FATOR”.


23 – VALOR DA DESPESA ANUALIZADO E EXPANDIDO (FATOR 2)
            Posição Inicial: 112
            Formato: N - Numérico
            Tamanho: 16 dígitos (16 bytes)
            Quantidade de Decimais: 2
 		Descrição Sumária: Identifica o valor resultante da operação do “VALOR DA DESPESA DEFLACIONADO” pelo NÚMERO DE MESES, pelo “FATOR DE ANUALIZAÇÃO” e pelo FATOR DE EXPANSÃO 2 (AJUSTADO P/ ESTIMATIVAS).


24 – VALOR DO INSS ANUALIZADO E EXPANDIDO (FATOR 2)
            Posição Inicial: 128
            Formato: N - Numérico
            Tamanho: 16 dígitos (16 bytes)
            Quantidade de Decimais: 2
 		Descrição Sumária: Identifica o valor resultante da operação do “VALOR DO INSS DEFLACIONADO”, pelo NÚMERO DE MESES, pelo “FATOR DE ANUALIZAÇÃO” e pelo FATOR DE EXPANSÃO 2 (AJUSTADO P/ ESTIMATIVAS).


25 – CÓDIGO DE IMPUTAÇÃO DA DESPESA
            Posição Inicial: 144
            Formato: N - Numérico
            Tamanho: 2 dígitos (2 bytes)
            Quantidade de Categorias: 4
 		Classificação: 902
Descrição Sumária: Ver descrição da variável 16 do REGISTRO TIPO 05 – INVENTÁRIO DE BENS DURÁVEIS.


26 – CÓDIGO DE IMPUTAÇÃO DO INSS
            Posição Inicial: 146
            Formato: N - Numérico
            Tamanho: 2 dígitos (2 bytes)
            Quantidade de Categorias: 4
 		Classificação: 902
Descrição Sumária: Ver descrição da variável 16 do REGISTRO TIPO 05 – INVENTÁRIO DE BENS DURÁVEIS.


27 - RENDA MONETÁRIA MENSAL DA UNIDADE DE CONSUMO
 Posição inicial: 148
 Formato: N - Numérico
 Tamanho: 16 dígitos (16 bytes)
 Quantidade de Decimais: 2
      Descrição Sumária: Ver descrição da variável 39 do REGISTRO TIPO 02 – PESSOAS.


 28 - RENDA NÃO MONETÁRIA MENSAL DA UNIDADE DE CONSUMO
 Posição inicial: 164
 Formato: N - Numérico
 Tamanho: 16 dígitos (16 bytes)
 Quantidade de Decimais: 2
      Descrição Sumária: Ver descrição da variável 40 do REGISTRO TIPO 02 – PESSOAS.


 29 - RENDA TOTAL MENSAL DA UNIDADE DE CONSUMO
 Posição inicial: 180
 Formato: N - Numérico
 Tamanho: 16 dígitos (16 bytes)
 Quantidade de Decimais: 2
 Descrição Sumária: Ver descrição da variável 41 do REGISTRO TIPO
02 – PESSOAS.


REGISTRO TIPO 10: ALUGUEL ESTIMADO – POF1 / QUADRO 2
```

#### **Tamanho: 149**

```
1 - TIPO DE REGISTRO (Item de Identificação do Dicionário)            Posição Inicial: 1
            Formato: N - Numérico
            Tamanho: 2 dígitos (2 bytes)
 		Descrição Sumária: Ver descrição da variável 1 do REGISTRO TIPO 01 – DOMICÍLIO.


  		2 – CÓDIGO DA UNIDADE DA FEDERAÇÃO
            Posição Inicial: 3
            Formato: N - Numérico
            Tamanho: 2 dígitos (2 bytes)
 		Quantidade de Categorias: 27
 		Classificação: 900
 		Descrição Sumária: Ver descrição da variável 2 do REGISTRO TIPO 01 – DOMICÍLIO.


  		3 – NÚMERO SEQUENCIAL
            Posição Inicial: 5
            Formato: N - Numérico
            Tamanho: 3 dígitos (3 bytes)
 		Descrição Sumária: Ver descrição da variável 3 do REGISTRO TIPO 01 – DOMICÍLIO.


  		4 – DV DO SEQUENCIAL
            Posição Inicial: 8
            Formato: N - Numérico
            Tamanho: 1 dígito (1 byte)
 		Descrição Sumária: Ver descrição da variável 4 do REGISTRO TIPO 01 – DOMICÍLIO.


  		5 – NÚMERO DO DOMICÍLIO
            Posição Inicial: 9
            Formato: N - Numérico
            Tamanho: 2 dígitos (2 bytes)
Descrição Sumária: Ver descrição da variável 5 do REGISTRO TIPO
01 – DOMICÍLIO.


  		6 - NÚMERO DA UC
             Posição inicial: 11
             Formato: N - Numérico
             Tamanho: 1 dígito (1 byte)
 		Descrição Sumária: Ver descrição da variável 6 do REGISTRO TIPO
02 – PESSOAS.


7 - ESTRATO GEOGRÁFICO
            Posição Inicial: 12
            Formato: N - Numérico
            Tamanho: 2 dígitos (2 bytes)
Quantidade de Categorias: 51
Classificação: 901
Descrição Sumária: Ver descrição da variável 6 do REGISTRO TIPO 01 – DOMICÍLIO.


  		8 – FATOR DE EXPANSÃO 1 (DESENHO AMOSTRAL)
            Posição Inicial: 14
            Formato: N - Numérico
            Tamanho: 14 dígitos (14 bytes)
            Quantidade de Decimais: 8
Descrição Sumária: Ver descrição da variável 7 do REGISTRO TIPO 01 – DOMICÍLIO.


  		9 – FATOR DE EXPANSÃO 2 (AJUSTADO P/ ESTIMATIVAS)
            Posição Inicial: 28
            Formato: N - Numérico
            Tamanho: 14 dígitos (14 bytes)
            Quantidade de Decimais: 8
Descrição Sumária: Ver descrição da variável 8 do REGISTRO TIPO 01 – DOMICÍLIO.


10 – NÚMERO DO QUADRO
            Posição Inicial: 42
            Formato: N - Numérico
            Tamanho: 2 dígitos (2 bytes)
 		Quantidade de Categorias: 56
Classificação: 906
Descrição Sumária: Ver descrição da variável 10 do REGISTRO TIPO 06 – DESPESA DE 90 DIAS.
Neste registro, o número do quadro é sempre 10. O item Aluguel Estimado foi pesquisado no quadro 2 do POF 1, mas para facilitar a geração dos resultados da pesquisa o mesmo foi tratado como um item do quadro 10 sendo que neste registro constam as mesmas variáveis do registro Tipo 7, referente as despesas de 12 meses.


   		11 – CÓDIGO DO ITEM
            Posição Inicial: 44
            Formato: N - Numérico
            Tamanho: 5 dígitos (5 bytes)
 		Quantidade de Categorias: 13476
            Classificação: 907
 		Descrição Sumária: Ver descrição da variável 11 do REGISTRO TIPO 06 – DESPESA DE 90 DIAS.


12 – FORMA DE AQUISIÇÃO
            Posição Inicial: 49
            Formato: N - Numérico
            Tamanho: 2 dígitos (2 bytes)
            Quantidade de Categorias: 12
 		Classificação: 903
 		Descrição Sumária: Ver descrição da variável 12 do REGISTRO TIPO 06 – DESPESA DE 90 DIAS.


13 – VALOR DA DESPESA / AQUISIÇÃO
            Posição Inicial: 51
            Formato: N - Numérico
            Tamanho: 11 dígitos (11 bytes)
            Quantidade de Decimais: 2
		Descrição Sumária: Ver descrição da variável 13 do REGISTRO TIPO 06 – DESPESA DE 90 DIAS.
Para os itens do quadro 10, o valor registrado corresponde ao valor da última despesa mensal realizada dentro do período de referência de 90 dias.


14 – MÊS DA ÚLTIMA DESPESA
            Posição Inicial: 62
            Formato: N – Numérico
            Tamanho: 2 dígitos (2 bytes)
            Quantidade de Categorias = 13
            Classificação: 904
Descrição Sumária: Ver descrição da variável 14 do REGISTRO TIPO 07 – DESPESA DE 12 MESES.


15 – NÚMERO DE MESES
            Posição Inicial: 64
            Formato: N – Numérico
            Tamanho: 2 dígitos (2 bytes)
            Descrição Sumária: Ver descrição da variável 15 do REGISTRO TIPO 07 – DESPESA DE 12 MESES.


   		16 - FATOR DE ANUALIZAÇÃO
            Posição Inicial: 66
            Formato: N - Numérico
            Tamanho: 2 dígitos (2 bytes)
		Descrição Sumária: Ver descrição da variável 14 do REGISTRO TIPO 06 – DESPESA DE 90 DIAS.


17 – DEFLATOR FATOR
            Posição Inicial: 68
            Formato: N - Numérico
            Tamanho: 5 dígitos (5 bytes)
            Quantidade de Decimais: 2
Descrição Sumária: Ver descrição da variável 15 do REGISTRO TIPO 06 – DESPESA DE 90 DIAS.


18 – VALOR DA DESPESA DEFLACIONADO
            Posição Inicial: 73
            Formato: N - Numérico
            Tamanho: 11 dígitos (11 bytes)
            Quantidade de Decimais: 2
Descrição Sumária: Ver descrição da variável 16 do REGISTRO TIPO 06 – DESPESA DE 90 DIAS.


19 – VALOR DA DESPESA ANUALIZADO E EXPANDIDO (FATOR 2)
            Posição Inicial: 84
            Formato: N - Numérico
            Tamanho: 16 dígitos (16 bytes)
            Quantidade de Decimais: 2
 		Descrição Sumária: Ver descrição da variável 17 do REGISTRO TIPO 06 – DESPESA DE 90 DIAS.


20 – CÓDIGO DE IMPUTAÇÃO
            Posição Inicial: 100
            Formato: N - Numérico
            Tamanho: 2 dígitos (2 bytes)
            Quantidade de Categorias: 4
            Classificação: 902
Descrição Sumária: Ver descrição da variável 16 do REGISTRO TIPO 05 – INVENTÁRIO DE BENS DURÁVEIS.


 21 - RENDA MONETÁRIA MENSAL DA UNIDADE DE CONSUMO
 Posição inicial: 102
 Formato: N - Numérico
 Tamanho: 16 dígitos (16 bytes)
 Quantidade de Decimais: 2
      Descrição Sumária: Ver descrição da variável 39 do REGISTRO TIPO 02 – PESSOAS.


 22 - RENDA NÃO MONETÁRIA MENSAL DA UNIDADE DE CONSUMO
 Posição inicial: 118
 Formato: N - Numérico
 Tamanho: 16 dígitos (16 bytes)
 Quantidade de Decimais: 2
      Descrição Sumária: Ver descrição da variável 40 do REGISTRO TIPO 02 – PESSOAS.


 23 - RENDA TOTAL MENSAL DA UNIDADE DE CONSUMO
 Posição inicial: 134
 Formato: N - Numérico
 Tamanho: 16 dígitos (16 bytes)
 Quantidade de Decimais: 2
 Descrição Sumária: Ver descrição da variável 41 do REGISTRO TIPO
02 – PESSOAS.


REGISTRO TIPO 11: CADERNETA DE DESPESA – POF 3
```

#### **Tamanho: 147**

```
1 - TIPO DE REGISTRO (Item de Identificação do Dicionário)            Posição Inicial: 1
            Formato: N - Numérico
            Tamanho: 2 dígitos (2 bytes)
 		Descrição Sumária: Ver descrição da variável 1 do REGISTRO TIPO 01 – DOMICÍLIO.


  		2 – CÓDIGO DA UNIDADE DA FEDERAÇÃO
            Posição Inicial: 3
            Formato: N - Numérico
            Tamanho: 2 dígitos (2 bytes)
 		Quantidade de Categorias: 27
 		Classificação: 900
 		Descrição Sumária: Ver descrição da variável 2 do REGISTRO TIPO 01 – DOMICÍLIO.


  		3 – NÚMERO SEQUENCIAL
            Posição Inicial: 5
            Formato: N - Numérico
            Tamanho: 3 dígitos (3 bytes)
 		Descrição Sumária: Ver descrição da variável 3 do REGISTRO TIPO 01 – DOMICÍLIO.


  		4 – DV DO SEQUENCIAL
            Posição Inicial: 8
            Formato: N - Numérico
            Tamanho: 1 dígito (1 byte)
 		Descrição Sumária: Ver descrição da variável 4 do REGISTRO TIPO 01 – DOMICÍLIO.


  		5 – NÚMERO DO DOMICÍLIO
            Posição Inicial: 9
            Formato: N - Numérico
            Tamanho: 2 dígitos (2 bytes)
Descrição Sumária: Ver descrição da variável 5 do REGISTRO TIPO
01 – DOMICÍLIO.


  		6 - NÚMERO DA UC
             Posição inicial: 11
             Formato: N - Numérico
             Tamanho: 1 dígito (1 byte)
 		Descrição Sumária: Ver descrição da variável 6 do REGISTRO TIPO
02 – PESSOAS.


7 - ESTRATO GEOGRÁFICO
            Posição Inicial: 12
            Formato: N - Numérico
            Tamanho: 2 dígitos (2 bytes)
Quantidade de Categorias: 51
Classificação: 901
Descrição Sumária: Ver descrição da variável 6 do REGISTRO TIPO 01 – DOMICÍLIO.


  		8 – FATOR DE EXPANSÃO 1 (DESENHO AMOSTRAL)
            Posição Inicial: 14
            Formato: N - Numérico
            Tamanho: 14 dígitos (14 bytes)
            Quantidade de Decimais: 8
Descrição Sumária: Ver descrição da variável 7 do REGISTRO TIPO 01 – DOMICÍLIO.


  		9 – FATOR DE EXPANSÃO 2 (AJUSTADO P/ ESTIMATIVAS)
            Posição Inicial: 28
            Formato: N - Numérico
            Tamanho: 14 dígitos (14 bytes)
            Quantidade de Decimais: 8
Descrição Sumária: Ver descrição da variável 8 do REGISTRO TIPO 01 – DOMICÍLIO.


10 – NÚMERO DO QUADRO
            Posição Inicial: 42
            Formato: N - Numérico
            Tamanho: 2 dígitos (2 bytes)
 		Quantidade de Categorias: 56
Classificação: 906
Descrição Sumária: Identifica o número do quadro do Questionário de Aquisição Coletiva (POF3) no qual o item de despesa foi registrado.
Neste registro, o número do quadro varia de 63 a 69.


11 – NÚMERO DO GRUPO DE DESPESA
                Posição Inicial: 44
             Formato: N - Numérico
             Tamanho: 2 dígitos (2 bytes)
            Quantidade de Categorias: 26
             Classificação: 600
             Descrição Sumária: Identifica o grupo de despesa ao qual pertence o produto adquirido pela unidade de consumo no período de referência da pesquisa. Esta variável existe somente neste registro onde são englobadas as informações registradas na Caderneta de Despesa Coletiva – POF3.

   		12 – CÓDIGO DO ITEM
            Posição Inicial: 46
            Formato: N - Numérico
            Tamanho: 5 dígitos (5 bytes)
Quantidade de Categorias: 13476
            Classificação: 907
 		Descrição Sumária: Identifica o tipo de despesa. No caso específico desse registro, esta variável deverá ser lida juntamente com a variável “NÚMERO DO GRUPO DE DESPESA”, que irá compor o código do produto completo com 7 dígitos.


13 – FORMA DE AQUISIÇÃO
            Posição Inicial: 51
            Formato: N - Numérico
            Tamanho: 2 dígitos (2 bytes)
            Quantidade de Categorias: 12
 		Classificação: 903
 		Descrição Sumária: Ver descrição da variável 12 do REGISTRO TIPO 06 – DESPESA DE 90 DIAS.


14 – VALOR DA DESPESA / AQUISIÇÃO
            Posição Inicial: 53
            Formato: N - Numérico
            Tamanho: 11 dígitos (11 bytes)
            Quantidade de Decimais: 2
		Descrição Sumária: Ver descrição da variável 13 do REGISTRO TIPO 06 – DESPESA DE 90 DIAS.


   		15 - FATOR DE ANUALIZAÇÃO
            Posição Inicial: 64
            Formato: N - Numérico
            Tamanho: 2 dígitos (2 bytes)
		Descrição Sumária: Ver descrição da variável 14 do REGISTRO TIPO 06 – DESPESA DE 90 DIAS.


16 – DEFLATOR FATOR
            Posição Inicial: 66
            Formato: N - Numérico
            Tamanho: 5 dígitos (5 bytes)
            Quantidade de Decimais: 2
Descrição Sumária: Ver descrição da variável 15 do REGISTRO TIPO 06 – DESPESA DE 90 DIAS.


17 – VALOR DA DESPESA DEFLACIONADO
            Posição Inicial: 71
            Formato: N - Numérico
            Tamanho: 11 dígitos (11 bytes)
            Quantidade de Decimais: 2
Descrição Sumária: Ver descrição da variável 16 do REGISTRO TIPO 06 – DESPESA DE 90 DIAS.


18 – VALOR DA DESPESA ANUALIZADO E EXPANDIDO (FATOR 2)
            Posição Inicial: 82
            Formato: N - Numérico
            Tamanho: 16 dígitos (16 bytes)
            Quantidade de Decimais: 2
 		Descrição Sumária: Ver descrição da variável 17 do REGISTRO TIPO 06 – DESPESA DE 90 DIAS.


19 – CÓDIGO DE IMPUTAÇÃO
            Posição Inicial: 98
            Formato: N - Numérico
            Tamanho: 2 dígitos (2 bytes)
            Quantidade de Categorias: 4
            Classificação: 902
Descrição Sumária: Ver descrição da variável 16 do REGISTRO TIPO 05 – INVENTÁRIO DE BENS DURÁVEIS.


 20 - RENDA MONETÁRIA MENSAL DA UNIDADE DE CONSUMO
 Posição inicial: 100
 Formato: N - Numérico
 Tamanho: 16 dígitos (16 bytes)
 Quantidade de Decimais: 2
      Descrição Sumária: Ver descrição da variável 39 do REGISTRO TIPO 02 – PESSOAS.


 21 - RENDA NÃO MONETÁRIA MENSAL DA UNIDADE DE CONSUMO
 Posição inicial: 116
 Formato: N - Numérico
 Tamanho: 16 dígitos (16 bytes)
 Quantidade de Decimais: 2
      Descrição Sumária: Ver descrição da variável 40 do REGISTRO TIPO 02 – PESSOAS.


 22 - RENDA TOTAL MENSAL DA UNIDADE DE CONSUMO
 Posição inicial: 132
 Formato: N - Numérico
 Tamanho: 16 dígitos (16 bytes)
 Quantidade de Decimais: 2
 Descrição Sumária: Ver descrição da variável 41 do REGISTRO TIPO
02 – PESSOAS.


23 – MÉTODO DA QUANTIDADE ADQUIRIDA
 Posição inicial: 148
 Formato: N - Numérico
 Tamanho: 2 dígitos (2 bytes)
 Quantidade de categorias: 10
 Classificação: 601
Descrição Sumária: Indica o método pelo qual foi calculado a quantidade adquirida em KILOGRAMAS dos ALIMENTOS E BEBIDAS.
 Categorias:
 (-2) Não aplicável (itens que não se referem a alimentos e bebidas)
 (-1) Não houve tratamento (itens agregados)
           (0) Quando a quantidade adquirida em Kg foi calculada diretamente pelos valores informados nas variáveis quantidade, unidade de medida e peso ou volume.
           (1) Quando o peso não foi informado na coleta e a quantidade adquirida em Kg foi imputada pelo valor médio/ Kg do produto
(2) Quando a quantidade não foi informada na coleta e a quantidade adquirida em Kg foi imputada pelo valor médio/ Kg do produto
(3) Quando o peso e a quantidade não foram informados na coleta e a quantidade adquirida em Kg foi imputada pelo valor médio/ Kg do produto
(4) Quando o peso não foi informado na coleta e a quantidade adquirida em Kg foi imputada pela variável unidade de medida do produto.
(5) Quando a quantidade adquirida em Kg calculada foi considerada como outlier na cada inferior (menor que P05) e posteriormente foi imputada pelo valor médio/ Kg do produto
(6) Quando a quantidade adquirida em Kg calculada foi considerada como outlier na cada superior (maior que P95) e posteriormente foi imputada pelo valor médio/ Kg do produto
(7) Quando a quantidade adquirida em kg não pode ser imputada pelo valor médio/KG do produto devido a inexistência de parâmetros. Neste caso, a mesma foi imputada pelo valor médio/kg do sub grupo agregado.


24 – QUANTIDADE ADQUIRIDA
 Posição inicial: 150
 Formato: N - Numérico
 Tamanho: 8 dígitos (8 bytes)
 Quantidade de Decimais: 3
 		Descrição Sumária: Indica a quantidade adquirida em KILOGRAMAS dos ALIMENTOS E BEBIDAS, após os procedimentos de transformação das             variáveis quantidade, unidade de medida e peso ou volume.


REGISTRO TIPO 12: DESPESA INDIVIDUAL – POF4 / QUADROS 22 A 50
```

#### **Tamanho: 147**

```
1 - TIPO DE REGISTRO (Item de Identificação do Dicionário)            Posição Inicial: 1
            Formato: N - Numérico
            Tamanho: 2 dígitos (2 bytes)
 		Descrição Sumária: Ver descrição da variável 1 do REGISTRO TIPO 01 – DOMICÍLIO.


  		2 – CÓDIGO DA UNIDADE DA FEDERAÇÃO
            Posição Inicial: 3
            Formato: N - Numérico
            Tamanho: 2 dígitos (2 bytes)
 		Quantidade de Categorias: 27
 		Classificação: 900
 		Descrição Sumária: Ver descrição da variável 2 do REGISTRO TIPO 01 – DOMICÍLIO.


  		3 – NÚMERO SEQUENCIAL
            Posição Inicial: 5
            Formato: N - Numérico
            Tamanho: 3 dígitos (3 bytes)
 		Descrição Sumária:
 		Descrição Sumária: Ver descrição da variável 3 do REGISTRO TIPO 01 – DOMICÍLIO.


  		4 – DV DO SEQUENCIAL
            Posição Inicial: 8
            Formato: N - Numérico
            Tamanho: 1 dígito (1 byte)
 		Descrição Sumária: Ver descrição da variável 4 do REGISTRO TIPO 01 – DOMICÍLIO.


  		5 – NÚMERO DO DOMICÍLIO
            Posição Inicial: 9
            Formato: N - Numérico
            Tamanho: 2 dígitos (2 bytes)
Descrição Sumária: Ver descrição da variável 5 do REGISTRO TIPO
01 – DOMICÍLIO.


  		6 - NÚMERO DA UC
             Posição inicial: 11
             Formato: N - Numérico
             Tamanho: 1 dígito (1 byte)
 		Descrição Sumária: Ver descrição da variável 6 do REGISTRO TIPO
02 – PESSOAS.


7 - NÚMERO DO INFORMANTE
             Posição inicial: 12
             Formato: N - Numérico
             Tamanho: 2 dígitos (2 bytes)
 		Descrição Sumária: Ver descrição da variável 7 do REGISTRO TIPO
02 – PESSOAS.


8 - ESTRATO GEOGRÁFICO
            Posição Inicial: 14
            Formato: N - Numérico
            Tamanho: 2 dígitos (2 bytes)
Quantidade de Categorias: 51
Classificação: 901
Descrição Sumária: Ver descrição da variável 6 do REGISTRO TIPO 01 – DOMICÍLIO.


  		9 – FATOR DE EXPANSÃO 1 (DESENHO AMOSTRAL)
            Posição Inicial: 16
            Formato: N - Numérico
            Tamanho: 14 dígitos (14 bytes)
            Quantidade de Decimais: 8
Descrição Sumária: Ver descrição da variável 7 do REGISTRO TIPO 01 – DOMICÍLIO.


  		10 – FATOR DE EXPANSÃO 2 (AJUSTADO P/ ESTIMATIVAS)
            Posição Inicial: 30
            Formato: N - Numérico
            Tamanho: 14 dígitos (14 bytes)
            Quantidade de Decimais: 8
Descrição Sumária: Ver descrição da variável 8 do REGISTRO TIPO 01 – DOMICÍLIO.


11 – NÚMERO DO QUADRO
            Posição Inicial: 44
            Formato: N - Numérico
            Tamanho: 2 dígitos (2 bytes)
 		Quantidade de Categorias: 56
Classificação: 906
Descrição Sumária: Identifica o número do quadro do Questionário de Aquisição Individual (POF 4) no qual o item de despesa foi registrado.
Neste registro, o número do quadro varia de 22 a 50.


   		12 – CÓDIGO DO ITEM
            Posição Inicial: 46
            Formato: N - Numérico
            Tamanho: 5 dígitos (5 bytes)
 		Quantidade de Categorias: 13476
            Classificação: 907
 		Descrição Sumária: Ver descrição da variável 11 do REGISTRO TIPO 06 – DESPESA DE 90 DIAS.


13 – FORMA DE AQUISIÇÃO
            Posição Inicial: 51
            Formato: N - Numérico
            Tamanho: 2 dígitos (2 bytes)
            Quantidade de Categorias: 12
 		Classificação: 903
 		Descrição Sumária: Ver descrição da variável 12 do REGISTRO TIPO 06 – DESPESA DE 90 DIAS.


14 – VALOR DA DESPESA / AQUISIÇÃO
            Posição Inicial: 53
            Formato: N - Numérico
            Tamanho: 11 dígitos (11 bytes)
            Quantidade de Decimais: 2
		Descrição Sumária: Ver descrição da variável 13 do REGISTRO TIPO 06 – DESPESA DE 90 DIAS.


   		15 - FATOR DE ANUALIZAÇÃO
            Posição Inicial: 64
            Formato: N - Numérico
            Tamanho: 2 dígitos (2 bytes)
		Descrição Sumária: Ver descrição da variável 14 do REGISTRO TIPO 06 – DESPESA DE 90 DIAS.


16 – DEFLATOR FATOR
            Posição Inicial: 66
            Formato: N - Numérico
            Tamanho: 5 dígitos (5 bytes)
            Quantidade de Decimais: 2
Descrição Sumária: Ver descrição da variável 15 do REGISTRO TIPO 06 – DESPESA DE 90 DIAS.


17 – VALOR DA DESPESA DEFLACIONADO
            Posição Inicial: 71
            Formato: N - Numérico
            Tamanho: 11 dígitos (11 bytes)
            Quantidade de Decimais: 2
Descrição Sumária: Ver descrição da variável 16 do REGISTRO TIPO 06 – DESPESA DE 90 DIAS.


18 – VALOR DA DESPESA ANUALIZADO E EXPANDIDO (FATOR 2)
            Posição Inicial: 82
            Formato: N - Numérico
            Tamanho: 16 dígitos (16 bytes)
            Quantidade de Decimais: 2
 		Descrição Sumária: Ver descrição da variável 17 do REGISTRO TIPO 06 – DESPESA DE 90 DIAS.


19 – CÓDIGO DE IMPUTAÇÃO
            Posição Inicial: 98
            Formato: N - Numérico
            Tamanho: 2 dígitos (2 bytes)
            Quantidade de Categorias: 4
            Classificação: 902
Descrição Sumária: Ver descrição da variável 16 do REGISTRO TIPO 05 – INVENTÁRIO DE BENS DURÁVEIS.


 20 - RENDA MONETÁRIA MENSAL DA UNIDADE DE CONSUMO
 Posição inicial: 100
 Formato: N - Numérico
 Tamanho: 16 dígitos (16 bytes)
 Quantidade de Decimais: 2
      Descrição Sumária: Ver descrição da variável 39 do REGISTRO TIPO 02 – PESSOAS.


 21 - RENDA NÃO MONETÁRIA MENSAL DA UNIDADE DE CONSUMO
 Posição inicial: 116
 Formato: N - Numérico
 Tamanho: 16 dígitos (16 bytes)
 Quantidade de Decimais: 2
      Descrição Sumária: Ver descrição da variável 40 do REGISTRO TIPO 02 – PESSOAS.


 22 - RENDA TOTAL MENSAL DA UNIDADE DE CONSUMO
 Posição inicial: 132
 Formato: N - Numérico
 Tamanho: 16 dígitos (16 bytes)
 Quantidade de Decimais: 2
 Descrição Sumária: Ver descrição da variável 41 do REGISTRO TIPO
02 – PESSOAS.


REGISTRO TIPO 13: DESPESA COM VEÍCULOS – POF4 / QUADRO 51
```

#### **Tamanho: 148**

```
1 - TIPO DE REGISTRO (Item de Identificação do Dicionário)            Posição Inicial: 1
            Formato: N - Numérico
            Tamanho: 2 dígitos (2 bytes)
 		Descrição Sumária: Ver descrição da variável 1 do REGISTRO TIPO 01 – DOMICÍLIO.


  		2 – CÓDIGO DA UNIDADE DA FEDERAÇÃO
 		Posição Inicial: 3
            Formato: N - Numérico
            Tamanho: 2 dígitos (2 bytes)
 		Quantidade de Categorias: 27
 		Classificação: 900
 		Descrição Sumária: Ver descrição da variável 2 do REGISTRO TIPO 01 – DOMICÍLIO.


  		3 – NÚMERO SEQUENCIAL
            Posição Inicial: 5
            Formato: N - Numérico
            Tamanho: 3 dígitos (3 bytes)
 		Descrição Sumária: Ver descrição da variável 3 do REGISTRO TIPO 01 – DOMICÍLIO.


  		4 – DV DO SEQUENCIAL
            Posição Inicial: 8
            Formato: N - Numérico
            Tamanho: 1 dígito (1 byte)
 		Descrição Sumária: Ver descrição da variável 4 do REGISTRO TIPO 01 – DOMICÍLIO.


  		5 – NÚMERO DO DOMICÍLIO
            Posição Inicial: 9
            Formato: N - Numérico
            Tamanho: 2 dígitos (2 bytes)
Descrição Sumária: Ver descrição da variável 5 do REGISTRO TIPO
01 – DOMICÍLIO.


  		6 - NÚMERO DA UC
             Posição inicial: 11
             Formato: N - Numérico
             Tamanho: 1 dígito (1 byte)
 		Descrição Sumária: Ver descrição da variável 6 do REGISTRO TIPO
02 – PESSOAS.


7 - NÚMERO DO INFORMANTE
             Posição inicial: 12
             Formato: N - Numérico
             Tamanho: 2 dígitos (2 bytes)
 		Descrição Sumária: Ver descrição da variável 7 do REGISTRO TIPO
02 – PESSOAS.


8 - ESTRATO GEOGRÁFICO
            Posição Inicial: 14
            Formato: N - Numérico
            Tamanho: 2 dígitos (2 bytes)
Quantidade de Categorias: 51
Classificação: 901
Descrição Sumária: Ver descrição da variável 6 do REGISTRO TIPO 01 – DOMICÍLIO.


  		9 – FATOR DE EXPANSÃO 1 (DESENHO AMOSTRAL)
            Posição Inicial: 16
            Formato: N - Numérico
            Tamanho: 14 dígitos (14 bytes)
            Quantidade de Decimais: 8
Descrição Sumária: Ver descrição da variável 7 do REGISTRO TIPO 01 – DOMICÍLIO.


  		10 – FATOR DE EXPANSÃO 2 (AJUSTADO P/ ESTIMATIVAS)
            Posição Inicial: 30
            Formato: N - Numérico
            Tamanho: 14 dígitos (14 bytes)
            Quantidade de Decimais: 8
Descrição Sumária: Ver descrição da variável 8 do REGISTRO TIPO 01 – DOMICÍLIO.


11 – NÚMERO DO QUADRO
            Posição Inicial: 44
            Formato: N - Numérico
            Tamanho: 2 dígitos (2 bytes)
 		Quantidade de Categorias: 56
Classificação: 906
Descrição Sumária: Ver descrição da variável 11 do REGISTRO TIPO 12 – DESPESA INDIVIDUAL.
Neste registro, o número do quadro é sempre 51.


   		12 – CÓDIGO DO ITEM
            Posição Inicial: 46
            Formato: N - Numérico
            Tamanho: 5 dígitos (5 bytes)
 		Quantidade de Categorias: 13476
            Classificação: 907
 		Descrição Sumária: Ver descrição da variável 11 do REGISTRO TIPO 06 – DESPESA DE 90 DIAS.


13 – FORMA DE AQUISIÇÃO
            Posição Inicial: 51
            Formato: N - Numérico
            Tamanho: 2 dígitos (2 bytes)
            Quantidade de Categorias: 12
 		Classificação: 903
 		Descrição Sumária: Ver descrição da variável 12 do REGISTRO TIPO 06 – DESPESA DE 90 DIAS.


14 – VALOR DA DESPESA / AQUISIÇÃO
            Posição Inicial: 53
            Formato: N - Numérico
            Tamanho: 11 dígitos (11 bytes)
            Quantidade de Decimais: 2
		Descrição Sumária: Ver descrição da variável 13 do REGISTRO TIPO 06 – DESPESA DE 90 DIAS.


15 – ESTADO DE AQUISIÇÃO
            Posição Inicial: 64
            Formato: N - Numérico
            Tamanho: 1 dígito (1 byte)
            Quantidade de Categorias: 3
 		Classificação: 905
Descrição Sumária: Identifica o estado do produto adquirido pelo informante no período de referencia da pesquisa. Categorias: (1) Novo, (3) Usado e 9 (Não Sabe).


   		16 - FATOR DE ANUALIZAÇÃO
            Posição Inicial: 65
            Formato: N - Numérico
            Tamanho: 2 dígitos (2 bytes)
		Descrição Sumária: Ver descrição da variável 14 do REGISTRO TIPO 06 – DESPESA DE 90 DIAS.


17 – DEFLATOR FATOR
            Posição Inicial: 67
            Formato: N - Numérico
            Tamanho: 5 dígitos (5 bytes)
            Quantidade de Decimais: 2
Descrição Sumária: Ver descrição da variável 15 do REGISTRO TIPO 06 – DESPESA DE 90 DIAS.


18 – VALOR DA DESPESA DEFLACIONADO
            Posição Inicial: 72
            Formato: N - Numérico
            Tamanho: 11 dígitos (11 bytes)
            Quantidade de Decimais: 2
Descrição Sumária: Ver descrição da variável 16 do REGISTRO TIPO 06 – DESPESA DE 90 DIAS.


19 – VALOR DA DESPESA ANUALIZADO E EXPANDIDO (FATOR 2)
            Posição Inicial: 83
            Formato: N - Numérico
            Tamanho: 16 dígitos (16 bytes)
            Quantidade de Decimais: 2
 		Descrição Sumária: Ver descrição da variável 17 do REGISTRO TIPO 06 – DESPESA DE 90 DIAS.


20 – CÓDIGO DE IMPUTAÇÃO
            Posição Inicial: 99
            Formato: N - Numérico
            Tamanho: 2 dígitos (2 bytes)
            Quantidade de Categorias: 4
            Classificação: 902
Descrição Sumária: Ver descrição da variável 16 do REGISTRO TIPO 05 – INVENTÁRIO DE BENS DURÁVEIS.


 21 - RENDA MONETÁRIA MENSAL DA UNIDADE DE CONSUMO
 Posição inicial: 101
 Formato: N - Numérico
 Tamanho: 16 dígitos (16 bytes)
 Quantidade de Decimais: 2
      Descrição Sumária: Ver descrição da variável 39 do REGISTRO TIPO 02 – PESSOAS.


 22 - RENDA NÃO MONETÁRIA MENSAL DA UNIDADE DE CONSUMO
 Posição inicial: 117
 Formato: N - Numérico
 Tamanho: 16 dígitos (16 bytes)
 Quantidade de Decimais: 2
      Descrição Sumária: Ver descrição da variável 40 do REGISTRO TIPO 02 – PESSOAS.


 23 - RENDA TOTAL MENSAL DA UNIDADE DE CONSUMO
 Posição inicial: 133
 Formato: N - Numérico
 Tamanho: 16 dígitos (16 bytes)
 Quantidade de Decimais: 2
 Descrição Sumária: Ver descrição da variável 41 do REGISTRO TIPO
02 – PESSOAS.


REGISTRO TIPO 14: RENDIMENTOS E DEDUÇÕES – POF5 / QUADRO 53
```

#### **Tamanho: 283**

```
1 - TIPO DE REGISTRO (Item de Identificação do Dicionário)
            Posição Inicial: 1
            Formato: N - Numérico
            Tamanho: 2 dígitos (2 bytes)
 		Descrição Sumária: Ver descrição da variável 1 do REGISTRO TIPO 01 – DOMICÍLIO.


  		2 – CÓDIGO DA UNIDADE DA FEDERAÇÃO
            Posição Inicial: 3
            Formato: N - Numérico
            Tamanho: 2 dígitos (2 bytes)
 		Quantidade de Categorias: 27
 		Classificação: 900
 		Descrição Sumária: Ver descrição da variável 2 do REGISTRO TIPO 01 – DOMICÍLIO.


  		3 – NÚMERO SEQUENCIAL
            Posição Inicial: 5
            Formato: N - Numérico
            Tamanho: 3 dígitos (3 bytes)
 		Descrição Sumária: Ver descrição da variável 3 do REGISTRO TIPO 01 – DOMICÍLIO.


  		4 – DV DO SEQUENCIAL
            Posição Inicial: 8
            Formato: N - Numérico
            Tamanho: 1 dígito (1 byte)
 		Descrição Sumária: Ver descrição da variável 4 do REGISTRO TIPO 01 – DOMICÍLIO.


  		5 – NÚMERO DO DOMICÍLIO
            Posição Inicial: 9
            Formato: N - Numérico
            Tamanho: 2 dígitos (2 bytes)
Descrição Sumária: Ver descrição da variável 5 do REGISTRO TIPO
01 – DOMICÍLIO.


  		6 - NÚMERO DA UC
             Posição inicial: 11
             Formato: N - Numérico
             Tamanho: 1 dígito (1 byte)
 		Descrição Sumária: Ver descrição da variável 6 do REGISTRO TIPO
02 – PESSOAS.


7 - NÚMERO DO INFORMANTE
             Posição inicial: 12
             Formato: N - Numérico
             Tamanho: 2 dígitos (2 bytes)
 		Descrição Sumária: Ver descrição da variável 7 do REGISTRO TIPO
02 – PESSOAS.


8 - ESTRATO GEOGRÁFICO
            Posição Inicial: 14
            Formato: N - Numérico
            Tamanho: 2 dígitos (2 bytes)
Quantidade de Categorias: 51
Classificação: 901
Descrição Sumária: Ver descrição da variável 6 do REGISTRO TIPO 01 – DOMICÍLIO.


  		9 – FATOR DE EXPANSÃO 1 (DESENHO AMOSTRAL)
            Posição Inicial: 16
            Formato: N - Numérico
            Tamanho: 14 dígitos (14 bytes)
            Quantidade de Decimais: 8
Descrição Sumária: Ver descrição da variável 7 do REGISTRO TIPO 01 – DOMICÍLIO.


  		10 – FATOR DE EXPANSÃO 2 (AJUSTADO P/ ESTIMATIVAS)
            Posição Inicial: 30
            Formato: N - Numérico
            Tamanho: 14 dígitos (14 bytes)
            Quantidade de Decimais: 8
Descrição Sumária: Ver descrição da variável 8 do REGISTRO TIPO 01 – DOMICÍLIO.

11 – NÚMERO DO QUADRO
            Posição Inicial: 44
            Formato: N - Numérico
            Tamanho: 2 dígitos (2 bytes)
 		Quantidade de Categorias: 56
Classificação: 906
Descrição Sumária: Identifica o número do quadro do Questionário de Trabalho e Rendimento Individual (POF 5) no qual o item de rendimento foi registrado.
Neste registro, o número do quadro é sempre 53.

12 – TIPO DE TRABALHO
            Posição Inicial: 46
            Formato: N - Numérico
            Tamanho: 1 dígito (1 byte)
            Quantidade de Categorias: 2
            Classificação: 700
            Descrição Sumária: Identifica o tipo de trabalho que o informante exerceu no período de referência da pesquisa. Categorias: (1) Principal e (2) Secundário.
A definição de trabalho principal deve levar em consideração um conjunto de critérios definidos a seguir.

a) Para a pessoa que trabalhou em mais de um trabalho durante o período de referência (últimos 12 meses), mas no momento da pesquisa tem apenas 1 trabalho, considera-se o trabalho atual como trabalho principal.

b) Para a pessoa que trabalhou em mais de um trabalho durante o período de referência (últimos 12 meses), mas no momento da pesquisa não possui nenhum trabalho, considera-se o último trabalho  como trabalho principal.

c) Para a pessoa classificada como tendo mais de um trabalho, ou seja, para a que trabalhava em mais de um empreendimento simultaneamente, adote os seguintes critérios respectivos, na ordem enumerada, sem fazer distinção entre trabalho remunerado e sem remuneração, para definir o principal:
```

1. O trabalho em que a pessoa **trabalhou o maior número de horas no último mês**.
2. Em caso de igualdade de horas trabalhadas no último mês, o principal deve ser aquele em que a pessoa **tinha mais tempo de permanência** no empreendimento.
3. Havendo igualdade no tempo que trabalhou nos empreendimentos, o principal deve ser aquele que proporcionava **maior rendimento mensal**.

```
            13 – POSIÇÃO NA OCUPAÇÃO
               Posição Inicial: 47
            Formato: N - Numérico
            Tamanho: 2 dígitos (2 bytes)
 		Quantidade de Categorias: 9
            Classificação: 701
            Descrição Sumária: Identifica a posição na ocupação da pessoa no trabalho informado. Categorias: (01) Empregado Privado, (02) Empregado Público, (03) Empregado Doméstico, (04) Empregado Temporário na Área Rural, (05) Empregador, (06) Conta-Própria, (07) Aprendiz ou Estagiário, (08) Não-Remunerado em Ajuda a Membro do Domicílio e (09) Trabalhador na Produção para o Próprio Consumo.


   		14 – FORMA DO ÚLTIMO RENDIMENTO
               Posição Inicial: 49
            Formato: N - Numérico
            Tamanho: 1 dígito (1 byte)
            Quantidade de Categorias: 4
            Classificação: 702
            Descrição Sumária: Identifica a forma do último rendimento bruto mensal recebido. Categorias: (0) Não tem, (1) Em dinheiro, (2) Em dinheiro e benefícios e (3) Somente benefícios


            15 – CÓDIGO DO ITEM
            Posição Inicial: 50
            Formato: N - Numérico
            Tamanho: 5 dígitos (5 bytes)
 		Quantidade de Categorias: 13476
            Classificação: 907
 		Descrição Sumária: Identifica o tipo de rendimento que é correspondente a variável posição na ocupação. Esta variável deverá ser lida juntamente com a variável “Número do Quadro”, que irá compor o código do produto completo com 7 dígitos.


   		16 – VALOR DO ÚLTIMO RENDIMENTO
            Posição Inicial: 55
            Formato: N – Numérico
            Tamanho: 11 dígitos (11 bytes)
            Quantidade de Decimais: 2
Descrição Sumária: Identifica o valor em reais (R$) do último rendimento bruto mensal recebido pela pessoa no período de referência da pesquisa. Nos casos de valores ignorados, este campo está preenchido com “Noves”. Nos casos em que não ocorreu rendimento, este campo esta preenchido com “Zeros”. O valor também é zero para os casos onde não se aplica.


17 – MÊS DO ÚLTIMO RENDIMENTO
            Posição Inicial: 66
            Formato: N – Numérico
            Tamanho: 2 dígitos (2 bytes)
            Quantidade de Categorias = 13
            Classificação: 904
Descrição Sumária: Identifica o mês último rendimento bruto mensal recebido pela pessoa no período de referência da pesquisa. Categorias: (00) Não se aplica, (01) Janeiro, (02) Fevereiro, (03) Março, (04) Abril, (05) Maio, (06) Junho, (07) Julho, (08) Agosto, (09) Setembro, (10) Outubro, (11) Novembro e (12) Dezembro.
Neste registro, esta variável é exclusiva para os itens do quadro 53.


            18 – NÚMERO DE MESES RECEBIDOS
            Posição Inicial: 68
            Formato: N – Numérico
            Tamanho: 2 dígitos (2 bytes)
Descrição Sumária: Identifica o número de meses que o rendimento bruto foi recebido pela pessoa no período de referência da pesquisa. O valor desta variável é “Zero” para os casos onde não se aplica.


19 – EXISTÊNCIA DE DEDUÇÃO
            Posição Inicial: 70
            Formato: N – Numérico
            Tamanho: 1 dígito (1 byte)
            Quantidade de Categorias: 2
            Classificação: 703
            Descrição Sumária: Identifica se houve ocorrência de dedução sobre o valor do último rendimento bruto mensal recebido pela pessoa no período de referência da pesquisa. Categorias: (0) Não se aplica, (1) Sim e (2) Não.

20 – CÓDIGO DE PREVIDÊNCIA PÚBLICA
            Posição Inicial: 71
            Formato: N - Numérico
            Tamanho: 5 dígitos (5 bytes)
            Quantidade de Categorias: 9
            Classificação: 704
 		Descrição Sumária: Identifica o tipo de dedução com previdência pública que incidiu sobre o rendimento. Esta variável deverá ser lida juntamente com a variável “Número do Quadro”, que irá compor o código do produto completo com 7 dígitos.


21 - VALOR DA PREVIDÊNCIA PÚBLICA
            Posição Inicial: 76
            Formato: N – Numérico
            Tamanho: 11 dígitos (11 bytes)
            Quantidade de Decimais: 2
            Descrição Sumária: Identifica o valor em reais (R$) da dedução com Previdência Pública que incidiu sobre o valor do último rendimento bruto mensal recebido pela pessoa no período de referência da pesquisa. Nos casos de valores ignorados, este campo está preenchido com “Noves”. Nos casos em que não ocorreu dedução, este campo esta preenchido com “Zeros”. O valor também é zero para os casos onde não se aplica.


22 – CÓDIGO DE IMPOSTO DE RENDA
            Posição Inicial: 87
            Formato: N - Numérico
            Tamanho: 5 dígitos (5 bytes)
            Quantidade de Categorias: 9
            Classificação: 705
 		Descrição Sumária: Identifica o tipo de dedução com imposto de renda que incidiu sobre o rendimento. Esta variável deverá ser lida juntamente com a variável “Número do Quadro”, que irá compor o código do produto completo com 7 dígitos.


23 - VALOR DO IMPOSTO DE RENDA
            Posição Inicial: 92
            Formato: N – Numérico
            Tamanho: 11 dígitos (11 bytes)
            Quantidade de Decimais: 2
Descrição Sumária: Identifica o valor em reais (R$) da dedução com Imposto de Renda que incidiu sobre o valor do último rendimento bruto mensal recebido pela pessoa no período de referência da pesquisa. Nos casos de valores ignorados, este campo está preenchido com “Noves”. Nos casos em que não ocorreu dedução, este campo esta preenchido com “Zeros”. O valor também é zero para os casos onde não se aplica.


24 – CÓDIGO DE OUTRAS DEDUÇÕES
            Posição Inicial: 103
            Formato: N - Numérico
            Tamanho: 5 dígitos (5 bytes)
            Quantidade de Categorias: 9
            Classificação: 706
 		Descrição Sumária: Identifica o tipo de outras deduções que incidiram sobre o rendimento. Esta variável deverá ser lida juntamente com a variável “Número do Quadro”, que irá compor o código do produto completo com 7 dígitos.


25 - VALOR DE OUTRAS DEDUÇÕES
            Posição Inicial: 108
            Formato: N – Numérico
            Tamanho: 11 dígitos (11 bytes)
            Quantidade de Decimais: 2
Descrição Sumária: Identifica o valor em reais (R$) de outras deduções que incidiram sobre o valor do último rendimento bruto mensal recebido pela pessoa no período de referência da pesquisa. Nos casos de valores ignorados, este campo está preenchido com “Noves”. Nos casos em que não ocorreu dedução, este campo esta preenchido com “Zeros”. O valor também é zero para os casos onde não se aplica.


26 - FATOR DE ANUALIZAÇÃO
            Posição Inicial: 119
            Formato: N - Numérico
            Tamanho: 2 dígitos (2 bytes)
		Descrição Sumária: Ver descrição da variável 14 do REGISTRO TIPO 06 – DESPESA DE 90 DIAS.


27 – DEFLATOR FATOR
            Posição Inicial: 121
            Formato: N - Numérico
            Tamanho: 5 dígitos (5 bytes)
            Quantidade de Decimais: 2
Descrição Sumária: Ver descrição da variável 15 do REGISTRO TIPO 06 – DESPESA DE 90 DIAS.


28 – VALOR DO ÚLTIMO RENDIMENTO DEFLACIONADO
            Posição Inicial: 126
            Formato: N - Numérico
            Tamanho: 11 dígitos (11 bytes)
            Quantidade de Decimais: 2
Descrição Sumária: Identifica o valor resultante da operação “VALOR DO ÚLTIMO RENDIMENTO” multiplicado pelo “DEFLATOR FATOR”.


29 – VALOR DA PREVIDÊNCIA PÚBLICA DEFLACIONADO
            Posição Inicial: 137
            Formato: N - Numérico
            Tamanho: 11 dígitos (11 bytes)
            Quantidade de Decimais: 2
Descrição Sumária: Identifica o valor resultante da operação “VALOR DA PREVIDÊNCIA PÚBLICA” multiplicado pelo “DEFLATOR FATOR”.


30 – VALOR DO IMPOSTO DE RENDA DEFLACIONADO
            Posição Inicial: 148
            Formato: N - Numérico
            Tamanho: 11 dígitos (11 bytes)
            Quantidade de Decimais: 2
Descrição Sumária: Identifica o valor resultante da operação “VALOR DO IMPOSTO DE RENDA” multiplicado pelo “DEFLATOR FATOR”.


31 – VALOR DE OUTRAS DEDUÇÕES DEFLACIONADO
            Posição Inicial: 159
            Formato: N - Numérico
            Tamanho: 11 dígitos (11 bytes)
            Quantidade de Decimais: 2
Descrição Sumária: Identifica o valor resultante da operação “VALOR DE OUTRAS DEDUÇÕES” multiplicado pelo “DEFLATOR FATOR”.


32 – VALOR DO ÚLTIMO RENDIMENTO ANUALIZADO E EXPANDIDO(FATOR 2)
            Posição Inicial: 170
            Formato: N - Numérico
            Tamanho: 16 dígitos (16 bytes)
            Quantidade de Decimais: 2
 		Descrição Sumária: Identifica o valor resultante da operação do “VALOR DO ÚLTIMO RENDIMENTO DEFLACIONADO pelo “NÚMERO DE MESES RECEBIDOS”, pelo “FATOR DE ANUALIZAÇÃO” e pelo FATOR DE EXPANSÃO 2 (AJUSTADO P/ ESTIMATIVAS).


33 – VALOR DA PREV. PÚBLICA ANUALIZADO E EXPANDIDO (FATOR 2)
            Posição Inicial: 186
            Formato: N - Numérico
            Tamanho: 16 dígitos (16 bytes)
            Quantidade de Decimais: 2
 		Descrição Sumária: Identifica o valor resultante da operação do “VALOR DA PREVIDÊNCIA PÚBLICA DEFLACIONADO multiplicado pelo “NÚMERO DE MESES RECEBIDOS”, pelo “FATOR DE ANUALIZAÇÃO” e pelo FATOR DE EXPANSÃO 2 (AJUSTADO P/ ESTIMATIVAS).


34 – VALOR DO IMPOSTO DE RENDA ANUALIZADO E EXPANDIDO (FATOR 2)
            Posição Inicial: 202
            Formato: N - Numérico
            Tamanho: 16 dígitos (16 bytes)
            Quantidade de Decimais: 2
 		Descrição Sumária: Identifica o valor resultante da operação do “VALOR DO IMPOSTO DE RENDA DEFLACIONADO” multiplicado pelo “NÚMERO DE MESES RECEBIDOS”, pelo “FATOR DE ANUALIZAÇÃO” e pelo FATOR DE EXPANSÃO 2 (AJUSTADO P/ ESTIMATIVAS).


35 – VALOR DE OUTRAS DEDUÇÕES ANUALIZADO E EXPANDIDO (FATOR 2)
            Posição Inicial: 218
            Formato: N - Numérico
            Tamanho: 16 dígitos (16 bytes)
            Quantidade de Decimais: 2
 		Descrição Sumária: Identifica o valor resultante da operação do “VALOR DE OUTRAS DEDUÇÕES DEFLACIONADO” multiplicado pelo “NÚMERO DE MESES RECEBIDOS”, pelo “FATOR DE ANUALIZAÇÃO” e pelo FATOR DE EXPANSÃO 2 (AJUSTADO P/ ESTIMATIVAS).


36 – CÓDIGO DE IMPUTAÇÃO
            Posição Inicial: 234
            Formato: N - Numérico
            Tamanho: 2 dígitos (2 bytes)
            Quantidade de Categorias: 4
            Classificação: 902
Descrição Sumária: Ver descrição da variável 16 do REGISTRO TIPO 05 – INVENTÁRIO DE BENS DURÁVEIS.


 37 - RENDA MONETÁRIA MENSAL DA UNIDADE DE CONSUMO
 Posição inicial: 236
 Formato: N - Numérico
 Tamanho: 16 dígitos (16 bytes)
 Quantidade de Decimais: 2
      Descrição Sumária: Ver descrição da variável 39 do REGISTRO TIPO 02 – PESSOAS.


 38 - RENDA NÃO MONETÁRIA MENSAL DA UNIDADE DE CONSUMO
 Posição inicial: 252
 Formato: N - Numérico
 Tamanho: 16 dígitos (16 bytes)
 Quantidade de Decimais: 2
      Descrição Sumária: Ver descrição da variável 40 do REGISTRO TIPO 02 – PESSOAS.


 39 - RENDA TOTAL MENSAL DA UNIDADE DE CONSUMO
 Posição inicial: 268
 Formato: N - Numérico
 Tamanho: 16 dígitos (16 bytes)
 Quantidade de Decimais: 2
 Descrição Sumária: Ver descrição da variável 41 do REGISTRO TIPO
02 – PESSOAS.


REGISTRO TIPO 15: OUTROS RENDIMENTOS – POF5 / QUADROS 54 A 57
```

#### **Tamanho: 192**

```
1 - TIPO DE REGISTRO (Item de Identificação do Dicionário)            Posição Inicial: 1
            Formato: N - Numérico
            Tamanho: 2 dígitos (2 bytes)
 		Descrição Sumária: Ver descrição da variável 1 do REGISTRO TIPO 01 – DOMICÍLIO.


  		2 – CÓDIGO DA UNIDADE DA FEDERAÇÃO
 		Posição Inicial: 3
            Formato: N - Numérico
            Tamanho: 2 dígitos (2 bytes)
 		Quantidade de Categorias: 27
 		Classificação: 900
 		Descrição Sumária: Ver descrição da variável 2 do REGISTRO TIPO 01 – DOMICÍLIO.


  		3 – NÚMERO SEQUENCIAL
            Posição Inicial: 5
            Formato: N - Numérico
            Tamanho: 3 dígitos (3 bytes)
 		Descrição Sumária: Ver descrição da variável 3 do REGISTRO TIPO 01 – DOMICÍLIO.


  		4 – DV DO SEQUENCIAL
            Posição Inicial: 8
            Formato: N - Numérico
            Tamanho: 1 dígito (1 byte)
 		Descrição Sumária: Ver descrição da variável 4 do REGISTRO TIPO 01 – DOMICÍLIO.


  		5 – NÚMERO DO DOMICÍLIO
            Posição Inicial: 9
            Formato: N - Numérico
            Tamanho: 2 dígitos (2 bytes)
Descrição Sumária: Ver descrição da variável 5 do REGISTRO TIPO
01 – DOMICÍLIO.


  		6 - NÚMERO DA UC
             Posição inicial: 11
             Formato: N - Numérico
             Tamanho: 1 dígito (1 byte)
 		Descrição Sumária: Ver descrição da variável 6 do REGISTRO TIPO
02 – PESSOAS.


7 - NÚMERO DO INFORMANTE
             Posição inicial: 12
             Formato: N - Numérico
             Tamanho: 2 dígitos (2 bytes)
 		Descrição Sumária: Ver descrição da variável 7 do REGISTRO TIPO
02 – PESSOAS.


8 - ESTRATO GEOGRÁFICO
            Posição Inicial: 14
            Formato: N - Numérico
            Tamanho: 2 dígitos (2 bytes)
Quantidade de Categorias: 51
Classificação: 901
Descrição Sumária: Ver descrição da variável 6 do REGISTRO TIPO 01 – DOMICÍLIO.


  		9 – FATOR DE EXPANSÃO 1 (DESENHO AMOSTRAL)
            Posição Inicial: 16
            Formato: N - Numérico
            Tamanho: 14 dígitos (14 bytes)
            Quantidade de Decimais: 8
Descrição Sumária: Ver descrição da variável 7 do REGISTRO TIPO 01 – DOMICÍLIO.


  		10 – FATOR DE EXPANSÃO 2 (AJUSTADO P/ ESTIMATIVAS)
            Posição Inicial: 30
            Formato: N - Numérico
            Tamanho: 14 dígitos (14 bytes)
            Quantidade de Decimais: 8
Descrição Sumária: Ver descrição da variável 8 do REGISTRO TIPO 01 – DOMICÍLIO.


11 – NÚMERO DO QUADRO
            Posição Inicial: 44
            Formato: N - Numérico
            Tamanho: 2 dígitos (2 bytes)
 		Quantidade de Categorias: 56
Classificação: 906
Descrição Sumária: Ver descrição da variável 11 do REGISTRO TIPO 14 – RENDIMENTOS E DEDUÇÕES.
Neste registro, o número do quadro varia de 54 a 57.


   		12 – CÓDIGO DO ITEM
            Posição Inicial: 46
            Formato: N - Numérico
            Tamanho: 5 dígitos (5 bytes)
 		Quantidade de Categorias: 13476
            Classificação: 907
 		Descrição Sumária: Identifica o tipo de rendimento. Esta variável deverá ser lida juntamente com a variável “Número do Quadro”, que irá compor o código do produto completo com 7 dígitos.


13 – VALOR DO RENDIMENTO
            Posição Inicial: 51
            Formato: N - Numérico
            Tamanho: 11 dígitos (11 bytes)
            Quantidade de Decimais: 2
		Descrição Sumária: Identifica o valor em reais (R$), do rendimento bruto recebido pela pessoa no período de referência do respectivo quadro. Nos casos de valores ignorados, este campo está preenchido com “Noves”.
Para os itens do quadro 54, o valor registrado corresponde ao último rendimento mensal recebido dentro do período de referência de 12 meses.


   		14 – VALOR DA DEDUÇÃO
            Posição Inicial: 62
            Formato: N - Numérico
            Tamanho: 11 dígitos (11 bytes)
            Quantidade de Decimais: 2
            Descrição Sumária: Identifica o valor em reais (R$) da Dedução que incidiu sobre o Rendimento recebido pela pessoa no período de referência do respectivo quadro. Nos casos de valores ignorados, este campo está preenchido com “Noves”. Nos casos em que não ocorreu dedução, este campo esta preenchido com “Zeros”. Nos quadros em que não foi pesquisada a variável dedução, este campo está preenchido com “Zeros”.


15 – CÓDIGO DA DEDUÇÃO
            Posição Inicial: 73
            Formato: N - Numérico
            Tamanho: 5 dígitos (5 bytes)
            Quantidade de Categorias: 206
            Classificação: 800
 		Descrição Sumária: Identifica o tipo de Dedução que incidiu sobre o Rendimento. Esta variável deverá ser lida juntamente com a variável “Número do Quadro”, que irá compor o código do produto completo com 7 dígitos.


16 – MÊS DO ÚLTIMO RENDIMENTO
            Posição Inicial: 78
            Formato: N – Numérico
            Tamanho: 2 dígitos (2 bytes)
            Quantidade de Categorias = 13
            Classificação: 904
Descrição Sumária: Identifica o último mês que o rendimento bruto foi recebido pela pessoa no período de referência da pesquisa. Categorias: (00) Não se aplica, (01) Janeiro, (02) Fevereiro, (03) Março, (4) Abril, (05) Maio, (06) Junho, (07) Julho, (08) Agosto, (09) Setembro, (10) Outubro, (11) Novembro e (12) Dezembro.
Neste registro, esta variável é exclusiva para os itens do quadro 54.


            17 – NÚMERO DE MESES RECEBIDOS
Posição Inicial: 80
            Formato: N – Numérico
            Tamanho: 2 dígitos (2 bytes)
Descrição Sumária: Identifica o número de meses que o rendimento foi recebido pela pessoa no período de referência da pesquisa. O valor desta variável é “Zero” para os casos onde não se aplica.
Neste registro, esta variável é exclusiva para os itens do quadro 54.


   		18 - FATOR DE ANUALIZAÇÃO
            Posição Inicial: 82
            Formato: N - Numérico
            Tamanho: 2 dígitos (2 bytes)
		Descrição Sumária: Ver descrição da variável 14 do REGISTRO TIPO 06 – DESPESA DE 90 DIAS.


19 – DEFLATOR FATOR
            Posição Inicial: 84
            Formato: N - Numérico
            Tamanho: 5 dígitos (5 bytes)
            Quantidade de Decimais: 2
Descrição Sumária: Ver descrição da variável 15 do REGISTRO TIPO 06 – DESPESA DE 90 DIAS.


20 – VALOR DO RENDIMENTO DEFLACIONADO
            Posição Inicial: 89
            Formato: N - Numérico
            Tamanho: 11 dígitos (11 bytes)
            Quantidade de Decimais: 2
Descrição Sumária: Identifica o valor resultante da operação “VALOR DO RENDIMENTO” multiplicado pelo “DEFLATOR FATOR”.


21 – VALOR DA DEDUÇÃO DEFLACIONADO
            Posição Inicial: 100
            Formato: N - Numérico
            Tamanho: 11 dígitos (11 bytes)
            Quantidade de Decimais: 2
Descrição Sumária: Identifica o valor resultante da operação “VALOR DA DEDUÇÃO” multiplicado pelo “DEFLATOR FATOR”.


22 – VALOR DO RENDIMENTO ANUALIZADO E EXPANDIDO (FATOR 2)
            Posição Inicial: 111
            Formato: N - Numérico
            Tamanho: 16 dígitos (16 bytes)
            Quantidade de Decimais: 2
 		Descrição Sumária: Identifica o valor resultante da operação do “VALOR DO RENDIMENTO DEFLACIONADO” multiplicado pelo “FATOR DE ANUALIZAÇÃO” e pelo FATOR DE EXPANSÃO 2 (AJUSTADO P/ ESTIMATIVAS).
 		Para os itens do quadro 54, deve-se multiplicar também pela variável NÚMERO DE MESES RECEBIDOS.


23 – VALOR DA DEDUÇÃO ANUALIZADA E EXPANDIDA (FATOR 2)
            Posição Inicial: 127
            Formato: N - Numérico
            Tamanho: 16 dígitos (16 bytes)
            Quantidade de Decimais: 2
 		Descrição Sumária: Identifica o valor resultante da operação do “VALOR DA DEDUÇÃO DEFLACIONADO”  multiplicado pelo “FATOR DE ANUALIZAÇÃO” e pelo FATOR DE EXPANSÃO 2 (AJUSTADO P/ ESTIMATIVAS).
 		Para os itens do quadro 54, deve-se multiplicar também pela variável NÚMERO DE MESES RECEBIDOS.


24 – CÓDIGO DE IMPUTAÇÃO
            Posição Inicial: 143
            Formato: N - Numérico
            Tamanho: 2 dígitos (2 bytes)
            Quantidade de Categorias: 4
            Classificação: 902
Descrição Sumária: Ver descrição da variável 16 do REGISTRO TIPO 05 – INVENTÁRIO DE BENS DURÁVEIS.


 25 - RENDA MONETÁRIA MENSAL DA UNIDADE DE CONSUMO
 Posição inicial: 145
 Formato: N - Numérico
 Tamanho: 16 dígitos (16 bytes)
 Quantidade de Decimais: 2
      Descrição Sumária: Ver descrição da variável 39 do REGISTRO TIPO 02 – PESSOAS.


 26 - RENDA NÃO MONETÁRIA MENSAL DA UNIDADE DE CONSUMO
 Posição inicial: 161
 Formato: N - Numérico
 Tamanho: 16 dígitos (16 bytes)
 Quantidade de Decimais: 2
      Descrição Sumária: Ver descrição da variável 40 do REGISTRO TIPO 02 – PESSOAS.


 27 - RENDA TOTAL MENSAL DA UNIDADE DE CONSUMO
 Posição inicial: 177
 Formato: N - Numérico
 Tamanho: 16 dígitos (16 bytes)
 Quantidade de Decimais: 2
 Descrição Sumária: Ver descrição da variável 41 do REGISTRO TIPO
02 – PESSOAS.
```
