# Supplementary material for: Can a poverty-reducing and progressive tax and transfer system hurt the poor?
Source: J Dev Econ. 2016 Sep;122:63–75. doi: 10.1016/j.jdeveco.2016.04.001 (PMC4988485; doi:10.1016/j.jdeveco.2016.04.001)
Supplement: Supplementary file 1 — Supplementary material. [file mmc1.zip › documentation/POF0809/Despesa total 2008 - 2009.doc]

**COMPONENTES DAS LINHAS DAS**

**TABELAS DE DESPESA MONETÁRIA E NÃO MONETÁRIA MÉDIA MENSAL FAMILIAR**

**Observações Iniciais:**

- Este documento apresenta os códigos dos produtos componentes de cada linha das tabelas de Despesa Monetária e Não Monetária Média Mensal Familiar.

- Cada código com 5 dígitos foi composto através dos 2 dígitos da variável “Número do Quadro” e dos 3 primeiros dígitos da variável “Código do Item”.

- Os 2 últimos dígitos da variável “Código do Item” se referem a números sequenciais, utilizados para identificar sinônimos ou diferentes denominações regionais de um mesmo produto. Como estes sequenciais são agregados em um único código na construção do plano tabular, não precisam ser descritos na construção das linhas da tabela.

**Despesa total** - 1 + 2 + 3 + -------------------------- + 14

**Despesas correntes** - 1 + 2 + 3 + --------------------- + 12

**Despesas de consumo** - 1 + 2 + 3 + ------------------ + 11

**1 - Alimentação** - todos os itens do quadro 24, todos os itens dos grupos do POF 3 (exceto grupos 86 a 89), 41006, 48033, 49026

**2 - Habitação** - 2.1 + 2.2 + 2.3 + 2.4 + 2.5 + 2.6 + 2.7 + 2.8

2.1 – Aluguel – 2.1.1 + 2.1.2

2.1.1 – Aluguel monetário – 10001, 10003, 10006, 10008

2.1.2 – Aluguel não monetário – 10090

2.2 – Condomínio – 10004, 10009

2.3 – Serviços e taxas – 2.3.1 + 2.3.2 + 2.3.3 + 2.3.4 +2.3.5 + 2.3.6 + 2.3.7

2.3.1 – Energia elétrica – 06002

2.3.2 – Telefone fixo – 06004

2.3.3 – Telefone celular – 28023, 28024

2.3.4 – Pacote de telefone, tv e internet – 06008 a 06011, 06013 a 06015

2.3.5 – Gás doméstico – 06003, 07001

2.3.6 – Água e esgoto – 06001

2.3.7 – Outros – 06005, 06007, 06012, 06999, 12003, 12005 a 12023, 12025, 12030 a 12035, 12999

2.4 – Manutenção do lar – 07002 a 07011, 07999, 08001 a 08068, 08070 a 08079, 08999, 19001 a 19030, 19099, 86057, 86083, 86090

2.5 – Artigos de limpeza – 86001 a 86008, 86011, 86014 a 86027, 86030 a 86034, 86042, 86045, 86056, 86058, 86060 a 86061, 86063 a 86066, 86069 a 86071, 86073, 86075, 86077, 86086, 86092 a 86095

2.6 – Mobiliários e artigos do lar – 15003, 15023, 15083, 15132 a 15140, 15145 a 15146, 15150 a 15154, 15158, 15161, 15164, 15168, 15177, 15182, 15185 a 15186, 15188, 15190 a 15194, 16016 a 16017, 16023, 17001 a 17091, 17999, 18001 a 18053, 18999, 37003 a 37006, 37010 a 37013, 37016, 37020, 37023 a 37024, 37031 a 37033, 37038 a 37040, 37043, 37045 a 37047, 39001 a 39099, 39999, 40014, 40019 a 40020, 86009 a 86010, 86012 a 86013, 86028 a 86029, 86035 a 86041, 86043 a 86044, 86046 a 86055, 86059, 86062, 86067 a 86068, 86072, 86074, 86076, 86078 a 86082, 86084 a 86085, 86087 a 86089, 86091

2.7 – Eletrodomésticos **–** 15001 a 15002, 15004 a 15022, 15024 a 15054, 15056 a 15059, 15061 a 15063, 15065 a 15069, 15071 a 15080, 15082, 15084 a 15124, 15128 a 15131, 15141 a 15144, 15147 a 15148, 15155 a 15156, 15159 a 15160, 15163, 15166, 15169 a 15171, 15173, 15175, 15180 a 15181, 15183, 15187, 15189, 15197, 15206, 15208 a 15214, 15216, 15999

2.8 – Consertos de artigos do lar – 09001 a 09147, 09149 a 09150, 09156 a 09162, 09999

**3 – Vestuário** – 3.1 + 3.2 + 3.3 + 3.4 + 3.5 + 3.6

3.1 – Roupa de homem – 34001 a 34033, 34999

3.2 – Roupa de mulher – 35001 a 35059, 35999

3.3 – Roupa de criança – 36001 a 36048, 36999

3.4 – Calçados e apetrechos – 38001 a 38052, 38054 a 38066, 38068, 38071 a 38072, 38999

3.5 – Jóias e bijuterias – 40001, 40022, 46001 a 46008, 46010 a 46011, 46999

3.6 – Tecidos e armarinhos – 37001 a 37002, 37008 a 37009, 37014 a 37015, 37017 a 37019, 37021, 37025 a 37030, 37034 a 37037, 37044, 37999

**4 – Transporte** – 4.1 + 4.2 + 4.3 + 4.4 + 4.5 + 4.6 + 4.7

4.1– Urbano – 23001 a 23005, 23010 a 23012, 23014 a 23016, 23020 a 23021, 23023, 23025 a 23026, 23999, 48034

4.2 – Gasolina - veículo próprio – 23007, 23028
4.3 – Álcool - veículo próprio – 23006

4.4 - Manutenção e acessórios – 23019, 43001 a 43008, 43011 a 43013, 43017, 43019, 43027, 43029, 43035, 43040, 43042, 43999

4.5 – Aquisição de veículos – 50012, 51001 a 51023, 51999

4.6 – Viagens esporádicas – 41001 a 41005, 41008 a 41017, 41020 a 41022, 41040 a 41042, 41999

4.7 – Outras – 23008 a 23009, 23013, 23017 a 23018, 23022, 50003, 50005 a 50008, 50010, 50013, 50017, 50999

**5 - Higiene e cuidados pessoais** - 5.1 + 5.2 + 5.3 + 5.4

5.1 - Perfume – 30002

5.2 - Produtos para cabelo – 30026, 89004

5.3 - Sabonete – 89003

5.4 - Instrumentos e produtos de uso pessoal – 29314, 30001, 30003 a 30021, 30023, 30025, 30029 a 30031, 30999, 89001 a 89002, 89005 a 89015

**6 - Assistência à saúde** - 6.1 + 6.2 + 6.3 + 6.4 + 6.5 + 6.6 + 6.7 + 6.8 + 6.9 + 6.10

6.1 - Remédios – 29001 a 29049, 29300, 29999

6.2 - Plano/Seguro saúde – 42001 a 42003, 42044 a 42045

6.3 - Consulta e tratamento dentário – 42023 a 42024

6.4 - Consulta médica – 42009 a 42012

6.5 - Tratamento médico e ambulatorial – 42004, 42008, 42020 a 42022, 42025, 42028 a 42034, 42047

6.6 - Serviços de cirurgia – 42005

6.7 - Hospitalização – 42006

6.8 - Exames diversos – 42013 a 42019, 42043, 42046

6.9 - Material de tratamento – 29301 a 29303, 29305 a 29309, 29311 a 29312, 42039 a 42041

6.10 - Outras – 29304, 29310, 29313, 42007, 42026 a 42027, 42035 a 42038, 42042, 42999

**7 - Educação** - 7.1 + 7.2 + 7.3 + 7.4 + 7.5 + 7.6

7.1 - Cursos regulares – 49001, 49031 a 49032

7.2 - Curso superior – 49033

7.3 - Outros cursos e atividades – 28055, 49002 a 49003, 49011, 49015, 49022, 49034 a 49041, 49043 a 49044, 49047, 49049 a 49052, 49054 a 49084, 49086 a 49092

7.4 - Livros didáticos e revistas técnicas – 49006 a 49008, 49045

7.5 - Artigos escolares – 32001 a 32002, 32999, 49019 a 49021, 49025, 49029

7.6 - Outras – 48003, 48013, 48036, 49004 a 49005, 49009 a 49010, 49012 a 49014, 49016 a 49018, 49024, 49027 a 49028, 49030, 49042, 49046, 49048, 49053, 49085, 49093, 49999

**8 - Recreação e cultura** - 8.1 + 8.2 + 8.3 + 8.4 + 8.5

8.1 - Brinquedos e jogos – 33002, 33012, 33016

8.2 - Celular e acessórios – 46009, 46012 a 46013

8.3 - Periódicos, livros e revistas não didáticos – 27001 a 27016, 27019 a 27021, 32004 a 32005

8.4 - Recreações e esportes – 28001 a 28005, 28011 a 28021, 28027 a 28028, 28030 a 28039, 28046, 28049, 28051, 28053 a 28054, 28056, 28058 a 28059, 28999, 41026, 41033 a 41039, 45014, 45016 a 45018

8.5 - Outras – 15125 a 15127, 15167, 15178 a 15179, 15184, 15200, 15207, 16001 a 16015, 16020 a 16022, 16028 a 16034, 16036 a 16038, 16040, 27017 a 27018, 27999, 28006 a 28010, 28022, 28025 a 28026, 28029, 28040 a 28045, 28047 a 28048, 28050, 28052, 28057, 33001, 33003 a 33011, 33013 a 33015, 33017 a 33026, 33999, 37041 a 37042

**9 - Fumo** – 25001 a 25018, 25999, 38053, 38067, 38069 a 38070

**10 - Serviços pessoais** - 10.1 + 10.2 + 10.3 + 10.4

10.1 - Cabeleireiro – 31001 a 31002, 31042 a 31043, 31047

10.2 - Manicuro e pedicuro – 31003

10.3 - Consertos de artigos pessoais – 31004, 31010, 31013 a 31016, 31018, 31020 a 31024, 31026 a 31027, 31029 a 31031, 31033, 31039, 31044 a 31045, 31051

10.4 - Outras – 31005 a 31009, 31011 a 31012, 31017, 31019, 31028, 31032, 31034 a 31038, 31040 a 31041, 31046, 31048 a 31050, 31052 a 31053, 31999

**11 - Despesas diversas** - 11.1 + 11.2 + 11.3 + 11.4 + 11.5 + 11.6

11.1 - Jogos e apostas - 26001 a 26018, 26020, 26022, 26024, 26027, 26029 a 26030, 26999

11.2 - Comunicação – 22001 a 22005, 22999

11.3 - Cerimônias e festas – 45001 a 45013, 45999

11.4 - Serviços profissionais – 44001 a 44019

11.5 - Imóveis de uso ocasional – 41025, 47005 a 47007, 47009 a 47020, 47023, 47025, 47999

11.6 – Outras – 11081 a 11084, 12004, 13001 a 13022, 13999, 16018 a 16019, 16024 a 16027, 16039, 16999, 32006, 32015, 37007, 37022, 40002 a 40013, 40015 a 40018, 40021, 40023 a 40026, 40999, 41007, 41018 a 41019, 41023 a 41024, 44020 a 44021, 44999, 87001 a 87010, 87012 a 87015, 88001

**12 - Outras despesas correntes** - 12.1 + 12.2 + 12.3 + 12.4 + 12.5 + 12.6

12.1 - Impostos – 10005, 10010 a 10014, 47008, 47022, 48035, 48038, 50001 a 50002, 50004, 50009, 50014 a 50016, 50018 a 50019, 53601 a 53609, 53701 a 53709

12.2 - Contribuições trabalhistas – 19501 a 19530, 19599, 48001, 48018, 48026, 48039, 53501 a 53509

12.3 - Serviços bancários – 44022 a 44033, 44035 a 44036, 44038, 44042, 44047

12.4 - Pensões, mesadas e doações – 48002, 48004 a 48005, 48021 a 48024, 48040, 48043 a 48044, 50011

12.5 - Previdência privada – 48006

# 12.6 - Outras – 12024, 47024, 48007 a 48010, 48014, 48017, 48019, 48025, 48028 a 48032, 48037, 48041, 48999, 54501 a 54538, 55501 a 55552, 55554 a 55559, 55562 a 55566

**13 - Aumento do ativo** - 13.1 + 13.2 + 13.3

13.1 - Imóvel (aquisição) – 12001 a 12002, 47001 a 47004, 47026

13.2 - Imóvel (reforma) – 11001 a 11067, 11069 a 11080, 11085 a 11089, 11999

13.3 - Outros investimentos – 15149, 15165, 15172, 15174, 47021, 47027, 48020, 48042

**14 - Diminuição do passivo** - 14.1 + 14.2

14.1- Empréstimo – 48011 a 48012, 48015, 48027, 48045

14.2 - Prestação de imóvel - 10002, 10007
