# Supplementary material for: Can a poverty-reducing and progressive tax and transfer system hurt the poor?
Source: J Dev Econ. 2016 Sep;122:63–75. doi: 10.1016/j.jdeveco.2016.04.001 (PMC4988485; doi:10.1016/j.jdeveco.2016.04.001)
Supplement: Supplementary file 1 — Supplementary material. [file mmc1.zip › documentation/POF0809/Renda 2008 - 2009.doc]

**COMPONENTES DAS LINHAS DAS**

**TABELAS DE RENDIMENTO TOTAL E VARIAÇÃO PATRIMONIAL MÉDIO MENSAL FAMILIAR**

**Observações Iniciais:**

- Este documento apresenta os códigos dos produtos componentes de cada linha das tabelas de Rendimento Total e Variação Patrimonial Médio Mensal Familiar

- Cada código com 5 dígitos foi composto através dos 2 dígitos da variável “Número do Quadro” e dos 3 primeiros dígitos da variável “Código do Item”.

- Os 2 últimos dígitos da variável “Código do Item” se referem a números sequenciais, utilizados para identificar sinônimos ou diferentes denominações regionais de um mesmo produto. Como estes sequenciais são agregados em um único código na construção do plano tabular, não precisam ser descritos na construção das linhas da tabela.

**Rendimento total e variação patrimonial** - 1 + 2

**1 - Rendimento total** - 1.1 + 1.2 + 1.3 + 1.4 + 1.5

**1.1 - Rendimento do trabalho -** 1.1.1 + 1.1.2 + 1.1.3

1.1.1 - Empregado – 53001 a 53004, 54015 a 54018, 54020, 54036 a 54038, 55001 a 55002, 55011, 55026, 55035, 55037 a 55043, 55045, 55062 a 55063

1.1.2 - Empregador – 53005, 55003

1.1.3 - Conta própria – 53006

**1.2 - Transferência -** 1.2.1 + 1.2.2 + 1.2.3 + 1.2.4 + 1.2.5 + 1.2.6

1.2.1 - Aposentadoria e pensão do INSS – 54001 a 54002, 55022 a 55023, 55064

1.2.2 - Aposentadoria, pensão da previdência pública – 54003 a 54004, 55065

1.2.3 - Aposentadoria, pensão da previdência privada - 54005, 54023, 55025, 55033, 55066

1.2.4 - Programas sociais federais – 54010 a 54012, 54024

1.2.5 - Pensão alimentícia, mesada ou doação – 54007, 54026, 54032, 55030

1.2.6 - Outras transferências – 54006, 54013 a 54014, 54019, 54021 a 54022, 54025, 54027 a 54031, 54035, 55017 a 55018, 55029, 55031, 55055 a 55059

**1.3** - **Rendimento de aluguel** - 54008 a 54009

**1.4 - Outras rendas** – 54033 a 54034, 55004, 55007, 55009, 55012 a 55013, 55015 a 55016, 55019 a 55021, 55024, 55027 a 55028, 55032, 55034, 55036, 55046 a 55052, 55054

**1.5 - Rendimento não monetário**

## 1 – Somar todos os valores referentes as despesas não monetárias (quando o quesito Forma de Aquisição é entre 7 e 11). Exceto, os valores do código 10090 (Aluguel Estimado).

2 – Subtrair do valor referente ao código 10090 (Aluguel Estimado) os valores das despesas monetárias (quando o quesito Forma de Aquisição é entre 1 e 6) dos seguintes códigos:

Códigos do quadro 8 – 08001 a 08017, 08019 a 08072 e 08077 a 08999

Códigos do quadro 10 – 10005, 10010, 10012 a 10013

## Códigos do quadro 12 – 12005 a 12025, 12030 a 12033 e 12999

Só considerar quando a diferença for > 0.

3 - Resultado final: (1+2).

**2 – Variação Patrimonial –** Somatório dos códigos 55005 a 55006, 55008, 55010, 55014, 55044 + para cada informante as seguintes diferenças:

Código 57001 – Código 56001 (só considerar quando a diferença for > 0)

Código 57002 – Código 56002 (só considerar quando a diferença for > 0)

Código 57003 – Código 56003 (só considerar quando a diferença for > 0)

Código 57004 – Código 56004 (só considerar quando a diferença for > 0)
